# Comprehensive Proteomics Analysis of Laticifer Latex Reveals New Insights into Ethylene Stimulation of Natural Rubber Production

**DOI:** 10.1038/srep13778

**Published:** 2015-09-08

**Authors:** Xuchu Wang, Dan Wang, Yong Sun, Qian Yang, Lili Chang, Limin Wang, Xueru Meng, Qixing Huang, Xiang Jin, Zheng Tong

**Affiliations:** 1 Key Laboratory of Biology and Genetic Resources for Tropical Crops, Institute of Tropical Biosciences and Biotechnology, Chinese Academy of Tropical Agricultural Sciences, Haikou Hainan 571101, China

## Abstract

Ethylene is a stimulant to increase natural rubber latex. After ethylene application, both fresh yield and dry matter of latex are substantially improved. Moreover, we found that ethylene improves the generation of small rubber particles. However, most genes involved in rubber biosynthesis are inhibited by exogenous ethylene. Therefore, we conducted a proteomics analysis of ethylene-stimulated rubber latex, and identified 287 abundant proteins as well as 143 ethylene responsive latex proteins (ERLPs) with mass spectrometry from the 2-DE and DIGE gels, respectively. In addition, more than 1,600 proteins, including 404 ERLPs, were identified by iTRAQ. Functional classification of ERLPs revealed that enzymes involved in post-translational modification, carbohydrate metabolism, hydrolase activity, and kinase activity were overrepresented. Some enzymes for rubber particle aggregation were inhibited to prolong latex flow, and thus finally improved latex production. Phosphoproteomics analysis identified 59 differential phosphoproteins; notably, specific isoforms of rubber elongation factor and small rubber particle protein that were phosphorylated mainly at serine residues. This post-translational modification and isoform-specific phosphorylation might be important for ethylene-stimulated latex production. These results not only deepen our understanding of the rubber latex proteome but also provide new insights into the use of ethylene to stimulate rubber latex production.

It has long been known that although ethylene stimulates natural rubber production, many genes involved in natural rubber biosynthesis are not induced upon treatment with ethephon^1^. Rubber latex is the cytoplasm of specialized cells called laticifers located in the bark phloem of rubber tree (*Hevea brasiliensis*), the only plant commercially cultivated to produce natural rubber^2^. The rubber particle, a specific organelle found in rubber latex, is the only site of rubber biosynthesis and storage in laticifers^3^. Natural rubber (cis-1,4-polyisoprene) is generated from rubber latex by a living carbocationic polymerization process that includes initiation, elongation and termination steps^4^. It is known that natural rubber biosynthesis occurs through a menvalonate (MVA) pathway that is catalyzed by many enzymes and cofactors^4^,^5^ and begins with the synthesis of isopentenyl pyrophosphate (IPP). In an early step, 3-hydroxy-3-methylglutaryl coenzyme A synthase (HMGS) and reductase (HMGR) activate the stored substrate. Rubber transferase (cis-prenyl transferase, CIPT) utilizes pyrophosphates to initiate rubber molecule synthesis, as well as IPP to form the polymer, and is thus a key enzyme in rubber biosynthesis. During elongation, rubber elongation factor (REF) and small rubber particle protein (SRPP) play key roles, and in isoprenoid biosynthesis, farnesyl diphosphate synthase (FADS) catalyzes the last common substrate^5^.

The use of ethylene has led to a technological revolution in natural rubber production^6^. As demonstrated in a ^14^C labeling experiment, ethylene was released from the zone to the upper leaf shortly after the introduction of an ethylene generator, such as ethephon or ethrel (chloro-2-ethyl phosphonic acid)^7^. In addition, the treatment of rubber tree bark with ethephon induces fresh latex production and stimulates latex regeneration between tappings^3^,^6^. This stimulation is associated with obvious changes in both the physiology and metabolism of laticifers^6^. As a result, ethylene-mediated technology is currently widely used in commercial latex production^2^.

Paradoxically, the expression of most genes involved in rubber biosynthesis are inhibited or unchanged upon ethylene stimulation^2^,^8^. Therefore, some researchers have even speculated that ethylene has little direct effect on rubber biosynthesis and have suggested that increases in latex yield could be attributed to a prolongation of latex flow^2^,^9^. Of the three key enzymes involved in rubber biosynthesis initiation, it has been reported that *FADS*^8^ and *CIPT*^2^ are not significantly affected by ethylene.

The third enzyme, HMGR, has 3 family members named *hmgr1*, *hmgr2* and *hmgr3* in rubber tree; while *hmgr1* is induced by ethylene, stimulation does not influence its enzyme activity^10^. In the first reported accelerative effect of ethephon on rubber biosynthesis, HMGS gene expression and enzyme activity were significantly enhanced upon the addition of ethylene^11^.

Based on these observations, we considered whether the regulation of rubber latex production by ethylene stimulation might occur not solely at the gene level but also at the protein level, with post-translational modifications (PTMs) playing crucial roles in controlling the final function of enzymes involved in rubber biosynthesis. We therefore conducted an in-depth comparative proteomics analysis of rubber latex exposed to different ethylene treatments and obtained the first comprehensive latex proteome profiles. In our study, 143 and 404 ethylene-responsive latex proteins (ERLPs) were generated by two-dimensional differential in-gel electrophoresis (2-D DIGE) and isobaric tags for relative and absolute quantitation (iTRAQ), respectively, and 59 differentially expressed phosphoproteins were identified *via* phosphoproteomics analysis. To our knowledge, this is the first in-depth comprehensive proteomics analysis of rubber latex following ethylene stimulation, and this information may help uncover new mechanisms for the stimulation of rubber latex production.

## Results

### Ethylene significantly improved rubber latex yield and small rubber particle generation

**Figure 1:**
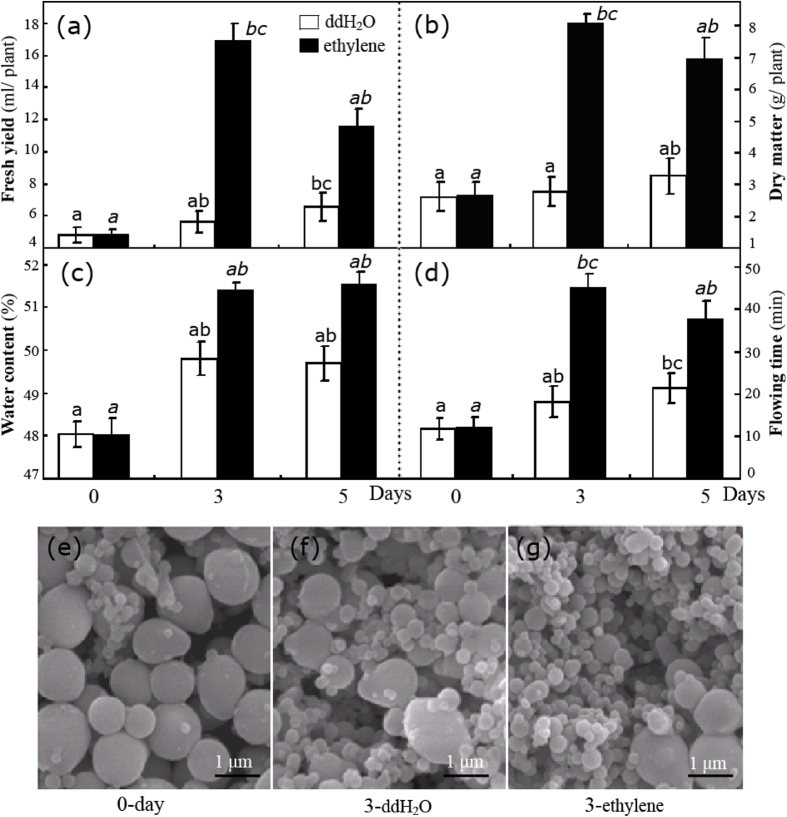
Changes in rubber latex parameters after different treatments. The fresh yield (**a**), dry matter (**b**), water content (**c**) and flow time (**d**) for latex collected from rubber trees treated with ethylene (3% ethephon, marked with black rectangle) and ddH_2_O (white) for 0, 3 and 5 days are presented. Total rubber particles from untreated plants (**e**, day 0) and plants treated with ddH_2_O (**f**) and ethylene (**g**) for 3 days were examined under SEM.

The effects of ethylene stimulation are more obvious in virgin plants than in mature trees^9^,^12^,^13^. A low-frequency tapping procedure referred to as “half-spiral and once tapping in three days plus ethrel” is the recent method of choice for latex collection^14^. Therefore, we used this procedure on virgin plants in this study. Overall, ethylene stimulation sharply improved the yield of fresh latex (Fig. 1a) and dry matter (Fig. 1b). Before treatment, approximately 5 ml of latex was recovered per plant. After ethylene treatment for 3 and 5 days, the volume of recovered latex reached ∼17 and 12 ml, respectively. Compared with the control, ddH_2_O-treated plants also exhibited a significant increase in latex (Fig. 1a), which were attributed to the mechanical effects of wounding caused by tapping^15^. Consistent with reported results^7^,^9^,^16^, ethylene also improved the latex water content (Fig. 1c) and markedly prolonged the latex flow time (Fig. 1d). *Hevea* rubber particles are spherical or pear-shaped, measuring approximately 0.02–3.0 μm diameter and, based on their bimodal size distribution, including both large and small rubber particles (LRPs and SRPs, respectively)^17^,^18^. In this study, the accumulation of SRPs was visibly induced by ethylene stimulation (Fig. 1e–g). In control, LRPs were the most frequently observed rubber particles (Fig. 1e). After tapping, the number of SRPs increased substantially (Fig. 1f). Notably, almost all examined rubber particles were SRPs following three days of ethylene (E-3) treatment (Fig. 1g). Recently, SRPP protein in SRPs has been reported to play a more important role than REF does in LRP natural rubber biosynthesis^5^,^18^,^19^. Taken with these reports^5^,^18^,^19^, our observations suggest that the ethylene stimulation of rubber latex can likely be attributed to the generation of SRPs.

### Identification and functional analysis of abundant proteins in rubber latex

**Figure 2:**
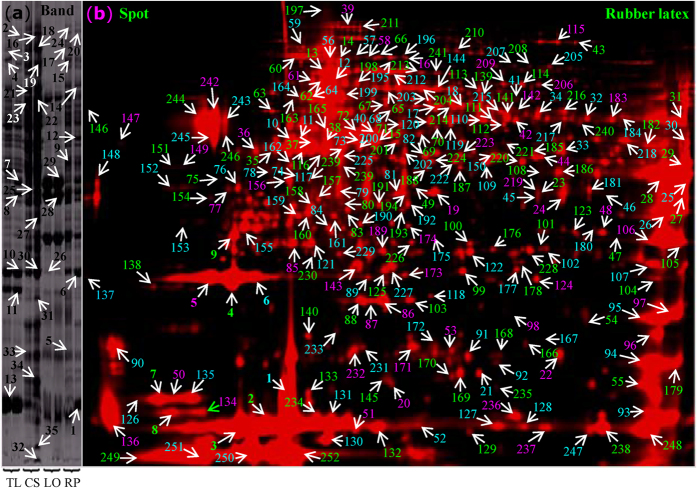
Proteome profiles and MS identification of main latex proteins. Protein bands of total latex (TL), C-serum (CS), lutoids (LO), and rubber particles (RP) from the D-3 and E-3 plants (from left to right) were identified by MS (**a**). Total latex proteins labeled with Cy5 on the pH 4–7 range 2-DE gels were visualized by a Typhoon scanner. The main protein spots were excised and identified by MS, which are marked with numbers in different colors (**b**). The protein identities are presented in Fig. S2 and Table S1.

To determine the protein profile of latex, the main proteins on both 1-DE and 2-DE gels were subjected to mass spectrometry (MS) analysis. The main bands of different latex fractions (Fig. 2a) were excised for in-gel digestion, and 35 proteins were positively identified. Then, the abundant protein spots (Vol% > 0.01) on the 2-DE gel (Fig. 2b) were subjected to MS analysis. Finally, 252 proteins were identified (Fig. S1; Table S1). Among them, 121 proteins were identified from *H. brasiliensis*, and 36 proteins were from *Ricinus communis* (Fig. S2a; Table S1). Radial chart analysis revealed that more than 90% of the proteins were distributed between the cyclical line 0.6 and 1.2 (Fig. S2b). These latex proteins belonged to 16 functional groups, the largest of which included 38 proteins involved in post-translational modification, followed by the categories of carbohydrate transport and metabolism, energy production, ribosomal structure and biogenesis (Fig. S1; Fig. S2c). Gene ontology (GO) pathway and biological process analysis verified that most of the proteins identified were involved in carbohydrate metabolism (Fig. S2d). An additional 19 proteins were related to abiotic stress responses, followed by 17 proteins involved with glycolysis. By GO cell biological analysis, 42 protein species were found to be overrepresented in the cytoplasm (Fig. S2e), in agreement with the fact that rubber latex is present in the cytoplasm of laticifers in *Hevea* phloem^15^.

Ten proteins belonged to the phosphopyruvate hydratase complex. TargetP analysis revealed that most latex proteins had no specific subcellular location. Only 11, 5, and 2 proteins localized to the secretary pathway, plastid, and mitochondrion, respectively. Finally, GO functional analysis demonstrated that the largest group of proteins was involved in adenosine triphosphate (ATP) binding, followed by metal ion binding and hydrolase activity (Fig. S2), indicating that proteins with ATP-binding activity might play crucial roles in latex.

Analysis of protein abundance revealed that most abundant protein spots contained REF isoforms, followed by SRPP and elicitor-responsive proteins. Prohevein, a 50-kDa protein, and chitinase were also enriched in latex (Fig. S2g). Notably, several spots found at different locations on 2-DE gels were identified as isoforms or different species of the same protein. For example, a total of 21 spots (17.2%) were identified as REF, and at least 13 spots (5.9%) were identified as SRPP (Fig. S2i), indicating that REF and SRPP are the two most abundant proteins in latex.

### Determination and identification of ERLPs by 2-D DIGE and iTRAQ

**Figure 3:**
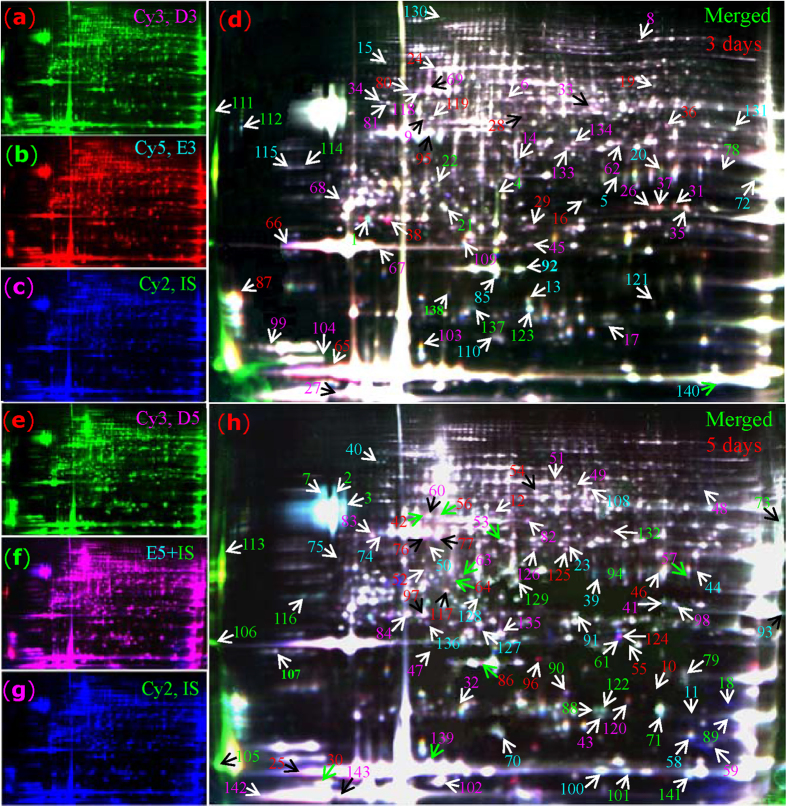
DIGE analysis of rubber latex proteins upon ethylene treatment. Proteins obtained from rubber trees treated with ethylene and ddH_2_O for 3 and 5 days (E-3, E-5; D-3, D-5) were labeled with different fluorescence dyes (Cy3 or Cy5). The combined images of each sample with internal standard (IS, labeled with Cy2) are presented. The differential spots (D1-D143) identified by MS are numbered with different colors (red, up-regulated upon ethylene; green, down-regulated).

2-D DIGE was performed to identify ERLPs. Typical DIGE gels for each treatment and the combined images are presented (Fig. 3a–h). The global protein patterns on DIGE gels with ∼1750 ± 38 spots remained consistent, and 143 protein spots (termed D1-D143) with a more than 1.3-fold change (confidence > 95%) following ethylene treatment were positively identified by MS (Fig. 3; Fig. S3; Table S2). In 3-day treated latex, 41 proteins had increased, while 43 had decreased. The top five induced proteins were mitogen-activated protein kinase (MAPK), aspartic proteinase precursor, enolase, regulator of ribonuclease, and caffeic acid O-methyltransferase (CAMT). Following 5 days of ethylene treatment, a greater number of differential proteins was observed. Among them, 50 proteins, including rubber elongation factor isoforms, Hev b7.02, esterase and proteasome, had increased, while 55 had decreased. Notably, although most spots containing rubber biosynthesis-related proteins, such as REF and SRPP, did not change significantly, some of their family members or isoforms were sharply increased by ethylene treatment. With regard to REF, 14 isoforms were significantly changed after ethylene treatment, including 10 that were sharply induced (D25, 27, 30, 32, 65, 86, 96, 139, 142 and 143). With regard to SRPP, at least 5 protein isoforms (D52, 66, 67, 99 and 104) increased. However, glucanase and hevein - two key activators of lutoid-mediated rubber particle aggregation (RPA) and sequential latex coagulation^19^ -were decreased upon ethylene treatment (Fig. 3; Fig. S3; Table S2). The decrease of accumulation of glucanase and hevein in ethylene-treated latex may inhibit RPA, thus maintaining latex flow.

**Figure 4:**
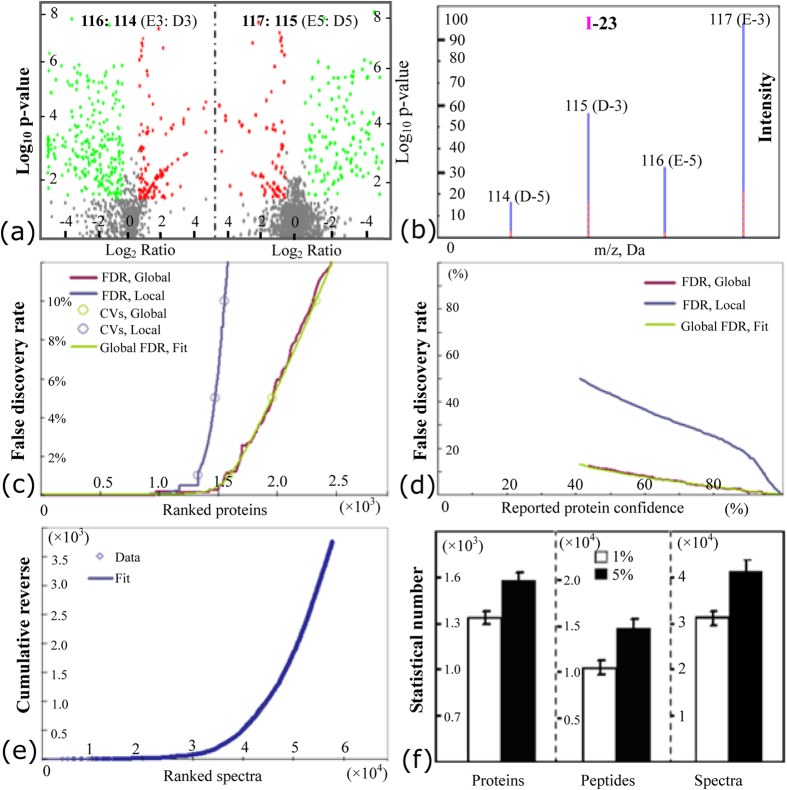
iTRAQ analysis of rubber latex proteins upon ethylene treatment. Peptides from D3, E3, D5 and E5 latex proteins were labeled with iTRAQ labeling reagents with molecular masses of 115, 117, 114 and 116 Da, respectively. The log_10_ p-value in the volcano plot for the ratios of 117:115 (E3/D3, left side) and 116:114 (E5/D5, right side) are demonstrated (**a**). Red spots, proteins significantly (fold change > 1.5, P < 0.05) induced in ethylene treated samples; green spots, proteins significantly reduced in ethylene treated samples. The typical peptide intensity of the four labeling reagents in I-23 is also highlighted (**b**). Then, false discovery rates for the ranked proteins (**c**) and reported protein confidence (**d**) at both the local and global levels are determined. The values of cumulative reverse for the ranked spectra are shown (**e**). Finally, the statistics describing proteins, peptides and spectra with 99% and 95% confidence are presented as the mean ± SD (**f**). Abbreviations: FDR, false discovery rate; CVs, confidence values.

Furthermore, iTRAQ was performed to detect additional ERLPs. The volcano plot p-values for the changed patterns of all identified proteins with 95% peptides >2 upon ethylene stimulation (Fig. 4a), the typical peptide of the protein I-23 (Fig. 4b), the false discovery rates for the ranked proteins (Fig. 4c) and reported protein confidence (Fig. 4d) at both the local and global levels, the cumulative reverse values for the ranked spectra (Fig. 4e), and the statistical values of proteins, peptides and spectra (Fig. 4f) are presented in Fig. 4. Finally, 1,596 ± 50 unique proteins (termed as protein I1-I1621) were identified from 14,977 distinct peptide sequences with 95% confidence (score higher than 1.3; error factor <2). Among them, 1,330 ± 45 proteins were identified with 99% confidence (Fig. 4f; Fig. S4; Table S3). Similar to the results obtained by 2-DE, REF was the most abundant protein with 553 matched peptides, followed by SRPP and hevamine. Abundant proteins, including glucanase, Hev b7.02, HEV1.2, elicitor-responsive protein, Hev b5, pro-hevein, ACAT and enolase, were identified by iTRAQ. Although many abundant proteins [e.g., REF (I1), SRPP (I4), and hevamine (I6)] showed no significant changes in general, some of their family members or protein isoforms (e.g., I63, I357 and I1207 for REF; I39 for SRPP) changed substantially upon ethylene treatment (Fig. S4), consistent with our observation in DIGE gels (Fig. 3).

After ethylene treatment, 404 proteins were significantly changed more than 1.5 fold. After 3 days of treatment, 140 proteins were substantially induced. Among them, the most highly induced was hydrolase, followed by an isoform of glucanase, potassium channel protein, ribosomal protein, galactosyltransferase, myo-inositol-1-phosphate synthase, and kinase receptor. In contrast, 264 proteins were down-regulated upon treatment. Among them were peptidyl-prolyl cis-trans isomerase, lactoylglutathione lyase, and thioredoxin. After increasing the ethylene treatment time to 5 days (E-5), most ERLPs demonstrated accumulation patterns similar to E-3 latex (Fig. S4; Table S3).

### Phosphoproteomics analysis of rubber latex upon ethylene stimulation

When the differentially expressed proteins recovered from DIGE and iTRAQ methods were combined, we noticed a large amount of ERLPs with phosphatase/phosphotransferase activity (33 proteins) and kinase activity (22 proteins). At the biological process level, 39 proteins actively responded to cadmium ions. Among them, 10 proteins showed phosphate-binding activity, and 7 proteins had strong phosphopyruvate hydratase activity (Fig. S3; Fig. S4). These results indicated that phosphorylation or dephosphorylation of proteins might be important in the ethylene-stimulation of rubber latex and suggested that rubber latex contains many phosphorylated enzymes.

**Figure 5:**
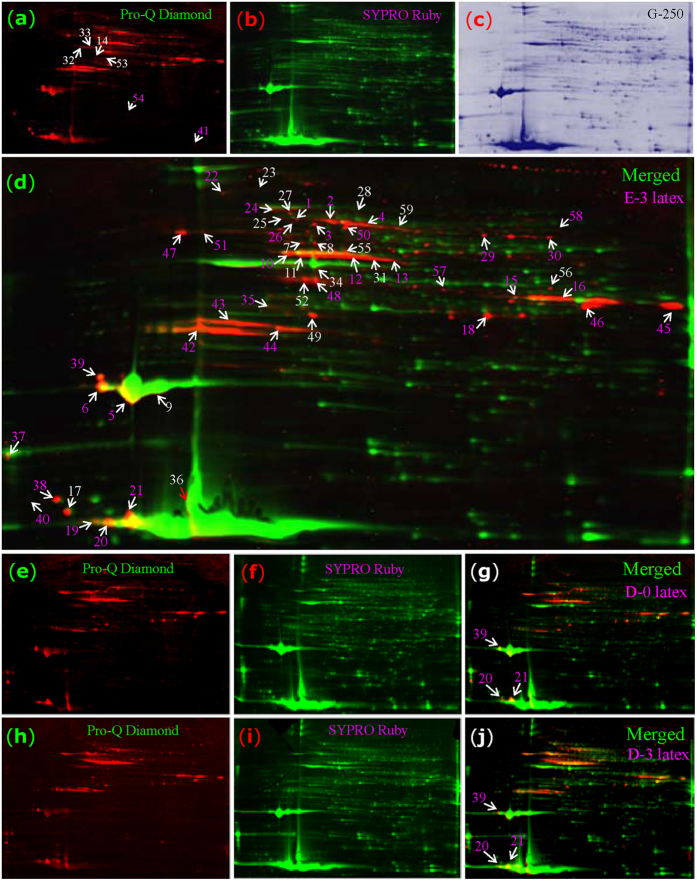
Phosphoproteomics analysis of rubber latex proteins upon ethylene treatment. The 2-DE gel for E-3 latex was stained with Pro-Q Diamond dye to detect phosphoproteins (**a**, red). It was then restained with SYPRO Ruby (**b**, green). The same gel was restained by G250 (**c**, blue). The combination image of Pro-Q Diamond and SYPRO Ruby are presented to demonstrate the specific phosphorylated proteins (**d**, merged). The latex proteins obtained from the D-0 (**e**-**g**) and D-3 (**h**-**j**) plants were also stained by Pro-Q Diamond and SYPRO Ruby. Finally, 59 phosphoproteins (P1-P59) were identified by MS (**d**).

Therefore, we confirmed the presence of phosphorylated proteins in rubber latex. Using Pro-Q Diamond dye, phosphospecific proteins in latex exposed to different ethylene treatment conditions were visualized and more than 200 protein spots were detected in 2-DE gels (Fig. 5). In the merged image of the Pro-Q Diamond- and SYPRO Ruby-stained gels, specific phosphorylated protein spots red in color were clearly observed. Many protein spots were visualized by Pro-Q Diamond for latex under the D-0 and D-3 treatments, indicating that these proteins were phosphorylated under control conditions. However, the phosphorylation was substantially more apparent after ethylene treatment (Fig. 5). Finally, the main spots of differentially expressed phosphoproteins were subjected to MS, and 59 differential proteins (Spots P1-P59) were positively identified (Fig. S5; Table S4). Among these phosphoproteins, REF (P19-21 and P36) was the most abundant, followed by the 7 isoforms of SRPP (P5, 6, 9, 17 and 38–40) and one glucanase isoform (P45). Other abundant phosphorylated proteins included malate dehydrogenase, cytoplasmic aldolase, linamarase, latex patatin homolog, Hev b7, and several hypothetical proteins (Fig. S5; Table S4). A large portion, including phosphoesterase, phosphoglycerate kinase, phosphoglucomutase, phospholipase, and 14-3-3 protein, had high phosphotransferase and kinase activity. These enzymes were involved in Ca^2+^ binding and signal transduction.

Although the main spots containing REF and SRPP were not stained red, several isoforms of REF (P19-21 and P36) and SRPP (P5, 6 and 39) did stain as phosphorylated (Fig. 5d), indicating that isoform-specific phosphorylation might has a positive correlation between phosphoprotein abundance and ethylene-stimulated rubber latex production. To determine which amino acid sites in SRPP were phosphorylated, spot P6 was collected from six independent 2-DE gels and subjected to tandem mass spectrometry (MS/MS), which revealed residue-specific phosphorylation. For example, the phosphorylation probability of the third serine (Ser-3) in the peptide QVSAQTYSVAQDAPR was 98.48%, while this value was only 0.03% in Ser-8. For threonine (T6), the phosphorylation probability was 1.48%. This value in tyrosine (Y7) was as low as 0.04% in this peptide (Fig. S5). These results provided evidence that some SRPP isoforms are phosphorylated and that the most phosphorylated site in SRPP was serine, followed by threonine and tyrosine.

### Functional analysis and clustering of ERLPs in rubber latex

**Figure 6:**
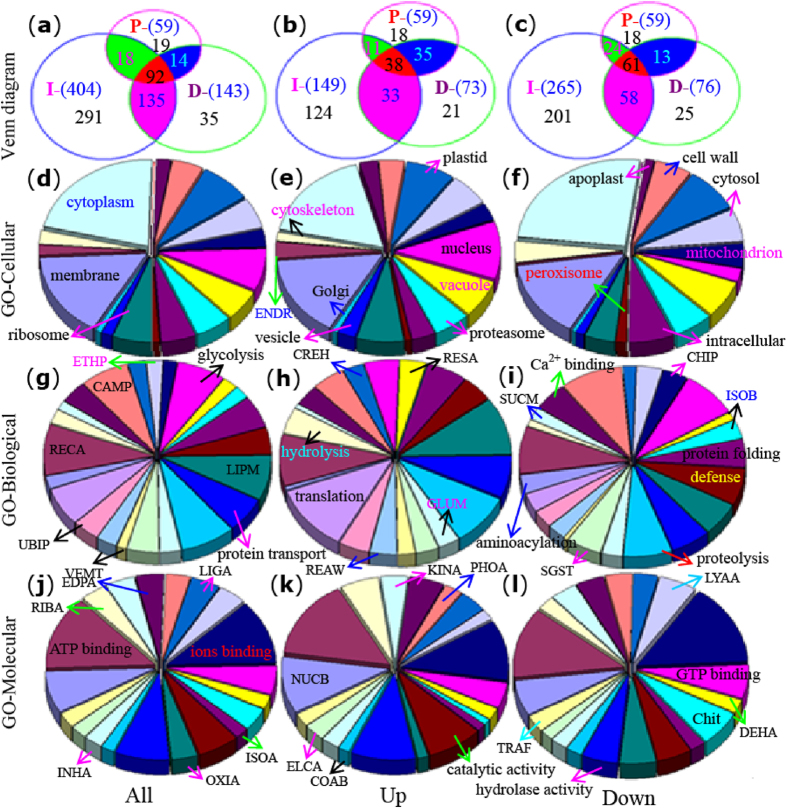
Functional classifications of the differential latex proteins upon ethylene treatment. All differentially expressed latex proteins after ethylene-treated for 3 and/or 5 days that identified by different methods, including D1-D143, P1-P59 and the 404 ERLPs from iTRAQ, are illustrated in a Venn diagram as differential proteins (**a**), up-regulated (**b**) and down-regulated (**c**) proteins. Proteins are clustered into 4 overlapping areas with numbers in different colors. Then, GO classification of all differential proteins (**d**,**g**,**j**), up-regulated (**e**,**h**,**k**), and down-regulated (**f**,**i**,**l**) proteins on cellular component (**d**–**f**), biological process (**g**–**i**) and molecular function (**j**–**l**) are presented. The abbreviations are listed in Supplemental Fig. S2.

ERLPs identified by DIGE (143 proteins, D1-D143) and iTRAQ (404 proteins) from E-3 and/or E-5 treatments, as well as the 59 differential phosphoproteins (P1-P59), were plotted in a Venn diagram, which revealed 92 shared protein spots representing 11 unique proteins (Fig. 6a–c; Fig. S5; Tables S2–S4): REF; SRPP; hevein; 14-3-3 protein; HSP70; glucanase; latex abundant protein; ELRP; Hev b7.02; phosphoglycerate kinase; and nucleoredoxin. Moreover, 135 protein species were shared between DIGE and iTRAQ (Fig. 6a). Of the up-regulated proteins, 38 were presented in all experiments and represented 4 unique proteins (REF, SRPP, Gluc and HSP70; Fig. 6b). In DIGE and iTRAQ, we identified 33 shared up-regulated protein species, including ETIF, ubiquitin, V-ATPase, glutathione-S-transferase, proteasome, aminopeptidase, esterase, HSP18 and RNA helicase. In addition, 9 unique proteins were down-regulated in all three experiments. In both DIGE and iTRAQ, 14 unique down-regulated proteins were identified, including chitinase, HMGR or HMGS, ubiquitin, proteasome, enolase, G3PD, and a 50-kDa protein (Fig. 6c; Fig. S5; Tables S3 and S4).

Next, we classified all differentially expressed proteins by COG into 16 functional groups. Among them, the largest group was the post-translational modification-related chaperones (59 protein species), followed by carbohydrate transport and metabolism (46 proteins) and energy production (Fig. S5). GO analysis of the differentially expressed proteins was performed for the categories of cellular component (Fig. 6d–f), biological process (Fig. 6g–i) and molecular function (Fig. 6j–l). For cellular component, the largest group was overrepresented in the cytoplasm (80 proteins), which is consistent with the fact that rubber latex is itself in the cytoplasm of laticifers^15^. Another large group was located in the plasma membrane (48 proteins). Forty-six proteins were located in the nucleolus, suggesting that ethylene stimulation activated gene expression (Fig. 6d). After ethylene treatment, many proteins located in the nucleolus, ribosome, apoplast and endoplasmic reticulum were induced (Fig. 6e), whereas a large group that localized to the cytoskeleton/microtubules, cytoplasm, intracellular compartments and cell wall was down-regulated (Fig. 6f).

Biological process and GO pathway analysis revealed that the largest group of ERLPs was involved in responding to cadmium ions (30 proteins). The second largest group, containing 28 proteins, was involved in carbohydrate metabolism. Some other important biological processes, such as lipid metabolism, proteolysis, glycolysis, protein transport, and translation were overrepresented (Fig. 6g). Many up-regulated proteins were involved in ATP hydrolysis, the lipid metabolic process, vesicle-mediated transport, or gene translation. However, proteins involved in isoprenoid biosynthesis (7 proteins), chitin catabolism (7 proteins) and aminoacylation were obviously decreased upon ethylene treatment (Fig. 6g; Fig. S5). Molecular function analysis demonstrated that the largest group of proteins (66 proteins) was involved in ATP binding, followed by metal ion binding (59 proteins), nucleic acid binding, hydrolase activity, phosphotransferase activity (33 proteins) and kinase activity (22 proteins). Of the induced proteins, most were involved in nucleic acid binding, ribosome activity, catalytic activity or hydrolase activity. In contrast, proteins involved in metal ion binding, aminopeptidase, GTPase, chitinase and oxidoreductase were down-regulated (Fig. 6j–l; Fig. S5).

### Integrative analysis of the proteins and genes involved in ethylene-stimulation of latex production

**Figure 7:**
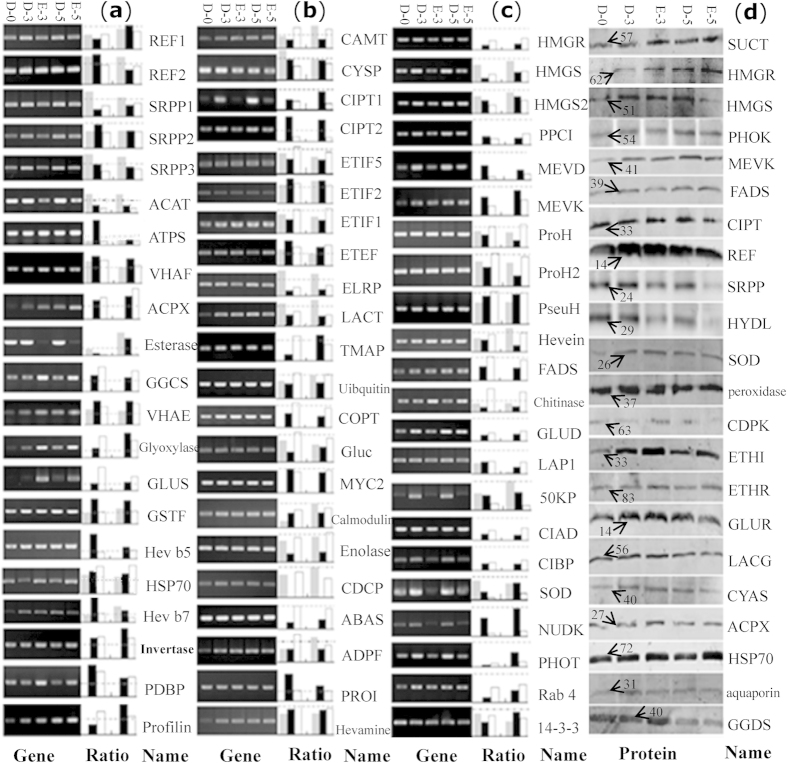
Comparison of protein and RNA patterns in ethylene-treated latex. The 65 identified proteins were assayed by RT-PCR, DIGE, and iTRAQ to compare their gene and protein expression patterns in the D-0, D-3, E-3, D-5 and E-5 plants (**a**–**c**). The actin gene from the rubber tree was used as a reference. Then, their relative changed ratios (E-3/D-3 and E-5/D-5) at both protein level (gray for data from DIGE; black for data from iTRAQ) and mRNA level (white) are presented right aside the semi-quantitative RT-PCR photographs. The dot line in each rectangle graph represents a 1.0 ratio value. The relative changed ratios at protein level over (**a**), near (**b**), and below (**c**) the 1.0 ratio line are classified into different groups. Finally, Western blotting for the 22 latex proteins was performed to determine the general protein accumulation in D-0, D-3, E-3, D-5 and E-5 plants (**d**). The approximately molecular weight (kDa) of the target bands is indicated with an arrow. Abbreviations, antibody information, and primers used for RT-PCR are listed in Supplemental Fig. S2, Fig. S6, and Fig. S7, respectively.

To further evaluate the correlation between the protein and transcript abundance, 65 ERLPs were selected for RT-PCR to assess gene expression patterns. The results demonstrated that the expression patterns of most detected proteins and their genes changed similarly upon ethylene treatment. Compared with H_2_O treated plants (D-3 and -5), only 13 genes were induced by ethylene (E-3 and -5), while 27 genes were inhibited (Fig. 7a–c). The other 25 transcripts either did not change or changed less than the level of their encoded proteins upon ethylene treatment. Among the down-regulated genes, were several enzymes known to be key factors in rubber biosynthesis, including HMGR, HMGS, MEVD, MEVK, REF, SRPP, FADS and CIPT (Fig. 7a–c).

Thirty-one proteins were significantly up-regulated upon ethylene treatment, including REF2, SRPP1, SRPP3, acetyltransferase, ATP synthase, CIPT 2, invertase, methyltransferase, glutamine synthetase, and HSP 70. In contrast, 34 protein species were down-regulated following E-3 and/or E-5 treatments, including REF1, SRPP2, CIPT 1, HMGR, HMGS, MEVD, MEVK, hevein, chitinase, Gluc, and enolase (Fig. 7a–c). These proteins are mainly involved in rubber biosynthesis and carbohydrate metabolism. We noticed that different protein isoforms, such as those of REF, SRPP and CIPT, showed different patterns upon ethylene treatment, indicating that different protein isoforms may play different roles in rubber biosynthesis.

Furthermore, 22 proteins known to be involved in rubber biosynthesis and ethylene response were assayed by Western blotting to determine their accumulation patterns (Fig. 7d). Compared with untreated plants, almost all proteins were induced after both water (mainly for wounding) and ethylene treatments except for HYDL, LACG and GGDS. For longer time periods, most induced proteins, including HMGS, REF, SRPP, ETHI, GLUR, SOD and peroxidase, decreased to some extent. However, some proteins, including PHOK, MEVK and HSP70, were most abundant in the 5-day-treated latex. Although most proteins were induced after tapping, ethylene stimulation did not enhance this trend. Compared with water, ethylene obviously improved the expression of some proteins following a 3-day treatment, including SUCT, MEVK, CIST, SOD, CDPK, ETHI, ETHR, CYAS, ACPX, aquaporin, GGDS and HSP70. However, protein and cognate transcript abundance for most rubber biosynthesis enzymes, including HMGR, HMGS, FADS and SRPP, were not significantly improved upon treatment with exogenous ethylene (Fig. 7), similar to observations made in our iTRAQ experiment (Fig. 3; Fig. S4).

**Figure 8:**
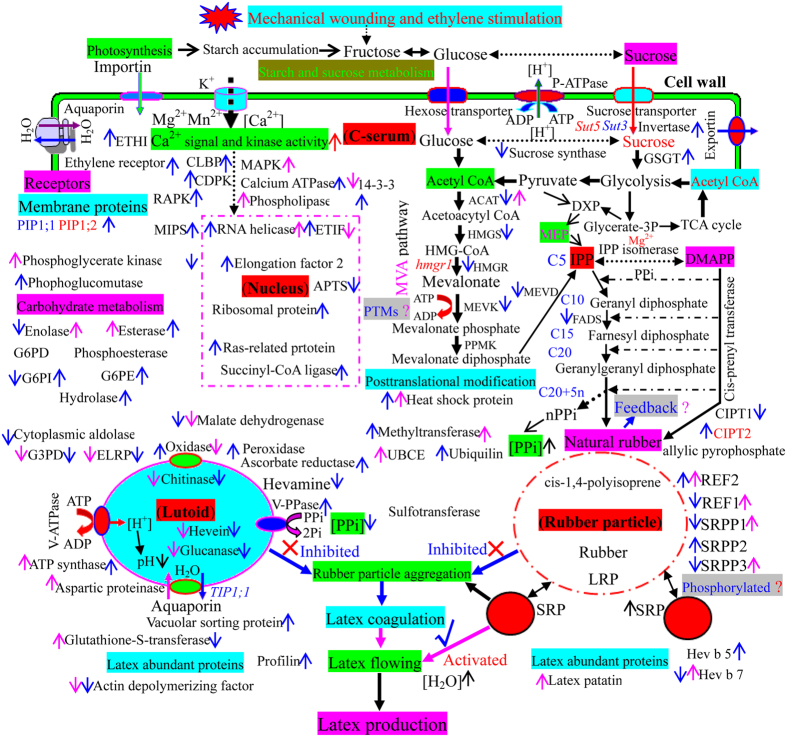
Schematic of enzyme localization and regulation of ethylene-induced pathways at the proteomic level in rubber latex cells. The ethylene-responsive proteins in rubber latex were localized in the C-serum, nucleus, lutoid and rubber particle based on GO analysis and their reported functions. The main biochemical processes and ethylene-responsive pathways are highlighted in different colors. Proteins activated (up) or inhibited (down) by ethylene are marked with arrows in different colors (pink, from DIGE; blue, from iTRAQ and/or Western blotting). Abbreviations are listed in Supplemental Fig. S2.

Given our in-depth proteomics analysis of rubber latex following ethylene treatment and the recent findings reported in the literatures, we have summarized the putative subcellular localization and main function of the identified proteins and have proposed a schematic for their regulation (Fig. 8). These results revealed that ethylene stimulation of rubber latex production might act neither at the level of gene expression nor on general protein accumulation. Our data point to the post-translational modification of several key enzymes and the involvement of carbohydrate metabolic process-related proteins as playing crucial roles in controlling natural rubber biosynthesis and latex production.

## Discussion

### In-depth proteomics analysis deepened our understanding of the main biological functions of rubber latex

Latex is composed of various macromolecules, including proteins, starches, sugars, tannins, resins, and alkaloids. Among them, proteins are key factors in rubber biosynthesis^20^. Using omics-based technologies, Cho *et al.* achieved a large-scale identification of the proteins involved in this process^21^. Proteomics analysis has been widely used as a powerful and straightforward tool for protein discovery and characterization of the latex of *H. brasiliensis*^19^,^21^,^22^,^23^, *Calotropis procera*^24^,^25^, *Chelidonium majus*^26^, *Lactuca sativa*^27^, and *Taraxacum brevicorniculatum*^28^. Recently, the analysis of 1,208 latex proteins from 20 plants demonstrated that these proteins have various biological functions including transcription, translation, protein degradation and response to environmental stimuli^21^.

Several rubber latex proteomics studies have been performed by traditional 1-DE and 2-DE methods^20^,^29^. In a pioneer proteomics analysis work, Martin found both the pellet fraction and cleared cytoplasm of rubber latex contained only a few proteins on 2-DE gels^30^. By the mid-1990s, latex allergens named Hev b9 (enolase) and Hev b10 (Mn SOD) had been identified^1^. Proteomics analysis of tapping panel dryness (TPD) barks produced 5 specific proteins, including an ethylene-inhibited 26-kDa protein^31^. Comparative proteomics analysis of rubber particles^18^,^32^, total latex^23^, C-serum^33^, lutoids^19^ and seeds^22^ were conducted in *Hevea* latex, but this analysis was somewhat low quality due to streaking on the 2-DE gels as well as the limited number of identified proteins, making this dataset a less comprehensive overview of the latex proteome^19^,^20^,^33^. To improve latex proteomics study, we developed a protocol for latex protein preparation and generated high-resolution 2-DE protein profiles^33^. Then, we identified 169 lutoid protein species and found that combination of chitinase and glucanase is crucial for lutoid-mediated RPA and latex coagulation^19^.

In this work, we provide the first high-resolution reference 2-DE profiles of rubber latex and identified almost all abundant latex proteins on 2-DE gels by MS. From the gels, we obtained 287 protein species, including 184 unique proteins (Fig. 2; Fig. S1; Table S1). By iTRAQ, we identified more than 1,600 proteins (Fig. S4; Table S3). In the past decade, only 313 proteins in total have been identified in the rubber tree, and among all rubber-producing plants, the number of proteins has only reached 1,208^20^. Of these, a large portion (803 proteins) were identified in lettuce latex by tandem MS^20^,^27^. Compared with previously reported work, this study has generated the most latex proteins and provided the best protein profiles for rubber latex (Fig. 2; Fig. S1). Among the identified rubber latex proteins, REF, known as Hev b1, facilitates the elongation of the CIPT molecule to the isoprene subunit^34^. It is tightly bound to rubber particles and coats the interface between aqueous serum and polyisoprene molecules. REF, with its highly charged N-terminus, comprises 10% to 60% of the total protein found in latex^34^. Our 2-DE results demonstrated that at least 17.2% of the protein found in latex was REF (Fig. 2; Fig. S1). In iTRAQ, 553 peptides were generated from REF (Fig. S4). Hevein, glucanase and chitinase are the three main proteins in lutoids^19^. Our results revealed that these proteins were enriched in total latex (Fig. 2; Fig. S1; Table S1). Functional analysis indicated that most latex proteins were involved in post-translational modification, carbohydrate metabolism, and energy production. These proteins were mainly located in the cytoplasm and play crucial roles in ATP binding, metal ion binding, abiotic stress responses, glycolysis, and catalysis (Fig. S2). These results are consistent with the observations of latex transcriptomics experiments^13^,^35^ and rubber genome analysis^36^,^37^. Notably, cellular location revealed that 10 proteins were significantly overrepresented in the phosphopyruvate hydratase complex (Fig. S2e; Fig. S1), indicating the importance of protein phosphorylation in rubber latex.

### Ethylene improved carbohydrate metabolism and energy production but inhibited RPA

Glutamine synthetase is highly abundant in the latex of rubber tree^38^ and lettuce^27^. It is a key enzyme involved in nitrogen assimilation that catalyzes the synthesis of glutamine, which is required for latex regeneration, from ammonium and glutamate^38^. In rubber tree, the activities of several enzymes, including glutamine synthetase^38^ and chitinase^6^, were specifically modulated upon ethylene treatment. In particular, ethylene increased invertase activity, which resulted in glycolysis acceleration, and finally increased the supply of carbon sources for rubber biosynthesis^39^. Sucrose, as a unique precursor of carbon sources used in rubber biosynthesis, is converted to mevalonate through an intermediate of acetyl-CoA in a NAD(P)H-dependent manner, but must cross the lactifer plasma membrane with the help of a sucrose transporter before being metabolized^35^. Accordingly, enzymes involved in carbohydrate metabolism were activated, and the gene expression of several sucrose transporters in *Hevea* bark tissues and latex-producing cells were induced by ethylene^35^. Several aquaporins were also up-regulated upon ethylene stimulation through the regulation of water exchange between the inner liber and latex cells^9^. Therefore, ethylene altered membrane permeability, prolonged latex flowing time, and as a feedback, finally regenerated the basic metabolism of rubber latex^6^.

In this proteomics analysis, we obtained 222 ethylene-induced proteins, many of which were involved in carbohydrate metabolism and energy production (Fig. 6). In DIGE gels, MAPK was markedly induced (Fig. S3). MAPK plays a role in ethylene signaling by controlling phosphatase activity to improve root cell expansion and is known to induce ethylene biosynthesis in *Arabidopsis*^40^. In the rubber tree, MAPK expression was induced upon jasmonate and ethylene stimulation^41^. The onset of TPD syndrome is triggered by MAPK which regulated MYB transcription factor to induce programmed cell death in rubber tree bark^42^. In addition, aspartic proteinase precursor increased 1.75 fold after treatment with ethylene for 3 days; however, when the treatment was extended to 5 days, its expression was substantially inhibited (Fig. S3; Table S2). This laticifer protein was also detected by 2-DE in *C. procera* latex^24^, and it is known to play a defensive role in rubber latex^25^. Enolase, also called 2-phosphoglycerate dehydratase or 2-phospho D-glycerate hydrolase, is ubiquitous in rubber-producing plants^20^ and in many living organisms^1^. In model plants, phosphoenolpyruvate enolase is associated with an abnormal phenotype^20^. This enzyme is a key player in glycolysis and gluconeogenesis in the carbohydrate metabolism pathway, where it catalyzes the reversible conversion of 2-phosphoglyceric acid to phosphoenolpyruvic acid^1^. This enzyme was detected in the C-serum of rubber latex and was identified as Hev b9 in a proteomics analysis of rubber latex^43^. It was also enriched in LRPs^18^. Herein, we detected two induced enolase isoforms (D-6 and D-33) and one decreased isoform (Enolase 1, D-108) after ethylene stimulation. In iTRAQ, this enzyme was generally down-regulated upon the addition of ethylene. Similarly, its gene expression was inhibited by ethylene (Fig. 7; Fig. S4).

In DIGE, a CAMT isoform (D-62) was induced by ethylene. CAMT is important for lignification^29^ and is induced upon ethylene and jasmonate stimulation in *Arabidopsis*^44^. In addition, CAMT is highly expressed in a high-yielding rubber tree clone^35^. However, this enzyme was generally decreased in our iTRAQ experiment (Fig. S4). In the 5-day-treated latex, esterase (Hev b13), an enzyme similar to hydroxynitrile lyase that is involved in plant defense, was obviously increased. Proteasome and Hev b7.02 were also induced. However, the levels of these proteins were low in 3-day-treated latex (Fig. S4), and the esterase gene was inhibited by ethylene treatment (Fig. 7). In iTRAQ, the most increased protein was hydrolase (31.3 fold), which hydrolyzes O-glycosyl compounds and is involved in many processing events in the vegetative vacuoles of plant cells, ranging from general metabolism to defense^45^. Hydrolase can also help to release ammonia from nitrogenous substrates under ethylene treatment through the modification of the membrane properties of glutamine synthetase^38^. Here, we noticed that all hydrolase family members were significantly increased upon ethylene treatment (Fig. S4). This protein was enriched in the latex of plants attacked by butterflies^25^ and in the self-rooting juvenile clones of the rubber tree^23^, and its gene was highly expressed in the latex of a high-yielding rubber tree clone^35^. In addition, a potassium channel protein (I-713) was induced upon ethylene treatment (Fig. S4; Table S3). This protein, in addition to V-ATPase, aids in the uptake of sugar and sucrose by facilitating their transfer across the plasma membrane in laticifer protoplast^46^.

As an early response to ethylene, the total amount of adenine nucleotides (i.e., ATP and ADP) in the adenylate pool, as well as the transtonoplast ΔpH within *Hevea* latex cell, are increased^15^. Similarly, the activity of tonoplast H^+^-pumping ATPase in rubber latex is significantly enhanced to improve vacuolar acidification upon ethylene treatment^12^,^15^. We observed that V-ATPase, an ATP-driven enzyme that transforms the energy derived from ATP hydrolysis^46^, was significantly induced upon ethylene. In rubber latex, this enzyme catalyzes vacuolar acidification and controls the availability of its substrate ATP and then activates energy-dependent metabolism during rubber latex production following ethylene stimulation^12^. A mitochondrial ATP synthase (I-188) was also induced by ethylene. This enzyme, together with other plasma-type ATPases (P-ATPases), is crucial for ethylene sensing *via* copper transport^47^.

Many of the induced proteins identified in this study are involved in ATP binding, ATP hydrolysis and energy production. Among them, CDPK5 was increased after 3 days of ethylene treatment (Fig. 7; Fig. S4; Table S3). It appears likely that CDPK, as well as MAPK, play a role in the ethylene signaling pathway by controlling phosphatase activity^48^ and sucrose metabolism^2^.

In the 402 down-regulated latex proteins, most enzymes, including prohevein/hevein, chitinase/hevamine and glucanase, were involved in chitin catabolism. These are key factors in lutoids that regulate RPA and latex production after tapping^19^. Our results demonstrated that almost all the investigated isoforms of these proteins were significantly inhibited upon ethylene treatment (Fig. 6; Fig. S4). Generally, hevein, chitinase and glucanase are the main three components of lutoid inclusions^3^. As defense-responsive proteins, they possess antimicrobial activity that is triggered in response to the presence of bacteria, insects, or fungi, and act to protect the cell or organism^19^,^30^. In a previous proteomics analysis, we confirmed that these three proteins are present at high concentrations in lutoids^19^. Chitinase, like hevamine, is a well-known plant defense protein that possesses lysozyme activity. It belongs to the chitinase class II subfamily of glycosyl hydrolases. A major difference between hevamine and chitinase is that the former is a basic protein localized in vacuoles, while the latter is an acidic extracellular one^19^. An increase in chitinase activity results in a dramatic decrease in the detection of Glc-NAc on the 22-kDa hevein receptor, indicating a possible role for chitinase in removing sugar moieties^49^. These lines of evidence supported a role for chitinase in plugging the latex vessel and in stopping latex flow^19^. Glucanase (Hev b2) is enriched in the lutoid membrane and possesses hydrolase activity. It is a basic vacuolar glycoprotein that tightly interacts with rubber particle glycoproteins, such as SRPP and REF^49^. This interaction promotes the rapid aggregation of rubber particles, resulting in latex coagulation when lutoids burst upon tapping of the rubber tree^19^. Hevein, or its precursor pro-hevein, is suggested to be the most abundant lutoid protein^50^. Both hevein and pro-hevein showed a strong chitin binding ability^19^,^50^. Immuno-blotting analysis of hevein revealed both mature hevein and a 20-kDa protein recognized by N domain-specific antibody^50^. Hevein can also stimulate RPA^19^,^49^.

Recently, based on a lutoid proteomics study, we proposed a new model for lutoid-mediated RPA. After tapping or other injury, rubber latex flows outside laticifers and the subsequent turgor pressure changes suddenly, resulting in the bursting of lutoids, which allows contact between chitinase and glucanase. When glucanase (an RPA activator) encounters the inhibitor chitinase in broken lutoids, rubber particles aggregate immediately and latex coagulates are formed, thus plugging latex vessel ends and inhibiting sustained latex flow^19^. Therefore, these three proteins in lutoids were considered to cause the negatively charged RPA and latex to coagulate in a rather narrow range of ratios of added proteins to rubber latex^19^. In this study, we found that ethylene treatment substantially inhibited the gene expression of hevein, chitinase and glucanase and, correspondingly, decreased their protein accumulation. The decrease in RPA-related enzymes, as well as the up-regulation of certain water and compound transportation proteins, such as aquaporin, potassium channel, exportin-1, invertase 2, and trafficking protein (Fig. 8), helped to inhibit RPA and prolonged the flow time of fresh latex, thus increasing the production of latex. We considered this process to be an important factor for ethylene-stimulated production of rubber latex.

### Ethylene induced many nucleotide-binding and translation-related proteins but generally depressed rubber biosynthesis enzymes

Several reports have uncovered that the genes for certain regulated proteins, such as WRKY transcription factor 1^51^, 14-3-3 protein^52^, metallothionein^53^, and ethylene-responsive factor^41^,^44^ and peroxidase^49^, responded positively to ethylene stimulation. Notably, some protein kinases, including CDPK^2^,^53^, MAPK^41^,^47^, serine/threonine protein kinase and CBL-interacting protein kinase^41^, were significantly induced in rubber latex by exogenous ethylene. Here, we found that 24 nucleic acid binding proteins were induced by ethylene, most of which are known to be nucleolus or nuclear envelope-binding proteins. Among them, the argonaute protein can bind a mature miRNA to guide a protein complex to target messages for post-transcriptional silencing^41^. In the rubber tree, this protein showed transcription activity in response to stimulation or TPD^54^. ETIF is controlled by deoxyhypusine synthase to activate protein post-translational modification^27^, and it is involved in the regulation of cell proliferation^13^. In the rubber tree, this protein plays a role not only in protein elongation and quality control but also in various stress responses^18^. ETIF is enriched in latex^35^,^42^ and SRPs^18^,^32^, and its gene is induced by TPD^13^. These results verified that ethylene could activate the expression of many genes encoding nuclear proteins in the rubber tree.

Of the many genes and proteins involved in rubber biosynthesis that were induced by tapping or mechanical wounding, most were inhibited by ethylene treatment (Figs 7 and 8). The mevalonate (MVA) pathway is the most-studied cytosolic pathway of rubber biosynthesis. Only in recent years has the plastidic methyl-D-erythritol 4-phosphate (MEP) pathway been considered a possible route for rubber biosynthesis by virtue of providing an alternative source of isopentenyl diphosphate^37^. Although the expression of 1-deoxy-D-xylulose 5-phosphate synthase (DXPS) has been reported in *Hevea* latex and leaves^37^, the main enzymes in the MEP pathway were not detected in this study. Only G3PD, which acts at the first step of MEP pathway, was identified, but it decreased after ethylene stimulation (Fig. S4), indicating that the MEP pathway is not crucial for rubber biosynthesis. In MVA pathway, CIPT^17^,^55^, HMGR^42^,^54^, HMGS^5^,^56^, FADS^8^,^56^, acetyl-CoA acetyltransferase, MEVD, MEVK, REF and SRPP are the key factors^5^,^56^. These proteins are closely related to the yield and quality of natural rubber^5^. CIPT, commonly called rubber transferase, localizes to the rubber particle membrane^32^. It produces rubber polymer (cis-1,4-polyisoprene) from IPP monomers, which it links in a chain-like manner to an allylic pyrophosphate (APP) initiator molecule. The rubber polymer grows as more isoprene units are added to the chain^4^,^19^. Rubber transferase, activated by the metal ion cofactor Mg^2+^ or Mn^2+^, is a key factor that determines the rubber-producing ability of rubber trees^6^,^57^. In addition, the activity of rubber transferase in SRPs is higher than in LRPs. It has also been reported that SRPs are composed of linear rubber molecules void of chain-end groups for forming branch points, while LRPs contain branched rubber molecules^19^. These results, as well as our observations (Fig. 1e–g), revealed that rubber transferase contained in SRPs may play additional important roles in ethylene-induced latex production. At the gene level, our observations, together with previously the reported results^2^, verified that CIPT1 and CIPT2 were repressed by ethylene. However, our results presented here show that ethylene significantly improved CIPT2 abundance in rubber latex (Fig. 7).

In rubber tree, HMGR and HMGS are involved in early steps of rubber biosynthesis and demonstrate a positive response to IPP substrate. HMGR, a key regulatory enzyme, provides a backbone by catalyzing mevalonate synthesis from HMG-CoA for isoprenoids^42^. Four HMGR (*hmgr1-4*) and two HMGS gene family members have been reported in rubber latex^5^. Among them, *hmgr1*, an ethylene induced-gene, is responsible for rubber biosynthesis^10^,^42^. However, ethylene did not influence the activity of HMGR^10^. It was reported in a recent study that ethylene influenced HMGS gene expression and improved its enzymatic activity, which was the first observation of the accelerative effect of ethylene on rubber biosynthesis^2^,^11^. Interestingly, we found the both gene and protein expression of HMGR and HMGS were inhibited by ethylene stimulation in this study (Figs 7 and 8). ACAT catalyzes a Claisen-type condensation of two acetyl-CoA units to form acetoacetyl-CoA, which is recognized as the first step in the MVA pathway^56^. In rubber tree, there are three ACAT genes^5^. Our results revealed that although both the gene and protein expression of most ACAT isoforms was depressed, one isoform was induced by ethylene. Two other important enzymes in the MVA pathway, mevalonate disphosphate decarboxylase (MEVD) and mevalonate kinase (MEVK), were inhibited by ethylene at both the gene and protein levels (Fig. 7). During rubber elongation, REF and SRPP are key proteins^5^. Our results revealed that although some family member proteins were markedly induced, REF and SRPP were not obviously changed at the gene or protein level upon ethylene treatment. This result is consistent with a report that neither ethylene treatment nor wounding changed the transcript level of the SRPP gene in *Hevea*^58^. This study revealed that ethylene could not directly induce most of the key enzymes in the MVA pathway at either the gene or protein expression level during rubber biosynthesis and that its stimulating effect was dependent on the activation of a small number of enzymes.

### Phosphorylation of specific isoforms of rubber biosynthesis-related enzymes might be important for ethylene-stimulated latex production

During ethylene signal transduction, MAPK cascades often regulate transcription directly by phosphorylating transcription factors. Two-component regulators are typically composed of a sensor protein with an input domain that receives signals, and a catalytic transmitter domain that autophosphorylates a conserved internal histidine residue^47^,^59^. MAPK3 and MAPK6 can influence the stability of the protein ethylene insensitive 3 (EIN3) by phosphorylation, and its level is regulated by ubiquitination and proteasome degradation^48^,^59^. In cotton, ethylene mediates the defense response to pathogen infection and oxidative stress by dephosphorylating MAPK^59^, and in *T. brevicornicula* latex, phosphorylation of the leucine zipper transcription factor contributes to plant stress tolerance^60^. Several serine/threonine kinases were identified in *C. majus* latex by proteomics analysis that phosphorylate several stress-induced proteins and are involved in cell viability^26^.

A proteomics study of lettuce latex identified 14 proteins that contributed to main complexes involved in oxidative phosphorylation in mitochondria^27^. Oxidative phosphorylation is the most important part of metabolic pathways, as it produces ATP, hydrogen peroxide and superoxide^27^. In rubber biosynthesis, the monomer IPP is phosphorylated to form an enzyme-IPP adduct, which is able to repeat and sustain IPP addition by the simultaneous loss of pyrophosphoric acid^4^. It is known that 14-3-3 isoforms can bind to phosphorylated client proteins to modulate their function, and the selective phosphorylation of 14-3-3 proteins at several known sites might be important for rubber biosynthesis^52^. In rubber latex, ethylene treatment improved ATP hydrolysis to generate ADP and AMP, followed by the rapid dephosphorylation of AMP, thus reducing the total adenine nucleotide pool^15^. In a proteomics analysis of rubber latex, 4 proteins were found in the combinatorial peptide ligand libraries that are known to be involved in phosphorylation processes^61^. HMGR1, a key rubber biosynthesis enzyme, was predicted to be phosphorylated at tyrosine and serine residues^42^.

Our comparative proteomics study identified many phosphoproteins with kinase activity (Figures S1–S4). Among them, REF and SRPP are two major components present on the surface of rubber particles that synthesize long-chain polyisoprene^34^,^62^. Genes encoding REF and SRPP have been cloned, and various isoforms have been identified. In addition, tapping is known to stimulate their gene expression. REF expression positively correlates with latex yield, and the amount of REF in latex is proportional to its rubber content. REF and SRPP are acidic proteins and are not believed be post-translationally modified in *Hevea* latex^1^,^62^. However, SRPP was recently suggested as a glycoprotein with amyloid properties likely to interact with lutoid lectin and susceptible to play a role in latex coagulation^49^,^62^. In our phosphoproteomics study, we found that at least 4 isoforms of REF and 7 SRPP family members were phosphorylated and that SRPP was phosphorylated at a serine residue (Fig. 5; Fig. S5; Table S4).

Based on the aforementioned comprehensive proteomics analysis and the recent literatures^4^,^5^,^37^,^63^, we proposed an intrinsically cellular-based mechanism associated with both the detailed localizations and the response mechanisms of main latex proteins after ethylene stimulation, and then suggested a new scheme of the ethylene-induced biochemical pathways in rubber latex cells at the protein level (Fig. 8). Similar to previous gene expression analyses^2^,^63^, our proteomics data revealed that mechanical wounding and ethylene stimulation induced many biochemical processes in laticifers, such as starch and sucrose metabolism, sucrose and glucose loading, water uptake and transport, nitrogen assimilation, carbohydrate transport and metabolism, hydrolase activity, metal ion binding, ATP binding, energy production, Ca^2+^ signal and kinase activity, and post-translational modification (Fig. 8). In contrast, the enzymes involved in RPA and latex coagulation were markedly inhibited upon ethylene stimulation, which depressed the formation of rubber coagulates in the vessel and subsequently prolonged the latex flow time. Natural rubber biosynthesis might be enhanced *via* a feedback mechanism; some isoforms of key rubber biosynthesis enzymes such as REF, SRPP and CIPT2 were activated by phosphorylation. Ethylene also increased the generation of SRPs, which are more important for rubber biosynthesis than LRPs. Finally, the natural rubber latex yield was significantly improved after treatment with exogenous ethylene. To our knowledge, this is the first in-depth quantitative proteomics study of rubber latex upon ethylene stimulation, and the first report proposing a role for the phosphorylation of REF and SRPP. We proposed that phosphorylation of specific isoforms of several key enzymes might have a positive correlation between phosphoprotein abundance and the ethylene-stimulation of rubber latex production.

## Materials and Methods

### Plant material and ethylene treatment

A total of 90 newly tapped mature (∼8-year-old) rubber trees (*H. Brasiliensis* Mull. Arg., clone RY 7-33-97) never before treated with ethylene were selected and randomized into six groups. These plants were grown at an experimental farm of the Chinese Academy of Tropical Agricultural Sciences in Danzhou City, Hainan Province, China. First, these virgin plants were tapped to collect latex for a negative control. On the same day, ethylene treatments in the form of 3% (V/V) ethephon dissolved in ddH_2_O were applied on the tapping panel as described^16^. For the control, an equal volume of ddH_2_O was used. Three days later, these plants were tapped again to collect fresh latex. After additional 2 days, these plants were tapped a third time. The latex samples collected from the plants treated with ddH_2_O (control) and 3% ethephon at days 0, 3 and 5 were termed D-0, D-3, D-5, E-3, and E-5, respectively. Three biological replicates were performed for each sample. After tapping, the first 10 drops were discarded. Subsequent latex drops were collected in ice-chilled glass beakers and taken for analysis.

### Determination of latex fresh weight, dry weight, and total water content

The latex fresh weight (FW) was determined immediately after tapping. The flowing time was calculated starting with the first latex drop, assuming a flow rate ≥1 drop/5 sec. The dry weight (DW) was determined after drying the fresh latex for 72 h in an oven at 60 °C. The total water content (TWC) was calculated as follows: TWC = [(FW − DW)/FW] × 100.

### SEM analysis of rubber particles

Rubber particles were isolated and purified as described^33^, and the purified rubber particles were examined under a scanning electron microscope (SEM) as described previously^17^,^18^. In brief, the collected rubber particles were suspended in a fixative solution (50 mM sodium dimethyl arsenic, 1% w/v tannic acid, 6% v/v glutaraldehyde) for 2 h at room temperature, then collected through centrifugation, washed three times with ddH_2_O, and further fixed with 1% w/v osmium tetroxide for 1 h at room temperature. These rubber particles were then washed twice with ddH_2_O and resuspended in an appropriate dilution. A small drop of the suspension was placed on a piece of tin foil and air dried. Samples were mounted on aluminum stubs, coated with gold particles, and examined under an S-3000 SEM (Hitachi, Japan).

### Protein extraction, 2-DE and 2-D DIGE analysis

Latex proteins were extracted as described^33^. Protein concentration was determined by Bradford assay with BSA as a standard. For common 2-DE, 1,000 μg of proteins were loaded onto the 24-cm, pH 4-7 linear gradient IPG strips (GE Healthcare, Uppsala, Sweden), and then, isoelectric focusing and gel electrophoresised were performed as described^19^,^64^. Three biological replicates for each separation were conducted.

2-D DIGE analysis was performed as described^64^. Proteins from the D-3, E-3, D-5 and E-5 latex samples were labeled with a ratio of 400 pmol Cy3 or Cy5 per 50 μg of proteins. For gel normalization, an internal standard was prepared by pooling an equal protein quantity from each of the four samples and labeling with Cy2. Then, the Cy2-, Cy3- and Cy5-labeled 2-DE images were acquired using a Typhoon Trio scanner (GE Healthcare, Piscataway, NJ, USA), and the DIGE images were analyzed using DeCyder 7.0 software (GE Healthcare). A differential in-gel analysis module was used for spot detection, and a biological variation analysis module was applied to the three biological repeats to identify the differentially expressed spots with higher than 95% confidence.

### Phosphoproteomics analysis

Approximately 1,000 μg of latex protein from the ethylene-treated for 3 days (E-3) rubber trees were subjected to 2-DE as described^19^,^33^, then, the 2-DE gels were stained with Pro-Q Diamond dye to detect phosphoproteins according to the manufacturer’s instructions (Molecular Probes, Eugene, USA). The gels were then restained with SYPRO Ruby fluorescence stain. Finally, the same gels were restained with Coomassie blue dye G-250 as described^64^. The images of these 2-DE gels stained by Pro-Q, SYPRO Ruby and G-250 were analyzed with ImageMaster software (GE Healthcare, USA). After spot detection and background subtraction, the 2-DE gels were aligned and matched, and the spot volumes were determined. Each of the gels has a Sypro Ruby (total protein) image plus a Pro-Q Diamond (phosphoprotein) image; the total protein images were selected as the internal standards. The individual Sypro Ruby spots from each image were normalized to the total Sypro Ruby spot volume of the image, while the Pro-Q Diamond images were normalized to a corresponding internal standard image. The spots with 2-fold change at least one treatment in normalized spot density with p-values less than 0.05 in all three biological replicates were considered as differentially regulated phosphoproteins (Table S4). The spots of interest were excised from the G-250 stained gels for subsequent MS analysis.

### Protein identification *via* mass spectrometry

The target protein spots were manually excised and in-gel digested with bovine trypsin as described^33^. Mass spectra of the peptides were acquired on an AB 5800 MALDI-TOF/TOF mass spectrometry (MS) instrument (AB SCIEX, Foster City, USA) as described^19^,^64^. The measured tryptic peptide masses were transferred to ProteinPilot Software (Version 4.5) and used for a Mascot Algorithm (version 2.3) search of the taxonomy of Viridiplantae (Green Plants, including 1,749,470 sequences) in the nonredundant NCBI (NCBInr) database (Released version NCBInr 20140323). Spots were considered identified with a Mascot threshold score of ≥75 and of 46 for the MS and MS/MS results, respectively. Furthermore, the remained unidentified proteins were subjected to nano-LC MALDI TOF/TOF MS. The samples were separated using a Pepmap C18 column (Dionex, USA) following a 30 min 5–35% organic gradient and spotted onto target plates using a Tempo spotting system (AB Sciex, USA). The tandem MS mode was used on a TOF/TOF 5800 analyzer. In addition, to determine the detail phosphorylated amino acid sites, the collected peptides from spot P6 were subjected to an LTQ Orbitrap Exactive mass spectrometer (Thermo Fisher Scientific), and the phosphospectra were extracted from Xcalibur software (version 2.2) in the data-dependent acquisition mode (Fig. S4; Table S3). All MS/MS data were combined to search the NCBI database. To avoid false positives, the identified proteins were subjected to an in-house BLAST search at NCBI (http://www.ncbi.nlm.) to confirm the matches.

### Protein labeling and quantification for iTRAQ analysis

Three separate iTRAQ (isotope tags for relative and absolute quantification) experiments were performed on the Triple TOF 5600 system (AB SCIEX) coupled with an Ultra 2D Plus HPLC with a Nanoflex microchip system (Eksigent, Dublin, USA). Approximately 100 μg of proteins were digested with trypsin, then, the vacuum dried peptides from latex proteins of D-3, E-3, D-5 and E-5 samples were respectively labeled with iTRAQ tags 114, 115, 116 and 117 Da, respectively. Strong cation exchange chromatography was performed for the combined iTRAQ-labeled peptides. The eluted fraction was desalted using Sep-Pak C18 cartridges (Waters), dried and then reconstituted for Nanoflow LC-MS/MS analysis.

The MS analysis was performed in Information Dependent Mode. Tandem MS was recorded in high sensitivity mode with rolling collision energy on and with iTRAQ reagent collision energy adjustment. Protein identification from green plants in the Swiss-Prot Database and relative iTRAQ quantification were performed with ProteinPilot™ Software 4.5 (AB SCIEX). For iTRAQ quantification, the peptide was automatically selected by the Pro GroupTM algorithm to calculate the reporter peak area, error factor (EF) and *p* value. A reverse database search strategy was adopted to estimate the FDR for peptide identification. A strict unused confidence score of >1.3, which corresponds to at least a peptide confidence level of 95%, was used as the qualification criterion. Identified proteins with at least two matched peptides higher than 95% confidence and an FDR value ≤1% were used to perform protein quantification. Subsequently, lists of proteins with at least a 1.5-fold change were finalized for the three biological replicates. Furthermore, rubber genome scaffolds from the BioProject ID: PRJNA80191 (www.ncbi.nlm.nih.gov/nuccore/448814761) and the draft genome (GenBank: AJJZ01000000) from the rubber tree^36^,^37^ were used for validating the protein identification.

### Protein classification and hierarchical cluster analysis

The identified proteins were searched against the UniProt database to confirm their functions. Proteins were then classified by using Functional Catalogue software to obtain their corresponding COG codes. These proteins were further analyzed by TargetP to predict their subcellular location. Then, GO pathway analysis was performed by Blast-2-GO software^19^,^64^. Finally, an in-house BLAST searching at UniProtKB was performed for each protein to find its homology and then to confirm its detailed cellular component, biological process and molecular function.

### RT-PCR and Western blotting analysis

RNA from the collected latex was used to generate cDNA as described^35^, which was used in RT-PCR reactions designed to assay target gene expression in latex after D-0, D-3, D-5, E-3 and E-5 treatments. The experiments were repeated at least three times. Approximately 1 μg of RNA was used for reverse transcription. The cDNA samples were diluted to 5–8 ng/μL. The *Hevea* actin gene (NCBI accession No. JF775488.1) was used as an internal control. The primers are provided in supplementary Fig. S6.

Western blotting was performed as described^19^. Approximately 10 μg of proteins were loaded per lane and 5% nonfat milk was used as a blocking protein, after which, the nitrocellulose membranes were incubated with the relevant polyclonal rabbit antibodies. The Peptide-Antigen Finder software (China Peptide Corp., Beijing, China) was used to design the 22 oligopeptides for immunizing rabbits to produce specific polyclonal antibodies by Beijing Genomics Institution (BGI, Beijing, China). Before Western blotting, the antibodies were purified using the respective oligopeptide as the affinity column tag. Total latex proteins extracted from the D-0, D-3, E-3, D-5 and E-5 plants were performed Western blotting. The antibody tag sequence and protein information are lisited in Supplemental Fig. S6. Bound rabbit IgG was detected in separate blots with alkaline phosphatase-conjugated anti-rabbit IgG. For validation of protein expression levels from iTRAQ and DIGE experiments, we performed 3 biological replicates for all Western blotting.

## Additional Information

**How to cite this article**: Wang, X. *et al.* Comprehensive Proteomics Analysis of Laticifer Latex Reveals New Insights into Ethylene Stimulation of Natural Rubber Production. *Sci. Rep.* **5**, 13778; doi: 10.1038/srep13778 (2015).

## Supplementary Material

Supplementary Information
