# Supplementary material for: Comprehensive Proteomics Analysis of Laticifer Latex Reveals New Insights into Ethylene Stimulation of Natural Rubber Production
Source: Sci Rep. 2015 Sep 8;5:13778. doi: 10.1038/srep13778 (PMC4562231; doi:10.1038/srep13778)
Supplement: Supplementary Information [file srep13778-s1.pdf]

Supplemental Information

Title: Comprehensive Proteomics Analysis of Laticifer Latex Reveals New Insights into Ethylene Stimulation of Natural Rubber Production

Authors:

Xuchu Wang (Corresponding author)

Key Laboratory of Biology and Genetic Resources for Tropical Crops, Institute of Tropical Biosciences and Biotechnology, Chinese Academy of Tropical Agricultural Sciences, Haikou Hainan 571101, China

E-mail: xchwanghainan@163.com

Dan Wang

Key Laboratory of Biology and Genetic Resources for Tropical Crops, Institute of Tropical Biosciences and Biotechnology, Chinese Academy of Tropical Agricultural Sciences, Haikou Hainan 571101, China

Yong Sun

Key Laboratory of Biology and Genetic Resources for Tropical Crops, Institute of Tropical Biosciences and Biotechnology, Chinese Academy of Tropical Agricultural Sciences, Haikou Hainan 571101, China

Qian Yang

Key Laboratory of Biology and Genetic Resources for Tropical Crops, Institute of Tropical Biosciences and Biotechnology, Chinese Academy of Tropical Agricultural Sciences, Haikou Hainan 571101, China

Lili Chang

Key Laboratory of Biology and Genetic Resources for Tropical Crops, Institute of

Tropical Biosciences and Biotechnology, Chinese Academy of Tropical Agricultural Sciences, Haikou Hainan 571101, China

Limin Wang

Key Laboratory of Biology and Genetic Resources for Tropical Crops, Institute of Tropical Biosciences and Biotechnology, Chinese Academy of Tropical Agricultural Sciences, Haikou Hainan 571101, China

Xueru Meng

Key Laboratory of Biology and Genetic Resources for Tropical Crops, Institute of Tropical Biosciences and Biotechnology, Chinese Academy of Tropical Agricultural Sciences, Haikou Hainan 571101, China

Qixing Huang

Key Laboratory of Biology and Genetic Resources for Tropical Crops, Institute of Tropical Biosciences and Biotechnology, Chinese Academy of Tropical Agricultural Sciences, Haikou Hainan 571101, China

Xiang Jin

Key Laboratory of Biology and Genetic Resources for Tropical Crops, Institute of Tropical Biosciences and Biotechnology, Chinese Academy of Tropical Agricultural Sciences, Haikou Hainan 571101, China

Zheng Tong

Key Laboratory of Biology and Genetic Resources for Tropical Crops, Institute of Tropical Biosciences and Biotechnology, Chinese Academy of Tropical Agricultural Sciences, Haikou Hainan 571101, China

Supplementary Figures and Tables

Supplementary Figure S1: Identification of the high abundant latex proteins in 2-DE gels.

Supplementary Figure S2: Classification and functional analysis of the identified main latex proteins.

Supplementary Figure S3: Identification of the differentially expressed latex proteins in DIGE gels.

Supplementary Figure S4: Identification of ethylene responsive latex proteins by iTRAQ.

Supplementary Figure S5: Information of the 59 phosphorlated latex proteins.

Supplementary Figure S6: Antibody information.

Supplementary Figure S7: Primers used in RT-PCR.

Supplementary Table S1: MS identification of high abundant rubber latex proteins 2-DE gels.

Supplementary Table S2: MS identification of the differential latex proteins in DIGE gels.

Supplementary Table S3: MS information of ethylene responsive latex proteins by iTRAQ.

Supplementary Table S4: MS identification of the phosphorlated latex proteins.

Supplementary Figure S1:

Annotated spectra for the 252 total latex protein spots and 35 bands identified by PMF and the combination results of PMF and PFF.

Spot numbers of the **252** proteins correspond to the proteins that listed in detail.

CID: collision induced desorption;

MALDI TOF:

matrix assisted laser desorption/ionization time of flight;

MS: mass spectrometry;

PMF: peptide mass fingerprinting;

PFF: peptide fragment fingerprinting.

Spot No.: **1**

NCBI accession No.: **gi|132270** Species: *Hevea brasiliensis*

Protein name：Rubber elongation factor protein; Short=REF;

AltName: Allergen=Hev b 1

Mascot score: **122** Sequence coverage %: **73**

Matched peptides No.: 9 Total peptides No.: **23**

Calculated Mr: **14713** Calculated *p*I: **5.04**

Probability Based Mowse Score:

Protein score is -10*Log(P), where P is the probability that the observed match is a random event.
Protein scores greater than 73 are significant (p<0.05).

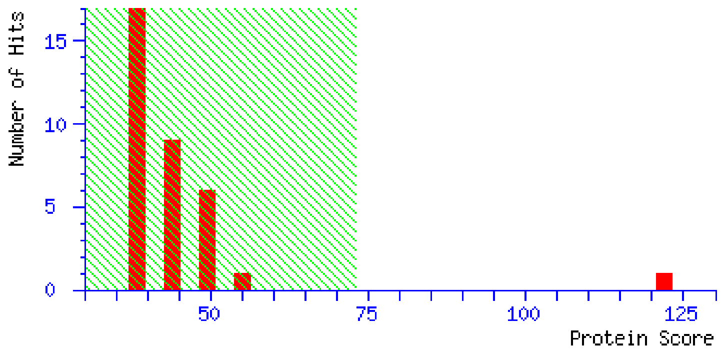

Matched peptide sequences: shown in Bold Red

```
  1 MAEDEDNQQG QGEGLKYLGF VQDAATYAVT TFSNVYLFAK DKSGPLQPGV
 51 DIIEGPVKNV AVPLYNRFSY IPNGALKFVD STVVASVTII DRSLPPIVKD
101 ASIQVVSAIR AAPEAARSLA SSLPGQTKIL AKVFYGEN
```

Spot No.: **2**

NCBI accession No.: **gi|132270** Species: *Hevea brasiliensis*

Protein name：Rubber elongation factor protein; Short=REF;

AltName: Allergen=Hev b 1

Mascot score: **142** Sequence coverage %: **81**

Matched peptides No.: **10** Total peptides No.: **17**

Calculated Mr: **14713** Calculated *p*I: **5.04**

Probability Based Mowse Score:

Protein score is -10*Log(P), where P is the probability that the observed match is a random event.
Protein scores greater than 73 are significant (p<0.05).

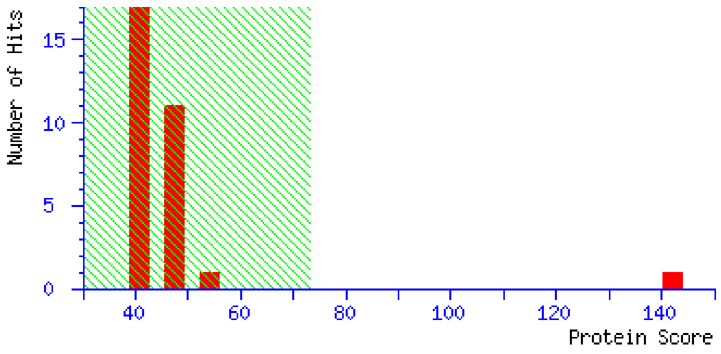

Matched peptide sequences: shown in Bold Red:

1 MAEDEDNQQG QGEGLKYLGF VQDAATYAVT TFSNVYLFAK DKSGPLQPGV
51 DIIEGPVKNV AVPLYNRFSY IPNGALKFVD STVVASVTII DRSLPPIVKD
101 ASIQVVSAIR AAPEAARSLA SSLPGQTKIL AKVFYGEN

Spot No.: **3**

NCBI accession No.: **gi|132270** Species: *Hevea brasiliensis*

Protein name：Rubber elongation factor protein; Short=REF;

AltName: Allergen=Hev b 1

Mascot score: **95** Sequence coverage %: **56**

Matched peptides No.: **8** Total peptides No.: **42**

Calculated Mr: **14713** Calculated *p*I: **5.04**

Probability Based Mowse Score:

Protein score is -10*Log(P), where P is the probability that the observed match is a random event.
Protein scores greater than 73 are significant (p<0.05).

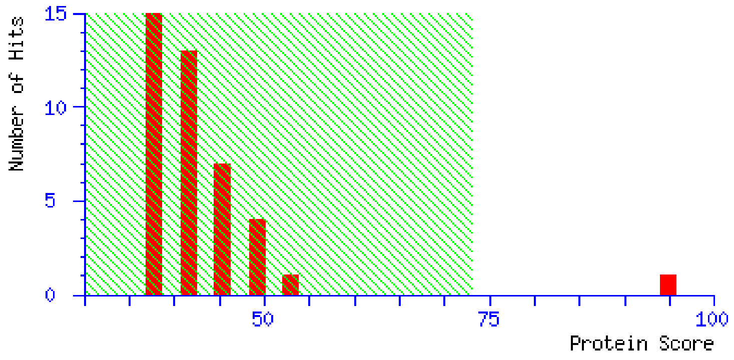

Matched peptide sequences: shown in Bold Red:

1 MAEDEDNQQG QGEGLKYLGF VQDAATYAVT TFSNVYLFAK DKSGPLQPGV
51 DIIEGPVKNV AVPLYNRFSY IPNGALKFVD STVVASVTII DRSLPPIVKD
101 ASIQVVSAIR AAPEAARSLA SSLPGQTKIL AKVFYGEN

Spot No.: **4**

NCBI accession No.: **gi|14423933** Species: *Hevea brasiliensis*

Protein name：Small rubber particle protein; Short=SRPP; AltName: Full=22 kDa rubber particle protein; Short=22 kDa RPP; AltName: Full=27 kDa natural rubber allergen; AltName: Full=Latex allergen Hev b 3

Mascot score: **119** Sequence coverage %: **75**

Matched peptides No.: **12** Total peptides No.: **52**

Calculated Mr: **22331** Calculated *p*I: **4.80**

Probability Based Mowse Score:

Protein score is -10*Log(P), where P is the probability that the observed match is a random event. Protein scores greater than 73 are significant (p<0.05).

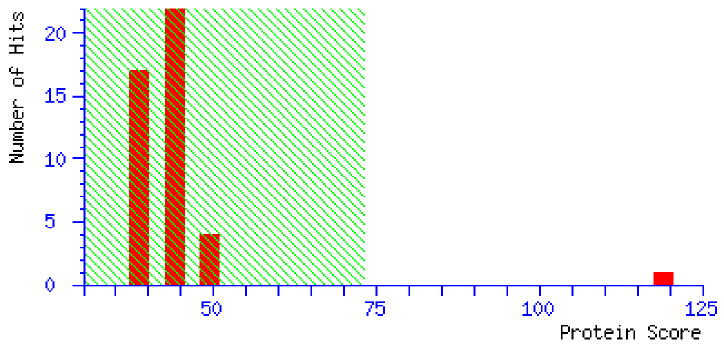

Matched peptide sequences: shown in Bold Red:

```
  1 MAEEVEEERL KYLDFVRAAG VYAVDSFSTL YLYAKDISGP LKPGVDTIEN
 51 VVKTVVTPVY YIPLEAVKFV DKTVDVSVTS LDGVVPPVIK QVSAQTYSVA
101 QDAPRIVLDV ASSVFNTGVQ EGAKALYANL EPKAEQYAVI TWRALNKLPL
151 VPQVANVVVP TAVYFSEKYN DVVRGTTEQG YRVSSYLPLL PTEKITKVFG
201 DEAS
```

Spot No.: **5**

NCBI accession No.: **gi|14423933** Species: *Hevea brasiliensis*

Protein name：Small rubber particle protein; Short=SRPP; AltName: Full=22 kDa rubber particle protein; Short=22 kDa RPP; AltName: Full=27 kDa natural rubber allergen; AltName: Full=Latex allergen Hev b 3

Mascot score: **122** Sequence coverage %: **85**

Matched peptides No.: **13** Total peptides No.: **55**

Calculated Mr: **22331** Calculated *p*I: **4.80**

Probability Based Mowse Score:

Protein score is -10*Log(P), where P is the probability that the observed match is a random event. Protein scores greater than 73 are significant ($p<0.05$).

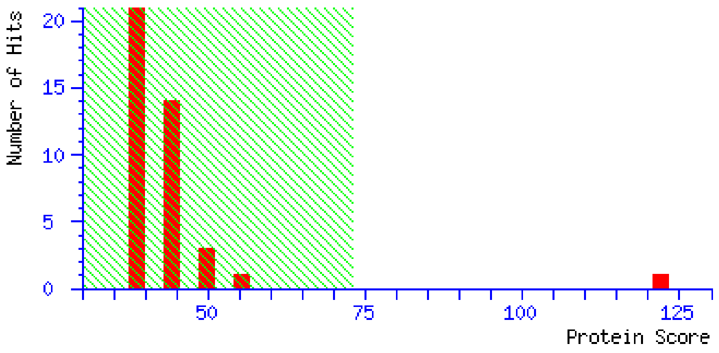

Matched peptide sequences: shown in Bold Red:

1 MAEEVEEERL KYLDFVRAAG VYAVDSFSTL YLYAKDISGP LKPGVDTIEN
51 VVKTVVTPVY YIPLEAVKFV DKTVDVSVTS LDGVVPPVIK QVSAQTYSVA
101 QDAPRIVLDV ASSVFNTGVQ EGAKALYANL EPKAEQYAVI TWRALNKLPL
151 VPQVANVVVP TAVYFSEKYN DVVRGTTEQG YRVSSYLPLL PTEKITKVFG
201 DEAS

Spot No.: **6**

NCBI accession No.: **gi|14423933** Species: *Hevea brasiliensis*

Protein name：Small rubber particle protein; Short=SRPP; AltName: Full=22 kDa rubber particle protein; Short=22 kDa RPP; AltName: Full=27 kDa natural rubber allergen; AltName: Full=Latex allergen Hev b 3

Mascot score: **135** Sequence coverage %: **75**

Matched peptides No.: **12** Total peptides No.: **31**

Calculated Mr: **22331** Calculated *p*I: **4.80**

Probability Based Mowse Score:

Protein score is -10*Log(P), where P is the probability that the observed match is a random event.
Protein scores greater than 73 are significant (p<0.05).

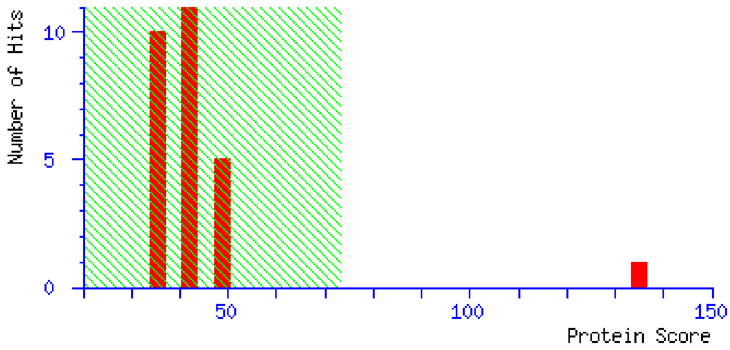

Matched peptide sequences: shown in Bold Red:

```
  1  MAEEVEEERL KYLDFVRAAG VYAVDSFSTL YLYAKDISGP LKPGVDTIEN
 51  VVKTVVTPVY YIPLEAVKFV DKTVDVSVTS LDGVVPPVIK QVSAQTYSVA
101  QDAPRIVLDV ASSVFNTGVQ EGAKALYANL EPKAEQYAVI TWRALNKLPL
151  VPQVANVVVP TAVYFSEKYN DVVRGTTEQG YRVSSYLPLL PTEKITKVFG
201  DEAS
```

Spot No.: **7**

NCBI accession No.: **gi| 29170601** Species: *Hevea brasiliensis*

PFF score: **[121]** Protein name：small rubber particle protein

Matched peptides No.: [2] Sequence coverage %: [23]

Matched sequences: K.ENENFQQEANEQEEK.L;

K.QLPTQACDTSVK.E

Calculated Mr: **12769** Calculated *p*I: **4.61**

Probability Based Mowse Score:

Ions score is -10*Log(P), where P is the probability that the observed match is a random event.
Individual ions scores > 44 indicate identity or extensive homology (p<0.05).
Protein scores are derived from ions scores as a non-probabilistic basis for ranking protein hits.

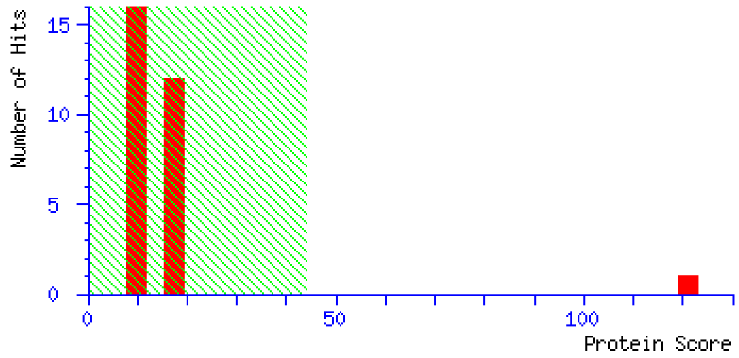

Matched peptide sequences: shown in Bold Red

```
  1 MAEGKENENF QQEANEQEEK LKYLEFVQAT TDNAVTALSN IYLYAKDNSG
 51 PLKPGVETIE GVAKTVVIPA SKIPTEAIKF ADRAVDASFT TLQNIVPSVL
101 KQLPTQACDT SVKESAE
```

Spot No.: **8**

NCBI accession No.: **gi| 29170601** Species: *Hevea brasiliensis*

PFF score: **[110]** Protein name：small rubber particle protein

Matched peptides No.: [1] Sequence coverage %: [15]

Matched sequences: K.DNSGPLKPGVETIEGVAK.T

Calculated Mr: **12769** Calculated *p*I: **4.61**

Probability Based Mowse Score:

Ions score is -10*Log(P), where P is the probability that the observed match is a random event.
Individual ions scores > 45 indicate identity or extensive homology (p<0.05).
Protein scores are derived from ions scores as a non-probabilistic basis for ranking protein hits.

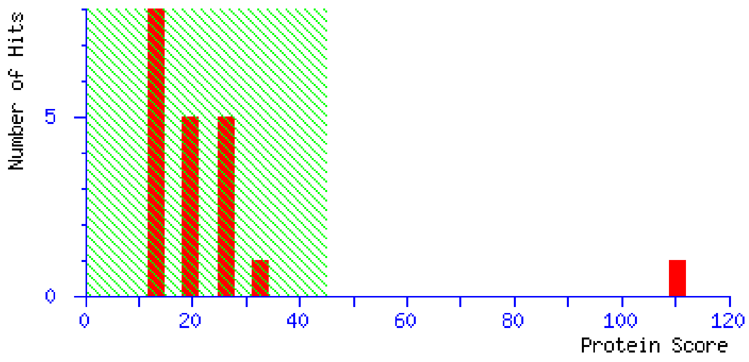

Matched peptide sequences: shown in Bold Red:

```
  1 MAEGKENENF QQEANEQEEK LKYLEFVQAT TDNAVTALSN IYLYAKDNSG
 51 PLKPGVETIE GVAKTVVIPA SKIPTEAIKF ADRAVDASFT TLQNIVPSVL
101 KQLPTQACDT SVKESAE
```

Spot No.: **9**

NCBI accession No.: **gi|388503664** Species: *Lotus japonicus*

Protein name： **unknown**

Mascot score: **93** Sequence coverage %: **45**

Matched peptides No.: **13** Total peptides No.: **48**

Calculated Mr: **29537** Calculated *p*I: **4.79**

Probability Based Mowse Score:

Protein score is -10*Log(P), where P is the probability that the observed match is a random event.
Protein scores greater than 73 are significant ($p<0.05$).

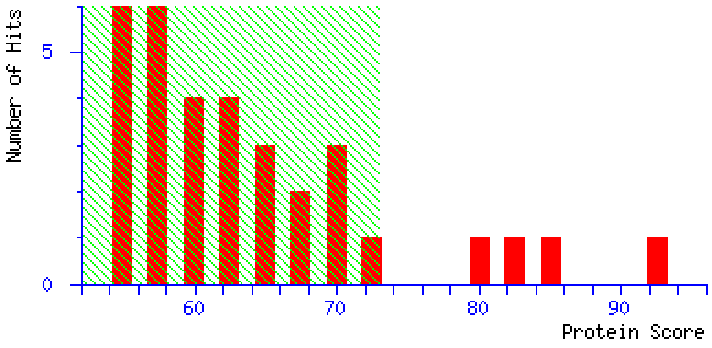

Matched peptide sequences: shown in Bold Red:

1 MASSK**DRGTF** **VYVAKLAEQA** **ER**YEEMVDSM KKVANLDVEL TVEER**NLLSV**
51 **GYKNVIGAR**R ASWR**ILSSIE** **QK**EETKGNDV NAKR**IKEYR**Q KVESELADIC
101 NDVMRVIDEH LIPSATAGES TVFYYKMK**GD** **YYRYLAEFK**S GNEK**KEAADQ**
151 **SMKAYESATT** **AAEAELPPTH** **PIRLGLALNF** **SVFYYEILNS** **PERACHLAK**Q
201 AFDEAISELD TLNEESYK**DS** **TLIMQLLR**DN LTLWTSDIPE EGEDSLKAND
251 TAKVGGGEDA E

Spot No.: **10**

NCBI accession No.: **gi|3288200** Species: *Hevea brasiliensis*

Protein name: **latex allergen**

Mascot score: **209** Sequence coverage %: **68**

Matched peptides No.: **22** Total peptides No.: **42**

Calculated Mr: **43020** Calculated *p*I: **5.00**

Probability Based Mowse Score:

Protein score is -10*Log(P), where P is the probability that the observed match is a random event. Protein scores greater than 73 are significant ($p<0.05$).

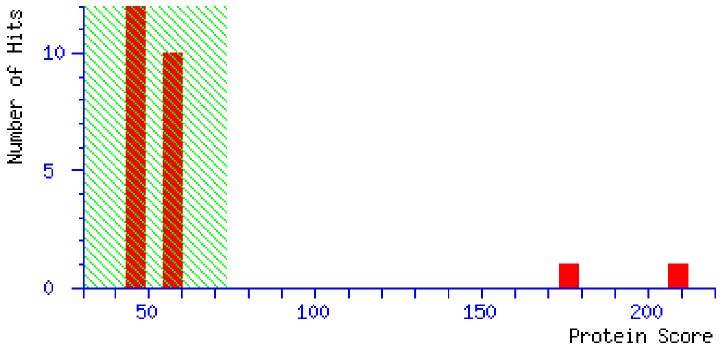

Matched peptide sequences: shown in Bold Red:

```
  1 MATGSTTLTQ GKKITVLSID GGGIRGIIPG IILASLESKL QDLDGPDARI
 51 ADYFDIIAGT STGGLITTML TAPNEDKKPI YQAKDIKDFY LENCPKIFPK
101 ESRDTYDPIH SIGPIYDGEY LRELCNNLLK DLTVKDTLTD VIIPAFDIKL
151 LLPVIFSSDD AKCNALKNAR LADVCISTSA APVLLPAHSF TTEDDKNIHT
201 FELIDGGVAA ANPTLLALTH IRNEIIRQNP RFIGANLTES KSRLVLSLGT
251 GKSEYKEKYN ADMTSKWRLY NWALYNGNSP AVDIFSNASS DMVDFHLSAL
301 FKSLDCEDYY LRIQDDTLTG EESSGHIATE ENLQRLVEIG TELLEKQESR
351 INLDTGRLES IPGAPTNEAA IAKFAKLLSE ERKLRQLK
```

Spot No.: **11**

NCBI accession No.: **gi|6707018** Species: *Hevea brasiliensis*

Protein name: **latex protein allergen Hev b 7**

Mascot score: **172** Sequence coverage %: **45**

Matched peptides No.: **17** Total peptides No.: **45**

Calculated Mr: **43107** Calculated *p*I: **5.00**

Probability Based Mowse Score:

Protein score is -10*Log(P), where P is the probability that the observed match is a random event. Protein scores greater than 73 are significant (p<0.05).

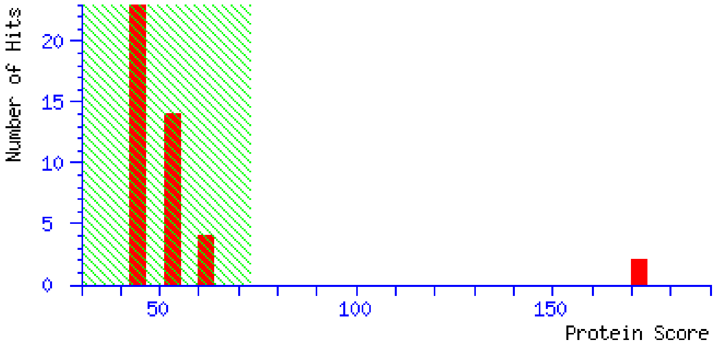

Matched peptide sequences: shown in Bold Red:

1 MATGSTPLTQ GKKITVLSID GGGIRGIIPG IILASLESKL QDLDGPDARI
51 ADYFDIIAGT STGGLITTML TAPNEDKKPM YQAKDIKDFY LENCPKIFPK
101 ESRDNYDPIH SIGPIYDGEY LRELCNNLLK DLTVKDTLTD VIIPTFDIKL
151 LLPVIFSSDD AKCNALKNAR LADVCISTSA APVLLPAHSF TTEDDKNIHT
201 FELIDGGVAA TNPTLLALTH IRNEIIRQNP RFIGANLTES KSRLVLSLGT
251 GKSEYKEKYN ADMTSKWRLY NWALYNGNSP AVDIFSNASS DMVDFHLSAL
301 FKSLDCEDYY LRIQDDTLTG EESSGHIATE ENLQRLVEIG TELLEKQESR
351 INLDTGRLES IPGAPTNEAA IAKFAKLLSE ERKLRQLK

Spot No.: **12**

NCBI accession No.: **gi|4235430** Species: *Hevea brasiliensis*

Protein name: **latex-abundant protein**

Mascot score: **82** Sequence coverage %: **49**

Matched peptides No.: **15** Total peptides No.: **29**

Calculated Mr: **46443** Calculated *p*I: **5.01**

Probability Based Mowse Score:

Protein score is -10*Log(P), where P is the probability that the observed match is a random event.
Protein scores greater than 73 are significant (p<0.05).

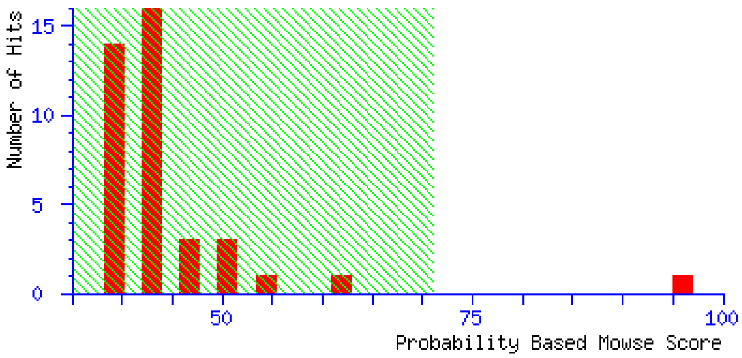

Matched peptide sequences: shown in Bold Red:

1 MAKKAVLIGI NYPGTKAELK GCINDVKRMY RCLVDR**YGFS** **EEDITVLIDI**
51 **DESYIQPTGK** NIR**RVLTDLV** **RSAEPGDLLF** **VHYSGHGTRL** **PAETGEDDDT**
101 **GFDECIVPCD** **MNLITDDDFR** **EFVDQVPHGC** **R**ITVVSDSCH SGGLIDEAKE
151 QIGESTKRKE EESESGFGFK SFLK**QTIQDA** **FESRGVHLPS** **DLHHHHGHRD**
201 **EEDFDNRVVE** **EDYGDSGYVK** SK**SLPLSTLI** **EILK**QKTGKD DIDVGK**LRPT**
251 **LFDMFGDDAS** **PK**VKKFMKVI LNKLR**HGDGE** **SGGGGFLGMV** **GSLAQEFLK**H
301 KLDENDESYV KPALETEVDS KQEVYAGKTK RSLPDGGILI SGCQTDQTSA
351 DASPSGKSSE AYGALSNAIQ TIIAETDGAV TNQELVLKAR KMLKKQGFTQ
401 K**PGLYCSDDH** **VEASFVC**

Spot No.: **13**

NCBI accession No.: **gi|4235430** Species: *Hevea brasiliensis*

Protein name: **latex-abundant protein**

Mascot score: **96** Sequence coverage %: **54**

Matched peptides No.: **15** Total peptides No.: **37**

Calculated Mr: **46443** Calculated *p*I: **5.01**

Probability Based Mowse Score:

Protein score is -10*Log(P), where P is the probability that the observed match is a random event.
Protein scores greater than 73 are significant (p<0.05).

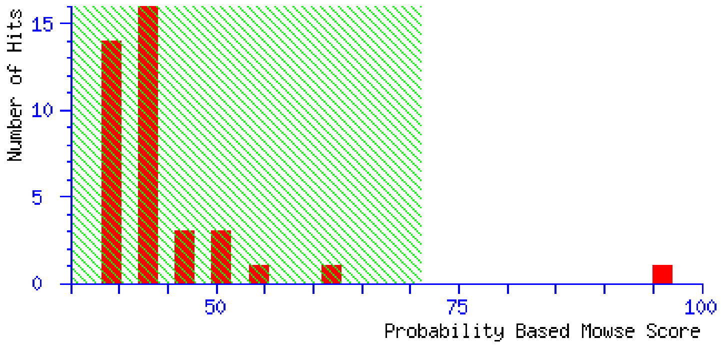

Matched peptide sequences: shown in Bold Red:

1 MAKKAVLIGI NYPGTKAELK GCINDVKRMY RCLVDR**YGFS** **EEDITVLIDI**
51 **DESYIQPTGK** NIR**RVLTDLV** **RSAEPGDLLF** **VHYSGHGTRL** **PAETGEDDDT**
101 **GFDECIVPCD** **MNLITDDDFR** **EFVDQVPHGC** **RITVVSDSCH** **SGGLIDEAK**E
151 QIGESTKRKE EESESGFGFK SFLK**QTIQDA** **FESRGVHLPS** **DLHHHHGHRD**
201 **EEDFDNRVVE** **EDYGDSGYVK** SK**SLPLSTLI** **EILK**QKTGKD DIDVGK**LRPT**
251 **LFDMFGDDAS** **PK**VKKFMKVI LNKLRHGDGE SGGGGFLGMV GSLAQEFLK**H**
301 **KLDENDESYV** **KPALETEVDS** **K**QEVYAGKTK RSLPDGGILI SGCQTDQTSA
351 DASPSGKSSE AYGALSNAIQ TIIAETDGAV TNQELVLKAR KMLK**KQGFTQ**
401 **KPGLYCSDDH** **VEASFVC**

Spot No.: **14**

NCBI accession No.: **gi|20559** Species: *Petunia x hybrida*

Protein name: **hsp70 (AA 6 - 651)**

Mascot score: **103** Sequence coverage %: **40**

Matched peptides No.: **24** Total peptides No.: **41**

Calculated Mr: **71137** Calculated *p*I: **5.07**

Probability Based Mowse Score:

Protein score is -10*Log(P), where P is the probability that the observed match is a random event.
Protein scores greater than 73 are significant (p<0.05).

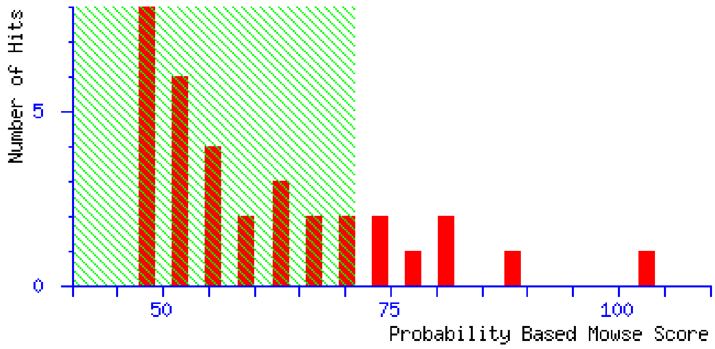

Matched peptide sequences: shown in Bold Red:

1	EGPAIGIDLG	TTYSCVGVWQ	HDRVEIIAND	QGNRTTPSYV	GFTDTERLIG
51	DAAKNQVAMN	PINTVFDAKR	LIGRRFSDPS	VQSDIKLWPF	KVIPGPGDKP
101	MIVVTYKGEE	KQFAAEEISS	MVLTK**MKEIA**	**EAYLGTTIKN**	**AVVTVPAYFN**
151	**DSQR**QATK**DA**	**GVIAGLNVMR**	**IINEPTAAAI**	**AYGLDKK**ASS	AGEKNVLIFD
201	LGGGTFDVSL	LTIEEGIFEV	K**ATAGDTHLG**	**GEDFDNRMVN**	**HFVQEFK**RKN
251	KK**DISGNPRA**	LRRLRTACER	AKR**TLSSTAQ**	**TTIEIDSLYE**	**GIDFYSTITR**
301	**ARFEELNMDL**	**FRKCMEPVEK**	CLRDAKMDK**S**	**SVHDVVLVGG**	**STR**IPKVQQL
351	LQDFFNGKEL	CKSINPDEAV	AYGAAVQAAI	LSGEGNEKVQ	DLLLLDVTPL
401	SLGLETAGGG	MTVLIPRNTT	IPTKK**EQVFS**	**TYSDNQPGVL**	**IQVYEGER**AR
451	TKDNNLLGKF	ELSGIPPAPR	**GVPQITVCFD**	**IDANGILNVS**	**AEDK**TTGQKN
501	KITITNDKGR	LSK**EEIERMV**	**QEAEK**YKSED	EELKKKVEAK	**NALENYAYNM**
551	**RNTIKDDKIN**	**SQLSAADKKR**	**IEDAIDEAIK**	WLDNNQLAEA	DEFEDK**MKEL**
601	**ESICNPIIAK**	MYQGGAGGAT	MDEDGPSVGG	SAGSQTGAGP	KIEEVD

Spot No.: **15**

NCBI accession No.: **gi|58013197**   Species: *Isatis tinctoria*

Protein name: **actin**

Mascot score: **142**   Sequence coverage %: **51**

Matched peptides No.: **16**   Total peptides No.: **36**

Calculated Mr: **42020**   Calculated *p*I: **5.31**

Probability Based Mowse Score:

Protein score is -10*Log(P), where P is the probability that the observed match is a random event.
Protein scores greater than 73 are significant (p<0.05).

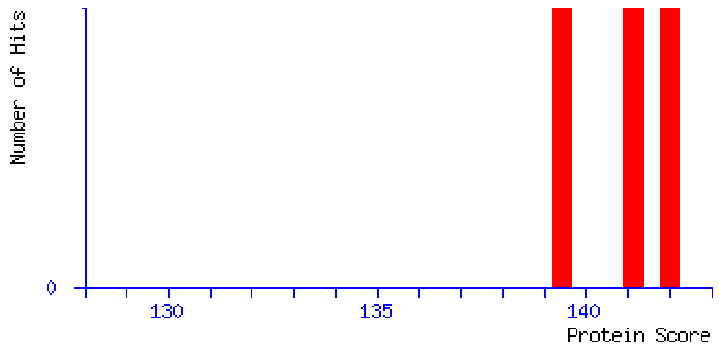

Matched peptide sequences: shown in Bold Red:

```
  1 MADGEDIQPL VCDNGTGMVK AGFAGDDAPR AVFPSIVGRP RHTGVTVWMG
 51 QKDAYVGDEA QSKRGILTLK YPIEHGIVSN WDDMEKIWHH TFYNELRVAP
101 EEHPVLLTEA PLNPKANREK MTQIMFETFN VPAMYVAIQA VLSLYASGRT
151 TGIVLDSGDG VSHTVPIYEG YALPHAILRL DLAGRDLTDS LMKILTERGY
201 MFTTTAEREI VRDIKEKLAY VALDYEQELE TAKSSSSVEK NYELPDGQVI
251 TIGAERFRCP EILFQPSLIG MEAPGIHETT YNSIMKCDVD IRKDLYGNIV
301 LSGGSTMFPG IADRMSKEIT ALAPSSMKIK VVAPPERKYS VWIGGSILAS
351 LSTFQQMWIS KGEYDESGPS IVHRKCF
```

Spot No.: **16**

NCBI accession No.: gi|2506211

Species: *Vigna radiata var. radiata*

Protein name: V-type proton ATPase catalytic subunit A

PMF Mascot score: **145** Sequence coverage %: **45**

Matched peptides No.: **27** Total peptides No.: **69**

Calculated Mr: **68923** Calculated *p*I: **5.30**

Probability Based Mowse Score:

Protein score is -10*Log(P), where P is the probability that the observed match is a random event.
Protein scores greater than 73 are significant (p<0.05).

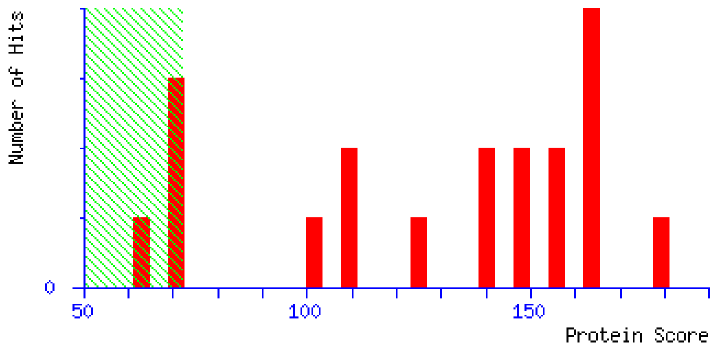

Matched peptide sequences: shown in Bold Red:

1 MPAVYGAR**LT** **TFEDSEKESE** **YGYVRKVSGP** **VVVADGMAGA** **AMYELVR**VGR
51 DNLIGEIIR**L** **EGDSATIQVY** **EETAGLMVND** **PVLRTHKPLS** **VELGPGILGN**
101 **IFDGIQRPLK** TIAKRSGDVY IPR**GVSVPAL** **DKDTLWEFQP** **K**KIGEGDLLT
151 GGDLYATVFE NTLMQHHIAL PPDAMGKITY IAPPGQYSIT DTVLELEFQG
201 VKK**KFTMLQT** **WPVR**TPRPVA SK**LAADTPLL** **TGQRVLDALF** **PSVLGGTCAI**
251 **PGAFGCGK**TV ISQALSKYSN SDAVVYVGCG ER**GNEMAEVL** **MDFPQLTMTL**
301 **PDGR**EESVMK RTTLVANTSN MPVAAR**EASI** **YTGITLAEYF** **R**DMGYNVSMM
351 ADSTSR**WAEA** **LR**EISGR**LAE** **MPADSGYPAY** **LAAR**LASFYE RPGKVKCLGG
401 PERTGSVTIV GAVSPPGGDF SDPVTSATLS IVQVFWGLDK KLAQR**KHFPS**
451 **VNWLISYSK**Y STALESFYEQ FDPDFINIRT KAR**EVLQRED** **DLNEIVQLVG**
501 **K**DALAEGDKI TLETAK**LLRE** **DYLAQNAFTP** **YDKFCPFYK**S VWMMR**NIIHF**
551 **YNLANQAVER** GAGSDGQKIT YSLIKHRVGD LFYRLVSQKF EDPAEGEAAL
601 VGQFQKLHED LSTGFRNLED ETR

Spot No.: **17**

NCBI accession No.: **gi|357480067**   Species: *Medicago truncatula*

Protein name: Leucine aminopeptidase

Mascot score: **92**   Sequence coverage %: **31**

Matched peptides No.: **16**   Total peptides No.: **69**

Calculated Mr: **59856**   Calculated *p*I: **7.56**

Probability Based Mowse Score:

Protein score is -10*Log(P), where P is the probability that the observed match is a random event.
Protein scores greater than 73 are significant (p<0.05).

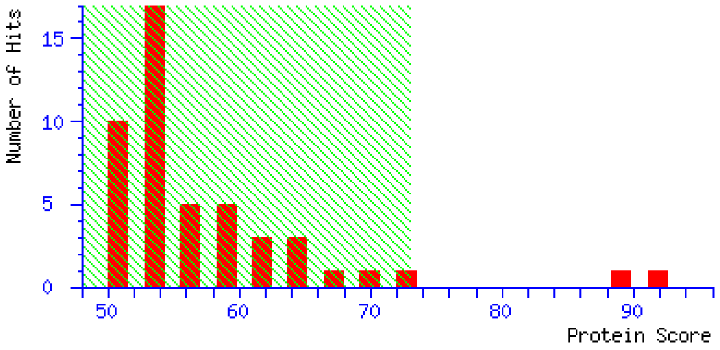

Matched peptide sequences: shown in Bold Red:

```
  1 MAAIASAIVI AFSKSSSSSL FLTSRIRFAS FPKRSFHSTT KLMSQSRYTT
 51 LGLTHPTNIE APKISFSATD VDVTEWKGDI LAVGVTEKDL TRDAKSRFEN
101 SILNKIDLKL DGLLSEASSE EDFSGKVGQS TVVRIKGLGS KRVGLIGLGQ
151 LPSTTALYKG LGEAVVAVAK SAQASNVAIV LASSEGLSSE SKLSTAYAIA
201 SGAVLGLFED QRYKSESKKP AVRSIDIIGL GTGPDLEKKL KYAGDVSSGI
251 IFGRELVNSP ANVLTPGVLA EEASKVASTY SDVFTAKILD ADQCKELKMG
301 SYLGVAAASA NPPRFIHLTY KPPSGSVKVK LALVGKGLTF DSGGYNIKTG
351 PGCSIELMKF DMGGSAAVLG AAKALGQIKP LGVEVHFIVA ACENMISGTG
401 MRPGDVVTAS NGKTIEVNNT DAEGRLTLAD ALVYACNQGV EKIIDLATLT
451 GACVVALGPS IAGVFSPNDE LVKEVLEASE VSGEKLWRLP IEESYWESMK
501 SGVADMVNTG GRPGGSITAA LFLKQFVDEK VQWLHIDMAG PVWNDKKRSA
551 TGFGVSTLVE WVLKNSS
```

Spot No.: **18**

NCBI accession No.: **gi|219885949** Species: *Zea mays*

Protein name: unknown

Mascot score: **81** Sequence coverage %: **35**

Matched peptides No.: **8** Total peptides No.: **46**

Calculated Mr: **30752** Calculated *p*I: **5.91**

Probability Based Mowse Score:

Protein score is -10*Log(P), where P is the probability that the observed match is a random event.
Protein scores greater than 73 are significant ($p<0.05$).

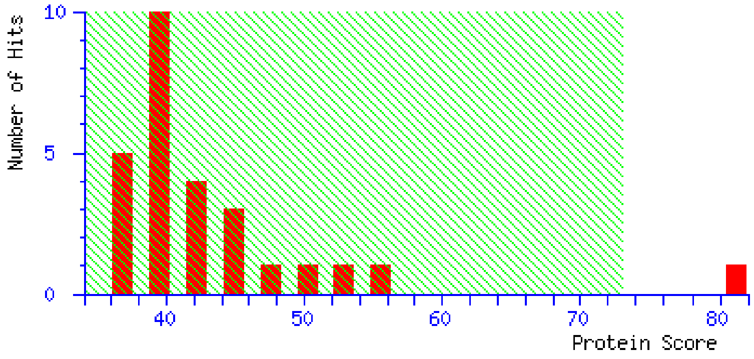

Matched peptide sequences: shown in Bold Red:

1 **MALAAVIRSS** **RATTMMELEI** **ELK**KASDKLK **SWDATSISLS** **AACDLFMRFV**
51 **TRTSHLEHEK** **FDAAK**SRLIE RGEK**FGEISL** **KARKTIAMLS** **QDFIYDGCTI**
101 **LVHGYSRVVL** **EVLK**LAASNR KLFRVLCTEG RPDRTGLRMS NELAALGIPV
151 KVLIDSAVAY SIDEVDMVFV GADGVVESGG IINMMGTYQI ALVAHTMDKP
201 VYVAAESHKF ARLYPLDQKD MTPAHRPIDF GVPIPIGVEV ETSARDYTPP
251 QYLTLLLTDL GVLTPSVVSD ELIQLYL

Spot No.: **19**

NCBI accession No.: **gi|242059605** Species: *Sorghum bicolor*

PFF score: **[101]**

Protein name： hypothetical protein SORBIDRAFT_03g043200

Matched peptides No.: [1] Sequence coverage %: [6]

Matched sequences: R.FFPSEFGNDVDHVHAVEPAK.S;

Calculated Mr: **34294** Calculated *p*I: **5.93**

Probability Based Mowse Score:

Ions score is -10*Log(P), where P is the probability that the observed match is a random event.
Individual ions scores > 43 indicate identity or extensive homology (p<0.05).
Protein scores are derived from ions scores as a non-probabilistic basis for ranking protein hits.

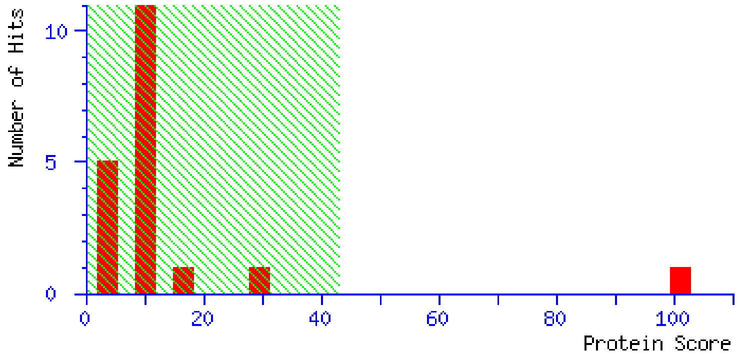

Matched peptide sequences: shown in Bold Red：

1	MSSAAGHKER	SRVLVIGGTG	YIGRFIVAAS	AREGHPTYVL	VRDPAPADPA
51	KAAVLQGFRD	AGVTLVKGDL	YNHESLVVAM	ESADVVISAV	GYAQLPDQTR
101	IISAIKDAGN	IKR**FFPSEFG**	**NDVDHVHAVE**	**PAK**SVFAAKA	SIRRAVEAEG
151	IPYTYISSNF	FAGRFLPAIG	QIGVTGLPID	KVLILGDGNV	KAIFGTEEDV
201	GTYTIKAVDD	PRTLNKILYL	RPPSNILSHN	ELISLWEKKV	GKTFERVYIP
251	EDDVLKKIQE	SPIPLNRALS	ISHSAWVKGD	HTNFEIDPFF	GVEATDLYPD
301	VKYTTVDEYL	NKFL			

Spot No.: **20**

NCBI accession No.: **gi|30575570** Species: *Citrus x paradisi*

PFF score: **[86]** Protein name：HSP19 class II, partial

Matched peptides No.: [1] Sequence coverage %: [15]

Matched sequences: K.VQVEDDNVLLISGER.K;

Calculated Mr: **11191** Calculated *p*I: **8.01**

Probability Based Mowse Score:

Ions score is -10*Log(P), where P is the probability that the observed match is a random event.
Individual ions scores > 44 indicate identity or extensive homology (p<0.05).
Protein scores are derived from ions scores as a non-probabilistic basis for ranking protein hits.

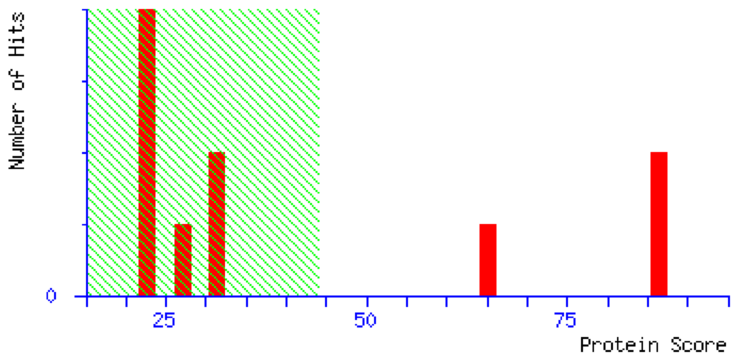

Matched peptide sequences: shown in Bold Red：

```
 1  YVFIVDMPGL  KSGDIKVQVE  DDNVLLISGE  RKREEEKDGA  KYVRMERRVG
51  KFMRKFVLPE  NANVEAISAV  CQDGVLTVTV  DKLPPPEPKK  PKTIEVKIA
```

Spot No.: **21**

NCBI accession No.: **gi|27449246** Species: *Hevea brasiliensis*

Protein name: Cu/Zn superoxide dismutase

Mascot score: **83** Sequence coverage %:**48**

Matched peptides No.: **7** Total peptides No.: **21**

Calculated Mr: **15726** Calculated *p*I: **5.73**

Probability Based Mowse Score:

Protein score is -10*Log(P), where P is the probability that the observed match is a random event.
Protein scores greater than 73 are significant ($p<0.05$).

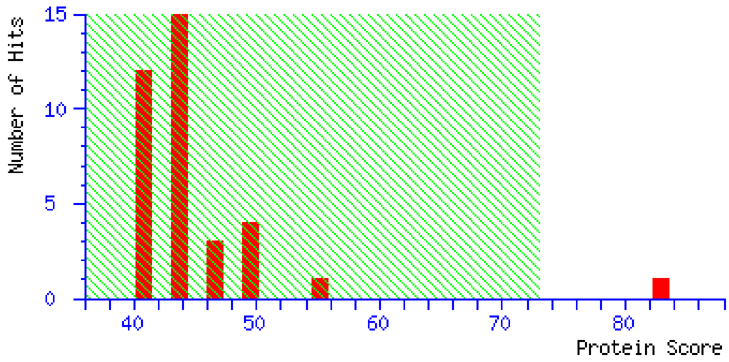

Matched peptide sequences: shown in Bold Red:

```
  1  MLKAVAVITS  SEGISGKIFF  TQEGDGPTTV  TGSVSGLKPG  LHGFHVHTFG
 51  DTTNGCLSTG  LHFNPASKDH  GGPEDENRHA  GDLGNVNVGD  DGTANFTIVD
101  KHIPLSGPHS  IAGRSVVFHE  GRDDLGKGGH  ELSKITGNAG  DRIACGIIGL
151  QE
```

Spot No.: **22**

NCBI accession No.: **gi|37806305**

Species: *Oryza sativa Japonica Group*

PFF score: **[60]** Protein name：hypothetical protein

Matched peptides No.: [2] Sequence coverage %: [3]

Matched sequences: K.LGTLVLGDRPR.L ; K.LGTLVLGDRPR.L

Calculated Mr: **35105** Calculated *p*I: **8.29**

Probability Based Mowse Score:

Ions score is -10*Log(P), where P is the probability that the observed match is a random event.
Individual ions scores > 45 indicate identity or extensive homology (p<0.05).
Protein scores are derived from ions scores as a non-probabilistic basis for ranking protein hits.

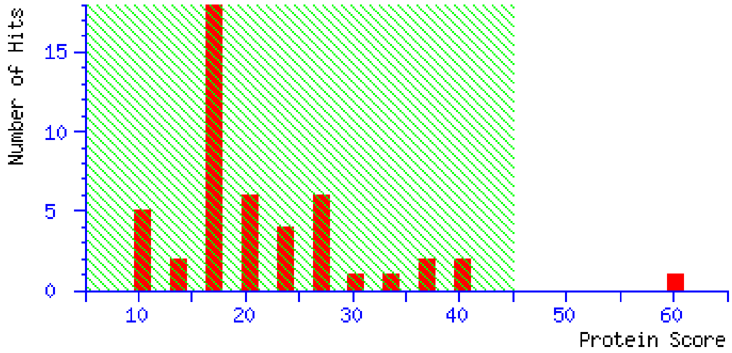

Matched peptide sequences: shown in Bold Red：

```
  1 MEVFQGVQFA RLRNWWEETY VTADEDGRSV YHYAPDRHRP AHEAIWAVQM
 51 VLAGAPPTQY VLLRGAYGRY LGAPDAVVRR WPLSCCWPAP VVGQRDFDQP
101 EVDAIMWRAV RRADHVLCLH DKSGRYLRGK LGTLVLGDRP RLTVGDGRLN
151 DDEKALRWEV LPVLPNPGRP ELPISIVPEA DLVGRLVKAC FLPLQREIQF
201 VEADDDGNIG EGQEVWDSFQ YEGRSVQLLR NELEDRVGYA ITVCVRAGRH
251 GQHSPLLINL PHSRETLHIV VLRRNSEADN QLTFPDPKAS SRRRYRHRRR
301 AIIQ
```

Spot No.: **23**

NCBI accession No.: **gi|10946499** Species: *Hevea brasiliensis*

Protein name: beta-1,3-glucanase

Mascot score: **85** Sequence coverage %:**43**

Matched peptides No.: **14** Total peptides No.: **53**

Calculated Mr: **35295** Calculated *p*I: **9.46**

Probability Based Mowse Score:

Protein score is -10*Log(P), where P is the probability that the observed match is a random event.
Protein scores greater than 73 are significant ($p<0.05$).

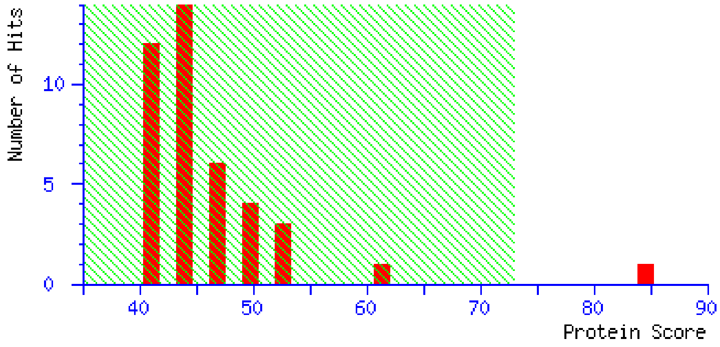

Matched peptide sequences: shown in Bold Red:

1 QVGVCYGMQG NNLPPVSEVI ALYKKSNITR MR**IYDPNRAV** **LEALR**GSNIE
51 LILGVPNSDL QSLTNPSNAK SWVQKNVRGF WSSVLFR**YIA** **VGNEISPVNR**
101 **GTAWLAQFVL** **PAMRNIHDAI** **RSAGLQDQIK** **VSTAIDLTLV** **GNSYPPSAGA**
151 **FRDDVRSYLN** **PIIRFLSSIR** SPLLANIYPY FTYAGNPRDI SLPYALFTSP
201 SVVVWDGQRG YK**NLFDATLD** **VLYSALER**AS GGSLEVVVSE SGWPSAGAFA
251 ATFDNGR**TYL** **SNLIQHVK**RG TPKRPNR**AIE** **TYLFAMFDEN** **K**KQPEVEKQF
301 GLFFPDKWQK YNLNFG

Spot No.: **24**

NCBI accession No.: **gi|78216493** Species: *Malus x domestica*

Protein name: cytosolic malate dehydrogenase

Mascot score: **82** Sequence coverage %:54

Matched peptides No.: **15** Total peptides No.: **50**

Calculated Mr: **35970** Calculated *p*I: **6.01**

Probability Based Mowse Score:

Protein score is -10*Log(P), where P is the probability that the observed match is a random event. Protein scores greater than 73 are significant ($p<0.05$).

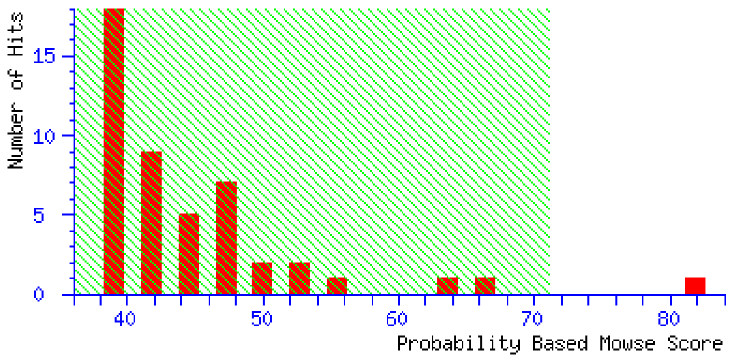

Matched peptide sequences: shown in Bold Red:

1	MAKEPVRVLV	TGAAGQIGYA	LVPMIARGIM	LGADQPVILH	LLDIPPAAEA
51	LNGVKMELVD	AAFPLLKGVV	ATTDVVEACT	GVNIAVMVGG	FPRKEGMERK
101	DVMTKNVSIY	KSQASALEKH	AAPNCKVLVV	ANPANTNALI	LKEFAPSIPE
151	KNVTCLTRLD	HNRALGQVSE	RLNVQVSDVK	NVIIWGNHSS	SQYPDVNHAT
201	VKTPSGEKCV	RELVADDAWL	NGEFISTVQQ	RGAAIIKARK	LSSALSAASS
251	ACDHIRDWVL	GTPEGTWVSM	GVYSDGSYDV	PSGLIFSFPV	TCQHGEWKIV
301	QGLSIDEFSR	KKLDATAEEL	SEEKALAYSC	LS	

Spot No.: **25**

NCBI accession No.: **gi|257219562** Species: *Jatropha curcas*

PFF score: [81] Protein name： annexin-like protein

Matched peptides No.: [2] Sequence coverage %: [5]

Matched sequences: K.AFEGWGTNEK.A ; R.WILDPEDR.D

Calculated Mr: **36015** Calculated *p*I: **6.41**

Probability Based Mowse Score:

Ions score is -10*Log(P), where P is the probability that the observed match is a random event.
Individual ions scores > 45 indicate identity or extensive homology (p<0.05).
Protein scores are derived from ions scores as a non-probabilistic basis for ranking protein hits.

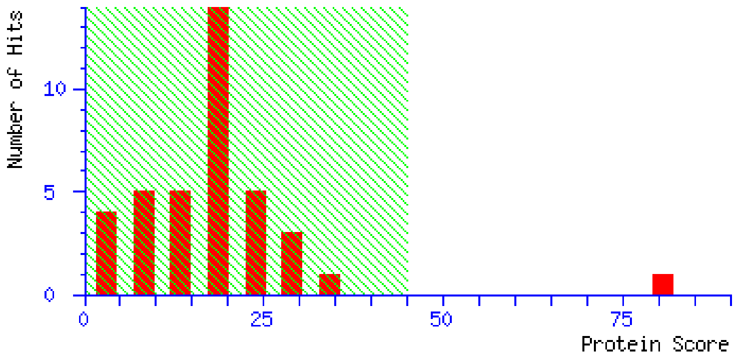

Matched peptide sequences: shown in Bold Red：

```
  1 MATIVVPANV SYVEDAETLR KAFEGWGTNE KAVIAVLGHR NAVQKKHIRQ
 51 AYWDLYQEDL VKRLESELGG DFERAMYRWI LDPEDRDAVL ANVALRKSGD
101 FHVIVEIACA RSAEELLLVR RAYQARYKHS LEEDVATHTT GDIRKLLVGL
151 VTAFMYEGAE INTRLAKSEA DVLQEAIKDK HFNHDEVIRI LTTRSKTQLN
201 TTFNHFKDDH GTSITKALLG EKADNEFVRL LSIAIRTMNE PLKYYEKVLR
251 NAIKRIGTDE DALTRVIVTR AEKDLLHIKE LYPKRNNVPL DHAVDKEIHG
301 DYKHFLLALL GHQD
```

Spot No.: **26**

NCBI accession No.: **gi|3451147** Species: *Hevea brasiliensis*

PFF score: [56] Protein name： chitinase

Matched peptides No.: [1] Sequence coverage %: [4]

Matched sequences: K.VYLTAAPQCPFPDR.Y

Calculated Mr: **34138** Calculated *p*I: **8.72**

Probability Based Mowse Score:

Ions score is -10*Log(P), where P is the probability that the observed match is a random event.
Individual ions scores > 44 indicate identity or extensive homology (p<0.05).
Protein scores are derived from ions scores as a non-probabilistic basis for ranking protein hits.

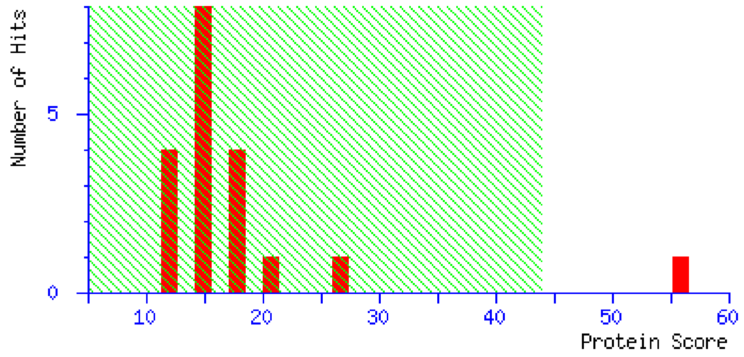

Matched peptide sequences: shown in Bold Red：

```
  1 MAKRTQAILL LLLAISLIMS SSHVDGGGIA IYWGQNGNEG TLTQTCSTRK
 51 YSYVNIAFLN KFGNGQTPQI NLAGHCNPAA GGCTIVSNGI RSCQIQGIKV
101 MLSLGGGIGS YTLASQADAK NVADYLWNNF LGGKSSSRPL GDAVLDGIDF
151 DIEHGSTLYW DDLARYLSAY SKQGKKVYLT AAPQCPFPDR YLGTALNTGL
201 FDYVWVQFYN NPPCQYSSGN INNIINSWNR WTTSINAGKI FLGLPAAPEA
251 AGSGYVPPDV LISRILPEIK KSPKYGGVML WSKFYDDKNG YSSSIRDSVL
301 FLHSEKCMTV L
```

Spot No.: **27**

NCBI accession No.: **gi|4469175** Species: *Hevea brasiliensis*

Protein name: beta-1,3-glucanase

Mascot score: **97** Sequence coverage %:46

Matched peptides No.: **16** Total peptides No.: **107**

Calculated Mr: **38930** Calculated *p*I: **9.45**

Probability Based Mowse Score:

Protein score is -10*Log(P), where P is the probability that the observed match is a random event.
Protein scores greater than 73 are significant ($p<0.05$).

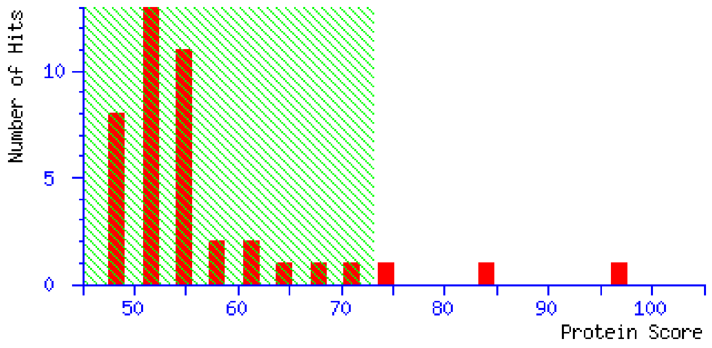

Matched peptide sequences: shown in Bold Red:

1 **MAISSSTSGT** **SSSLPSR**TTV MLLLFFFAAS VGITDAQVGV CYGMQGNNLP
51 PVSEVIALYK KSNITRMR**IY** **DPNRAVLEAL** **RGSNIELILG** **VPNSDLQSLT**
101 **NPSNAK**SWVQ KNVR**GFWSSV** **LFRYIAVGNE** **ISPVNRGTAW** **LAQFVLPAMR**
151 **NIHDAIRSAG** **LQDQIK**VSTA IDLTLVGNSY PPSAGAFRDD VR**SYLNPIIR**
201 **FLSSIR**SPLL ANIYPYFTYA GNPRDISLPY ALFTSPSVVV WDGQRGYK**NL**
251 **FDATLDVLYS** **ALER**ASGGSL EVVVSESGWP SAGAFAATFD NGR**TYLSNLI**
301 **QHVK**RGTPKR PNR**AIETYLF** **AMFDENKK**QP EVEKQFGLFF PDKWQKYNLN
351 FG

Spot No.: **28**

NCBI accession No.: **gi|124365253** Species: *Hevea brasiliensis*

Protein name: beta-1,3-glucanase

Mascot score: **99** Sequence coverage %:**37**

Matched peptides No.: **15** Total peptides No.: **44**

Calculated Mr: **41543** Calculated *p*I: **9.40**

Probability Based Mowse Score:

Protein score is -10*Log(P), where P is the probability that the observed match is a random event.
Protein scores greater than 73 are significant (p<0.05).

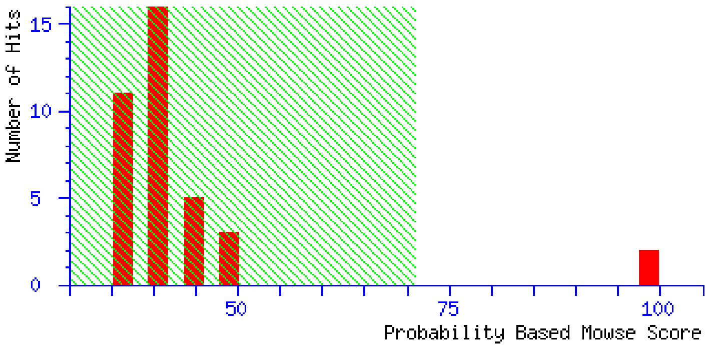

Matched peptide sequences: shown in Bold Red:

1 MAISSSTSGT SSSLPSRTTV MLLLFFFTAS VGITDAQVGV CYGMQGNNLP
51 PVSEVIALYK K**SNITRMRIY** **DPNQAVLEAL** **R**GSNIELILG VPNSDLQSLT
101 NPSNAKSWVQ KNVRGFWSSV RFR**YIAVGNE** **ISPVNR**GTAW LAQFVLPAMR
151 **NIHDAIRSAG** **LQDQIKVSTA** **IDLTLVGNSY** **PPSAGAFRDD** **VRSYLNPIIR**
201 **FLSSIR**SPLL ANIYPYFTYA GNPRDISLPY ALFTSPSVVV WDGQRGYK**NL**
251 **FDATLDALYS** **ALER**ASGGSL EVVVSESGWP SAGAFAATFD NGR**TYLSNLI**
301 **QHVK**RGTPKR PKR**AIETYLF** **AMFDENKK**QP EVEK**HFGLFF** **PNK**WQKYNLN
351 FSAEKNWDIS TEHNATILFL KSDM

Spot No.: **29**

NCBI accession No.: **gi|124365253** Species: *Hevea brasiliensis*

Protein name: beta-1,3-glucanase

Mascot score: **109** Sequence coverage %:**38**

Matched peptides No.: **15** Total peptides No.: **35**

Calculated Mr: **41543** Calculated *p*I: **9.40**

Probability Based Mowse Score:

Protein score is -10*Log(P), where P is the probability that the observed match is a random event. Protein scores greater than 73 are significant ($p<0.05$).

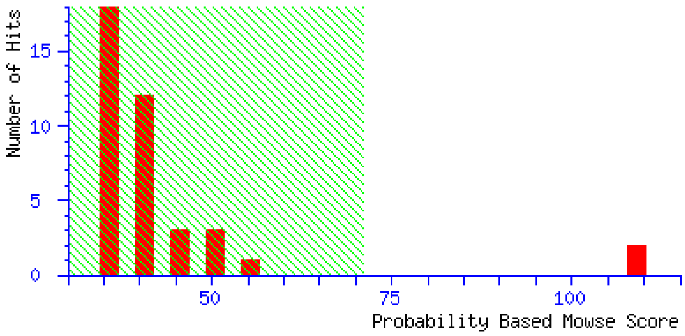

Matched peptide sequences: shown in Bold Red:

1 MAISSSTSGT SSSLPSRTTV MLLLFFFTAS VGITDAQVGV CYGMQGNNLP
51 PVSEVIALYK KSNITRMR**IY** **DPNQAVLEAL** **R**GSNIELILG VPNSDLQSLT
101 NPSNAKSWVQ KNVRGFWSSV RFR**YIAVGNE** **ISPVNR**GTAW LAQFVLPAMR
151 **NIHDAIRSAG** **LQDQIKVSTA** **IDLTLVGNSY** **PPSAGAFRDD** **VRSYLNPIIR**
201 **FLSSIRSPLL** **ANIYPYFTYA** **GNPR**DISLPY ALFTSPSVVV WDGQRGYK**NL**
251 **FDATLDALYS** **ALER**ASGGSL EVVVSESGWP SAGAFAATFD NGR**TYLSNLI**
301 **QHVK**RGTPKR PK**RAIETYLF** **AMFDENKK**QP EVEKHFGLFF PNKWQKYNLN
351 FSAEKNWDIS TEHNATILFL KSDM

Spot No.: **30**

NCBI accession No.: **gi|255540341** Species: *Ricinus communis*

Protein name: **glyceraldehyde 3-phosphate dehydrogenase, putative**

Mascot score: **86** Sequence coverage %:**42**

Matched peptides No.: **14** Total peptides No.: **57**

Calculated Mr: **36930** Calculated *p*I: **7.10**

Probability Based Mowse Score:

Protein score is -10*Log(P), where P is the probability that the observed match is a random event.
Protein scores greater than 73 are significant (p<0.05).

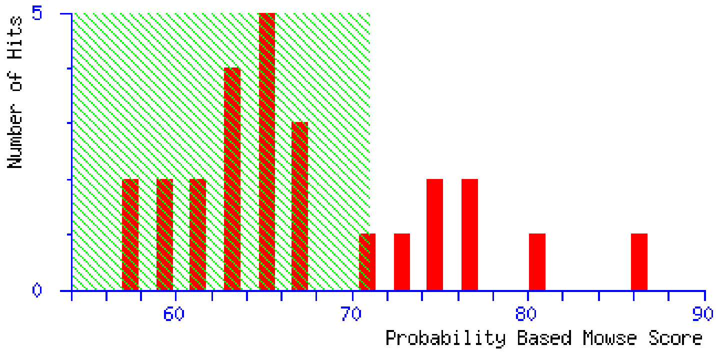

Matched peptide sequences: shown in Bold Red:

1	MAKIKIGING	FGRIGRLVAR	VALQRDDVEL	VAVNDPFIST	EYMTYMFKYD
51	TVHGQWKHHE	LKIKDDKTLL	FGEKPVTVFG	VRNPEEIPWA	ETGAEYIVES
101	TGVFTDKDKA	AAHLKGGAKK	VIISAPSKDA	PMFVVGVNEK	EYTPDLHIIS
151	NASCTTNCLA	PLAKVINDRF	GIVEGLMTTV	HSITATQKTV	DGPSMKDWRG
201	GRAASFNIIP	SSTGAAKAVG	KVLPSLNGKL	TGMSFRVPTV	DVSVVDLTVR
251	LEKSATYEQI	KAAIKEESEG	KLKGILGYTE	DDVVSTDFIG	DKRSSIFDAK
301	AGIALNDKFV	KLVSWYDNEW	GYSTRVVDLI	VHVASVH	

Spot No.: **31**

NCBI accession No.: **gi|4539543** Species: *Nicotiana tabacum*

Protein name: glyceraldehyde-3-phosphate dehydrogenase

Mascot score: **121** Sequence coverage %:**47**

Matched peptides No.: **19** Total peptides No.: **89**

Calculated Mr: **36816** Calculated *p*I: **7.70**

Probability Based Mowse Score:

Protein score is -10*Log(P), where P is the probability that the observed match is a random event.
Protein scores greater than 73 are significant (p<0.05).

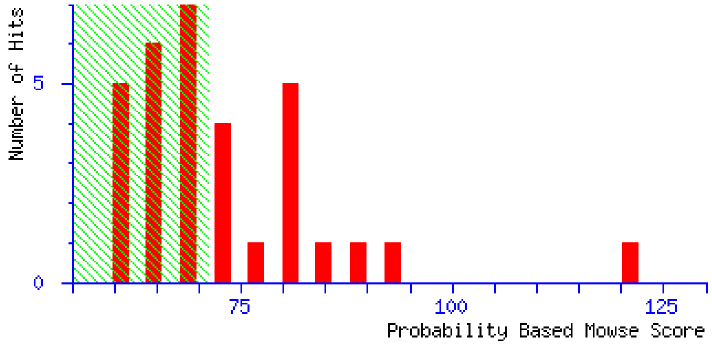

Matched peptide sequences: shown in Bold Red:

1 **MAKVKIGING** **FGR**IGRLVAR **VALQR**DDVEL VAVNDPFISV EYMTYMFKYD
51 SVHGQWKHHE LK**VKDDKTLL** **FGEK**AVTVFG FRNPEEIPWG QTGADYIVES
101 TGVFTDKDKA AAHLKGGAKK VIISAPSK**DA** **PMFVVGVNEK** EYKPELNIVS
151 NASCTTNCLA PLAKVINDR**F** **GIVEGLMTTV** **HSITATQK**TV DGPSAKDWRG
201 GR**AASFNIIP** **SSTGAAK**AVG KVLPALNGK**L** **TGMAFRVPTV** **DVSVVDLTVR**
251 LEKEATYDEI KAAIK**EESEG** **KLKGILGYTE** **DDVVSTDFVG** **DNRSSIFDAK**
301 **AGIALSK**NFV K**LVSWYDNEW** **GYSTR**VVDLI K**HMASVQ**

Spot No.: **32**

NCBI accession No.: **gi|32527831**

Species: *Populus tremula x Populus tremuloides*

Protein name: UDP-glucose pyrophosphorylase

Mascot score: **99**   Sequence coverage %:**40**

Matched peptides No.: **18**   Total peptides No.: **102**

Calculated Mr: **51949**   Calculated *p*I: **5.68**

Probability Based Mowse Score:

Protein score is -10*Log(P), where P is the probability that the observed match is a random event.
Protein scores greater than 73 are significant (p<0.05).

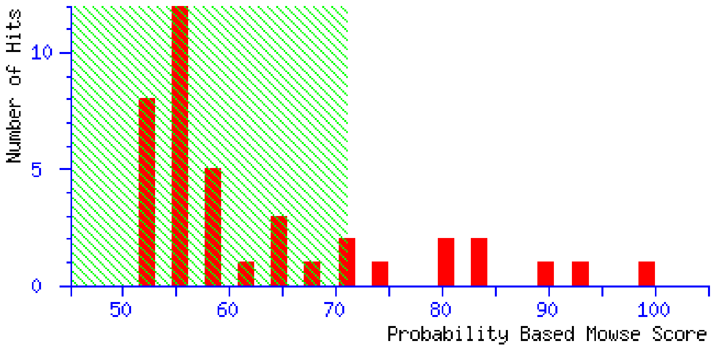

Matched peptide sequences: shown in Bold Red:

1 MATDTEKISQ LKSAVANLNQ ISESEKTGFV NLVSR**YLSGE** **AQQVEWSK**IQ
51 TPTDEVVVPY DTLESTPEDP EETKMLLDKL VVLK**LNGGLG** **TTMGCTGPKS**
101 **VIEVR**NGLTF LDLIVIQIES LNKKYGCSVP LLLMNSFNTH DDTQKIIEKY
151 SNSNIEIHTF NQSQYPR**LVA** **DDFVPLPSK**G HTDK**DGWYPP** **GHGDVFPSLK**
201 NSGKLDALLS QGKEYVFVAN SDNLGAVVDL KILNHLIR**NK** **NEYCMEVTPK**
251 **TLADVKGGTL** **ISYEGKVQLL** **EIAQVPDQHV** **NEFK**SIEKFK **IFNTNNLWVN**
301 **LKAIK**R**LVEA** **DALKMEIIPN** **PK**EVDGVK**VL** **QLETAAGAAI** **RFFDHAIGIN**
351 **VPR**SR**FLPVK** ASSDLLLVQS DIYTPVVDGF VIR**NPGRANP** **ANPSIELGPE**
401 **FK**KVASFLSR FKSIPSIIEL DSLKVAGDVW FGVNVTLKGK VSIVVKSGVK
451 LEIPEGVVLE NKEINGPKDL

Spot No.: **33**

NCBI accession No.: **gi|6707018**   Species: *Hevea brasiliensis*

Protein name: latex protein allergen Hev b 7

Mascot score: **171**   Sequence coverage %:**79**

Matched peptides No.: **26**   Total peptides No.: **67**

Calculated Mr: **43107**   Calculated *p*I: **5.00**

Probability Based Mowse Score:

Protein score is -10*Log(P), where P is the probability that the observed match is a random event.
Protein scores greater than 73 are significant (p<0.05).

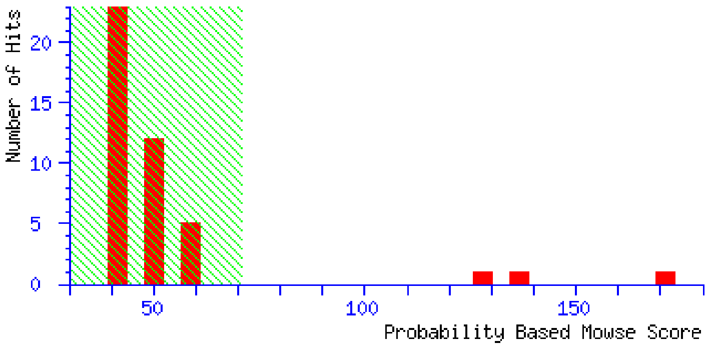

Matched peptide sequences: shown in Bold Red:

```
  1 MATGSTPLTQ GKKITVLSID GGGIRGIIPG IILASLESKL QDLDGPDARI
 51 ADYFDIIAGT STGGLITTML TAPNEDKKPM YQAKDIKDFY LENCPKIFPK
101 ESRDNYDPIH SIGPIYDGEY LRELCNNLLK DLTVKDTLTD VIIPTFDIKL
151 LLPVIFSSDD AKCNALKNAR LADVCISTSA APVLLPAHSF TTEDDKNIHT
201 FELIDGGVAA TNPTLLALTH IRNEIIRQNP RFIGANLTES KSRLVLSLGT
251 GKSEYKEKYN ADMTSKWRLY NWALYNGNSP AVDIFSNASS DMVDFHLSAL
301 FKSLDCEDYY LRIQDDTLTG EESSGHIATE ENLQRLVEIG TELLEKQESR
351 INLDTGRLES IPGAPTNEAA IAKFAKLLSE ERKLRQLK
```

Spot No.: **34**

NCBI accession No.: **gi|158144895** Species: *Gossypium hirsutum*

Protein name: enolase

Mascot score: **123** Sequence coverage %: **39**

Matched peptides No.: **14** Total peptides No.: **48**

Calculated Mr: **47982** Calculated *p*I: **5.49**

Probability Based Mowse Score:

Protein score is -10*Log(P), where P is the probability that the observed match is a random event. Protein scores greater than 73 are significant (p<0.05).

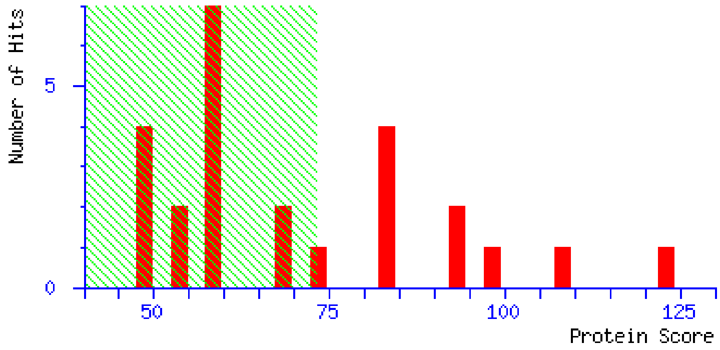

Matched peptide sequences: shown in Bold Red:

1 MAATIVSVKA RQIFDSRGNP TVEVDVETSN GIKARAAVPS GASTGIYEAL
51 ELRDGDFDYL GKGVSKAVAN VNTIIGPALI GKDPTEQTAI DNFMVQQLDG
101 TQNEWGWCKQ KLGANAILAV SLAVCKAGAE VKKLPLYKHI ANLAGNSKLV
151 LPVPAFNVIN GGSHAGNKLA MQEFMILPVG ASSFKEAMKM GVEVYHHLKS
201 VIKKKYGQDA TNVGDEGGFA PNIQENKEGL ELLNTAIAKA GYTGKVVIGM
251 DVAASEFYGT DKTYDLNFKE ENNDGKQKIS GDALKDLYKS FVAEYPIVSI
301 EDPFDQDDWE HYSKLTNEIG EKVQIVGDDL LVTNPKRVAK AIAEKTCNAL
351 LLKVNQIGSV TESIEAVKMS KQAGWGVMAS HRSGETEDTF IADLSVGLAT
401 GQIKTGAPCR SERLAKYNQL LRIEEELGAE AVYAGASFRA PVAPY

Spot No.: 35

NCBI accession No.: **gi|3288200** Species: *Hevea brasiliensis*

PFF score: [81]

Protein name： **latex allergen**

Matched peptides No.: [2] Sequence coverage %: [4]

Matched sequences: R.DTYDPIHSIGPIYDGEYLR.E；

R.DTYDPIHSIGPIYDGEYLR.E

Calculated Mr: **43020** Calculated *p*I: **5.00**

Probability Based Mowse Score:

Ions score is -10*Log(P), where P is the probability that the observed match is a random event.
Individual ions scores > 43 indicate identity or extensive homology (p<0.05).
Protein scores are derived from ions scores as a non-probabilistic basis for ranking protein hits.

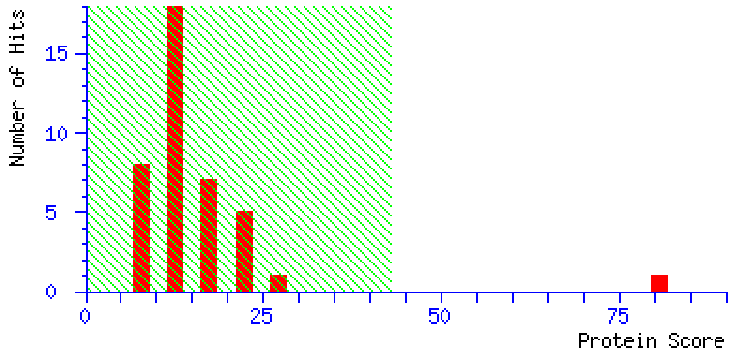

Matched peptide sequences: shown in Bold Red:

```
  1  MATGSTTLTQ  GKKITVLSID  GGGIRGIIPG  IILASLESKL  QDLDGPDARI
 51  ADYFDIIAGT  STGGLITTML  TAPNEDKKPI  YQAKDIKDFY  LENCPKIFPK
101  ESRDTYDPIH  SIGPIYDGEY  LRELCNNLLK  DLTVKDTLTD  VIIPAFDIKL
151  LLPVIFSSDD  AKCNALKNAR  LADVCISTSA  APVLLPAHSF  TTEDDKNIHT
201  FELIDGGVAA  ANPTLLALTH  IRNEIIRQNP  RFIGANLTES  KSRLVLSLGT
251  GKSEYKEKYN  ADMTSKWRLY  NWALYNGNSP  AVDIFSNASS  DMVDFHLSAL
301  FKSLDCEDYY  LRIQDDTLTG  EESSGHIATE  ENLQRLVEIG  TELLEKQESR
351  INLDTGRLES  IPGAPTNEAA  IAKFAKLLSE  ERKLRQLK
```

Spot No.: 36

NCBI accession No.: **gi|1916805**   Species: *Hevea brasiliensis*

PFF score: [138]

Protein name： **latex patatin homolog**

Matched peptides No.: [2]   Sequence coverage %: [8]

Matched sequences: K.SLDCEDYYLR.I；

R.IQDDTLTGEESSGHIATEENLQR.L

Calculated Mr: **43035**   Calculated *p*I: **5.00**

Probability Based Mowse Score:

Ions score is -10*Log(P), where P is the probability that the observed match is a random event.
Individual ions scores > 44 indicate identity or extensive homology (p<0.05).
Protein scores are derived from ions scores as a non-probabilistic basis for ranking protein hits.

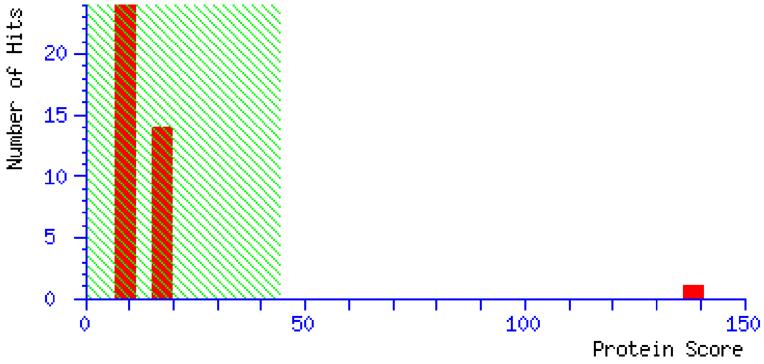

Matched peptide sequences: shown in Bold Red:

1 MATGSTTLTQ GKKITVLSID GGGIRGIIPG IILASLESKL QDLDGPDARI

51 ADYFDIIAGT STGGLITTML TAPNEDKKPM YQAKDIKDFY LENCPKIFPK

101 ESRDNYDPIH SIGPIYDGEY LRELCNNLLK DLTVKDTSTD VIIPTFDIKL

151 LLPVIFPSDD AKCNALKNAR LADVCISTSA APVLLPAHSF TTEDDKNIHT

201 FELIDGGVAA TNPTLLALTH IRNEIIRQNP RFIGANLTES KSRLVLSLGT

251 GKSEYKEKYN ADMTSKWRLY NWALYNGNSP AVDIFSNASS DMVDSHLSAL

301 FK**SLDCEDYY** **LRIQDDTLTG** **EESSGHIATE** **ENLQR**LVEIG TELLEKQESR

351 INLDTGRLES IPGAPTNEAA IAKFAKLLSE ERKLRQLK

Spot No.: 37

NCBI accession No.: **gi|41581137** Species: *Hevea brasiliensis*

Protein name: **putative latex allergen hev b 7.02**

PMF score: **152** Sequence coverage %: **44**

Matched peptides No.: **16** Total peptides No.: **39**

Calculated Mr: **43027** Calculated *p*I: **5.12**

Probability Based Mowse Score:

Protein score is -10*Log(P), where P is the probability that the observed match is a random event.
Protein scores greater than 73 are significant ($p<0.05$).

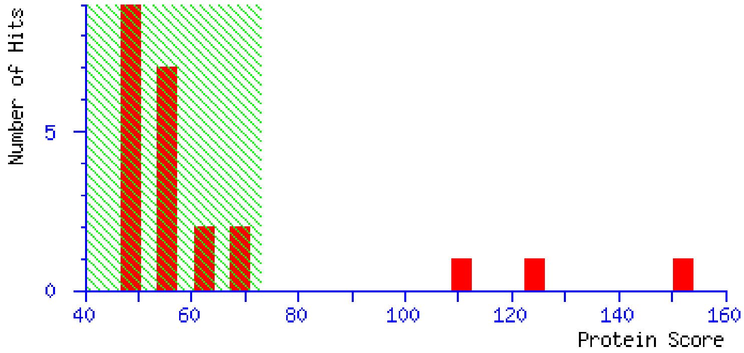

Matched peptide sequences: shown in Bold Red

```
  1 ATGSTTLTQG KKITVLSIDG GGIRGIIPGI ILASLESKLQ DLDGPDARIA
 51 DYFDIIAGTS TGGLITTMLT APNEDKKPMY QAKDIKDFYL ENCPKIFPKE
101 SRDNYDPIHS IGPIYDGEYL RELCNNLLKD LTVKDTLTDV IIPTFDIKLL
151 LPVIFSSDDA KCNALKNARL ADVCISTSAA PVLLPAHSFT TEDDKNIHTF
201 ELIDGGVAAT NPTLLALTHI RNEIIRQNPR FIGANLTESK SRLVLSLGTG
251 KSEYKEKYNA DMTSKWRLYN WALYNGNSPA VDIFSNASSD MVDFHLSALF
301 KSLDCEDYYL RIQDDTLTGE ESSGHIATEE NLQRLVEIGT RLLEKQESRI
351 NLDTGRLESI PGASTNEAAI TKFAKLLSEE RKLRQLK
```

Spot No.: 38

NCBI accession No.: **gi|41581137** Species: *Hevea brasiliensis*

Protein name： **putative latex allergen hev b 7.02**

PMF score: **142** Sequence coverage %: **42**

Matched peptides No.: **15** Total peptides No.: **51**

Calculated Mr: **43027** Calculated *p*I: **5.12**

Probability Based Mowse Score:

Protein score is -10*Log(P), where P is the probability that the observed match is a random event. Protein scores greater than 73 are significant (p<0.05).

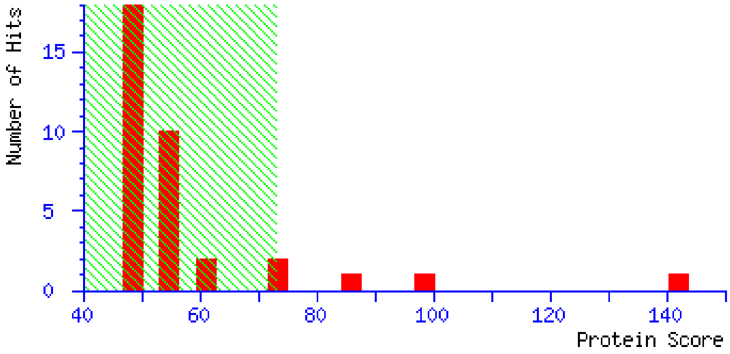

Matched peptide sequences: shown in Bold Red

```
  1  ATGSTTLTQG KKITVLSIDG GGIRGIIPGI ILASLESKLQ DLDGPDARIA
 51  DYFDIIAGTS TGGLITTMLT APNEDKKPMY QAKDIKDFYL ENCPKIFPKE
101  SRDNYDPIHS IGPIYDGEYL RELCNNLLKD LTVKDTLTDV IIPTFDIKLL
151  LPVIFSSDDA KCNALKNARL ADVCISTSAA PVLLPAHSFT TEDDKNIHTF
201  ELIDGGVAAT NPTLLALTHI RNEIIRQNPR FIGANLTESK SRLVLSLGTG
251  KSEYKEKYNA DMTSKWRLYN WALYNGNSPA VDIFSNASSD MVDFHLSALF
301  KSLDCEDYYL RIQDDTLTGE ESSGHIATEE NLQRLVEIGT RLLEKQESRI
351  NLDTGRLESI PGASTNEAAI TKFAKLLSEE RKLRQLK
```

Spot No.: 39

NCBI accession No.: **gi|255556934** Species: *Ricinus communis*

Protein name：**Transitional endoplasmic reticulum ATPase, putative**

PMF score: **136** Sequence coverage %: **31**

Matched peptides No.: **25** Total peptides No.: **59**

Calculated Mr: **90355** Calculated *p*I: **5.13**

Probability Based Mowse Score:

Protein score is -10*Log(P), where P is the probability that the observed match is a random event. Protein scores greater than 73 are significant (p<0.05).

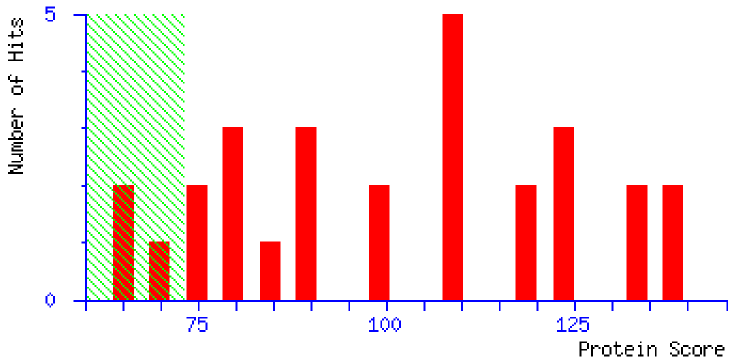

Matched peptide sequences: shown in Bold Red

```
  1 MSNQAESSDS KGTKRDFSTA ILERKKSPNR LVVDEAINDD NSVVSLHPET
 51 MEKLQLFRGD TILIKGKKRK DTICIALADD SCDEPKIRMN KVVRSNLRVR
101 LGDVVSVHQC PDVKYGKRVH ILPIDDTIEG VTGNLFDAYL KPYFLEAYRP
151 VRKGDLFLVR GGMRSVEFKV IETDPAEYCV VAPDTEIFCE GEPVRREDEN
201 RLDEVGYDDV GGVRKQMAQI RELVELPLRH PQLFKSIGVK PPKGILLYGP
251 PGSGKTLIAR AVANETGAFF FCINGPEIMS KLAGESESNL RKAFEEAEKN
301 APSIIFIDEI DSIAPKREKT HGEVERRIVS QLLTLMDGLK SRAHVIVIGA
351 TNRPNSIDPA LRRFGRFDRE IDIGVPDEVG RLEVLRIHTK NMKLAEDVDL
401 ERISKDTHGY VGADLAALCT EAALQCIREK MDVIDLEDET IDAEILNSMA
451 VSNEHFQTAL GTSNPSALRE TVVEVPNVSW EDIGGLENVK RELQETVQYP
501 VEHPEKFEKF GMSPSKGVLF YGPPGCGKTL LAKAIANECQ ANFISVKGPE
551 LLTMWFGESE ANVREIFDKA RQSAPCVLFF DELDSIATQR GSSVGDAGGA
601 ADRVLNQLLT EMDGMSAKKT VFIIGATNRP DIIDPALLRP GRLDQLIYIP
651 LPDEDSRHQI FKACLRKSPV SKDVDLRALA KYTQGFSGAD ITEICQRACK
701 YAIRENIEKD IERERRRRDN PEAMEEDVED DVAEIKAAHF EESMKYARRS
751 VSDADIRKYQ AFAQTLQQSR GFGSEFRFSE ATGAAAGADP FAASAGGEAD
801 DDDLYS
```

Spot No.: 40

NCBI accession No.: **gi|149938964** Species: *Actinidia deliciosa*

Protein name： **ACT1**

PMF score: **125** Sequence coverage %: **57**

Matched peptides No.: **19** Total peptides No.: **56**

Calculated Mr: **41865** Calculated *p*I: **5.31**

Probability Based Mowse Score:

Protein score is -10*Log(P), where P is the probability that the observed match is a random event. Protein scores greater than 73 are significant ($p<0.05$).

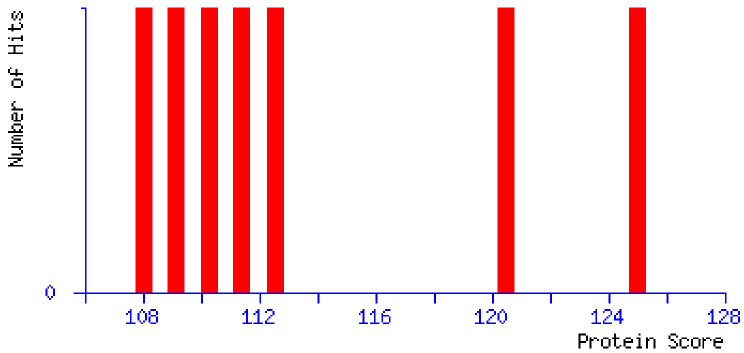

Matched peptide sequences: shown in Bold Red

```
  1 MADAEDIQPL VCDNGTGMVK AGFAGDDAPR AVFPSIVGRP RHTGVMVGMG
 51 QKDAYVGDEA QSKRGILTLK YPIEHGIVSN WDDMEKIWHH TFYNELRVAP
101 EEHPVLLTEA PLNPKANREK MTQIMFETFN VPAMYVAIQA VLSLYASGRT
151 TGIVLDSGDG VSHTVPIYEG YALPHAILRL DLAGRDLTDA LMKILTERGY
201 MFTTTAEREI VRDVKEKLAY VALDYEQELE TAKSSSSVEK NYELPDGQVI
251 TIGAERFRCP EVLFQPSLIG MEAAGIHETT YNSIMKCDVD IRKDLYGNIV
301 LSGGSTMFPG IADRMSKEIT ALAPSSMKIK VVAPPERKYS VWIGGSILAS
351 LSTFQQMWIS KGEYDESGPS IVHRKCF
```

Spot No.: 41

NCBI accession No.: **gi|159489771**

Species: *Chlamydomonas reinhardtii*

Protein name: **predicted protein**

PMF score: **83**

Sequence coverage %: **37**

Matched peptides No.: **13**

Total peptides No.: **42**

Calculated Mr: **41375**

Calculated *p*I: **9.34**

Probability Based Mowse Score:

Protein score is -10*Log(P), where P is the probability that the observed match is a random event. Protein scores greater than 73 are significant (p<0.05).

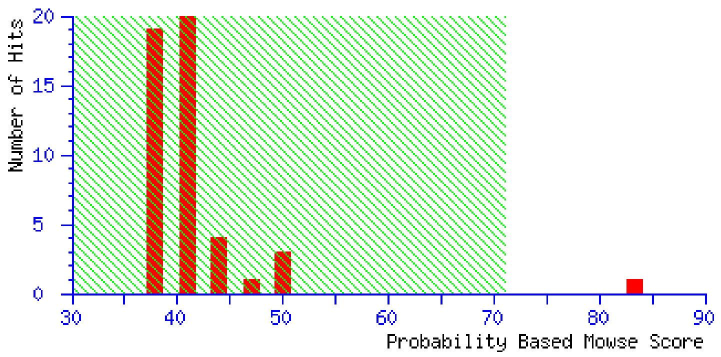

Matched peptide sequences: shown in Bold Red

```
  1 SGAAFIKWGQ WAATRVDLFP DDFCDTLANL HDRAPTHSFA FTRKQVERSF
 51 GLALERMFES FEQQPVASGS IAQIHRAVMR GSDGSRLQVA VKVVHPRVAL
101 RIRQDFALLR PAAAALGRLR SLRSLSLPET VSQFSATMTA QADLRVEAAH
151 LRRFYANFAA VAAHVTTPRP LPGLVRPEVM VETWEDGRSV SHYIRYPHPS
201 LNGRVVCLGL DTYLKMLLQD NFVHTDLHPG NIMVRANRSS RSSCRRAGVQ
251 LVLLDFGLAE ELSPVVRHHF ISFLQHLLRG DGVAAAGHLL RWTSRRQACA
301 DPAALTADMV ALAATQCDLR SEEGIDLDKV MKDVLRLARK HGVTIDSCYA
351 ALVIAVCVIV GFATSLDPGV NL
```

Spot No.: 42

NCBI accession No.: **gi|14423688**

Species: *Hevea brasiliensis*

Protein name：**Enolase 1; AltName: Full=2-phospho-D-glycerate hydro-lyase 1; AltName: Full=2-phosphoglycerate dehydratase 1; AltName: Allergen=Hev b 9**

PMF score: **97** Sequence coverage %: **39**

Matched peptides No.: **14** Total peptides No.: **24**

Calculated Mr: **48029** Calculated *p*I: **5.57**

Probability Based Mowse Score:

Protein score is -10*Log(P), where P is the probability that the observed match is a random event. Protein scores greater than 73 are significant (p<0.05).

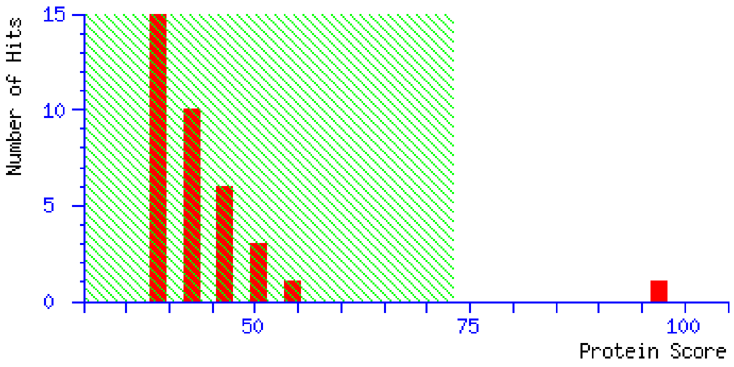

Matched peptide sequences: shown in Bold Red

```
  1 MAITIVSVRA RQIFDSRGNP TVEADVKLSD GYLARAAVPS GASTGIYEAL
 51 ELRDGGSDYL GKGVSKAVEN VNIIIGPALV GKDPTDQVGI DNFMVQQLDG
101 TVNEWGWCKQ KLGANAILAV SLAVCKAGAH VKGIPLYEHI ANLAGNKNLV
151 LPVPAFNVIN GGSHAGNKLA MQEFMILPVG ASSFKEAMKM GAEVYHHLKS
201 VIKKKYGQDA TNVGDEGGFA PNIQENKEGL ELLKTAIAKA GYTGKVVIGM
251 DVAASEFYGS DQTYDLNFKE ENNNGSQKIS GEALKDLYKS FVAEYPIVSI
301 EDPFDQDDWA HYAKLTSEIG EKVQIVGDDL LVTNPKRVEK AIKEKACNAL
351 LLKVNQIGSV TESIEAVKMS KRAGWGVMAS HRSGETEDTF IADLSVGLAT
401 GQIKTGAPCR SERLAKYNQL LRIEEELGSE AVYAGANFRK PVEPY
```

Spot No.: 44

NCBI accession No.: gi|382929047 Species: *Hypselodelphys hirsuta*

Protein name： maturase K (chloroplast)

PMF score: **82** Sequence coverage %: **28**

Matched peptides No.: **15** Total peptides No.: **41**

Calculated Mr: **62643** Calculated *p*I: **9.61**

Probability Based Mowse Score:

Protein score is -10*Log(P), where P is the probability that the observed match is a random event.
Protein scores greater than 73 are significant (p<0.05).

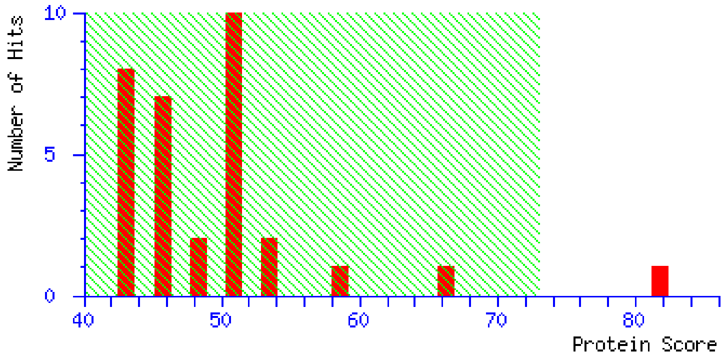

Matched peptide sequences: shown in Bold Red

```
  1 MEELQRYLEK DRSRQQHFLY PLLFQEYIYV FAYDHGLTSS IFYESVKLKS
 51 LGYDNKSSSI LVKRLIIRMY QQNFLIYSVI YSVNDSYQNR FVRHNNYFYS
101 HFFSQMISEG FAVIVEIPFS LQLVSSLEEK EIPKSHNLQS IHSVFPFLED
151 KLLHLNYLSD ILIPHFIHIE ILVQILKSWI QDVPSLHLLR FFLHEDYNWN
201 NLITPKKSIY VFSKENKRIF WVLYNSYLSE CEFVFIFLRK QSSYLRLTSS
251 GVFLERTYFY GKIEHLLVCR IFFQKTLWVF KDPFMHYVRY QGKAILGSRG
301 THSLMKKWKY YLVNFWQYHF HFWSQSCRIH INQLSKYSFY FLGYLSSVLI
351 NSLAVRNQML ENSFLMETLT RKFDTIIPRI PLIRSLFKAK FCTVSGHPIS
401 KPIWTDLSDC DIINRFGRVC RNLSHYHSGS SKKQSLYRMK YILRLSCART
451 LARKHKSTVR SFLQRLSSGL LEEFFTEEKQ VLSLIFPKIT FFSLYRSSRE
501 RIWYLDIIRI NDLVNNL
```

Spot No.: 45

NCBI accession No.: gi|4469175　　Species: *Hevea brasiliensis*

Protein name：beta-1,3-glucanase

PMF score: **156**　　Sequence coverage %: **55**

Matched peptides No.: **19**　　Total peptides No.: **37**

Calculated Mr: **38930**　　Calculated *p*I: **9.45**

Probability Based Mowse Score:

Protein score is -10*Log(P), where P is the probability that the observed match is a random event.
Protein scores greater than 73 are significant (p<0.05).

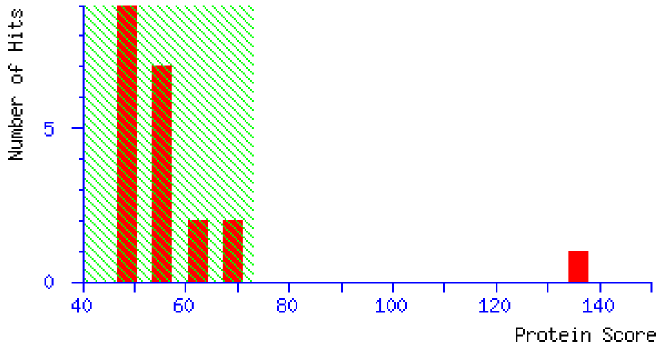

Matched peptide sequences: shown in Bold Red

```
  1 MAISSSTSGT SSSLPSRTTV MLLLFFFAAS VGITDAQVGV CYGMQGNNLP
 51 PVSEVIALYK KSNITRMRIY DPNRAVLEAL RGSNIELILG VPNSDLQSLT
101 NPSNAKSWVQ KNVRGFWSSV LFRYIAVGNE ISPVNRGTAW LAQFVLPAMR
151 NIHDAIRSAG LQDQIKVSTA IDLTLVGNSY PPSAGAFRDD VRSYLNPIIR
201 FLSSIRSPLL ANIYPYFTYA GNPRDISLPY ALFTSPSVVV WDGQRGYKNL
251 FDATLDVLYS ALERASGGSL EVVVSESGWP SAGAFAATFD NGRTYLSNLI
301 QHVKRGTPKR PNRAIETYLF AMFDENKKQP EVEKQFGLFF PDKWQKYNLN
351 FG
```

Spot No.: 46

NCBI accession No.: gi|4469175 Species: *Hevea brasiliensis*

Protein name： beta-1,3-glucanase

PMF score: **126** Sequence coverage %: **55**

Matched peptides No.: **19** Total peptides No.: **37**

Calculated Mr: **38930** Calculated *p*I: **9.45**

Probability Based Mowse Score:

Protein score is -10*Log(P), where P is the probability that the observed match is a random event.
Protein scores greater than 73 are significant (p<0.05).

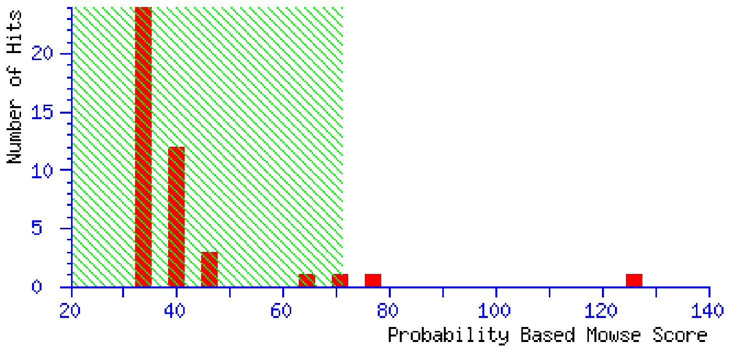

Matched peptide sequences: shown in Bold Red

```
  1 MAISSSTSGT SSSLPSRTTV MLLLFFFAAS VGITDAQVGV CYGMQGNNLP
 51 PVSEVIALYK KSNITRMRIY DPNRAVLEAL RGSNIELILG VPNSDLQSLT
101 NPSNAKSWVQ KNVRGFWSSV LFRYIAVGNE ISPVNRGTAW LAQFVLPAMR
151 NIHDAIRSAG LQDQIKVSTA IDLTLVGNSY PPSAGAFRDD VRSYLNPIIR
201 FLSSIRSPLL ANIYPYFTYA GNPRDISLPY ALFTSPSVVV WDGQRGYKNL
251 FDATLDVLYS ALERASGGSL EVVVSESGWP SAGAFAATFD NGRTYLSNLI
301 QHVKRGTPKR PNRAIETYLF AMFDENKKQP EVEKQFGLFF PDKWQKYNLN
351 FG
```

Spot No.: 47

NCBI accession No.: gi|225438145 Species: *Vitis vinifera*

PFF score: [65]

Protein name： PREDICTED: malate dehydrogenase, cytoplasmic

Matched peptides No.: [1] Sequence coverage %: [3]

Matched sequences: K.IVQGLHIDEFSR.K

Calculated Mr: **35881** Calculated *p*I: **6.18**

Probability Based Mowse Score:

Ions score is -10*Log(P), where P is the probability that the observed match is a random event.
Individual ions scores > 44 indicate identity or extensive homology (p<0.05).
Protein scores are derived from ions scores as a non-probabilistic basis for ranking protein hits.

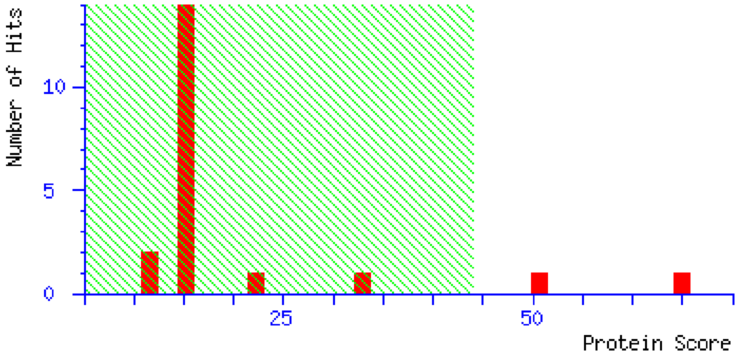

Matched peptide sequences: shown in Bold Red:

```
  1 MAKEPVRVLV TGAAGQIGYA LVPMIARGVM LGADQPVILH MLDIPPAAEA
 51 LNGVKMELVD AAFPLLKGVV ATTDVVEACT GVNIAVMVGG FPRKEGMERK
101 DVMSKNVSIY KSQASALENH AAANCKVLVV ANPANTNALI LKEFAPSIPE
151 KNISCLTRLD HNRALGQVSE RLNVQVSDVK NVIIWGNHSS TQYPDVNHAT
201 VKTPAGEKPV RGLVGDDAWL NGEFITTVQQ RGAAIIKARK LSSALSAASA
251 ACDHIRDWVL GTPEGTWVSM GVYSDGSYNV PAGLIYSFPV TCCAGEWKIV
301 QGLHIDEFSR KKLDLTAQEL SEEKELAYSC LS
```

Spot No.: 48

NCBI accession No.: gi|225438145 Species: *Vitis vinifera*

PFF score: [85]

Protein name： PREDICTED: malate dehydrogenase, cytoplasmic

Matched peptides No.: [1] Sequence coverage %: [3]

Matched sequences: K.IVQGLHIDEFSR.K

Calculated Mr: **35881** Calculated *p*I: **6.18**

Probability Based Mowse Score:

Ions score is -10*Log(P), where P is the probability that the observed match is a random event.
Individual ions scores > 44 indicate identity or extensive homology (p<0.05).
Protein scores are derived from ions scores as a non-probabilistic basis for ranking protein hits.

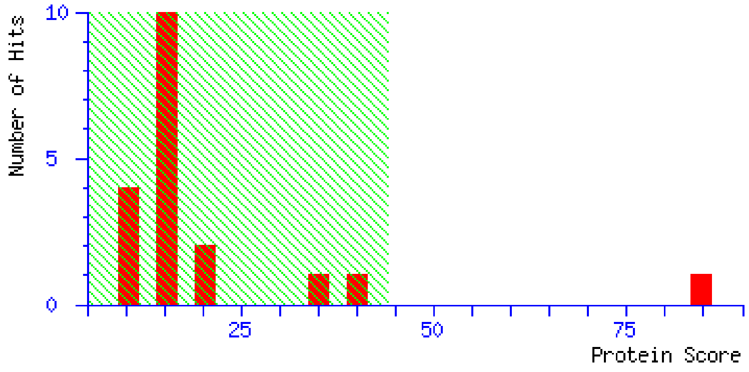

Matched peptide sequences: shown in Bold Red:

1	MAKEPVRVLV	TGAAGQIGYA	LVPMIARGVM	LGADQPVILH	MLDIPPAAEA
51	LNGVKMELVD	AAFPLLKGVV	ATTDVVEACT	GVNIAVMVGG	FPRKEGMERK
101	DVMSKNVSIY	KSQASALENH	AAANCKVLVV	ANPANTNALI	LKEFAPSIPE
151	KNISCLTRLD	HNRALGQVSE	RLNVQVSDVK	NVIIWGNHSS	TQYPDVNHAT
201	VKTPAGEKPV	RGLVGDDAWL	NGEFITTVQQ	RGAAIIKARK	LSSALSAASA
251	ACDHIRDWVL	GTPEGTWVSM	GVYSDGSYNV	PAGLIYSFPV	TCCAGEWK**IV**
301	**QGLHIDEFSR**	KKLDLTAQEL	SEEKELAYSC	LS	

Spot No.: 49

NCBI accession No.: gi|255552291 Species: *Ricinus communis*

PFF score: [75]

Protein name: short chain dehydrogenase, putative

Matched peptides No.: [1] Sequence coverage %: [5]

Matched sequences: K.DPIAIPTDVGFEENCR.K

Calculated Mr: **32148** Calculated *p*I: **6.50**

Probability Based Mowse Score:

Ions score is -10*Log(P), where P is the probability that the observed match is a random event.
Individual ions scores > 45 indicate identity or extensive homology (p<0.05).
Protein scores are derived from ions scores as a non-probabilistic basis for ranking protein hits.

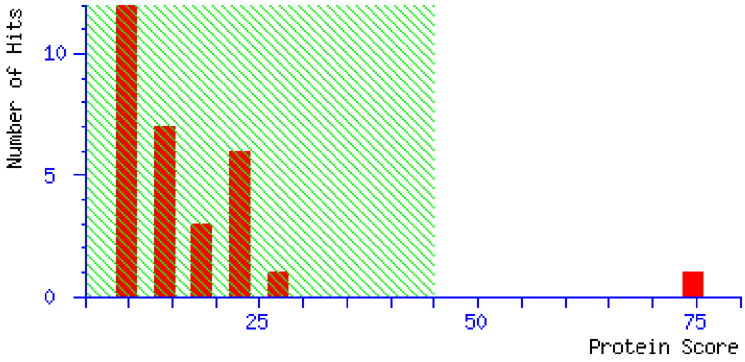

Matched peptide sequences: shown in **Bold Red:**

```
  1 MATRRGYRFP PQSQDKQPGK EYLMHPLPEF INPHYKPSNK LQDKVALVTG
 51 GDSGIGRAVS YYFTLEGATV AFTYVKGRED KDKDHILKIL HEVKAEGAKD
101 PIAIPTDVGF EENCRKVIDQ IMSEYGKIDI LVNNAGEAHY STTIEDITDP
151 MLERVFRTNI FGHFFMSRHA LKHMKEGGCI INTASVAAYA GFSFMVDYSS
201 TKGAVVAFTR SLALQLIDRG IRVNAVAPGS VWTPFQPSTL SAEQVAQVGS
251 GVPMDRAAQP YEIAPSFVFL ASNDCSSYFT GQVLHPNGMF
```

Spot No.: 50

NCBI accession No.: gi|29170601 Species: *Hevea brasiliensis*

PFF score: [240]

Protein name：small rubber particle protein

Matched peptides No.: [2] Sequence coverage %: [28]

Matched sequences: K.ENENFQQEANEQEEK.L；

K.DNSGPLKPGVETIEGVAK.T

Calculated Mr: **12769** Calculated *p*I: **4.61**

Probability Based Mowse Score:

Ions score is -10*Log(P), where P is the probability that the observed match is a random event.
Individual ions scores > 44 indicate identity or extensive homology ($p<0.05$).
Protein scores are derived from ions scores as a non-probabilistic basis for ranking protein hits.

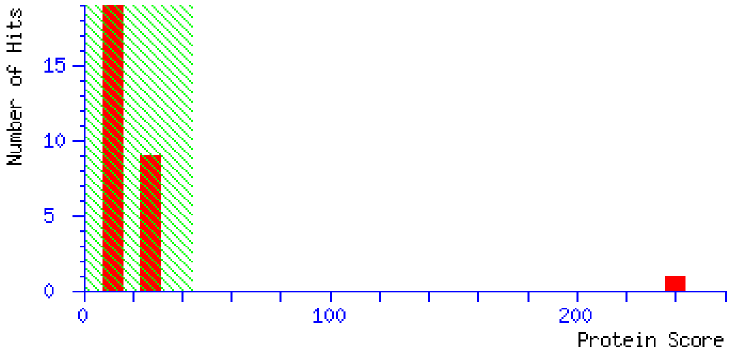

Matched peptide sequences: shown in Bold Red:

```
  1 MAEGKENENF QQEANEQEEK LKYLEFVQAT TDNAVTALSN IYLYAKDNSG
 51 PLKPGVETIE GVAKTVVIPA SKIPTEAIKF ADRAVDASFT TLQNIVPSVL
101 KQLPTQACDT SVKESAE
```

Spot No.: 51

NCBI accession No.: **gi|132270** Species: *Hevea brasiliensis*

Protein name： **Rubber elongation factor protein**

PMF score: **103** Sequence coverage %: **78**

Matched peptides No.: **10** Total peptides No.: **43**

Calculated Mr: **14713** Calculated *p*I: **5.04**

Probability Based Mowse Score:

Protein score is -10*Log(P), where P is the probability that the observed match is a random event. Protein scores greater than 73 are significant ($p<0.05$).

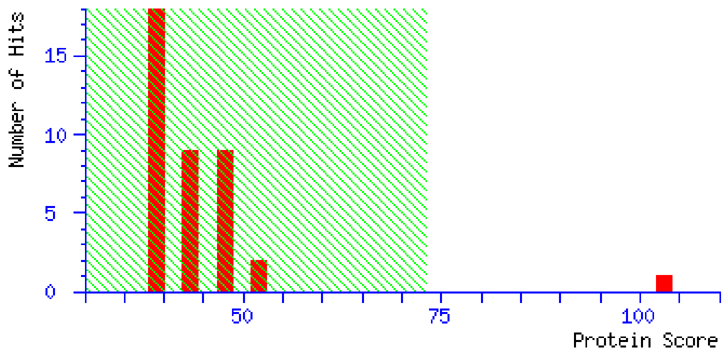

Matched peptide sequences: shown in Bold Red

1 MAEDEDNQQG QGEGLK**YLGF** **VQDAATYAVT** **TFSNVYLFAK** **DKSGPLQPGV**
51 **DIIEGPVKNV** **AVPLYNR**FSY IPNGALK**FVD** **STVVASVTII** **DRSLPPIVKD**
101 **ASIQVVSAIR** **AAPEAARSLA** SSLPGQTKIL AKVFYGEN

Spot No.: 52

NCBI accession No.: **gi|132270** Species: *Hevea brasiliensis*

Protein name： **Rubber elongation factor protein**

PMF score: **115** Sequence coverage %: **78**

Matched peptides No.: **10** Total peptides No.: **33**

Calculated Mr: **14713** Calculated *p*I: **5.04**

Probability Based Mowse Score:

Protein score is -10*Log(P), where P is the probability that the observed match is a random event.
Protein scores greater than 73 are significant (p<0.05).

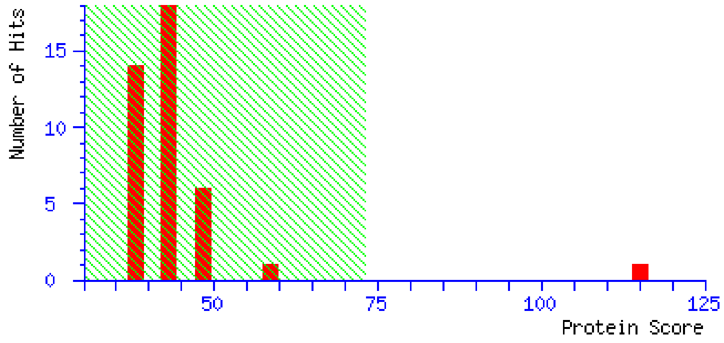

Matched peptide sequences: shown in Bold Red

```
  1 MAEDEDNQQG QGEGLKYLGF VQDAATYAVT TFSNVYLFAK DKSGPLQPGV
 51 DIIEGPVKNV AVPLYNRFSY IPNGALKFVD STVVASVTII DRSLPPIVKD
101 ASIQVVSAIR AAPEAARSLA SSLPGQTKIL AKVFYGEN
```

Spot No.: 53

NCBI accession No.: **gi|115187464** Species: *Arachis hypogaea*

PFF score: [61]

Protein name: **thioredoxin fold**

Matched peptides No.: [1] Sequence coverage %: [4]

Matched sequences: K.HVPGFIER.A

Calculated Mr: **17525** Calculated *p*I: **5.57**

Probability Based Mowse Score:

Ions score is -10*Log(P), where P is the probability that the observed match is a random event.
Individual ions scores > 44 indicate identity or extensive homology ($p<0.05$).
Protein scores are derived from ions scores as a non-probabilistic basis for ranking protein hits.

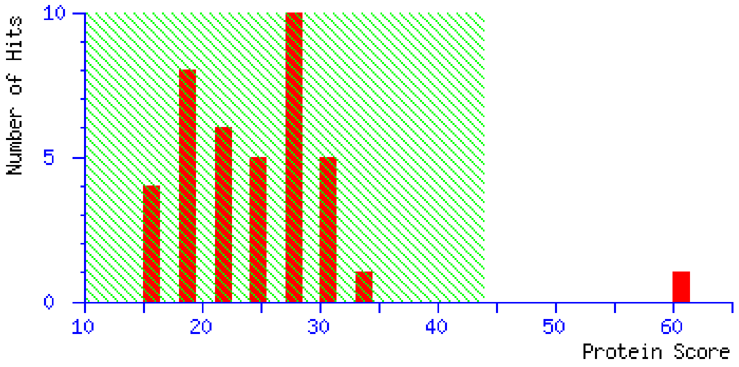

Matched peptide sequences: shown in Bold Red:

```
  1 MAPIAVGDVI PDGTLAFLDN DNKPQSVSIH SLAKGKKVII FGVPGAFTPT
 51 CSLKHVPGFI ERAEELKGKG VDEIICISVN DPFVMKSWAN TFPENKHVTF
101 LADGSAKYTH DLGLELDLSE KGLGIRSKRF ALLVEDLKVK VANIESGGEF
151 TVSSAEEIIK AL
```

Spot No.: 54

NCBI accession No.: **gi|34538473** Species: *Hordeum vulgare*

Protein name： **caleosin 1**

PMF score: **84** Sequence coverage %: **19**

Matched peptides No.: **10** Total peptides No.: **37**

Calculated Mr: **34330** Calculated *p*I: **6.11**

Probability Based Mowse Score:

Protein score is -10*Log(P), where P is the probability that the observed match is a random event.
Protein scores greater than 73 are significant ($p<0.05$).

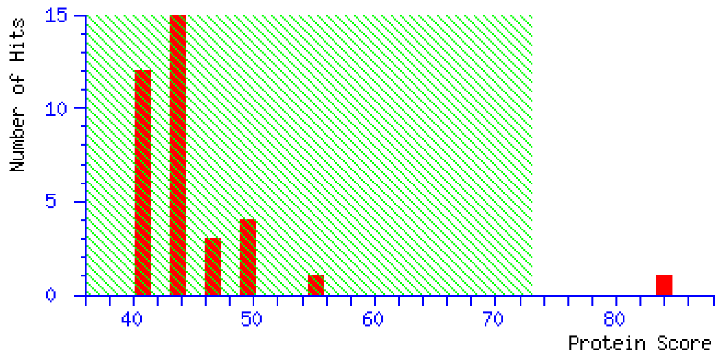

Matched peptide sequences: shown in Bold Red

1 **MATKARK**VEV **RDASRAEGKG** **DAADVHVLRE** **AMRADGK**GDH DTAGGANRAD
51 GHGDAGGRVG DSRGVDGKDS LKMVALQAPV TVERPVRGDL EEHVPKPYLA
101 RALAAPDMYH PEGTTTDDHQ HHNMSVLQQH VAFFDRDNNG IIYPWETYDG
151 CRAVGFNVFM SAFIAFLVNL VMSYPTLPGW LPNPLFPIYV HNIHKSK**HGS**
201 **DSGTYDK**EGR FMPVNFENIF SKYARTYPDR LSYR**EMWR**MT EGCREVFDFF
251 GWVAMKLEWS ILYALARDDE GYLSR**EAIRR** MYDGSLFEYM ERQR**MEHVKM**
301 **S**

Spot No.: 55

NCBI accession No.: **gi|225440278** Species: *Vitis vinifera*

Protein name：**PREDICTED: hypothetical protein**

PMF score: **88** Sequence coverage %: **41**

Matched peptides No.: **7** Total peptides No.: **14**

Calculated Mr: **19226** Calculated *p*I: **9.57**

Probability Based Mowse Score:

Protein score is -10*Log(P), where P is the probability that the observed match is a random event.
Protein scores greater than 73 are significant ($p<0.05$).

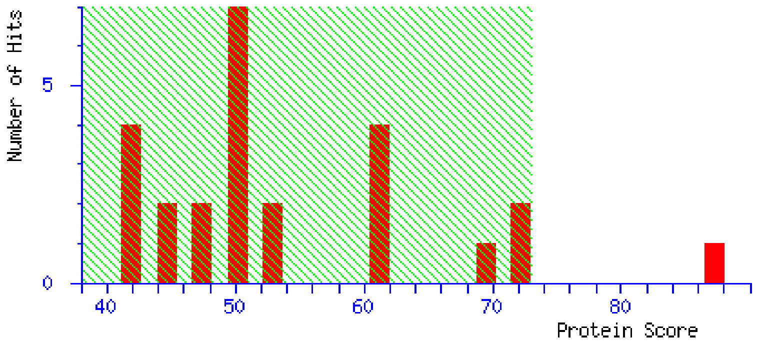

Matched peptide sequences: shown in Bold Red

```
  1 MDHYEALGIG RNASKGEIKE AFRKLALKYH PDKHAQSPKA VRDGATLRFK
 51 QVSEAYEVLS DDRKRSLYNL RSSTAPPGFS GGYAHSYHYN NERPRYSSRP
101 NADAFVSKFE VVLRFLTTRA FLLNVAFAGA LLGGMAIIDR SGETLWKMNN
151 SGKSFDEAME SIGKAKAHKD DM
```

Spot No.: **56**

NCBI accession No.: **gi|2213877** Species: *Hevea brasiliensis*

Protein name： glutamine synthetase

PMF score: **83** Sequence coverage %: **38**

Matched peptides No.: **14** Total peptides No.: **76**

Calculated Mr: **39504** Calculated *p*I: **5.81**

Probability Based Mowse Score:

Protein score is -10*Log(P), where P is the probability that the observed match is a random event. Protein scores greater than 73 are significant (p<0.05).

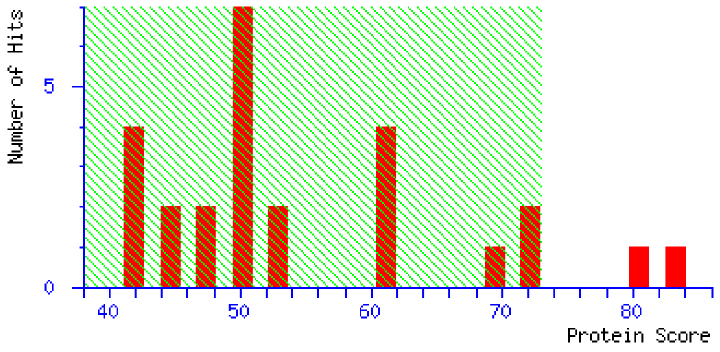

Matched peptide sequences: shown in Bold Red:

```
  1 MSLLSDLINL NLSDTTDKII AEYIWIGGSG MDMRSKARTL SGPVSDPSKL
 51 PKWNYDGSST GQAPGEDSEV ILYPQAIFKD PFRRGNNILV MCDAYTPAGE
101 PIPTNKRHAA AKIFSHPDVV AEEPWYGIEQ EYTLLQRDVK WPIGWPLGGY
151 PGPQGPYYCS IGADKSFGRD VVDSHYKSCL YAGINISGIN GEVMPGQWEF
201 QVGPAVGISA GDELWVARYI LERITEIAGV VLSFDPKPIQ GDWNGAGAHT
251 NYSTKSMRHD GGYEVIKKAI EKLGLRHKEH IAAYGEGNER RLTGRHETAD
301 INTFLWGVAN RGASIRVGRD TEKQGKGYFE DRRPASNMDP YVVTSMIAET
351 TILWKP
```

Spot No.: **57**

NCBI accession No.: **gi|172050753**   Species: *Capparis spinosa*

Protein name： 70 kDa heat shock protein

PMF score: **102**   Sequence coverage %: **36**

Matched peptides No.: **19**   Total peptides No.: **58**

Calculated Mr: **71456**   Calculated *p*I: **5.21**

Probability Based Mowse Score:

Protein score is -10*Log(P), where P is the probability that the observed match is a random event. Protein scores greater than 73 are significant (p<0.05).

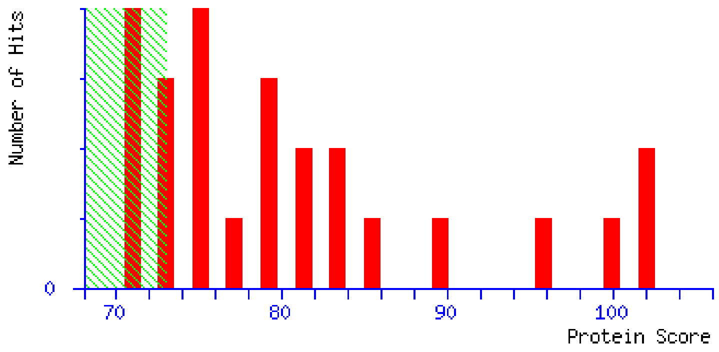

Matched peptide sequences: shown in Bold Red:

1 MAGKGEGPAI GIDLGTTYSC VGVWQHDR**VE** **IIANDQGNRT** **TPSYVAFTDT**
51 **ER**LIGDAAKN QVAMNPINTV FDAKRLIGRR YSDSSVQADM KLWPFKVVPG
101 SGDKPMIVVN YKGEEKQFAA EEISSMVLIK **MREIAEAFLG** **CTIK**NGVVTV
151 PAYFNDSQRQ ATK**DAGVIAG** **LNVMRIINEP** **TAAAIAYGLD** **K**KAASVGEKN
201 VLIFDLGGGT FDVSLLTIEE GIFEV**KATAG** **DTHLGGEDFD** **NRMVNHFVQE**
251 **FK**RKNKRDIS GNPRALRR**LR** **TACERAKRTL** **SSTAQTTIEI** **DSLYEGIDFY**
301 **TTITRARFEE** **LNMDLFRKCM** **EPVEK**CLRDA KMDKSNVHDI VLVGGSTRIP
351 **KVQQLLQDFF** **NGKELCKSIN** **PDEAVAYGAA** **VQAAILSGEG** **NEK**VQDLLLL
401 DVTPLSLGLE TAGGVMTVLI PRNTTIPTKK EQIFSTYSDN QPGVLIQVYE
451 GERARTK**DNN** **LLGKFELTGI** **PPAPRGVPQI** **NVCFDIDANG** **ILNVSAEDKT**
501 TGQKNKITIT NDKGRLSKEE IEKMVQEAEK YKSEDEEHKK KVEAK**NALEN**
551 **YAYNMR**NTIK DEKIGSKLDP ADKKIEDAI DQAIQWLDGN QLAEADEFKD
601 KMKELESICN PIIARMYQGA GGDMGGAGGA DDASPAGGSG AGPKIEEVD

Spot No.: **58**

NCBI accession No.: **gi|762844** Species: *Solanum lycopersicum*

PFF score: [151] Protein name： Hsc70

Matched peptides No.: [3] Sequence coverage %: [10]

Matched sequences: K.ATAGDTHLGGEDFDNR.M;
R.TLSSTAQTTIEIDSLYEGIDFYTTITR.A;
K.EQVFSTYSDNQPGVLIQVYEGER.A

Calculated Mr: **71869** Calculated *p*I: **5.18**

Probability Based Mowse Score:

Ions score is -10*Log(P), where P is the probability that the observed match is a random event.
Individual ions scores > 45 indicate identity or extensive homology (p<0.05).
Protein scores are derived from ions scores as a non-probabilistic basis for ranking protein hits.

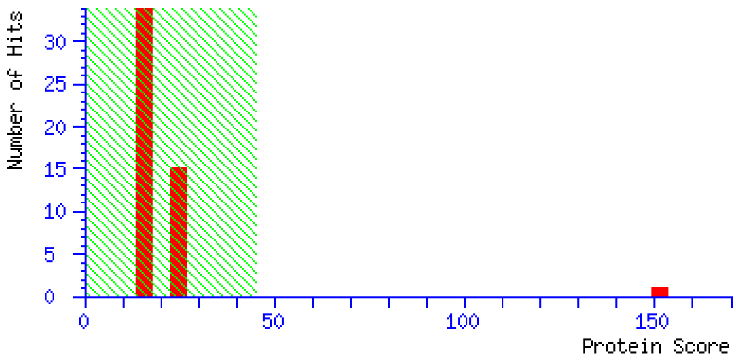

Matched peptide sequences: shown in Bold Red:

1 MAGKGEGPAI GIDLGTTYSC VGVWQHDRVE IIANDQGNRT TPSYVAFTDT
51 ERLIGDAAKN QVAMNPTNTV FDAKRLIGRR FSDPSVQSDM KLWPFKVIPG
101 PADKPMIVVN YKGEEKQFSA EEISSMVLIK MKEIAEAFLG TTIKNAVVTV
151 PAYFNDSQRQ ATKDAGTISG LNVMRIINEP TAAAIAYGLD KKISSTGEKT
201 VLIFDLGGGT FDVSLLTIEE GIFEVK**ATAG** **DTHLGGEDFD** **NR**MVNHFVQE
251 FKRKHKKDIS GNPRALRRLR TACERAKR**TL** **SSTAQTTIEI** **DSLYEGIDFY**
301 **TTITR**ARFEE LNMDLFRKCM EPVEKCLRDA KIDKSGVHDI VLVGGSTRIP
351 KVQQLLQDFF NGRELCKSIN PDEAVAYGAA VQAAILSGEG NEKVQDLLLL
401 DVTPLSLGLE TAGGVMTTLI PRNTTIPTKK **EQVFSTYSDN** **QPGVLIQVYE**
451 **GER**ARTRDNN LLGKFELTGI PPAPRGVPQI TVCFDIDANG ILNVSAEDKT
501 TGQKNKITIT NDKGRLSKDE IEKMVQEAEK YKAEDEEHKK KVEAKNALEN
551 YAYNMRNTIK DDKIASKLSP DEKKKIEDSV EQAIQWLDGN QLAEADEFED
601 KMKELESICN PIIAKMYQGR AGPDMAGGMD EDGPSAGASS AGAGPKIEEV
651 D

Spot No.: **59**

NCBI accession No.: **gi|356552478** Species: *Glycine max*

Protein name：PREDICTED: heat shock cognate protein 80-like

Mascot score: **107** Sequence coverage %: **25**

Matched peptides No.: **21** Total peptides No.: **54**

Calculated Mr: **80422** Calculated *p*I: **4.98**

Probability Based Mowse Score:

Protein score is -10*Log(P), where P is the probability that the observed match is a random event. Protein scores greater than 73 are significant ($p<0.05$).

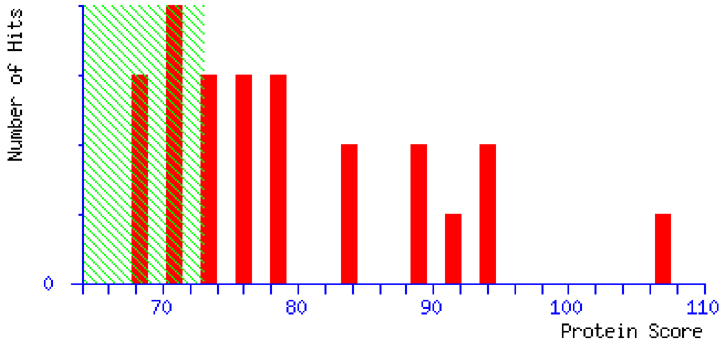

Matched peptide sequences: shown in Bold Red:

```
  1 MASETETFAF QAEINQLLSL IINTFYSNKE IFLRELISNA SDALDKIRFE
 51 SLTDKSKLDA QPELFIHIIP DKTNNTLSII DSGIGMTKAD LVNNLGTIAR
101 SGTKEFMEAL AAGADVSMIG QFGVGFYSAY LVADKVIVTT KHNDDEQYVW
151 ESHAGGSFTV TRDTSGENLG RGTKITLFLK EDQLEYLEER RLKDLIKKHS
201 EFISYPISLW IEKTTEKEIS DDEDEEEKKD EEGKVEDVDE DKEKEEKKKK
251 TIKEVSHEWS LVNKQKPIWM RKPEEITKEE YAAFYKSLTN DWEEHLAVKH
301 FSVEGQLEFK AVLFIPKRAP FDLFDTRKKP NNIKLYVRRV FIMDNCEELM
351 PEYLSFVKGI VDSEDLPLNI SREMLQQNKI LKVIRKNLVK KCIEMFFEIA
401 ENKEDYNKFY EAFSKNLKLG IHEDSQNKTK LAELLRYHST KSGDEMTSLK
451 DYVTRMKEGQ NDIYYITGES KKAVENSPFL EKLKKKGYEV LYMVDAIDEY
501 AVGQLKEFEG KKLVSATKEG LKLDESEDEK KKKEELKDKF EGLCHVIKDV
551 LGDKVEKVVV SDRVVDSPCC LVTGEYGWTA NMERIMKAQA LRDSSMAGYM
601 SSKKTMEINP ENPIMEELRK RADADKNDKS VKDLVLLLFE TALLTSGFSL
651 DDPNTFGNRI HRMLKLGLSI DEDAGEADAD MPPLEDADAD AEGSKMEEVD
```

Spot No.: **60**

NCBI accession No.: **gi|356510919** Species: *Glycine max*

PFF score: [51]

Protein name： PREDICTED: UDP-glycosyltransferase 75D1-like

Matched peptides No.: [1] Sequence coverage %: [2]

Matched sequences: K.CVEEVMGSGELR.R

Calculated Mr: **52131** Calculated *p*I: **5.07**

Probability Based Mowse Score:

Ions score is -10*Log(P), where P is the probability that the observed match is a random event.
Individual ions scores > 45 indicate identity or extensive homology (p<0.05).
Protein scores are derived from ions scores as a non-probabilistic basis for ranking protein hits.

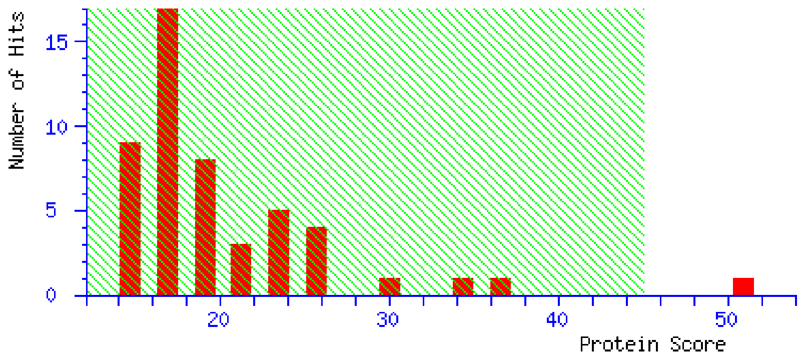

Matched peptide sequences: shown in Bold Red:

```
  1 MLHHRFLLVI YPGQGQINPA LQFAKRLTAM GARVTIPITL DMHRRMTNTT
 51 AVPGLSLAPF SDGYDDGFHA IRGTDSDYNL YASELKRRAS VFVSNLILSS
101 ANEGHPFTCL LYTLLVPWAP QVARGLNLPT AMLWIQPATV LDILYHYFHG
151 YADYINDETK ENIVLPGLSF SLSPRDIPSF LLTSKPSLLS FVFPLFEEQI
201 KQLDLEANPK VLVNTFEALE EEALRAVDKL NMIPIGPLIP TAFLGGKDPE
251 DTSFGGDLLQ VSNGYVEWLD SKEDKSVVYV SFGSYFELSK RQTEEIARAL
301 LGCSFPFLWV IRVKEEEKEE EEELCFREEL EGKGKLVKWC SQVEVLSHGS
351 VGCFVTHCGW NSTMESLVSG VPMVAFPQWS DQKTNAKLIE DVWKIGVRVE
401 NDGDGIVEKE EIRKCVEEVM GSGELRRNAE KWKGLAREAA KEGGPSERNL
451 KAFLDAMAAL QF
```

Spot No.: **61**

NCBI accession No.: **gi|255560725** Species: *Ricinus communis*

Protein name： dead box ATP-dependent RNA helicase, putative

Mascot score: **144** Sequence coverage %: **68**

Matched peptides No.: **25** Total peptides No.: **91**

Calculated Mr: **47097** Calculated *p*I: **5.49**

Probability Based Mowse Score:

Protein score is -10*Log(P), where P is the probability that the observed match is a random event.
Protein scores greater than 73 are significant (p<0.05).

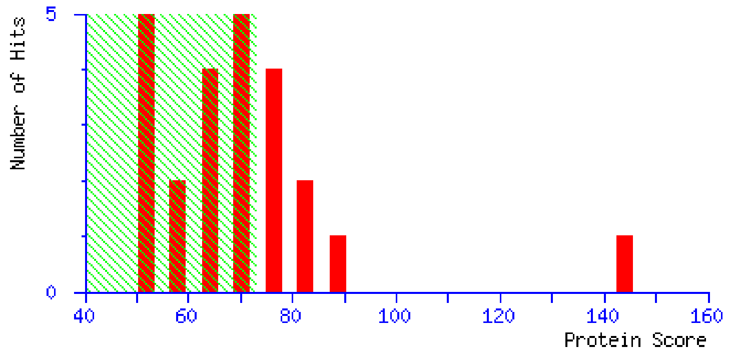

Matched peptide sequences: shown in Bold Red:

```
  1 MAGLAPEGSQ FDAKQYDAKM SELLSTEGQE FFTTYDEVYD SFDAMGLQEN
 51 LLRGIYAYGF EKPSAIQQRG IVPFCKGLDV IQQAQSGTGK TATFCSGILQ
101 QLDYGLVQCQ ALVLAPTREL AQQIEKVMRA LGDYLGVKVH ACVGGTSVRE
151 DQRILQAGVH VVVGTPGRVF DMLRRQSLRP DYIKMFVLDE ADEMLSRGFK
201 DQIYDIFQLL PAKVQVGVFS ATMPPEALEI TRKFMNKPVR ILVKRDELTL
251 EGIKQFYVNV EKEEWKLETL CDLYETLAIT QSVIFVNTRR KVDWLTDKMR
301 SRDHTVSATH GDMDQNTRDI IMREFRSGSS RVLITTDLLA RGIDVQQVSL
351 VINYDLPTQP ENYLHRIGRG GRFGRKGVAI NFVTRDDERM LFDIQKFYNV
401 VIEELPSNVA DLL
```

Spot No.: **62**

NCBI accession No.: **gi|4235430**   Species: *Hevea brasiliensis*

PFF score: [142]   Protein name：latex-abundant protein

Matched peptides No.: [9]   Sequence coverage %: [16]

Matched sequences: R.SAEPGDLLFVHYSGHGTR.L;
R.EFVDQVPHGCR.I;
R.EFVDQVPHGCR.I;
K.QTIQDAFESR.G;
K.QTIQDAFESR.G;
R.VVEEDYGDSGYVK.S;
R.VVEEDYGDSGYVK.S;
K.LRPTLFDMFGDDASPK.V +Oxidation(M);
K.LRPTLFDMFGDDASPK.V + Oxidation (M)

Calculated Mr: **46443**   Calculated *p*I: **5.01**

Probability Based Mowse Score:

Ions score is -10*Log(P), where P is the probability that the observed match is a random event.
Individual ions scores > 45 indicate identity or extensive homology (p<0.05).
Protein scores are derived from ions scores as a non-probabilistic basis for ranking protein hits.

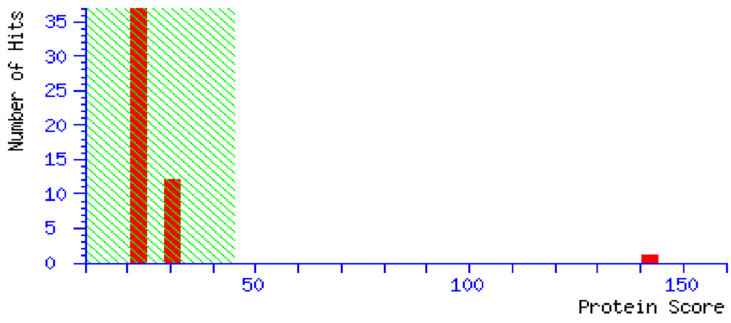

Matched peptide sequences: shown in Bold Red:

1	MAKKAVLIGI	NYPGTKAELK	GCINDVKRMY	RCLVDRYGFS	EEDITVLIDI
51	DESYIQPTGK	NIRRVLTDLV	R**SAEPGDLLF**	**VHYSGHGTR**L	PAETGEDDDT
101	GFDECIVPCD	MNLITDDDFR	**EFVDQVPHGC**	**R**ITVVSDSCH	SGGLIDEAKE
151	QIGESTKRKE	EESESGFGFK	SFLK**QTIQDA**	**FESR**GVHLPS	DLHHHHGHRD
201	EEDFDNR**VVE**	**EDYGDSGYVK**	SKSLPLSTLI	EILKQKTGKD	DIDVGK**LRPT**
251	**LFDMFGDDAS**	**PK**VKKFMKVI	LNKLRHGDGE	SGGGGFLGMV	GSLAQEFLKH
301	KLDENDESYV	KPALETEVDS	KQEVYAGKTK	RSLPDGGILI	SGCQTDQTSA
351	DASPSGKSSE	AYGALSNAIQ	TIIAETDGAV	TNQELVLKAR	KMLKKQGFTQ
401	KPGLYCSDDH	VEASFVC			

Spot No.: **63**

NCBI accession No.: **gi|255578278** Species: *Ricinus communis*

PFF score: [76]

Protein name:hypothetical protein RCOM_0537780

Matched peptides No.: [3] Sequence coverage %: [6]

Matched sequences: K.QESEYGSGGYGR.R;

R.KPSYGEEGGYGER.T ;

R.KPSYGEEGGYGER.T

Calculated Mr: **41389** Calculated *p*I: **4.65**

Probability Based Mowse Score:

Mascot Score Histogram

Ions score is -10*Log(P), where P is the probability that the observed match is a random event.
Individual ions scores > 44 indicate identity or extensive homology (p<0.05).
Protein scores are derived from ions scores as a non-probabilistic basis for ranking protein hits.

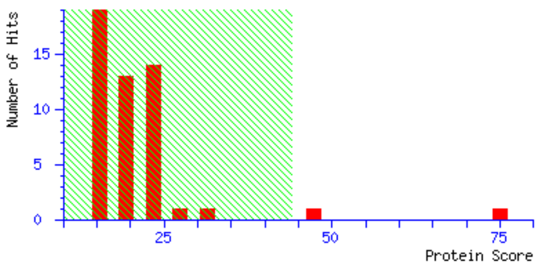

Matched peptide sequences: shown in Bold Red:

1	MPYYTRSEDD	VNDFDEFDPT	PYGGGYDLAL	TYGRPLPPSD	ETCYQNSAIA
51	DDDVDYDRPN	FTSYAEPSAY	NDDILQEEYN	SYSRPKPRPG	FIPGGGIGGD
101	PYSRPHAAPG	FQPGSGGYGG	VSEYEKPSGY	GRRPDSEYGS	GGYGGGTEYE
151	RPSGEDYGSG	HGRKQDSEYG	SGGYGRRPES	EYGSGGGYER	PSSEEYGSGY
201	GRK**QESEYGS**	**GGYGR**RPEAE	YGSGYGGRPE	TEYGGGGGSE	YGGGYGR**KPS**
251	**YGEEGGYGER**	TEYERPSYGD	DPPRRPGYGR	QDDEYERPSY	ERKSDDDNDG
301	SRRKYGGDDD	EKGYQKKYGG	YGDDDDEEGS	RRKYGYGGEE	ESYGRKKYGD
351	DGSDDDEKKH	HRHKHHQHKH	SDDE		

Spot No.: **64**

NCBI accession No.: **gi|5302811** Species: *Arabidopsis thaliana*

PFF score: [98] Protein name:putative serine protease-like protein

Matched peptides No.: [1] Sequence coverage %: [2]

Matched sequences: R.QEQAAFIEEISR.T

Calculated Mr: **47129** Calculated *p*I: **5.15**

Probability Based Mowse Score:

Ions score is -10*Log(P), where P is the probability that the observed match is a random event.
Individual ions scores > 45 indicate identity or extensive homology (p<0.05).
Protein scores are derived from ions scores as a non-probabilistic basis for ranking protein hits.

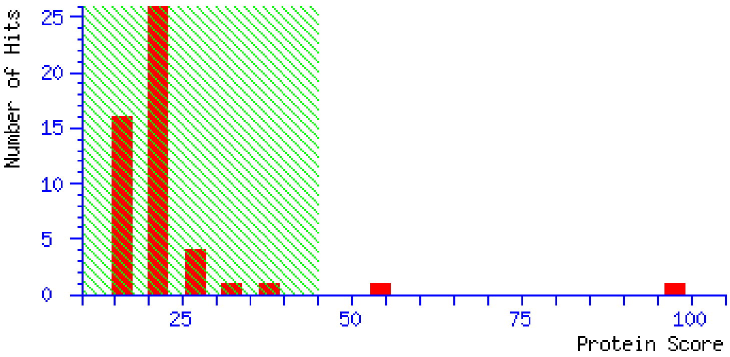

Matched peptide sequences: shown in Bold Red:

```
  1 MVVMMKATRG TRDPDLLGLM FVHHQFLFCV ISFFYLLLRD FEFLLNDSSS
 51 GIWTWSQVVS ETQALPYLQK AFNIQHFDFP TQEDQEENTY AAKVSEKQDF
101 SKEQWQDYKR PASEQHYSDE VETYGNLEPS QLELSGLSEA CNKLWELDLN
151 RLVPGRIIKS TAEMGKGSMR ELIWLKDYCS LGFCSLLDNY NPNEGYKEVV
201 TEEERQEQAA FIEEISRTSV IKYLHRYLVL KDVAPGSYLE FKRMLTSLWF
251 DLYGRGGTSG SSSAFEHVFV GEIKQSGGNK SLDSITGFST SKPVTDRETT
301 LLLRMQPDSE TQLLTIQFEW NGVLKSVSST LVGVSPEFEL ALYTMCFFMG
351 TEDNHIQLGP YNVNVKCYRL GNNRIALSFP HCRILKPLLM ALVTKYLPLA
401 QYCIELL
```

Spot No.: **65**

NCBI accession No.: **gi|2506277**   Species: *Pisum sativum*

Protein name： RuBisCO large subunit-binding protein subunit beta

PMF score: **102**   Sequence coverage %: **34**

Matched peptides No.: **17**   Total peptides No.: **54**

Calculated Mr: **63287**   Calculated *p*I: **5.85**

Probability Based Mowse Score:

Protein score is -10*Log(P), where P is the probability that the observed match is a random event.
Protein scores greater than 73 are significant ($p<0.05$).

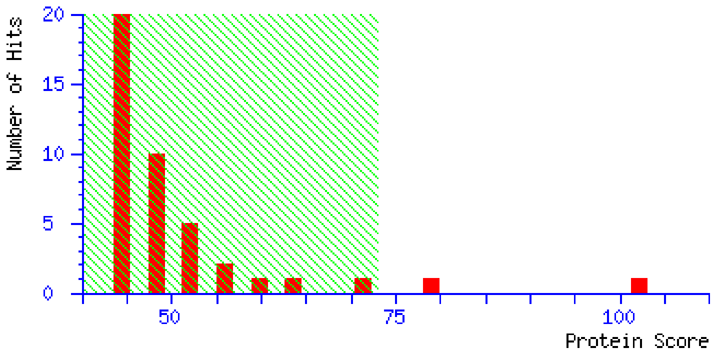

Matched peptide sequences: shown in Bold Red:

```
  1 MASTFSATTS SCNLSSSAAI SSFPLAAGKR NANKVVLPRK NRNVKVSAMA
 51 KELHFNKDGS AIKKLQNGVN KLADLVGVTL GPKGRNVVLE SKYGSPKIVN
101 DGVTVAKEVE LEDPVENIGA KLVRQAAAKT NDLAGDGTTT SVVLAQGLIA
151 EGVKVVAAGA NPVLITRGIE KTSKALVAEL KKMSKEVEDS ELADVAAVSA
201 GNNHEVGNMI AEALSKVGRK GVVTLEEGKS AENSLYVVEG MQFDRGYISP
251 YFVTDSEKMT VEFENCKLLL VDKKITNARD LINILEDAIR SGFPIVIIAE
301 DIEQEALATL VVNKLRGSLK IAALKAPGFG ERKSQYLDDI AILTGGTVIR
351 EEVGLTLDKA DKEVLGNAAK VVLTKDTTTI VGDGSTQEAV NKRVSQIKNQ
401 IEAAEQEYEK EKLSERIAKL SGGVAVIQVG AQTETELKEK KLRVEDALNA
451 TKAAVEEGIV VGGGCTLLRL ASKVDAIKDT LANDEEKVGA DIVKRALSYP
501 LKLIAKNAGV NGSVVSEKVL SSDNPKYGYN AATGKYEDLM AAGIIDPTKV
551 VRCCLEHASS VAKTFLMSDC VVVEIKEPES APVGNPMDNS GYGNI
```

Spot No.: **66**

NCBI accession No.: **gi|255543357** Species: *Ricinus communis*

Protein name： heat shock protein, putative

PMF score: **88** Sequence coverage %: **29**

Matched peptides No.: **18** Total peptides No.: **79**

Calculated Mr: **71965** Calculated *p*I: **5.27**

Probability Based Mowse Score:

Protein score is -10*Log(P), where P is the probability that the observed match is a random event.
Protein scores greater than 73 are significant (p<0.05).

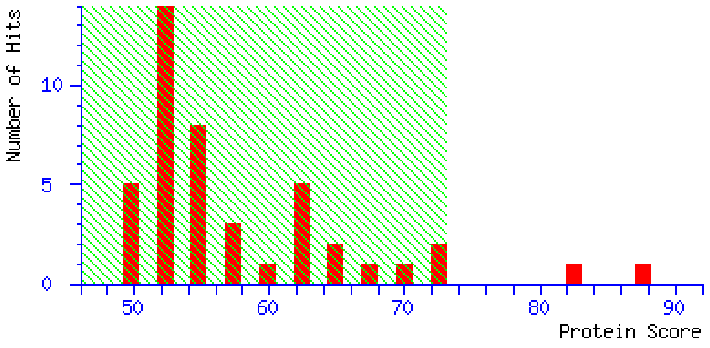

Matched peptide sequences: shown in Bold Red:

```
  1 MAKTEGKAVG IDLGTTYSCV GVWQNDRVEI IANDQGNRTT PSYVAFTDTE
 51 RLIGDAAKNQ VAMNPQNTVF DAKRLIGRRF SDPSVQSDMK HWPFKVVPGP
101 GDKPMIVVLY KGEEKQFAPE EISSMVLIKM KEIAEAYLGQ TVKNAVITVP
151 AYFNDSQRQA TKDAGAISGL NVMRIINEPT AAAIAYGLDK KGSRSGEKNV
201 LIFDLGGGTF DVSLLTIEEG IFEVKATAGD THLGGEDFDN RLVNHFVAEF
251 RRKHKKDIST NARALRRLRT ACERAKRTLS STSQTTIEID SLYEGIDFYS
301 TITRARFEEL NMDLFRKCME PVEKCLRDSK IDKSQVDDIV LVGGSTRIPK
351 VQQLLQDFFN GKELCKSINP DEAVAYGAAV QAAILSGEGD QKVQELLLLD
401 VTPLSLGIET AGGVMTVLIP RNTTIPAKKE QIFSTYSDNQ PGVLIQVYEG
451 ERARTKDNNL LGTFELKGIP PAPRGVPQIN VCFDIDANGI LNVSAEDKTA
501 GVKNKITITN DKGRLSKEDI ERMVQEAEKY KAEDEQVKKK VEAKNALENY
551 AYNMRNTVKD EKFAGKLDSA DKQKIEKAID ETIEWLDGNQ LAEVDEFEDK
601 LKELEGLCNP IVSKMYQGGA GGDVPMGGGA KPGSGYGNAS SGGSGSGPKI
651 EEVD
```

Spot No.: **67**

NCBI accession No.: **gi|224100473** Species: *Populus trichocarpa*

PFF score: [91] Protein name: predicted protein

Matched peptides No.: [1] Sequence coverage %: [3]

Matched sequences: R.SPPIEEVIQAGVVPR.F

Calculated Mr: **46093** Calculated *p*I: **4.54**

Probability Based Mowse Score:

Ions score is -10*Log(P), where P is the probability that the observed match is a random event.
Individual ions scores > 42 indicate peptides with significant homology.
Individual ions scores > 44 indicate identity or extensive homology (p<0.05).
Protein scores are derived from ions scores as a non-probabilistic basis for ranking protein hits.

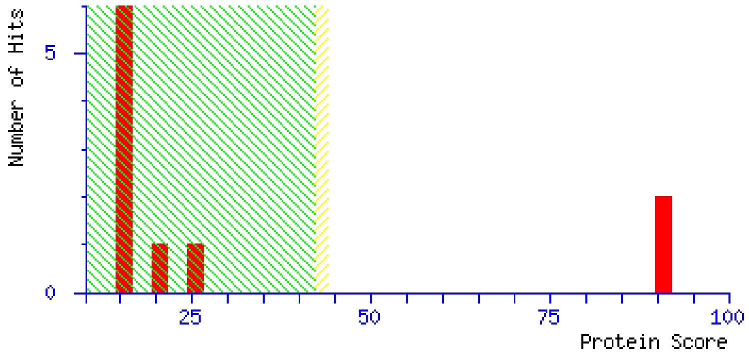

Matched peptide sequences: shown in Bold Red:

```
  1 GVWSEDGNLQ LESTTQFRKL LSIERSPPIE EVIQAGVVPR FVEFLVREDF
 51 PQLQFEAAWA LTNIASGTSE NTKVVIDHGA VPIFVKLLGS PSDDVREQAV
101 WALGNVAGDS PKCRDLVLSH GALIPLLAQL NEHAKLSMLR NATWTLSNFC
151 RGKPQPPFEQ VRPALPALER LVHSTDEEVL TDSCWALSYL SDGTDDKIQA
201 VIEAGVCPRL VELLLHPSPS VLVPALRTVG NIVTGDDMQT QVKALLAFCL
251 FVPSFAVIEN GLIGPLVNLL QNAEFDIKKE AAWAVSNATS GGTHEQIKFL
301 VSQGCIKPLC DLLVSPDPRI VTVSLEGLEN ILKVGEAEKN LGNSGDANFY
351 AQMIDDAEGL EKIENLQSHD NNEIYEKAVK ILETYWLEED DETLPSGDGA
401 QQGFQFGGNG VQVPSGGFNF
```

Spot No.: **68**

NCBI accession No.: **gi|31580730** Species: *Hevea brasiliensis*

Protein name: latex cyanogenic beta glucosidase

PMF score: **84** Sequence coverage %: **27**

Matched peptides No.: **12** Total peptides No.: **51**

Calculated Mr: **56325** Calculated *p*I: **5.72**

Probability Based Mowse Score:

Protein score is -10*Log(P), where P is the probability that the observed match is a random event.
Protein scores greater than 73 are significant ($p<0.05$).

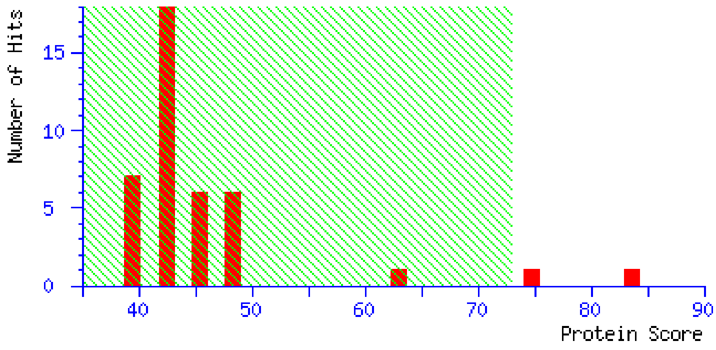

Matched peptide sequences: shown in Bold Red:

```
  1 MMKEKFLKEH PYLLEKEVSR SDFPPNFLFG VATSAYQIEG GCREGGRGPS
 51 IWDAFSHTKG TILDGSNGDV AVDHYHRYKE DIELIAKLGF DAYRFSLSWS
101 RIFPDGLGTK VNEEGIAFYN NIINALLEKG IEPYITLYHW DLPLHLQESM
151 GGWLNKEIVK YFAIYADTCF ASFGDRVKKW ITLNEPLQTA VNGFDTGILA
201 PGKHEHSYTE PFLASHHQIL AHATAVSIYR SMYKDNQGGE VGLVVDCEWA
251 ESNSDKIEDK AAAAKRLEFQ LGWYLHPLYY GDYPEVMRKI LGGGLPKFSE
301 EDKELLRNSL DFIGLNHYSS RFIKHVTDSP AECYYYKAQE IERLAKWEDG
351 EPIGERAASE WLYVRPWGLR KVLNYIVQRY NNPIIYVTEN GMDDEDSSAP
401 LHEMLDDKLR VRYFKGYLAA VAQAIKDGAD VRGYFAWSLL DNFEWAQGYT
451 KRFGLIYVDY KNGLARHPKS SAYWFMRFLK GDEGKNGKE
```

Spot No.: **69**

NCBI accession No.: **gi|2501850** Species: *Nicotiana tabacum*

Protein name: GDP dissociation inhibitor

PMF score: **83** Sequence coverage %: **34**

Matched peptides No.: **16** Total peptides No.: **62**

Calculated Mr: **50127** Calculated *p*I: **5.44**

Probability Based Mowse Score:

Protein score is -10*Log(P), where P is the probability that the observed match is a random event.
Protein scores greater than 73 are significant (p<0.05).

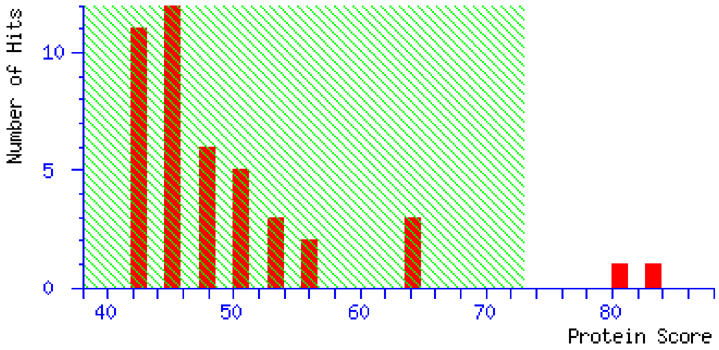

Matched peptide sequences: shown in Bold Red:

```
  1 MDEEYDVIVL GTGLKECILS GLLSVDGLKV LHMDRNDYYG GESTSLNLVQ
 51 LWKRFRGSDK PPAELGSSRD FNVDMIPKFI MANGALVRVL IHTDVTKYLY
101 FKAVDGSFVY NKGKVHKVPA TDMEALKSPL MGIFEKRRAR KFFIYVQDYN
151 ESDPKTHEGM DLTRVTTREL IAKYGLDDNT VDFIGHALAL HRDDRYLDEP
201 AQDTVKRMKL YAESLARFQG GSPYIYPLYG LGELPQAFAR LSAVYGGTYM
251 LNKPECKVEF DEEGKVCGVT SEGETAKCKK VVCDPSYLPN KVRKVGKVAR
301 AIAIMSHPIP NTNDSHSVQI ILPQKQLGRK SDMYLFCCSY THNVAPKGKF
351 IAFVSTEAET DNPESELKQG VNLLGPVDEI FYETYDRSEP VNEPSLDNCF
401 ISTSYDATTH FESTVDDVLN LYTKITGKVL DLNVDLSAAS AAEE
```

Spot No.: **70**

NCBI accession No.: **gi|2501850**   Species: *Nicotiana tabacum*

PFF score: [202]   Protein name: GDP dissociation inhibitor

Matched peptides No.: [5]   Sequence coverage %: [8]

Matched sequences: K.AVDGSFVYNK.G;
K.THEGMDLTR.V;
K.LYAESLAR.F;
K.KVVCDPSYLPNK.V;
K.VVCDPSYLPNK.V

Calculated Mr: **50127**   Calculated *p*I: **5.44**

Probability Based Mowse Score:

Ions score is -10*Log(P), where P is the probability that the observed match is a random event.
Individual ions scores > 45 indicate identity or extensive homology ($p<0.05$).
Protein scores are derived from ions scores as a non-probabilistic basis for ranking protein hits.

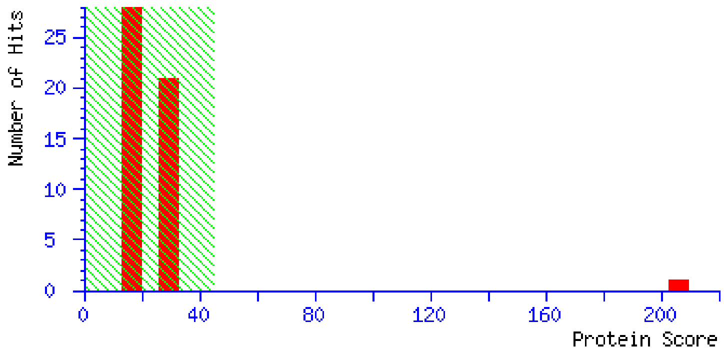

Matched peptide sequences: shown in Bold Red:

1	MDEEYDVIVL	GTGLKECILS	GLLSVDGLKV	LHMDRNDYYG	GESTSLNLVQ
51	LWKRFRGSDK	PPAELGSSRD	FNVDMIPKFI	MANGALVRVL	IHTDVTKYLY
101	FK**AVDGSFVY**	**NK**GKVHKVPA	TDMEALKSPL	MGIFEKRRAR	KFFIYVQDYN
151	ESDPK**THEGM**	**DLTR**VTTREL	IAKYGLDDNT	VDFIGHALAL	HRDDRYLDEP
201	AQDTVKRMK**L**	**YAESLAR**FQG	GSPYIYPLYG	LGELPQAFAR	LSAVYGGTYM
251	LNKPECKVEF	DEEGKVCGVT	SEGETAKCK**K**	**VVCDPSYLPN**	**K**VRKVGKVAR
301	AIAIMSHPIP	NTNDSHSVQI	ILPQKQLGRK	SDMYLFCCSY	THNVAPKGKF
351	IAFVSTEAET	DNPESELKQG	VNLLGPVDEI	FYETYDRSEP	VNEPSLDNCF
401	ISTSYDATTH	FESTVDDVLN	LYTKITGKVL	DLNVDLSAAS	AAEE

Spot No.: **71**

NCBI accession No.: **gi|359478860** Species: *Vitis vinifera*

Protein name： PREDICTED: 26S protease regulatory subunit 6B homolog

PMF score: **147** Sequence coverage %: **47**

Matched peptides No.: **18** Total peptides No.: **44**

Calculated Mr: **47196** Calculated *p*I: **5.42**

Probability Based Mowse Score:

Protein score is -10*Log(P), where P is the probability that the observed match is a random event. Protein scores greater than 73 are significant (p<0.05).

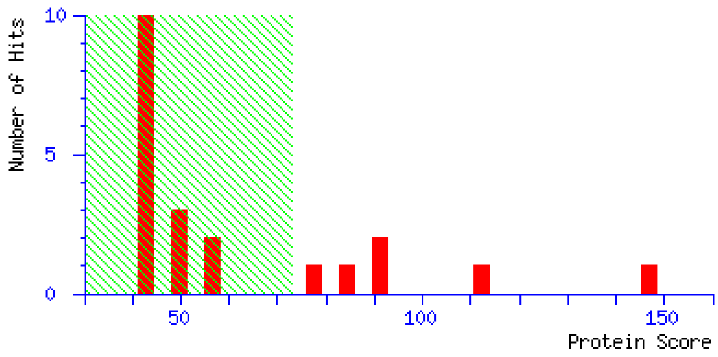

Matched peptide sequences: shown in Bold Red:

```
  1 MAVVLEPKPS SEPPPSFPST RSDIQGFSDQ SSSFSDEDDL YGRLKSLQRQ
 51 LEFIDIQEEY VKDEQKNLKR ELLRAQEEVK RIQSVPLVIG QFMEMVDQNN
101 GIVGSTTGSN YYVRILSTIN RELLKPSASV ALHRHSNALV DVLPPEADSS
151 ISLLSQSEKP DVTYNDIGGC DIQKQEIREA VELPLTHHEL YKQIGIDPPR
201 GVLLYGPPGT GKTMLAKAVA NHTTAAFIRV VGSEFVQKYL GEGPRMVRDV
251 FRLAKENAPA IIFIDEVDAI ATARFDAQTG ADREVQRILM ELLNQMDGFD
301 QTVNVKVIMA TNRADTLDPA LLRPGRLDRK IEFPLPDRRQ KRLVFQVCTA
351 KMNLSDEVDL EDYVSRPDKI SAAEIAAICQ EAGMHAVRKN RYVILPKDFE
401 KGYRTNVKKP DTDFEFYK
```

Spot No.: **72**

NCBI accession No.: **gi|224071449** Species: *Populus trichocarpa*

PFF score: [128] Protein name:predicted protein

Matched peptides No.: [3] Sequence coverage %: [4]

Matched sequences:R.EIYIAFR.G ;

K.NKIDVIPHYPGR.L ;

K.IDVIPHYPGR.L ;

Calculated Mr: **47121** Calculated *p*I: **5.28**

Probability Based Mowse Score:

Ions score is -10*Log(P), where P is the probability that the observed match is a random event.
Individual ions scores > 45 indicate identity or extensive homology (p<0.05).
Protein scores are derived from ions scores as a non-probabilistic basis for ranking protein hits.

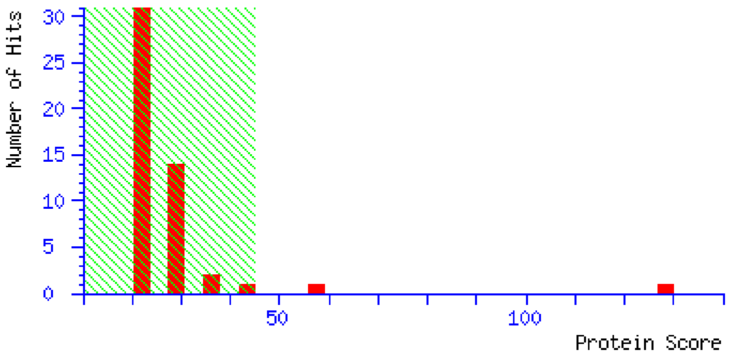

Matched peptide sequences: shown in **Bold Red:**

```
  1 MDGTSEATWP EILGSRNWDN LLDPLDLSLR KLILRCGDFC QATYDAFNND
 51 QNSRYCGTSR YGKRNFFHKV MLDNPENYQV SSFLYATARV SLPEAFLLHS
101 LSRDSWDRET NWIGYIAVTS DEQTKTLGRR EIYIAFRGTT RNYEWVDILG
151 AKLKSAKPLL RGATSTTHDQ ESSSSSDDDD DKVPKVMLGW LTMYISDDPN
201 SPFTKLSARA QLLAHIKELR ERYKDDDLSI IFTGHSLGAS LSILSAFDLV
251 ENGITDIPVS AFVFGSPQVG NKEFNERFNK YPNLKVLHIK NKIDVIPHYP
301 GRLMGYVYTG IEFEIDTRKS PSLKDSKNPS DWHNLQAMLH IVAGWNGEEQ
351 EFELKVKRSL ALVNKSSEFL KDECLVPGIW WVEKNKGMVR NEDGEWVLAP
401 PDEEDLPVPE C
```

Spot No.: **73**

NCBI accession No.: **gi|32186912** Species: *Gossypium hirsutum*

Protein name： **actin**

PMF score: **178** Sequence coverage %: **57**

Matched peptides No.: **19** Total peptides No.: **35**

Calculated Mr: **41936** Calculated *p*I: **5.37**

Probability Based Mowse Score:

Protein score is -10*Log(P), where P is the probability that the observed match is a random event.
Protein scores greater than 73 are significant ($p<0.05$).

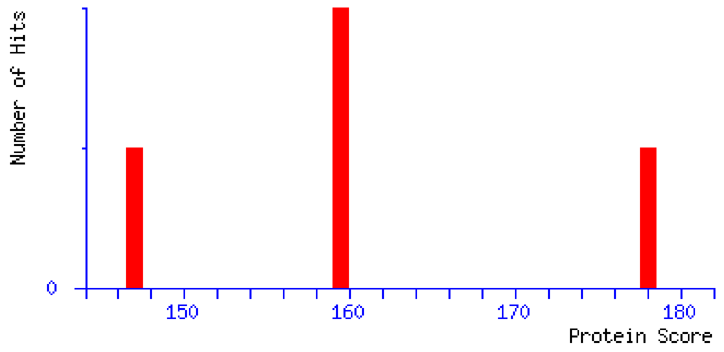

Matched peptide sequences: shown in Bold Red:

```
  1 MADGEDIQPL VCDNGTGMVK AGFAGDDAPR AVFPSIVGRP RHTGVMVGMG
 51 QKDAYVGDEA QSKRGILTLK YPIEHGIVSN WDDMEKIWHH TFYNELRVAP
101 EEHPVLLTEA PLNPKANREK MTHIIFETFN VPAMYVAIQA VLSLYASGRT
151 TGIVLDSGDG VSHTVPIYEG YALPHAILRL DLAGRDLTDA LMKILTERGY
201 MFTTTAEREI VRDMKEKLAY VALDYEQELE TAKSSSSVEK NYELPDGQVI
251 TIGAERFRCP EVLFQPSFIG MEAAGIHETT YNSIMKCDVD IRKDLYGNIV
301 LSGGSTMFPG IADRMSKEIT ALVPSSMKIK VVAPPERKYS VWIGGSILAS
351 LSTFQQMWIS KGEYDESGPS IVHRKCF
```

Spot No.: **74**

NCBI accession No.: **gi|297816956**

Species: Arabidopsis lyrata subsp. lyrata

PFF score: [70]

Protein name:hypothetical protein ARALYDRAFT_486071

Matched peptides No.: [3]   Sequence coverage %: [5]

Matched sequences:K.APLLIGCDVR.N ;
K.EVIAVNQDPLGVQGR.K;
K.EVIAVNQDPLGVQGR.K

Calculated Mr: **48033**   Calculated *p*I: **4.79**

Probability Based Mowse Score:

Ions score is -10*Log(P), where P is the probability that the observed match is a random event.
Individual ions scores > 45 indicate identity or extensive homology (p<0.05).
Protein scores are derived from ions scores as a non-probabilistic basis for ranking protein hits.

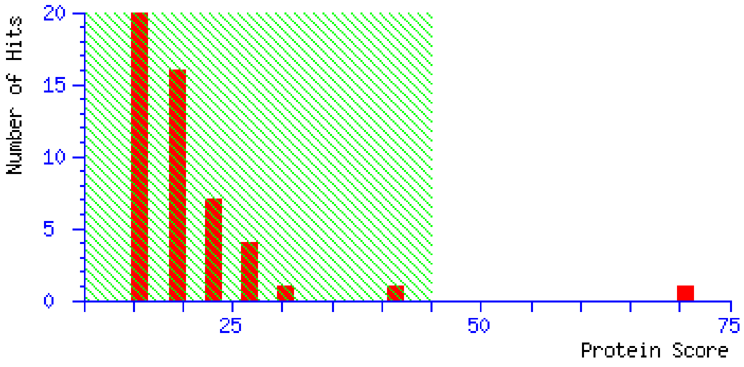

Matched peptide sequences: shown in Bold Red:

1	MKDSVLFLVG	LFSLSVLVSQ	SISGRVKAPL	LQSNTGGLVF	SKSFNSIYDT
51	SMYGRLQLNN	GLARTPQMGW	NSWNFFACNI	NETVIKETAD	ALLSSGLADL
101	GYIHVNIDDC	WSNLLRDSKG	QLVPHPETFP	SGIKLLADYV	HSKGLKLGIY
151	SDAGVFTCQV	RPGSLFHEVD	DADIFASWGV	DYLKYDNCFN	LGIKPIKRYP
201	PMRDALNATG	RSIFYSLCEW	GVDDPALWAK	EVGNSWRTTD	DINDTWASMT
251	TIADLNNKWA	AYAGPGGWND	PDMLEIGNGG	MTYEEYRGHF	SIWALMK**APL**
301	**LIGCDVR**NMT	AETFEILSNK	**EVIAVNQDPL**	**GVQGR**KIQAN	GEDDCQQVWS
351	GPLSGDRIVV	ALWNRCSEQA	TITASWDVIG	LESTISVSVR	DLWQHKDVTE
401	NASGSFEAQV	DAHDCHMYVL	TPQTVSHSDV		

Spot No.: **75**

NCBI accession No.: **gi|3264769** Species: *Prunus armeniaca*

PFF score: [74] Protein name: late embryogenesis-like protein

Matched peptides No.: [1] Sequence coverage %: [8]

Matched sequences: K.LVSGLIPDAGTIHAHGEETVK.V

Calculated Mr: **28937** Calculated *p*I: **4.72**

Probability Based Mowse Score:

Ions score is -10*Log(P), where P is the probability that the observed match is a random event.
Individual ions scores > 45 indicate identity or extensive homology (p<0.05).
Protein scores are derived from ions scores as a non-probabilistic basis for ranking protein hits.

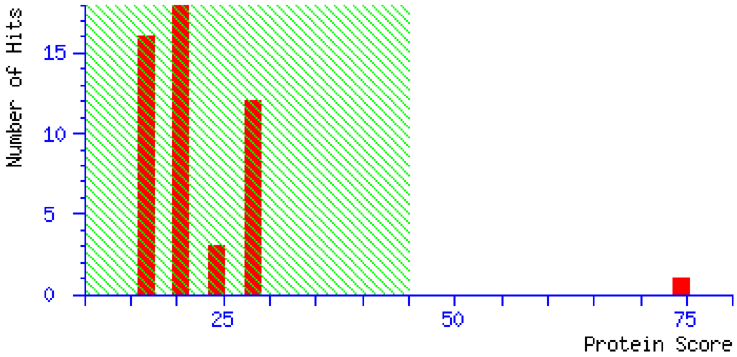

Matched peptide sequences: shown in Bold Red:

```
  1 DVTAIHIPSI NLEKAEIVVD VLIKNPNPVP IPLIDINYLI ESDGRKLVSG
 51 LIPDAGTIHA HGEETVKVPV HLVYDDIKNT YDDIKPGSII PYRFKVDLIV
101 DVPVLGRLTL PLEKTGEIPI PYKPDVDVEK IKFQAFSFEE TVAVLHVKLE
151 NMNDFDLGLN ALDYEIWLSE VNIGGAQLSQ SANLAKKGVT FIELPITFRP
201 KDFGSALWDM IRGKGTGYTI KGNINVDTPF GAMKLPIVKE GGTTRLRKNK
251 EDGGDDDDED ED
```

Spot No.: **76**

NCBI accession No.: **gi|168066110**

Species: **Physcomitrella patens subsp. patens**

Protein name： **predicted protein**

PMF score: **81** Sequence coverage %: **39**

Matched peptides No.: **15** Total peptides No.: **82**

Calculated Mr: **41963** Calculated *p*I: **5.24**

Probability Based Mowse Score:

Protein score is -10*Log(P), where P is the probability that the observed match is a random event.
Protein scores greater than 73 are significant (p<0.05).

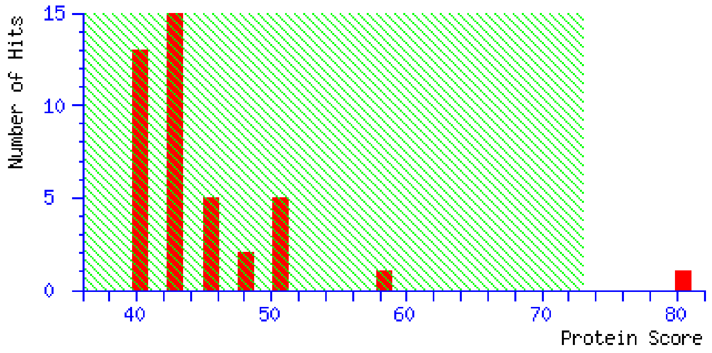

Matched peptide sequences: shown in Bold Red:

```
  1  MEASSHYIRK HWTRATTEVL VKAGDIEEPI VALVDHGFEI NLMSKDLYKK
 51  RKWPIDIEHR WVILAANNTR GELYRACSDV KIWIGDVATK RHFFVQDTTS
101  YPLILGQSYI TATRMETKVL DDGSTYARNY GGTKEEILSQ DEGTFDASFI
151  DIFESRNIIY KKVDDSKRKE IKRLYSIERE LRKLETLEDG EKILGVYEEN
201  GLEDSLELIA SIELGDNDKI VEIHSTEVYS ILESFQAPEV IVETKYKIVD
251  KKVKPLVRPL IEDSKEKMEE ASKEESLRDP KKIGHEFTKE TFEELKIGYD
301  GSLFPTEIEY FKKILAKRGK SFAFGSHEIG CVDPNIVALM LSRSFIDAAL
351  TSHVPLIDFL D
```

Spot No.: **77**

NCBI accession No.: **gi|388503664** Species: *Lotus japonicus*

Protein name： **unknown**

PMF score: **109** Sequence coverage %: **43**

Matched peptides No.: **13** Total peptides No.: **37**

Calculated Mr: **29537** Calculated *p*I: **4.79**

Probability Based Mowse Score:

Protein score is -10*Log(P), where P is the probability that the observed match is a random event.
Protein scores greater than 73 are significant (p<0.05).

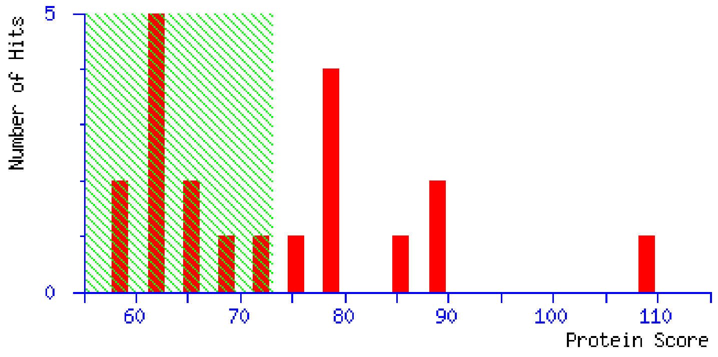

Matched peptide sequences: shown in Bold Red:

```
  1 MASSKDRGTF VYVAKLAEQA ERYEEMVDSM KKVANLDVEL TVEERNLLSV
 51 GYKNVIGARR ASWRILSSIE QKEETKGNDV NAKRIKEYRQ KVESELADIC
101 NDVMRVIDEH LIPSATAGES TVFYYKMKGD YYRYLAEFKS GNEKKEAADQ
151 SMKAYESATT AAEAELPPTH PIRLGLALNF SVFYYEILNS PERACHLAKQ
201 AFDEAISELD TLNEESYKDS TLIMQLLRDN LTLWTSDIPE EGEDSLKAND
251 TAKVGGGEDA E
```

Spot No.: **78**

NCBI accession No.: **gi|115468882**

Species: *Oryza sativa Japonica Group*

PFF score: [71]

Protein name:Os06g0608500

Matched peptides No.: [1]

Sequence coverage %: [6]

Matched sequences: R.LYEQQIEQLGNFQLR.I

Calculated Mr: **24269**

Calculated *p*I: **4.84**

Probability Based Mowse Score:

Ions score is -10*Log(P), where P is the probability that the observed match is a random event.
Individual ions scores > 42 indicate peptides with significant homology.
Individual ions scores > 43 indicate identity or extensive homology (p<0.05).
Protein scores are derived from ions scores as a non-probabilistic basis for ranking protein hits.

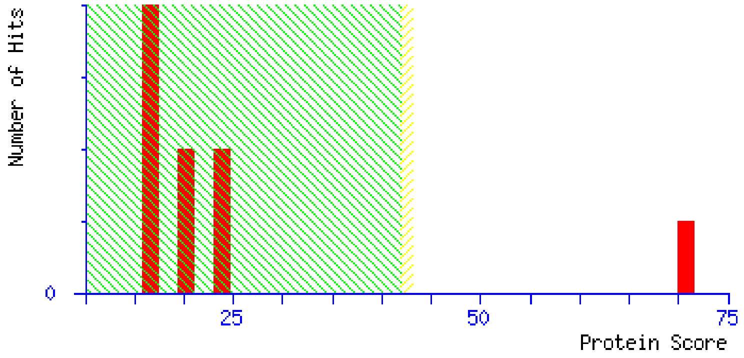

Matched peptide sequences: shown in Bold Red:

```
  1 MSGVFGKVFG KSKAQSQATA LASIDKLSET LEMLEKKENL LVKKANLEVE
 51 KAKTFTKAKN KRAAIQCLKR KRLYEQQIEQ LGNFQLRIHD QMIMLEGAKA
101 TTETVDALRT GASAMKAMHK ATNIDDVDKT MDEINDNMEN MRQIQDLLSA
151 PIGAAADFDE DELEAELADL EGEELEAELL APTTTAPTAP VRVQQPTRPS
201 AQSSKTEDDE LAALQAEMAM
```

(Matched peptide, shown in bold red: **LYEQQIEQLGNFQLR**)

Spot No.: **79**

NCBI accession No.: **gi|356532109** Species: *Glycine max*

PFF score: [104]

Protein name: 2,3-bisphosphoglycerate-independent phosphoglycerate mutase-like

Matched peptides No.: [3] Sequence coverage %: [7]

Matched sequences: K.ALEYENFDKFDR.V ;
K.ALEYENFDKFDR.V;
R.AHGTAVGLPTEDDMGNSEVGHNALGAGR.I + Oxidation (M)

Calculated Mr: **61059** Calculated *p*I: **5.51**

Probability Based Mowse Score:

Ions score is -10*Log(P), where P is the probability that the observed match is a random event.
Individual ions scores > 45 indicate identity or extensive homology ($p<0.05$).
Protein scores are derived from ions scores as a non-probabilistic basis for ranking protein hits.

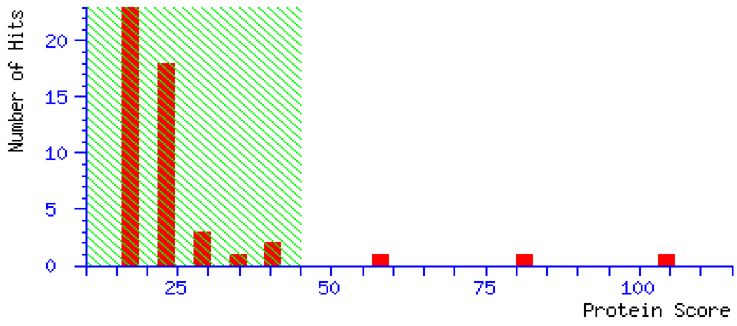

Matched peptide sequences: shown in Bold Red:

```
  1 MGSTGFSWKL ADHPKLPKGK TVAVVVLDGW GEATPNEYNC IHKAETPCMD
 51 SLKKGAPERW RLVRAHGTAV GLPTEDDMGN SEVGHNALGA GRIFAQGAKL
101 VDLALASGKF FEGGGFKYIK ESFENGTLHL IGLLSDGGVH SRLDQLQLLL
151 KGVSERGVKR VRVHILTDGR DVLDGSSVGF VETLENDLAN LREKGVDARI
201 ASGGGRMNVT MDRYENDWNV VKRGWDAQVL GEAPYKFTNA LEAVKKLRAE
251 PKANDQYLPP FVIVDESGKP VGPIVDGDAV VTFNFRADRM TMIAKALEYE
301 NFDKFDRVRF PKIRYAGMLE YDGELKLPSH YLVSPPEIDR TSGEYLVYNG
351 IRTFACSETV KFGHVTFFWN GNRSGYFNSE LEEYVEIPSD SGITFNEQPL
401 MKALQIAEKA RDAILSGKFD QVRVNLPNGD MVGHTGDIEA TVVACKAADA
451 AVKMILDAIE QVGGIYVVTA DHGNAEDMVK RDKSGKPLLG KDGKIQILTS
501 HTLEPVPIAI GGPGLAPGVR FRNDVPTGGL ANVAATVMNL HGFEAPSDYE
551 TTLVEVVDN
```

Spot No.: **80**

NCBI accession No.: **gi|224056489** Species: *Populus trichocarpa*

Protein name： **predicted protein**

Mascot score: **90** Sequence coverage %: **32**

Matched peptides No.: **17** Total peptides No.: **86**

Calculated Mr: **59210** Calculated *p*I: **5.11**

Probability Based Mowse Score:

Protein score is -10*Log(P), where P is the probability that the observed match is a random event. Protein scores greater than 73 are significant (p<0.05).

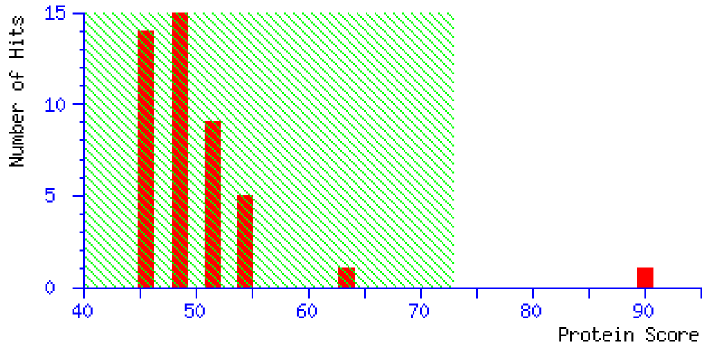

Matched peptide sequences: shown in Bold Red:

```
  1 MEESESRKQT LLSSLESAGI WIPDNVTSIG DLSPETFVSI CAQCVNLIDQ
 51 TASLPTTLPD AAIDKLKMCA DMGLAIKRLG FIGDMSYIKV IKPVTNCFVI
101 KLLYPSEDDL HKLTRFLVER LTESTRPVKI AEVKDVNARG KTKEDGSDIG
151 GRLDLNHQRE KLADPSLHNA VPEAEDIPFI GLSRQNLGKD KLPAFFYGEL
201 FDEEGKSGRD LFGNEETAAA GDDKDVQKAI SIKEQPSMVI IEKEELRNEE
251 KVLTEEVTAK TSELQHLEEE LELLKAAAEM AYGDQNSIDF CFEQLKGQVD
301 AKRCNITELK SQWDAFRMPL EDKKRNLEES LYANIPGSQE KLQKLRELEH
351 EKQLVLSEIT KREEEHSKLS ADLEKQPKLP SRTSYIERIK EITKSSQKQD
401 ADIKRILKET RELQLESNSI QDRLHRTYAV LDEIVCREAK RDLVGRKAYR
451 LLTSLHDCFG QISEKILTTD RISREMTELE KKLAAMASRS LNVDKLQADL
501 DAIVKENECL ELRLAENP
```

Spot No.: **81**

NCBI accession No.: **gi|255552291** Species: *Ricinus communis*

PFF score: [174]

Protein name:short chain dehydrogenase, putative

Matched peptides No.: [2] Sequence coverage %: [10]

Matched sequences: K.VALVTGGDSGIGR.A ;
K.DPIAIPTDVGFEENCR.K

Calculated Mr: **32148** Calculated *p*I: **6.50**

Probability Based Mowse Score:

Ions score is -10*Log(P), where P is the probability that the observed match is a random event.
Individual ions scores > 45 indicate identity or extensive homology (p<0.05).
Protein scores are derived from ions scores as a non-probabilistic basis for ranking protein hits.

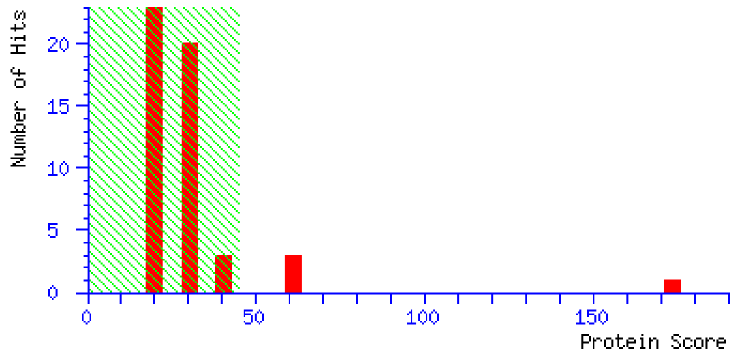

Matched peptide sequences: shown in **Bold Red:**

```
  1 MATRRGYRFP PQSQDKQPGK EYLMHPLPEF INPHYKPSNK LQDKVALVTG
 51 GDSGIGRAVS YYFTLEGATV AFTYVKGRED KDKDHILKIL HEVKAEGAKD
101 PIAIPTDVGF EENCRKVIDQ IMSEYGKIDI LVNNAGEAHY STTIEDITDP
151 MLERVFRTNI FGHFFMSRHA LKHMKEGGCI INTASVAAYA GFSFMVDYSS
201 TKGAVVAFTR SLALQLIDRG IRVNAVAPGS VWTPFQPSTL SAEQVAQVGS
251 GVPMDRAAQP YEIAPSFVFL ASNDCSSYFT GQVLHPNGMF
```

Spot No.: **82**

NCBI accession No.: **gi|2073448** Species: *Lotus japonicus*

Protein name: glutamine synthetase

PMF score: **91** Sequence coverage %: **46**

Matched peptides No.: **13** Total peptides No.: **89**

Calculated Mr: **39308** Calculated *p*I: **5.49**

Probability Based Mowse Score:

Protein score is -10*Log(P), where P is the probability that the observed match is a random event. Protein scores greater than 73 are significant (p<0.05).

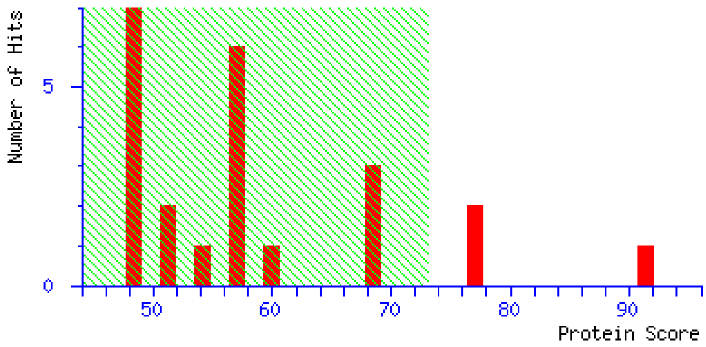

Matched peptide sequences: shown in Bold Red:

```
  1 MSLLSDLINL NLSETTDKII AEYIWIGGSG LDMRTKARTL PGPVSDPSQL
 51 PKWNYDGSST GQAPGEDSEV ILYPQAIFRD PFRRGSNILV ICDAYTPAGE
101 PIPTNKRHAA AKVFSHPDVV AEVPWYGIEQ EYTLLQKEVN WPVGWPIGGF
151 PGPQGPYYCG IGADKAFGRD IVDAHYKACL YAGVNISGIN GEVMPGQWEF
201 QVGPSVGISA GDEVWVARYI LERITEIAGV VLSFDPKPIK GDWNGAGAHT
251 NYSTKTMRED GGYEVIKKAI DKLGLRHKEH IAAYGEGNER RLTGRHETAD
301 INTFLWGVAN RGASIRVGRD TEKEGKGYFE DRRPASNMDP YVVTSMIADT
351 TILWKP
```

Spot No.: **83**

NCBI accession No.: **gi|192912956** Species: *Elaeis guineensis*

PFF score: [204]

Protein name: aluminum-induced protein

Matched peptides No.: [2] Sequence coverage %: [9]

Matched sequences: K.TANEVIIVIEAYR.S ;

R.GPYPADKVVR.D

Calculated Mr: 27466 Calculated *p*I: **6.07**

Probability Based Mowse Score:

Ions score is -10*Log(P), where P is the probability that the observed match is a random event.
Individual ions scores > 46 indicate identity or extensive homology (p<0.05).
Protein scores are derived from ions scores as a non-probabilistic basis for ranking protein hits.

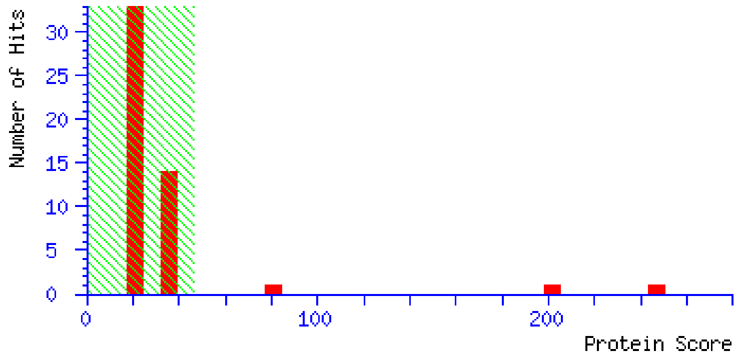

Matched peptide sequences: shown in Bold Red:

```
  1 MLAVFDRTVA KCPEGLRNMD SGPGEKGVGG VASLVDHFSA TREGAVTIRL
 51 GSSGAVAYTA EKQNLLLPRL FAAVDDIYCL FQGHIENIAH LKQQYGLNKT
101 ANEVIIVIEA YRSLRDRGPY PADKVVRDLN GKFAFILFDC SAKSTFIAAD
151 ADGSVPFYWG TDSGSHLVLS DDVDVIKKGC GKSFAPFPKG CFFTTSGGLQ
201 SFEHPLNEVK LMPRVDSQGE VCGATYEIDA QTKKESSMPR VGSAANWSSH
251 Y
```

Spot No.: 84

NCBI accession No.: gi|255559601   Species: ***Ricinus communis***

PFF score: [254]

Protein name：**alpha-soluble nsf attachment protein, putative**

Matched peptides No.:   [6]   Sequence coverage %:   [12]

Matched sequences: H EAAQAYVDAA HCYK ;

AIEIYEEIAR;

YQ DLDPTFSGTR

Calculated Mr: **32914**   Calculated *p*I: **5.12**

Probability Based Mowse Score:

Ions score is -10*Log(P), where P is the probability that the observed match is a random event.
Individual ions scores > 45 indicate identity or extensive homology ($p<0.05$).
Protein scores are derived from ions scores as a non-probabilistic basis for ranking protein hits.

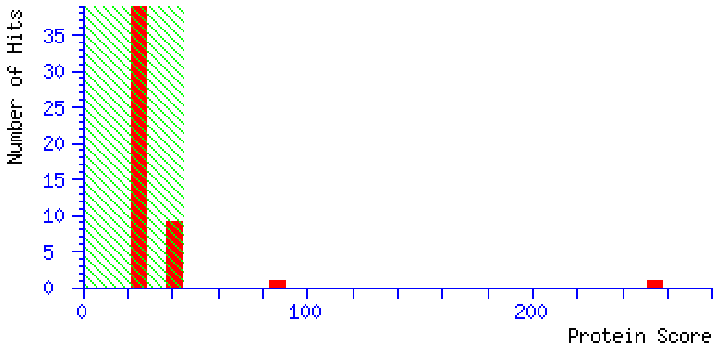

Matched peptide sequences: shown in **Bold Red:**

1	MSDQIAKGEE	FEKKAEKKLN	GWGLFGSKYE	DASDLFDKAA	NSFKLAKSWD
51	RAGSTYVKLA	NCHLKLDSK**H**	**EAAQAYVDAA**	**HCYK**KTTTNE	AISCLGQAVE
101	MFCDIGRISM	AARYYKEIAE	LYESEANVEK	AMDFYEKAAD	FFQGEEVTTS
151	ANQCKQKVAQ	FAAQLEQYQK	**AIEIYEEIAR**	HSLSNNLLKY	GVKGHLLNAG
201	ICHLCKGDVV	SVTNALER**YQ**	**DLDPTFSGTR**	EYRLLADVAA	AIDEEDVAKF
251	TDVVKEFDSM	TPLDSWKTTL	LLRVKEKLKA	KELEEDDLT	

Spot No.: 85

NCBI accession No.: **gi|224099853** Species: ***Populus trichocarpa***

PFF score: [69] Protein name: **putative protein**

Matched peptides No.: [2] Sequence coverage %: [5]

Matched sequences: S FEHPLNELKP VPR

Calculated Mr: **27448** Calculated *p*I: **5.84**

Probability Based Mowse Score:

Ions score is -10*Log(P), where P is the probability that the observed match is a random event.
Individual ions scores > 45 indicate identity or extensive homology (p<0.05).
Protein scores are derived from ions scores as a non-probabilistic basis for ranking protein hits.

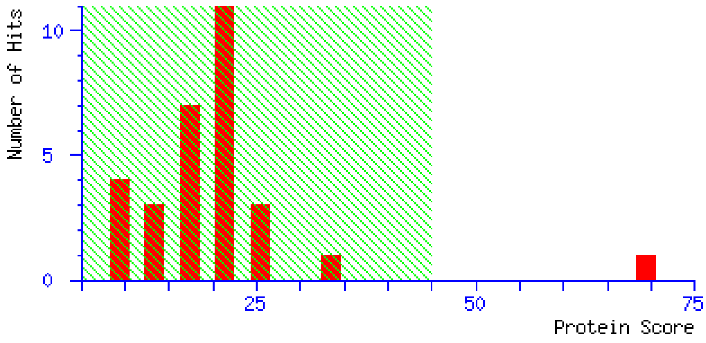

Matched peptide sequences: shown in Bold Red

```
  1 MLAVFDNTVA KCPDALQSPH SAPASSALKD GFLANHFASQ HPGSVTVNLG
 51 TSGLISHSVE KQNPFLPRLF AVVDDIFCLF QGHIDNVAVL KQQYGLNKTA
101 NEVIVVIEAY RTLRDRGPYP ADQVVKDIQG KFAFILYDST SKATFFAADA
151 DGSVPFFWGA DSEGNLVLSD DVQIVQKGCG KSFAPFPKGC FFTTSGGLRS
201 FEHPLNELKP VPRVDSSGQV CGATFKVDAE TKKESVGMPR VDSSYNWSSN
251 Y
```

Spot No.: 86

NCBI accession No.: **gi|38122474** Species: *Hevea brasiliensis*

PFF score: [410] Protein name： rubber elongation factor

Matched peptides No.: [4] Sequence coverage %: [26]

Matched sequences: YLDFVQAATVYAR ;
SGPFKPGVNTVESR;
SVVRPVYNK ;
RVDAYVTVLDR

Calculated Mr: **19570** Calculated *p*I: **5.06**

Probability Based Mowse Score:

Ions score is -10*Log(P), where P is the probability that the observed match is a random event.
Individual ions scores > 45 indicate identity or extensive homology ($p<0.05$).
Protein scores are derived from ions scores as a non-probabilistic basis for ranking protein hits.

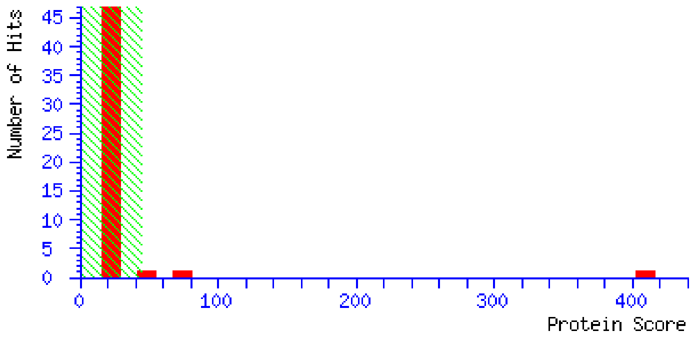

Matched peptide sequences: shown in Bold Red

```
  1  MAEGEEEVNI QEEANKGEEN PQEEANIQEE TNKGEENIQE EANIEEEANK
 51  EEESLKYLDF VQAATVYARA SFSKLYLFAK DKSGPFKPGV NTVESRFKSV
101  VRPVYNKFQP VPNKVLKFAD RRVDAYVTVL DRIVPPIVKR ASIQAYSVAP
151  GAALAVASYL PLHTKRLSKV LYGDG
```

Spot No.: 87

NCBI accession No.: **gi|38122474** Species: *Hevea brasiliensis*

PFF score: [226] Protein name： rubber elongation factor

Matched peptides No.: [4] Sequence coverage %: [26]

Matched sequences: YLDFVQAATVYAR ;
SGPFKPGVNTVESR;
SVVRPVYYNK ;
RVDAYVTVLDR

Calculated Mr: **19570** Calculated *p*I: **5.06**

Probability Based Mowse Score:

Ions score is -10*Log(P), where P is the probability that the observed match is a random event.
Individual ions scores > 46 indicate identity or extensive homology (p<0.05).
Protein scores are derived from ions scores as a non-probabilistic basis for ranking protein hits.

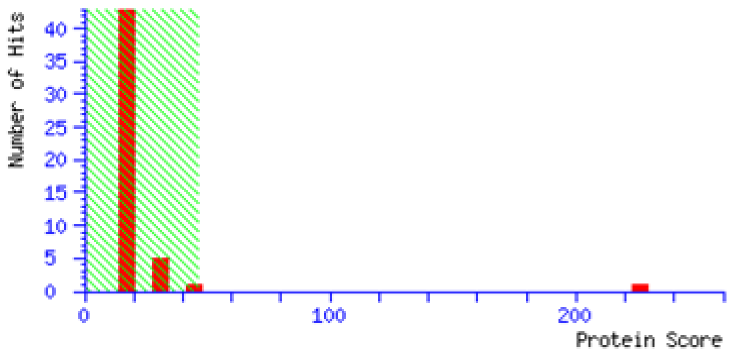

Matched peptide sequences: shown in Bold Red

```
  1  MAEGEEEVNI QEEANKGEEN PQEEANIQEE TNKGEENIQE EANIEEEANK
 51  EEESLKYLDF VQAATVYARA SFSKLYLFAK DKSGPFKPGV NTVESRFKSV
101  VRPVYNKFQP VPNKVLKFAD RRVDAYVTVL DRIVPPIVKR ASIQAYSVAP
151  GAALAVASYL PLHTKRLSKV LYGDG
```

Spot No.: 88

NCBI accession No.: **gi|38122474**   Species: *Hevea brasiliensis*

Protein name： rubber elongation factor

PMF score: **116**   Sequence coverage %: **54**

Matched peptides No.: **11**   Total peptides No.: **60**

Calculated Mr: **19570**   Calculated *p*I: **5.06**

Probability Based Mowse Score:

Protein score is -10*Log(P), where P is the probability that the observed match is a random event.
Protein scores greater than 73 are significant (p<0.05).

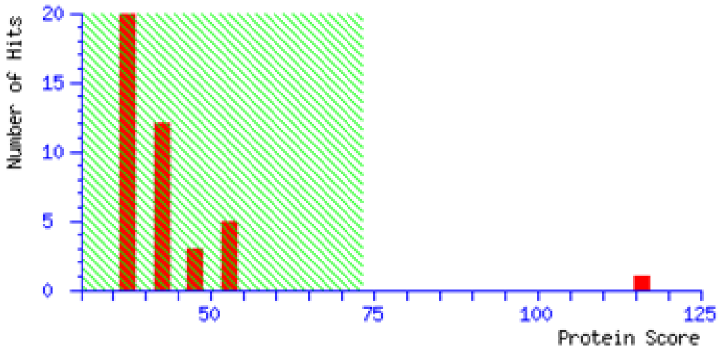

Matched peptide sequences: shown in Bold Red

```
  1 MAEGEEEVNI QEEANKGEEN PQEEANIQEE TNKGEENIQE EANIEEEANK
 51 EEESLKYLDF VQAATVYARA SFSKLYLFAK DKSGPFKPGV NTVESRFKSV
101 VRPVYNKFQP VPNKVLKFAD RRVDAYVTVL DRIVPPIVKR ASIQAYSVAP
151 GAALAVASYL PLHTKRLSKV LYGDG
```

Spot No.: 88

NCBI accession No.: **gi|38122474** Species: *Hevea brasiliensis*

Protein name： rubber elongation factor

PMF score: **99** Sequence coverage %: **455**

Matched peptides No.: **8** Total peptides No.: **39**

Calculated Mr: **19570** Calculated *p*I: **5.06**

Probability Based Mowse Score:

Protein score is -10*Log(P), where P is the probability that the observed match is a random event.
Protein scores greater than 73 are significant (p<0.05).

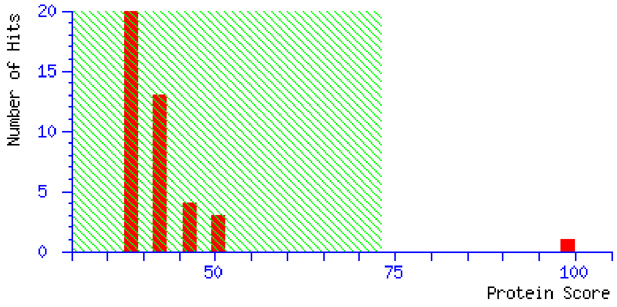

Matched peptide sequences: shown in Bold Red

1 MAEGEEEVNI QEEANKGEEN PQEEANIQEE TNK**GEENIQE** **EANIEEEANK**
51 **EEESLKYLDF** **VQAATVYAR**A SFSK**LYLFAK** DK**SGPFKPGV** **NTVESR**FKSV
101 **VRPVYNK**FQP VPNKVLK**FAD** **RRVDAYVTVL** **DR**IVPPIVKR ASIQAYSVAP
151 GAALAVASYL PLHTKRLSKV LYGDG

Spot No.: 90

NCBI accession No.: **gi|334854634** Species: *Hevea brasiliensis*

PFF score: [496] Protein name： elicitor-responsive protein

Matched peptides No.: [3] Sequence coverage %: [59]

Matched sequences: PLGTVEVLLVGAKGLENTDFLNGVDPYVVLACR;
GSEP EWNEKFSFEVSDGDTELTLK;
GEITVGLTFTPEVEMDNVGVDGYDFRL

Calculated Mr: **15249** Calculated *p*I: **4.06**

Probability Based Mowse Score:

Ions score is -10*Log(P), where P is the probability that the observed match is a random event.
Individual ions scores > 45 indicate identity or extensive homology ($p<0.05$).
Protein scores are derived from ions scores as a non-probabilistic basis for ranking protein hits.

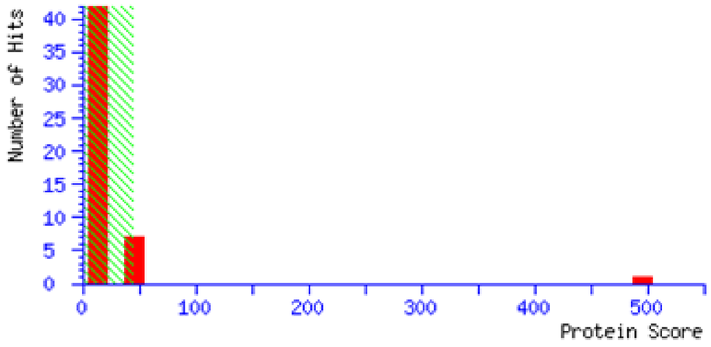

Matched peptide sequences: shown in Bold Red

```
  1 MPLGTVEVLL VGAKGLENTD FLNGVDPYVV LACRTQEQKS SVASGKGSEP
 51 EWNEKFSFEV SDGDTELTLK IMDSDVGAAD DFVGEATIPL EPLFLEGNLP
101 STAYKVVKEQ EYKGEITVGL TFTPEVEMDN VGVDGYDFRL
```

Spot No.: 91

NCBI accession No.: **gi|334854634** Species: *Hevea brasiliensis*

PFF score: [95]

Protein name:eukaryotic translation initiation factor 5A isoform VII

Matched peptides No.: [3]   Sequence coverage %: [28]

Matched sequences: TYPQQAGTIR;

K LEDIVPSSHNCDVPHVTR ;
TKDDLRLPTDENLLSQIK

Calculated Mr: **17689**   Calculated *p*I: **5.60**

Probability Based Mowse Score:

Ions score is -10*Log(P), where P is the probability that the observed match is a random event.
Individual ions scores > 45 indicate identity or extensive homology (p<0.05).
Protein scores are derived from ions scores as a non-probabilistic basis for ranking protein hits.

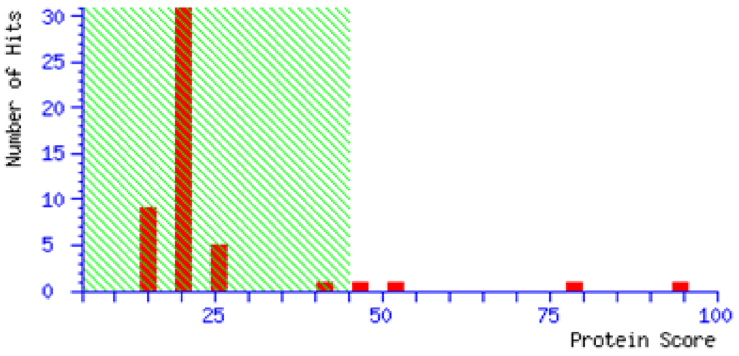

Matched peptide sequences: shown in Bold Red

1 MSDEEHHFES KADAGASK**TY** **PQQAGTIR**KN GYIVIKNRPC KVVDVSTSKT
51 GKHGHAKCHF VGIDIFNAK**K** **LEDIVPSSHN** **CDVPHVTR**TD YQLIDISEDG
101 FVSLLTENGN T**KDDLRLPTD** **ENLLSQIK**DG FAEGKDLVVT VMSSMGEEQI
151 CALKDIGPK

Spot No.: 92

NCBI accession No.: **gi|224144943** Species: *Populus trichocarpa*

PFF score: [180] Protein name: predicted protein

Matched peptides No.: [2] Sequence coverage %: [28]

Matched sequences: VFEDNVLIRVLVVDGGGSLR;

HVPITIG GTRISDGEWLYADTDGILISR

Calculated Mr: **17971** Calculated *p*I: **5.39**

Probability Based Mowse Score:

Ions score is -10*Log(P), where P is the probability that the observed match is a random event.
Individual ions scores > 44 indicate identity or extensive homology ($p<0.05$).
Protein scores are derived from ions scores as a non-probabilistic basis for ranking protein hits.

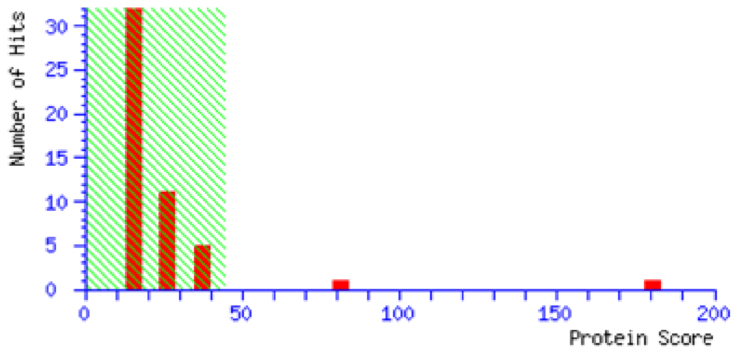

Matched peptide sequences: shown in **Bold Red**

```
  1 MALVTTAEVC DANPQLIVSG ELRALQPIFQ IYGRRQVFSG PVVTLKVFED
 51 NVLIREFLEE KGNGRVLVVD GGGSLRCAIL GGNPVVQAQN NGWAGIVVNG
101 CIRDVDEIMG CDIGVRALAS HPMKANKKGI GEKHVPITIG GTRISDGEWL
151 YADTDGILIS RTELAV
```

Spot No.: 93

NCBI accession No.: gi|253760141

Species: *Hevea brasiliensis*

Protein name： nucleoside diphosphate kinase 1

PMF score:84 Sequence coverage %: 44

Matched peptides No.: 8 Total peptides No.: 76

Calculated Mr: 16338 Calculated *p*I: 6.85

Probability Based Mowse Score:

Protein score is -10*Log(P), where P is the probability that the observed match is a random event.
Protein scores greater than 73 are significant (p<0.05).

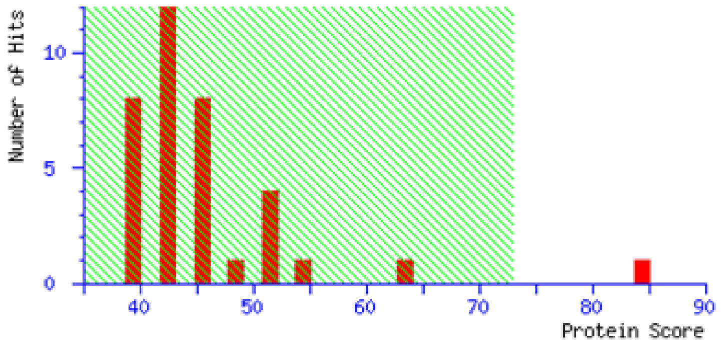

Matched peptide sequences: shown in Bold red:

```
  1 MEQTFIMIKP DGVQRGLVGE IIGRFEKKGF SLKGLKLITV ERLFAERHYA
 51 DLSAKPFFNG LVEYIISGPV VAMVWEGKNV VTTGRKIIGA TNPADSAPGT
101 IRGDYAIDIG RNVIHGSDSV ESARKEIALW FPEGPVSWSS SLHPWIYE
```

Spot No.: 94

NCBI accession No.: gi|123062 Species: *Hevea brasiliensis*

PFF score: [96]

Protein name: prohevein

Matched peptides No.: [1] Sequence coverage %: [8]

Matched sequences: IVDQ CSNGGLDLDV NVFR

Calculated Mr: 22700 Calculated *p*I: 5.63

Probability Based Mowse Score:

Ions score is -10*Log(P), where P is the probability that the observed match is a random event.
Individual ions scores > 44 indicate identity or extensive homology (p<0.05).
Protein scores are derived from ions scores as a non-probabilistic basis for ranking protein hits.

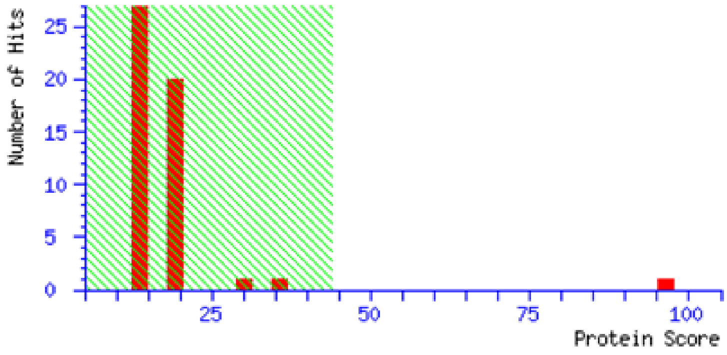

Matched peptide sequences: shown in Bold Red

```
  1 MNIFIVVLLC LTGVAIAEQC GRQAGGKLCP NNLCCSQWGW CGSTDEYCSP
 51 DHNCQSNCKD SGEGVGGGSA SNVLATYHLY NSQDHGWDLN AASAYCSTWD
101 ANKPYSWRSK YGWTAFCGPV GAHGQSSCGK CLSVTNTGTG AKTTVRIVDQ
151 CSNGGLDLDV NVFRQLDTDG KGYERGHITV NYQFVDCGDS FNPLFSVMKS
201 SVIN
```

Spot No.: 95

NCBI accession No.: **gi|313586569** Species: *Hevea brasiliensis*

PFF score: [70] Protein name: hypothetical protein 19

Matched peptides No.: [1] Sequence coverage %: [5]

Matched sequences: ADPNFFLTIR

Calculated Mr: **20724** Calculated *p*I: **7.77**

Probability Based Mowse Score:

Ions score is -10*Log(P), where P is the probability that the observed match is a random event.
Individual ions scores > 45 indicate identity or extensive homology (p<0.05).
Protein scores are derived from ions scores as a non-probabilistic basis for ranking protein hits.

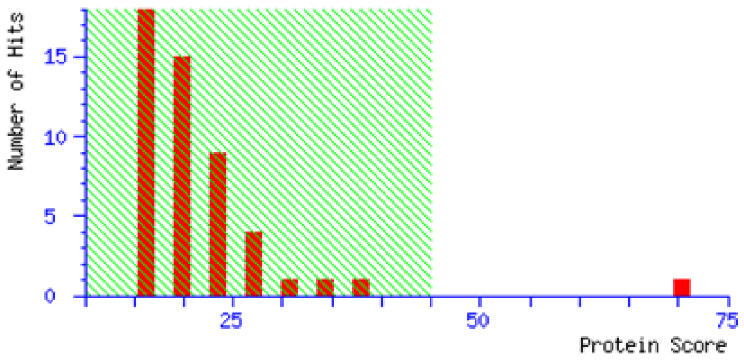

Matched peptide sequences: shown in Bold Red

1 MEELGSSHEK TESESSHCRK PHLPSGSYLS KKPSYKVYCK **ADPNFFLTIR**
51 DGKVVLAPPD PSDEFQHWYK DEKFSTMVKD EEGFPSFALV NKASGKALKH
101 SFGPTHPVLL TPYDPDDLEA SILWTESKDL GDGYRIVRML NNIRLNVEAL
151 RGDKKSGGVS NGTKVVLAYW NKGDHQKWRI APL

Spot No.: 96

NCBI accession No.: gi|313586569 Species: *Hevea brasiliensis*

Protein name: **hypothetical protein 19**

PMF score: **94** Sequence coverage %: **62**

Matched peptides No.: **11** Total peptides No.: **73**

Calculated Mr: **20724** Calculated *p*I: **7.77**

Probability Based Mowse Score:

Protein score is -10*Log(P), where P is the probability that the observed match is a random event. Protein scores greater than 73 are significant (p<0.05).

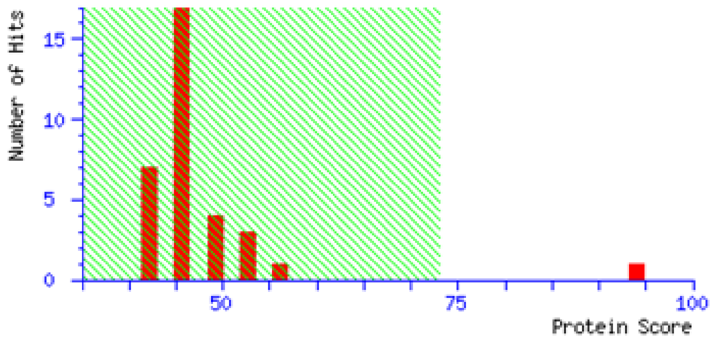

Matched peptide sequences: shown in Bold Red

```
  1  MEELGSSHEK TESESSHCRK PHLPSGSYLS KKPSYKVYCK ADPNFFLTIR
 51  DGKVVLAPPD PSDEFQHWYK DEKFSTMVKD EEGFPSFALV NKASGKALKH
101  SFGPTHPVLL TPYDPDDLEA SILWTESKDL GDGYRIVRML NNIRLNVEAL
151  RGDKKSGGVS NGTKVVLAYW NKGDHQKWRI APL
```

Spot No.: 97

NCBI accession No.: **gi|4835766** Species: ***Arabidopsis thaliana***

Protein name：Contains similarity to gb|AB017693 transfactor (WERBP-1) from Nicotiana tabacum

PMF score: **84** Sequence coverage %: **27**

Matched peptides No.: **11** Total peptides No.:**56**

Calculated Mr: **40677** Calculated *p*I: **8.94**

Probability Based Mowse Score:

Protein score is -10*Log(P), where P is the probability that the observed match is a random event. Protein scores greater than 73 are significant (p<0.05).

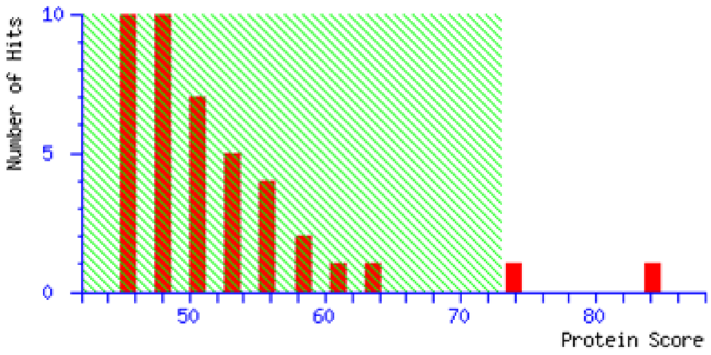

Matched peptide sequences: shown in Bold Red

```
  1 MFHAKKPSSM NGSYENRAMC VQGDSGLVLT TDPKPRLRWT VELHERFVDA
 51 VAQLGGPDKA TPKTIMRVMG VKGLTLYHLK SHLQKFRLGK QPHKEYGDHS
101 TKEGSRASAM DIQRNVASSS GMMSRNMNDN SHQVGLIRMQ MEVQRRLHEQ
151 LEVQRHLQLR IEAQGKYMQS ILERACQTLA GENMAAATAA AAVGGGYKGN
201 LGSSSLSAAV GPPPHPLSFP PFQDLNIYGN TTDQVLDHHN FHHQNIENHF
251 TGNNAADTNI YLGKKRPNPN FGNDVRKGLL MWSDQDHDLS ANQSIDDEHR
301 IQIQMATHVS TDLDSLSEIY ERKSGLSGDE GNNGGKLLER PSPRRSPLSP
351 MMNPNGGLIQ GRNSPFG
```

Spot No.: 98

NCBI accession No.: gi|38122474 Species: *Hevea brasiliensis*

Protein name: rubber elongation factor

PMF score: 99 Sequence coverage %: 45

Matched peptides No.: 8 Total peptides No.: 39

Calculated Mr: 19570 Calculated *p*I: 5.06

Probability Based Mowse Score:

Protein score is -10*Log(P), where P is the probability that the observed match is a random event.
Protein scores greater than 73 are significant (p<0.05).

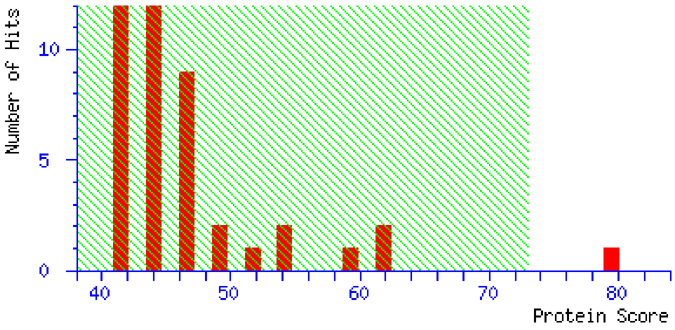

Matched peptide sequences: shown in Bold red:

1 MAEGEEEVNI QEEANKGEEN PQEEANIQEE TNKGEENIQE EANIEEEANK
51 EEESLKYLDF VQAATVYARA SFSKLYLFAK DKSGPFKPGV NTVESRFKSV
101 VRPVYNKFQP VPNKVLKFAD RRVDAYVTVL DRIVPPIVKR ASIQAYSVAP
151 GAALAVASYL PLHTKRLSKV LYGDG

Spot No.: **99**

NCBI accession No.: **gi|3377751**

Species: *Mesembryanthemum crystallinum*

PFF score: [73] Protein name: glutathione S-transferase

Matched peptides No.: [1] Sequence coverage %: [4]

Matched sequences: R.FWADFIDKK.L

Calculated Mr: **26154** Calculated *p*I: **5.71**

Probability Based Mowse Score:

Ions score is -10*Log(P), where P is the probability that the observed match is a random event.
Individual ions scores > 44 indicate identity or extensive homology (p<0.05).
Protein scores are derived from ions scores as a non-probabilistic basis for ranking protein hits.

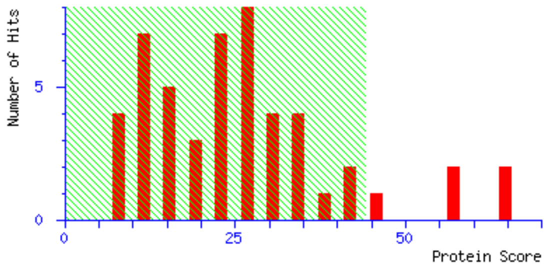

Matched peptide sequences: shown in Bold Red:

```
  1 MGEEVILLDF WPSMFGMRLR IALAEKGVKH ERKEEDLLIG KSPLLLQLNP
 51 VHKKIPVLVH NGKPINESMI AVQYIDEVWN EKSPSLLPSD PYQRAQARFW
101 ADFIDKKLYD ECRKIYTTKE AEIGEETYKG FLEIIKVLEA EVGEKSYFGG
151 DTFGFVDVAL IPFYSWFYAL EMLGKFNIET ECPKLIAWAK RCMERESVSK
201 SLPDRHKIYD FITLQLRKAL GVDQ
```

Spot No.: **100**

NCBI accession No.: **gi|1709761** Species: *Spinacia oleracea*

PFF score: [49]

Protein name: Proteasome subunit alpha type-4

Matched peptides No.: [1] Sequence coverage %: [3]

Matched sequences: R.TTIFSPEGR.L

Calculated Mr: **27545** Calculated *p*I: **6.17**

Probability Based Mowse Score:

Ions score is -10*Log(P), where P is the probability that the observed match is a random event.
Individual ions scores > 44 indicate identity or extensive homology (p<0.05).
Protein scores are derived from ions scores as a non-probabilistic basis for ranking protein hits.

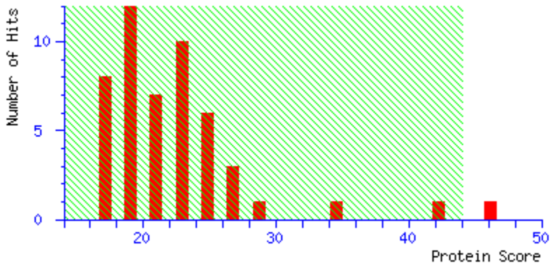

Matched peptide sequences: shown in Bold Red:

```
  1 MSRRYDSRTT IFSPEGRLYQ VEYAMEAIGN AGSAIGILAK DGVVLIGEKK
 51 VTSKLLQTST STEKMYKIDD HVACAVAGIM SDANILINTA RVQAQRYTFS
101 YQEPMPVEQL VQSLCDTKQG YTQFGGLRPF GVSFLFAGWD KNYGFQLYMS
151 DPSGNYGGWK ATAIGANNQA AQSMLKQDYK DDVTREDAVK LALKALSKTM
201 DSTSLTSEKL ELAEVYLLPS GKVKYQVHSP ESLNRLLTES GLTQPAAETS
```

Spot No.: **101**

NCBI accession No.: **gi|27449248** Species: *Hevea brasiliensis*

PFF score: [431] Protein name: ascorbate peroxidase

Matched peptides No.: [5] Sequence coverage %: [32]

Matched sequences: R.EDKPEPPPEGR.L;

R.IAWHSAGTYDANTK.T;
R.HAAEQAHAANNGLDIAVR.L;
K.YAADEDAFFADYAEAHVK.L;
K.TMGLSDKDIVVLSGGHTLGR.C + Oxidation (M)

Calculated Mr: **27526** Calculated *p*I: **5.78**

Probability Based Mowse Score:

Ions score is -10*Log(P), where P is the probability that the observed match is a random event.
Individual ions scores > 44 indicate identity or extensive homology (p<0.05).
Protein scores are derived from ions scores as a non-probabilistic basis for ranking protein hits.

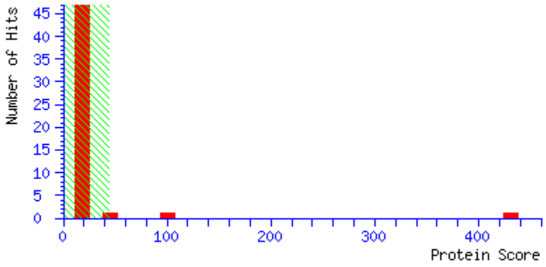

Matched peptide sequences: shown in Bold Red:

```
  1 MTKNYPKVSE EYQKAIDKAK RKLRGFIAEK GCAPLMLRIA WHSAGTYDAN
 51 TKTGGPFGTM RHAAEQAHAA NNGLDIAVRL LEPIKQQFPI LSYADFYQLA
101 GVVAVEITGG PEIPFHPGRE DKPEPPPEGR LPNATKGADH LREVFGKTMG
151 LSDKDIVVLS GGHTLGRCHK ERSGFDGPWT ANPLIFDNSF FTELLAGQKE
201 GLLQLPTDTV LVTDPVFRPY VEKYAADEDA FFADYAEAHV KLSELGFAEA
```

Spot No.: **102**

NCBI accession No.: **gi|211906460** Species: *Gossypium hirsutum*

PFF score: [193] Protein name: triosephosphate isomerase

Matched peptides No.: [3] Sequence coverage %: [18]

Matched sequences: K.FFVGGNWK.C;

K.VIACIGETLEQR.E;

K.ELAAQPDVDGFLVGGASLKPEFIDIIK.S

Calculated Mr: **27472** Calculated *p*I: **6.00**

Probability Based Mowse Score:

Ions score is -10*Log(P), where P is the probability that the observed match is a random event.
Individual ions scores > 45 indicate identity or extensive homology (p<0.05).
Protein scores are derived from ions scores as a non-probabilistic basis for ranking protein hits.

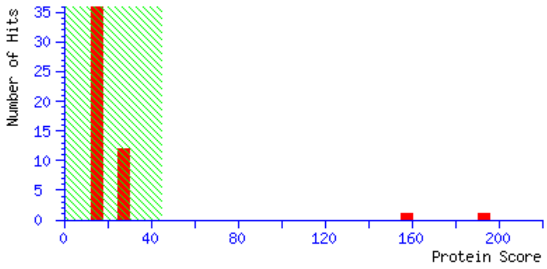

Matched peptide sequences: shown in Bold Red:

```
  1 MGRKFFVGGN WKCNGTTEEV NKIVSTLNAG EVPSQDVVEV VVSPPFVFLP
 51 LVKTSLRPDF HVAAQNCWVK KGGAFTGEVS AEMLVNLSIP WVIIGHSERR
101 LILKESNEFV ADKVAYALAQ GLKVIACIGE TLEQREAGST VAVVAEQTKA
151 IAAKISNWTD VVLAYEPVWA IGTGKVATPA QAQEVHCELR KWLQANVSPE
201 VAASTRIIYG GSVTAANCKE LAAQPDVDGF LVGGASLKPE FIDIIKSAEV
251 KKNA
```

Spot No.: 103

NCBI accession No.: gi|38122474 Species: ***Hevea Brasiliensis***

Protein name：rubber elongation factor

PMF score: **125** Sequence coverage %: **68**

Matched peptides No.: **11** Total peptides No.: **50**

Calculated Mr: **19570** Calculated *p*I: **5.06**

Probability Based Mowse Score:

Protein score is -10*Log(P), where P is the probability that the observed match is a random event.
Protein scores greater than 73 are significant (p<0.05).

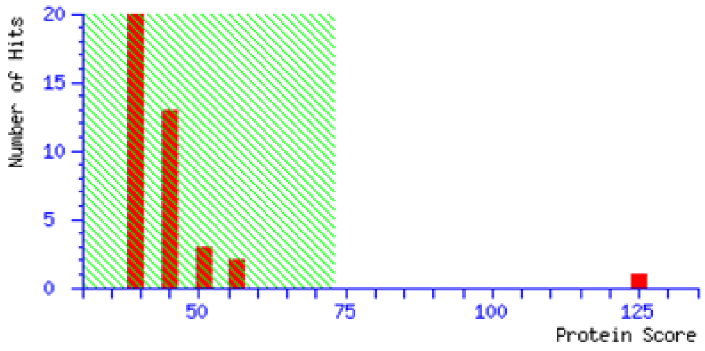

Matched peptide sequences: shown in Bold Red

```
  1 MAEGEEEVNI QEEANKGEEN PQEEANIQEE TNKGEENIQE EANIEEEANK
 51 EEESLKYLDF VQAATVYARA SFSKLYLFAK DKSGPFKPGV NTVESRFKSV
101 VRPVYNKFQP VPNKVLKFAD RRVDAYVTVL DRIVPPIVKR ASIQAYSVAP
151 GAALAVASYL PLHTKRLSKV LYGDG
```

Spot No.: 104

NCBI accession No.: gi|3451147 Species: *Hevea Brasiliensis*

Protein name： chitinase

PMF score: **135** Sequence coverage %: **66**

Matched peptides No.: **19** Total peptides No.: **77**

Calculated Mr: **34138** Calculated *p*I: **8.72**

Probability Based Mowse Score:

Protein score is -10*Log(P), where P is the probability that the observed match is a random event.
Protein scores greater than 73 are significant ($p<0.05$).

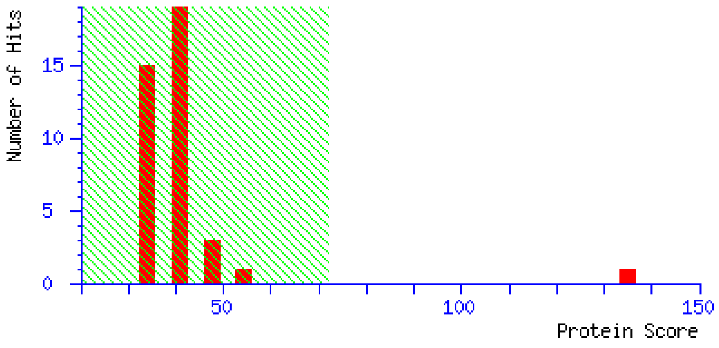

Matched peptide sequences: shown in Bold Red

```
  1 MAKRTQAILL LLLAISLIMS SSHVDGGGIA IYWGQNGNEG TLTQTCSTRK
 51 YSYVNIAFLN KFGNGQTPQI NLAGHCNPAA GGCTIVSNGI RSCQIQGIKV
101 MLSLGGGIGS YTLASQADAK NVADYLWNNF LGGKSSSRPL GDAVLDGIDF
151 DIEHGSTLYW DDLARYLSAY SKQGKKVYLT AAPQCPFPDR YLGTALNTGL
201 FDYVWVQFYN NPPCQYSSGN INNIINSWNR WTTSINAGKI FLGLPAAPEA
251 AGSGYVPPDV LISRILPEIK KSPKYGGVML WSKFYDDKNG YSSSIRDSVL
301 FLHSEKCMTV L
```

Spot No.:105

NCBI accession No.: gi|157831407 Species: *Hevea brasiliensis*

Protein name：Chain A, Crystal Structures Of Hevamine

PMF score: 93 Sequence coverage %:51

Matched peptides No.: 13 Total peptides No.: 76

Calculated Mr: 29874 Calculated *p*I: 8.44

Probability Based Mowse Score:

Protein score is -10*Log(P), where P is the probability that the observed match is a random event.
Protein scores greater than 73 are significant (p<0.05).

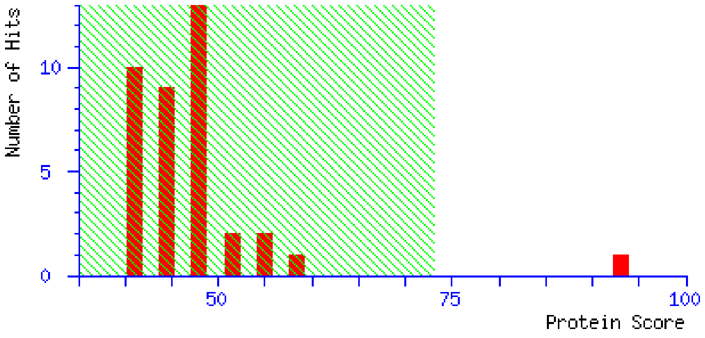

Matched peptide sequences: shown in Bold red:

1 GGIAIYWGQN GNEGTLTQTC STR**KYSYVNI** **AFLNK**FGNGQ TPQINLAGHC
51 NPAAGGCTIV SNGIR**SCQIQ** **GIKVMLSLGG** **GIGSYTLASQ** **ADAKNVADYL**
101 **WNNFLGGKSS** **SRPLGDAVLD** **GIDFDIEHGS** **TLYWDDLARY** LSAYSKQGK**K**
151 **VYLTAAPQCP** **FPDR**YLGTAL NTGLFDYVWV QFYNNPPCQY SSGNINNIIN
201 SWNRWTTSIN AGK**IFLGLPA** **APEAAGSGYV** **PPDVLISRIL** PEIKKSPK**YG**
251 **GVMLWSKFYD** **DK**NGYSSSIL DSV

Spot No.:106

NCBI accession No.: gi|157831407 Species: *Hevea brasiliensis*

Protein name：Chain A, Crystal Structures Of Hevamine

PMF score: 99 Sequence coverage %:42

Matched peptides No.: 12 Total peptides No.: 47

Calculated Mr: 29874 Calculated *p*I: 8.44

Probability Based Mowse Score:

Protein score is -10*Log(P), where P is the probability that the observed match is a random event. Protein scores greater than 73 are significant (p<0.05).

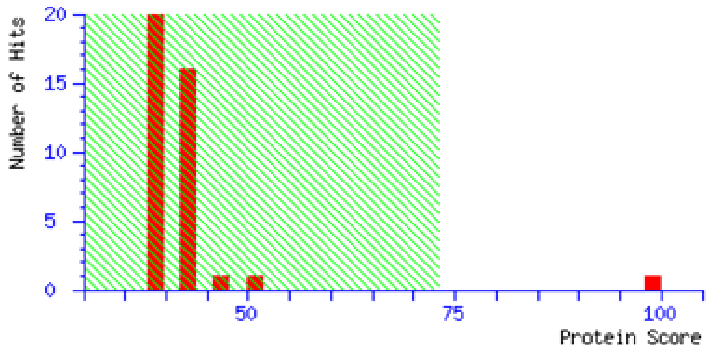

Matched peptide sequences: shown in **Bold Red**

```
  1 GGIAIYWGQN GNEGTLTQTC STRKYSYVNI AFLNKFGNGQ TPQINLAGHC
 51 NPAAGGCTIV SNGIRSCQIQ GIKVMLSLGG GIGSYTLASQ ADAKNVADYL
101 WNNFLGGKSS SRPLGDAVLD GIDFDIEHGS TLYWDDLARY LSAYSKQGKK
151 VYLTAAPQCP FPDRYLGTAL NTGLFDYVWV QFYNNPPCQY SSGNINNIIN
201 SWNRWTTSIN AGKIFLGLPA APEAAGSGYV PPDVLISRIL PEIKKSPKYG
251 GVMLWSKFYD DKNGYSSSIL DSV
```

Spot No.:107

NCBI accession No.: gi|3451147   Species: *Hevea Brasiliensis*

Protein name： chitinase

PMF score: **83**   Sequence coverage %: **48**

Matched peptides No.: **12**   Total peptides No.: **81**

Calculated Mr: **34138**   Calculated *p*I: **8.72**

Probability Based Mowse Score:

Protein score is -10*Log(P), where P is the probability that the observed match is a random event.
Protein scores greater than 73 are significant (p<0.05).

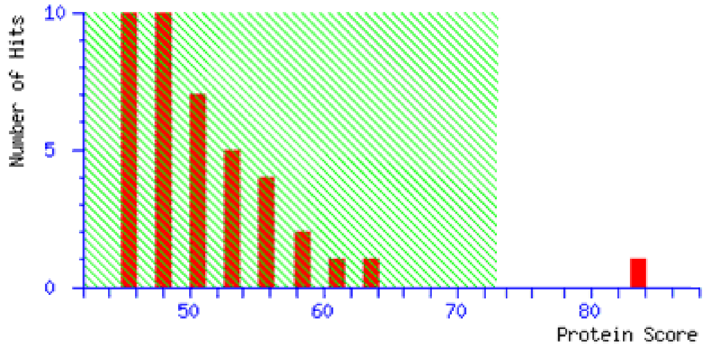

Matched peptide sequences: shown in Bold Red

1 MAKRTQAILL LLLAISLIMS SSHVDGGGIA IYWGQNGNEG TLTQTCSTRK
51 YSYVNIAFLN KFGNGQTPQI NLAGHCNPAA GGCTIVSNGI RSCQIQGIKV
101 MLSLGGGIGS YTLASQADAK NVADYLWNNF LGGKSSSRPL GDAVLDGIDF
151 DIEHGSTLYW DDLARYLSAY SKQGKKVYLT AAPQCPFPDR YLGTALNTGL
201 FDYVWVQFYN NPPCQYSSGN INNIINSWNR WTTSINAGKI FLGLPAAPEA
251 AGSGYVPPDV LISRILPEIK KSPKYGGVML WSKFYDDKNG YSSSIRDSVL
301 FLHSEKCMTV L

Spot No.: **108**

NCBI accession No.: **gi|2493895** Species: *Citrullus lanatus*

PFF score: [89]

Protein name: cysteine synthase

Matched peptides No.: [1] Sequence coverage %: [ ]

Matched sequences: K.EGLLVGISSGAAAAAAIR.I

Calculated Mr: **34495** Calculated *p*I: **6.26**

Probability Based Mowse Score:

Ions score is -10*Log(P), where P is the probability that the observed match is a random event.
Individual ions scores > 41 indicate identity or extensive homology (p<0.05).
Protein scores are derived from ions scores as a non-probabilistic basis for ranking protein hits.

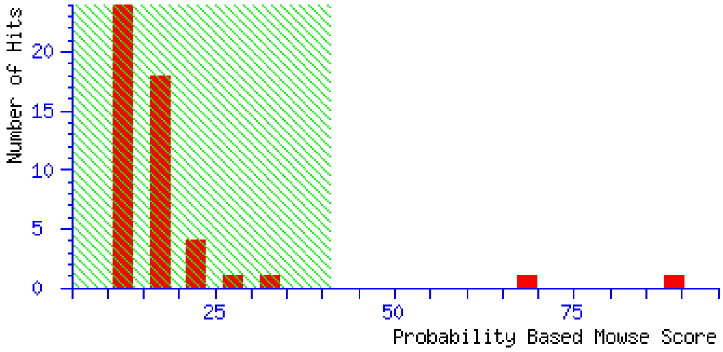

Matched peptide sequences: shown in Bold Red:

```
  1 MADAKSTIAK DVTELIGNTP LVYLNRVVDG CVARVAAKLE MMEPCSSVKD RIGYSMISDA
 61 ENKGLITPGE SVLIEPTSGN TGIGLAFIAA AKGYRLIICM PASMSLERRT ILRAFGAELV
121 LTDPARGMKG AVQKAEEIKA KTPNSYILQQ FENPANPKIH YETTGPEIWR GSGGKIDALV
181 SGIGTGGTVT GAGKYLKEQN PNIKLYGVEP VESAILSGGK PGPHKIQGIG AGFIPGVLDV
241 NLLDEVIQVS SEESIETAKL LALKEGLLVG ISSGAAAAAA IRIAKRPENA GKLIVAVFPS
301 FGERYLSTVL FESVKRETEN MVFEP
```

Spot No.: **109**

NCBI accession No.: gi|114199044 Species: *Malus x domestica*

PFF score: [163]

Protein name: caffeic acid O-methyltransferase

Matched peptides No.: [2] Sequence coverage %: [13]

Matched sequences: R.TEKEFEALAK.G;

K.AYGMTAFEYHGTDPR.F;
K.AYGMTAFEYHGTDPR.F + Oxidation (M);
K.WICHDWSDEHCLK.F

Calculated Mr: **39811** Calculated *p*I: **5.39**

Probability Based Mowse Score:

Ions score is -10*Log(P), where P is the probability that the observed match is a random event.
Individual ions scores > 44 indicate identity or extensive homology (p<0.05).
Protein scores are derived from ions scores as a non-probabilistic basis for ranking protein hits.

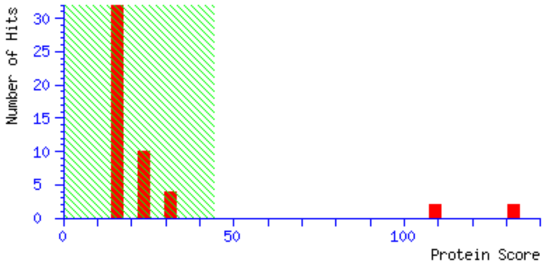

Matched peptide sequences: shown in Bold Red:

1 MGSTSESQSN SLTHTEDEAF LFAMQLCSAS VLPMVLKSAV ELDLLELMAK
51 AGPGAAISPS ELAAQLSTQN PEAPVMLDRM LRLLASYSVL NCTLRTLPDS
101 SVERLYSLAP VCKYLTKNAD GVSVAPLLLM NQDKVLMESW YHLKDAVLDG
151 GIPFNK**AYGM** **TAFEYHGTDP** **R**FNKVFNRGM SDHSTMSMKK ILEDYKGFEG
201 LNSIVDVGGG TGATVNMIVS KYPSIKGINF DLPHVIGDAP TYPGVEHVGG
251 DMFASVPKAD AIFMK**WICHD** **WSDEHCLK**FL KNCYEALPAN GKVIIAECIL
301 PEAPDTSLAT KNTVHVDIVM LAHNPGGKER **TEKEFEALAK** GAGFTGFARL
351 VALTTLGSWN STSN

Spot No.: 110

NCBI accession No.: gi|14423688 Species: *Hevea brasiliensis*

Protein name： Enolase 1

PMF score: 158 Sequence coverage %: 53

Matched peptides No.:18 Total peptides No.: 56

Calculated Mr: 48028 Calculated *p*I: 5.57

Probability Based Mowse Score:

Protein score is -10*Log(P), where P is the probability that the observed match is a random event. Protein scores greater than 73 are significant (p<0.05).

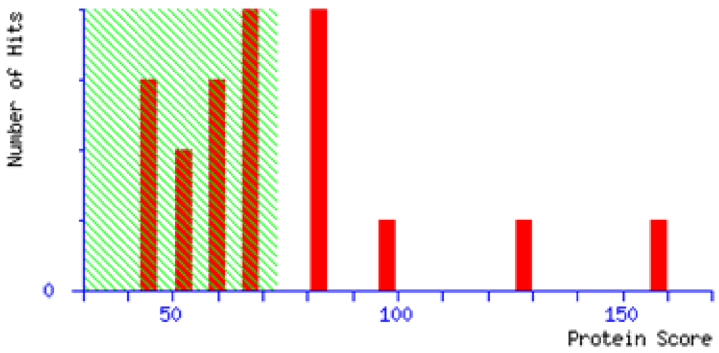

Matched peptide sequences: shown in Bold red

```
  1 MAITIVSVRA RQIFDSRGNP TVEADVKLSD GYLARAAVPS GASTGIYEAL
 51 ELRDGGSDYL GKGVSKAVEN VNIIIGPALV GKDPTDQVGI DNFMVQQLDG
101 TVNEWGWCKQ KLGANAILAV SLAVCKAGAH VKGIPLYEHI ANLAGNKNLV
151 LPVPAFNVIN GGSHAGNKLA MQEFMILPVG ASSFKEAMKM GAEVYHHLKS
201 VIKKKYGQDA TNVGDEGGFA PNIQENKEGL ELLKTAIAKA GYTGKVVIGM
251 DVAASEFYGS DQTYDLNFKE ENNNGSQKIS GEALKDLYKS FVAEYPIVSI
301 EDPFDQDDWA HYAKLTSEIG EKVQIVGDDL LVTNPKRVEK AIKEKACNAL
351 LLKVNQIGSV TESIEAVKMS KRAGWGVMAS HRSGETEDTF IADLSVGLAT
401 GQIKTGAPCR SERLAKYNQL LRIEEELGSE AVYAGANFRK PVEPY
```

Spot No.: 111

NCBI accession No.: gi|14423688 Species: *Hevea brasiliensis*

Protein name： Enolase 1

PMF score: 175 Sequence coverage %: 59

Matched peptides No.:25 Total peptides No.: 76

Calculated Mr: 48028 Calculated *p*I: 5.57

Probability Based Mowse Score:

Protein score is -10*Log(P), where P is the probability that the observed match is a random event.
Protein scores greater than 73 are significant (p<0.05).

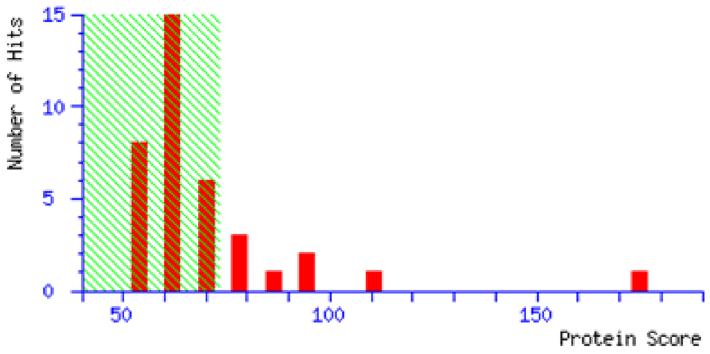

Matched peptide sequences: shown in Bold red

```
  1 MAITIVSVRA RQIFDSRGNP TVEADVKLSD GYLARAAVPS GASTGIYEAL
 51 ELRDGGSDYL GKGVSKAVEN VNIIIGPALV GKDPTDQVGI DNFMVQQLDG
101 TVNEWGWCKQ KLGANAILAV SLAVCKAGAH VKGIPLYEHI ANLAGNKNLV
151 LPVPAFNVIN GGSHAGNKLA MQEFMILPVG ASSFKEAMKM GAEVYHHLKS
201 VIKKKYGQDA TNVGDEGGFA PNIQENKEGL ELLKTAIAKA GYTGKVVIGM
251 DVAASEFYGS DQTYDLNFKE ENNNGSQKIS GEALKDLYKS FVAEYPIVSI
301 EDPFDQDDWA HYAKLTSEIG EKVQIVGDDL LVTNPKRVEK AIKEKACNAL
351 LLKVNQIGSV TESIEAVKMS KRAGWGVMAS HRSGETEDTF IADLSVGLAT
401 GQIKTGAPCR SERLAKYNQL LRIEEELGSE AVYAGANFRK PVEPY
```

Spot No.: 112

NCBI accession No.: gi|226897734 Species: *Thalictrum flavum*

Protein name： norcoclaurine 6-O-methyltransferase

PMF score: 85 Sequence coverage %: 45

Matched peptides No.:9 Total peptides No.:100

Calculated Mr: 25178 Calculated *p*I:6.05

Probability Based Mowse Score:

Protein score is -10*Log(P), where P is the probability that the observed match is a random event.
Protein scores greater than 73 are significant (p<0.05).

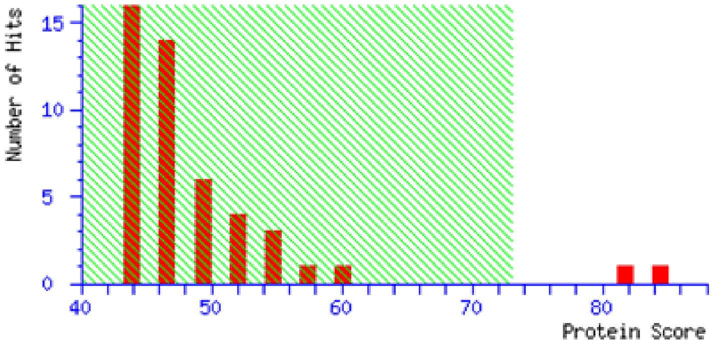

Matched peptide sequences: shown in Bold red:

1 **MEMINKENLS** **SQAK**LWNFIY GFADSLVLKS AVQLDLANII HNHGSPMTLS
51 ELSLHLPSQP VNQDALYRVL **RYLVHMKLFT** **KSSIDGELRY** GLAPPAKFLV
101 K**GWDKCMLGA** **ILTITDKDFM** **APWHYLK**EGI LNDGSTSTAF E**KALGTNIWD**
151 **YMAEHPEK**NQ LFNEGMANDT RLIMSALVK**E** **CSSMFDGITT** **IVDVGGGTGT**
201 **AVRNIAK**AFP HIKCTVYDLP HVIAD

Spot No.: **113**

NCBI accession No.: **gi| 255565071** Species: *Ricinus communis*

PFF score: [51]

Protein name: conserved hypothetical protein

Matched peptides No.: [2] Sequence coverage %: [2]

Matched sequences: R.LQVEELVFER.S; R.LQVEELVFER.S

Calculated Mr: **54103** Calculated *p*I: **5.53**

Probability Based Mowse Score:

Ions score is -10*Log(P), where P is the probability that the observed match is a random event.
Individual ions scores > 45 indicate identity or extensive homology (p<0.05).
Protein scores are derived from ions scores as a non-probabilistic basis for ranking protein hits.

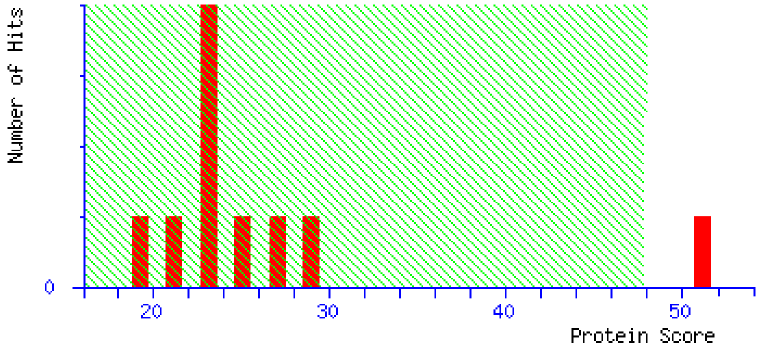

Matched peptide sequences: shown in Bold Red:

```
  1 MAPMLPKSVA IRSEFSRGYA YYMAENEAVR GFIRCEGENI LNPYVKIEVE
 51 TAQIDSKFVH LRFSHTNKYW GRSGPGFTQD DYWVSATSDQ PEEDTSKWSC
101 TLFEPIFDHG FLFLRHVQTG GRVRTDGPIG ATGPVSRLLL YFRNYDNVPY
151 LDFTFFDLGT LCILPKRVAF RGYNDMYLKA EWIERHEYLQ FSSDDVNEKA
201 AGYEITMMPD GHVRLRSNFF NKFWRRSPNW IWADSTDTVG SDRDTLFWPV
251 KVNDDTIALR NAGNNRFCSS LTAERKTDCL NAAVATITRE ARLQVEELVF
301 ERSIFNVRFL MEYARIFDER ATVAGWGSAE NQSEEAATLS ITVGYEDTTS
351 FTFSNSLAIT AGVTVGITTG LPRIAEGKIE ISTEVTNTLE WNRTTSETRT
401 ASATYLANVP ARSRIRIDYV ATRGTCNIPF SYTQRDRLSH DGSFATTQHV
451 DGVYTGVNYY SFHFEQPQIV PL
```

Spot No.: **114**

NCBI accession No.: **gi|1346735** Species: *Ricinus communis*

PFF score: [96]

Protein name: 2,3-bisphosphoglycerate-independent phosphoglycerate mutase

Matched peptides No.: [1] Sequence coverage %: [2]

Matched sequences: K.LPSHYLVSPPEIER.T

Calculated Mr: **57374** Calculated *p*I: **5.60**

Probability Based Mowse Score:

Ions score is -10*Log(P), where P is the probability that the observed match is a random event.
Individual ions scores > 45 indicate identity or extensive homology (p<0.05).
Protein scores are derived from ions scores as a non-probabilistic basis for ranking protein hits.

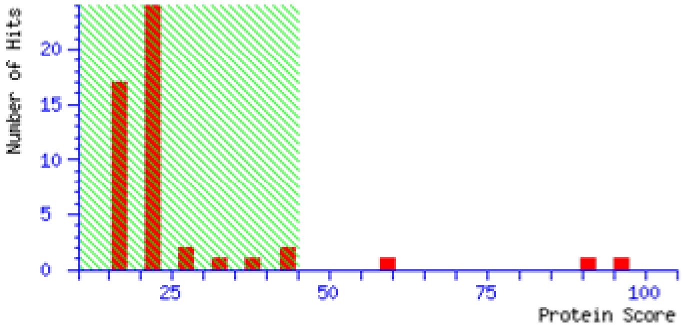

Matched peptide sequences: shown in Bold Red:

1 GEFTWKLADH PKLPKGKTIA MVVLDGWGEA KPDQYNCIHV AETPTMDSFK
51 KTAPERWRLI KAHGTAVGLP TEDDMGNSEV GHNALGAGRI YAQGAKLVDL
101 ALASGKIYEG EGFKYVKECF DKGTLHLIGL LSDGGVHSRL DQLQLLLKGA
151 AEHGAKRIRV HVLTDGRDVI DGTSVGFAET LEKDLENLRE KGVDAQVASG
201 GGRMYVTMDR YENDWNVVKR GWDAQVLGEA PYKFKSAVEA IKKLREEPKA
251 NDQYLPPFVI VDENGKPVGP IVDGDAVVTI NFRADRMVML AKALEYENFD
301 TFDRVRFPKI HYAGMLQYDG ELK**LPSHYLV** **SPPEIER**TSG EYLVHNGVHT
351 FACSETVKFG HVTFFWNGNR SGYFNPEMEE YVEIPSDVGI TFNVQPKMKA
401 IEIAEKARDA ILSGKFQQVR VNIPNGDMVG HTGDVEATVV GCKAADEAVK
451 MIIDAIEQVG GIYVVTADHG NAEDMVKRDK SGKPMADKSG KIQILTSHTL
501 QPVPIAIGGP GLTPGVRFRS DIPTGGLANV AATVMNLHGF EAPSDYEPTL
551 IEAVDN

Spot No.: 115

NCBI accession No.: gi|255565836   Species: *Ricinus communis*

Protein name： eukaryotic translation elongation factor, putative

PMF score: 141   Sequence coverage %: 40

Matched peptides No.:36   Total peptides No.:121

Calculated Mr: 94981   Calculated *p*I:5.87

Probability Based Mowse Score:

Protein score is -10*Log(P), where P is the probability that the observed match is a random event.
Protein scores greater than 73 are significant (p<0.05).

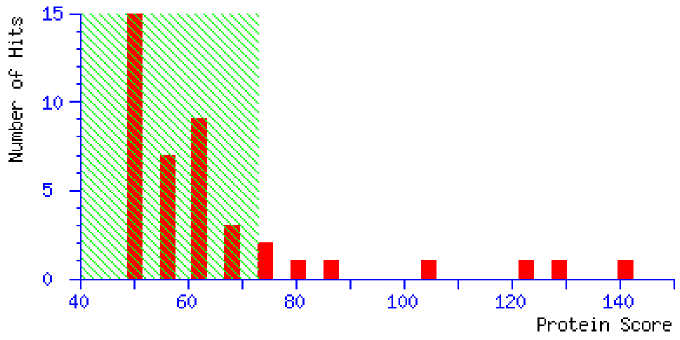

Matched peptide sequences: shown in Bold red:

```
  1 MVKFTAEELR RIMDYKHNIR NMSVIAHVDH GKSTLTDSLV AAAGIIAQEV
 51 AGDVRMTDTR QDEAERGITI KSTGISLYYE MTDEALKSFK GERQGNEYLI
101 NLIDSPGHVD FSSEVTAALR ITDGALVVVD CIEGVCVQTE TVLRQALGER
151 IRPVLTVNKM DRCFLELQVD GEEAYQTFSR VIENANVIMA TYEDPLLGDC
201 QVYPEKGTVA FSAGLHGWAF TLTNFAKMYA SKFGVDESKM MERLWGENFF
251 DPATKKWTSK NTGSPTCKRG FVQFCYEPIK QIINTCMNDQ KDKLWPMLQK
301 LGVTMKSDEK ELMGKALMKR VMQTWLPASS ALLEMMIFHL PSPAKAQRYR
351 VENLYEGPLD DPYATAIRNC DPEGPLMLYV SKMIPASDKG RFFAFGRVFA
401 GKVSTGLKVR IMGPNYVPGE KKDLYVKSVQ RTVIWMGKKQ ETVEDVPCGN
451 TVALVGLDQF ITKNATLTNE KEVDAHPIRA MKFSVSPVVR VAVQCKVASD
501 LPKLVEGLKR LAKSDPMVVC TIEESGEHII AGAGELHLEI CLKDLQDDFM
551 GGAEIIKSDP VVSFRETVLE KSCRVVMSKS PNKHNRLYME ARPMEEGLAE
601 AIDEGRIGPR DDPKARAKIL SEEFGWDKDL AKKIWCFGPE TTGPNMVVDM
651 CKGVQYLNEI KDSVVAGFQW ASKEGALAEE NMRGICFEVC DVVLHADAIH
701 RGGGQVIPTA RRVIYASQLT AKPRLLEPVY LVEIQAPEQA LGGIYSVLNQ
751 KRGHVFEELQ RPGTPLYNIK AYLPVIESFG FSGTLRAATS GQAFPQCVFD
801 HWDMMSSDPM EPGSQAANLV TEIRKRKGLK EQMTPLSEFE DKL
```

Spot No.: **116**

NCBI accession No.: **gi|6707018**   Species: *Hevea brasiliensis*

Protein name: latex protein allergen Hev b 7

PMF score: **147**   Sequence coverage %:**64**

Matched peptides No.: **17**   Total peptides No.: **60**

Calculated Mr: **43107**   Calculated *p*I: **5.00**

Probability Based Mowse Score:

Protein score is -10*Log(P), where P is the probability that the observed match is a random event.
Protein scores greater than 73 are significant ($p<0.05$).

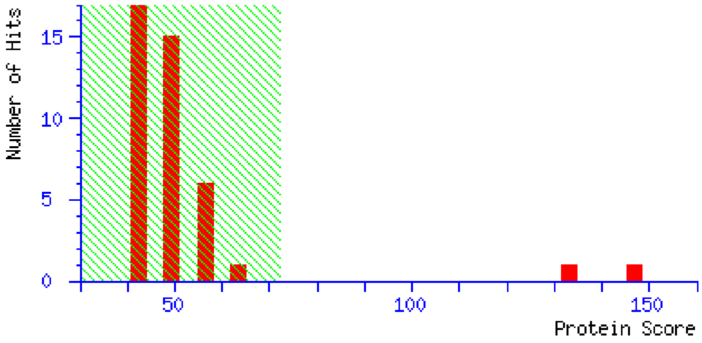

Matched peptide sequences: shown in Bold Red:

```
  1 MATGSTPLTQ GKKITVLSID GGGIRGIIPG IILASLESKL QDLDGPDARI
 51 ADYFDIIAGT STGGLITTML TAPNEDKKPM YQAKDIKDFY LENCPKIFPK
101 ESRDNYDPIH SIGPIYDGEY LRELCNNLLK DLTVKDTLTD VIIPTFDIKL
151 LLPVIFSSDD AKCNALKNAR LADVCISTSA APVLLPAHSF TTEDDKNIHT
201 FELIDGGVAA TNPTLLALTH IRNEIIRQNP RFIGANLTES KSRLVLSLGT
251 GKSEYKEKYN ADMTSKWRLY NWALYNGNSP AVDIFSNASS DMVDFHLSAL
301 FKSLDCEDYY LRIQDDTLTG EESSGHIATE ENLQRLVEIG TELLEKQESR
351 INLDTGRLES IPGAPTNEAA IAKFAKLLSE ERKLRQLK
```

Spot No.: **117**

NCBI accession No.: **gi|51315784** Species: *Hevea brasiliensis*

PFF score: [59]

Protein name: Esterase

Matched peptides No.: [1] Sequence coverage %: [4]

Matched sequences: K.AAAFYPLNPPYGETFFHR.S

Calculated Mr: **43871** Calculated *p*I: **5.00**

Probability Based Mowse Score:

Ions score is -10*Log(P), where P is the probability that the observed match is a random event.
Individual ions scores > 43 indicate identity or extensive homology (p<0.05).
Protein scores are derived from ions scores as a non-probabilistic basis for ranking protein hits.

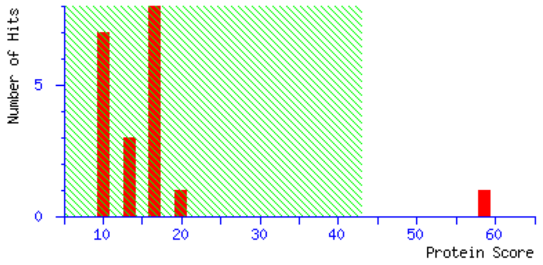

Matched peptide sequences: shown in Bold Red:

```
  1 MEFPETNNNP IITLSFLLCM LSLAYASETC DFPAIFNFGD SNSDTGGKAA
 51 AFYPLNPPYG ETFFHRSTGR YSDGRLIIDF IAESFNLPYL SPYLSSLGSN
101 FKHGADFATA GSTIKLPTTI IPAHGGFSPF YLDVQYSQFR QFIPRSQFIR
151 ETGGIFAELV PEEYYFEKAL YTFDIGQNDL TEGFLNLTVE EVNATVPDLV
201 NSFSANVKKI YDLGARTFWI HNTGPIGCLS FILTYFPWAE KDSAGCAKAY
251 NEVAQHFNHK LKEIVAQLRK DLPLATFVHV DIYSVKYSLF SEPEKHGFEF
301 PLITCCGYGG KYNFSVTAPC GDTVTADDGT KIVVGSCACP SVRVNWDGAH
351 YTEAANEYFF DQISTGAFSD PPVPLNMACH KTESLRTLAS V
```

Spot No.: 118

NCBI accession No.: **gi|38122474** Species: *Hevea brasiliensis*

Protein name： rubber elongation factor

PMF score: **98** Sequence coverage %: **57**

Matched peptides No.: **9** Total peptides No.: **44**

Calculated Mr: **19570** Calculated *p*I: **5.06**

Probability Based Mowse Score:

Protein score is -10*Log(P), where P is the probability that the observed match is a random event. Protein scores greater than 73 are significant (p<0.05).

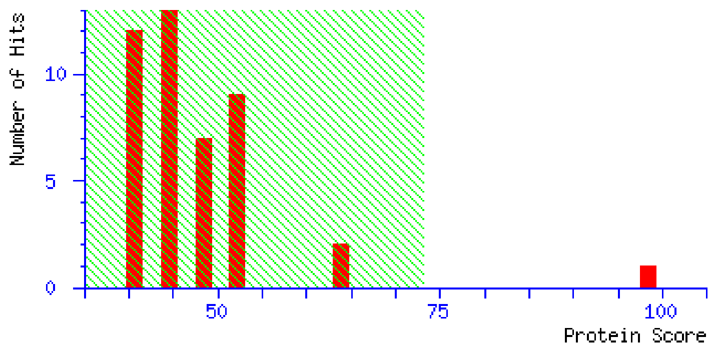

Matched peptide sequences: shown in Bold Red

```
  1 MAEGEEEVNI QEEANKGEEN PQEEANIQEE TNKGEENIQE EANIEEEANK
 51 EEESLKYLDF VQAATVYARA SFSKLYLFAK DKSGPFKPGV NTVESRFKSV
101 VRPVYNKFQP VPNKVLKFAD RRVDAYVTVL DRIVPPIVKR ASIQAYSVAP
151 GAALAVASYL PLHTKRLSKV LYGDG
```

Spot No.: **119**

NCBI accession No.: **gi| 3023685** Species: *Alnus glutinosa*

PFF score: [51]

Protein name: **Enolase; AltName: Full=2-phospho-D-glycerate hydro-lyase; AltName: Full=2-phosphoglycerate dehydratase**

Matched peptides No.: [1] Sequence coverage %: [3]

Matched sequences: R.IEEELGSEAVYAGANFR.T

Calculated Mr: **47796** Calculated *p*I: **5.41**

Probability Based Mowse Score:

Ions score is -10*Log(P), where P is the probability that the observed match is a random event.
Individual ions scores > 40 indicate peptides with significant homology.
Individual ions scores > 44 indicate identity or extensive homology (p<0.05).
Protein scores are derived from ions scores as a non-probabilistic basis for ranking protein hits.

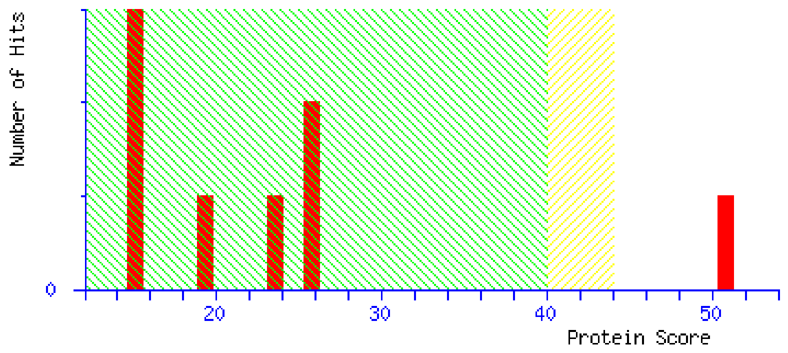

Matched peptide sequences: shown in Bold Red:

1 MAEITHIKAR QIFDSRGNPT VEAEVTTANG VVSRAAVPSG ASTGVYEALE
51 LRDGGSDYLG KGVLKAVDNV NKIIGPALIG KDATEQTAID IDFMFQQLDG
101 TVNEWGWCKQ KLGANAILAV SLAVCKAGAS VKKIPLYKHI ANLAGNPKLV
151 LPVPAFNVIN GGSHAGNKLA MQEFMILPVG ASSFKEAMKM GVEVYHHLKA
201 VIKKKYGQDA TNVGDEGGFA PNIQENKEGL ELLKTAIAKA GYVVIGMDVA
251 ASEFYEKDKD ITNFKEENND GSQKISADQL KDLYKSFVDE YPIVSIEDPF
301 DQDDWEHYSK LTAEIGEKVQ IVGDDLLVTN PKRVEKAIKE KACNALLLKV
351 NQIGSVTESI EAVKMSKRAG WGVMAHRSGE TEDTFIADLS VGLATGQIKT
401 GAPCRSERLA KYNQLLR**IEE** **ELGSEAVYAG** **ANFR**TPVEPY

Spot No.: 120

NCBI accession No.: **gi**| 351723039   Species: *Glycine max*

Protein name： uncharacterized protein LOC100305754 precursor

PMF score: **81**   Sequence coverage %: **51**

Matched peptides No.: **8**   Total peptides No.: **28**

Calculated Mr: **21968**   Calculated *p*I: **4.83**

Probability Based Mowse Score:

Protein score is -10*Log(P), where P is the probability that the observed match is a random event. Protein scores greater than 73 are significant (p<0.05).

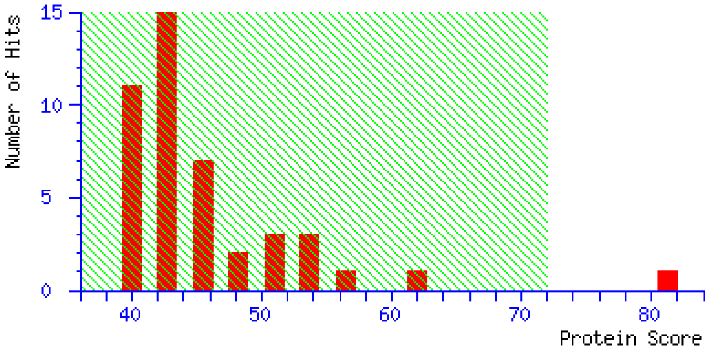

Matched peptide sequences: shown in Bold Red

```
  1  MSMRSIGTSL  SLMVWLVIAT  SALAKSDNPP  VLDTQGNPLE  PGKDYYIKPA
 51  ITDVGGRVTL  LSRNNPCPLY  VGQENSDAAE  GLPLFFTPFA  EEDDVVKVNR
101  DFKLTFSAAS  ICVQGTNWNL  AEKDSESGRR  LIAASGRDDY  FRITETPIKG
151  SYYIGWCPTD  VCPFCRFDCG  IVGGLRENGK  ILLALDGNVL  PVVFEKAY
```

Spot No.: **121**

NCBI accession No.: **gi| 255554865** Species: *Ricinus communis*

PFF score: [55]

Protein name: lactoylglutathione lyase, putative

Matched peptides No.: [1] Sequence coverage %: [3]

Matched sequences: K.FYTECFGMK.L

Calculated Mr: **31641** Calculated *p*I: **7.63**

Probability Based Mowse Score:

Ions score is -10*Log(P), where P is the probability that the observed match is a random event.
Individual ions scores > 40 indicate peptides with significant homology.
Individual ions scores > 44 indicate identity or extensive homology (p<0.05).
Protein scores are derived from ions scores as a non-probabilistic basis for ranking protein hits.

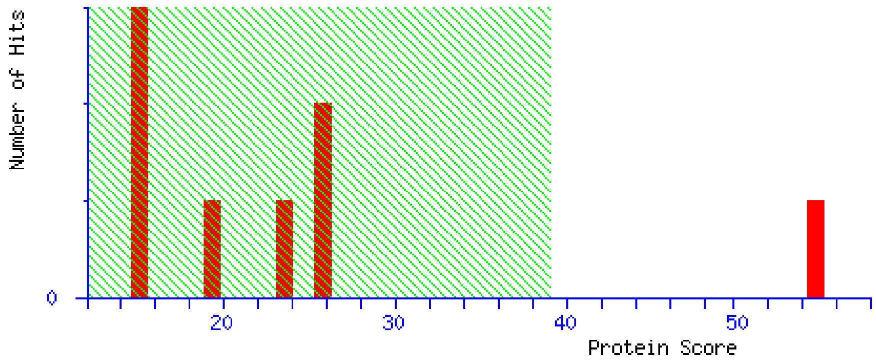

Matched peptide sequences: shown in Bold Red:

```
  1  MAAEATAPNA  DVLEWPKKDK  RRLLHAVYRV  GDLDRTIKFY  TECFGMKLLR
 51  KRDIPEEKYS  NAFLGFGPEE  TNFVVELTYN  YGVTSYDIGT  GFGHFAIATQ
101  DVYKLVEEVL  AKGGAVTREP  GPVKGGTTVI  AFVKDPDGYI  FELIQRGPTP
151  EPLCQVMLRV  GDLDRSIRFY  EKALGMKLLR  KVDKPEYKYT  LAMMGYADEY
201  ETTVLELTYN  YGVTEYTKGN  AYAQVAISTD  DVYKSAEVVN  LVTQELGGKI
251  TRQPGPIPGL  NTKITSFLDP  DGWKTKRKRR
```

Spot No.: **122**

NCBI accession No.: **gi|118485535** Species: *Populus trichocarpa*

PFF score: [83]

Protein name: unknown

Matched peptides No.: [1] Sequence coverage %: [8]

Matched sequences: K.VATPAQAQEVHLELR.K

Calculated Mr: **18429** Calculated *p*I: **5.59**

Probability Based Mowse Score:

Ions score is -10*Log(P), where P is the probability that the observed match is a random event.
Individual ions scores > 45 indicate identity or extensive homology (p<0.05).
Protein scores are derived from ions scores as a non-probabilistic basis for ranking protein hits.

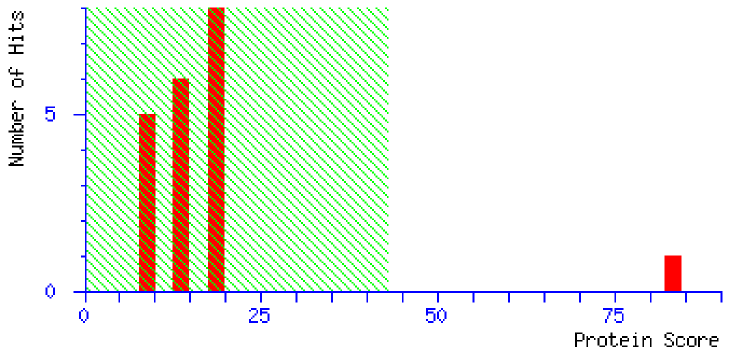

Matched peptide sequences: shown in Bold Red:

```
  1 MLVNLGIPWV ILGHSERRSL LNESNEFVGD KVAYALSLGL KVIACVGETL
 51 QQRESGSTMA VVAAQTKAIA ANVSYWDNVV MAYEPVWAIG TGKVATPAQA
101 QEVHLELRKW FHDNVGAEVA ASTRIIYGGS VNGANCKELA GQPDVDGFLV
151 GGASLKPEFI DIIKSATVKS QL
```

Spot No.: **123**

NCBI accession No.: **gi|15225839** Species: *Arabidopsis thaliana*

Protein name: endopeptidase/ peptidase/ threonine-type endopeptidase

PMF Mascot score: **86** Sequence coverage %: **40**

Matched peptides No.: **14** Total peptides No.: **56**

Calculated Mr: **27645** Calculated *p*I: **5.93**

Probability Based Mowse Score:

Protein score is -10*Log(P), where P is the probability that the observed match is a random event. Protein scores greater than 73 are significant ($p<0.05$).

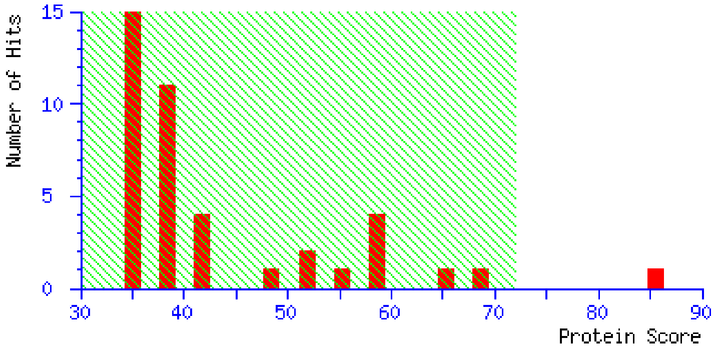

Matched peptide sequences: shown in Bold Red:

1 MSSIGTGYDL SVTTFSPDGR **VFQIEYAAK**A VDNSGTVVGI KCKDGIVMGV
51 EKLIASK**MML** **PGSNRRIHSV** **HRHAGMAVAG** **LAADGRQIVA** **RAKSEARSYE**
101 **SVYGDAVPVK** ELSERVASYV HLCTLYWWLR PFGCGVILGG YDR**DGPQLYM**
151 **IEPSGISYRY** **FGAAIGK**GKQ AAKTEIEKLN LSEMTCKEGV IEVAKIIYKL
201 HDEAK**DKAFE** **LEMSWICEES** **K**REHQKVPDD LLEEAKTAAK TALEEMDAD

Spot No.: **124**

NCBI accession No.: **gi| 10862818** Species: *Hevea brasiliensis*

Protein name: IgE-binding protein Mn SOD

PMF Mascot score: **81** Sequence coverage %: **37**

Matched peptides No.: **5** Total peptides No.: **8**

Calculated Mr: **22958** Calculated *p*I: **6.06**

Probability Based Mowse Score:

Protein score is -10*Log(P), where P is the probability that the observed match is a random event.
Protein scores greater than 73 are significant ($p<0.05$).

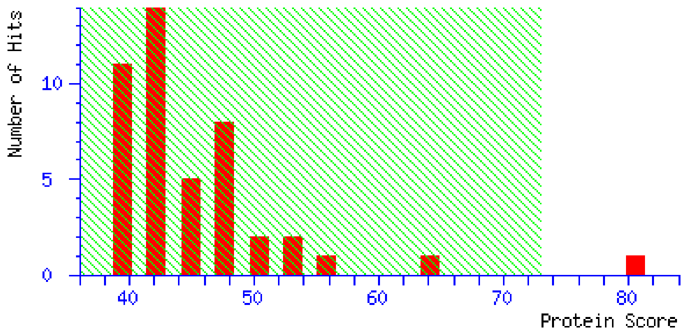

Matched peptide sequences: shown in Bold Red:

```
  1  QTFSLPDLPY DYGALEPAIS GEIMQLHHQK HHQTYITNYN KALEQLNDAI
 51  EKGDSAAVVK LQSAIKFNGG GHVNHSIFWK NLAPVREGGG ELPHGSLGWA
101  IDADFGSLEK LIQLMNAEGA ALRGSGWVWL ALDKELKKLV VETTANQDPL
151  VTKGPTLVPL LGIDVWEHAY YLQYKNVRPD YLKNIWKVMN WKYASEVYAK
201  ECPSS
```

Spot No.: **125**

NCBI accession No.: **gi|1170781** Species: *Glycine max*

PFF score: [51]

Protein name: Glutathione S-transferase 3

Matched peptides No.: [1] Sequence coverage %: [5]

Matched sequences: K.SPLMPSDPYKR.S+Oxidation (M)

Calculated Mr: **26112** Calculated *p*I: **5.58**

Probability Based Mowse Score:

Ions score is -10*Log(P), where P is the probability that the observed match is a random event.
Individual ions scores > 45 indicate identity or extensive homology (p<0.05).
Protein scores are derived from ions scores as a non-probabilistic basis for ranking protein hits.

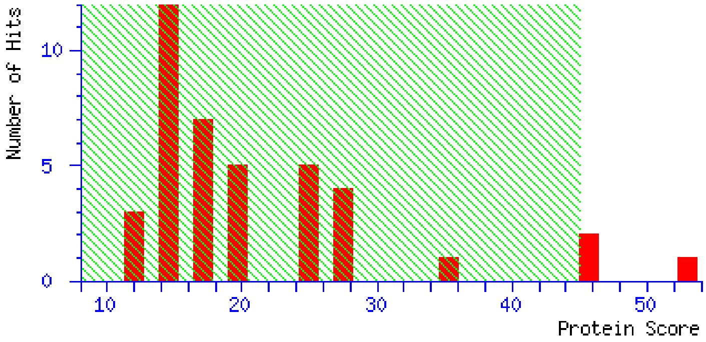

Matched peptide sequences: shown in Bold Red:

```
  1 MSDEVVLLDT WASMYGMRAR IALAEKGVRY EYKEENLMNR SPLLLQMNPI
 51 HKKIPVLIHN GKPICESAII VQYIDEVWND KSPLMPSDPY KRSQARFWVD
101 YIDKKIYDTW KKMWLSKGEE HEEGKKELIS IFKQLEETLT DKPFYGDDTF
151 GFVDLCLITF SSWFYTYETY GNFKMEEECP KLMAWVKRCM ERETVSNTLP
201 DAKKVYGLIV ELQKTLESK
```

(Matched in bold red: SPLMPSDPY KR)

Spot No.: **126**

NCBI accession No.: **gi|29170601** Species: *Hevea brasiliensis*

PFF score: [97]

Protein name: small rubber particle protein

Matched peptides No.: [1] Sequence coverage %: [12]

Matched sequences: K.ENENFQQEANEQEEK.L

Calculated Mr: **12769** Calculated *p*I: **4.61**

Probability Based Mowse Score:

Ions score is -10*Log(P), where P is the probability that the observed match is a random event.
Individual ions scores > 44 indicate identity or extensive homology (p<0.05).
Protein scores are derived from ions scores as a non-probabilistic basis for ranking protein hits.

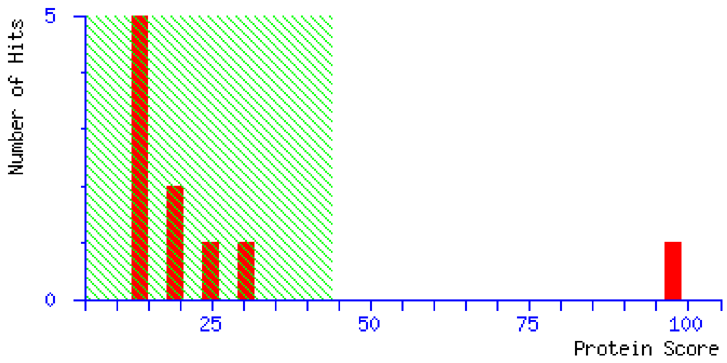

Matched peptide sequences: shown in Bold Red:

```
  1 MAEGKENENF QQEANEQEEK LKYLEFVQAT TDNAVTALSN IYLYAKDNSG
 51 PLKPGVETIE GVAKTVVIPA SKIPTEAIKF ADRAVDASFT TLQNIVPSVL
101 KQLPTQACDT SVKESAE
```

Spot No.: 127

NCBI accession No.: **gi|2832430** Species: *Hevea brasiliensis*

PFF score: [84] Protein name: prohevein

Matched peptides No.: [1] Sequence coverage %: [10]

Matched sequences: K.YGWTAFCGPVGAHGQPSCGK.C

Calculated Mr: **20852** Calculated *p*I: **5.64**

Probability Based Mowse Score:

Ions score is -10*Log(P), where P is the probability that the observed match is a random event.
Individual ions scores > 43 indicate identity or extensive homology (p<0.05).
Protein scores are derived from ions scores as a non-probabilistic basis for ranking protein hits.

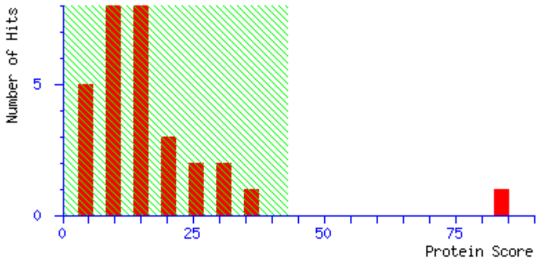

Matched peptide sequences: shown in Bold Red:

```
  1 EQCGRQAGGK LCPNNLCCSQ WGWCGSTDEY CSPDHNCQSN CKDSGEGVGG
 51 GSASNVLATY HLYNSQDHGW DLNAASAYCS TWDANKPYSW RSKYGWTAFC
101 GPVGAHGQPS CGKCLSVTNT GTGAKATVRI VDQCSNGGLD LDVNVFRQLD
151 TDGKGYERGH LTVNYQFVDC GDSFNPLFSV MKSSVIN
```

Spot No.: **128**

NCBI accession No.: **gi|224054224** Species: *Populus trichocarpa*

PFF score: [68] Protein name: predicted protein

Matched peptides No.: [2] Sequence coverage %: [12]

Matched sequences: R.AYVGDRPTWR.N;

R.NPQHPWR.V

Calculated Mr: **15003** Calculated *p*I: **6.82**

Probability Based Mowse Score:

Ions score is -10*Log(P), where P is the probability that the observed match is a random event.
Individual ions scores > 45 indicate identity or extensive homology (p<0.05).
Protein scores are derived from ions scores as a non-probabilistic basis for ranking protein hits.

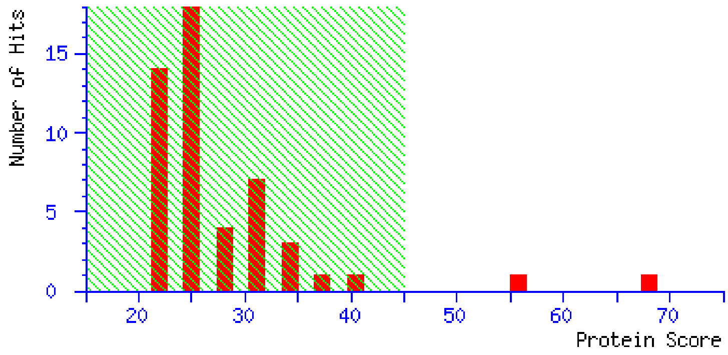

Matched peptide sequences: shown in Bold Red:

```
  1 MTVKLVDATI SSFGSVFEKF KAEAPKNKVN LILFLADNDP STNLSWCPDC
 51 VRAEPVILKK LEALPDDVAL LRAYVGDRPT WRNPQHPWRV DSRFKLKGVP
101 TLISWENDAV KGRLEDYEAH LEHKINALVS GN
```

Spot No.: **129**

NCBI accession No.: **gi| 329750601** Species: *Hevea brasiliensis*

PFF score: [61] Protein name: thioredoxin H-type 2

Matched peptides No.: [2] Sequence coverage %: [12]

Matched sequences: R.AYVGDRPTWR.N;

R.NPQHPWR.V

Calculated Mr: **13295** Calculated *p*I: **5.89**

Probability Based Mowse Score:

Ions score is -10*Log(P), where P is the probability that the observed match is a random event.
Individual ions scores > 45 indicate identity or extensive homology (p<0.05).
Protein scores are derived from ions scores as a non-probabilistic basis for ranking protein hits.

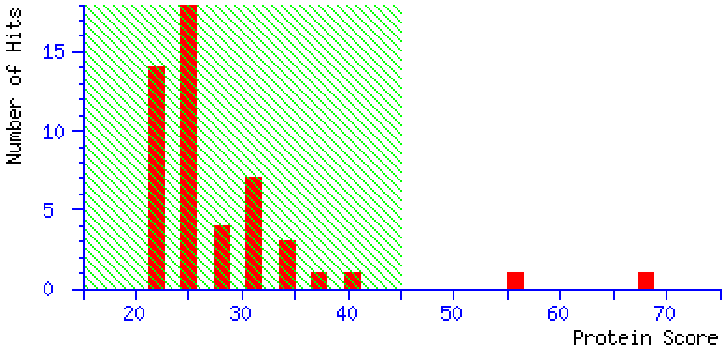

Matched peptide sequences: shown in Bold Red:

```
  1 MTVKLVDATI SSFGSVFEKF KAEAPKNKVN LILFLADNDP STNLSWCPDC
 51 VRAEPVILKK LEALPDDVAL LRAYVGDRPT WRNPQHPWRV DSRFKLKGVP
101 TLISWENDAV KGRLEDYEAH LEHKINALVS GN
```

Spot No.: **130**

NCBI accession No.: **gi|268321205**

Plantspecies: ***Hevea brasiliensis*** Protein name：**latex cystatin**

PMF score: **81** Sequence coverage %: **48**

Matched peptides No.: **6** Total peptides No.:**42**

Calculated Mr: **11248** Calculated *p*I: **5.45**

Probability Based Mowse Score:

Protein score is -10*Log(P), where P is the probability that the observed match is a random event.
Protein scores greater than 73 are significant (p<0.05).

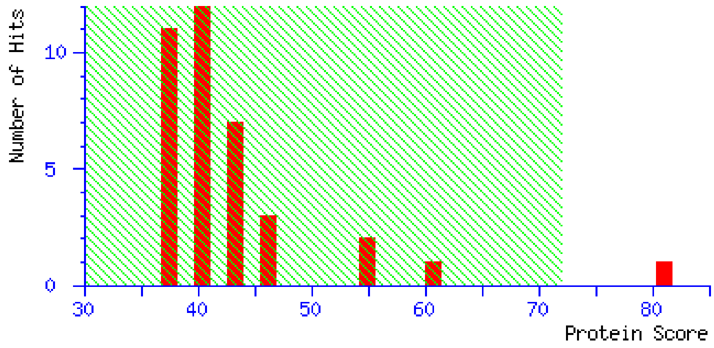

Matched peptide sequences: shown in Bold Red

```
  1 MAKLGGVKEV EGSANSVEIN SLARYAVDDY NQKQNALLEF KKVVNAKQQV
 51 VAGTIYYITL EVIDGGQKKV YEAKVWEKPW LNFKEVQEFK LIGDAPSDST
101 A
```

Spot No.: **131**

NCBI accession No.: **gi|132270** Species: *Hevea brasiliensis*

PFF score: [539]

Protein name: small rubber particle protein

Matched peptides No.: [5] Sequence coverage %: [39]

Matched sequences: K.DKSGPLQPGVDIIEGPVK.N;
K.SGPLQPGVDIIEGPVK.N;
K.FVDSTVVASVTIIDR.S;
K.DASIQVVSAIR.A;
R.SLASSLPGQTK.I

Calculated Mr: **14713** Calculated *p*I: **5.04**

Probability Based Mowse Score:

Ions score is -10*Log(P), where P is the probability that the observed match is a random event.
Individual ions scores > 44 indicate identity or extensive homology (p<0.05).
Protein scores are derived from ions scores as a non-probabilistic basis for ranking protein hits.

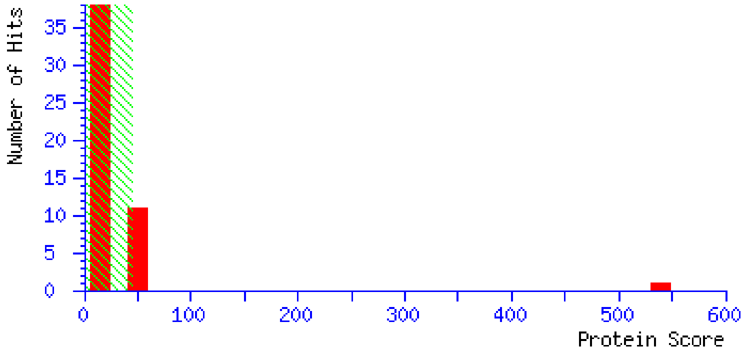

Matched peptide sequences: shown in Bold Red:

```
  1 MAEDEDNQQG QGEGLKYLGF VQDAATYAVT TFSNVYLFAK DKSGPLQPGV
 51 DIIEGPVKNV AVPLYNRFSY IPNGALKFVD STVVASVTII DRSLPPIVKD
101 ASIQVVSAIR AAPEAARSLA SSLPGQTKIL AKVFYGEN
```

Spot No.: 132

NCBI accession No.: **gi|132270** Species: *Hevea brasiliensis*

Protein name: **Rubber elongation factor protein**

PMF score: **138** Sequence coverage %: **81**

Matched peptides No.: **10** Total peptides No.: **28**

Calculated Mr: **14713** Calculated *p*I: **5.04**

Probability Based Mowse Score:

Protein score is -10*Log(P), where P is the probability that the observed match is a random event.
Protein scores greater than 73 are significant (p<0.05).

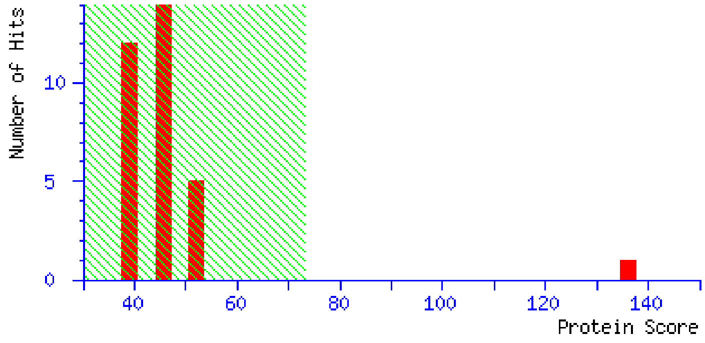

Matched peptide sequences: shown in Bold Red

```
  1 MAEDEDNQQG QGEGLKYLGF VQDAATYAVT TFSNVYLFAK DKSGPLQPGV
 51 DIIEGPVKNV AVPLYNRFSY IPNGALKFVD STVVASVTII DRSLPPIVKD
101 ASIQVVSAIR AAPEAARSLA SSLPGQTKIL AKVFYGEN
```

Spot No.: **133**

NCBI accession No.: **gi|242083244**

Plantspecies: ***Sorghum bicolor***

Protein name： **hypothetical protein SORBIDRAFT_08g008236**

PMF score: **81** Sequence coverage %: **89**

Matched peptides No.: **7** Total peptides No.:**33**

Calculated Mr: **8620** Calculated *p*I: **6.4**

Probability Based Mowse Score:

Protein score is -10*Log(P), where P is the probability that the observed match is a random event. Protein scores greater than 73 are significant (p<0.05).

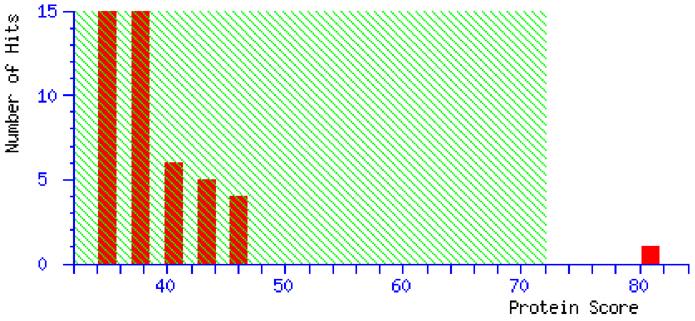

Matched peptide sequences: shown in Bold Red

```
 1 CVENIILSVL FWSKVQLCLR ASQPLLVALR IVDGDETPID PKIMVAMDVA
51 KAIIKESLKD KPILLNEVLK YYDNR
```

Spot No.: **134**

NCBI accession No.: **gi|29170601** Species: *Hevea brasiliensis*

PFF score: [85]

Protein name: small rubber particle protein

Matched peptides No.: [1] Sequence coverage %: [15]

Matched sequences: R.AVDASFTTLQNIVPSVLK.Q

Calculated Mr: **12769** Calculated *p*I: **4.61**

Probability Based Mowse Score:

Ions score is -10*Log(P), where P is the probability that the observed match is a random event.
Individual ions scores > 39 indicate peptides with significant homology.
Individual ions scores > 44 indicate identity or extensive homology (p<0.05).
Protein scores are derived from ions scores as a non-probabilistic basis for ranking protein hits.

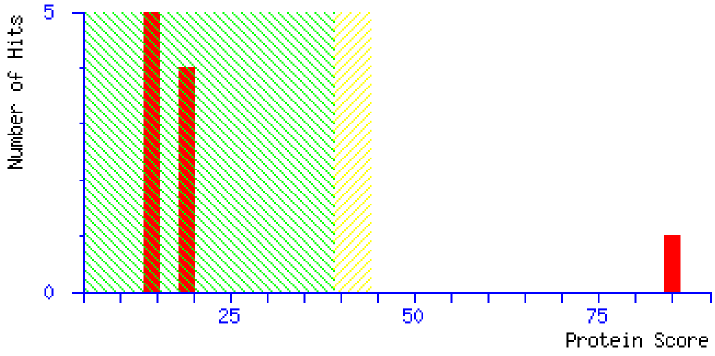

Matched peptide sequences: shown in Bold Red:

```
  1 MAEGKENENF QQEANEQEEK LKYLEFVQAT TDNAVTALSN IYLYAKDNSG
 51 PLKPGVETIE GVAKTVVIPA SKIPTEAIKF ADRAVDASFT TLQNIVPSVL
101 KQLPTQACDT SVKESAE
```

Spot No.: **135**

NCBI accession No.: **gi|29170601** Species: *Hevea brasiliensis*

PFF score: [485]

Protein name: small rubber particle protein

Matched peptides No.: [4] Sequence coverage %: [53]

Matched sequences: K.ENENFQQEANEQEEK.L;
K.DNSGPLKPGVETIEGVAK.T;
R.AVDASFTTLQNIVPSVLK.Q;
K.QLPTQACDTSVK.E

Calculated Mr: **12769** Calculated *p*I: **4.61**

Probability Based Mowse Score:

Ions score is -10*Log(P), where P is the probability that the observed match is a random event.
Individual ions scores > 44 indicate identity or extensive homology (p<0.05).
Protein scores are derived from ions scores as a non-probabilistic basis for ranking protein hits.

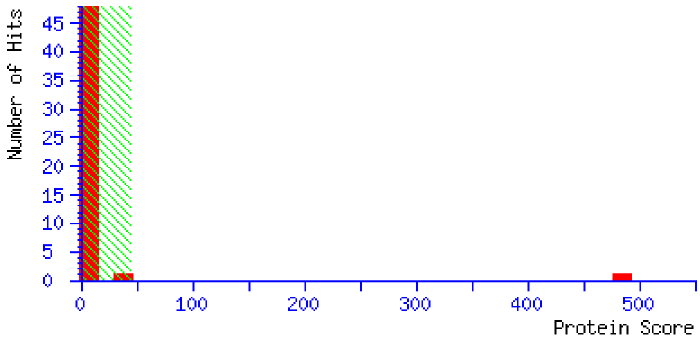

Matched peptide sequences: shown in Bold Red:

```
  1 MAEGKENENF QQEANEQEEK LKYLEFVQAT TDNAVTALSN IYLYAKDNSG
 51 PLKPGVETIE GVAKTVVIPA SKIPTEAIKF ADRAVDASFT TLQNIVPSVL
101 KQLPTQACDT SVKESAE
```

Spot No.: 136

NCBI accession No.: **gi|354318** Species: *Triticum aestivum*

PFF score: [128] Protein name: calmodulin

Matched peptides No.: [1] Sequence coverage %: [10]

Matched sequences: R.VFDKDQDGFISAAELR.H

Calculated Mr: **16876** Calculated *p*I: **4.10**

Probability Based Mowse Score:

Ions score is -10*Log(P), where P is the probability that the observed match is a random event.
Individual ions scores > 45 indicate identity or extensive homology (p<0.05).
Protein scores are derived from ions scores as a non-probabilistic basis for ranking protein hits.

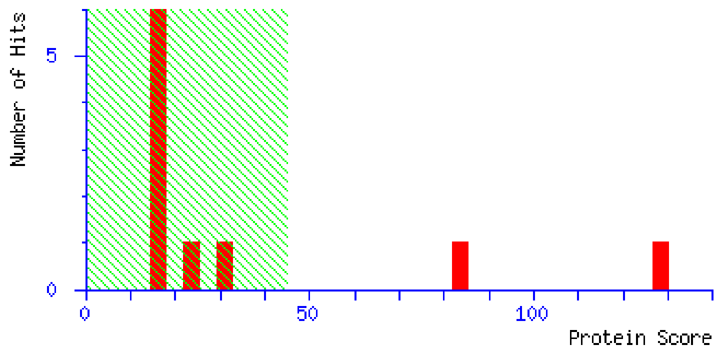

Matched peptide sequences: shown in Bold Red:

```
  1 ADQLTDEQNI AEFKEAFSLF DKDGDGCITT KELGTVMRSL GQNPTEAELQ
 51 DMINEVDADG NGTIDFPEFL NLMARKMKDT DSEEELKEAF RVFDKDQDGF
101 ISAAELRHVM TNLGEKLTDE EVDEMIREAD VDGDGQINYE EFVKVMMAK
```

Spot No.: 137

NCBI accession No.: gi|14423933 Species: *Hevea brasiliensis*

Protein name: Small rubber particle protein

PMF score: **84** Sequence coverage %: **65**

Matched peptides No.: **9** Total peptides No.:**43**

Calculated Mr: **22331** Calculated *p*I: **4.80**

Probability Based Mowse Score:

Protein score is -10*Log(P), where P is the probability that the observed match is a random event. Protein scores greater than 73 are significant (p<0.05).

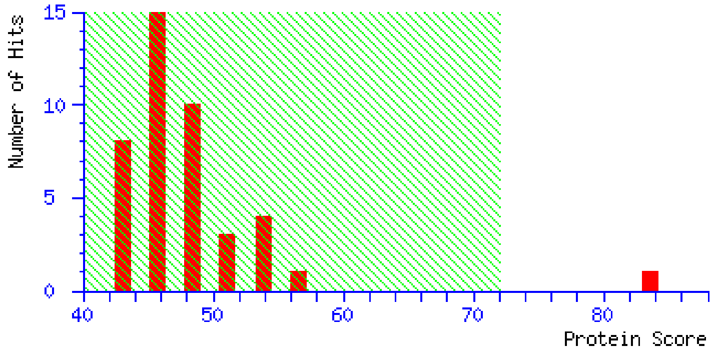

Matched peptide sequences: shown in Bold Red

```
  1 MAEEVEEERL KYLDFVRAAG VYAVDSFSTL YLYAKDISGP LKPGVDTIEN
 51 VVKTVVTPVY YIPLEAVKFV DKTVDVSVTS LDGVVPPVIK QVSAQTYSVA
101 QDAPRIVLDV ASSVFNTGVQ EGAKALYANL EPKAEQYAVI TWRALNKLPL
151 VPQVANVVVP TAVYFSEKYN DVVRGTTEQG YRVSSYLPLL PTEKITKVFG
201 DEAS
```

Spot No.: 138

NCBI accession No.: gi|14423933   Species: *Hevea brasiliensis*

Protein name: Small rubber particle protein

PMF score: **162**   Sequence coverage %: **85**

Matched peptides No.: **13**   Total peptides No.:**43**

Calculated Mr: **22331**   Calculated *p*I: **4.80**

Probability Based Mowse Score:

Protein score is -10*Log(P), where P is the probability that the observed match is a random event. Protein scores greater than 73 are significant (p<0.05).

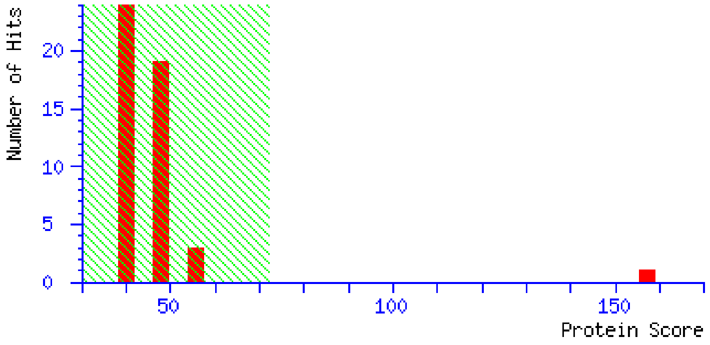

Matched peptide sequences: shown in Bold Red

```
  1 MAEEVEEERL KYLDFVRAAG VYAVDSFSTL YLYAKDISGP LKPGVDTIEN
 51 VVKTVVTPVY YIPLEAVKFV DKTVDVSVTS LDGVVPPVIK QVSAQTYSVA
101 QDAPRIVLDV ASSVFNTGVQ EGAKALYANL EPKAEQYAVI TWRALNKLPL
151 VPQVANVVVP TAVYFSEKYN DVVRGTTEQG YRVSSYLPLL PTEKITKVFG
201 DEAS
```

Spot No.: 139

NCBI accession No.: gi|14423688 Species: *Hevea brasiliensis*

Protein name： **Enolase 1**

PMF score: 107 Sequence coverage %: 44

Matched peptides No.:14 Total peptides No.:47

Calculated Mr: 48029 Calculated *p*I:5.57

Probability Based Mowse Score:

Protein score is -10*Log(P), where P is the probability that the observed match is a random event.
Protein scores greater than 73 are significant (p<0.05).

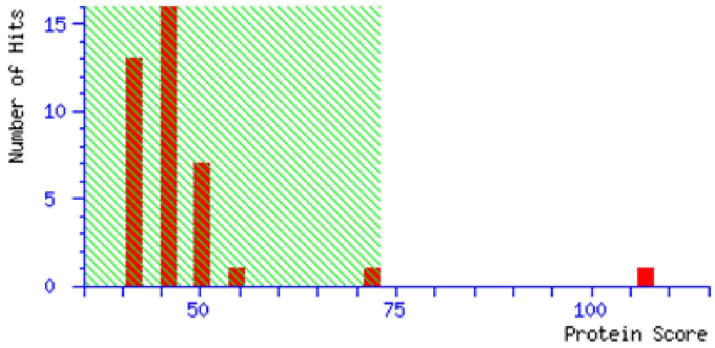

Matched peptide sequences: shown in Bold red:

1	MAITIVSVRA	RQIFDSRGNP	TVEADVKLSD	GYLARAAVPS	GASTGIYEAL
51	ELRDGGSDYL	GKGVSKAVEN	VNIIIGPALV	GKDPTDQVGI	DNFMVQQLDG
101	TVNEWGWCKQ	KLGANAILAV	SLAVCKAGAH	VKGIPLYEHI	ANLAGNKNLV
151	LPVPAFNVIN	GGSHAGNKLA	MQEFMILPVG	ASSFKEAMKM	GAEVYHHLKS
201	VIKKKYGQDA	TNVGDEGGFA	PNIQENKEGL	ELLKTAIAKA	GYTGKVVIGM
251	DVAASEFYGS	DQTYDLNFKE	ENNNGSQKIS	GEALKDLYKS	FVAEYPIVSI
301	EDPFDQDDWA	HYAKLTSEIG	EKVQIVGDDL	LVTNPKRVEK	AIKEKACNAL
351	LLKVNQIGSV	TESIEAVKMS	KRAGWGVMAS	HRSGETEDTF	IADLSVGLAT
401	GQIKTGAPCR	SERLAKYNQL	LRIEEELGSE	AVYAGANFRK	PVEPY

Spot No.: 140

NCBI accession No.: **gi|132270** Species: *Hevea brasiliensis*

PFF score: [52] Protein name： Rubber elongation factor protein

Matched peptides No.: [1] Sequence coverage %: [10]

Matched sequences: K.FVDSTVVASVTIIDR.S

Calculated Mr: **14713** Calculated *p*I: **5.04**

Probability Based Mowse Score:

Ions score is -10*Log(P), where P is the probability that the observed match is a random event.
Individual ions scores > 45 indicate identity or extensive homology (p<0.05).
Protein scores are derived from ions scores as a non-probabilistic basis for ranking protein hits.

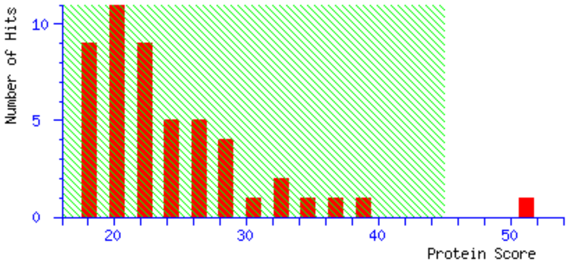

Matched peptide sequences: shown in Bold Red

```
  1 MAEDEDNQQG QGEGLKYLGF VQDAATYAVT TFSNVYLFAK DKSGPLQPGV
 51 DIIEGPVKNV AVPLYNRFSY IPNGALKFVD STVVASVTII DRSLPPIVKD
101 ASIQVVSAIR AAPEAARSLA SSLPGQTKIL AKVFYGEN
```

Spot No.: **141**

NCBI accession No.: **gi|14423688** Species: *Hevea brasiliensis*

Protein name: Enolase 1

PMF Mascot score: **165** Sequence coverage %: **52**

Matched peptides No.: **22** Total peptides No.: **64**

Calculated Mr: **48029** Calculated p*I*: **5.57**

Probability Based Mowse Score:

Protein score is -10*Log(P), where P is the probability that the observed match is a random event. Protein scores greater than 73 are significant (p<0.05).

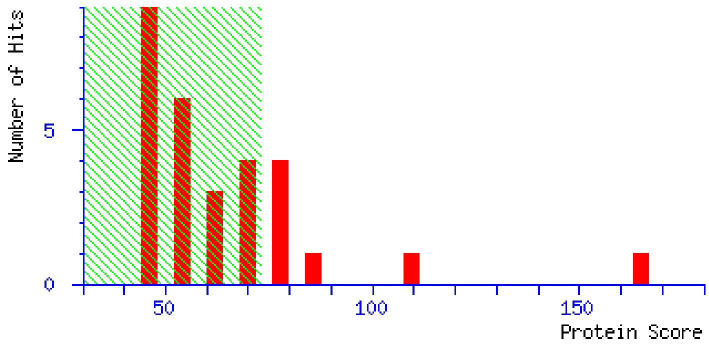

Matched peptide sequences: shown in Bold Red:

```
  1 MAITIVSVRA RQIFDSRGNP TVEADVKLSD GYLARAAVPS GASTGIYEAL
 51 ELRDGGSDYL GKGVSKAVEN VNIIIGPALV GKDPTDQVGI DNFMVQQLDG
101 TVNEWGWCKQ KLGANAILAV SLAVCKAGAH VKGIPLYEHI ANLAGNKNLV
151 LPVPAFNVIN GGSHAGNKLA MQEFMILPVG ASSFKEAMKM GAEVYHHLKS
201 VIKKKYGQDA TNVGDEGGFA PNIQENKEGL ELLKTAIAKA GYTGKVVIGM
251 DVAASEFYGS DQTYDLNFKE ENNNGSQKIS GEALKDLYKS FVAEYPIVSI
301 EDPFDQDDWA HYAKLTSEIG EKVQIVGDDL LVTNPKRVEK AIKEKACNAL
351 LLKVNQIGSV TESIEAVKMS KRAGWGVMAS HRSGETEDTF IADLSVGLAT
401 GQIKTGAPCR SERLAKYNQL LRIEEELGSE AVYAGANFRK PVEPY
```

Spot No.: **142**

NCBI accession No.: **gi|14423687** Species: *Hevea brasiliensis*

PFF score: [766] Protein name: Enolase 2

Matched peptides No.: [10] Sequence coverage %: [32]

Matched sequences: K.LAMQEFMILPVGASSFK.E + Oxidation (M);
K.MGAEVYHHLK.S;
K.MGAEVYHHLK.S + Oxidation (M);
K.YGQDATNVGDEGGFAPNIQENK.E;
K.VVIGMDVAASEFYGSDK.T;
K.VQIVGDDLLVTNPK.R;
K.VNQIGSVTESIEAVK.M;
R.AGWGVMASHR.S + Oxidation (M);
R.SGETEDTFIADLSVGLATGQIK.T;
R.IEEELGAEAVYAGANFR.T

Calculated Mr: **48112** Calculated *p*I: **5.92**

Probability Based Mowse Score:

Ions score is -10*Log(P), where P is the probability that the observed match is a random event.
Individual ions scores > 45 indicate identity or extensive homology (p<0.05).
Protein scores are derived from ions scores as a non-probabilistic basis for ranking protein hits.

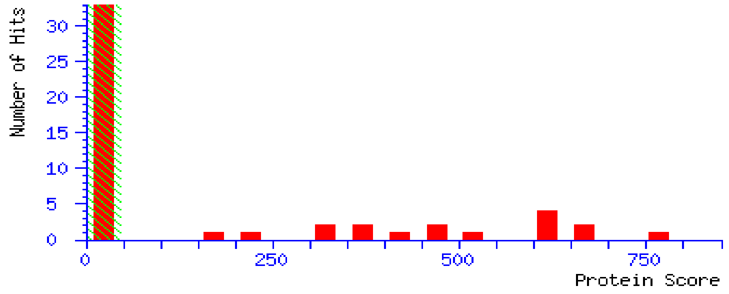

Matched peptide sequences: shown in Bold Red:

1	MAITIVSVRA	RQIFDSRGNP	TVEADVKLSD	GYLARAAVPR	GASTGIYEAL
51	ELRDGGSDYL	GKGVSKAVEN	VNIIIGPALV	GKDPTDQVGI	DNFMVQQLDG
101	TVNEWGWCKQ	KLGANAILAV	SLAVCKAGAH	VKGIPLYKHV	ANLAGNKNLV
151	LPVPAFNVIN	GGSHAGNK**LA**	**MQEFMILPVG**	**ASSFK**EAMK**M**	**GAEVYHHLK**S
201	VIKKK**YGQDA**	**TNVGDEGGFA**	**PNIQENK**EGL	ELLKTAIAKA	GYTGK**VVIGM**
251	**DVAASEFYGS**	**DK**TYDLNFKE	ENNNGSQKIS	GDVLKDLYKS	FVTEYPIVSI
301	EDPFDQDDWE	HYAKLTSEIG	VK**VQIVGDDL**	**LVTNPK**RVEK	AIKEKACNAL
351	LLK**VNQIGSV**	**TESIEAVK**MS	KR**AGWGVMAS**	**HRSGETEDTF**	**IADLSVGLAT**
401	**GQIK**TGAPCR	SERLAKYNQL	LR**IEEELGAE**	**AVYAGANFR**T	PVEPY

Spot No.: **143**

NCBI accession No.: **gi|333690873**   Species: *Hevea brasiliensis*

PFF score: [52]

Protein name: glutathione-S-transferase tau 1

Matched peptides No.: [2]   Sequence coverage %: [10]

Matched sequences: K.YEYREEDLR.N;

K.DKSPFLPSDPYQR.A

Calculated Mr: 25595   Calculated *p*I: **5.48**

Probability Based Mowse Score:

Ions score is -10*Log(P), where P is the probability that the observed match is a random event.
Individual ions scores > 45 indicate identity or extensive homology (p<0.05).
Protein scores are derived from ions scores as a non-probabilistic basis for ranking protein hits.

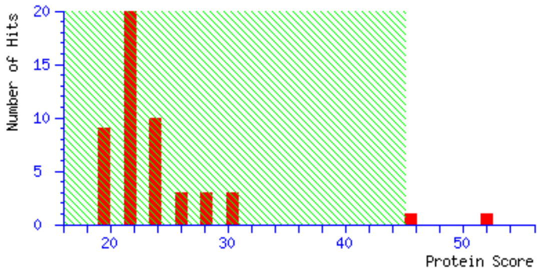

Matched peptide sequences: shown in Bold Red:

```
  1 MAEEVILLDF WPSPFGMRVR IALAEKGVKY EYREEDLRNK SPLLLQMNPV
 51 HKKIPVLIHN GKPICESLIA VQYVDEVWKD KSPFLPSDPY QRAQARFWAD
101 FIDKKIYDIG RKIWTTKGDE QEAAKKEFIE ALKLLEGELG NKPYFGGESM
151 GYVDVALIPF YSWFYAYETC GNFSIEPECP VLIAWAKRCL QKESVSESVP
201 DPEKVYEFVL VLKKKFGIE
```

Spot No.: **144**

NCBI accession No.: **gi|242080619** Species: *Sorghum bicolor*

Protein name: hypothetical protein SORBIDRAFT_07g003800

PMF Mascot score: **82** Sequence coverage %: **34**

Matched peptides No.: **13** Total peptides No.: **69**

Calculated Mr: **43483** Calculated *p*I: **8.78**

Probability Based Mowse Score:

Protein score is -10*Log(P), where P is the probability that the observed match is a random event. Protein scores greater than 73 are significant (p<0.05).

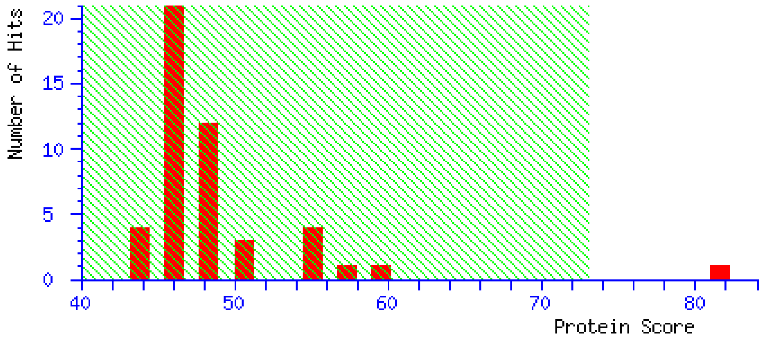

Matched peptide sequences: shown in Bold Red:

```
  1 MAEANVAAAS LFGADSRLCS ADILAPPEAK VRARIEVAVL NFLAALASPS
 51 SPAISVLPLI SRSSANCSLR SGLLNDVSSV YLSHTFCKRS LMHNPKAFVR
101 VWKVMEMCYK ILGEGKLVHQ RELFYKLLSD SPKYFSCQRH VNQAIQDVVS
151 LLRCTRQSLG VMASSRGALI GRLVLHEPDE EQIDCSILGA SGHAITGDLN
201 LLSKLNLSSD ARYIIVVEKD AIFQRLAEDR LYNQLPCILI TAKGYPDIAT
251 RFILHRLSQT FPNMPIFALV DWNPAGLAIM CTYKYGSISM GLESYRYACN
301 VKWLGMRGDD LQLIPESAFQ VLKPRDLQTC TNYLRPQFHQ ESHRAELTLM
351 VEKGKRADIE ALYSHGFDYL GKYIARKIVQ GDYI
```

Spot No.: **145**

NCBI accession No.: **gi|30575570** Species: *Citrus x paradisi*

PFF score: [87]

Protein name: HSP19 class II

Matched peptides No.: [1] Sequence coverage %: [15]

Matched sequences: K.VQVEDDNVLLISGER.K

Calculated Mr: **11191** Calculated *p*I: **8.01**

Probability Based Mowse Score:

Ions score is -10*Log(P), where P is the probability that the observed match is a random event.
Individual ions scores > 45 indicate identity or extensive homology ($p<0.05$).
Protein scores are derived from ions scores as a non-probabilistic basis for ranking protein hits.

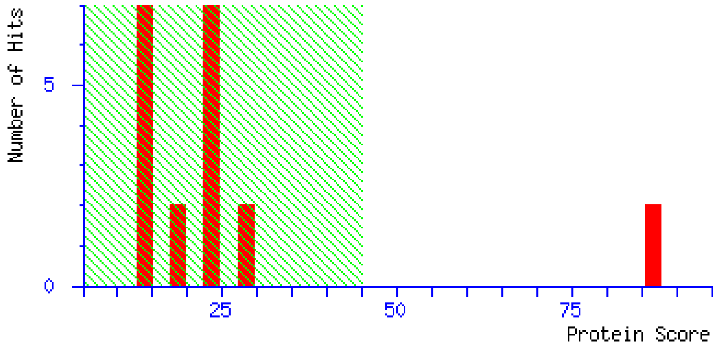

Matched peptide sequences: shown in Bold Red:

```
 1 YVFIVDMPGL KSGDIKVQVE DDNVLLISGE RKREEEKDGA KYVRMERRVG
51 KFMRKFVLPE NANVEAISAV CQDGVLTVTV DKLPPPEPKK PKTIEVKIA
```

Spot No.: **146**

NCBI accession No.: **gi|37901124** Species: *Hevea brasiliensis*

PFF score: [88]

Protein name: latex abundant protein 1

Matched peptides No.: [1] Sequence coverage %: [11]

Matched sequences: R.NKDDINCFNEYNPR.P

Calculated Mr: **14618** Calculated *p*I: **5.01**

Probability Based Mowse Score:

Ions score is -10*Log(P), where P is the probability that the observed match is a random event.
Individual ions scores > 43 indicate identity or extensive homology (p<0.05).
Protein scores are derived from ions scores as a non-probabilistic basis for ranking protein hits.

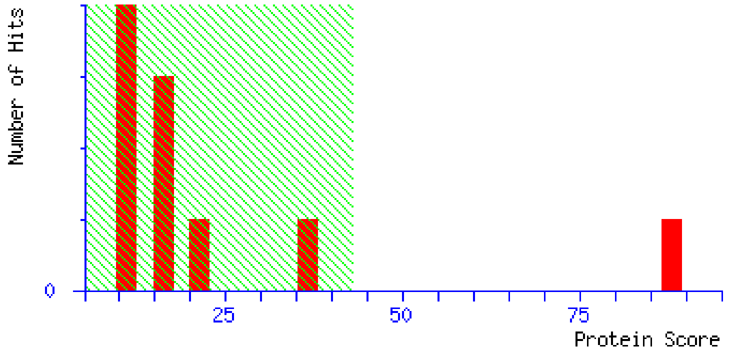

Matched peptide sequences: shown in Bold Red:

```
  1 MPYYTRNKDD INCFNEYNPR PYIGGYDMAL TYGRPISACE ETCYPRSSSA
 51 NEIDYDYPDF TWYVEPSAYN DDHLQEEYTS YARPKPRPAP NSLGSCPILG
101 GNDEEKKHHH CHHQHEKHCD DE
```

Spot No.: **147**

NCBI accession No.: **gi|359806300** Species: *Glycine max*

PFF score: [173]

Protein name: uncharacterized protein LOC100784610

Matched peptides No.: [3] Sequence coverage %: [9]

Matched sequences: K.NTALHYAAGYGR.K;
R.YWNNEEVLQK.L;
R.TALHFACGYGEVK.C

Calculated Mr: **38027** Calculated *p*I: **4.53**

Probability Based Mowse Score:

Ions score is -10*Log(P), where P is the probability that the observed match is a random event.
Individual ions scores > 45 indicate identity or extensive homology (p<0.05).
Protein scores are derived from ions scores as a non-probabilistic basis for ranking protein hits.

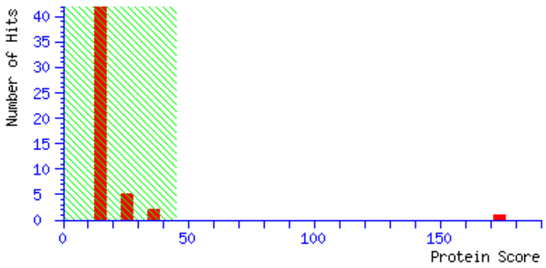

Matched peptide sequences: shown in Bold Red:

```
  1 MASDSKKDFP ADDKAGTEES KTSKDETSSK DSPAEQRATA TFGPRPGPAG
 51 HPGNPFDLSA MSGLLNDPSI KELAEQIAKD PSFNQMAEQL QKTFQGAPQD
101 AIPSFDNQQY FSTMQQVMQN PNFMTMAERL GNALMQDPSM SAMLESFSNP
151 SNKDQLEERM ARIKEDPSLK HILEEIETGG PAAMMRYWNN EEVLQKLGQA
201 MGLANSGDAG ASAENSGADE TEDLGNEDES IVHHTASVGD VEGLKNALAS
251 GADKDEEDSE GRTALHFACG YGEVKCAQVL LEAGAKVDAL DKNKNTALHY
301 AAGYGRKECV ALLENGAAV TLQNMDGKTP IDVAKLNNQN EVLKLLEKDA
351 FL
```

Spot No.: **148**

NCBI accession No.: **gi|37625031** Species: *Vitis aestivalis*

PFF score: [166]

Protein name: putative ankyrin-repeat protein

Matched peptides No.: [3] Sequence coverage %: [9]

Matched sequences: K.LNNQHEVLK.L;

K.NTALHYAAGYGR.K;

R.TALHFACGYGEVK.C

Calculated Mr: **38089** Calculated *p*I: **4.53**

Probability Based Mowse Score:

Ions score is -10*Log(P), where P is the probability that the observed match is a random event.
Individual ions scores > 45 indicate identity or extensive homology (p<0.05).
Protein scores are derived from ions scores as a non-probabilistic basis for ranking protein hits.

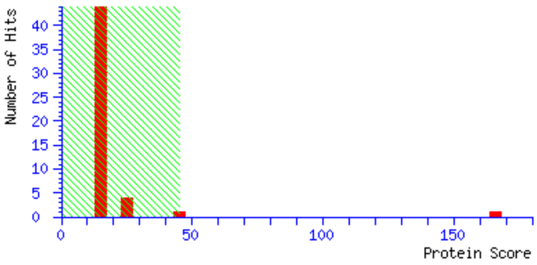

Matched peptide sequences: shown in Bold Red:

1 MASNSDKDTP AGSAENSKSD SSSGESHSEQ RRAAAAASVP GAGLPTNPFD
51 FSAMTGLLND PSIKELAEQI AKDPAFNQMA EQLQKTFHGA AVEESIPQFD
101 TQQYYSTMQQ VMQNPQFMTM AERLGNALMQ DPSMSSMLEN LANPTHKDQL
151 EERMARIKED PSLKPILDEI ETGGPAAMMR YWNDKDVLQK LGEAMGLAVS
201 GDAAASADNS GLDEAEELAN EDESIAHHHS ESIVHDTASV GDVEGLKNAL
251 ASGADKDEED SEGR**TALHFA** **CGYGEVK**CAQ ILVEAGATVD ALDKNK**NTAL**
301 **HYAAGYGR**KE CVALLLENGA AVTLQNMDGK TPIDVAK**LNN** **QHEVLK**LLEK
351 DAFL

Spot No.: **149**

NCBI accession No.: **gi|13310811** Species: *Nicotiana tabacum*

PFF score: [71]

Protein name: putative ankyrin-repeat protein

Matched peptides No.: [2] Sequence coverage %: [7]

Matched sequences: K.NTALHYAAGYGR.K;
R.TALHFACGYGEVK.C

Calculated Mr: **37522** Calculated *p*I: **4.45**

Probability Based Mowse Score:

Ions score is -10*Log(P), where P is the probability that the observed match is a random event.
Individual ions scores > 45 indicate identity or extensive homology (p<0.05).
Protein scores are derived from ions scores as a non-probabilistic basis for ranking protein hits.

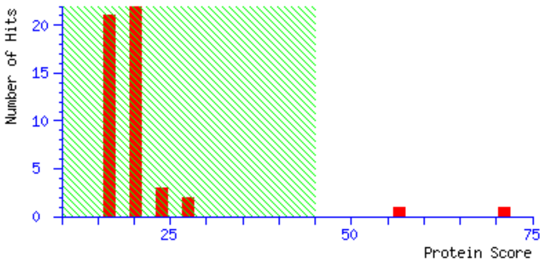

Matched peptide sequences: shown in Bold Red:

```
  1 MSEGEKVLPT ASADEKSGAS ENKKSSESSS TEAPSGEART TSTAAAGAGL
 51 QNPFDFSAMS GLLNDPSIKE LAEQIAKDPA FNQMAEQLQK TFQGAAVEES
101 VPNFDSQQYY STMQQVMQNP QFMTMAERLG NALMQDPSMS GMLESLSNPA
151 QKEQIEERMA RIKEDPSLKP ILEEIESGGP AAMMRYWNDQ ETLKKIGEAM
201 GFAAGGEGAT SSAIPGTDET EEANEDESVV HQCASVGDAE GLKAALTAGA
251 DKDEEDSEGR TALHFACGYG EVKCAQILLE AGAKVDALDK NKNTALHYAA
301 GYGRKECVAL LLENGAAVTL QNLDGKTPID VAKLNNQQEV LKLLEKDVFL
```

Spot No.: **150**

NCBI accession No.: gi|129916   Species: *Triticum aestivum*

PFF score: [180]

Protein name: Phosphoglycerate kinase, cytosolic

Matched peptides No.: [3]   Sequence coverage %: [7]

Matched sequences: K.KPFAAIVGGSK.V;

K.LASVADLYVNDAFGTAHR.A;
K.KLASVADLYVNDAFGTAHR.A

Calculated Mr: **42153**   Calculated *p*I: **5.64**

Probability Based Mowse Score:

Ions score is -10*Log(P), where P is the probability that the observed match is a random event.
Individual ions scores > 45 indicate identity or extensive homology (p<0.05).
Protein scores are derived from ions scores as a non-probabilistic basis for ranking protein hits.

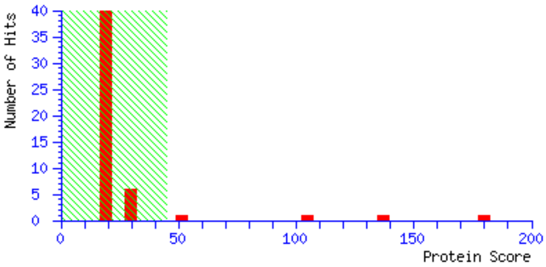

Matched peptide sequences: shown in Bold Red:

```
  1 MATKRSVGTL GEADLKGKKV FVRADLNVPL DDAQKITDDT RIRASIPTIK
 51 YLLEKGAKVI LASHLGRPKG VTPKFSLKPL VARLSELLGL EVVMAPDCIG
101 EEVEKLAAAL PDGGVLLLEN VRFYKEEEKN DPEFAKKLAS VADLYVNDAF
151 GTAHRAHAST EGVTKFLRPS VAGFLMQKEL DYLVGAVANP KKPFAAIVGG
201 SKVSSKIGVI ESLLAKVDIL ILGGGMIFTF YKAQGLAVGK SLVEEDKLEL
251 ATSLIETAKS KGVKLLLPTD VVVADKFAAD AESKIVPATA IPDGWMGLDV
301 GPDSIKTFAE ALDTTKTVIW NGPMGVFEFE KFAAGTDAIA KQLAELTGKG
351 VTTIIGGGDS VAAVEKAGLA DKMSHISTGG GASLELLEGK PLPGVLALDE
401 A
```

Spot No.: **151**

NCBI accession No.: **gi|378464888**

Species: *Ammopiptanthus mongolicus*

PFF score: [117]

Protein name: translation elongation factor

Matched peptides No.: [2]   Sequence coverage %: [11]

Matched sequences: R.WFNHIDALLR.I;

K.SSVLLDVKPWDDETDMK.K

Calculated Mr: **25300**   Calculated *p*I: **4.50**

Probability Based Mowse Score:

Ions score is -10*Log(P), where P is the probability that the observed match is a random event.
Individual ions scores > 45 indicate identity or extensive homology (p<0.05).
Protein scores are derived from ions scores as a non-probabilistic basis for ranking protein hits.

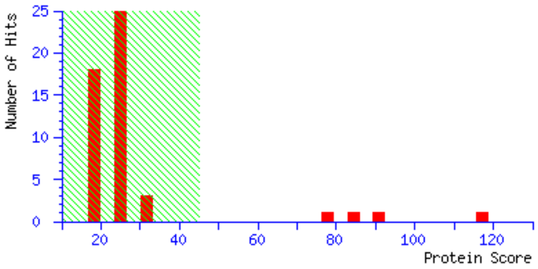

Matched peptide sequences: shown in Bold Red:

```
  1 MVLTLYNLSS EPGLKKLDEY LLSRSYITGY QASKDDLTVY AALPEVPSDK
 51 YVNVSRWFNH IDALLRISGV SGEGSGVTVE GSAPVAEETI ATPPAADTKA
101 TAAEDDDDDV DLFGEETEEE KKAAEERAAS VKASVKKKES GKSSVLLDVK
151 PWDDETDMKK LKEAVRSVSM EGLHWGASKL VPVGYGIKKL QIMLTIVDDL
201 VSVDNLIEEQ LQVEPINEYV QSCDIVAFNK I
```

Spot No.: **152**

NCBI accession No.: **gi|225456295** Species: *Vitis vinifera*

PFF score: [107]

Protein name: elongation factor 1-delta-like

Matched peptides No.: [4] Sequence coverage %: [17]

Matched sequences: K.APSSEYVNVSR.W;

R.SIKMEGLLWGASK.L;

R.SIKMEGLLWGASK.L + Oxidation (M);

K.SSVLLDVKPWDDETDMK.K

Calculated Mr: **25060** Calculated *p*I: **4.50**

Probability Based Mowse Score:

Ions score is -10*Log(P), where P is the probability that the observed match is a random event.
Individual ions scores > 45 indicate identity or extensive homology (p<0.05).
Protein scores are derived from ions scores as a non-probabilistic basis for ranking protein hits.

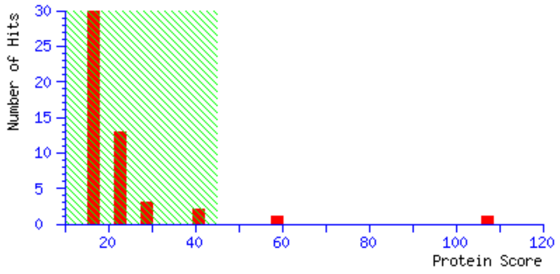

Matched peptide sequences: shown in Bold Red:

```
  1 MAVTFYGVNS GSGLKKLDEY LLTRSYISGY QASKDDITVH AALSKAPSSE
 51 YVNVSRWYNH IESLLRISGV SGEGCGVTIE GFAPVDAVAT PPAEDTKATA
101 AEDDDDDDVD LFGEETEEEK KAAEARAAAV KASGKKKESG KSSVLLDVKP
151 WDDETDMKKL EEAVRSIKME GLLWGASKLV PVGYGIKKLQ IMLTIVDDLV
201 SVDDLIEEQL TVEPTNEYVQ SCDIVAFNKI
```

Spot No.: **153**

NCBI accession No.: **gi|22261807** Species: *Solanum tuberosum*

PFF score: [132]

Protein name: Light-induced protein, chloroplastic

Matched peptides No.: [1] Sequence coverage %: [8]

Matched sequences: K.FEEGIIGTPQLTDSIVLPENVEFLGQK.I

Calculated Mr: **35671** Calculated *p*I: **5.26**

Probability Based Mowse Score:

Ions score is -10*Log(P), where P is the probability that the observed match is a random event.
Individual ions scores > 45 indicate identity or extensive homology (p<0.05).
Protein scores are derived from ions scores as a non-probabilistic basis for ranking protein hits.

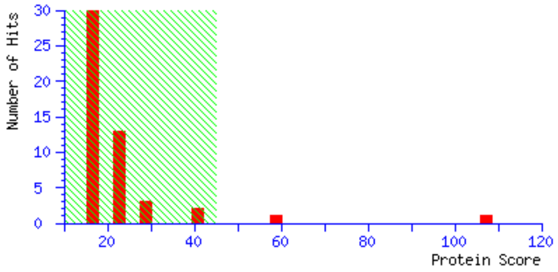

Matched peptide sequences: shown in Bold Red:

```
  1  MAVTFYGVNS  GSGLKKLDEY  LLTRSYISGY  QASKDDITVH  AALSKAPSSE
 51  YVNVSRWYNH  IESLLRISGV  SGEGCGVTIE  GFAPVDAVAT  PPAEDTKATA
101  AEDDDDDDVD  LFGEETEEEK  KAAEARAAAV  KASGKKKESG  KSSVLLDVKP
151  WDDETDMKKL  EEAVRSIKME  GLLWGASKLV  PVGYGIKKLQ  IMLTIVDDLV
201  SVDDLIEEQL  TVEPTNEYVQ  SCDIVAFNKI
```

Spot No.: **154**

NCBI accession No.: **gi|345103803** Species: *Gossypium thurberi*

Protein name: 14-3-3-like protein, partial

PMF Mascot score: **83** Sequence coverage %: **37**

Matched peptides No.: **11** Total peptides No.: **41**

Calculated Mr: **28861** Calculated *p*I: **4.82**

Probability Based Mowse Score:

Protein score is -10*Log(P), where P is the probability that the observed match is a random event.
Protein scores greater than 73 are significant (p<0.05).

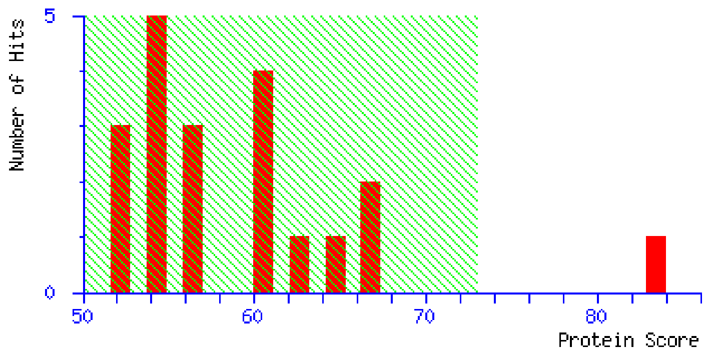

Matched peptide sequences: shown in Bold Red:

```
  1 MEKEREQQVY LARLAEQAER YDEMVEAMKS VAKLDVELTV EERNLLSVGY
 51 KNVIGARRAS WRILSSIEQK EEAKGNEQNV KRIKDYRQRV EDELSKICND
101 ILSVIDKHLI PSSSTGESTV FYYKMKGDYF RYLAEFKAGD DRKEAADQSL
151 KAYEAATSTA SSDLPPTHPI RLGLALNFSV FYYEILNSPE RACHLAKQAF
201 DEAIAELDSL NEESYKDSTL IMQLLRDNLT LWTSDLPEEG GEQSKADEPQ
251 AE
```

Spot No.: **155**

NCBI accession No.: **gi|326694865** Species: *Hevea brasiliensis*

PFF score: [384]

Protein name: 14-3-3 protein 2

Matched peptides No.: [9] Sequence coverage %: [32]

Matched sequences: R.GNEDHVTIIK.E;
K.DSTLIMQLLR.D;
K.DSTLIMQLLR.D + Oxidation (M);
R.YEEMVEFMEK.V;
R.YEEMVEFMEK.V + Oxidation (M),K.TVDVEELTVEER.N;
K.SAQDIALADLAPTHPIR.L;
K.LAEQAERYEEMVEFMEK.V;
R.LGLALNFSVFYYEILNSPDR.A

Calculated Mr: **35671** Calculated *p*I: **5.26**

Probability Based Mowse Score:

Ions score is -10*Log(P), where P is the probability that the observed match is a random event.
Individual ions scores > 45 indicate identity or extensive homology (p<0.05).
Protein scores are derived from ions scores as a non-probabilistic basis for ranking protein hits.

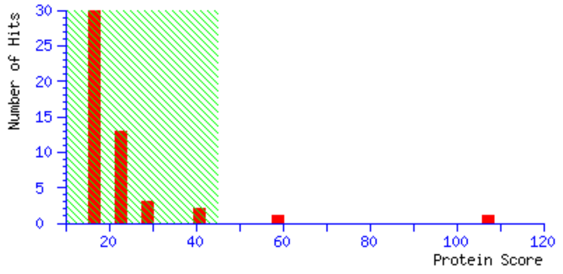

Matched peptide sequences: shown in Bold Red:

```
  1 MAVTFYGVNS GSGLKKLDEY LLTRSYISGY QASKDDITVH AALSKAPSSE
 51 YVNVSRWYNH IESLLRISGV SGEGCGVTIE GFAPVDAVAT PPAEDTKATA
101 AEDDDDDDVD LFGEETEEEK KAAEARAAAV KASGKKKESG KSSVLLDVKP
151 WDDETDMKKL EEAVRSIKME GLLWGASKLV PVGYGIKKLQ IMLTIVDDLV
201 SVDDLIEEQL TVEPTNEYVQ SCDIVAFNKI
```

Spot No.: **156**

NCBI accession No.: **gi|225458231**   Species: *Vitis vinifera*

PFF score: [53]

Protein name: PREDICTED: proteasome subunit alpha type-1-B

Matched peptides No.: [3]   Sequence coverage %: [17]

Matched sequences: K.VDDHIGVAIAGLTADGR.V;

R.NQYDTDVTTWSPAGR.L;

R.SECINYSFTYESPLPVGR.L

Calculated Mr: **30912**   Calculated *p*I: **5.02**

Probability Based Mowse Score:

Ions score is -10*Log(P), where P is the probability that the observed match is a random event.
Individual ions scores > 45 indicate identity or extensive homology (p<0.05).
Protein scores are derived from ions scores as a non-probabilistic basis for ranking protein hits.

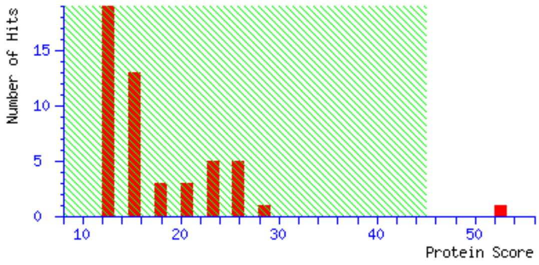

Matched peptide sequences: shown in **Bold Red:**

```
  1 MFRNQYDTDV TTWSPAGRLF QVEYAMEAVK QGSAAIGLRS KTHVVLACVN
 51 KANSELSSHQ KKIFKVDDHI GVAIAGLTAD GRVLSRYMRS ECINYSFTYE
101 SPLPVGRLVV QLADKAQVCT QRSWKRPYGV GLLVAGLDES GAHLYYNCPS
151 GNYFEYQAFA IGSRSQAAKT YLERRFGTFQ NSSREDLIKD ALFAIRETLQ
201 GEKLKSSICT VTVLGVGEPF HILDQETVQG LINAFEMAGE EAPAADQGAA
251 GEEGAAGEVV AAAEEGAAPE EGAATEEGAA PMDI
```

Spot No.: **157**

NCBI accession No.: **gi|211906514** Species: *Gossypium hirsutum*

PFF score: [80]

Protein name: lactoylglutathione lyase

Matched peptides No.: [3]   Sequence coverage %: [6]

Matched sequences: K.FYTECFGMK.L;
K.FYTECFGMK.L + Oxidation (M);
K.ITSFLDPDGWK.T

Calculated Mr: **32609**   Calculated *p*I: **5.69**

Probability Based Mowse Score:

Ions score is -10*Log(P), where P is the probability that the observed match is a random event.
Individual ions scores > 45 indicate identity or extensive homology (p<0.05).
Protein scores are derived from ions scores as a non-probabilistic basis for ranking protein hits.

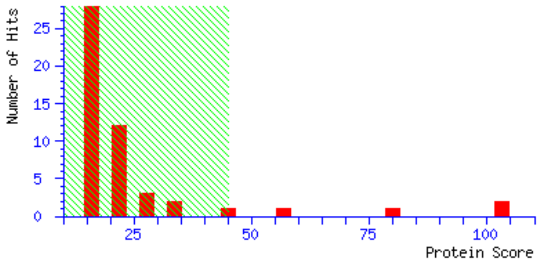

Matched peptide sequences: shown in Bold Red:

```
  1 MAEGSAAVPS TELLEWPKKD KRRFLHAVYR VGDLDRTIKF YTECFGMKLL
 51 RKRDIPEEKY SNAFLGFGPE ESHFVVELTY NYGVTSYDIG TGFGHFAIAT
101 PDVYKMVEDI RAKGGNITRE PGPVKGGSSV IAFVKDPDGY IFELIQRAST
151 PEPLCQVMLR VGDLDRSVKF YEKALGMKLV KKVDRPEYKY SIAMMGYAEE
201 HETTVLELTY NYGVTEYTKG NAYAQVAIST DDVYKSGEVV EHVIQELGGK
251 VTRKPGPIPG INTKITSFLD PDGWKTVLVD NEDFLKELK
```

Spot No.: **159**

NCBI accession No.: **gi|340033945** Species: *Crithmum maritimum*

Protein name: ATP synthase CF1 beta subunit

PMF Mascot score: **83** Sequence coverage %: **29**

Matched peptides No.: **15** Total peptides No.: **69**

Calculated Mr: **53692** Calculated *p*I: **5.08**

Probability Based Mowse Score:

Protein score is -10*Log(P), where P is the probability that the observed match is a random event. Protein scores greater than 73 are significant ($p<0.05$).

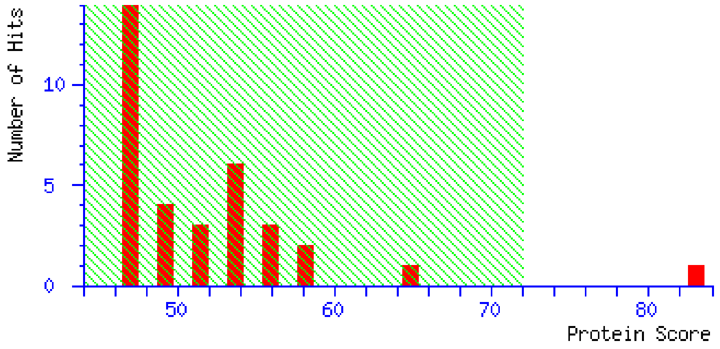

Matched peptide sequences: shown in Bold Red:

```
  1 MRINPTTSSS VASTLEKKNL GNITQIIGPV LDVAFPPGKM PNIYNALVVK
 51 GRDTAGQQIN VTCEVQQLLG NNRVRAVAMS ATDGLTRGME VIDTGAPLSV
101 PVGGTTLGRI FNVLGEPVDN LGPVDTRTTS PIHRSAPAFI QLDTKLSIFE
151 TGIKVVDLLA PYRRGGKIGL FGGAGVGKTV LIMELINNIA KAHGGVSVFG
201 GVGERTREGN DLYMEMKESG VINEENIAES KVALVYGQMN EPPGARMRVG
251 LTALTMAEYF RDVNEQDVLL FIDNIFRFVQ AGSEVSALLG RMPSAVGYQP
301 TLSTEMGTLQ ERITSTKEGS ITSIQAVYVP ADDLTDPAPA TTFAHLDATT
351 VLSRGLAAKG IYPAVDPLDS TSTMLQPRIV GEDHYETAQR VKQTLQRYKE
401 LQDIIAILGL DELSEEDRLT VARARKIERF LSQPFFVAEV FTGSPGKYVG
451 LAETIIGFRL ILSGELDSLP EQAFYLVGNI DEATAKAMNL EMESNSNK
```

Spot No.: **160**

NCBI accession No.: gi|4388534 Species: *Sorghum bicolor*

Protein name: F1-ATP synthase, beta subunit

PMF Mascot score: **84** Sequence coverage %: **19**

Matched peptides No.: **8** Total peptides No.: **20**

Calculated Mr: **49239** Calculated *p*I: **5.41**

Probability Based Mowse Score:

Protein score is -10*Log(P), where P is the probability that the observed match is a random event.
Protein scores greater than 73 are significant (p<0.05).

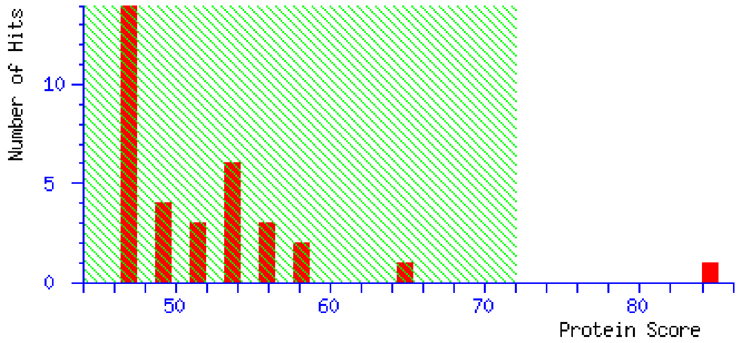

Matched peptide sequences: shown in Bold Red:

1	IGQVCQVIGA	VVDVR**FDEGL**	**PPILTALEVL**	**DNNIR**LVLEV	AQHLGENMVR
51	TIAMDGTEGL	VRGQRVLNTG	SPITVPVGRA	TLGRIINVIG	EPIDERGDIT
101	TNHFLPIHRE	APAFVEQATE	QQILVTGIKV	VDLLAPYQRG	GKIGLFGGAG
151	VGKTVLIMEL	INNVAK**AHGG**	**FSVFAGVGER**	**TREGNDLYRE**	**MIESGVIKLG**
201	**DK**QSESK**CAL**	**VYGQMNEPPG**	**AR**ARVGLTGL	TVAEHFRDAE	GQDVLLFIDN
251	IFRFTQANSE	VSALLGRIPS	AVGYQPTLAT	DLGGLQERIT	TTKKGSITSV
301	QAIYVPADDL	TDPAPATTYA	HLDATTVLSR	QISER**GIYPA**	**VDPLDSTSRM**
351	LSPHVLGEDH	YNTARGVQKV	LQKYKNLQDI	IAILGMDELS	EDDKLTVARA
401	RKIQRFLSQP	FHVAEVFTGA	PGKYVELKES	VK**SFQGVLDG**	**K**YDDLPAQSF
451	YMVG				

Spot No.: **161**

NCBI accession No.: **gi|147805616** Species: *Vitis vinifera*

PFF score: [136]

Protein name: hypothetical protein VITISV_028800

Matched peptides No.: [3] Sequence coverage %: [10]

Matched sequences: R.GPYPADQVVR.D;

R.DRGPYPADQVVR.D;
K.TANEVIIVIEAYR.T

Calculated Mr: 27385 Calculated *p*I: **5.64**

Probability Based Mowse Score:

Ions score is -10*Log(P), where P is the probability that the observed match is a random event.
Individual ions scores > 45 indicate identity or extensive homology (p<0.05).
Protein scores are derived from ions scores as a non-probabilistic basis for ranking protein hits.

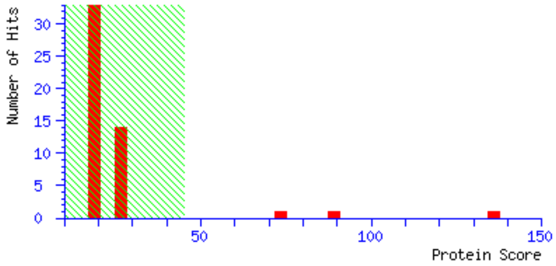

Matched peptide sequences: shown in Bold Red:

```
  1 MLAVFHKSIA EAPEGLRTPD SASLPALKDG FLPQFFASLH PSAVTVNLGS
 51 SGAISYSVDK QNPLLPRLFA VVDDIFCLFQ GHIENVALLK QQYGLNKTAN
101 EVIIVIEAYR TLRDRGPYPA DQVVRDLHGK FAFVLYDSSN RTAFLAADAD
151 ESVPFFWGVD SEGHLVLSDD EETVKKGCGK SFAPFPKGCF FTTSGGLRSF
201 EHPLNELRAE PRVDSSGQVC GANFKVDVEA KKETGMPRVG SAANWSTHY
```

Spot No.: **162**

NCBI accession No.: **gi|326492265**

Species: *Hordeum vulgare subsp. vulgare*

Protein name: **predicted protein**

PMF score: **83** Sequence coverage %: **10**

Matched peptides No.: **58** Total peptides No.: **32**

Calculated Mr: **54666** Calculated *p*I: **8.89**

Probability Based Mowse Score:

Protein score is -10*Log(P), where P is the probability that the observed match is a random event
Protein scores greater than 73 are significant (p<0.05).

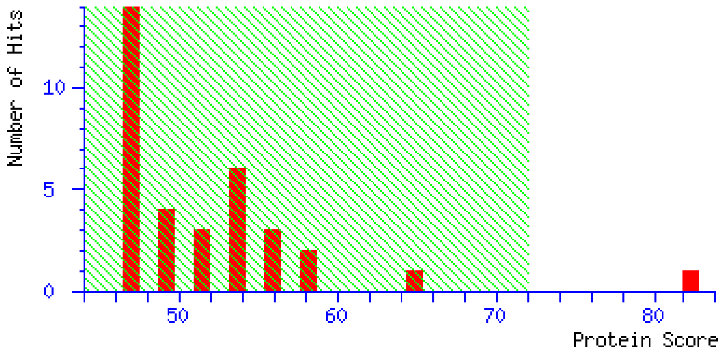

Matched peptide sequences: shown in Bold Red:

1 MSPYAVLVAV LLAFFYAVWK ARGSSSSSSS RLPPSPPSRP LLGHLHLLGQ
51 LPHRSLRELH ARYGTDGGLL LLQLGRRRTL VVSTAAAAAD LYKNHDLAFA
101 SRVPSVPVDK LTYGSINISF SPYGDVWRRS KKIAVVHLLS PRRVDSFAPV
151 RAVEVAALVA GARRGAEAGE SVELRELLCA YSNAVVTRAT TGAAGATAEK
201 LNKLLGNSAA LMSGLQADDV LPGAAAKVVR WATGLEKRLD AELELWDKFL
251 SQIIAEHLEK KKCDGSAGEE DFLDVLLRLR EEGGAGLELT DDRIKSIIKD
301 IIAAGTDTSS VTLEWAMAEL AGNPRAMAKL QDEITRVTQG KPTIQEADLS
351 RMEYLKAVLK EVLRLHPAAP LLVPHQSTTT AVVQGYEIPA KTALFVNAWA
401 IGRDPAAWGA TAEEFRPERF LGSGGAEGVD LRGNDYQLLP FGAGRRLCPG
451 IGFAMPVLEI ALSSLVRHFD WELPAGARLD MSEAPGLTTP LLAPLRLVPK
501 CRRQQ

Spot No.: **163**

NCBI accession No.: **gi|225428086** Species: *Vitis vinifera*

PFF score: [343]

Protein name： V-type proton ATPase subunit B 1

Matched peptides No.: [10] Sequence coverage %: [30]

Matched sequences: K.YQEIVNIR.L;

K.FVAQGAYDTR.N;
R.RGQVLEVDGEK.A;
K.YTTVQFTGEVLK.T;
R.QIYPPINVLPSLSR.L;
R.VTLFLNLANDPTIER.I;
R.GYPGYMYTDLATIYER.A;
R.RDHADVSNQLYANYAIGK.D;
K.IPLFSAAGLPHNEIAAQICR.Q;
K.AVVGEEALSSEDLLYLEFLDKFER.K

Calculated Mr: **54367** Calculated *p*I: **5.04**

Probability Based Mowse Score:

Mascot Score Histogram

Ions score is -10*Log(P), where P is the probability that the observed match is a random event.
Individual ions scores > 45 indicate identity or extensive homology (p<0.05).
Protein scores are derived from ions scores as a non-probabilistic basis for ranking protein hits.

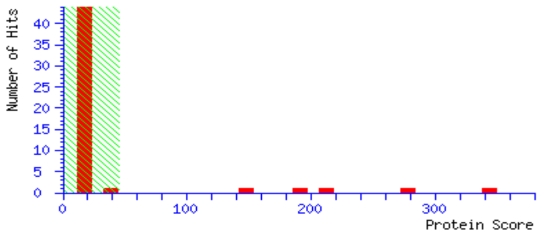

Matched peptide sequences: shown in Bold Red:

```
  1 MGVEQNNHDA EEGTLLVGME YRTVSGVAGP LVILEKVKGP KYQEIVNIRL
 51 GDGTTRRGQV LEVDGEKAVV QVFEGTSGID NKYTTVQFTG EVLKTPVSLD
101 MLGRIFNGSG KPIDNGPPIL PEAYLDISGS SINPSERTYP EEMIQTGIST
151 IDVMNSIARG QKIPLFSAAG LPHNEIAAQI CRQAGLVKRL EKSGSLLEDV
201 EEDNFAIVFA AMGVNMETAQ FFKRDFEENG SMERVTLFLN LANDPTIERI
251 ITPRIALTTA EYLAYECGKH VLVILTDMSS YADALREVSA AREEVPGRRG
301 YPGYMYTDLA TIYERAGRIE GRKGSITQIP ILTMPNDDIT HPTPDLTGYI
351 TEGQIYIDRQ LHNRQIYPPI NVLPSLSRLM KSAIGEGMTR RDHADVSNQL
401 YANYAIGKDV QAMKAVVGEE ALSSEDLLYL EFLDKFERKF VAQGAYDTRN
451 IFQSLDLAWT LLRIFPRELL HRIPAKTLDQ YYSREASN
```

Spot No.: **164**

NCBI accession No.: **gi|225428086** Species: *Vitis vinifera*

PFF score: [377]

Protein name：V-type proton ATPase subunit B 1

Matched peptides No.: [10] Sequence coverage %: [23]

Matched sequences: K.YQEIVNIR.L;

K.FVAQGAYDTR.N;
R.DFEENGSMER.V;
R.RGQVLEVDGEK.A;
R.KFVAQGAYDTR.N;
K.YTTVQFTGEVLK.T;
R.QIYPPINVLPSLSR.L;
R.VTLFLNLANDPTIER.I;
R.GYPGYMYTDLATIYER.A;
R.RDHADVSNQLYANYAIGK.D

Calculated Mr: **54367** Calculated *p*I: **5.04**

Probability Based Mowse Score:

Mascot Score Histogram

Ions score is -10*Log(P), where P is the probability that the observed match is a random event.
Individual ions scores > 45 indicate identity or extensive homology (p<0.05).
Protein scores are derived from ions scores as a non-probabilistic basis for ranking protein hits.

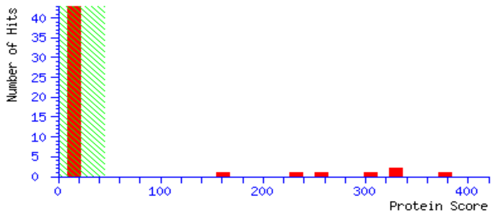

Matched peptide sequences: shown in Bold Red:

1 MGVEQNNHDA EEGTLLVGME YRTVSGVAGP LVILEKVKGP KYQEIVNIRL
51 GDGTTRRGQV LEVDGEKAVV QVFEGTSGID NKYTTVQFTG EVLKTPVSLD
101 MLGRIFNGSG KPIDNGPPIL PEAYLDISGS SINPSERTYP EEMIQTGIST
151 IDVMNSIARG QKIPLFSAAG LPHNEIAAQI CRQAGLVKRL EKSGSLLEDV
201 EEDNFAIVFA AMGVNMETAQ FFKRDFEENG SMERVTLFLN LANDPTIERI
251 ITPRIALTTA EYLAYECGKH VLVILTDMSS YADALREVSA AREEVPGRRG
301 YPGYMYTDLA TIYERAGRIE GRKGSITQIP ILTMPNDDIT HPTPDLTGYI
351 TEGQIYIDRQ LHNRQIYPPI NVLPSLSRLM KSAIGEGMTR RDHADVSNQL
401 YANYAIGKDV QAMKAVVGEE ALSSEDLLYL EFLDKFERKF VAQGAYDTRN
451 IFQSLDLAWT LLRIFPRELL HRIPAKTLDQ YYSREASN

Spot No.: **165**

NCBI accession No.: **gi|224071449** Species: *Populus trichocarpa*

PFF score: [59]

Protein name: predicted protein

Matched peptides No.: [1]   Sequence coverage %: [2]

Matched sequences: K.IDVIPHYPGR.L

Calculated Mr: **47121**   Calculated *p*I: **5.28**

Probability Based Mowse Score:

Ions score is -10*Log(P), where P is the probability that the observed match is a random event.
Individual ions scores > 44 indicate identity or extensive homology ($p<0.05$).
Protein scores are derived from ions scores as a non-probabilistic basis for ranking protein hits.

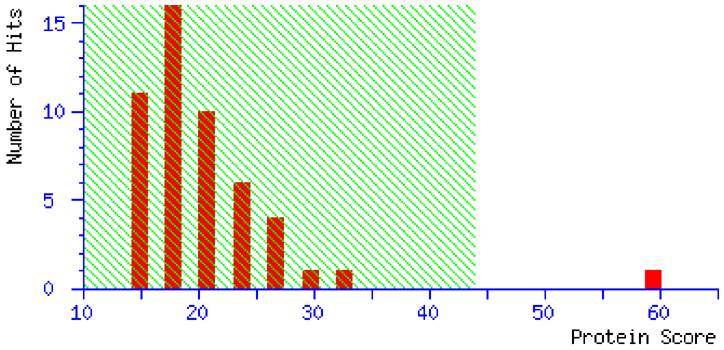

Matched peptide sequences: shown in Bold Red:

```
  1 MDGTSEATWP EILGSRNWDN LLDPLDLSLR KLILRCGDFC QATYDAFNND
 51 QNSRYCGTSR YGKRNFFHKV MLDNPENYQV SSFLYATARV SLPEAFLLHS
101 LSRDSWDRET NWIGYIAVTS DEQTKTLGRR EIYIAFRGTT RNYEWVDILG
151 AKLKSAKPLL RGATSTTHDQ ESSSSSDDDD DKVPKVMLGW LTMYISDDPN
201 SPFTKLSARA QLLAHIKELR ERYKDDDLSI IFTGHSLGAS LSILSAFDLV
251 ENGITDIPVS AFVFGSPQVG NKEFNERFNK YPNLKVLHIK NKIDVIPHYP
301 GRLMGYVYTG IEFEIDTRKS PSLKDSKNPS DWHNLQAMLH IVAGWNGEEQ
351 EFELKVKRSL ALVNKSSEFL KDECLVPGIW WVEKNKGMVR NEDGEWVLAP
401 PDEEDLPVPE C
```

Spot No.: **166**

NCBI accession No.: **gi|116789736** Species: *Picea sitchensis*

Protein name: unknown

PMF Mascot score: **81** Sequence coverage %: **60**

Matched peptides No.: **6** Total peptides No.: **48**

Calculated Mr: **13436** Calculated *p*I: **5.91**

Probability Based Mowse Score:

Protein score is -10*Log(P), where P is the probability that the observed match is a random event. Protein scores greater than 73 are significant (p<0.05).

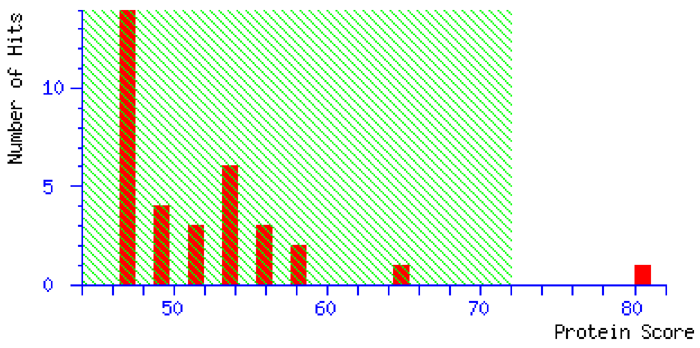

Matched peptide sequences: shown in Bold Red:

```
  1 MPSHSLIGTT TTNRRGSMED YPNPFNPETI HELFKLVWSQ TNKARAQKEG
 51 GETELEVGAA ASKKTRTTTA NSNALKLSCE FLRLFVTEAV QRAAIVAEVE
101 GSTQIEGTHL ERILPQLLLD F
```

Spot No.: 167

NCBI accession No.: **gi|115392253**

Species: *Cercocarpus betuloides*

PFF score: [51]　　Protein name: polyphenol oxidase

Matched peptides No.: [1]　　Sequence coverage %: [3]

Matched sequences: K.EKEQEEEILVIEGIEFDR.D

Calculated Mr: **51023**　　Calculated *p*I: **5.98**

Probability Based Mowse Score:

Ions score is -10*Log(P), where P is the probability that the observed match is a random event.
Individual ions scores > 45 indicate identity or extensive homology (p<0.05).
Protein scores are derived from ions scores as a non-probabilistic basis for ranking protein hits.

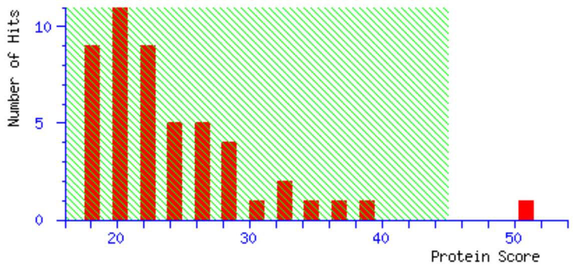

Matched peptide sequences: shown in Bold Red:

```
  1 PFAFAKPVAP PDVSKCGAAD LPTGAQPTNC CPPVASKIID FKLPSPTPLR VRPAAHAVDK
 61 AYIEKYSKAI ELMKALPDDD PRSFKQQANV HCAYCDGAYD QAGFPDLELQ IHNSWLFFPF
121 HRYYLYFHEK ILGSLIGDPT FALPFWNWDA PAGMPLPAIY ANPKSPLYDK FRAANHQPPT
181 LVDLDFNGTE DAASNETQIN ANLKIMYRQM VSNAKTPQLF FGNPYRAGDE PDPGGGSIEG
241 TPHGPVHLWT GDNTQPNFED MGNFYSAGRD PIFFAHHSNV DRLWSIWKTL GGKRTDITDT
301 DWLDSGFLFY DENAQMVRVK VRDCLESKNL GYVYQDANIP WLESKPTPRR VKAALGKIAK
361 KLHVAHAADN SSASKVVARA AFPIAKLDTK MSTVVARPKQ KKRSKKEKEQ EEEILVIEGI
421 EFDRDVAVKF DVYVNDEDDL PSGPDKSEFA GSFVS
```

Spot No.: 168

NCBI accession No.: **gi|225430941** Species: *Vitis vinifera*

PFF score: [210]

Protein name: regulator of ribonuclease-like protein 2

Matched peptides No.: [3] Sequence coverage %: [24]

Matched sequences: K.VFEDNVLIR.E;

R.DVDEINGCDIGVR.A;

R.ISDGEWLYADTDGILISR.T

Calculated Mr: **17984** Calculated *p*I: **5.39**

Probability Based Mowse Score:

Ions score is -10*Log(P), where P is the probability that the observed match is a random event.
Individual ions scores > 45 indicate identity or extensive homology (p<0.05).
Protein scores are derived from ions scores as a non-probabilistic basis for ranking protein hits.

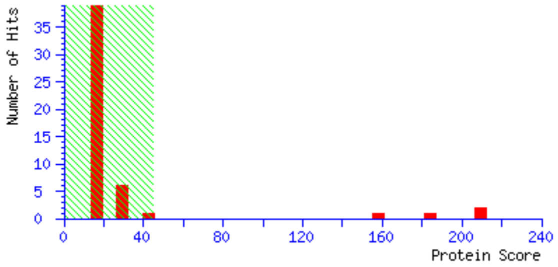

Matched peptide sequences: shown in Bold Red

```
  1 MALVTTAEVC DANPQLIVSG ELRALQPVFQ IYGRRPVFSG PIVTLKVFED
 51 NVLIREFLEE KGNGRVLVVD GGGSMRCAIL GGNPVVQAQN NGWAGIVVNG
101 CIRDVDEING CDIGVRALNS HPMKANKKGI GEKHVPIAIA GTRISDGEWL
151 YADTDGILIS RTELSV
```

Spot No.: **169**

NCBI accession No.: **gi|2832430** Species: *Hevea brasiliensis*

PFF score: [152] Protein name: prohevein

Matched peptides No.: [2] Sequence coverage %: [20]

Matched sequences: R.IVDQCSNGGLDLDVNVFR.Q;

K.YGWTAFCGPVGAHGQPSCGK.C

Calculated Mr: **20852** Calculated *p*I: **5.64**

Probability Based Mowse Score:

Ions score is -10*Log(P), where P is the probability that the observed match is a random event.
Individual ions scores > 45 indicate identity or extensive homology (p<0.05).
Protein scores are derived from ions scores as a non-probabilistic basis for ranking protein hits.

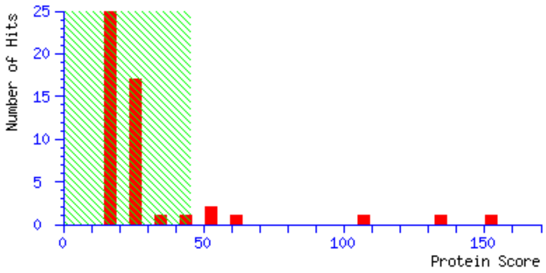

Matched peptide sequences: shown in Bold Red:

```
  1 EQCGRQAGGK LCPNNLCCSQ WGWCGSTDEY CSPDHNCQSN CKDSGEGVGG
 51 GSASNVLATY HLYNSQDHGW DLNAASAYCS TWDANKPYSW RSKYGWTAFC
101 GPVGAHGQPS CGKCLSVTNT GTGAKATVRI VDQCSNGGLD LDVNVFRQLD
151 TDGKGYERGH LTVNYQFVDC GDSFNPLFSV MKSSVIN
```

Spot No.: **170**

NCBI accession No.: **gi|298362843** Species: *Hevea brasiliensis*

PFF score: [110]

Protein name: actin-depolymerizing factor

Matched peptides No.: [1] Sequence coverage %: [15]

Matched sequences: K.LGEPTQSYEDFTASLPADECR.Y

Calculated Mr: **16220** Calculated *p*I: **5.92**

Probability Based Mowse Score:

Ions score is -10*Log(P), where P is the probability that the observed match is a random event.
Individual ions scores > 44 indicate identity or extensive homology ($p<0.05$).
Protein scores are derived from ions scores as a non-probabilistic basis for ranking protein hits.

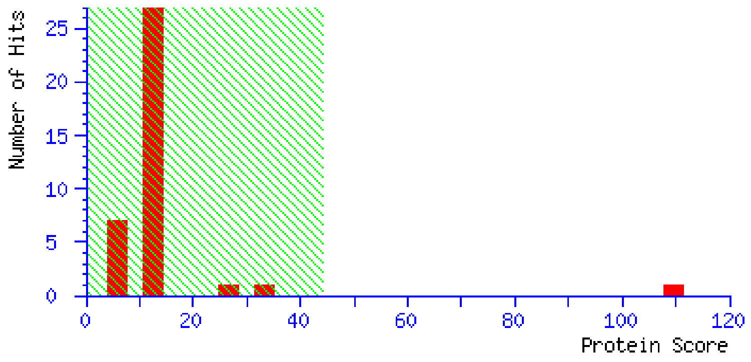

Matched peptide sequences: shown in Bold Red:

```
  1 MANAASGMAV HDDCKLRFLE LKAKRTYRYI VFKIEEKAKQ VIVEKLGEPT
 51 QSYEDFTASL PADECRYAVY DFDFVTEENC QKSRIFFIAW SPDTSRVRSK
101 MIYASSKDRF KRELDGIQVE LQATDPTEMG LDVFKSRAS
```

Spot No.: 171

NCBI accession No.: **gi|115392253**

Species: *Cercocarpus betuloides*

PFF score: [104]   Protein name: actin depolymerizing factor

Matched peptides No.: [1]   Sequence coverage %: [4]

Matched sequences: K.EKEQEEEILVIEGIEFDR.D

Calculated Mr: **58369**   Calculated *p*I: **9.23**

Probability Based Mowse Score:

Ions score is -10*Log(P), where P is the probability that the observed match is a random event.
Individual ions scores > 40 indicate peptides with significant homology.
Individual ions scores > 44 indicate identity or extensive homology (p<0.05).
Protein scores are derived from ions scores as a non-probabilistic basis for ranking protein hits.

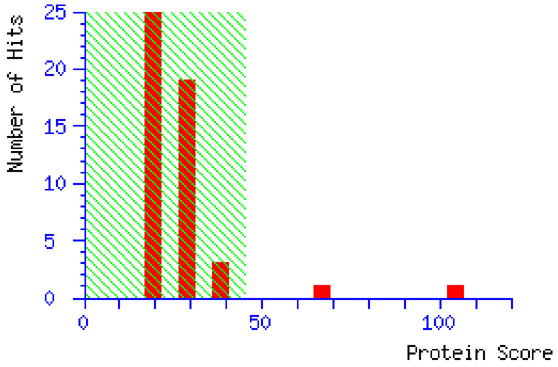

Matched peptide sequences: shown in Bold Red:

```
  1 PFAFAKPVAP PDVSKCGAAD LPTGAQPTNC CPPVASKIID FKLPSPTPLR VRPAAHAVDK
 61 AYIEKYSKAI ELMKALPDDD PRSFKQQANV HCAYCDGAYD QAGFPDLELQ IHNSWLFFPF
121 HRYYLYFHEK ILGSLIGDPT FALPFWNWDA PAGMPLPAIY ANPKSPLYDK FRAANHQPPT
181 LVDLDFNGTE DAASNETQIN ANLKIMYRQM VSNAKTPQLF FGNPYRAGDE PDPGGGSIEG
241 TPHGPVHLWT GDNTQPNFED MGNFYSAGRD PIFFAHHSNV DRLWSIWKTL GGKRTDITDT
301 DWLDSGFLFY DENAQMVRVK VRDCLESKNL GYVYQDANIP WLESKPTPRR VKAALGKIAK
361 KLHVAHAADN SSASKVVARA AFPIAKLDTK MSTVVARPKQ KKRSKKEKEQ EEEILVIEGI
421 EFDRDVAVKF DVYVNDEDDL PSGPDKSEFA GSFVS
```

Spot No.: 172

NCBI accession No.: **gi|2832430** Species: *Hevea brasiliensis*

PFF score: [252]

Protein name: prohevein

Matched peptides No.: [3] Sequence coverage %: [26]

Matched sequences: R.QLDTDGKGYER.G;

R.IVDQCSNGGLDLDVNVFR.Q;
K.YGWTAFCGPVGAHGQPSCGK.C

Calculated Mr: **20852** Calculated *p*I: **5.64**

Probability Based Mowse Score:

Ions score is -10*Log(P), where P is the probability that the observed match is a random event.
Individual ions scores > 45 indicate identity or extensive homology (p<0.05).
Protein scores are derived from ions scores as a non-probabilistic basis for ranking protein hits.

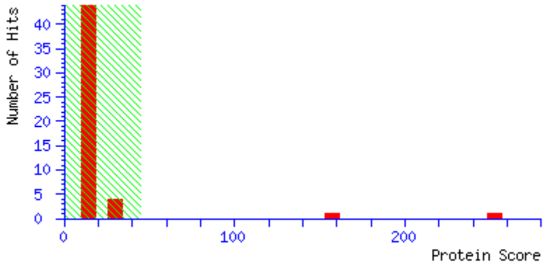

Matched peptide sequences: shown in Bold Red

```
  1  EQCGRQAGGK LCPNNLCCSQ WGWCGSTDEY CSPDHNCQSN CKDSGEGVGG
 51  GSASNVLATY HLYNSQDHGW DLNAASAYCS TWDANKPYSW RSKYGWTAFC
101  GPVGAHGQPS CGKCLSVTNT GTGAKATVRI VDQCSNGGLD LDVNVFRQLD
151  TDGKGYERGH LTVNYQFVDC GDSFNPLFSV MKSSVIN
```

Spot No.: **173**

NCBI accession No.: **gi|255574159** Species: *Ricinus communis*

Protein name: proteasome subunit beta type 6,9, putative

PMF Mascot score: **101** Sequence coverage %: **51**

Matched peptides No.: **14** Total peptides No.: **60**

Calculated Mr: **24912** Calculated *p*I: **5.17**

Probability Based Mowse Score:

Protein score is -10*Log(P), where P is the probability that the observed match is a random event. Protein scores greater than 73 are significant (p<0.05).

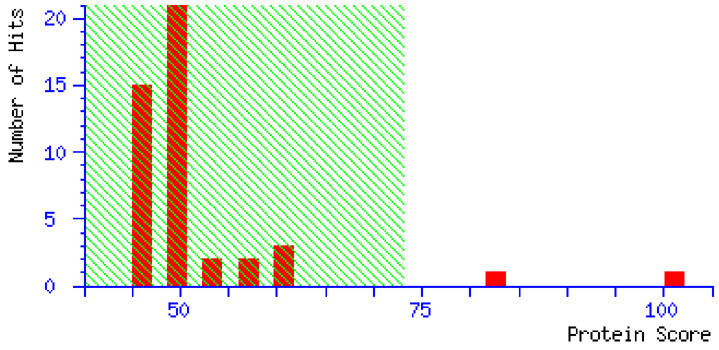

Matched peptide sequences: shown in Bold Red:

```
  1 MDLKAPHSMG TTIIGVTYNG GVVLGADSRT STGMYVANRA SDKITQLTDN
 51 VYVCRSGSAA DSQIVSDYVR YFLHQHTIQL GQPATVKVAA NLVRLISYNN
101 KNMLETGLIV GGWDKYDGGK IYGIPLGGTI IEQPFAIGGS GSSYLYGFFD
151 QAWKEGMTKD EAEQLVVKAV SLAMARDGAS GGVVRTVIIN SEGVTRNFYP
201 GDSLPLWHEE LEPQNSLLDI LNASSPEPMN I
```

Spot No.: **174**

NCBI accession No.: gi|242059605   Species: *Sorghum bicolor*

PFF score: [117]

Protein name: hypothetical protein SORBIDRAFT_03g043200

Matched peptides No.: [2]   Sequence coverage %: [6]

Matched sequences: K.RFFPSEFGNDVDHVHAVEPAK.S;

R.FFPSEFGNDVDHVHAVEPAK.S

Calculated Mr: **34294**   Calculated *p*I: **5.93**

Probability Based Mowse Score:

Ions score is -10*Log(P), where P is the probability that the observed match is a random event.
Individual ions scores > 45 indicate identity or extensive homology (p<0.05).
Protein scores are derived from ions scores as a non-probabilistic basis for ranking protein hits.

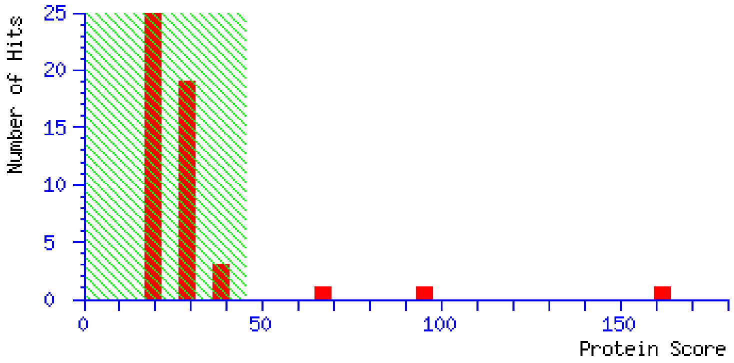

Matched peptide sequences: shown in Bold Red:

```
  1 MSSAAGHKER SRVLVIGGTG YIGRFIVAAS AREGHPTYVL VRDPAPADPA
 51 KAAVLQGFRD AGVTLVKGDL YNHESLVVAM ESADVVISAV GYAQLPDQTR
101 IISAIKDAGN IKRFFPSEFG NDVDHVHAVE PAKSVFAAKA SIRRAVEAEG
151 IPYTYISSNF FAGRFLPAIG QIGVTGLPID KVLILGDGNV KAIFGTEEDV
201 GTYTIKAVDD PRTLNKILYL RPPSNILSHN ELISLWEKKV GKTFERVYIP
251 EDDVLKKIQE SPIPLNRALS ISHSAWVKGD HTNFEIDPFF GVEATDLYPD
301 VKYTTVDEYL NKFL
```

Spot No.: **175**

NCBI accession No.: **gi|2832654** Species: *Arabidopsis thaliana*

Protein name: chloroplast protein cs/ch-42

PMF Mascot score: **86** Sequence coverage %: **37**

Matched peptides No.: **16** Total peptides No.: **78**

Calculated Mr: **66408** Calculated *p*I: **6.44**

Probability Based Mowse Score:

Protein score is -10*Log(P), where P is the probability that the observed match is a random event. Protein scores greater than 73 are significant (p<0.05).

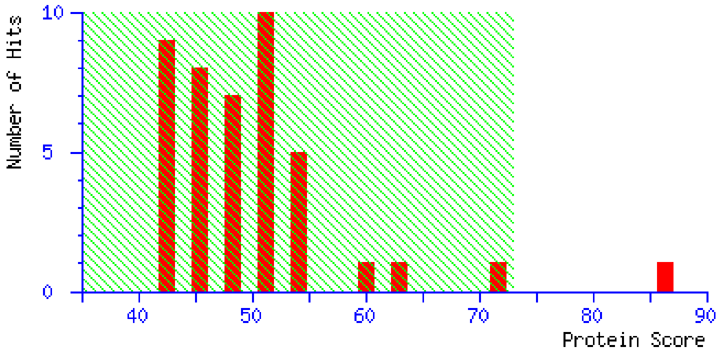

Matched peptide sequences: shown in Bold Red:

1	MLCFSASRLD	DFDLGSSPPK	KGSKTTTKSM	DCEEICASSK	SDKSDDLDFG
51	LDLPITRQVP	SKANTDVQAK	ASAEKESQNY	KTTDTLVVNK	SKNSNQAALE
101	SMGDFEAVES	PQGSRKKASQ	THTMCVQPQS	VDTSPLKTSC	SKVEEKNEPC
151	PSNETIAPSP	LHASEIAHIA	VNRETSPDIH	ELCRSGTKED	CPIDPENANK
201	KMITTMESSY	EKIEQTSPSI	SSHLCSDKIE	HQQEEMGTDT	QAEIQDNTKG
251	ALYNSDAGHS	LTTLSGKISP	GTRTSQTAKV	QDLSEKLPLD	PSHSMAGLDN
301	LRAMQNKDLG	LIRSRFFKKP	EKPESHVLES	SPIETEIQPV	IRENIGSSLN
351	PTNDTSSHEK	IIHKDHSNGK	TVENVAGQMD	HLKLQAKNTT	REKSILQINI
401	SSKLDASSLT	QKLSNHLSSG	VESLQKPKLN	SLGRPKLGNI	MADLRAVKTQ
451	RNVEELTHRA	VQGKVRFTNR	SVILIVTRRK	SFLCQESRID	TKTTKEPVKE
501	SPQTKSHYQN	INMANLEIPI	TENADNIEKA	EAYTKELENV	NRALFSIMNI
551	LNIICNILKK	KHEEAKELLV	RAVVDNNKLL	MLNHPLYEDQ	ISFL

Spot No.: **176**

NCBI accession No.: **gi|27449248** Species: *Hevea brasiliensis*

PFF score: [487] Protein name: ascorbate peroxidase

Matched peptides No.: [5] Sequence coverage %: [34]

Matched sequences: R.EDKPEPPPEGR.L;

R.IAWHSAGTYDANTK.T;
R.HAAEQAHAANNGLDIAVR.L;
K.YAADEDAFFADYAEAHVK.L;
K.EGLLQLPTDTVLVTDPVFRPYVEK.Y

Calculated Mr: **27526** Calculated *p*I: **5.78**

Probability Based Mowse Score:

Ions score is -10*Log(P), where P is the probability that the observed match is a random event.
Individual ions scores > 45 indicate identity or extensive homology (p<0.05).
Protein scores are derived from ions scores as a non-probabilistic basis for ranking protein hits.

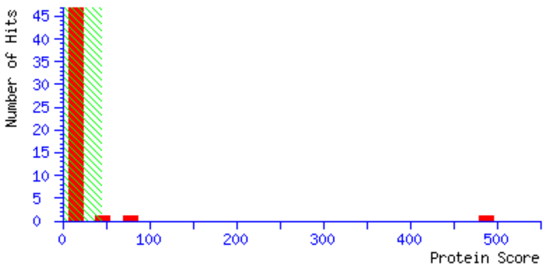

Matched peptide sequences: shown in Bold Red:

```
  1 MTKNYPKVSE EYQKAIDKAK RKLRGFIAEK GCAPLMLRIA WHSAGTYDAN
 51 TKTGGPFGTM RHAAEQAHAA NNGLDIAVRL LEPIKQQFPI LSYADFYQLA
101 GVVAVEITGG PEIPFHPGRE DKPEPPPEGR LPNATKGADH LREVFGKTMG
151 LSDKDIVVLS GGHTLGRCHK ERSGFDGPWT ANPLIFDNSF FTELLAGQKE
201 GLLQLPTDTV LVTDPVFRPY VEKYAADEDA FFADYAEAHV KLSELGFAEA
```

Spot No.: **177**

NCBI accession No.: **gi|357480975** Species: *Medicago truncatula*

PFF score: [369]

Protein name: Proteasome subunit beta type

Matched peptides No.: [7] Sequence coverage %: [42]

Matched sequences: K.NVALYQFR.N;
R.VQFTEYIQK.N;
R.LVVAPPNFVIK.I;
R.NGIPLTTAAAANFTR.G;
K.GAFGYGSYFSLSMMDR.H;
K.ETGPSLYYIDYIATLHK.L;
K.LIAASGEPGDRVQFTEYIQK.N

Calculated Mr: **22572** Calculated *p*I: **6.06**

Probability Based Mowse Score:

Ions score is -10*Log(P), where P is the probability that the observed match is a random event.
Individual ions scores > 45 indicate identity or extensive homology (p<0.05).
Protein scores are derived from ions scores as a non-probabilistic basis for ranking protein hits.

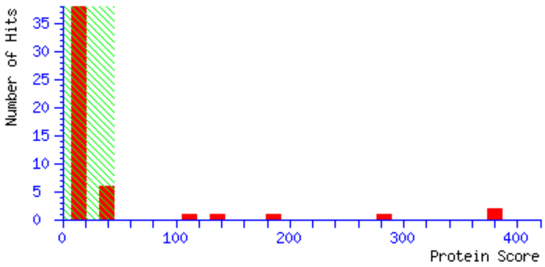

Matched peptide sequences: shown in Bold Red:

```
  1 MECVFGLVGN GFAIVVADTS AVHSILVHKS NEDKIMFLDS HKLIAASGEP
 51 GDRVQFTEYI QKNVALYQFR NGIPLTTAAA ANFTRGELAT ALRKNPYSVN
101 ILLAGYDKET GPSLYYIDYI ATLHKLEKGA FGYGSYFSLS MMDRHFHSGM
151 NVEEAIDLVD KCILEIRSRL VVAPPNFVIK IVDKDGAREY AWRESVKDTP
201 ASA
```

Spot No.: **178**

NCBI accession No.: **gi|255541320** Species: *Ricinus communis*

PFF score: [302]

Protein name: proteasome subunit beta type, putative

Matched peptides No.: [4] Sequence coverage %: [28]

Matched sequences: K.HLIYQHQHNK.Q;
K.GCVYTYDAVGSYER.V;
R.FFPYYAFNVLGGLDSEGK.G;
R.VGYSAQGSGSTLIMPFLDNQLK.S

Calculated Mr: **24880** Calculated *p*I: **6.08**

Probability Based Mowse Score:

Ions score is -10*Log(P), where P is the probability that the observed match is a random event.
Individual ions scores > 45 indicate identity or extensive homology (p<0.05).
Protein scores are derived from ions scores as a non-probabilistic basis for ranking protein hits.

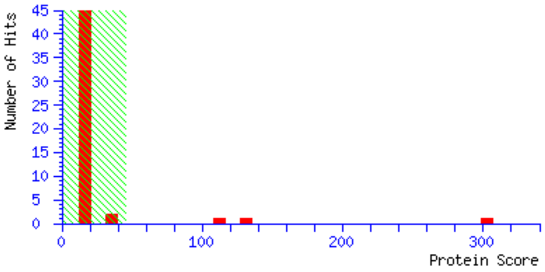

Matched peptide sequences: shown in Bold Red:

1 MTKQQANWSP YDNNGGSCVA IAGADYCVIA ADTRMSTGYN ILTRDYSKIC
51 QLAEKSVMAS SGFQADVQAL QKHLKAK**HLI** **YQHQHNK**QMS CPAMGQLLSN
101 TLYFKR**FFPY** **YAFNVLGGLD** **SEGKGCVYTY** **DAVGSYERVG** **YSAQGSGSTL**
151 **IMPFLDNQLK** SPSPLLLPAQ DAVTPLSEPE AVDLVKTVFA SATERDIYTG
201 DKVEIVVLNA DGIRREYMEL RKD

Spot No.: 179

NCBI accession No.: gi|350537255

Species: *Solanum lycopersicum*

Protein name：small zinc finger-like protein

PMF score: 82 Sequence coverage %: 70

Matched peptides No.: 7 Total peptides No.: 40

Calculated Mr: 9669 Calculated *p*I: 6.56

Probability Based Mowse Score:

Protein score is -10*Log(P), where P is the probability that the observed match is a random event.
Protein scores greater than 73 are significant (p<0.05).

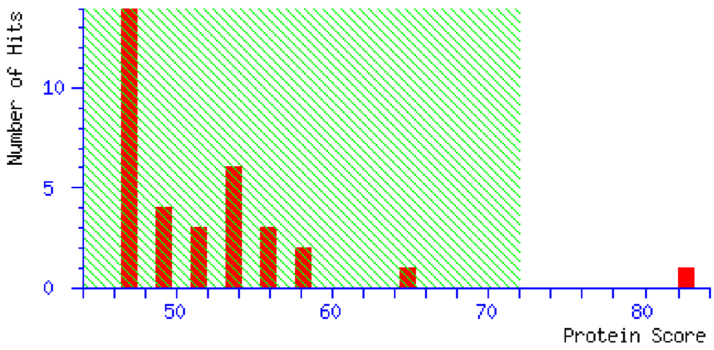

Matched peptide sequences: shown in Bold red:

```
 1 MAGVPSNLER EQIFSMAEKE MEYRVEMFNK LTHTCFKKCV ENKYKDSELN
51 MGENSCIDRC VSKYWQVTNL VGTLLGNTRP M
```

Spot No.: **180**

NCBI accession No.: **gi|37622210** Species: *Hevea brasiliensis*

Protein name: small rubber particle protein

PMF Mascot score: **107** Sequence coverage %: **60**

Matched peptides No.: **10** Total peptides No.: **38**

Calculated Mr: **18929** Calculated *p*I: **8.99**

Probability Based Mowse Score:

Protein score is -10*Log(P), where P is the probability that the observed match is a random event.
Protein scores greater than 73 are significant (p<0.05).

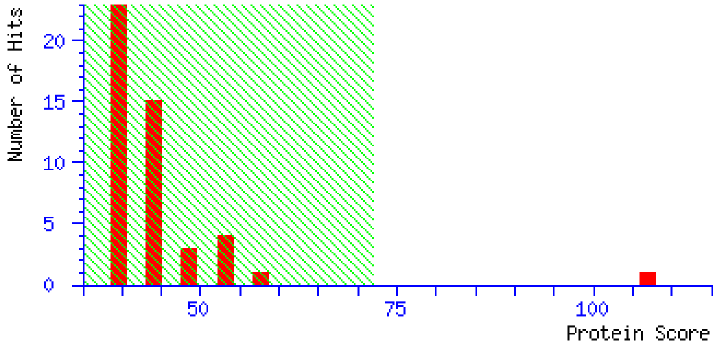

Matched peptide sequences: shown in Bold Red:

```
  1  KFHDVPNEVL KFVDRKVDES VTSLDSRVPP VVKQVSAQAY SVAREAPVAA
 51  RAVASEVHQS GVKETASGLA KTLYTKYEPK AKELYSKYEP KAEQCAVTAW
101  RRLNQLPLFP QVAQVVVPTA AYCSEKYNQT VLSTFEKGYR VSSYLPLVPT
151  ERIAKVFSDD VAQSMPLVSS
```

Spot No.: 181

NCBI accession No.: gi|168038495

Species: *Physcomitrella patens subsp. patens*

Protein name：predicted protein

PMF score: **81** Sequence coverage %: **36**

Matched peptides No.: **18** Total peptides No.: **81**

Calculated Mr: **61349** Calculated *p*I:**5.80**

Probability Based Mowse Score:

Protein score is -10*Log(P), where P is the probability that the observed match is a random event. Protein scores greater than 73 are significant (p<0.05).

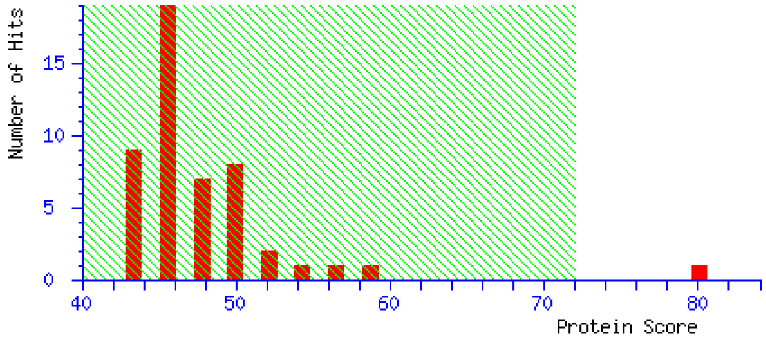

Matched peptide sequences: shown in Bold red:

1 MQAPVLVLNE STKRETGGRV HHANIQASKA VADVIRTTLG PRSMLKMLLD
51 ASGGIVLTND GNAILRELDV SHPAAKSMIE LSRTQDEEVG DGTTSVIILA
101 GEMLYVAEQF LDRQFHPTVI CRAYTKALED AVAALKEIAF DIDITDRTMM
151 LNIVRSCIGT KFTSRFGTLI ADLAIDSVAT ISVDLGNGLR EVDIKKYIKV
201 EKIPGGQLED SKVLKGVMFN KDVVSPGKMR RKILNPRIIL LDSPLEYKKG
251 ENQTNAEIMK EEDWATLLRM EEEYIEQLCV QIIKFKPDLV ITEKGLSDLA
301 SHYLSKAGIS AIRRLRKTDN NRIARACGAT IVNRPEELQE SDVGTRCGLF
351 EVKKIGDEYF TFIVQCNEPK ACTVLLRGPS KDLLNEVERN LADAMGVARN
401 VIKDAKLVPG GGASEMAVSA ILKKNSMSIE GVEQWPYRAV AQALEVIPRT
451 LAQNCGVNVI RTMTALQAKH ANAENPMFGI DGNTGELTDM KELGVWDSFG
501 VKLQTFKTAI EAACMLLRID DIVSGIKKKQ APGAAPKQPM TSTGEDVDSE
551 QMIPE

Spot No.: **182**

NCBI accession No.: **gi|164472662** Species: *Triticum aestivum*

Protein name: calcium-dependent protein kinase

PMF Mascot score: **82** Sequence coverage %: **28**

Matched peptides No.: **17** Total peptides No.: **74**

Calculated Mr: **61353** Calculated *p*I: **6.04**

Probability Based Mowse Score:

Protein score is -10*Log(P), where P is the probability that the observed match is a random event. Protein scores greater than 73 are significant (p<0.05).

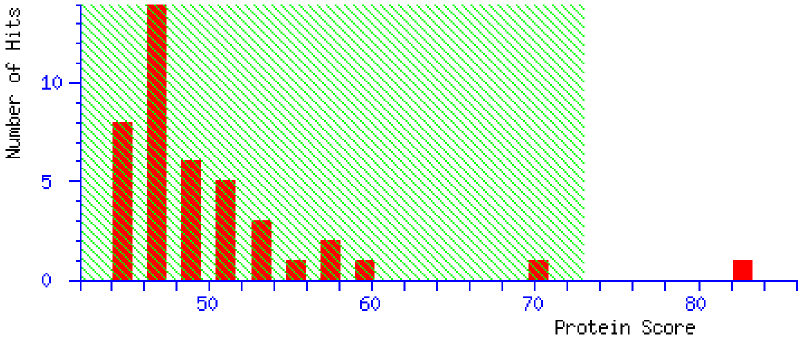

Matched peptide sequences: shown in Bold Red:

```
  1 MGNCCRSPAA AAREDVSSSH FPASAGKRKP HQARNGGGGG AGGGGGGGGA
 51 GAGGEKKRLS VLGEEGPDVS GGIDDKYALD RELGRGEFGV TYLCMDRGSK
101 ELLACKSISK RKLRTPVDVE DVRREVAIMR HLPRSASIVT LREACEDDGA
151 GHLVMELCEG GELFDRIVAR GHYTERAAAA VTRTIVEVVQ LCHHHGVIHR
201 DLKPENFLFA NKKENSPLKA IDFGLSIFFK PGEKFSEIVG SPYYMAPEVL
251 KRNYGPEIDI WSAGVILYIL LCGVPPFWAE TEQGVAQAIL RGNIDFKREP
301 WPHVSDNAKD LDRQMLQPDP KIRLTAKQVL EHTWLQNAKK APNVPLGDIV
351 KSRLKQFSRM NRFKRRALRV IADHLSAEEV EDIKEMFKVM DTDNDGIVSY
401 EELKSGIAKF GSHLAESEVQ MLIEAVDTNG RGALDYGEFL AVSLHLQRMA
451 NDEHLRRAFL FFDKDGDGFI EPEELQEALA EDGAVDITEV VKDILQEVDT
501 DKDGKISFEE FVAMMKTGTD WRKASRHYSR GRFNSLSIRL IKDGSVKMGN
551 E
```

Spot No.: **183**

NCBI accession No.: **gi|32527831**

Species: *Populus tremula x Populus tremuloides*

Protein name: UDP-glucose pyrophosphorylase

PMF Mascot score: **92** Sequence coverage %: **29**

Matched peptides No.: **12** Total peptides No.: **45**

Calculated Mr: **51949** Calculated *p*I: **5.68**

Probability Based Mowse Score:

Protein score is -10*Log(P), where P is the probability that the observed match is a random event. Protein scores greater than 73 are significant (p<0.05).

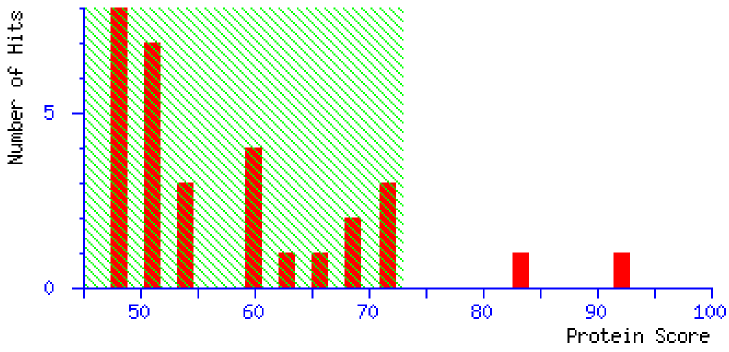

Matched peptide sequences: shown in Bold Red:

1 MATDTEKISQ LKSAVANLNQ ISESEKTGFV NLVSR**YLSGE** **AQQVEWSK**IQ
51 TPTDEVVVPY DTLESTPEDP EETKMLLDKL VVLK**LNGGLG** **TTMGCTGPKS**
101 **VIEVR**NGLTF LDLIVIQIES LNKKYGCSVP LLLMNSFNTH DDTQKIIEKY
151 SNSNIEIHTF NQSQYPR**LVA** **DDFVPLPSKG** HTDK**DGWYPP** **GHGDVFPSLK**
201 NSGKLDALLS QGKEYVFVAN SDNLGAVVDL KILNHLIR**NK** **NEYCMEVTPK**
251 TLADVK**GGTL** **ISYEGKVQLL** **EIAQVPDQHV** **NEFK**SIEKFK IFNTNNLWVN
301 LKAIKRLVEA DALK**MEIIPN** **PKEVDGVKVL** **QLETAAGAAI** **R**FFDHAIGIN
351 VPRSRFLPVK ASSDLLLVQS DIYTPVVDGF VIRNPGRANP ANPSIELGPE
401 FKKVASFLSR FKSIPSIIEL DSLKVAGDVW FGVNVTLKGK VSIVVKSGVK
451 LEIPEGVVLE NK**EINGPKDL**

Spot No.: **184**

NCBI accession No.: **gi|356576773** Species: *Glycine max*

Protein name: seryl-tRNA synthetase-like

PMF Mascot score: **92** Sequence coverage %:**28**

Matched peptides No.: **17** Total peptides No.: **53**

Calculated Mr: **51822** Calculated *p*I:**6.04**

Probability Based Mowse Score:

Protein score is -10*Log(P), where P is the probability that the observed match is a random event. Protein scores greater than 73 are significant (p<0.05).

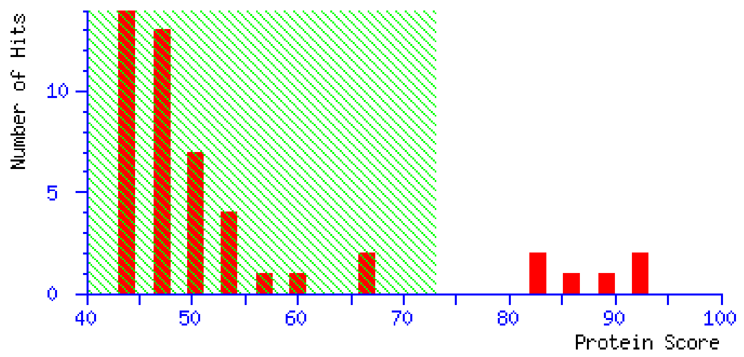

Matched peptide sequences: shown in Bold Red

```
  1 MLDINLFREE KGHNPEFIRE SQRRRFASVE VVDEVINLDK EWRKRQFELE
 51 NLRKEFNKIN KEVSKLKRAG EDATKFISES EETKKSIGEK EVEVRETLNL
101 LNSKLETIGN LVHDSVPISQ DEANNVVVRS WGEKRVEPKL KNHVDLVELL
151 GIADTKKGAD VAGGRGFYLK GDGVRLNQAL INFGLDFLEK REYTLLHTPF
201 FMRKDIMSKC AQLAQFDEEL YKVTGEGDDK YLIATAEQPL CAYHLDDWIH
251 PTQLPLRYAG YSSCFRKEAG SHGRDTLGIF RVHQFEKVEQ FCLTSPNDND
301 SWDMHEEMLK NSEDFYKALN LPYQVVSIVS GALNDAAAKK YDLEAWFPAS
351 QAYRELVSCS NCTDYQARRL EIRYGQKKSN EQMKQYVHLL NSTLTATERT
401 ICCILENNQK EDGVEIPEAL RPFMGGKTFL PFKNQPSNEA KGKKSKA
```

Spot No.: **185**

NCBI accession No.: gi|357514973   Species: *Medicago truncatula*

PFF score: [51]

Protein name: annexin, putative

Matched peptides No.: [2]   Sequence coverage %: [7]

Matched sequences: R.SSEELLGAR.K;

R.FENALVIWAMHPWER.D + Oxidation (M)

Calculated Mr: **35872**   Calculated *p*I: **6.04**

Probability Based Mowse Score:

Ions score is -10*Log(P), where P is the probability that the observed match is a random event.
Individual ions scores > 44 indicate identity or extensive homology (p<0.05).
Protein scores are derived from ions scores as a non-probabilistic basis for ranking protein hits.

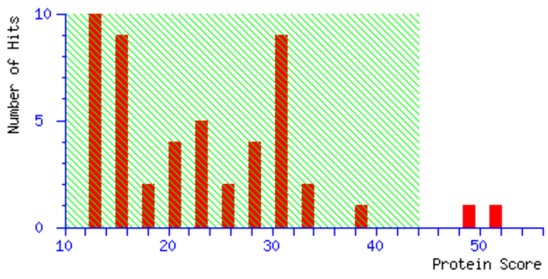

Matched peptide sequences: shown in Bold Red:

```
  1 MAHPEELEAL TKAFSGLGVD EKSLISILGK SHPEHRKSFR KGSPHLFIED
 51 ERSFERWDDD SVHLLRQEFA RFENALVIWA MHPWERDARL IYEALREGPQ
101 SYGVIVEIAC TRSSEELLGA RKAYHSLFDH SIEEDVATHI SGTERKLLVA
151 LASAYRYEGP KVKEDSAKFE AKIFANAVKN GDKTNPIEDD EVIRILSTRS
201 KPHLKAVYKH YKEISGNGII EDLGAANLIL KETVECLCTP HAFFSKVLDK
251 AMRKDADHNT KKALTRVIVT QADIDLKEIS EQYNSLYGIP LSKKVEETAN
301 GNYKDFLLAL ISREN
```

Spot No.: **186**

NCBI accession No.: **gi| 1351887** Species: *Malus x domestica*

PFF score: [63]

Protein name: Alcohol dehydrogenase

Matched peptides No.: [1] Sequence coverage %: [7]

Matched sequences: K.SMGMPDEDEAAVELAWSR.F

Calculated Mr: 42066 Calculated *p*I: **6.48**

Probability Based Mowse Score:

Ions score is -10*Log(P), where P is the probability that the observed match is a random event.
Individual ions scores > 45 indicate identity or extensive homology (p<0.05).
Protein scores are derived from ions scores as a non-probabilistic basis for ranking protein hits.

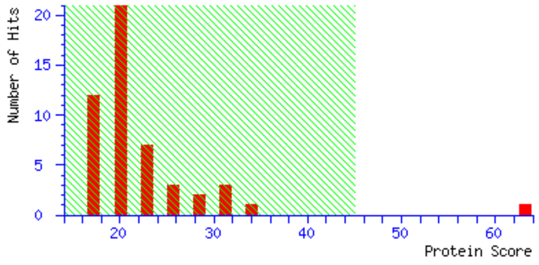

Matched peptide sequences: shown in Bold Red:

```
  1 MLAAAIRSLS PSSTSSIFNK SALPPLLRSL SPSLSNSRFQ IRSMADSASS
 51 PFKKVQIHRD NTTFDAYVIG KDDAPGIVVV QEWWGVDFEI KNHAEKISQL
101 EPGFKALIPD LYRGKVGLDV AEAQHLMDGL DWQGAVKDIG ASVNWLKANG
151 SKKAGVTGFC MGGALAIASS VLVPEVDAVV AFYGVPPSEL ADATQAKAPI
201 QAHFGELDNF VGFADITAAK ALEEKLKASG VPSEVHIYPG NAHAFMNRSA
251 EGVKRRKSMG MPDEDEAAVE LAWSRFRSWM NQYLSA
```

Spot No.: **187**

NCBI accession No.: **gi|380468126** Species: *Hevea brasiliensis*

PFF score: [123]

Protein name: caffeic acid 3-O-methyltransferase

Matched peptides No.: [3] Sequence coverage %: [11]

Matched sequences: K.NPEAPVLLDR.M;
K.SWYHLNEAILEGGTPFNR.A;
R.AYGMNQFEYPGTDQR.F + Oxidation (M)

Calculated Mr: **40957** Calculated *p*I: **5.54**

Probability Based Mowse Score:

Ions score is -10*Log(P), where P is the probability that the observed match is a random event.
Individual ions scores > 45 indicate identity or extensive homology (p<0.05).
Protein scores are derived from ions scores as a non-probabilistic basis for ranking protein hits.

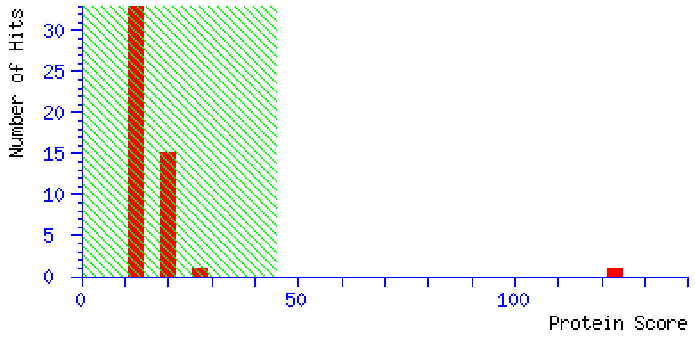

Matched peptide sequences: shown in Bold Red:

1 MTGSEQSNSA SIMSGRSDEE TWNLAIDLAN TVILPMVLKS ALELNVIDII
51 STAGNSGASL SAPEIAQRIP EAK**NPEAPVL** **LDR**MLRLLAT YDIVKCSSNT
101 KENGEVERLY APGPICKFLT KNKNGSGSAA PLLLLHHDEV FMK**SWYHLNE**
151 **AILEGGTPFN** **RAYGMNQFEY** **PGTDQR**FNRV FNDAMSSYTT YLVKKILDAY
201 KGFDGLKSLV DVGGNSGVTL NSITSKYPHI KGINYDLPHV LADAPSYPGV
251 EHVAGDMFKS VPKGDAIILK WVLHDWNDDL CLKLLKNCWE ALPSNGKVIV
301 VESILPTVPE NNVTSQVLHK EDLMLLSFNV GGKERTRQEF EALASKSGFS
351 SCEFICCAYN SWVIEFHK

Spot No.: **188**

NCBI accession No.: **gi|380468126** Species: *Hevea brasiliensis*

PFF score: [117]

Protein name: caffeic acid 3-O-methyltransferase

Matched peptides No.: [2] Sequence coverage %: [8]

Matched sequences: K.SWYHLNEAILEGGTPFNR.A;

R.AYGMNQFEYPGTDQR.F + Oxidation (M)

Calculated Mr: **40957** Calculated *p*I: **5.54**

Probability Based Mowse Score:

Ions score is -10*Log(P), where P is the probability that the observed match is a random event.
Individual ions scores > 44 indicate identity or extensive homology (p<0.05).
Protein scores are derived from ions scores as a non-probabilistic basis for ranking protein hits.

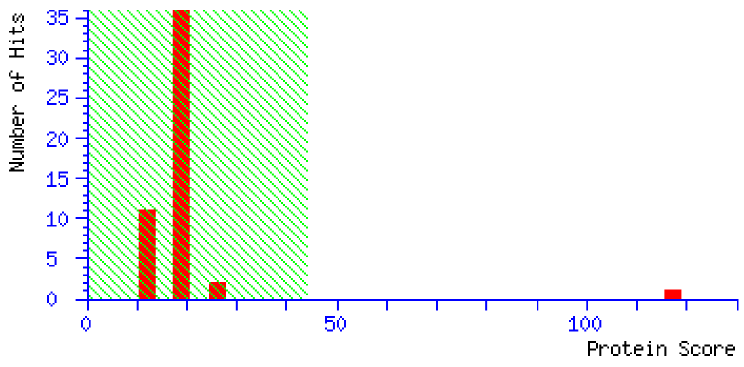

Matched peptide sequences: shown in Bold Red:

1	MTGSEQSNSA	SIMSGRSDEE	TWNLAIDLAN	TVILPMVLKS	ALELNVIDII
51	STAGNSGASL	SAPEIAQRIP	EAKNPEAPVL	LDRMLRLLAT	YDIVKCSSNT
101	KENGEVERLY	APGPICKFLT	KNKNGSGSAA	PLLLLHHDEV	FMK**SWYHLNE**
151	**AILEGGTPFN**	**RAYGMNQFEY**	**PGTDQR**FNRV	FNDAMSSYTT	YLVKKILDAY
201	KGFDGLKSLV	DVGGNSGVTL	NSITSKYPHI	KGINYDLPHV	LADAPSYPGV
251	EHVAGDMFKS	VPKGDAIILK	WVLHDWNDDL	CLKLLKNCWE	ALPSNGKVIV
301	VESILPTVPE	NNVTSQVLHK	EDLMLLSFNV	GGKERTRQEF	EALASKSGFS
351	SCEFICCAYN	SWVIEFHK			

Spot No.: **189**

NCBI accession No.: **gi|284433794** Species: *Jatropha curcas*

PFF score: [159]

Protein name: glutathione S-transferase omega

Matched peptides No.: [3] Sequence coverage %: [10]

Matched sequences: K.IIGESLDLIK.Y;

K.YDITAGRPK.L;

K.ELVEFYK.K

Calculated Mr: **27480** Calculated *p*I: **5.41**

Probability Based Mowse Score:

Ions score is -10*Log(P), where P is the probability that the observed match is a random event.
Individual ions scores > 45 indicate identity or extensive homology (p<0.05).
Protein scores are derived from ions scores as a non-probabilistic basis for ranking protein hits.

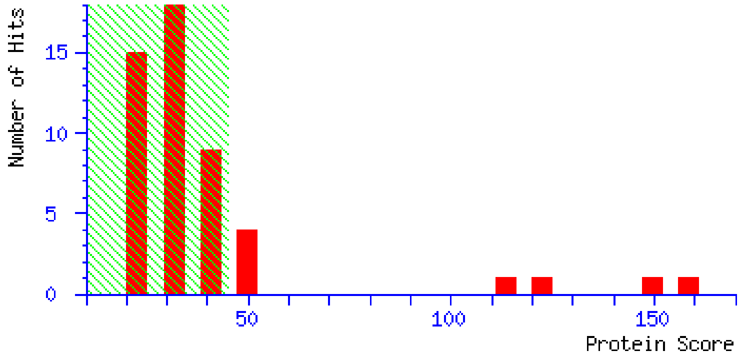

Matched peptide sequences: shown in Bold Red:

```
  1 MAATALDKSL PEKLPPILDA TAEQPPLFDG TIRLYTSYAC PFAQRVWITR
 51 NYKGLQDKIK LVPLNLQNRP SWYGEKVYPA NKVPALEHNG KIIGESLDLI
101 KYIDANFEGP SLLPDDPAKK EFAEELFSYT DTFTKTVFTS FKGDAVKDAS
151 PAFDYLENAL HKFEDGPFLL GQFSLVDIAY IPFVERFQIF FSEVFKYDIT
201 AGRPKLAAWI EEMNKIEAYK QTKTDPKELV EFYKKRLLAQ
```

Spot No.: **190**

NCBI accession No.: **gi|255567721** Species: *Ricinus communis*

PFF score: [52]

Protein name: carboxymethylenebutenolidase, putative

Matched peptides No.: [1] Sequence coverage %: [6]

Matched sequences: K.SMGMPDEDEAAVELAWSR.F + 2 Oxidation (M)

Calculated Mr: 30833 Calculated *p*I: **6.09**

Probability Based Mowse Score:

Ions score is -10*Log(P), where P is the probability that the observed match is a random event.
Individual ions scores > 45 indicate identity or extensive homology (p<0.05).
Protein scores are derived from ions scores as a non-probabilistic basis for ranking protein hits.

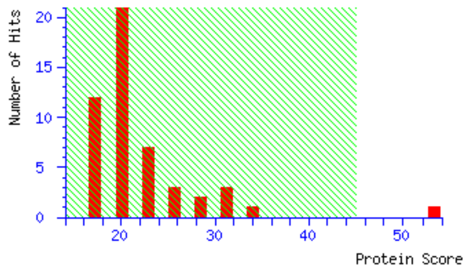

Matched peptide sequences: shown in Bold Red:

```
  1 MLAAAIRSLS PSSTSSIFNK SALPPLLRSL SPSLSNSRFQ IRSMADSASS
 51 PFKKVQIHRD NTTFDAYVIG KDDAPGIVVV QEWWGVDFEI KNHAEKISQL
101 EPGFKALIPD LYRGKVGLDV AEAQHLMDGL DWQGAVKDIG ASVNWLKANG
151 SKKAGVTGFC MGGALAIASS VLVPEVDAVV AFYGVPPSEL ADATQAKAPI
201 QAHFGELDNF VGFADITAAK ALEEKLKASG VPSEVHIYPG NAHAFMNRSA
251 EGVKRRKSMG MPDEDEAAVE LAWSRFRSWM NQYLSA
```

Spot No.: **191**

NCBI accession No.: **gi|125573095**

Species: *Oryza sativa Japonica Group*

PFF score: [59]   Protein name: hypothetical protein OsJ_04535

Matched peptides No.: [1]   Sequence coverage %: [8]

Matched sequences: M.SSWNSPYYDTSSYGAGSGGGGGGGR.R

Calculated Mr: **29680**   Calculated *p*I: **10.36**

Probability Based Mowse Score:

Ions score is -10*Log(P), where P is the probability that the observed match is a random event.
Individual ions scores > 45 indicate identity or extensive homology (p<0.05).
Protein scores are derived from ions scores as a non-probabilistic basis for ranking protein hits.

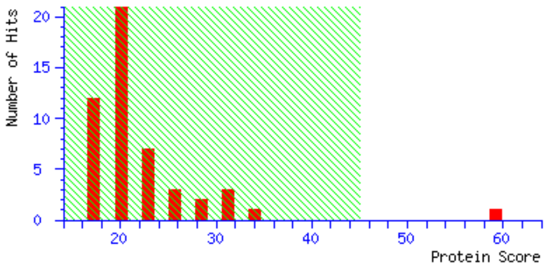

Matched peptide sequences: shown in Bold Red:

```
  1 MSSWNSPYYD TSSYGAGSGG GGGGGRRSSS PPRGAGDKKE TKTKDYQSYT
 51 SNNNNNGSDD DKDKNKHKIT SSHKHKDDEK DRNNHSKDSH GGGNSSNYNK
101 DSYGGNSGNP NNYYGSSTGV VAGSGSYYGG GGGYGGGNTS YGGSLSYGKD
151 GGYGGSNSPY GGGSSIIISG AAPIPHNNFG FYLNRLAGSA AAAGRRVRGG
201 AGVHPRGGGE GHLPPHAARP RLIVVVVLAV AEVRERGRQE EERRRWIRIR
251 KQQRRRRRRV LRPSVPRRRR LHRPVCVN
```

Spot No.: **192**

NCBI accession No.: **gi|255542380** Species: *Ricinus communis*

Protein name: cysteine synthase, putative

PMF Mascot score: **82** Sequence coverage %: **37**

Matched peptides No.: **13** Total peptides No.: **59**

Calculated Mr: **34435** Calculated *p*I: **5.50**

Probability Based Mowse Score:

Protein score is -10*Log(P), where P is the probability that the observed match is a random event. Protein scores greater than 73 are significant (p<0.05).

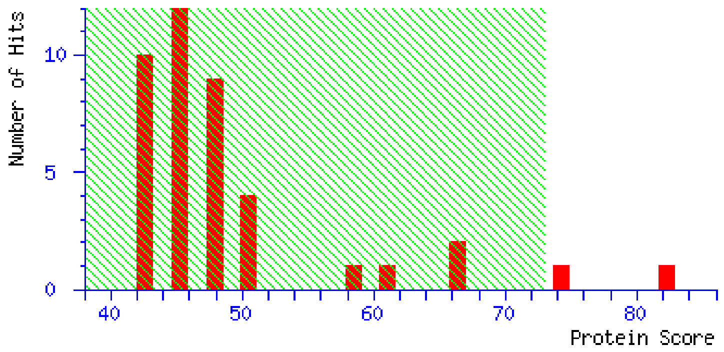

Matched peptide sequences: shown in Bold Red:

```
  1 MVEEKSAIAK DVTELVGKTP LVYLNHVVDG CVARIAAKLE MMEPCSSVKD
 51 RIGYSMITDA EEKGLIKPGE SVLIEPTSGN TGIGLAFMAA AKGYKLIITM
101 PASMSLERRM VLRAFGAELV LTDPARGMKG AVQKAEEILA KTPNSYILQQ
151 FENPANPKIH YETTGPEIWK GSGGKVDFFV SGIGTGGTVT GAGGYLREQN
201 PDIKLIGVEP VESAVLSGGK PGPHKIQGIG AGFIPGVLDV SLLDEVVQIS
251 SEEAIETAKL LALKEGLLVG ISSGAAAAAA IKIARRPENA GKLIVVIFPS
301 FGERYLSSVL FESVKREAES MVFEP
```

Spot No.: **193**

NCBI accession No.: **gi|255562276** Species: *Ricinus communis*

PFF score: [57]

Protein name: pyridoxine kinase, putative

Matched peptides No.: [1] Sequence coverage %: [4]

Matched sequences: K.LYVPPELVAVYR.E

Calculated Mr: **32436** Calculated *p*I: **5.56**

Probability Based Mowse Score:

Ions score is -10*Log(P), where P is the probability that the observed match is a random event.
Individual ions scores > 35 indicate peptides with significant homology.
Individual ions scores > 45 indicate identity or extensive homology (p<0.05).
Protein scores are derived from ions scores as a non-probabilistic basis for ranking protein hits.

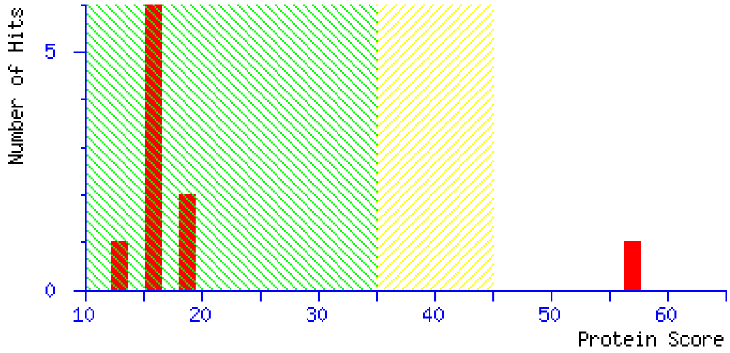

Matched peptide sequences: shown in Bold Red:

```
  1  MAPPILSLAL  PSGTGRVLSI  QSHTVQGYVG  NKSAVFPLQI  LGYDVDPINS
 51  VQFSNHTGYP  SVKGQVLNGQ  QLWDLIEGLE  GNDLLYYTHL  LTGYIGSVSF
101  LNTILEVVNK  LRSINPKLTY  VCDPVMGDEG  KLYVPPELVA  VYREKVVPVA
151  SMLTPNQFEA  EQLTGFRIVT  EKDGREACNT  LHAAGPSKVI  ITSINIDGNL
201  LLVGSHQKEK  DQPPEQFKIE  IPKIPAYFTK  YPDNLGTAAE  LAVSSLQALL
251  QRTVNDYRSV  GYDPQSSSLE  IRLIQSQDDI  RHPQVKFKAE  KYD
```

Spot No.: **194**

NCBI accession No.: **gi|255552291**   Species: *Ricinus communis*

PFF score: [303]

Protein name: short chain dehydrogenase, putative

Matched peptides No.: [3]   Sequence coverage %: [11]

Matched sequences: K.VALVTGGDSGIGR.A;

K.DPIAIPTDVGFEENCR.K;

K.AEGAKDPIAIPTDVGFEENCR.K

Calculated Mr: **32148**   Calculated *p*I: **6.50**

Probability Based Mowse Score:

Ions score is -10*Log(P), where P is the probability that the observed match is a random event.
Individual ions scores > 45 indicate identity or extensive homology (p<0.05).
Protein scores are derived from ions scores as a non-probabilistic basis for ranking protein hits.

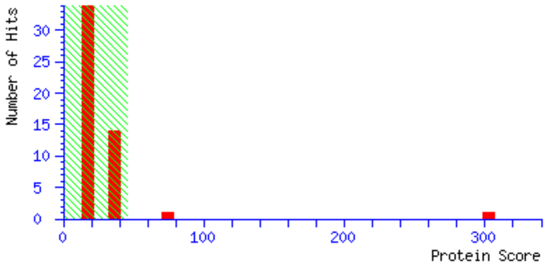

Matched peptide sequences: shown in Bold Red:

```
  1 MATRRGYRFP PQSQDKQPGK EYLMHPLPEF INPHYKPSNK LQDKVALVTG
 51 GDSGIGRAVS YYFTLEGATV AFTYVKGRED KDKDHILKIL HEVKAEGAKD
101 PIAIPTDVGF EENCRKVIDQ IMSEYGKIDI LVNNAGEAHY STTIEDITDP
151 MLERVFRTNI FGHFFMSRHA LKHMKEGGCI INTASVAAYA GFSFMVDYSS
201 TKGAVVAFTR SLALQLIDRG IRVNAVAPGS VWTPFQPSTL SAEQVAQVGS
251 GVPMDRAAQP YEIAPSFVFL ASNDCSSYFT GQVLHPNGMF
```

Spot No.: **195**

NCBI accession No.: gi|255543357 Species: *Ricinus communis*

Protein name: heat shock protein, putative

PMF Mascot score: **102** Sequence coverage %: **27**

Matched peptides No.: **18** Total peptides No.: **61**

Calculated Mr: **71.965** Calculated *p*I: **5.27**

Probability Based Mowse Score:

Protein score is -10*Log(P), where P is the probability that the observed match is a random event.
Protein scores greater than 73 are significant (p<0.05).

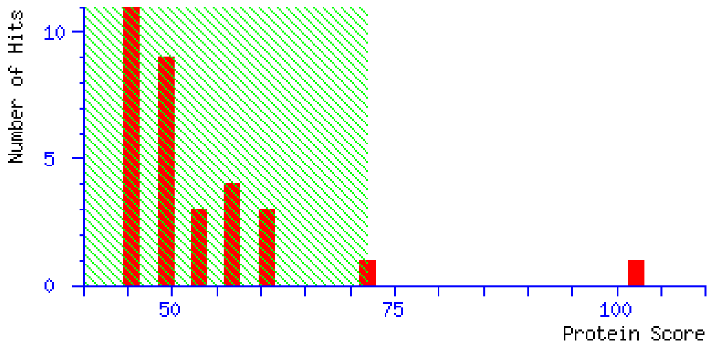

Matched peptide sequences: shown in Bold Red:

```
  1 MAKTEGKAVG IDLGTTYSCV GVWQNDRVEI IANDQGNRTT PSYVAFTDTE
 51 RLIGDAAKNQ VAMNPQNTVF DAKRLIGRRF SDPSVQSDMK HWPFKVVPGP
101 GDKPMIVVLY KGEEKQFAPE EISSMVLIKM KEIAEAYLGQ TVKNAVITVP
151 AYFNDSQRQA TKDAGAISGL NVMRIINEPT AAAIAYGLDK KGSRSGEKNV
201 LIFDLGGGTF DVSLLTIEEG IFEVKATAGD THLGGEDFDN RLVNHFVAEF
251 RRKHKKDIST NARALRRLRT ACERAKRTLS STSQTTIEID SLYEGIDFYS
301 TITRARFEEL NMDLFRKCME PVEKCLRDSK IDKSQVDDIV LVGGSTRIPK
351 VQQLLQDFFN GKELCKSINP DEAVAYGAAV QAAILSGEGD QKVQELLLLD
401 VTPLSLGIET AGGVMTVLIP RNTTIPAKKE QIFSTYSDNQ PGVLIQVYEG
451 ERARTKDNNL LGTFELKGIP PAPRGVPQIN VCFDIDANGI LNVSAEDKTA
501 GVKNKITITN DKGRLSKEDI ERMVQEAEKY KAEDEQVKKK VEAKNALENY
551 AYNMRNTVKD EKFAGKLDSA DKQKIEKAID ETIEWLDGNQ LAEVDEFEDK
601 LKELEGLCNP IVSKMYQGGA GGDVPMGGGA KPGSGYGNAS SGGSGSGPKI
651 EEVD
```

Spot No.: **196**

NCBI accession No.: **gi|224139936** Species: *Populus trichocarpa*

Protein name：predicted protein

PMF score: **82** Sequence coverage %: **15**

Matched peptides No.: **80** Total peptides No.: **22**

Calculated Mr: **68859** Calculated *p*I: **7.90**

Probability Based Mowse Score:

Protein score is -10*Log(P), where P is the probability that the observed match is a random event.
Protein scores greater than 73 are significant (p<0.05).

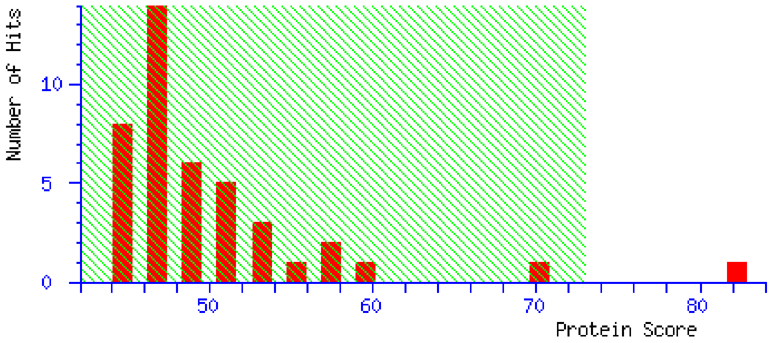

Matched peptide sequences: shown in Bold Red:

1 **MGSMFLNSPL** **PASNK**LIRVS SK**CDWCFLR**W RKRNVVVYCQ GGGGKAIR**SS**
51 **GFPSVLTERS** AVVSDQEHIA SVRDAGSLVL SPNEKGQPEI AVEDFVPYGG
101 PTSSSLLEMQ DGIGIVKFLR GKGLFISGAT GFLAK**VLIEK** **ILRTMPDVGK**
151 IYVLIKAESK EAAITR**LKNE** **IINAELFKCL** **R**QTHGKSYQS FMLNKLVPVV
201 GNVCESNLGL EEDLADKIAN EVDIIVNSAA NTTFDER**YDV** **AIDVNTR**GTC
251 HLMSFAKKCP KLK**LFLQVST** **AYVNGQR**QGR IMEKPFLFGD CIARENLIIS
301 ESTPRFAPAL DIEHEMNLAL DSKEAFQENE VAQKMKELGL ERARK**YGWQD**
351 **TYVFTKAMGE** **MVVDNMRGDI** **PVVIIRPSVI** **ESTCK**EPFPG WMEGNR**MMDP**
401 **IVLYYGK**GQL TGFLVDPNGV LDVVPADMVV NATLAAMAWH GMEQKPDINV
451 YQIASSVVNP LVFQDLATLL HEHYNSSPYM DSNGRPIHVP SMKLFSSMEE
501 FSAHLWRYVT QRSRLAGMAT SDRKLSQKHE NICRKSVEQA KYLASIYEPY
551 TFYGGRFDNS NTQKLMERMS ENEKGIFGFD VGSIDWRDYI TNVHIPGLRR
601 HVMKGRGMCG

Spot No.: **197**

NCBI accession No.: **gi|351727028** Species: *Glycine max*

Protein name: cell division cycle protein 48 homolog

PMF Mascot score: **174** Sequence coverage %: **42**

Matched peptides No.: **37** Total peptides No.: **90**

Calculated Mr: **90512** Calculated *p*I: **5.18**

Probability Based Mowse Score:

Protein score is -10*Log(P), where P is the probability that the observed match is a random event. Protein scores greater than 73 are significant (p<0.05).

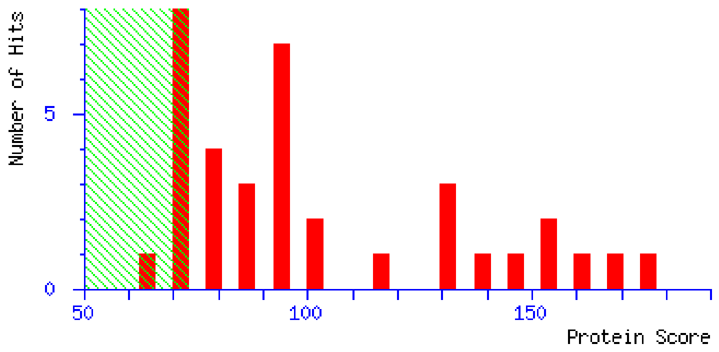

Matched peptide sequences: shown in Bold Red:

1 MSQQGESSDP KSGKK**DFSTA** **ILER**KKSPNR LVVDEAVNDD NSVVTMHPQT

51 MEK**LQLFR**GD TILIKGKKRK DTICIALADE NCEEPKIRMN KVVRSNLRVR

101 **LGDVVSVHQC** **PDVK**YGKRVH ILPIDDTIEG VTGNLFDAFL KPYFLEAYRP

151 VR**KGDLFLVR** GGMRSVEFKV VETDPGEYCV VAPDTEIFCE GEPLKREDEE

201 RLDEVGYDDV GGVR**KQMAQI** **RELVELPLRH** **PQLFK**SIGVK PPK**GILLYGP**

251 **PGSGK**TLIAR AVANETGAFF FCINGPEIMS K**LAGESESNL** **R**KAFEEAEKN

301 APSIIFIDEI DSIAPKREK**T** **HGEVERRIVS** **QLLTLMDGLK** **SRAHVIVIGA**

351 **TNRPNSIDPA** **LRRFGRFDRE** **IDIGVPDEVG** **RLEVLR**IHTK NMKLSDDVDL

401 ERIAK**DTHGY** **VGADLAALCT** **EAALQCIR**EK MDVIDLEDET IDAEVLNSMA

451 VTNEHFQTAL GTSNPSALR**E** **TVVEVPNVSW** **EDIGGLENVK** **RELQETVQYP**

501 **VEHPEKFEK**F GMSPSK**GVLF** **YGPPGCGK**TL LAK**AIANECQ** **ANFISVKGPE**

551 **LLTMWFGESE** **ANVR**EIFDKA R**QSAPCVLFF** **DELDSIATQR** **GSSVGDAGGA**

601 **ADRVLNQLLT** **EMDGMSAK**KT VFIIGATNRP DIIDPALLRP GR**LDQLIYIP**

651 **LPDEDSRHQI** **FK**ACLRKSPI AKNVDLRALA RHTQGFSGAD ITEICQRACK

701 YAIRENIEKD IERERKSREN PEAMDEDTVD DEVAEIKAAH FEESMKFARR

751 **SVSDADIRKY** **QAFAQTLQQS** **R**GFGSEFRFP ESGDRTTTGS DPFAASAGGA

801 DEDDLYS

Spot No.: **198**

NCBI accession No.: **gi|4235430** Species: *Hevea brasiliensis*

Protein name: latex-abundant protein

PMF Mascot score: **180** Sequence coverage %: **76**

Matched peptides No.: **22** Total peptides No.: **58**

Calculated Mr: **46443** Calculated *p*I: **5.01**

Probability Based Mowse Score:

Protein score is -10*Log(P), where P is the probability that the observed match is a random event. Protein scores greater than 73 are significant (p<0.05).

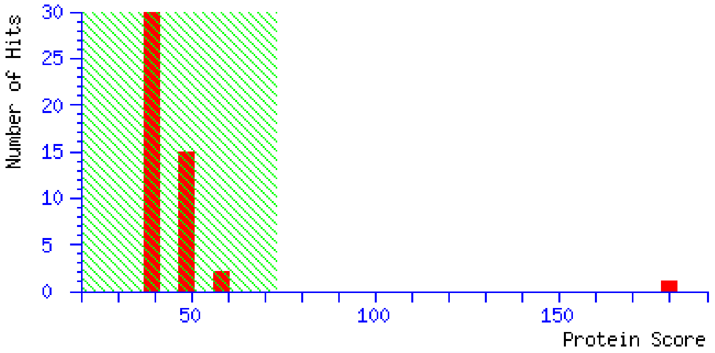

Matched peptide sequences: shown in Bold Red:

```
  1 MAKKAVLIGI NYPGTKAELK GCINDVKRMY RCLVDRYGFS EEDITVLIDI
 51 DESYIQPTGK NIRRVLTDLV RSAEPGDLLF VHYSGHGTRL PAETGEDDDT
101 GFDECIVPCD MNLITDDDFR EFVDQVPHGC RITVVSDSCH SGGLIDEAKE
151 QIGESTKRKE EESESGFGFK SFLKQTIQDA FESRGVHLPS DLHHHHGHRD
201 EEDFDNRVVE EDYGDSGYVK SKSLPLSTLI EILKQKTGKD DIDVGKLRPT
251 LFDMFGDDAS PKVKKFMKVI LNKLRHGDGE SGGGGFLGMV GSLAQEFLKH
301 KLDENDESYV KPALETEVDS KQEVYAGKTK RSLPDGGILI SGCQTDQTSA
351 DASPSGKSSE AYGALSNAIQ TIIAETDGAV TNQELVLKAR KMLKKQGFTQ
401 KPGLYCSDDH VEASFVC
```

Spot No.: **199**

NCBI accession No.: **gi|198400319** Species: *Camellia sinensis*

PFF score: [57]

Protein name: senescence-related protein

Matched peptides No.: [1] Sequence coverage %: [2]

Matched sequences: K.GILWCGDVTVDR.L

Calculated Mr: **48662** Calculated *p*I: **4.96**

Probability Based Mowse Score:

Ions score is -10*Log(P), where P is the probability that the observed match is a random event.
Individual ions scores > 45 indicate identity or extensive homology (p<0.05).
Protein scores are derived from ions scores as a non-probabilistic basis for ranking protein hits.

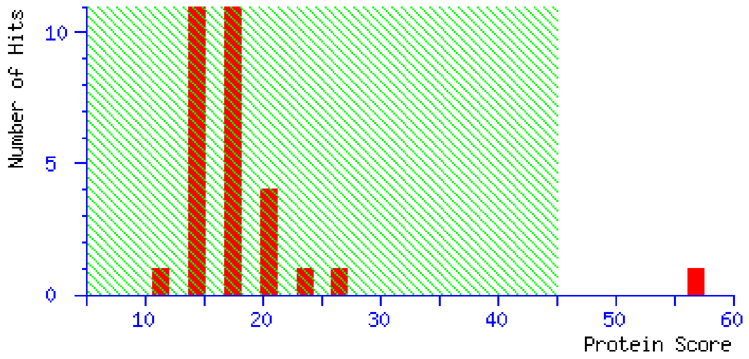

Matched peptide sequences: shown in Bold Red:

1	MSSQNPKNSL	YPQVDQSNPE	AISSSSSSPS	SSTIYPSIDM	KDLAENLFPD
51	NDSQNPNSQS	QLESSEEILI	RVPGTIVHLI	DKEQSVELAC	GELTIVRLLQ
101	GGNVVAVLAR	IDDQIQWPLA	KDEAAVKLDE	SHYFFTLRVP	SEAGFDNEED
151	NDEVNMESEN	LLNYGVTIAS	KGQEGLLEAF	DSILEHYSAF	SVQKVSEAVD
201	GSVVARETSP	EEMESEEEKR	EMMEGSSAAY	WTTLAPNVED	YSGNVARMIA
251	VGSGQLIKG**I**	**LWCGDVTVDR**	LKWGNEFLKK	KLGPASQTKI	SPQAMRRMKR
301	VKLLTKMSEE	VATGILSGVV	KVSGFFTSSI	VNSSVGKKFF	SLLPGEIVLA
351	SLDGFNKVCD	AVEVAGRNVM	STTSVVTTGL	VSQRYGEQAA	KVTNEGFDAA
401	GHAIGIAWAV	FKIRKALNPK	SVIKPTTLAK	AAAETNYAAL	KAKSNKQT

Spot No.: **200**

NCBI accession No.: **gi|255570457** Species: *Ricinus communis*

PFF score: [282]

Protein name: protease C56, putative

Matched peptides No.: [3] Sequence coverage %: [8]

Matched sequences: R.APEYLAMNESVLDCVR.K;

R.APEYLAMNESVLDCVR.K + Oxidation (M);

R.APEYLALDETVIALVK.Q

Calculated Mr: **42437** Calculated *p*I: **5.60**

Probability Based Mowse Score:

Ions score is -10*Log(P), where P is the probability that the observed match is a random event.
Individual ions scores > 43 indicate identity or extensive homology (p<0.05).
Protein scores are derived from ions scores as a non-probabilistic basis for ranking protein hits.

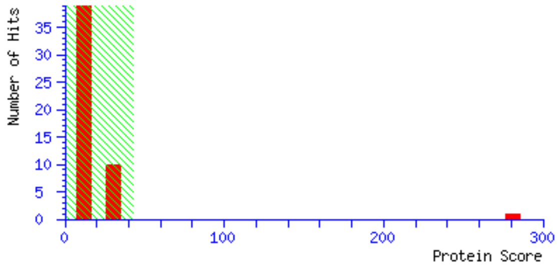

Matched peptide sequences: shown in **Bold Red:**

```
  1 MSHQKTVFLL CGDYMEDYEA MVPFQALLAY GVAVDAACPG KKAGDYCRTA
 51 IHESSGHQTY SESRGHNFTL NSTFDDIDFG KYDGLVIPGG RAPEYLAMNE
101 SVLDCVRKFA DAGKPIASVC HGQLILAAAG LVKGRKCTAY PPVRPVLIAA
151 GAHWVEPETM ASCVADGNLI TGATYEGHPE FIQLFVKALG GSITGADKRI
201 LFLCGDYMED YEVTVPFQSL QALGCHVNAV CPKKKAGDSC PTAVHDFEGD
251 QTYSEKPGHH FTLTASYVGL DVSSYDALVL PGGRAPEYLA LDETVIALVK
301 QFMQSKKPVA SICHGQQILA AAGVLKGKKC TAYPAVKLNV VLSGATWLEP
351 DPIDRCYTDE NLVTGAAWPG HPEFISQLMA LLGIRVSF
```

Spot No.: **201**

NCBI accession No.: **gi|32186896** Species: *Gossypium hirsutum*

Protein name: actin

PMF Mascot score: **149** Sequence coverage %: **57**

Matched peptides No.: **21** Total peptides No.: **38**

Calculated Mr: **41935** Calculated *p*I: **5.44**

Probability Based Mowse Score:

Protein score is -10*Log(P), where P is the probability that the observed match is a random event. Protein scores greater than 73 are significant (p<0.05).

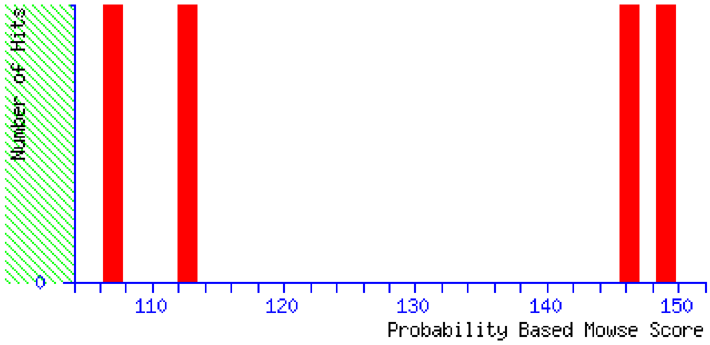

Matched peptide sequences: shown in Bold Red:

```
  1 MADGEDIQPL VCDNGTGMVK AGFAGDDAPR AVFPSIVGRP RHTGVMVGMG
 51 HKDAYVGDEA QSKRGILTLK YPIEHGIVSN WDDMEKIWHH TFYNELRVAP
101 EEHPVLLTEA PLNPKANREK MTHIMFETFN VPAMYVAIQA VLSLYASGRT
151 TGIVLDSGDG VSHTVPIYEG YALPHAILRL DLAGRDLTDA LMKILTERGY
201 MFTTTAEREI VRDMKEKLAY VALDYEQELE TAKSSSSVEK NYELPDGQVI
251 TIGAERFRCP EVLFQPSFIG MEAAGIHETT YNSIMKCDVD IRKDLYGNIV
301 LSGGSTMFPG IADRMSKEIT ALAPSSMKIK VVAPPERKYS VWIGGSILAS
351 LSTFQQMWIS KGEYDESGPS IVHRKCF
```

Spot No.: **202**

NCBI accession No.: **gi|255560725** Species: *Ricinus communis*

Protein name: dead box ATP-dependent RNA helicase, putative

PMF Mascot score: **109** Sequence coverage %: **54**

Matched peptides No.: **21** Total peptides No.: **76**

Calculated Mr: **47097** Calculated *p*I: **5.49**

Probability Based Mowse Score:

Protein score is -10*Log(P), where P is the probability that the observed match is a random event.
Protein scores greater than 73 are significant (p<0.05).

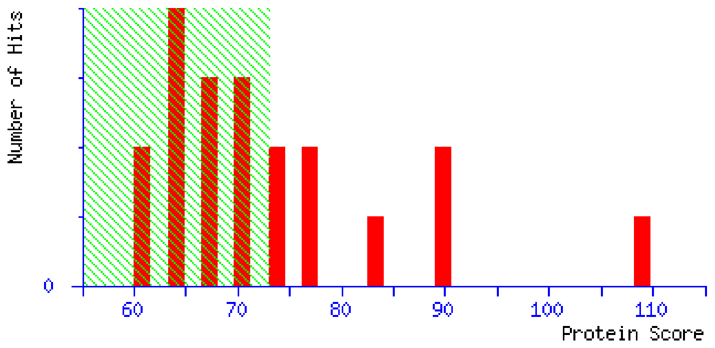

Matched peptide sequences: shown in Bold Red:

```
  1 MAGLAPEGSQ FDAKQYDAKM SELLSTEGQE FFTTYDEVYD SFDAMGLQEN
 51 LLRGIYAYGF EKPSAIQQRG IVPFCKGLDV IQQAQSGTGK TATFCSGILQ
101 QLDYGLVQCQ ALVLAPTREL AQQIEKVMRA LGDYLGVKVH ACVGGTSVRE
151 DQRILQAGVH VVVGTPGRVF DMLRRQSLRP DYIKMFVLDE ADEMLSRGFK
201 DQIYDIFQLL PAKVQVGVFS ATMPPEALEI TRKFMNKPVR ILVKRDELTL
251 EGIKQFYVNV EKEEWKLETL CDLYETLAIT QSVIFVNTRR KVDWLTDKMR
301 SRDHTVSATH GDMDQNTRDI IMREFRSGSS RVLITTDLLA RGIDVQQVSL
351 VINYDLPTQP ENYLHRIGRG GRFGRKGVAI NFVTRDDERM LFDIQKFYNV
401 VIEELPSNVA DLL
```

Spot No.: **203**

NCBI accession No.: **gi|14423688** Species: *Hevea brasiliensis*

PFF score: [229] Protein name: Enolase 1

Matched peptides No.: [3] Sequence coverage %: [10]

Matched sequences: R.AGWGVMASHR.S + Oxidation (M);

R.AAVPSGASTGIYEALELR.D;

R.IEEELGSEAVYAGANFR.K

Calculated Mr: **48029** Calculated *p*I: **5.57**

Probability Based Mowse Score:

Ions score is -10*Log(P), where P is the probability that the observed match is a random event.
Individual ions scores > 45 indicate identity or extensive homology (p<0.05).
Protein scores are derived from ions scores as a non-probabilistic basis for ranking protein hits.

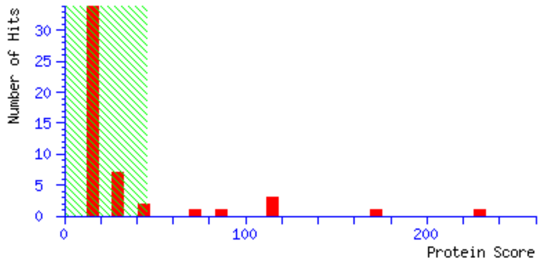

Matched peptide sequences: shown in **Bold Red:**

```
  1 MAITIVSVRA RQIFDSRGNP TVEADVKLSD GYLARAAVPS GASTGIYEAL
 51 ELRDGGSDYL GKGVSKAVEN VNIIIGPALV GKDPTDQVGI DNFMVQQLDG
101 TVNEWGWCKQ KLGANAILAV SLAVCKAGAH VKGIPLYEHI ANLAGNKNLV
151 LPVPAFNVIN GGSHAGNKLA MQEFMILPVG ASSFKEAMKM GAEVYHHLKS
201 VIKKKYGQDA TNVGDEGGFA PNIQENKEGL ELLKTAIAKA GYTGKVVIGM
251 DVAASEFYGS DQTYDLNFKE ENNNGSQKIS GEALKDLYKS FVAEYPIVSI
301 EDPFDQDDWA HYAKLTSEIG EKVQIVGDDL LVTNPKRVEK AIKEKACNAL
351 LLKVNQIGSV TESIEAVKMS KRAGWGVMAS HRSGETEDTF IADLSVGLAT
401 GQIKTGAPCR SERLAKYNQL LRIEEELGSE AVYAGANFRK PVEPY
```

Spot No.: **204**

NCBI accession No.: **gi|186898205** Species: *Hevea brasiliensis*

PFF score: [311] Protein name: senescence-related protein

Matched peptides No.: [10] Sequence coverage %: [13]

Matched sequences: R.LSKEEIER.M;
R.LVNHFVAEFK.G;
R.FEELNMDLFR.K;
R.FEELNMDLFR.K + Oxidation (M);
R.TTPSYVAFTDTER.L;
R.ARFEELNMDLFR.K;
R.ARFEELNMDLFR.K + Oxidation (M);
K.ATAGDTHLGGEDFDNR.L;
K.NAVITVPAYFNDSQR.Q;
R.IINEPTAAAIAYGLDKK.G

Calculated Mr: **72036** Calculated *p*I: **5.27**

Probability Based Mowse Score:

Ions score is -10*Log(P), where P is the probability that the observed match is a random event.
Individual ions scores > 46 indicate identity or extensive homology (p<0.05).
Protein scores are derived from ions scores as a non-probabilistic basis for ranking protein hits.

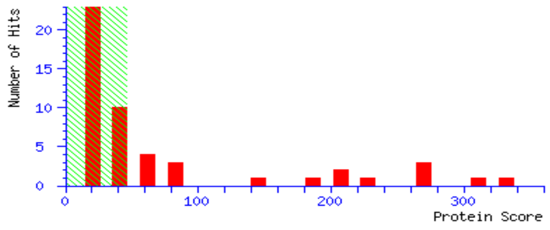

Matched peptide sequences: shown in Bold Red:

```
  1 MAPKTEGKAI GIDLGTTYSC VGVWQNDRVE IIANDQGNRT TPSYVAFTDT
 51 ERLIGDAAKN QVAMNPQNTV FDAKRLIGRR FSDPSVQSDM KHWPFKVISG
101 PGDKPMIVVQ YKGEEKQFAP EEISSMVLTK MKEIAEAYLG QTVKNAVITV
151 PAYFNDSQRQ ATKDAGAIAG VNVMRIINEP TAAAIAYGLD KKGSSTGEKN
201 VLIFDLGGGT FDVSLLTIEE GIFEVKATAG DTHLGGEDFD NRLVNHFVAE
251 FKGKHKKDIS TNARALRRLR TARERAKRTL SSTSQTTIEI DSLYEGIDFY
301 ATITRARFEE LNMDLFRKCM EPVEKCLRDS KIDKSQVHDV VLVGGSTRIP
351 KVQQLLQDIF NGKELCKSIN PDEAVAYGAA VQAAILSGEG DEKVQDLLLL
401 DVTPLSLGIE TAGGVMTVLI PRNTTIPTKK EQIFSTYSDN QPGVLIQVYE
451 GERARTKDNN LLGKFELTGI PPAPRGVPQI NVCFDIDANG IVNVSAEDKT
501 AGVKNKITIT NDKGRLSKEE IERMVQEAEK YKAEDEEVKK KVEAKNSLEN
551 YAYNMRNTVK DDKFAGKLAP ADKGKIEKAI DETIEWLDRN QLAEVEEFED
601 KLKELEGLCN PIIAKMYQGG AGGDVPVGGA EMPNSNYGKT SSGGSGAGPK
651 IEEVD
```

Spot No.: **205**

NCBI accession No.: **gi|255537027** Species: *Ricinus communis*

PFF score: [63]

Protein name: heat shock protein 70 (HSP70)-interacting protein,

Matched peptides No.: [2] Sequence coverage %: [5]

Matched sequences: K.LVNAGIVQIR.-;

K.HDPQNQELLDGVR.R

Calculated Mr: **65386** Calculated *p*I: **5.60**

Probability Based Mowse Score:

Ions score is -10*Log(P), where P is the probability that the observed match is a random event.
Individual ions scores > 45 indicate identity or extensive homology (p<0.05).
Protein scores are derived from ions scores as a non-probabilistic basis for ranking protein hits.

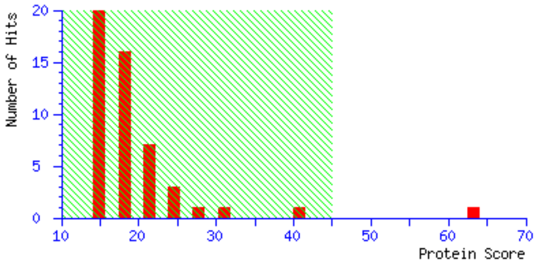

Matched peptide sequences: shown in Bold Red:

1	MAEEAKAKGN	AAFSAGDFTT	AVKHFTEAIS	IAPSNHVLYS	NRSAALASLH
51	NYADALTDAK	KTVELKPDWS	KGYSRLGAAY	LGLNQTQDAI	SAYKKGLDID
101	PSNEALKSGL	ADAQARFSAP	PPPPSPFGDA	FNGPEMWAKL	TADPSTRMYL
151	QQPDFVKMMQ	EIQKNPNNLN	LYLKDQRVMQ	ALGVLLNLKF	RAPNAGAEDM
201	EVPQEDDHSS	SPPPQAEQPA	KKAPQPEPEP	EPMDVITEED	KEAKERKAQA
251	VKEKELGNAA	YKQKDFETAI	SHYTTALELD	DEDISYLTNR	AAVYLEMGKY
301	EDCIKDCDKA	VERGRELRSD	FKMIARALTR	KGTALVKMAK	SSKDYDSAIE
351	TFQKALTEHR	NPETLKKLNE	AERAKKELEQ	QEYFDPKLAD	EEREKGNEYF
401	KQQKYPEAVK	HYTESLRRNP	EDPRAYSNRA	ACYTKLGALP	EGLKDAEKCI
451	ELDPTFTKGY	TRKGAVQFFM	KEYDKALETY	QEGLK**HDPQN**	**QELLDGVR**RC
501	VEQLNKASRG	DLSPEELKER	QAKAMQDPEI	QNILSDPVMR	QVLVDFQENP
551	KSAQDHMKNP	MVMNKIQK**LV**	**NAGIVQIR**		

Spot No.: 206

NCBI accession No.: gi|302853426

Species: *Volvox carteri f. nagariensis*

Protein name: **hypothetical protein VOLCADRAFT_77867**

PMF score: 81 Sequence coverage %: 33

Matched peptides No.:14 Total peptides No.:69

Calculated Mr: 65036 Calculated *p*I:8.96

Probability Based Mowse Score:

Protein score is -10*Log(P), where P is the probability that the observed match is a random event.
Protein scores greater than 73 are significant (p<0.05).

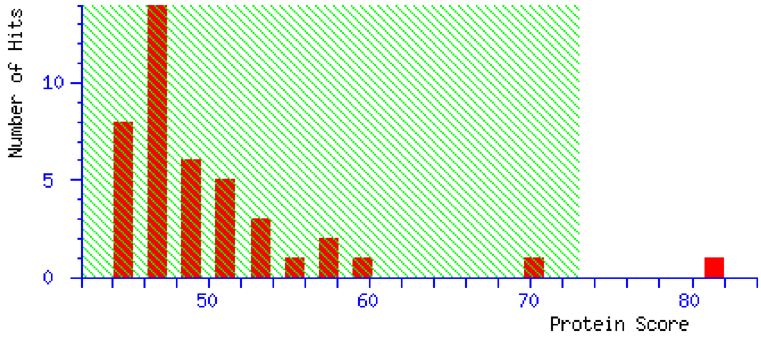

Matched peptide sequences: shown in Bold red:

1 **MIKQQYLGQE** **KIK**KRVSRNG GMGSRCSCRW RRGR**WSLKPI** **VTNTPLTPCS**
51 **EAALMFGRGM** **R**AGIDRREQK KAAASLEAEL LRKARSAAGL VETAETREHD
101 RERKR**LADQY** **DGFDMRIEK**H WTEKSLEEMT ERDWRIFRED FNIGYRGVNT
151 VLPIRKWEES GLPSLLIKAI DRVGYKK**PSP** **IQMAAIPLGL** **QQRDVIGIAE**
201 **TGSGK**TAAFV LPMLSYIMRQ PPMTEENEAD GPYAVVLAPT RELAQQIEEE
251 THKLAYYTNY R**IASVVGGQS** **IEEQGAK**LRK **GCEIVIATPG** **RLLDCIDR**HY
301 AVLNQCNYVV LDEADRMIDL GFEPQVIGVL DAMPSSFLKP DEDGAVLEAN
351 R**TYRTTYMFS** **ATMPPAVERL** **AK**KYLRRPVV VVIGSAGKVT DNVTQRVFVV
401 KENEK**PRVLE** **QEMESVDEK**R VIVFVNTQRQ CDNVHRHLEE LGYRCTILHG
451 GKTQDQREAG IKGFRDGTYN CLIATDVAGR **GIDVPDVALV** **INYDMPNNIE**
501 **NYTHR**IGRTG RAGKKGVAVT FLTLGDTGVF YDLKKLLEES K**AAVPPELAR**
551 **HEASK**LKPGS IEAKSRKDQT VFAA

Spot No.: **207**

NCBI accession No.: **gi|255537027** Species: *Ricinus communis*

PFF score: [266]

Protein name: heat shock protein 70 (HSP70)-interacting protein,

Matched peptides No.: [3] Sequence coverage %: [5]

Matched sequences: K.LVNAGIVQIR.-;
K.NPNNLNLYLK.D;
R.MYLQQPDFVK.M + Oxidation (M);
R.QVLVDFQENPK.S;
K.LGALPEGLKDAEK.C;
K.HDPQNQELLDGVR.R;
K.ALETYQEGLKHDPQNQELLDGVR.R

Calculated Mr: **65386** Calculated *p*I: **5.60**

Probability Based Mowse Score:

Ions score is -10*Log(P), where P is the probability that the observed match is a random event.
Individual ions scores > 45 indicate identity or extensive homology (p<0.05).
Protein scores are derived from ions scores as a non-probabilistic basis for ranking protein hits.

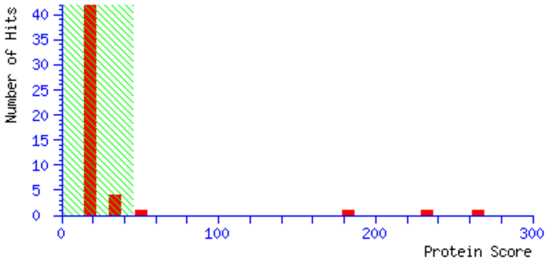

Matched peptide sequences: shown in Bold Red:

```
  1 MAEEAKAKGN AAFSAGDFTT AVKHFTEAIS IAPSNHVLYS NRSAALASLH
 51 NYADALTDAK KTVELKPDWS KGYSRLGAAY LGLNQTQDAI SAYKKGLDID
101 PSNEALKSGL ADAQARFSAP PPPPSPFGDA FNGPEMWAKL TADPSTRMYL
151 QQPDFVKMMQ EIQKNPNNLN LYLKDQRVMQ ALGVLLNLKF RAPNAGAEDM
201 EVPQEDDHSS SPPPQAEQPA KKAPQPEPEP EPMDVITEED KEAKERKAQA
251 VKEKELGNAA YKQKDFETAI SHYTTALELD DEDISYLTNR AAVYLEMGKY
301 EDCIKDCDKA VERGRELRSD FKMIARALTR KGTALVKMAK SSKDYDSAIE
351 TFQKALTEHR NPETLKKLNE AERAKKELEQ QEYFDPKLAD EEREKGNEYF
401 KQQKYPEAVK HYTESLRRNP EDPRAYSNRA ACYTKLGALP EGLKDAEKCI
451 ELDPTFTKGY TRKGAVQFFM KEYDKALETY QEGLKHDPQN QELLDGVRRC
501 VEQLNKASRG DLSPEELKER QAKAMQDPEI QNILSDPVMR QVLVDFQENP
551 KSAQDHMKNP MVMNKIQKLV NAGIVQIR
```

Spot No.: **208**

NCBI accession No.: **gi|255537027** Species: *Ricinus communis*

PFF score: [68]

Protein name: heat shock protein 70 (HSP70)-interacting protein,

Matched peptides No.: [2] Sequence coverage %: [5]

Matched sequences: K.LVNAGIVQIR.-;

K.HDPQNQELLDGVR.R

Calculated Mr: **65386** Calculated *p*I: **5.60**

Probability Based Mowse Score:

Ions score is -10*Log(P), where P is the probability that the observed match is a random event.
Individual ions scores > 45 indicate identity or extensive homology (p<0.05).
Protein scores are derived from ions scores as a non-probabilistic basis for ranking protein hits.

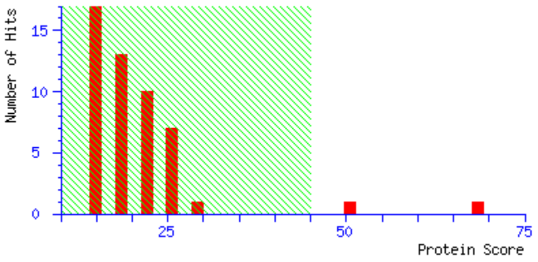

Matched peptide sequences: shown in Bold Red:

1 MAEEAKAKGN AAFSAGDFTT AVKHFTEAIS IAPSNHVLYS NRSAALASLH
51 NYADALTDAK KTVELKPDWS KGYSRLGAAY LGLNQTQDAI SAYKKGLDID
101 PSNEALKSGL ADAQARFSAP PPPPSPFGDA FNGPEMWAKL TADPSTRMYL
151 QQPDFVKMMQ EIQKNPNNLN LYLKDQRVMQ ALGVLLNLKF RAPNAGAEDM
201 EVPQEDDHSS SPPPQAEQPA KKAPQPEPEP EPMDVITEED KEAKERKAQA
251 VKEKELGNAA YKQKDFETAI SHYTTALELD DEDISYLTNR AAVYLEMGKY
301 EDCIKDCDKA VERGRELRSD FKMIARALTR KGTALVKMAK SSKDYDSAIE
351 TFQKALTEHR NPETLKKLNE AERAKKELEQ QEYFDPKLAD EEREKGNEYF
401 KQQKYPEAVK HYTESLRRNP EDPRAYSNRA ACYTKLGALP EGLKDAEKCI
451 ELDPTFTKGY TRKGAVQFFM KEYDKALETY QEGLK**HDPQN** **QELLDGVR**RC
501 VEQLNKASRG DLSPEELKER QAKAMQDPEI QNILSDPVMR QVLVDFQENP
551 KSAQDHMKNP MVMNKIQK**LV** **NAGIVQIR**

Spot No.: **209**

NCBI accession No.: **gi|1346735** Species: *Ricinus communis*

PFF score: [161]

Protein name: 2,3-bisphosphoglycerate-independent phosphoglycerate mutase

Matched peptides No.: [3] Sequence coverage %: [9]

Matched sequences: K.FGHVTFFWNGNR.S;
K.LPSHYLVSPPEIER.T;
K.AHGTAVGLPTEDDMGNSEVGHNALGAGR.I + Oxidation (M)

Calculated Mr: **57374** Calculated *p*I: **5.60**

Probability Based Mowse Score:

Ions score is -10*Log(P), where P is the probability that the observed match is a random event.
Individual ions scores > 45 indicate identity or extensive homology (p<0.05).
Protein scores are derived from ions scores as a non-probabilistic basis for ranking protein hits.

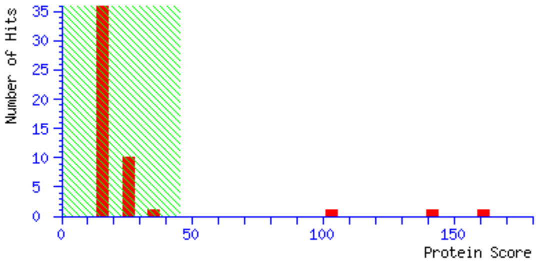

Matched peptide sequences: shown in Bold Red:

```
  1 GEFTWKLADH PKLPKGKTIA MVVLDGWGEA KPDQYNCIHV AETPTMDSFK
 51 KTAPERWRLI KAHGTAVGLP TEDDMGNSEV GHNALGAGRI YAQGAKLVDL
101 ALASGKIYEG EGFKYVKECF DKGTLHLIGL LSDGGVHSRL DQLQLLLKGA
151 AEHGAKRIRV HVLTDGRDVI DGTSVGFAET LEKDLENLRE KGVDAQVASG
201 GGRMYVTMDR YENDWNVVKR GWDAQVLGEA PYKFKSAVEA IKKLREEPKA
251 NDQYLPPFVI VDENGKPVGP IVDGDAVVTI NFRADRMVML AKALEYENFD
301 TFDRVRFPKI HYAGMLQYDG ELKLPSHYLV SPPEIERTSG EYLVHNGVHT
351 FACSETVKFG HVTFFWNGNR SGYFNPEMEE YVEIPSDVGI TFNVQPKMKA
401 IEIAEKARDA ILSGKFQQVR VNIPNGDMVG HTGDVEATVV GCKAADEAVK
451 MIIDAIEQVG GIYVVTADHG NAEDMVKRDK SGKPMADKSG KIQILTSHTL
501 QPVPIAIGGP GLTPGVRFRS DIPTGGLANV AATVMNLHGF EAPSDYEPTL
551 IEAVDN
```

(Matched, shown in bold red: AHGTAVGLP TEDDMGNSEV GHNALGAGR (51–); LPSHYLV SPPEIER (301–); FG HVTFFWNGNR (351–))

Spot No.: **210**

NCBI accession No.: **gi|255537027**   Species: *Ricinus communis*

PFF score: [69]

Protein name: heat shock protein 70 (HSP70)-interacting protein

Matched peptides No.: [3]   Sequence coverage %: [5]

Matched sequences: K.LVNAGIVQIR.-;

K.HDPQNQELLDGVR.R;

K.ALETYQEGLKHDPQNQELLDGVR.R

Calculated Mr: **65386**   Calculated *p*I: **5.60**

Probability Based Mowse Score:

Ions score is -10*Log(P), where P is the probability that the observed match is a random event.
Individual ions scores > 45 indicate identity or extensive homology (p<0.05).
Protein scores are derived from ions scores as a non-probabilistic basis for ranking protein hits.

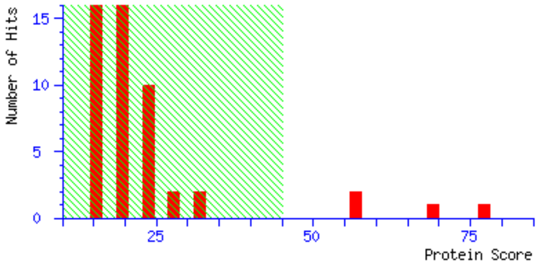

Matched peptide sequences: shown in **Bold Red:**

1 MAEEAKAKGN AAFSAGDFTT AVKHFTEAIS IAPSNHVLYS NRSAALASLH
51 NYADALTDAK KTVELKPDWS KGYSRLGAAY LGLNQTQDAI SAYKKGLDID
101 PSNEALKSGL ADAQARFSAP PPPPSPFGDA FNGPEMWAKL TADPSTRMYL
151 QQPDFVKMMQ EIQKNPNNLN LYLKDQRVMQ ALGVLLNLKF RAPNAGAEDM
201 EVPQEDDHSS SPPPQAEQPA KKAPQPEPEP EPMDVITEED KEAKERKAQA
251 VKEKELGNAA YKQKDFETAI SHYTTALELD DEDISYLTNR AAVYLEMGKY
301 EDCIKDCDKA VERGRELRSD FKMIARALTR KGTALVKMAK SSKDYDSAIE
351 TFQKALTEHR NPETLKKLNE AERAKKELEQ QEYFDPKLAD EEREKGNEYF
401 KQQKYPEAVK HYTESLRRNP EDPRAYSNRA ACYTKLGALP EGLKDAEKCI
451 ELDPTFTKGY TRKGAVQFFM KEYDK**ALETY QEGLKHDPQN QELLDGVR**RC
501 VEQLNKASRG DLSPEELKER QAKAMQDPEI QNILSDPVMR QVLVDFQENP
551 KSAQDHMKNP MVMNKIQK**LV NAGIVQIR**

Spot No.: **211**

NCBI accession No.: gi|255556934 Species: *Ricinus communis*

PFF score: [1185]

Protein name：Transitional endoplasmic reticulum ATPase, putative

Matched peptides No.: [15] Sequence coverage %: [26]

Matched sequences: R.DFSTAILER.K; K.GVLFYGPPGCGK.T;
R.EIDIGVPDEVGR.L; R.LDEVGYDDVGGVR.K;
K.YQAFAQTLQQSR.G; R.LGDVVSVHQCPDVK.Y;
R.KYQAFAQTLQQSR.G; R.LDQLIYIPLPDEDSR.H;
K.YTQGFSGADITEICQR.A; R.EDENRLDEVGYDDVGGVR.K;
R.AHVIVIGATNRPNSIDPALR.R; R.QSAPCVLFFDELDSIATQR.G;
R.ELQETVQYPVEHPEKFEK.F; R.ETVVEVPNVSWEDIGGLENVKR.E;
K.DTHGYVGADLAALCTEAALQCIR.E

Calculated Mr: **90355** Calculated *p*I: **5.13**

Probability Based Mowse Score:

Ions score is -10*Log(P), where P is the probability that the observed match is a random event.
Individual ions scores > 45 indicate identity or extensive homology ($p<0.05$).
Protein scores are derived from ions scores as a non-probabilistic basis for ranking protein hits.

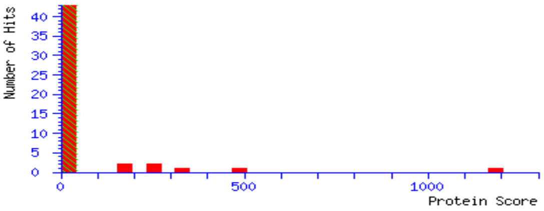

Matched peptide sequences: shown in Bold Red:

1 MSNQAESSDS KGTKR**DFSTA** **ILER**KKSPNR LVVDEAINDD NSVVSLHPET
51 MEKLQLFRGD TILIKGKKRK DTICIALADD SCDEPKIRMN KVVRSNLRVR
101 **LGDVVSVHQC** **PDVK**YGKRVH ILPIDDTIEG VTGNLFDAYL KPYFLEAYRP
151 VRKGDLFLVR GGMRSVEFKV IETDPAEYCV VAPDTEIFCE GEPVRR**EDEN**
201 **RLDEVGYDDV** **GGVR**KQMAQI RELVELPLRH PQLFKSIGVK PPKGILLYGP
251 PGSGKTLIAR AVANETGAFF FCINGPEIMS KLAGESESNL RKAFEEAEKN
301 APSIIFIDEI DSIAPKREKT HGEVERRIVS QLLTLMDGLK SR**AHVIVIGA**
351 **TNRPNSIDPA** **LR**RFGRFDR**E** **IDIGVPDEVG** **R**LEVLRIHTK NMKLAEDVDL
401 ERISK**DTHGY** **VGADLAALCT** **EAALQCIR**EK MDVIDLEDET IDAEILNSMA
451 VSNEHFQTAL GTSNPSALR**E** **TVVEVPNVSW** **EDIGGLENVK** **RELQETVQYP**
501 **VEHPEKFEK**F GMSPSK**GVLF** **YGPPGCGK**TL LAKAIANECQ ANFISVKGPE
551 LLTMWFGESE ANVREIFDKA R**QSAPCVLFF** **DELDSIATQR** GSSVGDAGGA
601 ADRVLNQLLT EMDGMSAKKT VFIIGATNRP DIIDPALLRP GR**LDQLIYIP**
651 **LPDEDSR**HQI FKACLRKSPV SKDVDLRALA K**YTQGFSGAD** **ITEICQR**ACK
701 YAIRENIEKD IERERRRRDN PEAMEEDVED DVAEIKAAHF EESMKYARRS
751 VSDADIR**KYQ** **AFAQTLQQSR** GFGSEFRFSE ATGAAAGADP FAASAGGEAD
801 DDDLYS

Spot No.: **212**

NCBI accession No.: gi|4588474   Species: *Brassica napus*

PFF score: [166]

Protein name：phosphoinositide-specific phospholipase C

Matched peptides No.: [3]   Sequence coverage %: [3]

Matched sequences: K.NDIDVLHGR.T;

R.VIELDIWPNSNK.N;

R.VIELDIWPNSNKNDIDVLHGR.T

Calculated Mr: **66632**   Calculated *p*I: **5.79**

Probability Based Mowse Score:

Ions score is -10*Log(P), where P is the probability that the observed match is a random event.
Individual ions scores > 45 indicate identity or extensive homology (p<0.05).
Protein scores are derived from ions scores as a non-probabilistic basis for ranking protein hits.

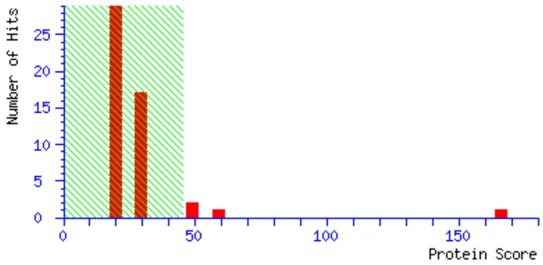

Matched peptide sequences: shown in Bold Red:

```
  1 MSKQTYKVCF CFNRRFRYTA SEAPRDVKTL FDKYSENGVM TVDHLQRFLI
 51 DVQKQDKATK EDAQSIINAA SSLLHSNGLH LDAFFKYLFG DSNPPLALHE
101 VHQDMDAPIS HYFIFTGHNS YLTGNQLSSD CSEVPIIDAL KKGVRVIELD
151 IWPNSNKNDI DVLHGRTLTS PVELIKCLRA IKTHAFEVSD YPVVVTLEDH
201 LTPELQSKVA EMVTEIFGEI LFTPPVGESL KEFPSPNSLK RRIIISTKPP
251 KEYKEGKDED VVQKGKALGD EEVWGREVPS FIERNKSGDK DDLDDEEDND
301 EDDDVEKFKK NAPPQYKHLI AIHAGKPKGS ITACLKVDPD KVRRLSLSEE
351 QLEKAAEKYA KQIVRFTQQN LLRIYPKGTR VTSSNYNPLV GWSHGAQMVA
401 FNMQGYGRSL WLMQGMFRAN GGCGYIKKPD ILLKGGSDSD IFDPKTTLPV
451 KTTLRVTIYM GEGWYFDFRH THFDQYSPPD FYTRVGIAGV PADTVMKKTK
501 TLEDNWVPSW DEVFEFPLTV PELALLRLEV HEYDMSEKDD FGGQTCLPVW
551 ELQEGIRSFP LHNRKEEKYK SVKLLVKVEF V
```

Spot No.: **213**

NCBI accession No.: gi|4588474 Species: *Brassica napus*

PFF score: [116]

Protein name： phosphoinositide-specific phospholipase C

Matched peptides No.: [2] Sequence coverage %: [3]

Matched sequences: K.NDIDVLHGR.T;

R.VIELDIWPNSNKNDIDVLHGR.T

Calculated Mr: **66632** Calculated *p*I: **5.79**

Probability Based Mowse Score:

Ions score is -10*Log(P), where P is the probability that the observed match is a random event.
Individual ions scores > 45 indicate identity or extensive homology (p<0.05).
Protein scores are derived from ions scores as a non-probabilistic basis for ranking protein hits.

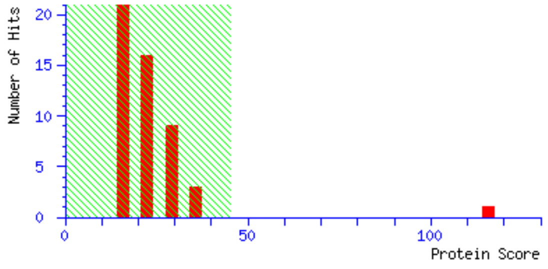

Matched peptide sequences: shown in Bold Red:

1 MSKQTYKVCF CFNRRFRYTA SEAPRDVKTL FDKYSENGVM TVDHLQRFLI
51 DVQKQDKATK EDAQSIINAA SSLLHSNGLH LDAFFKYLFG DSNPPLALHE
101 VHQDMDAPIS HYFIFTGHNS YLTGNQLSSD CSEVPIIDAL KKGVR**VIELD**
151 **IWPNSNKNDI** **DVLHGR**TLTS PVELIKCLRA IKTHAFEVSD YPVVVTLEDH
201 LTPELQSKVA EMVTEIFGEI LFTPPVGESL KEFPSPNSLK RRIIISTKPP
251 KEYKEGKDED VVQKGKALGD EEVWGREVPS FIERNKSGDK DDLDDEEDND
301 EDDDVEKFKK NAPPQYKHLI AIHAGKPKGS ITACLKVDPD KVRRLSLSEE
351 QLEKAAEKYA KQIVRFTQQN LLRIYPKGTR VTSSNYNPLV GWSHGAQMVA
401 FNMQGYGRSL WLMQGMFRAN GGCGYIKKPD ILLKGGSDSD IFDPKTTLPV
451 KTTLRVTIYM GEGWYFDFRH THFDQYSPPD FYTRVGIAGV PADTVMKKTK
501 TLEDNWVPSW DEVFEFPLTV PELALLRLEV HEYDMSEKDD FGGQTCLPVW
551 ELQEGIRSFP LHNRKEEKYK SVKLLVKVEF V

Spot No.: 215

NCBI accession No.: gi|32967699　　Species: *Arabidopsis thaliana*

PFF score: [289]

Protein name：S-adenosyl-L-homocystein hydrolase

Matched peptides No.: [4]　　Sequence coverage %: [10]

Matched sequences: R.ITIKPQTDR.W;
K.SKFDNLYGCR.H;
K.GETLQEYWWCTER.A;
R.WVFPDTKSGIIVLAEGR.L

Calculated Mr: **53776**　　Calculated *p*I: **5.66**

Probability Based Mowse Score:

Ions score is -10*Log(P), where P is the probability that the observed match is a random event.
Individual ions scores > 46 indicate identity or extensive homology (p<0.05).
Protein scores are derived from ions scores as a non-probabilistic basis for ranking protein hits.

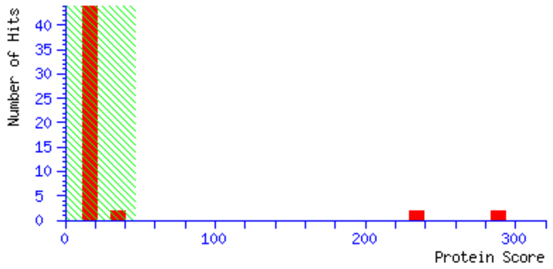

Matched peptide sequences: shown in **Bold Red:**

1	MALIVEKTSG	GREYKVKDMS	QADFGRLELE	LAEVEMPGLM	ACRTEFGPAQ
51	PFKGARITGS	LHMTIQTAVL	IETLTALGAE	VRWCSCNIFS	TQDHAAAAIA
101	RDSAAVFAWK	**GETLQEYWWC**	**TER**ALDWGPG	GGPDLIVDDG	GDATLLIHEG
151	VKAEEIFEKT	GQVPDPTSTD	NPEFQIVLSI	IKEGLQVDPK	KYHKMKGRLV
201	GVSEETTTGV	KRLYQMQESG	ALLFPAINVN	DSVTK**SKFDN**	**LYGCR**HSLPD
251	GLMRATDVMI	AGKVAVICGY	GDVGKGCAAA	MKTAGARVIV	TEIDPICALQ
301	AMMEGLQVLT	LEDVVSEADI	FVTTTGNKDI	IMVDHMRKMK	NNAIVCNIGH
351	FDNEIDMQGL	ETFPGVKR**IT**	**IKPQTDRWVF**	**PDTKSGIIVL**	**AEGR**LMNLGC
401	ATGHPSFVMS	CSFTNQVIAQ	LELWNEKSSG	KYEKKVYVLP	KHLDEKVAAL
451	HLGKLGAKLT	KLTKDQSDYV	SIPIEGPYKP	PHYRY	

Spot No.: **216**

NCBI accession No.: gi|336390551 Species: *Glycine max*

PFF score: [327]

Protein name： adenosylhomocysteinase

Matched peptides No.: [7] Sequence coverage %: [21]

Matched sequences: R.HSLPDGLMR.A + Oxidation (M);
R.ITIKPQTDR.W;
K.SKFDNLYGCR.H;
K.GETLQEYWWCTER.A;
R.WVFPETNTGIIVLAEGR.L;
R.WCSCNIFSTQDHAAAAIAR.D;
R.ALDWGPGGGPDLIVDDGGDATLLIHEGVK.A

Calculated Mr: **53761** Calculated *p*I: **5.69**

Probability Based Mowse Score:

Ions score is -10*Log(P), where P is the probability that the observed match is a random event.
Individual ions scores > 45 indicate identity or extensive homology (p<0.05).
Protein scores are derived from ions scores as a non-probabilistic basis for ranking protein hits.

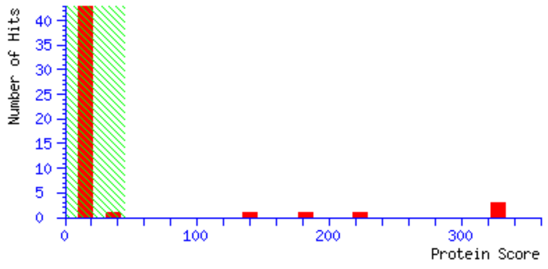

Matched peptide sequences: shown in Bold Red:

1 MALLVEKTTS GREYKVKDLS QADFGRLEIE LAEVEMPGLM ACRTEFGPSQ
51 PFKGARITGS LHMTIQTAVL IETLTALGAE VR**WCSCNIFS** **TQDHAAAAIA**
101 **R**DSAAVFAWK **GETLQEYWWC** **TERALDWGPG** **GGPDLIVDDG** **GDATLLIHEG**
151 **VK**AEELYEKT GELPDPNSTD NAEFQIVLTI IRDGLKTDPT RYRKMKERLV
201 GVSEETTTGV KRLYQMQANG TLLFPAINVN DSVTK**SKFDN** **LYGCRHSLPD**
251 **GLMR**ATDVMI AGKVAVVAGY GDVGKGCAAA MKQAGARVIV TEIDPICALQ
301 ALMEGLQVLT LEDVVSEADI FVTTTGNKDI IMVDHMRKMK NNAIVCNIGH
351 FDNEIDMLGL ENYPGVKR**IT** **IKPQTDRWVF** **PETNTGIIVL** **AEGR**LMNLGC
401 ATGHPSFVMS CSFTNQVIAQ LGLWKEKSIG KYEKKVYVLP KHLDEKVAAL
451 HLGKLGAKLT QLSKSQADYI SVPVEGPYKP AHYRY

Spot No.: **217**

NCBI accession No.: gi|6136111   Species: *Hordeum vulgare*

PFF score: [52]

Protein name: UTP--glucose-1-phosphate uridylyltransferase

Matched peptides No.: [1]   Sequence coverage %: [2]

Matched sequences: R.FFEKAIGINVPR.S

Calculated Mr: **51783**   Calculated *p*I: **5.20**

Probability Based Mowse Score:

Ions score is -10*Log(P), where P is the probability that the observed match is a random event.
Individual ions scores > 46 indicate identity or extensive homology (p<0.05).
Protein scores are derived from ions scores as a non-probabilistic basis for ranking protein hits.

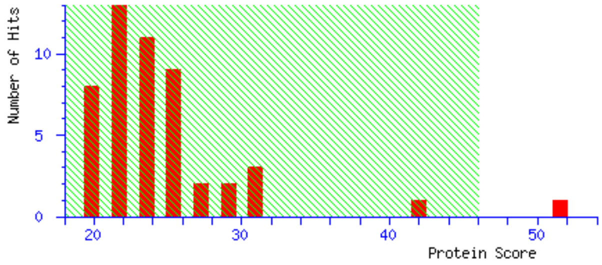

Matched peptide sequences: shown in Bold Red:

```
  1 MAAAAVAADS KIDGLRDAVA KLGEISENEK AGFISLVSRY LSGEAEQIEW
 51 SKIQTPTDEV VVPYDTLAPP PEDLDAMKAL LDKLVVLKLN GGLGTTMGCT
101 GPKSVIEVRN GFTFLDLIVI QIESLNKKYG CSVPLLLMNS FNTHDDTQKI
151 VEKYSNSNIE IHTFNQSQYP RIVTEDFLPL PSKGQTGKDG WYPPGHGDVF
201 PSLNNSGKLD TLLSQGKEYV FVANSDNLGA IVDIKILNHL IHNQNEYCME
251 VTPKTLADVK GGTLISYEGR VQLLEIAQVP DEHVDEFKSI EKFKIFNTNN
301 LWVNLKAIKR LVDAEALKME IIPNPKEVDG VKVLQLETAA GAAIRFFEKA
351 IGINVPRSRF LPVKATSDLL LVQSDLYTLV DGYVIRNPAR VKPSNPSIEL
401 GPEFKKVANF LARFKSIPSI VELDSLKVSG DVSFGSGVVL KGNVTIAAKA
451 GVKLEIPDGA VLENKDINGP EDI
```

(Matched peptide in bold red: FFEKA / IGINVPR, residues 346–357)

Spot No.: 218

NCBI accession No.: gi|14916100 Species: *Hevea brasiliensis*

PFF score: [802]

Protein name: hydroxymethylglutaryl coenzyme A

Matched peptides No.: [10] Sequence coverage %: [35]

Matched sequences: R.LVFNDFVR.N;
K.ARHELPPEK.F;
K.TFLMQIFEK.F + Oxidation (M);
K.VSQQVAKPLYDAK.V;
K.LAPFSNLSGDESYQNR.D;
R.YGLVVCTDSAVYAEGPAR.P;
K.QFSISDAEYFVFHSPYNK.L;
K.DCSLLASGTYYLTEVDSLYR.R;
R.GSHMSHAYDFYKPNLASEYPVVDGK.L + Oxidation (M);
K.NVGILAVDIYFPPTFVQQEALEAHDGASK.G

Calculated Mr: **51761** Calculated *p*I: **6.86**

Probability Based Mowse Score:

Ions score is -10*Log(P), where P is the probability that the observed match is a random event.
Individual ions scores > 45 indicate identity or extensive homology (p<0.05).
Protein scores are derived from ions scores as a non-probabilistic basis for ranking protein hits.

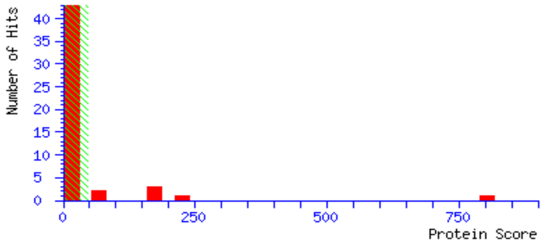

Matched peptide sequences: shown in Bold Red:

1 MAKNVGILAV DIYFPPTFVQ QEALEAHDGA SKGKYTIGLG QDCMAFCTEV
51 EDVISMSLTA VTSLLDKYNI DPKQIGRLEV GSETVIDKSK SIKTFLMQIF
101 EKFGNTDIEG VDSTNACYGG TAALFNCVNW VESSSWDGRY GLVVCTDSAV
151 YAEGPARPTG GAAAIAILVG PDAPIAFESK FRGSHMSHAY DFYKPNLASE
201 YPVVDGKLSQ TCYLMALDSC YKHFCAKYEK FEGKQFSISD AEYFVFHSPY
251 NKLVQKSFAR LVFNDFVRNA RSIDETAKEK LAPFSNLSGD ESYQNRDLEK
301 VSQQVAKPLY DAKVKPTTLI PKQVGNMYTA SLYAAFASLL HSKHTELAGK
351 RVTLFSYGSG LTATMFSLRL HEGQHPFSLS NIASVMNVAG KLKARHELPP
401 EKFVNIMKLM EHRYGAKDFV RSKDCSLLAS GTYYLTEVDS LYRRFYAQKA
451 VGNTVENGLL ANGH

Spot No.: **219**

NCBI accession No.: **gi|307707110** Species: *Prunus armeniaca*

PFF score: [181]

Protein name： NAD-dependent malate dehydrogenase

Matched peptides No.: [4] Sequence coverage %: [19]

Matched sequences: R.LNVQVSDVK.N;

K.VLVVANPANTNALILK.E;
R.KLSSALSAASSACDHIR.D;
K.NVIIWGNHSSTQYPDVNHATVK.T

Calculated Mr: **34812** Calculated *p*I: **5.92**

Probability Based Mowse Score:

Ions score is -10*Log(P), where P is the probability that the observed match is a random event.
Individual ions scores > 45 indicate identity or extensive homology (p<0.05).
Protein scores are derived from ions scores as a non-probabilistic basis for ranking protein hits.

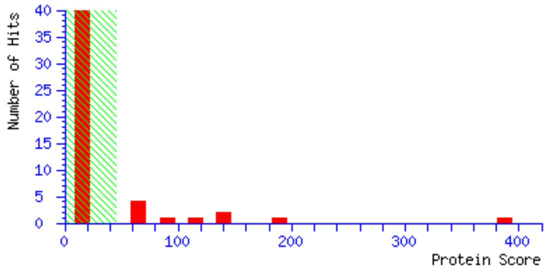

Matched peptide sequences: shown in Bold Red:

```
  1 VTGAAGQIGY ALVPMIARGV MLGADQPVIL HLLDIPPAAE ALNGVKMELV
 51 DAAFPLLKGV VATTDVVEAC TGVNIAVMVG GFPRKEGMER KDVMSKNVSI
101 YKSQASALEK HAAANCKVLV VANPANTNAL ILKEFAPSIP EKNITCLTRL
151 DHNRALGQVS ERLNVQVSDV KNVIIWGNHS STQYPDVNHA TVKTPSGEKA
201 VRELVADDAW LTGEFITTVQ QRGAAIIKAR KLSSALSAAS SACDHIRDWV
251 LGTPEGTWVS MGVYSDGSYN VPSGLIYSFP VTCQNGEWKN VQGLSIDEFS
301 RKKLDATADE LSEEKALAYS CLS
```

Spot No.: **220**

NCBI accession No.: **gi|357137038**

Species: *Brachypodium distachyon*

PFF score: [357]

Protein name： phosphoglycerate kinase, cytosolic-like

Matched peptides No.: [6]　　　　Sequence coverage %: [20]

Matched sequences: K.KPFAAIVGGSK.V;
K.ELDYLVGAVANPK.K;
K.GVTTIIGGGDSVAAVEK.A;
K.LASVADLYVNDAFGTAHR.A;
K.KLASVADLYVNDAFGTAHR.A;
K.VVPASAIPDGWMGLDVGPDAIK.T + Oxidation (M)

Calculated Mr: **42207**　　　　Calculated *p*I: **5.64**

Probability Based Mowse Score:

Ions score is -10*Log(P), where P is the probability that the observed match is a random event.
Individual ions scores > 45 indicate identity or extensive homology (p<0.05).
Protein scores are derived from ions scores as a non-probabilistic basis for ranking protein hits.

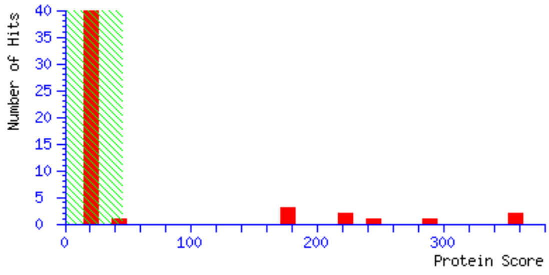

Matched peptide sequences: shown in **Bold Red:**

```
  1 MATKRSVGTL GEADLKGKKV FVRADLNVPL DDAQKITDDT RIRASVPTIK
 51 FLLEKGAKVI LASHLGRPKG VTPKFSLKPL VPRLSELLGI DVVMANDCIG
101 EEVEKLAAAL PDGGVLLLEN VRFYKEEEKN DPEFAKKLAS VADLYVNDAF
151 GTAHRAHAST EGVTKFLRPS VAGFLMQKEL DYLVGAVANP KKPFAAIVGG
201 SKVSSKIGVI ESLLAKVDIL ILGGGMIFTF YKAQGLAVGK SLVEEDKLEL
251 ATSLIETAKA KGVKLLLPID VVVADKFAAD AESKVVPASA IPDGWMGLDV
301 GPDAIKTFSE ALDTCKTIIW NGPMGVFEFE KFAAGTDAIA KQLAELTAKG
351 VTTIIGGGDS VAAVEKAGLA DKMSHISTGG GASLELLEGK PLPGVLALDE
401 A
```

Spot No.: **221**

NCBI accession No.: **gi|357514973** Species: *Medicago truncatula*

PFF score: [99]

Protein name: **Annexin D4**

Matched peptides No.: [2] Sequence coverage %: [3]

Matched sequences: K.LLVALVSAYR.Y;

R.KLLVALVSAYR.Y

Calculated Mr: **36283** Calculated *p*I: **8.64**

Probability Based Mowse Score:

Ions score is -10*Log(P), where P is the probability that the observed match is a random event.
Individual ions scores > 45 indicate identity or extensive homology (p<0.05).
Protein scores are derived from ions scores as a non-probabilistic basis for ranking protein hits.

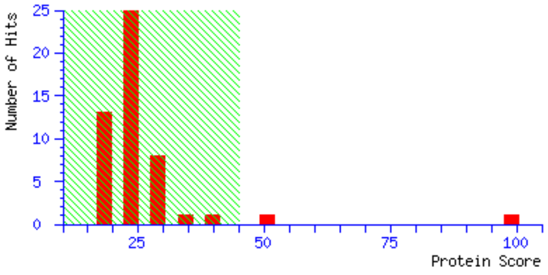

Matched peptide sequences: shown in Bold Red:

```
  1 MAFNQELEAI TQAFSGHGVD EKSLIAVLGK WDPLERETYR KKTSHFFIED
 51 HERQFQRWND HCVRLLKHEF VRFKNAVVLW SMHPWERDAR LAKEALKKGS
101 ISYGVLIEIA CTRSSEELLG ARKAYHSLFD HSIEEDVASH IHGNDRKLLV
151 ALVSAYRYEG TKVKDDTAKS EAKTLSNAIK NAQNKPIVED DEVIRILATR
201 SKLHLQAVYK HYKEISGKNL EEDLNDLRFK ETVQCLCTPQ VYFSKVLDAA
251 LKNDVNKNIK KSLTRVIVTR ADIDMKEIKA EYNNLYGVSL PQKIEETAKG
301 NYKDFLLTLI ARGG
```

Spot No.: 222

NCBI accession No.: gi|21536853 Species: *Arabidopsis thaliana*

PFF score: [314]

Protein name: phosphoglycerate kinase, putative

Matched peptides No.: [6] Sequence coverage %: [17]

Matched sequences: K.YSLKPLVPR.L;

K.ELDYLVGAVANPK.K;

K.YLKPSVAGFLMQK.E + Oxidation (M);

K.GVSLLLPTDVVIADK.F;

K.LAALADVYVNDAFGTAHR.A;

K.KLAALADVYVNDAFGTAHR.A

Calculated Mr: **42178** Calculated *p*I: **5.49**

Probability Based Mowse Score:

Ions score is -10*Log(P), where P is the probability that the observed match is a random event.
Individual ions scores > 45 indicate identity or extensive homology (p<0.05).
Protein scores are derived from ions scores as a non-probabilistic basis for ranking protein hits.

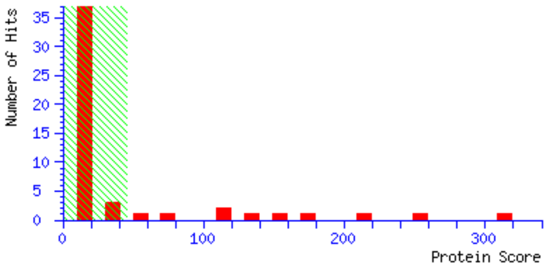

Matched peptide sequences: shown in Bold Red:

```
  1 MATKRSVGTL KEADLKGKSV FVRVDLNVPL DDNSNITDDT RIRAAVPTIK
 51 YLMGNGSRVV LCSHLGRPKG VTPKYSLKPL VPRLSELLGV EVVMANDSIG
101 EEVQKLVAGL PEGGVLLLEN VRFYAEEEKN DPEFAKKLAA LADVYVNDAF
151 GTAHRAHAST EGVAKYLKPS VAGFLMQKEL DYLVGAVANP KKPFAAIVGG
201 SKVSTKIGVI ESLLNTVDIL LLGGGMIFTF YKAQGLSVGS SLVEEDKLDL
251 AKSLMEKAKA KGVSLLLPTD VVIADKFAPD ANSKIVPATA IPDGWMGLDI
301 GPDSIKTFSE ALDTTKTIIW NGPMGVFEFD KFAAGTEAVA KQLAELSGKG
351 VTTIIGGGDS VAAVEKVGLA DKMSHISTGG GASLELIEGK PLPGVLALDE
401 A
```

Spot No.: 223

NCBI accession No.: gi|255547137 Species: *Ricinus communis*

PFF score: [77]

Protein name： Alpha-1,4-glucan-protein synthase

Matched peptides No.: [2] Sequence coverage %: [5]

Matched sequences: K.DINALEQHIK.N;

K.VICDHLGFGVK.T

Calculated Mr: **41557** Calculated *p*I: **5.82**

Probability Based Mowse Score:

Ions score is -10*Log(P), where P is the probability that the observed match is a random event.
Individual ions scores > 46 indicate identity or extensive homology (p<0.05).
Protein scores are derived from ions scores as a non-probabilistic basis for ranking protein hits.

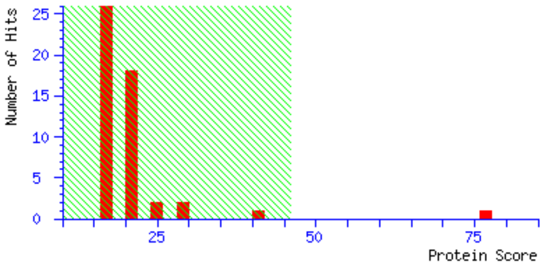

Matched peptide sequences: shown in Bold Red:

```
  1 MADFAAISPT PLLKDELDIV IPTIRNLDFL EMWRPFFQPY HLIIVQDGDP
 51 SKIIKVPEGF DYELYNRNDI NRILGPKASC ISFKDSACRC FGYMVSKKKY
101 IYTIDDDCFV AKDPSGKDIN ALEQHIKNLL CPSTPFFFNT LYDPYRTGAD
151 FVRGYPFSLR EGVPTAVSHG LWLNIPDYDA PTQLVKPLER NTRYVDAVMT
201 IPKGTLFPMC GMNLAFNREL IGPAMYFGLM GDGQPIGRYD DMWAGWCTKV
251 ICDHLGFGVK TGLPYIYHSK ASNPFVNLKK EYKGIYWQEE LIPFFQSAVL
301 PKECTTVQKC YIELSKQVRA KLGKIDEYFV KLADAMVTWV EAWDELNPSG
351 NSGELPNGAA K
```

Spot No.: 224

NCBI accession No.: gi|380468126   Species: *Hevea brasiliensis*

PFF score: [922]

Protein name： caffeic acid 3-O-methyltransferase

Matched peptides No.: [9]   Sequence coverage %: [42]

Matched sequences: R.LYAPGPICK.F;
K.NPEAPVLLDR.M;
R.TRQEFEALASK.S;
K.WVLHDWNDDLCLK.L;
R.AYGMNQFEYPGTDQR.F + Oxidation (M);
K.SWYHLNEAILEGGTPFNR.A;
K.VIVVESILPTVPENNVTSQVLHK.E;
K.SALELNVIDIISTAGNSGASLSAPEIAQR.I;
K.GINYDLPHVLADAPSYPGVEHVAGDMFK.S + Oxidation (M)

Calculated Mr: **40957**   Calculated *p*I: **5.54**

Probability Based Mowse Score:

Ions score is -10*Log(P), where P is the probability that the observed match is a random event.
Individual ions scores > 45 indicate identity or extensive homology (p<0.05).
Protein scores are derived from ions scores as a non-probabilistic basis for ranking protein hits.

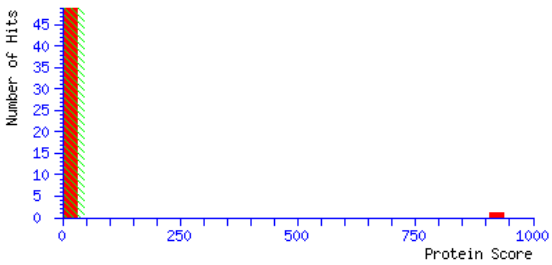

Matched peptide sequences: shown in Bold Red:

```
  1 MTGSEQSNSA SIMSGRSDEE TWNLAIDLAN TVILPMVLKS ALELNVIDII
 51 STAGNSGASL SAPEIAQRIP EAKNPEAPVL LDRMLRLLAT YDIVKCSSNT
101 KENGEVERLY APGPICKFLT KNKNGSGSAA PLLLLHHDEV FMKSWYHLNE
151 AILEGGTPFN RAYGMNQFEY PGTDQRFNRV FNDAMSSYTT YLVKKILDAY
201 KGFDGLKSLV DVGGNSGVTL NSITSKYPHI KGINYDLPHV LADAPSYPGV
251 EHVAGDMFKS VPKGDAIILK WVLHDWNDDL CLKLLKNCWE ALPSNGKVIV
301 VESILPTVPE NNVTSQVLHK EDLMLLSFNV GGKERTRQEF EALASKSGFS
351 SCEFICCAYN SWVIEFHK
```

Spot No.: 225

NCBI accession No.: gi|255570457 Species: *Ricinus communis*

PFF score: [237]

Protein name： protease C56, putative

Matched peptides No.: [3] Sequence coverage %: [10]

Matched sequences: K.YDGLVIPGGR.A;

R.APEYLALDETVIALVK.Q;

R.APEYLAMNESVLDCVR.K + Oxidation (M)

Calculated Mr: **42437** Calculated *p*I: **5.60**

Probability Based Mowse Score:

Ions score is -10*Log(P), where P is the probability that the observed match is a random event.
Individual ions scores > 45 indicate identity or extensive homology (p<0.05).
Protein scores are derived from ions scores as a non-probabilistic basis for ranking protein hits.

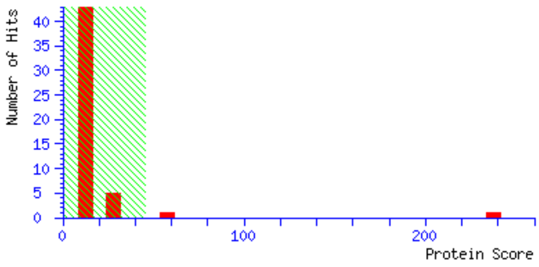

Matched peptide sequences: shown in Bold Red:

```
  1 MSHQKTVFLL CGDYMEDYEA MVPFQALLAY GVAVDAACPG KKAGDYCRTA
 51 IHESSGHQTY SESRGHNFTL NSTFDDIDFG KYDGLVIPGG RAPEYLAMNE
101 SVLDCVRKFA DAGKPIASVC HGQLILAAAG LVKGRKCTAY PPVRPVLIAA
151 GAHWVEPETM ASCVADGNLI TGATYEGHPE FIQLFVKALG GSITGADKRI
201 LFLCGDYMED YEVTVPFQSL QALGCHVNAV CPKKKAGDSC PTAVHDFEGD
251 QTYSEKPGHH FTLTASYVGL DVSSYDALVL PGGRAPEYLA LDETVIALVK
301 QFMQSKKPVA SICHGQQILA AAGVLKGKKC TAYPAVKLNV VLSGATWLEP
351 DPIDRCYTDE NLVTGAAWPG HPEFISQLMA LLGIRVSF
```

Spot No.: 226

NCBI accession No.: gi|224131618 Species: *Populus trichocarpa*

PFF score: [172]

Protein name：predicted protein

Matched peptides No.: [2] Sequence coverage %: [18]

Matched sequences: K.ASGIPYEVHIYPGNAHAFMNR.S + Oxidation (M);

K.APVQAHFGELDNFVGFSDVTAAK.A

Calculated Mr: **26246** Calculated *p*I: **5.24**

Probability Based Mowse Score:

Ions score is -10*Log(P), where P is the probability that the observed match is a random event.
Individual ions scores > 45 indicate identity or extensive homology (p<0.05).
Protein scores are derived from ions scores as a non-probabilistic basis for ranking protein hits.

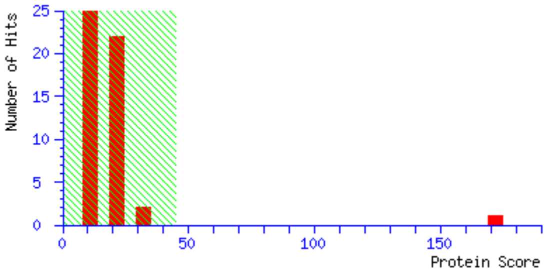

Matched peptide sequences: shown in Bold Red:

1 MAGSASQPFK KIQIQRDDTT FDAYVVGKED APGIVVLQEW WGVDFEIKNH
51 AVKISQLGPG FKALIPDLYR GKVGLDVAEA QHLMDGLDWQ GAVKDIQASV
101 NWLKTNGSSK AGVTGFCMGG ALSIASSVLV PEVDAVVAFY GVPSSQLADP
151 AQAK**APVQAH FGELDNFVGF SDVTAAK**ALE EKLK**ASGIPY EVHIYPGNAH**
201 **AFMNR**SPEGV MRRKGMGLPD EDEASAELAW SRFTTWMTRY LSA

Spot No.: 227

NCBI accession No.: gi|333690873   Species: *Hevea brasiliensis*

PFF score: [386]

Protein name: glutathione-S-transferase tau 1

Matched peptides No.: [8]   Sequence coverage %: [29]

Matched sequences: R.FWADFIDK.K;
K.VYEFVLVLK.K;
R.FWADFIDKK.I;
K.YEYREEDLR.N;
K.SPFLPSDPYQR.A;
K.SPLLLQMNPVHK.K + Oxidation (M);
K.DKSPFLPSDPYQR.A;
K.ESVSESVPDPEKVYEFVLVLK.K

Calculated Mr: **25595**   Calculated *p*I: **5.48**

Probability Based Mowse Score:

Ions score is -10*Log(P), where P is the probability that the observed match is a random event.
Individual ions scores > 46 indicate identity or extensive homology (p<0.05).
Protein scores are derived from ions scores as a non-probabilistic basis for ranking protein hits.

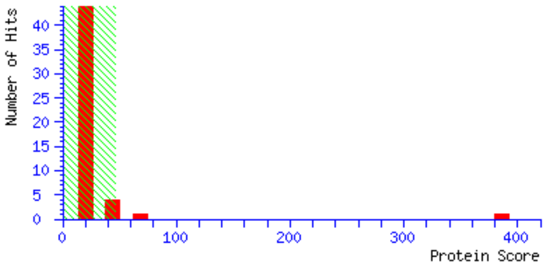

Matched peptide sequences: shown in Bold Red:

1 MAEEVILLDF WPSPFGMRVR IALAEKGVKY EYREEDLRNK SPLLLQMNPV
51 HKKIPVLIHN GKPICESLIA VQYVDEVWKD KSPFLPSDPY QRAQARFWAD
101 FIDKKIYDIG RKIWTTKGDE QEAAKKEFIE ALKLLEGELG NKPYFGGESM
151 GYVDVALIPF YSWFYAYETC GNFSIEPECP VLIAWAKRCL QKESVSESVP
201 DPEKVYEFVL VLKKKFGIE

Spot No.: 228

NCBI accession No.: gi|211906460 Species: *Gossypium hirsutum*

PFF score: [243]

Protein name： triosephosphate isomerase

Matched peptides No.: [2] Sequence coverage %: [15]

Matched sequences: K.VIACIGETLEQR.E;

K.ELAAQPDVDGFLVGGASLKPEFIDIIK.S

Calculated Mr: **27472** Calculated *p*I: **6.00**

Probability Based Mowse Score:

Ions score is -10*Log(P), where P is the probability that the observed match is a random event.
Individual ions scores > 45 indicate identity or extensive homology (p<0.05).
Protein scores are derived from ions scores as a non-probabilistic basis for ranking protein hits.

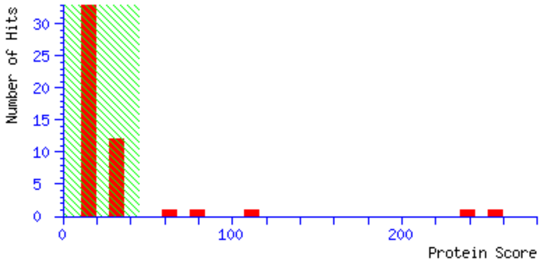

Matched peptide sequences: shown in Bold Red:

```
  1 MGRKFFVGGN WKCNGTTEEV NKIVSTLNAG EVPSQDVVEV VVSPPFVFLP
 51 LVKTSLRPDF HVAAQNCWVK KGGAFTGEVS AEMLVNLSIP WVIIGHSERR
101 LILKESNEFV ADKVAYALAQ GLKVIACIGE TLEQREAGST VAVVAEQTKA
151 IAAKISNWTD VVLAYEPVWA IGTGKVATPA QAQEVHCELR KWLQANVSPE
201 VAASTRIIYG GSVTAANCKE LAAQPDVDGF LVGGASLKPE FIDIIKSAEV
251 KKNA
```

Spot No.: 229

NCBI accession No.: gi|255568460 Species: *Ricinus communis*

PFF score: [93]

Protein name： glutathione-s-transferase omega, putative

Matched peptides No.: [2] Sequence coverage %: [13]

Matched sequences: K.YDITAGRPK.L;

K.FDDGPFLLGQFSLVDIAYIPFVER.L

Calculated Mr: **27477** Calculated *p*I: **5.13**

Probability Based Mowse Score:

Ions score is -10*Log(P), where P is the probability that the observed match is a random event.
Individual ions scores > 45 indicate identity or extensive homology (p<0.05).
Protein scores are derived from ions scores as a non-probabilistic basis for ranking protein hits.

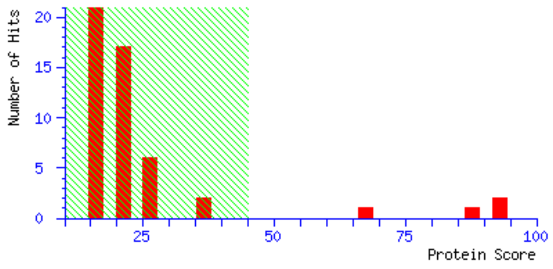

Matched peptide sequences: shown in Bold Red:

```
  1 MAAATALDKS AQENLPPILD AAADQPPLFD GTIRLYTAYA CPFAQRVWIT
 51 RNYKGLQDSI KLVPLNLQNR PSWYPEKVYS VNKVPALEHN GKIIGESLDL
101 IKYVDSNFEG PSLFPDDPAK REFAEELFSY TDTFSRTVFT SFKGDVAKEA
151 GPAFDYLENA LQKFDDGPFL LGQFSLVDIA YIPFVERLEI FLSEVFKYDI
201 TAGRPKLAAW VGEVNKIGAY KQTKTDPKEL VEYYKKRFFA QQ
```

Spot No.: 230

NCBI accession No.: gi|2970051   Species: *Vigna radiata*

PFF score: [104]

Protein name：ARG10

Matched peptides No.: [2]   Sequence coverage %: [12]

Matched sequences: K.SANEVILVIEAYK.A;

K.ITAVPAEEEEIWGATFK.V

Calculated Mr: **25750**   Calculated *p*I: **5.62**

Probability Based Mowse Score:

Ions score is -10*Log(P), where P is the probability that the observed match is a random event.
Individual ions scores > 46 indicate identity or extensive homology (p<0.05).
Protein scores are derived from ions scores as a non-probabilistic basis for ranking protein hits.

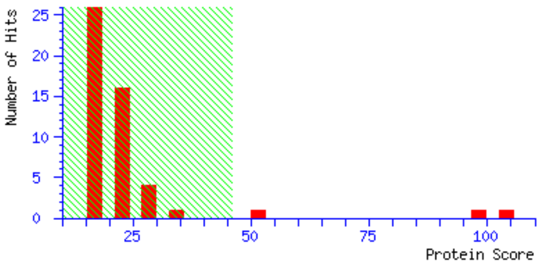

Matched peptide sequences: shown in Bold Red:

1 MLGIFSSSVV SPPEELVAAG SRTPSPKTTA GKLLTRFVES KASAVSLQVG
51 EHVQLAYTHH SESPWYPRSF AVKDEIFCLF EGALDNLGSL RQQYGLAK**SA**
101 **NEVILVIEAY** **K**ALRDRAPYP ANRVVCHLSG SFAFIVFDKS TSTVFVASDQ
151 AGKVPLYWGI TADGYVAFAD DADLLKGSCG KSLASFPQGC FYSTAVGGLR
201 CYENPKNK**IT** **AVPAEEEEIW** **GATFK**VEGSA VLAATEY

Spot No.: 231

NCBI accession No.: gi|2832430 Species: *Hevea brasiliensis*

PFF score: [217]

Protein name： prohevein

Matched peptides No.: [2] Sequence coverage %: [20]

Matched sequences: R.IVDQCSNGGLDLDVNVFR.Q;

K.YGWTAFCGPVGAHGQPSCGK.C

Calculated Mr: **20852** Calculated *p*I: **5.64**

Probability Based Mowse Score:

Ions score is -10*Log(P), where P is the probability that the observed match is a random event.
Individual ions scores > 45 indicate identity or extensive homology (p<0.05).
Protein scores are derived from ions scores as a non-probabilistic basis for ranking protein hits.

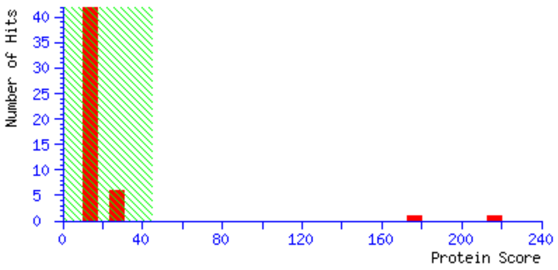

Matched peptide sequences: shown in Bold Red:

```
  1 EQCGRQAGGK LCPNNLCCSQ WGWCGSTDEY CSPDHNCQSN CKDSGEGVGG
 51 GSASNVLATY HLYNSQDHGW DLNAASAYCS TWDANKPYSW RSKYGWTAFC
101 GPVGAHGQPS CGKCLSVTNT GTGAKATVRI VDQCSNGGLD LDVNVFRQLD
151 TDGKGYERGH LTVNYQFVDC GDSFNPLFSV MKSSVIN
```

Spot No.: 232

NCBI accession No.: gi|2832430 Species: *Hevea brasiliensis*

PFF score: [112]

Protein name： prohevein

Matched peptides No.: [2] Sequence coverage %: [16]

Matched sequences: R.QLDTDGKGYER.G;

K.YGWTAFCGPVGAHGQPSCGK.C

Calculated Mr: **20852** Calculated *p*I: **5.64**

Probability Based Mowse Score:

Ions score is -10*Log(P), where P is the probability that the observed match is a random event.
Individual ions scores > 45 indicate identity or extensive homology (p<0.05).
Protein scores are derived from ions scores as a non-probabilistic basis for ranking protein hits.

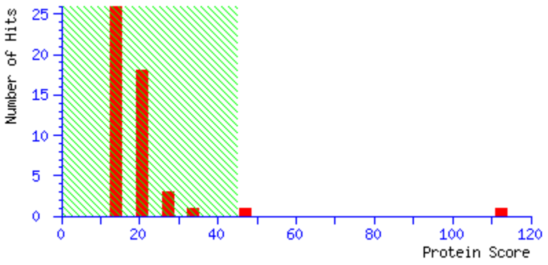

Matched peptide sequences: shown in Bold Red:

```
  1 EQCGRQAGGK LCPNNLCCSQ WGWCGSTDEY CSPDHNCQSN CKDSGEGVGG
 51 GSASNVLATY HLYNSQDHGW DLNAASAYCS TWDANKPYSW RSKYGWTAFC
101 GPVGAHGQPS CGKCLSVTNT GTGAKATVRI VDQCSNGGLD LDVNVFRQLD
151 TDGKGYERGH LTVNYQFVDC GDSFNPLFSV MKSSVIN
```

Spot No.: 233

NCBI accession No.: gi|14423933 Species: *Hevea brasiliensis*

PFF score: [384]

Protein name: Small rubber particle protein

Matched peptides No.: [5] Sequence coverage %: [36]

Matched sequences: K.AEQYAVITWR.A;
R.VSSYLPLLPTEK.I;
K.QVSAQTYSVAQDAPR.I;
K.DISGPLKPGVDTIENVVK.T;
R.IVLDVASSVFNTGVQEGAK.A

Calculated Mr: **22331** Calculated *p*I: **4.80**

Probability Based Mowse Score:

Ions score is -10*Log(P), where P is the probability that the observed match is a random event.
Individual ions scores > 45 indicate identity or extensive homology (p<0.05).
Protein scores are derived from ions scores as a non-probabilistic basis for ranking protein hits.

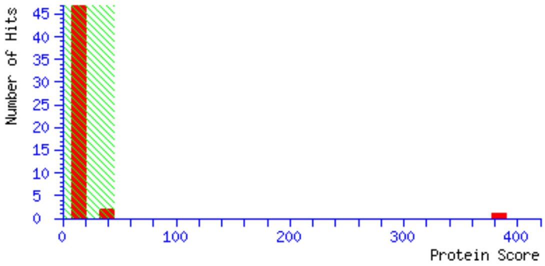

Matched peptide sequences: shown in Bold Red:

```
  1 MAEEVEEERL KYLDFVRAAG VYAVDSFSTL YLYAKDISGP LKPGVDTIEN
 51 VVKTVVTPVY YIPLEAVKFV DKTVDVSVTS LDGVVPPVIK QVSAQTYSVA
101 QDAPRIVLDV ASSVFNTGVQ EGAKALYANL EPKAEQYAVI TWRALNKLPL
151 VPQVANVVVP TAVYFSEKYN DVVRGTTEQG YRVSSYLPLL PTEKITKVFG
201 DEAS
```

Spot No.: 234

NCBI accession No.: gi|132270 Species: *Hevea brasiliensis*

PFF score: [379]

Protein name： Rubber elongation factor protein

Matched peptides No.: [4] Sequence coverage %: [38]

Matched sequences: K.NVAVPLYNR.F;

K.DASIQVVSAIR.A;
K.FVDSTVVASVTIIDR.S;
K.DKSGPLQPGVDIIEGPVK.N

Calculated Mr: **14713** Calculated *p*I: **5.04**

Probability Based Mowse Score:

Ions score is -10*Log(P), where P is the probability that the observed match is a random event.
Individual ions scores > 45 indicate identity or extensive homology (p<0.05).
Protein scores are derived from ions scores as a non-probabilistic basis for ranking protein hits.

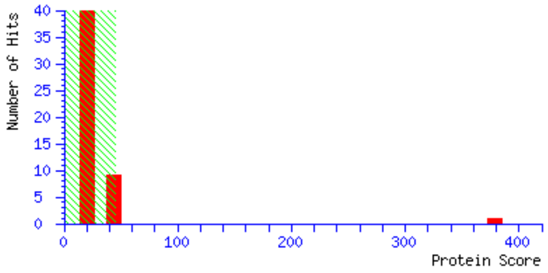

Matched peptide sequences: shown in Bold Red:

1 MAEDEDNQQG QGEGLKYLGF VQDAATYAVT TFSNVYLFAK **DKSGPLQPGV**
51 **DIIEGPVKNV AVPLYNR**FSY IPNGALK**FVD STVVASVTII DR**SLPPIVK**D**
101 **ASIQVVSAIR** AAPEAARSLA SSLPGQTKIL AKVFYGEN

Spot No.: 235

NCBI accession No.: gi|123550 Species: *Pisum sativum*

PFF score: [128]

Protein name：17.1 kDa class II heat shock protein

Matched peptides No.: [1] Sequence coverage %: [9]

Matched sequences: K.VQVEDENVLLISGER.K

Calculated Mr: **17059** Calculated *p*I: **6.32**

Probability Based Mowse Score:

Ions score is -10*Log(P), where P is the probability that the observed match is a random event.
Individual ions scores > 45 indicate identity or extensive homology (p<0.05).
Protein scores are derived from ions scores as a non-probabilistic basis for ranking protein hits.

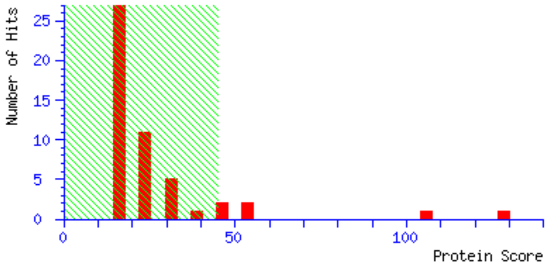

Matched peptide sequences: shown in Bold Red:

```
  1 MDLDSPLFNT LHHIMDLTDD TTEKNLNAPT RTYVRDAKAM AATPADVKEH
 51 PNSYVFMVDM PGVKSGDIKV QVEDENVLLI SGERKREEEK EGVKYLKMER
101 RIGKLMRKFV LPENANIEAI SAISQDGVLT VTVNKLPPPE PKKPKTIQVK
151 VA
```

Spot No.: 236

NCBI accession No.: gi|312985293 Species: *Hevea brasiliensis*

PFF score: [272]

Protein name： nucleoside diphosphate kinase 2

Matched peptides No.: [5] Sequence coverage %: [27]

Matched sequences: K.LMSVERPFAEK.H;

K.LMSVERPFAEK.H + Oxidation (M);
R.NVIHGSDSVENAK.K;
R.NVIHGSDSVENAKK.E;
K.IIGATKPSESDPGTIR.G

Calculated Mr: **16463** Calculated *p*I: **5.96**

Probability Based Mowse Score:

Ions score is -10*Log(P), where P is the probability that the observed match is a random event.
Individual ions scores > 45 indicate identity or extensive homology (p<0.05).
Protein scores are derived from ions scores as a non-probabilistic basis for ranking protein hits.

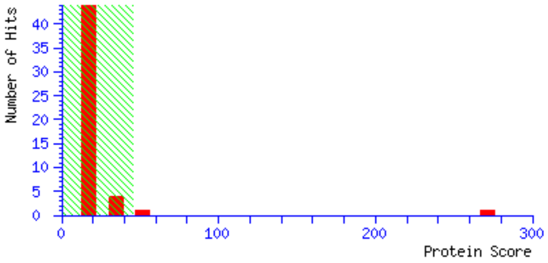

Matched peptide sequences: shown in Bold Red:

```
  1 MEQTFIMIKP DGVQRGLVGE IISRFEKKGF SLKGLKLMSV ERPFAEKHYE
 51 ELSSKPFFEG LVDYIISGPV AAMVWEGKNV VATGRKIIGA TKPSESDPGT
101 IRGDFAVEVG RNVIHGSDSV ENAKKEIALW FPDGTVNWQS SLHSWIYE
```

Spot No.: 237

NCBI accession No.: gi|132270 Species: *Hevea brasiliensis*

PFF score: [90]

Protein name：Rubber elongation factor protein

Matched peptides No.: [2] Sequence coverage %: [23]

Matched sequences: K.FVDSTVVASVTIIDR.S;

K.DKSGPLQPGVDIIEGPVK.N

Calculated Mr: **14713** Calculated *p*I: **5.04**

Probability Based Mowse Score:

Ions score is -10*Log(P), where P is the probability that the observed match is a random event.
Individual ions scores > 45 indicate identity or extensive homology (p<0.05).
Protein scores are derived from ions scores as a non-probabilistic basis for ranking protein hits.

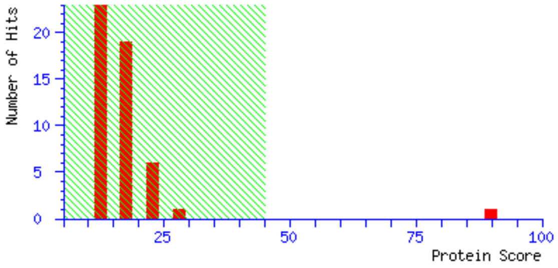

Matched peptide sequences: shown in Bold Red:

1 MAEDEDNQQG QGEGLKYLGF VQDAATYAVT TFSNVYLFAK **DKSGPLQPGV**
51 **DIIEGPVK**NV AVPLYNRFSY IPNGALK**FVD** **STVVASVTII** **DR**SLPPIVKD
101 ASIQVVSAIR AAPEAARSLA SSLPGQTKIL AKVFYGEN

Spot No.: 238

NCBI accession No.: gi|2832430 Species: *Hevea brasiliensis*

PFF score: [344]

Protein name： prohevein

Matched peptides No.: [3] Sequence coverage %: [26]

Matched sequences: R.QLDTDGKGYER.G;

R.IVDQCSNGGLDLDVNVFR.Q;

K.YGWTAFCGPVGAHGQPSCGK.C

Calculated Mr: **20852** Calculated *p*I: **5.64**

Probability Based Mowse Score:

Ions score is -10*Log(P), where P is the probability that the observed match is a random event.
Individual ions scores > 45 indicate identity or extensive homology (p<0.05).
Protein scores are derived from ions scores as a non-probabilistic basis for ranking protein hits.

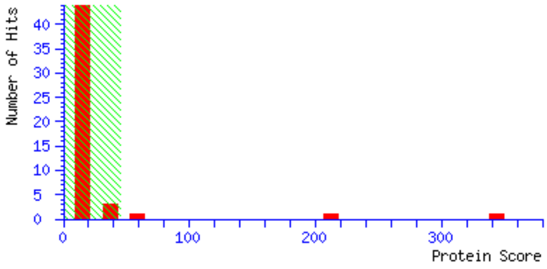

Matched peptide sequences: shown in Bold Red:

```
  1 EQCGRQAGGK LCPNNLCCSQ WGWCGSTDEY CSPDHNCQSN CKDSGEGVGG
 51 GSASNVLATY HLYNSQDHGW DLNAASAYCS TWDANKPYSW RSKYGWTAFC
101 GPVGAHGQPS CGKCLSVTNT GTGAKATVRI VDQCSNGGLD LDVNVFRQLD
151 TDGKGYERGH LTVNYQFVDC GDSFNPLFSV MKSSVIN
```

Spot No.: 239

NCBI accession No.: gi|255570457 Species: *Ricinus communis*

PFF score: [193]

Protein name： protease C56, putative

Matched peptides No.: [2] Sequence coverage %: [8]

Matched sequences: R.APEYLALDETVIALVK.E;

R.APEYLAMNESVLDCVR.K + Oxidation (M)

Calculated Mr: **42437** Calculated *p*I: **5.60**

Probability Based Mowse Score:

Ions score is -10*Log(P), where P is the probability that the observed match is a random event.
Individual ions scores > 45 indicate identity or extensive homology (p<0.05).
Protein scores are derived from ions scores as a non-probabilistic basis for ranking protein hits.

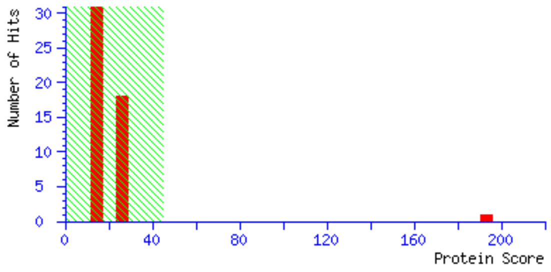

Matched peptide sequences: shown in Bold Red:

```
  1 MSHQKTVFLL CGDYMEDYEA MVPFQALLAY GVAVDAACPG KKAGDYCRTA
 51 IHESSGHQTY SESRGHNFTL NSTFDDIDFG KYDGLVIPGG RAPEYLAMNE
101 SVLDCVRKFA DAGKPIASVC HGQLILAAAG LVKGRKCTAY PPVRPVLIAA
151 GAHWVEPETM ASCVADGNLI TGATYEGHPE FIQLFVKALG GSITGADKRI
201 LFLCGDYMED YEVTVPFQSL QALGCHVNAV CPKKKAGDSC PTAVHDFEGD
251 QTYSEKPGHH FTLTASYVGL DVSSYDALVL PGGRAPEYLA LDETVIALVK
301 QFMQSKKPVA SICHGQQILA AAGVLKGKKC TAYPAVKLNV VLSGATWLEP
351 DPIDRCYTDE NLVTGAAWPG HPEFISQLMA LLGIRVSF
```

Spot No.: 240

NCBI accession No.: gi|727357 Species: *Arabidopsis thaliana*

PFF score: [58]

Protein name： DnaJ homolog

Matched peptides No.: [2] Sequence coverage %: [6]

Matched sequences: R.EIYDQYGEDALK.E;

K.ELAQAYEVLSDPEKR.E

Calculated Mr: **47042** Calculated *p*I: **5.68**

Probability Based Mowse Score:

Ions score is -10*Log(P), where P is the probability that the observed match is a random event.
Individual ions scores > 45 indicate identity or extensive homology (p<0.05).
Protein scores are derived from ions scores as a non-probabilistic basis for ranking protein hits.

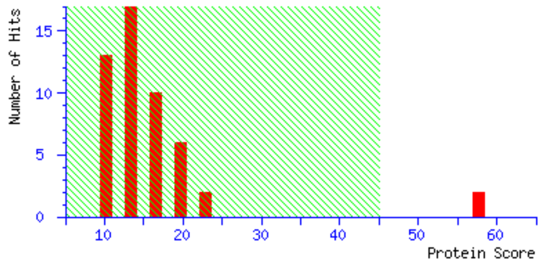

Matched peptide sequences: shown in Bold Red:

```
  1 MFGRGPSKKS DNTKFYEILG VPKSASPEDL KKAYKKAAIK NHPDKGGDPE
 51 KFKELAQAYE VLSDPEKREI YDQYGEDALK EGMGGGGGGH DPFDIFSSFF
101 GGGPFGGNTS RQRRQRRGED VVHPLKVSLE DVYLGTMKKL SLSRNALCSK
151 CNGKGSKSGA SLKCGGCQGS GMKVSIRQLG PGMIQQMQHA CNECKGTGET
201 INDRDRCPQC KGDKVIPEKK VLEVNVEKGM QHSQKITFEG QADEAPDTVT
251 GDIVFVLQQK EHPQFKRKGE DLFVEHTLSL TEALCGFQFV LTHLDGRSLL
301 IKSNPGEVVK PDSYKAISDE GMPIYQRPFM KGKLYIHFTV EFPDSLSPDQ
351 TKALEAVLPK PSTAQLSDME IDECEETTLH DVNIEDEMRR KAQAQREAYD
401 DDDEDDDHPG GAQRVQCAQQ
```

Spot No.: 241

NCBI accession No.: gi|255568794 Species: *Ricinus communis*

PFF score: [100]

Protein name: nucleoredoxin, putative

Matched peptides No.: [3] Sequence coverage %: [5]

Matched sequences: K.LIEAYHEIK.A;

K.KCDFDLHPK.C;

R.VEYFRQEEENAK.K

Calculated Mr: **63728** Calculated *p*I: **5.46**

Probability Based Mowse Score:

Ions score is -10*Log(P), where P is the probability that the observed match is a random event.
Individual ions scores > 45 indicate identity or extensive homology (p<0.05).
Protein scores are derived from ions scores as a non-probabilistic basis for ranking protein hits.

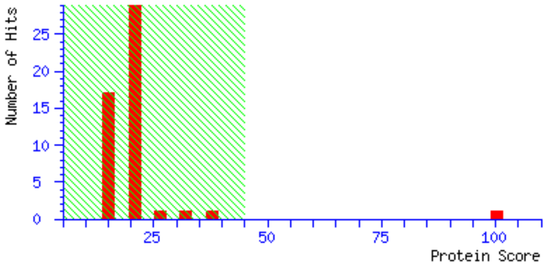

Matched peptide sequences: shown in Bold Red:

```
  1 MADDPVIDVS HDLLSLLSSD DRDFLIRSNG DQVKISNLVG KIVGLYFSGS
 51 WCGPCRHFTP NLVQVYEELS LKGDFEVVFI SSDRDAESFD AYFSKMPWLA
101 IPFSDQETCK HLKDLFKVRG IPNLVFLDAD GKVSCDQGVR FIREYGAEGY
151 PFTPERVEYF RQEEENAKKN QTLSSILVSS SRDFLISKDG TKIPVSELEG
201 KMVGLYFSVH SHRLCLDFTP RLEEVYKKLK EKGEKFEVVL ISMDYDENNF
251 KQGLETMPWL ALPFEDKSRE RLARYFELSA LPTLVIIGED GKTLNKNVAE
301 LIEGHGIQAY PFTPEKLVEL AEIEKARLEA QTLESVLVHG DKDFVIEESG
351 SKVPVSELVG KNILLYFSAK WCPPCRAFLP KLIEAYHEIK AKDNAFEIIF
401 ISSDRDQSSF DEFYTEMPWL ALPFGDDRKT ILQRKFKIKG IPAAIAISPT
451 GKTLTKEARE HITAYGADAY PFNEDHLKQL NDKQEEIAKG WPEKVRHELH
501 PEHELVRMKR NGYGCDGCKE AGSGWSFYCK KCDFDLHPKC ALKKEENGEK
551 VKK
```

Spot No.: **242**

NCBI accession No.: **gi|37719658** Species: *Hevea brasiliensis*

Protein name： **50 kDa protein**

Mascot score: **93** Sequence coverage %: **31**

Matched peptides No.: **9** Total peptides No.: **33**

Calculated Mr: **41480** Calculated *p*I: **4.60**

Probability Based Mowse Score:

Protein score is -10*Log(P), where P is the probability that the observed match is a random event. Protein scores greater than 73 are significant (p<0.05).

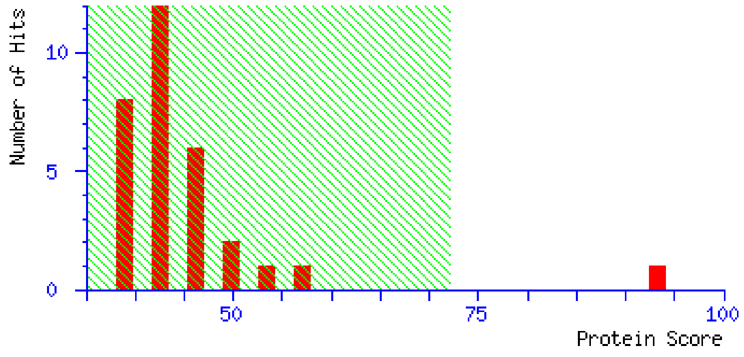

Matched peptide sequences: shown in **Bold Red:**

```
  1 MASLAYSLFI LSLFTFTLLN PVCSELDEYL FSFGDGLYDA GNAKFIYPDK
 51 YLPSYHHPYG TTFFDYPTGR FSDGRTVVDF VAENVSLPRI PPFKNKEANF
101 TYGANFASEG ATASDSNPLI DFRSQIRDFG ELKLEWAVQL VNVTELARRL
151 KKAVYLISFG ADDYLNYEIP SEASREQLES IVDVVLGNIS DRIKELYDFG
201 ARKFVVENVA PLGLIPFIKQ TSDNSTLFYE LASLHAMKLP QILEKIQDGY
251 LFPEFNYTVF NYFGIIKEII DAPGEHGFKY GDIACCGNST YRGQACGFLD
301 YEFCVCGNKT EYLFFDGTHN TDAANNLLAE LMWDKESGFI SPYGVKDFFP
351 SPTTIQTLLT EATALG
```

Spot No.: **243**

NCBI accession No.: **gi|37719658** Species: ***Hevea brasiliensis***

Protein name: **50 kDa protein**

Mascot score: **93** Sequence coverage %: **36**

Matched peptides No.: **11** Total peptides No.: **44**

Calculated Mr: **41480** Calculated *p*I: **4.60**

Probability Based Mowse Score:

Protein score is -10*Log(P), where P is the probability that the observed match is a random event. Protein scores greater than 73 are significant ($p<0.05$).

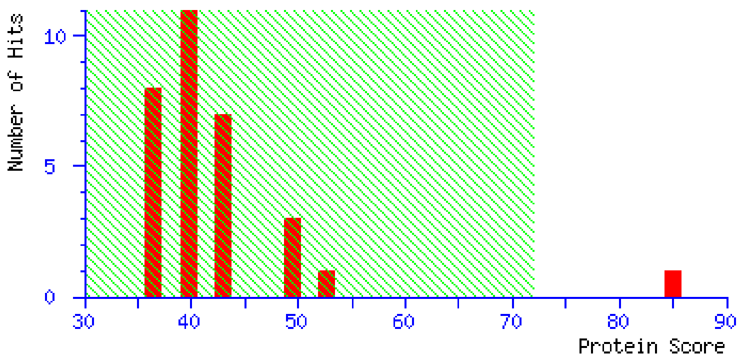

Matched peptide sequences: shown in Bold Red:

```
  1 MASLAYSLFI LSLFTFTLLN PVCSELDEYL FSFGDGLYDA GNAKFIYPDK
 51 YLPSYHHPYG TTFFDYPTGR FSDGRTVVDF VAENVSLPRI PPFKNKEANF
101 TYGANFASEG ATASDSNPLI DFRSQIRDFG ELKLEWAVQL VNVTELARRL
151 KKAVYLISFG ADDYLNYEIP SEASREQLES IVDVVLGNIS DRIKELYDFG
201 ARKFVVENVA PLGLIPFIKQ TSDNSTLFYE LASLHAMKLP QILEKIQDGY
251 LFPEFNYTVF NYFGIIKEII DAPGEHGFKY GDIACCGNST YRGQACGFLD
301 YEFCVCGNKT EYLFFDGTHN TDAANNLLAE LMWDKESGFI SPYGVKDFFP
351 SPTTIQTLLT EATALG
```

Spot No.: **244**

NCBI accession No.: **gi| 37719658** Species: *Hevea brasiliensis*

Protein name: **50 kDa protein**

Mascot score: **95** Sequence coverage %: **42**

Matched peptides No.: **14** Total peptides No.: **66**

Calculated Mr: **41480** Calculated *p*I: **4.60**

Probability Based Mowse Score:

Protein score is -10*Log(P), where P is the probability that the observed match is a random event.
Protein scores greater than 73 are significant (p<0.05).

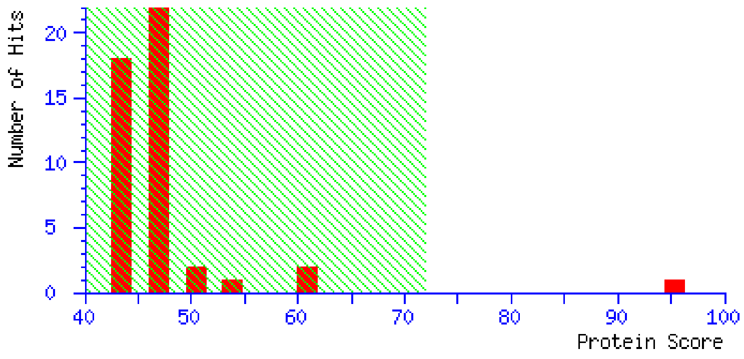

Matched peptide sequences: shown in Bold Red:

1 MASLAYSLFI LSLFTFTLLN PVCSELDEYL FSFGDGLYDA GNAK**FIYPDK**

51 **YLPSYHHPYG TTFFDYPTGR FSDGRTVVDF VAENVSLPRI PPFK**NKEANF

101 TYGANFASEG ATASDSNPLI DFRSQIR**DFG ELK**LEWAVQL VNVTELARRL

151 KK**AVYLISFG ADDYLNYEIP SEASREQLES IVDVVLGNIS DRIKELYDFG**

201 **ARKFVVENVA PLGLIPFIK**Q TSDNSTLFYE LASLHAMK**LP QILEK**IQDGY

251 LFPEFNYTVF NYFGIIK**EII DAPGEHGFKY GDIACCGNST YR**GQACGFLD

301 YEFCVCGNKT EYLFFDGTHN TDAANNLLAE LMWDKESGFI SPYGVKDFFP

351 SPTTIQTLLT EATALG

Spot No.: **245**

NCBI accession No.: **gi|37719658** Species: *Hevea brasiliensis*

Protein name： **50 kDa protein**

Mascot score: **101** Sequence coverage %: **49**

Matched peptides No.: **16** Total peptides No.: **67**

Calculated Mr: **41480** Calculated *p*I: **4.60**

Probability Based Mowse Score:

Protein score is -10*Log(P), where P is the probability that the observed match is a random event.
Protein scores greater than 73 are significant (p<0.05).

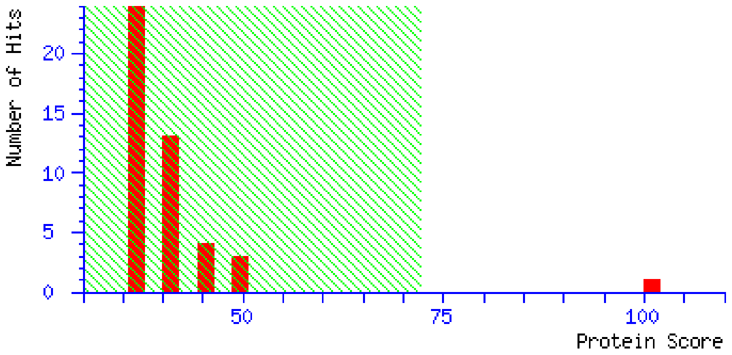

Matched peptide sequences: shown in Bold Red:

```
  1 MASLAYSLFI LSLFTFTLLN PVCSELDEYL FSFGDGLYDA GNAKFIYPDK
 51 YLPSYHHPYG TTFFDYPTGR FSDGRTVVDF VAENVSLPRI PPFKNKEANF
101 TYGANFASEG ATASDSNPLI DFRSQIRDFG ELKLEWAVQL VNVTELARRL
151 KKAVYLISFG ADDYLNYEIP SEASREQLES IVDVVLGNIS DRIKELYDFG
201 ARKFVVENVA PLGLIPFIKQ TSDNSTLFYE LASLHAMKLP QILEKIQDGY
251 LFPEFNYTVF NYFGIIKEII DAPGEHGFKY GDIACCGNST YRGQACGFLD
301 YEFCVCGNKT EYLFFDGTHN TDAANNLLAE LMWDKESGFI SPYGVKDFFP
351 SPTTIQTLLT EATALG
```

Spot No.: **246**

NCBI accession No.: **gi| 37719658** Species: *Hevea brasiliensis*

Protein name： **50 kDa protein**

Mascot score: **118** Sequence coverage %: **47**

Matched peptides No.: **15** Total peptides No.: **46**

Calculated Mr: **41480** Calculated *p*I: **4.60**

Probability Based Mowse Score:

Protein score is -10*Log(P), where P is the probability that the observed match is a random event.
Protein scores greater than 73 are significant (p<0.05).

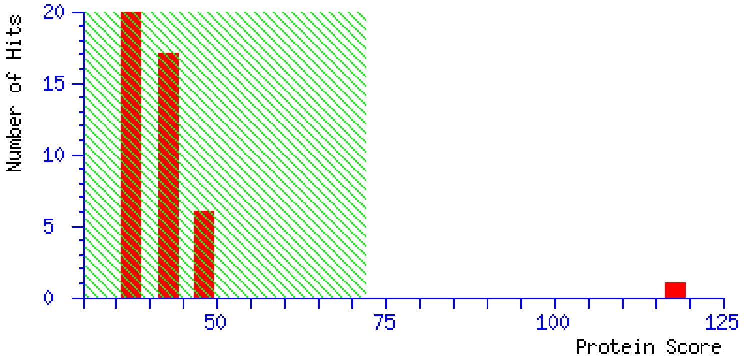

Matched peptide sequences: shown in **Bold Red:**

1 MASLAYSLFI LSLFTFTLLN PVCSELDEYL FSFGDGLYDA GNAKFIYPDK
51 **YLPSYHHPYG TTFFDYPTGR FSDGRTVVDF VAENVSLPRI PPFK**NKEANF
101 TYGANFASEG ATASDSNPLI DFR**SQIR**DFG ELKLEWAVQL VNVTELARRL
151 KK**AVYLISFG ADDYLNYEIP SEASREQLES IVDVVLGNIS DRIKELYDFG**
201 **ARKFVVENVA PLGLIPFIK**Q TSDNSTLFYE LASLHAMK**LP QILEK**IQDGY
251 LFPEFNYTVF NYFGIIK**EII DAPGEHGFKY GDIACCGNST YRGQACGFLD**
301 **YEFCVCGNK**T EYLFFDGTHN TDAANNLLAE LMWDK**ESGFI SPYGVK**DFFP
351 SPTTIQTLLT EATALG

Spot No.: **247**

NCBI accession No.: **gi|123062** Species: *Hevea brasiliensis*

PFF score: [158]

Protein name: Pro-hevein

Matched peptides No.: [2] Sequence coverage %: [11]

Matched sequences: K.CLSVTNTGTGAK.T;
R.QLDTDGKGYER.G

Calculated Mr: **22700** Calculated *p*I: **5.63**

Probability Based Mowse Score:

Ions score is -10*Log(P), where P is the probability that the observed match is a random event.
Individual ions scores > 45 indicate identity or extensive homology (p<0.05).
Protein scores are derived from ions scores as a non-probabilistic basis for ranking protein hits.

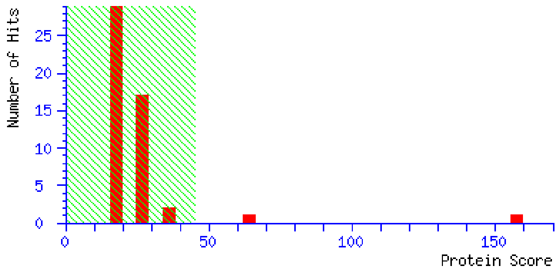

Matched peptide sequences: shown in Bold Red:

```
  1 MNIFIVVLLC LTGVAIAEQC GRQAGGKLCP NNLCCSQWGW CGSTDEYCSP
 51 DHNCQSNCKD SGEGVGGGSA SNVLATYHLY NSQDHGWDLN AASAYCSTWD
101 ANKPYSWRSK YGWTAFCGPV GAHGQSSCGK CLSVTNTGTG AKTTVRIVDQ
151 CSNGGLDLDV NVFRQLDTDG KGYERGHITV NYQFVDCGDS FNPLFSVMKS
201 SVIN
```

Spot No.: **248**

NCBI accession No.: **gi|2832430** Species: *Hevea brasiliensis*

PFF score: [446] Protein name: prohevein

Matched peptides No.: [4] Sequence coverage %: [32]

Matched sequences: K.CLSVTNTGTGAK.A;
R.QLDTDGKGYER.G;
R.IVDQCSNGGLDLDVNVFR.Q;
K.YGWTAFCGPVGAHGQPSCGK.C

Calculated Mr: **20852** Calculated *p*I: **5.64**

Probability Based Mowse Score:

Ions score is -10*Log(P), where P is the probability that the observed match is a random event.
Individual ions scores > 46 indicate identity or extensive homology (p<0.05).
Protein scores are derived from ions scores as a non-probabilistic basis for ranking protein hits.

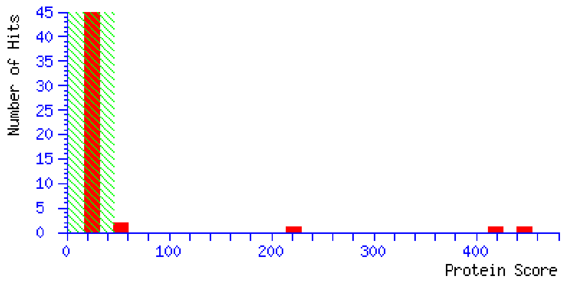

Matched peptide sequences: shown in Bold Red:

```
  1  EQCGRQAGGK  LCPNNLCCSQ  WGWCGSTDEY  CSPDHNCQSN  CKDSGEGVGG
 51  GSASNVLATY  HLYNSQDHGW  DLNAASAYCS  TWDANKPYSW  RSKYGWTAFC
101  GPVGAHGQPS  CGKCLSVTNT  GTGAKATVRI  VDQCSNGGLD  LDVNVFRQLD
151  TDGKGYERGH  LTVNYQFVDC  GDSFNPLFSV  MKSSVIN
```

Spot No.: **249**

NCBI accession No.: **gi|132270** Species: *Hevea brasiliensis*

PFF score: [405]

Protein name: Rubber elongation factor protein

Matched peptides No.: [4] Sequence coverage %: [38]

Matched sequences: K.NVAVPLYNR.F;
K.DASIQVVSAIR.A;
K.FVDSTVVASVTIIDR.S;
K.DKSGPLQPGVDIIEGPVK.N

Calculated Mr: **14713** Calculated *p*I: **5.04**

Probability Based Mowse Score:

Ions score is -10*Log(P), where P is the probability that the observed match is a random event.
Individual ions scores > 46 indicate identity or extensive homology (p<0.05).
Protein scores are derived from ions scores as a non-probabilistic basis for ranking protein hits.

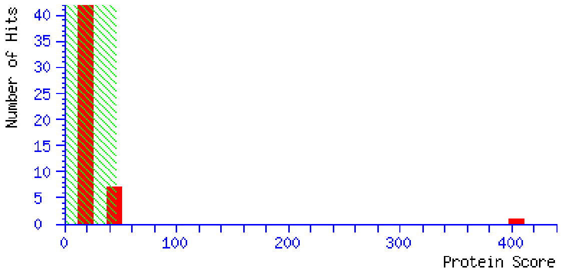

Matched peptide sequences: shown in Bold Red:

1 MAEDEDNQQG QGEGLKYLGF VQDAATYAVT TFSNVYLFAK **DKSGPLQPGV**
51 **DIIEGPVKNV** **AVPLYNR**FSY IPNGALK**FVD** **STVVASVTII** **DR**SLPPIVKD
101 **ASIQVVSAIR** AAPEAARSLA SSLPGQTKIL AKVFYGEN

Spot No.: **250**

NCBI accession No.: **gi|132270**   Species: *Hevea brasiliensis*

PFF score: [340]

Protein name: Rubber elongation factor protein

Matched peptides No.: [4]   Sequence coverage %: [38]

Matched sequences: K.NVAVPLYNR.F;

K.DASIQVVSAIR.A;
K.FVDSTVVASVTIIDR.S;
K.DKSGPLQPGVDIIEGPVK.N

Calculated Mr: **14713**   Calculated *p*I: **5.04**

Probability Based Mowse Score:

Ions score is -10*Log(P), where P is the probability that the observed match is a random event.
Individual ions scores > 46 indicate identity or extensive homology (p<0.05).
Protein scores are derived from ions scores as a non-probabilistic basis for ranking protein hits.

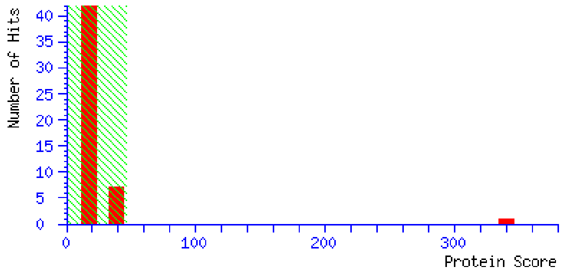

Matched peptide sequences: shown in Bold Red:

```
  1 MAEDEDNQQG QGEGLKYLGF VQDAATYAVT TFSNVYLFAK DKSGPLQPGV
 51 DIIEGPVKNV AVPLYNRFSY IPNGALKFVD STVVASVTII DRSLPPIVKD
101 ASIQVVSAIR AAPEAARSLA SSLPGQTKIL AKVFYGEN
```

Spot No.: **251**

NCBI accession No.: **gi|132270** Species: *Hevea brasiliensis*

PFF score: [470]

Protein name: Rubber elongation factor protein

Matched peptides No.: [4] Sequence coverage %: [27]

Matched sequences: K.NVAVPLYNR.F;
K.DASIQVVSAIR.A;
K.SGPLQPGVDIIEGPVK.N;
K.DKSGPLQPGVDIIEGPVK.N

Calculated Mr: **14713** Calculated *p*I: **5.04**

Probability Based Mowse Score:

Ions score is -10*Log(P), where P is the probability that the observed match is a random event.
Individual ions scores > 46 indicate identity or extensive homology (p<0.05).
Protein scores are derived from ions scores as a non-probabilistic basis for ranking protein hits.

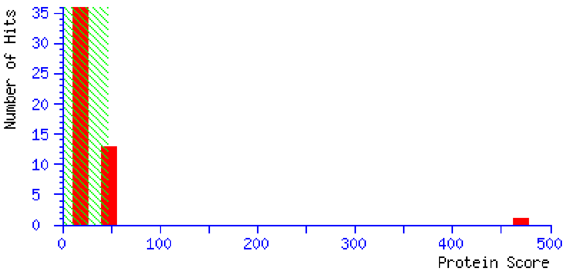

Matched peptide sequences: shown in Bold Red:

```
  1 MAEDEDNQQG QGEGLKYLGF VQDAATYAVT TFSNVYLFAK DKSGPLQPGV
 51 DIIEGPVKNV AVPLYNRFSY IPNGALKFVD STVVASVTII DRSLPPIVKD
101 ASIQVVSAIR AAPEAARSLA SSLPGQTKIL AKVFYGEN
```

Spot No.: **252**

NCBI accession No.: **gi|132270** Species: *Hevea brasiliensis*

PFF score: [402]

Protein name: Rubber elongation factor protein

Matched peptides No.: [4] Sequence coverage %: [38]

Matched sequences: K.NVAVPLYNR.F;

K.DASIQVVSAIR.A;
K.FVDSTVVASVTIIDR.S;
K.DKSGPLQPGVDIIEGPVK.N

Calculated Mr: **14713** Calculated *p*I: **5.04**

Probability Based Mowse Score:

Ions score is -10*Log(P), where P is the probability that the observed match is a random event.
Individual ions scores > 46 indicate identity or extensive homology (p<0.05).
Protein scores are derived from ions scores as a non-probabilistic basis for ranking protein hits.

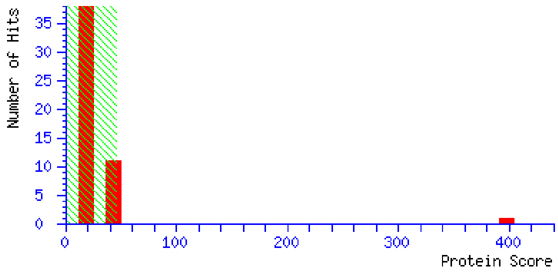

Matched peptide sequences: shown in Bold Red:

```
  1 MAEDEDNQQG QGEGLKYLGF VQDAATYAVT TFSNVYLFAK DKSGPLQPGV
 51 DIIEGPVKNV AVPLYNRFSY IPNGALKFVD STVVASVTII DRSLPPIVKD
101 ASIQVVSAIR AAPEAARSLA SSLPGQTKIL AKVFYGEN
```

Band No.: B1

NCBI accession No.: **gi|132270** Species: *Hevea brasiliensis*

PFF score: [282]

Protein name: **Rubber elongation factor protein; Short=REF;**

AltName: Allergen=Hev b 1

Matched peptides No.: [4] Sequence coverage %: [38]

Matched sequences: K.DKSGPLQPGVDIIEGPVK.N; K.NVAVPLYNR.F;

K.FVDSTVVASVTIIDR.S; K.DASIQVVSAIR.A

Calculated Mr: **14713** Calculated *p*I: **5.04**

Probability Based Mowse Score:

Ions score is -10*Log(P), where P is the probability that the observed match is a random event.
Individual ions scores > 45 indicate identity or extensive homology (p<0.05).
Protein scores are derived from ions scores as a non-probabilistic basis for ranking protein hits.

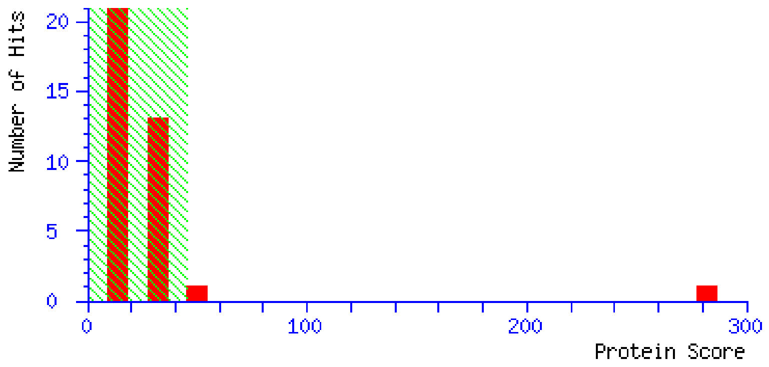

Matched peptide sequences: shown in Bold Red:

```
  1 MAEDEDNQQG QGEGLKYLGF VQDAATYAVT TFSNVYLFAK DKSGPLQPGV
 51 DIIEGPVKNV AVPLYNRFSY IPNGALKFVD STVVASVTII DRSLPPIVKD
101 ASIQVVSAIR AAPEAARSLA SSLPGQTKIL AKVFYGEN
```

Band No.: **B2**

NCBI accession No.: **gi|225440045** Species: ***Vitis vinifera***

Protein name： **cell division cycle protein 48 homolog**

PMF score: **90** Sequence coverage %: **32**

Matched peptides No.: **23** Total peptides No.: **49**

Calculated Mr: **90331** Calculated *p*I: **5.14**

Probability Based Mowse Score:

Protein score is -10*Log(P), where P is the probability that the observed match is a random event.
Protein scores greater than 73 are significant ($p<0.05$).

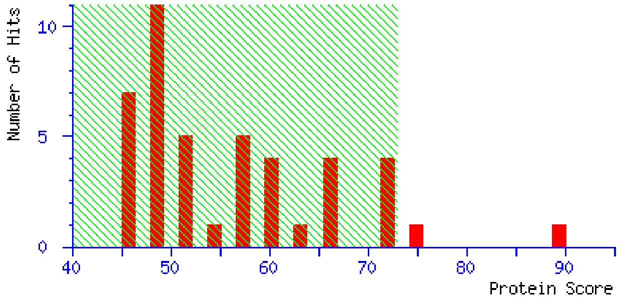

Matched peptide sequences: shown in Bold Red

```
  1 MSNQAESSDS KGTKRDFSTA ILERKKAANR LVVDEAVNDD NSVVALHPDT
 51 MEKLQLFRGD TILIKGKKRK DTICIALADD TCDEPKIRMN KVVRSNLRVR
101 LGDVVSVHQC PDVKYGKRVH ILPVDDTIEG VTGNLFDAYL KPYFLEAYRP
151 VRKGDLFLVR GGMRSVEFKV IETDPAEYCV VAPDTEIFCE GEPVRREDED
201 RLDEVGYDDV GGVRKQMAQI RELVELPLRH PQLFKSIGVK PPKGILLYGP
251 PGSGKTLIAR AVANETGAFF FCINGPEIMS KLAGESESNL RKAFEEAEKN
301 APSIIFIDEI DSIAPKREKT HGEVERRIVS QLLTLMDGLK SRAHVIVIGA
351 TNRPNSIDPA LRRFGRFDRE IDIGVPDEVG RLEVLRIHTK NMKLSEDVDL
401 ERIAKDTHGY VGADLAALCT EAALQCIREK MDVIDLEDES IDAEILNSMA
451 VTDEHFKTAL GTSNPSALRE TVVEVPNVSW EDIGGLENVK RELQETVQYP
501 VEHPEKFEKF GMSPSKGVLF YGPPGCGKTL LAKAIANECQ ANFISVKGPE
551 LLTMWFGESE ANVREIFDKA RQSAPCVLFF DELDSIATQR GSSVGDAGGA
601 ADRVLNQLLT EMDGMSAKKT VFIIGATNRP DIIDPALLRP GRLDQLIYIP
651 LPDEDSRHQI FKACLRKSPV SKDVDLRALA KYTQGFSGAD ITEICQRACK
701 YAIRENIEKD IERERRREN PEAMEEDVDE EVAEIKAAHF EESMKYARRS
751 VSDADIRKYQ AFAQTLQQSR GFGTEFRFSE TSTGAAGSDP FAASAGGAAD
801 EDDLYS
```

Band No.: **B3**

NCBI accession No.: **gi|** 88193697 Species: *Arachis hypogaea*

PFF score: **[100]** Protein name： phospholipase D alpha 2

Matched peptides No.: [2] Sequence coverage %: [2]

Matched sequences: R.SIDGGAAFGFPETPEDAAR.A;

R.SIDGGAAFGFPETPEDAAR.A

Calculated Mr: **92340** Calculated *p*I: **5.54**

Probability Based Mowse Score:

Ions score is -10*Log(P), where P is the probability that the observed match is a random event.
Individual ions scores > 44 indicate identity or extensive homology ($p<0.05$).
Protein scores are derived from ions scores as a non-probabilistic basis for ranking protein hits.

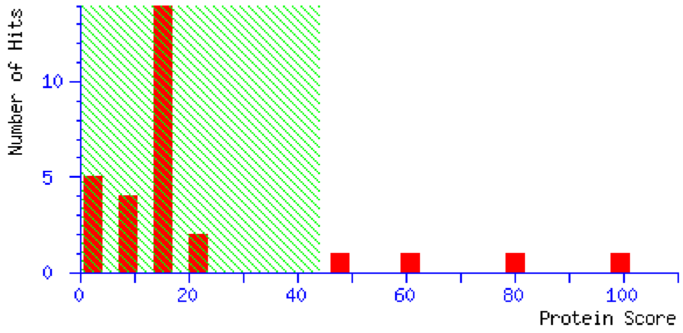

Matched peptide sequences: shown in Bold Red

1	MAQILLHGTL	HVTIYEVDKL	KTSGGNVFTK	LVQNIEETVG	FGKGVTKLYA
51	TIDLEKARVG	RTRIIEKDHS	NPRWYESFHI	YCAHMASNII	FTVKDDNPIG
101	ATLIGRAYVP	VEDVLDGEEV	DRWVEILDED	KNPIHGNSKI	HVKLQYFDVT
151	KDKNWARGVR	SAKFPGVPYT	FFSQRRGCKV	SLYQDAHVPD	NFVPKIPLAG
201	GQTYQPHRCW	EDVFDAIEKA	RHLIYITGWS	VYTEITLVRD	SRRPKPGGDL
251	TIGELLKKKA	NEGVRVLMLV	WDDRTSVPLL	KKDGLMATHD	EETEKYFQGT
301	EVHCILCPRN	PDDGGSIIQD	LQISTMFTHH	QKIVVVDSDM	PSGDSGKRRI
351	VSFVGGIDLC	DGRYDTQFHS	LFRTLDTAHH	DDFHQPNFAG	ASIMKGGPRE
401	PWHDIHSRLE	GPIAWDVLFN	FEQRWRKQGG	KDLLIPLREL	EDVIIPPSPV
451	TFAEDQETWN	VQLFR**SIDGG**	**AAFGFPETPE**	**DAAR**AGLVSG	KDNIIDRSIQ
501	DAYIHAIRRA	KNFIYIENQY	FLGSCFGWSP	DDIKPEDIGA	LHLIPKELSL
551	KIVSKIEAGE	RFTVYIVVPM	WPEGFPESGS	VQAILDWQRR	TMEMMYKDIV
601	EALNAKGIVE	DPRNYLTFFC	LGNREVKKPG	EYEPSERPDP	DSDYIKAQEN
651	RRFMIYVHAK	MMIVDDEYII	IGSANINQRS	MDGARDSEIA	MGAYQPYYLS
701	ARELARGQIH	GFRLALWYEH	LGMIRESFLQ	PESEECIKMV	NQVADKYWDL
751	YSSESLDHDL	PGHLLRYPVG	ISGDGTVTEL	PGFEFFPDTK	GRVLGTKTDY
801	LPPILTT				

Band No.: B4

NCBI accession No.: **gi| 225440324** Species: ***Vitis vinifera***

Protein name：heat shock cognate 70 kDa protein isoform 1

Mascot score: **102** Sequence coverage %: **36**

Matched peptides No.: **22** Total peptides No.: **68**

Calculated Mr: **71420** Calculated *p*I: **5.17**

Probability Based Mowse Score:

Protein score is -10*Log(P), where P is the probability that the observed match is a random event.
Protein scores greater than 73 are significant ($p<0.05$).

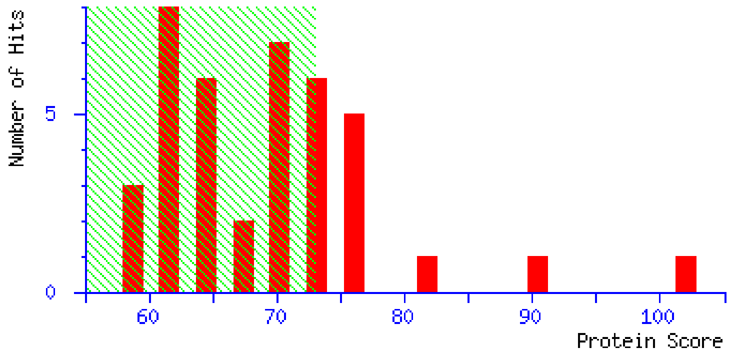

Matched peptide sequences: shown in Bold Red

1	MAGKGEGPAI	GIDLGTTYSC	VGVWQHDRVE	IIANDQGNR**T**	**TPSYVAFTDT**
51	**ER**LVGDAAK**N**	**QVAMNPINTV**	**FDAKR**LIGRR	FSDATVQSDI	KLWPFKVVAG
101	PGDKPMIVVN	YKGEEK**QFAA**	**EEISSMVLMK**	**MREIAEAYLG**	**SAVKNAVVTV**
151	**PAYFNDSQRQ**	**ATKDAGVIAG**	**LNVMRIINEP**	**TAAAIAYGLD**	**KK**ATSVGEKN
201	VLIFDLGGGT	FDVSLLTIEE	GIFEVK**ATAG**	**DTHLGGEDFD**	**NRMVNHFVQE**
251	**FKR**KHKK**DIS**	**GNPR**ALRRLR	TACERAKRTL	SSTAQTTIEI	DSLYEGVDFY
301	TTITR**ARFEE**	**LNMDLFR**KCM	EPVEKCLRDA	KMDK**SSVHDV**	**VLVGGSTRIP**
351	KVQQLLQDFF	NGKELCKSIN	PDEAVAYGAA	VQAAILSGEG	NDKVQDLLLL
401	DVTPLSLGLE	TAGGVMTVLI	PRNTTIPTKK	**EQVFSTFSDN**	**QPGVLIQVYE**
451	**GERTR**TRDNN	LLGKFELSGI	PPAPRGVPQI	TVCFDIDANG	ILNVSAEDKT
501	TGQKNKITIT	NDKGRLSKEE	IEK**MVQEAEK**	YKSEDEEHKK	KVDAK**NALEN**
551	**YAYNMR**NTVK	DEKIGAKLPP	ADKKKIEDAI	EQAIQWLDNN	QLAEADEFED
601	KMKELESICN	PIIAK**MYQGA**	**GGPDMGGGMD**	**EDGPSAGGSG**	**AGPK**IEEVD

Band No.: B5

NCBI accession No.: gi|253760141 Species: *Sorghum bicolor*

Protein name： hypothetical protein SORBIDRAFT_1058s002010

Mascot score: 95 Sequence coverage %: 57

Matched peptides No.: 15 Total peptides No.: 59

Calculated Mr: 23743 Calculated *p*I: 6.77

Probability Based Mowse Score:

Protein score is -10*Log(P), where P is the probability that the observed match is a random event. Protein scores greater than 73 are significant (p<0.05).

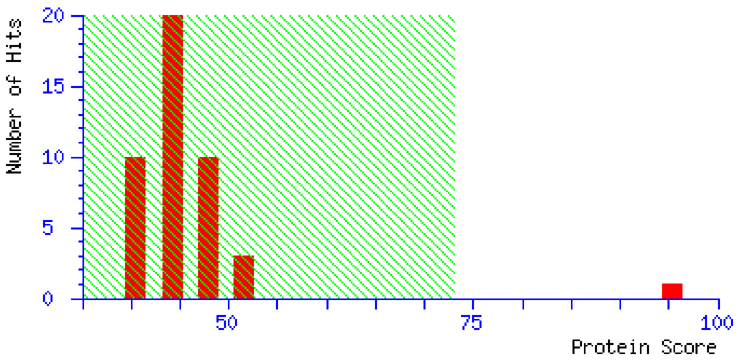

Matched peptide sequences: shown in Bold red:

```
  1  MASAVASMVA APKPVFLMDL LASPSASSRH LFRTGDARPR SSSDGVKDST
 51  GKDDAAAADG RPERDPSIPK LFTGDVLDLF PEAAKRDLLL ALAEDGGGAG
101  SMITGLAPRG WWISKKDGDA LQLKVAMPGL GKEHVKMRAE KNVLVIKGEG
151  DKDAEGDKDA EGDDDKVPVP VPVPARYIYR IGLPSQAFKM DQIKAEMKNG
201  VLILTMPKIK GEGRKDVFGI KIE
```

Band No.: B6

NCBI accession No.: gi|14423933 Species: *Hevea brasiliensis*

PFF score: [348]

Protein name: **Small rubber particle protein; Short=SRPP; AltName: Full=22 kDa rubber particle protein; Short=22 kDa RPP; AltName: Full=27 kDa natural rubber allergen; AltName: Full=Latex allergen Hev b 3; AltName: Allergen=Hev b 3**

Matched peptides No.: [4] Sequence coverage %: [21]

Matched sequences: K.QVSAQTYSVAQDAPR.I; R.IVLDVASSVFNTGVQEGAK.A; R.IVLDVASSVFNTGVQEGAK.A; K.AEQYAVITWR.A

Calculated Mr: 22331 Calculated *p*I: 4.80

Probability Based Mowse Score:

Ions score is -10*Log(P), where P is the probability that the observed match is a random event.
Individual ions scores > 44 indicate identity or extensive homology (p<0.05).
Protein scores are derived from ions scores as a non-probabilistic basis for ranking protein hits.

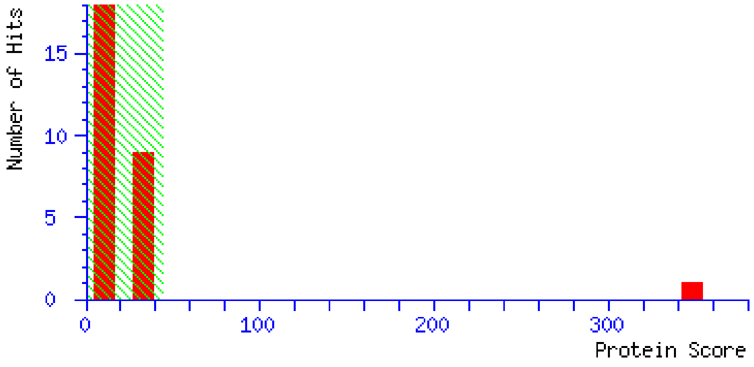

Matched peptide sequences: shown in Bold Red:

```
  1 MAEEVEEERL KYLDFVRAAG VYAVDSFSTL YLYAKDISGP LKPGVDTIEN
 51 VVKTVVTPVY YIPLEAVKFV DKTVDVSVTS LDGVVPPVIK QVSAQTYSVA
101 QDAPRIVLDV ASSVFNTGVQ EGAKALYANL EPKAEQYAVI TWRALNKLPL
151 VPQVANVVVP TAVYFSEKYN DVVRGTTEQG YRVSSYLPLL PTEKITKVFG
201 DEAS
```

Band No.: B7

NCBI accession No.: **gi|260099871** Species: ***Hevea Brasiliensis***

Protein name: **beta-1,3-glucanase**

Mascot score: **125** Sequence coverage %: **40**

Matched peptides No.: **16** Total peptides No.: **38**

Calculated Mr: **41543** Calculated *p*I: **9.40**

Probability Based Mowse Score:

Protein score is -10*Log(P), where P is the probability that the observed match is a random event.
Protein scores greater than 73 are significant ($p<0.05$).

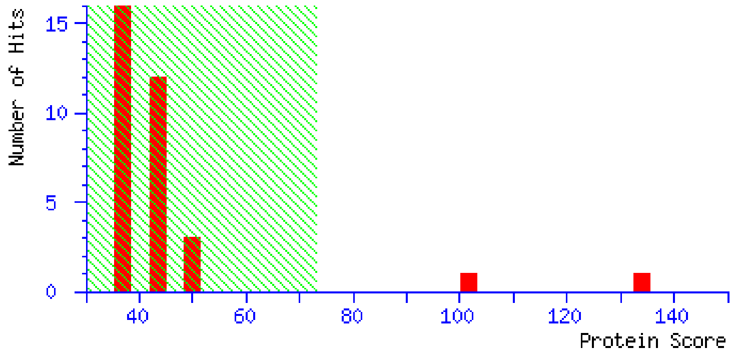

Matched peptide sequences: shown in **Bold Red**

1 MAISSSTSGT SSSLPSRTTV MLLLFFFTAS VGITDAQVGV CYGMQGNNLP
51 PVSEVIALYK KSNITRMR**IY** **DPNQAVLEAL** **RGSNIELILG** **VPNSDLQSLT**
101 **NPSNAK**SWVQ KNVRGFWSSV RFR**YIAVGNE** **ISPVNR**GTAW LAQFVLPAMR
151 **NIHDAIRSAG** **LQDQIKVSTA** **IDLTLVGNSY** **PPSAGAFRDD** **VRSYLNPIIR**
201 **FLSSIRSPLL** **ANIYPYFTYA** **GNPR**DISLPY ALFTSPSVVV WDGQRGYKNL
251 FDATLDALYS ALERASGGSL EVVVSESGWP SAGAFAATFD NGR**TYLSNLI**
301 **QHVK**RGTPKR PK**RAIETYLF** **AMFDENKK**QP EVEKHFGLFF PNKWQKYNLN
351 FSAEKNWDIS TEHNATILFL KSDM

Band No.: B8

NCBI accession No.: **gi| 10946499** Species: ***Hevea Brasiliensis***

Protein name： **beta-1,3-glucanase**

Mascot score: **187** Sequence coverage %: **61**

Matched peptides No.: **20** Total peptides No.: **62**

Calculated Mr: **35295** Calculated *p*I: **9.46**

Probability Based Mowse Score:

Protein score is -10*Log(P), where P is the probability that the observed match is a random event.
Protein scores greater than 73 are significant ($p<0.05$).

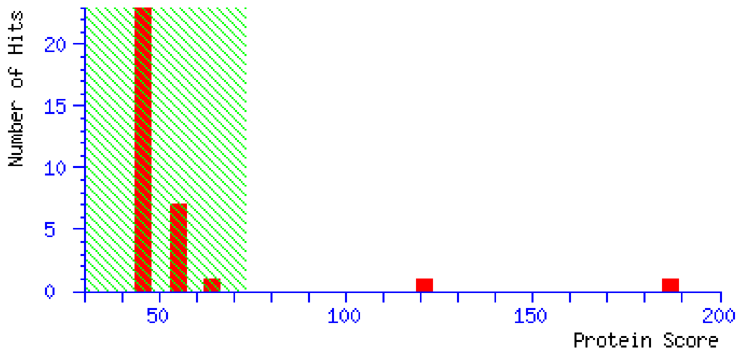

Matched peptide sequences: shown in Bold Red

1 QVGVCYGMQG NNLPPVSEVI ALYKKSNITR MR**IYDPNRAV** **LEALRGSNIE**
51 **LILGVPNSDL** **QSLTNPSNAK** **SWVQK**NVRGF **WSSVLFRYIA** **VGNEISPVNR**
101 **GTAWLAQFVL** **PAMRNIHDAI** **RSAGLQDQIK** **VSTAIDLTLV** **GNSYPPSAGA**
151 **FRDDVRSYLN** **PIIRFLSSIR** **SPLLANIYPY** **FTYAGNPR**DI SLPYALFTSP
201 SVVVWDGQRG YK**NLFDATLD** **VLYSALER**AS GGSLEVVVSE SGWPSAGAFA
251 ATFDNGR**TYL** **SNLIQHVK**RG TPKRPNR**AIE** **TYLFAMFDEN** **KK**QPEVEKQF
301 GLFFPDKWQK YNLNFG

Band No.: B9

NCBI accession No.: gi|255540341 Species: *Ricinus communis*

Protein name： glyceraldehyde 3-phosphate dehydrogenase, putative

Mascot score: 103 Sequence coverage %: 48

Matched peptides No.: 15 Total peptides No.: 82

Calculated Mr: 36930 Calculated *p*I: 7.10

Probability Based Mowse Score:

Protein score is -10*Log(P), where P is the probability that the observed match is a random event. Protein scores greater than 73 are significant (p<0.05).

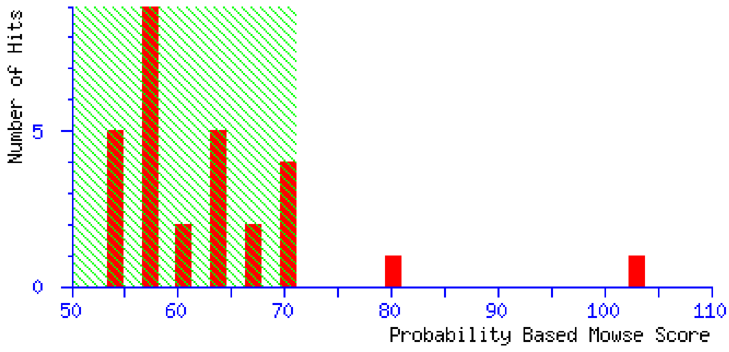

Matched peptide sequences: shown in Bold red:

```
  1 MAKIKIGING FGRIGRLVAR VALQRDDVEL VAVNDPFIST EYMTYMFKYD
 51 TVHGQWKHHE LKIKDDKTLL FGEKPVTVFG VRNPEEIPWA ETGAEYIVES
101 TGVFTDKDKA AAHLKGGAKK VIISAPSKDA PMFVVGVNEK EYTPDLHIIS
151 NASCTTNCLA PLAKVINDRF GIVEGLMTTV HSITATQKTV DGPSMKDWRG
201 GRAASFNIIP SSTGAAKAVG KVLPSLNGKL TGMSFRVPTV DVSVVDLTVR
251 LEKSATYEQI KAAIKEESEG KLKGILGYTE DDVVSTDFIG DKRSSIFDAK
301 AGIALNDKFV KLVSWYDNEW GYSTRVVDLI VHVASVH
```

Band No.: **B10**

NCBI accession No.: **gi|14423933** Species: ***Hevea Brasiliensis***

Protein name: **Small rubber particle protein**

Mascot score: **160** Sequence coverage %: **77**

Matched peptides No.: **13** Total peptides No.: **44**

Calculated Mr: **22331** Calculated *p*I: **4.80**

Probability Based Mowse Score:

Protein score is -10*Log(P), where P is the probability that the observed match is a random event. Protein scores greater than 73 are significant (p<0.05).

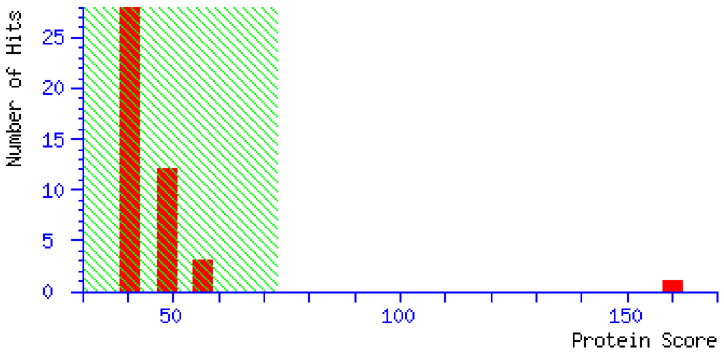

Matched peptide sequences: shown in Bold Red

```
  1  MAEEVEEERL KYLDFVRAAG VYAVDSFSTL YLYAKDISGP LKPGVDTIEN
 51  VVKTVVTPVY YIPLEAVKFV DKTVDVSVTS LDGVVPPVIK QVSAQTYSVA
101  QDAPRIVLDV ASSVFNTGVQ EGAKALYANL EPKAEQYAVI TWRALNKLPL
151  VPQVANVVVP TAVYFSEKYN DVVRGTTEQG YRVSSYLPLL PTEKITKVFG
201  DEAS
```

Band No.: **B11**

NCBI accession No.: **gi| 38122474** Species: ***Hevea Brasiliensis***

Protein name: **rubber elongation factor**

Mascot score: **106** Sequence coverage %: **42**

Matched peptides No.: **10** Total peptides No.: **29**

Calculated Mr: **19570** Calculated *p*I: **5.06**

Probability Based Mowse Score:

Protein score is -10*Log(P), where P is the probability that the observed match is a random event. Protein scores greater than 73 are significant ($p<0.05$).

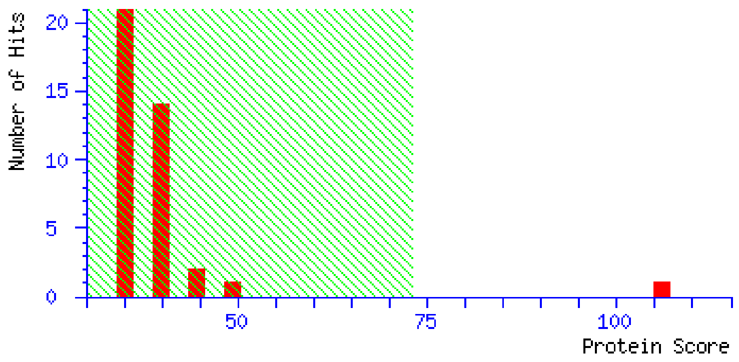

Matched peptide sequences: shown in Bold Red

```
  1 MAEGEEEVNI QEEANKGEEN PQEEANIQEE TNKGEENIQE EANIEEEANK
 51 EEESLKYLDF VQAATVYARA SFSKLYLFAK DKSGPFKPGV NTVESRFKSV
101 VRPVYNKFQP VPNKVLKFAD RRVDAYVTVL DRIVPPIVKR ASIQAYSVAP
151 GAALAVASYL PLHTKRLSKV LYGDG
```

Band No.: B12

NCBI accession No.: gi|3288200 Species: *Hevea brasiliensis*

Protein name： latex allergen

Mascot score: 87 Sequence coverage %: 41

Matched peptides No.: 12 Total peptides No.: 43

Calculated Mr: 43020 Calculated *p*I: 5.00

Probability Based Mowse Score:

Protein score is -10*Log(P), where P is the probability that the observed match is a random event. Protein scores greater than 73 are significant (p<0.05).

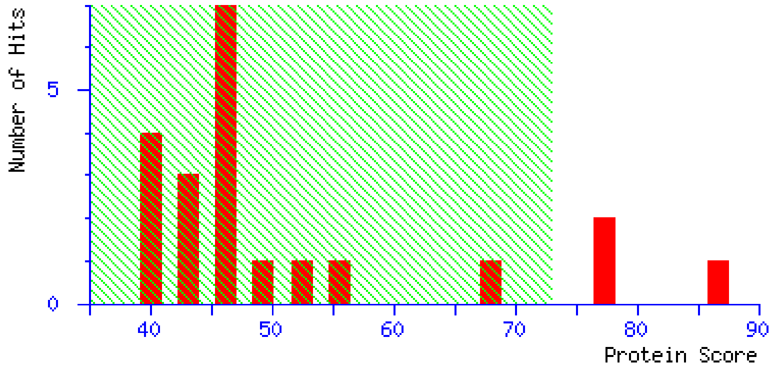

Matched peptide sequences: shown in Bold red:

```
  1 MATGSTTLTQ GKKITVLSID GGGIRGIIPG IILASLESKL QDLDGPDARI
 51 ADYFDIIAGT STGGLITTML TAPNEDKKPI YQAKDIKDFY LENCPKIFPK
101 ESRDTYDPIH SIGPIYDGEY LRELCNNLLK DLTVKDTLTD VIIPAFDIKL
151 LLPVIFSSDD AKCNALKNAR LADVCISTSA APVLLPAHSF TTEDDKNIHT
201 FELIDGGVAA ANPTLLALTH IRNEIIRQNP RFIGANLTES KSRLVLSLGT
251 GKSEYKEKYN ADMTSKWRLY NWALYNGNSP AVDIFSNASS DMVDFHLSAL
301 FKSLDCEDYY LRIQDDTLTG EESSGHIATE ENLQRLVEIG TELLEKQESR
351 INLDTGRLES IPGAPTNEAA IAKFAKLLSE ERKLRQLK
```

Band No.: B13

NCBI accession No.: **gi| 334854628** Species: ***Hevea brasiliensis***

PFF score: [271] Protein name: **rubber elongation factor**

Matched peptides No.: [3] Sequence coverage %: [27]

Matched sequences: K.DKSGPLQPGVDIIEGPVK.N; K.NVAVPLYNR.F;

K.DASIQVVSAIR.A

Calculated Mr: **14889** Calculated *p*I: **5.04**

Probability Based Mowse Score:

Ions score is -10*Log(P), where P is the probability that the observed match is a random event.
Individual ions scores > 45 indicate identity or extensive homology (p<0.05).
Protein scores are derived from ions scores as a non-probabilistic basis for ranking protein hits.

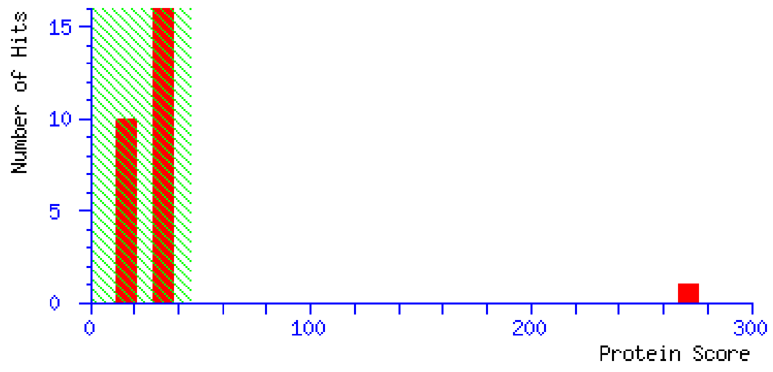

Matched peptide sequences: shown in Bold Red

```
  1 MAEDEDNQQG QGEGLKYLGF VQDAATYAVT TFSNVYLFAK DKSGPLQPGV
 51 DIIEGPVKNV AVPLYNRFSY IPNGALKFVD SPVVASVTII DRSLPPIVKD
101 ASIQVVSAIR AAPEAARFLA FFLPGQTKIL AKVFYGEN
```

Band No.: B14

NCBI accession No.: gi|14423688 Species: *Hevea brasiliensis*

Protein name: Enolase 1; AltName: Full=2-phospho-D-glycerate hydro-lyase 1; AltName: Full=2-phosphoglycerate dehydratase 1; AltName: Allergen=Hev b 9

Mascot score: 117 Sequence coverage %: 48

Matched peptides No.: 20 Total peptides No.: 71

Calculated Mr: 48029 Calculated *p*I: 5.57

Probability Based Mowse Score:

Protein score is -10*Log(P), where P is the probability that the observed match is a random event.
Protein scores greater than 73 are significant ($p<0.05$).

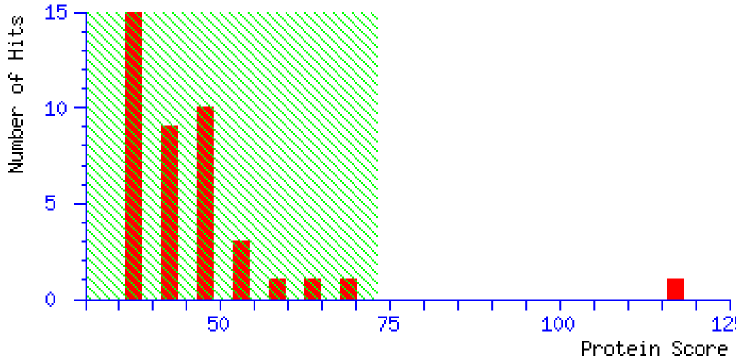

Matched peptide sequences: shown in Bold red:

1	MAITIVSVRA	RQIFDSRGNP	TVEADVKLSD	GYLARAAVPS	GASTGIYEAL
51	ELRDGGSDYL	GKGVSKAVEN	VNIIIGPALV	GKDPTDQVGI	DNFMVQQLDG
101	TVNEWGWCKQ	KLGANAILAV	SLAVCKAGAH	VKGIPLYEHI	ANLAGNKNLV
151	LPVPAFNVIN	GGSHAGNKLA	MQEFMILPVG	ASSFKEAMKM	GAEVYHHLKS
201	VIKKKYGQDA	TNVGDEGGFA	PNIQENKEGL	ELLKTAIAKA	GYTGKVVIGM
251	DVAASEFYGS	DQTYDLNFKE	ENNNGSQKIS	GEALKDLYKS	FVAEYPIVSI
301	EDPFDQDDWA	HYAKLTSEIG	EKVQIVGDDL	LVTNPKRVEK	AIKEKACNAL
351	LLKVNQIGSV	TESIEAVKMS	KRAGWGVMAS	HRSGETEDTF	IADLSVGLAT
401	GQIKTGAPCR	SERLAKYNQL	LRIEEELGSE	AVYAGANFRK	PVEPY

Band No.: B15

NCBI accession No.: gi|255543357 Species: *Ricinus communis*

Protein name： heat shock protein, putative

Mascot score: 85 Sequence coverage %: 22

Matched peptides No.: 9 Total peptides No.: 17

Calculated Mr: 71965 Calculated *p*I: 5.27

Probability Based Mowse Score:

Protein score is -10*Log(P), where P is the probability that the observed match is a random event.
Protein scores greater than 73 are significant (p<0.05).

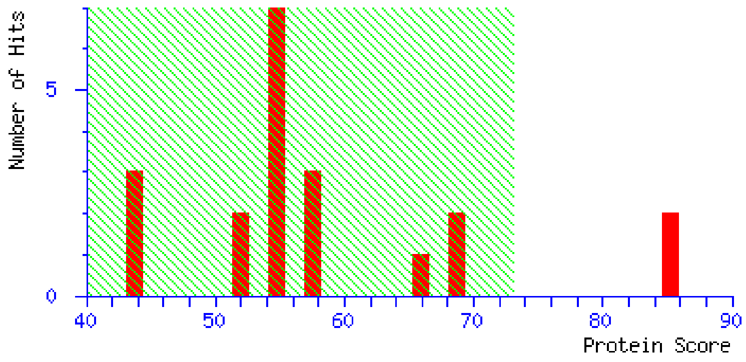

Matched peptide sequences: shown in Bold red:

1 MAKTEGKAVG IDLGTTYSCV GVWQNDRVEI IANDQGNRTT PSYVAFTDTE
51 RLIGDAAKNQ VAMNPQNTVF DAKRLIGRRF SDPSVQSDMK HWPFKVVPGP
101 GDKPMIVVLY KGEEKQFAPE EISSMVLIKM KEIAEAYLGQ TVKNAVITVP
151 AYFNDSQRQA TKDAGAISGL NVMRIINEPT AAAIAYGLDK KGSRSGEKNV
201 LIFDLGGGTF DVSLLTIEEG IFEVKATAGD THLGGEDFDN RLVNHFVAEF
251 RRKHKKDIST NARALRRLRT ACERAKRTLS STSQTTIEID SLYEGIDFYS
301 TITRARFEEL NMDLFRKCME PVEKCLRDSK IDKSQVDDIV LVGGSTRIPK
351 VQQLLQDFFN GKELCKSINP DEAVAYGAAV QAAILSGEGD QKVQELLLLD
401 VTPLSLGIET AGGVMTVLIP RNTTIPAKKE QIFSTYSDNQ PGVLIQVYEG
451 ERARTKDNNL LGTFELKGIP PAPRGVPQIN VCFDIDANGI LNVSAEDKTA
501 GVKNKITITN DKGRLSKEDI ERMVQEAEKY KAEDEQVKKK VEAKNALENY
551 AYNMRNTVKD EKFAGKLDSA DKQKIEKAID ETIEWLDGNQ LAEVDEFEDK
601 LKELEGLCNP IVSKMYQGGA GGDVPMGGGA KPGSGYGNAS SGGSGSGPKI
651 EEVD

Band No.: B16

NCBI accession No.: gi|225456951 Species: *Vitis vinifera*

Protein name： cell division cycle protein 48 homolog

Mascot score: 88 Sequence coverage %: 33

Matched peptides No.: 27 Total peptides No.: 121

Calculated Mr: 91020 Calculated *p*I: 5.12

Probability Based Mowse Score:

Protein score is -10*Log(P), where P is the probability that the observed match is a random event.
Protein scores greater than 73 are significant (p<0.05).

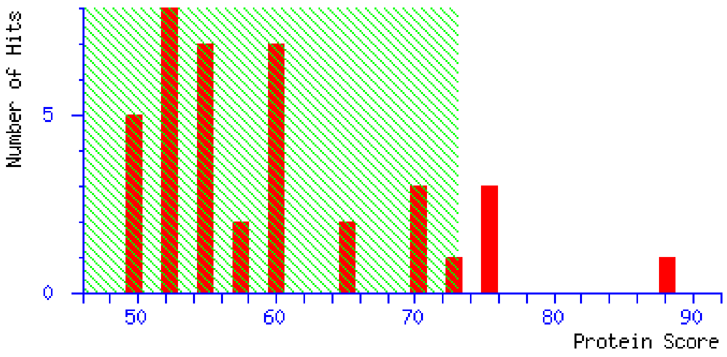

Matched peptide sequences: shown in Bold red

1	MADPSSSGPS	SSPEVKSVKK	DFSTAILERK	KSPNRLVVDE	AVNDDNSVVS
51	MNPATMEKLQ	FFRGDTVLIK	GKKRKDTVCI	VLVDEQCEEP	KIRMNKVVRA
101	NLRVRLGDVV	SVHQCPDVKY	GKRVHILPID	DTIEGVTGNL	FDAYLKPYFL
151	ESYRPVRKGD	LFLVRGGMRS	VEFKVIETDP	GEYCVVAPDT	EIFCEGEPIK
201	REDEERLNEV	GYDDVGGVRK	QMAQIRELVE	LPLRHPQLFK	SIGVKPPKGI
251	LLYGPPGSGK	TLIARAVANE	TGAFFFLING	PEIMSKLAGE	SESNLRKAFE
301	EAEKNAPSII	FIDELDSIAP	KREKTHGEVE	RRIVSQLLTL	MDGLKTRAHV
351	IVIGATNRPN	SIDPALRRFG	RFDREIDIGV	PDEVGRLEVL	RIHTKNMKLS
401	DDVDLERVAK	DTHGYVGADL	AALCTEAALQ	CIREKMDVID	LEDETIDAEV
451	LNSMAVTNEH	FQTALGSSNP	SALRETVVEV	PNVSWEDIGG	LDNVKRELQE
501	TVQYPVEHPE	KFEKFGMSPS	KGVLFYGPPG	CGKTLLAKAI	ANECQANFIS
551	VKGPELLTMW	FGESEANVRE	IFDKARQSAP	CVLFFDELDS	IATQRGSSVG
601	DAGGAADRVL	NQLLTEMDGM	TAKKTVFIIG	ATNRPDIIDP	ALLRPGRLDQ
651	LIYIPLPDEA	SRLQIFKACL	RKSPVSRDVD	LVALARYTHG	FSGADITEIC
701	QRSCKYAIRE	NIEKDIERER	KKTENPEAME	EDDVDDVPEI	KAAHFEESMK
751	FARRSVSDAD	IRKYQLFAQT	LQQSRGFGSE	FRFPDQPNNA	TAASTAADPF
801	SSAAAAGDDD	DLYS			

Band No.: B17

NCBI accession No.: gi|66735596 Species: *Arachis hypogaea*

PFF score: [67] Protein name: phospholipase D alpha

Matched peptides No.: [1] Sequence coverage %: [10]

Matched sequences: R.SIDGGAAFGFPETPEDAAR.A

Calculated Mr: 21036 Calculated *p*I: 5.22

Probability Based Mowse Score:

Ions score is -10*Log(P), where P is the probability that the observed match is a random event.
Individual ions scores > 44 indicate identity or extensive homology (p<0.05).
Protein scores are derived from ions scores as a non-probabilistic basis for ranking protein hits.

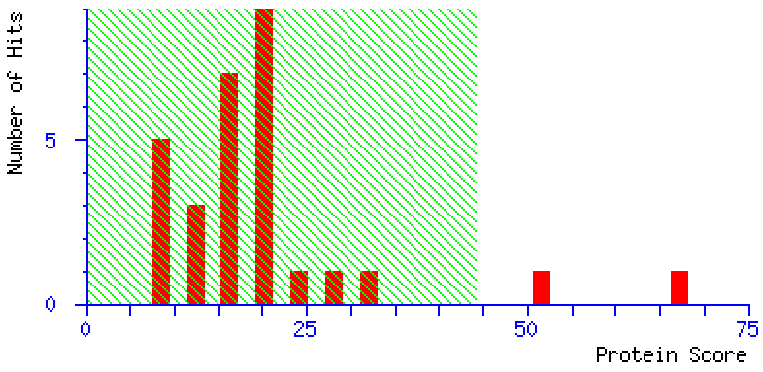

Matched peptide sequences: shown in Bold Red:

```
  1 HHDDFHQPNF AGASIMKGGP REPWHDIHSR LEGPIAWDVL FNFEQRWRKQ
 51 GGKDLLIPLR ELEDVIIPPS PVTFAEDQET WNVQLFRSID GGAAFGFPET
101 PEDAARAGLV SGKDNIIDRS IQDAYIHAIR RAKNFIYIEN QYFLGSCFGW
151 SPDDIKPEDI GALHLIPKEL SLKNVSKIEA GERFT
```

Band No.: B18

NCBI accession No.: gi|33326375 Species: *Hevea brasiliensis*

Protein name： heat shock protein

Mascot score: 90 Sequence coverage %: 28

Matched peptides No.:17 Total peptides No.: 52

Calculated Mr: 80380 Calculated *p*I: 5.02

Probability Based Mowse Score:

Protein score is -10*Log(P), where P is the probability that the observed match is a random event.
Protein scores greater than 73 are significant (p<0.05).

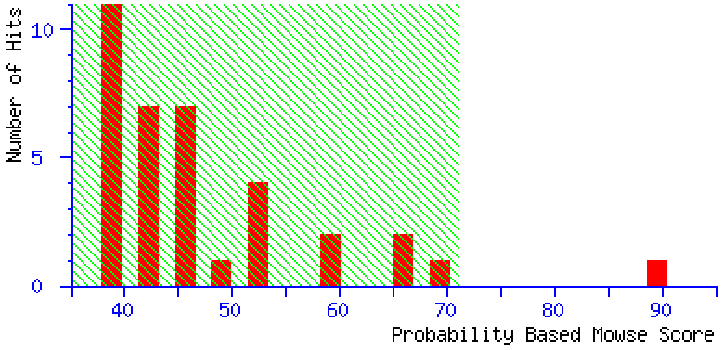

Matched peptide sequences: shown in **Bold red**

1 MADAETFAFQ AEINQLLSLI INTFYSNKEI FLREIISNAS DALDKIRFES

51 LTDKSK**LDAQ** **PELFIR**LVPD KANKTLSIID SGIGMTK**ADL** **VNNLGTIARS**

101 GTKEFMEALQ AGADVSMIGQ FGVGFYSAYL VAEKVIVTTK **HNDDEQYIWE**

151 **SQAGGSFTVT** RDVNGDQLGR GTK**ITLFLKE** **DQLEYLEER**R IKDLVKKHSE

201 FISYPIYLWT EKTTEKEISD DEDDEPKKEE EGDVEDVDEE KETKSKKKK**I**

251 **KEVSHEWQLI** **NKQKPIWLRK** **PEEITKEEYA** **SFYKSLTNDW** **EDHLAVKHFS**

301 **VEGQLEFK**AI LFVPK**RAPFD** **LFDTR**KKMSN IKLYVR**RVFI** **MDNCEELIPE**

351 **YLGFVKGVVD** **SDDLPLNISR** EMLQQNKILK VIRKNLVKKC IEMFNEIAEN

401 KEDYNKFYEA FSKNLKLGIH EDSQNRAKLA DLLR**YHSTKS** **GDEMTSLK**DY

451 VTRMKEGQKD IYYITGESKK **AVENSPFLER** LKKKGYEVLF MVDAIDEYAV

501 GQLKEYDGKK LVSATKEGLK LDDETEEEKK KKEEKKKSFE NLCKTIKDIL

551 GDKVEKVVVS DRIVDSPCCL VTGEYGWTAN MERIMKAQAL RDSSMSSYMS

601 SKK**TMEINPD** **NVIVEELRK**R AEADKNDKSV KDLVLLLFET ALLTSGFSLD

651 DPNTFSARIH RMLKLGLSID DDETAGDDAD MPALEEDGAE ESKMEEVD

Band No.: B19

NCBI accession No.: gi|450880 Species: *Arabidopsis thaliana*

Protein name： heat shock cognate 70-1

Mascot score: 107 Sequence coverage %: 47

Matched peptides No.: 28 Total peptides No.: 83

Calculated Mr: 70427 Calculated *p*I: 5.11

Probability Based Mowse Score:

Protein score is -10*Log(P), where P is the probability that the observed match is a random event. Protein scores greater than 73 are significant (p<0.05).

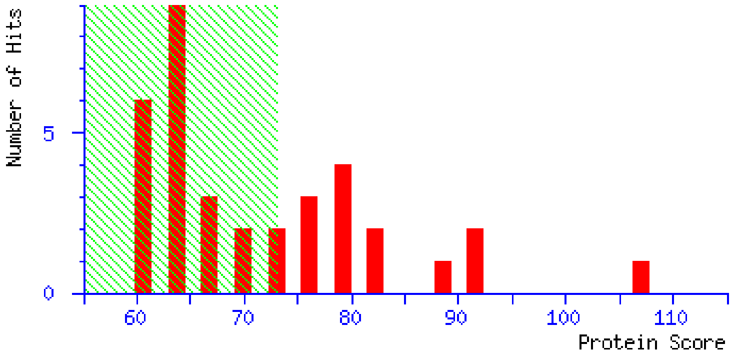

Matched peptide sequences: shown in Bold red

```
  1 GTTYSCVGVW QHDRVEIIAN DQGNRTTPSY VAFTDSERLI GDAAKNQVAM
 51 SPVNTVFDAK RLIGRRFSDS SVQSDMKLWP FKIQAGPADK PMIYVEYKGE
101 EKEFAAEEIS SMVLIKMREI AEAYLGVTIK NAVVTVPAYF NDSQRQATKD
151 AGVIAGLNVM RIINEPTAAA IAYGLDKKAT RVGEKNVLIF DLGGGTFDVS
201 LLTIEEGIFE VKATAGDTHL GGEDFDNRMV NHFVQEFKRK SKKDITGNPR
251 ALRRLRTSCE RAKRTLSSTA QTTIEIDSLY EGIDFYSTIT RARFEELNMD
301 LFRKCMEPVE KCLRDAKMDK STVHDVVLVG GSTRIPKVQQ LLQDFFNGKE
351 LCKSINPVEA VVYGAAVQGA ILSGEGNEKV QDLLLLDVTP LSLGLETAGG
401 VMTTLIPRNT TIPTKKEQVF STYSDNQPGV LIQVYEGERA RTKDNNLLGK
451 FELSGIPPAP RGVPQITVCF DIDANGILNV SAEDKTTGQK NKVTITNDKG
501 RLSKDEIEKM VQEAEKYKSE DEEHKKKVEA KNALENYAYN MRNTIQDEKI
551 GEKLPAADKK KIEDSIEQAI QWLEGNQLAE ADEFEDKMKE LESICNPIIA
601 KMYQGAGGEA GGPGASGMDD DAPPASGGAG PKIEEVD
```

Band No.: B20

NCBI accession No.: gi|379068234

Species: *Rhododendron formosanum*

Protein name： nucleotide-binding site leucine-rich repeat protein

Mascot score: 81 Sequence coverage %: 56

Matched peptides No.:12 Total peptides No.: 60

Calculated Mr: 28360 Calculated *p*I: 7.01

Probability Based Mowse Score:

Protein score is -10*Log(P), where P is the probability that the observed match is a random event. Protein scores greater than 73 are significant (p<0.05).

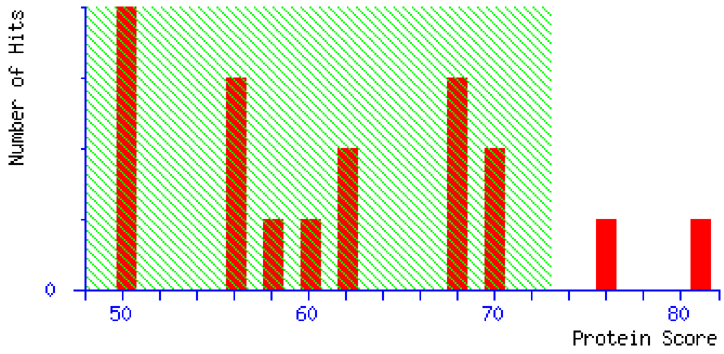

Matched peptide sequences: shown in **Bold red:**

```
  1 QVAKNTKEEK LFDDVVMATV SQNLDARKIQ GEIADLLGFK FEQESDSGRA
 51 DVLRGHLKQK ARVLVILDDV WKRFELNDIG IPFGEDHKGC KILVTSRNEE
101 VCNDMGAQKN FPVQILHKEE AWNLFKEMAG IPEDETNFRS TKMAVANECG
151 GLPIALVTVA RALKGKGKSS WGSALEALRK SIGKNVREVV DKVFKSLELS
201 FNFLKSKEAQ RCFLLCSLYS EDYDIPIEDL VRYGYGQKLF GGIKSVGEAR
251 A
```

Band No.: B21

NCBI accession No.: **gi|**14423688 Species: *Hevea brasiliensis*

Protein name：Enolase 1; 2-phosphoglycerate dehydratase 1

Mascot score: 101 Sequence coverage %: 35

Matched peptides No.: 10 Total peptides No.: 21

Calculated Mr: 48029 Calculated *p*I: 5.57

Probability Based Mowse Score:

Protein score is -10*Log(P), where P is the probability that the observed match is a random event.
Protein scores greater than 73 are significant (p<0.05).

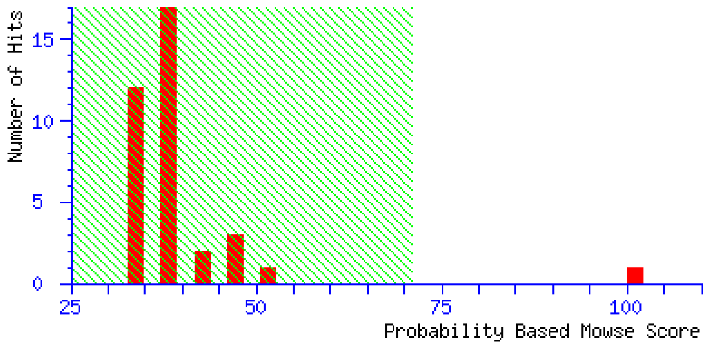

Matched peptide sequences: shown in Bold red

1 MAITIVSVRA R**QIFDSR**GNP TVEADVKLSD GYLAR**AAVPS** **GASTGIYEAL**
51 **ELRDGGSDYL** **GK**GVSKAVEN VNIIIGPALV GKDPTDQVGI DNFMVQQLDG
101 TVNEWGWCKQ KLGANAILAV SLAVCKAGAH VK**GIPLYEHI** **ANLAGNK**NLV
151 LPVPAFNVIN GGSHAGNKLA MQEFMILPVG ASSFKEAMKM GAEVYHHLKS
201 VIKKK**YGQDA** **TNVGDEGGFA** **PNIQENKEGL** **ELLK**TAIAKA GYTGKVVIGM
251 DVAASEFYGS DQTYDLNFKE ENNNGSQKIS GEALKDLYK**S** **FVAEYPIVSI**
301 **EDPFDQDDWA** **HYAKLTSEIG** **EKVQIVGDDL** **LVTNPK**RVEK AIKEKACNAL
351 LLKVNQIGSV TESIEAVKMS KR**AGWGVMAS** **HR**SGETEDTF IADLSVGLAT
401 GQIKTGAPCR SERLAK**YNQL** **LRIEEELGSE** **AVYAGANFR**K PVEPY

Band No.: B22

NCBI accession No.: **gi|3288200** Species: ***Hevea brasiliensis***

Protein name： latex allergen

Mascot score: 121 Sequence coverage %: 47

Matched peptides No.: 16 Total peptides No.: 62

Calculated Mr: 43020 Calculated *p*I: 5.00

Probability Based Mowse Score:

Protein score is -10*Log(P), where P is the probability that the observed match is a random event.
Protein scores greater than 73 are significant (p<0.05).

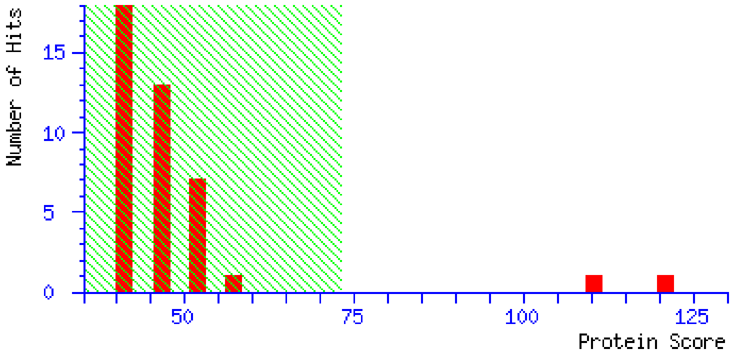

Matched peptide sequences: shown in Bold red

```
  1 MATGSTTLTQ GKKITVLSID GGGIRGIIPG IILASLESKL QDLDGPDARI
 51 ADYFDIIAGT STGGLITTML TAPNEDKKPI YQAKDIKDFY LENCPKIFPK
101 ESRDTYDPIH SIGPIYDGEY LRELCNNLLK DLTVKDTLTD VIIPAFDIKL
151 LLPVIFSSDD AKCNALKNAR LADVCISTSA APVLLPAHSF TTEDDKNIHT
201 FELIDGGVAA ANPTLLALTH IRNEIIRQNP RFIGANLTES KSRLVLSLGT
251 GKSEYKEKYN ADMTSKWRLY NWALYNGNSP AVDIFSNASS DMVDFHLSAL
301 FKSLDCEDYY LRIQDDTLTG EESSGHIATE ENLQRLVEIG TELLEKQESR
351 INLDTGRLES IPGAPTNEAA IAKFAKLLSE ERKLRQLK
```

Band No.: B23

NCBI accession No.: gi|113217 Species: *Daucus carota*

PFF score: [81] Protein name：Actin-1

Matched peptides No.: [2] Sequence coverage %: [7]

Matched sequences: R.AVFPSIVGRPR.H；K.NYELPDGQVITIGAER.F

Calculated Mr: 42246 Calculated *p*I: 5.64

Probability Based Mowse Score:

Ions score is -10*Log(P), where P is the probability that the observed match is a random event.
Individual ions scores > 44 indicate identity or extensive homology (p<0.05).
Protein scores are derived from ions scores as a non-probabilistic basis for ranking protein hits.

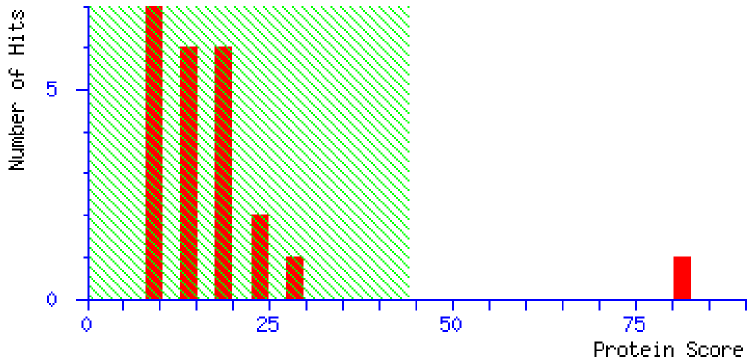

Matched peptide sequences: shown in Bold Red:

```
  1 MADGEDIQPL VCDNGTGMVK AGFAGDDAPR AVFPSIVGRP RHTGVMVGMG
 51 QKDAYVGDEA QSKRGIITLK YPIEHGIVSN WDDMRIWHHT FYNELRASPE
101 EHPVLLTEAP LNPKANREKM TQIMIETFNV PAMYVAIQAV LSLYASGRTT
151 GIVLDSGDGV SHTVPIYEGY ALPHAILRLD LAGRDLTDGL MKILTEKSIC
201 HYTTAEREIV RDMKEKLAYV ALDYEQELET AKRRSAVEKN YELPDGQVIT
251 IGAERFRCPQ VLFQPSMIGM ESAGIHETTY NSIMKCDVDI RKDLYGNIVL
301 SGGSTMFPGI ADRMSKEITA LAPSSMKIKV VAPPERKYSD LWIGGSILAS
351 LSTFQQMWIS KGEYDESGPS IVHRKCLLAG
```

Band No.: B24

NCBI accession No.: gi|147835357   Species: *Vitis vinifera*

Protein name： hypothetical protein

Mascot score: 84   Sequence coverage %: 34

Matched peptides No.: 25   Total peptides No.: 106

Calculated Mr: 97236   Calculated *p*I: 6.08

Probability Based Mowse Score:

Protein score is -10*Log(P), where P is the probability that the observed match is a random event. Protein scores greater than 73 are significant (p<0.05).

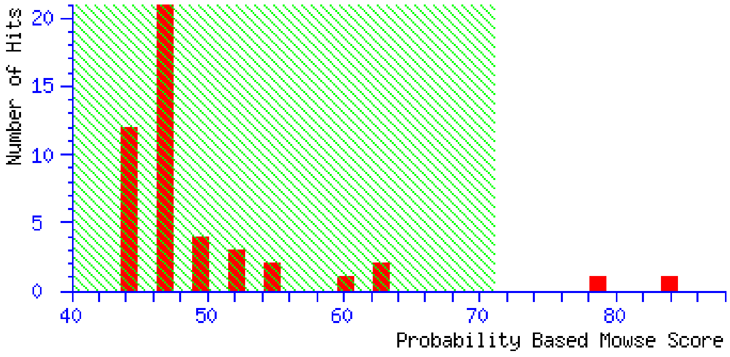

Matched peptide sequences: shown in Bold red:

```
  1 MVDAVVTVFL EKLLNALEEE GRVLFDFREQ FERLRDELRL MQSFLKDAER
 51 LKRKNETLRT VMIGLRELIY EAEDILADCK VQSEGSQDIS GWYAVCFYPT
101 NLPFKYQTGK RLREINEKIT RIKQNIPSFL GVPILSQAEI ADSRNLPVDR
151 WSSSVFDHSQ VVGIEGDTRK VKNWLLEAKD GILAIGVVGM GGVGKTTLAQ
201 VVFNDREMEA RFERRMWVSV TGTPNEKRIL RSMLRNLGDM NVGDDCGELL
251 RKINQYLLGK RFLLVMDDVG ENTNTWWRKI SDGLPKGNGS SIIITTRTKE
301 VATMMGVEEE RTHRPKVLSK DDSWLLFRNV AFAANGGICT SSELENIGRE
351 IVXKCGGLPL AIKAAGGMML YQQPYYHDWK RIADHFRDEL AEEDGSVMAS
401 LELSYEELPS HLKSCFLCLS LYPEDCEITK EQLIHWWIAE GFVPLRRGRL
451 STEAGEDCFS GLTNRCLIEV VEKSYTGAIQ TCKIHDMVRD LVIKKAEDDA
501 FSGPTTASCR HLGIEGDIDR KYDMPNQKLR ALLSTIKTGE VNKVASSNAK
551 KFCDCRYLRV LDXSKTIFDK SLTGLLDHIG FLQHLTYLSL SNTHPLTEVP
601 PALEXLRNLQ VLDLSYCQNL KMLPSYVTTF EKLTVLDVSH CGSLRYLPKG
651 LGSLSNLQVL LGFKPSKSNQ LEGCRIAELR SLTKLRRLGL QLTQGDEIGD
701 NDDNVLVGLR GLQFLVISCF DSHGDDLIPK LDKLSPPQQL HELSLRFYPG
751 KMNPGWLNPF SLPILRYLSI SSGNLTNMSQ RFWGDGDNTW KIEGLMLESL
801 SDLGMEWSMV QQVMPRLRIV NVSWCPDLDS FPIEDVGFRG GVWKKGERPS
```

Band No.: B25

NCBI accession No.: **gi**|170525118 Species: *Physalis mendocina*

Protein name： geranylgeranyl reductase

Mascot score: 81 Sequence coverage %: 76

Matched peptides No.: 7 Total peptides No.: 54

Calculated Mr: 5.67 Calculated *p*I: 11741

Probability Based Mowse Score:

Protein score is -10*Log(P), where P is the probability that the observed match is a random event. Protein scores greater than 73 are significant (p<0.05).

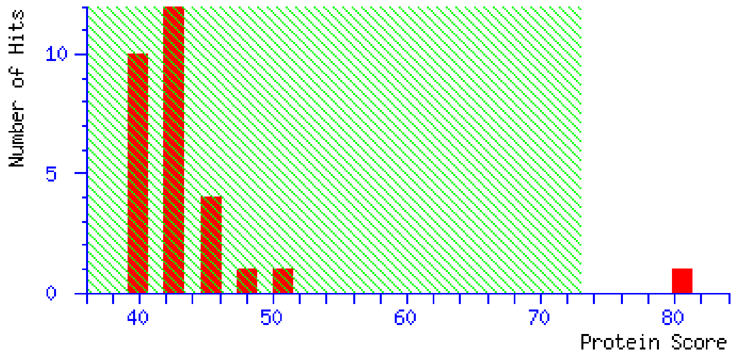

Matched peptide sequences: shown in Bold red:

```
  1 LKPHEYIGMV RREVLDAYLR DRAAEAGASV LNGLFLKMDM PKAPNAPYVL
 51 HYSAYDSKTN GAGEKRTPEV DAVIGADGAN SRVAKSINAG DYEYAIAFQE
101 RIRISDD
```

Band No.: B26

NCBI accession No.: gi|145346735

Species: *Ostreococcus lucimarinus CCE9901*

Protein name： predicted protein

Mascot score: 83 Sequence coverage %: 22

Matched peptides No.: 9 Total peptides No.: 25

Calculated Mr: 43211 Calculated *p*I: 8.11

Probability Based Mowse Score:

Protein score is -10*Log(P), where P is the probability that the observed match is a random event. Protein scores greater than 73 are significant (p<0.05).

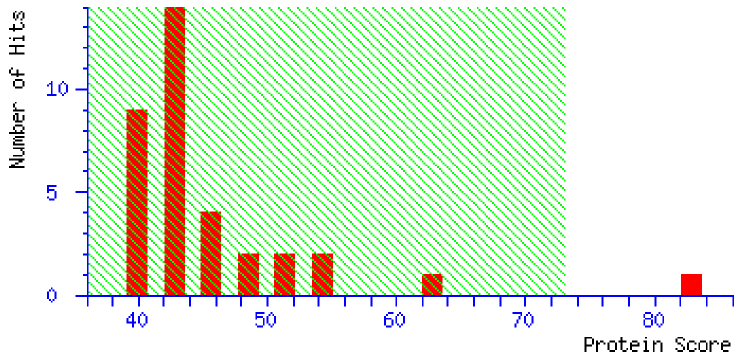

Matched peptide sequences: shown in Bold red:

```
  1 MSGGARCNVL PTSATANDFT TESANRRLKA VLASWSVERC REWLETEIGL
 51 GLGIEIASNK YFPLSNSSRE VRDRLVAACE KAGVEFKYET SVDGVVRGSD
101 GMGWTLRVRG SGDERARAIV LAMGGLSFPA VGTDGTGYAI ARRDLGHALH
151 EPYPALVPLT GPHPGGEPLP GVSVEVELKV AAANADGGGG KKKAQAIRKG
201 FLFTHRGYSG PSVLDLSHHL VRPLVRANES KGGSLRDEDF DAPTSSSPRM
251 VINWSGIKRE EWQERLTAPP GRALVVSRLR EALPSRLADA LVAESGVDSR
301 CKIAELKKDD RAKLLRVLTE YEIAVKGHQG YRKAEVTGGG VALDELDTAS
351 MASLKAPGIY MCGEVCDVFG RIGGFNFLWA WCSGRLAGMS AAKAISSADD
401 E
```

Band No.: B27

NCBI accession No.: gi| 37903393

Species: *Saccharum hybrid cultivar CP65-357*

PFF score: [101]

Protein name：14-3-3-like protein

Matched peptides No.: [1]

Sequence coverage %: [4]

Matched sequences: K.TVDVEELTVEER.N

Calculated Mr: 28978

Calculated *p*I: 4.79

Probability Based Mowse Score:

Ions score is -10*Log(P), where P is the probability that the observed match is a random event.
Individual ions scores > 45 indicate identity or extensive homology (p<0.05).
Protein scores are derived from ions scores as a non-probabilistic basis for ranking protein hits.

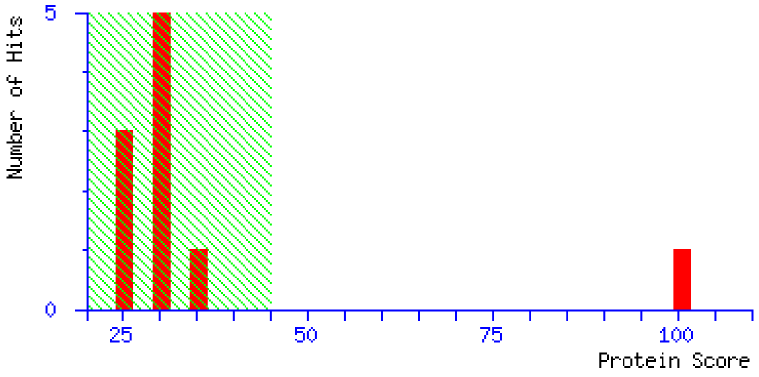

Matched peptide sequences: shown in Bold Red:

```
  1 MSREENVYMA KLAEQAERYE EMVEYMEKVA KTVDVEELTV EERNLLSVAY
 51 KNVIGARRAS WRIVSSIEQK EESRKNEEHV NLIKEYRGKI EAELSNICDG
101 ILKLLDSHLV PSSTAAESKV FYLKMKGDYH RYLAEFKTGA ERKESAESTM
151 VAYKAAQDIA LAELAPTHPI RLGLALNFSV FYYEILNSPD KACNLAKQAF
201 DEAISELDTL GEESYKDSTL IMQLLRDNLT LWTSDLTEDG ADEGKEASKG
251 DAGEGQ
```

Band No.: B28

NCBI accession No.: gi|10946499 Species: *Hevea brasiliensis*

Protein name： beta-1,3-glucanase

Mascot score: 181 Sequence coverage %: 66

Matched peptides No.:18 Total peptides No.:45

Calculated Mr: 35295 Calculated *p*I: 9.46

Probability Based Mowse Score:

Protein score is -10*Log(P), where P is the probability that the observed match is a random event. Protein scores greater than 73 are significant ($p<0.05$).

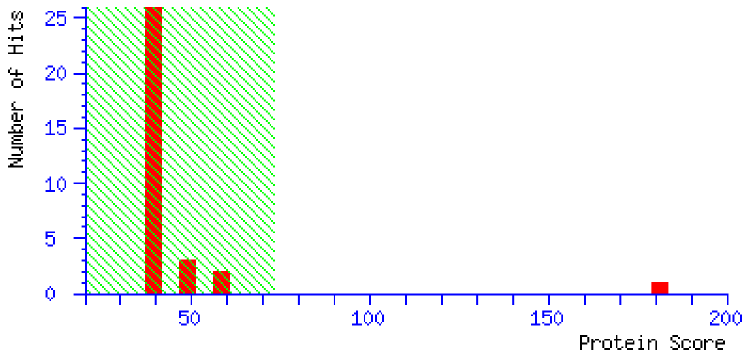

Matched peptide sequences: shown in Bold red:

```
  1  QVGVCYGMQG NNLPPVSEVI ALYKKSNITR MRIYDPNRAV LEALRGSNIE
 51  LILGVPNSDL QSLTNPSNAK SWVQKNVRGF WSSVLFRYIA VGNEISPVNR
101  GTAWLAQFVL PAMRNIHDAI RSAGLQDQIK VSTAIDLTLV GNSYPPSAGA
151  FRDDVRSYLN PIIRFLSSIR SPLLANIYPY FTYAGNPRDI SLPYALFTSP
201  SVVVWDGQRG YKNLFDATLD VLYSALERAS GGSLEVVVSE SGWPSAGAFA
251  ATFDNGRTYL SNLIQHVKRG TPKRPNRAIE TYLFAMFDEN KKQPEVEKQF
301  GLFFPDKWQK YNLNFG
```

Band No.: B29

NCBI accession No.: gi|124365253 Species: *Hevea brasiliensis*

Protein name： beta-1,3-glucanase

Mascot score: 105 Sequence coverage %: 38

Matched peptides No.: 10 Total peptides No.: 35

Calculated Mr: 41543 Calculated *p*I: 9.4

Probability Based Mowse Score:

Protein score is -10*Log(P), where P is the probability that the observed match is a random event.
Protein scores greater than 73 are significant (p<0.05).

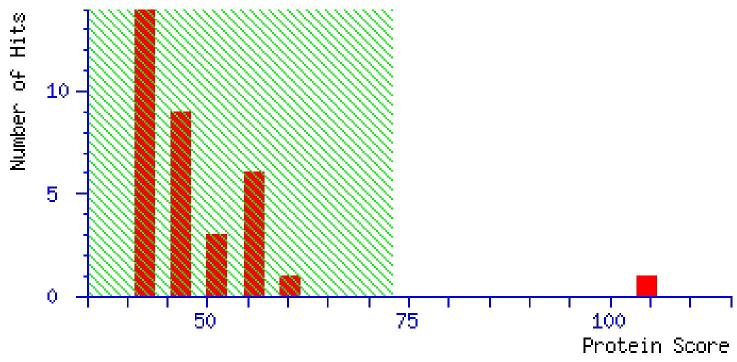

Matched peptide sequences: shown in Bold red:

1 MAISSSTSGT SSSLPSRTTV MLLLFFFTAS VGITDAQVGV CYGMQGNNLP
51 PVSEVIALYK KSNITRMR**IY** **DPNQAVLEAL** **R**GSNIELILG VPNSDLQSLT
101 NPSNAKSWVQ KNVRGFWSSV RFR**YIAVGNE** **ISPVNR**GTAW LAQFVLPAMR
151 **NIHDAIRSAG** **LQDQIKVSTA** **IDLTLVGNSY** **PPSAGAFR**DD VR**SYLNPIIR**
201 **FLSSIR**SPLL ANIYPYFTYA GNPR**DISLPY** **ALFTSPSVVV** **WDGQR**GYK**NL**
251 **FDATLDALYS** **ALERASGGSL** **EVVVSESGWP** **SAGAFAATFD** **NGR**TYLSNLI
301 QHVKRGTPKR PKRAIETYLF AMFDENKKQP EVEKHFGLFF PNKWQKYNLN
351 FSAEKNWDIS TEHNATILFL KSDM

Band No.: B30

NCBI accession No.: gi|14423933 Species: *Hevea brasiliensis*

Protein name：Small rubber particle protein; Short=SRPP; AltName: Full=22 kDa rubber particle protein; Short=22 kDa RPP; AltName: Full=27 kDa natural rubber allergen; AltName: Full=Latex allergen Hev b 3; AltName: Allergen=Hev b 3

Mascot score: 130 Sequence coverage %: 51

Matched peptides No.:10 Total peptides No.: 26

Calculated Mr: 22331 Calculated *p*I: 4.80

Probability Based Mowse Score:

Protein score is -10*Log(P), where P is the probability that the observed match is a random event. Protein scores greater than 73 are significant ($p<0.05$).

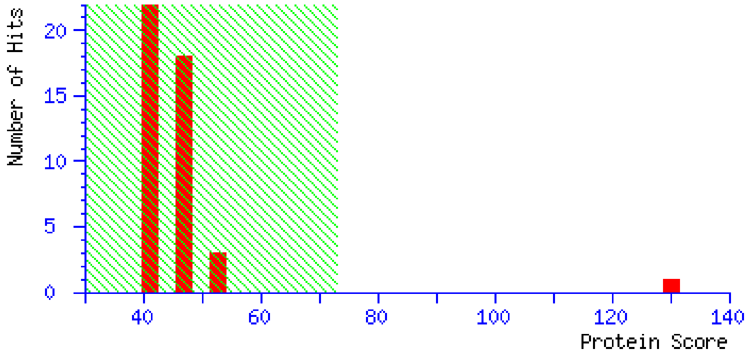

Matched peptide sequences: shown in Bold red:

```
  1 MAEEVEEERL KYLDFVRAAG VYAVDSFSTL YLYAKDISGP LKPGVDTIEN
 51 VVKTVVTPVY YIPLEAVKFV DKTVDVSVTS LDGVVPPVIK QVSAQTYSVA
101 QDAPRIVLDV ASSVFNTGVQ EGAKALYANL EPKAEQYAVI TWRALNKLPL
151 VPQVANVVVP TAVYFSEKYN DVVRGTTEQG YRVSSYLPLL PTEKITKVFG
201 DEAS
```

Band No.: B31

NCBI accession No.: gi|313586569 Species: *Hevea brasiliensis*

Protein name： hypothetical protein 19

Mascot score: 132 Sequence coverage %: 48

Matched peptides No.: 12 Total peptides No.: 36

Calculated Mr: 20724 Calculated *p*I: 7.77

Probability Based Mowse Score:

Protein score is -10*Log(P), where P is the probability that the observed match is a random event. Protein scores greater than 73 are significant (p<0.05).

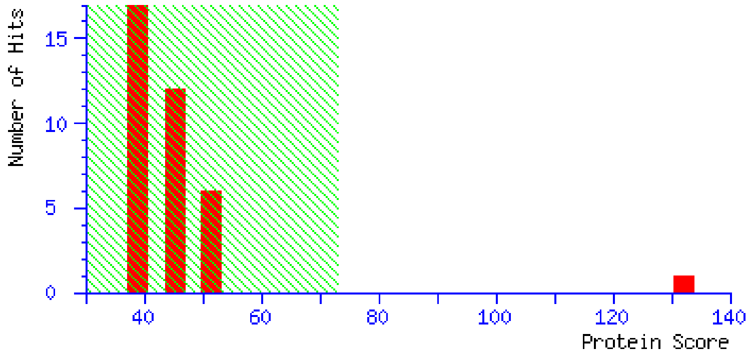

Matched peptide sequences: shown in Bold red:

```
  1 MEELGSSHEK TESESSHCRK PHLPSGSYLS KKPSYKVYCK ADPNFFLTIR
 51 DGKVVLAPPD PSDEFQHWYK DEKFSTMVKD EEGFPSFALV NKASGKALKH
101 SFGPTHPVLL TPYDPDDLEA SILWTESKDL GDGYRIVRML NNIRLNVEAL
151 RGDKKSGGVS NGTKVVLAYW NKGDHQKWRI APL
```

Band No.: B32

NCBI accession No.: gi|327492451

Species: *Mesembryanthemum crystallinum*

Protein name： N-terminal end of polyubiquitin

Mascot score: 162

Sequence coverage %: 96

Matched peptides No.: 12

Total peptides No.: 38

Calculated Mr: 8463

Calculated *p*I: 6.56

Probability Based Mowse Score:

Protein score is -10*Log(P), where P is the probability that the observed match is a random event.
Protein scores greater than 73 are significant ($p<0.05$).

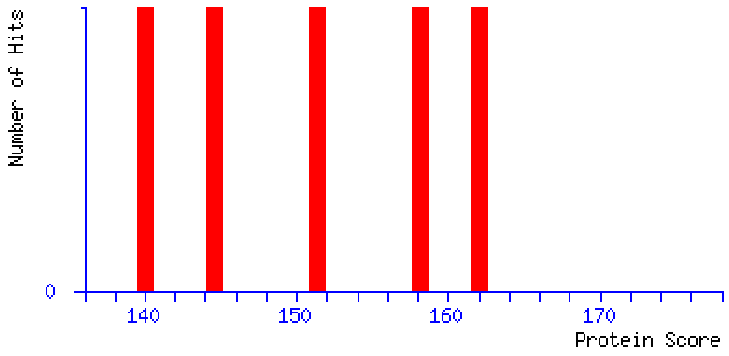

Matched peptide sequences: shown in Bold red:

```
 1 MQIFVKTLTG KTITLEVESS DTIDNVKAKI QDKEGIPPDQ QRLIFAGKQL
51 EDGRTLADYN IQKESTLHLV LRLRG
```

Band No.: B33

NCBI accession No.: **gi**|27449246 Species: *Hevea brasiliensis*

Protein name： Cu/Zn superoxide dismutase

Mascot score: 87 Sequence coverage %: 38

Matched peptides No.: 6 Total peptides No.: 22

Calculated Mr: 15726 Calculated *p*I: 5.73

Probability Based Mowse Score:

Protein score is -10*Log(P), where P is the probability that the observed match is a random event.
Protein scores greater than 73 are significant (p<0.05).

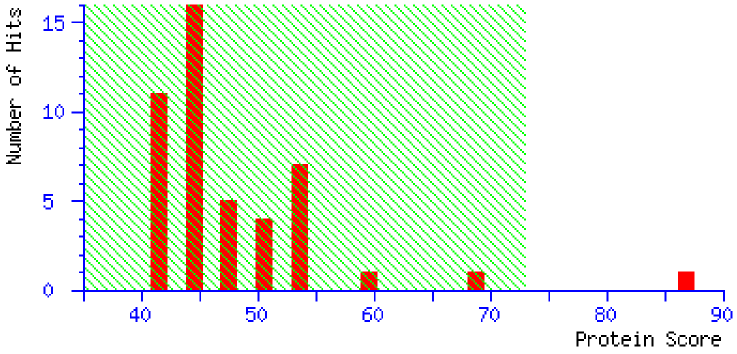

Matched peptide sequences: shown in Bold red:

```
  1 MLKAVAVITS SEGISGKIFF TQEGDGPTTV TGSVSGLKPG LHGFHVHTFG
 51 DTTNGCLSTG LHFNPASKDH GGPEDENRHA GDLGNVNVGD DGTANFTIVD
101 KHIPLSGPHS IAGRSVVFHE GRDDLGKGGH ELSKITGNAG DRIACGIIGL
151 QE
```

Band No.: B34

NCBI accession No.: gi| 29170601 Species: *Hevea brasiliensis*

Protein name： small rubber particle protein

Mascot score: 84 Sequence coverage %: 61

Matched peptides No.: 7 Total peptides No.: 31

Calculated Mr: 12769 Calculated *p*I: 4.61

Probability Based Mowse Score:

Protein score is -10*Log(P), where P is the probability that the observed match is a random event. Protein scores greater than 73 are significant (p<0.05).

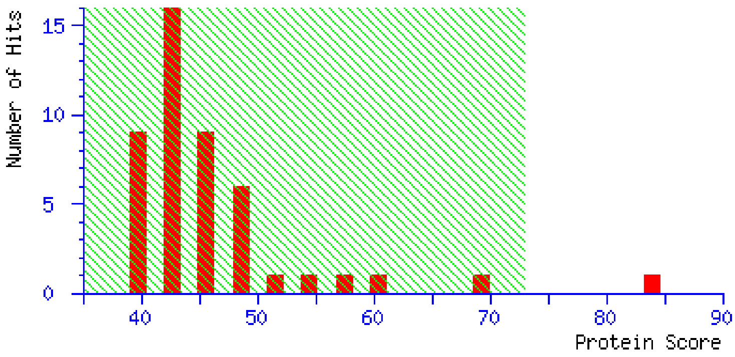

Matched peptide sequences: shown in Bold red:

```
  1 MAEGKENENF QQEANEQEEK LKYLEFVQAT TDNAVTALSN IYLYAKDNSG
 51 PLKPGVETIE GVAKTVVIPA SKIPTEAIKF ADRAVDASFT TLQNIVPSVL
101 KQLPTQACDT SVKESAE
```

Spot No.: B35

NCBI accession No.: gi|242080683 Species: *Sorghum bicolor*

PFF score: [118]

Protein name： hypothetical protein SORBIDRAFT_07g004260

Matched peptides No.: [2] Sequence coverage %: [15]

Matched sequences: M.GLQEEFEEYAEK.A; M.GLQEEFEEYAEKAK.T

Calculated Mr: 10341 Calculated *p*I: 5.01

Probability Based Mowse Score:

Ions score is -10*Log(P), where P is the probability that the observed match is a random event.
Individual ions scores > 45 indicate identity or extensive homology (p<0.05).
Protein scores are derived from ions scores as a non-probabilistic basis for ranking protein hits.

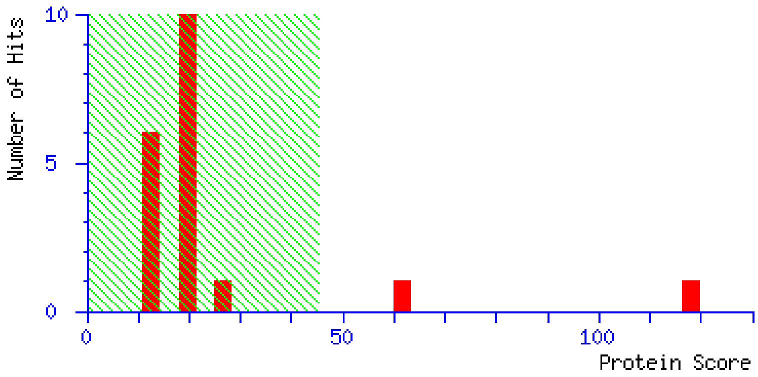

Matched peptide sequences: shown in Bold Red:

1 **MGLQEEFEEY AEKAK**TLPES TSNENKLILY GLYKQATVGN VNTDRPGIFY
51 QKDRAKWDAW KAVEGKPKEE AMNDYITKVK QLQEEAAAAT S

Supplementary Figure S2

Figure S2:

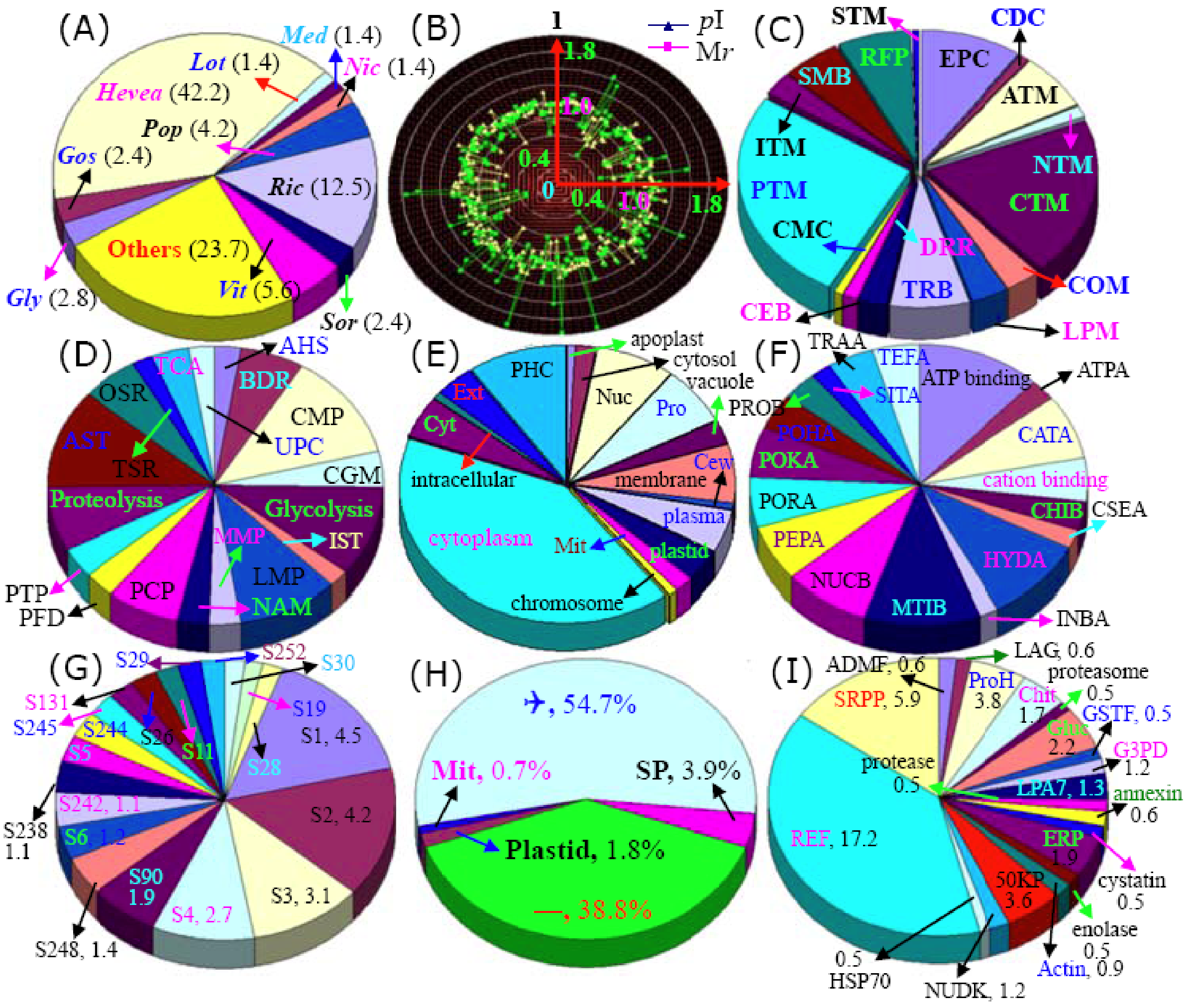

Figure S2: Classification and functional analysis of the identified main latex proteins. A total of 287 proteins including 184 unique proteins were positively identified from 252 spots and 35 bands by MS. The distributions of the identified proteins in different plant species were demonstrated (A). To evaluate the quality of identified proteins, the theoretical and experimental ratios of molecular mass (M*r*) and isoelectric point (*p*I) were calculated and presented in radial chart as radial and annular radar axis labels respectively (B). Then, each protein was functionally classified by COG and the proportion of each functional category was shown (C). The GO classification results including biological process (D), cellular component (E), and molecular function (F) were presented. The cellular locations of all the identified

proteins were also determined by TargetP (H). To detect the protein expression abundance in rubber latex, the most abundant 20 protein spots (G) as well as the most abundant 20 kinds of proteins on the reference 2-DE gel were also determined. The spot number (S), protein name and the volume value (Vol%) in the reference 2-DE gel were presented.

The abbreviations used in this study were listed as the following.

The abbreviations for proteins:

ABAS, abscisic acid stress ripening-related protein; ACAT, Acetyl-CoA C-acetyltransferase; ACPX, ascorbate peroxidase; ADMF, actin depolymerizing factor; ADPF, actin-depolymerizing factor; APTS, asparaginyl-tRNA synthetase; ATPS, ATP synthase subunit beta; CAMT, caffeic acid 3-O-methyltransferase; CDCP, cell division cycle protein; CDPK, calcium-dependent protein kinase; Chit, chitinase; CIAD, cinnamyl alcohol dehydrogenase; CIBP, citrate-binding protein; CIPT, cis-prenyl transferase; CLBP, calcium lipid binding protein; COPC, COP9 signalosome complex subunit 1; COPT, copper transport protein; CVMP, coated vesicle membrane protein; CYAS, beta-cyanoalanine synthase; CYSP, cysteine protease; DMAPP, dimethylallyl diphosphate; ELRP, elicitor-responsive protein; ETEF, eukaryotic translation elongation factor; ETIF, eukaryotic translation initiation factor; ETHI, ethylene-inducible protein; ETHR, ethylene receptor; FADS, farnesyl diphosphate synthase; GLUD, glutaredoxin; GGCS, gamma-glutamylcysteine synthetase; GGDS, geranylgeranyl diphosphate synthase; Gluc, beta-1,3-glucanase; GLUR, glutathione reductase; GLUS, glutamine synthetase; GSGT, galactinol-sucrose galactosyltransferase; GSTF, glutathione-S-transferase; G3PD, glyceraldehyde 3-phosphate dehydrogenase; G6PD, glucose-6-phosphate dehydrogenase; G6PE, glucose-6-phosphate 1-epimerase; G6PI, glucose-6-phosphate isomerase; HMGR, hydroxymethylglutaryl coenzyme A reductase; HMGS, hHydroxymethylglutaryl coenzyme A synthase; HSP 70, heat shock protein 70; HYDL, hydroxynitrile lyase; LACG, latex cyanogenic beta glucosidase; LACT, latex cystatin; LAP, latex abundant protein; LAG, latex allergen; LPA7, latex protein allergen Hev b 7; MEVD, mevalonate disphosphate decarboxylase; MEVK, mevalonate kinase; MIPS, myo-inositol-1-phosphate synthase; NUDK, nucleoside diphosphate kinase; PDBP, pyridoxal biosynthesis protein; PHOK, phosphomevalonate kinase; PHOT, phosphoglyceride transfer family protei; PMPP, pyrophosphate-energized membrane proton pump; PPCI, peptidyl-prolyl cis-trans isomerase; ProH, prohevein; PROI, protease inhibitor protein; PseuH, pseudo-hevein; RAPK, receptor for activated protein kinase; REF, rubber elongation factor; RRMP, RNA recognition motif-containing protein; SOD, superoxide dismutase; SRPP, small rubber particle protein; SUCT, sucrose transporter; TMAP, translation machinery-associated protein; UBCE, ubiquitin-conjugating enzyme; UBPL,

ubiquitin-protein ligase; VHAE, V-type proton ATPase subunit E; VHAF, V-type proton ATPase subunit; V-PPase, vacuolar-type inorganic pyrophosphatase; 50KP, 50 kDa protein.

The abbreviations for COG classification of each protein:

EPC, Energy production and conversion; CDC, Cell division and chromosome partitioning; ATM, Amino acid transport and metabolism; NTM, Nucleotide transport and metabolism; CTM, Carbohydrate transport and metabolism; COM, Coenzyme transport and metabolism; LPM, Lipid metabolism; TRB, Translation, ribosomal structure and biogenesis; DRR, DNA replication, recombination and repair; CMC, Cell envelope biogenesis, outer membrane/ G Carbohydrate transport and metabolism; PTM, Posttranslational modification, protein turnover, chaperones.

The abbreviations for GO-Biological process:

AHS, ATP hydrolysis and synthesis; BDR, biotic defense response; CMP, carbohydrate metabolic process; CGM, cellulose biosynthetic and glucose metabolic process; IST, intracellular signal transduction; LMP, lipid metabolic process; MMP, malate metabolic process; NAM, nuclear acid metabolic process; PCP, protein catabolic process; PFD, protein folding; PTP, protein transport; AST, abiotic stress response; OSR, oxidative stress response; TRR, translational regulation; TCA, tricarboxylic acid cycle; UPC, ubiquitin-dependent protein catabolic process.

The abbreviations for GO-Cellular component:

Nuc, nucleus; Pro, proteasome;Cew, cell wall;Mit, mitochondria; Cyt, cytoskeleton; Ext, extracellular region; PHC, phosphopyruvate hydratase complex.

The abbreviations for GO-Molecular function:

ATPA, ATPase activity; CATA, catalytic activity; CHIB, chitin binding; CSEA, cysteine synthase and endopeptidase activity; HYDA, hydrolase activity; INBA, inhibitor activity; MTIB, metal ion binding; NUCB, nucleotide binding; PEPA, peptidase activity; PORA, peroxidase and reductase activity; POKA, phosphate kinase activity; POHY, phosphopyruvate hydratase activity; PROB, protein binding; SITA, signal transducer activity;TRAA, transferase activity; TEFA, translation elongation factor activity.

The abbreviations for the cellular location in TargetP:

Mit, Mitochondrion, i.e. the sequence contains mTP, a mitochondrial targeting peptide; Plastid, Chloroplast or other plastids,, i.e. the sequence contains cTP, a chloroplast transit peptide; _, Any other location; Q , Don't know location, indicates that cutoff restrictions were set and the winning network output score was below the requested cutoff for that category; SP, Secretory pathway, i.e. the sequence contains SP, a signal peptide.

Supplementary Figure S3

Supplemental spectra and MALDI TOF/TOF MS /MS identification information for the ethylene responsive latex proteins

Annotated spectra 143 differential latex proteins on DIGE gels identified by PMF and the combination results of PMF and PFF.

CID: collision induced desorption;

MALDI TOF:

matrix assisted laser desorption/ionization time of flight;

MS: mass spectrometry;

PMF: peptide mass fingerprinting;

PFF: peptide fragment fingerprinting.

Spot No.: **D1**

NCBI accession No.: **gi|255545912** Species: *Ricinus communis*

PFF score: [278]

Protein name: 14-3-3 protein, putative

Matched peptides No.: [3] Sequence coverage %: [17]

Matched sequences: R.IVSSIEQKEEGR.K;
K.LVLGSTPSGELTVEER.N;
K.AAQDIALADLAPTHPIR.L

Calculated Mr: **28647** Calculated *p*I: **4.71**

Probability Based Mowse Score:

Ions score is -10*Log(P), where P is the probability that the observed match is a random event.
Individual ions scores > 46 indicate identity or extensive homology (p<0.05).
Protein scores are derived from ions scores as a non-probabilistic basis for ranking protein hits.

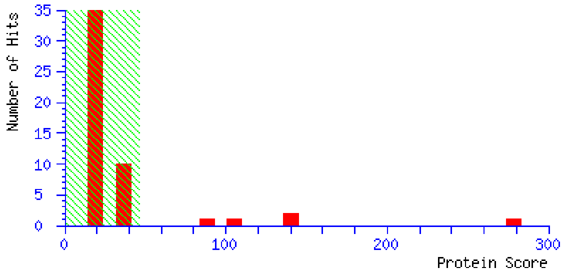

Matched peptide sequences: shown in Bold Red:

```
  1  MAAAATVPDN LTRDQYVYLA KLAEQAERYE EMVQFMQKLV LGSTPSGELT
 51  VEERNLLSVA YKNVIGSLRA AWRIVSSIEQ KEEGRKNEEH VVLVKEYRSK
101  VENELSDVCA SILSLLDSNL IPSASASESK VFYLKMKGDY HRYLAEFKAG
151  DERKTAAEDT MLAYKAAQDI ALADLAPTHP IRLGLALNFS VFYYEILNQS
201  DKACSMAKQA FEEAIAELDT LGEESYKDST LIMQLLRDNL TLWTSDVQDQ
251  LDEP
```

Spot No.: **D2**

NCBI accession No.: **gi|37719658** Species: *Hevea brasiliensis*

Protein name: **50 kDa protein**

Mascot score: **93** Sequence coverage %: **31**

Matched peptides No.: **9** Total peptides No.: **33**

Calculated Mr: **41480** Calculated *p*I: **4.60**

Probability Based Mowse Score:

Protein score is -10*Log(P), where P is the probability that the observed match is a random event. Protein scores greater than 73 are significant (p<0.05).

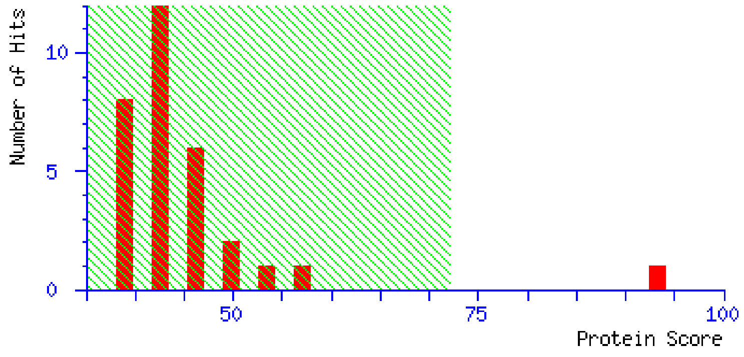

Matched peptide sequences: shown in Bold Red:

```
  1 MASLAYSLFI LSLFTFTLLN PVCSELDEYL FSFGDGLYDA GNAKFIYPDK
 51 YLPSYHHPYG TTFFDYPTGR FSDGRTVVDF VAENVSLPRI PPFKNKEANF
101 TYGANFASEG ATASDSNPLI DFRSQIRDFG ELKLEWAVQL VNVTELARRL
151 KKAVYLISFG ADDYLNYEIP SEASREQLES IVDVVLGNIS DRIKELYDFG
201 ARKFVVENVA PLGLIPFIKQ TSDNSTLFYE LASLHAMKLP QILEKIQDGY
251 LFPEFNYTVF NYFGIIKEII DAPGEHGFKY GDIACCGNST YRGQACGFLD
301 YEFCVCGNKT EYLFFDGTHN TDAANNLLAE LMWDKESGFI SPYGVKDFFP
351 SPTTIQTLLT EATALG
```

Spot No.: **D3**

NCBI accession No.: **gi|37719658** Species: *Hevea brasiliensis*

Protein name: **50 kDa protein**

Mascot score: **93** Sequence coverage %: **36**

Matched peptides No.: **11** Total peptides No.: **44**

Calculated Mr: **41480** Calculated *p*I: **4.60**

Probability Based Mowse Score:

Protein score is -10*Log(P), where P is the probability that the observed match is a random event. Protein scores greater than 73 are significant (p<0.05).

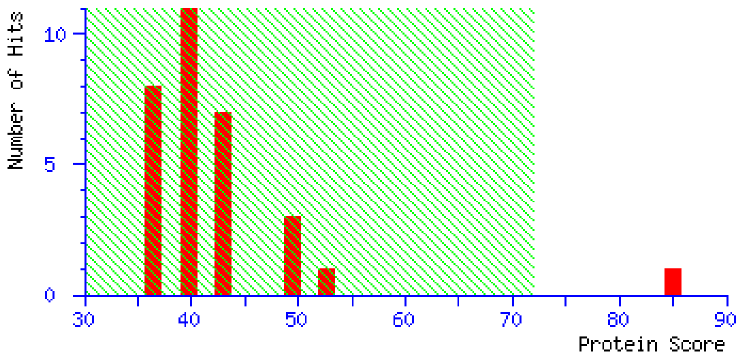

Matched peptide sequences: shown in Bold Red:

```
  1 MASLAYSLFI LSLFTFTLLN PVCSELDEYL FSFGDGLYDA GNAKFIYPDK
 51 YLPSYHHPYG TTFFDYPTGR FSDGRTVVDF VAENVSLPRI PPFKNKEANF
101 TYGANFASEG ATASDSNPLI DFRSQIRDFG ELKLEWAVQL VNVTELARRL
151 KKAVYLISFG ADDYLNYEIP SEASREQLES IVDVVLGNIS DRIKELYDFG
201 ARKFVVENVA PLGLIPFIKQ TSDNSTLFYE LASLHAMKLP QILEKIQDGY
251 LFPEFNYTVF NYFGIIKEII DAPGEHGFKY GDIACCGNST YRGQACGFLD
301 YEFCVCGNKT EYLFFDGTHN TDAANNLLAE LMWDKESGFI SPYGVKDFFP
351 SPTTIQTLLT EATALG
```

Spot No.: **D4**

NCBI accession No.: **gi|147805616**   Species: *Vitis vinifera*

PFF score: [324]

Protein name: hypothetical protein VITISV_028800

Matched peptides No.: [4]   Sequence coverage %: [14]

Matched sequences: R.GPYPADQVVR.D;

K.GCFFTTSGGLR.S;

R.DRGPYPADQVVR.D;

K.TANEVIIVIEAYR.T

Calculated Mr: **27385**   Calculated *p*I: **5.64**

Probability Based Mowse Score:

Ions score is -10*Log(P), where P is the probability that the observed match is a random event.
Individual ions scores > 46 indicate identity or extensive homology (p<0.05).
Protein scores are derived from ions scores as a non-probabilistic basis for ranking protein hits.

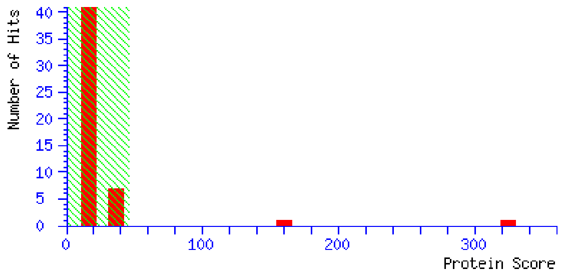

Matched peptide sequences: shown in **Bold Red:**

```
  1 MLAVFHKSIA EAPEGLRTPD SASLPALKDG FLPQFFASLH PSAVTVNLGS
 51 SGAISYSVDK QNPLLPRLFA VVDDIFCLFQ GHIENVALLK QQYGLNKTAN
101 EVIIVIEAYR TLRDRGPYPA DQVVRDLHGK FAFVLYDSSN RTAFLAADAD
151 ESVPFFWGVD SEGHLVLSDD EETVKKGCGK SFAPFPKGCF FTTSGGLRSF
201 EHPLNELRAE PRVDSSGQVC GANFKVDVEA KKETGMPRVG SAANWSTHY
```

Spot No.: **D5**

NCBI accession No.: **gi|224137430**

Species: *Populus trichocarpa*

Protein name: predicted protein PFF score: [122]

Matched peptides No.: [2] Sequence coverage %: [6]

Matched sequences: R.VYKDGTIER.L;

R.LLVIVAEKDILR.D

Calculated Mr: **35782** Calculated *p*I: **5.30**

Probability Based Mowse Score:

Ions score is -10*Log(P), where P is the probability that the observed match is a random event.
Individual ions scores > 45 indicate identity or extensive homology (p<0.05).
Protein scores are derived from ions scores as a non-probabilistic basis for ranking protein hits.

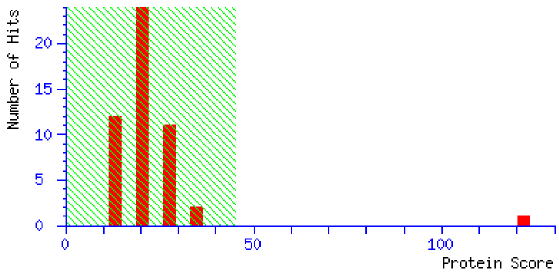

Matched peptide sequences: shown in **Bold Red:**

1 MDQSKEIARD VFPFLR**VYKD** **GTIER**LAGTE VSHAGLDPET GVLSKDTVIV
51 PETGVSARLY RPNSAKGNRK LPLVIYYHGG GFFISSAADP KYHNSLNRLV
101 AEANIVLVSV DYRIAPENPL PAAYDDSWAA LQWVAAHAKE DGGSEAWLKD
151 YVDFGRVFLA GDSCGANVAH HFALKLKDCE LGHQINIQAI AMIFPYFWGK
201 DPIGVEVTDQ ARKSMVDNWW LLVCPSEKGC DDPLINPFAD GSPSLESLAC
251 KR**LLVIVAEK** **DILR**DRGRLY YEKMVNSEWQ GTAEFMEVQG EDHVFHIHNP
301 DCENAKSMFK GLASFINQA

Spot No.: D6

NCBI accession No.: **gi|14423688** Species: *Hevea brasiliensis*

PFF score: [229] Protein name: Enolase 1

Matched peptides No.: [3] Sequence coverage %: [10]

Matched sequences: R.AGWGVMASHR.S + Oxidation (M);

R.AAVPSGASTGIYEALELR.D;

R.IEEELGSEAVYAGANFR.K

Calculated Mr: **48029** Calculated *p*I: **5.57**

Probability Based Mowse Score:

Ions score is -10*Log(P), where P is the probability that the observed match is a random event.
Individual ions scores > 45 indicate identity or extensive homology (p<0.05).
Protein scores are derived from ions scores as a non-probabilistic basis for ranking protein hits.

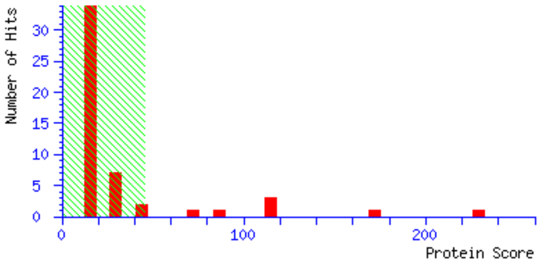

Matched peptide sequences: shown in Bold Red

1	MAITIVSVRA	RQIFDSRGNP	TVEADVKLSD	GYLAR**AAVPS**	**GASTGIYEAL**
51	**ELR**DGGSDYL	GKGVSKAVEN	VNIIIGPALV	GKDPTDQVGI	DNFMVQQLDG
101	TVNEWGWCKQ	KLGANAILAV	SLAVCKAGAH	VKGIPLYEHI	ANLAGNKNLV
151	LPVPAFNVIN	GGSHAGNKLA	MQEFMILPVG	ASSFKEAMKM	GAEVYHHLKS
201	VIKKKYGQDA	TNVGDEGGFA	PNIQENKEGL	ELLKTAIAKA	GYTGKVVIGM
251	DVAASEFYGS	DQTYDLNFKE	ENNNGSQKIS	GEALKDLYKS	FVAEYPIVSI
301	EDPFDQDDWA	HYAKLTSEIG	EKVQIVGDDL	LVTNPKRVEK	AIKEKACNAL
351	LLKVNQIGSV	TESIEAVKMS	KR**AGWGVMAS**	**HR**SGETEDTF	IADLSVGLAT
401	GQIKTGAPCR	SERLAKYNQL	LR**IEEELGSE**	**AVYAGANFR**K	PVEPY

Spot No.: **D7**

NCBI accession No.: **gi| 37719658** Species: *Hevea brasiliensis*

Protein name： **50 kDa protein**

Mascot score: **95** Sequence coverage %: **42**

Matched peptides No.: **14** Total peptides No.: **66**

Calculated Mr: **41480** Calculated *p*I: **4.60**

Probability Based Mowse Score:

Protein score is -10*Log(P), where P is the probability that the observed match is a random event.
Protein scores greater than 73 are significant ($p<0.05$).

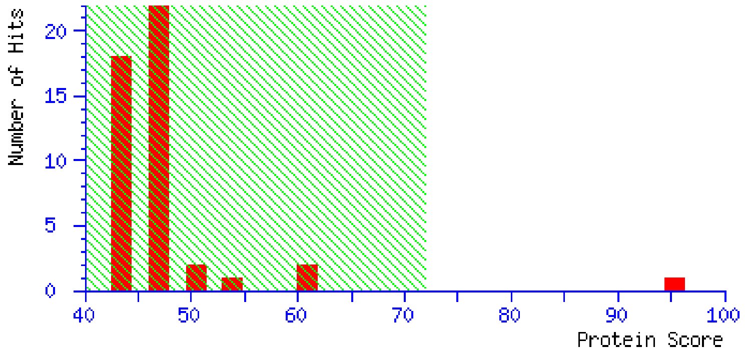

Matched peptide sequences: shown in Bold Red:

```
  1 MASLAYSLFI LSLFTFTLLN PVCSELDEYL FSFGDGLYDA GNAKFIYPDK
 51 YLPSYHHPYG TTFFDYPTGR FSDGRTVVDF VAENVSLPRI PPFKNKEANF
101 TYGANFASEG ATASDSNPLI DFRSQIRDFG ELKLEWAVQL VNVTELARRL
151 KKAVYLISFG ADDYLNYEIP SEASREQLES IVDVVLGNIS DRIKELYDFG
201 ARKFVVENVA PLGLIPFIKQ TSDNSTLFYE LASLHAMKLP QILEKIQDGY
251 LFPEFNYTVF NYFGIIKEII DAPGEHGFKY GDIACCGNST YRGQACGFLD
301 YEFCVCGNKT EYLFFDGTHN TDAANNLLAE LMWDKESGFI SPYGVKDFFP
351 SPTTIQTLLT EATALG
```

Spot No.: **D8**

NCBI accession No.: **gi|255565836** Species: *Ricinus communis*

Protein name： eukaryotic translation elongation factor, putative

Mascot score: **123** Sequence coverage %: **30**

Matched peptides No.: **26** Total peptides No.: **56**

Calculated Mr: **94981** Calculated *p*I: **5.87**

Probability Based Mowse Score:

Protein score is -10*Log(P), where P is the probability that the observed match is a random event.
Protein scores greater than 72 are significant (p<0.05).

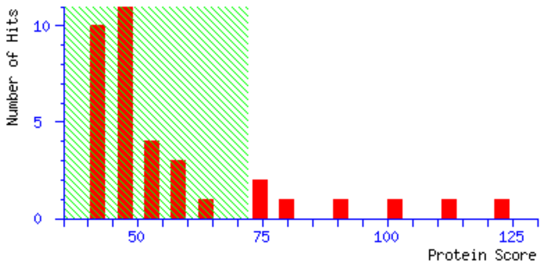

Matched peptide sequences: shown in **Bold Red**

1	MVK**FTAEELR**	RIMDYKHNIR	**NMSVIAHVDH**	**GKSTLTDSLV**	**AAAGIIAQEV**
51	**AGDVR**MTDTR	QDEAERGITI	KSTGISLYYE	MTDEALKSFK	GERQGNEYLI
101	NLIDSPGHVD	FSSEVTAALR	ITDGALVVVD	CIEGVCVQTE	TVLR**QALGER**
151	IRPVLTVNKM	DRCFLELQVD	GEEAYQTFSR	VIENANVIMA	TYEDPLLGDC
201	QVYPEKGTVA	FSAGLHGWAF	TLTNFAK**MYA**	**SK**FGVDESKM	MER**LWGENFF**
251	**DPATK**KWTSK	**NTGSPTCKRG**	**FVQFCYEPIK**	QIINTCMNDQ	KDKLWPMLQK
301	**LGVTMKSDEK**	ELMGKALMKR	VMQTWLPASS	ALLEMMIFHL	PSPAKAQR**YR**
351	**VENLYEGPLD**	**DPYATAIRNC**	**DPEGPLMLYV**	**SK**MIPASDKG	R**FFAFGR**VFA
401	GKVSTGLKVR	**IMGPNYVPGE**	**K**KDLYVK**SVQ**	**RTVIWMGK**KQ	ETVEDVPCGN
451	TVALVGLDQF	ITKNATLTNE	KEVDAHPIRA	MK**FSVSPVVR**	VAVQCKVASD
501	LPKLVEGLKR	LAKSDPMVVC	TIEESGEHII	AGAGELHLEI	CLK**DLQDDFM**
551	**GGAEIIK**SDP	VVSFRETVLE	KSCRVVMSKS	PNKHNRLYME	ARPMEEGLAE
601	AIDEGRIGPR	DDPKARAK**IL**	**SEEFGWDKDL**	**AK**KIWCFGPE	TTGPNMVVDM
651	CKGVQYLNEI	K**DSVVAGFQW**	**ASKEGALAEE**	**NMR**GICFEVC	DVVLHADAIH
701	R**GGGQVIPTA**	**RRVIYASQLT**	**AKPR**LLEPVY	LVEIQAPEQA	LGGIYSVLNQ
751	KR**GHVFEELQ**	**RPGTPLYNIK**	AYLPVIESFG	FSGTLRAATS	GQAFPQCVFD
801	HWDMMSSDPM	EPGSQAANLV	TEIRKRKGLK	EQMTPLSEFE	DKL

Spot No.: **D9**

NCBI accession No.: **gi|225428086** Species: *Vitis vinifera*

Protein name: PREDICTED: V-type proton ATPase subunit B 1

Mascot score: **146**

Sequence coverage %: **52**

Matched peptides No.: 29

Total peptides No.: **52**

Calculated Mr: **54367**

Calculated *p*I: **5.04**

Probability Based Mowse Score:

Protein score is -10*Log(P), where P is the probability that the observed match is a random event.
Protein scores greater than 73 are significant (p<0.05).

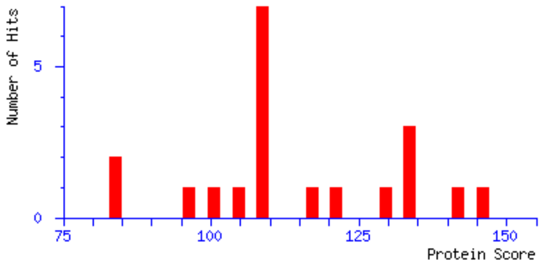

Matched peptide sequences: shown in Bold Red

1 MGVEQNNHDA EEGTLLVGME YRTVSGVAGP LVILEKVKGP K**YQEIVNIRL**
51 GDGTTRRGQV LEVDGEK**AVV QVFEGTSGID NK**YTTVQFTG EVLK**TPVSLD**
101 **MLGR**IFNGSG KPIDNGPPIL PEAYLDISGS SINPSER**TYP EEMIQTGIST**
151 **IDVMNSIAR**G QK**IPLFSAAG LPHNEIAAQI CR**QAGLVKRL EKSGSLLEDV
201 EEDNFAIVFA AMGVNMETAQ FFK**RDFEENG SMERVTLFLN LANDPTIER**I
251 ITPR**IALTTA EYLAYECGKH VLVILTDMSS YADALR**EVSA AR**EEVPGRRG**
301 **YPGYMYTDLA TIYER**AGRIE GRKGSITQIP ILTMPNDDIT HPTPDLTGYI
351 TEGQIYIDRQ LHNR**QIYPPI NVLPSLSRLM KSAIGEGMTR RDHADVSNQL**
401 **YANYAIGK**DV QAMK**AVVGEE ALSSEDLLYL EFLDKFERKF VAQGAYDTRN**
451 **IFQSLDLAWT LLR**IFPRELL HRIPAK**TLDQ YYSR**EASN

Spot No.: **D10**

NCBI accession No.: **gi| 2832430** Species: *Hevea brasiliensis*

PFF score: [446]

Protein name: Pro-hevein

Matched peptides No.: [4] Sequence coverage %: [32]

Matched sequences: K.YGWTAFCGPVGAHGQPSCGK.C; K.CLSVTNTGTGAK.A; R.IVDQCSNGGLDLDVNVFR.Q; R.QLDTDGKGYER.G

Calculated Mr: **20852** Calculated *p*I: **5.65**

Probability Based Mowse Score:

Ions score is -10*Log(P), where P is the probability that the observed match is a random event.
Individual ions scores > 45 indicate identity or extensive homology (p<0.05).
Protein scores are derived from ions scores as a non-probabilistic basis for ranking protein hits.

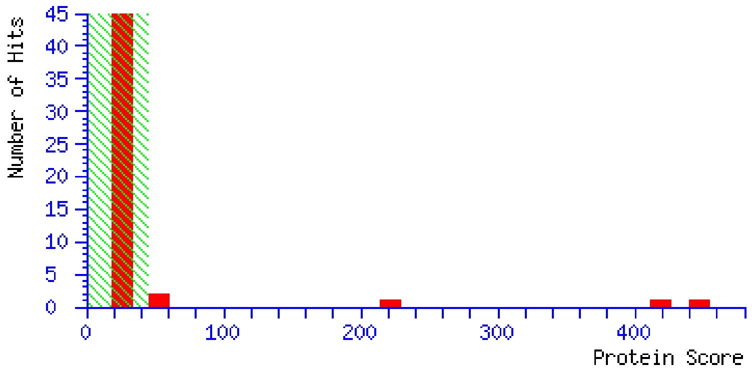

Matched peptide sequences: shown in Bold Red:

```
  1 EQCGRQAGGK LCPNNLCCSQ WGWCGSTDEY CSPDHNCQSN CKDSGEGVGG
 51 GSASNVLATY HLYNSQDHGW DLNAASAYCS TWDANKPYSW RSKYGWTAFC
101 GPVGAHGQPS CGKCLSVTNT GTGAKATVRI VDQCSNGGLD LDVNVFRQLD
151 TDGKGYERGH LTVNYQFVDC GDSFNPLFSV MKSSVIN
```

Spot No.: **D11**

NCBI accession No.: **gi|123062** Species: *Hevea brasiliensis*

PFF score: [158]

Protein name: Pro-hevein

Matched peptides No.: [2] Sequence coverage %: [11]

Matched sequences: K.CLSVTNTGTGAK.T;
R.QLDTDGKGYER.G

Calculated Mr: **22700** Calculated *p*I: **5.63**

Probability Based Mowse Score:

Ions score is -10*Log(P), where P is the probability that the observed match is a random event.
Individual ions scores > 45 indicate identity or extensive homology (p<0.05).
Protein scores are derived from ions scores as a non-probabilistic basis for ranking protein hits.

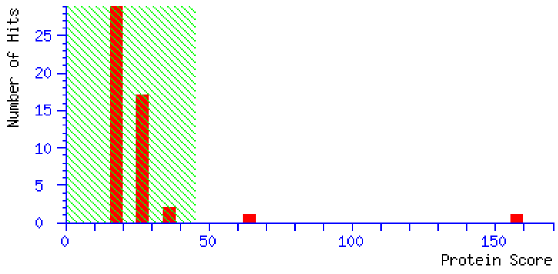

Matched peptide sequences: shown in Bold Red:

```
  1 MNIFIVVLLC LTGVAIAEQC GRQAGGKLCP NNLCCSQWGW CGSTDEYCSP
 51 DHNCQSNCKD SGEGVGGGSA SNVLATYHLY NSQDHGWDLN AASAYCSTWD
101 ANKPYSWRSK YGWTAFCGPV GAHGQSSCGK CLSVTNTGTG AKTTVRIVDQ
151 CSNGGLDLDV NVFRQLDTDG KGYERGHITV NYQFVDCGDS FNPLFSVMKS
201 SVIN
```

Spot No.: **D12**

NCBI accession No.: **gi|15237159** Species: *Arabidopsis thaliana*

PFF score: [77]

Protein name: regulatory particle triple-A ATPase 3

Matched peptides No.: [4] Sequence coverage %: [13]

Matched sequences: K.AVANHTTAAFIR.V;
K.KPDTDFEFYK.-;
R.EAVELPLTHHELYK.Q;
K.ENAPAIIFIDEVDAIATAR.F

Calculated Mr: **45894** Calculated *p*I: **5.42**

Probability Based Mowse Score:

Ions score is -10*Log(P), where P is the probability that the observed match is a random event.
Individual ions scores > 46 indicate identity or extensive homology (p<0.05).
Protein scores are derived from ions scores as a non-probabilistic basis for ranking protein hits.

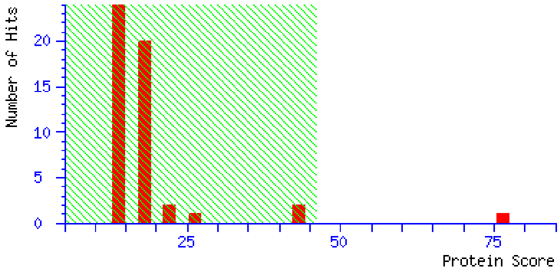

Matched peptide sequences: shown in Bold Red:

```
  1 MASAAVASMV LDPKASPALM DLSTADEEDL YGRLKSLERQ LEFTDIQEEY
 51 VKDEQKNLKR ELLRAQEEVK RIQSVPLVIG QFMEMVDQNN GIVGSTTGSN
101 YYVRILSTIN RELLKPSASV ALHRHSNALV DVLPPEADSS ISLLSQSEKP
151 DVSYNDIGGC DIQKQEIREA VELPLTHHEL YKQIGIDPPR GVLLYGPPGT
201 GKTMLAKAVA NHTTAAFIRV VGSEFVQKYL GEGPRMVRDV FRLAKENAPA
251 IIFIDEVDAI ATARFDAQTG ADREVQRILM ELLNQMDGFD QTVNVKVIMA
301 TNRADTLDPA LLRPGRLDRK IEFPLPDRRQ KRLVFQVCTS KMNLSDEVDL
351 EDYVSRPDKI SAAEIAAICQ EAGMHAVRKN RYVILPKDFE KGYRANVKKP
401 DTDFEFYK
```

Spot No.: **D13**

NCBI accession No.: **gi|123062** Species: *Hevea brasiliensis*

PFF score: [129] Protein name: Pro-hevein

Matched peptides No.: [1] Sequence coverage %: [8]

Matched sequences: R.IVDQCSNGGLDLDVNVFR.Q

Calculated Mr: **22700** Calculated *p*I: **5.63**

Probability Based Mowse Score:

Ions score is -10*Log(P), where P is the probability that the observed match is a random event.
Individual ions scores > 45 indicate identity or extensive homology (p<0.05).
Protein scores are derived from ions scores as a non-probabilistic basis for ranking protein hits.

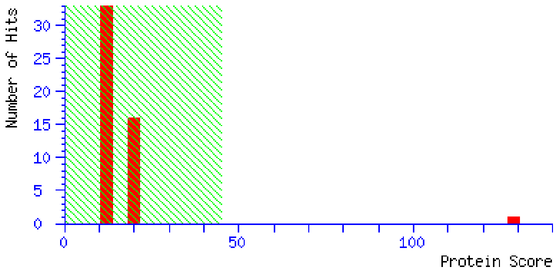

Matched peptide sequences: shown in Bold Red:

1 MNIFIVVLLC LTGVAIAEQC GRQAGGKLCP NNLCCSQWGW CGSTDEYCSP
51 DHNCQSNCKD SGEGVGGGSA SNVLATYHLY NSQDHGWDLN AASAYCSTWD
101 ANKPYSWRSK YGWTAFCGPV GAHGQSSCGK CLSVTNTGTG AKTTVR**IVDQ**
151 **CSNGGLDLDV** **NVFR**QLDTDG KGYERGHITV NYQFVDCGDS FNPLFSVMKS
201 SVIN

Spot No.: **D14**

NCBI accession No.: **gi|225446010** Species: *Vitis vinifera*

PFF score: [196]

Protein name: putative glucose-6-phosphate 1-epimerase

Matched peptides No.: [4] Sequence coverage %: [14]

Matched sequences: R.VALGPGGDLMLTSR.I;
R.VALGPGGDLMLTSR.I + Oxidation (M);
K.DGLPDAVVWNPWDKK.A;
R.VEGLETLDYLDNLQNK.E

Calculated Mr: **35638** Calculated *p*I: **5.75**

Probability Based Mowse Score:

Ions score is -10*Log(P), where P is the probability that the observed match is a random event.
Individual ions scores > 46 indicate identity or extensive homology (p<0.05).
Protein scores are derived from ions scores as a non-probabilistic basis for ranking protein hits.

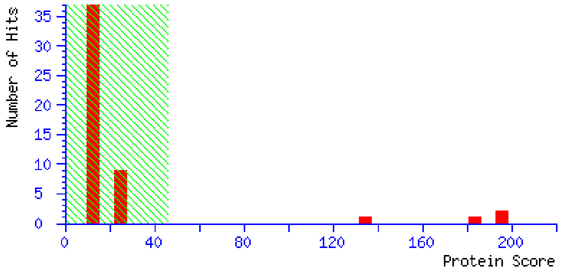

Matched peptide sequences: shown in Bold Red:

```
  1 MSSERVPVEL CKGINGLEKV VLREVRGSSA EVYLYGGQVT SWKNDHGEEL
 51 LFVSSKAIFK PPKAIRGGIP ICFPQFGNHG SLEQHGFARN RVWSIDVDPP
101 PFPTNTSSRA FIDLILKPSE EDMKIWPHSY EFRLRVALGP GGDLMLTSRI
151 RNTSTEGKPL TFTFAYHTYF SVSDISEVRV EGLETLDYLD NLQNKERFTE
201 QGDAITFESE VDKIYVSTPT KIAILDHEKK RTFVIRKDGL PDAVVWNPWD
251 KKAKAMADFG DDEYKHMLCV EAAAVEKPIT LKPGEEWRGR QELSAVPSSY
301 CSGQLDPQKV LQCS
```

Spot No.: **D15**

NCBI accession No.: **gi|1143427** Species: *Cucumis sativus*

PFF score: [338] Protein name: heat shock protein 70

Matched peptides No.: [5] Sequence coverage %: [8]

Matched sequences: K.DIDEVILVGGSTR.I;
K.QFAAEEISAQVLR.K;
K.AVVTVPAYFNDSQR.T;
K.LSFKDIDEVILVGGSTR.I;
K.KQDITITGASTLPSDEVER.M

Calculated Mr: **75480** Calculated *p*I: **5.15**

Probability Based Mowse Score:

Ions score is -10*Log(P), where P is the probability that the observed match is a random event.
Individual ions scores > 45 indicate identity or extensive homology (p<0.05).
Protein scores are derived from ions scores as a non-probabilistic basis for ranking protein hits.

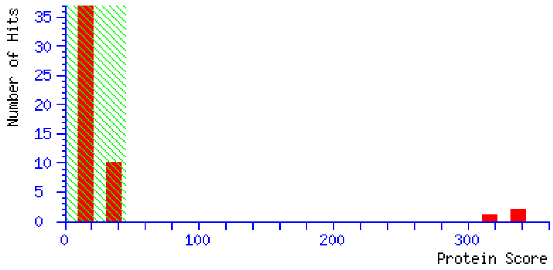

Matched peptide sequences: shown in Bold Red:

1	MGASTAQIHG	LGAPSFAAAS	MRKSNNVSSR	SVFFGQKLGN	SSAFPAAAFL
51	NLRSNTSRRN	SSVRPLRIVN	EKVVGIDLGT	TNSAVAAMEG	GKPTIVTNAE
101	GQRTTPSVVA	YTKNGDRLVG	QIAKRQAVVN	PENTFFSVKR	FIGRKMSEVD
151	EESKQVSYRV	ERDENGNVKL	ECPAIGK**QFA**	**AEEISAQVLR**	KLVDDASKFL
201	NDKVTK**AVVT**	**VPAYFNDSQR**	TATKDAGRIA	GLEVLRIINE	PTAASLAYGF
251	EKKSNETILV	FDLGGGTFDV	SVLEVGDGVF	EVLSTSGDTH	LGGDDFDKRI
301	VDWLAANFKR	DEGIDLLKDK	QALQRLTETA	EKAKMELSSL	TQANISLPFI
351	TATADGPKHI	ETTITRAKFE	ELCSDLLDRL	KTPVENSLRD	AK**LSFKDIDE**
401	**VILVGGSTRI**	PAVQELVKKM	TGKEPNVTVN	PDEVVALGAA	VQAGVLAGDV
451	SDIVLLDVSP	LSLGLETLGG	VMTKIIPRNT	TLPTSKSEVF	STAADGQTSV
501	EINVLQGERE	FVRDNKSLGS	FRLDGIPPAP	RGVPQIEVKF	DIDANGILSV
551	TAIDKGSGK**K**	**QDITITGAST**	**LPSDEVER**MV	SEADKFAKED	KEKRDAIDTK
601	NQADSVVYQT	EKQLKELGDK	VPGPVKEKVE	SKLGELKEAI	SGGSTEAIKE
651	AMAALNQEVM	QLGQSLYNQP	GAGAAPGPGA	SSESGPSEST	GKGPEGDVID
701	ADFSDSK				

Spot No.: **D16**

NCBI accession No.: **gi|255558037** Species: *Ricinus communis*

PFF score: [198]

Protein name: nucleic acid binding protein, putative

Matched peptides No.: [2] Sequence coverage %: [9]

Matched sequences: K.HQFTSTATSK.V;

R.YVLTGASWVTGAFNR.V

Calculated Mr: **67285** Calculated *p*I: **5.36**

Probability Based Mowse Score:

Ions score is -10*Log(P), where P is the probability that the observed match is a random event.
Individual ions scores > 46 indicate identity or extensive homology (p<0.05).
Protein scores are derived from ions scores as a non-probabilistic basis for ranking protein hits.

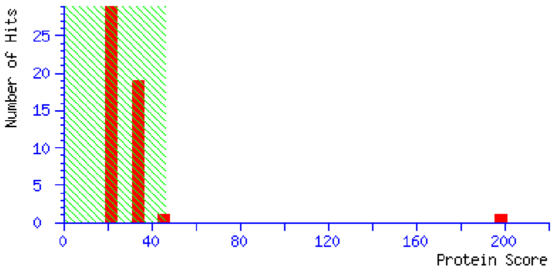

Matched peptide sequences: shown in Bold Red:

```
  1 MAITTVKVSN VSLGATEQDI KEFFSFSGDI DYVELLGDNE RSQIAYVTFK
 51 DQQGAETAVL LSGATIVDQS VTIELAPDYK LPAAASVPTT ATESSTASGA
101 ESAFQKAEDI VSSMLAKGFI LGKDAVNKAK AFDEKHQFTS TATSKVASLD
151 EKIGLTEKIS AGTTLVNDKV REVDEKFRVS ETTKSAFAAA EQTVSNAGSA
201 IMKNRYVLTG ASWVTGAFNR VAKAAGEVGQ KTKEKVLAEE EQNQVAEGYT
251 QIHGTDSPNM SEQMSKSSH
```

Spot No.: **D17**

NCBI accession No.: **gi|27449246** Species: *Hevea brasiliensis*

PFF score: [105] Protein name: heat shock protein 70

Matched peptides No.: [3] Sequence coverage %: [23]

Matched sequences: K.DHGGPEDENR.H;

K.HIPLSGPHSIAGR.S;

R.SVVFHEGRDDLGK.G

Calculated Mr: **15726** Calculated *p*I: **5.73**

Probability Based Mowse Score:

Ions score is -10*Log(P), where P is the probability that the observed match is a random event.
Individual ions scores > 46 indicate identity or extensive homology (p<0.05).
Protein scores are derived from ions scores as a non-probabilistic basis for ranking protein hits.

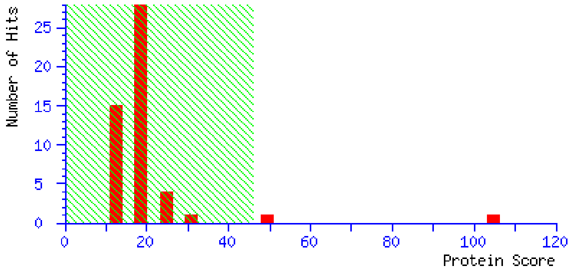

Matched peptide sequences: shown in Bold Red:

```
  1 MLKAVAVITS SEGISGKIFF TQEGDGPTTV TGSVSGLKPG LHGFHVHTFG
 51 DTTNGCLSTG LHFNPASKDH GGPEDENRHA GDLGNVNVGD DGTANFTIVD
101 KHIPLSGPHS IAGRSVVFHE GRDDLGKGGH ELSKITGNAG DRIACGIIGL
151 QE
```

Spot No.: **D18**

NCBI accession No.: **gi|2832430** Species: *Hevea brasiliensis*

PFF score: [446] Protein name: prohevein

Matched peptides No.: [4] Sequence coverage %: [32]

Matched sequences: K.CLSVTNTGTGAK.A;
R.QLDTDGKGYER.G;
R.IVDQCSNGGLDLDVNVFR.Q;
K.YGWTAFCGPVGAHGQPSCGK.C

Calculated Mr: **20852** Calculated *p*I: **5.64**

Probability Based Mowse Score:

Ions score is -10*Log(P), where P is the probability that the observed match is a random event.
Individual ions scores > 46 indicate identity or extensive homology (p<0.05).
Protein scores are derived from ions scores as a non-probabilistic basis for ranking protein hits.

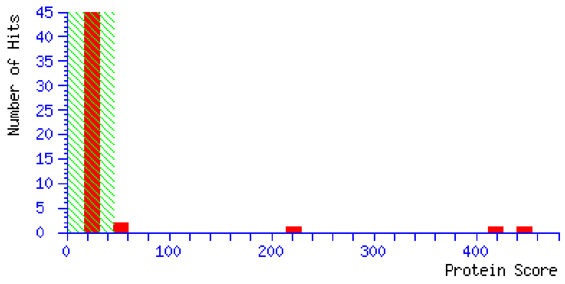

Matched peptide sequences: shown in Bold Red:

```
  1 EQCGRQAGGK LCPNNLCCSQ WGWCGSTDEY CSPDHNCQSN CKDSGEGVGG
 51 GSASNVLATY HLYNSQDHGW DLNAASAYCS TWDANKPYSW RSKYGWTAFC
101 GPVGAHGQPS CGKCLSVTNT GTGAKATVRI VDQCSNGGLD LDVNVFRQLD
151 TDGKGYERGH LTVNYQFVDC GDSFNPLFSV MKSSVIN
```

Spot No.: **D19**

NCBI accession No.: **gi|255551408**   Species: *Ricinus communis*

PFF score: [301]

Protein name: chaperonin containing t-complex protein 1

Matched peptides No.: [6]   Sequence coverage %: [14]

Matched sequences: R.HKFDVDTR.L;
K.GPNDHTIAQIK.D;
K.GIDPPSLDLLAR.A;
R.LVEGLVLDHGSR.H;
K.SEINAGFFYSNAEQR.E;
K.NTIEDEAVVLGAGAFELAAR.K

Calculated Mr: **59556**   Calculated *p*I: **6.08**

Probability Based Mowse Score:

Ions score is -10*Log(P), where P is the probability that the observed match is a random event.
Individual ions scores > 46 indicate identity or extensive homology (p<0.05).
Protein scores are derived from ions scores as a non-probabilistic basis for ranking protein hits.

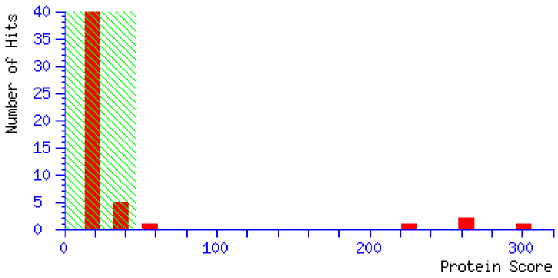

Matched peptide sequences: shown in Bold Red:

1 MSLRVLNPNA EVLNKSAALH MNINAAKGLQ DVLKSNLGPK GTIKMLVGGA
51 GDIKLTKDGN TLLKEMQIQN PTAIMIARTA VAQDDISGDG TTSTVIFIGE
101 LMKQSERYIG EGMHPRVLVD GFEIAKRATL QFLEKFKTPV VMGDEPDKEI
151 LKMVARTTLR TKLYETLADQ LTDIVVNAVL CIRKPEEAID LFMVEIMHMR
201 **HKFDVDTRLV** **EGLVLDHGSR** HPDMKRRAEN CYILTCNVSL EYDK**SEINAG**
251 **FFYSNAEQRE** KMVAAERRQV DERVEKIIEL KNKVCSGNDN NFVVINQK**GI**
301 **DPPSLDLLAR** AGIIALRRAK RRNMERLVLA CGGEAVNSVD DLTPDCLGWA
351 GLVYEHILGE EKYTFVEHVK NPHSCTILIK **GPNDHTIAQI** **K**DAVRDGLRA
401 VK**NTIEDEAV** **VLGAGAFELA** **AR**KYLISEVK KTVKGRAQLG IEAFADALLV
451 IPKTLAENSG LDTQDEIVSL TGEHDRENIV GLNLQTGGPL DPQMEGIFDN
501 YSVKRQLINS GPVIASQLLL VDEVIRAGRN MRKPN

Spot No.: **D20**

NCBI accession No.: **gi|225438145** Species: *Vitis vinifera*

PFF score: [75]

Protein name: PREDICTED: malate dehydrogenase, cytoplasmic

Matched peptides No.: [1] Sequence coverage %: [3]

Matched sequences: K.IVQGLHIDEFSR.K

Calculated Mr: **35881** Calculated *p*I: **6.18**

Probability Based Mowse Score:

Ions score is -10*Log(P), where P is the probability that the observed match is a random event.
Individual ions scores > 46 indicate identity or extensive homology (p<0.05).
Protein scores are derived from ions scores as a non-probabilistic basis for ranking protein hits.

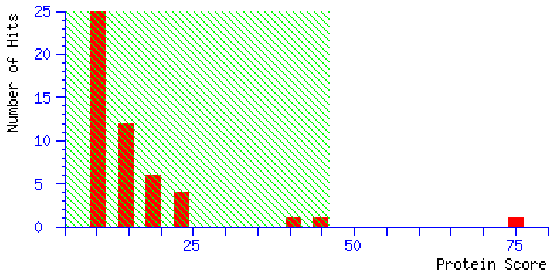

Matched peptide sequences: shown in Bold Red:

```
  1 MAKEPVRVLV TGAAGQIGYA LVPMIARGVM LGADQPVILH MLDIPPAAEA
 51 LNGVKMELVD AAFPLLKGVV ATTDVVEACT GVNIAVMVGG FPRKEGMERK
101 DVMSKNVSIY KSQASALENH AAANCKVLVV ANPANTNALI LKEFAPSIPE
151 KNISCLTRLD HNRALGQVSE RLNVQVSDVK NVIIWGNHSS TQYPDVNHAT
201 VKTPAGEKPV RGLVGDDAWL NGEFITTVQQ RGAAIIKARK LSSALSAASA
251 ACDHIRDWVL GTPEGTWVSM GVYSDGSYNV PAGLIYSFPV TCCAGEWKIV
301 QGLHIDEFSR KKLDLTAQEL SEEKELAYSC LS
```

Spot No.: **D21**

NCBI accession No.: **gi|224056377** Species: *Ricinus communis*

PFF score: [116]

Protein name: Aspartic proteinase precursor

Matched peptides No.: [3] Sequence coverage %: [7]

Matched sequences: K.GEHTYVPVTQK.G;
K.EPVFSFWFNR.N;
K.NYMDAQYFGEIGIGTPPQK.F + Oxidation (M)

Calculated Mr: **56596** Calculated *p*I: **5.19**

Probability Based Mowse Score:

Ions score is -10*Log(P), where P is the probability that the observed match is a random event.
Individual ions scores > 46 indicate identity or extensive homology (p<0.05).
Protein scores are derived from ions scores as a non-probabilistic basis for ranking protein hits.

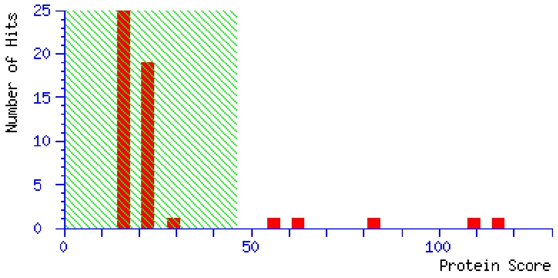

Matched peptide sequences: shown in Bold Red:

```
  1 MGTIFKPALF FCLILLPLVC ATASSSNDGL VRIGLKKRKF DQNNRVAAQF
 51 ESKEGEAFRA SIKKYHIRGN LGDAEDIDIV SLKNYMDAQY FGEIGIGTPP
101 QKFTVIFDTG SSNLWVPSSK CYFSVACYFH SKYKSGQSST YKKNGKSADI
151 HYGTGAISGF FSQDNVKVGE LVIKNQEFIE ATREPSITFL VAKFDGILGL
201 GFQEISVGNA VPVWYNMVNQ GLVKEPVFSF WFNRNADEDE GGEIVFGGMD
251 PNHYKGEHTY VPVTQKGYWQ FDMGDVLIDG KTTGICSSGC AAIADSGTSL
301 LAGPTTIITE VNHAIGATGV VSQECKAVVA QYGETIIAML LAKDQPQKIC
351 SQIGLCTFDG SRGVSMGIES VVNEKIQEVA GGLHDAMCST CEMAVVWMQN
401 QLKQNQTQEH ILNYVNELCE RLPSPMGESA VDCGSLSTMP NVSFTIGGRV
451 FDLAPEQYVL KVGDGEAAQC ISGFTALDVP PPRGPLWILG DVFMGPFHTV
501 FDYGNKRVGF AEVA
```

Spot No.: **D22**

NCBI accession No.: **gi|255554865** Species: *Ricinus communis*

PFF score: [145]

Protein name: lactoylglutathione lyase, putative

Matched peptides No.: [4] Sequence coverage %: [16]

Matched sequences: K.FYTECFGMK.L;

K.ITSFLDPDGWK.T;

K.DPDGYIFELIQR.G;

R.GPTPEPLCQVMLR.V + Oxidation (M)

Calculated Mr: **31641** Calculated *p*I: **7.63**

Probability Based Mowse Score:

Ions score is -10*Log(P), where P is the probability that the observed match is a random event.
Individual ions scores > 46 indicate identity or extensive homology (p<0.05).
Protein scores are derived from ions scores as a non-probabilistic basis for ranking protein hits.

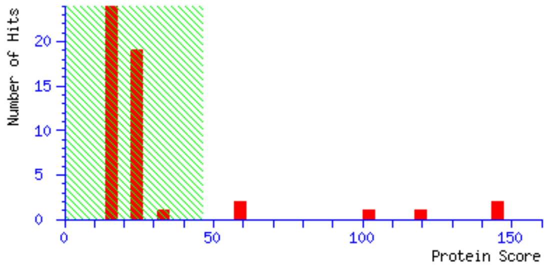

Matched peptide sequences: shown in Bold Red:

```
  1 MAAEATAPNA DVLEWPKKDK RRLLHAVYRV GDLDRTIKFY TECFGMKLLR
 51 KRDIPEEKYS NAFLGFGPEE TNFVVELTYN YGVTSYDIGT GFGHFAIATQ
101 DVYKLVEEVL AKGGAVTREP GPVKGGTTVI AFVKDPDGYI FELIQRGPTP
151 EPLCQVMLRV GDLDRSIRFY EKALGMKLLR KVDKPEYKYT LAMMGYADEY
201 ETTVLELTYN YGVTEYTKGN AYAQVAISTD DVYKSAEVVN LVTQELGGKI
251 TRQPGPIPGL NTKITSFLDP DGWKTKRKRR
```

Spot No.: **D23**

NCBI accession No.: **gi|356517215** Species: *Glycine max*

PFF score: [466]

Protein name: uncharacterized protein LOC100817735

Matched peptides No.: [5] Sequence coverage %: [22]

Matched sequences: R.LGLSAVIMGSR.G;
K.IHIVKDHDMK.E + Oxidation (M);
K.IGVAVDLSDESAYAVR.W;
R.KIGVAVDLSDESAYAVR.W;
K.LGSVSDYCVHHCVCPVVVVR.Y

Calculated Mr: **27677** Calculated *p*I: **5.83**

Probability Based Mowse Score:

Ions score is -10*Log(P), where P is the probability that the observed match is a random event.
Individual ions scores > 46 indicate identity or extensive homology (p<0.05).
Protein scores are derived from ions scores as a non-probabilistic basis for ranking protein hits.

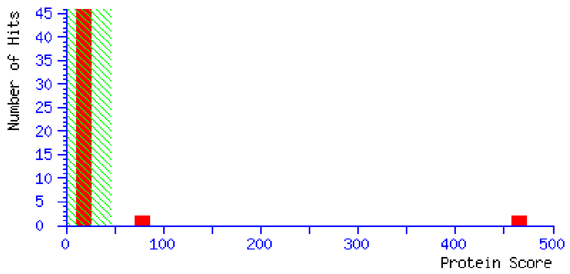

Matched peptide sequences: shown in Bold Red:

```
  1 MQPHQNPPAL DSDPQLPQIK IHHPASPRHH HPSSAGAATP TPTAGARRKI
 51 GVAVDLSDES AYAVRWAVQH YIRPGDAVIL LHVSATNVLF GADWGSIDLS
101 INTDPNSDED AVSAVNNSND HNSKRKLEDD FDAFTASKAA DLAKPLRELQ
151 IPFKIHIVKD HDMKERLCLE VERLGLSAVI MGSRGFGAVR RGSDGKLGSV
201 SDYCVHHCVC PVVVVRYPDD KDAVAAATTA EPVVAVKDGD EGEAVVQPVP
251 HKKED
```

Spot No.: **D24**

NCBI accession No.: **gi|356569000** Species: *Glycine max*

PFF score: [505]

Protein name: heat shock cognate 70 kDa protein-like

Matched peptides No.: [11] Sequence coverage %: [19]

Matched sequences:

K.ITITNDKGR.L;K.DAGVIAGLNVMR.I;K.DAGVIAGLNVMR.I + Oxidation (M);R.MVNHFVQEFK.R + Oxidation (M);K.NALENYAYNMR.N;K.NALENYAYNMR.N (M);K.SSVHDVVLVGGSTR.I;R.TTPSYVAFTDTER.L;K.ATAGDTHLGGEDFDNR.M; R.IINEPTAAAIAYGLDKK.A;K.EQVFSTYSDNQPGVLIQVYEGER.T

Calculated Mr: **71194** Calculated *p*I: **5.10**

Probability Based Mowse Score:

Ions score is -10*Log(P), where P is the probability that the observed match is a random event.
Individual ions scores > 46 indicate identity or extensive homology ($p<0.05$).
Protein scores are derived from ions scores as a non-probabilistic basis for ranking protein hits.

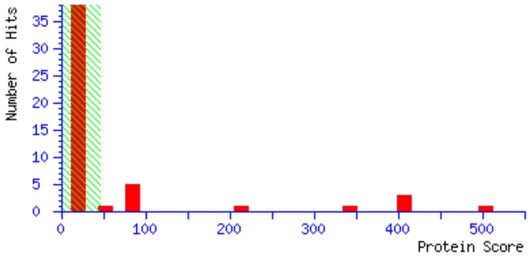

Matched peptide sequences: shown in Bold Red:

1 MAGKGEGLAI GIDLGTTYSC VGVWQHDRVE IIANDQGNR**T** **TPSYVAFTDT**
51 **ER**LIGDAAKN QVAMNPINTV FDAKRLIGRR VSDPSVQSDM KLWPFKVTAG
101 AGEKPMIGVN YKGEEKQFAA EEISSMVLTK MREIAEAYLG STVKNAVVTV
151 PAYFNDSQRQ ATK**DAGVIAG** **LNVMRIINEP** **TAAAIAYGLD** **KK**ATSVGEKN
201 VLIFDLGGGT FDVSLLTIEE GIFEVK**ATAG** **DTHLGGEDFD** **NRMVNHFVQE**
251 **FK**RKNKKDIT GNPRALRRLR TSCERAKRTL SSTAQTTIEI DSLFEGIDFY
301 STITRARFEE LNMDLFRKCM EPVEKCLRDA KMDK**SSVHDV** **VLVGGSTR**IP
351 KVQQLLQDFF NGKDLCKSIN PDEAVAYGAA VQAAILSGEG NEKVQDLLLL
401 DVTPLSLGLE TAGGVMTVLI PRNTTIPTKK **EQVFSTYSDN** **QPGVLIQVYE**
451 **GER**TRTRDNN LLGKFELSGI PPAPRGVPQI TVCFDIDANG ILNVSAEDKT
501 TGQKNK**ITIT** **NDKGR**LSKEE IEKMVQEAEK YKSEDEEHKK KVEGK**NALEN**
551 **YAYNMR**NTIK DEKISSKLSS EDKTKIDNAI EQAIQWLDTN QLAEADEFED
601 KMKELEGICN PIIAKMYQGG AGTGGDVDDD APPAGGSGAG PKIEEVD

Spot No.: **D25**

NCBI accession No.: **gi|132270**   Species: *Hevea brasiliensis*

PFF score: [405]

Protein name: Rubber elongation factor protein

Matched peptides No.: [4]   Sequence coverage %: [38]

Matched sequences: K.NVAVPLYNR.F;
K.DASIQVVSAIR.A;
K.FVDSTVVASVTIIDR.S;
K.DKSGPLQPGVDIIEGPVK.N

Calculated Mr: **14713**   Calculated *p*I: **5.04**

Probability Based Mowse Score:

Ions score is -10*Log(P), where P is the probability that the observed match is a random event.
Individual ions scores > 46 indicate identity or extensive homology (p<0.05).
Protein scores are derived from ions scores as a non-probabilistic basis for ranking protein hits.

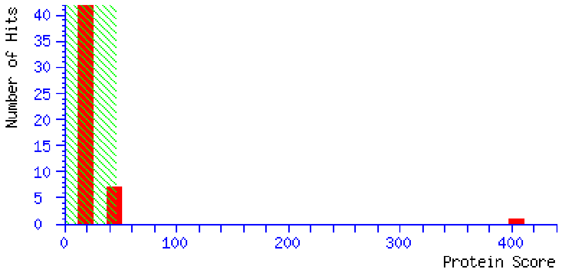

Matched peptide sequences: shown in Bold Red:

1 MAEDEDNQQG QGEGLKYLGF VQDAATYAVT TFSNVYLFAK **DKSGPLQPGV**
51 **DIIEGPVKNV** **AVPLYNR**FSY IPNGALK**FVD** **STVVASVTII** **DR**SLPPIVKD
101 **ASIQVVSAIR** AAPEAARSLA SSLPGQTKIL AKVFYGEN

Spot No.: **D26**

NCBI accession No.: **gi|6624721** Species: *Pisum sativum*

PFF score: [79]

Protein name: putative cysteine protease

Matched peptides No.: [2] Sequence coverage %: [7]

Matched sequences: R.SGQIDSNELR.D;

R.ALSSYNQSFSLR.T

Calculated Mr: **31321** Calculated *p*I: **7.81**

Probability Based Mowse Score:

Ions score is -10*Log(P), where P is the probability that the observed match is a random event.
Individual ions scores > 45 indicate identity or extensive homology (p<0.05).
Protein scores are derived from ions scores as a non-probabilistic basis for ranking protein hits.

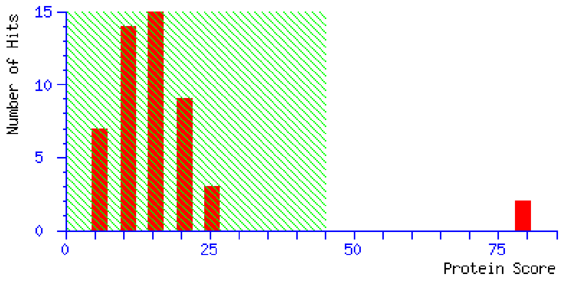

Matched peptide sequences: shown in Bold Red:

```
  1 RFFLLLGKTL SPFNMSGYPN QSPNYGYGYN APPPTQSYGA PPPSQSYGAP
 51 PPSQSYGAPP PSQYGAPPPG QSYSASPYGQ PSAPYAAPHQ KPPKEESHSS
101 GGGAYPPPAH GSPFASLLPS TFPPGTDPSI VACFQVADQD GSGLIDDKEL
151 QRALSSYNQS FSLRTVHLLM YHFTNTSVKI GPKEFTSLFY SLQSWRGIFE
201 RFDKDRSGQI DSNELRDALL SLGYAVSPTV LDLLVSKFDK TGGKHKAVEY
251 DNFIECCLTV KGLTDKFKEK DTGILALQHF PMRRLC
```

(Bold red: ALSSYNQS FSLR (153–164); SGQI DSNELR (207–216))

Spot No.: **D27**

NCBI accession No.: **gi|132270** Species: *Hevea brasiliensis*

PFF score: [340]

Protein name: Rubber elongation factor protein

Matched peptides No.: [4] Sequence coverage %: [38]

Matched sequences: K.NVAVPLYNR.F;

K.DASIQVVSAIR.A;
K.FVDSTVVASVTIIDR.S;
K.DKSGPLQPGVDIIEGPVK.N

Calculated Mr: **14713** Calculated *p*I: **5.04**

Probability Based Mowse Score:

Ions score is -10*Log(P), where P is the probability that the observed match is a random event.
Individual ions scores > 46 indicate identity or extensive homology (p<0.05).
Protein scores are derived from ions scores as a non-probabilistic basis for ranking protein hits.

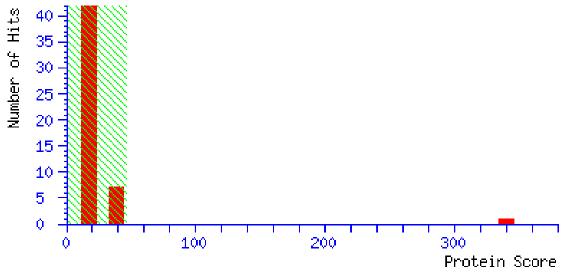

Matched peptide sequences: shown in Bold Red:

```
  1 MAEDEDNQQG QGEGLKYLGF VQDAATYAVT TFSNVYLFAK DKSGPLQPGV
 51 DIIEGPVKNV AVPLYNRFSY IPNGALKFVD STVVASVTII DRSLPPIVKD
101 ASIQVVSAIR AAPEAARSLA SSLPGQTKIL AKVFYGEN
```

Spot No.: **D28**

NCBI accession No.: **gi|314991144**

Species: *Gossypium hirsutum*

PFF score: [115]

Protein name: mitogen-activated protein kinase

Matched peptides No.: [3] Sequence coverage %: [9]

Matched sequences: K.YIHSANVLHR.D;
R.HMDHENVVAIR.D;
R.VTSESDFMTEYVVTR.W

Calculated Mr: **46237** Calculated *p*I: **5.47**

Probability Based Mowse Score:

Ions score is -10*Log(P), where P is the probability that the observed match is a random event.
Individual ions scores > 45 indicate identity or extensive homology (p<0.05).
Protein scores are derived from ions scores as a non-probabilistic basis for ranking protein hits.

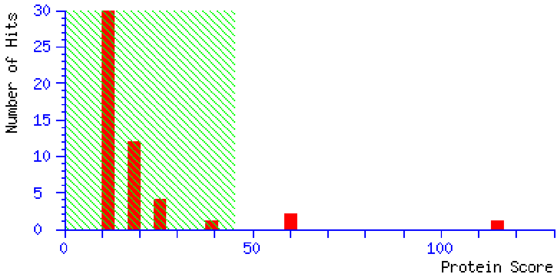

Matched peptide sequences: shown in Bold Red:

1	MEGGGPPQAA	DTEMAEAAQQ	QPQHHQQRPP	QVAAGLENIP	ATLSHGGRFI
51	QYNIFGNIFE	VTAKYKPPIM	PIGKGAYGIV	CSALNSETNE	HVALKKIANA
101	FDNKIDAKRT	LREIKLLR**HM**	**DHENVVAIR**D	IIPPPQRECF	NDVYIAYELM
151	DTDLHQIIRS	NQALSEEHCQ	YFPYQILRGL	**KYIHSANVLH**	**R**DLKPSNLLL
201	NANCDLKICD	FGLAR**VTSES**	**DFMTEYVVTR**	WYRPPELLLN	SSDYTAAIDV
251	WSVGCIFMEL	MDRKPLFPGR	DHVHQLRLLM	ELIGTPSEAE	LEFLNENAKR
301	YIRQLPLYRR	QSFTEKFPNV	PPLAIDLVEK	MLTFDPRQRI	TVEDALAHPY
351	LTSLHDISDE	PVCMTPFSFD	FEQHALTEEQ	MKELIYREAL	AFNPEYLQQ

Spot No.: **D29**

NCBI accession No.: **gi|118485535**

Species: *Populus trichocarpa*

PFF score: [82]   Protein name: unknown

Matched peptides No.: [1]   Sequence coverage %: [8]

Matched sequences: K.VATPAQAQEVHLELR.K

Calculated Mr: **18429**   Calculated *p*I: **5.59**

Probability Based Mowse Score:

Ions score is -10*Log(P), where P is the probability that the observed match is a random event.
Individual ions scores > 46 indicate identity or extensive homology (p<0.05).
Protein scores are derived from ions scores as a non-probabilistic basis for ranking protein hits.

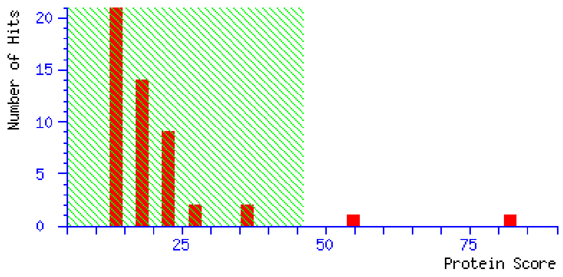

Matched peptide sequences: shown in **Bold Red:**

```
  1 MLVNLGIPWV ILGHSERRSL LNESNEFVGD KVAYALSLGL KVIACVGETL
 51 QQRESGSTMA VVAAQTKAIA ANVSYWDNVV MAYEPVWAIG TGKVATPAQA
101 QEVHLELRKW FHDNVGAEVA ASTRIIYGGS VNGANCKELA GQPDVDGFLV
151 GGASLKPEFI DIIKSATVKS QL
```

Bold red: VATPAQA (residues 94–100) and QEVHLELR (residues 101–108).

Spot No.: **D30**

NCBI accession No.: **gi|132270** Species: *Hevea brasiliensis*

PFF score: [470]

Protein name: Rubber elongation factor protein

Matched peptides No.: [4] Sequence coverage %: [27]

Matched sequences: K.NVAVPLYNR.F;
K.DASIQVVSAIR.A;
K.SGPLQPGVDIIEGPVK.N;
K.DKSGPLQPGVDIIEGPVK.N

Calculated Mr: **14713** Calculated *p*I: **5.04**

Probability Based Mowse Score:

Ions score is -10*Log(P), where P is the probability that the observed match is a random event.
Individual ions scores > 46 indicate identity or extensive homology (p<0.05).
Protein scores are derived from ions scores as a non-probabilistic basis for ranking protein hits.

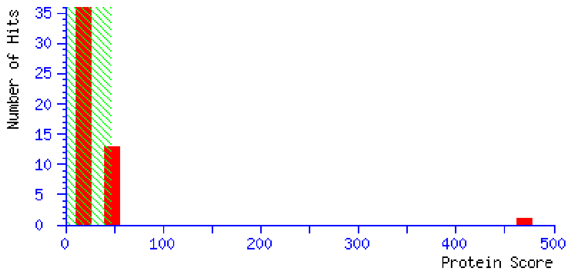

Matched peptide sequences: shown in Bold Red:

1 MAEDEDNQQG QGEGLKYLGF VQDAATYAVT TFSNVYLFAK **DKSGPLQPGV**
51 **DIIEGPVKNV AVPLYNR**FSY IPNGALKFVD STVVASVTII DRSLPPIVK**D**
101 **ASIQVVSAIR** AAPEAARSLA SSLPGQTKIL AKVFYGEN

Spot No.: **D31**

NCBI accession No.: **gi|470127750**

Species: *Fragaria vesca subsp. Vesca* PFF score: [80]

Protein name: uncharacterized protein LOC101300446

Matched peptides No.: [1] Sequence coverage %: [3]

Matched sequences: K.KLDLVITSPLLR.T

Calculated Mr: **36660** Calculated *p*I: **6.43**

Probability Based Mowse Score:

Ions score is -10*Log(P), where P is the probability that the observed match is a random event.
Individual ions scores > 46 indicate identity or extensive homology (p<0.05).
Protein scores are derived from ions scores as a non-probabilistic basis for ranking protein hits.

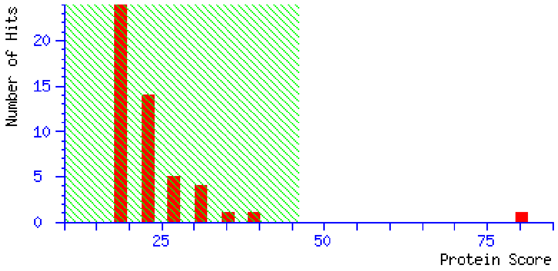

Matched peptide sequences: shown in Bold Red:

```
  1 MTVSISSIYT PHISLIPLSV PTSTSPSSKF PKSKHHSILL CFSSPPDMDA
 51 AGATSLYPLH RSKTIHLVRH AQGIHNVEGE KDHLAYMSHD LFDAHLTPLG
101 WKQVDNLHKH VQACGLTKKL DLVITSPLLR TMQTAVGVFG GEAYTDGIDV
151 PPLMVENAGN SLHAAISSLN CPPFVAVELC REHLGVHPCD KRRSISEYRP
201 LFPAIDFSLI ENDDDILWTP DIREKNEEVA ARGLKFLNWL WTREEKNIAI
251 VTHSGFLYHT LSAFGNDCHS SIKNEISTHF ANCELRSMVI VDRGLIGSNS
301 TSATNYPGKN PQGLDLPSDL ADKKNPVSK
```

Spot No.: **D32**

NCBI accession No.: **gi|132270** Species: *Hevea brasiliensis*

PFF score: [398]

Protein name: Rubber elongation factor protein

Matched peptides No.: [4] Sequence coverage %: [38]

Matched sequences: K.NVAVPLYNR.F;
K.DASIQVVSAIR.A;
K.FVDSTVVASVTIIDR.S;
K.DKSGPLQPGVDIIEGPVK.N

Calculated Mr: **14713** Calculated *p*I: **5.04**

Probability Based Mowse Score:

Ions score is -10*Log(P), where P is the probability that the observed match is a random event.
Individual ions scores > 46 indicate identity or extensive homology (p<0.05).
Protein scores are derived from ions scores as a non-probabilistic basis for ranking protein hits.

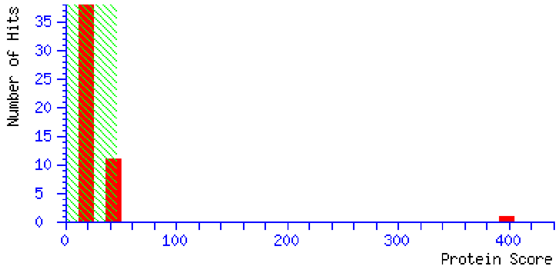

Matched peptide sequences: shown in Bold Red:

```
  1 MAEDEDNQQG QGEGLKYLGF VQDAATYAVT TFSNVYLFAK DKSGPLQPGV
 51 DIIEGPVKNV AVPLYNRFSY IPNGALKFVD STVVASVTII DRSLPPIVKD
101 ASIQVVSAIR AAPEAARSLA SSLPGQTKIL AKVFYGEN
```

Spot No.: **D33**

NCBI accession No.: **gi|351724891** Species: *Glycine max*

PFF score: [106] Protein name: enolase

Matched peptides No.: [2] Sequence coverage %: [7]

Matched sequences: R.AAVPSGASTGIYEALELR.D;

R.IEEELGAEAVYAGANFR.T

Calculated Mr: **47975** Calculated *p*I: **5.31**

Probability Based Mowse Score:

Ions score is -10*Log(P), where P is the probability that the observed match is a random event.
Individual ions scores > 45 indicate identity or extensive homology (p<0.05).
Protein scores are derived from ions scores as a non-probabilistic basis for ranking protein hits.

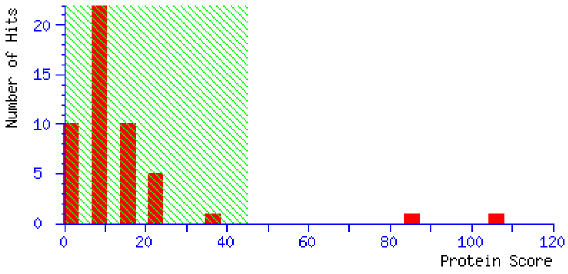

Matched peptide sequences: shown in Bold Red:

```
  1 MATIVSINAR QIFDSRGNPT VEVDLTCSDG TFARAAVPSG ASTGIYEALE
 51 LRDGGSDYLG KGVSKAVDNV NTVIGPALIG KDPTEQTAID NLMVQQLDGT
101 VNEWGWCKQK LGANAILAVF LAVCKAGASV LKIPLYKHIA NIAGNKKLVL
151 PVPAFNVING GSHAGNKLAM QEFMVLPVGA SSFKEAMKMG VEVYHNLKSV
201 IKKKYGQDAI NVGDEGGFAP NIQENKEGLE LLKTAIAKAG YTGKVVIGMD
251 VAASEFYKED KTYDLNFKED NNDGSQRISG DALKDLYKSF VSEYPIVSIE
301 DPFDQDDWEH YAKLTAEVGA NVQIVGDDLL VTNPKRVQKA IDTKACNALL
351 LKVNQIGSVT ESIEAVRMSK KAGWGVMASH RSGETEDTFI ADLSVGLATG
401 QIKTGAPCRS ERLAKYNQLL RIEEELGAEA VYAGANFRTP VEPY
```

Spot No.: **D34**

NCBI accession No.: **gi|255578278** Species: *Ricinus communis*

PFF score: [154]

Protein name: hypothetical protein RCOM_0537780

Matched peptides No.: [2] Sequence coverage %: [6]

Matched sequences: K.QESEYGSGGYGR.R;

R.KPSYGEEGGYGER.T

Calculated Mr: **41389** Calculated *p*I: **4.65**

Probability Based Mowse Score:

Ions score is -10*Log(P), where P is the probability that the observed match is a random event.
Individual ions scores > 46 indicate identity or extensive homology (p<0.05).
Protein scores are derived from ions scores as a non-probabilistic basis for ranking protein hits.

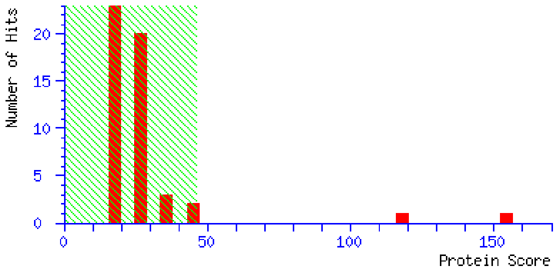

Matched peptide sequences: shown in Bold Red:

```
  1 MPYYTRSEDD VNDFDEFDPT PYGGGYDLAL TYGRPLPPSD ETCYQNSAIA
 51 DDDVDYDRPN FTSYAEPSAY NDDILQEEYN SYSRPKPRPG FIPGGGIGGD
101 PYSRPHAAPG FQPGSGGYGG VSEYEKPSGY GRRPDSEYGS GGYGGGTEYE
151 RPSGEDYGSG HGRKQDSEYG SGGYGRRPES EYGSGGGYER PSSEEYGSGY
201 GRKQESEYGS GGYGRRPEAE YGSGYGGRPE TEYGGGGGSE YGGGYGRKPS
251 YGEEGGYGER TEYERPSYGD DPPRRPGYGR QDDEYERPSY ERKSDDDNDG
301 SRRKYGGDDD EKGYQKKYGG YGDDDDEEGS RRKYGYGGEE ESYGRKKYGD
351 DGSDDDEKKH HRHKHHQHKH SDDE
```

Spot No.: **D35**

NCBI accession No.: **gi|1143427** Species: *Cucumis sativus*

PFF score: [378] Protein name: heat shock protein 70

Matched peptides No.: [5] Sequence coverage %: [8]

Matched sequences: K.DIDEVILVGGSTR.I;
K.QFAAEEISAQVLR.K;
K.AVVTVPAYFNDSQR.T;
K.LSFKDIDEVILVGGSTR.I;
K.KQDITITGASTLPSDEVER.M

Calculated Mr: **75480** Calculated *p*I: **5.15**

Probability Based Mowse Score:

Ions score is -10*Log(P), where P is the probability that the observed match is a random event.
Individual ions scores > 45 indicate identity or extensive homology (p<0.05).
Protein scores are derived from ions scores as a non-probabilistic basis for ranking protein hits.

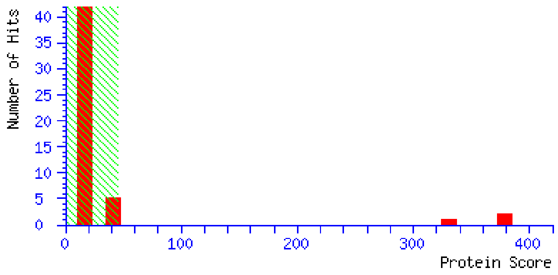

Matched peptide sequences: shown in Bold Red:

1 MGASTAQIHG LGAPSFAAAS MRKSNNVSSR SVFFGQKLGN SSAFPAAAFL
51 NLRSNTSRRN SSVRPLRIVN EKVVGIDLGT TNSAVAAMEG GKPTIVTNAE
101 GQRTTPSVVA YTKNGDRLVG QIAKRQAVVN PENTFFSVKR FIGRKMSEVD
151 EESKQVSYRV ERDENGNVKL ECPAIGK**QFA** **AEEISAQVLR** KLVDDASKFL
201 NDKVTK**AVVT** **VPAYFNDSQR** TATKDAGRIA GLEVLRIINE PTAASLAYGF
251 EKKSNETILV FDLGGGTFDV SVLEVGDGVF EVLSTSGDTH LGGDDFDKRI
301 VDWLAANFKR DEGIDLLKDK QALQRLTETA EKAKMELSSL TQANISLPFI
351 TATADGPKHI ETTITRAKFE ELCSDLLDRL KTPVENSLRD AK**LSFKDIDE**
401 **VILVGGSTR**I PAVQELVKKM TGKEPNVTVN PDEVVALGAA VQAGVLAGDV
451 SDIVLLDVSP LSLGLETLGG VMTKIIPRNT TLPTSKSEVF STAADGQTSV
501 EINVLQGERE FVRDNKSLGS FRLDGIPPAP RGVPQIEVKF DIDANGILSV
551 TAIDKGSGK**K** **QDITITGAST** **LPSDEVER**MV SEADKFAKED KEKRDAIDTK
601 NQADSVVYQT EKQLKELGDK VPGPVKEKVE SKLGELKEAI SGGSTEAIKE
651 AMAALNQEVM QLGQSLYNQP GAGAAPGPGA SSESGPSEST GKGPEGDVID
701 ADFSDSK

Spot No.: **D36**

NCBI accession No.: **gi|164604960** Species: *Hevea brasiliensis*

PFF score: [120] Protein name: acetyl-CoA C-acetyltransferase

Matched peptides No.: [2] Sequence coverage %: [5]

Matched sequences: R.ILVTLLGVLR.K;

M.APVAAAEIKPR.D

Calculated Mr: **41798** Calculated *p*I: **6.96**

Probability Based Mowse Score:

Ions score is -10*Log(P), where P is the probability that the observed match is a random event.
Individual ions scores > 45 indicate identity or extensive homology (p<0.05).
Protein scores are derived from ions scores as a non-probabilistic basis for ranking protein hits.

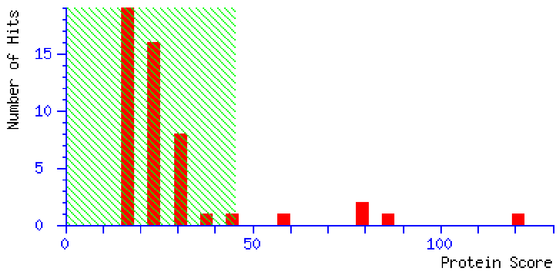

Matched peptide sequences: shown in Bold Red:

```
  1 MAPVAAAEIK PRDVCIVGVA RTPMGGFLGL LSTLPATKLG SIAIEAALKR
 51 ASVDPSLVQE VFFGNVLSAN LGQAPARQAA LGAGIPNSVV CTTVNKVCAS
101 GMKATMLAAQ SIQLGINDVV VAGGMESMSN APKYLAEARK GSRLGHDSLV
151 DGMLKDGLWD VYNDVGMGSC AEICADNHSI TREDQDKFAI HSFERGIAAQ
201 ESGAFAWEIV PVEVSGGRGK PLTIVDKDEG LGKFDPVKLR KLRPSFKENG
251 GTVTAGNASS ISDGAAALIL VSGETALKLG LQVIAKIRGY ADAAQAPELF
301 TTAPALAIPK TIANAGLDAS QVDYYEINEA FAVVALANQK LLGLNPEKVN
351 VHGGAVSLGH PLGCSGARIL VTLLGVLRKK NAKYGVGGVC NGGGGASALV
401 VELL
```

Spot No.: **D37**

NCBI accession No.: **gi|37622210** Species: *Hevea brasiliensis*

PFF score: [77] Protein name: small rubber particle protein

Matched peptides No.: [2] Sequence coverage %: [14]

Matched sequences: R.AVASEVHQSGVK.E;

R.VSSYLPLVPTER.I

Calculated Mr: **18929** Calculated *p*I: **8.99**

Probability Based Mowse Score:

Ions score is -10*Log(P), where P is the probability that the observed match is a random event.
Individual ions scores > 45 indicate identity or extensive homology (p<0.05).
Protein scores are derived from ions scores as a non-probabilistic basis for ranking protein hits.

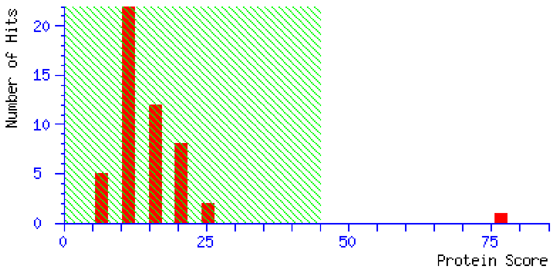

Matched peptide sequences: shown in Bold Red:

```
  1 KFHDVPNEVL KFVDRKVDES VTSLDSRVPP VVKQVSAQAY SVAREAPVAA
 51 RAVASEVHQS GVKETASGLA KTLYTKYEPK AKELYSKYEP KAEQCAVTAW
101 RRLNQLPLFP QVAQVVVPTA AYCSEKYNQT VLSTFEKGYR VSSYLPLVPT
151 ERIAKVFSDD VAQSMPLVSS
```

Spot No.: **D38**

NCBI accession No.: **gi|255555641** Species: *Ricinus communis*

PFF score: [109]

Protein name: 26S proteasome non-atpase regulatory subunit

Matched peptides No.: [2] Sequence coverage %: [12]

Matched sequences: K.HAVELEQSFMEGAYNR.V + Oxidation (M);
R.LPPSSQEYMILGLNLLR.L + Oxidation (M)

Calculated Mr: **30793** Calculated *p*I: **4.87**

Probability Based Mowse Score:

Ions score is -10*Log(P), where P is the probability that the observed match is a random event.
Individual ions scores > 46 indicate identity or extensive homology (p<0.05).
Protein scores are derived from ions scores as a non-probabilistic basis for ranking protein hits.

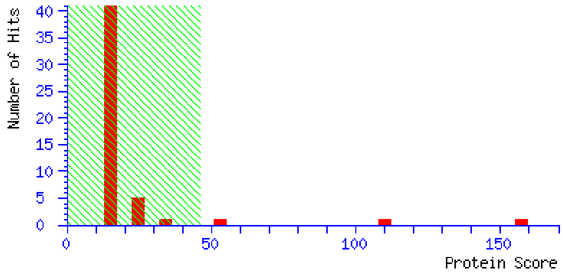

Matched peptide sequences: shown in Bold Red:

```
  1 MDPKITEASQ LFERFKAACV REDVDACTKL LSSLKVMLTE FRSLPPLFED
 51 TPNAIQELTL ARDIYEHAVL LNVKIGDQDA FERDFFQLKP YYTDASGRLP
101 PSSQEYMILG LNLLRLLVQN RIAEFHTELE LLSPAALENP AIKHAVELEQ
151 SFMEGAYNRV LSAKQTVPYK GYDYFMDLLA KTVRDEIAGC SEKAYDYLSI
201 NDARQMLLFS SDTELLQYIK EDHPEWEVKN GVVIFQKAKE SAPCKEIPSL
251 QLINQTLSYA RELERIV
```

(Bold red: **LP** (99–100), **PSSQEYMILG LNLLR** (101–115), **HAVELEQ** (144–150), **SFMEGAYNR** (151–159))

Spot No.: **D39**

NCBI accession No.: **gi|255541320**   Species: *Ricinus communis*

PFF score: [157]

Protein name: proteasome subunit beta type, putative

Matched peptides No.: [2]   Sequence coverage %: [10]

Matched sequences: K.HLIYQHQHNK.Q;
K.GCVYTYDAVGSYER.V

Calculated Mr: **24880**   Calculated *p*I: **6.08**

Probability Based Mowse Score:

Ions score is -10*Log(P), where P is the probability that the observed match is a random event.
Individual ions scores > 46 indicate identity or extensive homology (p<0.05).
Protein scores are derived from ions scores as a non-probabilistic basis for ranking protein hits.

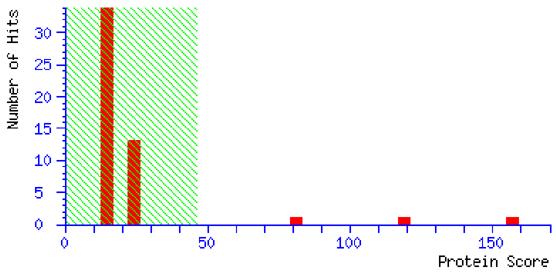

Matched peptide sequences: shown in Bold Red:

```
  1 MTKQQANWSP YDNNGGSCVA IAGADYCVIA ADTRMSTGYN ILTRDYSKIC
 51 QLAEKSVMAS SGFQADVQAL QKHLKAKHLI YQHQHNKQMS CPAMGQLLSN
101 TLYFKRFFPY YAFNVLGGLD SEGKGCVYTY DAVGSYERVG YSAQGSGSTL
151 IMPFLDNQLK SPSPLLLPAQ DAVTPLSEPE AVDLVKTVFA SATERDIYTG
201 DKVEIVVLNA DGIRREYMEL RKD
```

Spot No.: **D40**

NCBI accession No.: **gi|255570990** Species: *Ricinus communis*

PFF score: [459] Protein name: heat shock protein, putative

Matched peptides No.: [8] Sequence coverage %: [16]

Matched sequences: K.DIDEVILVGGSTR.I;K.QFAAEEISAQVLR.K;
K.AVVTVPAYFNDSQR.T;K.MSEVDEESKQVSYR.V + Oxidation (M);
R.QAVVNPENTFFSVKR.F;K.LSFKDIDEVILVGGSTR.I;
K.KQDITITGASTLPSDEVER.M;K.SEVFSTAADGQTSVEINVLQGER.E

Calculated Mr: **75431** Calculated *p*I: **5.35**

Probability Based Mowse Score:

Ions score is -10*Log(P), where P is the probability that the observed match is a random event.
Individual ions scores > 45 indicate identity or extensive homology (p<0.05).
Protein scores are derived from ions scores as a non-probabilistic basis for ranking protein hits.

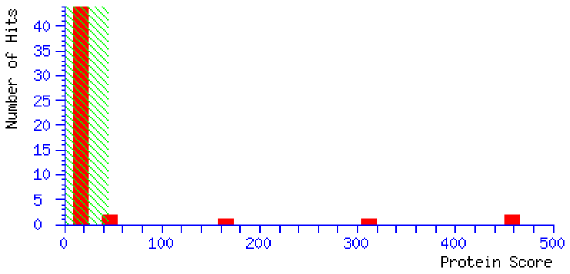

Matched peptide sequences: shown in Bold Red:

```
  1 MASSTAQIHV LGGIGFPSSK SRKPSFSFPK KTVFLGSNLK SHQTAAFLRQ
 51 SNTNARRKYG PLRVVNEKVV GIDLGTTNSA VAAMEGGKPT IVTNAEGQRT
101 TPSVVAYTKN GDRLVGQIAK RQAVVNPENT FFSVKRFIGR KMSEVDEESK
151 QVSYRVVRDE NGNVKLDCPA IGKQFAAEEI SAQVLRKLVD DASKFLNDKV
201 TKAVVTVPAY FNDSQRTATK DAGRIAGLEV LRIINEPTAA SLAYGFEKKN
251 NETILVFDLG GGTFDVSVLE VGDGVFEVLS TSGDTHLGGD DFDKRIVDWL
301 AGNFKRDEGI DLLKDKQALQ RLTETAEKAK MELSSLTQTN ISLPFITATA
351 DGPKHIETTI TRAKFEELCS DLLDRLKTPV ENSLRDAKLS FKDIDEVILV
401 GGSTRIPAVQ ELVKKMTGKD PNVTVNPDEV VALGAAVQAG VLSGDVSDIV
451 LLDVTPLSLG LETLGGVMTK IIPRNTTLPT SKSEVFSTAA DGQTSVEINV
501 LQGEREFVRD NKSLGSFRLD GIPPAPRGVP QIEVKFDIDA NGILSVTAID
551 KGTGKKQDIT ITGASTLPSD EVERMVSEAE RFAKEDKEKR DAIDTKNQAD
601 SVVYQTEKQL KELGDKVPAP VKEKVEAKLK ELKDAIEGGS TQGMKDAMAA
651 LNQEVMQLGQ SLYNQPGAGG AGPAPGGEAG PSSDSSSKGP EGDVIDADFT
701 DSK
```

Spot No.: **D41**

NCBI accession No.: **gi|351726646** Species: *Glycine max*

PFF score: [97]

Protein name: uncharacterized protein LOC100499738

Matched peptides No.: [5] Sequence coverage %: [28]

Matched sequences: K.NVALYQFR.N;
K.LIAASGEPGDR.V;
K.SNEDKIMLLDSHK.L + Oxidation (M);
R.HYHSGMSVEEAIDLVDK.C;
K.LIAASGEPGDRVQFTEYIQK.N

Calculated Mr: **22699** Calculated *p*I: **5.86**

Probability Based Mowse Score:

Ions score is -10*Log(P), where P is the probability that the observed match is a random event.
Individual ions scores > 46 indicate identity or extensive homology (p<0.05).
Protein scores are derived from ions scores as a non-probabilistic basis for ranking protein hits.

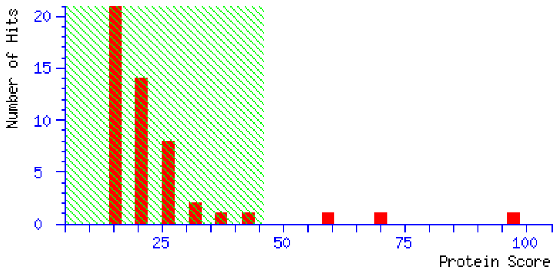

Matched peptide sequences: shown in Bold Red:

```
  1 MECVFGLVGN GFAIVVADSS AVHSILVHKS NEDKIMLLDS HKLIAASGEP
 51 GDRVQFTEYI QKNVALYQFR NGIPLTTAAA ANFTRGELAT ALRKNPYSVN
101 ILLAGYDKET GPELYYIDYI ATLHKLEKGA FGYGSYFSLS MMDRHYHSGM
151 SVEEAIDLVD KCIMEIRSRL VVAPPNFVIK IVDKDGAREY AWRESVKDTP
201 VPSA
```

Spot No.: **D42**

NCBI accession No.: **gi|166627** Species: *Arabidopsis thaliana*

PFF score: [111]

Protein name: nucleotide-binding subunit of vacuolar ATPase

Matched peptides No.: [3] Sequence coverage %: [7]

Matched sequences: K.YQEIVNIR.L;
R.QIYPPINVLPSLSR.L;
R.GYPGYMYTDLATIYER.A + Oxidation (M)

Calculated Mr: **54819** Calculated *p*I: **4.98**

Probability Based Mowse Score:

Ions score is -10*Log(P), where P is the probability that the observed match is a random event.
Individual ions scores > 46 indicate identity or extensive homology (p<0.05).
Protein scores are derived from ions scores as a non-probabilistic basis for ranking protein hits.

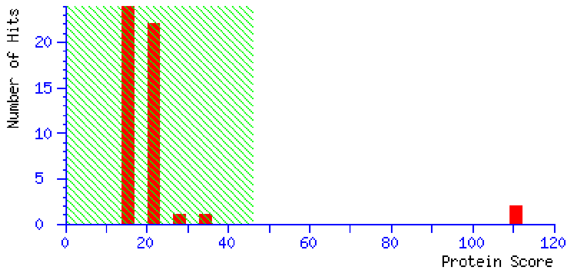

Matched peptide sequences: shown in Bold Red:

```
  1 MSLSVNMGTN DLDIEEGTLE IGMEYRTVSG VAGPLVILDK VKGPKYQEIV
 51 NIRLGDGSTR RGQVLEVDGE KAVVQVFEGT SGIDNKFTTV QFTGEVLKTP
101 VSLDMLGRIF NGSGKPIDNG PPILPEAYLD ISGSSINPSE RTYPEEMIQT
151 GISTIDVMNS IARGQKIPLF SAAGLPHNEI AAQICRQAGL VKRLEKTVDL
201 LEDHGEDNFA IVFAAMGVNM ETAQFFKRDF EENGSMERVT LFLNLANDPT
251 IERIITPRIA LTTAEYLAYE CGKHVLVILT DMSSYADALR EVSAAREEVP
301 GRRGYPGYMY TDLATIYERA GRIEGRKGSI TQIPILTMPN DDITHPTPDL
351 TGYITEGQIY IDRQLHNRQI YPPINVLPSL SRLMKSAIGE GMTRKDHSDV
401 SNQLYANYAI GKDVQAMKAV VGEEALSSED LLYLEFLDKF ERKFVMQGAY
451 DTRNIFQSLD LAWTLLRIFP RELLHRIPAK TLDQFYSRDS TS
```

Spot No.: **D43**

NCBI accession No.: **gi|132270** Species: *Hevea brasiliensis*

PFF score: [55]

Protein name: Rubber elongation factor protein

Matched peptides No.: [2] Sequence coverage %: [17]

Matched sequences: K.NVAVPLYNR.F;

K.FVDSTVVASVTIIDR.S

Calculated Mr: **14713** Calculated *p*I: **5.04**

Probability Based Mowse Score:

Ions score is -10*Log(P), where P is the probability that the observed match is a random event.
Individual ions scores > 46 indicate identity or extensive homology (p<0.05).
Protein scores are derived from ions scores as a non-probabilistic basis for ranking protein hits.

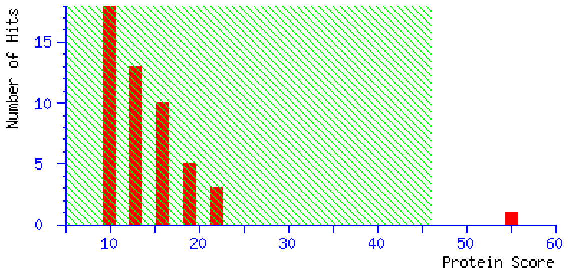

Matched peptide sequences: shown in Bold Red:

```
  1 MAEDEDNQQG QGEGLKYLGF VQDAATYAVT TFSNVYLFAK DKSGPLQPGV
 51 DIIEGPVKNV AVPLYNRFSY IPNGALKFVD STVVASVTII DRSLPPIVKD
101 ASIQVVSAIR AAPEAARSLA SSLPGQTKIL AKVFYGEN
```

Spot No.: **D44**

NCBI accession No.: **gi|255583952** Species: *Ricinus communis*

PFF score: [366]

Protein name: proteasome subunit alpha type, putative

Matched peptides No.: [6] Sequence coverage %: [26]

Matched sequences: K.ATEIEVGVVR.T;R.HITIFSPEGR.L;
K.EQEAINFLEK.K;K.YGYEMPVDVLAK.W + Oxidation (M);
K.LLDHTSVTHLFPITK.Y;K.ATSAGLKEQEAINFLEK.K

Calculated Mr: **27504** Calculated *p*I: **5.84**

Probability Based Mowse Score:

Ions score is -10*Log(P), where P is the probability that the observed match is a random event.
Individual ions scores > 46 indicate identity or extensive homology (p<0.05).
Protein scores are derived from ions scores as a non-probabilistic basis for ranking protein hits.

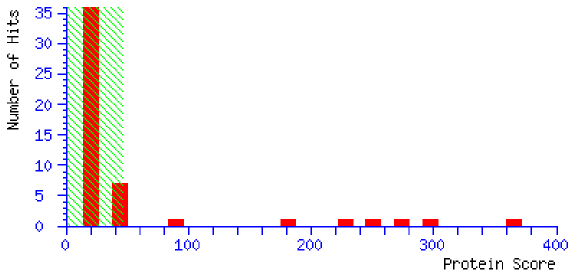

Matched peptide sequences: shown in Bold Red:

```
  1 MSRGSGGGYD RHITIFSPEG RLFQVEYAFK AVKASGVTSI GVCGKDSVCV
 51 VTQKKVPDKL LDHTSVTHLF PITKYLGLLA TGMTADARTL VQQARNEAAE
101 FRFKYGYEMP VDVLAKWIAD KSQIYTQHAY MRPLGVVAMV LGIDDEYGPR
151 LYKCDPAGHF FGHKATSAGL KEQEAINFLE KKMKNDPAFT YEETVQTAIS
201 ALQSVLQEDF KATEIEVGVV RTENPVFRVL STEEIDEHLT AISERD
```

Spot No.: **D45**

NCBI accession No.: **gi|356547978** Species: *Glycine max*

PFF score: [189]

Protein name: eukaryotic translation initiation factor 3 subunit K-like

Matched peptides No.: [3] Sequence coverage %: [11]

Matched sequences: K.NAADSVPLEHITR.I;
K.TLIVLSHYLETGR.F;
K.KNAADSVPLEHITR.I

Calculated Mr: **26266** Calculated *p*I: **5.48**

Probability Based Mowse Score:

Ions score is -10*Log(P), where P is the probability that the observed match is a random event.
Individual ions scores > 46 indicate identity or extensive homology (p<0.05).
Protein scores are derived from ions scores as a non-probabilistic basis for ranking protein hits.

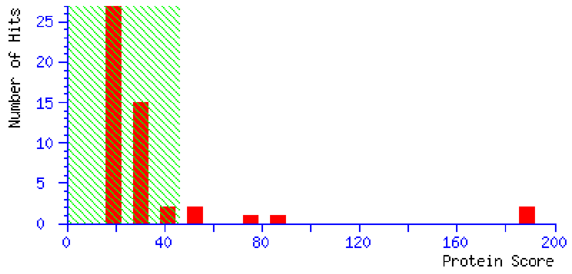

Matched peptide sequences: shown in Bold Red:

```
  1 MGREKETTKV AYAVEQLVAF NRYNPDILPD LENYVNDQVS SQTYSLDANL
 51 CLLRLYQFEP EKMSSQIVAR ILVKALMAMP APDFSLCLFL IPERVQMEEQ
101 FKTLIVLSHY LETGRFRQFW DEAAKSRHIV EAVPGFEQAI QGYAIHVLSL
151 TYQRIPRTVL AEAINIEGLS LDKFLENQVA NNGWVIEKSQ GRAQLIVLPR
201 NEFNDPALKK NAADSVPLEH ITRIFPILS
```

Spot No.: **D46**

NCBI accession No.: **gi|310772392** Species: *Malpighia glabra*

PFF score: [78]

Protein name: dehydroascorbate reductase

Matched peptides No.: [1] Sequence coverage %: [4]

Matched sequences: K.EHVIAGWEPK.V

Calculated Mr: **23725** Calculated *p*I: **6.40**

Probability Based Mowse Score:

Ions score is -10*Log(P), where P is the probability that the observed match is a random event.
Individual ions scores > 46 indicate identity or extensive homology (p<0.05).
Protein scores are derived from ions scores as a non-probabilistic basis for ranking protein hits.

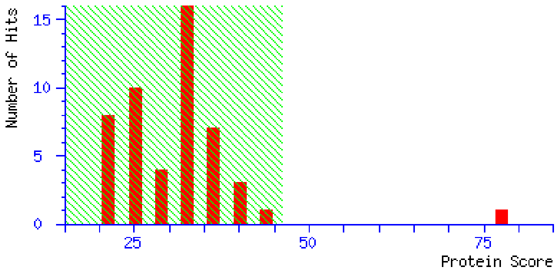

Matched peptide sequences: shown in Bold Red:

```
  1 MALEICVKAA VGAPNVLGDC PFSQRALLTL EEKKLSYKRH LFDLSNKPQW
 51 FLEISPEGKV PVLKLDDKWV ADSDVIVGLL EEKYPEPSLK TPPEFSSVGS
101 NIFSSFVKFL KSKDANDGTE KALLEELVAL DDHLKAHGPF VAGERVTAVD
151 LSLAPKLYHL EIALGHFKKW TVPESLSHVR NYMKALFARE SFEKTKAAKE
201 HVIAGWEPKV NA
```

Spot No.: **D47**

NCBI accession No.: **gi|255547584** Species: *Ricinus communis*

PFF score: [370]

Protein name: Regulator of ribonuclease activity A, putative

Matched peptides No.: [5] Sequence coverage %: [33]

Matched sequences: R.VLVVDGGGSLR.C;

K.VFEDNVLIR.E;
R.ALQPIFQIYGR.R;
R.RQVFSGPVVTLK.V;
R.DVDEINGCDIGVR.A

Calculated Mr: **17958** Calculated *p*I: **5.39**

Probability Based Mowse Score:

Ions score is -10*Log(P), where P is the probability that the observed match is a random event.
Individual ions scores > 46 indicate identity or extensive homology (p<0.05).
Protein scores are derived from ions scores as a non-probabilistic basis for ranking protein hits.

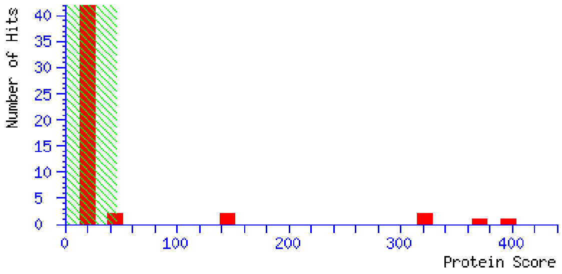

Matched peptide sequences: shown in Bold Red:

```
  1 MALVTTAEVC DANPQLIVSG ELRALQPIFQ IYGRRQVFSG PVVTLKVFED
 51 NVLIREFLEE KGNGRVLVVD GGGSLRCAIL GGNPVVQAQN NGWAGILVNG
101 CIRDVDEING CDIGVRALAS HPMKANKKGI GEKHVPITIG GTRISDGEFL
151 YADTDGILIS RNELSV
```

Spot No.: **D48**

NCBI accession No.: **gi|255568992** Species: *Ricinus communis*

PFF score: [128]

Protein name: conserved hypothetical protein

Matched peptides No.: [2] Sequence coverage %: [8]

Matched sequences: K.WNKGDNQR.W;

R.MVNNIHLNVDAFHGDKK.S

Calculated Mr: **35473** Calculated *p*I: **6.29**

Probability Based Mowse Score:

Ions score is -10*Log(P), where P is the probability that the observed match is a random event.
Individual ions scores > 45 indicate identity or extensive homology (p<0.05).
Protein scores are derived from ions scores as a non-probabilistic basis for ranking protein hits.

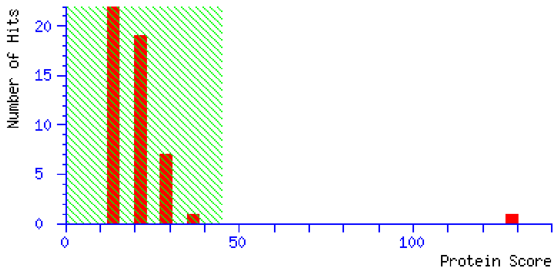

Matched peptide sequences: shown in Bold Red:

```
  1 MEFPFGHQSH THHHHQRNNA DDDDESRQFY PPPASAPPPP YTRDNEFAPP
 51 PPYFQEPDFP PSPTRPYYRE NEYAPPPPPP PVQQAHVYHS SHNQELDSDY
101 YPPRPPTQVT HVSHERIGTE TQTHHSFQPH FPSFLQHHTH QSGSASGLDL
151 SNKPTYKVYS KAEPNFHLTI RDGKVVLAPS DPSDEFQNWY KDERYSTRVK
201 DEEGFPCFAL VNKASGQAMK HSIGGTHPVQ LIPYNPNVLD ESILWTESKD
251 LGDGYRAVRM VNNIHLNVDA FHGDKKSGGV HNGTTIVLWK WNKGDNQRWR
301 ITPHCKSLN
```

Spot No.: **D49**

NCBI accession No.: **gi|356522666** Species: *Glycine max*

PFF score: [206]

Protein name: ubiquitin carboxyl-terminal hydrolase 6-like

Matched peptides No.: [3] Sequence coverage %: [7]

Matched sequences: R.YLTVQFVR.F;

K.CQLYDLTGVPPER.Q;

K.CHISQEVNHLHEGIK.H

Calculated Mr: **54602** Calculated *p*I: **5.78**

Probability Based Mowse Score:

Ions score is -10*Log(P), where P is the probability that the observed match is a random event.
Individual ions scores > 46 indicate identity or extensive homology (p<0.05).
Protein scores are derived from ions scores as a non-probabilistic basis for ranking protein hits.

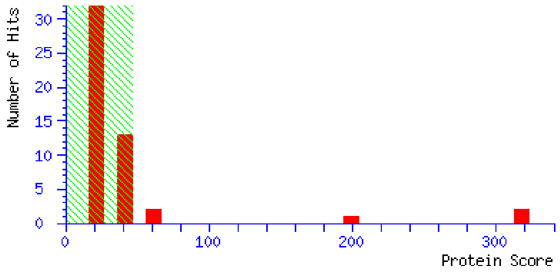

Matched peptide sequences: shown in Bold Red:

```
  1 MITVSVRWQK EIFKDVELDT TQSPYVVFKCQ LYDLTGVPPE RQKIMVKGGL
 51 LKDDADWSTV GVKEGQKLMM MGTADEVVKT PEKGTVFVED LPEEEQVVAV
101 GHTAGLFNLG NTCYMNSTLQ CLHSVPELKS ALTNYSHSGR NNDVDQSSHL
151 LTIATRDLFS ELDKSVKPVA PMQFWMVLRK KYPQFGQLHN GVFMQQDAEE
201 CWTQLLYTLS QSLRSPGSSE NPDAVKALFG IELISRIHCQ ESKEESSEAE
251 SVYSLKCHIS QEVNHLHEGI KHGLKSELEK ASLVLGRSAI HLKESRINAL
301 PRYLTVQFVR FFWKRESNQK AKILRKVDYP LELDVYDFCS DELRKKLEAP
351 RQILRNEEGK KLGLKVNEKS SVQKENDVKM SDAEGATSGG GEPSVVPMEE
401 GEKETQMTGI YDLVAVLTHK GRSADSGHYV GWVKQENGKW IEFDDDNPKP
451 KLEEDITRLS GGGDWHMAYI IMYKARVVSV
```

Spot No.: **D50**

NCBI accession No.: **gi|224099853**

Species: *Populus trichocarpa* PFF score: [120]

Protein name: conserved hypothetical protein

Matched peptides No.: [2] Sequence coverage %: [9]

Matched sequences: K.GCFFTTSGGLR.S;

R.SFEHPLNELKPVPR.V

Calculated Mr: **27448** Calculated *p*I: **5.84**

Probability Based Mowse Score:

Ions score is -10*Log(P), where P is the probability that the observed match is a random event.
Individual ions scores > 46 indicate identity or extensive homology (p<0.05).
Protein scores are derived from ions scores as a non-probabilistic basis for ranking protein hits.

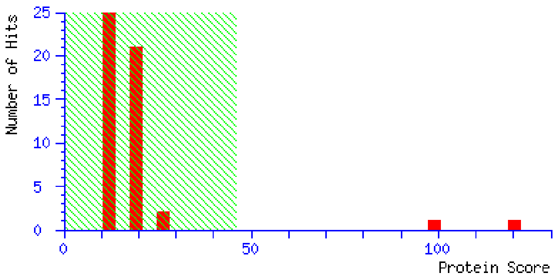

Matched peptide sequences: shown in Bold Red:

```
  1 MLAVFDNTVA KCPDALQSPH SAPASSALKD GFLANHFASQ HPGSVTVNLG
 51 TSGLISHSVE KQNPFLPRLF AVVDDIFCLF QGHIDNVAVL KQQYGLNKTA
101 NEVIVVIEAY RTLRDRGPYP ADQVVKDIQG KFAFILYDST SKATFFAADA
151 DGSVPFFWGA DSEGNLVLSD DVQIVQKGCG KSFAPFPKGC FFTTSGGLRS
201 FEHPLNELKP VPRVDSSGQV CGATFKVDAE TKKESVGMPR VDSSYNWSSN
251 Y
```

Spot No.: **D51**

NCBI accession No.: **gi|255568794**

Species: *Ricinus communis*

Protein name: nucleoredoxin, putative PFF score: [380]

Matched peptides No.: [6] Sequence coverage %: [9]

Matched sequences: K.LIEAYHEIK.A;K.KCDFDLHPK.C;
K.LVELAEIEKAR.L;K.NQTLSSILVSSSR.D;
K.KNQTLSSILVSSSR.D;R.VEYFRQEEENAK.K

Calculated Mr: **63728** Calculated *p*I: **5.46**

Probability Based Mowse Score:

Ions score is -10*Log(P), where P is the probability that the observed match is a random event.
Individual ions scores > 46 indicate identity or extensive homology (p<0.05).
Protein scores are derived from ions scores as a non-probabilistic basis for ranking protein hits.

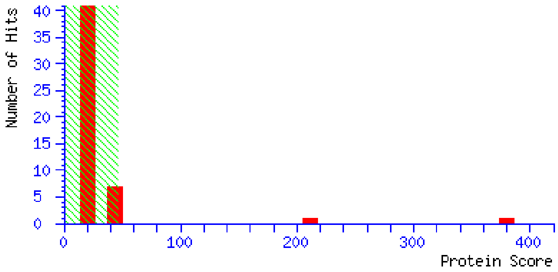

Matched peptide sequences: shown in Bold Red:

```
  1 MADDPVIDVS HDLLSLLSSD DRDFLIRSNG DQVKISNLVG KIVGLYFSGS
 51 WCGPCRHFTP NLVQVYEELS LKGDFEVVFI SSDRDAESFD AYFSKMPWLA
101 IPFSDQETCK HLKDLFKVRG IPNLVFLDAD GKVSCDQGVR FIREYGAEGY
151 PFTPERVEYF RQEEENAKKN QTLSSILVSS SRDFLISKDG TKIPVSELEG
201 KMVGLYFSVH SHRLCLDFTP RLEEVYKKLK EKGEKFEVVL ISMDYDENNF
251 KQGLETMPWL ALPFEDKSRE RLARYFELSA LPTLVIIGED GKTLNKNVAE
301 LIEGHGIQAY PFTPEKLVEL AEIEKARLEA QTLESVLVHG DKDFVIEESG
351 SKVPVSELVG KNILLYFSAK WCPPCRAFLP KLIEAYHEIK AKDNAFEIIF
401 ISSDRDQSSF DEFYTEMPWL ALPFGDDRKT ILQRKFKIKG IPAAIAISPT
451 GKTLTKEARE HITAYGADAY PFNEDHLKQL NDKQEEIAKG WPEKVRHELH
501 PEHELVRMKR NGYGCDGCKE AGSGWSFYCK KCDFDLHPKC ALKKEENGEK
551 VKK
```

Spot No.: **D52**

NCBI accession No.: **gi|14423933** Species: *Hevea brasiliensis*

Protein name: Small rubber particle protein PFF score: [137]

Matched peptides No.: [2] Sequence coverage %: [16]

Matched sequences: K.QVSAQTYSVAQDAPR.I;

K.DISGPLKPGVDTIENVVK.T

Calculated Mr: **22331** Calculated *p*I: **4.80**

Probability Based Mowse Score:

Ions score is -10*Log(P), where P is the probability that the observed match is a random event.
Individual ions scores > 46 indicate identity or extensive homology (p<0.05).
Protein scores are derived from ions scores as a non-probabilistic basis for ranking protein hits.

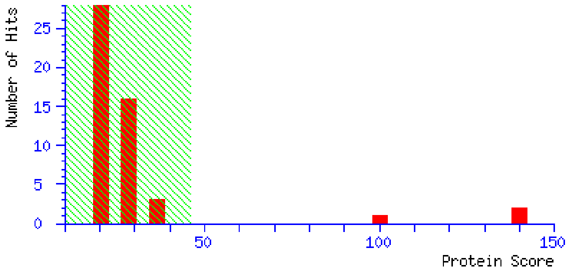

Matched peptide sequences: shown in Bold Red:

```
  1 MAEEVEEERL KYLDFVRAAG VYAVDSFSTL YLYAKDISGP LKPGVDTIEN
 51 VVKTVVTPVY YIPLEAVKFV DKTVDVSVTS LDGVVPPVIK QVSAQTYSVA
101 QDAPRIVLDV ASSVFNTGVQ EGAKALYANL EPKAEQYAVI TWRALNKLPL
151 VPQVANVVVP TAVYFSEKYN DVVRGTTEQG YRVSSYLPLL PTEKITKVFG
201 DEAS
```

Spot No.: **D53**

NCBI accession No.: **gi|333690873** Species: *Hevea brasiliensis*

PFF score: [132]

Protein name: Small rubber particle protein

Matched peptides No.: [4] Sequence coverage %: [18]

Matched sequences: R.FWADFIDKK.I;

K.YEYREEDLR.N;

K.SPFLPSDPYQR.A;

K.SPLLLQMNPVHK.K + Oxidation (M)

Calculated Mr: **25595** Calculated *p*I: **5.48**

Probability Based Mowse Score:

Ions score is -10*Log(P), where P is the probability that the observed match is a random event.
Individual ions scores > 46 indicate identity or extensive homology (p<0.05).
Protein scores are derived from ions scores as a non-probabilistic basis for ranking protein hits.

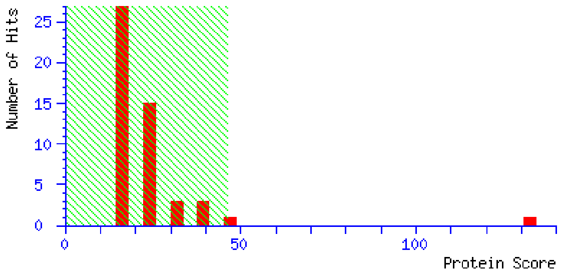

Matched peptide sequences: shown in Bold Red:

```
  1 MAEEVILLDF WPSPFGMRVR IALAEKGVKY EYREEDLRNK SPLLLQMNPV
 51 HKKIPVLIHN GKPICESLIA VQYVDEVWKD KSPFLPSDPY QRAQARFWAD
101 FIDKKIYDIG RKIWTTKGDE QEAAKKEFIE ALKLLEGELG NKPYFGGESM
151 GYVDVALIPF YSWFYAYETC GNFSIEPECP VLIAWAKRCL QKESVSESVP
201 DPEKVYEFVL VLKKKFGIE
```

Spot No.: **D54**

NCBI accession No.: **gi|356543702** Species: *Glycine max*

PFF score: [105]

Protein name: PREDICTED: (+)-pulegone reductase-like

Matched peptides No.: [1] Sequence coverage %: [4]

Matched sequences: K.YFPDGIDVYLDNVGGK.M

Calculated Mr: **38361** Calculated *p*I: **5.71**

Probability Based Mowse Score:

Ions score is -10*Log(P), where P is the probability that the observed match is a random event.
Individual ions scores > 46 indicate identity or extensive homology (p<0.05).
Protein scores are derived from ions scores as a non-probabilistic basis for ranking protein hits.

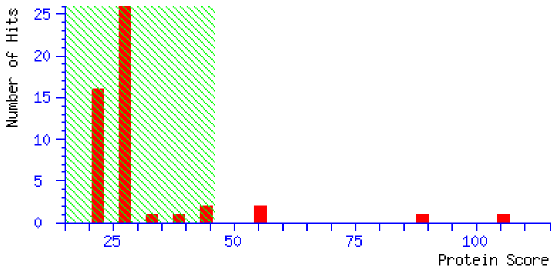

Matched peptide sequences: shown in Bold Red:

```
  1 MGSVVESREW YLAAYSPHGV PNSDNLKLRT VALSLSSDSI PDGHVSLQIL
 51 FLSVDPYLRT RLTGTLDGLY IQQYPLNEVI TAYGVGRVIG SKDSKYTEGD
101 LILTPSAPVA EYCILPSSRV IRKIDAASGI SLPDYLSALG VPGFAAWVGI
151 VVLGDPKPGS NVFISAASGA VGMSAGQLAK IRGCRVIGST GSDEKVKLIK
201 EEFGYDDGFN YNKESDFDAA LSKYFPDGID VYLDNVGGKM LESVLNHVNK
251 YARIPLCGMI SQYNKVWTER EGVRNLLNMV GKEVRMEGFM LESYWHRFED
301 FAKEMEGYIK EGKVTSKNKI NIGIESFLDS LASLFSSSNI GKVVVQVNKA
```

Spot No.: **D55**

NCBI accession No.: **gi|351721881** Species: *Glycine max*

PFF score: [56]

Protein name: 18.5 kDa class I heat shock protein

Matched peptides No.: [3] Sequence coverage %: [16]

Matched sequences: R.ENSAFVSTR.V;

K.ETPEAHVFK.A;

K.EDKNDTWHR.V

Calculated Mr: **18491** Calculated *p*I: **5.82**

Probability Based Mowse Score:

Ions score is -10*Log(P), where P is the probability that the observed match is a random event.
Individual ions scores > 45 indicate identity or extensive homology (p<0.05).
Protein scores are derived from ions scores as a non-probabilistic basis for ranking protein hits.

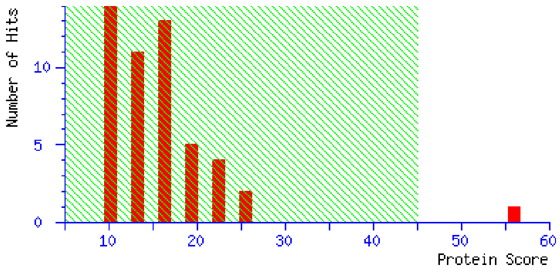

Matched peptide sequences: shown in Bold Red:

```
  1 MSLIPNFFGG RRNNVFDPFS LDVWDPFKDF PFPNTLSSAS FPEFSRENSA
 51 FVSTRVDWKE TPEAHVFKAD IPGLKKEEVK VQIEDDKVLQ ISGERNVEKE
101 DKNDTWHRVE RSSGKFMRRF RLPENAKVEQ VKASMENGVL TVTVPKEEVK
151 KPDVKAIEIS G
```

Spot No.: **D56**

NCBI accession No.: **gi|462406561** Species: *Prunus persica*

PFF score: [96]

Protein name: hypothetical protein PRUPE_ppa020028mg, partial

Matched peptides No.: [2] Sequence coverage %: [5]

Matched sequences: R.VDYQGYIFPR.R;

K.QEQAAFIEEISR.T

Calculated Mr: **48394** Calculated *p*I: **5.02**

Probability Based Mowse Score:

Ions score is -10*Log(P), where P is the probability that the observed match is a random event.
Individual ions scores > 46 indicate identity or extensive homology (p<0.05).
Protein scores are derived from ions scores as a non-probabilistic basis for ranking protein hits.

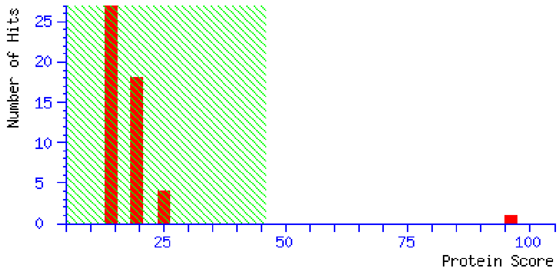

Matched peptide sequences: shown in Bold Red:

```
  1 MEGLIKGLID VALGHDENRN EDPGPRDRQE QSRSTWAQVV SGEEQDENDS
 51 DRPHGSKQSH WNRQEQSHLG RAEWDNVDSE PSRRPHKAAH EGYERNDDGG
101 RHGYNQNYSR RKDEEENKDG WETVGKRPPR QQPHKIQMGQ WNGYKRPSTE
151 QEYSDEAEVG AKIEPSEEEL SDLSQACSKI WELDLNRLVP GKDYEIDCGG
201 GKKVYQKEDL AEGSLFTWLS EDIFRRPTFS RFCSLLDNYN PNEGCKEVIT
251 SEEKQEQAAF IEEISRTAPI KYLHKYLSRK GILSESYQDF KRTLTTLWFD
301 LYGRGGASAS SSAFEHVFVG EIKQRGEEEV SGFHNWLQFY LEEAKGRVDY
351 QGYIFPRRRG QTPDSETQLL TIQFEWNGVL KAVSSTLVGV SPEFEIALYT
401 LCFFMGGEDN HIELGPYQ
```

Spot No.: **D57**

NCBI accession No.: **gi|255570177** Species: *Ricinus communis*

PFF score: [56]

Protein name: proteasome subunit beta type 7,10, putative

Matched peptides No.: [1] Sequence coverage %: [3]

Matched sequences: R.EGLNRDEGIK.L

Calculated Mr: **30600** Calculated *p*I: **7.60**

Probability Based Mowse Score:

Ions score is -10*Log(P), where P is the probability that the observed match is a random event.
Individual ions scores > 46 indicate identity or extensive homology (p<0.05).
Protein scores are derived from ions scores as a non-probabilistic basis for ranking protein hits.

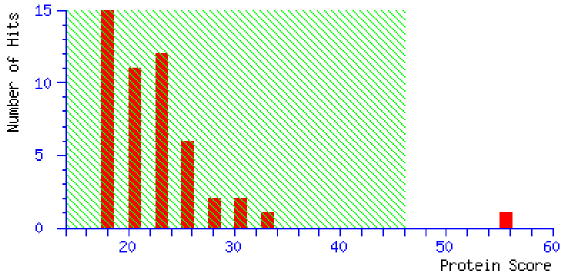

Matched peptide sequences: shown in Bold Red:

```
  1 MSKSAIDVPP KGGFSFDLCK RNEMLAKKGV NLPSSYRKTG TTIVGLIFQD
 51 GVILGADTRA TEGPIVCDKN CEKIHYMAPN IYCCGAGTAA DTEAVTDMVS
101 SQLQLHRYHT GRESRVITAL TLLKKHLFNY QGYVQAALVL GGVDCTGPHL
151 HTIYPHGSTD TLPFATMGSG SLAAMAIFES KYREGLNRDE GIKLVTEAIC
201 SGVFNDLGSG SNVDVCVITK GHKEYLRNHL LPNPRTYINP KGYVFSKKTE
251 VLLTKITPLK EPSKEKEGVS EGGEAMEE
```

Matched (bold red): **EGLNRDE GIK** (residues 184–193)

Spot No.: **D58**

NCBI accession No.: **gi|3834310**   Species: *Arabidopsis thaliana*

PFF score: [197]

Protein name: Similar to Ubiquitin-conjugating enzyme E2-17 KD gb|D83004 from Homo sapiens

Matched peptides No.: [3]   Sequence coverage %: [24]

Matched sequences: K.DKWSPALQIR.T;
K.IYHPNIDKLGR.I;
R.LLSEPAPGISASPSEDNMR.Y + Oxidation (M)

Calculated Mr: **18339**   Calculated *p*I: **5.93**

Probability Based Mowse Score:

Ions score is -10*Log(P), where P is the probability that the observed match is a random event.
Individual ions scores > 46 indicate identity or extensive homology (p<0.05).
Protein scores are derived from ions scores as a non-probabilistic basis for ranking protein hits.

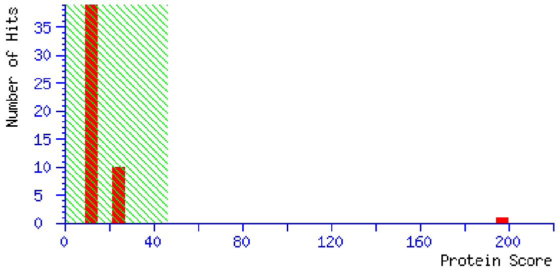

Matched peptide sequences: shown in Bold Red:

1 MANSNLPRRI IKETQR**LLSE** **PAPGISASPS** **EDNMR**YFNVM ILGPTQSPYE
51 GGVFKLELFL PEEYPMAAPK VRFLTK**IYHP** **NIDKLGR**ICL DILK**DKWSPA**
101 **LQIR**TVLLRS IQALLSAPNP DDPLSENIAK HWKSNEAEAV DTGDLRNGPV
151 FMQAAHDKLE GEN

Spot No.: **D59**

NCBI accession No.: **gi|315937232**   Species: *Jatropha curcas*

PFF score: [256]

Protein name: ubiquitin-conjugating family protein

Matched peptides No.: [6]   Sequence coverage %: [48]

Matched sequences: K.KEMAAPHNR.K + Oxidation (M);
K.FGLLANWQR.E;
M.TLGSGGSSVVVPR.N;
R.KLVQPPEGTYF.-;
R.EYTMEDILTQLK.K + Oxidation (M);
R.SWTGTIIGPHNTVHEGR.I

Calculated Mr: **16706**   Calculated *p*I: **6.20**

Probability Based Mowse Score:

Ions score is -10*Log(P), where P is the probability that the observed match is a random event.
Individual ions scores > 46 indicate identity or extensive homology (p<0.05).
Protein scores are derived from ions scores as a non-probabilistic basis for ranking protein hits.

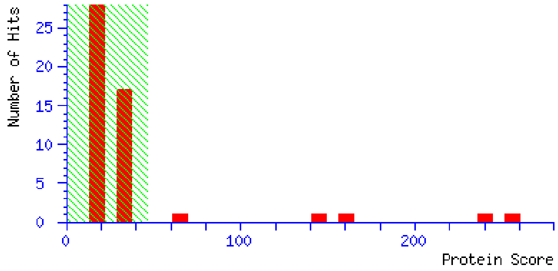

Matched peptide sequences: shown in Bold Red:

1 **MTLGSGGSSV VVPR**NFRLLE ELERGEKGIG DGTVSYGMDD GDDIYMR**SWT**
51 **GTIIGPHNTV HEGR**IYQLKL FCDKDYPEKP PSVRFHSRIN MTCVNHETGV
101 VEPKK**FGLLA NWQREYTMED ILTQLKKEMA APHNRKLVQP PEGTYF**

Spot No.: **D60**

NCBI accession No.: **gi|226531470** Species: *Zea mays*

PFF score: [271]

Protein name: vacuolar ATP synthase subunit B

Matched peptides No.: [8] Sequence coverage %: [20]

Matched sequences: K.YQEIVNIR.L;
K.TLDQYYSR.D;
K.FVTQGAYDTR.N;
R.RGQVLEVDGEK.A;
R.QIYPPINVLPSLSR.L;
R.VTLFLNLANDPTIER.I;
R.GYPGYMYTDLATIYER.A + Oxidation (M);
K.IPLFSAAGLPHNEIAAQICR.Q

Calculated Mr: **54142** Calculated *p*I: **5.07**

Probability Based Mowse Score:

Ions score is -10*Log(P), where P is the probability that the observed match is a random event.
Individual ions scores > 46 indicate identity or extensive homology (p<0.05).
Protein scores are derived from ions scores as a non-probabilistic basis for ranking protein hits.

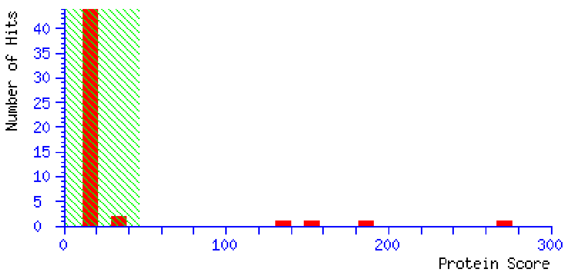

Matched peptide sequences: shown in Bold Red:

1	MGLVKEGVDM	EEGTLEIGME	YRTVSGVAGP	LVILDKVKGP	K**YQEIVNIR**L
51	GDGTTR**RGQV**	**LEVDGEK**AVV	QVFEGTSGID	NKYTTVQFTG	EVLKTPVSLD
101	MLGRIFNGSG	KPIDNGPPIL	PEAYLDISGS	SINPSERTYP	EEMIQTGIST
151	IDVMNSIARG	QK**IPLFSAAG**	**LPHNEIAAQI**	**CR**QAGLVKTL	EKGKHAEGGE
201	DDNFAIVFAA	MGVNMETAQF	FKRDFEENGS	MER**VTLFLNL**	**ANDPTIER**II
251	TPRIALTTAE	YLAYECGKHV	LVILTDMSSY	ADALREVSAA	REEVPGRR**GY**
301	**PGYMYTDLAT**	**IYER**AGRIEG	RTGSITQIPI	LTMPNDDITH	PTPDLTGYIT
351	EGQIYIDRQL	HNR**QIYPPIN**	**VLPSLSR**LMK	SAIGEGMTRR	DHSDVSNQLY
401	ANYAIGKDVQ	AMKAVVGEEA	LSSEDLLYLE	FLDKFERK**FV**	**TQGAYDTR**NI
451	FQSLDLAWTL	LRIFPRELLH	RIPAK**TLDQY**	**YSR**DASH	

Spot No.: **D61**

NCBI accession No.: **gi|2832430** Species: *Hevea brasiliensis*

PFF score: [175] Protein name: prohevein

Matched peptides No.: [3] Sequence coverage %: [22]

Matched sequences: K.CLSVTNTGTGAK.A;

R.QLDTDGKGYER.G;

K.YGWTAFCGPVGAHGQPSCGK.C

Calculated Mr: **20852** Calculated *p*I: **5.64**

Probability Based Mowse Score:

Ions score is -10*Log(P), where P is the probability that the observed match is a random event.
Individual ions scores > 46 indicate identity or extensive homology (p<0.05).
Protein scores are derived from ions scores as a non-probabilistic basis for ranking protein hits.

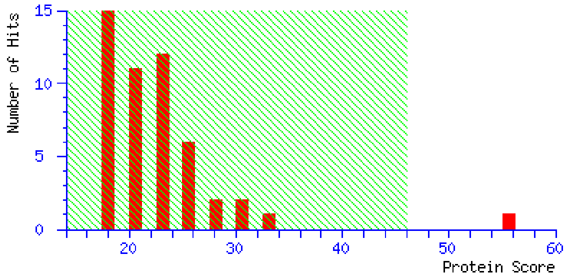

Matched peptide sequences: shown in Bold Red:

```
  1 MSKSAIDVPP KGGFSFDLCK RNEMLAKKGV NLPSSYRKTG TTIVGLIFQD
 51 GVILGADTRA TEGPIVCDKN CEKIHYMAPN IYCCGAGTAA DTEAVTDMVS
101 SQLQLHRYHT GRESRVITAL TLLKKHLFNY QGYVQAALVL GGVDCTGPHL
151 HTIYPHGSTD TLPFATMGSG SLAAMAIFES KYREGLNRDE GIKLVTEAIC
201 SGVFNDLGSG SNVDVCVITK GHKEYLRNHL LPNPRTYINP KGYVFSKKTE
251 VLLTKITPLK EPSKEKEGVS EGGEAMEE
```

Spot No.: **D62**

NCBI accession No.: **gi|114199044** Species: *Malus x domestica*

PFF score: [195]

Protein name: caffeic acid O-methyltransferase

Matched peptides No.: [4] Sequence coverage %: [12]

Matched sequences: R.TLPDGKVER.L;

R.TEKEFEALAK.G;
R.VLASYSILTYSLR.T;
K.AYGMTAFEYHGTDPR.F + Oxidation (M)

Calculated Mr: **40047** Calculated *p*I: **5.54**

Probability Based Mowse Score:

Ions score is -10*Log(P), where P is the probability that the observed match is a random event.
Individual ions scores > 45 indicate identity or extensive homology (p<0.05).
Protein scores are derived from ions scores as a non-probabilistic basis for ranking protein hits.

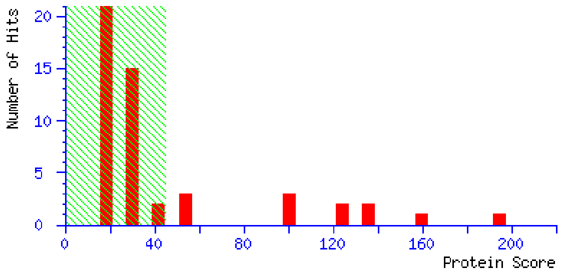

Matched peptide sequences: shown in Bold Red:

```
  1 MGSTPETQMT PTQVSDEEAN LFAMQLASAS VLPMVLKAAI ELDLLEIMAK
 51 AGPGAFVSPA DLSSQLPTKN PDAPVMLDRM LRVLASYSIL TYSLRTLPDG
101 KVERLYGLGP VCKFLTKSVD GASIGSLCLM NQDKVLMESW YNLKDAVLEG
151 GIPFNKAYGM TAFEYHGTDP RFNRVFNKGM ADHSTITMKK LLENYNGFEG
201 LTSIVDVGGG TGAVLNMIVS KYPSIKGINF DLPHVIEDAP QYPGVEHVGG
251 DMFVSVPKGD AIFMKWICHD WSDEHCLKFL KNCYAALPDN GKVIVAECIL
301 PVAPDSSLAT KGVVHIDAIM LAHNPGGKER TEKEFEALAK GSGFQGFRVV
351 CSAFNTYAIE FLKKN
```

Spot No.: **D63**

NCBI accession No.: **gi|255538084** Species: *Ricinus communis*

PFF score: [77]

Protein name: Adenine phosphoribosyltransferase, putative

Matched peptides No.: [1] Sequence coverage %: [7]

Matched sequences: R.ALVIDDLIATGGTLCAAIR.L

Calculated Mr: **27683** Calculated *p*I: **8.33**

Probability Based Mowse Score:

Ions score is -10*Log(P), where P is the probability that the observed match is a random event.
Individual ions scores > 46 indicate identity or extensive homology (p<0.05).
Protein scores are derived from ions scores as a non-probabilistic basis for ranking protein hits.

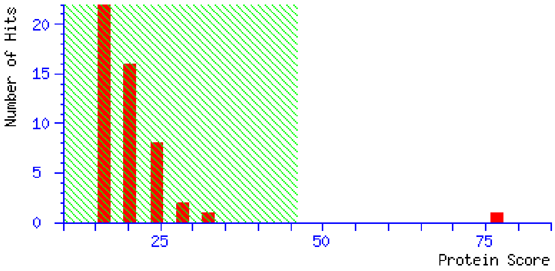

Matched peptide sequences: shown in Bold Red:

```
  1 MLKSIHLRAP NSILPSRQLS PPSPNKQLSA RIVIAAPSTG QQAPSISLRN
 51 YPPARPSLRC SAGGSDSQNQ KMAAQDDKDN RIERISSSIR VIPDFPKPGI
101 LFQDITTLLL DTKAFKDTID LFVERYKERD ISVVAGIEAR GFIFGPPIAL
151 AIGAKFVPMR KPNKLPGEVV SEEYSLEYGT DKMEMHVGAV QAGERALVID
201 DLIATGGTLC AAIRLLERVG VEVVECACVI ELPELKGRER LGQTPLFVLV
251 SSA
```

Spot No.: **D64**

NCBI accession No.: **gi|255546213** Species: *Ricinus communis*

PFF score: [94]

Protein name: o-methyltransferase, putative

Matched peptides No.: [1] Sequence coverage %: [7]

Matched sequences: R.VQISHASLGDGITICR.R

Calculated Mr: **25951** Calculated *p*I: **5.55**

Probability Based Mowse Score:

Ions score is -10*Log(P), where P is the probability that the observed match is a random event.
Individual ions scores > 46 indicate identity or extensive homology (p<0.05).
Protein scores are derived from ions scores as a non-probabilistic basis for ranking protein hits.

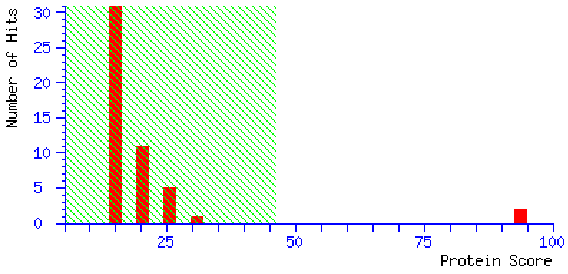

Matched peptide sequences: shown in Bold Red:

1 MQKGLLKSEE LYQYVLETSV FPREPEPLRE LRNITANHPW AKMATSPDAG
51 QFMAMLLQLV NAKKTIEVGV FTGYSLLLTA LSIPEDGKIV AIDVDREAYE
101 IGLPIIRKAG VEHKIDFMES EALPVLDKLL KEHGNESSFD FAFVDADKIN
151 YWNYHERLLK LVKVGGIVIY DNSLWRGSVA VPEEEAPEHL RFCRQLTIEH
201 NKFLAADSRV **QISHASLGDG ITICR**RIC

Spot No.: **D65 (S3)**

NCBI accession No.: **gi|132270**   Species: *Hevea brasiliensis*

Protein name：Rubber elongation factor protein; Short=REF;

AltName: Allergen=Hev b 1

Mascot score: **95**   Sequence coverage %: **56**

Matched peptides No.: **8**   Total peptides No.: **42**

Calculated Mr: **14713**   Calculated *p*I: **5.04**

Probability Based Mowse Score:

Protein score is -10*Log(P), where P is the probability that the observed match is a random event.
Protein scores greater than 73 are significant (p<0.05).

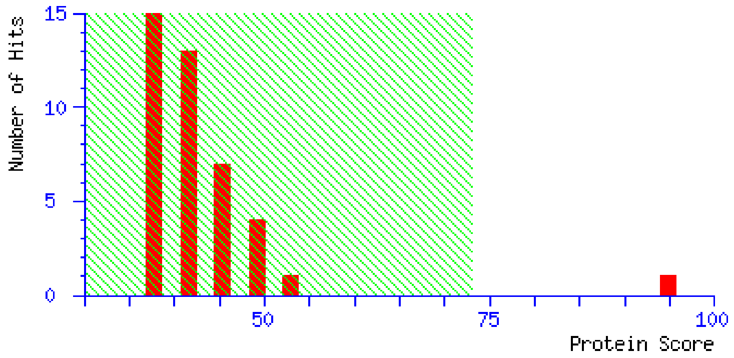

Matched peptide sequences: shown in Bold Red:

```
  1 MAEDEDNQQG QGEGLKYLGF VQDAATYAVT TFSNVYLFAK DKSGPLQPGV
 51 DIIEGPVKNV AVPLYNRFSY IPNGALKFVD STVVASVTII DRSLPPIVKD
101 ASIQVVSAIR AAPEAARSLA SSLPGQTKIL AKVFYGEN
```

Spot No.: **D66 (S5)**

NCBI accession No.: **gi|14423933** Species: *Hevea brasiliensis*

Protein name：Small rubber particle protein; Short=SRPP; AltName: Full=22 kDa rubber particle protein; Short=22 kDa RPP; AltName: Full=27 kDa natural rubber allergen; AltName: Full=Latex allergen Hev b 3

Mascot score: **122** Sequence coverage %: **85**

Matched peptides No.: **13** Total peptides No.: **55**

Calculated Mr: **22331** Calculated *p*I: **4.80**

Probability Based Mowse Score:

Protein score is -10*Log(P), where P is the probability that the observed match is a random event.
Protein scores greater than 73 are significant (p<0.05).

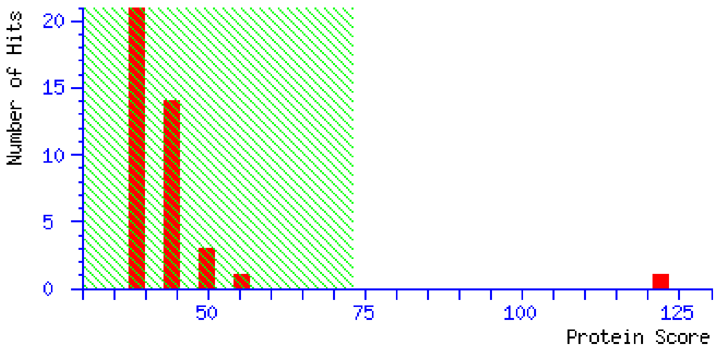

Matched peptide sequences: shown in Bold Red:

```
  1 MAEEVEEERL KYLDFVRAAG VYAVDSFSTL YLYAKDISGP LKPGVDTIEN
 51 VVKTVVTPVY YIPLEAVKFV DKTVDVSVTS LDGVVPPVIK QVSAQTYSVA
101 QDAPRIVLDV ASSVFNTGVQ EGAKALYANL EPKAEQYAVI TWRALNKLPL
151 VPQVANVVVP TAVYFSEKYN DVVRGTTEQG YRVSSYLPLL PTEKITKVFG
201 DEAS
```

Spot No.: **D67 (S6)**

NCBI accession No.: **gi|14423933** Species: *Hevea brasiliensis*

Protein name：Small rubber particle protein; Short=SRPP; AltName: Full=22 kDa rubber particle protein; Short=22 kDa RPP; AltName: Full=27 kDa natural rubber allergen; AltName: Full=Latex allergen Hev b 3

Mascot score: **135** Sequence coverage %: **75**

Matched peptides No.: **12** Total peptides No.: **31**

Calculated Mr: **22331** Calculated *p*I: **4.80**

Probability Based Mowse Score:

Protein score is -10*Log(P), where P is the probability that the observed match is a random event. Protein scores greater than 73 are significant (p<0.05).

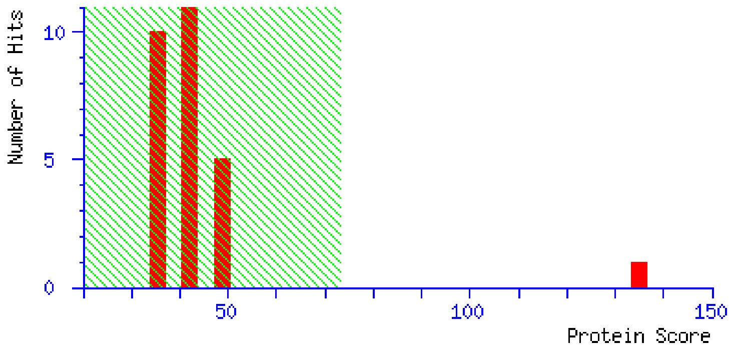

Matched peptide sequences: shown in Bold Red:

```
  1  MAEEVEEERL KYLDFVRAAG VYAVDSFSTL YLYAKDISGP LKPGVDTIEN
 51  VVKTVVTPVY YIPLEAVKFV DKTVDVSVTS LDGVVPPVIK QVSAQTYSVA
101  QDAPRIVLDV ASSVFNTGVQ EGAKALYANL EPKAEQYAVI TWRALNKLPL
151  VPQVANVVVP TAVYFSEKYN DVVRGTTEQG YRVSSYLPLL PTEKITKVFG
201  DEAS
```

Spot No.: **D68 (S9)**

NCBI accession No.: **gi|388503664** Species: *Lotus japonicus*

Protein name: **unknown**

Mascot score: **93** Sequence coverage %: **45**

Matched peptides No.: **13** Total peptides No.: **48**

Calculated Mr: **29537** Calculated *p*I: **4.79**

Probability Based Mowse Score:

Protein score is -10*Log(P), where P is the probability that the observed match is a random event.
Protein scores greater than 73 are significant (p<0.05).

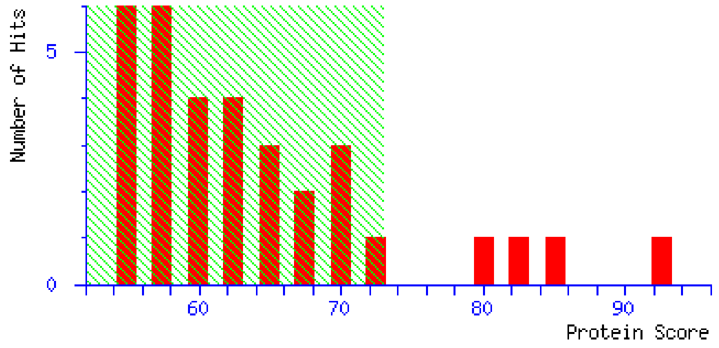

Matched peptide sequences: shown in Bold Red:

```
  1 MASSKDRGTF VYVAKLAEQA ERYEEMVDSM KKVANLDVEL TVEERNLLSV
 51 GYKNVIGARR ASWRILSSIE QKEETKGNDV NAKRIKEYRQ KVESELADIC
101 NDVMRVIDEH LIPSATAGES TVFYYKMKGD YYRYLAEFKS GNEKKEAADQ
151 SMKAYESATT AAEAELPPTH PIRLGLALNF SVFYYEILNS PERACHLAKQ
201 AFDEAISELD TLNEESYKDS TLIMQLLRDN LTLWTSDIPE EGEDSLKAND
251 TAKVGGGEDA E
```

Spot No.: Spot No.: **D69 (S13)**

NCBI accession No.: **gi|4235430** Species: *Hevea brasiliensis*

Protein name: **latex-abundant protein**

Mascot score: **96** Sequence coverage %: **54**

Matched peptides No.: **15** Total peptides No.: **37**

Calculated Mr: **46443** Calculated *p*I: **5.01**

Probability Based Mowse Score:

Protein score is -10*Log(P), where P is the probability that the observed match is a random event. Protein scores greater than 73 are significant (p<0.05).

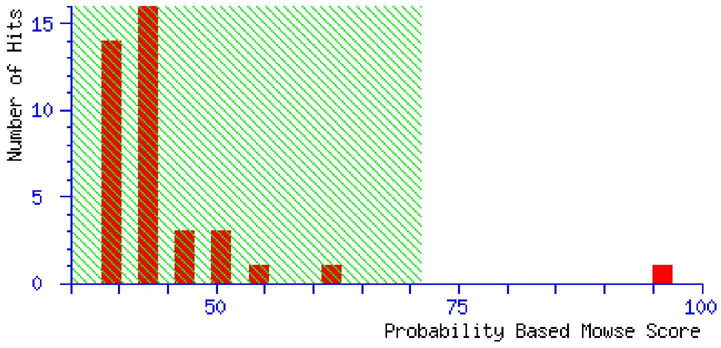

Matched peptide sequences: shown in Bold Red:

1 MAKKAVLIGI NYPGTKAELK GCINDVKRMY RCLVDR**YGFS** **EEDITVLIDI**

51 **DESYIQPTGK** NIR**RVLTDLV** **RSAEPGDLLF** **VHYSGHGTRL** **PAETGEDDDT**

101 **GFDECIVPCD** **MNLITDDDFR** **EFVDQVPHGC** **RITVVSDSCH** **SGGLIDEAK**E

151 QIGESTKRKE EESESGFGFK SFLK**QTIQDA** **FESRGVHLPS** **DLHHHHGHRD**

201 **EEDFDNRVVE** **EDYGDSGYVK** SK**SLPLSTLI** **EILK**QKTGKD DIDVGK**LRPT**

251 **LFDMFGDDAS** **PK**VKKFMKVI LNKLRHGDGE SGGGGFLGMV GSLAQEFLK**H**

301 **KLDENDESYV** **KPALETEVDS** **K**QEVYAGKTK RSLPDGGILI SGCQTDQTSA

351 DASPSGKSSE AYGALSNAIQ TIIAETDGAV TNQELVLKAR KMLK**KQGFTQ**

401 **KPGLYCSDDH** **VEASFVC**

Spot No.: **D70 (S20)**

NCBI accession No.: **gi|30575570** Species: *Citrus x paradisi*

PFF score: **[86]** Protein name: HSP19 class II, partial

Matched peptides No.: [1] Sequence coverage %: [15]

Matched sequences: K.VQVEDDNVLLISGER.K;

Calculated Mr: **11191** Calculated *p*I: **8.01**

Probability Based Mowse Score:

Ions score is -10*Log(P), where P is the probability that the observed match is a random event.
Individual ions scores > 44 indicate identity or extensive homology (p<0.05).
Protein scores are derived from ions scores as a non-probabilistic basis for ranking protein hits.

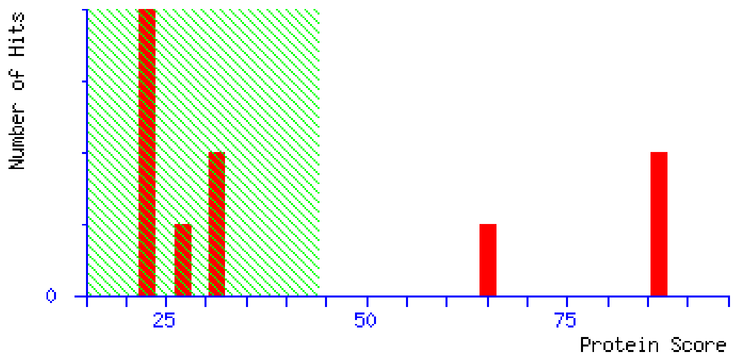

Matched peptide sequences: shown in Bold Red:

```
 1  YVFIVDMPGL KSGDIKVQVE DDNVLLISGE RKREEEKDGA KYVRMERRVG
51  KFMRKFVLPE NANVEAISAV CQDGVLTVTV DKLPPPEPKK PKTIEVKIA
```

Spot No.: **D71 (S22)**

NCBI accession No.: **gi|37806305**

Species: *Oryza sativa Japonica Group*

PFF score: **[60]** Protein name： hypothetical protein

Matched peptides No.: [2] Sequence coverage %: [3]

Matched sequences: K.LGTLVLGDRPR.L ; K.LGTLVLGDRPR.L

Calculated Mr: **35105** Calculated *p*I: **8.29**

Probability Based Mowse Score:

Ions score is -10*Log(P), where P is the probability that the observed match is a random event.
Individual ions scores > 45 indicate identity or extensive homology (p<0.05).
Protein scores are derived from ions scores as a non-probabilistic basis for ranking protein hits.

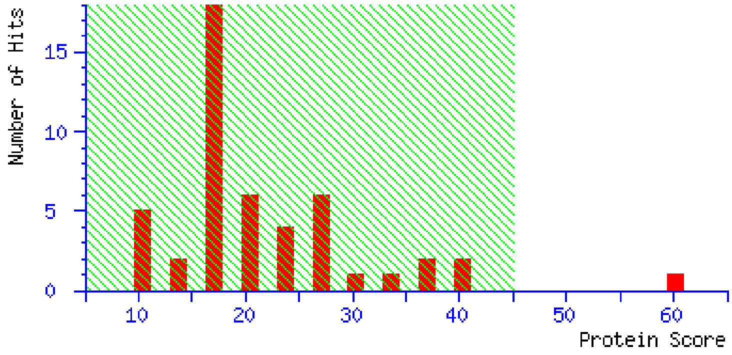

Matched peptide sequences: shown in Bold Red：

```
  1 MEVFQGVQFA RLRNWWEETY VTADEDGRSV YHYAPDRHRP AHEAIWAVQM
 51 VLAGAPPTQY VLLRGAYGRY LGAPDAVVRR WPLSCCWPAP VVGQRDFDQP
101 EVDAIMWRAV RRADHVLCLH DKSGRYLRGK LGTLVLGDRP RLTVGDGRLN
151 DDEKALRWEV LPVLPNPGRP ELPISIVPEA DLVGRLVKAC FLPLQREIQF
201 VEADDDGNIG EGQEVWDSFQ YEGRSVQLLR NELEDRVGYA ITVCVRAGRH
251 GQHSPLLINL PHSRETLHIV VLRRNSEADN QLTFPDPKAS SRRRYRHRRR
301 AIIQ
```

Spot No.: **D72 (S26)**

NCBI accession No.: **gi|3451147** Species: *Hevea brasiliensis*

PFF score: [56] Protein name: chitinase

Matched peptides No.: [1] Sequence coverage %: [4]

Matched sequences: K.VYLTAAPQCPFPDR.Y

Calculated Mr: **34138** Calculated *p*I: **8.72**

Probability Based Mowse Score:

Ions score is -10*Log(P), where P is the probability that the observed match is a random event.
Individual ions scores > 44 indicate identity or extensive homology (p<0.05).
Protein scores are derived from ions scores as a non-probabilistic basis for ranking protein hits.

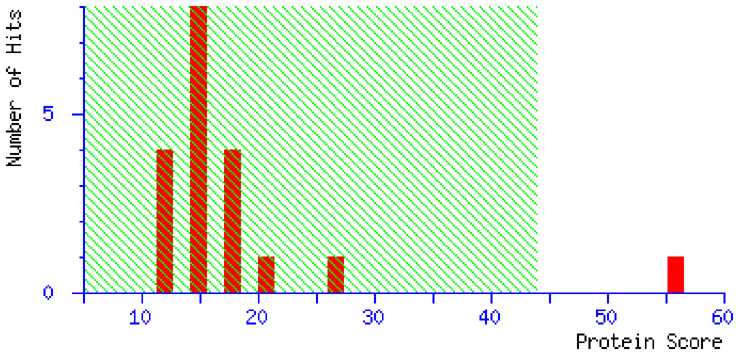

Matched peptide sequences: shown in Bold Red:

```
  1 MAKRTQAILL LLLAISLIMS SSHVDGGGIA IYWGQNGNEG TLTQTCSTRK
 51 YSYVNIAFLN KFGNGQTPQI NLAGHCNPAA GGCTIVSNGI RSCQIQGIKV
101 MLSLGGGIGS YTLASQADAK NVADYLWNNF LGGKSSSRPL GDAVLDGIDF
151 DIEHGSTLYW DDLARYLSAY SKQGKKVYLT AAPQCPFPDR YLGTALNTGL
201 FDYVWVQFYN NPPCQYSSGN INNIINSWNR WTTSINAGKI FLGLPAAPEA
251 AGSGYVPPDV LISRILPEIK KSPKYGGVML WSKFYDDKNG YSSSIRDSVL
301 FLHSEKCMTV L
```

Spot No.: **D73 (S30)**

NCBI accession No.: **gi|255540341** Species: *Ricinus communis*

Protein name: **glyceraldehyde 3-phosphate dehydrogenase, putative**

Mascot score: **86** Sequence coverage %:**42**

Matched peptides No.: **14** Total peptides No.: **57**

Calculated Mr: **36930** Calculated *p*I: **7.10**

Probability Based Mowse Score:

Protein score is -10*Log(P), where P is the probability that the observed match is a random event.
Protein scores greater than 73 are significant (p<0.05).

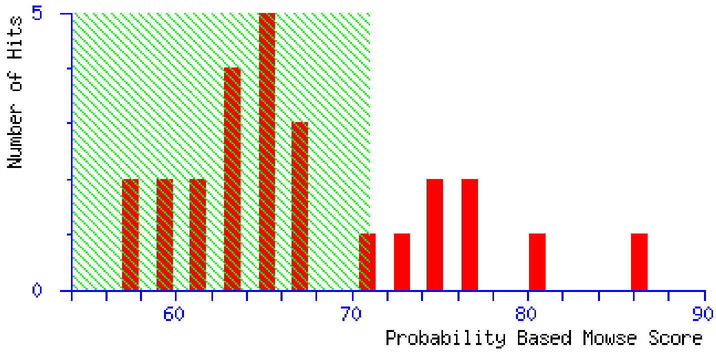

Matched peptide sequences: shown in **Bold Red:**

1	MAKIK**IGING**	**FGR**IGRLVAR	**VALQR**DDVEL	VAVNDPFIST	EYMTYMFK**YD**
51	**TVHGQWK**HHE	LKIKDDKTLL	FGEKPVTVFG	VR**NPEEIPWA**	**ETGAEYIVES**
101	**TGVFTDKDK**A	AAHLKGGAKK	VIISAPSK**DA**	**PMFVVGVNEK**	EYTPDLHIIS
151	NASCTTNCLA	PLAKVINDRF	GIVEGLMTTV	HSITATQK**TV**	**DGPSMK**DWRG
201	GR**AASFNIIP**	**SSTGAAKAVG**	**K**VLPSLNGK**L**	**TGMSFRVPTV**	**DVSVVDLTVR**
251	LEKSATYEQI	KAAIK**EESEG**	**KLK**GILGYTE	DDVVSTDFIG	DKRSSIFDAK
301	**AGIALNDKFV**	**KLVSWYDNEW**	**GYSTR**VVDLI	VHVASVH	

Spot No.: **D74 (S35)**

NCBI accession No.: **gi|3288200**   Species: *Hevea brasiliensis*

PFF score: [81]

Protein name: **latex allergen**

Matched peptides No.: [2]   Sequence coverage %: [4]

Matched sequences: R.DTYDPIHSIGPIYDGEYLR.E;

R.DTYDPIHSIGPIYDGEYLR.E

Calculated Mr: **43020**   Calculated *p*I: **5.00**

Probability Based Mowse Score:

Ions score is -10*Log(P), where P is the probability that the observed match is a random event.
Individual ions scores > 43 indicate identity or extensive homology (p<0.05).
Protein scores are derived from ions scores as a non-probabilistic basis for ranking protein hits.

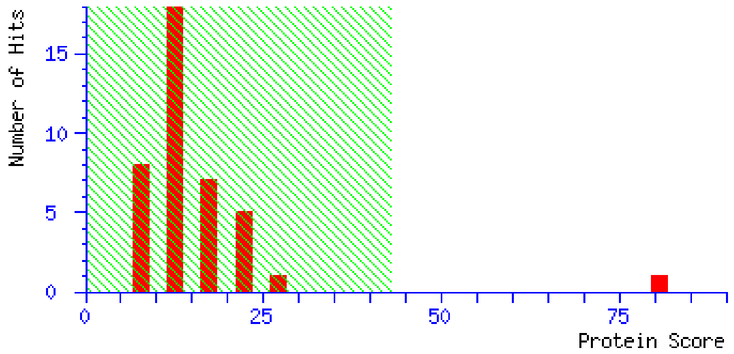

Matched peptide sequences: shown in **Bold Red:**

```
  1  MATGSTTLTQ  GKKITVLSID  GGGIRGIIPG  IILASLESKL  QDLDGPDARI
 51  ADYFDIIAGT  STGGLITTML  TAPNEDKKPI  YQAKDIKDFY  LENCPKIFPK
101  ESRDTYDPIH  SIGPIYDGEY  LRELCNNLLK  DLTVKDTLTD  VIIPAFDIKL
151  LLPVIFSSDD  AKCNALKNAR  LADVCISTSA  APVLLPAHSF  TTEDDKNIHT
201  FELIDGGVAA  ANPTLLALTH  IRNEIIRQNP  RFIGANLTES  KSRLVLSLGT
251  GKSEYKEKYN  ADMTSKWRLY  NWALYNGNSP  AVDIFSNASS  DMVDFHLSAL
301  FKSLDCEDYY  LRIQDDTLTG  EESSGHIATE  ENLQRLVEIG  TELLEKQESR
351  INLDTGRLES  IPGAPTNEAA  IAKFAKLLSE  ERKLRQLK
```

Spot No.: **D75 (S36)**

NCBI accession No.: **gi|1916805** Species: *Hevea brasiliensis*

PFF score: [138]

Protein name： **latex patatin homolog**

Matched peptides No.: [2] Sequence coverage %: [8]

Matched sequences: K.SLDCEDYYLR.I；

R.IQDDTLTGEESSGHIATEENLQR.L

Calculated Mr: **43035** Calculated *p*I: **5.00**

Probability Based Mowse Score:

Ions score is -10*Log(P), where P is the probability that the observed match is a random event.
Individual ions scores > 44 indicate identity or extensive homology (p<0.05).
Protein scores are derived from ions scores as a non-probabilistic basis for ranking protein hits.

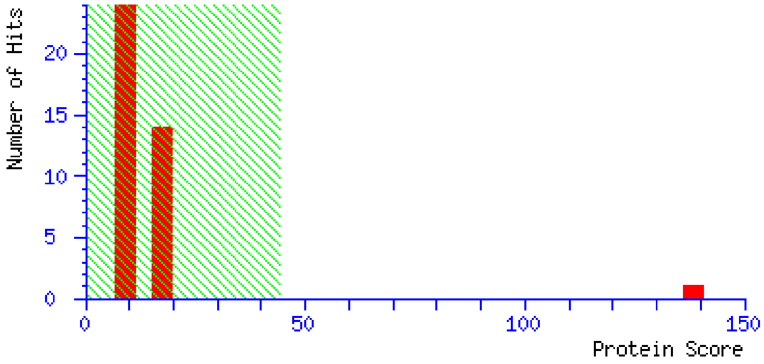

Matched peptide sequences: shown in **Bold Red:**

```
  1 MATGSTTLTQ GKKITVLSID GGGIRGIIPG IILASLESKL QDLDGPDARI
 51 ADYFDIIAGT STGGLITTML TAPNEDKKPM YQAKDIKDFY LENCPKIFPK
101 ESRDNYDPIH SIGPIYDGEY LRELCNNLLK DLTVKDTSTD VIIPTFDIKL
151 LLPVIFPSDD AKCNALKNAR LADVCISTSA APVLLPAHSF TTEDDKNIHT
201 FELIDGGVAA TNPTLLALTH IRNEIIRQNP RFIGANLTES KSRLVLSLGT
251 GKSEYKEKYN ADMTSKWRLY NWALYNGNSP AVDIFSNASS DMVDSHLSAL
301 FKSLDCEDYY LRIQDDTLTG EESSGHIATE ENLQRLVEIG TELLEKQESR
351 INLDTGRLES IPGAPTNEAA IAKFAKLLSE ERKLRQLK
```

Spot No.: **D76 (S37)**

NCBI accession No.: **gi|41581137** Species: *Hevea brasiliensis*

Protein name: **putative latex allergen hev b 7.02**

PMF score: **152** Sequence coverage %: **44**

Matched peptides No.: **16** Total peptides No.: **39**

Calculated Mr: **43027** Calculated *p*I: **5.12**

Probability Based Mowse Score:

Protein score is -10*Log(P), where P is the probability that the observed match is a random event. Protein scores greater than 73 are significant (p<0.05).

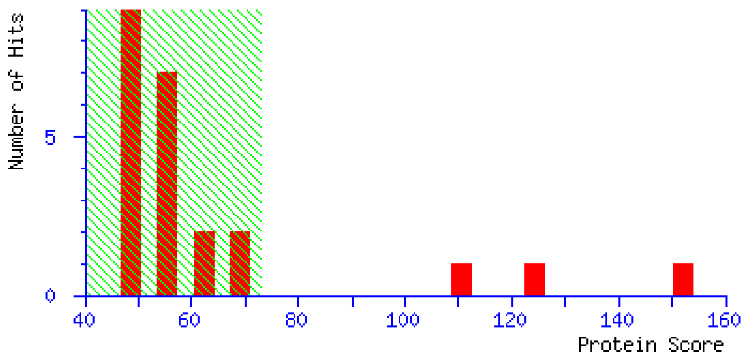

Matched peptide sequences: shown in Bold Red

```
  1 ATGSTTLTQG KKITVLSIDG GGIRGIIPGI ILASLESKLQ DLDGPDARIA
 51 DYFDIIAGTS TGGLITTMLT APNEDKKPMY QAKDIKDFYL ENCPKIFPKE
101 SRDNYDPIHS IGPIYDGEYL RELCNNLLKD LTVKDTLTDV IIPTFDIKLL
151 LPVIFSSDDA KCNALKNARL ADVCISTSAA PVLLPAHSFT TEDDKNIHTF
201 ELIDGGVAAT NPTLLALTHI RNEIIRQNPR FIGANLTESK SRLVLSLGTG
251 KSEYKEKYNA DMTSKWRLYN WALYNGNSPA VDIFSNASSD MVDFHLSALF
301 KSLDCEDYYL RIQDDTLTGE ESSGHIATEE NLQRLVEIGT RLLEKQESRI
351 NLDTGRLESI PGASTNEAAI TKFAKLLSEE RKLRQLK
```

Spot No.: **D77 (S38)**

NCBI accession No.: **gi|41581137** Species: *Hevea brasiliensis*

Protein name: **putative latex allergen hev b 7.02**

PMF score: **142** Sequence coverage %: **42**

Matched peptides No.: **15** Total peptides No.: **51**

Calculated Mr: **43027** Calculated *p*I: **5.12**

Probability Based Mowse Score:

Protein score is -10*Log(P), where P is the probability that the observed match is a random event.
Protein scores greater than 73 are significant (p<0.05).

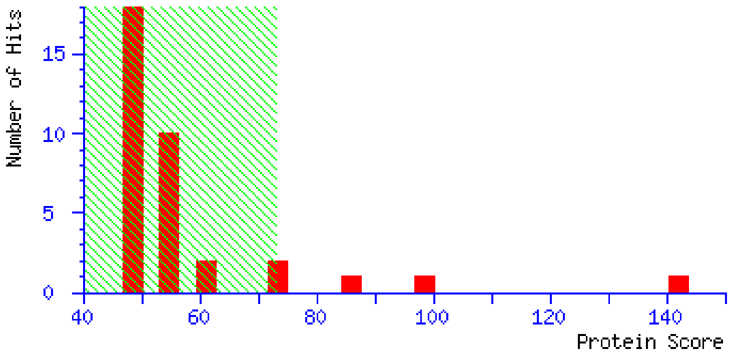

Matched peptide sequences: shown in Bold Red

1	ATGSTTLTQG	KKITVLSIDG	GGIRGIIPGI	ILASLESKLQ	DLDGPDARIA
51	DYFDIIAGTS	TGGLITTMLT	APNEDKKPMY	QAKDIKDFYL	ENCPKIFPKE
101	SRDNYDPIHS	IGPIYDGEYL	RELCNNLLKD	LTVKDTLTDV	IIPTFDIKLL
151	LPVIFSSDDA	KCNALKNARL	ADVCISTSAA	PVLLPAHSFT	TEDDKNIHTF
201	ELIDGGVAAT	NPTLLALTHI	RNEIIRQNPR	FIGANLTESK	SRLVLSLGTG
251	KSEYKEKYNA	DMTSKWRLYN	WALYNGNSPA	VDIFSNASSD	MVDFHLSALF
301	KSLDCEDYYL	RIQDDTLTGE	ESSGHIATEE	NLQRLVEIGT	RLLEKQESRI
351	NLDTGRLESI	PGASTNEAAI	TKFAKLLSEE	RKLRQLK	

Spot No.: **D78 (S46)**

NCBI accession No.: gi|4469175 Species: *Hevea brasiliensis*

Protein name： beta-1,3-glucanase

PMF score: **126** Sequence coverage %: **55**

Matched peptides No.: **19** Total peptides No.: **37**

Calculated Mr: **38930** Calculated *p*I: **9.45**

Probability Based Mowse Score:

Protein score is -10*Log(P), where P is the probability that the observed match is a random event.
Protein scores greater than 73 are significant (p<0.05).

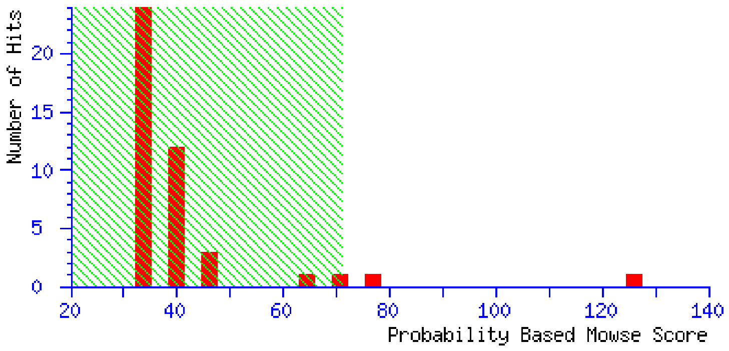

Matched peptide sequences: shown in Bold Red

```
  1  MAISSSTSGT SSSLPSRTTV MLLLFFFAAS VGITDAQVGV CYGMQGNNLP
 51  PVSEVIALYK KSNITRMRIY DPNRAVLEAL RGSNIELILG VPNSDLQSLT
101  NPSNAKSWVQ KNVRGFWSSV LFRYIAVGNE ISPVNRGTAW LAQFVLPAMR
151  NIHDAIRSAG LQDQIKVSTA IDLTLVGNSY PPSAGAFRDD VRSYLNPIIR
201  FLSSIRSPLL ANIYPYFTYA GNPRDISLPY ALFTSPSVVV WDGQRGYKNL
251  FDATLDVLYS ALERASGGSL EVVVSESGWP SAGAFAATFD NGRTYLSNLI
301  QHVKRGTPKR PNRAIETYLF AMFDENKKQP EVEKQFGLFF PDKWQKYNLN
351  FG
```

Spot No.: **D79 (S54)**

NCBI accession No.: **gi|34538473**   Species: *Hordeum vulgare*

Protein name: **caleosin 1**

PMF score: **84**   Sequence coverage %: **19**

Matched peptides No.: **10**   Total peptides No.: **37**

Calculated Mr: **34330**   Calculated *p*I: **6.11**

Probability Based Mowse Score:

Protein score is -10*Log(P), where P is the probability that the observed match is a random event.
Protein scores greater than 73 are significant ($p<0.05$).

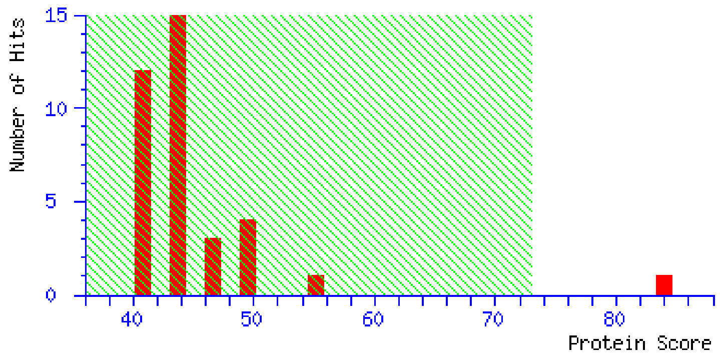

Matched peptide sequences: shown in Bold Red

```
  1 MATKARKVEV RDASRAEGKG DAADVHVLRE AMRADGKGDH DTAGGANRAD
 51 GHGDAGGRVG DSRGVDGKDS LKMVALQAPV TVERPVRGDL EEHVPKPYLA
101 RALAAPDMYH PEGTTTDDHQ HHNMSVLQQH VAFFDRDNNG IIYPWETYDG
151 CRAVGFNVFM SAFIAFLVNL VMSYPTLPGW LPNPLFPIYV HNIHKSKHGS
201 DSGTYDKEGR FMPVNFENIF SKYARTYPDR LSYREMWRMT EGCREVFDFF
251 GWVAMKLEWS ILYALARDDE GYLSREAIRR MYDGSLFEYM ERQRMEHVKM
301 S
```

Spot No.: **D80 (S61)**

NCBI accession No.: **gi|255560725** Species: *Ricinus communis*

Protein name： dead box ATP-dependent RNA helicase, putative

Mascot score: **144** Sequence coverage %: **68**

Matched peptides No.: **25** Total peptides No.: **91**

Calculated Mr: **47097** Calculated *p*I: **5.49**

Probability Based Mowse Score:

Protein score is -10*Log(P), where P is the probability that the observed match is a random event.
Protein scores greater than 73 are significant ($p<0.05$).

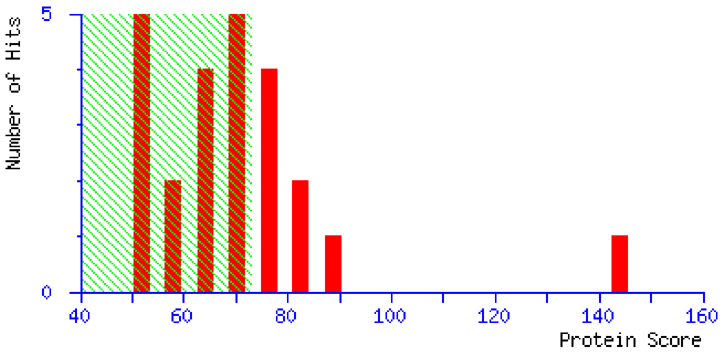

Matched peptide sequences: shown in Bold Red:

```
  1 MAGLAPEGSQ FDAKQYDAKM SELLSTEGQE FFTTYDEVYD SFDAMGLQEN
 51 LLRGIYAYGF EKPSAIQQRG IVPFCKGLDV IQQAQSGTGK TATFCSGILQ
101 QLDYGLVQCQ ALVLAPTREL AQQIEKVMRA LGDYLGVKVH ACVGGTSVRE
151 DQRILQAGVH VVVGTPGRVF DMLRRQSLRP DYIKMFVLDE ADEMLSRGFK
201 DQIYDIFQLL PAKVQVGVFS ATMPPEALEI TRKFMNKPVR ILVKRDELTL
251 EGIKQFYVNV EKEEWKLETL CDLYETLAIT QSVIFVNTRR KVDWLTDKMR
301 SRDHTVSATH GDMDQNTRDI IMREFRSGSS RVLITTDLLA RGIDVQQVSL
351 VINYDLPTQP ENYLHRIGRG GRFGRKGVAI NFVTRDDERM LFDIQKFYNV
401 VIEELPSNVA DLL
```

Spot No.: **D81 (S63)**

NCBI accession No.: **gi|255578278**   Species: *Ricinus communis*

PFF score: [76]

Protein name:hypothetical protein RCOM_0537780

Matched peptides No.: [3]   Sequence coverage %: [6]

Matched sequences: K.QESEYGSGGYGR.R;
R.KPSYGEEGGYGER.T ;
R.KPSYGEEGGYGER.T

Calculated Mr: **41389**   Calculated *p*I: **4.65**

Probability Based Mowse Score:

Mascot Score Histogram

Ions score is -10*Log(P), where P is the probability that the observed match is a random event.
Individual ions scores > 44 indicate identity or extensive homology (p<0.05).
Protein scores are derived from ions scores as a non-probabilistic basis for ranking protein hits.

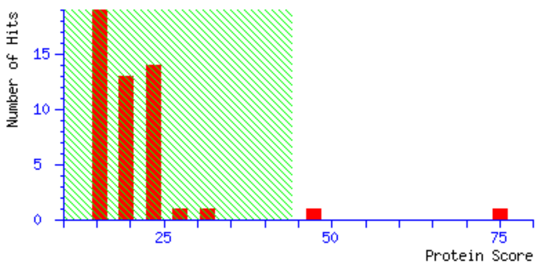

Matched peptide sequences: shown in Bold Red:

```
  1 MPYYTRSEDD VNDFDEFDPT PYGGGYDLAL TYGRPLPPSD ETCYQNSAIA
 51 DDDVDYDRPN FTSYAEPSAY NDDILQEEYN SYSRPKPRPG FIPGGGIGGD
101 PYSRPHAAPG FQPGSGGYGG VSEYEKPSGY GRRPDSEYGS GGYGGGTEYE
151 RPSGEDYGSG HGRKQDSEYG SGGYGRRPES EYGSGGGYER PSSEEYGSGY
201 GRKQESEYGS GGYGRRPEAE YGSGYGGRPE TEYGGGGGSE YGGGYGRKPS
251 YGEEGGYGER TEYERPSYGD DPPRRPGYGR QDDEYERPSY ERKSDDDNDG
301 SRRKYGGDDD EKGYQKKYGG YGDDDDEEGS RRKYGYGGEE ESYGRKKYGD
351 DGSDDDEKKH HRHKHHQHKH SDDE
```

Bold red (matched): 204–215 QESEYGSGGYGR; 248–260 KPSYGEEGGYGER

Spot No.: **D82 (S69)**

NCBI accession No.: **gi|2501850** Species: *Nicotiana tabacum*

Protein name: GDP dissociation inhibitor

PMF score: **83** Sequence coverage %: **34**

Matched peptides No.: **16** Total peptides No.: **62**

Calculated Mr: **50127** Calculated *p*I: **5.44**

Probability Based Mowse Score:

Protein score is -10*Log(P), where P is the probability that the observed match is a random event.
Protein scores greater than 73 are significant (p<0.05).

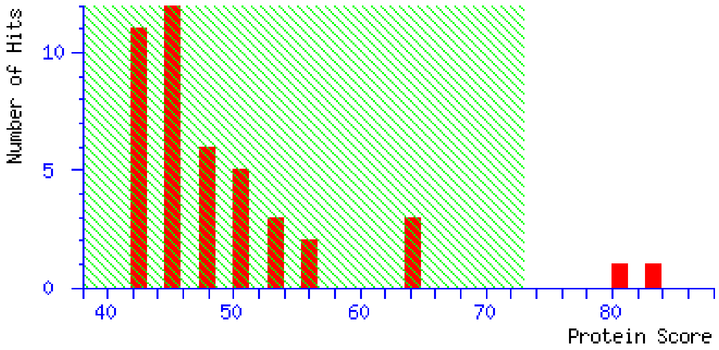

Matched peptide sequences: shown in Bold Red:

1	MDEEYDVIVL	GTGLKECILS	GLLSVDGLKV	LHMDRNDYYG	GESTSLNLVQ
51	LWKRFRGSDK	PPAELGSSRD	FNVDMIPKFI	MANGALVRVL	IHTDVTKYLY
101	FKAVDGSFVY	NKGKVHKVPA	TDMEALKSPL	MGIFEKRRAR	KFFIYVQDYN
151	ESDPKTHEGM	DLTRVTTREL	IAKYGLDDNT	VDFIGHALAL	HRDDRYLDEP
201	AQDTVKRMKL	YAESLARFQG	GSPYIYPLYG	LGELPQAFAR	LSAVYGGTYM
251	LNKPECKVEF	DEEGKVCGVT	SEGETAKCKK	VVCDPSYLPN	KVRKVGKVAR
301	AIAIMSHPIP	NTNDSHSVQI	ILPQKQLGRK	SDMYLFCCSY	THNVAPKGKF
351	IAFVSTEAET	DNPESELKQG	VNLLGPVDEI	FYETYDRSEP	VNEPSLDNCF
401	ISTSYDATTH	FESTVDDVLN	LYTKITGKVL	DLNVDLSAAS	AAEE

Spot No.: **D83 (S75)**

NCBI accession No.: **gi|3264769** Species: *Prunus armeniaca*

PFF score: [74] Protein name: late embryogenesis-like protein

Matched peptides No.: [1] Sequence coverage %: [8]

Matched sequences: K.LVSGLIPDAGTIHAHGEETVK.V

Calculated Mr: **28937** Calculated *p*I: **4.72**

Probability Based Mowse Score:

Ions score is -10*Log(P), where P is the probability that the observed match is a random event.
Individual ions scores > 45 indicate identity or extensive homology (p<0.05).
Protein scores are derived from ions scores as a non-probabilistic basis for ranking protein hits.

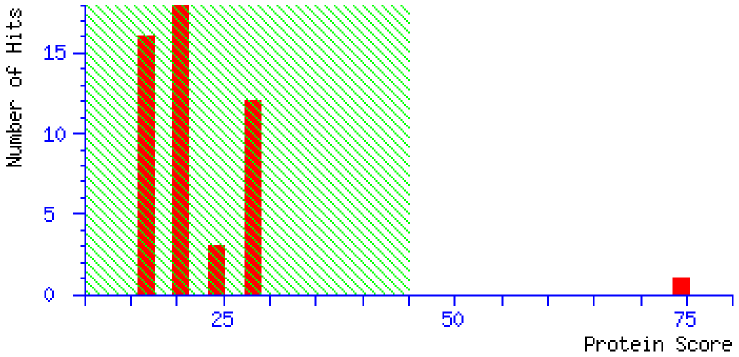

Matched peptide sequences: shown in Bold Red:

```
  1 DVTAIHIPSI NLEKAEIVVD VLIKNPNPVP IPLIDINYLI ESDGRKLVSG
 51 LIPDAGTIHA HGEETVKVPV HLVYDDIKNT YDDIKPGSII PYRFKVDLIV
101 DVPVLGRLTL PLEKTGEIPI PYKPDVDVEK IKFQAFSFEE TVAVLHVKLE
151 NMNDFDLGLN ALDYEIWLSE VNIGGAQLSQ SANLAKKGVT FIELPITFRP
201 KDFGSALWDM IRGKGTGYTI KGNINVDTPF GAMKLPIVKE GGTTRLRKNK
251 EDGGDDDDED ED
```

Spot No.: **D84 (S85)**

NCBI accession No.: **gi|224099853** Species: ***Populus trichocarpa***

PFF score: [69] Protein name: **putative protein**

Matched peptides No.: [2] Sequence coverage %: [5]

Matched sequences: S FEHPLNELKP VPR

Calculated Mr: **27448** Calculated *p*I: **5.84**

Probability Based Mowse Score:

Ions score is -10*Log(P), where P is the probability that the observed match is a random event.
Individual ions scores > 45 indicate identity or extensive homology (p<0.05).
Protein scores are derived from ions scores as a non-probabilistic basis for ranking protein hits.

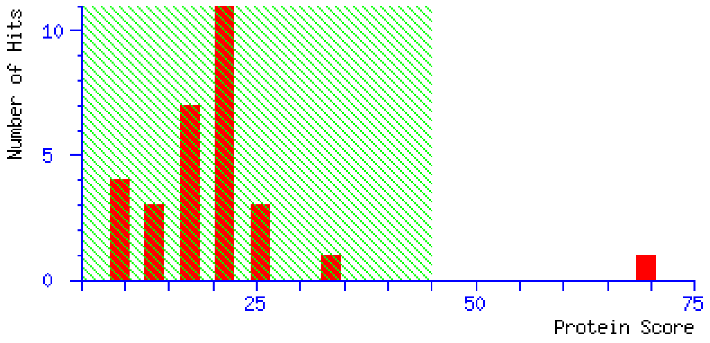

Matched peptide sequences: shown in Bold Red

```
  1 MLAVFDNTVA KCPDALQSPH SAPASSALKD GFLANHFASQ HPGSVTVNLG
 51 TSGLISHSVE KQNPFLPRLF AVVDDIFCLF QGHIDNVAVL KQQYGLNKTA
101 NEVIVVIEAY RTLRDRGPYP ADQVVKDIQG KFAFILYDST SKATFFAADA
151 DGSVPFFWGA DSEGNLVLSD DVQIVQKGCG KSFAPFPKGC FFTTSGGLRS
201 FEHPLNELKP VPRVDSSGQV CGATFKVDAE TKKESVGMPR VDSSYNWSSN
251 Y
```

Spot No.: **D85 (S86)**

NCBI accession No.: **gi|38122474** Species: *Hevea brasiliensis*

PFF score: [410] Protein name： rubber elongation factor

Matched peptides No.: [4] Sequence coverage %: [26]

Matched sequences: YLDFVQAATVYAR ;
SGPFKPGVNTVESR;
SVVRPVYNK ;
RVDAYVTVLDR

Calculated Mr: **19570** Calculated *p*I: **5.06**

Probability Based Mowse Score:

Ions score is -10*Log(P), where P is the probability that the observed match is a random event.
Individual ions scores > 45 indicate identity or extensive homology (p<0.05).
Protein scores are derived from ions scores as a non-probabilistic basis for ranking protein hits.

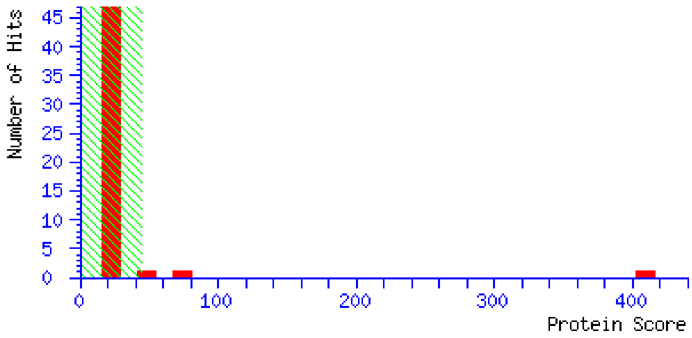

Matched peptide sequences: shown in **Bold Red**

```
  1  MAEGEEEVNI  QEEANKGEEN  PQEEANIQEE  TNKGEENIQE  EANIEEEANK
 51  EEESLKYLDF  VQAATVYARA  SFSKLYLFAK  DKSGPFKPGV  NTVESRFKSV
101  VRPVYNKFQP  VPNKVLKFAD  RRVDAYVTVL  DRIVPPIVKR  ASIQAYSVAP
151  GAALAVASYL  PLHTKRLSKV  LYGDG
```

Spot No.: **D86 (S87)**

NCBI accession No.: **gi|38122474** Species: *Hevea brasiliensis*

PFF score: [226] Protein name： rubber elongation factor

Matched peptides No.: [4] Sequence coverage %: [26]

Matched sequences: YLDFVQAATVYAR ;

SGPFKPGVNTVESR;

SVVRPVYNK ;

RVDAYVTVLDR

Calculated Mr: **19570** Calculated *p*I: **5.06**

Probability Based Mowse Score:

Ions score is -10*Log(P), where P is the probability that the observed match is a random event.
Individual ions scores > 46 indicate identity or extensive homology (p<0.05).
Protein scores are derived from ions scores as a non-probabilistic basis for ranking protein hits.

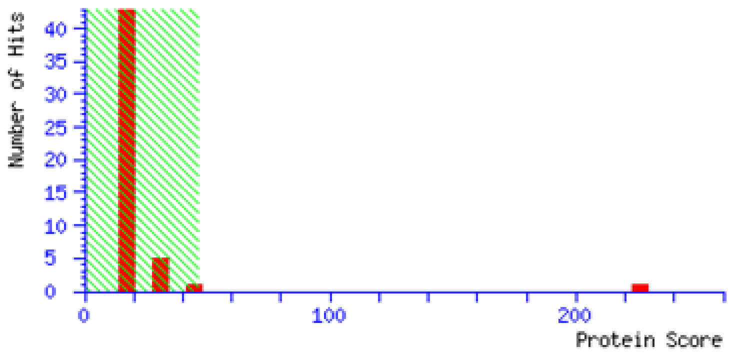

Matched peptide sequences: shown in Bold Red

```
  1  MAEGEEEVNI QEEANKGEEN PQEEANIQEE TNKGEENIQE EANIEEEANK
 51  EEESLKYLDF VQAATVYARA SFSKLYLFAK DKSGPFKPGV NTVESRFKSV
101  VRPVYNKFQP VPNKVLKFAD RRVDAYVTVL DRIVPPIVKR ASIQAYSVAP
151  GAALAVASYL PLHTKRLSKV LYGDG
```

Spot No.: **D87 (S90)**

NCBI accession No.: **gi|334854634** Species: *Hevea brasiliensis*

PFF score: [490] Protein name： elicitor-responsive protein

Matched peptides No.: [3] Sequence coverage %: [59]

Matched sequences: PLGTVEVLLVGAKGLENTDFLNGVDPYVVLACR;
GSEP EWNEKFSFEVSDGDTELTLK;
GEITVGLTFTPEVEMDNVGVDGYDFRL

Calculated Mr: **15249** Calculated *p*I: **4.06**

Probability Based Mowse Score:

Ions score is -10*Log(P), where P is the probability that the observed match is a random event.
Individual ions scores > 45 indicate identity or extensive homology (p<0.05).
Protein scores are derived from ions scores as a non-probabilistic basis for ranking protein hits.

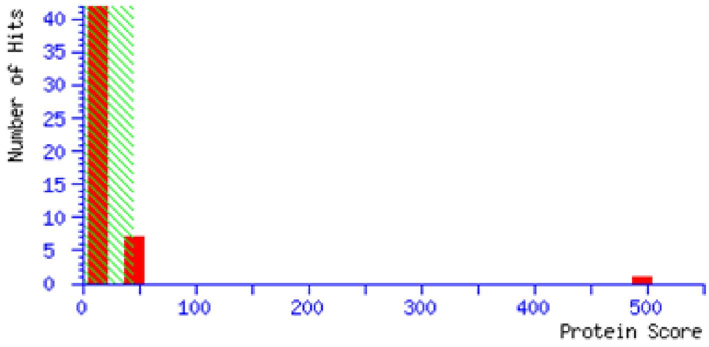

Matched peptide sequences: shown in Bold Red

```
  1 MPLGTVEVLL VGAKGLENTD FLNGVDPYVV LACRTQEQKS SVASGKGSEP
 51 EWNEKFSFEV SDGDTELTLK IMDSDVGAAD DFVGEATIPL EPLFLEGNLP
101 STAYKVVKEQ EYKGEITVGL TFTPEVEMDN VGVDGYDFRL
```

Spot No.: **D88 (S92)**

NCBI accession No.: **gi|224144943** Species: *Populus trichocarpa*

PFF score: [180] Protein name: predicted protein

Matched peptides No.: [2] Sequence coverage %: [28]

Matched sequences: VFEDNVLIRVLVVDGGGSLR;

HVPITIG GTRISDGEWLYADTDGILISR

Calculated Mr: **17971** Calculated *p*I: **5.39**

Probability Based Mowse Score:

Ions score is -10*Log(P), where P is the probability that the observed match is a random event.
Individual ions scores > 44 indicate identity or extensive homology (p<0.05).
Protein scores are derived from ions scores as a non-probabilistic basis for ranking protein hits.

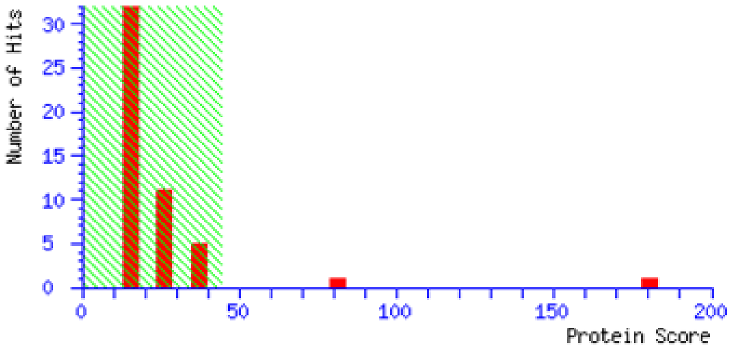

Matched peptide sequences: shown in Bold Red

```
  1  MALVTTAEVC  DANPQLIVSG  ELRALQPIFQ  IYGRRQVFSG  PVVTLKVFED
 51  NVLIREFLEE  KGNGRVLVVD  GGGSLRCAIL  GGNPVVQAQN  NGWAGIVVNG
101  CIRDVDEIMG  CDIGVRALAS  HPMKANKKGI  GEKHVPITIG  GTRISDGEWL
151  YADTDGILIS  RTELAV
```

Spot No.: **D89 (S94)**

NCBI accession No.: gi|123062 Species: *Hevea brasiliensis*

PFF score: [96]

Protein name: prohevein

Matched peptides No.: [1] Sequence coverage %: [8]

Matched sequences: IVDQ CSNGGLDLDV NVFR

Calculated Mr: 22700 Calculated *p*I: 5.63

Probability Based Mowse Score:

Ions score is -10*Log(P), where P is the probability that the observed match is a random event.
Individual ions scores > 44 indicate identity or extensive homology (p<0.05).
Protein scores are derived from ions scores as a non-probabilistic basis for ranking protein hits.

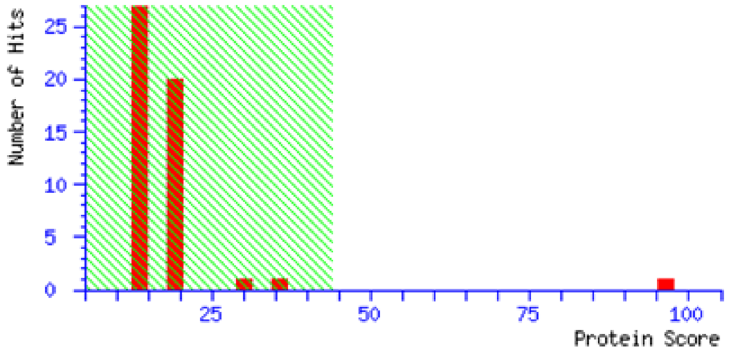

Matched peptide sequences: shown in Bold Red

```
  1 MNIFIVVLLC LTGVAIAEQC GRQAGGKLCP NNLCCSQWGW CGSTDEYCSP
 51 DHNCQSNCKD SGEGVGGGSA SNVLATYHLY NSQDHGWDLN AASAYCSTWD
101 ANKPYSWRSK YGWTAFCGPV GAHGQSSCGK CLSVTNTGTG AKTTVRIVDQ
151 CSNGGLDLDV NVFRQLDTDG KGYERGHITV NYQFVDCGDS FNPLFSVMKS
201 SVIN
```

Spot No.: **D90 (S98)**

NCBI accession No.: gi|38122474 Species: *Hevea brasiliensis*

Protein name: rubber elongation factor

PMF score: 99 Sequence coverage %: 45

Matched peptides No.: 8 Total peptides No.: 39

Calculated Mr: 19570 Calculated *p*I: 5.06

Probability Based Mowse Score:

Protein score is -10*Log(P), where P is the probability that the observed match is a random event.
Protein scores greater than 73 are significant (p<0.05).

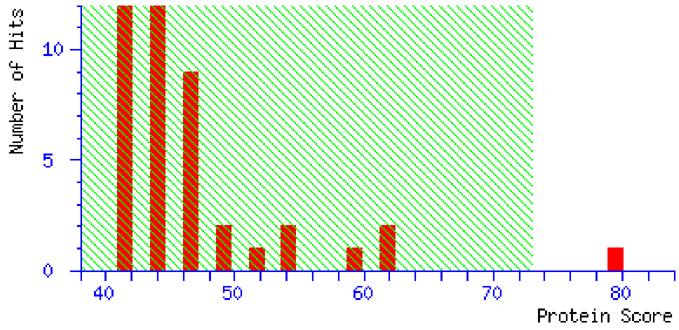

Matched peptide sequences: shown in Bold red:

```
  1 MAEGEEEVNI QEEANKGEEN PQEEANIQEE TNKGEENIQE EANIEEEANK
 51 EEESLKYLDF VQAATVYARA SFSKLYLFAK DKSGPFKPGV NTVESRFKSV
101 VRPVYNKFQP VPNKVLKFAD RRVDAYVTVL DRIVPPIVKR ASIQAYSVAP
151 GAALAVASYL PLHTKRLSKV LYGDG
```

Spot No.: **D91 (S100)**

NCBI accession No.: **gi|1709761** Species: *Spinacia oleracea*

PFF score: [49]

Protein name: Proteasome subunit alpha type-4

Matched peptides No.: [1] Sequence coverage %: [3]

Matched sequences: R.TTIFSPEGR.L

Calculated Mr: **27545** Calculated *p*I: **6.17**

Probability Based Mowse Score:

Ions score is -10*Log(P), where P is the probability that the observed match is a random event.
Individual ions scores > 44 indicate identity or extensive homology (p<0.05).
Protein scores are derived from ions scores as a non-probabilistic basis for ranking protein hits.

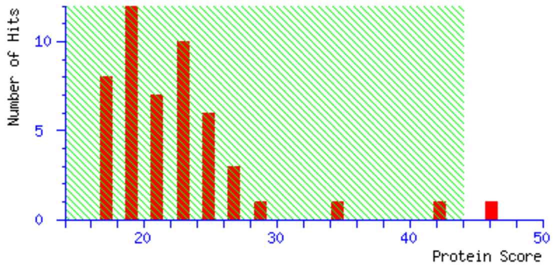

Matched peptide sequences: shown in Bold Red:

```
  1  MSRRYDSRTT IFSPEGRLYQ VEYAMEAIGN AGSAIGILAK DGVVLIGEKK
 51  VTSKLLQTST STEKMYKIDD HVACAVAGIM SDANILINTA RVQAQRYTFS
101  YQEPMPVEQL VQSLCDTKQG YTQFGGLRPF GVSFLFAGWD KNYGFQLYMS
151  DPSGNYGGWK ATAIGANNQA AQSMLKQDYK DDVTREDAVK LALKALSKTM
201  DSTSLTSEKL ELAEVYLLPS GKVKYQVHSP ESLNRLLTES GLTQPAAETS
```

Spot No.: **D92 (S103)**

NCBI accession No.: gi|38122474 Species: ***Hevea Brasiliensis***

Protein name: rubber elongation factor

PMF score: **125** Sequence coverage %: **68**

Matched peptides No.: **11** Total peptides No.: **50**

Calculated Mr: **19570** Calculated *p*I: **5.06**

Probability Based Mowse Score:

Protein score is -10*Log(P), where P is the probability that the observed match is a random event. Protein scores greater than 73 are significant (p<0.05).

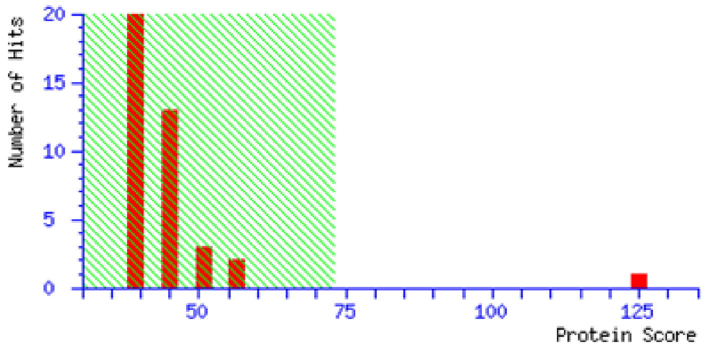

Matched peptide sequences: shown in Bold Red

```
  1 MAEGEEEVNI QEEANKGEEN PQEEANIQEE TNKGEENIQE EANIEEEANK
 51 EEESLKYLDF VQAATVYARA SFSKLYLFAK DKSGPFKPGV NTVESRFKSV
101 VRPVYNKFQP VPNKVLKFAD RRVDAYVTVL DRIVPPIVKR ASIQAYSVAP
151 GAALAVASYL PLHTKRLSKV LYGDG
```

Spot No.: **D93 (S105)**

NCBI accession No.: gi|157831407 Species: *Hevea brasiliensis*

Protein name： Chain A, Crystal Structures Of Hevamine

PMF score: 93 Sequence coverage %:51

Matched peptides No.: 13 Total peptides No.: 76

Calculated Mr: 29874 Calculated *p*I: 8.44

Probability Based Mowse Score:

Protein score is -10*Log(P), where P is the probability that the observed match is a random event.
Protein scores greater than 73 are significant (p<0.05).

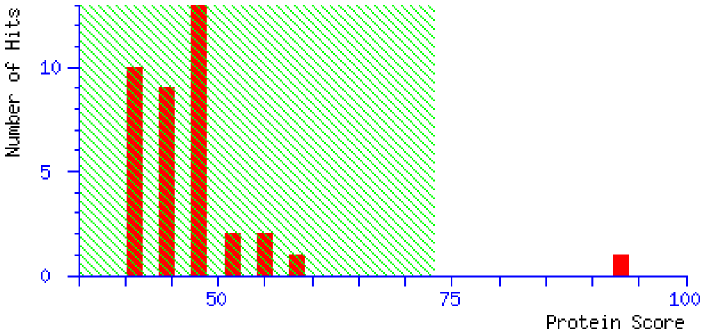

Matched peptide sequences: shown in Bold red:

1 GGIAIYWGQN GNEGTLTQTC STR**KYSYVNI** **AFLNK**FGNGQ TPQINLAGHC
51 NPAAGGCTIV SNGIR**SCQIQ** **GIKVMLSLGG** **GIGSYTLASQ** **ADAKNVADYL**
101 **WNNFLGGKSS** **SRPLGDAVLD** **GIDFDIEHGS** **TLYWDDLARY** LSAYSKQGK**K**
151 **VYLTAAPQCP** **FPDR**YLGTAL NTGLFDYVWV QFYNNPPCQY SSGNINNIIN
201 SWNRWTTSIN AGK**IFLGLPA** **APEAAGSGYV** **PPDVLISRIL** PEIKKSPK**YG**
251 **GVMLWSKFYD** **DK**NGYSSSIL DSV

Spot No.: **D94 (S108)**

NCBI accession No.: **gi|2493895** Species: *Citrullus lanatus*

PFF score: [89]

Protein name: cysteine synthase

Matched peptides No.: [1] Sequence coverage %: [ ]

Matched sequences: K.EGLLVGISSGAAAAAAIR.I

Calculated Mr: **34495** Calculated *p*I: **6.26**

Probability Based Mowse Score:

Ions score is -10*Log(P), where P is the probability that the observed match is a random event.
Individual ions scores > 41 indicate identity or extensive homology (p<0.05).
Protein scores are derived from ions scores as a non-probabilistic basis for ranking protein hits.

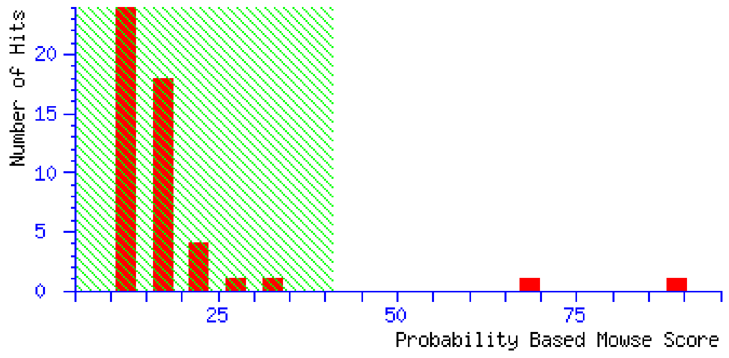

Matched peptide sequences: shown in Bold Red:

```
  1 MADAKSTIAK DVTELIGNTP LVYLNRVVDG CVARVAAKLE MMEPCSSVKD RIGYSMISDA
 61 ENKGLITPGE SVLIEPTSGN TGIGLAFIAA AKGYRLIICM PASMSLERRT ILRAFGAELV
121 LTDPARGMKG AVQKAEEIKA KTPNSYILQQ FENPANPKIH YETTGPEIWR GSGGKIDALV
181 SGIGTGGTVT GAGKYLKEQN PNIKLYGVEP VESAILSGGK PGPHKIQGIG AGFIPGVLDV
241 NLLDEVIQVS SEESIETAKL LALKEGLLVG ISSGAAAAAA IRIAKRPENA GKLIVAVFPS
301 FGERYLSTVL FESVKRETEN MVFEP
```

Spot No.: **D95 (S117)**

NCBI accession No.: **gi|51315784** Species: *Hevea brasiliensis*

PFF score: [59]

Protein name: Esterase

Matched peptides No.: [1] Sequence coverage %: [4]

Matched sequences: K.AAAFYPLNPPYGETFFHR.S

Calculated Mr: **43871** Calculated *p*I: **5.00**

Probability Based Mowse Score:

Ions score is -10*Log(P), where P is the probability that the observed match is a random event.
Individual ions scores > 43 indicate identity or extensive homology (p<0.05).
Protein scores are derived from ions scores as a non-probabilistic basis for ranking protein hits.

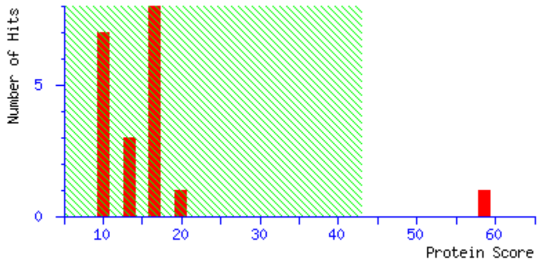

Matched peptide sequences: shown in Bold Red:

```
  1 MEFPETNNNP IITLSFLLCM LSLAYASETC DFPAIFNFGD SNSDTGGKAA
 51 AFYPLNPPYG ETFFHRSTGR YSDGRLIIDF IAESFNLPYL SPYLSSLGSN
101 FKHGADFATA GSTIKLPTTI IPAHGGFSPF YLDVQYSQFR QFIPRSQFIR
151 ETGGIFAELV PEEYYFEKAL YTFDIGQNDL TEGFLNLTVE EVNATVPDLV
201 NSFSANVKKI YDLGARTFWI HNTGPIGCLS FILTYFPWAE KDSAGCAKAY
251 NEVAQHFNHK LKEIVAQLRK DLPLATFVHV DIYSVKYSLF SEPEKHGFEF
301 PLITCCGYGG KYNFSVTAPC GDTVTADDGT KIVVGSCACP SVRVNWDGAH
351 YTEAANEYFF DQISTGAFSD PPVPLNMACH KTESLRTLAS V
```

Spot No.: **D96 (S118)**

NCBI accession No.: **gi|38122474** Species: *Hevea brasiliensis*

Protein name： rubber elongation factor

PMF score: **98** Sequence coverage %: **57**

Matched peptides No.: **9** Total peptides No.: **44**

Calculated Mr: **19570** Calculated *p*I: **5.06**

Probability Based Mowse Score:

Protein score is -10*Log(P), where P is the probability that the observed match is a random event.
Protein scores greater than 73 are significant (p<0.05).

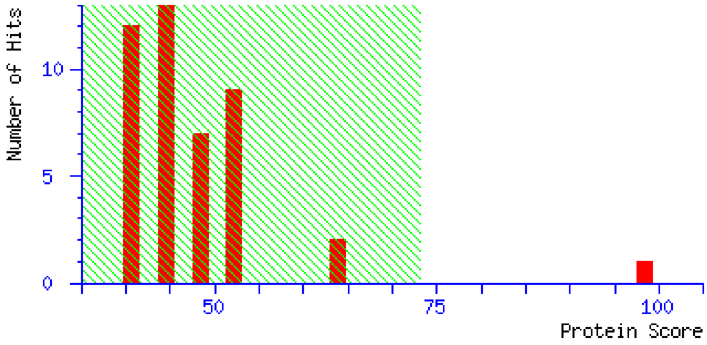

Matched peptide sequences: shown in Bold Red

```
  1 MAEGEEEVNI QEEANKGEEN PQEEANIQEE TNKGEENIQE EANIEEEANK
 51 EEESLKYLDF VQAATVYARA SFSKLYLFAK DKSGPFKPGV NTVESRFKSV
101 VRPVYNKFQP VPNKVLKFAD RRVDAYVTVL DRIVPPIVKR ASIQAYSVAP
151 GAALAVASYL PLHTKRLSKV LYGDG
```

Spot No.: **D97 (S121)**

NCBI accession No.: **gi| 255554865** Species: *Ricinus communis*

PFF score: [55]

Protein name: lactoylglutathione lyase, putative

Matched peptides No.: [1] Sequence coverage %: [3]

Matched sequences: K.FYTECFGMK.L

Calculated Mr: **31641** Calculated *p*I: **7.63**

Probability Based Mowse Score:

Ions score is -10*Log(P), where P is the probability that the observed match is a random event.
Individual ions scores > 40 indicate peptides with significant homology.
Individual ions scores > 44 indicate identity or extensive homology (p<0.05).
Protein scores are derived from ions scores as a non-probabilistic basis for ranking protein hits.

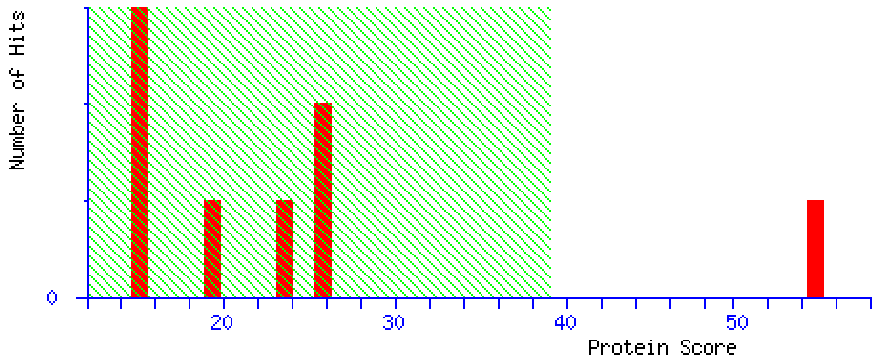

Matched peptide sequences: shown in Bold Red:

```
  1 MAAEATAPNA DVLEWPKKDK RRLLHAVYRV GDLDRTIKFY TECFGMKLLR
 51 KRDIPEEKYS NAFLGFGPEE TNFVVELTYN YGVTSYDIGT GFGHFAIATQ
101 DVYKLVEEVL AKGGAVTREP GPVKGGTTVI AFVKDPDGYI FELIQRGPTP
151 EPLCQVMLRV GDLDRSIRFY EKALGMKLLR KVDKPEYKYT LAMMGYADEY
201 ETTVLELTYN YGVTEYTKGN AYAQVAISTD DVYKSAEVVN LVTQELGGKI
251 TRQPGPIPGL NTKITSFLDP DGWKTKRKRR
```

Spot No.: **D98 (S123)**

NCBI accession No.: **gi|15225839** Species: *Arabidopsis thaliana*

Protein name: endopeptidase/ peptidase/ threonine-type endopeptidase

PMF Mascot score: **86** Sequence coverage %: **40**

Matched peptides No.: **14** Total peptides No.: **56**

Calculated Mr: **27645** Calculated *p*I: **5.93**

Probability Based Mowse Score:

Protein score is -10*Log(P), where P is the probability that the observed match is a random event. Protein scores greater than 73 are significant (p<0.05).

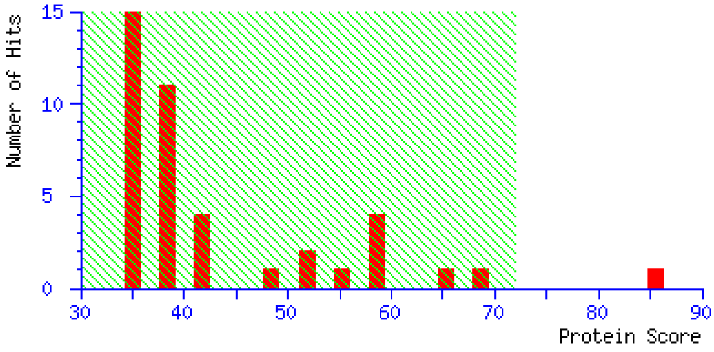

Matched peptide sequences: shown in Bold Red:

```
  1 MSSIGTGYDL SVTTFSPDGR VFQIEYAAKA VDNSGTVVGI KCKDGIVMGV
 51 EKLIASKMML PGSNRRIHSV HRHAGMAVAG LAADGRQIVA RAKSEARSYE
101 SVYGDAVPVK ELSERVASYV HLCTLYWWLR PFGCGVILGG YDRDGPQLYM
151 IEPSGISYRY FGAAIGKGKQ AAKTEIEKLN LSEMTCKEGV IEVAKIIYKL
201 HDEAKDKAFE LEMSWICEES KREHQKVPDD LLEEAKTAAK TALEEMDAD
```

Spot No.: **D99 (S126)**

NCBI accession No.: **gi|29170601** Species: *Hevea brasiliensis*

PFF score: [97]

Protein name: small rubber particle protein

Matched peptides No.: [1] Sequence coverage %: [12]

Matched sequences: K.ENENFQQEANEQEEK.L

Calculated Mr: **12769** Calculated *p*I: **4.61**

Probability Based Mowse Score:

Ions score is -10*Log(P), where P is the probability that the observed match is a random event.
Individual ions scores > 44 indicate identity or extensive homology (p<0.05).
Protein scores are derived from ions scores as a non-probabilistic basis for ranking protein hits.

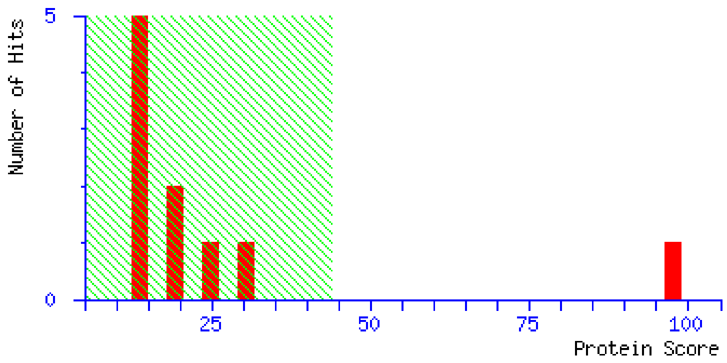

Matched peptide sequences: shown in Bold Red:

```
  1 MAEGKENENF QQEANEQEEK LKYLEFVQAT TDNAVTALSN IYLYAKDNSG
 51 PLKPGVETIE GVAKTVVIPA SKIPTEAIKF ADRAVDASFT TLQNIVPSVL
101 KQLPTQACDT SVKESAE
```

Spot No.: **D100 (S127)**

NCBI accession No.: **gi|2832430** Species: *Hevea brasiliensis*

PFF score: [84] Protein name: prohevein

Matched peptides No.: [1] Sequence coverage %: [10]

Matched sequences: K.YGWTAFCGPVGAHGQPSCGK.C

Calculated Mr: **20852** Calculated *p*I: **5.64**

Probability Based Mowse Score:

Ions score is -10*Log(P), where P is the probability that the observed match is a random event.
Individual ions scores > 43 indicate identity or extensive homology (p<0.05).
Protein scores are derived from ions scores as a non-probabilistic basis for ranking protein hits.

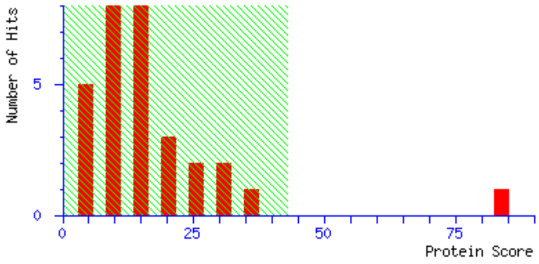

Matched peptide sequences: shown in Bold Red:

```
  1 EQCGRQAGGK LCPNNLCCSQ WGWCGSTDEY CSPDHNCQSN CKDSGEGVGG
 51 GSASNVLATY HLYNSQDHGW DLNAASAYCS TWDANKPYSW RSKYGWTAFC
101 GPVGAHGQPS CGKCLSVTNT GTGAKATVRI VDQCSNGGLD LDVNVFRQLD
151 TDGKGYERGH LTVNYQFVDC GDSFNPLFSV MKSSVIN
```

Spot No.: **D101 (S128)**

NCBI accession No.: **gi|224054224** Species: *Populus trichocarpa*

PFF score: [68] Protein name: predicted protein

Matched peptides No.: [2] Sequence coverage %: [12]

Matched sequences: R.AYVGDRPTWR.N;

R.NPQHPWR.V

Calculated Mr: **15003** Calculated *p*I: **6.82**

Probability Based Mowse Score:

Ions score is -10*Log(P), where P is the probability that the observed match is a random event.
Individual ions scores > 45 indicate identity or extensive homology (p<0.05).
Protein scores are derived from ions scores as a non-probabilistic basis for ranking protein hits.

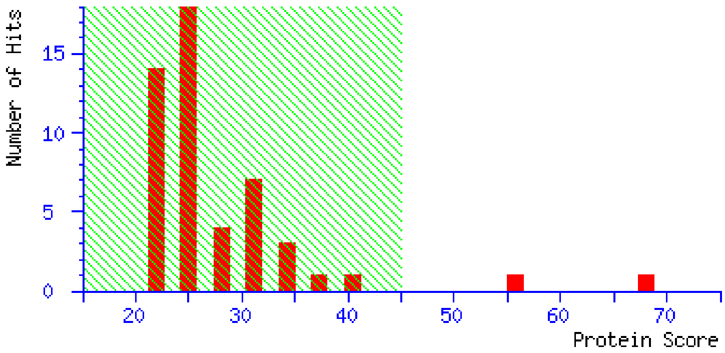

Matched peptide sequences: shown in Bold Red:

```
  1 MTVKLVDATI SSFGSVFEKF KAEAPKNKVN LILFLADNDP STNLSWCPDC
 51 VRAEPVILKK LEALPDDVAL LRAYVGDRPT WRNPQHPWRV DSRFKLKGVP
101 TLISWENDAV KGRLEDYEAH LEHKINALVS GN
```

Spot No.: **D102 (S130)**

NCBI accession No.: **gi|268321205**

Plantspecies: ***Hevea brasiliensis*** Protein name：**latex cystatin**

PMF score: **81** Sequence coverage %: **48**

Matched peptides No.: **6** Total peptides No.:**42**

Calculated Mr: **11248** Calculated *p*I: **5.45**

Probability Based Mowse Score:

Protein score is -10*Log(P), where P is the probability that the observed match is a random event.
Protein scores greater than 73 are significant (p<0.05).

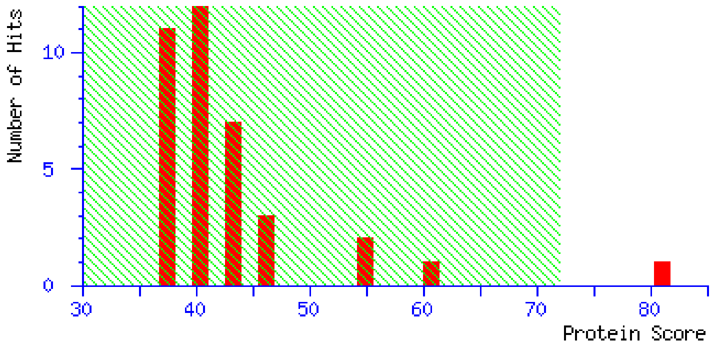

Matched peptide sequences: shown in Bold Red

```
  1 MAKLGGVKEV EGSANSVEIN SLARYAVDDY NQKQNALLEF KKVVNAKQQV
 51 VAGTIYYITL EVIDGGQKKV YEAKVWEKPW LNFKEVQEFK LIGDAPSDST
101 A
```

Spot No.: **D103 (S133)**

NCBI accession No.: **gi|242083244**

Plantspecies: ***Sorghum bicolor***

Protein name： **hypothetical protein SORBIDRAFT_08g008236**

PMF score: **81** Sequence coverage %: **89**

Matched peptides No.: **7** Total peptides No.:**33**

Calculated Mr: **8620** Calculated *p*I: **6.4**

Probability Based Mowse Score:

Protein score is -10*Log(P), where P is the probability that the observed match is a random event.
Protein scores greater than 73 are significant (p<0.05).

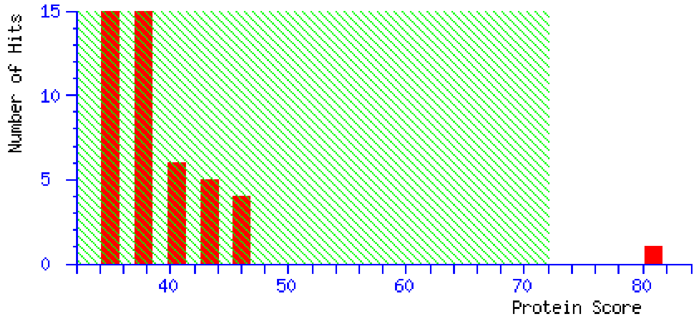

Matched peptide sequences: shown in **Bold Red**

```
 1 CVENIILSVL FWSKVQLCLR ASQPLLVALR IVDGDETPID PKIMVAMDVA
51 KAIIKESLKD KPILLNEVLK YYDNR
```

Spot No.: **D104 (S134)**

NCBI accession No.: **gi|29170601** Species: *Hevea brasiliensis*

PFF score: [85]

Protein name: small rubber particle protein

Matched peptides No.: [1] Sequence coverage %: [15]

Matched sequences: R.AVDASFTTLQNIVPSVLK.Q

Calculated Mr: **12769** Calculated *p*I: **4.61**

Probability Based Mowse Score:

Ions score is -10*Log(P), where P is the probability that the observed match is a random event.
Individual ions scores > 39 indicate peptides with significant homology.
Individual ions scores > 44 indicate identity or extensive homology (p<0.05).
Protein scores are derived from ions scores as a non-probabilistic basis for ranking protein hits.

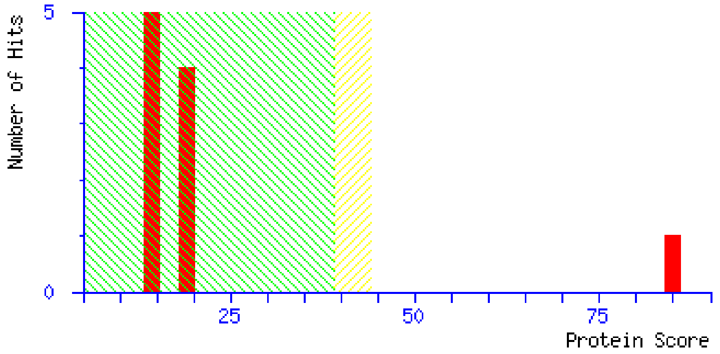

Matched peptide sequences: shown in Bold Red:

```
  1 MAEGKENENF QQEANEQEEK LKYLEFVQAT TDNAVTALSN IYLYAKDNSG
 51 PLKPGVETIE GVAKTVVIPA SKIPTEAIKF ADRAVDASFT TLQNIVPSVL
101 KQLPTQACDT SVKESAE
```

Spot No.: **D105 (S136)**

NCBI accession No.: **gi|354318** Species: *Triticum aestivum*

PFF score: [128] Protein name: calmodulin

Matched peptides No.: [1] Sequence coverage %: [10]

Matched sequences: R.VFDKDQDGFISAAELR.H

Calculated Mr: **16876** Calculated *p*I: **4.10**

Probability Based Mowse Score:

Ions score is -10*Log(P), where P is the probability that the observed match is a random event.
Individual ions scores > 45 indicate identity or extensive homology (p<0.05).
Protein scores are derived from ions scores as a non-probabilistic basis for ranking protein hits.

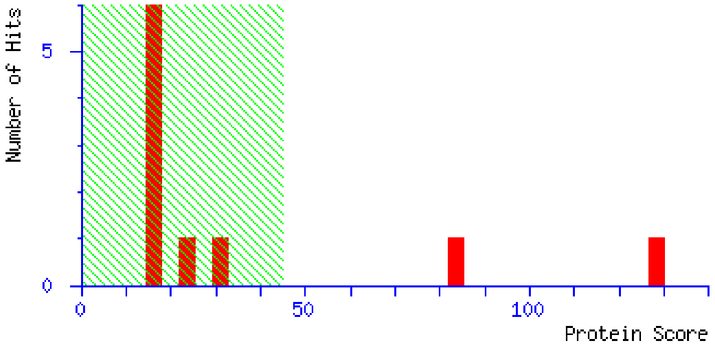

Matched peptide sequences: shown in Bold Red:

```
  1 ADQLTDEQNI AEFKEAFSLF DKDGDGCITT KELGTVMRSL GQNPTEAELQ
 51 DMINEVDADG NGTIDFPEFL NLMARKMKDT DSEEELKEAF RVFDKDQDGF
101 ISAAELRHVM TNLGEKLTDE EVDEMIREAD VDGDGQINYE EFVKVMMAK
```

Spot No.: **D106 (S137)**

NCBI accession No.: gi|14423933 Species: *Hevea brasiliensis*

Protein name： Small rubber particle protein

PMF score: **84** Sequence coverage %: **65**

Matched peptides No.: **9** Total peptides No.:**43**

Calculated Mr: **22331** Calculated *p*I: **4.80**

Probability Based Mowse Score:

Protein score is -10*Log(P), where P is the probability that the observed match is a random event.
Protein scores greater than 73 are significant (p<0.05).

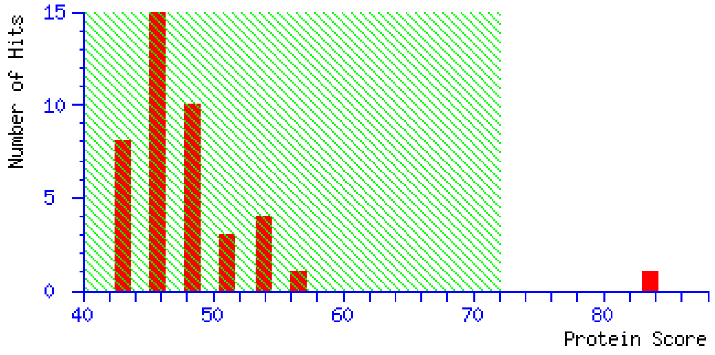

Matched peptide sequences: shown in Bold Red

```
  1 MAEEVEEERL KYLDFVRAAG VYAVDSFSTL YLYAKDISGP LKPGVDTIEN
 51 VVKTVVTPVY YIPLEAVKFV DKTVDVSVTS LDGVVPPVIK QVSAQTYSVA
101 QDAPRIVLDV ASSVFNTGVQ EGAKALYANL EPKAEQYAVI TWRALNKLPL
151 VPQVANVVVP TAVYFSEKYN DVVRGTTEQG YRVSSYLPLL PTEKITKVFG
201 DEAS
```

Spot No.: **D107 (S138)**

NCBI accession No.: gi|14423933 Species: *Hevea brasiliensis*

Protein name： Small rubber particle protein

PMF score: **162** Sequence coverage %: **85**

Matched peptides No.: **13** Total peptides No.:**43**

Calculated Mr: **22331** Calculated *p*I: **4.80**

Probability Based Mowse Score:

Protein score is -10*Log(P), where P is the probability that the observed match is a random event.
Protein scores greater than 73 are significant ($p<0.05$).

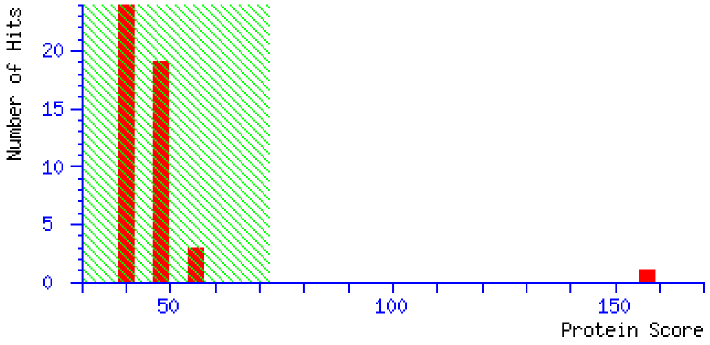

Matched peptide sequences: shown in Bold Red

```
  1 MAEEVEEERL KYLDFVRAAG VYAVDSFSTL YLYAKDISGP LKPGVDTIEN
 51 VVKTVVTPVY YIPLEAVKFV DKTVDVSVTS LDGVVPPVIK QVSAQTYSVA
101 QDAPRIVLDV ASSVFNTGVQ EGAKALYANL EPKAEQYAVI TWRALNKLPL
151 VPQVANVVVP TAVYFSEKYN DVVRGTTEQG YRVSSYLPLL PTEKITKVFG
201 DEAS
```

Spot No.: **D108 (S139)**

NCBI accession No.: gi|14423688   Species: *Hevea brasiliensis*

Protein name： **Enolase 1**

PMF score: 107   Sequence coverage %: 44

Matched peptides No.:14   Total peptides No.:47

Calculated Mr: 48029   Calculated *p*I:5.57

Probability Based Mowse Score:

Protein score is -10*Log(P), where P is the probability that the observed match is a random event.
Protein scores greater than 73 are significant (p<0.05).

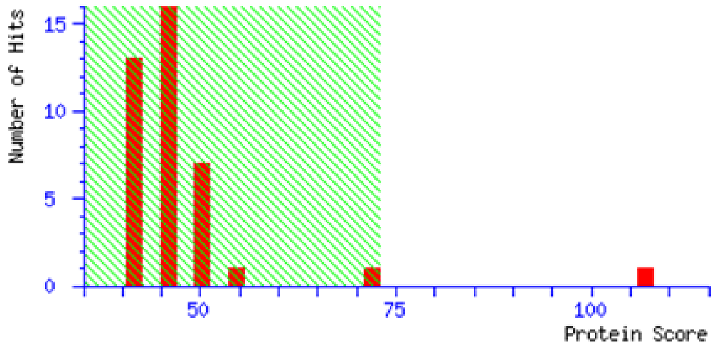

Matched peptide sequences: shown in Bold red:

```
  1  MAITIVSVRA RQIFDSRGNP TVEADVKLSD GYLARAAVPS GASTGIYEAL
 51  ELRDGGSDYL GKGVSKAVEN VNIIIGPALV GKDPTDQVGI DNFMVQQLDG
101  TVNEWGWCKQ KLGANAILAV SLAVCKAGAH VKGIPLYEHI ANLAGNKNLV
151  LPVPAFNVIN GGSHAGNKLA MQEFMILPVG ASSFKEAMKM GAEVYHHLKS
201  VIKKKYGQDA TNVGDEGGFA PNIQENKEGL ELLKTAIAKA GYTGKVVIGM
251  DVAASEFYGS DQTYDLNFKE ENNNGSQKIS GEALKDLYKS FVAEYPIVSI
301  EDPFDQDDWA HYAKLTSEIG EKVQIVGDDL LVTNPKRVEK AIKEKACNAL
351  LLKVNQIGSV TESIEAVKMS KRAGWGVMAS HRSGETEDTF IADLSVGLAT
401  GQIKTGAPCR SERLAKYNQL LRIEEELGSE AVYAGANFRK PVEPY
```

Spot No.: **D109 (S143)**

NCBI accession No.: **gi|333690873** Species: *Hevea brasiliensis*

PFF score: [52]

Protein name: glutathione-S-transferase tau 1

Matched peptides No.: [2] Sequence coverage %: [10]

Matched sequences: K.YEYREEDLR.N;

K.DKSPFLPSDPYQR.A

Calculated Mr: 25595 Calculated *p*I: **5.48**

Probability Based Mowse Score:

Ions score is -10*Log(P), where P is the probability that the observed match is a random event.
Individual ions scores > 45 indicate identity or extensive homology (p<0.05).
Protein scores are derived from ions scores as a non-probabilistic basis for ranking protein hits.

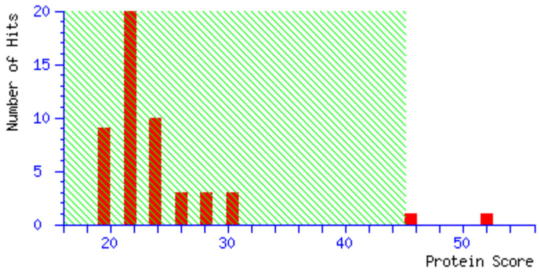

Matched peptide sequences: shown in Bold Red:

```
  1 MAEEVILLDF WPSPFGMRVR IALAEKGVKY EYREEDLRNK SPLLLQMNPV
 51 HKKIPVLIHN GKPICESLIA VQYVDEVWKD KSPFLPSDPY QRAQARFWAD
101 FIDKKIYDIG RKIWTTKGDE QEAAKKEFIE ALKLLEGELG NKPYFGGESM
151 GYVDVALIPF YSWFYAYETC GNFSIEPECP VLIAWAKRCL QKESVSESVP
201 DPEKVYEFVL VLKKKFGIE
```

Spot No.: **D110 (S145)**

NCBI accession No.: **gi|30575570** Species: *Citrus x paradisi*

PFF score: [87]

Protein name: HSP19 class II

Matched peptides No.: [1] Sequence coverage %: [15]

Matched sequences: K.VQVEDDNVLLISGER.K

Calculated Mr: **11191** Calculated *p*I: **8.01**

Probability Based Mowse Score:

Ions score is -10*Log(P), where P is the probability that the observed match is a random event.
Individual ions scores > 45 indicate identity or extensive homology (p<0.05).
Protein scores are derived from ions scores as a non-probabilistic basis for ranking protein hits.

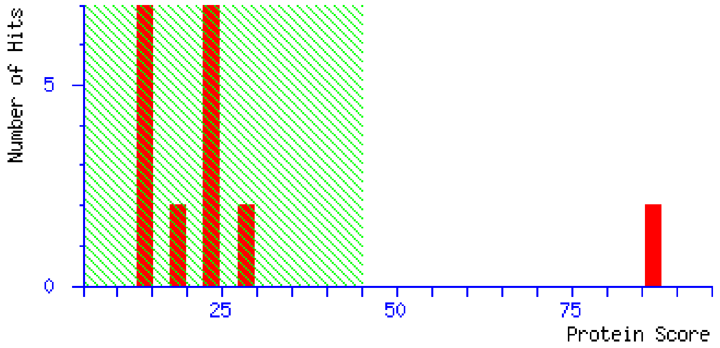

Matched peptide sequences: shown in Bold Red:

```
 1 YVFIVDMPGL KSGDIKVQVE DDNVLLISGE RKREEEKDGA KYVRMERRVG
51 KFMRKFVLPE NANVEAISAV CQDGVLTVTV DKLPPPEPKK PKTIEVKIA
```

Spot No.: **D111 (S146)**

NCBI accession No.: **gi|37901124** Species: *Hevea brasiliensis*

PFF score: [88]

Protein name: latex abundant protein 1

Matched peptides No.: [1] Sequence coverage %: [11]

Matched sequences: R.NKDDINCFNEYNPR.P

Calculated Mr: **14618** Calculated *p*I: **5.01**

Probability Based Mowse Score:

Ions score is -10*Log(P), where P is the probability that the observed match is a random event.
Individual ions scores > 43 indicate identity or extensive homology (p<0.05).
Protein scores are derived from ions scores as a non-probabilistic basis for ranking protein hits.

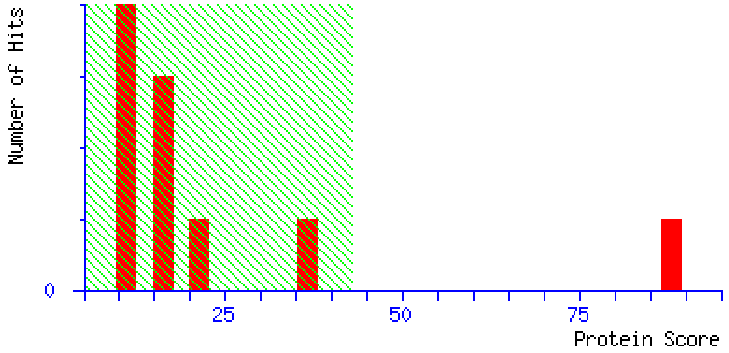

Matched peptide sequences: shown in Bold Red:

```
  1 MPYYTRNKDD INCFNEYNPR PYIGGYDMAL TYGRPISACE ETCYPRSSSA
 51 NEIDYDYPDF TWYVEPSAYN DDHLQEEYTS YARPKPRPAP NSLGSCPILG
101 GNDEEKKHHH CHHQHEKHCD DE
```

(Matched: **NKDD INCFNEYNPR**)

Spot No.: **D112 (S147)**

NCBI accession No.: **gi|359806300** Species: *Glycine max*

PFF score: [173]

Protein name: uncharacterized protein LOC100784610

Matched peptides No.: [3] Sequence coverage %: [9]

Matched sequences: K.NTALHYAAGYGR.K;

R.YWNNEEVLQK.L;

R.TALHFACGYGEVK.C

Calculated Mr: **38027** Calculated *p*I: **4.53**

Probability Based Mowse Score:

Ions score is -10*Log(P), where P is the probability that the observed match is a random event.
Individual ions scores > 45 indicate identity or extensive homology (p<0.05).
Protein scores are derived from ions scores as a non-probabilistic basis for ranking protein hits.

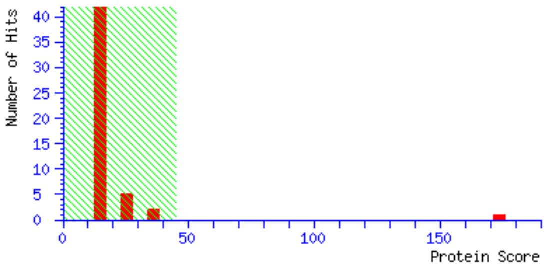

Matched peptide sequences: shown in **Bold Red:**

```
  1 MASDSKKDFP ADDKAGTEES KTSKDETSSK DSPAEQRATA TFGPRPGPAG
 51 HPGNPFDLSA MSGLLNDPSI KELAEQIAKD PSFNQMAEQL QKTFQGAPQD
101 AIPSFDNQQY FSTMQQVMQN PNFMTMAERL GNALMQDPSM SAMLESFSNP
151 SNKDQLEERM ARIKEDPSLK HILEEIETGG PAAMMRYWNN EEVLQKLGQA
201 MGLANSGDAG ASAENSGADE TEDLGNEDES IVHHTASVGD VEGLKNALAS
251 GADKDEEDSE GRTALHFACG YGEVKCAQVL LEAGAKVDAL DKNKNTALHY
301 AAGYGRKECV ALLLENGAAV TLQNMDGKTP IDVAKLNNQN EVLKLLEKDA
351 FL
```

Spot No.: **D113 (S148)**

NCBI accession No.: **gi|37625031** Species: *Vitis aestivalis*

PFF score: [166]

Protein name: putative ankyrin-repeat protein

Matched peptides No.: [3] Sequence coverage %: [9]

Matched sequences: K.LNNQHEVLK.L;

K.NTALHYAAGYGR.K;

R.TALHFACGYGEVK.C

Calculated Mr: **38089** Calculated *p*I: **4.53**

Probability Based Mowse Score:

Ions score is -10*Log(P), where P is the probability that the observed match is a random event.
Individual ions scores > 45 indicate identity or extensive homology (p<0.05).
Protein scores are derived from ions scores as a non-probabilistic basis for ranking protein hits.

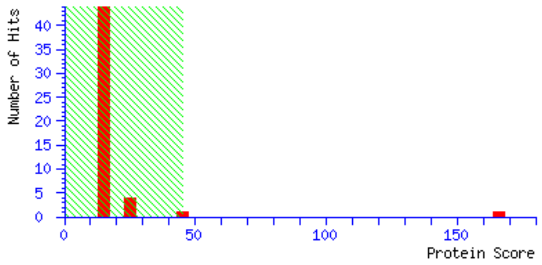

Matched peptide sequences: shown in Bold Red:

1 MASNSDKDTP AGSAENSKSD SSSGESHSEQ RRAAAAASVP GAGLPTNPFD
51 FSAMTGLLND PSIKELAEQI AKDPAFNQMA EQLQKTFHGA AVEESIPQFD
101 TQQYYSTMQQ VMQNPQFMTM AERLGNALMQ DPSMSSMLEN LANPTHKDQL
151 EERMARIKED PSLKPILDEI ETGGPAAMMR YWNDKDVLQK LGEAMGLAVS
201 GDAAASADNS GLDEAEELAN EDESIAHHHS ESIVHDTASV GDVEGLKNAL
251 ASGADKDEED SEGR**TALHFA CGYGEVK**CAQ ILVEAGATVD ALDKNK**NTAL**
301 **HYAAGYGR**KE CVALLLENGA AVTLQNMDGK TPIDVAK**LNN QHEVLK**LLEK
351 DAFL

Spot No.: **D114 (S149)**

NCBI accession No.: **gi|13310811** Species: *Nicotiana tabacum*

PFF score: [71]

Protein name: putative ankyrin-repeat protein

Matched peptides No.: [2] Sequence coverage %: [7]

Matched sequences: K.NTALHYAAGYGR.K;

R.TALHFACGYGEVK.C

Calculated Mr: **37522** Calculated *p*I: **4.45**

Probability Based Mowse Score:

Ions score is -10*Log(P), where P is the probability that the observed match is a random event.
Individual ions scores > 45 indicate identity or extensive homology (p<0.05).
Protein scores are derived from ions scores as a non-probabilistic basis for ranking protein hits.

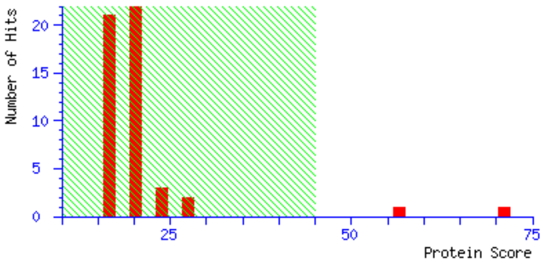

Matched peptide sequences: shown in Bold Red:

```
  1 MSEGEKVLPT ASADEKSGAS ENKKSSESSS TEAPSGEART TSTAAAGAGL
 51 QNPFDFSAMS GLLNDPSIKE LAEQIAKDPA FNQMAEQLQK TFQGAAVEES
101 VPNFDSQQYY STMQQVMQNP QFMTMAERLG NALMQDPSMS GMLESLSNPA
151 QKEQIEERMA RIKEDPSLKP ILEEIESGGP AAMMRYWNDQ ETLKKIGEAM
201 GFAAGGEGAT SSAIPGTDET EEANEDESVV HQCASVGDAE GLKAALTAGA
251 DKDEEDSEGR TALHFACGYG EVKCAQILLE AGAKVDALDK NKNTALHYAA
301 GYGRKECVAL LLENGAAVTL QNLDGKTPID VAKLNNQQEV LKLLEKDVFL
```

Spot No.: **D115 (S151)**

NCBI accession No.: **gi|378464888**

Species: *Ammopiptanthus mongolicus*

PFF score: [117]

Protein name: translation elongation factor

Matched peptides No.: [2]   Sequence coverage %: [11]

Matched sequences: R.WFNHIDALLR.I;

K.SSVLLDVKPWDDETDMK.K

Calculated Mr: **25300**   Calculated *p*I: **4.50**

Probability Based Mowse Score:

Ions score is -10*Log(P), where P is the probability that the observed match is a random event.
Individual ions scores > 45 indicate identity or extensive homology (p<0.05).
Protein scores are derived from ions scores as a non-probabilistic basis for ranking protein hits.

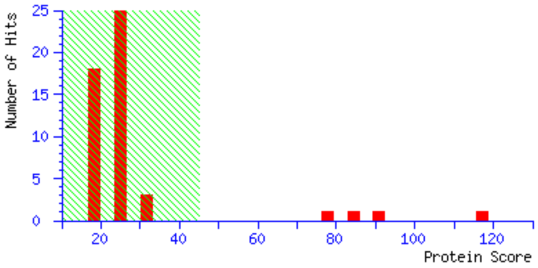

Matched peptide sequences: shown in Bold Red:

```
  1 MVLTLYNLSS EPGLKKLDEY LLSRSYITGY QASKDDLTVY AALPEVPSDK
 51 YVNVSRWFNH IDALLRISGV SGEGSGVTVE GSAPVAEETI ATPPAADTKA
101 TAAEDDDDDV DLFGEETEEE KKAAEERAAS VKASVKKKES GKSSVLLDVK
151 PWDDETDMKK LKEAVRSVSM EGLHWGASKL VPVGYGIKKL QIMLTIVDDL
201 VSVDNLIEEQ LQVEPINEYV QSCDIVAFNK I
```

Spot No.: **D116 (S153)**

NCBI accession No.: **gi|22261807** Species: *Solanum tuberosum*

PFF score: [132]

Protein name: Light-induced protein, chloroplastic

Matched peptides No.: [1] Sequence coverage %: [8]

Matched sequences: K.FEEGIIGTPQLTDSIVLPENVEFLGQK.I

Calculated Mr: **35671** Calculated *p*I: **5.26**

Probability Based Mowse Score:

Ions score is -10*Log(P), where P is the probability that the observed match is a random event.
Individual ions scores > 45 indicate identity or extensive homology (p<0.05).
Protein scores are derived from ions scores as a non-probabilistic basis for ranking protein hits.

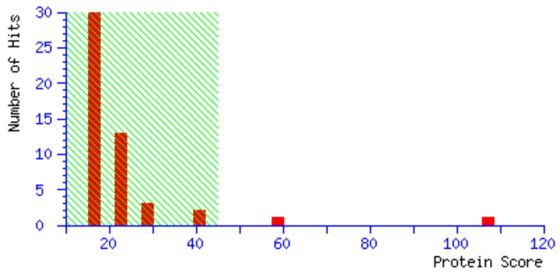

Matched peptide sequences: shown in Bold Red:

```
  1 MAVTFYGVNS GSGLKKLDEY LLTRSYISGY QASKDDITVH AALSKAPSSE
 51 YVNVSRWYNH IESLLRISGV SGEGCGVTIE GFAPVDAVAT PPAEDTKATA
101 AEDDDDDDVD LFGEETEEEK KAAEARAAAV KASGKKKESG KSSVLLDVKP
151 WDDETDMKKL EEAVRSIKME GLLWGASKLV PVGYGIKKLQ IMLTIVDDLV
201 SVDDLIEEQL TVEPTNEYVQ SCDIVAFNKI
```

Spot No.: **D117 (S161)**

NCBI accession No.: **gi|147805616** Species: *Vitis vinifera*

PFF score: [136]

Protein name: hypothetical protein VITISV_028800

Matched peptides No.: [3] Sequence coverage %: [10]

Matched sequences: R.GPYPADQVVR.D;
R.DRGPYPADQVVR.D;
K.TANEVIIVIEAYR.T

Calculated Mr: 27385 Calculated *p*I: **5.64**

Probability Based Mowse Score:

Ions score is -10*Log(P), where P is the probability that the observed match is a random event.
Individual ions scores > 45 indicate identity or extensive homology ($p<0.05$).
Protein scores are derived from ions scores as a non-probabilistic basis for ranking protein hits.

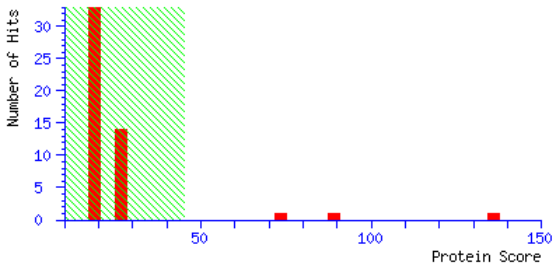

Matched peptide sequences: shown in Bold Red:

```
  1 MLAVFHKSIA EAPEGLRTPD SASLPALKDG FLPQFFASLH PSAVTVNLGS
 51 SGAISYSVDK QNPLLPRLFA VVDDIFCLFQ GHIENVALLK QQYGLNKTAN
101 EVIIVIEAYR TLRDRGPYPA DQVVRDLHGK FAFVLYDSSN RTAFLAADAD
151 ESVPFFWGVD SEGHLVLSDD EETVKKGCGK SFAPFPKGCF FTTSGGLRSF
201 EHPLNELRAE PRVDSSGQVC GANFKVDVEA KKETGMPRVG SAANWSTHY
```

Spot No.: **D118 (S164)**

NCBI accession No.: **gi|225428086**   Species: *Vitis vinifera*

PFF score: [377]

Protein name： V-type proton ATPase subunit B 1

Matched peptides No.: [10]   Sequence coverage %: [23]

Matched sequences: K.YQEIVNIR.L;K.FVAQGAYDTR.N;
R.DFEENGSMER.V;R.RGQVLEVDGEK.A;
R.KFVAQGAYDTR.N;K.YTTVQFTGEVLK.T;
R.QIYPPINVLPSLSR.L;R.VTLFLNLANDPTIER.I;
R.GYPGYMYTDLATIYER.A;
R.RDHADVSNQLYANYAIGK.D

Calculated Mr: **54367**   Calculated *p*I: **5.04**

Probability Based Mowse Score:

Mascot Score Histogram

Ions score is -10*Log(P), where P is the probability that the observed match is a random event.
Individual ions scores > 45 indicate identity or extensive homology (p<0.05).
Protein scores are derived from ions scores as a non-probabilistic basis for ranking protein hits.

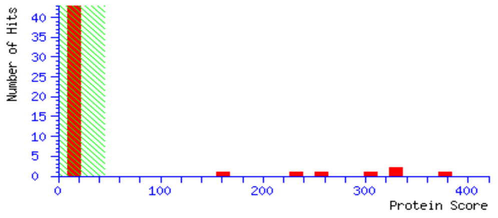

Matched peptide sequences: shown in Bold Red:

```
  1 MGVEQNNHDA EEGTLLVGME YRTVSGVAGP LVILEKVKGP KYQEIVNIRL
 51 GDGTTRRGQV LEVDGEKAVV QVFEGTSGID NKYTTVQFTG EVLKTPVSLD
101 MLGRIFNGSG KPIDNGPPIL PEAYLDISGS SINPSERTYP EEMIQTGIST
151 IDVMNSIARG QKIPLFSAAG LPHNEIAAQI CRQAGLVKRL EKSGSLLEDV
201 EEDNFAIVFA AMGVNMETAQ FFKRDFEENG SMERVTLFLN LANDPTIERI
251 ITPRIALTTA EYLAYECGKH VLVILTDMSS YADALREVSA AREEVPGRRG
301 YPGYMYTDLA TIYERAGRIE GRKGSITQIP ILTMPNDDIT HPTPDLTGYI
351 TEGQIYIDRQ LHNRQIYPPI NVLPSLSRLM KSAIGEGMTR RDHADVSNQL
401 YANYAIGKDV QAMKAVVGEE ALSSEDLLYL EFLDKFERKF VAQGAYDTRN
451 IFQSLDLAWT LLRIFPRELL HRIPAKTLDQ YYSREASN
```

Spot No.: **D119 (S165)**

NCBI accession No.: **gi|224071449** Species: *Populus trichocarpa*

PFF score: [59]

Protein name: predicted protein

Matched peptides No.: [1] Sequence coverage %: [2]

Matched sequences: K.IDVIPHYPGR.L

Calculated Mr: **47121** Calculated *p*I: **5.28**

Probability Based Mowse Score:

Ions score is -10*Log(P), where P is the probability that the observed match is a random event.
Individual ions scores > 44 indicate identity or extensive homology ($p<0.05$).
Protein scores are derived from ions scores as a non-probabilistic basis for ranking protein hits.

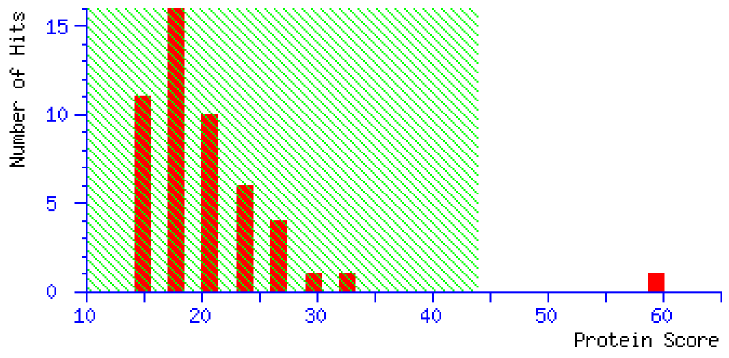

Matched peptide sequences: shown in Bold Red:

```
  1 MDGTSEATWP EILGSRNWDN LLDPLDLSLR KLILRCGDFC QATYDAFNND
 51 QNSRYCGTSR YGKRNFFHKV MLDNPENYQV SSFLYATARV SLPEAFLLHS
101 LSRDSWDRET NWIGYIAVTS DEQTKTLGRR EIYIAFRGTT RNYEWVDILG
151 AKLKSAKPLL RGATSTTHDQ ESSSSSDDDD DKVPKVMLGW LTMYISDDPN
201 SPFTKLSARA QLLAHIKELR ERYKDDDLSI IFTGHSLGAS LSILSAFDLV
251 ENGITDIPVS AFVFGSPQVG NKEFNERFNK YPNLKVLHIK NKIDVIPHYP
301 GRLMGYVYTG IEFEIDTRKS PSLKDSKNPS DWHNLQAMLH IVAGWNGEEQ
351 EFELKVKRSL ALVNKSSEFL KDECLVPGIW WVEKNKGMVR NEDGEWVLAP
401 PDEEDLPVPE C
```

Spot No.: **D120 (S166)**

NCBI accession No.: **gi|116789736** Species: *Picea sitchensis*

Protein name: unknown

PMF Mascot score: **81** Sequence coverage %: **60**

Matched peptides No.: **6** Total peptides No.: **48**

Calculated Mr: **13436** Calculated *p*I: **5.91**

Probability Based Mowse Score:

Protein score is -10*Log(P), where P is the probability that the observed match is a random event. Protein scores greater than 73 are significant (p<0.05).

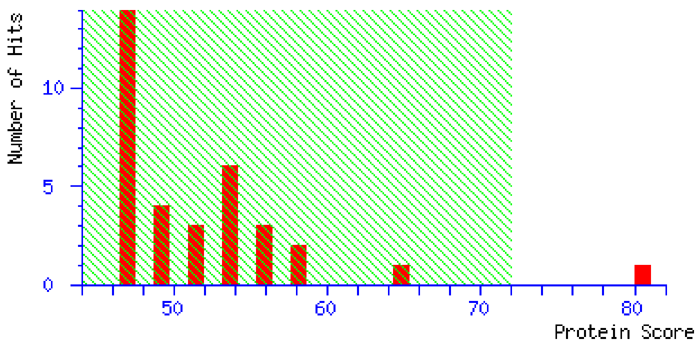

Matched peptide sequences: shown in Bold Red:

```
  1 MPSHSLIGTT TTNRRGSMED YPNPFNPETI HELFKLVWSQ TNKARAQKEG
 51 GETELEVGAA ASKKTRTTTA NSNALKLSCE FLRLFVTEAV QRAAIVAEVE
101 GSTQIEGTHL ERILPQLLLD F
```

Spot No.: **D121 (S167)**

NCBI accession No.: **gi|115392253**

Species: *Cercocarpus betuloides*

PFF score: [51]   Protein name: polyphenol oxidase

Matched peptides No.: [1]   Sequence coverage %: [3]

Matched sequences: K.EKEQEEEILVIEGIEFDR.D

Calculated Mr: **51023**   Calculated *p*I: **5.98**

Probability Based Mowse Score:

Ions score is -10*Log(P), where P is the probability that the observed match is a random event.
Individual ions scores > 45 indicate identity or extensive homology (p<0.05).
Protein scores are derived from ions scores as a non-probabilistic basis for ranking protein hits.

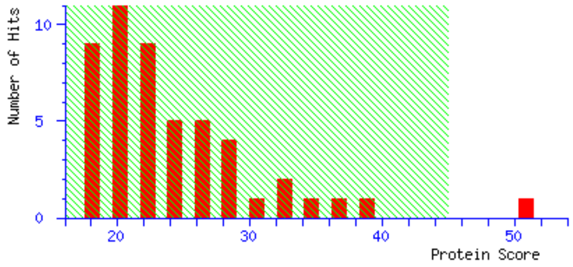

Matched peptide sequences: shown in **Bold Red:**

```
  1 PFAFAKPVAP PDVSKCGAAD LPTGAQPTNC CPPVASKIID FKLPSPTPLR VRPAAHAVDK
 61 AYIEKYSKAI ELMKALPDDD PRSFKQQANV HCAYCDGAYD QAGFPDLELQ IHNSWLFFPF
121 HRYYLYFHEK ILGSLIGDPT FALPFWNWDA PAGMPLPAIY ANPKSPLYDK FRAANHQPPT
181 LVDLDFNGTE DAASNETQIN ANLKIMYRQM VSNAKTPQLF FGNPYRAGDE PDPGGGSIEG
241 TPHGPVHLWT GDNTQPNFED MGNFYSAGRD PIFFAHHSNV DRLWSIWKTL GGKRTDITDT
301 DWLDSGFLFY DENAQMVRVK VRDCLESKNL GYVYQDANIP WLESKPTPRR VKAALGKIAK
361 KLHVAHAADN SSASKVVARA AFPIAKLDTK MSTVVARPKQ KKRSKKEKEQ EEEILVIEGI
421 EFDRDVAVKF DVYVNDEDDL PSGPDKSEFA GSFVS
```

Spot No.: **D122 (S168)**

NCBI accession No.: **gi|225430941** Species: *Vitis vinifera*

PFF score: [210]

Protein name: regulator of ribonuclease-like protein 2

Matched peptides No.: [3] Sequence coverage %: [24]

Matched sequences: K.VFEDNVLIR.E;

R.DVDEINGCDIGVR.A;

R.ISDGEWLYADTDGILISR.T

Calculated Mr: **17984** Calculated *p*I: **5.39**

Probability Based Mowse Score:

Ions score is -10*Log(P), where P is the probability that the observed match is a random event.
Individual ions scores > 45 indicate identity or extensive homology (p<0.05).
Protein scores are derived from ions scores as a non-probabilistic basis for ranking protein hits.

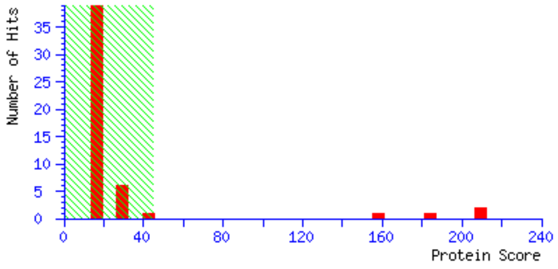

Matched peptide sequences: shown in **Bold Red**

```
  1 MALVTTAEVC DANPQLIVSG ELRALQPVFQ IYGRRPVFSG PIVTLKVFED
 51 NVLIREFLEE KGNGRVLVVD GGGSMRCAIL GGNPVVQAQN NGWAGIVVNG
101 CIRDVDEING CDIGVRALNS HPMKANKKGI GEKHVPIAIA GTRISDGEWL
151 YADTDGILIS RTELSV
```

Spot No.: **D123 (S171)**

NCBI accession No.: **gi|115392253**

Species: *Cercocarpus betuloides*

PFF score: [104]   Protein name: actin depolymerizing factor

Matched peptides No.: [1]   Sequence coverage %: [4]

Matched sequences: K.EKEQEEEILVIEGIEFDR.D

Calculated Mr: **58369**   Calculated *p*I: **9.23**

Probability Based Mowse Score:

Ions score is -10*Log(P), where P is the probability that the observed match is a random event.
Individual ions scores > 40 indicate peptides with significant homology.
Individual ions scores > 44 indicate identity or extensive homology (p<0.05).
Protein scores are derived from ions scores as a non-probabilistic basis for ranking protein hits.

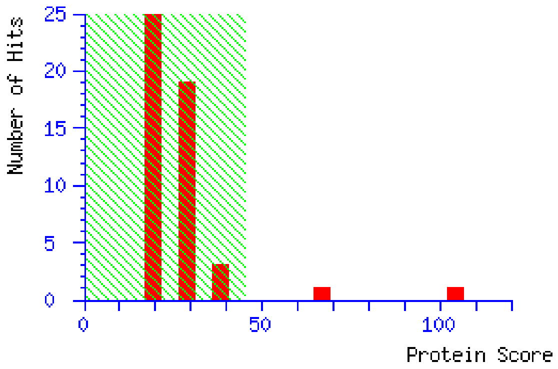

Matched peptide sequences: shown in Bold Red:

```
  1 PFAFAKPVAP PDVSKCGAAD LPTGAQPTNC CPPVASKIID FKLPSPTPLR VRPAAHAVDK
 61 AYIEKYSKAI ELMKALPDDD PRSFKQQANV HCAYCDGAYD QAGFPDLELQ IHNSWLFFPF
121 HRYYLYFHEK ILGSLIGDPT FALPFWNWDA PAGMPLPAIY ANPKSPLYDK FRAANHQPPT
181 LVDLDFNGTE DAASNETQIN ANLKIMYRQM VSNAKTPQLF FGNPYRAGDE PDPGGGSIEG
241 TPHGPVHLWT GDNTQPNFED MGNFYSAGRD PIFFAHHSNV DRLWSIWKTL GGKRTDITDT
301 DWLDSGFLFY DENAQMVRVK VRDCLESKNL GYVYQDANIP WLESKPTPRR VKAALGKIAK
361 KLHVAHAADN SSASKVVARA AFPIAKLDTK MSTVVARPKQ KKRSKKEKEQ EEEILVIEGI
421 EFDRDVAVKF DVYVNDEDDL PSGPDKSEFA GSFVS
```

Spot No.: **D124 (S177)**

NCBI accession No.: **gi|357480975** Species: *Medicago truncatula*

PFF score: [369]

Protein name: Proteasome subunit beta type

Matched peptides No.: [7] Sequence coverage %: [42]

Matched sequences: K.NVALYQFR.N;
R.VQFTEYIQK.N;
R.LVVAPPNFVIK.I;
R.NGIPLTTAAAANFTR.G;
K.GAFGYGSYFSLSMMDR.H;
K.ETGPSLYYIDYIATLHK.L;
K.LIAASGEPGDRVQFTEYIQK.N

Calculated Mr: **22572** Calculated *p*I: **6.06**

Probability Based Mowse Score:

Ions score is -10*Log(P), where P is the probability that the observed match is a random event.
Individual ions scores > 45 indicate identity or extensive homology (p<0.05).
Protein scores are derived from ions scores as a non-probabilistic basis for ranking protein hits.

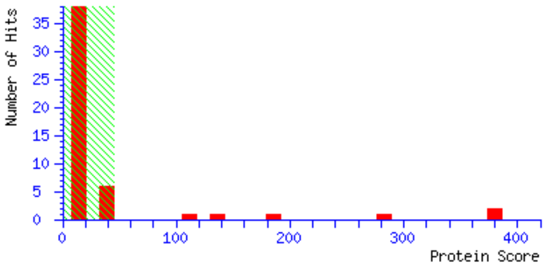

Matched peptide sequences: shown in Bold Red:

1 MECVFGLVGN GFAIVVADTS AVHSILVHKS NEDKIMFLDS HK**LIAASGEP**
51 **GDRVQFTEYI QKNVALYQFR NGIPLTTAAA ANFTR**GELAT ALRKNPYSVN
101 ILLAGYDK**ET GPSLYYIDYI ATLHK**LEKGA **FGYGSYFSLS MMDR**HFHSGM
151 NVEEAIDLVD KCILEIRSR**L VVAPPNFVIK** IVDKDGAREY AWRESVKDTP
201 ASA

Spot No.: **D125 (S187)**

NCBI accession No.: **gi|380468126** Species: *Hevea brasiliensis*

PFF score: [123]

Protein name: caffeic acid 3-O-methyltransferase

Matched peptides No.: [3] Sequence coverage %: [11]

Matched sequences: K.NPEAPVLLDR.M;

K.SWYHLNEAILEGGTPFNR.A;

R.AYGMNQFEYPGTDQR.F + Oxidation (M)

Calculated Mr: **40957** Calculated *p*I: **5.54**

Probability Based Mowse Score:

Ions score is -10*Log(P), where P is the probability that the observed match is a random event.
Individual ions scores > 45 indicate identity or extensive homology (p<0.05).
Protein scores are derived from ions scores as a non-probabilistic basis for ranking protein hits.

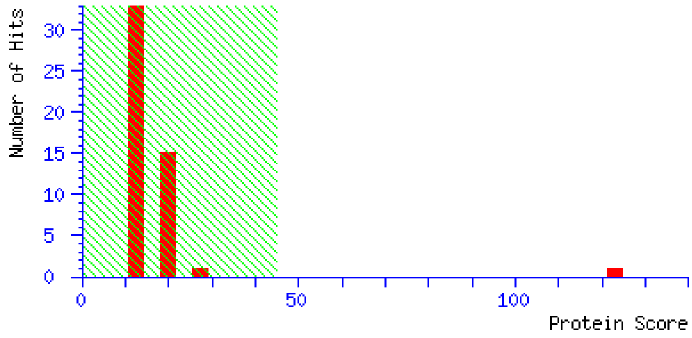

Matched peptide sequences: shown in **Bold Red:**

```
  1 MTGSEQSNSA SIMSGRSDEE TWNLAIDLAN TVILPMVLKS ALELNVIDII
 51 STAGNSGASL SAPEIAQRIP EAKNPEAPVL LDRMLRLLAT YDIVKCSSNT
101 KENGEVERLY APGPICKFLT KNKNGSGSAA PLLLLHHDEV FMKSWYHLNE
151 AILEGGTPFN RAYGMNQFEY PGTDQRFNRV FNDAMSSYTT YLVKKILDAY
201 KGFDGLKSLV DVGGNSGVTL NSITSKYPHI KGINYDLPHV LADAPSYPGV
251 EHVAGDMFKS VPKGDAIILK WVLHDWNDDL CLKLLKNCWE ALPSNGKVIV
301 VESILPTVPE NNVTSQVLHK EDLMLLSFNV GGKERTRQEF EALASKSGFS
351 SCEFICCAYN SWVIEFHK
```

Spot No.: **D126 (S188)**

NCBI accession No.: **gi|380468126** Species: *Hevea brasiliensis*

PFF score: [117]

Protein name: caffeic acid 3-O-methyltransferase

Matched peptides No.: [2] Sequence coverage %: [8]

Matched sequences: K.SWYHLNEAILEGGTPFNR.A;

R.AYGMNQFEYPGTDQR.F + Oxidation (M)

Calculated Mr: **40957** Calculated *p*I: **5.54**

Probability Based Mowse Score:

Ions score is -10*Log(P), where P is the probability that the observed match is a random event.
Individual ions scores > 44 indicate identity or extensive homology (p<0.05).
Protein scores are derived from ions scores as a non-probabilistic basis for ranking protein hits.

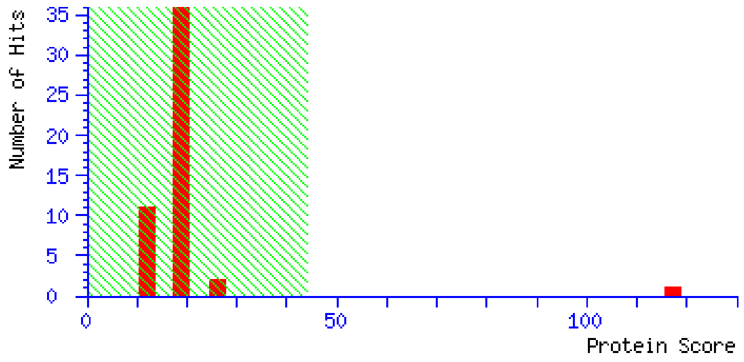

Matched peptide sequences: shown in **Bold Red:**

```
  1 MTGSEQSNSA SIMSGRSDEE TWNLAIDLAN TVILPMVLKS ALELNVIDII
 51 STAGNSGASL SAPEIAQRIP EAKNPEAPVL LDRMLRLLAT YDIVKCSSNT
101 KENGEVERLY APGPICKFLT KNKNGSGSAA PLLLLHHDEV FMKSWYHLNE
151 AILEGGTPFN RAYGMNQFEY PGTDQRFNRV FNDAMSSYTT YLVKKILDAY
201 KGFDGLKSLV DVGGNSGVTL NSITSKYPHI KGINYDLPHV LADAPSYPGV
251 EHVAGDMFKS VPKGDAIILK WVLHDWNDDL CLKLLKNCWE ALPSNGKVIV
301 VESILPTVPE NNVTSQVLHK EDLMLLSFNV GGKERTRQEF EALASKSGFS
351 SCEFICCAYN SWVIEFHK
```

Spot No.: **D127 (S189)**

NCBI accession No.: **gi|284433794** Species: *Jatropha curcas*

PFF score: [159]

Protein name: glutathione S-transferase omega

Matched peptides No.: [3] Sequence coverage %: [10]

Matched sequences: K.IIGESLDLIK.Y;
K.YDITAGRPK.L;
K.ELVEFYK.K

Calculated Mr: **27480** Calculated *p*I: **5.41**

Probability Based Mowse Score:

Ions score is -10*Log(P), where P is the probability that the observed match is a random event.
Individual ions scores > 45 indicate identity or extensive homology (p<0.05).
Protein scores are derived from ions scores as a non-probabilistic basis for ranking protein hits.

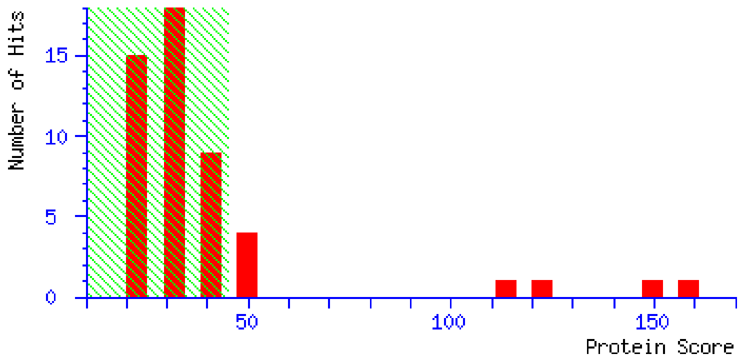

Matched peptide sequences: shown in Bold Red:

```
  1  MAATALDKSL  PEKLPPILDA  TAEQPPLFDG  TIRLYTSYAC  PFAQRVWITR
 51  NYKGLQDKIK  LVPLNLQNRP  SWYGEKVYPA  NKVPALEHNG  KIIGESLDLI
101  KYIDANFEGP  SLLPDDPAKK  EFAEELFSYT  DTFTKTVFTS  FKGDAVKDAS
151  PAFDYLENAL  HKFEDGPFLL  GQFSLVDIAY  IPFVERFQIF  FSEVFKYDIT
201  AGRPKLAAWI  EEMNKIEAYK  QTKTDPKELV  EFYKKRLLAQ
```

Spot No.: **D128 (S190)**

NCBI accession No.: **gi|255567721** Species: *Ricinus communis*

PFF score: [52]

Protein name: carboxymethylenebutenolidase, putative

Matched peptides No.: [1] Sequence coverage %: [6]

Matched sequences: K.SMGMPDEDEAAVELAWSR.F + 2 Oxidation (M)

Calculated Mr: 30833 Calculated *p*I: **6.09**

Probability Based Mowse Score:

Ions score is -10*Log(P), where P is the probability that the observed match is a random event.
Individual ions scores > 45 indicate identity or extensive homology (p<0.05).
Protein scores are derived from ions scores as a non-probabilistic basis for ranking protein hits.

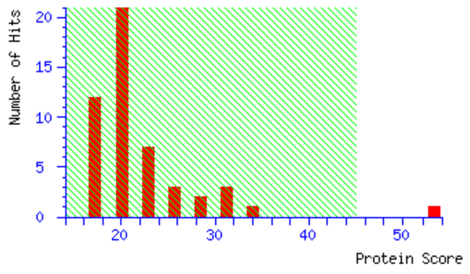

Matched peptide sequences: shown in Bold Red:

```
  1 MLAAAIRSLS PSSTSSIFNK SALPPLLRSL SPSLSNSRFQ IRSMADSASS
 51 PFKKVQIHRD NTTFDAYVIG KDDAPGIVVV QEWWGVDFEI KNHAEKISQL
101 EPGFKALIPD LYRGKVGLDV AEAQHLMDGL DWQGAVKDIG ASVNWLKANG
151 SKKAGVTGFC MGGALAIASS VLVPEVDAVV AFYGVPPSEL ADATQAKAPI
201 QAHFGELDNF VGFADITAAK ALEEKLKASG VPSEVHIYPG NAHAFMNRSA
251 EGVKRRKSMG MPDEDEAAVE LAWSRFRSWM NQYLSA
```

Spot No.: **D192 (S129)**

NCBI accession No.: **gi|255542380** Species: *Ricinus communis*

Protein name: cysteine synthase, putative

PMF Mascot score: **82** Sequence coverage %: **37**

Matched peptides No.: **13** Total peptides No.: **59**

Calculated Mr: **34435** Calculated *p*I: **5.50**

Probability Based Mowse Score:

Protein score is -10*Log(P), where P is the probability that the observed match is a random event.
Protein scores greater than 73 are significant (p<0.05).

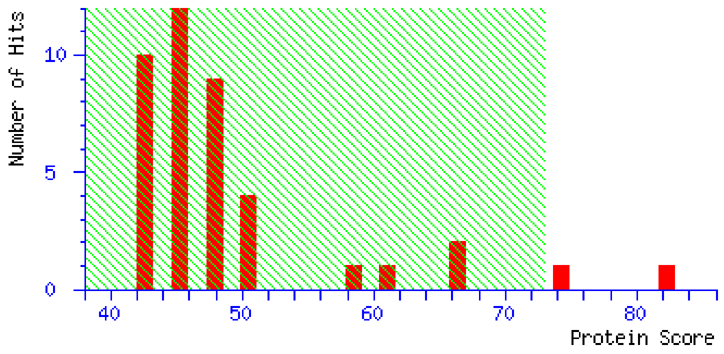

Matched peptide sequences: shown in Bold Red:

1 MVEEKSAIAK DVTELVGK**TP** **LVYLNHVVDG** **CVARIAAKLE** **MMEPCSSVK**D
51 RIGYSMITDA EEK**GLIKPGE** **SVLIEPTSGN** **TGIGLAFMAA** **AKGYKLIITM**
101 **PASMSLERRM** **VLRAFGAELV** **LTDPAR**GMKG AVQKAEEILA KTPNSYILQQ
151 FENPANPKIH YETTGPEIWK GSGGKVDFFV SGIGTGGTVT GAGGYLREQN
201 PDIKLIGVEP VESAVLSGGK PGPHKIQGIG AGFIPGVLDV SLLDEVVQIS
251 SEEAIETAKL LALK**EGLLVG** **ISSGAAAAAA** **IK**IARR**PENA** **GK**LIVVIFPS
301 FGER**YLSSVL** **FESVKR**EAES MVFEP

Spot No.: **D130 (S197)**

NCBI accession No.: **gi|351727028** Species: *Glycine max*

Protein name: cell division cycle protein 48 homolog

PMF Mascot score: **174** Sequence coverage %: **42**

Matched peptides No.: **37** Total peptides No.: **90**

Calculated Mr: **90512** Calculated *p*I: **5.18**

Probability Based Mowse Score:

Protein score is -10*Log(P), where P is the probability that the observed match is a random event.
Protein scores greater than 73 are significant (p<0.05).

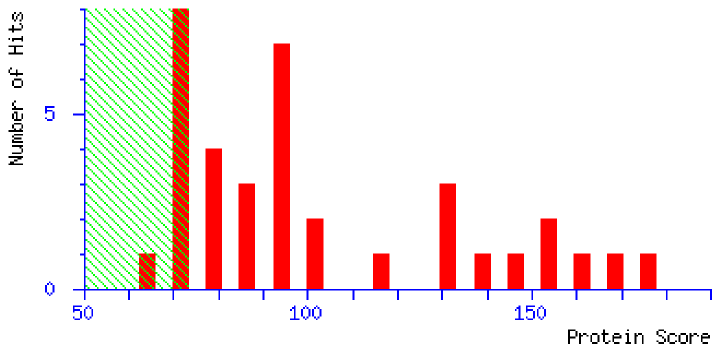

Matched peptide sequences: shown in **Bold Red:**

1	MSQQGESSDP	KSGKK**DFSTA**	**ILER**KKSPNR	LVVDEAVNDD	NSVVTMHPQT
51	MEK**LQLFR**GD	TILIKGKKRK	DTICIALADE	NCEEPKIRMN	KVVRSNLRVR
101	**LGDVVSVHQC**	**PDVK**YGKRVH	ILPIDDTIEG	VTGNLFDAFL	KPYFLEAYRP
151	VR**KGDLFLVR**	GGMRSVEFKV	VETDPGEYCV	VAPDTEIFCE	GEPLKREDEE
201	RLDEVGYDDV	GGVR**KQMAQI**	**RELVELPLRH**	**PQLFK**SIGVK	PPK**GILLYGP**
251	**PGSGK**TLIAR	AVANETGAFF	FCINGPEIMS	K**LAGESESNL**	**R**KAFEEAEKN
301	APSIIFIDEI	DSIAPKREK**T**	**HGEVERRIVS**	**QLLTLMDGLK**	**SRAHVIVIGA**
351	**TNRPNSIDPA**	**LRRFGRFDRE**	**IDIGVPDEVG**	**RLEVLR**IHTK	NMKLSDDVDL
401	ERIAK**DTHGY**	**VGADLAALCT**	**EAALQCIR**EK	MDVIDLEDET	IDAEVLNSMA
451	VTNEHFQTAL	GTSNPSALRE	**TVVEVPNVSW**	**EDIGGLENVK**	**RELQETVQYP**
501	**VEHPEKFEK**F	GMSPSK**GVLF**	**YGPPGCGK**TL	**LAKAIANECQ**	**ANFISVKGPE**
551	**LLTMWFGESE**	**ANVR**EIFDKA	R**QSAPCVLFF**	**DELDSIATQR**	**GSSVGDAGGA**
601	**ADRVLNQLLT**	**EMDGMSAK**KT	VFIIGATNRP	DIIDPALLRP	GR**LDQLIYIP**
651	**LPDEDSRHQI**	**FK**ACLRKSPI	AKNVDLRALA	RHTQGFSGAD	ITEICQRACK
701	YAIRENIEKD	IERERKSREN	PEAMDEDTVD	DEVAEIKAAH	FEESMKFARR
751	**SVSDADIRKY**	**QAFAQTLQQS**	RGFGSEFRFP	ESGDRTTTGS	DPFAASAGGA
801	DEDDLYS				

Spot No.: **D131 (S218)**

NCBI accession No.: gi|14916100   Species: *Hevea brasiliensis*

PFF score: [802]

Protein name： hydroxymethylglutaryl coenzyme A

Matched peptides No.: [10]   Sequence coverage %: [35]

Matched sequences: R.LVFNDFVR.N;K.ARHELPPEK.F;
K.TFLMQIFEK.F + Oxidation (M);
K.VSQQVAKPLYDAK.V;K.LAPFSNLSGDESYQNR.D;
R.YGLVVCTDSAVYAEGPAR.P;
K.QFSISDAEYFVFHSPYNK.L;
K.DCSLLASGTYYLTEVDSLYR.R;
R.GSHMSHAYDFYKPNLASEYPVVDGK.L + Oxidation (M);
K.NVGILAVDIYFPPTFVQQEALEAHDGASK.G

Calculated Mr: **51761**   Calculated *p*I: **6.86**

Probability Based Mowse Score:

Ions score is -10*Log(P), where P is the probability that the observed match is a random event.
Individual ions scores > 45 indicate identity or extensive homology (p<0.05).
Protein scores are derived from ions scores as a non-probabilistic basis for ranking protein hits.

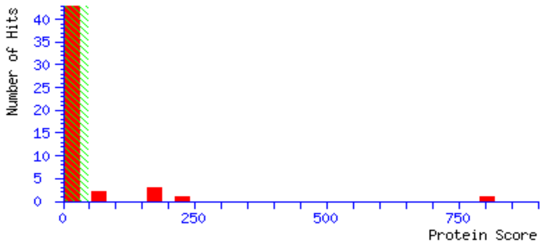

Matched peptide sequences: shown in Bold Red:

```
  1 MAKNVGILAV DIYFPPTFVQ QEALEAHDGA SKGKYTIGLG QDCMAFCTEV
 51 EDVISMSLTA VTSLLDKYNI DPKQIGRLEV GSETVIDKSK SIKTFLMQIF
101 EKFGNTDIEG VDSTNACYGG TAALFNCVNW VESSSWDGRY GLVVCTDSAV
151 YAEGPARPTG GAAAIAILVG PDAPIAFESK FRGSHMSHAY DFYKPNLASE
201 YPVVDGKLSQ TCYLMALDSC YKHFCAKYEK FEGKQFSISD AEYFVFHSPY
251 NKLVQKSFAR LVFNDFVRNA RSIDETAKEK LAPFSNLSGD ESYQNRDLEK
301 VSQQVAKPLY DAKVKPTTLI PKQVGNMYTA SLYAAFASLL HSKHTELAGK
351 RVTLFSYGSG LTATMFSLRL HEGQHPFSLS NIASVMNVAG KLKARHELPP
401 EKFVNIMKLM EHRYGAKDFV RSKDCSLLAS GTYYLTEVDS LYRRFYAQKA
451 VGNTVENGLL ANGH
```

Spot No.: **D132 (S221)**

NCBI accession No.: **gi|357514973** Species: *Medicago truncatula*

PFF score: [99]

Protein name: **Annexin D4**

Matched peptides No.: [2] Sequence coverage %: [3]

Matched sequences: K.LLVALVSAYR.Y;

R.KLLVALVSAYR.Y

Calculated Mr: **36283** Calculated *p*I: **8.64**

Probability Based Mowse Score:

Ions score is -10*Log(P), where P is the probability that the observed match is a random event.
Individual ions scores > 45 indicate identity or extensive homology (p<0.05).
Protein scores are derived from ions scores as a non-probabilistic basis for ranking protein hits.

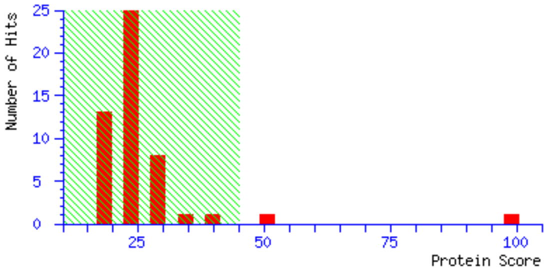

Matched peptide sequences: shown in Bold Red:

```
  1 MAFNQELEAI TQAFSGHGVD EKSLIAVLGK WDPLERETYR KKTSHFFIED
 51 HERQFQRWND HCVRLLKHEF VRFKNAVVLW SMHPWERDAR LAKEALKKGS
101 ISYGVLIEIA CTRSSEELLG ARKAYHSLFD HSIEEDVASH IHGNDRKLLV
151 ALVSAYRYEG TKVKDDTAKS EAKTLSNAIK NAQNKPIVED DEVIRILATR
201 SKLHLQAVYK HYKEISGKNL EEDLNDLRFK ETVQCLCTPQ VYFSKVLDAA
251 LKNDVNKNIK KSLTRVIVTR ADIDMKEIKA EYNNLYGVSL PQKIEETAKG
301 NYKDFLLTLI ARGG
```

Spot No.: **D133 (S222)**

NCBI accession No.: gi|21536853 Species: *Arabidopsis thaliana*

PFF score: [314]

Protein name： phosphoglycerate kinase, putative

Matched peptides No.: [6] Sequence coverage %: [17]

Matched sequences: K.YSLKPLVPR.L;
K.ELDYLVGAVANPK.K;
K.YLKPSVAGFLMQK.E + Oxidation (M);
K.GVSLLLPTDVVIADK.F;
K.LAALADVYVNDAFGTAHR.A;
K.KLAALADVYVNDAFGTAHR.A

Calculated Mr: **42178** Calculated *p*I: **5.49**

Probability Based Mowse Score:

Ions score is -10*Log(P), where P is the probability that the observed match is a random event.
Individual ions scores > 45 indicate identity or extensive homology (p<0.05).
Protein scores are derived from ions scores as a non-probabilistic basis for ranking protein hits.

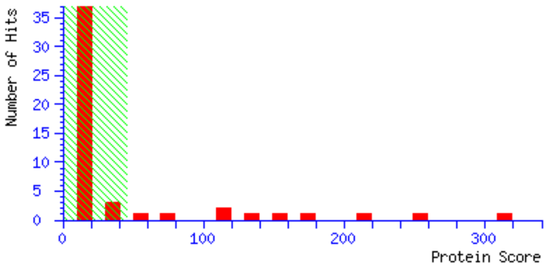

Matched peptide sequences: shown in Bold Red:

```
  1 MATKRSVGTL KEADLKGKSV FVRVDLNVPL DDNSNITDDT RIRAAVPTIK
 51 YLMGNGSRVV LCSHLGRPKG VTPKYSLKPL VPRLSELLGV EVVMANDSIG
101 EEVQKLVAGL PEGGVLLLEN VRFYAEEEKN DPEFAKKLAA LADVYVNDAF
151 GTAHRAHAST EGVAKYLKPS VAGFLMQKEL DYLVGAVANP KKPFAAIVGG
201 SKVSTKIGVI ESLLNTVDIL LLGGGMIFTF YKAQGLSVGS SLVEEDKLDL
251 AKSLMEKAKA KGVSLLLPTD VVIADKFAPD ANSKIVPATA IPDGWMGLDI
301 GPDSIKTFSE ALDTTKTIIW NGPMGVFEFD KFAAGTEAVA KQLAELSGKG
351 VTTIIGGGDS VAAVEKVGLA DKMSHISTGG GASLELIEGK PLPGVLALDE
401 A
```

Spot No.: **D134 (S223)**

NCBI accession No.: gi|255547137 Species: *Ricinus communis*

PFF score: [77]

Protein name: Alpha-1,4-glucan-protein synthase

Matched peptides No.: [2] Sequence coverage %: [5]

Matched sequences: K.DINALEQHIK.N;

K.VICDHLGFGVK.T

Calculated Mr: **41557** Calculated *p*I: **5.82**

Probability Based Mowse Score:

Ions score is -10*Log(P), where P is the probability that the observed match is a random event.
Individual ions scores > 46 indicate identity or extensive homology (p<0.05).
Protein scores are derived from ions scores as a non-probabilistic basis for ranking protein hits.

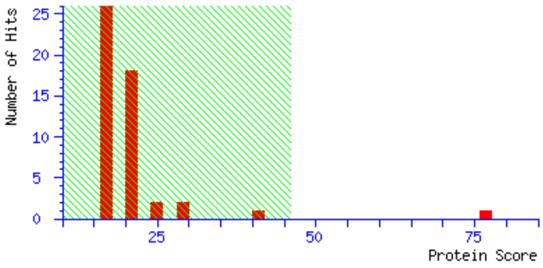

Matched peptide sequences: shown in Bold Red:

```
  1  MADFAAISPT  PLLKDELDIV  IPTIRNLDFL  EMWRPFFQPY  HLIIVQDGDP
 51  SKIIKVPEGF  DYELYNRNDI  NRILGPKASC  ISFKDSACRC  FGYMVSKKKY
101  IYTIDDDCFV  AKDPSGKDIN  ALEQHIKNLL  CPSTPFFFNT  LYDPYRTGAD
151  FVRGYPFSLR  EGVPTAVSHG  LWLNIPDYDA  PTQLVKPLER  NTRYVDAVMT
201  IPKGTLFPMC  GMNLAFNREL  IGPAMYFGLM  GDGQPIGRYD  DMWAGWCTKV
251  ICDHLGFGVK  TGLPYIYHSK  ASNPFVNLKK  EYKGIYWQEE  LIPFFQSAVL
301  PKECTTVQKC  YIELSKQVRA  KLGKIDEYFV  KLADAMVTWV  EAWDELNPSG
351  NSGELPNGAA  K
```

Spot No.: **D135 (S227)**

NCBI accession No.: gi|333690873 Species: *Hevea brasiliensis*

PFF score: [386]

Protein name：glutathione-S-transferase tau 1

Matched peptides No.: [8] Sequence coverage %: [29]

Matched sequences: R.FWADFIDK.K;

K.VYEFVLVLK.K;
R.FWADFIDKK.I;
K.YEYREEDLR.N;
K.SPFLPSDPYQR.A;
K.SPLLLQMNPVHK.K + Oxidation (M);
K.DKSPFLPSDPYQR.A;
K.ESVSESVPDPEKVYEFVLVLK.K

Calculated Mr: **25595** Calculated *p*I: **5.48**

Probability Based Mowse Score:

Ions score is -10*Log(P), where P is the probability that the observed match is a random event.
Individual ions scores > 46 indicate identity or extensive homology (p<0.05).
Protein scores are derived from ions scores as a non-probabilistic basis for ranking protein hits.

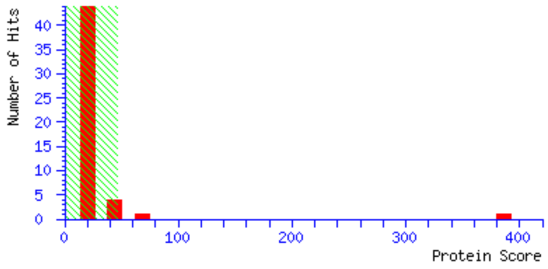

Matched peptide sequences: shown in Bold Red:

```
  1 MAEEVILLDF WPSPFGMRVR IALAEKGVKY EYREEDLRNK SPLLLQMNPV
 51 HKKIPVLIHN GKPICESLIA VQYVDEVWKD KSPFLPSDPY QRAQARFWAD
101 FIDKKIYDIG RKIWTTKGDE QEAAKKEFIE ALKLLEGELG NKPYFGGESM
151 GYVDVALIPF YSWFYAYETC GNFSIEPECP VLIAWAKRCL QKESVSESVP
201 DPEKVYEFVL VLKKKFGIE
```

Spot No.: **D136 (S230)**

NCBI accession No.: gi|2970051   Species: *Vigna radiata*

PFF score: [104]

Protein name： ARG10

Matched peptides No.: [2]   Sequence coverage %: [12]

Matched sequences: K.SANEVILVIEAYK.A;

K.ITAVPAEEEEIWGATFK.V

Calculated Mr: **25750**   Calculated *p*I: **5.62**

Probability Based Mowse Score:

Ions score is -10*Log(P), where P is the probability that the observed match is a random event.
Individual ions scores > 46 indicate identity or extensive homology (p<0.05).
Protein scores are derived from ions scores as a non-probabilistic basis for ranking protein hits.

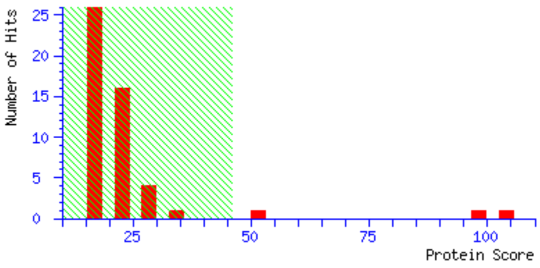

Matched peptide sequences: shown in Bold Red:

```
  1 MLGIFSSSVV SPPEELVAAG SRTPSPKTTA GKLLTRFVES KASAVSLQVG
 51 EHVQLAYTHH SESPWYPRSF AVKDEIFCLF EGALDNLGSL RQQYGLAKSA
101 NEVILVIEAY KALRDRAPYP ANRVVCHLSG SFAFIVFDKS TSTVFVASDQ
151 AGKVPLYWGI TADGYVAFAD DADLLKGSCG KSLASFPQGC FYSTAVGGLR
201 CYENPKNKIT AVPAEEEEIW GATFKVEGSA VLAATEY
```

Spot No.: **D137 (S231)**

NCBI accession No.: gi|2832430 Species: *Hevea brasiliensis*

PFF score: [217]

Protein name： prohevein

Matched peptides No.: [2] Sequence coverage %: [20]

Matched sequences: R.IVDQCSNGGLDLDVNVFR.Q;

K.YGWTAFCGPVGAHGQPSCGK.C

Calculated Mr: **20852** Calculated p*I*: **5.64**

Probability Based Mowse Score:

Ions score is -10*Log(P), where P is the probability that the observed match is a random event.
Individual ions scores > 45 indicate identity or extensive homology (p<0.05).
Protein scores are derived from ions scores as a non-probabilistic basis for ranking protein hits.

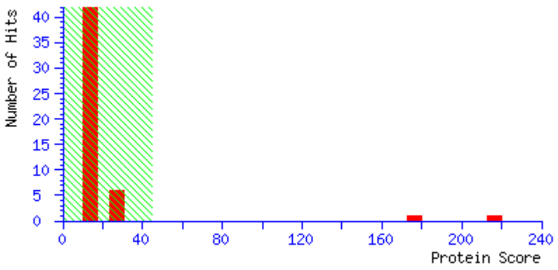

Matched peptide sequences: shown in Bold Red:

```
  1 EQCGRQAGGK LCPNNLCCSQ WGWCGSTDEY CSPDHNCQSN CKDSGEGVGG
 51 GSASNVLATY HLYNSQDHGW DLNAASAYCS TWDANKPYSW RSKYGWTAFC
101 GPVGAHGQPS CGKCLSVTNT GTGAKATVRI VDQCSNGGLD LDVNVFRQLD
151 TDGKGYERGH LTVNYQFVDC GDSFNPLFSV MKSSVIN
```

Spot No.: **D138 (S233)**

NCBI accession No.: gi|14423933 Species: *Hevea brasiliensis*

PFF score: [384]

Protein name： Small rubber particle protein

Matched peptides No.: [5] Sequence coverage %: [36]

Matched sequences: K.AEQYAVITWR.A;
R.VSSYLPLLPTEK.I;
K.QVSAQTYSVAQDAPR.I;
K.DISGPLKPGVDTIENVVK.T;
R.IVLDVASSVFNTGVQEGAK.A

Calculated Mr: **22331** Calculated *p*I: **4.80**

Probability Based Mowse Score:

Ions score is -10*Log(P), where P is the probability that the observed match is a random event.
Individual ions scores > 45 indicate identity or extensive homology (p<0.05).
Protein scores are derived from ions scores as a non-probabilistic basis for ranking protein hits.

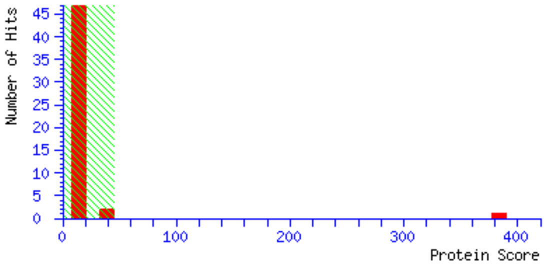

Matched peptide sequences: shown in **Bold Red:**

```
  1 MAEEVEEERL KYLDFVRAAG VYAVDSFSTL YLYAKDISGP LKPGVDTIEN
 51 VVKTVVTPVY YIPLEAVKFV DKTVDVSVTS LDGVVPPVIK QVSAQTYSVA
101 QDAPRIVLDV ASSVFNTGVQ EGAKALYANL EPKAEQYAVI TWRALNKLPL
151 VPQVANVVVP TAVYFSEKYN DVVRGTTEQG YRVSSYLPLL PTEKITKVFG
201 DEAS
```

Spot No.: **D139 (S234)**

NCBI accession No.: gi|132270 Species: *Hevea brasiliensis*

PFF score: [379]

Protein name： Rubber elongation factor protein

Matched peptides No.: [4] Sequence coverage %: [38]

Matched sequences: K.NVAVPLYNR.F;

K.DASIQVVSAIR.A;
K.FVDSTVVASVTIIDR.S;
K.DKSGPLQPGVDIIEGPVK.N

Calculated Mr: **14713** Calculated *p*I: **5.04**

Probability Based Mowse Score:

Ions score is -10*Log(P), where P is the probability that the observed match is a random event.
Individual ions scores > 45 indicate identity or extensive homology (p<0.05).
Protein scores are derived from ions scores as a non-probabilistic basis for ranking protein hits.

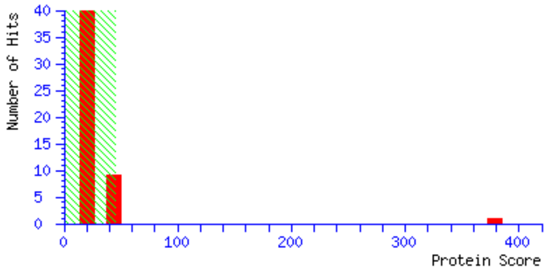

Matched peptide sequences: shown in Bold Red:

```
  1 MAEDEDNQQG QGEGLKYLGF VQDAATYAVT TFSNVYLFAK DKSGPLQPGV
 51 DIIEGPVKNV AVPLYNRFSY IPNGALKFVD STVVASVTII DRSLPPIVKD
101 ASIQVVSAIR AAPEAARSLA SSLPGQTKIL AKVFYGEN
```

Spot No.: **D140 (S238)**

NCBI accession No.: gi|2832430 Species: *Hevea brasiliensis*

PFF score: [344]

Protein name： prohevein

Matched peptides No.: [3] Sequence coverage %: [26]

Matched sequences: R.QLDTDGKGYER.G;

R.IVDQCSNGGLDLDVNVFR.Q;

K.YGWTAFCGPVGAHGQPSCGK.C

Calculated Mr: **20852** Calculated *p*I: **5.64**

Probability Based Mowse Score:

Ions score is -10*Log(P), where P is the probability that the observed match is a random event.
Individual ions scores > 45 indicate identity or extensive homology (p<0.05).
Protein scores are derived from ions scores as a non-probabilistic basis for ranking protein hits.

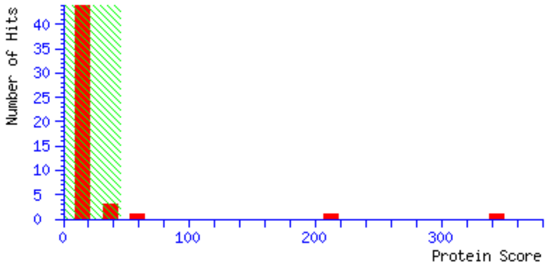

Matched peptide sequences: shown in Bold Red:

```
  1 EQCGRQAGGK LCPNNLCCSQ WGWCGSTDEY CSPDHNCQSN CKDSGEGVGG
 51 GSASNVLATY HLYNSQDHGW DLNAASAYCS TWDANKPYSW RSKYGWTAFC
101 GPVGAHGQPS CGKCLSVTNT GTGAKATVRI VDQCSNGGLD LDVNVFRQLD
151 TDGKGYERGH LTVNYQFVDC GDSFNPLFSV MKSSVIN
```

Spot No.: **D141 (S247)**

NCBI accession No.: **gi|123062** Species: *Hevea brasiliensis*

PFF score: [158]

Protein name: Pro-hevein

Matched peptides No.: [2] Sequence coverage %: [11]

Matched sequences: K.CLSVTNTGTGAK.T;
R.QLDTDGKGYER.G

Calculated Mr: **22700** Calculated *p*I: **5.63**

Probability Based Mowse Score:

Ions score is -10*Log(P), where P is the probability that the observed match is a random event.
Individual ions scores > 45 indicate identity or extensive homology (p<0.05).
Protein scores are derived from ions scores as a non-probabilistic basis for ranking protein hits.

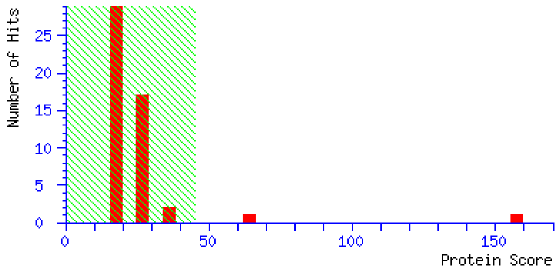

Matched peptide sequences: shown in Bold Red:

```
  1 MNIFIVVLLC LTGVAIAEQC GRQAGGKLCP NNLCCSQWGW CGSTDEYCSP
 51 DHNCQSNCKD SGEGVGGGSA SNVLATYHLY NSQDHGWDLN AASAYCSTWD
101 ANKPYSWRSK YGWTAFCGPV GAHGQSSCGK CLSVTNTGTG AKTTVRIVDQ
151 CSNGGLDLDV NVFRQLDTDG KGYERGHITV NYQFVDCGDS FNPLFSVMKS
201 SVIN
```

Spot No.: **D142 (S249)**

NCBI accession No.: **gi|132270** Species: *Hevea brasiliensis*

PFF score: [405]

Protein name: Rubber elongation factor protein

Matched peptides No.: [4] Sequence coverage %: [38]

Matched sequences: K.NVAVPLYNR.F;
K.DASIQVVSAIR.A;
K.FVDSTVVASVTIIDR.S;
K.DKSGPLQPGVDIIEGPVK.N

Calculated Mr: **14713** Calculated *p*I: **5.04**

Probability Based Mowse Score:

Ions score is -10*Log(P), where P is the probability that the observed match is a random event.
Individual ions scores > 46 indicate identity or extensive homology (p<0.05).
Protein scores are derived from ions scores as a non-probabilistic basis for ranking protein hits.

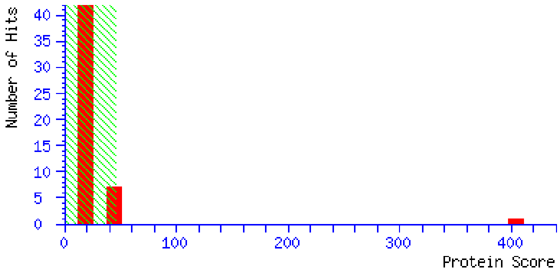

Matched peptide sequences: shown in Bold Red:

```
  1 MAEDEDNQQG QGEGLKYLGF VQDAATYAVT TFSNVYLFAK DKSGPLQPGV
 51 DIIEGPVKNV AVPLYNRFSY IPNGALKFVD STVVASVTII DRSLPPIVKD
101 ASIQVVSAIR AAPEAARSLA SSLPGQTKIL AKVFYGEN
```

Spot No.: **D143 (S251)**

NCBI accession No.: **gi|132270**   Species: *Hevea brasiliensis*

PFF score: [470]

Protein name: Rubber elongation factor protein

Matched peptides No.: [4]   Sequence coverage %: [27]

Matched sequences: K.NVAVPLYNR.F;
K.DASIQVVSAIR.A;
K.SGPLQPGVDIIEGPVK.N;
K.DKSGPLQPGVDIIEGPVK.N

Calculated Mr: **14713**   Calculated *p*I: **5.04**

Probability Based Mowse Score:

Ions score is -10*Log(P), where P is the probability that the observed match is a random event.
Individual ions scores > 46 indicate identity or extensive homology (p<0.05).
Protein scores are derived from ions scores as a non-probabilistic basis for ranking protein hits.

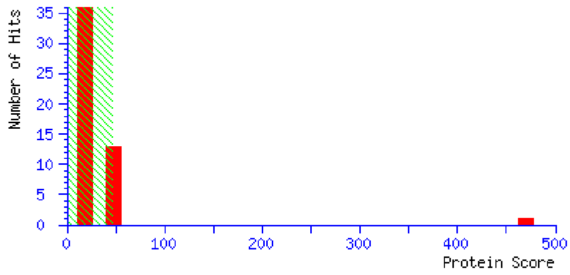

Matched peptide sequences: shown in Bold Red:

```
  1 MAEDEDNQQG QGEGLKYLGF VQDAATYAVT TFSNVYLFAK DKSGPLQPGV
 51 DIIEGPVKNV AVPLYNRFSY IPNGALKFVD STVVASVTII DRSLPPIVKD
101 ASIQVVSAIR AAPEAARSLA SSLPGQTKIL AKVFYGEN
```

Supplementary Figure S4

Identification of ethylene responsive latex proteins by iTRAQ

1. Labeled samples:

117: E3TL (total latex)

116: E5TL

115: D3TL

114: D5TL

Needed values: 117: 115= E3TL: D3TL

116: 114= E5TL: D5TL

2.

14. Details of All Protein Summary for Rubber Latex _iTRAQ 4 plex

The 405 different proteins identified by ITRAQ.

Protein No.: I-2

Protein name and Species:

Beta-1,3-glucanase form 'RRII Gln 2' OS=Hevea brasiliensis PE=3 SV=1

Accession: Tr|D1M8S7|D1M8S7_HEVBR

Unused ProtScore: 66.5 **Seq Cov %: 67.1**

ITRAQ Changed Ratio:

117: 115=E3TL: D3TL=0.38

116: 114=E5TL: D5TL=0.57

Annotated typical Precursor MS Region:

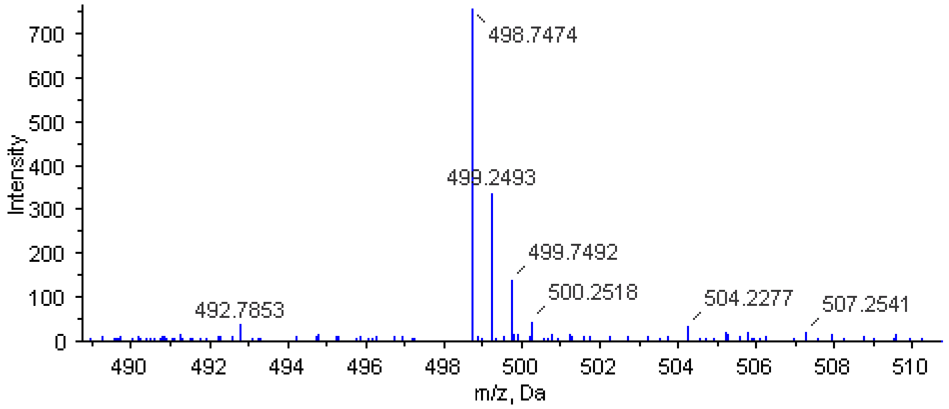

Sequence Coverage and Matched peptides: in different color

MAICSSTSGTSSSLPSRTTVMLLLFFFTASVG**ITDAQVGVCYGMQGNNLPPVSEVIALYK**KSNIT**RMRI**
YDPNQAVLEALRGSNIELILGVPNSDLQSLTNPSNAKSWVQKNVRGFWSSVR**FRYIAVGNEISPVNRGT**
AWLAQFVLPAMRNIHDAIRSAGLQDQIKVSTAIDLTLVGNSYPPSAGAFRDDVR**SYLNPIIR**FLSSIR**S**
PLLANIYPYFTYAGNPRDISLPYALFTSPSVVVWDGQRGYK**NLFDATLDALYSALER**ASGGSLEVVVSE
SGWPSAGAF**AATFDNGRTYLSNLIQHVKR**GTPKRPKR**AIETYLFAMFDENK**KQPEVEK**HFGLFFPNK**WQ
K**YNLNFSAEK**NWDISTEHNATILFLKSDM

Protein No.: I-5

Protein name and Species:

Major latex allergen Hev b 5 OS=Hevea brasiliensis PE=1 SV=3

Accession:

sp|Q39967|ALL5_HEVBR

Unused ProtScore: 58.21 **Seq Cov %: 95.4**

ITRAQ Changed Ratio:

117: 115=E3TL: D3TL=3.25

116: 114=E5TL: D5TL=1.54

Annotated typical Precursor MS Region:

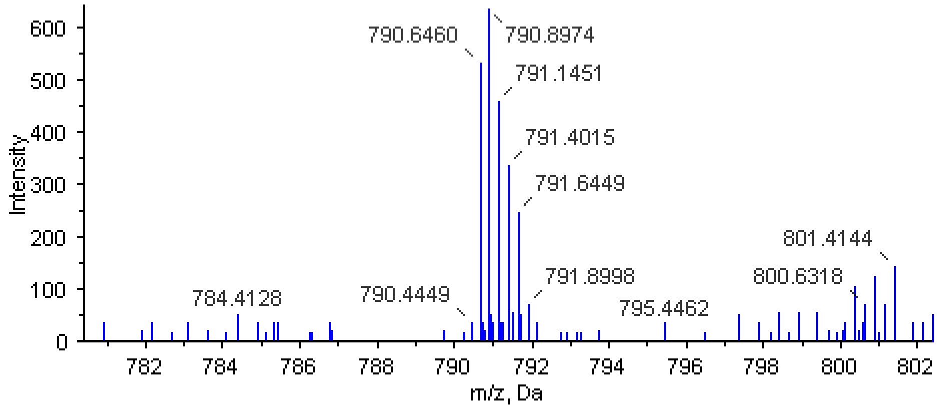

Sequence Coverage and Matched peptides: in different color

MASVEVESAATALPKNETPEVTKAEETKTEEPAAPPASEQETADATPEKEEPTAAPAEPEAPAPETEKA
EEVEKIEKTEEPAPEADQTTPEEKPAEPEPVAEEEPKHETKETETEAPAAPAEGEKPAEEEKPITEAAE
TATTEVPVEKTEE

Protein No.: I-7

Protein name and Species:

Latex allergen hev b 7.02 (Fragment) OS=Hevea brasiliensis PE=2 SV=1

Accession: tr|Q706V4|Q706V4_HEVBR

Unused ProtScore: 54.77

Seq Cov %: 71.6

ITRAQ Changed Ratio:

117: 115=E3TL: D3TL=0.33

116: 114=E5TL: D5TL=0.30

Annotated typical Precursor MS Region:

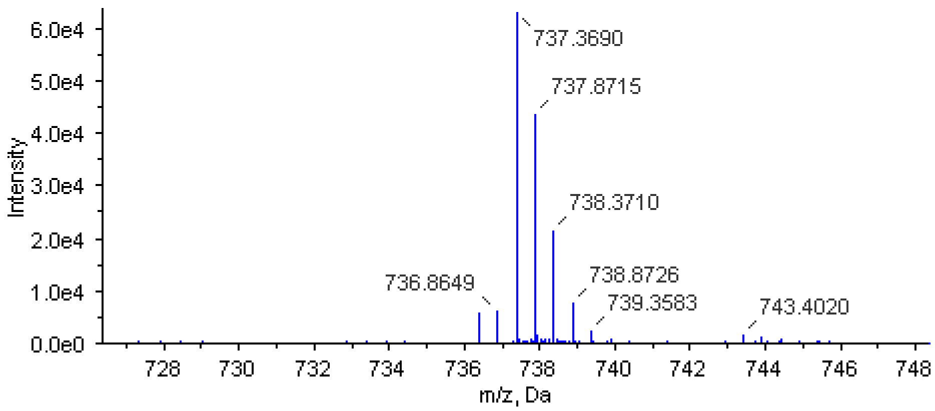

Sequence Coverage and Matched peptides: in different color

ATGSTTLTQGKKITVLSIDGGGIRGIIPGIILASLESKLQDLDGPDARIADYFDIIAGTSTGGLITTML
TAPNEDKKPMYQAKDIKDFYLENCPKIFPKESR**DNYDPIHSIGPIYDGEYLRELCNNLLK**DLTV**KDTLT**
DVIIPTFDIKLLLPVIFSSDDAKCNALKNAR**LADVCISTSAAPVLLPAHSFTTEDDK**NIH**TFELIDGGV**
AATNPTLLALTHIRNEIIRQNPR**FIGANLTESK**SR**LVLSLGTGK**SEYKEK**YNADMTSK**WRLYNWALYNG
NSPAVDIFSNASSDMVDFHLSALFK**SLDCEDYYLRIQDDTLTGEESSGHIATEENLQR**LVEIGTRLLEK
QESR**INLDTGRLESIPGASTNEAAITK**FAKLLSEERKLRQLK

Protein No.: I-10

Protein name and Species:

Actin OS=Morus alba PE=3 SV=2

Accession:

tr|E5FR85|E5FR85_MORAL

Unused ProtScore: **44.55**

Seq Cov %: **56.2**

ITRAQ Changed Ratio:

117: 115=E3TL: D3TL=0.39

116: 114=E5TL: D5TL=0.75

Annotated typical Precursor MS Region:

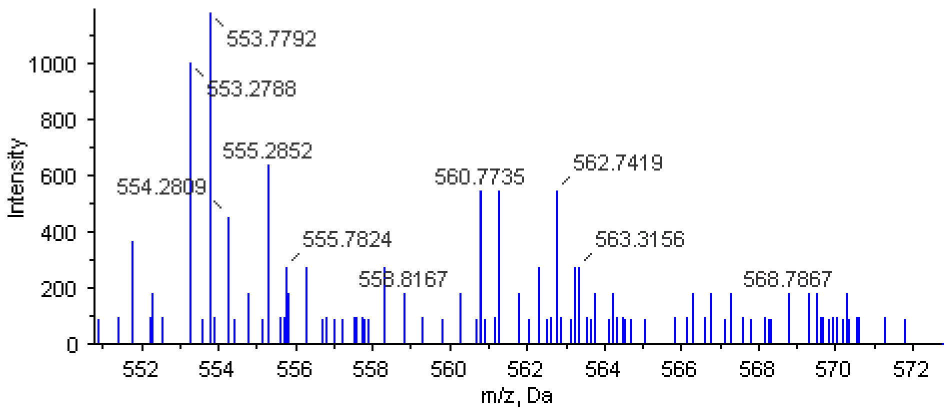

Sequence Coverage and Matched peptides: in different color

MADAEDIQPLVCDNGTGMVK**AGFAGDDAPRAVFPSIVGRPRHTGVMVGMGQKDAYVGDEAQSK**RGILTL
K**YPIEHGIVSNWDDMEK**IWHHTFYNELR**VAPEEHPVLLTEAPLNPK**ANREKMTQIMFETFNVPAMYVAI
QAVLSLYASGR**TTGIVLDSGDGVSHTVPIYEGYALPHAILR**LDLAGR**DLTDALMK**ILTER**GYMFTTTAE**
REIVRDMKGK**LAYVALDYEQELETAK**SGSSVEK**NYELPDGQVITIGAER**YRCPQVLFQPSLIGMEAAGI
HETTYNSIMKCDVDIRK**DLYGNIVLSGGSTMFPGIADR**MSK**EITALAPSSMKIKVVAPPERK**YSVWIGG
SILASLSTFQQMWISK**GEYDESGPSIVHR**KCF

Protein No.: I-11

Protein name and Species:

Heat shock protein OS=Hevea brasiliensis PE=2 SV=1

Accession: tr|Q71EE1|Q71EE1_HEVBR

Unused ProtScore: 43.62

Seq Cov %: 38.3

ITRAQ Changed Ratio:

117: 115=E3TL: D3TL=1.58

116: 114=E5TL: D5TL=1.42

Annotated typical Precursor MS Region:

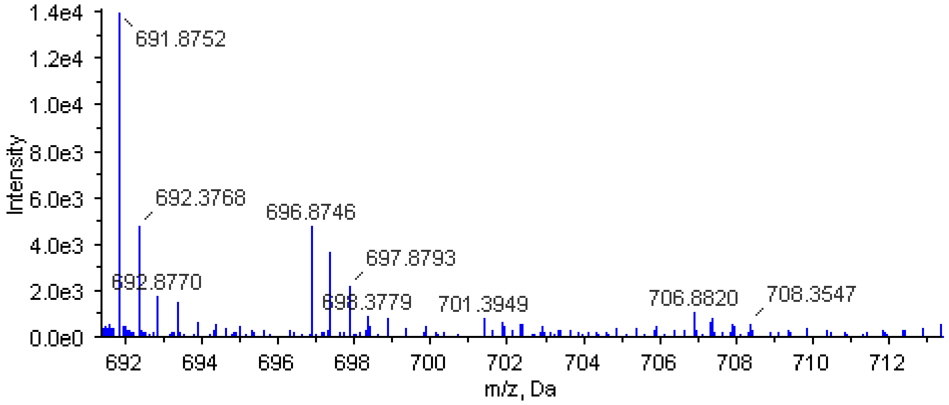

Sequence Coverage and Matched peptides: in different color

MADAETFAFQAEINQLLSLIINTFYSNKEIFLR**EIISNASDALDKIRFESLTDKSKLDAQPELFIR**LVP
DKANK**TLSIIDSGIGMTKADLVNNLGTIAR**SGTKEFMEALQAGADVSMIGQFGVGFYSAYLVAEKVIVT
TKHNDDEQYIWESQAGGSFTVTRDVNGDQLGRGTK**ITLFLKEDQLEYLEERR**IKDLVKKHSEFISYPIY
LWTEKTTEKEISDDEDDEPKKEEEGDVEDVDEEKETKSKKKKIKEVSHEWQLINKQKPIWLRKPEEITK
EEYASFYKSLTNDWEDHLAVKHFSVEGQLEFKAILFVPK**RAPFDLFDTR**KKMSNIKLYVRR**VFIMDNCE**
ELIPEYLGFVKGVVDSDDLPLNISREMLQQNKILKVIRKNLVKK**CIEMFNEIAENK**EDYNK**FYEAFSK**N
LK**LGIHEDSQNR**AK**LADLLR**YHSTK**SGDEMTSLKDYVTR**MKEGQK**DIYYITGESKKAVENSPFLER**LKK
KGYEVLF**MVDAIDEYAVGQLK**EYDGKK**LVSATK**EGLK**LDDETEEEK**KKKEEKKK**SFENLCK**TIK**DILGD**
KVEK**VVVSDRIVDSPCCLVTGEYGWTANMER**IMKAQALRDSSMSSYMSSKK**TMEINPDNVIVEELR**KRA
EADKNDKSVKDLVLLLFETALLTSGFSLDDPNTFSARIHRMLK**LGLSIDDDETAGDDADMPALEEDGAE**
ESKMEEVD

Protein No.: I-12

Protein name and Species:

50 kDa protein; Major latex allergen Hev b 4 OS=Hevea brasiliensis PE=2 SV=1

Accession: tr|Q5VLJ6|Q5VLJ6_HEVBR

Unused ProtScore: 42.03

Seq Cov %: 35.4

ITRAQ Changed Ratio:

117: 115=E3TL: D3TL=0.46

116: 114=E5TL: D5TL=0.56

Annotated typical Precursor MS Region:

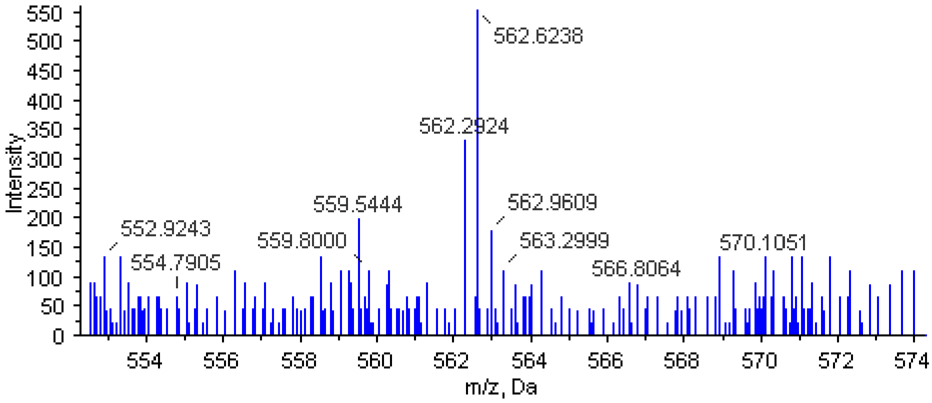

Sequence Coverage and Matched peptides: in different color

MAGKGEGPAIGIDLGTTYSCVGVWQHDRVEIIANDQGNRTTPSYVGFTDTERLIGDAAKNQVAMNPINT
VFDAKRLIGRRFTDASVQSDIKLWPFKVFSGPGDKPMIQVNYKGEDKQFSAEEISSMVLMKMREIAEAY
LGTTVKNAVVTVPAYFNDSQRQATKDAGVIAGLNVMRIINEPTAAAIAYGLDKKATSVGEKNVLIFDLG
GGTFDVSLLTIEEGIFEVKATAGDTHLGGEDFDNRMVNHFVQEFKRKNKKDISGNPRALRRLRTACERA
KRTLSSTAQTTIEIDSLYEGIDFYSTITRARFEELNMDLFRKCMEPVEKCLRDAKMDKRTVHDVVLVGG
STRIPKVQQLLQDFFNGKELCKSINPDEAVAYGAAVQAAILSGEGNEKVQDLLLLDVTPLSLGLETAGG
VMTVLIPRNTTIPTKKEQVFSTYSDNQPGVLIQVYEGERTRTRDNNLLGKFELSGIPPAPRGVPQITVC
FDIDANGILNVSAEDKTTGQKNKITITNDKGRLSKEEIEKMVQEAEKYKSEDEEHKKKVEAKNALENYA
YNMRNTVKDEKIGGKLDPADKKKIEDAVEQTIQWLDSNQLGEADEFEDKMKELESICNPIIAKMYQGGA
GPDMGGGMDDDVPAGGSGAGPKIEEVD

Protein No.: I-13

Protein name and Species:

Acetyl-CoA C-acetyltransferase OS=Hevea brasiliensis GN=HbAACT PE=2 SV=1

Accession: tr|A9ZMZ4|A9ZMZ4_HEVBR

Unused ProtScore: 41.88

Seq Cov %: 76

ITRAQ Changed Ratio:

117: 115=E3TL: D3TL=0.65

116: 114=E5TL: D5TL=0.45

Annotated typical Precursor MS Region:

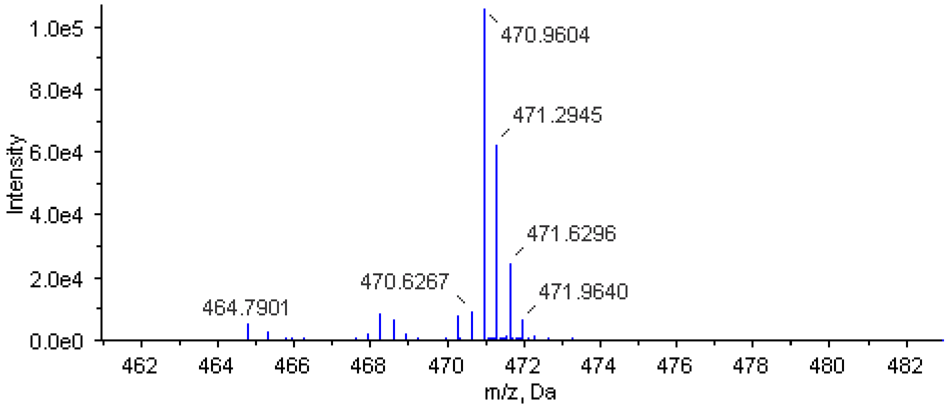

Sequence Coverage and Matched peptides: in different color

MAPVAAAEIKPRDVCIVGVARTPMGGFLGLLSTLPATKLGSIAIEAALKRASVDPSLVQEVFFGNVLSA
NLGQAPARQAALGAGIPNSVVCTTVNKVCASGMKATMLAAQSIQLGINDVVVAGGMESMSNAPKYLAEA
RKGSRLGHDSLVDGMLKDGLWDVYNDVGMGSCAEICADNHSITREDQDKFAIHSFERGIAAQESGAFAW
EIVPVEVSGGRGKPLTIVDKDEGLGKFDPVKLRKLRPSFKENGGTVTAGNASSISDGAAALILVSGETA
LKLGLQVIAKIRGYADAAQAPELFTTAPALAIPKTIANAGLDASQVDYYEINEAFAVVALANQKLLGLN
PEKVNVHGGAVSLGHPLGCSGARILVTLLGVLRKKNAKYGVGGVCNGGGGASALVVELL

Protein No.: I-15

Protein name and Species:

Enolase 1 OS=Hevea brasiliensis GN=ENO1 PE=1 SV=1

Accession: sp|Q9LEJ0|ENO1_HEVBR

Unused ProtScore: 39.75

Seq Cov %: 45.2

ITRAQ Changed Ratio:

117: 115=E3TL: D3TL=0.30

116: 114=E5TL: D5TL=0.37

Annotated typical Precursor MS Region:

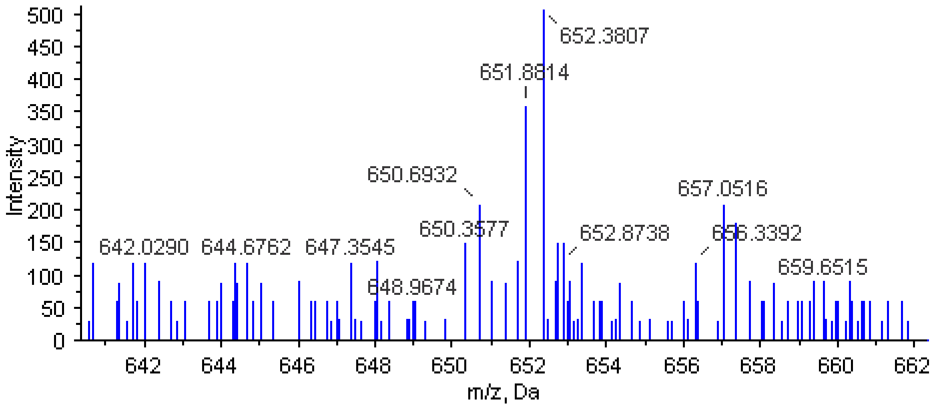

Sequence Coverage and Matched peptides: in different color

M**AITIVSVR**ARQIFDSR**GNPTVEADVKLSDGYLARAAVPSGASTGIYEALELRDGGSDYLGK**GVSK**AVE**
NVNIIIGPALVGKDPTDQVGIDNFMVQQLDGTVNEWGWCKQKLGANAILAVSLAVCK**AGAHVK**GIPLYE
HIANLAGNKNLVLPVPAFNVINGGSHAGNKLAMQEFMILPVGASSFKEAMK**MGAEVYHHLK**SVIKK**KYG**
QDATNVGDEGGFAPNIQENK**EGLELLK**TAIAKAGYTGKVVIGMDVAASEFYGSDQ**TYDLNFKEENNNGS**
QKISGEALKDLYKSFVAEYPIVSIEDPFDQDDWAHYAK**LTSEIGEKVQIVGDDLLVTNPK**RVEKAIKEK
ACNALLLKVNQIGSVTESIEAVK**MSK**R**AGWGVMASHR**SGETEDTFIADLSVGLATGQIKTGAPCRSERL
AKYNQLLR**IEEELGSEAVYAGANFR**KPVEPY

Protein No.: I-17

Protein name and Species:

Latex-abundant protein; caspase; type II metacaspase [Hevea brasiliensis] OS=Hevea brasiliensis GN=LAR PE=2 SV=1

Accession: tr|Q9ZSP8|Q9ZSP8_HEVBR

Unused ProtScore: 37.25

Seq Cov %: 65.5

ITRAQ Changed Ratio:

117: 115=E3TL: D3TL=0.52

116: 114=E5TL: D5TL=0.30

Annotated typical Precursor MS Region:

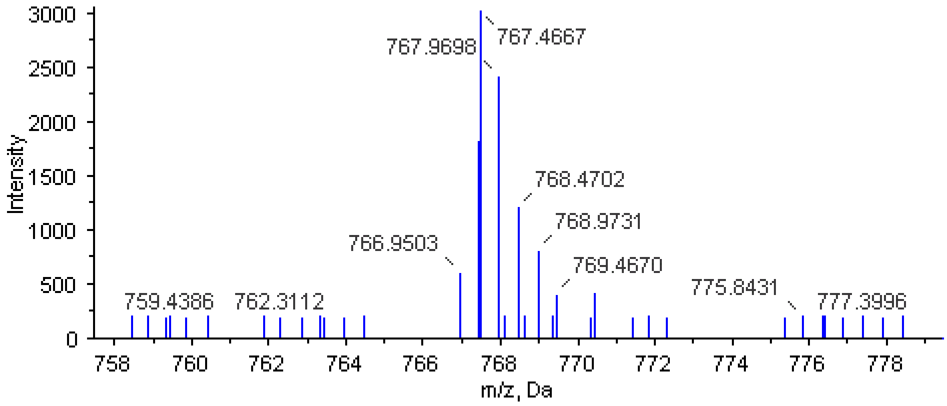

Sequence Coverage and Matched peptides: in different color

MAKKAVLIGINYPGTKAELKGCINDVKRMYRCLVDRYGFSEEDITVLIDIDESYIQPTGKNIRRVLTDL
VRSAEPGDLLFVHYSGHGTRLPAETGEDDDTGFDECIVPCDMNLITDDDFREFVDQVPHGCRITVVSDS
CHSGGLIDEAKEQIGESTKRKEEESESGFGFKSFLKQTIQDAFESRGVHLPSDLHHHHGHRDEEDFDNR
VVEEDYGDSGYVKSKSLPLSTLIEILKQKTGKDDIDVGKLRPTLFDMFGDDASPKVKKFMKVILNKLRH
GDGESGGGGFLGMVGSLAQEFLKHKLDENDESYVKPALETEVDSKQEVYAGKTKRSLPDGGILISGCQT
DQTSADASPSGKSSEAYGALSNAIQTIIAETDGAVTNQELVLKARKMLKKQGFTQKPGLYCSDDHVEAS
FVC

Protein No.: I-18

Protein name and Species:

Mevalonate disphosphate decarboxylase OS=Hevea brasiliensis PE=2 SV=1

Accession: tr|Q944G0|Q944G0_HEVBR

Unused ProtScore: 34.69

Seq Cov %: 43.4

ITRAQ Changed Ratio:

117: 115=E3TL: D3TL=0.49

116: 114=E5TL: D5TL=0.35

Annotated typical Precursor MS Region:

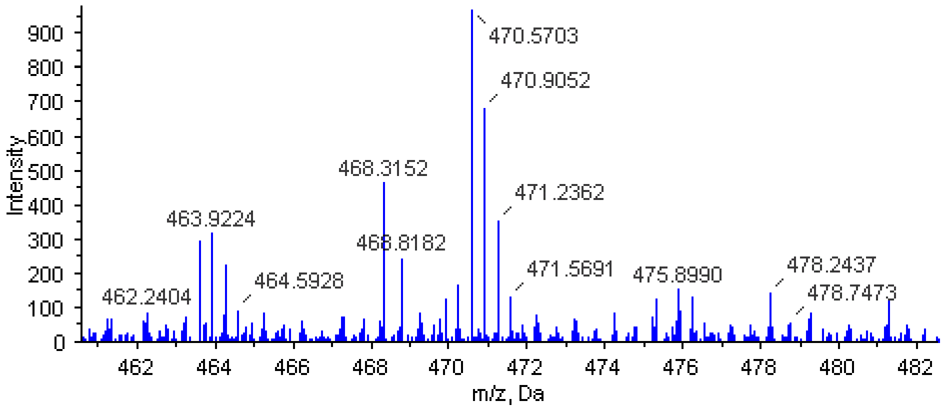

Sequence Coverage and Matched peptides: in different color

MAESWVI**MVTAQTPTNIAVIK**YWGKRDEKLILPVNDSISVTLDPAHLCTTTTVAVSPSFAQDRMWLNGK
EISLSGGRYQNCLREIRAR**ACDVEDKER**GIKISKKDWEKLYVHIASYNNFPTAAGLASSAAGFACLVFA
LAKLMNAK**EDNSELSAIAR**QGSGSACR**SLFGGFVK**WKMGK**VEDGSDSLAVQVVDEK**HWDDLVIIIAVVS
SRQKETSSTTGMR**ETVETSLLLQHR**AKEIVPKR**IVQMEESIK**NRNFASFAHLTCADSNQFHAVCMDTCP
PIFYMNDTSHR**IISCVEK**WNR**SVGTPQVAYTFDAGPNAVLIAHNR**KAAAQLLQK**LLFYFPPNSDTELNS**
YVLGDKSILK**DAGIEDLKDVEALPPPPEIK**DAPR**YKGDVSYFICTRPGQGPVLLSDESQALLSPETGLP**
K

Protein No.: I-19

Protein name and Species:

glyceraldehyde-3-phosphate dehydrogenase， NADP-dependent OS=Pisum sativum GN=GAPN PE=1 SV=1

Accession: sp|P81406|GAPN_PEA

Unused ProtScore: 32.73

Seq Cov %: 46.6

ITRAQ Changed Ratio:

117: 115=E3TL: D3TL=0.50

116: 114=E5TL: D5TL=0.77

Annotated typical Precursor MS Region:

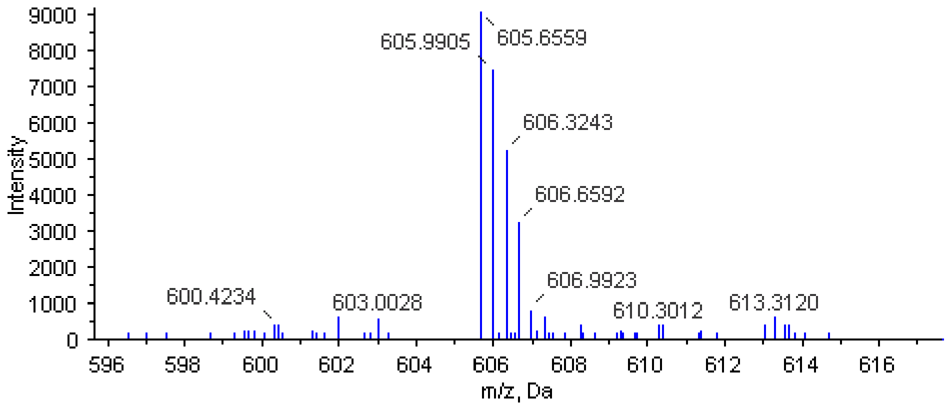

Sequence Coverage and Matched peptides: in different color

MAATGVLAEIIDGDVYKYYADGEWKKSTSGKSVAIINPTTRKPQYKVQACSQEEVNKVMDSAKSAQKSW
AKTPLWKRAELLHKAAAILKEHKAAIAECLVKEIAKPAKDAVTEVVRSGDLVSYCAEEGVRILGEGKFL
VSDSFPGNERTKYCLTSKIPLGVILAIPPFNYPVNLAVSKIAPALIAGNSIVLKPPTQGAVAALHMVHC
FHLAGFPKGLISCVTGKGSEIGDFLTMHPGVNCISFTGGDTGIAISKKSGMIPLQMELGGKDACIVLED
ADLDLVAANIIKGGFSYSGQRCTAVKVVLVMESVADALVEKVKVKVAKLSVGPPEDDSDITPVVSESSA
NFIEGLVNDAKEKGATFCQEYKREGNLIWPLLLDNVRPDMRIAWEEPFGPVLPVIRINSVEEGIHHCNA
SNFGLQGCVFTKDINKAIMISDAMESGTVQINSAPARGPDHFPFQGIKDSGIGSQGITNSINMMTKVKT
TVINLPSPSYTMG

Protein No.: I-22

Protein name and Species:

Hydroxymethylglutaryl coenzyme A synthase OS=Hevea brasiliensis PE=2 SV=1

Accession: tr|Q944F8|Q944F8_HEVBR

Unused ProtScore: 31.1

Seq Cov %: 42.7

ITRAQ Changed Ratio:

117: 115=E3TL: D3TL=0.19

116: 114=E5TL: D5TL=0.44

Annotated typical Precursor MS Region:

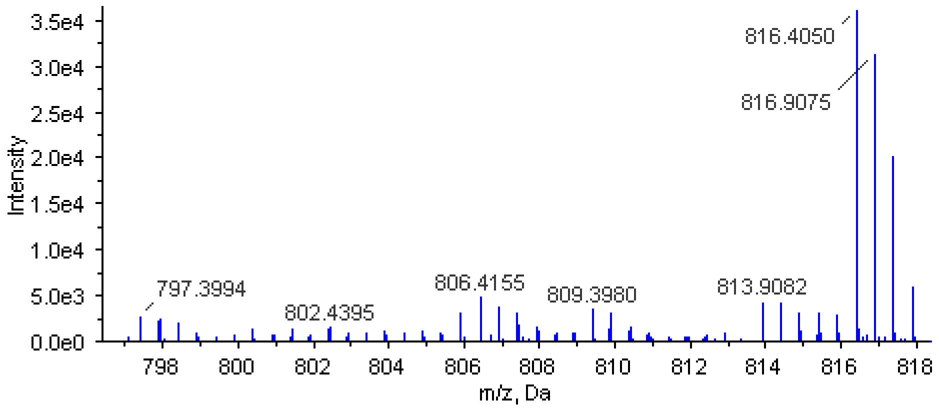

Sequence Coverage and Matched peptides: in different color

MAKNVGILAVDIYFPPTFVQQEALEAHDGASKGKYTIGLGQDCMAFCTEVEDVISMSLTAVTSLLDKYN
IDPKQIGR**LEVGSETVIDK**SKSIK**TFLMQIFEK**FGNTDIEGVDSTNACYGGTAALFNCVNWVESSSWDG
R**YGLVVCTDSAVYAEGPARPTGGAAAIAILVGPDAPIAFESK**FRGSHMSHAYDFYK**PNLASEYPVVDGK**
LSQTCYLMALDSCYKHFCAKYEKFEGKQFSISDAEYFVFHSPYNKLVQKSFAR**LVFNDFVRNASSIDET**
AKEK**LAPFSNLSGDESYQNRDLEKVSQQVAKPLYDAKVKPTTLIPK**QVGNMYTASLYAAFASLLHSKHT
ELAGKRVTLFSYGSGLTATMFSLR**LHEGQHPF****SLSNIASVMNVAGK**LKARHELPPEKFVDIMKLMEHRY
GAKDFVTSK**DCSLLASGTYYLTEVDSLYR**RFYAQK**AVGNTVENGLLANGH**

Protein No.: I-23

Protein name and Species:

Methyl transferase; 5-methyltetrahydropteroyltriglutamate-homocysteine methyltransferase, putative OS=Ricinus communis GN=RCOM_1321820 PE=3 SV=1

Accession: tr|B9SI90|B9SI90_RICCO

Unused ProtScore: 30.18

Seq Cov %: 25.5

ITRAQ Changed Ratio:

117: 115=E3TL: D3TL=2.17

116: 114=E5TL: D5TL=1.57

Annotated typical Precursor MS Region:

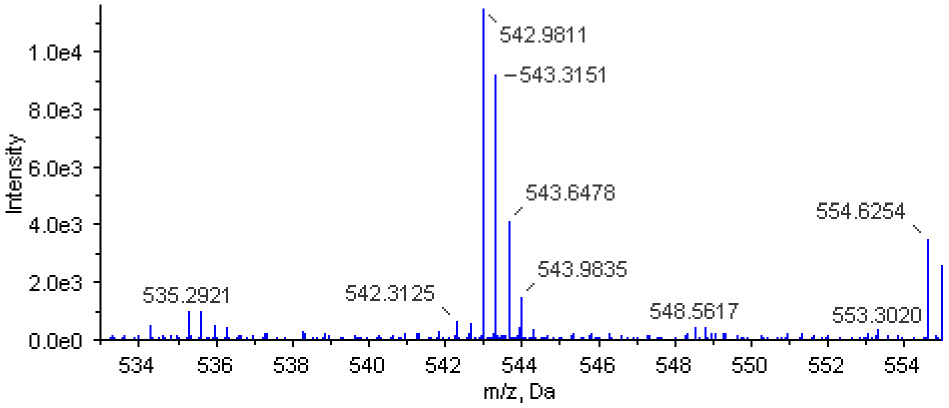

Sequence Coverage and Matched peptides: in different color

M**ASHIVGYPR**MGPKRELK**FALESFWDGKSSAQDLEK**VASDLRESIWKQMAAAGIKHIPSNTFSYYDQVL
DTTAMLGAVPPRYGWNGGEIGFDTYFSMAR**GNASVPAMEMTK**WFDTNYHFIVPELGPDVNFSYASHKAV
TEYKEAKAHGVDTVPVLVGPVSYLLLSKPAKGVEK**TFSLLSLLGK**ILPVYKEVISELKAAGASWIQFDE
PTLVLDLDSHKLQAFTDAYSELESTLSGLNVLIETYFADIPAEAFKTLTSLK**GVTAYGFDLVR**GTKTLD
LIKSEFPKGK**YLFAGVVDGR**NIWANDLAASLTILHELEGIVGKDKLVVSTSSSLLHTAVDLVNETKLDK
ELK**SWLAFAAQK**IVEVNALAK**ALAGEKDEAFFSANAAAQASR**KSSPRVTNEAVQKAAAALKGSDHRRAT
NVSARLDAQQK**KLNLPILPTTTIGSFPQTIELR**RVRREYKAKKVSEDDYVKAIKEEISKVVKLQEELDI
DVLVHGEPERNDMVEYFGEQLSGFAFTVNGWVQSYGSR**CVKPPIIYGDVSRPNPM**TVFWSSTAQSMTAR
PMK**GMLTGPVTILNWSFVR**NDQPRFETCYQIALAIKDEVEDLEK**AGINVIQIDEAALR**EGLPLRKSEQA
FYLDWAVHSFRITNVGVQDTTQIHTHMCYSNFNDIIHSIIDMDADVITIENSRSDEKLLSVFREGVK**YG**
AGIGPGVYDIHSPRIPSTEEIADRINKMLAVLEM**NILWVNPDCGLK**TRK**YSEVKPALSNMVAAAK**LLRT
QLASAK

Protein No.: I-24

Protein name and Species:

ATP-citrate synthase, putative OS=Ricinus communis GN=RCOM_1000580 PE=4 SV=1

Accession: tr|B9RZR0|B9RZR0_RICCO

Unused ProtScore: 30.15

Seq Cov %: 42

ITRAQ Changed Ratio:

117: 115=E3TL: D3TL=0.31

116: 114=E5TL: D5TL=0.59

Annotated typical Precursor MS Region:

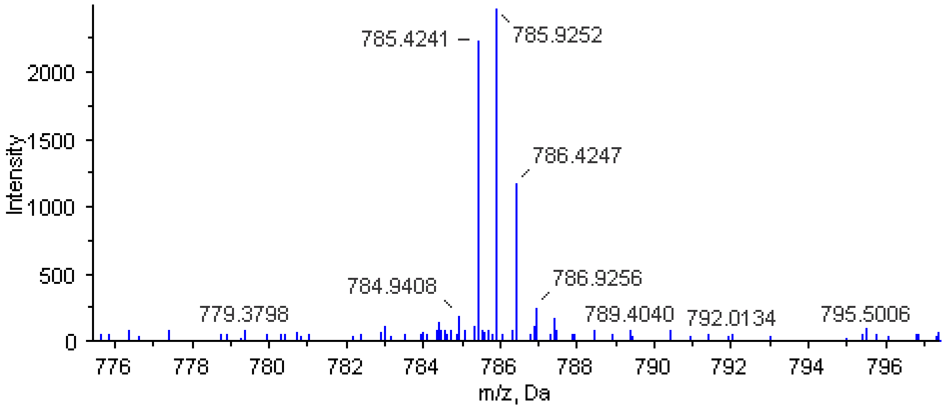

Sequence Coverage and Matched peptides: in different color

MATGQLFSR**TTQALFYNYK**QLPIQR**MLDFDFLCGRETPSVAGIINPGSEGFQK**LFFGQEEIAIPVHSAI
EAACAAHPTADVFINFASFR**SAAASSMAALKQPTIRVVAIIAEGVPESDTK**QLIAYARANNK**VVIGPAT**
VGGIQAGAFKIGDTAGTIDNIIACKLYRPGSVGFVSKSGGMSNELYNTIARVTDGIYEGIAIGGDVFPG
STLSDHVLRFNNIPQVKMIVVLGELGGR**DEYSLVEALK**QGKVNKPVVAWVSGTCARLFK**SEVQFGHAGA**
KSGGEMESAQAKNQALK**DAGAVVPTSYEAFETAIK**ETFQKLVEEGKITPVK**EVKPPQIPEDLNTAIK**SG
KVR**APTHIISTISDDR**GEEPCYAGVPMSSIVEQGYGVGDVISLLWFKRSLPRYCTHFIEICIMLCADHG
PCVSGAHNTIVTARAGK**DLVSSLVSGLLTIGPRFGGAIDDAAR**YFKDAYDRGLAPYEFVESMKKKGIRV
PGIGHRIKRGDNRDKRVELLQRFARTHFPSVK**YMEYAVEVETYTLSK**ANNLVLNVDGAIGSLFLDLLAG
SGMFTKQEIDEIVEIGYLNGLFVLARSIGLIGNRSE

Protein No.: I-25

Protein name and Species:

Phosphofructokinase, putative OS=Ricinus communis GN=RCOM_1602260 PE=4 SV=1

Accession: tr|B9R8U5|B9R8U5_RICCO

Unused ProtScore: 29.94

Seq Cov %: 42.5

ITRAQ Changed Ratio:

117: 115=E3TL: D3TL=0.34

116: 114=E5TL: D5TL=0.36

Annotated typical Precursor MS Region:

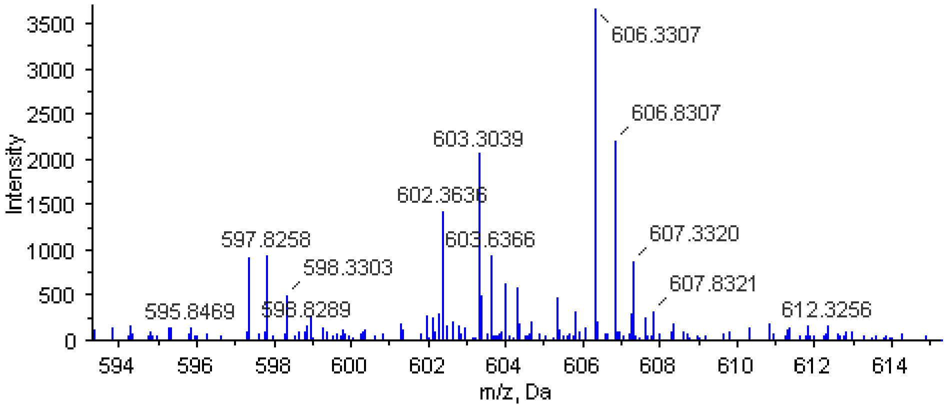

Sequence Coverage and Matched peptides: in different color

MDSDFGIPRELSDLQKLR**SLYQPELPPCLQGTTVR**VELGDGTTAADEAGAHTISR**SFPH****TYGQPLAHFL**
RATAKVADAHIISEHPAMRVGVVFCGRQSPGGHNVVWGLHNALKIHNPN**STLLGFLGGSEGLFAQK****TLE**
VTDDILSTYKNQGGYDLLGRTKDQIR**TTEQVHAALTTCK**NLK**LDGLVIIGGVTSNTDAAQLAETFAEAK**
CPTK**VVGVPVTLNGDLKNQFVETNVGFDTICKVNSQLISNVCTDALSAEK**YYYFIRLMGRK**ASHVALEC**
TLQSHPNMVILGEEVAASKLTLFDLTKQVCDAVQARAEQDKYHGVILLPEGLIESIPEVYALLK**EIHGL**
LRQGVSPNNISSQLSPWASALFEFLPPFIKK**QLLLYPESDDSAQLSQIETEKLLAHLVEAEMNK**RLKEG
TYKGKK**FNAICHFFGYQAR**GSLPSKFDCDYAYVLGHICYHVLAAGLNGYMATATNLKNPVNKWRCGAAP
IAAMMTVKRWAQNPGATSIGK**PAIHPAAVDLK**GKAYELLRLNAVK**FLMDDLYRNPGPLQFEGPGADAKP**
ITLCVEDQDYMGRIKKLQEYLDKVR**TIVKPGCSQEVLK**AALSVMASVTDVLLTMSSTSLDGQRPL

Protein No.: I-27

Protein name and Species:

14-3-3 protein 2 OS=Hevea brasiliensis PE=2 SV=1

Accession: tr|F2YZ43|F2YZ43_HEVBR

Unused ProtScore: 27.55

Seq Cov %: 51.5

ITRAQ Changed Ratio:

117: 115=E3TL: D3TL=0.53

116: 114=E5TL: D5TL=0.86

Annotated typical Precursor MS Region:

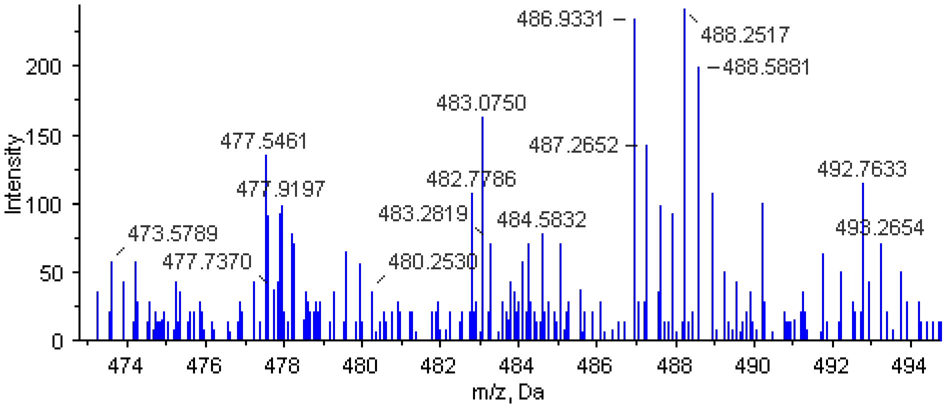

Sequence Coverage and Matched peptides: in different color

MSPTESSREENVYMAKLAEQAERYEEMVEFMEKVAKTVDVEELTVEERNLLSVAYKNVIGARRASWRII
SSIEQKEESRGNEDHVTIIKEYRGKIESELSKICDGILSLLESHLIPSASSAESKVFYLKMKGDYHRYL
AEFKTAGERKEAAESTLLAYKSAQDIALADLAPTHPIRLGLALNFSVFYYEILNSPDRACNLAKQAFDE
AISELDTLGEESYKDSTLIMQLLRDNLTLWTSDITDEAGDEIKEASKRESGEGQPQQ

Protein No.: I-28

Protein name and Species:

RAB2A OS=Lotus japonicus GN=rab2A PE=2 SV=1

Accession: tr|Q40208|Q40208_LOTJA

Unused ProtScore: 27.3

Seq Cov %: 65.4

ITRAQ Changed Ratio:

117: 115=E3TL: D3TL=0.38

116: 114=E5TL: D5TL=0.39

Annotated typical Precursor MS Region:

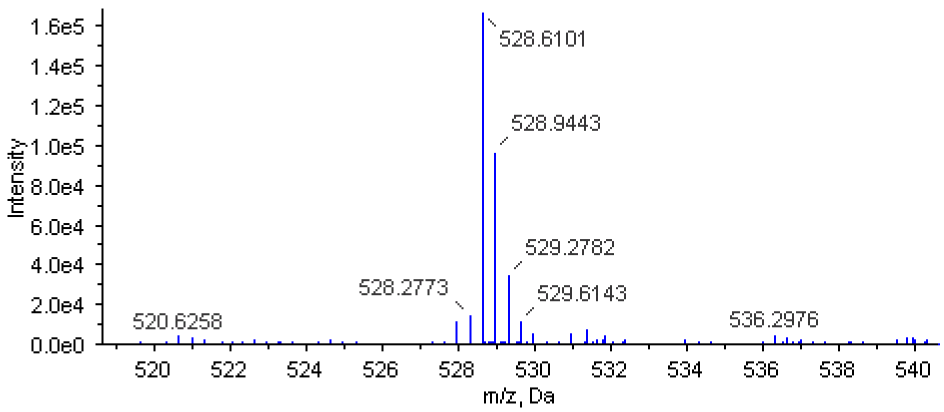

Sequence Coverage and Matched peptides: in different color

MSYAYLFKYIIIGDTGVGKSCLLLQFTDKRFQPVHDLTIGVEFGARMITIDNKPIKLQIWDTAGQESFR
SITRSYYRGAAGALLVYDITRRETFNHLASWLEDARQHANANMTIMLIGNKCDLAHRRAVSTEEGEQFA
KEHGLIFMEASAKTAQNVEEAFIKTAATIYKKIQDGVFDVSNESYGIKVGYGGIPGPSGGRDGPSASAG
GCCS

Protein No.: I-30

Protein name and Species:

Elicitor-responsive protein OS=Hevea brasiliensis GN=ERP PE=2 SV=1

Accession: tr|F8SKC5|F8SKC5_HEVBR

Unused ProtScore: 27.13

Seq Cov %: 90

ITRAQ Changed Ratio:

117: 115=E3TL: D3TL=0.33

116: 114=E5TL: D5TL=0.40

Annotated typical Precursor MS Region:

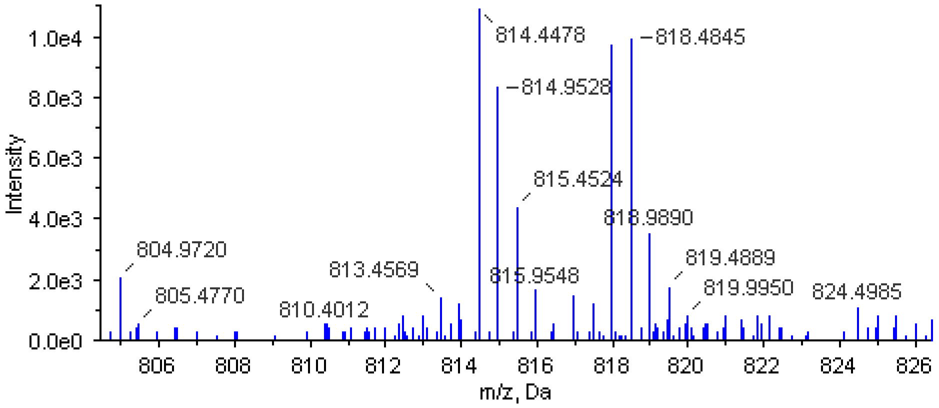

Sequence Coverage and Matched peptides: in different color

MPLGTVEVLLVGAKGLENTDFLNGVDPYVVLACRTQEQKSSVASGKGSEPEWNEKFSFEVSDGDTELTL
KIMDSDVGAADDFVGEATIPLEPLFLEGNLPSTAYKVVKEQEYKGEITVGLTFTPEVEMDNVGVDGYDF
RL

Protein No.: I-31

Protein name and Species:

phospholipase D alpha 1-like [Glycine max] OS=Glycine max PE=2 SV=1

Accession:

tr|C6TEK4|C6TEK4_SOYBN

Unused ProtScore: 27.02

Seq Cov %: 37.6

ITRAQ Changed Ratio:

117: 115=E3TL: D3TL=1.60

116: 114=E5TL: D5TL=1.32

Annotated typical Precursor MS Region:

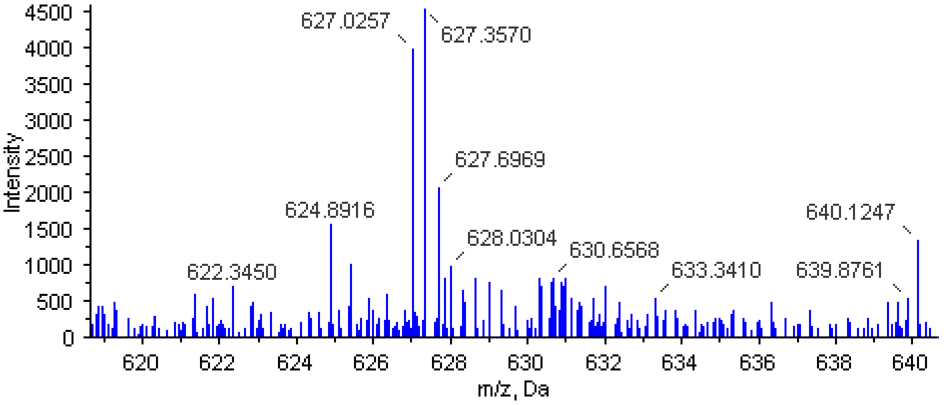

Sequence Coverage and Matched peptides: in different color

MATEHEDDFKDEK**NPRPLDEDDIALLK**TYGLGPYSTSIKKAEKEIKEMAKK**VNDLCGIK**ESDTGLAAPS
QWDLVSDK**QMMQEEQPLQVAR**CTKIINPNSEDAKYV**INVK**QIAK**FVVGLGDK**VSPTDIEEGMRVGVDRN
KYQIQIPLPPK**IDPSVTMMTVEEKPDVTYNDVGGCK**EQIEKMR**EVVELPMLHPEK**FVKLGIDPPK**GVLC**
YGPPGTGKTLLARAVANR**TDACFIRVIGSELVQK**YVGEGARMVRELFQMARSKK**ACIVFFDEVDAIGGA**
RFDDGVGGDNEVQRTMLEIVNQLDGFDARGNIK**VLMATNR**PDTLDPALLRPGRLDR**KVEFGLPDLESR**T
QIFKIHTRTMNCERDIRFKLLARLCPNSTGADIR**SVCTEAGMYAIR**ARRKTVTEKDFLDAVNKVIKGYQ
KFSATPKYMVYN

Protein No.: I-32

Protein name and Species:

Phospholipase D OS=Ricinus communis GN=RCOM_0899520 PE=3 SV=1

Accession: tr|B9RV56|B9RV56_RICCO

Unused ProtScore: 27.02

Seq Cov %: 22.7

ITRAQ Changed Ratio:

117: 115=E3TL: D3TL=0.63

116: 114=E5TL: D5TL=0.55

Annotated typical Precursor MS Region:

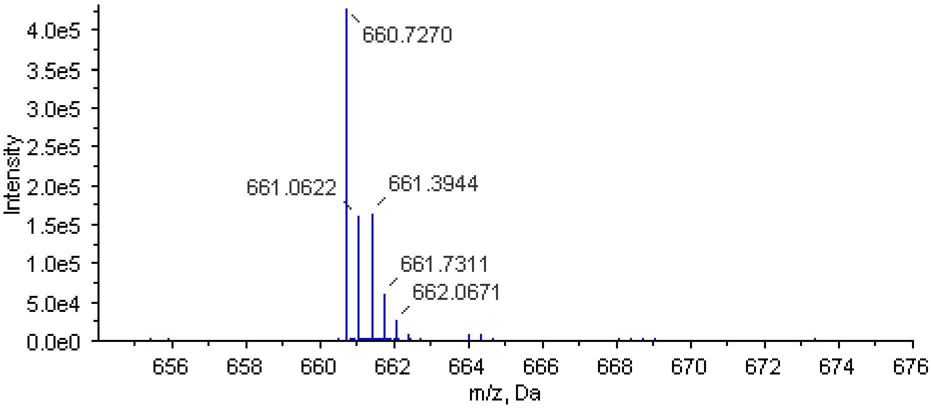

Sequence Coverage and Matched peptides: in different color

MAQISLHGTLHVTIYEVDKLHSGGGPHFFRKLVENIEETVGFGKGVSKLYATIDLEKARVGRTRILENE
QSNPRWYESFHVYCAHQASNVIFTVKDDNPIGATLIGRAYVPVEELLDGEEIDRWVEILDEDKNPVHSG
SKIHVKLQYFEVTKDRNWGQGIRSSKYPGVPYTYFSQRQGCKVSLYQDAHIPDKFVPQIPLAGGNYYEP
HRCWEDVFDAITNAKHLIYITGWSVYTEISLIRDSRRPKPGGDITLGELLKKKASEGVRVLMLVWDDRT
SVGLLKKDGLMATHDEETEHFFQNTDVHCVLCPRNPDDGGSFVQDLQISTMFTHHQKIVVVDSAMPNGD
SQRRRIVSFVGGLDLCDGRYDSPFHSLFRTLDSAHHDDFHQPNFAGASIEKGGPREPWHDIHSRLEGPI
AWDVLFNFEQRWRKQGGKDLLIQLRELEDVIIPPSPVMYPDDFEAWNVQLFRSIDGGAAFGFPETPEDA
ARAGLVSGKDNIIDRSIQDAYIHAIRRAKNFIYIENQYFLGSSFGWSPDGIKPEDINALHLIPKELSLK
ILSKIAAGERFTVYIVVPMWPEGIPESASVQAILDWQKRTMEMMYKDIVQALKANGIIEDPRNYLTFFC
LGNREVKKSGEYEPAEKPEPDTDYIRAQEARRFMIYVHTKMMIVDDEYIIIGSANINQRSMDGARDSEI
AMGAYQPHHLSTRQPARGQIHGFRMSLWYEHLGMLDESFLNPESEECVRKVNQMAEKYWDLYSSETLEH
DLPGHLLRYPIGVASEGDVTELPGTEFFPDTKARVLGAKSDYLPPILTT

Protein No.: I-33

Protein name and Species:

Peptidyl-prolyl cis-trans isomerase OS=Hevea brasiliensis PE=2 SV=1

Accession: tr|B3FNQ1|B3FNQ1_HEVBR

Unused ProtScore: 26.07

Seq Cov %: 64.5

ITRAQ Changed Ratio:

117: 115=E3TL: D3TL=0.06

116: 114=E5TL: D5TL=0.28

Annotated typical Precursor MS Region:

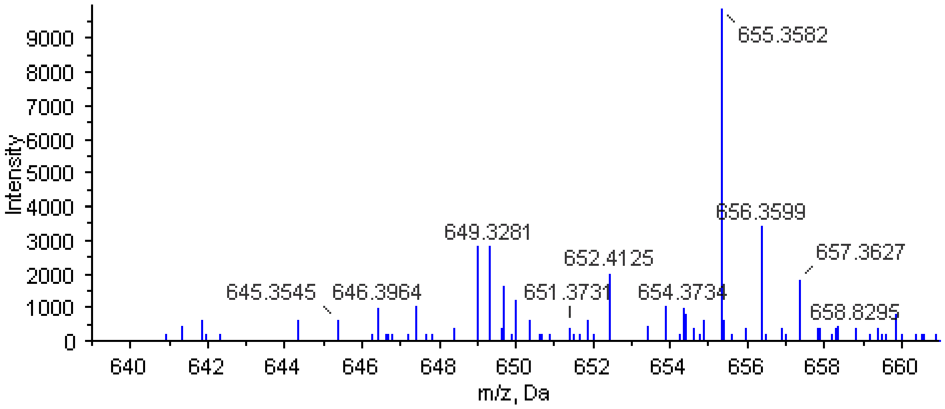

Sequence Coverage and Matched peptides: in different color

MANPKVFFDMTIGGAPAGRIVMELFADTTPRTAENFRALCTGEKGIGRSRKPLHYKGSSFHRVIPGFMC
QGGDFTAGNGTGGESIYGAKFADENFIKKHTGPGILSMANAGPGTNGSQFFICTAKTEWLDGKHVVFGR
VVEGMDVVKTIEKVGSSSGRTSKPVVIADCGQLA

Protein No.: I-39

Protein name and Species:

Small rubber particle protein; REF-like stress related protein 2 OS=Hevea brasiliensis GN=SRPP PE=4 SV=1

Accession: tr|Q84T88|Q84T88_HEVBR

Unused ProtScore: 24.89

Seq Cov %: 97.4

ITRAQ Changed Ratio:

117: 115=E3TL: D3TL=0.39

116: 114=E5TL: D5TL=0.61

Annotated typical Precursor MS Region:

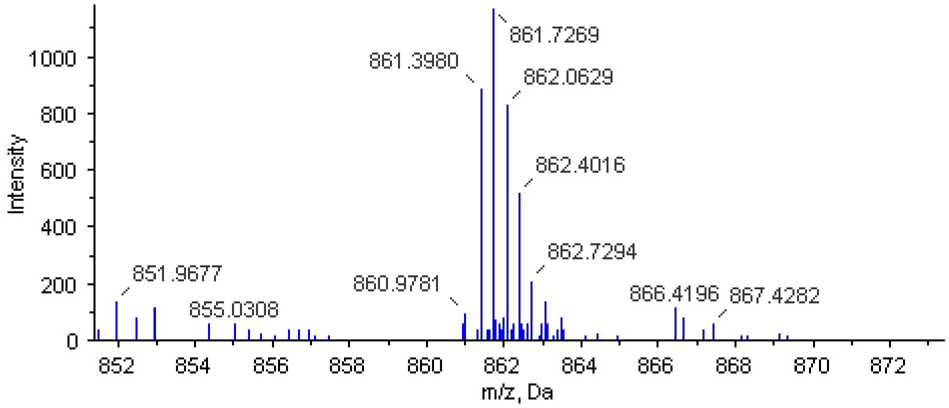

Sequence Coverage and Matched peptides: in different color

MAEGKENENFQQEANEQEEKLKYLEFVQATTDNAVTALSNIYLYAKDNSGPLKPGVETIEGVAKTVVIP
ASKIPTEAIKFADRAVDASFTTLQNIVPSVLKQLPTQACDTSVKESAE

Protein No.: I-40

Protein name and Species:

eukaryotic translation elongation factor 1-alpha OS=Bruguiera sexangula GN=bsef-1A PE=2 SV=1

Accession: tr|Q8H9B1|Q8H9B1_9ROSI

Unused ProtScore: 24.28

Seq Cov %: 45.2

ITRAQ Changed Ratio:

117: 115=E3TL: D3TL=2.07

116: 114=E5TL: D5TL=1.56

Annotated typical Precursor MS Region:

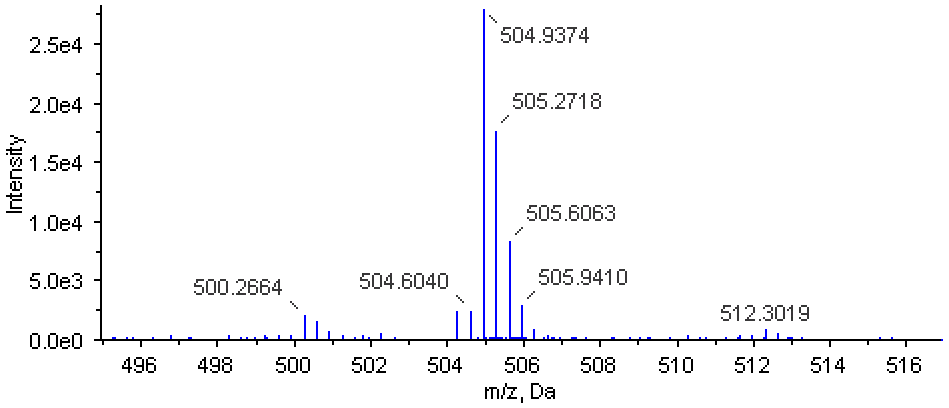

Sequence Coverage and Matched peptides: in different color

MGKEKIHIN**IVVIGHVDSGKSTTTGHLIYK**LGGIDKRVIER**FEKEAAEMNK**RSFKYAWVLDKLKAERER
GITIDIALWKFETTK**YYCTVIDAPGHR**DFIK**NMITGTSQADCAVLIIDSTTGGFEAGISK**DGQTREHAL
LAFTLGVK**QMICCCNK**MDATTSKYSKARYDEIVK**EVSSYLK**K**VGYNPEKIPFVPISGFEGDNMIERSTN**
LDWYKGPTLLEALDMIQEPKRPSDKPLR**LPLQDVYKIGGIGTVPVGRVETGVLKPGMVVTFGPSGLTTE**
VKSVEMHHEALQEALPGDNVGFNVKNVSVKDLKRGYVASNSKDDPAKEASSFTSQVIIMNHPGQIGNGY
APVLDCHTSHIAVKFSEILTKIDRRSGKELEKEPKFLKNGDAGFVK**MIPTKPMVVETFSEYPPLGR**FAV
RDMR**QTVAVGVIKSVEK**KEPSGAKVTKSAAKKGGK

Protein No.: I-42

Protein name and Species:

Phosphoglucomutase, putative OS=Ricinus communis GN=RCOM_0628900 PE=3 SV=1

Accession: tr|B9SP64|B9SP64_RICCO

Unused ProtScore: 24.12

Seq Cov %: 36.8

ITRAQ Changed Ratio:

117: 115=E3TL: D3TL=1.87

116: 114=E5TL: D5TL=1.11

Annotated typical Precursor MS Region:

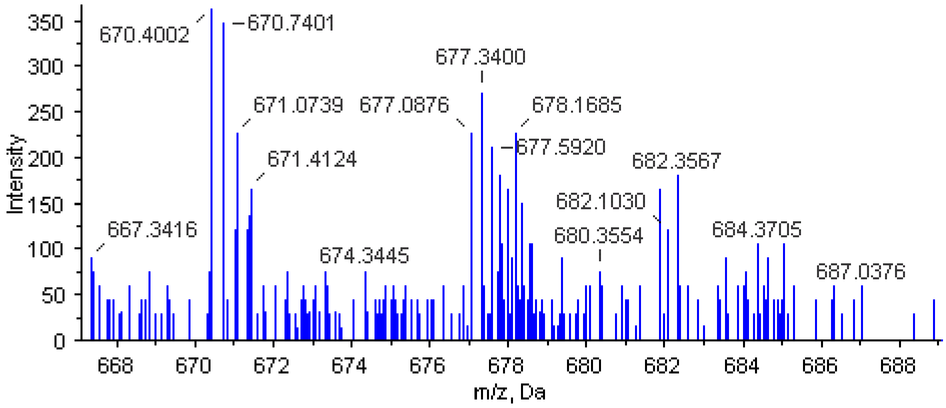

Sequence Coverage and Matched peptides: in different color

MVFEVSRVSTTPFDGQKPGTSGLRKKVKVFTQPN**YLHNFVQSTFDALTAEK**VR**GATLVVSGDGR**YFSKD
AIQIIIKMSAANGVRR**VWVGQNGLLSTPAVSAVIR**ERVGLDGSK**ATGAFILTASHNPGGPHEDFGIKYN**
MENGGPAPEGITDKIYENTKTIKEYLISDLPDVDISAIGVTNFSGPEGQFDVDVFDSADDYVKLMKSIF
DFESIKKLISSPN**FTFCYDALHGVAGAYAK**RIFVEELCAQESSLLNCVPK**EDFGGGHPDPNLTYAK**ELV
ARMGLGK**SNSEVEPPEFGAAADGDADR**NMILGKRFFVTPSDSVAIIAANAVEAIPYFSAGLKGVARSMP
TSAALDIVAKHLNLKFFEVPTGWKFFGNLMDAGLCSVCGEESFGTGSDHIREKDGIWAVLAWLSILAYK
NKENLNGGKLVSVEDIVRNHWATYGRHYYTR**YDYENVDAGAAKELMAHLVKLQSSLGEVNEIVK**GIRSD
VAKVVHGDEFEYKDPVDGSISSHQGIRYLFEDGSRLVFR**LSGTGSEGATIRLYIEQYEK**DSSKTGR**DSQ**
EALAPLVEVALKLSK**MQEFTGR**SAPTVIT

Protein No.: I-43

Protein name and Species:

Trehalose-6-phosphate synthase, putative OS=Ricinus communis GN=RCOM_1251280 PE=4 SV=1

Accession: tr|B9S8D6|B9S8D6_RICCO

Unused ProtScore: 23.88

Seq Cov %: 22.7

ITRAQ Changed Ratio:

117: 115=E3TL: D3TL=0.59

116: 114=E5TL: D5TL=0.70

Annotated typical Precursor MS Region:

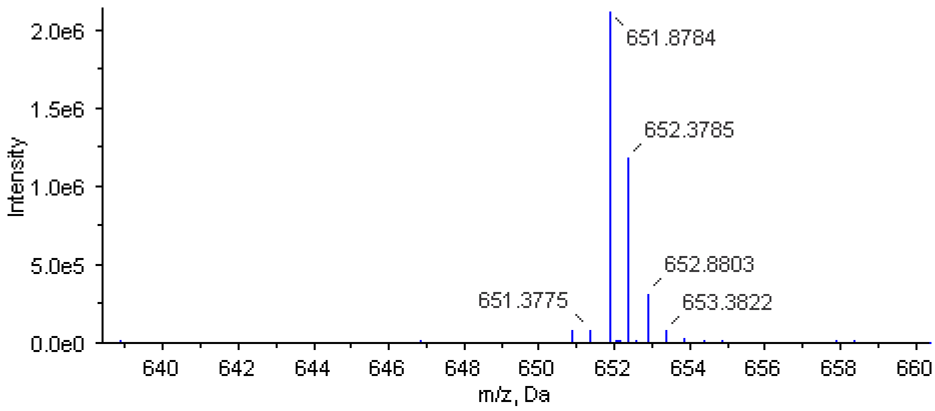

Sequence Coverage and Matched peptides: in different color

MMSR**SYTNLLDLASGNFPVMGQPR**EKKRLPRVMTVPGVISELDDDQANSVASDVPSSLVQDR**IIIVANQ**
LPVKAKRRPDNKGWSFSWDDDSLLLQLK**DGLPEDMEVIYVGSLRVEVDMSEQDDVSQLLLDR**FK**CVPAF**
LPPDILSKFYHGFCKQHLWPLFHYMLPFSANHGGRFDR**SLWEAYVAANK**IFSQRVIEVINPEDDYVWIH
DYHLMVLPTFLRRRFNRLRMGFFLHSPFPSSEIYR**TLPVREEILK**ALLNSDLIGFHTFDYARHFLSCCS
R**MLGLEYQSK**R**GYIGLEYYGR**TVGIKIMPVGIHMGQIQSVLKLADKEWRVGELKQQFEGK**TVLLGVDDM**
DIFKGVNLK**LLAMEQMLK**QHPKWQGR**AVLVQIANPAR**GKGKDLLEIQAEIQASCKRINESFGQPGYEPI
VFIDRPVSLSERAAYYTIAECVVVAAVR**DGMNLTPYEYIVCR**QGVSGSESSSESNGPKK**SMLVVSEFIG**
CSPSLSGAIRVNPWNIEATAEAMNEAISMSDSEKQLRHEKHYRYVSTHDVAYWARSFFQDMERTCRDHF
RRRCWGIGLSFGFR**VVALDPNFR**KLSIDAIVSAYLRSKNRAILLDYDGTVMPQTSINKSPSQEVISIID
TLCSDAKNTVFVVSGRGRDSLGKWFSPCRK**LGIAAEHGYFMR**WSADRQWETCGQTTDFGWIQMAEPVMK
LYTESTDGSSIETKESALVWHHR**DADPGFGASQAKEMLDHLESVLANEPVAVK**SGQFIVEVKPQGISKG
FVAEKIFTSMAENGRQADFVLCIGDDRSDEDMFEIIGNAISSGVLSSSTSVFACTVGQKPSKAKYYVDD
TGEVINMLEALAEASSPSPSPPNSP

Protein No.: I-49

Protein name and Species: **Exportin-1 OS=Populus trichocarpa GN=POPTRDRAFT_774005 PE=4 SV=1**

Accession: **tr|B9I8G4|B9I8G4_POPTR**

Unused ProtScore: **22.33**

Seq Cov %: **12.4**

ITRAQ Changed Ratio:

117: 115=E3TL: D3TL=1.84

116: 114=E5TL: D5TL=2.19

Annotated typical Precursor MS Region:

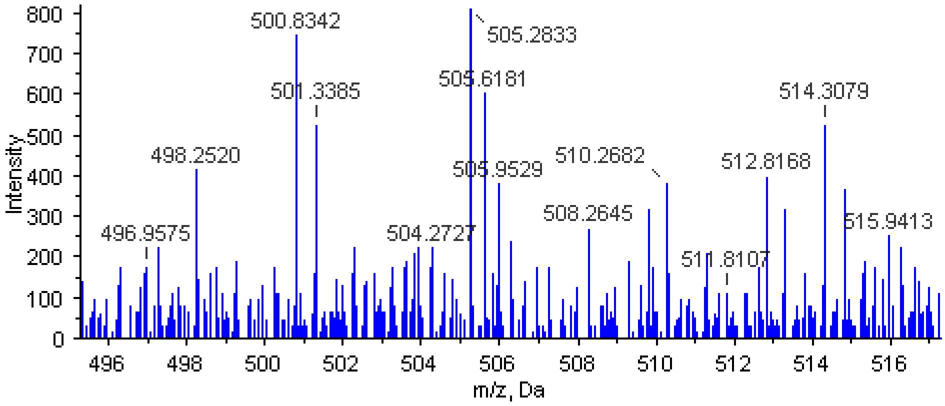

Sequence Coverage and Matched peptides: in different color

MAAEKFRDLSQAIDVPLLDATVAAFYGTGSKEERVAADRILQDLQNNPDMWLQVVHILQNTKNLNTKFF
ALQVLEGVIKYRWNALPVEQRDGMKNYISEVIVQLSSNEASFRMERLYVNKLNVTLVQILKHEWPARWR
SFIPDLVAAAKTSETICENCMVILKLLSEEVFDFSRGEMTQQKIKELKQSLNSEFQLIHELCLYVLSAS
QRTELIRATLSTLHAFLSWIPLGYIFESPLLETLLKFFPMPSYRNLTLQCLTEVAALNFGDFYNIQYVK
MYNFFMVQLQAILPLTTNIPEAYANGSSEEQAFIQNLALFFTSFYKSHIQVLESTQENITALLMGLEYL
INICYVDDTEVFKVCLDYWNSLVLELFEARHNLDNPAVAVNMMGLQMPLLHGMVDGLGSQILQRRQLYA
TPMSKLRMLMICR**MAKPEEVLIVEDENGNIVR**ETMK**DNDVLVQYK**IMRETLIYLSHLDHEDTEKQMLKK
LSKQLSGEDWNWNNLNTLCWAIGSISGSMMEEQENRFLVMVIR**DLLNLCEITK**GKDNK**AVIASNIMYVV**
GQYPRFLRAHWKFLKTVVNKLFEFMHETHPGVQDMACDTFLKIVQKCKRKFVIVQVGESEPFVSELLAG
LPTTVADLEPHQIHTFYESVGHMIQAESDPQKRDEYLQR**LMDLPNQK**WAEIIGQAR**QSVDFLK**DQDVIR
TVLNIMQTNTSVASALGTYFLSQISLIFLDMLNVYR**MYSELISSSIAEGGPYASK**TSYVKLLRSVKRET
LK**LIETFLDK**AEDQTQIGK**QFVPPMMDPVLGDYAR**NLPDARESEVLSLFATIINKYKAAMIEDVPRIFE
AVFQCTLEMITKNFEDYPEHRLKFFSLLR**AIATHCFPALIR**LSSEQLKLVMDSIIWAFRHTERNIAETG
LNLLVEMLK**NFQASEFCNQFYR**SYFLTIEQEIFAVLTDTFHKPGFKLHVLVLQHLFCLVESGALTEPLW
DAATISYSYPNNAMFVREYTIKLLGTSFPNMTASEVTQFVNGLFESRNDLSAFKNHIRDFLVQSK**EFSA**
QDNKDLYAEEAAAQRERERQRMLSIPGLIAPNEIQDEMLDS

Protein No.: I-50

Protein name and Species:

Invertase 2 OS=Hevea brasiliensis GN=NIN2 PE=2 SV=1

Accession: tr|E9KF95|E9KF95_HEVBR

Unused ProtScore: 22.29

Seq Cov %: 26

ITRAQ Changed Ratio:

117: 115=E3TL: D3TL=1.51

116: 114=E5TL: D5TL=2.20

Annotated typical Precursor MS Region:

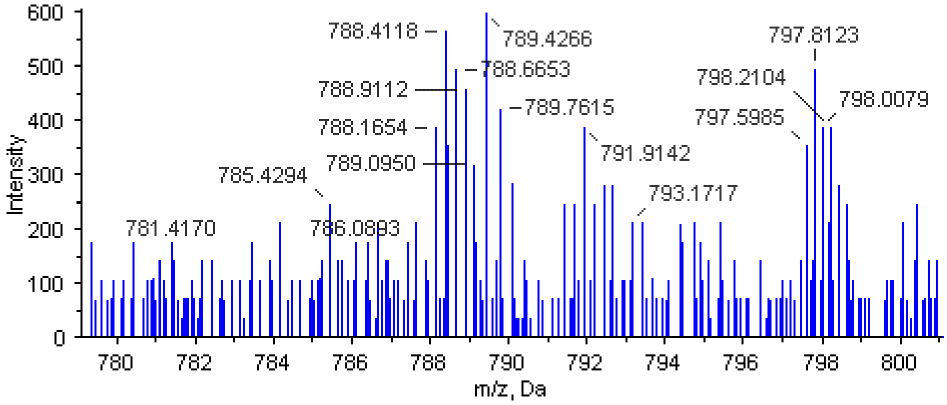

Sequence Coverage and Matched peptides: in different color

MDGTKEVGLR**NVSSTCSISEMDDFDLSR**LLDKPRLNIERQRSFDER**SLSELSIGLTRGGLDYCEITYSP**
GGRSGLDTPVSSARNSFEPHPMVADAWEALRR**SIVYFR**GQPVGTIAAIDHASEEVLNYDQVFVR**DFVPS**
ALAFLMNGEPEIVKNFLLKTLHLQGWEKRIDRFKLGEGVMPASFK**VLHDPVR****KTDTLMADFGESAIGR**V
APVDSGFWW**IILLR**AYTK**STGDLSLAETPECQK**GMRLILTLCLSEGFDTFPTLLCADGCSMIDRRMGIY
GYPIEIQALFFMALR**CALSMLK**HDTEGKECIERIVKRLHALSYHIRSYFWLDFQQLNDIYRYK**TEEYSH**
TAVNKFNVIPDSIPDWVFDFMPTR**GGYFIGNISPAR**MDFRWFALGNCVAILSSLATPEQSMAIMDLIES
RWEELVGEMPLK**IAYPAIESHDWR**IVTGCDPKNTRWSYHNGGSWPVLLWLLTAACIKTGRPQIARRAID
LAETRLLKDSWPEYYDGKLGKFIGKQARKYQTW**SIAGYLVAKMMLEDPSHLGMISLEEDK**QMKPVIKRS
SSWTC

Protein No.: I-53

Protein name and Species:

Sucrose synthase OS=Manihot esculenta PE=2 SV=1

Accession: tr|A9NIV2|A9NIV2_MANES

Unused ProtScore: 21.98

Seq Cov %: 15.9

ITRAQ Changed Ratio:

117: 115=E3TL: D3TL=0.57

116: 114=E5TL: D5TL=0.62

Annotated typical Precursor MS Region:

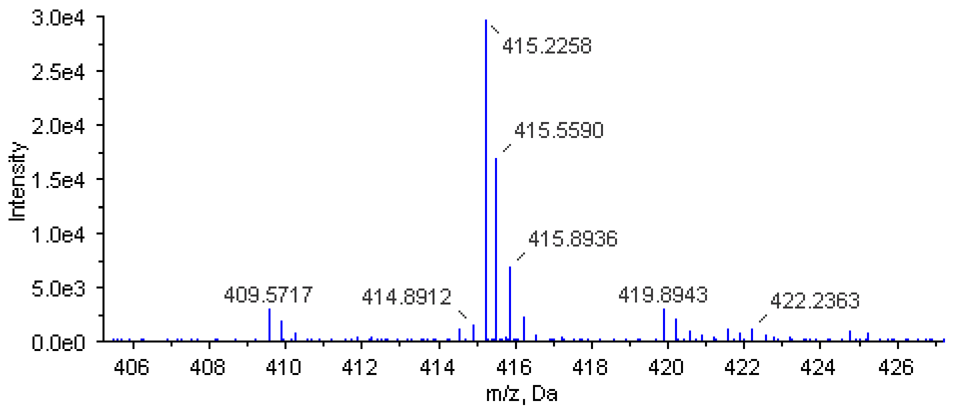

Sequence Coverage and Matched peptides: in different color

MAERVITRVQSIRER**LDETLSAHRNEIVALLSR**IEGKGKGILQHHQIIAEFEEIPEENRKK**LLESVFGE**
VLRSAQEAIVLPPWVALAVRPRPGVWEYIR**VNVHALVVEELR**VAEYLHFKEELVDGSVNGNFVLELDFE
PFNASFPRPTLSK**YIGNGVEFLNR**HLSAKLFHDK**ESLHPLLEFLK**VHCHKGKNMMLNDR**IHNLDSLQYV**
LRKAEEYLTALAPETPYSQFEHK**FQEIGLER**GWGDTAERVLEMIRLLLDLLEAPDPCTLETFLGRIPMV
FNVVIMSPHGYFAQDNVLGYPDTGGQVVYILDQVR**ALETEMLHR**IKQQGLDITPRILIITR**LLPDAVGT**
TCGQRLEK**VFGTEHSDILR**IPFKTEKGIVRKWISRFEVWPYLETYTEDVATEIGKEFQGKPDLIFGNYS
DGNIVASLLAHK**LGVTECTIAHALEK**TKYPESDIYWKKFDEKYHFSCQFTADLIAMNHTDFIITSTFQE
IAGSKDTVGQYESHTALTLPGLYR**VVHGIDVFDPK**FNIVSPGADETIYFSYTEEKRRLTSFHPEIEELL
YSPVENEEHLCVLKDRSKPIIFTMARLDRVKNLTGLVEWYGKNAKLRELANLVVVGGDRRKESK**DLEEQ**
AEMKKMHSLIEK**YNLNGHFR**WISSQMNRVRNGELYRYICDTKGVFVQPAIYEAFGLTVVEAM**TCGLPTF**
ATCNGGPAEIIVHGKSGFNIDPYHGDQAAELLVQFFEKCKADPSHWIKISEGAMQRIQEKYTWQIYSQR
LLTLTGVYGFWKHVSKLDRRESRR**YLEMFYALK**YRKLAESVPLTVED

Protein No.: I-54

Protein name and Species:

Malate dehydrogenase OS=Populus trichocarpa GN=POPTRDRAFT_747123 PE=3 SV=1

Accession: tr|B9NBW3|B9NBW3_POPTR

Unused ProtScore: 21.97

Seq Cov %: 32.8

ITRAQ Changed Ratio:

117: 115=E3TL: D3TL=0.31

116: 114=E5TL: D5TL=0.92

Annotated typical Precursor MS Region:

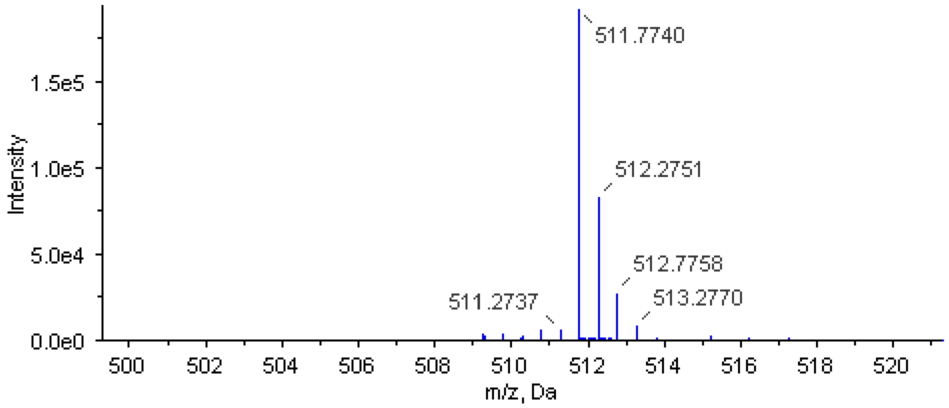

Sequence Coverage and Matched peptides: in different color

MAKEPARVLVTGAAGQIGYALVPMIARGVMLGPDQPVILHMLDIPPAAEALNGVKMELVDAAFPLLKGV
VATTDVVEACTGVNIAVMVGGFPRKEGMERKDVMSKNVSIYKSQASALEKHAAANCKVLVVANPANTNA
LILKEFAPSIPEKNITCLTRLDHNRALGQISERLNVQVSDVKNVIIWGNHSSTQYPDVNHATVKTPAGE
KPVRELVKDDAWLNAEFITTVQQRGAAIIKARKLSSALSAASSACDHIHDWVLGTPEGTWVSMGVYSDG
SYNVPAGLIYSFPVTCQNGEWKIVQGLSIDEFSRKKLDLTADELSEEKALAYSCLT

Protein No.: I-55

Protein name and Species:

Chaperonin containing t-complex protein 1, zeta subunit, tcpz, putative OS=Ricinus communis GN=RCOM_1727810 PE=3 SV=1

Accession: tr|B9RSN1|B9RSN1_RICCO

Unused ProtScore: 21.39

Seq Cov %: 30.8

ITRAQ Changed Ratio:

117: 115=E3TL: D3TL=1.51

116: 114=E5TL: D5TL=1.63

Annotated typical Precursor MS Region:

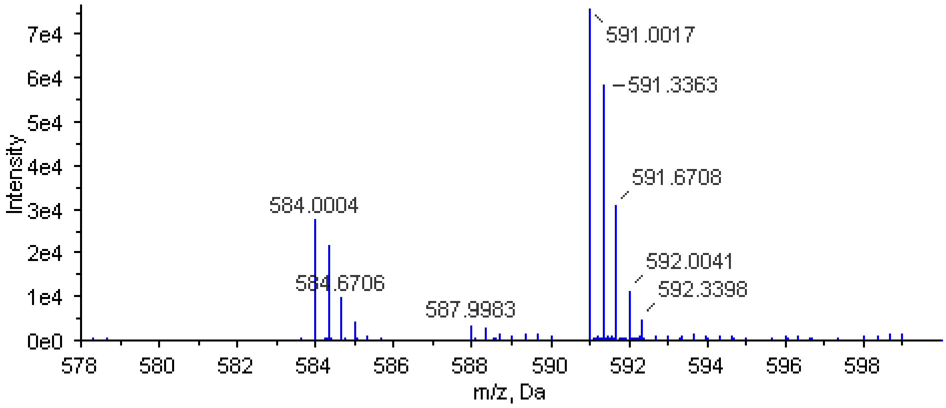

Sequence Coverage and Matched peptides: in different color

MSLRVLNPNAEVLNKSAALHMNINAAKGLQDVLKSNLGPKGTIKMLVGGAGDIKLTKDGNTLLKEMQIQ
NPTAIMIARTAVAQDDISGDGTTSTVIFIGELMKQSERYIGEGMHPRVLVDGFEIAKRATLQFLEKFKT
PVVMGDEPDKEILKMVARTTLRTKLYETLADQLTDIVVNAVLCIRKPEEAIDLFMVEIMHMRHKFDVDT
RLVEGLVLDHGSRHPDMKRRAENCYILTCNVSLEYDKSEINAGFFYSNAEQREKMVAAERRQVDERVEK
IIELKNKVCSGNDNNFVVINQKGIDPPSLDLLARAGIIALRRAKRRNMERLVLACGGEAVNSVDDLTPD
CLGWAGLVYEHILGEEKYTFVEHVKNPHSCTILIKGPNDHTIAQIKDAVRDGLRAVKNTIEDEAVVLGA
GAFELAARKYLISEVKKTVKGRAQLGIEAFADALLVIPKTLAENSGLDTQDEIVSLTGEHDRENIVGLN
LQTGGPLDPQMEGIFDNYSVKRQLINSGPVIASQLLLVDEVIRAGRNMRKPN

Protein No.: I-57

Protein name and Species: Luminal binding protein (BiP), Nucleotide-Binding Domain of the sugar kinase/HSP70/actin superfamily OS=Thellungiella halophila PE=2 SV=1

Accession: tr|E4MXI2|E4MXI2_THEHA

Unused ProtScore: 21.05 **Seq Cov %: 24.2**

ITRAQ Changed Ratio:

117: 115=E3TL: D3TL=3.50

116: 114=E5TL: D5TL=2.24

Annotated typical Precursor MS Region:

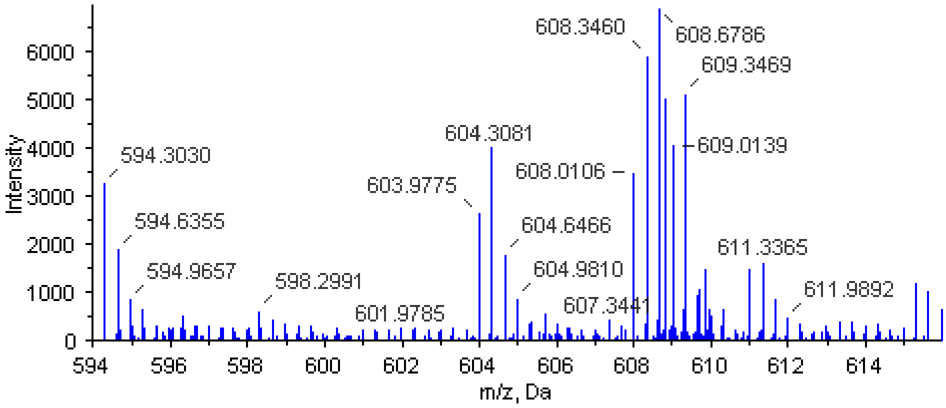

Sequence Coverage and Matched peptides: in different color

MARSFGANGTVVLAIIFFGCLFAFSTAKEEATKLGTVIGIDLGTTYSCVGVYK**NGHVEIIANDQGNR**IT
PSWVGFTDSERLIGEAAKNQAAVNPER**TIFDVK**RLIGRKFEDKEVQKDKKLVPYQIVNKDGKPYIQVKI
KDGETKVFSPEEISAMILTKMKETAEAYLGKK**IKDAVVTVPAYFNDAQR**QATKDAGVIAGLNVAR**IINE**
PTAAAIAYGLDKKGGEKNILVFDLGGGTFDVSVLTIDNGVFEVL**STNGDTHLGGEDFDHR**IMDYFIKLI
KKKHQKDISKDNKALGKLRRECERAKRALSSQHQVR**VEIESLFDGVDFSEPLTR**AR**FEELNNDLFR**KTM
GPVKKAMDDAGLQKS**QIDEIVLVGGSTR**IPK**VQQLLK**DFFEGKEPNK**GVNPDEAVAYGAAVQGGILSGE**
GGDETKDILLLDVAPLTLGIETVGGVMTKLIPRNTVIPTKKSQVFTTYQDQQTTVSIQVFEGERSLTKD
CRLLGK**FDLTGIPPAPR**GTPQIEVTFEVDANGILNVKAEDKASGKSEKITITNEKGR**LSQEEIER**MVK**E**
AEEFAEEDKKVKERIDAR**NSLETYVYNMK**NQVSDKDKLADKLEADEKDKIEAATKEALEWLDENQNSEK
EDYEEKLK**EVEAVCNPIITAVYQR**SGGAPGAAGGDSATDEEDESHDEL

Protein No.: I-58

Protein name and Species: Glucosamine-fructose-6-phosphate aminotransferase, putative OS=Ricinus communis GN=RCOM_1077800 PE=4 SV=1

Accession: tr|B9RM60|B9RM60_RICCO

Unused ProtScore: 21.05

Seq Cov %: 22.7

ITRAQ Changed Ratio:

117: 115=E3TL: D3TL=0.36

116: 114=E5TL: D5TL=0.70

Annotated typical Precursor MS Region:

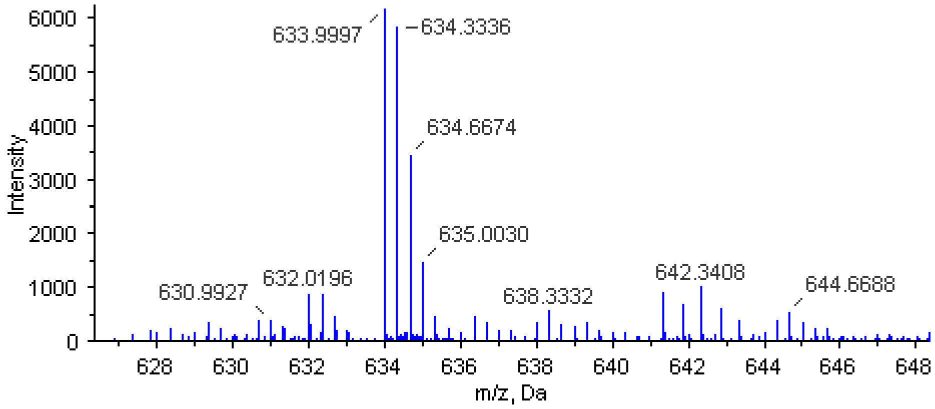

Sequence Coverage and Matched peptides: in different color

MCGIFAYLNYNVNRERRYILQVLFNGLRRLEYRGYDSAGISIENSIPSESSDPIPSSSSHSPSPLVFR**Q**
EGNIESLVKSVYQDVATIDLNLEESFSVHAGIAHTR**WATHGEPAPR**NSHPQTSGAGNEFLVVHNGVITN
YEVLKETLVRHGFTFESETDTEVIPKLAKYVFDKANEGEGAQPVTFSQVVLEVMRHLEGAYALIFKSRH
YPNELIACKR**GSPLLLGVKELNEDMNSGSAFHDAK**SLSKDGHAK**ELFLSSDANAIIEHTK**RVLMIEDGE
VVHLKDGSVSILKFENAKGRHGGALSRPASVQR**ALSVLEMEVEQINK**GNYEHYMQK**EIHEQPESLTTTM**
RGRLIRGGSCKAK**TVLLGGLK**DHLKTIRRSRR**IVFVGCGTSYNAALAAR**PILEELSGVPVTMEIASDLV
DRQGPIYREDTAVFVSQSGETADTLHALEYALENGALCVGITNTVGSAIARNTHCGIHINAGAEIGVAS
TKAYTSQIVVMAMLALAIGGDAISSQAKR**EAIIDGLFDLPNR**VREVLKLDQEMKDLAELLMAEQSLLVF
GR**GYNYATALEGALK**VK**EVALMHSEGMLAGEMK**HGPLALVDENLPIIVIATRDACFRK**QQSVIQQLHAR**
KGRLIVMCSKGDAASVCPGESCRVIEVPQVEDCLQPVVNIVPLQLLAYHLTVLR**GYNVDQPR**NLAKSVT
TQ

Protein No.: I-60

Protein name and Species:

Leucine aminopeptidase, putative OS=Ricinus communis GN=RCOM_0623200 PE=3 SV=1

Accession: tr|B9STR1|B9STR1_RICCO

Unused ProtScore: 20.86

Seq Cov %: 29.1

ITRAQ Changed Ratio:

117: 115=E3TL: D3TL=0.29

116: 114=E5TL: D5TL=0.40

Annotated typical Precursor MS Region:

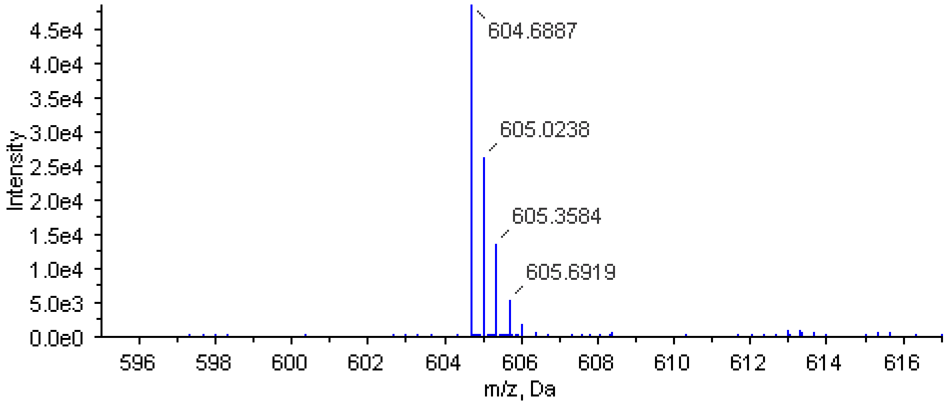

Sequence Coverage and Matched peptides: in different color

MAARVVSLAASISSFAVPFSSTSSSLFLTRLRSPSVLRFSFAVAPLSYRSRGGGGGGGGKLMAHSLAR**A**
TLGLTQPANVDVPKISFSAKEIDVVEWK**GDILAVGVTEK**DMTKDKSTKFDNSLLKK**LDTHLGGLLSEAS**
SEEDFTGKAGQSMVLRLPGLGSKRIGLIGLGQSASTTFAFRNFGETIAATAKAAQASNVAIVLASAESI
SNELKLNTASAIASGTVLGTYEDARFKSESKKPVLKSVDILGFGTGPDLAKKLK**YAEDVSSAVIFGKEL**
VNSPANVLTPAVLAEEASKIASTYSDVLSANILSAEQCKELKMGSYLGVAAASANPPHFIHLHYKPPGG
TVKAK**LALVGKGLTFDSGGYNIKTGPGCSIELMKFDMGGSAAVLGAAK**AIGQIKPPGVEVHFIVAACEN
MISGTGMR**PGDIVTASNGKTIEVNNTDAEGRLTLADALVYACNQGVDK**IVDLATLTGACVVALGPSIAG
VFTPSDDLANEVFTAAEASGEKLWRMPLEESYWENMK**SGVADMVNTGGRQGGAITAALFLK**QFINEKVQ
WMHIDMAGPVWNEKKRTATGFGISTLVEWILHNSS

Protein No.: I-61

Protein name and Species:

Rab GDP dissociation inhibitor OS=Ricinus communis GN=RCOM_1750690 PE=4 SV=1

Accession: tr|B9RIY3|B9RIY3_RICCO

Unused ProtScore: 20.57

Seq Cov %: 41.2

ITRAQ Changed Ratio:

117: 115=E3TL: D3TL=0.63

116: 114=E5TL: D5TL=0.39

Annotated typical Precursor MS Region:

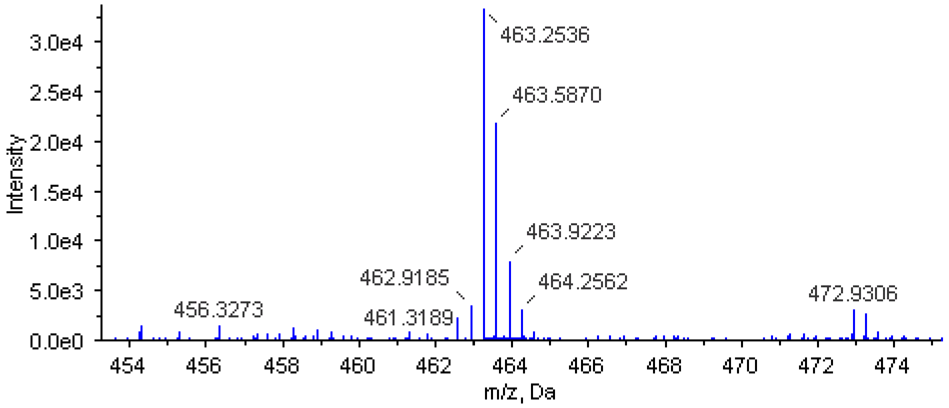

Sequence Coverage and Matched peptides: in different color

MDEEYDVIVLGTGLK**ECILSGLLSVDGLK**VLHMDKNDYYGGESASLNLNQLWKRFR**GSDKPPAHLGSSR**
DYNVDMIPKFMMANGNLVRVLIHTDVTKYLYFK**AVDGSFVYNK**GKVHKVPATDMEALKSPLMGIFEKRR
ARK**FFIYVQDYNESDPKTHEGMDLTR**VTTRELIAKYGLDDNTMDFIGHALALHR**DDRYLDEPALDTVMR**
MK**LYAESLAR**FQGGSPY**IYPLYGLGELPQAFARLSAVYGGTYMLNKPECKVEFDEEGKVVGVTSEGETA**
KCK**KVVCDPSYLPNK**ARKVSRVAR**AIAIMSHPIPNTNDSHSAQVILPQK**QLGRRSDMYLFCCSYSHNVA
PKGKYIAFVSSEAETDNPEVELKPGIDLLGPVDEIFFEVYDRYEPVNEPSLDNCFISTSYDATTHFEST
VTDVLNMYSMITGKVLDLSVDLSAASAAEE

Protein No.: I-64

Protein name and Species:

Alpha-1,4-glucan-protein synthase [UDP-forming], putative OS=Ricinus communis GN=RCOM_1468610 PE=4 SV=1

Accession: tr|B9RLK7|B9RLK7_RICCO

Unused ProtScore: 19.65

Seq Cov %: 36.3

ITRAQ Changed Ratio:

117: 115=E3TL: D3TL=0.60

116: 114=E5TL: D5TL=0.44

Annotated typical Precursor MS Region:

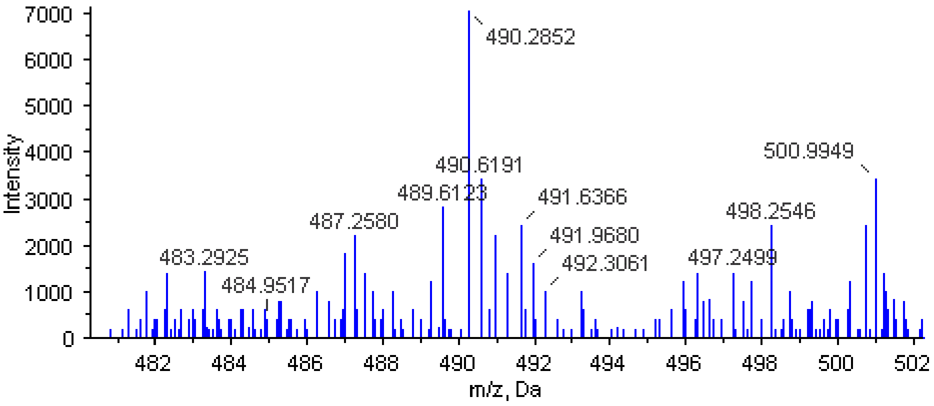

Sequence Coverage and Matched peptides: in different color

MADFAAISPTPLLKDELDIVIPTIRNLDFLEMWRPFFQPYHLIIVQDGDPSKIIKVPEGFDYELYNRND
INRILGPKASCISFKDSACRCFGYMVSKKKYIYTIDDDCFVAKDPSGKDINALEQHIKNLLCPSTPFFF
NTLYDPYRTGADFVRGYPFSLREGVPTAVSHGLWLNIPDYDAPTQLVKPLERNTRYVDAVMTIPKGTLF
PMCGMNLAFNRELIGPAMYFGLMGDGQPIGRYDDMWAGWCTKVICDHLGFGVKTGLPYIYHSKASNPFV
NLKKEYKGIYWQEELIPFFQSAVLPKECTTVQKCYIELSKQVRAKLGKIDEYFVKLADAMVTWVEAWDE
LNPSGNSGELPNGAAK

Protein No.: I-69

Protein name and Species:

Heat shock protein 70 (HSP70)-interacting protein, putative OS=Ricinus communis GN=RCOM_1679700 PE=4 SV=1

Accession: tr|B9RBP6|B9RBP6_RICCO

Unused ProtScore: 18.74

Seq Cov %: 19.2

ITRAQ Changed Ratio:

117: 115=E3TL: D3TL=0.58

116: 114=E5TL: D5TL=0.57

Annotated typical Precursor MS Region:

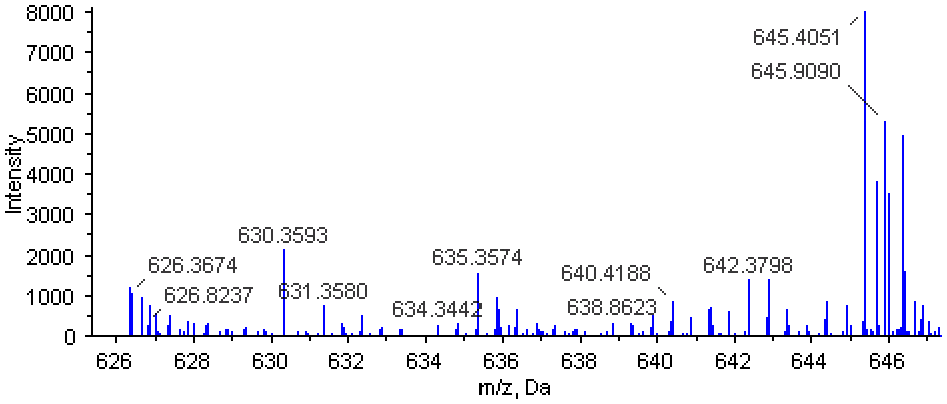

Sequence Coverage and Matched peptides: in different color

MAEEAKAKGNAAFSAGDFTTAVKHFTEAISIAPSNHVLYSNRSAALASLHNYADALTDAKKTVELKPDW
SKGYSRLGAAYLGLNQTQDAISAYKKGLDIDPSNEALKSGLADAQARFSAPPPPPSPFGDAFNGPEMWA
KLTADPSTRMYLQQPDFVKMMQEIQKNPNNLNLYLKDQRVMQALGVLLNLKFRAPNAGAEDMEVPQEDD
HSSSPPPQAEQPAKKAPQPEPEPEPMDVITEEDKEAKERKAQAVKEKELGNAAYKQKDFETAISHYTTA
LELDDEDISYLTNRAAVYLEMGKYEDCIKDCDKAVERGRELRSDFKMIARALTRKGTALVKMAKSSKDY
DSAIETFQKALTEHRNPETLKKLNEAERAKKELEQQEYFDPKLADEEREKGNEYFKQQKYPEAVKHYTE
SLRRNPEDPRAYSNRAACYTKLGALPEGLKDAEKCIELDPTFTKGYTRKGAVQFFMKEYDKALETYQEG
LKHDPQNQELLDGVRRCVEQLNKASRGDLSPEELKERQAKAMQDPEIQNILSDPVMRQVLVDFQENPKS
AQDHMKNPMVMNKIQKLVNAGIVQIR

Protein No.: I-70

Protein name and Species:

DnaJ protein OS=Hevea brasiliensis PE=2 SV=1

Accession: tr|Q9ZSZ6|Q9ZSZ6_HEVBR

Unused ProtScore: 18.66

Seq Cov %: 38.1

ITRAQ Changed Ratio:

117: 115=E3TL: D3TL=0.52

116: 114=E5TL: D5TL=0.45

Annotated typical Precursor MS Region:

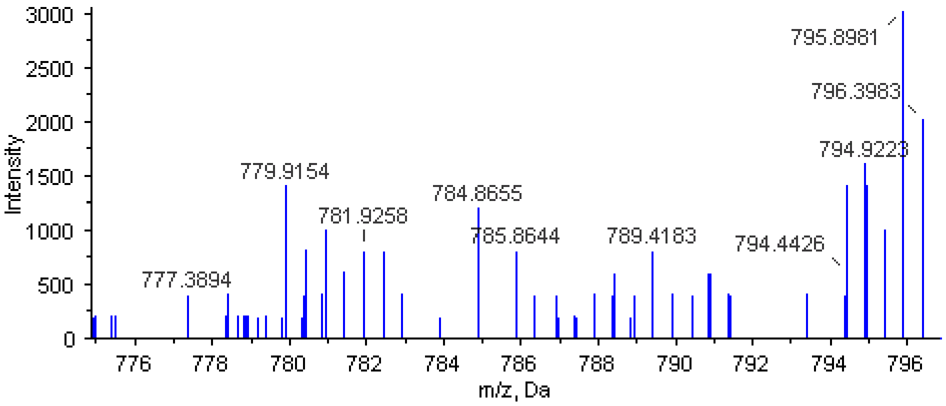

Sequence Coverage and Matched peptides: in different color

MFGRAPKKSDNTK**YYEILGVSK**NASQDDLKKAYRKAAIKNHPDKGGDPEKFK**ELAQAYEVLSDPEKREI**
YDQYGEDALKEGMGSGGGAHDPFDIFQSFFGGNPFGGGGSSRGRRKE**GEDVIHPLK****VSLEDLYNGTSK**K
LSLSRNVICSKCKGKGSKSGASMKCSGCQGSGMKVSIR**QLGPSMIQQMQHPCNECKGTGETINDKDR**CP
QCKGEKVVQEKK**VLEVIVEK**GMQNGQRITFPGEADEAPDTITGDIVFVLQQKEHPKFKRKGDDLIVDHT
LSLTEALCASQFILTHLDGDLLIK**SQPGEVVKPDQFKAINDEGMPMYQR**PFMRGKLYIHFSVDFPDSLP
PDQCK**ALEAVLPSR****TSVQLSDMELDECEETTLHDVNFDEEMR**RK**QQQAQEAYDEDDDMHGGGQR**VQCAQ
Q

Protein No.: I-71

Protein name and Species:

Phosphoglycerate kinase OS=Ricinus communis GN=RCOM_1574570 PE=3 SV=1

Accession: tr|B9RHY3|B9RHY3_RICCO

Unused ProtScore: 18.31

Seq Cov %: 33.9

ITRAQ Changed Ratio:

117: 115=E3TL: D3TL=0.54

116: 114=E5TL: D5TL=0.63

Annotated typical Precursor MS Region:

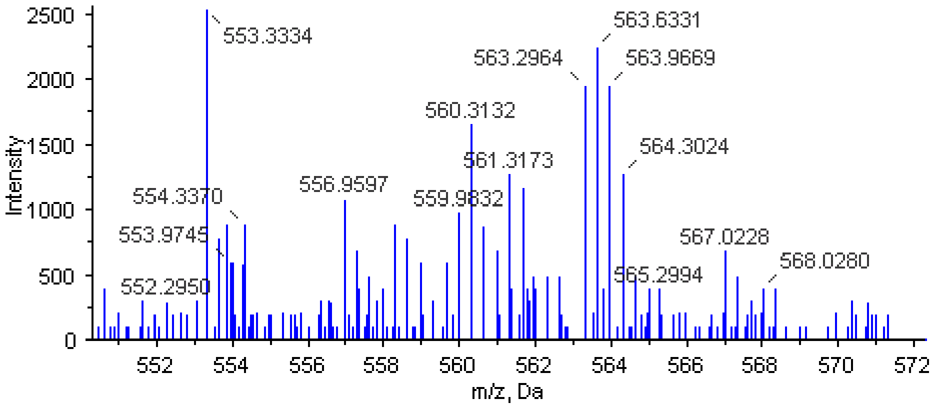

Sequence Coverage and Matched peptides: in different color

MATKKSVSSLKEADLKGKRVFVRVDLNVPLDDNLNITDDTRIRAAVPTIKYLMDHGARVILCTHLGRPK
GVTPKYSLKPLVPRLSELLGVEVKMASDCIGEEVEKLVAEIPEGGVLLLENVRFHKEEEKNEPEFAKKL
AGLADVYVNDAFGTAHRAHASTEGVAKYLKPSVAGFLMQKELDYLVGAVANPKKPFAAIVGGSKVSTKI
GVIESLLEKVDILILGGGMIFTFYKAQGHSVGSSLVEGDKLDLASSLIEKAKAKGVDLLLPTDVVIADK
FAADANSKVVPASSIPDGWMGLDVGPDAIKTFCEALDTTQTIIWNGPMGVFEFDKFAVGTEAIAKKLAE
LSGKGVTTIIGGGDSVAAVEKVGLADKMSHISTGGGASLELLEGKPLPGVLALDDA

Protein No.: I-73

Protein name and Species:

Alanyl-tRNA synthetase, putative OS=Ricinus communis GN=RCOM_0036070 PE=3 SV=1

Accession: tr|B9SYR5|B9SYR5_RICCO

Unused ProtScore: 18.11

Seq Cov %: 13.1

ITRAQ Changed Ratio:

117: 115=E3TL: D3TL=0.63

116: 114=E5TL: D5TL=0.52

Annotated typical Precursor MS Region:

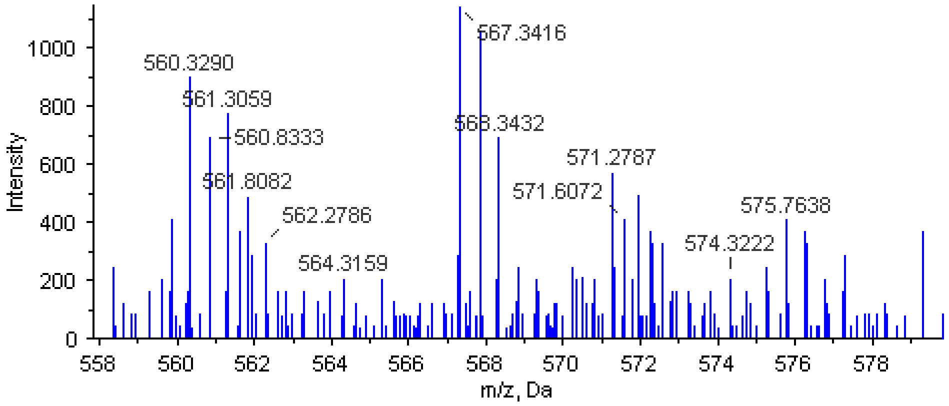

Sequence Coverage and Matched peptides: in different color

MKRITSTAAAAAASSSLSVTLHRTALHSPHLLAAIVVAAARVSLLHPSRATLFASFSSSSSSPLLSHIA
AMPGVDPQEMEWPAKKVRDTFIS**FFEDK**KHVHWKSSPVVPVNDPTLLFANAGMNQFK**PIFLGTADPNTA**
LSKLSRACNTQKCIRAGGK**HNDLDDVGK**DTYHHTFFEMLGNWSFGDYFKREAIGWAWELLTKIYKLPAD
RIYATYFGGDEKAGLAPDAEARDFWLQFLPPERVLPFGCKDNFWEMGDTGPCGPCTEIHYDRVGNRDAS
QLVNNDDPTCIEIWNLVFIQFNRESDGSLKPLPAK**HVDTGMGFERLTSVLQNK**MSNYDTDVFLPIFDAI
QQATGARPYSGKVGSDDVDRVDMAYRVVADHIR**TLSFAIADGSCPGNEGR**EYVLRRILRRAVRYGSEVL
KAQEGFFNGLVNIVVKVMGDVFPELIQNEVRIREIIKEEEASFGKTLLKGIEKFKKAAQEVQGKVSCLQ
DAFVLWDTYGFPLDLTQLMAEERGLW**VDVEGFNNAMDEAR**ERSRNAQNKQAGGDIIMDADATSALHKKG
VSVTDDSYKFIWFQDHESVIKAIYTGTEFLGSTTSSNEVGIVLESTSFYAEQGGQIFDTGSLEGTFGSF
QVSNVQIFGGFVVHIGSLTRAAPRLSVGDKVICKVDYDRRT**LIAPNHTCTHMLNFALREVLGNHVDQK**G
SIVLPEKLRFDFSHGKPVDPALLR**KIESIVNEQIKAELEVSAK**EATLSEAKRINGLR**AVFGEVYPDPVR**
VVAIGRKVEDLLADPENKEWSSISAELCGGTHISNTKEAK**AFALLSEEGIAK**GVRRITAVTTDGAFKAM
ELASLLEQEADEISKTEGSLLEKKVASLKSRVDSAPIPAAKKFDIRTKISALQDEVRKAQKKISEENMQ
KAVKIATEMAEVAASEGKQFCISR**VDVGLDAAAVR**EAVSKVLQKKGLSAMVFSIDETTNK**VVVCAGVAE**
KGDKSKQLEVSEWLTAALKPLNGRCGKGKGGLATGQGTEGVHLDAAMNLAAEFAQMKLR

Protein No.: I-76

Protein name and Species:

T-complex protein 1 subunit theta-like OS=Populus trichocarpa GN=POPTRDRAFT_823706 PE=3 SV=1

Accession: tr|B9I5Y4|B9I5Y4_POPTR

Unused ProtScore: 17.33

Seq Cov %: 21.8

ITRAQ Changed Ratio:

117: 115=E3TL: D3TL=1.72

116: 114=E5TL: D5TL=1.29

Annotated typical Precursor MS Region:

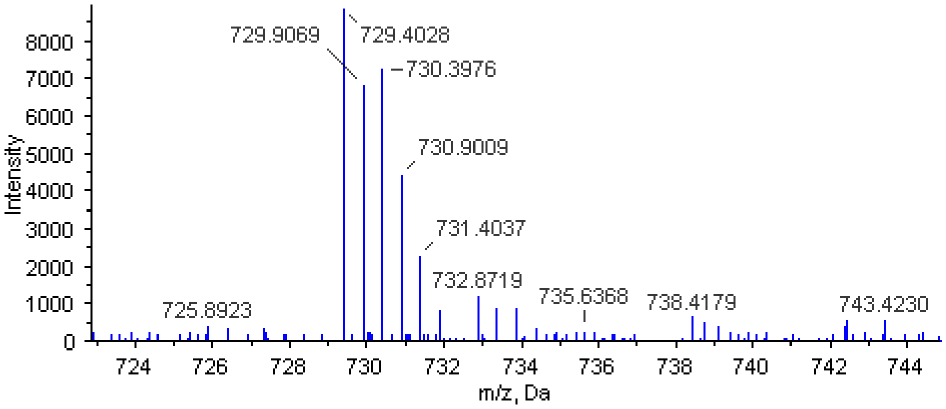

Sequence Coverage and Matched peptides: in different color

MGFSMQPYGMQSMLKEGHK**HLSGLDEAVLK**NI**DACKQLSAITR****TSLGPNGMNK****MVINHLDK****LFVTNDAA**
TIVNELEVQHPAAK**ILVLAGK**AQQEEIGDGANLTISFAGEMLQN**ADELIR****MGLHPSEIISGYNKAINK**A
IEILSELVEPGSENMDVRNKEQVICRMKAAVASKQFGQEDILCNLIADACIQVCPK**NPVNFNVDNVR**VA
KLVGGGLINSSIVRGMVLKGDTVGTIKRVEKAK**VAVFAGGVDTSATETKGTVLIHSAEQLENYAK**TEEA
K**VEELIK**AVAETGAKVIVSGAAVGEMALHFCERYK**LMVLK**ISSK**FELR**RFCR**TTGAVAILK**LSPPSPDD
LGCVDSISVEEIGGARVTIVR**NEEGGNSVCTVVLRGSTDSILDDLER****AVDDGVNTYK**AMCRDSRIIPGG
AATEIELAKRLR**EFSFK****ETGLDQYAIAK**FAESFEMIPKTLAENAGLNAMEIISSLYAEHASGNTK**VGID**
LEEGVFKDVSTTNIWDLYVTKFFALK**YAADAACTVLR**VDQIIMAKPAGGPRRDAAPGGMDED

Protein No.: I-77

Protein name and Species:

Cis-prenyl transferase II (Rubber transferase) OS=Hevea brasiliensis GN=Hpt3 PE=2 SV=1

Accession: tr|Q8L3U6|Q8L3U6_HEVBR

Unused ProtScore: 17.27

Seq Cov %: 42.2

ITRAQ Changed Ratio:

117: 115=E3TL: D3TL=2.23

116: 114=E5TL: D5TL=1.96

Annotated typical Precursor MS Region:

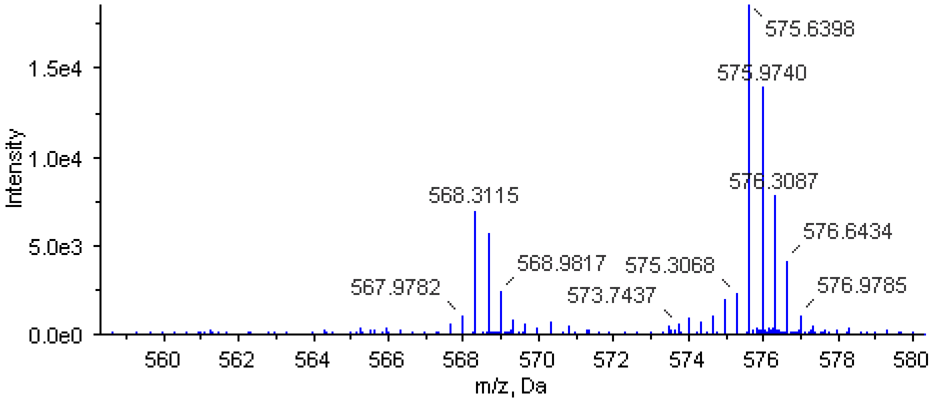

Sequence Coverage and Matched peptides: in different color

MEIYTGQRPSVFRIFGKYMRKGLYSILTQGPIPTHLAFIMDGNRRFAKKHKMKEAEGYKAGYLALLRTL
TYCYELGVRYVTIYAFSIDNFRRQPREVQCVMNLMMEKIEEIIVEESIMNAYDVGVRIVGNLNLLDEPI
RIAAEKIMRATANNSRFVLLIAVAYSSTDEIVHAVEESSKDKLNSNEVCNNGIEAEQEFKEANGTGNSV
IPVQKTESYSGINLADLEKNTYVNPHPDVLIRTSGLSRLSNYLLWQTSNCILYSPFALWPEIGLRHLVW
TVMNFQRHHSYLEKHKEYLK

Protein No.: I-80

Protein name and Species:

GTP-binding protein ras-like protein OS=Ricinus communis GN=RCOM_1012250 PE=3 SV=1

Accession: tr|B9S943|B9S943_RICCO

Unused ProtScore: 16.77

Seq Cov %: 39.6

ITRAQ Changed Ratio:

117: 115=E3TL: D3TL=0.47

116: 114=E5TL: D5TL=0.72

Annotated typical Precursor MS Region:

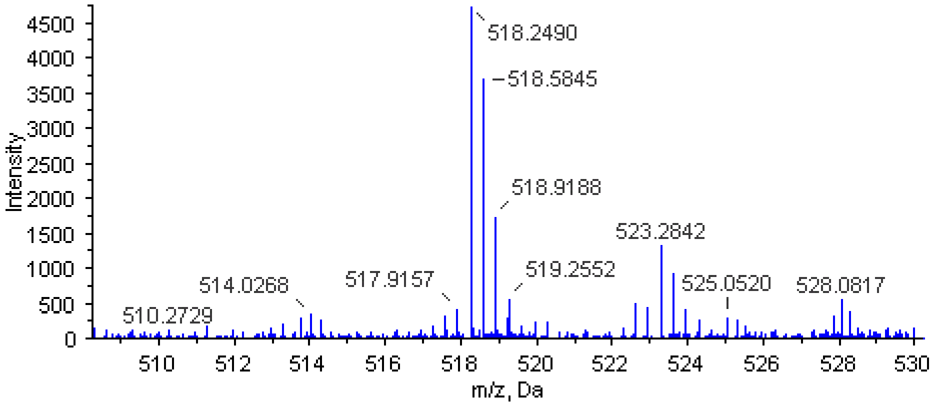

Sequence Coverage and Matched peptides: in different color

MGAYR**ADDDYDYLFKVVLIGDSGVGKSNLLSR**FTR**NEFSLESKSTIGVEFATR**SIHVDDKVVK**AQIWDT
AGQER**YR**AITSAYYRGAVGALLVYDVTRHVTFENVER**WLKELRDHTDANIVIMLVGNKADLRHLRAITI
EDATAFAQRQNTFFMETSALESMNVENAFTEVLTQIYRVVSR**KALDIGDDPAALPR**GQTINVGSRDDVS
AVKKAGCCSS

Protein No.: I-83

Protein name and Species: cullin-associated NEDD8-dissociated protein 1-like [Vitis vinifera] OS=Vitis vinifera GN=VIT_12s0035g01840 PE=4 SV=1

Accession: tr|F6HJZ9|F6HJZ9_VITVI

Unused ProtScore: 16.69

Seq Cov %: 7.1

ITRAQ Changed Ratio:

117: 115=E3TL: D3TL=0.23

116: 114=E5TL: D5TL=0.66

Annotated typical Precursor MS Region:

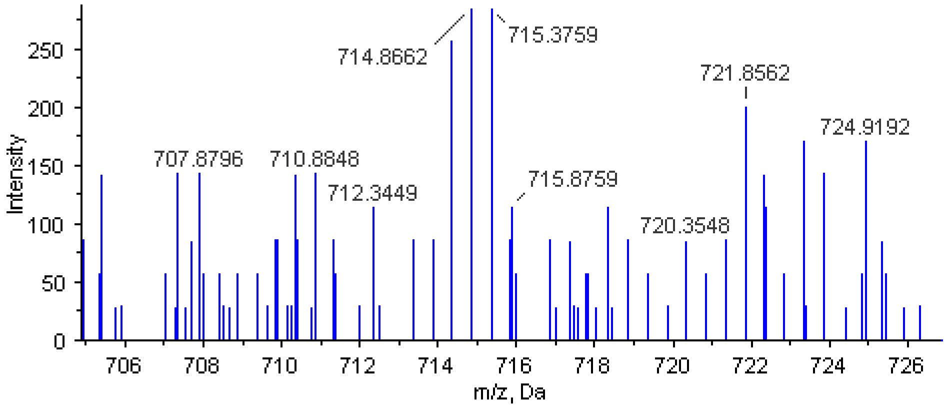

Sequence Coverage and Matched peptides: in different color

MANLAITSILEKMTGKDKDYRYMATSDLLNELNKEGFRADADLEIKLSNIVLQQLDDAAGDVSGLAVKC
LAPLVKKVSETRLVEMTNKLCDKLLNGKDQHRDIASIALKTIVSEVTTSAVAQCVLVSLSPQLIKGITS
PGMTTEMKCECLDILCDVLHKFGNLMATDHELLLGALLSQLSSNQASVRKKTVSCIASLASSLSDDLLA
KATVEVVRNLRSKGVKPEMTRTNIQMIGALSRAVGYRFGSHLGDTVPVLINYCTSASENDEELREYSLQ
ALESFLLRCPRDISSYCDEILHLTLEYLSYDPNFTDNMEEDTDDENHEEEEDDESATEYTDDEDVSWKV
RRAAAKCLAALIVSRPEMLSKLYEEACPKLIDRFKEREENVKMDVFNTFIELLRQTGNVTKGQTDMNEL
SPRWLLKQEVPKIVKSINRQLREKTIKTKVGAFSVLKELVVVLPDCLADHIGSLISGIEKALSDKSSTS
NLKIEALIFTRLVLASHSPSVFHPYIKALSSPVLSAVGERYYKVTAEALRVCGELVRVVRPNIEGYGFD
FKPYVHPIYNAIMTRLTNQDQDQEVKECAISCMGLLVSTFGDNLRAELPACLPVLVDRMGNEITRLTAV
KAFAVIATSPLNIDLSCVLEHVIAELTAFLRKANRALRQATLGTLNSLIVAYGDKIGSSAYEVIIVELS
SLISDSDLHMTALALELCCTLMADKRASPNVGLAVRNKVLPQALTLIKSSLLQGQALMALQNFFATLVY
SANTSFDALLDSLLSSAKPSPQSGGVAKQALCSIAQCVAVLCLAAGDQKCSTTVKMLTDILRDDSSSNS
AKQHLALLCLGEIGRRKDLSSHAHIENIVIESFQSPFEEIKSAASYALGNIAVGNLSKYLPFILDQIDN
QQKKQYLLLHSLKEVIVRQSVDKAEFQDSSVEKILKLLFNHCESEEEGVRNVVAECLGKIALIEPAKLV
PALKVRTASPAAFTRATVVIAVKYSIVERPEKIDEIIYPEISSFLMLIKDHDRHVRRAAVLALSTAAHN
KPNLIKGLLPELLPLLYDQTIVKQELIRTVDLGPFKHIVDDGLELRKAAFECVDTLLDSCLDQVNPSSF
IVPYLKSGLDDHYDVKMPCHLILSKLADKCPSAVLAVLDSLVDPLLKTINFKPKQDAVKQEVDRNEDMI
RSALRAIASLNRISGGDCSLKFKHLMNEISKSSTLWEKYHSIRNE

Protein No.: I-85

Protein name and Species:

Proteasome subunit alpha type OS=Ricinus communis GN=RCOM_0711430 PE=3 SV=1

Accession: tr|B9RR89|B9RR89_RICCO

Unused ProtScore: 16.45

Seq Cov %: 40.7

ITRAQ Changed Ratio:

117: 115=E3TL: D3TL=1.71

116: 114=E5TL: D5TL=1.12

Annotated typical Precursor MS Region:

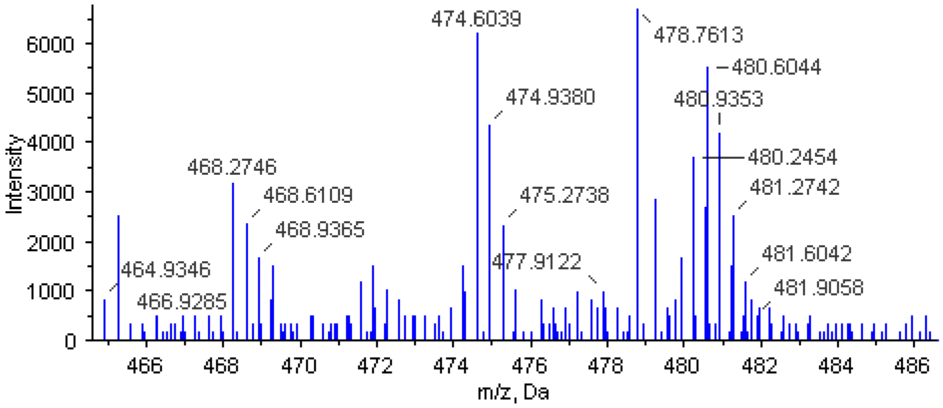

Sequence Coverage and Matched peptides: in different color

MSRGSGGGYDR**HITIFSPEGR**LFQVEYAFKAVK**AAAVTSIGVRGK**DSVCVVTQKKVPDK**LLDQTSVTHL**
FPITKYLGLLATGMTADARTLVQQAR**NEAAEFR**FRYGYEMPVDVLARWIADK**SQVYTQHAYMR**PLGVIA
MVLGIDEENGPQLYKCDPAGHFFGHKATSAGLK**EQEAINFLEK**KMKNDRAFTYEETVQTAISALQSVLQ
EDFK**ATEIEVGVVR**ADDRVFR**VLSTEEIDEHLTAISERD**

Protein No.: I-86

Protein name and Species:

Proteasome subunit alpha type OS=Glycine max PE=2 SV=1

Accession: tr|C6TKI4|C6TKI4_SOYBN

Unused ProtScore: 16.41

Seq Cov %: 37.5

ITRAQ Changed Ratio:

117: 115=E3TL: D3TL=2.07

116: 114=E5TL: D5TL=1.87

Annotated typical Precursor MS Region:

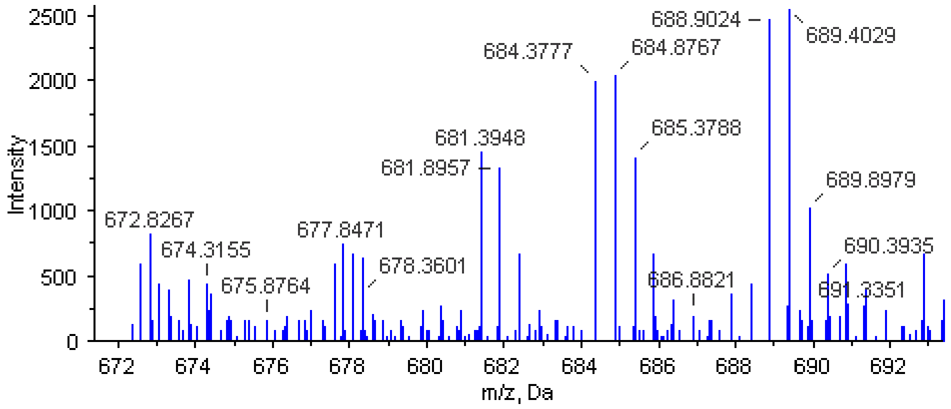

Sequence Coverage and Matched peptides: in different color

MGDSQYSFSLTTFSPSGKLVQIEHALTAVGSGQTSLGIKAANGVVIATEKKLPSILVDETSVQKIQLST
PNIGVVYSGMGPDFRVLVRKSRKQAEQYHRLYKEPIPVTQLVREVAAVMQEFTQSGGVRPFGVSLLVAG
FDDNGPQLYQVDPSGSYFSWKASAMGKNVSNAKTFLEKRYTDDMELDDAVHTSILTLKEGFEGQISGKN
IEIGIIGADKKFRVLTPAEIDDYLAEVE

Protein No.: I-87

Protein name and Species:

Glucose-6-phosphate isomerase OS=Ricinus communis GN=RCOM_1594100 PE=3 SV=1

Accession: tr|B9R7S8|B9R7S8_RICCO

Unused ProtScore: 16.33

Seq Cov %: 16.6

ITRAQ Changed Ratio:

117: 115=E3TL: D3TL=0.41

116: 114=E5TL: D5TL=0.26

Annotated typical Precursor MS Region:

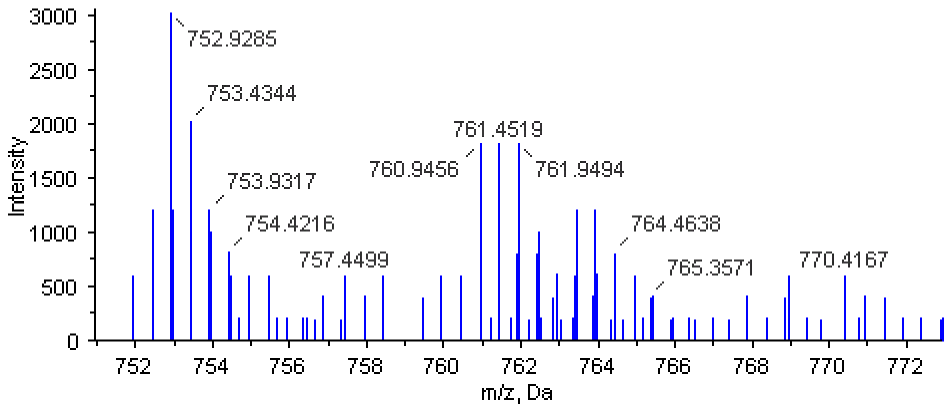

Sequence Coverage and Matched peptides: in different color

MALSALICDTQPWKDLKAHVEDIKKTHLRNLLGDTERCKSMLVEFDGIMLDYSRQTATIDTLDK**LYNLA**
EAAHLKEKIHRMFNGERINSTENR**SVLHVALR**APRDAIVNSDGK**NVVPDVWNVLDK**IKEFSERVRNGSW
VGATGKPLTDVIAIGIGGSFLGPLFVHTALQTDPEASKSAKGRQLRFLANVDPIDVARNIAGLSPETTL
VVVVSK**TFTTAETMLNAR**TLRAWISR**ELGPSAVAK**HMVAVSTNLTLVEKFGIDPNNAFAFWDWVGGRYS
VCSAVGVLPLSLQYGFSIVEKFLKGASSIDQHFSSAPFEKNIPVLLGLLSVWNVSVLGYPAR**AILPYSQ**
ALEKLAPHIQQVSMESNGKGVSMDGVPLPFETGEIDFGEPGTNGQHSFYQLIHQGR**VIPCDFIGVVK**SQ
QPVYLEGEVVNNHDELMSNFFAQPDALAYGKTAEQLQKENVPQHLIPHKTFSGNRPSVSILLSSLDAYK
IGQLLAIYEHRVAVQGFVWGINSFDQWGVELGKSLATQVRKQLNASR**TKGEPVEGFNFSTTTMLTRYLE**
ETSEVPAKPQTLLPRI

Protein No.: I-89

Protein name and Species:

26S proteasome regulatory subunit S3, putative OS=Ricinus communis GN=RCOM_0977830 PE=4 SV=1

Accession: tr|B9S5K6|B9S5K6_RICCO

Unused ProtScore: 16.31

Seq Cov %: 19.8

ITRAQ Changed Ratio:

117: 115=E3TL: D3TL=2.11

116: 114=E5TL: D5TL=1.37

Annotated typical Precursor MS Region:

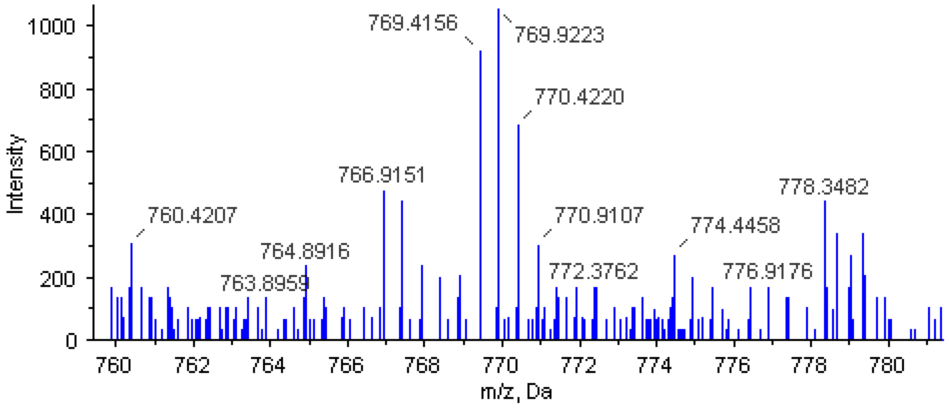

Sequence Coverage and Matched peptides: in different color

MTQDVEMKEVPAAAAAPSNSTTSSSPSTLQHLK**EIASLIETGAYAR**EVRRIVRAVRLTMALRRKLKAQL
LSAFLNSILTPGSDPYTRLSSFLPKEDDHEMEVDTATSVTQAPAKHPLPELEMYCYLLVLIYLIDQKKY
NEAKACSTACIARLKNLNRRTVDVLASRLYFYYSLSYELTGDLAEIRGNLLALHRIATLRHDELGQETL
LNLLLR**NYLHYNLYDQAEK**LRSKAPR**FEAHSNQQFCR**YLFYLGKIR**TIQLEYTDAK**ESLLQAARKSPVA
ALGFRVQCNKWAIIVRLLLGEIPERTVFMQKGMEKALRPYFELTNAVR**IGDLELFK**SVAEKFSTIFSAD
RTHNLIVRLRHNVIRTGLR**NISISYSRISLADVAK**KLR**LDSANPVADAESIVAK**AIRDGAIDATLDHAN
GWMVSK**ETGDIYSTNEPQIAFNSR**IAFCLNMHNEAVRALRFPPNSHKEKESAEKRRERQQQEQELAK**HI
AEEDDDEF**

Protein No.: I-95

Protein name and Species:

UDP-D-glucose pyrophosphorylase OS=Gossypium hirsutum PE=2 SV=1

Accession: tr|D2D2Z1|D2D2Z1_GOSHI

Unused ProtScore: 16.17

Seq Cov %: 27.3

ITRAQ Changed Ratio:

117: 115=E3TL: D3TL=0.47

116: 114=E5TL: D5TL=0.74

Annotated typical Precursor MS Region:

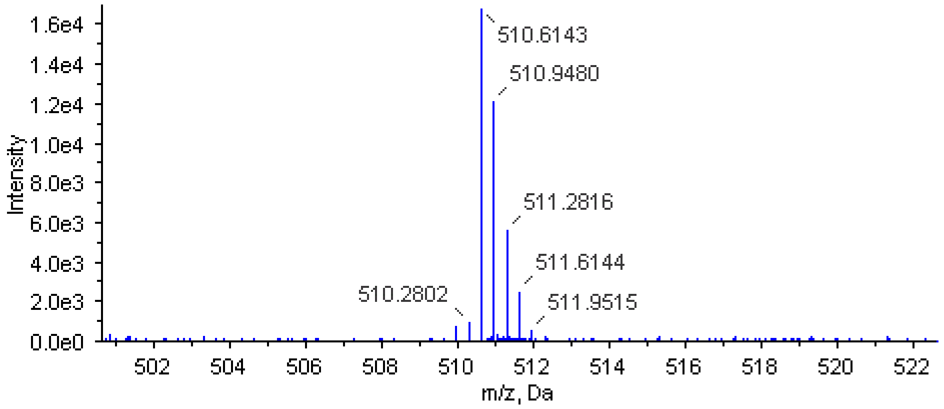

Sequence Coverage and Matched peptides: in different color

MEKLEHLKSAVAALSEISENEKNGFINLVSRYLSGEAQHIEWSKIQTPTDEVVVPYDTLSPSPDDPAET
KKLLDKLVVLKLNGGLGTTMGCTGPKSVIEVRNGLTFLDLIVIQIENLNSKYGCNVPLVLMNSFNTHDD
TLKIVDKYSNSNIEIHTFNQSQYPRLVVEDFAPLPSKGQHGKDGWYPPGHGDVFPSLMNSGKLDAFLSQ
GKEYVFVANSDNLGAIVDMKILNHLVQNKNEYCMEVTPKTLADVKGGTLISYEGKVQLLEIAQVPDEHV
NEFKSIEKFKIFNTNNLWVNLNAIKRLVEADALKMEIIPNPKEVNGIKVLQLETAAGAAIRFFDHAIGI
NVPRSRFLPVKATSDLLLVQSDLYTLVDGFVIRNKDRANPTNPSIELGPEFKKVGNFLSRFKSIPSIIE
LDSLKVTGDVWFGAGIVLKGKVSIAAKPGVKLEIPDGAVIEKKEINVPEDI

Protein No.: I-98

Protein name and Species:

Eukaryotic translation initiation factor 2c, putative OS=Ricinus communis GN=RCOM_0605410 PE=4 SV=1

Accession: tr|B9SJV6|B9SJV6_RICCO

Unused ProtScore: 15.62

Seq Cov %: 14.3

ITRAQ Changed Ratio:

117: 115=E3TL: D3TL=0.61

116: 114=E5TL: D5TL=0.59

Annotated typical Precursor MS Region:

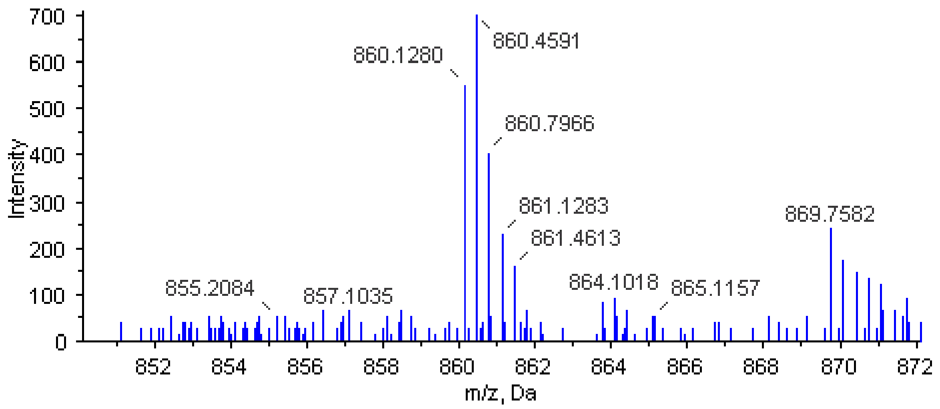

Sequence Coverage and Matched peptides: in different color

MVRKRRTEAPASGGESSEPHEAASGGSQRPYERNAPPQQGPGGPYQGGRSWGPQSQQGGRGGGGRGRSG
GMSQQQQYGGGPEYQGRGRGPPQQGGRGGYGGGRSSSNRGGPPSVGPSRPPVPELHQATLAPYQAGVSP
QLMPSEGSSSSGPPEPSPVVVAQQMQELSIQQEVSSSQPIQAPPPSSKSMRFPLRPGKGSTGIRCIVKA
NHFFAELPDKDLHQYDVTITPEVTSRGVNRAVMEQLVKLYRESHLGK**RLPAYDGR**K**SLYTAGPLPFISK**
EFKITLIDEDDGSGGQRREREFRVVIKLAARADLHHLGLFLQGR**QADAPQEALQVLDIVLR**ELPTTRYC
PVGR**SFYSPDLGR**RQPLGEGLESWRGFYQSIRPTQMGLSLNIDMSSTAFIEPLPVIDFVNQLLNR**DVSS**
RPLSDADRVKIKKALRGVKVEVTHRGNMRRKYRISGLTSQATR**ELTFPVDER**GTMKSVVEYFYETYGFV
IQHTQWPCLQVGNQQRPN**YLPMEVCK**VVEGQRYSKRLNERQITALLKVTCQRPQERERDIMQTVHHNAY
GNDPYAKEFGIKISEKLASVEARILPAPWLKYHDTGREKDCLPQVGQWNMMNKKMVNGGTVNNWICINF
SRNVQDSVARGFCYELAQMCY**ISGMAFNPEPVLPPVSARPEQVEK**VLKTRYHDAMTKLQQGKELDLLIV
ILPDNNGSLYGELKR**ICETDLGLVSQCCLTK**HVFRMNKQYLANVALKINVKVGGRNTVLVDALSRRIPL
VSDRPTIIFGADVTHPHPGEDSSPSIAAVVASQDWPEVTK**YAGLVCAQAHRQELIQDLFK**EWQDPVRGR
VTGGMIK**ELLISFR**RATGQKPQRIIFYRDGVSEGQFYQVLLYELDAIRK**ACASLEPNYQPPVTFVVVQK**
RHHTRLFANNHNDRNAVDKSGNILPGTVVDSKICHPTEFDFYLCSHAGIQGTSRPAHYHVLWDENKFTA
DGLQSLTNNLCYTYARCTR**SVSIVPPAYYAHLAAFR**ARFYMEPETSDSGSMTSGPVGGRGGMGGGAGAR
STRGP**AASAAVRPLPALK**ENVKRVMFYC

Protein No.: I-99

Protein name and Species:

Coatomer subunit beta OS=Vitis vinifera GN=VIT_04s0008g02320 PE=3 SV=1

Accession: tr|A5AHP0|A5AHP0_VITVI

Unused ProtScore: 15.49

Seq Cov %: 11.9

ITRAQ Changed Ratio:

117: 115=E3TL: D3TL=1.80

116: 114=E5TL: D5TL=1.49

Annotated typical Precursor MS Region:

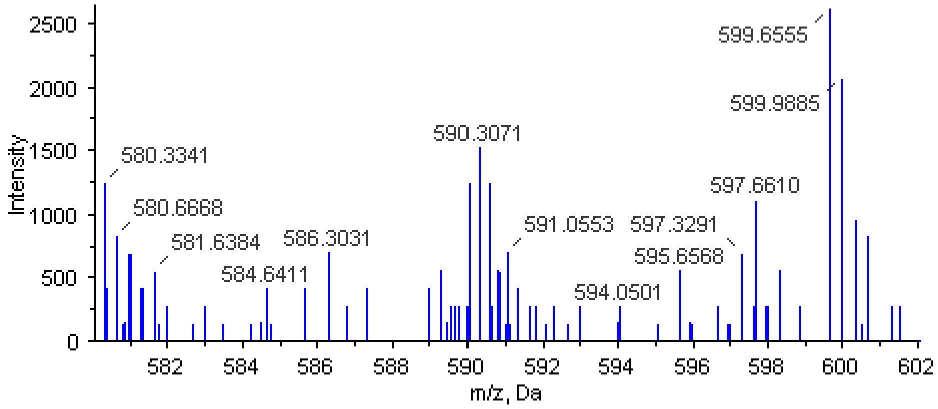

Sequence Coverage and Matched peptides: in different color

MEKSCSLLIYFDK**GTPAIANEIK**EALEGNDDYAKIEAMKKAIMLLLNGETLPQLFITIVRYVLPSEDHT
VQKLLLLYLEIIEKTDAKGKVMPEMILICQNLR**NNLQHPNEYIR**GVTLRFLCRLNEAEIIEPLIPSVLQ
NLEHRHPFIRRNAILAVMSIYK**LPQGEQLLVDAPEMIEK**VLSTEQDPSAKR**NAFLMLFTCAQDRAINYL**
LTHVDRVPEWGELLQMVV**LELIR**KVCRTNRGEKGKYIKIIISLLNAPSTAVIYECAGTLVSLSSAPTAI
R**AAANTYCQLLLSQSDNNVK**LIVLDRLNELKSSHREIMVDMIMDVLRALSSPNLDIRRKTLDIVLELIT
PRNINEVVLTLKKEVVKTQSGELEKNGEYRQMLIQAIHSCAIKFPEVASTVVHLLMDFLGDSNVASAID
VVVFVREIIETNPKLRVSIITR**LLDTFYQIR**AARVCSCALWIIGEYCLSLSEVESGITTIKQCLGDLPF
FSVSEEGEASDSSKKVQQVNATTVSSRRPAVLADGTYATQSAASETAFSPPTLVQGSLSSGNLRSLLLT
GDFFLGAVVACTLTKLVLRLEEVQPSKAEVNKVSSQALLIMVSMLQLGQSSVLPHPIDNDSYDRIVLCI
RLLCNTGDDIRKIWLQSCRQSYVKMLADKQLRETEEIKAKAQISYAQPDDLIDFYHLKSRK**GMSQLELE**
DEVQDDLKRATGEFIKDGDDANKLNRILQLTGFSDPVYAEAYVTVHHYDIVLDVTVINRTKETLQNLCL
ELATMGDLK**LVDRPQNYTLAPESSK**QIKANIKVSSTETGVIFGNIVYETSNVHERMVVVLNDIHIDIMD
YISPAVCTDVAFRTMWAEFEWENKVAVNTVLQNEKEFLEHIIKSTNMKCLTASSALDGDCGFLAANLYA
KSVFGEDALVNISIEKQADGKLSGYIRIRSK**TQGIALSLGDK**ITLKQKGGS

Protein No.: I-102

Protein name and Species:

Nucleoredoxin, putative OS=Ricinus communis GN=RCOM_0528050 PE=4 SV=1

Accession: tr|B9SH99|B9SH99_RICCO

Unused ProtScore: 15.18

Seq Cov %: 18.4

ITRAQ Changed Ratio:

117: 115=E3TL: D3TL=0.42

116: 114=E5TL: D5TL=0.59

Annotated typical Precursor MS Region:

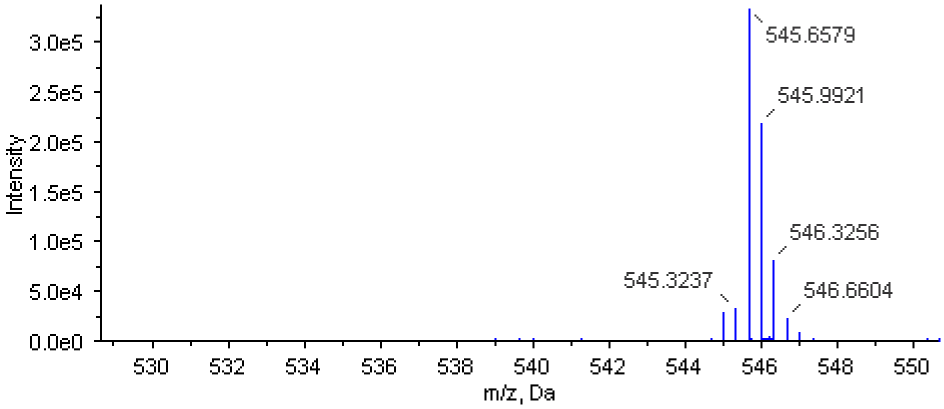

Sequence Coverage and Matched peptides: in different color

MADDPVIDVSHDLLSLLSSDDRDFLIRSNGDQVKISNLVGKIVGLYFSGSWCGPCRHFTPNLVQVYEEL
SLKGDFEVVFISSDRDAESFDAYFSKMPWLAIPFSDQETCKHLKDLFKVRGIPNLVFLDADGKVSCDQG
VRFIREYGAEGYPFTPERVEYFRQEEENAKKNQTLSSILVSSSRDFLISKDGTKIPVSELEGKMVGLYF
SVHSHRLCLDFTPRLEEVYKKLKEKGEKFEVVLISMDYDENNFKQGLETMPWLALPFEDKSRERLARYF
ELSALPTLVIIGEDGKTLNKNVAELIEGHGIQAYPFTPEKLVELAEIEKARLEAQTLESVLVHGDKDFV
IEESGSKVPVSELVGKNILLYFSAKWCPPCRAFLPKLIEAYHEIKAKDNAFEIIFISSDRDQSSFDEFY
TEMPWLALPFGDDRKTILQRKFKIKGIPAAIAISPTGKTLTKEAREHITAYGADAYPFNEDHLKQLNDK
QEEIAKGWPEKVRHELHPEHELVRMKRNGYGCDGCKEAGSGWSFYCKKCDFDLHPKCALKKEENGEKVK
K

Protein No.: I-106

Protein name and Species:

L-lactate dehydrogenase OS=Ricinus communis GN=RCOM_0540790 PE=3 SV=1

Accession: tr|B9SIT3|B9SIT3_RICCO

Unused ProtScore: 14.96

Seq Cov %: 32.3

ITRAQ Changed Ratio:

117: 115=E3TL: D3TL=0.44

116: 114=E5TL: D5TL=0.76

Annotated typical Precursor MS Region:

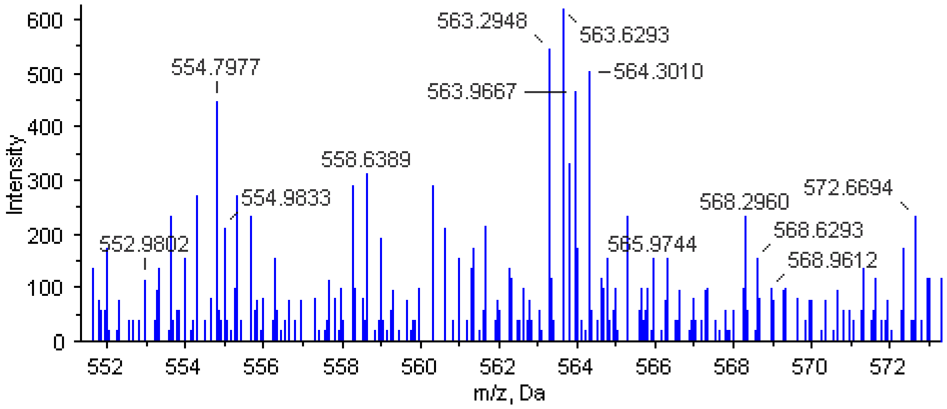

Sequence Coverage and Matched peptides: in different color

MQQSSSAASLGPGGLDLSQVFFKPIHNTSPPSPTKRHTKISVIGTGNVGMAIAQTILTQDLADELALVD
VNPNKLR**GEMLDLQHAAAFLPR**TKILASPDYSVTIGSDLCIVTAGARQIPGESR**LNLLQR**NVTLFKNII
PPLAKYSPDSILMIVSNPVDILTFIAWK**LSGFPSNR**VVGSGTNLDSSRFRFLIADHLDVNAQDVQAYIV
GEHGDSSVALWSSISVGGVPILSFLEKQDIAYEK**ETLENIHKAVVDSAYEVISLKGYTSWAIGYSAANL**
ARSLLRDQRK**IHPVSVLAKGFYGIDGGDVFLSLPAQLGRGGVLGVTNVHLTEEEAQR**LRDSAKTLLEVQ
SQLGI

Protein No.: I-108

Protein name and Species:

26S proteasome regulatory subunit 4 homolog A [Vitis vinifera] OS=Vitis vinifera GN=VIT_07s0005g05160 PE=3 SV=1

Accession: tr|A5BEF3|A5BEF3_VITVI

Unused ProtScore: 14.87

Seq Cov %: 27.6

ITRAQ Changed Ratio:

117: 115=E3TL: D3TL=1.51

116: 114=E5TL: D5TL=1.47

Annotated typical Precursor MS Region:

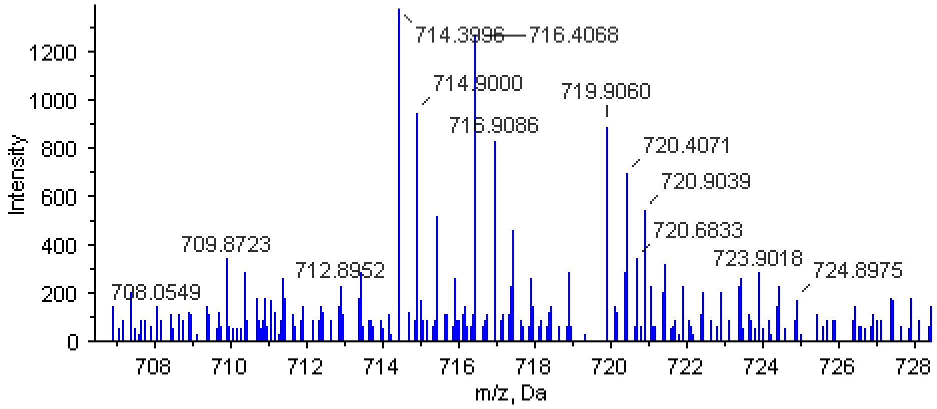

Sequence Coverage and Matched peptides: in different color

MGQGASGGLNRQGLPGDRKNDGDKKEK**KFEPAAPPAR**VGRKQRKQKGPEAAARLPTVTPLSKCKLRLLK
LERIKDYLLMEEEFVSNQERLKPQEEKTEEDRSKVDDLRGSPMSVGNLEELIDENHAIVSSSVGPEYYV
GILS**FVDK**DQLEPGCAILMHNKVLSVVGLLQDEVDPMVSVMKVEK**APLESYADIGGLDAQIQEIK**EAVE
LPLTHPELYEDIGIKPPK**GVILYGEPGTGK**TLLAK**AVANSTSATFLRVVGSELIQK**YLGDGPKLVRELF
R**VADDLSPSIVFIDEIDAVGTK**RYDAHSGGEREIQR**TMLELLNQLDGFDSR**GDVKVILATNR**IESLDPA**
LLRPGRIDRKIEFPLPDIKTRRR**IFQIHTSRMTLADDVNLEEFVMTKDEFSGADIKAICTEAGLLALR**E
RRMKVTHVDFKKAKEKVMFKK**KEGVPEGLYM**

Protein No.: I-109

Protein name and Species:

Chaperonin containing t-complex protein 1, eta subunit, tcph, putative OS=Ricinus communis GN=RCOM_0395630 PE=3 SV=1

Accession: tr|B9SS36|B9SS36_RICCO

Unused ProtScore: 14.75

Seq Cov %: 17.6

ITRAQ Changed Ratio:

117: 115=E3TL: D3TL=0.55

116: 114=E5TL: D5TL=0.72

Annotated typical Precursor MS Region:

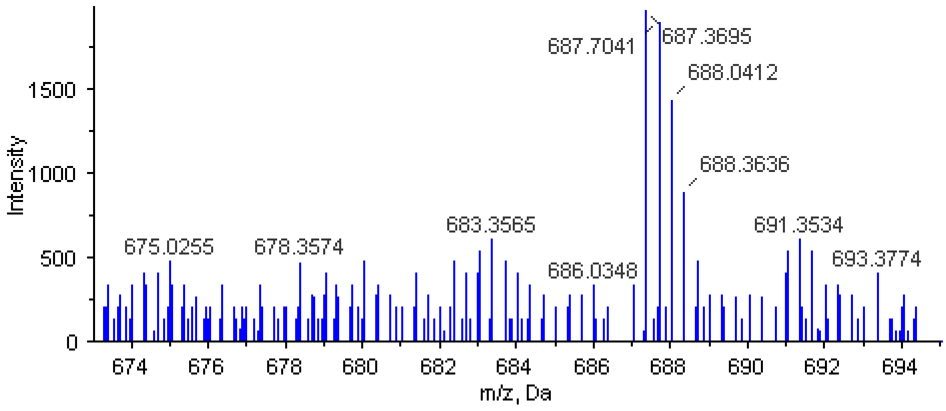

Sequence Coverage and Matched peptides: in different color

MAAMLQPQIILLKEGTDTSQGKAQLVSNINACTAVADVVRTTLGPRGMDKLIHDDKGNVTISNDGATIM
KLLDIVHPAAKILVDIAKSQDSEVGDGTTTVVLLAAEFLKEAKPFIEDGVHSQNLIRSYRTACNLALEK
IRELAFSIEGKSIEEKKSLLAKCASTTLSSKLIGGEKEFFAQMVVDAVIAIGNEDRLNMIGIKKVPGGN
MRDSFLVNGVAFKKTFSYAGFEQQPKKFVNPKLLLLNIELELKSEKENAEIRLSDPSEYQSIVDAEWNI
IYDKLDKCVQSGAKVVLSRLAIGDLATQYFADRDIFCAGRVAEEDLQRVAAATGGTVQTTVNNIIDEVL
GTCEIFEEKQVGNERFNIFSGCPSGKTATIVLRGGADQFIEEAERSLHDAIMIVRRAMKNSTVVAGGGA
IDMEISRFLRQHARTIAGKSQLFINSYAKALEVIPRQLCDNAGFDATDVLNKLRQKHALPSGEGAPFGV
DINTGGIADSFANFVWEPAVVKINAINAATEAACLILSVDETVKNPKSESAQGEAAAGAMGGRGRGGAA
FRGRGRGMRRR

Protein No.: I-112

Protein name and Species:

Lactoylglutathione lyase, putative OS=Ricinus communis GN=RCOM_0904280 PE=4 SV=1

Accession: tr|B9RXK1|B9RXK1_RICCO

Unused ProtScore: 14.47

Seq Cov %: 30

ITRAQ Changed Ratio:

117: 115=E3TL: D3TL=0.08

116: 114=E5TL: D5TL=0.10

Annotated typical Precursor MS Region:

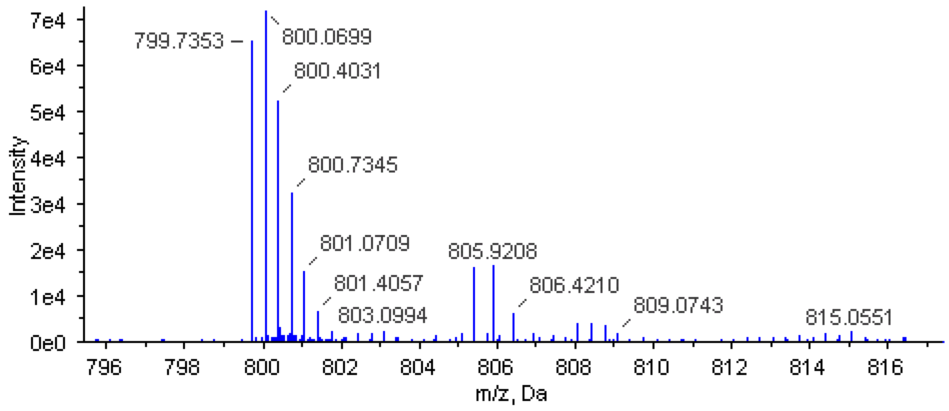

Sequence Coverage and Matched peptides: in different color

MAAEATAPNADVLEWPKKDKRRLLHAVYRVGDLDRTIKFYTECFGMKLLRKRDIPEEKYSNAFLGFGPE
ETNFVVELTYNYGVTSYDIGTGFGHFAIATQDVYKLVEEVLAKGGAVTREPGPVKGGTTVIAFVKDPDG
YIFELIQRGPTPEPLCQVMLRVGDLDRSIRFYEKALGMKLLRKVDKPEYKYTLAMMGYADEYETTVLEL
TYNYGVTEYTKGNAYAQVAISTDDVYKSAEVVNLVTQELGGKITRQPGPIPGLNTKITSFLDPDGWKTK
RKRR

Protein No.: I-115

Protein name and Species:

Glucose 4-6-dehydratase, putative OS=Ricinus communis GN=RCOM_1185970 PE=4 SV=1

Accession: tr|B9SN65|B9SN65_RICCO

Unused ProtScore: 14.31

Seq Cov %: 28.7

ITRAQ Changed Ratio:

117: 115=E3TL: D3TL=3.10

116: 114=E5TL: D5TL=1.52

Annotated typical Precursor MS Region:

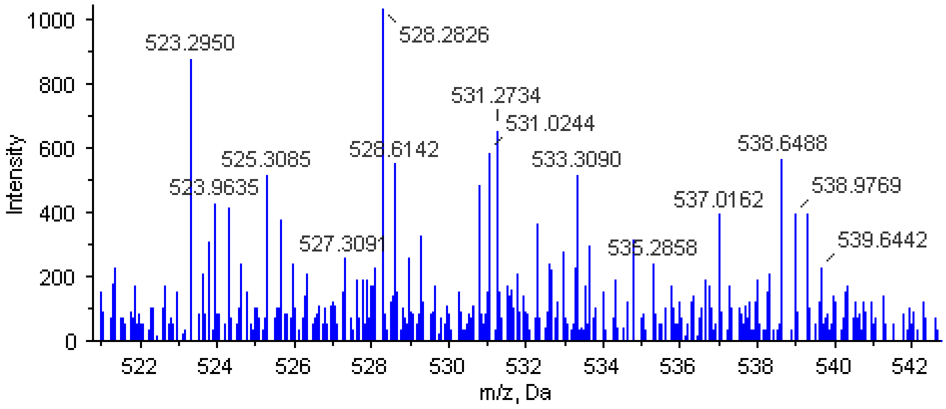

Sequence Coverage and Matched peptides: in different color

MANR**VDLDGR**PIKEMTIC**MIGAGGFIGSHLCEK**ILNETPHK**ILALDVYNDK**IKHLLEPDSLPWAGRIQF
HRINIKHDSR**LEGLIK****MADLTINLAAICTPADYNTR**PLDTIYSNFIDA**LPVVK**YCSENNKRLIHFSTXX
SYFSWDPAYYILKEDESPCIFGSIEKQRWSYACAKQLIERLIYAEGAENGLEFTIVRPFNWIGPR**MDFI
PGIDGPSEGVPR**VLACFSNALLRGEPLKLVDGGQSQR**TFVYIK****DAIEAVLLMIENPARANGHIFNVGNP
HNEVTVK**QLAEMMTEVYSKVSGEPVLEVPTVDISSK**EFYGEGYDDSDKR****IPDMTIINR**QLGWNPKTSLW
DLLESTLTYQHRTY**AEAIK**KVLAKPTSS

Protein No.: I-117

Protein name and Species:

Regulator of ribonuclease activity A OS=Populus trichocarpa GN=POPTRDRAFT_738638 PE=2 SV=1

Accession: tr|A9PFQ6|A9PFQ6_POPTR

Unused ProtScore: 14.13

Seq Cov %: 42.8

ITRAQ Changed Ratio:

117: 115=E3TL: D3TL=0.30

116: 114=E5TL: D5TL=0.37

Annotated typical Precursor MS Region:

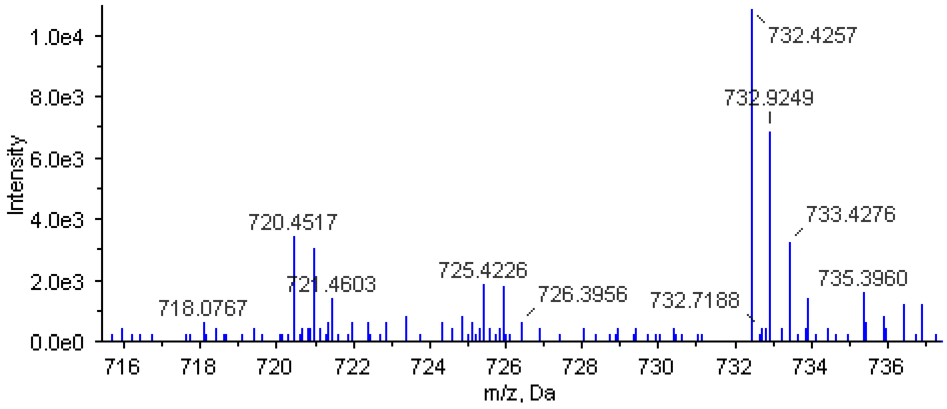

Sequence Coverage and Matched peptides: in different color

MALVTTAEVCDANPQLIVSGELR**ALQPIFQIYGRRQVFSGPVVTLKVFEDNVLIR****EFLEEK**GNGR**VLVVDGGGSLR**CAILGGNPVVQAQNNGWAGIVVNGCIRDVDEIMGCDIGVRALASHPMKANKKGIGEK**HVPITIGGTRISDGEWLYADTDGILISR**TELAV

Protein No.: I-119

Protein name and Species:

Hydroxymethylglutaryl coenzyme A reductase OS=Hevea brasiliensis PE=2 SV=1

Accession: tr|Q547W6|Q547W6_HEVBR

Unused ProtScore: 14.02

Seq Cov %: 22.1

ITRAQ Changed Ratio:

117: 115=E3TL: D3TL=0.22

116: 114=E5TL: D5TL=0.28

Annotated typical Precursor MS Region:

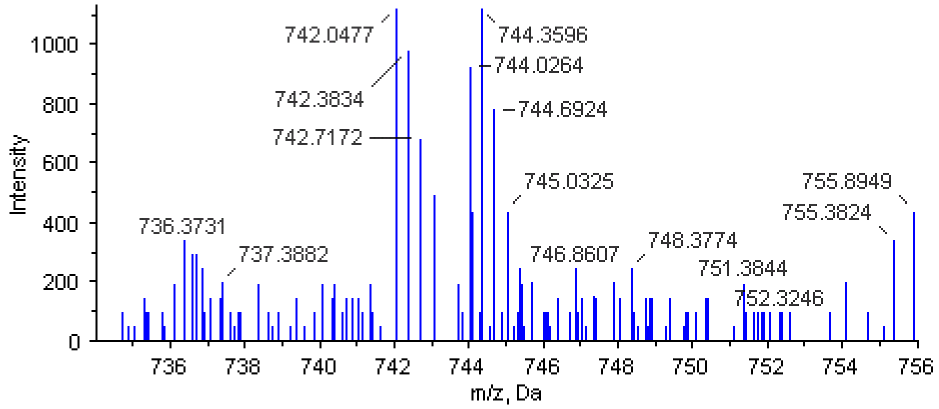

Sequence Coverage and Matched peptides: in different color

MDTTGRLHHRKHATPVEDRSPTTPKASDALPLPLYLTNAVFFTLFFSVAYYLLHRWRDKIRNSTPLHIV
TLSEIVAIVSLIASFIYLLGFFGIDFVQSFIARASHDVWDLEDTDPNYLIDEDHR**LVTCPPANISTKTT**
IIAAPTKLPTSEPLIAPLVSEEDEMIVNSVVDGKIPSYSLESKLGDCKRAAAIRREALQRMTRRSLEGL
PVEGFDYESILGQCCEMPVGYVQIPVGIAGPLLLNGR**EYSVPMATTEGCLVASTNR**GCK**AIYLSGGATS**
VLLKDGMTRAPVVRFASATRAAELKFFLEDPDNFDTLAVVFNKSSRFARLQGIKCSIAGKNLYIR**FSCS**
TGDAMGMNMVSKGVQNVLEFLQSDFSDMDVIGISGNFCSDKKPAAVNWIEGRGKSVVCEAIIKEEVVKK
VLK**TNVASLVELNMLKNLAGSAVAGALGGFNAH**AGNIVSAIFIATGQDPAQNVESSHCITMMEAVNDGK
DLHISVTMPSIEVGTVGGGTQLASQSACLNLLGVKGANKESPGSNSRLLAAIVAGSVLAGELSLMSAIA
AGQLVKSHMKYNRSSKDMSKAAS

Protein No.: I-122

Protein name and Species:

villin-2 OS=Populus trichocarpa GN=POPTRDRAFT_576360 PE=4 SV=1

Accession: tr|B9II82|B9II82_POPTR

Unused ProtScore: 13.95

Seq Cov %: 13.5

ITRAQ Changed Ratio:

117: 115=E3TL: D3TL=0.58

116: 114=E5TL: D5TL=1.19

Annotated typical Precursor MS Region:

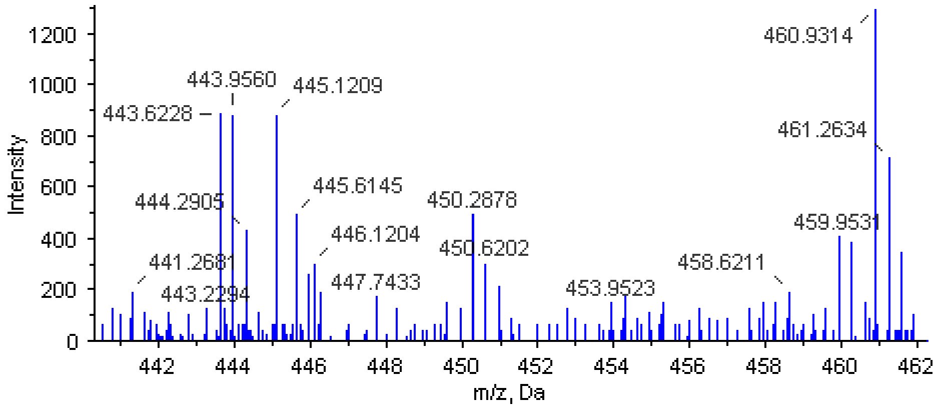

Sequence Coverage and Matched peptides: in different color

MSSSAKALDPAFQGVGQRPGTEIWRIENFQPVPLPKSDHGKFYMGDSYIVLQTTTGKGGAYLYDIHFWI
GKDTSQDEAGTAAIKTVELDAVLGGRAVQHRELQGHESDKFLSYFKPCIIPLEGGVATGFKKVEEEAFE
IRLYVCRGKRVVRLKQVPFARSSLNHDDVFILDTEKKIYQFNGANSNIQERGKALEVIQFLKEKYHEGT
CDVAIVDDGKLDTESDSGEFWVLFGGFAPIGKKVANEDDIIPETTPAKLYSITDGEVKIVEGELSKGLL
ENNKCYLLDCGAEIFVWVGRVTQVEERKAASQAAEEFVASQNRPKTTQLTRLIQGYETRSFKTNFDSWP
AGSAAPGAEEGRGKVAALLKQQGVGLKGMTKSAPVNEEVPPLLEGGGKMEVWCINGSSKTPLPKEDVGK
FYSGDCYIILYTYHSGDRKEDYLLCCWFGNDSSEIHGHPIQEDQKMAARLANTMSNSLKGRPVQGRIFQ
GKEPPQFVALFQPIVILKGGLSSGYKKSIAEKGLSDETYTADSVALFRISGTSVHNDKAVQVDAVATSL
NSAECFLLQSGSSIFTWHGNQSTFEQQQLAAKIAEFLKPGVALKHAKEGTESSAFWFALGGKQSYTSKK
FSPETVRDPHLFTFSFNKGKFQVEEVYNFSQDDLLTEDILILDTHAEVFVWVGQYVDPKEKQNVFDIGQ
KYIEMAVSLDGLSPNVPLYKVTEGNEPSFFTTYFSWDLTKATVQGNSFQKKAALLFGLGHHVVEERSNG
NQGGPTQRASALAALSSAFNPSSGKSSLLDRSNGSNQGGTTQRASALAALSSAFNSSPGSKTTASRPSG
TGQGSQRRAAVAALSSVLTAEKKQTPETSPSRSPPSETNLPEGSEGVAEVKEMEETASVSESNGGEDSE
RKQDTEHGESDDGNGQSTFCYDQLKAHSDNPVKGIDFKRREAYLSDEEFQTIFGVTKEAFYKMPKWKQD
MQKKKFDLF

Protein No.: I-125

Protein name and Species:

Cysteine synthase OS=Vitis vinifera GN=VIT_14s0030g01560 PE=3 SV=1

Accession: tr|F6HTU8|F6HTU8_VITVI

Unused ProtScore: 13.43

Seq Cov %: 11.8

ITRAQ Changed Ratio:

117: 115=E3TL: D3TL=0.17

116: 114=E5TL: D5TL=0.40

Annotated typical Precursor MS Region:

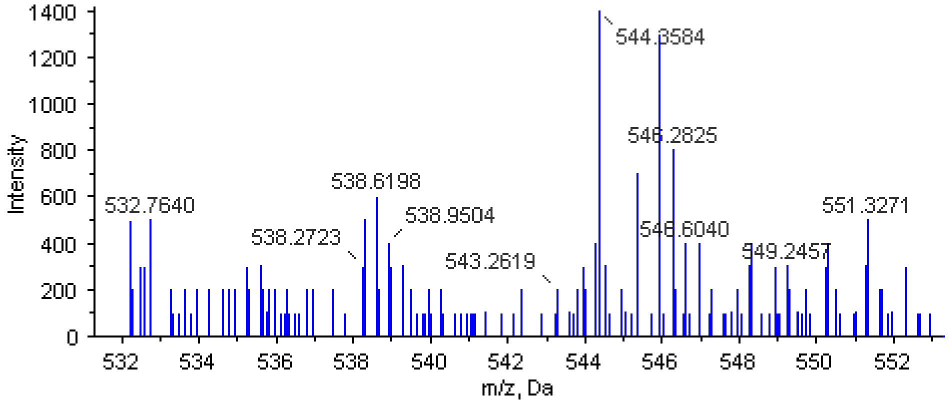

Sequence Coverage and Matched peptides: in different color

MIQKKSELILIYNIKLGNLILNLILFVRFIQEKQFAILPSLFIKVVSVETVGDVQKPQNRKKKQRIRKG
DLVVGISGDLCEMAEEKCFIAK**DVTELIGK**TPLVYLNNVVDGCVARIAAK**LEMMEPCSSVK**DRIGYSMI
TDAEEKGLITPGESVLIEPTSGNTGIGLAFMAAAKGYK**LIITMPASMSLER**RIILRAFGAELVLTDPAK
GMKGAVQK**AEEILAKTPNSYILQQFENPANPK**IHYETTGPEIWRGTGGKVDAFVSGIGTGGTITGAGKF
LKEQNSDIK**LYGVEPVESAVLSGGKPGPHK**IQGIGAGFIPGVLDVNLLDEVVQVSSEEAIETAK**LLALK**
EGLLVGISSGGAAAAAIKLAK**RPENAGKLIVVVFPSFGER**YLSSVLFDSVKREAENMLKKKHGGQMLDC
KRCYRTSIAVKLEAMEPCSSVKDRIALSMIKDVEDKGLIQLGKTVLIEPTSGNSGIELAYIAAVKGYKL
ILAMPASMSRERRIVLRAFGDELHLTDPVKGIKGIHEKAELLEKTPYGYMLKQFANPANPKIHYETTGT
EIWRGCGGKVDVFVSGVGTGGTVTGVGRFLKEKNPDIKVYGVEPAESAVLNGGEPGLHKIQGIDPGFIT
PILDVSILDEVVPISSEEAIETAK**LLALK**EGLLVGKPSGVAAAAAIKIAK**RPENAGKLIVVVFPSFGER**
YLSTVLFDSIR

Protein No.: I-129

Protein name and Species:

Proteasome subunit alpha type OS=Glycine max PE=2 SV=1

Accession: tr|C6TGU2|C6TGU2_SOYBN

Unused ProtScore: 13.13

Seq Cov %: 39.2

ITRAQ Changed Ratio:

117: 115=E3TL: D3TL=0.52

116: 114=E5TL: D5TL=0.66

Annotated typical Precursor MS Region:

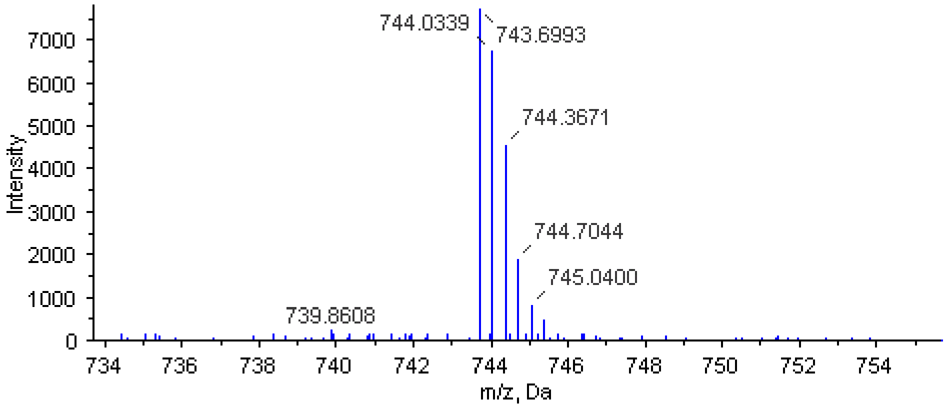

Sequence Coverage and Matched peptides: in different color

MFLTRTEYDRGVNTFSPEGRLFQVEYAIEAIKLGSTAIGLKTKEGVVLAVEKRITSPLLEPSSVEKIME
IDEHIGCAMSGLIADARTLVEHARVETQNHRFSYGEPMTVESTTQALCDLALRFGEGDEESMSRPFGVS
LLIAGHDENGPSLYYTDPSGTFWQCNGKAIGSGSEGADSSLQEQYNKDLTLQEAETIALSILKQVMEEK
VTPNNVDIAKVAPTYHLYTPSEVEAVISRL

Protein No.: I-131

Protein name and Species:

40S ribosomal protein SA OS=Glycine max PE=2 SV=1

Accession: sp|O22518|RSSA_SOYBN

Unused ProtScore: 12.95

Seq Cov %: 31.6

ITRAQ Changed Ratio:

117: 115=E3TL: D3TL=2.47

116: 114=E5TL: D5TL=1.62

Annotated typical Precursor MS Region:

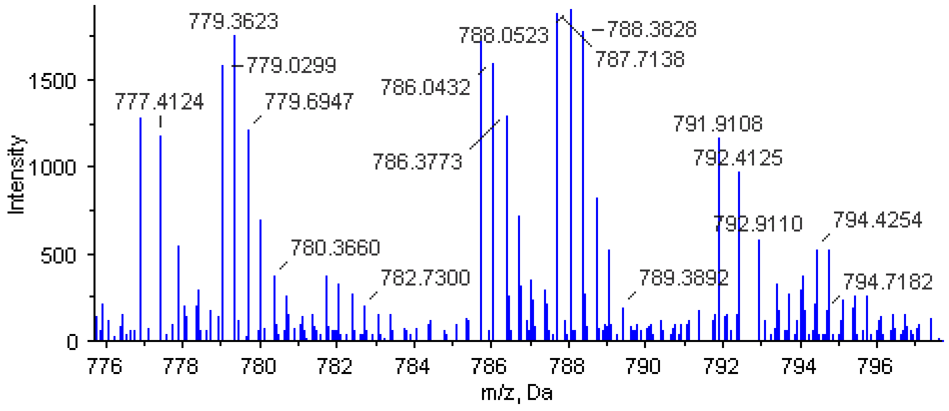

Sequence Coverage and Matched peptides: in different color

MATATNAAAAPPRQLSQKEADIQMMLAADVHLGTKNCDFQMERYIFKRRNDGIYIINLGKTWEKLQLAA
RVIVAIENPQDIIVQSARPYGQRAVLKFAQYTGAHAIAGRHTPGTFTNQLQTSFSEPRLLILTDPRTDH
QPIKEAALGNIPTIAFCDTDSPMRYVDIGIPANNKGKHSIGCLFWLLARMVLQMRGTIRPGLKWDVMVD
LFFYREPEEAKQQEEEEAPAVDYAITDFNAGAIAADGQWPGTIDQSWSDAVPQPIPAVPGVNWGAPAEA
PAAAGGDWGEAVPPPQQIPVPPSGIDTVQPSGWD

Protein No.: I-132

Protein name and Species:

UDP-D-glucose dehydrogenase OS=Gossypium hirsutum PE=2 SV=1

Accession: tr|D2D2Z2|D2D2Z2_GOSHI

Unused ProtScore: 12.91

Seq Cov %: 19.8

ITRAQ Changed Ratio:

117: 115=E3TL: D3TL=2.27

116: 114=E5TL: D5TL=1.66

Annotated typical Precursor MS Region:

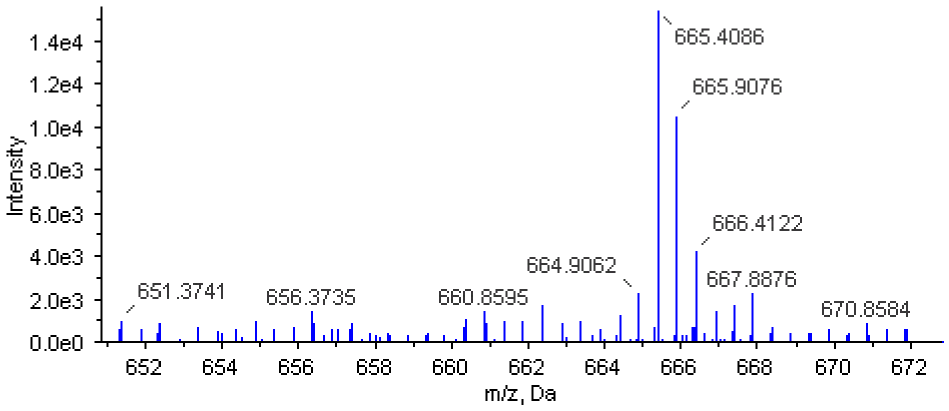

Sequence Coverage and Matched peptides: in different color

MK**ICCIGAGYVGGPTMAVIALK**CPDIEVAVVDISVPRIAAWNSDQLPIYEPGLDGVVKECRGRNLFFST
DVEKHVREADIVFVSVNTPTKTRGLGAGKAADLTYWESAARMIADVSKSDKIVVEKSTVPVKTAEAIEK
ILTHNSKGIKFQILSNPEFLAEGTAIQDLFNPDRVLIGGRETPEGNKAVQALKEVYAHWVPEER**ILTTN**
LWSAELSKLAANAFLAQRISSVNAM**SALCEATGADVTQVSYAVGK**DTRIGPKFLNASVGFGGSCFQKDI
LNLVYICECNGLPEVAEYWKQVIKINDYQKNRFVNR**VVSSMFNTVSNK**K**IAILGFAFK**KDTGDTR**ETPA**
IDVCKGLLGDKAR**LSIYDPQVTEDQVQR**DLSMNKFDWDHPLHLQPMSPTTVKQVSWVWDAYEATKDAHG
ICILTEWDEFKKLDFKR**IYDNMQKPAFVFDGR**NIVNADELREIGFIVYSIGKPLDPWLKDMPAVA

Protein No.: I-133

Protein name and Species:

Farnesyl diphosphate synthase OS=Hevea brasiliensis GN=FDP PE=2 SV=1

Accession: tr|Q8L7F4|Q8L7F4_HEVBR

Unused ProtScore: 12.91

Seq Cov %: 25.4

ITRAQ Changed Ratio:

117: 115=E3TL: D3TL=0.56

116: 114=E5TL: D5TL=0.73

Annotated typical Precursor MS Region:

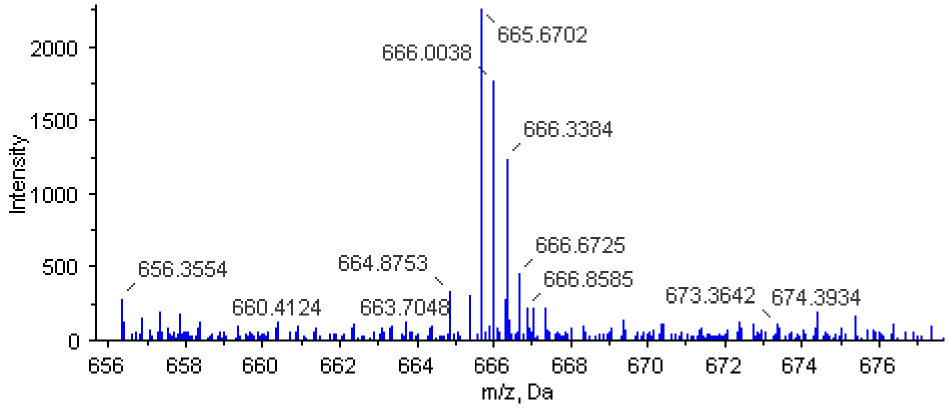

Sequence Coverage and Matched peptides: in different color

MADLKSTFLKVYSVLKQELLEDPAFEWTPDSRQWVERMLDYNVPGGKLNRGLSVIDSYKLLKEGQELTE
EEIFLASALGWCIEWLQAYFLVLDDIMDSSHTRRGQPCWFRVPKVGLIAANDGILLRNHIPRILKKHFR
GKAYYVDLLDLFNEVEFQTASGQMIDLITTLEGEKDLSKYTLSLHRRIVQYKTAYYSFYLPVACALLIA
GENLDNHIVVKDILVQMGIYFQVQDDYLDCFGDPETIGKIGTDIEDFKCSWLVVKALELCNEEQKKVLY
EHYGKADPASVAKVKVLYNELKLQGVFTEYENESYKKLVTSIEAHPSKPVQAVLKSFLAKIYKRQK

Protein No.: I-134

Protein name and Species:

Serine/threonine-protein phosphatase OS=Vitis vinifera GN=VIT_13s0156g00060 PE=3 SV=1

Accession: tr|F6HPS0|F6HPS0_VITVI

Unused ProtScore: 12.79

Seq Cov %: 27.6

ITRAQ Changed Ratio:

117: 115=E3TL: D3TL=0.21

116: 114=E5TL: D5TL=0.77

Annotated typical Precursor MS Region:

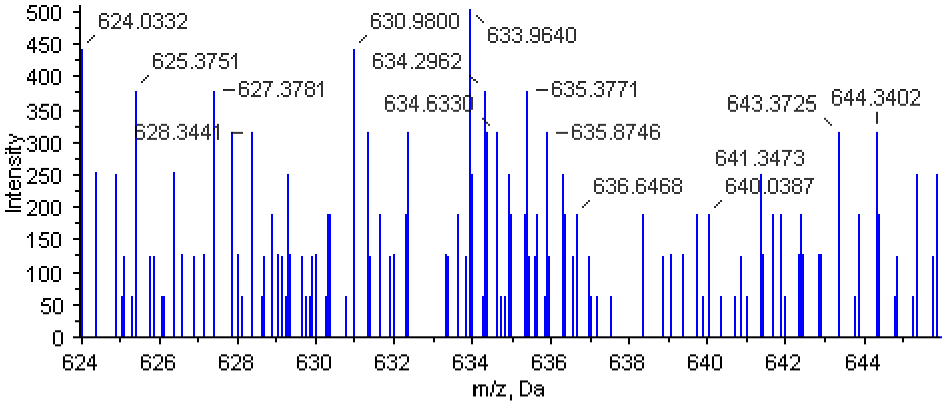

Sequence Coverage and Matched peptides: in different color

MDLISSDSHGNLDAQISQLMQCK**PLSEQEVR**TLCEKAK**EILMEESNVQPVK**SPVTICGDIHGQFHD**LAE**
LFRIGGK**CPDTNYLFMGDYVDR**GYYSVETVTMFVKSNKQDRVSKSPTKFEYRTMSLACSKIIWLRGLLA
ELDFSETDPTPLHADNTSANQITTNPVYHERTKHIEVDCHSIRAAFEARLLVALKVRYPQR**ITILR**GNH
ESR**QITQVYGFYDECLR**KYGNANVWKIFTDLFDYFPLTALVESEIFCLHGGLSPSIETLDNIRNFDRVQ
EVPHEGPMCDLLWSDPDDRCGWGISPR**GAGYTFGQDISEQFNHTNNLK**LIARAHQLVMEGFNWGHEQK**V**
VTIFSAPNYCYRCGNMASILEVDDCKGHTFIQFEPAPRRGEPDVTRRTPDYFL

Protein No.: I-135

Protein name and Species:

Calcium-dependent protein kinase, putative OS=Ricinus communis GN=RCOM_1689450 PE=4 SV=1

Accession: tr|B9RCK2|B9RCK2_RICCO

Unused ProtScore: 12.78

Seq Cov %: 18.2

ITRAQ Changed Ratio:

117: 115=E3TL: D3TL=0.63

116: 114=E5TL: D5TL=1.20

Annotated typical Precursor MS Region:

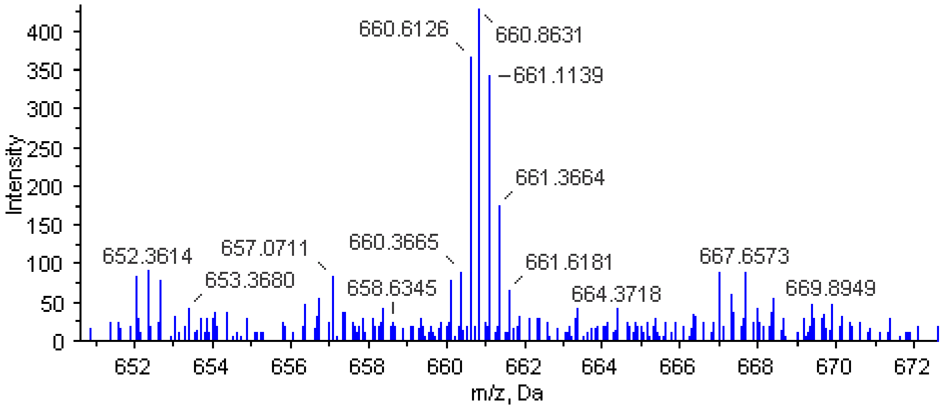

Sequence Coverage and Matched peptides: in different color

MGNCSSNLPSTSTFTADRPPPPPPSHGITVHPPTASPPPRPPPPHSLQPTSNNRPSAAPAAVGR**VLGRP**
MEDVRSIYAFGRELGR**GQFGVTYLVTHK**ETKQQFACKSIATRKLINRDDIEDVRREVQIMHHLTGHRNI
VELKGAYEDRHSVNLVMELCAGGELFDRIIAKGHYSERAAANLCRQIVTVVHDCHSMGVIHR**DLKPENF**
LFLSTDENSPLKATDFGLSVFFKPGDTFK**DLVGSAYYVAPEVLR**RNYGPAIDIWSAGVILYILLSGVPP
FWGETEQAIFDTVLRGHIDFSSDPWPSISSSAKDLVKKMLTADAKDRISAAEVLNHPWMR**VDGDASDKP**
LDIAVLTRMKQFRAMNKLKKVALK**VIAENLSEEEIMGLK**EMFKSMDTDNNGTITYEELKAGLPKLGTKL
SESEVKQLMEAADVDGNGTIDYIEFITATMHMNRMEREDHLYKAFEYFDKDK**SGYITMEELEHALK**EYN
MGDARTIKEIIAEVDTDHDGR**INYEEFVAMMR**KGNPELVTNRRRK

Protein No.: I-136

Protein name and Species:

obg-like ATPase 1 [Vitis vinifera] OS=Vitis vinifera GN=VIT_10s0003g05020 PE=4 SV=1

Accession: tr|D7TKK5|D7TKK5_VITVI

Unused ProtScore: 12.7

Seq Cov %: 18.5

ITRAQ Changed Ratio:

117: 115=E3TL: D3TL=0.64

116: 114=E5TL: D5TL=0.79

Annotated typical Precursor MS Region:

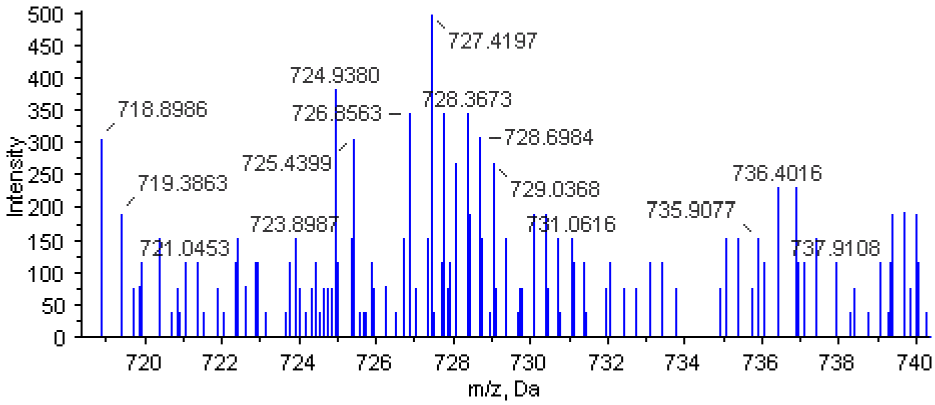

Sequence Coverage and Matched peptides: in different color

MPPKSSKSKEAPAERPILGRFSSHLKIGIVGLPNVGKSTLFNTLTKLAIPAENFPFCTIEPNEARVNIP
DERFEWLCQLYKPKSEVSAFLEIHDIAGLVRGAHQGQGLGNSFLSHIRAVDGIFHVLRAFEDPDIIHVD
DSVDPVRDLEVISEELRLKDIEFIEKRIEDVEKSMKRSNDKQLKIELELCLKVKAFLEDGKDIRLGDWK
AADVEILNTFQLLTAKPVVYLVNMNEKDYQRKKNKFLPKIHAWVQDHGGETIIPFSCALERNLADMPED
EAAKYCEENKVQSALPKIIKTGFSAINLIYFFTAGPDEVKCWQIRRQTKAPQAAGTIHSDFERGFICAE
VMKFDDLKELGSEGAVKAAGKYRQEGKTYVVQDADIIFFKFNVSGGGKK

Protein No.: I-138

Protein name and Species:

GTP-binding nuclear protein Ran-3-like [Vitis vinifera] OS=Vitis vinifera GN=VIT_04s0008g05030 PE=4 SV=1

Accession: tr|D7SUQ2|D7SUQ2_VITVI

Unused ProtScore: 12.64

Seq Cov %: 19.3

ITRAQ Changed Ratio:

117: 115=E3TL: D3TL=0.46

116: 114=E5TL: D5TL=0.69

Annotated typical Precursor MS Region:

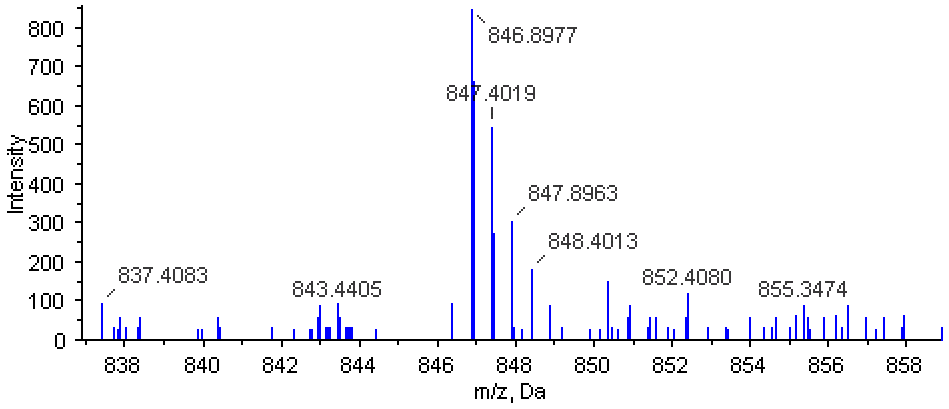

Sequence Coverage and Matched peptides: in different color

MPFCYNNNYCYFIKSKKIVITKLFRDLIQRLREIASIKGYNENTRRLLIYIRLKLLLPPHSKLKPSFQS
APWRLPYEKLTLTLQMALPNQQTVDYPSFKLVIVGDGGTGKTTFVKRHLTGEFEKKYEPTIGVEVHPLD
FFTNCGKIRFYCWDTAGQEKFGGLRDGYYIHGQCAIIMFDVTARLTYKNVPTWHRDLCRVCENIPIVLC
GNKVDVKNRQVKAKQVTFHRKKNLQYYEISAKSNYNFEKPFLYLARKLAGDVNLHFVESPALAPPEVQI
DMAAQAHHEAELAAAAAQPLPDDDDDTFE

Protein No.: I-141

Protein name and Species:

Aspartate aminotransferase OS=Vitis vinifera GN=VIT_04s0008g03770 PE=3 SV=1

Accession: tr|D7SUD7|D7SUD7_VITVI

Unused ProtScore: 12.36

Seq Cov %: 27

ITRAQ Changed Ratio:

117: 115=E3TL: D3TL=0.55

116: 114=E5TL: D5TL=0.47

Annotated typical Precursor MS Region:

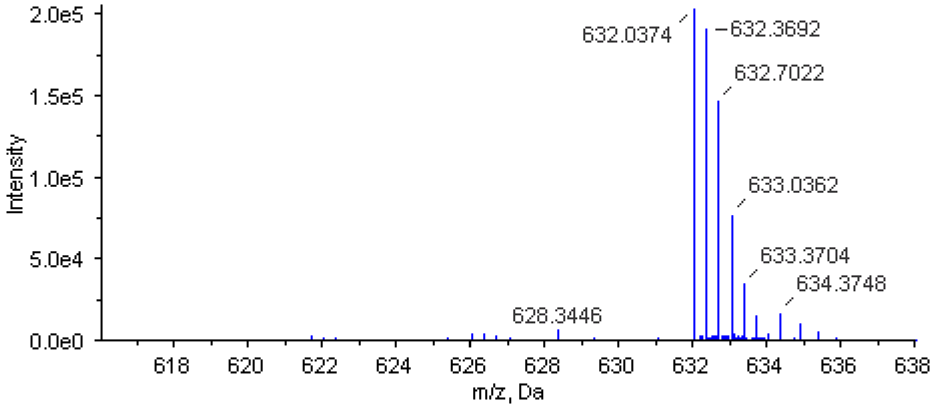

Sequence Coverage and Matched peptides: in different color

MDPNHTISASATSGSDFGGSVFDHVVQ**APEDPILGVTVAYNK**DPSPVK**LNLGVGAYRTEEGKPLVLNVV**
RRAEQLLVNDRSRVK**EYLPIVGLAEFNK**LSAKLMFGADSPAIQVK**RVTTVQCLSGTGSLR**VGAEFLARH
HHQRTIYIPQPTWGNHGKIFTLAGLSVKTYRYYDPATRGLNFQGLLEDLSLAPSGAIVLLHACAHNPTG
VDPTLQQWEQIRQLIRLK**GLLPFFDSAYQGFASGSLDADAQSVR**MFVADGGECLAAQSYAKNMGLYGER
VGALSIVCKTADVASRVESQVKLVIRPMFSNPPIHGASIVAIILKNRDMYNEWTVELKAMADRIISMRQ
QLFDALRARGTPGDWNHILKQIGMFTFTGLNTAQVAFMTKEYHVYMTSDGR**ISMAGLSSKTVPHLADAI**
HAAVTRAI

Protein No.: I-145

Protein name and Species:

Citrate-binding protein OS=Hevea brasiliensis GN=CBP PE=1 SV=1

Accession: sp|Q39962|CBPR_HEVBR

Unused ProtScore: 12.21

Seq Cov %:34.9

ITRAQ Changed Ratio:

117: 115=E3TL: D3TL=0.29

116: 114=E5TL: D5TL=0.38

Annotated typical Precursor MS Region:

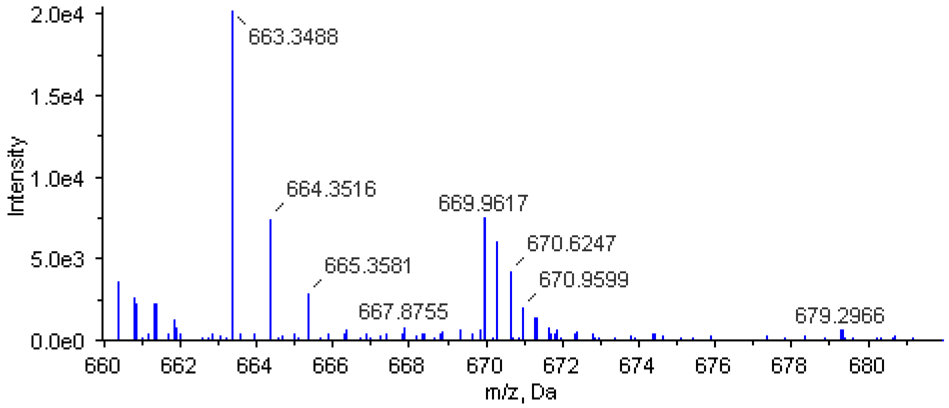

Sequence Coverage and Matched peptides: in different color

MKMKRSPYCFCCSFALLLLVSFLKDRHFCSA**DPTDGFTEVPLTEDNFVIQKPY**DKPLNDRYSYKNGIRR
LWVYENDKPFKVGSPTRPRTEIRIKGHDYSSGVWQFEGQVHVPEGTSGVTVMQVFGAVNK**ATALQLR**VY
NGDLKSYK**SNSVATDIYNK**WLR**VNVIHK**VGK**GEITVFINGQQKLVVNDDGPAEHYFKCGVYAAPDGSSN**
YMESRWKNIKLYKSDNKLEGCNNNHGTWLVQ

Protein No.: I-148

Protein name and Species:

Acetyl-CoA C-acetyltransferase OS=Hevea brasiliensis GN=HbAACT PE=2 SV=1

Accession: tr|A9ZMZ5|A9ZMZ5_HEVBR

Unused ProtScore: 12

Seq Cov %: 61.8

ITRAQ Changed Ratio:

117: 115=E3TL: D3TL=0.55

116: 114=E5TL: D5TL=0.66

Annotated typical Precursor MS Region:

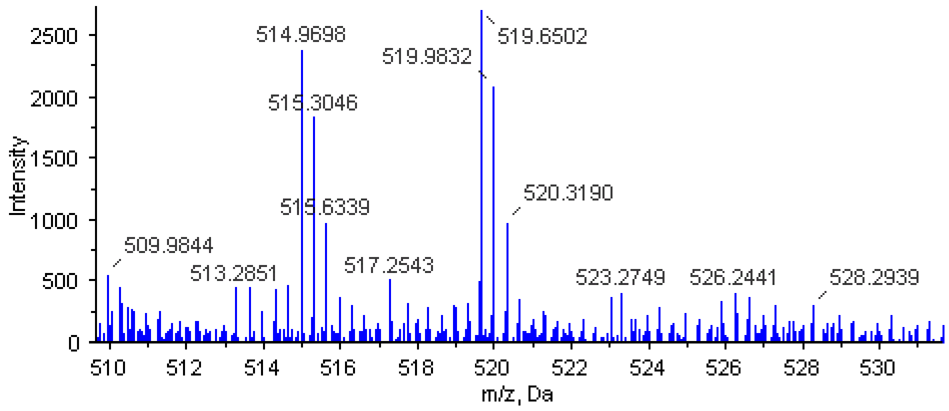

Sequence Coverage and Matched peptides: in different color

MAPAAATAVAAEIKPRDVCIVGVARTPMGGFLGSLCTLSATKLGSIAIEAALKRANVDPSLVQEVFFGN
VLSANLGQAPARQAALGAGIPNSVVCTTVNKVCASGMKATMLAAQSIQLGINDVVVAGGMESMSNAPKY
LAEARKGSRLGHDSLVDGMLKDGLWDVYNDVGMGSCAEICADNHSITREDQDKFAIHSFERGIAAQESG
AFAWEIVPVEVSGGRGKPSTIVDKDEGLGKFDPVKLRKLRPSFKENGGTVTAGNASSISDGAAAVILVS
GETALKLGLQVIAKIGGYADAAQAPELFTTSPALAIPKTIANGGLDASQVDYYEINEAFAVVALANQKL
LGLNPEKINVHGGAVSLGHPLGCSGARILVTLLGVLRQKNGKYGVGGVCNGGGGASALVVELL

Protein No.: I-150

Protein name and Species:

Pyridoxal biosynthesis protein PDX1 OS=Hevea brasiliensis GN=PDX1 PE=2 SV=1

Accession: sp|Q39963|PDX1_HEVBR

Unused ProtScore: 11.87

Seq Cov %: 29.8

ITRAQ Changed Ratio:

117: 115=E3TL: D3TL=2.40

116: 114=E5TL: D5TL=1.42

Annotated typical Precursor MS Region:

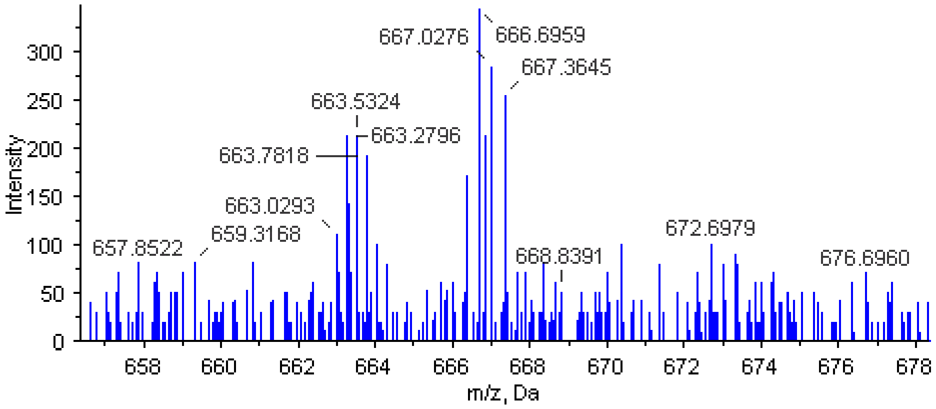

Sequence Coverage and Matched peptides: in different color

MAGTGVVAVYGNGAITETKKSPFSVKVGLAQMLRGGVIMDVVNPEQARIAEEAGACAVMALERVPADIR
AQGGVARMSDPQLIKEIKQSVTIPVMAKARIGHFVEAQILEAIGIDYVDESEVLTPADEENHINKHNFR
IPFVCGCRNLGEALRRIREGAAMIRTKGEAGTGNVIEAVRHVRSVMGDIRLLRNMDDDEVFTFAKKIAA
PYDLVMQTKQLGRLPVVQFAAGGVATPADAALMMQLGCDGVFVGSGVFKSGDPARRARAIVQAVTHYSD
PDMLAEVSCGLGEAMVGINLNDKKVERFANRSE

Protein No.: I-151

Protein name and Species:

NAD dependent epimerase/dehydratase, putative OS=Ricinus communis GN=RCOM_1683000 PE=4 SV=1

Accession: tr|B9RC03|B9RC03_RICCO

Unused ProtScore: 11.81

Seq Cov %: 27.5

ITRAQ Changed Ratio:

117: 115=E3TL: D3TL=1.75

116: 114=E5TL: D5TL=1.51

Annotated typical Precursor MS Region:

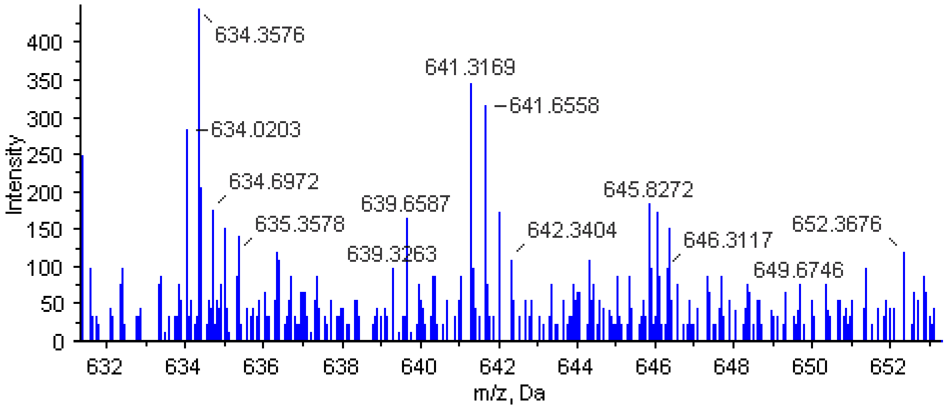

Sequence Coverage and Matched peptides: in different color

MGFQSNGATDNKKPYKFLIYGRTGWIGGLLGKLCESQGIDYTYGNGRLENRVSLENDIASINPTHVFNA
AGVTGRPNVDWCESHKVETIRTNVAGTLTLADVCREKGLVLINYATGCIFEYDDKHPLGSGIGFKEEDT
PNFIGSFYSKTKAMVEELLKNYENVCTLRVRMPISSDLANPRNFITKITRYEKVVDIPNSMTILDELLP
ISIEMAKRNLTGIWNFTNPGVVSHNEILEMYRDYVDPNFTWKNFTLEEQAKVIVAPRSNNELDATKLSK
EFPEMLPIKESLIKYVFKPNQKTAAA

Protein No.: I-152

Protein name and Species:

Calmodulin OS=Clematoclethra scandens subsp. tomentella GN=CaM PE=4 SV=1

Accession: tr|B1NDK1|B1NDK1_9ERIC

Unused ProtScore: 11.77

Seq Cov %: 50

ITRAQ Changed Ratio:

117: 115=E3TL: D3TL=0.61

116: 114=E5TL: D5TL=0.63

Annotated typical Precursor MS Region:

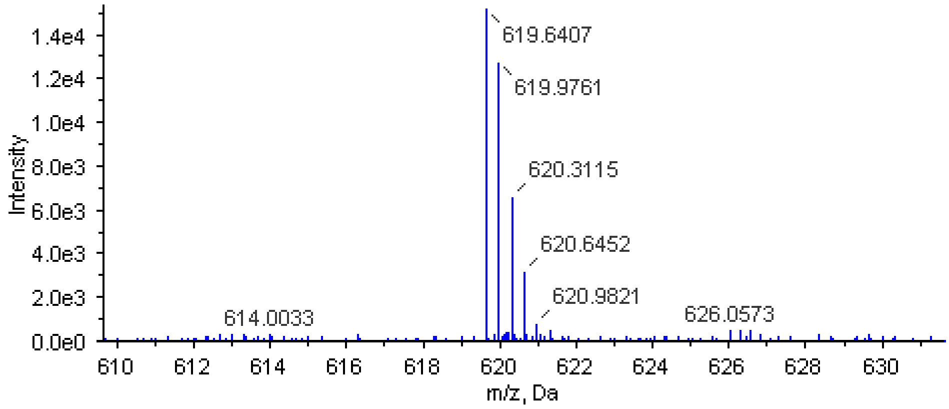

Sequence Coverage and Matched peptides: in different color

MADPLTDDQISEFK**EAFSLFDKDGDGCITTK**ELGTVMRSLGQNPTEAELQDMINEVDADGNGTIDFPEF
LNLMARKMK**DTDSEEELKEAFRVFDKDQNGFISAAELR**HVMANLGEK**LTDEEVDEMIREADVDGDGQIN**
YEEFVKVMMA

Protein No.: I-160

Protein name and Species:

26S proteasome non-atpase regulatory subunit, putative OS=Ricinus communis GN=RCOM_1507340 PE=4 SV=1

Accession: **tr|B9RAM4|B9RAM4_RICCO**

Unused ProtScore: **11.38**

Seq Cov %: **20.7**

ITRAQ Changed Ratio:

117: 115=E3TL: D3TL=0.44

116: 114=E5TL: D5TL=0.62

Annotated typical Precursor MS Region:

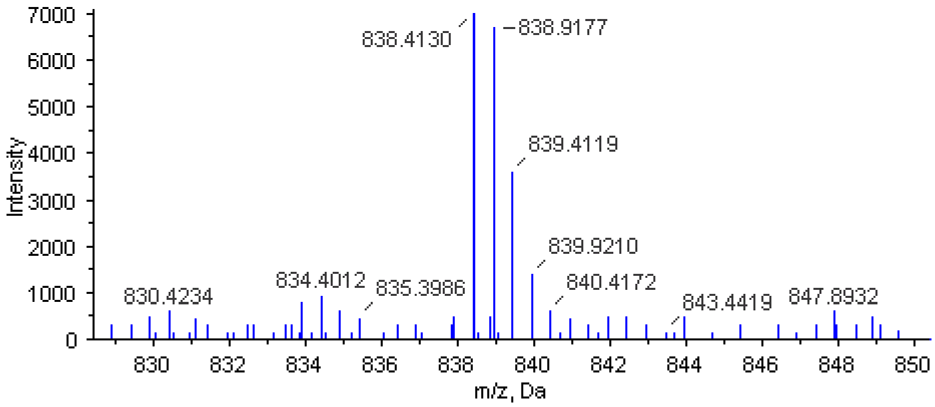

Sequence Coverage and Matched peptides: in different color

MEGQEGTQQPHLILAHKRFLLTHPDVQDIEKVRLKEEVFAAVKSDDMAPLYETLITEGLLEKDQSILDL
MRAKNEDELKKIDEKIADAEENLGESEVREAHLAKSLFYIRIGDKEKALEQLKVTESKTVAVGQKMDLV
FYTLQLGFFYMDFDLISKSIDKAKNLFEEGGDWERKNRLKVYEGLYCMSTRNFTKAATLFLDSISTFTT
YELFPYDTFIFYTVLTSIITLDRVSLKQKVVDAPEILTVIGKIPYLSEFLNSLYFCQYKSFFSAFAGIA
EQIKLDRYLHPHFRYYMREVRTVVYSQFLESYKSVTIEAMAKAFGVTVEFIDLELSRFIAAGKLHCKID
KVAGVLETNRPDAKNALYQATIKQGDFLLNRIQKLSRVIDL

Protein No.: I-161

Protein name and Species:

Abscisic acid stress ripening-related protein 1 OS=Hevea brasiliensis GN=ASRLP1 PE=4 SV=1

Accession: **tr|Q6XNP8|Q6XNP8_HEVBR**

Unused ProtScore: **11.37**

Seq Cov %: **21.3**

ITRAQ Changed Ratio:

117: 115=E3TL: D3TL=0.31

116: 114=E5TL: D5TL=0.42

Annotated typical Precursor MS Region:

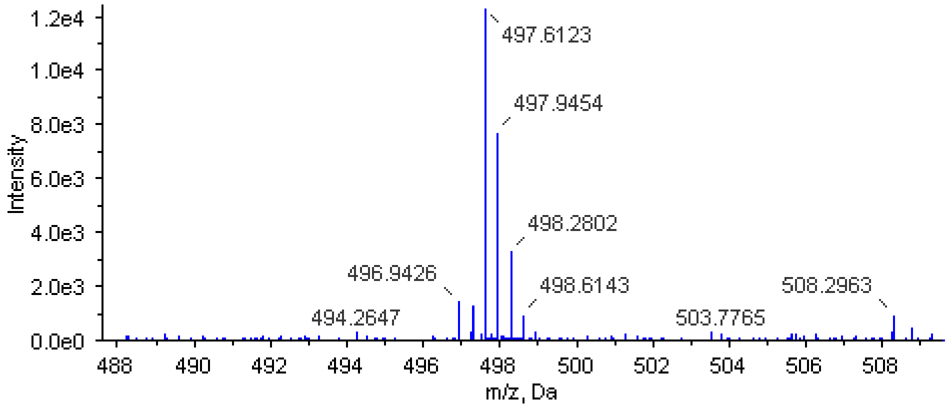

Sequence Coverage and Matched peptides: in different color

MAEEKHHHLFHHHKEGQEAVDCRKEEKHHKHLCQLGKLGAAAAGTYALHEKHAAKKDPEHAHGHKIKEE
VAAAAAIGAGGFAFHEHHEKKEAKKKNEEGHGKKHHHPF

Protein No.: I-167

Protein name and Species:

Myo-inositol-1-phosphate synthase OS=Ricinus communis GN=MIPS PE=2 SV=1

Accession: tr|C7B9C0|C7B9C0_RICCO

Unused ProtScore: 11.12

Seq Cov %: 18

ITRAQ Changed Ratio:

117: 115=E3TL: D3TL=10.47

116: 114=E5TL: D5TL=2.51

Annotated typical Precursor MS Region:

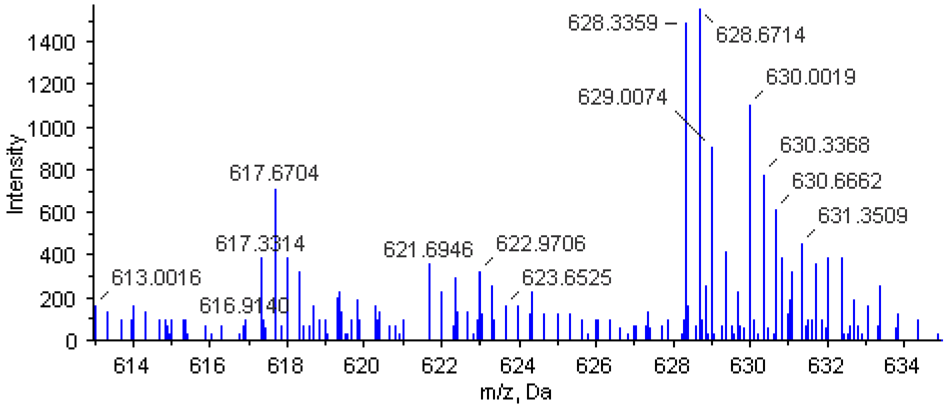

Sequence Coverage and Matched peptides: in different color

MFIEKFQVESPNVKYSEDEIHSVYNYETTELVHENRNGTYQWIVKPKTVQYEFKTDVHVPKLGVMLVGW
GGNNGSTLTGGVIANREGISWATKDKVQQANYFGSLTQASSIRVGSFNGEEIYAPFKSLLPMVNPDDIV
FGGWDISDMNLADAMARAKVLDIDLQKQLRPYMESMVPLPGIYDPDFIAANQGSRANNVIKGTKKEQME
QIVKDIREFKEKTKVDKVVVLWTANTERYSNVVVGLNDTMESLLASLERNEAEISPSTLYALACIFENV
PFINGSPQNTFVPGVIDLAIKGNSLIGGDDFKSGQTKMKSVLVDFLVGAGIKPTSIVSYNHLGNNDGMN
LSAPQTFRSKEISKSNVVDDMVSSNGILYEPGEHPDHVVVIKYVPYVGDSKRAMDEYTSEIFMGGKSTI
VLHNTCEDSLLAAPIILDLVLLAELSTRIQLKAEGEGKFHSFHPVATILSYLTKAPLVPPGTPVVNALS
KQRAMLENILRACVGLAPENNMILEYK

Protein No.: I-168

Protein name and Species:

Catalytic, putative OS=Ricinus communis GN=RCOM_0291690 PE=4 SV=1

Accession: tr|B9SZU0|B9SZU0_RICCO

Unused ProtScore: 10.98

Seq Cov %: 22.2

ITRAQ Changed Ratio:

117: 115=E3TL: D3TL= 0.45

116: 114=E5TL: D5TL= 0.64

Annotated typical Precursor MS Region:

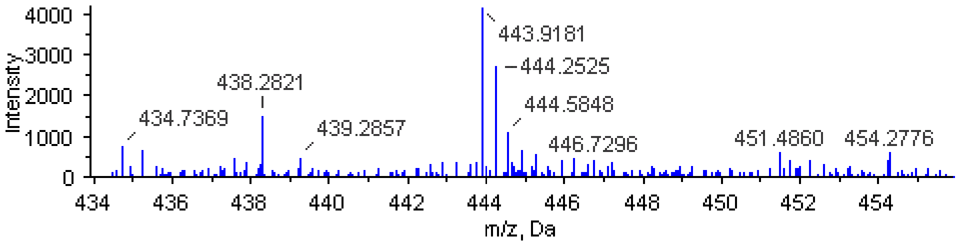

Sequence Coverage and Matched peptides: in different color

MVPITVLLSSFLLLFTVSHGFAPPRSFLQSLSGSNTYLTTKELWFNQILDHYSPYDHRRFQQRYYEYLD
YFRAPGGPIFLK**ICGESSCNGIANDYISVLAK**K**FGAAVVSLEHR**YYGKSTPFKSSETKNLRYLSSK**QAL**
FDLAVFRQHYQEALNLKLNRTNVENPWIVFGISYSGALSAWYRLKFPHLTCGSVASSAVVLAVYNFTEF
DQQIGESAGAECKAALQETTQLVDERLASNRKAVKTLFNAAELEIDGDFLYFLADAAVIAFQYGNPDK**L**
CSPLVEAKK**AGEDLVEAYAK**YVKEYYVGSFGVSVETYNQKHLKDTAINENSSDRLWWFQVCTEVAYFQV
APSNDSIRSSKVNTR**YHLDLCK****NVFGEGIYPEVDTTNIYYGGTK**IAGSKIVFTNGSQDPWRHASK**QISS**
PDTPSYIITCHNCGHGTDMRGCPQSPLSLEGNAQNCSSPDAVQKVRQQVIEHIDLWLSECEASGRSYI

Protein No.: I-169

Protein name and Species:

Proliferation-associated 2g4, putative OS=Ricinus communis GN=RCOM_0489730 PE=4 SV=1

Accession: tr|B9SWY5|B9SWY5_RICCO

Unused ProtScore: 10.97

Seq Cov %: 15.2

ITRAQ Changed Ratio:

117: 115=E3TL: D3TL= 1.53

116: 114=E5TL: D5TL= 1.66

Annotated typical Precursor MS Region:

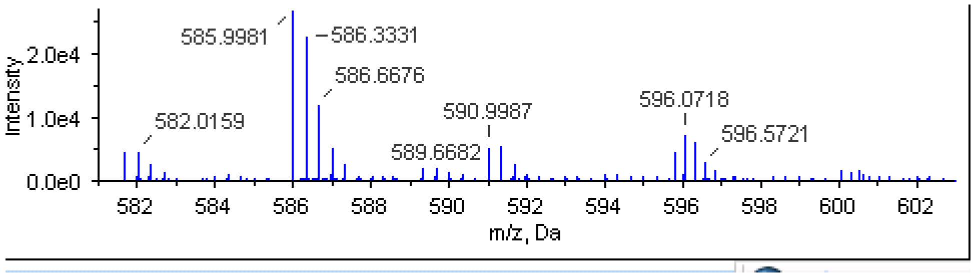

Sequence Coverage and Matched peptides: in different color

MSDDEREEK**ELDLTSPEVVTK**YK**SAAEIVNK**ALQLVISECKPKAK**IVDLCEK**GDAFIREQTGNMYKNVK
KKIERGVAFPTCVSVNNTVCHCSPLASDETLLEEGDVVKIDMGCHIDGFIAVVGHTHVLQAGPVTGR**AA
DVIAAANTAAEVALR**LVRPGKKNKDVTEAIQK**VAAAYDCKIVEGVLSHQLKQFVIDGNK**VILSVSNPET
RVDDAEFEENEVYAIDIVTTTGEGKPKLLDEKHTTIYKRAVDKNYHLKMKASR**FIFSEISQK**FPIMPFT
ARALEEKRARLGLVECVNHDLLQPYPVLHEKPGDIVAHIK**FTVLLMPNGSDR**ITSHSLQELQPTKTIDD
PEIKAWLALGTKTKKKGGGKKKKAKKGDKAEESTEAEPMDATNGAETQE

Protein No.: I-170

Protein name and Species:

Aspartic proteinase, putative OS=Ricinus communis GN=RCOM_1303660 PE=3 SV=1

Accession: tr|B9SVA7|B9SVA7_RICCO

Unused ProtScore: 10.95

Seq Cov %: 26.1

ITRAQ Changed Ratio:

117: 115=E3TL: D3TL= 0.30

116: 114=E5TL: D5TL= 0.52

Annotated typical Precursor MS Region:

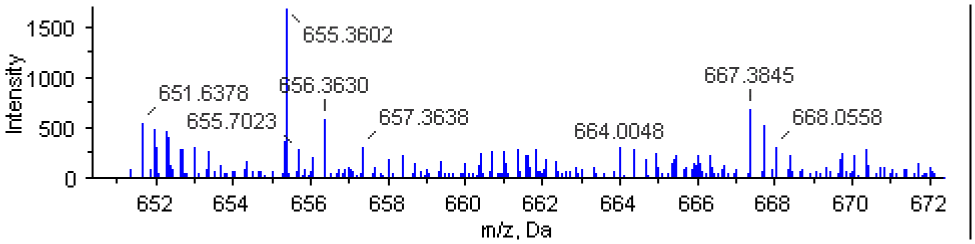

Sequence Coverage and Matched peptides: in different color

MGTIFKPALFFCLILLPLVCATASSSNDGLVRIGLKKRKFDQNNRVAAQFESKEGEAFRASIKKYHIRG
NLGDAEDIDIVSLKNYMDAQYFGEIGIGTPPQKFTVIFDTGSSNLWVPSSKCYFSVACYFHSKYKSGQS
STYKKNGKSADIHYGTGAISGFFSQDNVKVGELVIKNQEFIEATREPSITFLVAKFDGILGLGFQEISV
GNAVPVWYNMVNQGLVKEPVFSFWFNRNADEDEGGEIVFGGMDPNHYKGEHTYVPVTQKGYWQFDMGDV
LIDGKTTGICSSGCAAIADSGTSLLAGPTTIITEVNHAIGATGVVSQECKAVVAQYGETIIAMLLAKDQ
PQKICSQIGLCTFDGSRGVSMGIESVVNEKIQEVAGGLHDAMCSTCEMAVVWMQNQLKQNQTQEHILNY
VNELCERLPSPMGESAVDCGSLSTMPNVSFTIGGRVFDLAPEQYVLKVGDGEAAQCISGFTALDVPPPR
GPLWILGDVFMGPFHTVFDYGNKRVGFAEVA

Protein No.: I-175

Protein name and Species:

Phosphoglyceride transfer family protein OS=Hevea brasiliensis PE=2 SV=1

Accession: tr|Q6J6I5|Q6J6I5_HEVBR

Unused ProtScore: 10.83

Seq Cov %: 33.5

ITRAQ Changed Ratio:

117: 115=E3TL: D3TL= 0.08

116: 114=E5TL: D5TL= 0.59

Annotated typical Precursor MS Region:

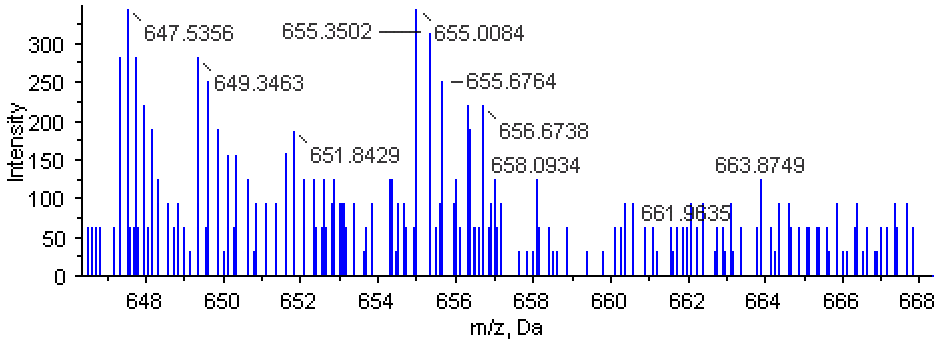

Sequence Coverage and Matched peptides: in different color

MLTGTLKWRATYKPEEIRWHEISHEVEKGKVFRANFHDRYGRTVLIMRPEMQNTTSAVDNIRHLAYVIE
NSILNLAEGQEQMSWLIDFTGLSLSNSMSIGTGVDIIKILQNHYPERLAVAFVYNPPRIFEAFWKAVKC
VLNSKTSEKVKFVYPNNKESEEVMRHFFDVDNLPGEFGGKATMKYDHEEFSRLMAQDDVKTAKYWGFDE
KPTHIANAQLRPQLAPEPTPFAPPAS

Protein No.: I-181

Protein name and Species:

Polyadenylate-binding protein, putative OS=Ricinus communis GN=RCOM_0812530 PE=4 SV=1

Accession: tr|B9RYE9|B9RYE9_RICCO

Unused ProtScore: 10.63

Seq Cov %: 15.2

ITRAQ Changed Ratio:

117: 115=E3TL: D3TL= 1.75

116: 114=E5TL: D5TL= 1.24

Annotated typical Precursor MS Region:

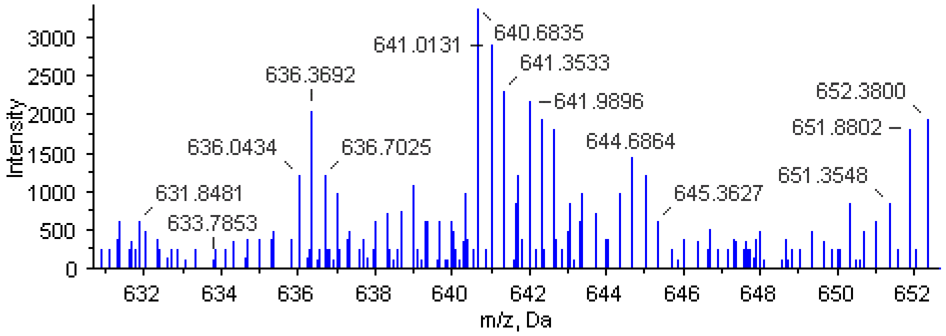

Sequence Coverage and Matched peptides: in different color

MAQVQVPVQGQNVNGGANAPYVTTSLYVGDLEANVTDSHLYDLFNQVGQVVSVRVCRDLTTRR**SLGYGY**
VNYSSPQDAARALDMLNFTPLNGSPIRIMYSHRDPSVRK**SGSGNIFIK**NLDKGIDHKALHDTFSAFGNI
LSCKVATDSSGQSKGYGFVQFDNEESAQKAIEKLNGMLLNDKQVYVGPFLRKQERESAIDKTRFNNVYV
KNLSETTTEEDLKKAFGEYGTITSAVVMRDGDGKTKCFGFVNFENADDAATAVEALNGKKFDDKEWFVG
KAQKKNERENELKVR**FEQSMKEAADKFQGANLYIK****NLDDSIGDDRLK**QLFSPFGTITSCKVMRDPNGIS
RGSGFVAFSTPEEASKALMEMNGK**MVVSKPLYVALAQR**KEDRRARLQAQFSQIRPVAMAPSVAPRMPMY
PPGGPGLGQQIFYGQAPPAIIPPQPGFGYQQQLVPGMRPGGAPMPNFFVPMVQQGQQGQRPGGRRAGAA
QQSQQPVPLMQQQMVPRGRVYRYPPGRGIPDVPMTGVAGGMLSVPYDMSGMPMRDAALSQPIPIGALAS
ALANASPEQQR**TMLGENLYPLVEQLEPDAAAK**VTGMLLEMDQTEVLHLLESPEALKAK**VAEAMEVLR**SV
QQQQAGGAADQLASLSLNDNLVS

Protein No.: I-186

Protein name and Species:

Proteasome subunit beta type OS=Ricinus communis GN=RCOM_0205360 PE=3 SV=1

Accession: tr|B9SPS6|B9SPS6_RICCO

Unused ProtScore: 10.47

Seq Cov %: 29

ITRAQ Changed Ratio:

117: 115=E3TL: D3TL= 1.60

116: 114=E5TL: D5TL= 1.41

Annotated typical Precursor MS Region:

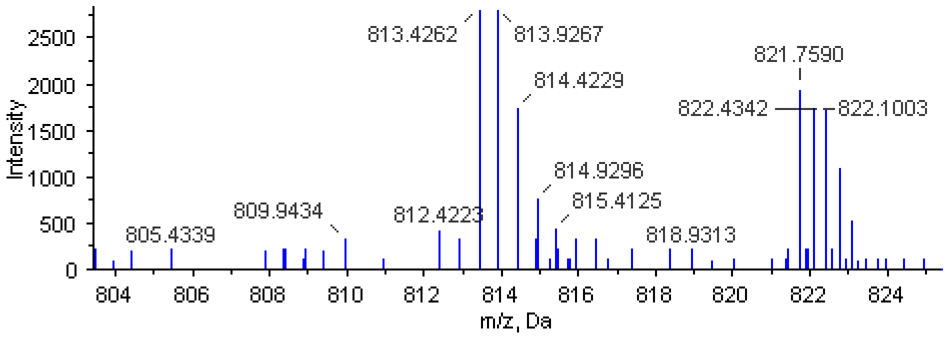

Sequence Coverage and Matched peptides: in different color

MDLKAPHSMGTTIIGVTYNGGVVLGADSRTSTGMYVANRASDKITQLTDNVYVCRSGSAADSQIVSDYV
RYFLHQHTIQLGQPATVKVAANLVRLISYNNKNMLETGLIVGGWDKYDGGKIYGIPLGGTIIEQPFAIG
GSGSSYLYGFFDQAWKEGMTKDEAEQLVVKAVSLAMARDGASGGVVRTVIINSEGVTRNFYPGDSLPLW
HEELEPQNSLLDILNASSPEPMNI

Protein No.: I-187

Protein name and Species:

ras-related protein RABC1 isoform 1 OS=Populus trichocarpa GN=POPTRDRAFT_798654 PE=3 SV=1

Accession: tr|B9GTP5|B9GTP5_POPTR

Unused ProtScore: 10.45

Seq Cov %: 42.2

ITRAQ Changed Ratio:

117: 115=E3TL: D3TL= 0.52

116: 114=E5TL: D5TL= 0.70

Annotated typical Precursor MS Region:

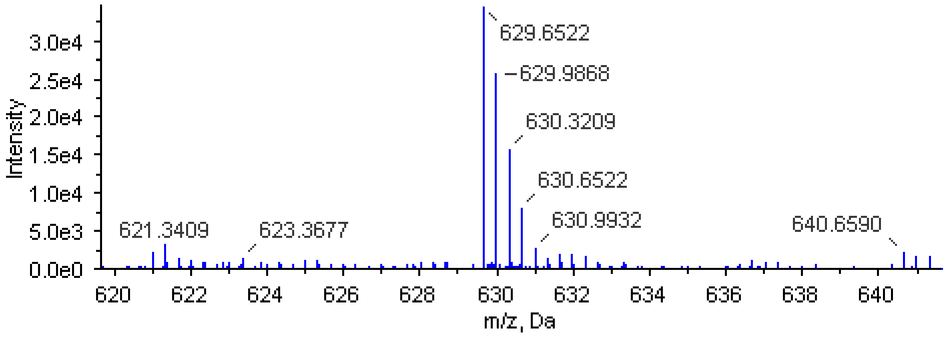

Sequence Coverage and Matched peptides: in different color

MEVPSTSQQEFEYLFKLLLIGDSGVGKSTLLLSFTSKNFEDLSPTIGVDFKVKHVTIGGKKLKLAIWDT
AGQERFRTLTSSYYRGAQGIIMVYDVTRRETFTNLSEIWAKEIDLYSTNQDCIKMLVGNKVDKESERVV
TKKEGIDFAREYGCLFLECSAKTRVNVEQCFEELVLKILETPSLLAEGSSGVKKNVFKQKPPEDVTTSS
CCSW

Protein No.: I-188

Protein name and Species:

ATP synthase subunit beta, mitochondrial OS=Hevea brasiliensis GN=ATPB PE=2 SV=1

Accession: sp|P29685|ATPBM_HEVBR

Unused ProtScore: 10.41

Seq Cov %: 17.3

ITRAQ Changed Ratio:

117: 115=E3TL: D3TL= 7.52

116: 114=E5TL: D5TL= 1.63

Annotated typical Precursor MS Region:

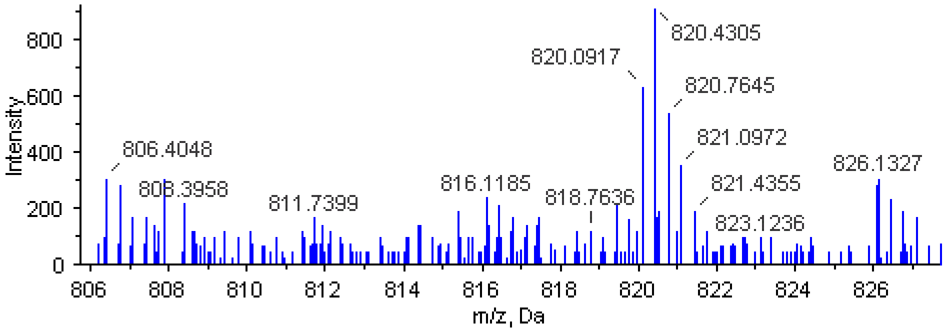

Sequence Coverage and Matched peptides: in different color

MASRRLLSSLLRSSSRRSVSKSPISNINPKLSSSSPSSKSRASPYGYLLTRAAEYATSAAAAAPPQPPP
AKPEGGKGGGK**ITDEFTGK**GAIGQVCQVIGAVVDVRFDEGLPPILTSLEVLDHSIRLVLEVAQHMGEGM
VRTIAMDGTEGLVRGQRVLNTGSPITVPVGRANPWTYHEVIGEPIDERGDIKTSHFLPIHR**EAPAFVDQ**
ATEQQILVTGIKVVDLLAPYQRGGKIGLFGGAGVGKTVLIMELINNVAKAHGGFSVFAGVGERTREGND
LYREMIESGVIKLGDKQADSKCALVYGQMNEPPGARAR**VGLTGLTVAEHFR**DAEGQDVLLFIDNIFR**FT**
QANSEVSALLGRIPSAVGYQPTLATDLGGLQERITTTKKGSITSVQAIYVPADDLTDPAPATTFAHLDA
TTVLSRQISELGIYPAVDPLDSTSRMLSPH**ILGEEHYNTAR**GVQKVLQNYKNLQDIIAILGMDELSEDD
KLTVARARKIQRFLSQPFHVAEVFTGAPGKYVELKESITSFQGVLDGKYDDLPEQSFYMVGGIDEVIAK
ADKIAKESAS

Protein No.: I-191

Protein name and Species:

Acid phosphatase, putative OS=Ricinus communis GN=RCOM_0854040 PE=4 SV=1

Accession: tr|B9RUL1|B9RUL1_RICCO

Unused ProtScore: 10.38

Seq Cov %: 7

ITRAQ Changed Ratio:

117: 115=E3TL: D3TL= 0.58

116: 114=E5TL: D5TL=0.60

Annotated typical Precursor MS Region:

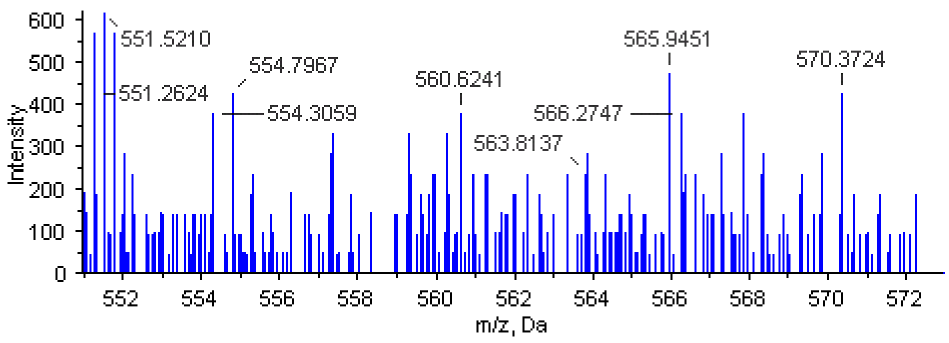

Sequence Coverage and Matched peptides: in different color

MEEEGGEMEKKIKIGVCVMEKKVLSAPMGQIMDRLQAFGEFEIIHFGDKVIAEDPIESWPICDCLIAFY
SSGYPLEK**AEAYAALR**KPFLVNELEPQHLLHDRRKVYQRLEMYGIPVPRYALVNREFPYQELDYFSEEE
DFVEVHGNRFWKPFVEKPIDGDNHSIMIYYPSSAGGGMKELFRKVGNRSSEFHPEVRRVRREGSYIYEE
FMPTGGTDVK**VYTVGPEYAHAEAR**KSPVVDGVVMRNPDGKEVRYPVLLTPNEKQMAREVCIAFRQAVCG
FDLLRCEGRSYVCDVNGWSFVKNSYKYYDDAACVLRKMFLDAKAPHLSSTIPPTLPWKINEPVQPSEGL
TRQGSGIIGTFGQSEELRCVITVMRHGDRTPKQKVKLKVTEEK**LLNLMLK**YNGGRPRSETKLK**SAIQLQ**
DLLDATRILVPRIRPGR**ESDSEAEDIEHAEK**LRQVK**AVLEEGGHFSGIYR**KVQLKPLKWVKIPKSTGEA
EEERPVEA**LMVLK**YGGVLTHAGRKQAEELGRYFRNNMYPGEGTGLLRLHSTYRHDLKIYSSDEGRVQMS
AAAFAKGLLDLEGQLTPILVSLVSKDSSMLDGLDNASSEMEEAKARLNEIITSSTKAANSNESPEFPWM
TDGAGLPPNASELLPELVKLTKKVTEQVRLLAKDEDEELTETSSYNVIPPYDQAKALGKINIDVDRIAA
GLPCGSEGFLLMYARWRKLERDLYNERKDR**FDITQIPDVYDSCK**YDLLHNAHLNLEGLDELFK**VAQLLA**
DGVIPNEYGINPKQKLKIGSKIARRLLGKILIDLRNTREEAISVAELKSNQDQHSTSTKNEKEDADYQS
KLFIKNEDTRRTSTSEISTDHDDDDDKETKYRLDPKYANVKTPERHVRTRLYFTSESHIHSLVNVLRYC
NLDESLQEEDSLVCHNALERLHKTKELDYMSYIVLRMFENTEVPLEDPKRYRIEMTYSRGADLSPLEKN
DSEANSLHQEHTLPIMGPERLQEVGSYLTLEKMETMIRPFAMPAEDFPPPSTPAGFSGYFSKSAAVLER
LVNLWPFHKHDKHASANGK

Protein No.: I-195

Protein name and Species:

Putative uncharacterized protein OS=Ricinus communis GN=RCOM_1341920 PE=4 SV=1

Accession: tr|B9RN01|B9RN01_RICCO

Unused ProtScore: 10.23

Seq Cov %: 4.2

ITRAQ Changed Ratio:

117: 115=E3TL: D3TL=5.50

116: 114=E5TL: D5TL= 2.29

Annotated typical Precursor MS Region:

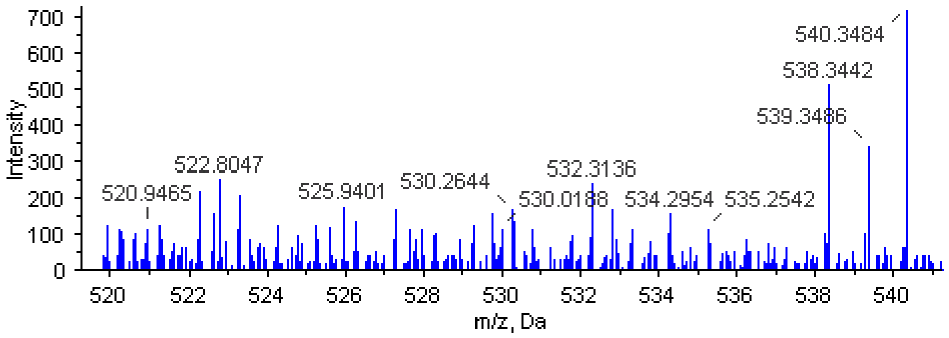

Sequence Coverage and Matched peptides: in different color

MSIPKELEQVMKLRGGSVLGKKTILKSDHFPGCQNKRLTPQIDGAPNYRQADSLPVHGVAIPTTEGIRN
VLKHIGAQKDGKRVQVIWFNLREEPVVYINGRPFVLR**DVERPFSNLEYTGINR**SRVEQMESRLKEDILM
EAARYGNKILVTDELPDGQMVDQWEPVSRDSANEELQLEGYLFDYERVPVTDEKSPEEPDFDILVDKIY
QADLNTEIIFNCQMGRGRTTTGMVIATLVYLNRIGASGIPRTNSIGRVFDTGPTVTDNLPNSEEAIRRG
EYAVIRSLTR**VLEGGVEGK**RQVDKVIDKCASMQNLR**EAIANYR**NRILRQPDEMKREASLSFFVEYLERY
YFLICFAVYIHSERDALRSSSFGHSSFADWMR**ARPELYSILR**RLLRRDPMGALGYASSKPSLMKIAESA
DGRPHEMGVVAALRNGEVLGSQTVLKSDHCPGCQIHTLPERVEGAPNFREVPGFPVYGVANPTIDGILS
VIQRIGSSKGGRPIFWHNMREEPVIYINGKPFVLREVERPYKNMLEYSGIDRERVQGMEARLKEDILRE
AESYGGAIMVIHETDDGQIFDAWEHVNFDSVKTPLEVFKCLEVDGFPIKYARVPITDGKAPKSSDFDTL
AVNIASASKDTAFVFNCQMGRGRTTTGTVIACLLKLRIDYGRPIRVLVDDMACEEADSGSSSGEETGGN
AARSPPSNTRMRTGTEQARAFGIDDILLLWKITRLFDNGVECREALDAVIDRCSALQNIRQAVLHYRK**V**
VNQQHVEPRVRRVALNRGAEYLERYFRLIAFAAYLGSEAFDGFCGQGESRMTFKTWLHQRPEVQAMKWS
IRLRPGR**FFTIPEELRAPQESQHGDAVMEATIK**ARNGSVLGTGSILKMYFFPGQRTSSHLQIHGAPHVY
KVDGYPVYSMATPTIAGAKEMLAYLGAKPNGEGSLAQKVILTDLREEAVVYINGTPFVLRELHKPVDTL
KHVGITGPLVEHMEAR**LKEDIVSEVR**ESGGRMLLHREEYNPATNQSSVIGYWENIFANDVKTPAEVYAA
LKDEGYDMTYRRIPLTRERDALASDVDAIQYCKDDCAGSYLFVSHTGFGGIAYAMAIICLRLGAEATFT
AEIPQTLVDTESFSVHEEILPSQLSEEETFRMGDYRDILSLTRVLMYGPKSKADVDIVIDKCVGAGHLR
DDILYYSKELRKCPHHDDEQLAHLMDMGVKALRRYFFLITFRSYLYCAKPTETRFTSWMNARPELGHLC
NNLRIDK

Protein No.: I-197

Protein name and Species:

chitinase Class I, putative OS=Ricinus communis GN=RCOM_0806420 PE=4 SV=1

Accession: tr|B9SIC4|B9SIC4_RICCO

Unused ProtScore: 10.18

Seq Cov %: 24.9

ITRAQ Changed Ratio:

117: 115=E3TL: D3TL= 0.55

116: 114=E5TL: D5TL= 0.59

Annotated typical Precursor MS Region:

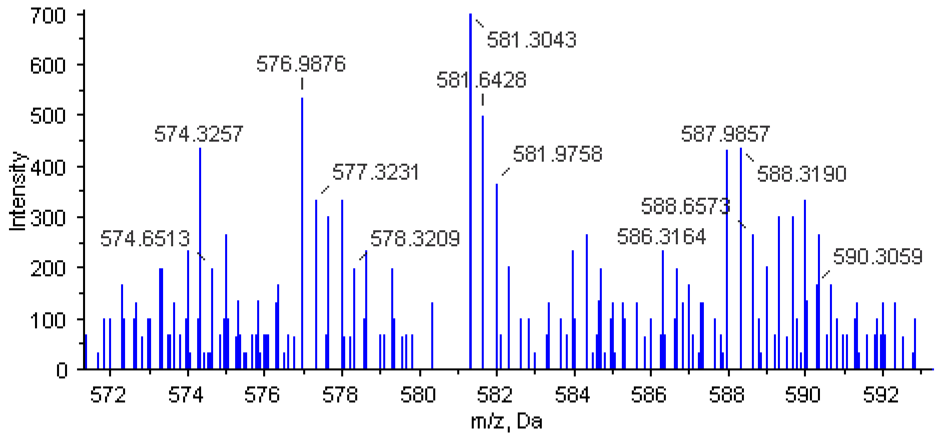

Sequence Coverage and Matched peptides: in different color

MLPKMEVYAFTVFYIFFSFLLGGLA**EQCGR**QADGALCPGGLCCSQYGWCGNTDPYCGGGCQSQCGGGG**G**
GGGGDLGSIISRSTFDQLLKHRNDGACPGK**GFYTYDAFISAAKDFPGFGTTGDVATR**KGEIAAFFGQTS
HETTGGWPSAPDGPYAWGYCFIR**EQNPGSYCSPSSTYPCAPNK**QYYGR**GPIQLTWNYNYGQCGR**AIGVD
LLNNPDLVATDPVISFKAAIWFWMTPQSPK**PSCHDVITGRWSPSAADR**SAGRLAGYGVLTNIINGALEC
GRGWDSRVDDR**IGFYK**RYCDLLGIGYGNNLDCYNQRSFGNGAFGLVDTM

Protein No.: I-198

Protein name and Species:

Stem-specific protein TSJT1, putative OS=Ricinus communis GN=RCOM_0910970 PE=4 SV=1

Accession: tr|B9RTJ4|B9RTJ4_RICCO

Unused ProtScore: 10.16

Seq Cov %: 20.1

ITRAQ Changed Ratio:

117: 115=E3TL: D3TL= 0.52

116: 114=E5TL: D5TL= 0.31

Annotated typical Precursor MS Region:

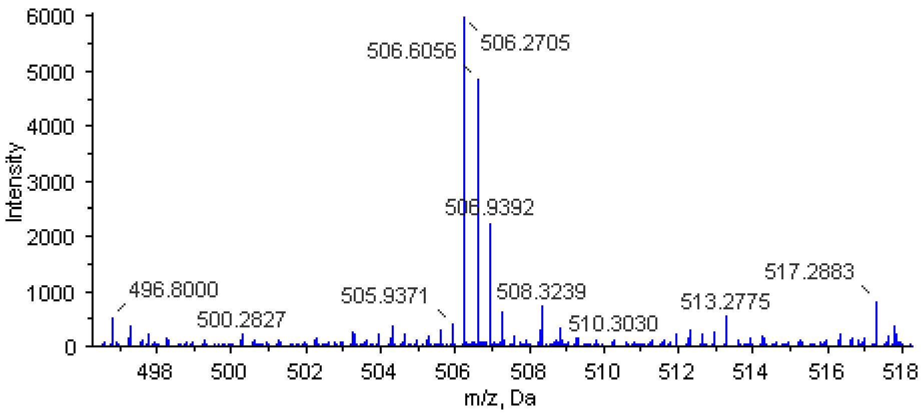

Sequence Coverage and Matched peptides: in different color

MLAVFDNTVAKCPDALQSPHSASSSALKDGFLAKHFGSIHPASVTVNLGTAGIIAYSLDK**QNPLLPR**LF
AVVDDIFCLFQGHIENIAVLKQQYGLNKTANEVIIVIEAFRTLR**DRGPYPADQVVR**DIQGKFAFVLYDS
TSKATFIAADADGSVPFFWGADSERHLVLSDDAQILQQACGK**SFAPFPKGCFFTTSGGLR**SYEHPLNEL
KPVPR**VDSSGQVCGATFK**VDAETKKEGGMPRVGSAYDWSSNY

Protein No.: I-204

Protein name and Species:

Pyruvate kinase OS=Populus trichocarpa GN=POPTRDRAFT_563414 PE=2 SV=1

Accession: tr|A9PFJ7|A9PFJ7_POPTR

Unused ProtScore: 9.98

Seq Cov %: 21.7

ITRAQ Changed Ratio:

117: 115=E3TL: D3TL= 1.85

116: 114=E5TL: D5TL= 1.56

Annotated typical Precursor MS Region:

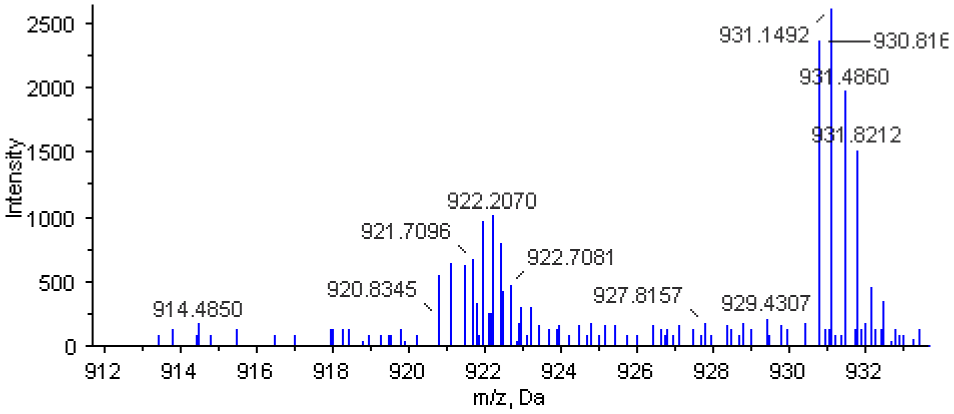

Sequence Coverage and Matched peptides: in different color

MANID**IEGLLK**EHQDDEEGRVPRTKIVCTLGPSSRSVPMLEKLLRAGMNVARFNFSHGTHEYHQETLNN
LRIAMQNTNILAAVMLDTKGPEIRTGFLKDGNPIQLKEGQEITITTDYSIK**GDTDMISMSYK**KLPVDIK
PRNTILCADGTITLTVLSCDPKAGTVRCRCENTAMLGERKNVNLPGVVVDLPTLTDKDKEDILGWGVPN
N**IDMIALSFVR**KGSDLVNVRKVLGPHAKHIQLMSK**VENQEGVVNFDEILR**ETDSFMVAR**GDLGMEIPVE**
KIFLAQKMMIYKCNIVGKPVVTATQMLESMIKSPRPTR**AEATDVANAVLDGTDCVMLSGESAAGAYPEL**
AVKTMRRICIEAESSLDYAAIFKEMIR**STPLPMSPLESLASSAVR**TANKARAKLIVVLTRGGTTAKLVA
KYRPAVPILSVVVPVLTTDSFDWTCSDETPARHSLIYR**GLIPLLAEGSAK**ATDAESTEVILEAALKSAT
KRGLCKPGDAVVALHRIGAASVIKICLVK

Protein No.: I-205

Protein name and Species:

Peptidyl-prolyl cis-trans isomerase OS=Hevea brasiliensis PE=2 SV=1

Accession: tr|F8RW92|F8RW92_HEVBR

Unused ProtScore: 9.88

Seq Cov %: 66.3

ITRAQ Changed Ratio:

117: 115=E3TL: D3TL= 0.66

116: 114=E5TL: D5TL= 0.75

Annotated typical Precursor MS Region:

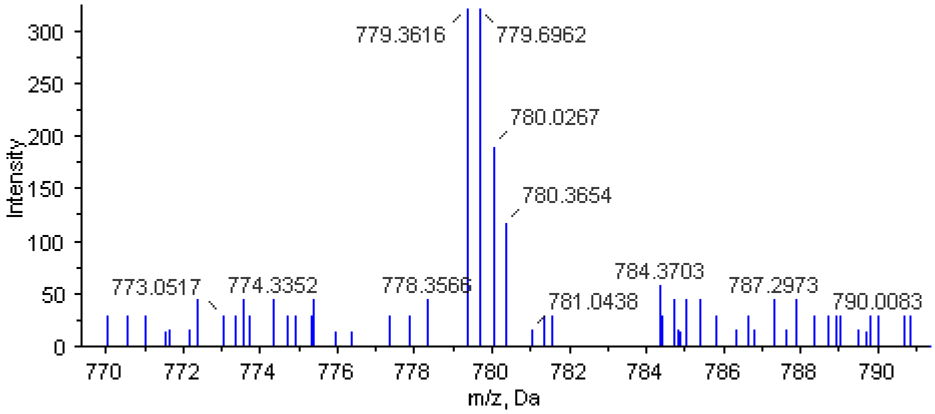

Sequence Coverage and Matched peptides: in different color

MANPKVFFDMSVGGHPAGKIVMELYADVVPRTAENFRALCTGEKGIGKSGKPLHYKGSSFHRVIPGFMC
QGGDFTAGNGTGGESIYGAKFADENFTKKHTGPGVLSMANAGPGTNGSQFFVCTAKTEWLDGKHVVFGQ
VVEGMDVVKAIEKVGSSSGRTSKPVVIADCGQLS

Protein No.: I-208

Protein name and Species:

Importin beta-3, putative OS=Ricinus communis GN=RCOM_1361100 PE=4 SV=1

Accession: tr|B9T1E9|B9T1E9_RICCO

Unused ProtScore: 9.79

Seq Cov %: 6.4

ITRAQ Changed Ratio:

117: 115=E3TL: D3TL= 3.31

116: 114=E5TL: D5TL= 2.11

Annotated typical Precursor MS Region:

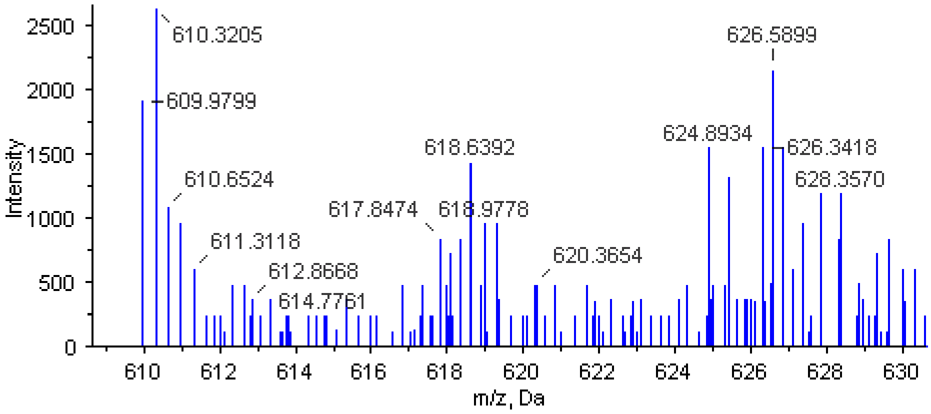

Sequence Coverage and Matched peptides: in different color

MDESAQQLQLQQAQLAAILGQDSAPFETLISSLMSSSNEQRSQAEVLFNLCKQTDPNTLSLKLSHLLQF
SPHVDARAMSAVLLRKLLTRDNHVVVDTDSSSYLWPR**LSLATQSSIK**SILLTSIQHESTKSILKKLCDT
VSELAANILPDNGWPELLPFMFNCVSSDSAKLQESAFLIFAQLSQYIGESLIPFIKDLHTVFLQCLGSS
PSFDVKIAALNAVINFIQCLTSSADRDRFQDLLPAMMRTLTEALNNGNEATAQEALELLIELAGTEPRF
LRRQLVDVVGSMLQIAEAESLEEGTRHLAIEFVITLAEARERAPGMMRKLPQFISRLFAILMRMLLDIE
DDPAWHSAENEDEDAGETSNYSVGQECLDRLAISLGGNTIVPVASEQLPAYLAAPEWQKHHAALIALAQ
IAEGCSKVMIKNLEHVVSMVLNSFHDPHPRVRWAAINAIGQLSTDLGPDLQNQYHQRILPALASAMDDF
QNPRVQAHAASAVLNFSENCTPEILTPYLDGIVSKLLVLLQNGK**QMVQEGALTALASVADSSQEHFQKY**
YDAVMPYLKTILVNATDKSNRMLRAKSMECISLVGMAVGKDKFREDAKQVMEVLMSLQGSQMETDDPTT
SYMLQPDVTITSADSDNDIDDSDDESMETITLGDKRIGIKTSVLEEKATACNMLCCYADELKEGFFPWI
DQVAPTLVPLLKFYFHEEVRKAAVSAMPELLRSAKLAVEKGLSQGRNESYVKQLSDYIIPALVEALHKE
PDTEICANMLDALNECLQISGTLVDEGQVRSIVDEIKQVITASSSRKKERADRAKAEDFDAEEGELIKE
ENEQEEEVFDQVGEILGTLIKTFKASFLPFFDELSTYLTPMWGKDKTAEERR**IAICIFDDVAEQCR**ETA
LKYYDTYLPFLLEACNDENPDVRQAAVYGLGVCAEFGGSVFKPLVGEALSRLNVVIQHPNAKQSENVMA
YDNAVSALGKICQFHRESINSAQIVPAWLNCLPITGDLIEAK**VVHEQLCSMVER**SDSELLGPNNQYLPK
IVSVFAEVLCGKDLATEQTASRMVNLLRHLQQTLPPATLASTWSLLHPQQQMALQSILSS

Protein No.: I-210

Protein name and Species:

Profilin OS=Mangifera indica GN=Man i 3.01 PE=2 SV=1

Accession: tr|Q2XPH2|Q2XPH2_MANIN

Unused ProtScore: 9.66

Seq Cov %: 31.3

ITRAQ Changed Ratio:

117: 115=E3TL: D3TL= 1.67

116: 114=E5TL: D5TL= 1.53

Annotated typical Precursor MS Region:

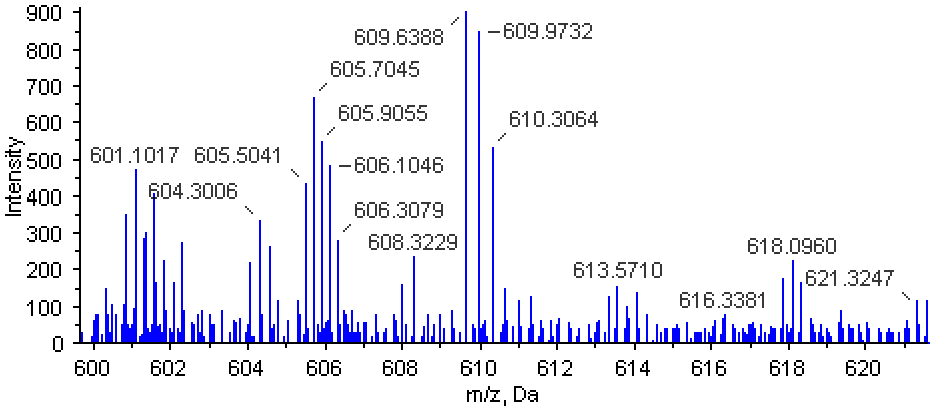

Sequence Coverage and Matched peptides: in different color

MSWQAYVDEHLMCDIEGHHLTAAAIVGLDGSVWAQSANFPKLNPEEITAINK**DFDEPGSLAPTGLHLGG**
TKYMVIQGEPGAVIRGK**KGPGGVTVK**KTNMAFVIGIYDEPMTPGQCNMIVERLGDYLVEQGL

Protein No.: I-212

Protein name and Species:

Superoxide dismutase [Cu-Zn] OS=Hevea brasiliensis GN=Cu/Zn-SOD PE=2 SV=1

Accession: tr|Q8GZP2|Q8GZP2_HEVBR

Unused ProtScore: 9.6

Seq Cov %: 37.5

ITRAQ Changed Ratio:

117: 115=E3TL: D3TL= 0.35

116: 114=E5TL: D5TL= 0.72

Annotated typical Precursor MS Region:

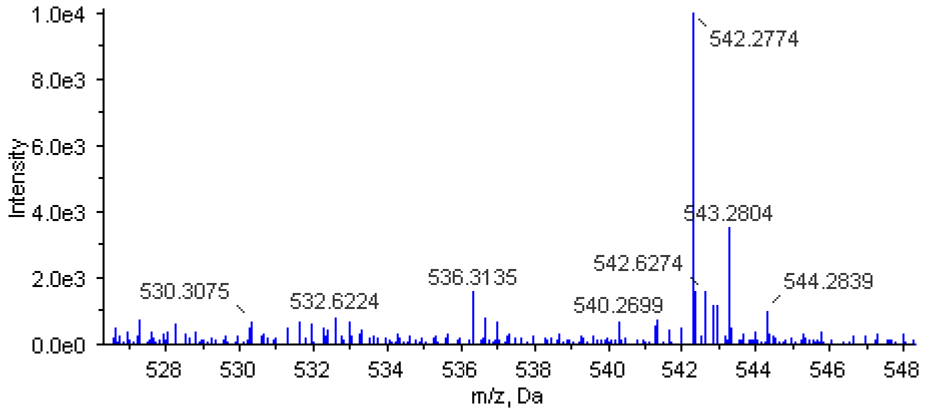

Sequence Coverage and Matched peptides: in different color

MLK**AVAVITSSEGISGK**IFFTQEGDGPTTVTGSVSGLKPGLHGFHVHTFGDTTNGCLSTGLHFNPASKD
HGGPEDENR**HAGDLGNVNVGDDGTANFTIVDKHIPLSGPHSIAGR**SVVFHEGRDDLGK**GGHELSK**ITGN
AGDRIACGIIGLQE

Protein No.: I-213

Protein name and Species:

Seryl-tRNA synthetase, putative OS=Ricinus communis GN=RCOM_1564380 PE=3 SV=1

Accession: tr|B9RKX9|B9RKX9_RICCO

Unused ProtScore: 9.57

Seq Cov %: 13.9

ITRAQ Changed Ratio:

117: 115=E3TL: D3TL= 0.66

116: 114=E5TL: D5TL= 0.53

Annotated typical Precursor MS Region:

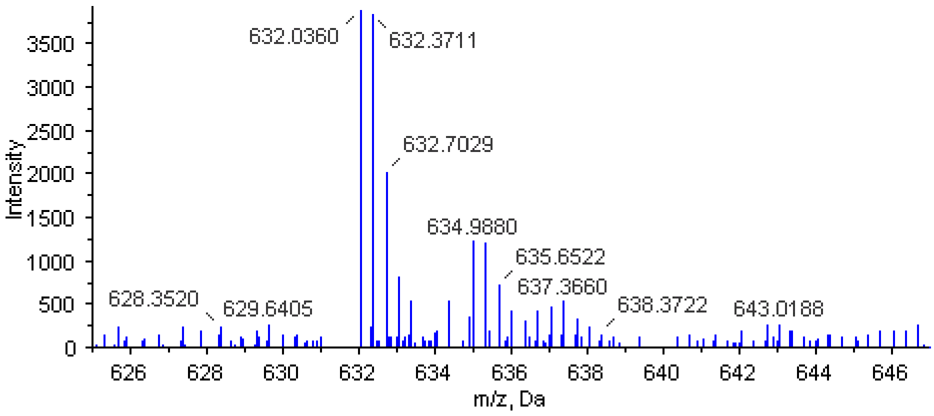

Sequence Coverage and Matched peptides: in different color

MLDINLFREDKGHNPQLIRESQRRRFANVNVVGEIILLDKEWRQRQYELDNLRKDFNKINKLVAKLKIA
GEDATEMIKDTNENKRLTAEKEAQVQEAALALNKKLEEVGNLVHDSVPVSNDEANNAVIRVWGEKRLEP
KLKNHVDLVELLGIADTKKGANVAGGRGFYLKGDGVRLNQALINVALDFLEKRGYTALQTPFFMRKDIM
AK**CAQLAQFDEELYK**VTGEGEDKYLIATAEQPLCAYHLDDWIHPSQLPIR**YAGYSSCFR**KEAGSHGR**DT**
LGIFRVHQFEKVEQFCITSPNGNDSWDMHEEMIKNSEDFYKMLNIPYQVVAIVSGALNDAAAKKYDLEA
WFPASNTYR**ELVSCSNCTDYQSR**KLEIRYGQKKNNEQAK**QYVHLLNSTLTATERTLCCILENYQK**ENGV
EIPEPLRGYMGGKSFLPFQNNPPTEGKGKKSKA

Protein No.: I-216

Protein name and Species:

Phospholipase D OS=Populus trichocarpa GN=POPTRDRAFT_829577 PE=3 SV=1

Accession: tr|B9GH43|B9GH43_POPTR

Unused ProtScore: 9.47

Seq Cov %: 18.8

ITRAQ Changed Ratio:

117: 115=E3TL: D3TL= 0.36

116: 114=E5TL: D5TL= 0.77

Annotated typical Precursor MS Region:

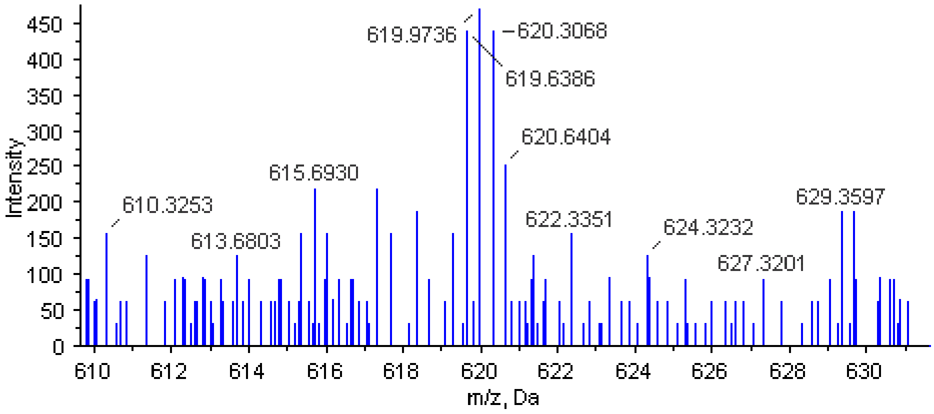

Sequence Coverage and Matched peptides: in different color

MAQILLHGNLHVTIYEVDKIGEGGGHGFFHKLVGKVGEKVGIGNGISR**LYATIDLEK**ARVGRTRILENE
ATNPRWYESFHIYCAHMASNVIFTVK**DVNPIGATLIGR**AYIPVEEILDGEEIDRWVEILDGDKNPIRAG
SKIHVKLQYFDITNDHNWGRGIRGSK**YPGVPYTFYSQR**QGCRVSLYQDAHIPDKFIPKIPLASGEYYEP
HR**CWEDVFDAITNAK**HLIYITGWSVYTEISLVRDSRRPK**PGGDITLGELLK**KKASEGVRVLMLIWDDR**T**
SVGLLKRDGLMATHDEETEHYFQNTDVHCILCPRNPDDGGSIVQDLQISTMFTHHQK**IVVVDSAMPNGD**
SQRRRIVSYIGGIDLCDGR**YDTPFHSLFRTLDTAHHDDFHQPNFTGASIQK**GGPREPWHDIHSRLEGPI
AWDVLFNFEQRWKKQGGKDLLVQLRELEDVIIPPSPVTYPDDHETWNVQLFRSIDGGAAFGFPETPEDA
AKAGLVSGKDNIIDRSIQDAYINAIRRAKNFIYIENQYFLGSSFCWSADGIKPEDINALHLIPKELSLK
IVSK**IEAGER**FTVYVVVPMWPEGIPESASVQAILDWQRRTMDMMYKDVIQALRAKGLEEDPR**NYLTFFC**
LGNREVKK**SGEYEPSEKPEPDSDYIR**AQEARR**FMIYVHAK**MMIVDDEYIIIGSANINQR**SMDGARDSEI
AMGGYQPYHLATRQPARGQIHGFRLGLWYEHLGMLDDTFLKPENEDCIR**KVNQIADK**YWDLYSSETLEG
DLPGHLLRYPIGISSEGNVTELPGTEYFPDTKARVLGAKSDYMPPILTT

Protein No.: I-219

Protein name and Species:

14-3-3 protein 3 OS=Hevea brasiliensis PE=2 SV=1

Accession: tr|F2YZ44|F2YZ44_HEVBR

Unused ProtScore: 9.44

Seq Cov %: 47.7

ITRAQ Changed Ratio:

117: 115=E3TL: D3TL= 0.65

116: 114=E5TL: D5TL= 0.66

Annotated typical Precursor MS Region:

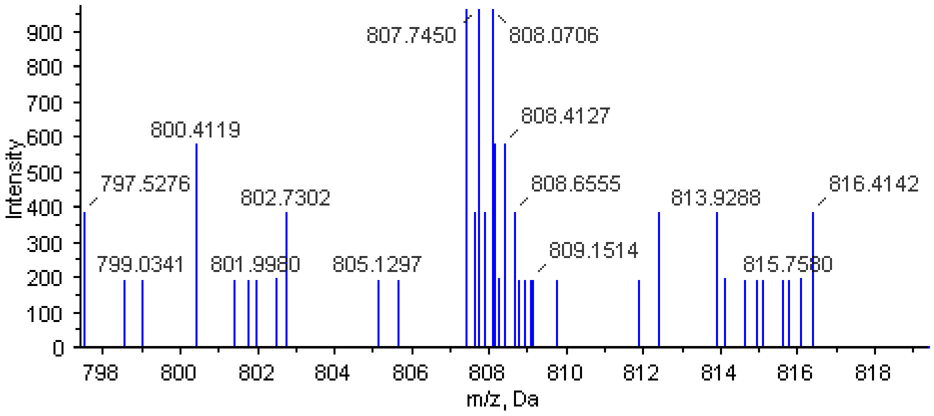

Sequence Coverage and Matched peptides: in different color

MAVAPSSREESIYMAKLAEQAERYEEMVEFMEKVSASVEKEELTVEERNLLSVAYKNVIGARRASWRII
SSIEQKEESRGNENHVSMIRDYRAKIESELSSICDGILKLLDTRLIPPASPGDSKVFYLKMKGDYHRYL
AEFKTGAERKEAAESTLTAYKSAQDIATAELAPTHPIRLGLALNFSVFYYEILNSPDRACSLAKQAFDE
AIAELDTLGEESYKDSTLIMQLLRDNLTLWTSDIADEAGDEIKEASKRESGEGQPQQ

Protein No.: I-220

Protein name and Species:

Caffeic acid O-methyltransferase OS=Boehmeria nivea GN=COMT PE=2 SV=1

Accession: tr|Q14VV8|Q14VV8_BOENI

Unused ProtScore: 9.44

Seq Cov %: 19.2

ITRAQ Changed Ratio:

117: 115=E3TL: D3TL= 0.61

116: 114=E5TL: D5TL= 0.63

Annotated typical Precursor MS Region:

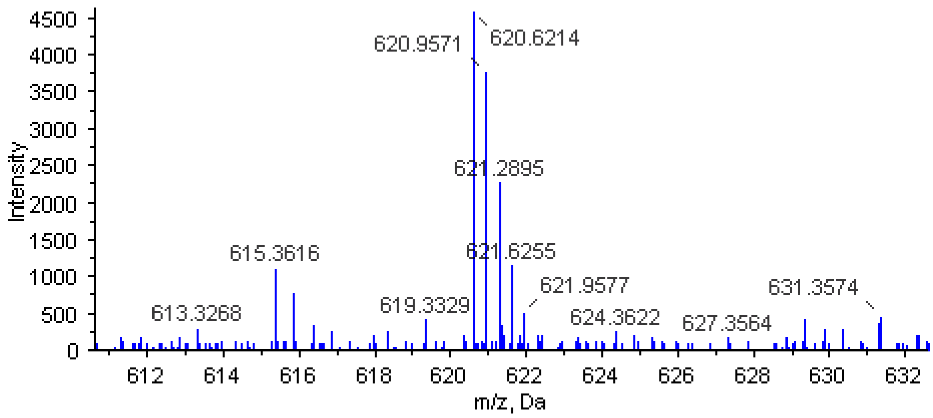

Sequence Coverage and Matched peptides: in different color

MGSTGETQMTPTQVSDEEANLFAMQLASASVLPMVLKTAIELDLLEIIAKAGPGAWLSPAEIAAQLPTT
NPAAPVMLDRILRLLACYSVVACQLRTLPGGAAER**LYGLAPVCK**FLTKNNDGVSISPLCLMNQDKVLME
SWYYLK**DAVLEGGIPFNKAYGMTAFEYHGTDPR**FNKVFNR**GMSDHSTITMK**KLLETYKGFEGLNSVVDV
GGGTGAVLSMIVSNYPSIKGINFDLPHVIEDAPAYPGVEHVGGDMFVSVPK**GDAIFMK**WICHDWSDEHC
LKFLKSCYDALPNNGKVIVAECILPVAPDTSLATKGVAHIDVIMLAHNPGGKERAEKEFQALAK**GAGFQ**
GFRVVCSAFNTYVMEFLKKP

Protein No.: I-221

Protein name and Species:

Pathogenesis-related thaumatin (Fragment) OS=Populus deltoides GN=PR52-1 PE=4 SV=1

Accession: **tr|D2JWP4|D2JWP4_POPDE**

Unused ProtScore: **9.34**

Seq Cov %: **35.3**

ITRAQ Changed Ratio:

117: 115=E3TL: D3TL= 0.28

116: 114=E5TL: D5TL= 0.53

Annotated typical Precursor MS Region:

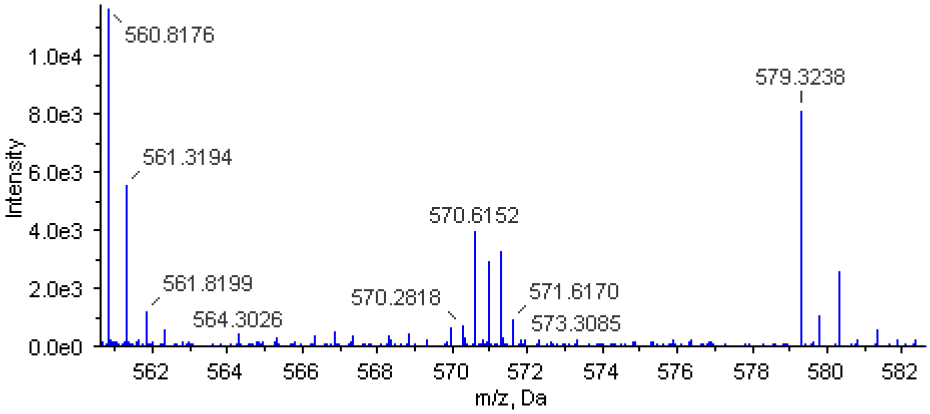

Sequence Coverage and Matched peptides: in different color

MSHLTKFLTSSLLFSLLIISTNAATFEIR**NNCPYTVWAAASPGGGRRLDRGQTWYLNVPAGTSMAR**IWG
R**TNCNFDGSGR**GRCQTGDCTGGLECKGWGVPPNTLAEYALNQFGNLDFYDISLVDGFNIPMEFSPTSSG
GSGKCQALLCTADINGQCPNELR**APGGCNNPCTVFK**TNEYCCTNGQGSCGPTYFSRFFKDR**CPTSYSYP**
QDDPSSTFTCPGGTNYRVIFCPWGSPHFPLEMVE

Protein No.: I-224

Protein name and Species:

Glutathione transferase, Lambda class OS=Populus trichocarpa PE=2 SV=1

Accession: tr|D2WL72|D2WL72_POPTR

Unused ProtScore: 9.24

Seq Cov %: 32.6

ITRAQ Changed Ratio:

117: 115=E3TL: D3TL= 0.52

116: 114=E5TL: D5TL= 0.61

Annotated typical Precursor MS Region:

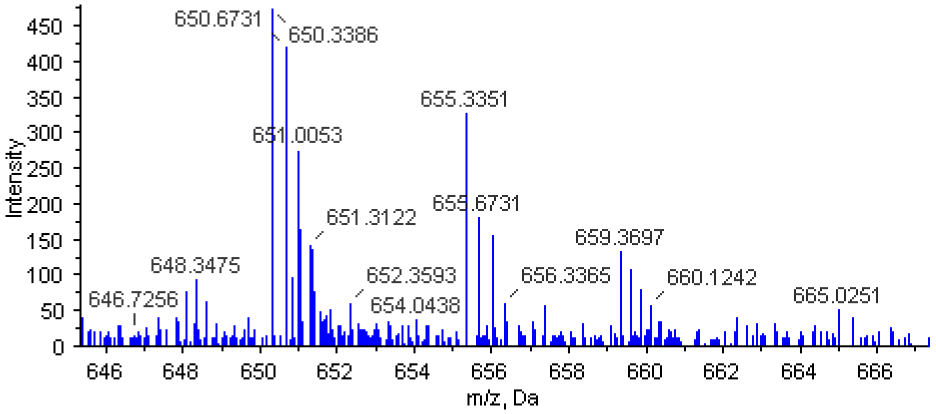

Sequence Coverage and Matched peptides: in different color

MELPRLYTCYTCPFAHRVWITRNFKGLQDEIKLVPLILQNRPAWYSEKVYPPNKVPSLEHNGKITGESL
DLIKYLESNFQGPSLLPEDPAKKEFAEELFSYTDTFNRTVFTSFKGDPAKEAGPAFDHLENALHKFGDG
PFFLGQEFSLVDIAYIPFVERFCIFLSEVFKYDITAGRPKLAAWIEELNKIEAYKQTKTDPKEMVEVYK
KRFMA

Protein No.: I-226

Protein name and Species:

Latex cystatin OS=Hevea brasiliensis PE=4 SV=1

Accession: tr|E8Z9H3|E8Z9H3_HEVBR

Unused ProtScore: 9.2

Seq Cov %: 32.7

ITRAQ Changed Ratio:

117: 115=E3TL: D3TL= 0.57

116: 114=E5TL: D5TL= 0.52

Annotated typical Precursor MS Region:

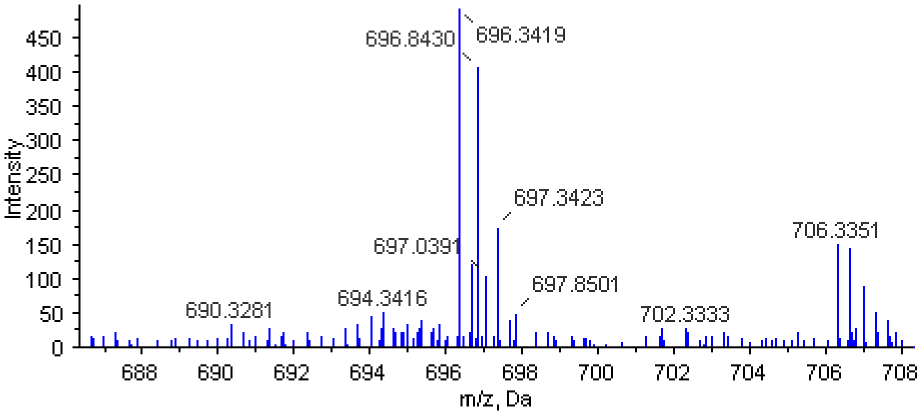

Sequence Coverage and Matched peptides: in different color

MAKLGGVKEVEGSANSVEINSLARYAVDDYNQKQNALLEFKKVVNAKQQVVAGTIYYITLEVIDGGQKK
VYEAKVWEKPWLNFKEVQEFKLIGDAPSDSTA

Protein No.: I-227

Protein name and Species:

Rab4 OS=Hevea brasiliensis PE=2 SV=1

Accession: tr|F2VXU6|F2VXU6_HEVBR

Unused ProtScore: 9.17

Seq Cov %: 40.6

ITRAQ Changed Ratio:

117: 115=E3TL: D3TL= 0.17

116: 114=E5TL: D5TL=0.40

Annotated typical Precursor MS Region:

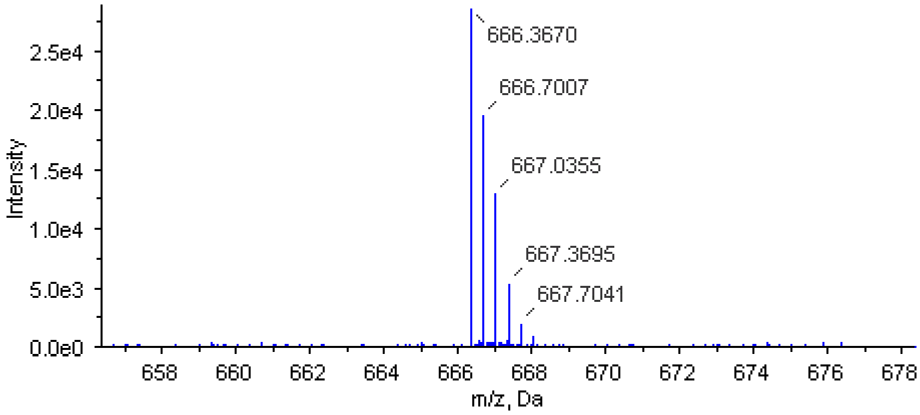

Sequence Coverage and Matched peptides: in different color

MSNEYDYLFK**LLLIGDSSVGKSCLLLRFADDSYVDSYISTIGVDFK**IR**TVELDGK**TIK**LQIWDTAGQER**
FR**TITSSYYR**GAHGIIIVYDVTEMESFNNVK**QWLNEIDRYANDSVCK**LLVGNKCDLVDNKVVDTQTAK**A**
FADELGIPFLETSAKDSINVEQAFLTMAGEIKKK**MGSQPAASK**STGTTVQMKGQPIQQKNNCCG

Protein No.: I-230

Protein name and Species:

6-phosphofructokinase 3 OS=Populus trichocarpa GN=POPTRDRAFT_866992 PE=4 SV=1

Accession: tr|B9H8X6|B9H8X6_POPTR

Unused ProtScore: 9.12

Seq Cov %: 14.9

ITRAQ Changed Ratio:

117: 115=E3TL: D3TL= 0.54

116: 114=E5TL: D5TL= 0.62

Annotated typical Precursor MS Region:

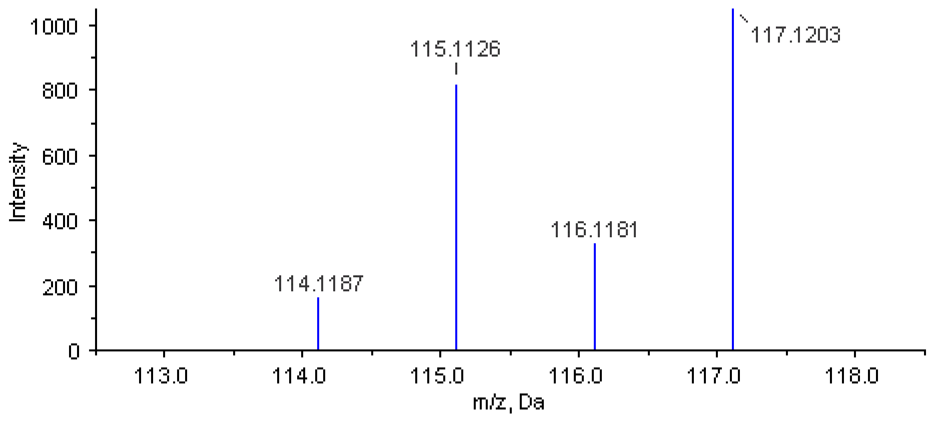

Sequence Coverage and Matched peptides: in different color

MASNDVSKPKIITGDFGYVLEDVPHFTDYIPDLSTYSNPLQDNPAFCVVK**QYFVHVDDSVPQK**IVVHKD
SPRGIHFRRAGPRQKVYFDSDEVHACIVTCGGLCPGLNTVIREIVYSLYHMYGVTT**VLGIDGGYR**GFYA
RNTIALTPKVVNDIHKRGGTILGTSRGGHDTSKIVDSIQDRGINQVYIIGGDGTQK**GASVIFEEIR**RRG
LKVVVAGIPKTIDNDIPVIDK**SFGFDTAVEEAQRAINAAHVEAESVENGIGLVK**LMGRYSGFIAMYATL
ASRDVDCCLIPESPFYLDGKGGLFEYIEKQLKENGHMVIVIAEGAGQELLSESMQSRNQQDASGNKLLQ
DVGLWISQGIKDYFSRQKTMAINLK**YIDPTYMIR**AVPSNASDNVYSTLLAQSAVHGAMAGYTGFTSGLV
NGRQTYIPFYRIIEKQNKVVITDRMWARLLSSTNQPSFMSDKDVIEDKTECISGEKKEEDPATQFLDNG
NRADGVLENKEVSSV

Protein No.: I-241

Protein name and Species:

Acyl-CoA-binding protein (Fragment) OS=Jatropha curcas PE=2 SV=1

Accession: **tr|D6BR55|D6BR55_9ROSI**

Unused ProtScore: **8.78**

Seq Cov %: **35.9**

ITRAQ Changed Ratio:

117: 115=E3TL: D3TL= 0.40

116: 114=E5TL: D5TL= 0.58

Annotated typical Precursor MS Region:

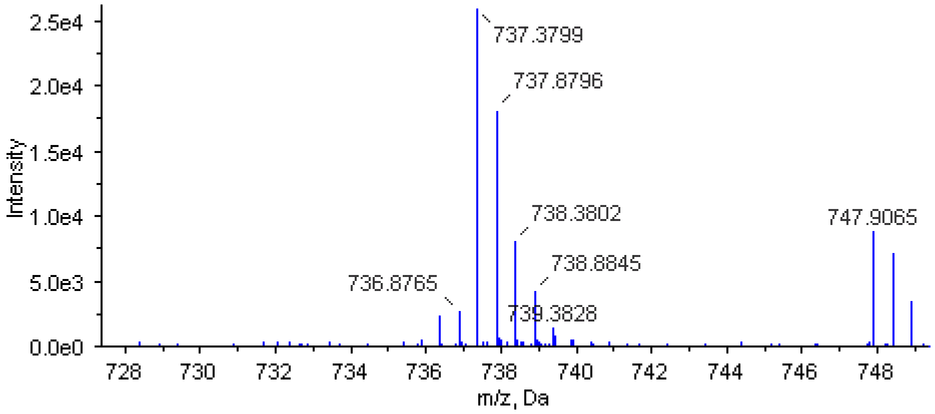

Sequence Coverage and Matched peptides: in different color

MGLKEEFEEYAEKAKTLPENTTNENKLILYGLFKQATVGPVNTSRPGMFNMRDRAKWDAWKAVEGKSKE
EAMSDYITKVKLLLEEAAAAASA

Protein No.: I-242

Protein name and Species:

Actin-depolymerizing factor OS=Hevea brasiliensis GN=ADF PE=2 SV=1

Accession: tr|D9I9X9|D9I9X9_HEVBR

Unused ProtScore: 8.71

Seq Cov %: 61.9

ITRAQ Changed Ratio:

117: 115=E3TL: D3TL= 0.58

116: 114=E5TL: D5TL= 0.58

Annotated typical Precursor MS Region:

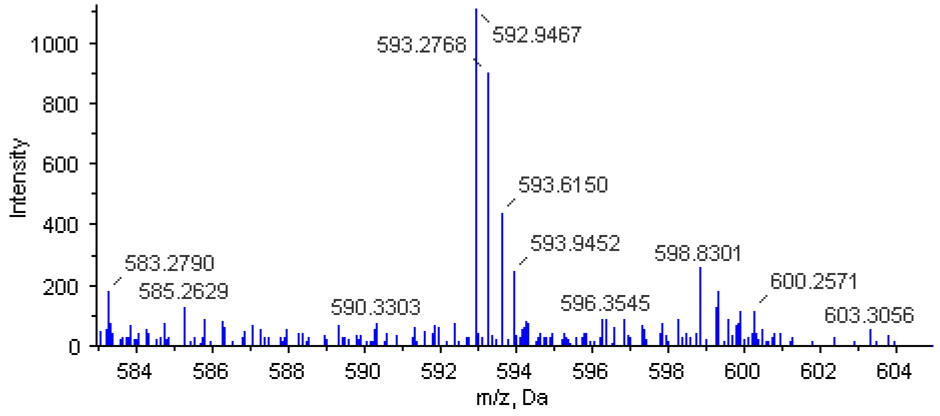

Sequence Coverage and Matched peptides: in different color

MANAASGMAVHDDCKLRFLELKAKRTYRYIVFKIEEKAKQVIVEKLGEPTQSYEDFTASLPADECRYAV
YDFDFVTEENCQKSRIFFIAWSPDTSRVRSKMIYASSKDRFKRELDGIQVELQATDPTEMGLDVFKSRA
S

Protein No.: I-244

Protein name and Species:

Proteasome subunit alpha type OS=Ricinus communis GN=RCOM_1595040 PE=3 SV=1

Accession: tr|B9R7X1|B9R7X1_RICCO

Unused ProtScore: 8.65

Seq Cov %: 23.3

ITRAQ Changed Ratio:

117: 115=E3TL: D3TL= 1.61

116: 114=E5TL: D5TL=1.50

Annotated typical Precursor MS Region:

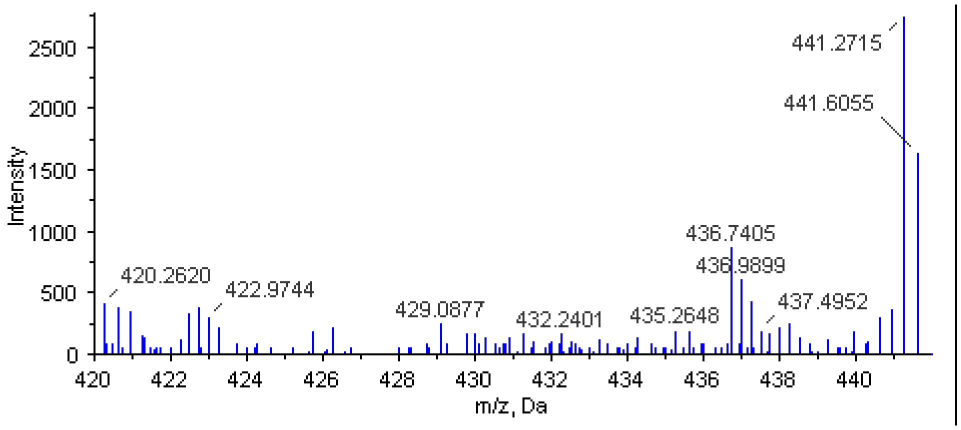

Sequence Coverage and Matched peptides: in different color

MFR**NQYDTDVTTWSPAGRLFQVEYAMEAVKQGSAAIGLR**SK**THVVLACVNK**ANSELSSHQKKIFK**VDDH**
IGVAIAGLTADGRVLSRYMR**SECINYSFTYESPLPVGRLVVQLADK**AQVCTQRSWKRPYGVGLLVAGLD
ESGAHLYYNCPSGNYFEYQAFAIGSRSQAAKTYMER**RFENFMDSSR**DDLIKDALIAVRETLQGETLKSS
ICTVAVVGVGEAFHVLDQETVQQLIDAFEIVGEPEAPAAEPDAAAEGGGGAAADQGGAAADQGGAAADE
GVAPMDI

Protein No.: I-245

Protein name and Species:

ubiquitin-protein ligase 1 OS=Hevea brasiliensis PE=2 SV=1

Accession: tr|F8RWS9|F8RWS9_HEVBR

Unused ProtScore: 8.65

Seq Cov %: 41.8

ITRAQ Changed Ratio:

117: 115=E3TL: D3TL= 0.65

116: 114=E5TL: D5TL=0.70

Annotated typical Precursor MS Region:

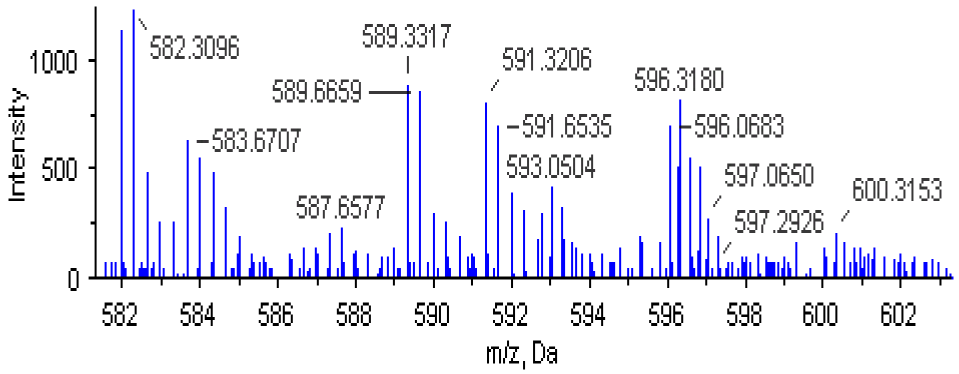

Sequence Coverage and Matched peptides: in different color

MTLGSGGSSVVVPRNFRLLEELERGEKGIGDGTVSYGMDDGDDIYMRSWTGTIIGPHNTVHEGRIYQLK
LFCDKDYPEKPPSVRFHSRINMTCVNHETGVVEPKKFGLLANWQREYTMEDILTQLKKEMAAPHNRKLV
QPPEGTYF

Protein No.: I-246

Protein name and Species:

Plant synaptotagmin OS=Populus trichocarpa GN=NTMC2T1.2 PE=4 SV=1

Accession: **tr|B9HR67|B9HR67_POPTR**

Unused ProtScore: **8.6**

Seq Cov %: **12.1**

ITRAQ Changed Ratio:

117: 115=E3TL: D3TL= 1.79

116: 114=E5TL: D5TL= 0.69

Annotated typical Precursor MS Region:

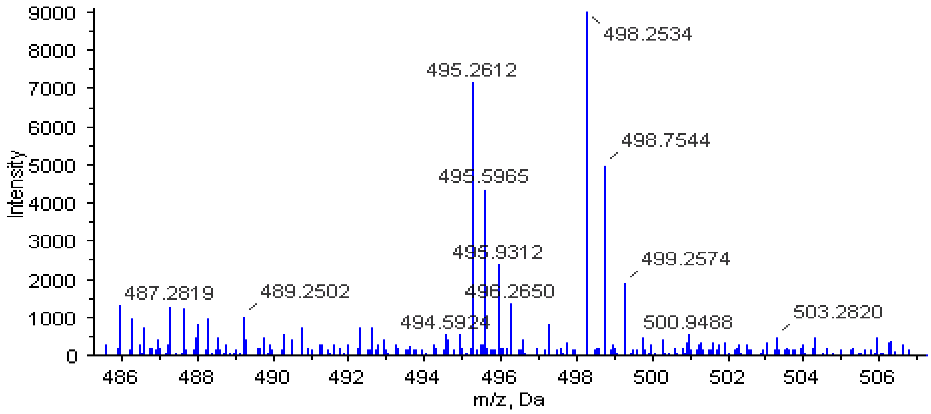

Sequence Coverage and Matched peptides: in different color

MGFLSTISGFCGFGVGLSTGLTIGYYLFIYFQPTDVKDPEVR**PLAEQDSETLQR**ILPEIPLWVKNPDYD
RIDWLNRFIQLMWPYLDKAICKTVKNIAKPIIAEQIPKYKIDAVEFETLTLGTLPPTFHGMKVYVTDEK
ELIMEPCIK**WAGNPNVTVAVK**AFGLKATAQVVDLQVFASPRITLKPLVPSFPCFANIYVSLMEKPHVDF
GLK**LLGADLMSIPGLYR**V**VQEIIK**DQVANMYLWPKTLEVPILDPAKAMKLKKK**DLMGASDPYVK**IKLTE
DKLPAKKTTVKHKNLNPEWNEEFNLVVKDPESQALELRVYDWEQVGKHDR**MGMNVVPLK**DLTPEEPK**VM**
TLDLLKNMDLNDPQNEKSRGQLMVELTYKPFKEDDLNKSFKDEVEQKAPEGTPAGGGLLVVTIHEAQDV
EGKHHTNPYVRLLFRGEEMKTKRVKKNRDPRWEEEFQFTLEEPPVNAKLH**VEVVSTSSR**IGLLHPKESL
GYVEINLSDVVSNRRINERYHLIDSKNGKIQIELQWRPAS

Protein No.: I-247

Protein name and Species: P5CS, Delta 1-pyrroline-5-carboxylate synthetase, putative OS=Ricinus communis GN=RCOM_1482350 PE=3 SV=1

Accession: tr|B9SE11|B9SE11_RICCO

Unused ProtScore: 8.57

Seq Cov %: 6.7

ITRAQ Changed Ratio:

117: 115=E3TL: D3TL= 2.03

116: 114=E5TL: D5TL= 1.66

Annotated typical Precursor MS Region:

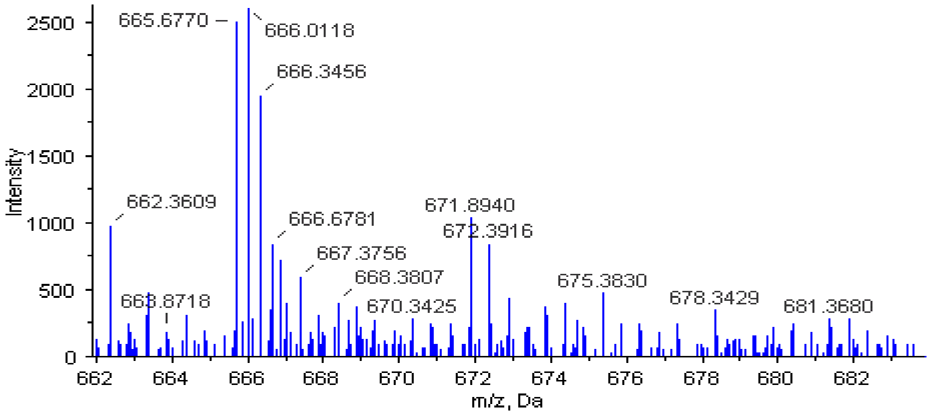

Sequence Coverage and Matched peptides: in different color

MNGGMDRSRAFVKDVKRLVIKVGTAVVTRGDGRLALGRLGAFCEQVKELNSQGYEVIVVSSGAVGLGRQ
RLRYRKLVNSSFADLQKPQVEIDGKASAAVGQSSLMALYDTMFSQLDVTSAQLLVTDNDFRDKDFRKQL
NETVESLLSLRVIPIFNENDAVSTRKAPYEDASGIFWDNDSLSALLALELKADLLVLLSDVDGLYSGPP
SDPRSKLIHTYIKEIHHGEITFGDKSRVGRGGMTAKVKAAVNAAYAGIPVIITSGFARENILKVLQGER
VGTLFHQDAHIWVPIKEVCGREMAVAARESSRRLQALSSQERKRILLDIADALEANEEQIKIENEADVA
AAQLAGLEKSLISRLALKSGKIKSLANSIRVLANMEDPIGCVLKRTELADGLLLEKTSSPLGVLLIIFE
SRPDALVQIASLAIRSGNGLLLKGGKEAKRSNEILHKIITAAIPETIGGKLIGLVTSRDEIPDLLKLDD
VIDLVIPRGSNKLVSQIKASTKIPVLGHADGICHVYVDKSANMEMAKRIVLDAKIDYPAACNAMETLLV
HKDLVQTGGLNELTVDLRTEGVNLYGGPRASKELNVPEVDSFHHEYNSMACTIEIVDDVYAAIEHIHEH
GSAHTDCIITEDHEVAEVFLCQVDSAAVFHNASTRFSDGARFGLGAEVGISTSRIHARGPVGVEGLLTT
RWILRGNGQVVDGDKGVIYTHKDVTHIHSNGFVPASLTKEDN

Protein No.: I-248

Protein name and Species:

adenosine kinase 2 [Vitis vinifera] OS=Vitis vinifera GN=VIT_13s0019g04480 PE=4 SV=1

Accession: tr|F6HNF4|F6HNF4_VITVI

Unused ProtScore: 8.56

Seq Cov %: 16.7

ITRAQ Changed Ratio:

117: 115=E3TL: D3TL= 0.34

116: 114=E5TL: D5TL= 0.50

Annotated typical Precursor MS Region:

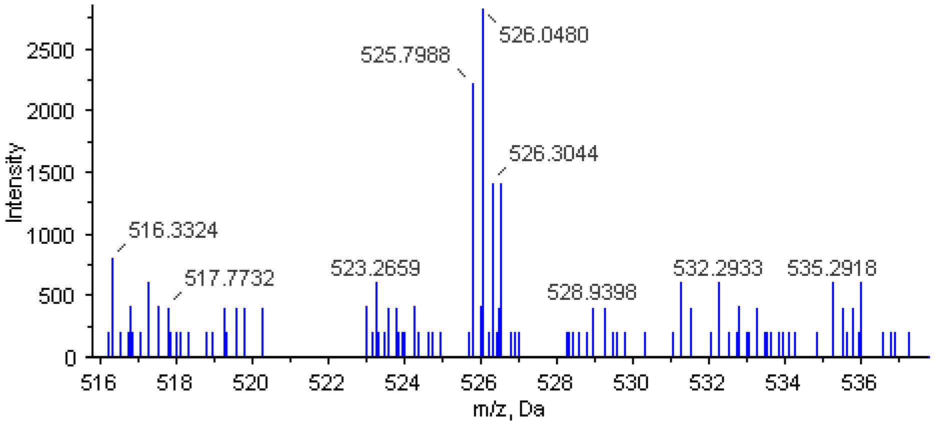

Sequence Coverage and Matched peptides: in different color

MAYEGILLGMGNPLLDISSVVDEEFLQRYDIK**LNNAILAEDKHLPMYDEMASKYNVEYIAGGATQNSIR**
VCQWMLQIPGATSYMGCIGKDK**FGEEMK**KNSKLAGVNVHYREDETAPTGTCAVCVVGGER**SLIANLSAA**
NCYKSEHLKRPENWALVEKAKYFYIAGFFLTVSPESILLVAEHAAANNKVFMMNLSAPFICEFFKDQQE
KALPYMDYVFGNETEARTFSR**VHGWETDNVEEIAIK**ISQWPKASGTHKR**ITVITQGADPVVVAEDGK**VK
SFPVILLPKEKLVDTNGAGDAFVGGFLSQLVQEKPIEECVRAGCYASHVIIQR**SGCTYPEKPDFS**

Protein No.: I-256

Protein name and Species:

Fructose-bisphosphate aldolase OS=Populus trichocarpa GN=POPTRDRAFT_832739 PE=3 SV=1

Accession: tr|B9HJ73|B9HJ73_POPTR

Unused ProtScore: 8.36

Seq Cov %: 19.2

ITRAQ Changed Ratio:

117: 115=E3TL: D3TL= 0.66

116: 114=E5TL: D5TL= 0.75

Annotated typical Precursor MS Region:

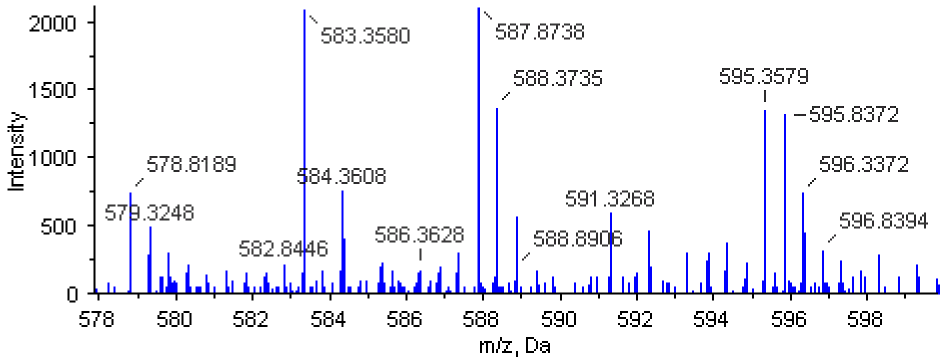

Sequence Coverage and Matched peptides: in different color

MACASFVKLNAASSSWTGQKSSFGKRSPGSSTRRVSFSIRASSYTDELVQTAKLIASPGR**GILAIDESN**
ATCGKRLASIGLDNTETNRQAYRQLLLTTPGLGEYISGAILFEETLYQSTTDGRK**FVDCLRDENIVPGI**
KVDK**GLVPLPGSNNESWCQGLDGLASR**SAEYYKQGARFAKWR**TVVSIPCGPSALAVK**EAAWGLARYAAI
SQDNGLVPIVEPEILLDGDHPIDRTLEVAEKVWAEVFYYLAENNVVFEGILLKPSMVTPGAEHKEKASP
DTIAKYTLTMLKRRVPPAVPGIMFLSGGQSEVQATLNLNAMNQSPNPWHVSFSYAR**ALQNTVLK**TWQGR
PENVEAAQKSLLVRAKANSLAQLGRYSAEGESEEAKKGMFVKGYTY

Protein No.: I-261

Protein name and Species:

Arginyl-tRNA synthetase, putative OS=Ricinus communis GN=RCOM_1049090 PE=3 SV=1

Accession: tr|B9RKG2|B9RKG2_RICCO

Unused ProtScore: 8.15

Seq Cov %: 14.2

ITRAQ Changed Ratio:

117: 115=E3TL: D3TL= 0.53

116: 114=E5TL: D5TL= 0.96

Annotated typical Precursor MS Region:

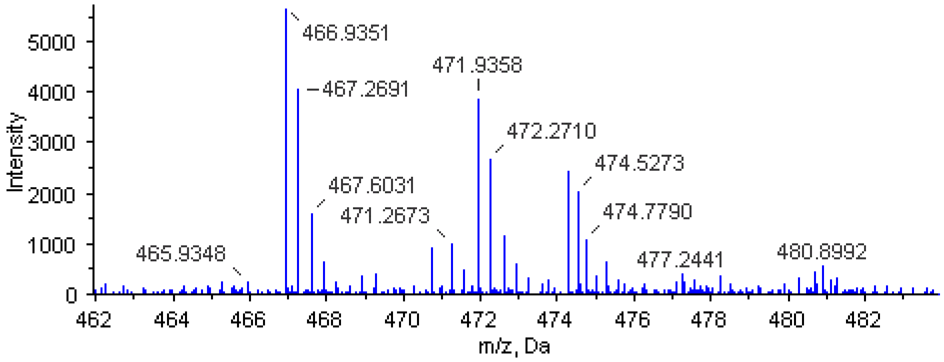

Sequence Coverage and Matched peptides: in different color

MATNAENEDNVGNLKKQIAKLFEASLRETVPSEPDVEPLVAACTAKFGDYQCNNAMSLWSKIKGKNTEF
KGPPAVGQAIMKNLPPSEVIESCSVAGPGFVNVVLSKKWMSKKIQKMLIDGIDSWAPKLLVKRAVVDFS
SPNIAKEMHVGHLRSTIIGDTLARMLEFSKVDVLRRNHVGDWGTQFGMLIEFLFEKFPNFEDVNERAIG
DLQAFYKASKQRFDADSEFKERAQKAVVRLQGGEPKYRQAWAQICDISRKEFDKVYQRLGVQLEEKGES
FYNPYIPGIIDALSNQGLVEESEGARVIFIEGVNIPLIVVKSDGGFNYASTDLTALWYRLNEEKAEWII
YVTDVGQQQHFDMVFKAAKRASWLPADDSMFPKASHVGFGLVLGDDGKRFRTRATEVVRLVDLLDEAKT
RSKAVLVERGKAEEWTEQELEQIAEAIGYGAVKYADLKNNRLTNYTFNYDQMLNDKGNTAVYLLYAHAR
ICSIIRKSGRDMEELKKMGSLVLDHPDERSLGLHLLQFAETVEEACTNLLPNVLCEYLYNLSEYFTKFY
SNCKVVESPEETSRLLLCEATAVVMRKCFFLLGIVPVYKI

Protein No.: I-262

Protein name and Species: Carotenoid cleavage dioxygenase 1 OS=Manihot esculenta GN=CCD1 PE=2 SV=1

Accession: tr|E2DQG6|E2DQG6_MANES

Unused ProtScore: 8.15

Seq Cov %: 10.3

ITRAQ Changed Ratio:

117: 115=E3TL: D3TL= 0.20

116: 114=E5TL: D5TL= 0.65

Annotated typical Precursor MS Region:

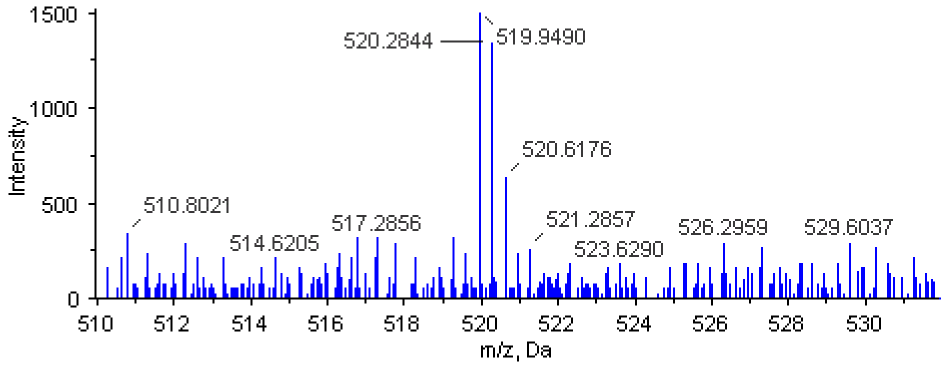

Sequence Coverage and Matched peptides: in different color

MAEEKSEVEKKQGNSGGAGVPIVEVKPKPRKGLASKFVDLLEKLIVKLMFDASKPLHYLSGNFAPVTDE
TPPVRDLSVKGHLPDCLNGEFVRVGPNPKFAPVAGYHWFDGDGMIHGMRIKNGKATYVRRYVRTSR**IQQ**
EEFFGGSKIMKVGDLKGLFGLFMVNMQILRAKLKVLDMSYGNGTANTALIYHHGKLLALQEADKPYVVK
VLEDGDLQTVGMLDYDKRLKHSFTAHPKVDPNTGEMFTFGYAHEPPYITYRVISKDGVMHDPVPITISD
PIMMHDFAITENYAIFLDLPLYFRPKEMVKDKKLIFTFDATKKARFGVLPRYAKDDHQIRWFELPNCFI
FHNANAWEEEDEVVLITCRLENPDLDMVSGNVKEKLENFANELYEMRFNMKTGVASQKRLSAPAVDFPR
VNESYTGRKQR**YVYGTILDSIAK**VTGIIK**FDLHAEPQQGK**TKLEVGGNIKGIFDLGPGRFGSEAVFVPR
EPGTSSEEDDGYLIFFTHDENTGKSSVNVIDAK**TMSSDPVAVVELPHR**VPYGFHAFFVTEEQLQAQATL

Protein No.: I-264

Protein name and Species:

Glutaredoxin OS=Hevea brasiliensis PE=4 SV=1

Accession: **tr|B3FNP8|B3FNP8_HEVBR**

Unused ProtScore: **8.09**

Seq Cov %: **43**

ITRAQ Changed Ratio:

117: 115=E3TL: D3TL= 0.28

116: 114=E5TL: D5TL= 0.31

Annotated typical Precursor MS Region:

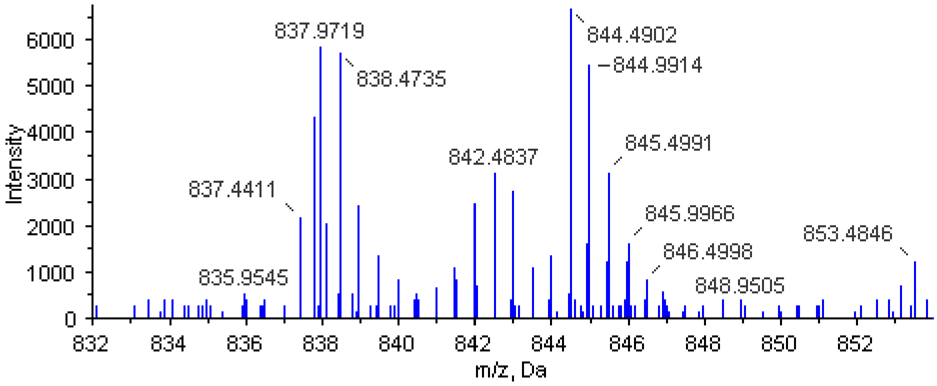

Sequence Coverage and Matched peptides: in different color

MAMTKAK**ELVSSNSVVVFSKTYCPYCTSVK**K**LLDELGANYK**TVELDTEGDGSQVQSALAEWTGQR**SVPN**
VFISGKHIGGCDTTTGMHKEGK**LIPLLTEAGALAK**ASA

Protein No.: I-266

Protein name and Species:

ras-related protein RABA3 OS=Ricinus communis GN=RCOM_0143940 PE=3 SV=1

Accession: tr|B9T509|B9T509_RICCO

Unused ProtScore: 8.05

Seq Cov %: 42

ITRAQ Changed Ratio:

117: 115=E3TL: D3TL= 0.46

116: 114=E5TL: D5TL= 0.58

Annotated typical Precursor MS Region:

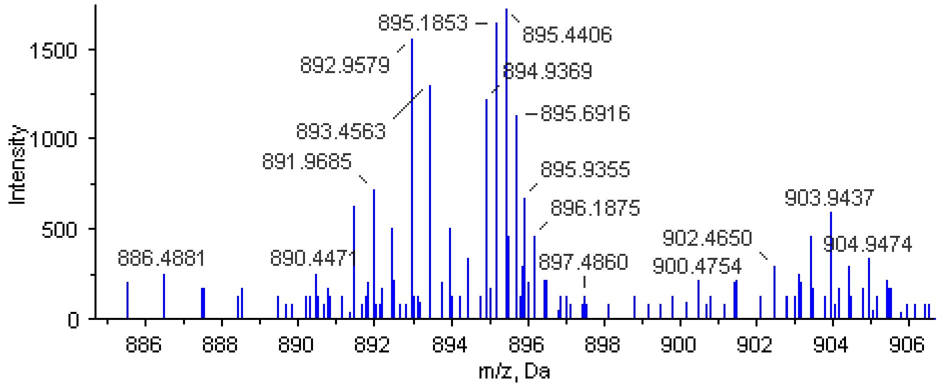

Sequence Coverage and Matched peptides: in different color

MNQEMNGDDQENLQEKIDYVFKVVVIGDSAVGKTQLLSRFTKNEFCFDSKSTIGVEFQTRTVTIKGKVV
KAQIWDTAGQERYRAVTSAYYRGALGAMLVYDITKRATFDHVARWVEELRAHADNSIVITLIGNKADLV
DQRAVPTEDAVEFAEDQGLFFSETSALSGDNVDRAFFRLLEEIYGVISKKSLECGGHKTNGADSVMLRG
SKIDVISGSDLEISEMKKLSACSC

Protein No.: I-267

Protein name and Species:

Peroxiredoxin, putative OS=Ricinus communis GN=RCOM_1052060 PE=4 SV=1

Accession: tr|B9RKM9|B9RKM9_RICCO

Unused ProtScore: 8.02

Seq Cov %: 29

ITRAQ Changed Ratio:

117: 115=E3TL: D3TL= 0.39

116: 114=E5TL: D5TL= 0.12

Annotated typical Precursor MS Region:

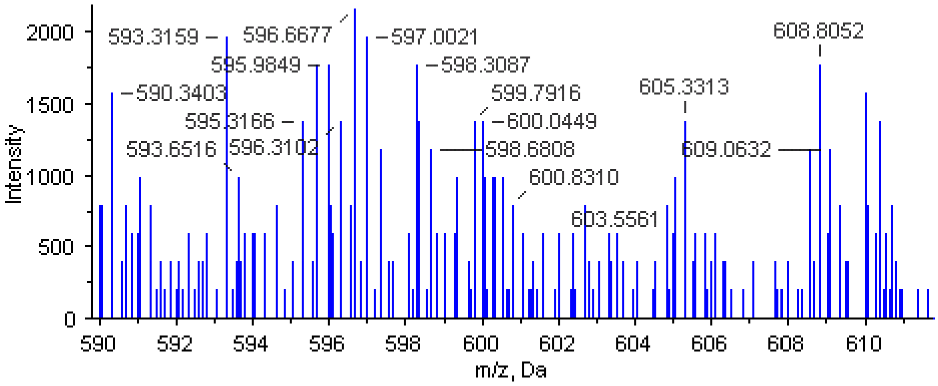

Sequence Coverage and Matched peptides: in different color

MAPIAAGDTLPDGTLAYFDEQDQLQQVSIHSLAAGK**KVVIVGVPGAFTPTCSLK**HVP**GFIEK**AEELKSK

GVAEILCLSVNDPFVMKAWAKTYPENKHVKFLADGSATYTH**ALGLELDLK**EKGLGTRSR**RFALLVDDLK**

VK**AANLEEGGEFTVSSVDEILK**AL

Protein No.: I-268

Protein name and Species:

Ubiquilin, putative OS=Ricinus communis GN=RCOM_0873810 PE=4 SV=1

Accession: **tr|B9S6T7|B9S6T7_RICCO**

Unused ProtScore: **8**

Seq Cov %: **9.3**

ITRAQ Changed Ratio:

117: 115=E3TL: D3TL= 1.53

116: 114=E5TL: D5TL= 1.67

Annotated typical Precursor MS Region:

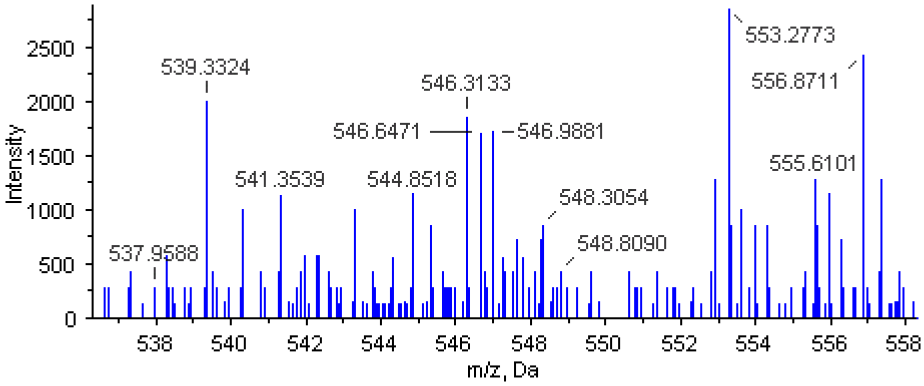

Sequence Coverage and Matched peptides: in different color

MGGGNGASVDESSNGGDEVNVNIRCSNGSKFSVGISLSESVETFKTVLAGKCDVPADQQRLIYKGRILK

DDQTLQSYGLEADHTVHLVRGFAPAAAANTAPPSNTGGSNPASTNTRAVGSNEGGALGGSDLGLFPGMR

LNGLGGSAGLFGAGLPEFEQVQQQLTRNPNIMREIMNTPVVQNLMNNPEIMRNLIMNNPQMR**EIIDRNP**

ELAHILNDPSTLRQTLEAARNPELMREMMRNTDR**AMSNIESSPEGFNMLR**RMYETVQEPFLNATTMAGN

TGGDSANPFAALLGNQAGNQASDATTNPSIASSESTTGSPAPNTNPLPNPWTATGTGGALNNTARSSNT

GDARPQTPVGLGGLGLPEFDSMFGAMPDSNVMSQMLQNPAISQMMQSLLSNPQYMNQMLGSNPQFRSML

DSNSQFREMMQNPEFLRQLTSPETMQQLLTFQQALLSQFGRQQSNQEPGQTGAGTGTPNNMGLDMLMNM

FGGLGAGSLAVPNRSNVPPEELYATQLSQLQEMGFFDTQENIR**ALIATAGNVHAAVER**LLGNSGQ

Protein No.: I-269

Protein name and Species: **2,3-bisphosphoglycerate-independent phosphoglycerate mutase OS=Populus trichocarpa GN=POPTRDRAFT_825441 PE=4 SV=1**

Accession: **tr|B9IGY9|B9IGY9_POPTR**

Unused ProtScore: **7.99**

Seq Cov %: **7.5**

ITRAQ Changed Ratio:

117: 115=E3TL: D3TL= 0.61

116: 114=E5TL: D5TL= 0.57

Annotated typical Precursor MS Region:

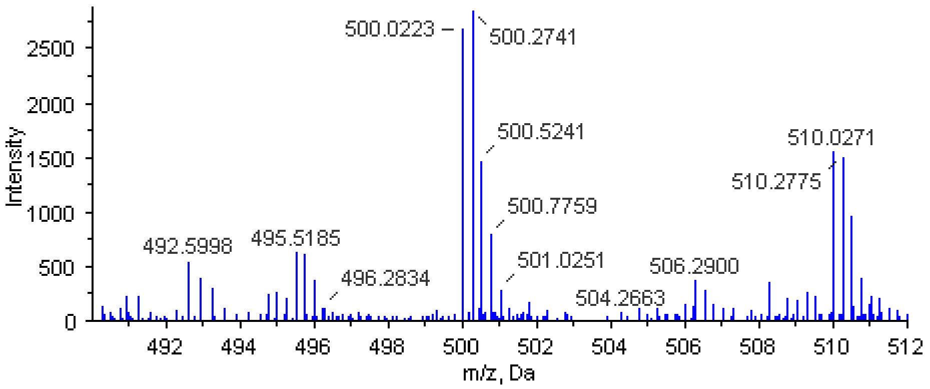

Sequence Coverage and Matched peptides: in different color

MGSPGQNAWKLADHPKLPKGKTIAMVVLDGWGEAKPDQYNCIHVAHTPTMDSFKTTAPEKWRLIKAHGT
AVGLPSEDDMGNSEVGHNALGAGRIFAQGAK**LVDLALASGKIYDGEGFK**YIKECFDNGTLHLIGLLSDG
GVHSR**LDQLQLLLK**GAVENGAKRIRVHILTDGR**DVLDGSSIGFVETLEK**DLSNLREKGIDAQIASGGGR
MYVTMDRYENDWDVVKRGWDAQVLGEAPYKFRNAVEAVKKLREEPKANDQYLPPFVIVDESGNPVGPIK
DGDAVVTFNFRADRMAMLAK**ALEYEDFDKFDR**VRVPK**IHYAGMLQYDGELK**LPSHYLVSPPEIDR**TSGE**
YLVHNGIRTFACSETVKFGHVTFFWNGNRSGYFNQEMEEYVEIPSDV**GITFNVQPK**MK**AIEIAEK**ARDA
ILSGKFDQVR**VNLPNGDMVGHTGDIEATVVACK**VADDAVKMIIDAIEQVGGIFVITADHGNAEDMVKRD
KSGKPLLDKNGNLQILTSHTLQPVPIAIGGPGLAPGARFRNDVPTGGLANVAATVMNLHGFEAPSDYEP
TLIEVVDN

Protein No.: I-278

Protein name and Species:

26S proteasome non-atpase regulatory subunit, putative OS=Ricinus communis GN=RCOM_0974240 PE=4 SV=1

Accession: tr|B9SXW5|B9SXW5_RICCO

Unused ProtScore: 7.82

Seq Cov %: 20.6

ITRAQ Changed Ratio:

117: 115=E3TL: D3TL= 0.58

116: 114=E5TL: D5TL= 0.62

Annotated typical Precursor MS Region:

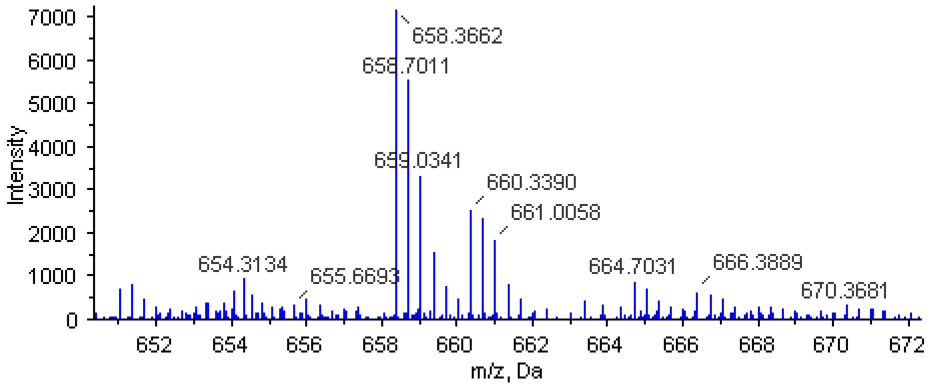

Sequence Coverage and Matched peptides: in different color

MVLEATMICIDNSEWMRNGDYNPSRFHAQADAVNLICGAKTQSNPENTVGVLTMAGKGVRVLVTPTSDL
GKILACMHGLEIGGEMNLAAGIQVAQLALKHRQNKKQQQRIIVFAGSSIKHDKKALEMIGKKLKKNNVA
LDIVDFGEEDDEKAEKLEALLAAVNSNDTSHIVHVPPGPSALSDVLISTPVFTGDGEGGSGFAAAAAAA
AAGGVSGFEFGVDPNLDPELALALRVSMEEERARQEAAAKKAAEEAAKQEKGGEQPSSSQDATMTESAS
VTASEADNKRHDLTNEENALLQQALAMSMDDPASSQELRDTDMSEAAADDPELALALQLSVQDSPKDSG
NPTDMSKLLADQSFVSSILASLPGVDPNDPSVKDLLASMQSQSEPQEKKDEDKPKEEK

Protein No.: I-279

Protein name and Species:

FAS-associated factor 2 OS=Populus trichocarpa GN=POPTRDRAFT_554543 PE=4 SV=1

Accession: tr|B9GXV5|B9GXV5_POPTR

Unused ProtScore: 7.78

Seq Cov %: 14.4

ITRAQ Changed Ratio:

117: 115=E3TL: D3TL= 0.47

116: 114=E5TL: D5TL= 0.67

Annotated typical Precursor MS Region:

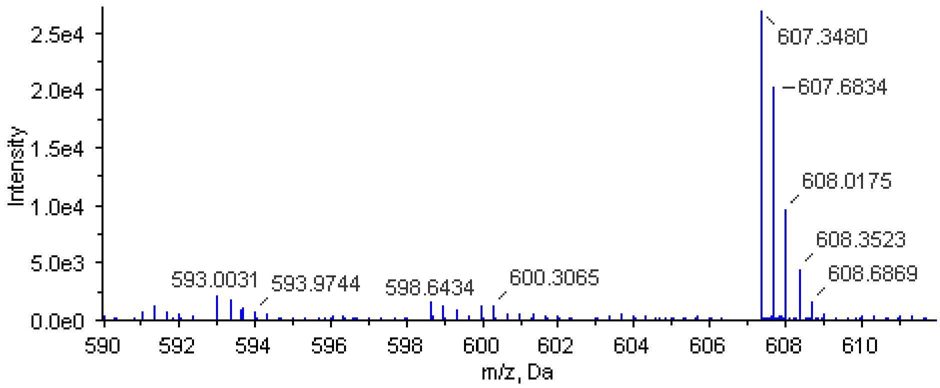

Sequence Coverage and Matched peptides: in different color

MVDVADKLAYFQAITGLEDPDLCTQILQAHNWDLELAISSFTSNHNNDPLSYSSSTATATTSTVVEPPI
SSIHRSDSTAIAPVAPPPPGLGWKIITLPISIVSGSLRLISGAVGFGFWAAGSIFSYSLGFIGFSSNSG
RGGDSSAQLVTVSAASREALEFIAQFERDYGGSGSSSRPNFVGEGFMDALTRSRNSFKLLFVYLHSPDH
PDTPAFCEGTLCSEVFSAFVNENFVAWGGSLRGSEGFKMSNSLKASR**FPFCAVVMPATNQRIALLQQVE**
GPKSPEEMLMTLQRVLEESAPVLVAARLEAEERTTNMR**LREEQDAAYR**AALEADQARERQLREEQERLE
REAAEAERKRKEEEEAQERATREAAEKEAALARMRQEK**ALSLGAEPEKGPNVTQVLVR**FPTGERKERRF
HSTATIQSLYDYVDSLGCLDVENYSLVSNFPRVVYGTDKVSLSLKEAGLHPQASLFVELN

Protein No.: I-280

Protein name and Species:

Putative uncharacterized protein OS=Ricinus communis GN=RCOM_0799000 PE=4 SV=1

Accession: tr|B9RRX1|B9RRX1_RICCO

Unused ProtScore: 7.72

Seq Cov %: 3.6

ITRAQ Changed Ratio:

117: 115=E3TL: D3TL= 0.34

116: 114=E5TL: D5TL= 0.50

Annotated typical Precursor MS Region:

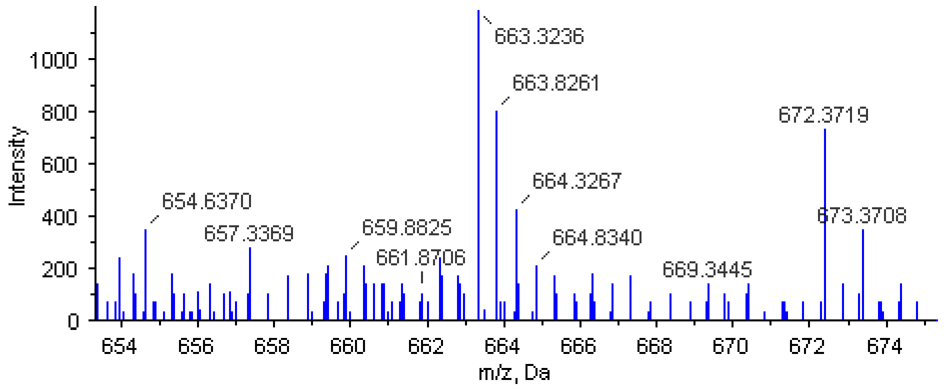

Sequence Coverage and Matched peptides: in different color

MARFYSSVTLFFNFAIIITTTLISNPNFVISSNNVHTSFSIIDYDSNLFHQDYSPPSPPPPPPHAPSVS
CTDDLGGIGSLDTTCRIISNVNLTRDVYIAGKGNFYIHPGVSFNCLSFGCSVTINITGNFTLSINASIV
TSSFELVAYNASFSNNSVVNTTGLAGNPPPQTSGTPQGIDGAGGGHGGR**GACCLVDDK**KLPEDVWGGDA
YSWSSLQIPNSYGSRGGSTSKEVNYGGGGGGKVKFTISEYLVVDGGILADGGDGGSKGGGGSGGSIFIK
AYKMTGSGR**ISACGGSGFAGGGGGRVSVDIFSR**HDDPQIFVHGGSSF**GCPENAGAAGTLYDAVPR**SLIV
SNHNMSTDTETLLLDFPYQPLWTNVYVRNHARATVPLLWSRVQVQGQISLLCHGVLSFGLAHYASSEFE
LLAEELLMSDSVIKVYGALRMTVKIFLMWNSKMIVDGGEDTTVTTSWLEASNLIVLKESSVIQSNANLG
VHGQGLLNLSGPGDSIEAQRLVLSLFYSIHVGPGSVLRGPLQNATSDAVTPRLYCELQDCPIELLHPPE
DCNVNSSLSFTLQICRVEDITVEGLIKGSVVHFHRARTVSVLSSGRISASGMGCTGGVGRGHVLENGIG
SGGGHGGKGGLGCYNGSCIEGGMSYGNVELPCELGSGSGDESSAGSTAGGGIIVMGSLDHPLSSLSVEG
SVRADGESFQQTVKLGKLTVKNDTTGGPGGGSGGTILMFLHTLDLSESAVLSSGGGYGSQNGAGGGGGG
RIHFHWSDIPTGDVYQPIASVKGSILFGGGTGRDEGCAGENGTVTGKACPKGLFGVFCEECPAGTFKNV
TGSERSLCHPCPANELPHRAVYVAVRGGIAETPCPYKCISDRFHMPHCYTALEELIYTFGGPWLFCLLL
VALLILLALVLSVARMKFVGVDELPGPAPTQHGSQIDHSFPFLESLNEVLETNRAEESQNHVHRMYFMG
PNTFSEPWHLPHTPPEQIKEIVYESAYNSFVDEINAITAYQWWEGAMYSILSALLYPLAWSWQQWRRRI
KLQKLREFVRSEYDHACLRSCRSRALYEGLKVAATPDLMLAYLDFFLGGDEKRTDLPPRLHQRFPMSII
FGGDGSYMAPFSIQSDNILTSLMSQTVPPTTWYRMVAGLNAQLRLVRRGRLRVTFRSVIKWLETHANPA
LRIHGIRVDLAWFQATACGYCQYGLLVYAIEEETGESIDGGKQTLQESRENYTRRKKSYWGSIDTNNLQ
MLEEKRDIFCLLSFIIHNTKPVGHQDLVGLVISMLLLGDFSLVLLTLLQLYSISLVDVLLVLLILPLGI
LLPFPAGINALFSHGPRRSAGLARIYALWNVMSLINVVVAFVCGYVHYHSQSSSSKKFPFQPWNISMDE
SEWWIFPAGLVLCKILQSQLVNWHVANLEIQDRSLYSSDFELFWQS

Protein No.: I-281

Protein name and Species:

Glutamate decarboxylase, putative OS=Ricinus communis GN=RCOM_0112580 PE=3 SV=1

Accession: tr|B9SR96|B9SR96_RICCO

Unused ProtScore: 7.7

Seq Cov %: 12.3

ITRAQ Changed Ratio:

117: 115=E3TL: D3TL= 3.25

116: 114=E5TL: D5TL= 0.90

Annotated typical Precursor MS Region:

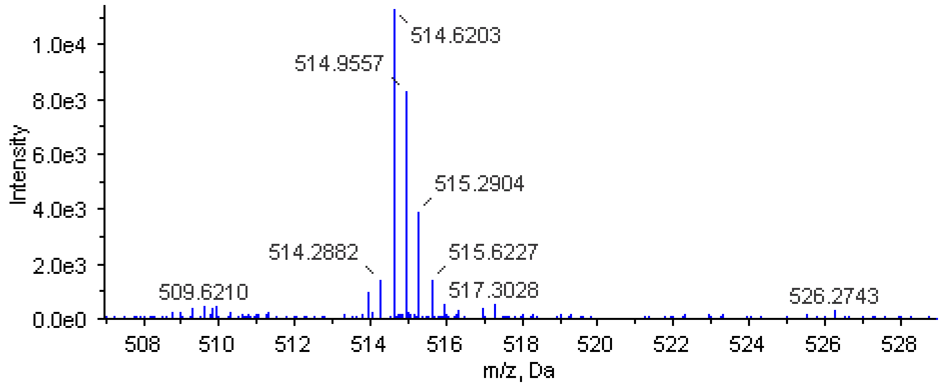

Sequence Coverage and Matched peptides: in different color

MVLSKTASESDVSVHSTFASRYVRASLPRFKMPESSIPK**EAAYQIINDELMLDGNPR**LNLASFVTTWME

PECDKLIMASINK**NYVDMDEYPVTTELQNR**CVNMIAHLFNAPLGDSETAVGVGTVGSSEAIMLAGLAFK

RKWQNKRKAEGKPYDKPNIVTGANVQVCWEKFARYFEVELKEVKLRDGYYVMDPVKAVEMVDENTICVA

AILGSTLNGEFEDVKLLNDLLTEKNKQTGWDTPIHVDAASGGFIAPFLYPELEWDFRLPLVKSINVSGH

KYGLVYAGIGWVIWRNKDDLPDELIFHINYLGADQPTFTLNFSK**GSSQVIAQYYQLIR**LGYEGYRNIME

NCRDNMLVLKDGLEKTGRFTIVSK**DNGVPLVAFSLK**DNSSHNEFEISELLRRFGWIVPAYTMPPDAQHV

TVLRVVIREDFSRTFAER**LVLDIEK**VLHELDTLPSRLREKIAIVEEKEKNGTKAAKKSVIETQREITTI

WKKFVLEKKKMNGVC

Protein No.: I-284

Protein name and Species:

hydroxycinnamoyltransferase ; Quinate O-hydroxycinnamoyltransferase/shikimate O-hydroxycinnamoyltransferase OS=Populus trichocarpa PE=2 SV=1

Accession: tr|B2Z6Q5|B2Z6Q5_POPTR

Unused ProtScore: 7.66

Seq Cov %: 15

ITRAQ Changed Ratio:

117: 115=E3TL: D3TL= 0.38

116: 114=E5TL: D5TL= 1.00

Annotated typical Precursor MS Region:

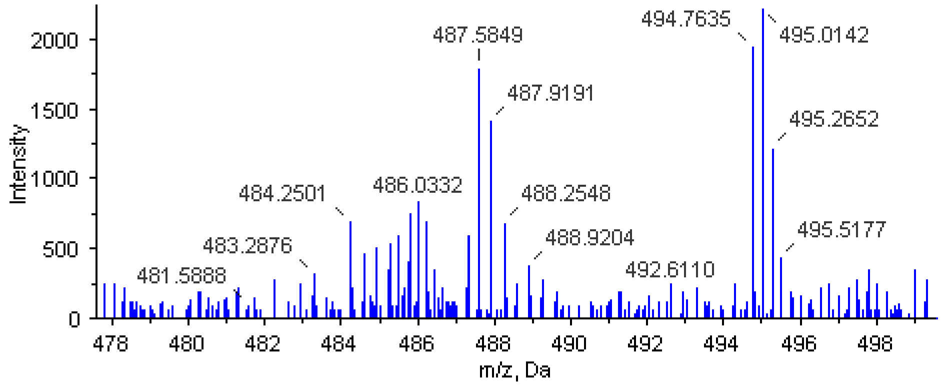

Sequence Coverage and Matched peptides: in different color

MIINVKESTMVQPAEETPRRGLWNSNVDLVVPR**FHTPSVYFYR**PTGASNFFDAKVLKEALSK**ALVPFYP**
MAGRLRRDDDGRIEIDCNAEGVLFVEAGTASVVADFGDFAPTLELKQLIPTVDYSGGISTYPLLVLQVT
YFKCGGVSLGVGMQHHAADGFSGLHFVNTWSDMAR**GLDLTIPPFIDR**TLLRAR**DPPQPAFHHVEYQPPP**
AMKTVLETSKPESTAVSIFKLTRDQLNTLKAKAKEGGNNIGYSSYEMLAGHVWRSACKARGLPDDQETK
LYIATDGRSRLRPTLPPGYFGNVIFTATPIAVAGEIQSKPTWYAAGKIHDSLVR**MDNDYLRSALDFLEL**
QPDLSALVRGAHTFR**CPNLGITSWVR**LPIHDADFGWGRPIFMGPGGIAYEGLSFIIPSSTNDGSLSVAI
SLQAEHMKLFEKFIYDIKE

Protein No.: I-289

Protein name and Species:

Eukaryotic translation initiation factor 2c, putative OS=Ricinus communis GN=RCOM_0476750 PE=4 SV=1

Accession: tr|B9SCN8|B9SCN8_RICCO

Unused ProtScore: 7.53

Seq Cov %: 7.3

ITRAQ Changed Ratio:

117: 115=E3TL: D3TL= 1.50

116: 114=E5TL: D5TL= 1.39

Annotated typical Precursor MS Region:

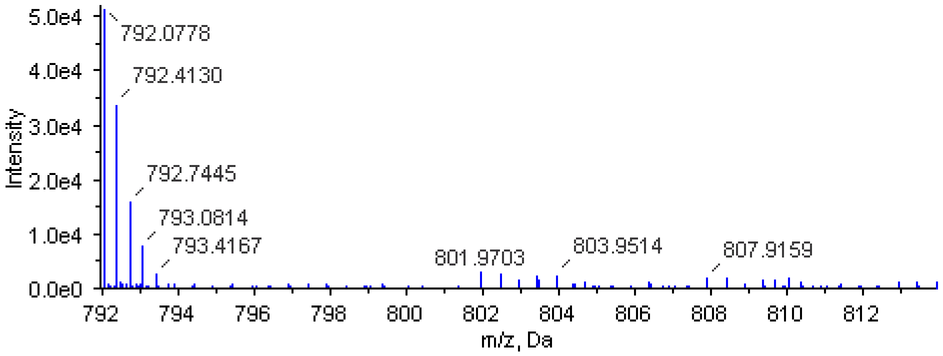

Sequence Coverage and Matched peptides: in different color

MSHRGGGRRQESQRDRQSSATSPSFNRGGGGGRGGRGRGGAGSFYAQPAPPPAGSDFPSLSRPPTTSSR
AAAAPQAAPPSSSSRAPPAPAPAPAPPPAAAASTSTQIEKLTHGVEQLTTTAGAPTPSSSKEIRFPNRP
GYGSIGMKCVVKANHFLVDVADRDLRQYDVSITPELTSKKINRDVISQLIRMFRQSHLGNRRAAYDGRK
SLYTAGPLPFESKEFVVKLVESNKNAGSSVSSKKEREFKVAIKFASKPDIHHLKQFLIGRQMDCPQETI
QVLDIVLRETPSEKYTPVGRSFFSPDLGQKGELGDGIEYWRGYYQSLRPTQMGLSLNIDVSARSFYEPI
IVTDFVSKYLKLRDMSRPLSDQDRIKVKKALKSVKVQILHREYAKSYKVTGISNKPLNQIFFKLDDKST
DISVVQYFREKYNIGLKYTSLPALQAGSDAKPIYLPMELCKIVDGQRYSKKLNERQVTALLRATCQRPH
EREESIKQMVKRNSYNQDVLVRDEFGIQVK**EELTFVDAR**VLPAPMLNYHETGRESRVDPRCGQWNMINK
KMVNGGSVNFWTCVNFSLNINRDLPAEFCR**QLIQMCVSK**GMAFNPNPIIPISSAHPGQIGKTLNDIKRQ
CEAKLVKQLQLLIIILPDISGSYGIIKR**VCETELGIVSQCCQPR**QAAKLSKQYFENVALKINVKVGGRN
TVLNDAVQRRIPLVTDCPTIIFGADVTHPPPGEDSSPSIAAVVASMDWPEVTKYRGIVSAQAHR**EEIIQ**
DLYKSFQDPQGILKHSGMIRELFVAFRRETGMKPKRIIFYRDGVSEGQFSQVLLYEMDAIRKACASLEE
GYLPPVTFVVVQKRHHTRLFPVDRGQTDR**SGNILPGTVIDTK**ICHQREFDFYLNSHAGIQGTSRPTHYH
VLYDENHFTADNLQVLTNNLCYTFARCTR**SVSIVPPAYYAH**LAAFRARYYIEGEMSDGGSTSGKSTTGR
SKEVQPLPVIKDNVKDVMFYC

Protein No.: I-290

Protein name and Species:

Fructose-bisphosphate aldolase OS=Mesembryanthemum crystallinum PE=2 SV=1

Accession: tr|O04975|O04975_MESCR

Unused ProtScore: 7.51

Seq Cov %: 21.9

ITRAQ Changed Ratio:

117: 115=E3TL: D3TL= 0.66

116: 114=E5TL: D5TL= 1.37

Annotated typical Precursor MS Region:

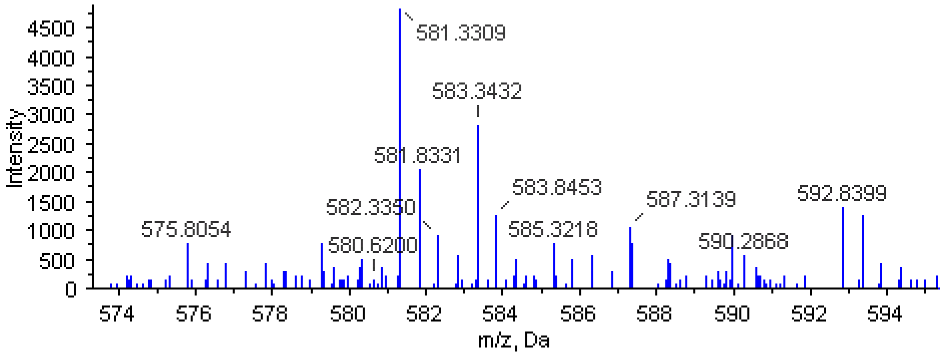

Sequence Coverage and Matched peptides: in different color

MTAYRGK**YADELIANAAYIGTPGKGILAADESTGTIGKR**FAGINVENVESNRRALRELLFTTPGAVQYL
SGVILFEETLYQKTAAGKPFVEVLKEGNVLPGIKVDKGVVELAGTNGETTTQGLDGLGARCAQYYAAGA
RFAKWRAVLKIGPTEPSPLAILENANGLARYAIICQENGLVPIVEPEILVDGPHDIDRCAEVTER**VLAA**
CYKALNDHHVLLEGTLLKPNMVTPGSESKKVAPEVIAEYTVRALQRTVPPAVPAVMFLSGGQSEEEATV
NLNAMNKLQGKKPWTLSFSYGRALQSSTLKAWSGKEENVEKAQAVFLARAKGNSEATLGKYQGGAGGAD
ASESLHVKDYKY

Protein No.: I-293

Protein name and Species:

Nuclear protein localization, putative OS=Ricinus communis GN=RCOM_0557110 PE=4 SV=1

Accession: **tr|B9S3Y6|B9S3Y6_RICCO**

Unused ProtScore: **7.44**

Seq Cov %: **18.2**

ITRAQ Changed Ratio:

117: 115=E3TL: D3TL= 0.11

116: 114=E5TL: D5TL= 0.68

Annotated typical Precursor MS Region:

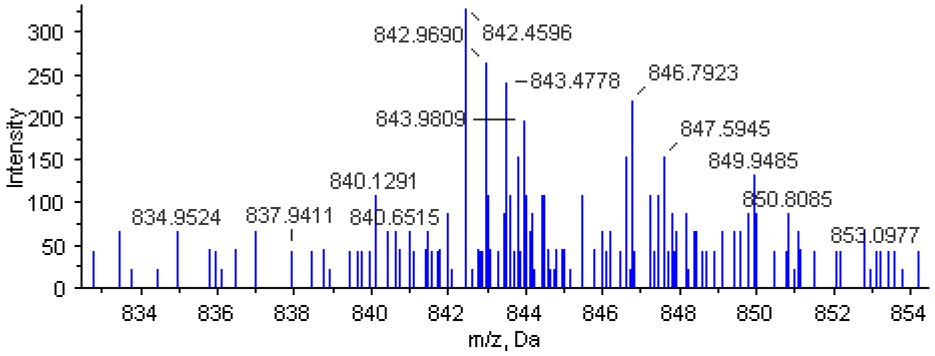

Sequence Coverage and Matched peptides: in different color

MMIRIRSRDGLER**VSVENPNITVSQLK**TLIQNQLNIPIQNQTLSTNQNLLLAKSPSDVLKFTDMSNPNT

PLSSLNITHGSILFLTYDGERTIAGPAVNPAGSFGRKMTMDDLIAKQMRITRQENPHCESVSFDRDCAN

AFQHIPMMVKLRFIIIYEPPQQGTEENLMLLRDPEEEKLVDAIAVGLGMRR**VGFIFTQTITQDK**KDYTL

SNR**EVLQAAELHAESELK**EWVTAIVKLEVNEDGGADVHFEAFQMSDMCIRLFKEDWFEKEIAEDSDPKL

SKMKKDVVVGGKDVR**EVDNDFFLVVVKILDHLGPLSSSFPIENR**INQVTKRALKSHLDRLKNLSFVKRI

SDFHLLLFLGRFLDVSDVPALAECVLTQTAVPEGYQLIIESVANS

Protein No.: I-297

Protein name and Species: Nucleic acid binding protein, putative OS=Ricinus communis GN=RCOM_1326600 PE=4 SV=1

Accession: tr|B9S228|B9S228_RICCO

Unused ProtScore: 7.32

Seq Cov %: 21.9

ITRAQ Changed Ratio:

117: 115=E3TL: D3TL= 0.50

116: 114=E5TL: D5TL= 0.64

Annotated typical Precursor MS Region:

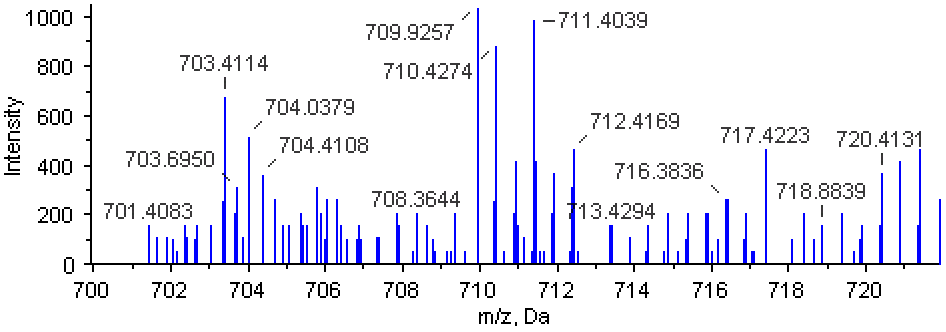

Sequence Coverage and Matched peptides: in different color

MAITTVK**VSNVSLGATEQDIK**EFFSFSGDIDYVELLGDNER**SQIAYVTFK**DQQGAETAVLLSGATIVDQ

SVTIELAPDYKLPAAASVPTTATESSTASGAESAFQK**AEDIVSSMLAKGFILGK**DAVNKAKAFDEKHQF

TSTATSKVASLDEKIGLTEK**ISAGTTLVNDK**VREVDEKFRVSETTK**SAFAAAEQTVSNAGSAIMK**NR**YV**

LTGASWVTGAFNRVAKAAGEVGQKTKEKVLAEEEQNQVAEGYTQIHGTDSPNMSEQMSKSSH

Protein No.: I-299

Protein name and Species:

Proteasome subunit beta type OS=Populus trichocarpa GN=POPTRDRAFT_814139 PE=3 SV=1

Accession: tr|B9HUT6|B9HUT6_POPTR

Unused ProtScore: 7.28

Seq Cov %: 28.7

ITRAQ Changed Ratio:

117: 115=E3TL: D3TL= 1.57

116: 114=E5TL: D5TL= 0.84

Annotated typical Precursor MS Region:

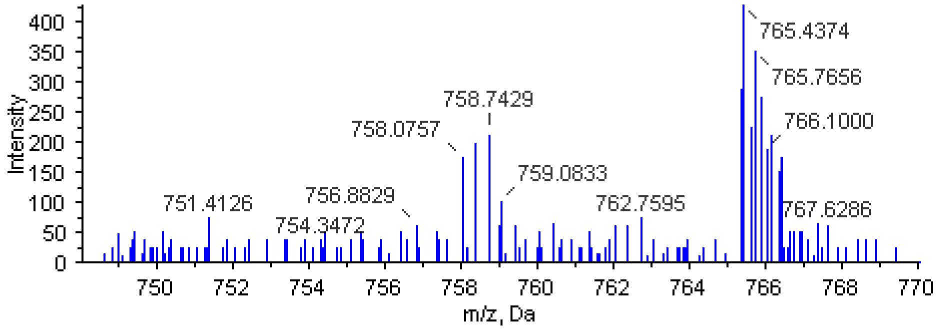

Sequence Coverage and Matched peptides: in different color

MECVFGLVGDGFVIVASDTSAVNSILVHKTNEDK**IMKLDSHK**LVAASGESGDR**VQFTEYIQKNVALYQF**

RNGIPLTTAAAANFTRGELATALRKNPYMVNILLAGYDK**ETGPSLYYIDYIATLHK**VDRGAFGYGSYFC

LSMMDRHYHSGMSVEEAVELVDKCITEIQSR**LVVAPPNFVIK**IVDR**DGAREYAWRESVK**DTPTAQPEAL

GV

Protein No.: I-306

Protein name and Species:

Ganglioside induced differentiation associated protein, putative OS=Populus trichocarpa GN=POPTRDRAFT_765804 PE=4 SV=1

Accession: tr|B9HIC5|B9HIC5_POPTR

Unused ProtScore: 7.1

Seq Cov %: 12.1

ITRAQ Changed Ratio:

117: 115=E3TL: D3TL= 2.78

116: 114=E5TL: D5TL= 1.59

Annotated typical Precursor MS Region:

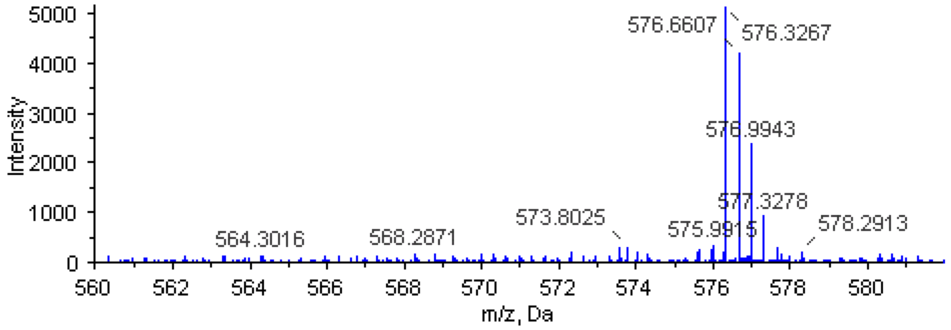

Sequence Coverage and Matched peptides: in different color

MYQRVPAAVTTR**GGSPTDNGDSVVTLDQVPR**WSDAESRSSFGYDNEDPSFTNPFFPDPLTSPSEGESSS
CGMVSR**FPVDHEINSK**IYLWRGNPWNLEVDAVVNSTNELLDEAHSSPGLHAAAGPGLAEECTTLGGCRT
GMAKVTNAYDLPARRVIHTVGPKYAMKYHTAAENALSHCYRSCLELLIENGLQSIAMGCIYTESKNYPR
EPAAHVAIRTVRRFLEKQKNKITAVVFCTTTSTDTEIYKRLLPLYFPREKHEEEVAIAK**LPADVGDENG**
ETIIDERKIRIKPLPKKNIPRPSQPLADLPVIDVGLVRRNSSYLDSYLDPAFMSVIKDPDQRRKEQWEK
TAQAQSGWNCAKIFGFGDLGGPTLSAAEEYSLHSRYLAK**ANSLNLSEIAEMK**IVYRGGVDSEGRPVMVV
VGAHFLLRCLDLERFVLHVVKEFEPLIQKPYTIVYFHSAASLQVQPDLGWIRRLQQILTRKHQRNLHAI
YVLHPNFHLKATIFALQV**FVDK**VTWKKVVYVDRLVQLFRYVPREQLTIPDFVFQHDLEVNGGKGLIVDP
RTKYVYHRP

Protein No.: I-310

Protein name and Species:

40S ribosomal protein S2, putative OS=Ricinus communis GN=RCOM_1577790 PE=3 SV=1

Accession: tr|B9RIC4|B9RIC4_RICCO

Unused ProtScore: 7.05

Seq Cov %: 18.2

ITRAQ Changed Ratio:

117: 115=E3TL: D3TL= 1.51

116: 114=E5TL: D5TL= 1.12

Annotated typical Precursor MS Region:

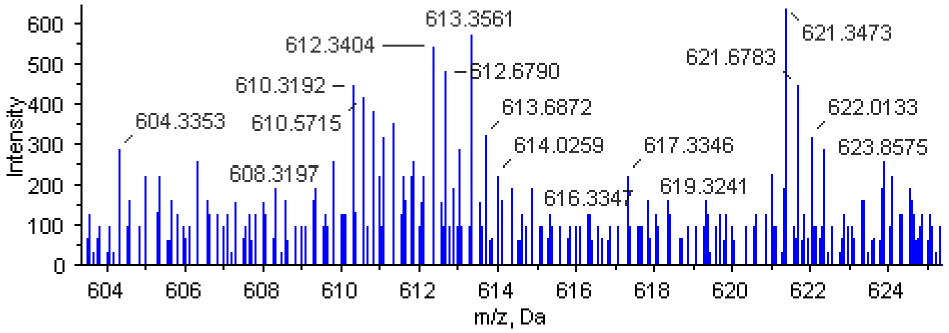

Sequence Coverage and Matched peptides: in different color

MSERPGGGERGAFRRGFGGAGRGDRGGRGRRRPRREEEEKWVPVTKLGRLVKEGKIR**SLEQIYLHSLPI**
KEHQIIDTLVGPSLKDEVMKITPVQKQTRAGQRTRFKAFVVVGDGNGHVGLGVKCSKEVATAIRGAIIL
AKLSVIPVRRGYWGNKIGKPHTVPCKVTGKCGSVTVRMVPAPRGAGIVAARVPKK**VLQFAGIEDVFTSS**
RGSTK**TLGNFVK**ATFDCLLKTYGFLTPDFWRETHFTKSPFQEYTDLLGKPTKVLIEDVERVDA

Protein No.: I-314

Protein name and Species:

DNA damage-inducible protein OS=Populus trichocarpa GN=POPTRDRAFT_744891 PE=2 SV=1

Accession: tr|A9PEP6|A9PEP6_POPTR

Unused ProtScore: 6.98

Seq Cov %: 13.9

ITRAQ Changed Ratio:

117: 115=E3TL: D3TL= 0.47

116: 114=E5TL: D5TL= 0.52

Annotated typical Precursor MS Region:

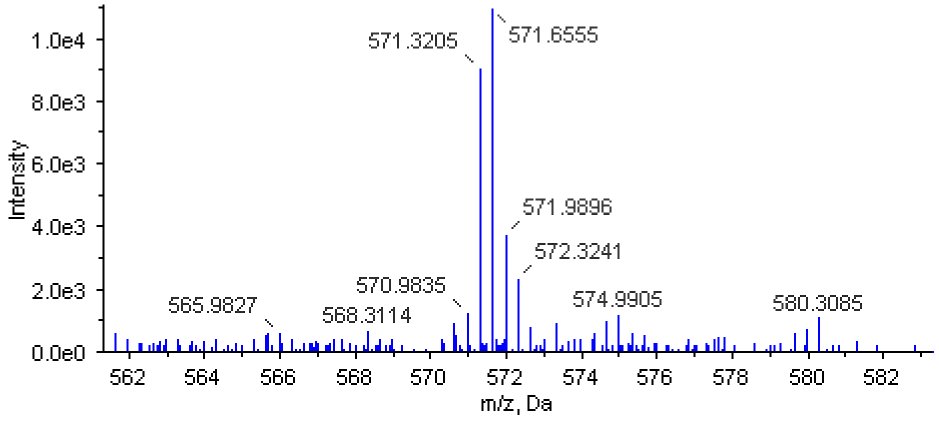

Sequence Coverage and Matched peptides: in different color

MRITVMTADEQIISLEVDPHESVENVK**ALLEVETQVPLQQQQLLYNGR**EMRNNEK**LSALGVK**DEDLVMM
VSNAAALSAPSNNLGLNPDGSAMNPGAFQQQLRNDSNTMAQLFQADPELAQVILGNDLNK**LQDLLR**QRH
RQRSELHRQQEEEFALLEADPFDVEAQKKIEAAIRQK**GIDENWAAALEYNPEAFAR**VIMLYVDMEVNGV
PLK**AFVDSGAQSTIISK**SCAERCGLLRLLDQRYKGIAHGVGQSEILGR**IHVAPIK**IGNIFYPCSFMVLD
SPNMEFLFGLDMLRKHQCIIDLKENVLR**VGGGEVSVPFLQEK**DIPPRFLDEERYSKEASSSGNPVTSGK
AEKKNDPPAMGQSSGVARSSVTQGPDFEAKVAK**LVELGFGR**EAVIQALKLFDGNEEQAAGFLFGG

Protein No.: I-315

Protein name and Species:

Putative uncharacterized protein OS=Vitis vinifera GN=VIT_18s0001g08370 PE=3 SV=1

Accession: tr|F6H158|F6H158_VITVI

Unused ProtScore: 6.98

Seq Cov %: 5

ITRAQ Changed Ratio:

117: 115=E3TL: D3TL= 0.44

116: 114=E5TL: D5TL= 0.60

Annotated typical Precursor MS Region:

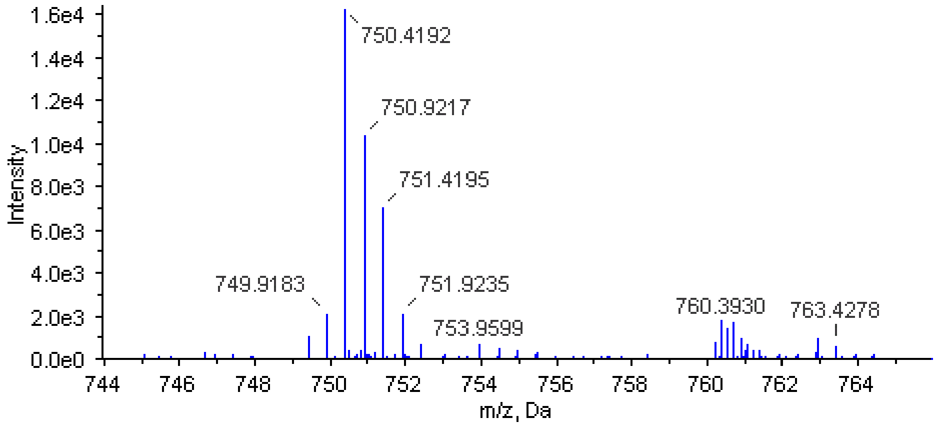

Sequence Coverage and Matched peptides: in different color

MRRIWPCCFTRRVSNTHQKAEDIQRSIGTMENPKEGNADHGVSEKQNESTIKSRDSLSSNIILNHDFSR
GLHSWNLNCCNGSVVSAESGFLEGISVKSGGNYAVITNRKECWQGLEQDITSRVSLGSTYSVSACVGVS
GSLQGSAVVQATLKLEYQGSATSYLFIGRTSVSREQWKK**LEGTFSLSTMPDR**VVFYLEGPSPGLDLLIE
SVVIFCSSPTEEESSSTRCAAAGDENIILNPIFEDGVNNWSGRGCKILLHDSMGGGKIVPQSGKFFASA
TERTQSWNGIQQEITGRVQR**KLAYEVAAVVR**IFGNNVTSADVRVTLWVQTPNLREQYIGVANSQATDKD
WIQLQGKFLLNASPSRVVIYLEGPPPGTDILVNSLVVKHAEKIPPSPPPVIEDPAFGINTIQNSNLNDG
SNGWFPLGSCTLSVATGSPRILPPMARDSLGAHNPLSGHYILVTNRTQTWMGPAQMITDRVKLYLTYQV
SAWVRIGPGATAPQNVNVALGVDSQWVNGGQANVSDDRWYEIGGSFRIEKQPLKVMVYVQGPASGVDLM
VAGLQIFPVDRHARFRHLKKETDKIRKRDVILNFSGSGTGTSIGTFVKVRQTQNSFGFGSCVSRTNIDN
EDFVDFFVK**NFNWAVFGNELK**WYWTESQQGNFNYRDADELLDLCKSHNMETRGHCIFWEVEGTVQPWVK
SLNKNDLMTAVQNRLTGLLTRYKGKFRHYDVNNEMLHGSFYQDRLGKDIRANMFKTANQLDSSAALFVN
DYHVEDGCDTRSSPEKYIEQVIDLQKQGAPVGGIGIQGHIDSPVGPIVCSALDKLGVLGLPIWFTELDV
SSINECIRADDLEVMLREAFAHPAVDGIMLWGFWELFMSRNNAHLVNAEGEINETGWRYLALRKEWLSH
AHGHIDEQGEFMFRGFHGSYVVEIGTGSKKISKTFVVDNGESPLVVSIGL

Protein No.: I-321

Protein name and Species:

Ubiquitin-protein ligase, putative OS=Ricinus communis GN=RCOM_1154930 PE=4 SV=1

Accession: tr|B9SG36|B9SG36_RICCO

Unused ProtScore: 6.86

Seq Cov %: 6.5

ITRAQ Changed Ratio:

117: 115=E3TL: D3TL= 1.80

116: 114=E5TL: D5TL= 1.08

Annotated typical Precursor MS Region:

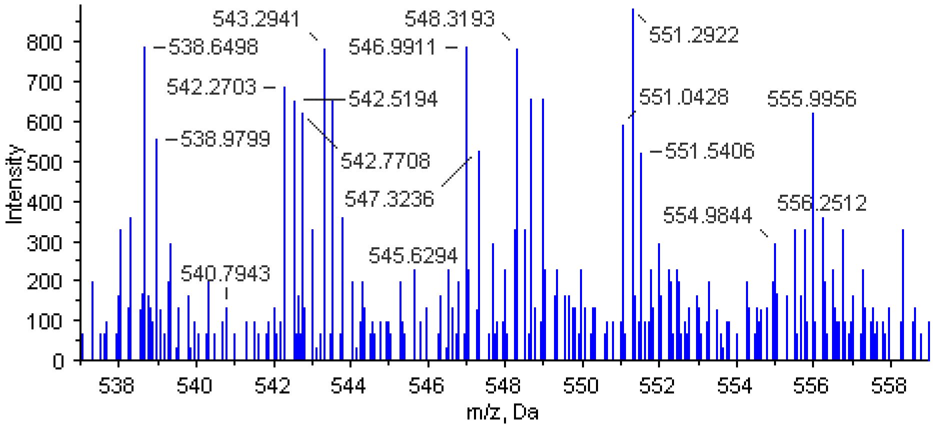

Sequence Coverage and Matched peptides: in different color

MSVMGYNLAGDVTSANLFHKESSVMEISLLKALLSNISSFLHLSSIDNICSDLVQKCYQK**AEEILK**LLK
PILDAIVDSEIASDEVLTKAFDELGQSVDELRELFENWQPLSSKVFFVLQIESLISKIRTLGLDIFQLL
KSSHEHLPDELSTSSLEEILIEAVALEKLKENAEQAEKPREAELFDQMISLSQTCSHVPIPADFCCPLS
LELMTDPVIVGSGQTYERAFIKNWIELGLTVCPKTRQTLAHTNLIPNYTVKALIANWCESNNVKLPDPV
KSVSFNQPSALLIHAESGTPRGSHGFYSRGNQPMSPESTRSTDSPDRNWISSSVHRESTSPCHPRSTSD
SSLSGIVGNEQGLDMARISLASSEERSVNLEGRNRDSGVRHSVSPSRNEVSNAVRVGEPIAQSHSRNAS
ASSINASFSQGAQVDTNDSSEVLNHLTSYSSDNSGEVKAETQASTALNSPRPHREPEFAPRFIETRSRS
QTIWRRPSDRLIPRIVSSPAIETRADLSGVETQVRNLVEDLKSDSIDVQRAATAELRLLAKHNMDNRIV
IANCGAINILVNLLRSADAKIQENAVTALLNLSINDNNKTAIANADAIGPLIHVLETGSPEAKENSAAT
LFSLSVIEDNKVRIGRSGAVGPLVDLLGNGTPRGKKDAATALFNLSIFHENKARIVQAGAVK**HLVELMD**
PAAGMVDKAVAVLANLATIPEGRTAIGQEGGIPVLVEVVELGSARGKENAAAALLQLCTNSNRFCNTVL
QEGAVPPLVALSQSGTPRAKEKAQALLSFFRNQRHGNARG

Protein No.: I-322

Protein name and Species:

alanine aminotransferase OS=Populus trichocarpa GN=POPTRDRAFT_1073568 PE=4 SV=1

Accession: tr|B9GXZ6|B9GXZ6_POPTR

Unused ProtScore: 6.86

Seq Cov %: 20.2

ITRAQ Changed Ratio:

117: 115=E3TL: D3TL= 0.44

116: 114=E5TL: D5TL= 2.25

Annotated typical Precursor MS Region:

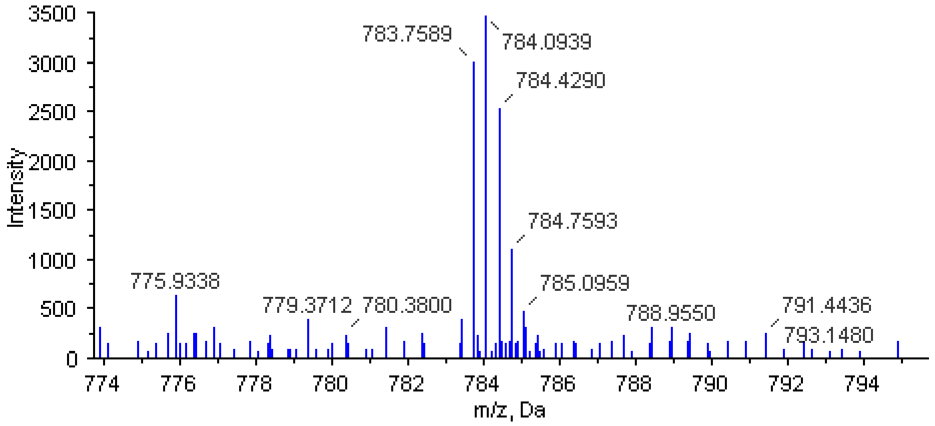

Sequence Coverage and Matched peptides: in different color

MPPVSLDNINPKVLKCEYAVRGEIVTLAQALQEELKSKPGSHPFDEILYCNIGNPQSLGQQPITFFREV
LALCDHPSILDR**SETQGLFSADAIER**AWQILDQIPGR**ATGAYSHSQGIK**GLRDTIAAGIEARDGFPADP
NDIFLTDGASPAVHMMMQLLIRSEKDGILCPIPQYPLYSASIALHGGALVPYYLDEATGWGLEVSELKK
QLADAKSKGITPR**ALVVINPGNPTGQVLAEDNQR**GIVEFCK**QEGLVLLADEVYQENVYVPEK**KFDSFKK
VARSMGYGEKDISLVSFQSVSKGYYGECGKRGGYMEVTGFGPEIREQIYKVASVNLCSNISGQILASLV
MSPPK**VGDESYESYSAEK**DGILSSLARRAKTLEDAFNNLEAVTCNKAEGAMYLFPCICLPEKAIKAAEA
AKTAPDNFYCRRLLNATGIVFVPGSGFGQLPGTWHFR**CTILPQEDKIPAVVSR**LTEFHKSFMDEFRD

Protein No.: I-324

Protein name and Species:

ATP-citrate synthase alpha chain OS=Vitis vinifera GN=VITISV_017316 PE=4 SV=1

Accession: tr|A5AQP2|A5AQP2_VITVI

Unused ProtScore: 6.85

Seq Cov %: 19.6

ITRAQ Changed Ratio:

117: 115=E3TL: D3TL= 0.51

116: 114=E5TL: D5TL= 0.71

Annotated typical Precursor MS Region:

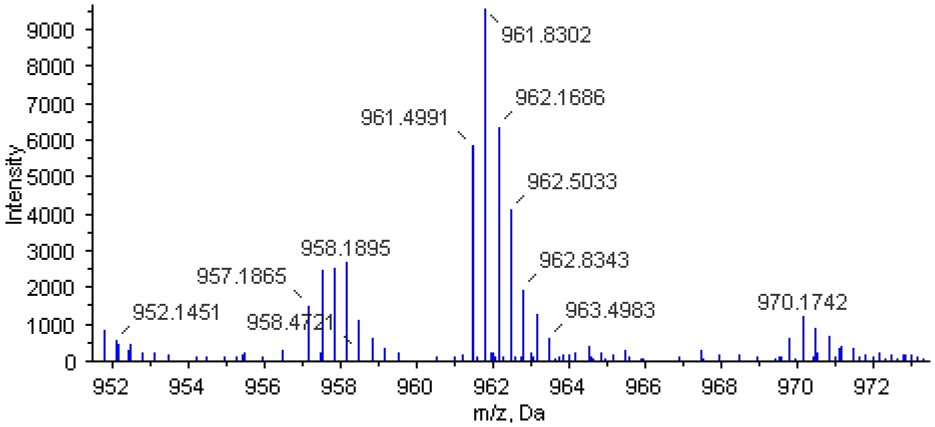

Sequence Coverage and Matched peptides: in different color

MARKKIREYDSKRLLKEHLKRLVGIDLQICSAQVTEATDFAELTNKEPWLSSTRLVVKPDMLFGKRGKS
GLVALNLDLAQVAEFVKARLGVEVEMGGCKAPITTFIVEPFVPHDQEYYLSIVSDRLGCTISFSECGGI
EIEENWDKVKTINLPTEKPMTPETCAPLIATLPLEVRGKIGDFIKGVFAVFQDLDFSFLEMNPFTLVNG
EPYPLDMRGELDDTAAFKNFKTWGNIEFPLPFGRVLSPTEGYIHLLDEKTSASLKFTVLNPKGRIWTMV
AGGGASVIYADTVGDLGYASELGNYAEYSGAPNEEEVLQYARVVLDCATADPDGRKRALLIGGGIANFT
DVAATFNGIIRALKEKESKLKASRMHIYVRRGGPNYQTGLARMRALGEELGIPLEVYGPEATMTGICKQ
AIDCIMSTA

Protein No.: I-327

Protein name and Species:

Heat shock protein 70 OS=Hevea brasiliensis GN=HSP70 PE=2 SV=1

Accession: tr|B2MW33|B2MW33_HEVBR

Unused ProtScore: 6.81

Seq Cov %: 33.3

ITRAQ Changed Ratio:

117: 115=E3TL: D3TL= 0.65

116: 114=E5TL: D5TL=0.70

Annotated typical Precursor MS Region:

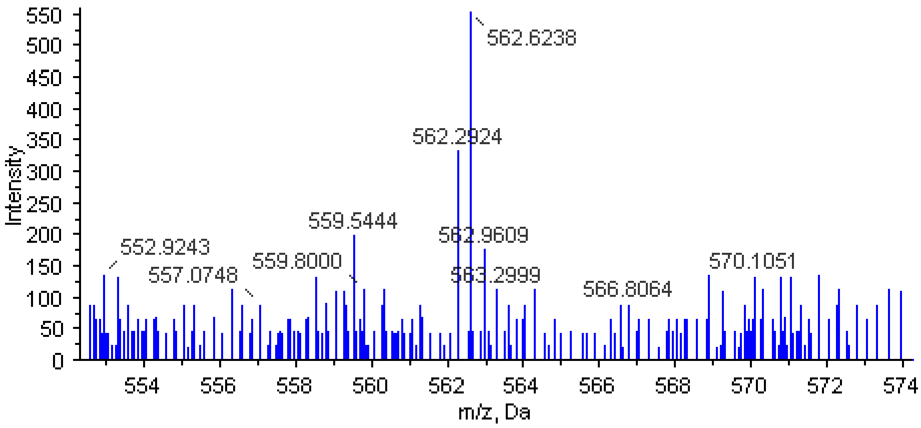

Sequence Coverage and Matched peptides: in different color

MAPKTEGKAIGIDLGTTYSCVGVWQNDRVEIIANDQGNRTTPSYVAFTDTERLIGDAAKNQVAMNPQNT
VFDAKRLIGRRFSDPSVQSDMKHWPFKVISGPGDKPMIVVQYKGEEKQFAPEEISSMVLTKMKEIAEAY
LGQTVKNAVITVPAYFNDSQRQATKDAGAIAGVNVMRIINEPTAAAIAYGLDKKGSSTGEKNVLIFDLG
GGTFDVSLLTIEEGIFEVKATAGDTHLGGEDFDNRLVNHFVAEFKGKHKKDISTNARALRRLRTARERA
KRTLSSTSQTTIEIDSLYEGIDFYATITRARFEELNMDLFRKCMEPVEKCLRDSKIDKSQVHDVVLVGG
STRIPKVQQLLQDIFNGKELCKSINPDEAVAYGAAVQAAILSGEGDEKVQDLLLLDVTPLSLGIETAGG
VMTVLIPRNTTIPTKKEQIFSTYSDNQPGVLIQVYEGERARTKDNNLLGKFELTGIPPAPRGVPQINVC
FDIDANGIVNVSAEDKTAGVKNKITITNDKGRLSKEEIERMVQEAEKYKAEDEEVKKKVEAKNSLENYA
YNMRNTVKDDKFAGKLAPADKGKIEKAIDETIEWLDRNQLAEVEEFEDKLKELEGLCNPIIAKMYQGGA
GGDVPVGGAEMPNSNYGKTSSGGSGAGPKIEEVD

Protein No.: I-328

Protein name and Species:

Esterase D, putative OS=Ricinus communis GN=RCOM_0893450 PE=4 SV=1

Accession: tr|B9SCD6|B9SCD6_RICCO

Unused ProtScore: 6.77

Seq Cov %: 17.7

ITRAQ Changed Ratio:

117: 115=E3TL: D3TL= 0.32

116: 114=E5TL: D5TL= 2.32

Annotated typical Precursor MS Region:

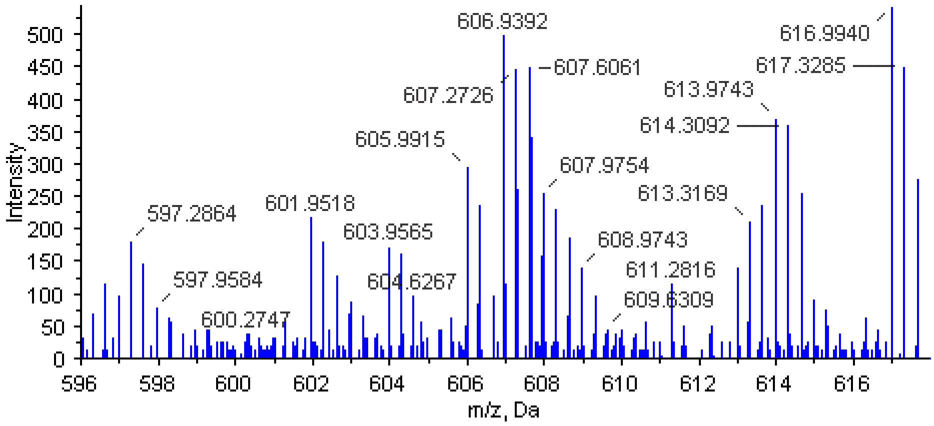

Sequence Coverage and Matched peptides: in different color

MKSVAQRCLEDTIKDTDISILYFLSGLTCTDENFIIKSGAQR**AASSEGVALIAPDTSPR**GLNVEGEADS
WDFGVGAGFYLNATQEKWKNWRMYDYVVKELPK**LLSDNFPQLDTSR**ASLSGHSMGGHGALTIYLKNLDK
YKSVSAFAPISNPINCPWGQKAFTNYLGSSKGDWEEYDATSLVSKFHDVSATILIDQGEDDKFLHDKQL
LPQK**FEEACRSANVPLLLR**MQPGYDHSYFFISTFIDDHIRHHAQALNL

Protein No.: I-329

Protein name and Species:

Predicted protein OS=Populus trichocarpa GN=POPTRDRAFT_647799 PE=4 SV=1

Accession: tr|B9H2B8|B9H2B8_POPTR

Unused ProtScore: 6.77

Seq Cov %: 30.3

ITRAQ Changed Ratio:

117: 115=E3TL: D3TL= 0.23

116: 114=E5TL: D5TL= 0.75

Annotated typical Precursor MS Region:

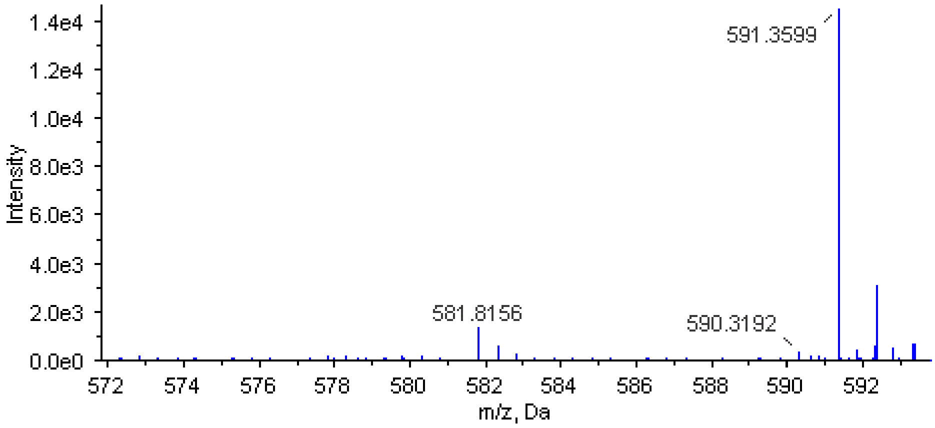

Sequence Coverage and Matched peptides: in different color

MTSQHIATHREKAEIHTGESLCKQKSLELLEEIH**LPMGLLPLDDIVEVGYNR**TTGFVWLKQKKRKDHRF
LKIGRQVSYDT**EVTAFVENR**RMRRLTGVKTKELLFWVSISDIYVDEKDLE**KITFGNPTGISRTFPVSAF**
ELEEEKK

Protein No.: I-335

Protein name and Species:

L-asparaginase, putative OS=Ricinus communis GN=RCOM_1467530 PE=4 SV=1

Accession: **tr|B9RLJ0|B9RLJ0_RICCO**

Unused ProtScore: **6.7**

Seq Cov %: **24.3**

ITRAQ Changed Ratio:

117: 115=E3TL: D3TL= 0.37

116: 114=E5TL: D5TL= 0.53

Annotated typical Precursor MS Region:

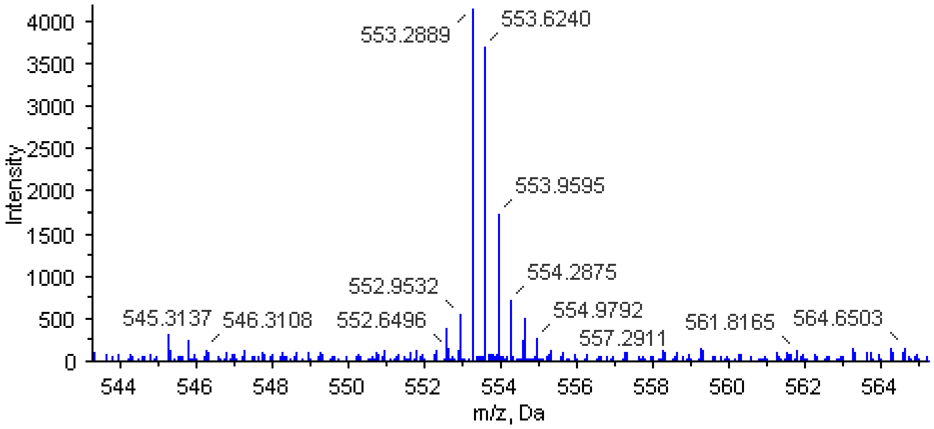

Sequence Coverage and Matched peptides: in different color

MGWAIALHGGAGDIPLNLPPERRLPREAALRHCLEIGVAALQAQK**HPLDVVELVVRELENHPNFNAGK**G
SVLTTEGTVEMEACIMDGKTKKCGAVSGLTTVVNAISLARLVMEKTPHIYLAFDGAEAFAR**EQGVETVD**
SSQFVTPENVERLKQAKEANR**VQIDYTQPIQK**NEETQNPNADGDSQIG**TVGCVAVDTNGNLASATSTGG**
LVNKMVGR**IGDTPIIGAGTYANNLCAVSATGK**GEFIIRGTVARDVAALMEYKGLSLMEAAAYVVEQCVP
RATVGLVAVSATGEVTMPFNTTGMFRACATEDGYSEIAIWPPVQD

Protein No.: I-338

Protein name and Species:

Chorismate synthase OS=Populus trichocarpa GN=CS1 PE=3 SV=1

Accession: tr|B9HLM0|B9HLM0_POPTR

Unused ProtScore: 6.66

Seq Cov %: 12.1

ITRAQ Changed Ratio:

117: 115=E3TL: D3TL= 0.61

116: 114=E5TL: D5TL= 1.26

Annotated typical Precursor MS Region:

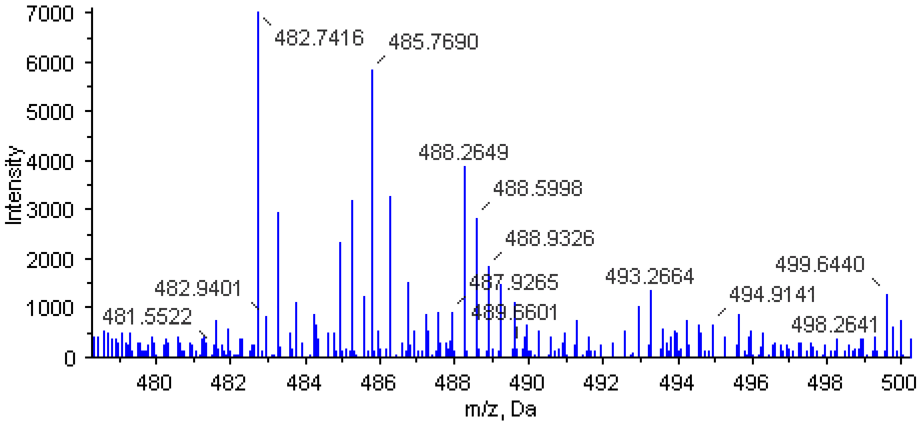

Sequence Coverage and Matched peptides: in different color

MASSTLTSKSFLDSSRIDTASLSSAHLRKLSFSSVQISSRSRLPKKLQINAAGSTFGNNFR**VTTFGESH**
GGGVGCIIDGCPPRIPLSESDMQFDLDRRRPGQSRITTPRKETDTCKISSGVSEGQTTGTPIHVFVPNT
DQRGFDYNEMSVAYRPSHADATYDMKYGVRSVQGGGRSSARETIGRVAAGAVAKKILKLYAGTEILAYV
SQVHKVVLPEGVVNHDSLSLDQIESNIVRCPDPEYAEKMIAAIDAVRVK**GDSVGGVVTCIVR**NAPRGLG
SPVFDK**LEAELAK**AAMSLPATKGFEFGSGFSGTFLSGSEHNDEFYTDEHGRIRTRTNRSGGIQGGISNG
EIINLR**IAFKPTSTIGR**KQHTVTRDKKEIELIARGRHDPCVVPRAVPMVEAMVALVLMDQLMAQFSQSY
LFPINPDLQEPLTMPRLEAANASV

Protein No.: I-343

Protein name and Species:

40S ribosomal protein S11, putative OS=Ricinus communis GN=RCOM_0658740 PE=3 SV=1

Accession: **tr|B9SKG4|B9SKG4_RICCO**

Unused ProtScore: **6.6**

Seq Cov %: **17.6**

ITRAQ Changed Ratio:

117: 115=E3TL: D3TL= 2.09

116: 114=E5TL: D5TL= 0.82

Annotated typical Precursor MS Region:

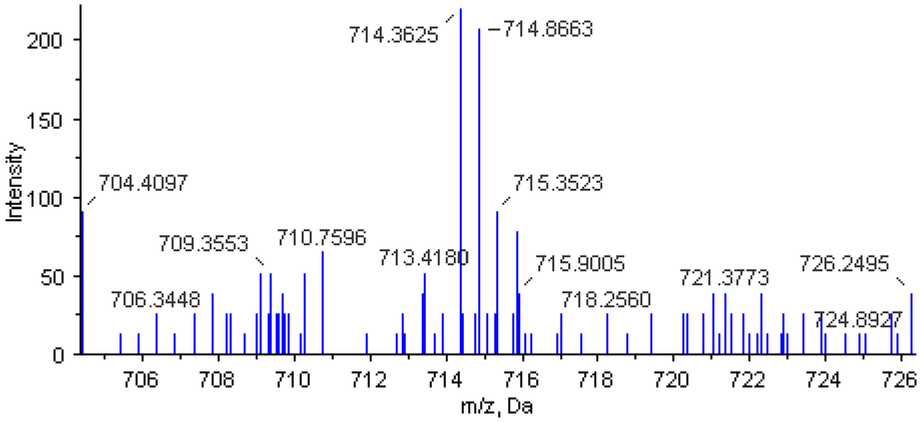

Sequence Coverage and Matched peptides: in different color

MAEQTEKAFLKQPKVFLSSKKTGKGKRPGKGGNRFWKSIGLGFKTPREAIEGTYIDKKCPFTGTVSIRG
RILAGTCHSAKMNRTIIVRRNYLHFIKKYQRYEKRHSNIPAHISPCFRVKEGDHVIIGQCRPLSKTVRF
NVLKVIPAGSSGGAKKAFTAM

Protein No.: I-345

Protein name and Species:

JHL07K02.17 protein OS=Jatropha curcas GN=JHL07K02.17 PE=4 SV=1

Accession: **tr|E6NU17|E6NU17_9ROSI**

Unused ProtScore: **6.54**

Seq Cov %: **6.8**

ITRAQ Changed Ratio:

117: 115=E3TL: D3TL= 0.33

116: 114=E5TL: D5TL= 1.27

Annotated typical Precursor MS Region:

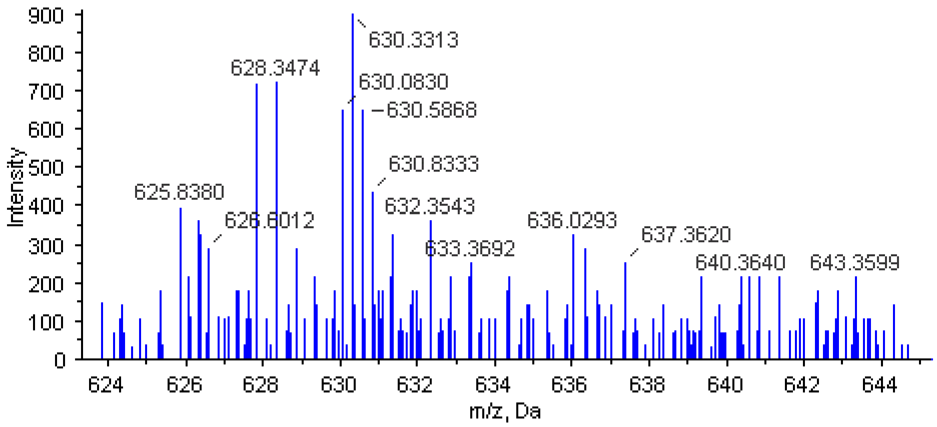

Sequence Coverage and Matched peptides: in different color

MGNCCSDEAGGRAAVGGTGTASAGHTTTANDAIDYYLKSRGFRGMFFQIELSFSATNLRDRDVLSK**SDP**
MVVVYTKGRDEPPTEVFRTEVVLNSLNPTWITKYSITFQFEVVQTLQFHVYDVDTQFHNVDVKMLKLDE
QQFLGEASCVLSEIVTRPNRTLTLDLVHKDSTISSQPRCGQLVVHAEECISSKTTTQMTLRCSDLEHK**D**
LFSRINPFLVISKTVESGMPIPVCKTEVLKNDHSPVWKPVFLNIQQVGSKDSPLVIECYNFNSSGK**HDL**
IGEVRKSLADLEKLCSTGAGQNLFLQTAVGHDHSKALKSQLFVDNFSESIQYTFLDYLKGGCELNFMVA
IDFTASNGNPR**LPDSLHYIDPSGR**PNAYQQAILEVGEVLQFYDSDKRFPAWGFGARPIDGPVSHCFNLS
GSTNHCEVEGIQGIMTSYTSALFNVSLAGPTLFGPVISNAAHIAGQSIANGGKKYFVLLIITDGVVTDL
QETKDAIVKASDLPLSILIVGVGGADFKEMEILDADKGERLESSTGRVASR**DIVQFVPFR**SIQSQQISV
VQALLAELPTQFLTYMRSRNIQPNI

Protein No.: I-348

Protein name and Species:

Hydrolase, hydrolyzing O-glycosyl compounds, putative OS=Ricinus communis GN=RCOM_0750500 PE=4 SV=1

Accession: tr|B9SUE9|B9SUE9_RICCO

Unused ProtScore: 6.52

Seq Cov %: 7

ITRAQ Changed Ratio:

117: 115=E3TL: D3TL= 31.33

116: 114=E5TL: D5TL= 2.51

Annotated typical Precursor MS Region:

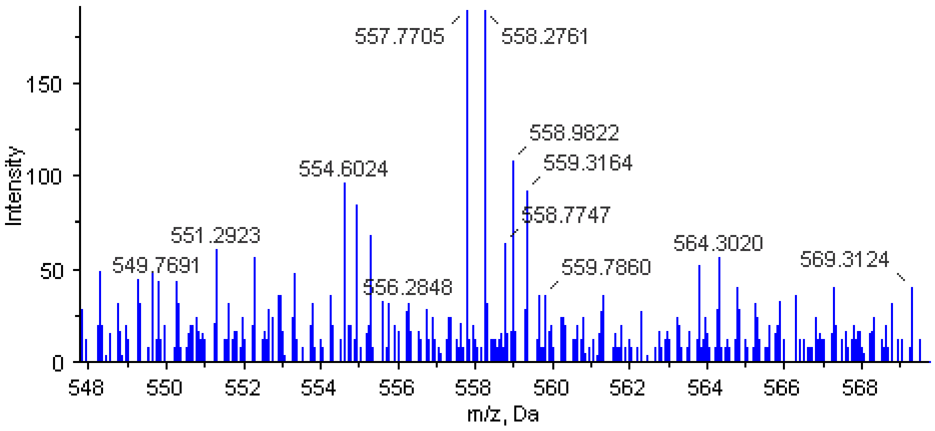

Sequence Coverage and Matched peptides: in different color

MTVTPN**ISINDGNLVVHGK**TILTRVPDNIVLTPGSGVGLLAGAFIGASAADSKSLHVFPVGVLEGLRFM
CCFRFKLWWMTQRMGTCGKDIPLETQFMLVESKDGGEGVDQDDAQTIYTVFLPLLEGQFRAVLQGNEKS
EMEICLESGDTAVETNQGHYLVYMHAGTNPFEVINQAVKAVEKHMQTFLHREKKKVTISFFFFYIKNFY
HKHCEAESASISSFVLLVLCSCPLFLTGLAGLKHVVEDAKKHHNVKYVYAWHALAGYWGGVKPAAAGME
HYDTALAYPMSSPGVKGNQPDIVMDSLSVHGLGLVHPKKVFNFYNELHAYLASCGIDGVK**VDVQNIIET**
LGAGHGGRVSLTR**SYHQALEASIAR**NFPDNGCIACMCHNTDGLYSAKQTAVVR**ASDDFYPR**DPASHTIH
ISSVVYNSLFLGEFMQPDWDMFHSLHPAADYHGAARAIGGCPIYVSDKPGNHNFELLKKLVLPDGSVLR
AQLPGRPTRDCLFADPARDGTSLLKVWNMNKCTGVVGVFNCQGAGWCKIEKKTR**IHDASPGTLTASVR**A
SDVDCIAQVANADWNGETVVYAHRSADVIRLPKGASLPVTLKVLEYELFHFCPIKEIISNISFAPIGLL
DMFNSSGAVDQVEIRTASDGKPELFDGEVSSELTTSLSENRSPTATIEMRRPLKCLVGGAETDFNYDSD
SGLLTLTLPVPEEDMYRWPVEIHV

Protein No.: I-349

Protein name and Species:

60S ribosomal protein L12, putative OS=Ricinus communis GN=RCOM_0153900 PE=3 SV=1

Accession: tr|B9SWB0|B9SWB0_RICCO

Unused ProtScore: 6.51

Seq Cov %: 31.9

ITRAQ Changed Ratio:

117: 115=E3TL: D3TL= 2.42

116: 114=E5TL: D5TL= 1.28

Annotated typical Precursor MS Region:

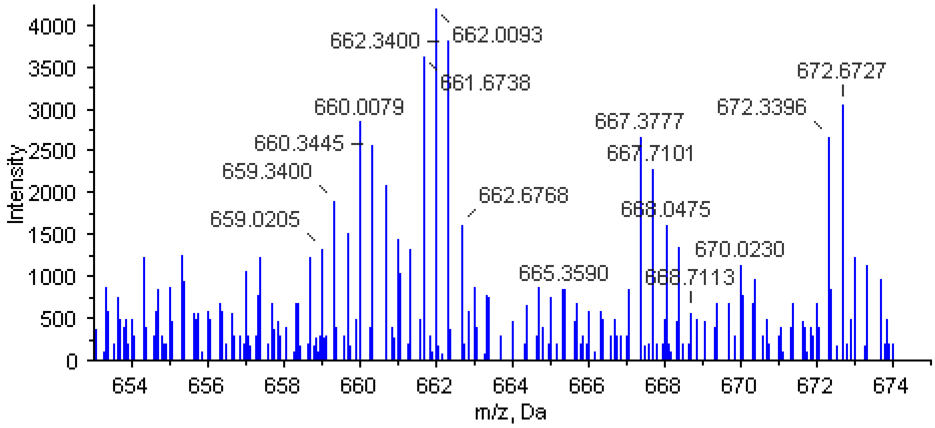

Sequence Coverage and Matched peptides: in different color

MPPKFDPSQVVDVFVR**VTGGEVGAASSLAPKIGPLGLSPK**KIGEDIAKETAKDWKGLRVTVKLTVQNRQ
AK**VTVVPSAAALVIK**ALKEPERDRKKTKNIKHSGNISLDDVIEIAKVMKPRSMAKDLSGTVK**EILGTCV**
SVGCTVDGKDPKDLQQEITDGDVEVPLD

Protein No.: I-350

Protein name and Species:

developmentally-regulated GTP-binding protein OS=Populus trichocarpa GN=POPTRDRAFT_718912 PE=4 SV=1

Accession: tr|B9HG22|B9HG22_POPTR

Unused ProtScore: 6.49

Seq Cov %: 14.7

ITRAQ Changed Ratio:

117: 115=E3TL: D3TL= 0.48

116: 114=E5TL: D5TL= 0.84

Annotated typical Precursor MS Region:

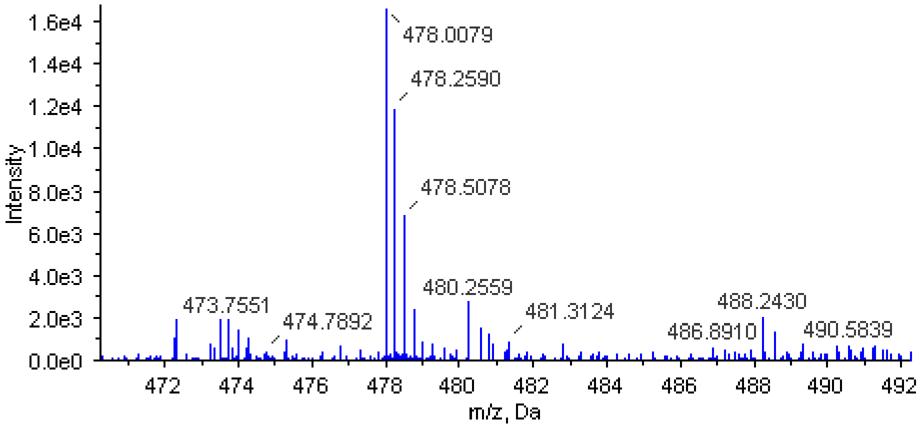

Sequence Coverage and Matched peptides: in different color

MSTVMQKIKDIEDEMARTQKNKATAHHLGLLKAKLAKLRRELLEPSAK**GGGGAGQGFDVTK**SGDAR**VGL**
VGFPSVGKSTLLNKLTGTFSEVASYEFTTLTCIPGVIVYKGAKIQLLDLPGIIEGAKDGKGRGRQVIST
ARTCNCILIVLDAIKPITHKRLIEKELEGFGIRLNKEPPNLTFRKKDKGGINFTSTVANTHLDLDTVK**A**
ICSEYRMHNADITLR**YDATADDLIDVIEGSR**IYMPCIYVVNK**IDQITLEELEILDK**LPHYCPVSAHLEW
NLDGLLDKVWEYLNLTRIYTKPKGMNPDYEDPVILSSKKRTVEDFCERIHKDMLKQFKYALVWGSSAKH
KPQRVGKEHELEDEDVVQIIKKV

Protein No.: I-351

Protein name and Species:

Cis-prenyl transferase OS=Hevea brasiliensis GN=HbCPT PE=2 SV=1

Accession: **tr|A9ZN23|A9ZN23_HEVBR**

Unused ProtScore: **6.48**

Seq Cov %: **37.8**

ITRAQ Changed Ratio:

117: 115=E3TL: D3TL= 0.39

116: 114=E5TL: D5TL= 0.72

Annotated typical Precursor MS Region:

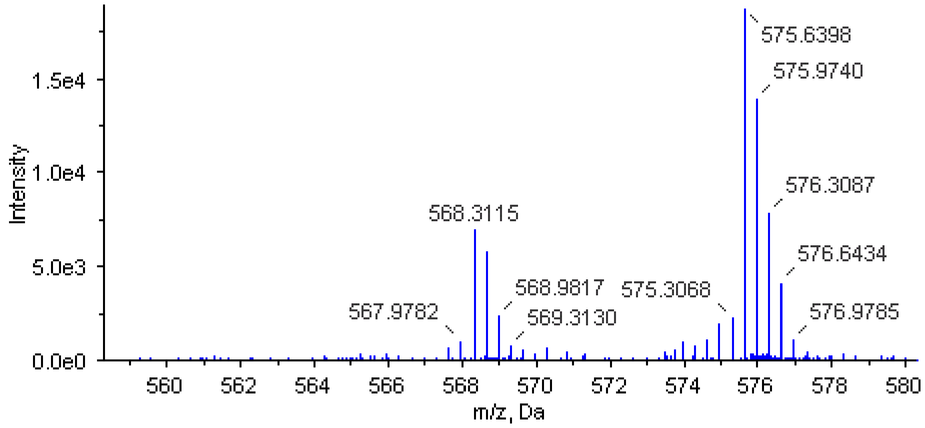

Sequence Coverage and Matched peptides: in different color

MELYNGERPSVFRLLGKYMRKGLYGILTQGPIPTHLAFILDGNRRFAKKHKLPEGGGHKAGFLALLNVL
TYCYELGVKYATIYAFSIDNFRRKPHEVQYVMDLMLEKIEGMIMEESIINAYDICVRFVGNLKLLSEPV
KTAADQSMRATANNSRFVLLIAVAYSSTDEIVHAVEESSKDKLNSNEVCNNGIEAEQEFKEANGTGNSV
IPVQKTESYSGIKLVDLEKNTYINPYPDVLIRTSGETRLSNYLLWQTTNCILYSPYALWPEIGLRHVVW
SVINFQRHYSYLEKHKEYLK

Protein No.: I-355

Protein name and Species:

40S ribosomal protein S14, putative OS=Ricinus communis GN=RCOM_0632900 PE=3 SV=1

Accession: tr|B9S120|B9S120_RICCO

Unused ProtScore: 6.46

Seq Cov %: 22.7

ITRAQ Changed Ratio:

117: 115=E3TL: D3TL= 2.27

116: 114=E5TL: D5TL= 0.86

Annotated typical Precursor MS Region:

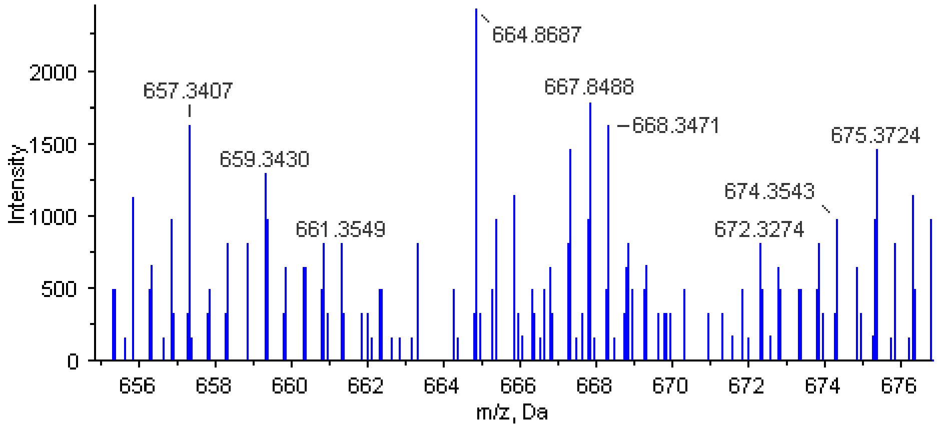

Sequence Coverage and Matched peptides: in different color

MSKKKTREPKEENVTLGPAVREGEHVFGVAHIFASFNDTFIHVTDLSGRETLVRITGGMKVKADRDESS
PYAAMLAAQDVTQRCKELGITALHIKLRATGGNKTKTPGPGAQSALRALARSGMRIGRIEDVTPIPTDS
TRRKGGRRGRRL

Protein No.: I-357

Protein name and Species:

Rubber elongation factor OS=Hevea brasiliensis GN=REF PE=2 SV=1

Accession: **tr|Q6T708|Q6T708_HEVBR**

Unused ProtScore: **6.44**

Seq Cov %: **64**

ITRAQ Changed Ratio:

117: 115=E3TL: D3TL= 0.51

116: 114=E5TL: D5TL= 1.17

Annotated typical Precursor MS Region:

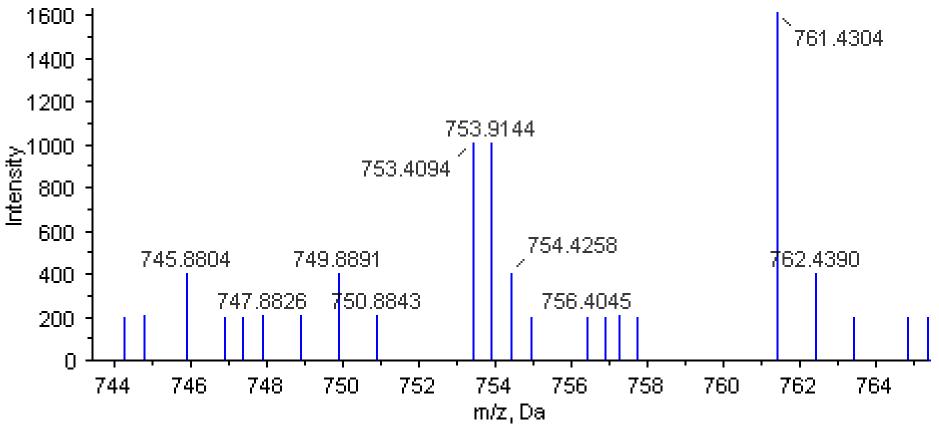

Sequence Coverage and Matched peptides: in different color

MAEGEEEVNIQEEANK**GEENPQEEANIQEETNKGEENIQEEANIEEEANKEEESLKYLDFVQAATVYAR**
ASFSKLYLFAKDK**SGPFKPGVNTVESR**FK**SVVRPVYNK**FQPVPNKVLKFADR**RVDAYVTVLDR**IVPPIV
KR**ASIQAYSVAPGAALAVASYLPLHTK**RLSKVLYGDG

Protein No.: I-364

Protein name and Species:

chitinase，Class I (Fragment) OS=Gossypium hirsutum PE=2 SV=1

Accession: tr|Q9ZTT8|Q9ZTT8_GOSHI

Unused ProtScore: 6.34

Seq Cov %: 13.9

ITRAQ Changed Ratio:

117: 115=E3TL: D3TL=0.60

116: 114=E5TL: D5TL=0.62

Annotated typical Precursor MS Region:

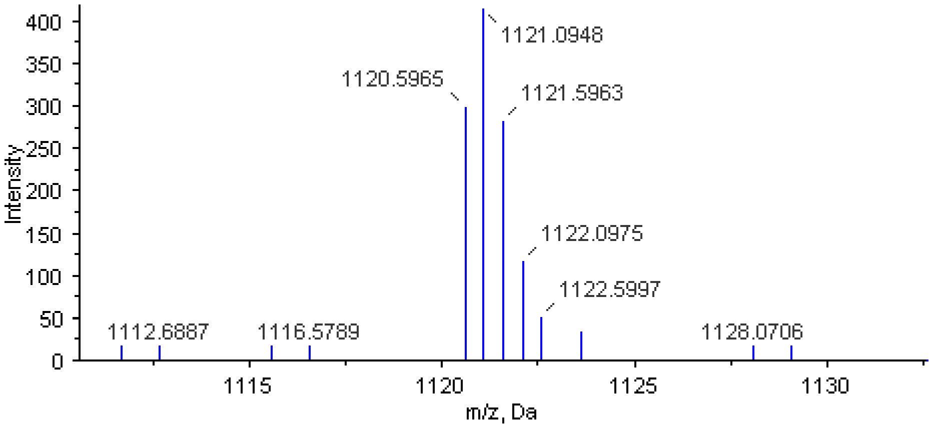

Sequence Coverage and Matched peptides: in different color

EQCGRQAGGALCPGGLCCSQFGWCGSTADYCTVPGCQSQCSGSGPAPGPGGLTNLISRETFNRMLLHRN
DGACPARGFYTYDAFIAAARSFPAFATTGDQATRKREIAAFLAQTSHETTGGAGWAAPDGPYAWGYCYN
RELNPPSSYCASGPNYPCSPGKQYFGRGPMQLSWNYNYGPCGRAIGVDLLNNPDLLSSDPTVSFKSAFW
FWMTPQSPKPSCHNVIIGAWSPSSSDRAAGRATGYGVITNIINGGLECGKGWNAQVEDRIGFYKRYCDI
LGVSYGNNLDCYNQRPFGNGVSVDSM

Protein No.: I-366

Protein name and Species:

Protease inhibitor protein OS=Hevea brasiliensis PE=4 SV=1

Accession: tr|B3FNP9|B3FNP9_HEVBR

Unused ProtScore: 6.31

Seq Cov %: 67.1

ITRAQ Changed Ratio:

117: 115=E3TL: D3TL=2.78

116: 114=E5TL: D5TL=1.56

Annotated typical Precursor MS Region:

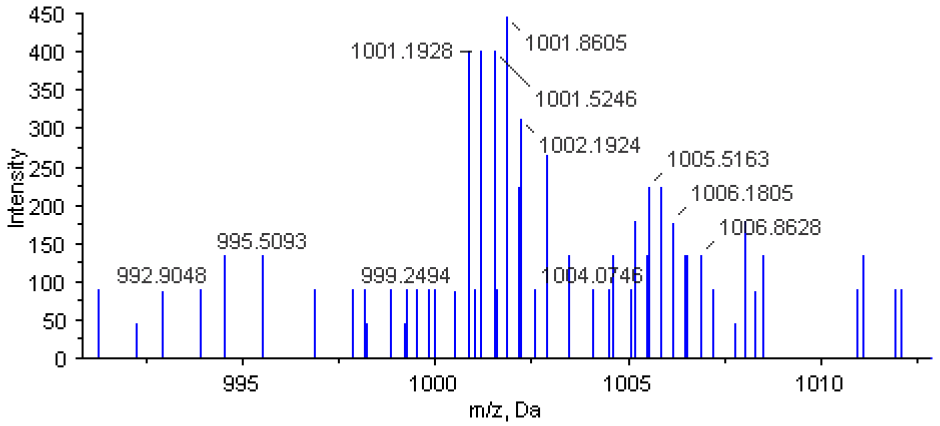

Sequence Coverage and Matched peptides: in different color

MASQCPVK**DAWPELIGTNGDIAAGIIETENANVK**AIVLK**KGSPMTMEYNLCRVLVFVDDNR**VVTQAPVI
G

Protein No.: I-369

Protein name and Species:

Ras-related protein RABB1b-like OS=Populus trichocarpa GN=POPTRDRAFT_648984 PE=2 SV=1

Accession: tr|A9P8X5|A9P8X5_POPTR

Unused ProtScore: 6.2

Seq Cov %: 51.7

ITRAQ Changed Ratio:

117: 115=E3TL: D3TL=2.61

116: 114=E5TL: D5TL=1.12

Annotated typical Precursor MS Region:

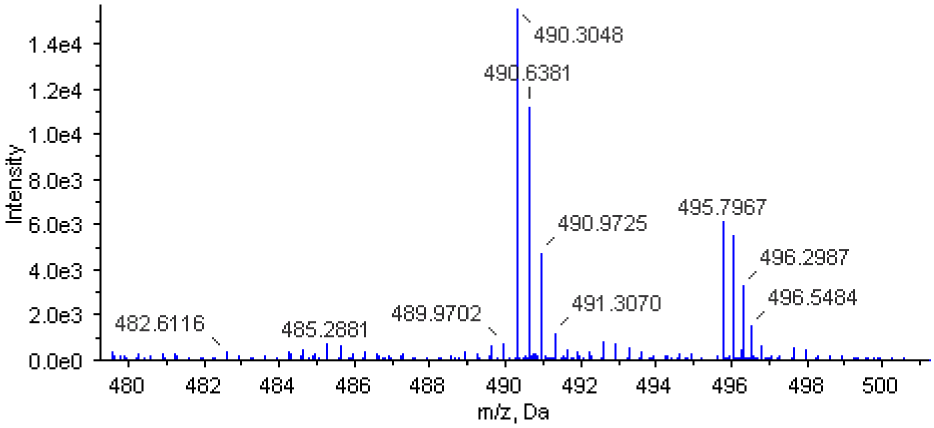

Sequence Coverage and Matched peptides: in different color

MSYDYLFKYIIIGDTGVGKSCLLLQFTDKRFQPVHDLTIGVEFGARMVTIDARPIKLQIWDTAGQESFR
SITRSYYRGAAGALLVYDITRRETFNHLASWLEDARQHANPNMSIMLIGNKCDLAHRRAVSKEEGEQFA
KENGLLFLEASARTAQNVEEAFIGTAGKILQNIQEGVFDVSNESSGIKVGYGRPQGASGARDGTVAQRG
GCCS

Protein No.: I-370

Protein name and Species:

Asparaginyl-tRNA synthetase, cytoplasmic 1-like [Vitis vinifera]

OS=Vitis vinifera GN=VIT_11s0016g02680 PE=3 SV=1

Accession: **tr|F6HGX0|F6HGX0_VITVI**

Unused ProtScore: **6.18**

Seq Cov %: **11.2**

ITRAQ Changed Ratio:

117: 115=E3TL: D3TL=1.64

116: 114=E5TL: D5TL=1.17

Annotated typical Precursor MS Region:

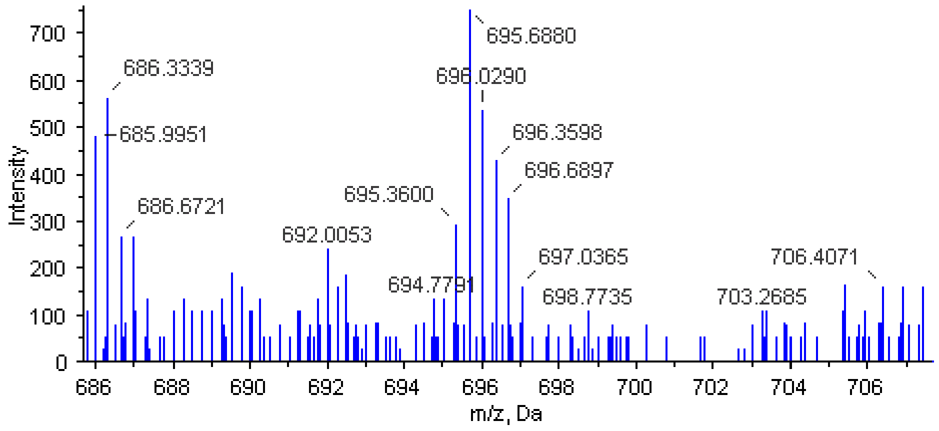

Sequence Coverage and Matched peptides: in different color

MAEDSVPPVDQMALATLNDDVSTPFVKAQFSDRHLIRTILSRPDGGAGLAGQTVKVGGWVKTGREQGKG
SFAFLELNDGSCPANLQVIVDAAVAPLGQLVQTGTCVHVEGLLKVPPEGTKQRVELRVEKVHHVGPVDP
AKYPLPKTR**LTLEFLR**DFVHFRPR**TNTISAVAR**IRNALAYATHTFFQNHGFLYIHTPIITTSDCEGAGE
MFQVTTLISDAEKVEK**ELIKNPPPSEADIEAAK**ALVKEKGEAVAQLKSAKASKGEITASVAELNKAKEN
LSRLEERSKLKPGIPQKDGKIDYSQDFFARQAFLTVSGQLQVETYACAVSSVYTFGPTFRAEHSHTSRH
LAEFWMVEPEIAFADLKDDMNCAEAYVKFLCQWLLDNCIDDMEFMAKNFDKGCIDRLRMVASTPFER**IS**
YTEAIKLLEEAVKKDKKFENKVEWGIDLASEHERFLTEVLFKKPVIVHDYPKGIKAFYMRLNDDMK**TVA**
AMDVLVPKVGELIGGSQREERYEVIEKRILEMGLPLEPYEWYLDLRRYGTVKHCGFGLGFER**MILFATG**
IDNIRDVIPFPRYPGRADL

Protein No.: I-371

Protein name and Species:

S-adenosyl-methionine-sterol-C-methyltransferase OS=Ricinus communis PE=2 SV=1

Accession: tr|O24328|O24328_RICCO

Unused ProtScore: 6.18

Seq Cov %: 17.6

ITRAQ Changed Ratio:

117: 115=E3TL: D3TL=0.63

116: 114=E5TL: D5TL=1.24

Annotated typical Precursor MS Region:

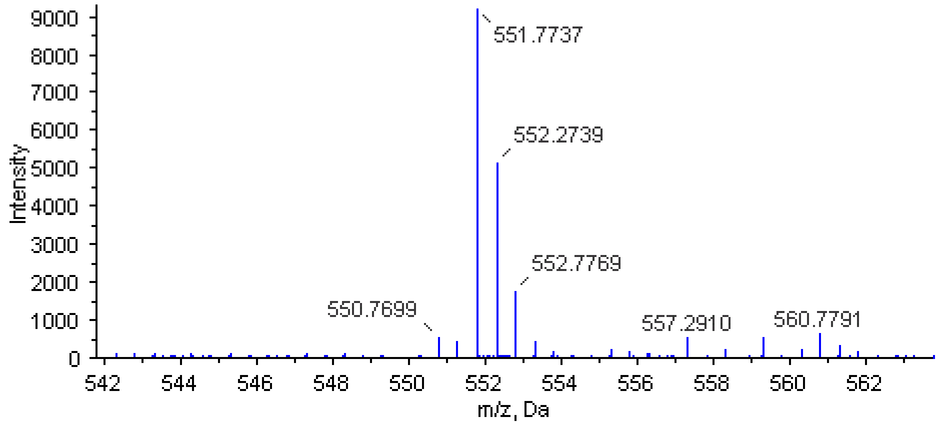

Sequence Coverage and Matched peptides: in different color

MSKAGALDLASGVGGKIDKNEVLSAVEKYEKYHVYYGGAEEERKANYTDMVNKYYDLVTSFYEFGWGES
FHFAHRFKGESLRESIKRHEHFLALQLGLKPEQKVLDVGCGIGGPLREISRFSLTSVTGLNNNEYQITR
GKVLNRIAGVDKTCDFVKADFMKMPFPANSFDAVYAIEATCHAPDAYGCYSEIYRVLKPGQFFAAYEWC
MTDSFDPNNQEHQKIKAEIEIGDGLPDIRLTGQCIEALKKAGFDVIWSGDLTASSPVPWYLPLDKNQYS
LTGFRLTAIGRFFTRNMIKALEFAGLAPKGSQRVQDFLEKAAEGLVEGGRKEIFTPMFFFLAQKPHSEN
Q

Protein No.: I-372

Protein name and Species:

60S ribosomal protein L4 [Vitis vinifera] OS=Vitis vinifera GN=VIT_08s0040g03200 PE=4 SV=1

Accession: tr|F6HQV3|F6HQV3_VITVI

Unused ProtScore: 6.14

Seq Cov %: 8.1

ITRAQ Changed Ratio:

117: 115=E3TL: D3TL=2.31

116: 114=E5TL: D5TL=2.14

Annotated typical Precursor MS Region:

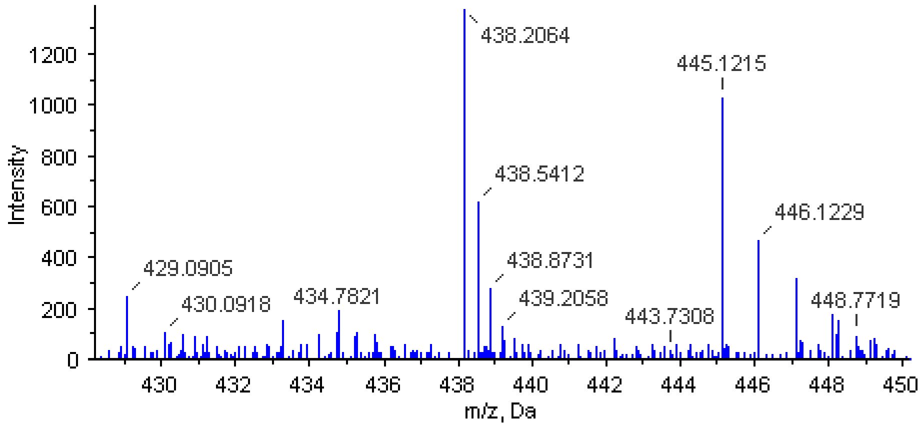

Sequence Coverage and Matched peptides: in different color

MAAAAARPLVTVQVLKSDMATDGGNAVPLPDVMKASIRPDIVTFVHSNISKNSRQPYAVSRRAGHQTSA
ESWGTGRAVSRIPRVPGGGTHR**AGQGAFGNMCR**GGRMFAPTKIWRRWHRKINVNQKRYAVVSAIAASAI
PSLVMARGHRIESVPELPLVIGDSAESVEKTSAAIDILKQVGAYPDAEKAKDSHAIRPGKGKMRNRRYI
SRKGPLIVYGTEGAKLVKAFR**NIPGVEVANVDR**LNLLKLAPGGHLGRFVIWTKSAFEK**LDSIYGSFEK**T
SEKKNGYLLPRSKMVNADLARIINSDEVQSVVRPIKKEVKRAPMKKNPLKNLNTMLKLNPYAKTARRMA
LLAEAQRVKAKQEKLDKKRKQIPKEEVAAIKAAGKAWYQTMISDSDYTEFDNFSKWLGVSQ

Protein No.: I-373

Protein name and Species:

Malate dehydrogenase OS=Ricinus communis GN=RCOM_1520380 PE=3 SV=1

Accession: tr|B9SE47|B9SE47_RICCO

Unused ProtScore: 6.14

Seq Cov %: 13.6

ITRAQ Changed Ratio:

117: 115=E3TL: D3TL=0.28

116: 114=E5TL: D5TL=0.31

Annotated typical Precursor MS Region:

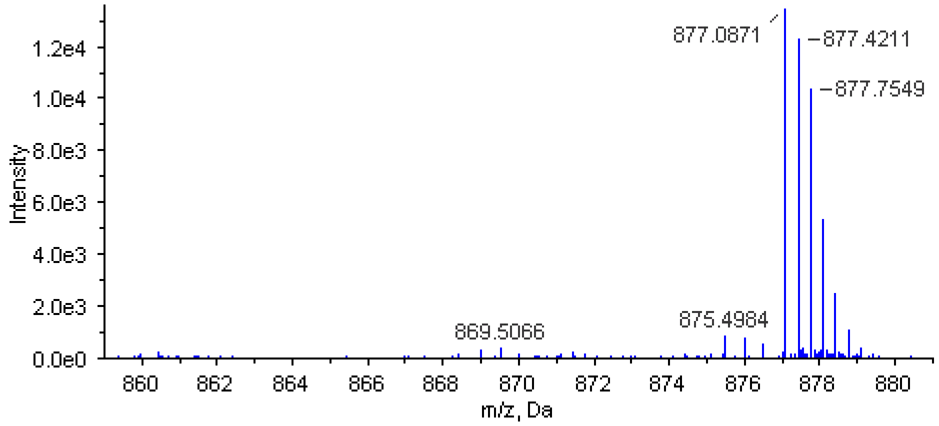

Sequence Coverage and Matched peptides: in different color

MRSSLFRSVKALGSTSSSSHLLRRGYASEAVPDRK**VAVLGAAGGIGQPLALLMK**LNPLVSNLALYDIAN
TPGVAADVSHINTRSDVKGYVGEDQLGK**ALEGSDVVIIPAGVPR**KPGMTR**DDLFNINAGIVK**GLCEAIA
KYCPNALVNMISNPVNSTVPIAAEVFKKAGTYDEKRLFGVTTLDVVRAKTFYAGKAKVPVAEVNVPVVG
GHAGITILPLLSQATPKANLPDEEIVALTKRTQDGGTEVVEAKAGKGSATLSMAYAGAIFADACLKGLN
GVPDVVECSFVQSTVTELPFFASKVRLGK**NGVEEVLGLGPLSDYEK**EGLEKLKPELLSSIEKGINFANK

Protein No.: I-374

Protein name and Species:

Alcohol dehydrogenase, putative OS=Ricinus communis GN=RCOM_0605880 PE=4 SV=1

Accession: tr|B9SUZ4|B9SUZ4_RICCO

Unused ProtScore: 6.12

Seq Cov %: 18.5

ITRAQ Changed Ratio:

117: 115=E3TL: D3TL=0.09

116: 114=E5TL: D5TL=0.29

Annotated typical Precursor MS Region:

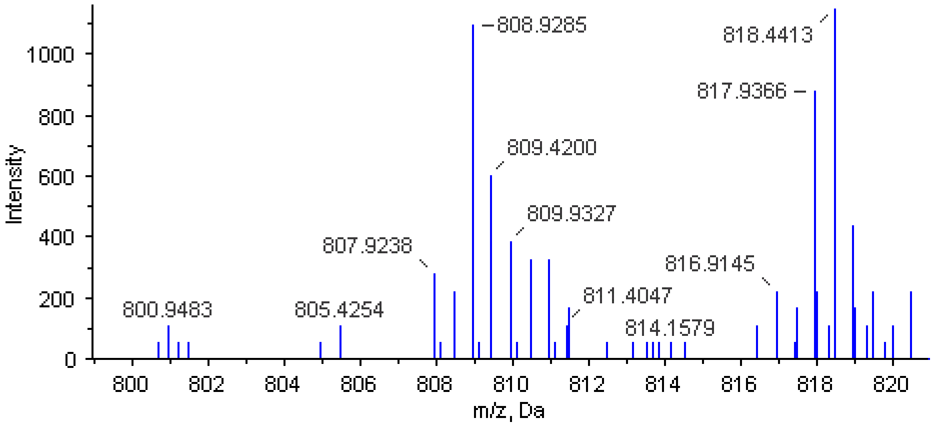

Sequence Coverage and Matched peptides: in different color

MTSKESVHRNSRRKAIDYREMASEAEVVSNKQVIFKEYVSGFPKESDMYMTTSSVKLQVEEGSTSVLVK
NLYLSCDPYMRGRMRNTPSDDPEFSPLHPGSPVFGLGVAK**VVDSGHPGFK**KGDLVWGSTRWEEYTLITA
PEGLFKIHHTDIPLSYYTGLLGMPGITAYFGLNDICTPKKGER**VYVSAASGAVGQLVGQFAKLMGCYVV**
GSAGSKEKVDLLKNKFGFDEAFNYREEHDWNAALKR**YFPEGIDIYFENVGGKMLDAVLLNMR**THGRIAA
CGMISQYNLEHPDGVHNLIVVVHKRIRIQGFISFDYFGQYSK**FLDFVLPYIR**EGKITYVEDIAEGIESA
PAALVGLFSGRNVGK**QVVAVAHE**

Protein No.: I-375

Protein name and Species:

Methionine synthase OS=Camellia sinensis PE=2 SV=1

Accession: tr|G3G858|G3G858_CAMSI

Unused ProtScore: 6.11

Seq Cov %: 17.5

ITRAQ Changed Ratio:

117: 115=E3TL: D3TL=0.39

116: 114=E5TL: D5TL=0.51

Annotated typical Precursor MS Region:

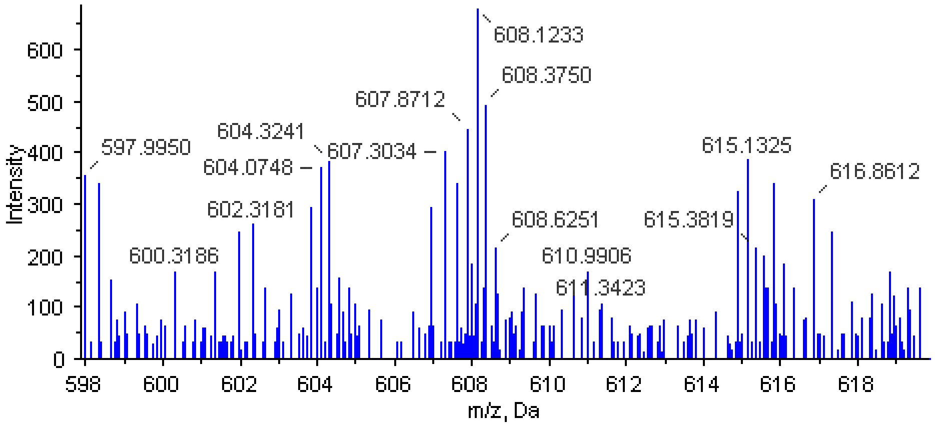

Sequence Coverage and Matched peptides: in different color

M**ASHIVGYPR**MGPKRELK**FALESFWDK**KSSAEDLKKVAADLRSSIWKQMASAGIKYIPSNTFSYYDQVL
DTTAMLGAVPPRYGWTGGEIGFDTYFSMARGNASVPAMEMAKWFDTNYHFIVPELGPEIKFSYASHKAV
DEYKEAK**ALGVDTVPVLVGPVSYLLLSK**PAKGVEK**SFSLLSLLDK**ILPIYKEVVAELKAAGASWIQFDE
PTLVKDLESHQLQAFTAAYSELESALSGLNVIVETYFADVPAEAFKTLTSLKCVTGFGFDLVRGAQTLD
LITSGFPSGE**YLFAGVVDGR**NIWANDLAASLSLLQTLEGIVGKDKLVVSTSCSLLHTAVDLVNETKFDK
ELK**SWLAFAAQK**VVEVNSLAKALAGHKDEAFFSENAAAQASRKSSPRVTNEAVQKAAAALRGSDHRRAT
NVSARLDAQQK**KLNLPILPTTTIGSFPQTIELR**RVRREYKAKKISEEEYVKAIKEEINKVVKLQEELDI
DVLVHGEPERNDMVEYFGEQLSGFAFSANGWVQSYGSRCVKPPIIFGDVSRPKAMTVFWSSMAQSMTTR
PMK**GMLTGPVTILNWSFVR**NDQPRFETCYQIALAIKDE**VEDLEK**AGINVIQMDEAALREGLPLRKSEQA
FYLDWAVHSFRITNCGVTDTTQIHTHMCYSNFNDIIHSIIDMDADVITIENSRSDEKLLSVFREGVK**YG**
AGIGPGVYDIHSPRIPSTEEIADRINKMLQVLET**NILWVNPDCGLK**TRKYHEVKPALLNMVAAAKQIRT
QLASAK

Protein No.: I-382

Protein name and Species:

Trafficking protein particle complex subunit, putative OS=Ricinus communis GN=RCOM_0183430 PE=4 SV=1

Accession: tr|B9SA27|B9SA27_RICCO

Unused ProtScore: 6.03

Seq Cov %: 24.2

ITRAQ Changed Ratio:

117: 115=E3TL: D3TL=1.71

116: 114=E5TL: D5TL=1.47

Annotated typical Precursor MS Region:

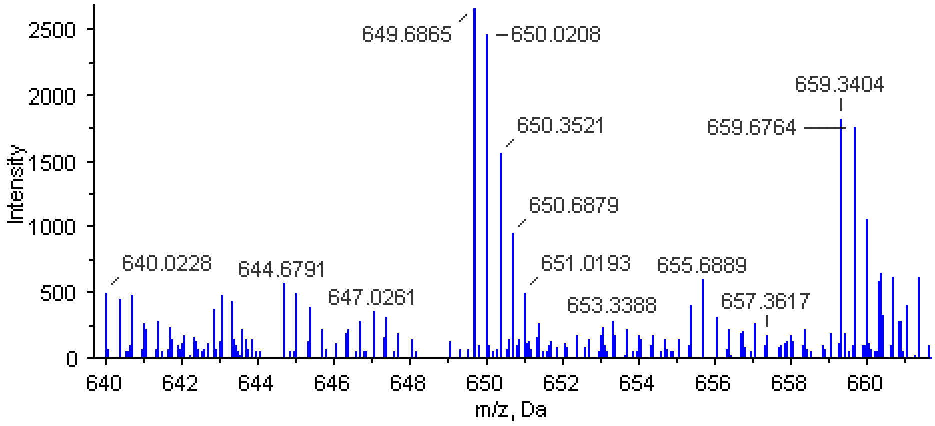

Sequence Coverage and Matched peptides: in different color

MAPVGPRSGDAIFASVERVNAELFTLTYGAIVRQLLTDLEEVEEVNKQLDQMGYNIGIRLVDEFLAKSN
VNRCVDFRETAEVIAKVGFKMFLGVSASVTNWDADGTCCSIILEDNPLVDFVELPDTCQGLYYCNILSG
VIRGALEMVSMKTEVTWARDMLRGDDAYELQVKLLKQVPEEYPYKDDE

Protein No.: I-383

Protein name and Species:

Sucrose phosphate phosphatase OS=Malus domestica PE=2 SV=1

Accession: tr|Q5J3P0|Q5J3P0_MALDO

Unused ProtScore: 6.02

Seq Cov %: 9.9

ITRAQ Changed Ratio:

117: 115=E3TL: D3TL=0.50

116: 114=E5TL: D5TL=0.58

Annotated typical Precursor MS Region:

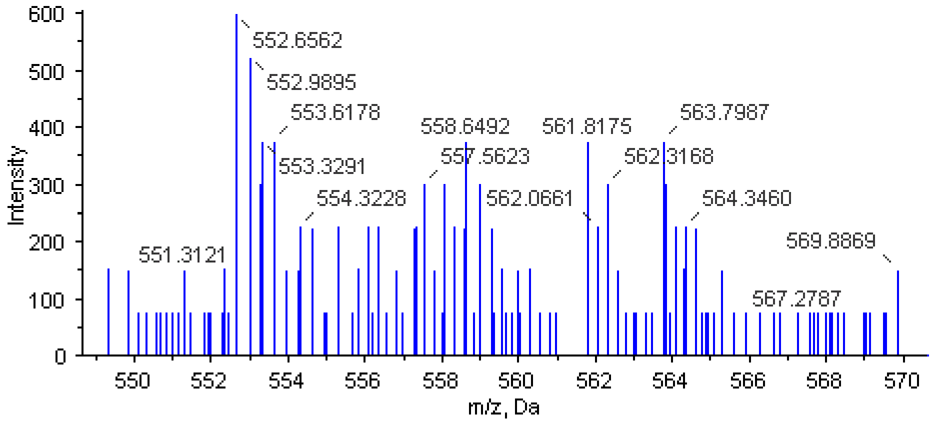

Sequence Coverage and Matched peptides: in different color

MDRLEAPARLMIVSDLDHTMVDHHDTENLSLLRFNSLWEANYC**HDSLLVFSTGR**SPTLYKELRKEKPML
TPDITIMSVGTEITYGNAMVPDDGWVEVLNQKWDRNIVKEEASKYSELKLQAETEQRPHKVSFYVEKAK
AQAVTKALSEVFEKRGLDVK**IIYSGGMDLDILPQGAGK**GQALAYLLKKFKSEGSSPVNTLVCGDSGNDA
ELFSIPEVYGVMVSNAQEELLQWHAENAKGNTRIIHATER**CAAGIIQAIGHFK**LGPSLPPRDIADFSDF
KLENPNPGHELVKFFLFYEKWRRAEVENSEIYLASLKADCSPSGTFVHPSGVEHSLSDSINALRNCYGD
KQGKQFRVWVDGVLATHVGSNTWLVKFDKWELSGEERYAIKGTAVISSKGSGVSDGFTWIRVHQTWYKG
YEAKDDSTWFF

Protein No.: I-386

Protein name and Species:

60S ribosomal protein L9, putative OS=Ricinus communis GN=RCOM_1315040 PE=4 SV=1

Accession: tr|B9RYZ1|B9RYZ1_RICCO

Unused ProtScore: 6

Seq Cov %: 19.1

ITRAQ Changed Ratio:

117: 115=E3TL: D3TL=2.19

116: 114=E5TL: D5TL=1.74

Annotated typical Precursor MS Region:

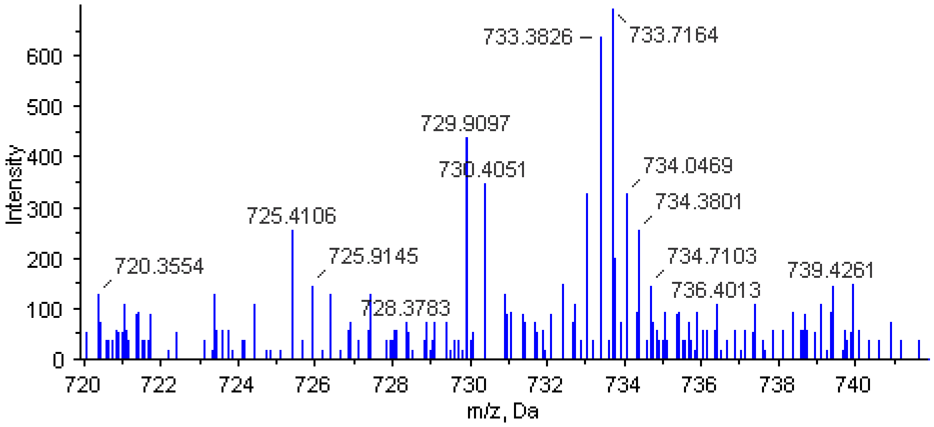

Sequence Coverage and Matched peptides: in different color

MKTILSSETMDIPDGVKIKVNAKIIEVEGPRGKLTRNFKHLNLDFQLIKDEETGKRKLKIEAWFGSRKT
SAAIR**TALSHVENLITGVTK**GYRYKMRFVYAHFPINASITNSNSAIEIRNFLGEKKVR**KVDMLEGVTVV**
RSEKVKDELVLDGNDIELVSRSAALINQKCHVKNKDIRK**FLDGIYVSEK**GTVAEQE

Protein No.: I-389

Protein name and Species:

Predicted protein OS=Populus trichocarpa GN=POPTRDRAFT_571988 PE=4 SV=1

Accession: tr|B9IBL7|B9IBL7_POPTR

Unused ProtScore: 6

Seq Cov %: 6.5

ITRAQ Changed Ratio:

117: 115=E3TL: D3TL=6.19

116: 114=E5TL: D5TL=1.20

Annotated typical Precursor MS Region:

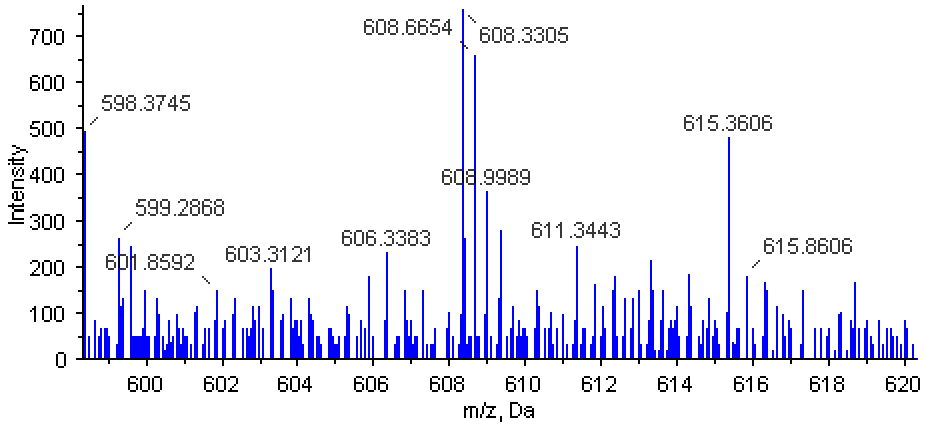

Sequence Coverage and Matched peptides: in different color

MSWLARSIANSLKFEDDDQQPDKNTTATTTSAPTTDNVNNNKDLSNSESDQQQSATPRGVKEDLTDLKK
TLTRQLWGVASFLAPPPEPPLTENRSHTSDPDGSNDDDDDAALIAGIR**SDFAEIGGR**FKSGITKLSSNK
TVSEFTKIASNLLQLGSESHEIGTTASAVGVTEEVVGFARDVAMHPETWLDFPLPDVQGFEDFDMSDAQ
QEHALAVEHLAPRLAALRIELCPGYMSEGCFWK**IYFVLLHPR**LSK**HDAELLSTPQIVEAR**AMLSHELQN
KAKAKSTPDWSGVDTSNVKADLPHEESLSVPSRAKSESVPIMTSGIEAVSPTLAAKTSDNEAAPSFVSV
ESETEKHPVESTQMQIIDKSVVEEGKVDQTKHQHSSSSSSSGILEEKFDDDGDDWLKDDSSEMIGVSGS
SMPLGNDEDVSFSDLEEDDGDEPASYKKVASGSDCATKGSQDWVQLSRSSADSVKDIKPVSIKNAGSEK
VSARNSENKESSDWLDVDDIDVI

Protein No.: I-390

Protein name and Species:

Putative uncharacterized protein OS=Vitis vinifera GN=VIT_19s0015g02610 PE=3 SV=1

Accession: tr|F6I510|F6I510_VITVI

Unused ProtScore: 5.99

Seq Cov %: 18

ITRAQ Changed Ratio:

117: 115=E3TL: D3TL=0.39

116: 114=E5TL: D5TL=0.71

Annotated typical Precursor MS Region:

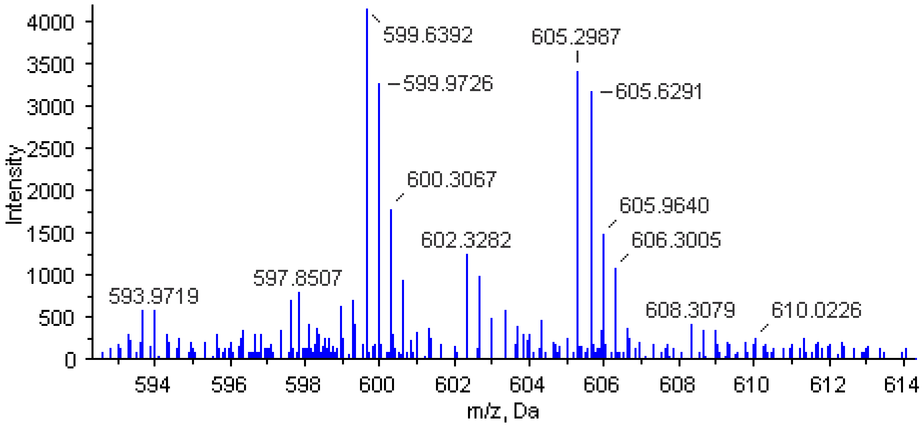

Sequence Coverage and Matched peptides: in different color

MADEIILLDFWPSMFGMRVR**LALAEK**GLK**YEYRQEDLR**NK**SPLLLEMNPVHK**K**IPVLIHNGK**PICESLI
IVQYIDEVWK**DKSPLLPSDPYQR**AQARFWADYVDKKLYELGGKIWSTKGEEQETGK**KEFIECLKLLEGE**
LGEKPYFGGEKIGFVDVALVTFSCWFYAYETFGNFSIEAECPKLVAWTKRCMEKESVSSSLEDPHKVHG
FIMGMRKRFGIEVR**LALAEK**GLK**YEYKEEDLR**NK**SPLLLEMNPVHK**K**IPVLIHNGK**PICESLIIVQYID
EVWH**DKSPLLPSDPYQR**AQARFWADYVDKKGFVQNAGFFFTHSLPKAPSSS

Protein No.: I-391

Protein name and Species:

Carbonic anhydrase OS=Populus trichocarpa GN=POPTRDRAFT_666464 PE=3 SV=1

Accession: **tr|B9IER7|B9IER7_POPTR**

Unused ProtScore: **5.98**

Seq Cov %: **11.8**

ITRAQ Changed Ratio:

117: 115=E3TL: D3TL=0.54

116: 114=E5TL: D5TL=1.06

Annotated typical Precursor MS Region:

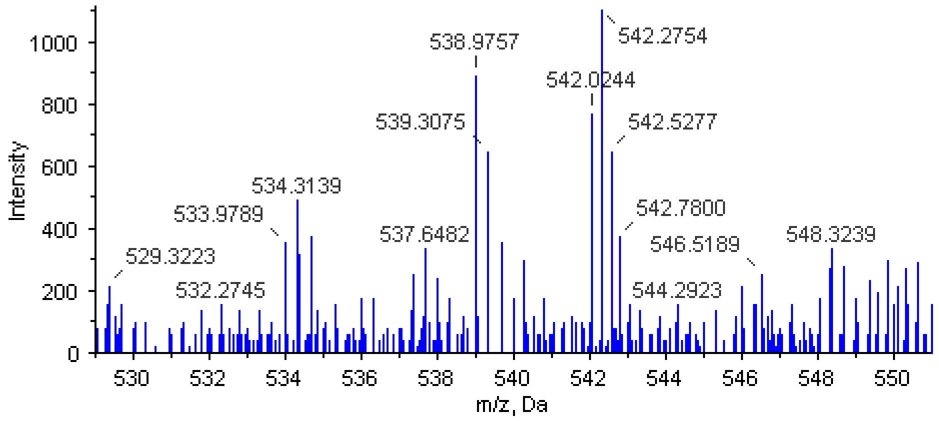

Sequence Coverage and Matched peptides: in different color

MSNLSHEGAIEGSKKLLIDNEKDDQLNKEVEAKIEKLIGELQGKRLPDHCDPVGRILEGFHRFKTTKFD
KYPELYRELAEGQSPKFLVFACSDSR**VSPSHVLDFQPGEAFMVRNIANLVPAFNQLR**YSGVGATIEYAV
ATLGVENILVIGHSRCGGIERLMTLPEDGSTANDFVDDWVKIGLPAKAKVEAEFGHLPLPEQIHKCEKE
AVNLSLINLQTYPYVQERMAEGALALRGGYYDFVKGCFELWEVKSTVTPPISTCCK

Protein No.: I-392

Protein name and Species:

Protein SEY1, putative; root hair defective 3 OS=Ricinus communis GN=RCOM_0312010 PE=4 SV=1

Accession: tr|B9SN36|B9SN36_RICCO

Unused ProtScore: 5.95

Seq Cov %: 7.6

ITRAQ Changed Ratio:

117: 115=E3TL: D3TL=5.06

116: 114=E5TL: D5TL=1.78

Annotated typical Precursor MS Region:

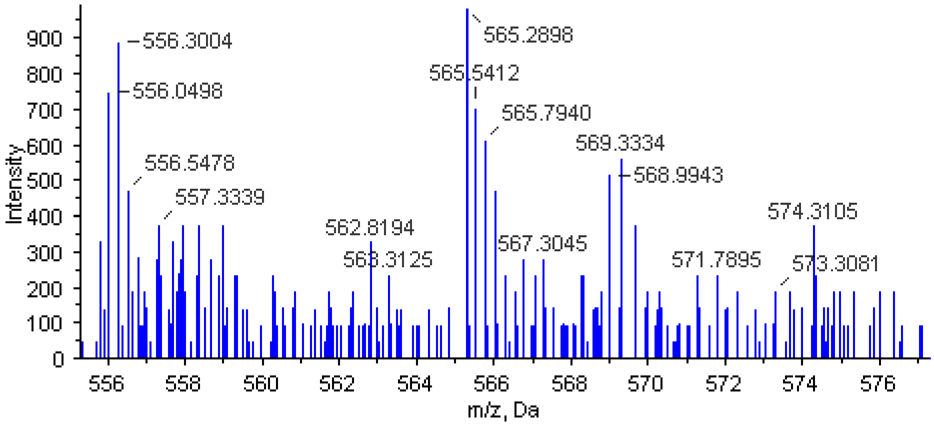

Sequence Coverage and Matched peptides: in different color

MANSEESCSTQLIDGDGTFNAAGLEHFTKEVRLGECGLSYAVVSIMGPQSSGKSTLLNNLFGTNFREMD
AFRGRSQTTKGIWLARCAGIEPCTLVMDLEGTDGRERGEDDTAFEKQSALFALAVSDIVLINMWCHDIG
REQAANKPLLKTVFQVMMRLFSPRKTTLMFVIRDKTRTPLENLEPVLREDIQKIWDAVPKPQEHKETPL
SEFFNVEVVALSSYEEKEEQFKEQVASLRQRFFHSIAPGGLAGDRRGVVPASGFSFSAQQMWKVIKENK
DLDLPAHKVMVATVRCEEIANEKYANFTTNEEWHQIEEAVQSGPVSGFGKKLSSTLSTSFSEYDAEAIY
FDEGVRSAKRKQLEEKLLQLVQPAHQSMLGHIRSGTLDKFKEAFDKALAAGEGFSSAAYSCTQYYMTVF
DEGCTDAIIEQASWDTSKVRDKLRRDIDAHVASVRAAKLSELTSSFEAKLNEALSGPVEALLDGATSET
WPAIRKLLQRESESAVSGLSSALAGFDMDKQSKDKMLSSLETYARGVVEAKAKEEAGRVLIRMKDRFSM
LFSHDSDSMPRVWTGKEDIRAITKTARSASLKLLSVMVAIRLDDEVDNVESTLSSVFLDTKNNAAVTER
SITKTDPLASSTWDEVPSSKTLITPVQCKSLWRQFKAETEYSVTQAISAQEANKRNNNWLPPPWAIVAL
VVLGFNEFMTLLRNPLYLGFIFVVFLLVKALWVQLDVSGEFRNGALPGLISLSTKFLPTIMNLIKKLAE
EGQKPATNDPQRNPALAAKSFRNGVGSSDDMSTASSGVTSTENGTEFSSASKDD

Protein No.: I-395

Protein name and Species:

Receptor for activated protein kinase C, putative OS=Ricinus communis GN=RCOM_0571360 PE=4 SV=1

Accession: tr|B9SU88|B9SU88_RICCO

Unused ProtScore: 5.85

Seq Cov %: 17.4

ITRAQ Changed Ratio:

117: 115=E3TL: D3TL=1.75

116: 114=E5TL: D5TL=1.46

Annotated typical Precursor MS Region:

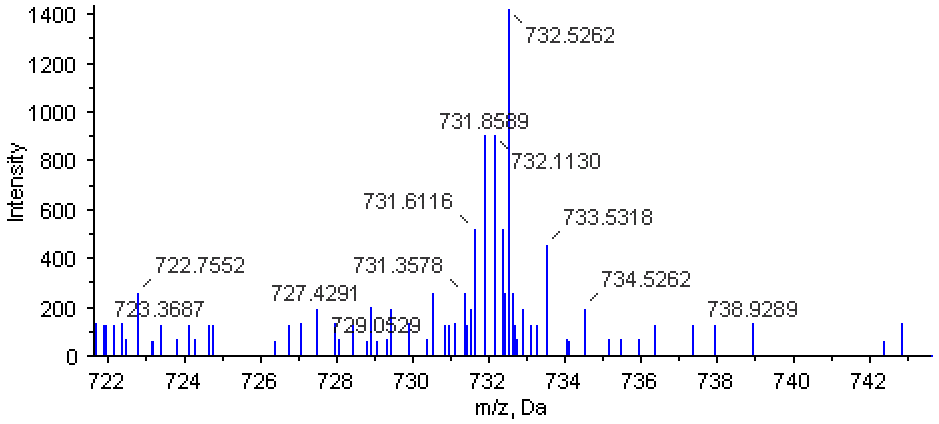

Sequence Coverage and Matched peptides: in different color

MAERLILLGTMRAHTDQVTAIATPIDNSDMIVTASRDKSIILWKLIKDEKTYGVAHRRLTGHSHFVQDV
VLSSDGQFALSGSWDGELRLWDLAAGVSARRFVGHTKDVLSVAFSIDNRQIVSASRDRTIKLWNTLGEC
KYTIQDGDAHNDWVSCVRFSPNTLQPTIVSASWDRTVKVWNLTNCKLRCTLAGHGGYVNTVAVSPDGSL
CASGGKDGVILLWDLAEGKRLYNLDAGAVIHALCFSPNRYWLCAATENSIKIWDLESKSIVEDLKVDLK
AEAEKSDGSTVASTGAKKKNIYCTSLSWNADGSTLFSGYTDGVIRVWGIGRF

Protein No.: I-401

Protein name and Species:

Fumarylacetoacetate hydrolase (Fragment) OS=Dimocarpus longan PE=2 SV=1

Accession: tr|G0YC94|G0YC94_9ROSI

Unused ProtScore: 5.79

Seq Cov %: 22.9

ITRAQ Changed Ratio:

117: 115=E3TL: D3TL=1.61

116: 114=E5TL: D5TL=3.14

Annotated typical Precursor MS Region:

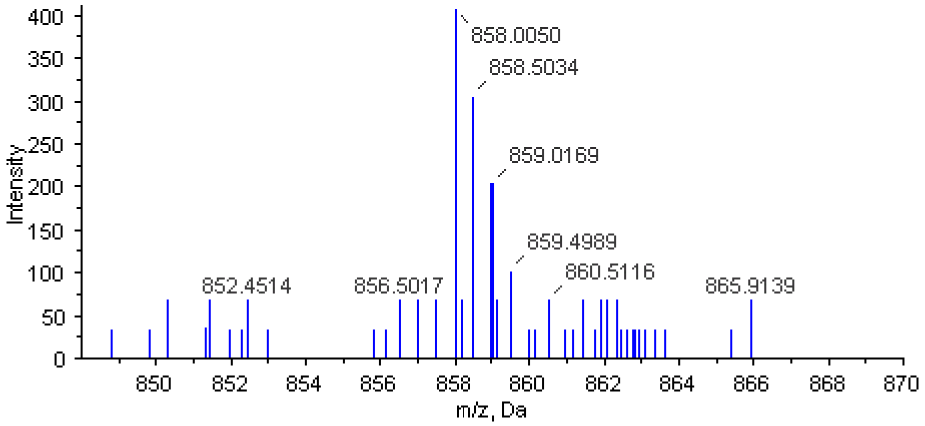

Sequence Coverage and Matched peptides: in different color

MAVLQSFVEVKPDSHFPIQNLPYGVFKPDPTSSPRPGIAIGDYVLDLSEIAKAGLFNGPTLS**GSDCFLQ**
PNLNKFLALGRPAWKEARATLQK**LLSSSEAALRDNASLR**QKSLVSMDKVEMVVPIAIGDYTDFFSSMHH
AK**NCGTIFR**GPQNPVPANWFHLPIAYHGR**ASSIVISGTDIVRPR**GQGVPSGNSPPPFGPSQK**LDFELEM**
AAVVGPGNELGKPVDVNEAADHIFGVVLMNDWSAR**DIQAWEYVPLGPFLGK**SFGTTISPWIVTL

Protein No.: I-408

Protein name and Species:

Protease inhibitor HPI OS=Hevea brasiliensis GN=PI1 PE=1 SV=2

Accession: **sp|Q6XNP7|HPI_HEVBR**

Unused ProtScore: **5.72**

Seq Cov %: **65.7**

ITRAQ Changed Ratio:

117: 115=E3TL: D3TL=1.51

116: 114=E5TL: D5TL=1.34

Annotated typical Precursor MS Region:

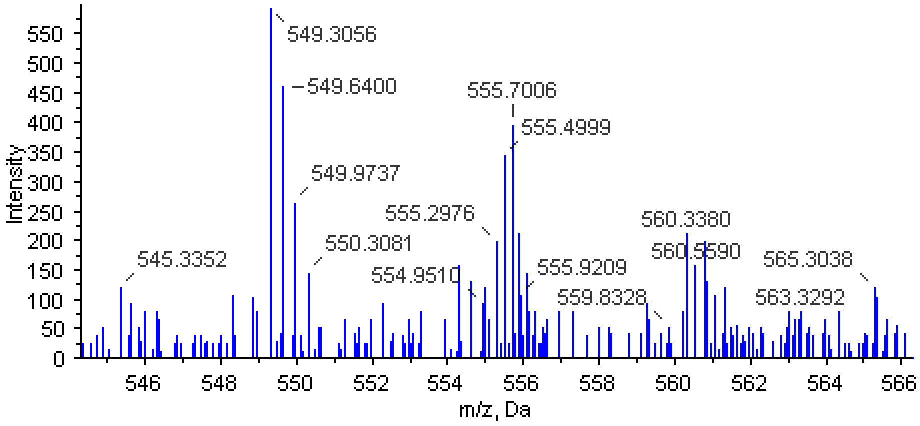

Sequence Coverage and Matched peptides: in different color

MASQCPVK**NSWPELVGTNGDIAAGIIQTENANVK**AIVVK**EGLPITQDLNFNR**VR**VFVDENR**VVTQVPAI
G

Protein No.: I-409

Protein name and Species:

1-phosphatidylinositol-4,5-bisphosphate phosphodiesterase, putative OS=Ricinus communis GN=RCOM_0339640 PE=4 SV=1

Accession: tr|B9T5Y1|B9T5Y1_RICCO

Unused ProtScore: 5.7

Seq Cov %: 13.6

ITRAQ Changed Ratio:

117: 115=E3TL: D3TL=0.58

116: 114=E5TL: D5TL=0.50

Annotated typical Precursor MS Region:

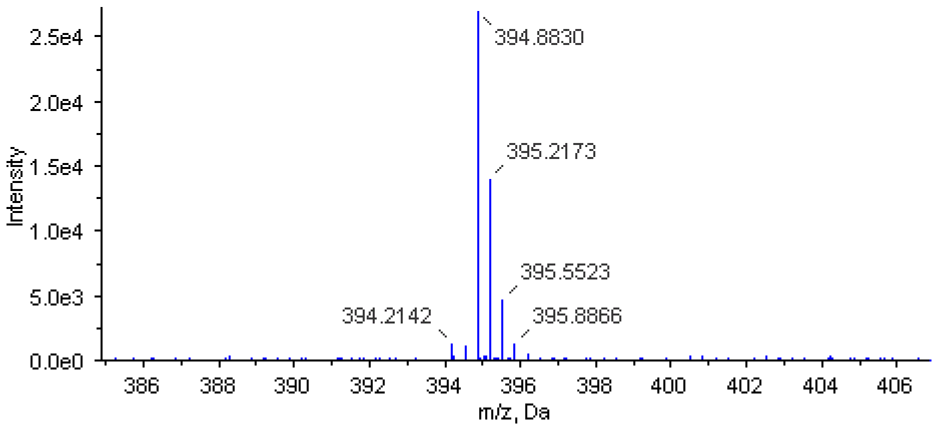

Sequence Coverage and Matched peptides: in different color

MSKQTYRVCFCFRRRFRLAVAEAPQEIKSLFDQYSDNNGLMSIDQLRRFLVEIQKQDNATTEDAQAIFN
NLHELKHLNIFHRKGLNLEAFFK**YLFGDINPPLDVK**RGVHHDMTCPLSHYFIYTGHNSYLTGNQLSSDC
SDVPIIQALQRGVRVIELDIWPNSTK**DNVEVLHGR**TLTTPVELIKCLRSIKEHAFRASDYPVVITLEDH
LTPDLQAK**VAEMITQTFGDILFTPGSECLKEFPSPESLK**KRIIVSTKPPKEYLEVREIREREGDSQSGK
PGSDEETWGKEIPDLKGHFRVDDKNELDEDNNDDEDAAPDGDNKSQQNIAPEYK**HLIAIHAGKPKGGIE**
ECLKVDPDKVRRLSLSEQQLEKAAETHGKEIVRFTQRNILRVYPKGIRVDSSNYNPLIGWMHGAQMVAF
NMQGHGRSLWLMQGMFR**ANGGCGYVK**KPDFLLKSGPHGEVFDPRAKLPVKTTLKVKVYMGEGWYYDFDR
THFDAYSPPDFYARVGIAGVPADTIMKKTKTLEDNWIPVWNEEFEFPLTVPELALLRVEVHEYDMSEKD
DFGGQSCLPVSELRKGIRAIPLHDRKGVKYNSVKLLVRFDFV

Protein No.: I-410

Protein name and Species:

Protein disulfide isomerase, putative OS=Populus trichocarpa GN=POPTRDRAFT_643603 PE=3 SV=1

Accession: tr|B9GU26|B9GU26_POPTR

Unused ProtScore: 5.68

Seq Cov %: 8.9

ITRAQ Changed Ratio:

117: 115=E3TL: D3TL=2.29

116: 114=E5TL: D5TL=2.13

Annotated typical Precursor MS Region:

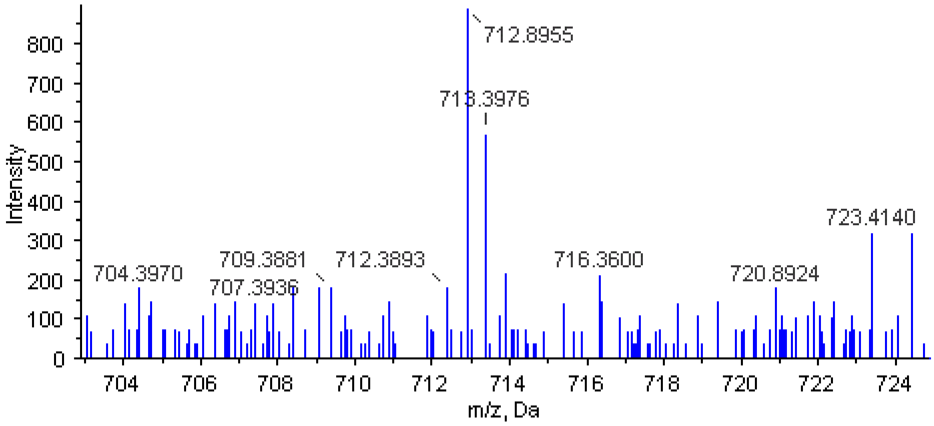

Sequence Coverage and Matched peptides: in different color

MASTVSFWSCIFLLSLIVALSAGEDESKEYVLTLDHSNFNETVSKHDFIVVEFYAPWCGHCKKLAPEYE
KAASILSSNDPQVVLAKVDANEDANKEIASQYDVKGFPTIVILRKGGKSVQEYKGPR**EADGIVEYLK**KQ
SGPASAELKSDDDATGFIGDKKVVIVGVFPK**FSGEEFENFLAVAEK**LR**SDYEFGHTLDAK**YLPRGESSV
SGPLVRLFKPFDELFVDSK**DFNVDALEK**FVEESSIPIVTLFNKDPSNHPFVVKYFDSPLAKAMLFMNFS
SENGDSIRTKYQEVAGLHKGDGLVFLLGDVEASQGALQYFGLKEDQVPLIVIQTTDGQKYLKPNLVSDQ
IAPWLKEYKEGKVPPFKKSEPIPEVNDEPVKVVVADSLDELVTKSGKNVFLEFYAPWCGHCQKLAPILE
EVAISFQSDADVVIAKLDATANDIPSDTYDVKGFPTIFFR**SATGKLVQYEGDR**TKQDIIDFIEKNRDKI
GQQEPAKEEEPAKEQETAKDEL

Protein No.: I-412

Protein name and Species:

DEAD-box ATP-dependent RNA helicase 37-like [Vitis vinifera]

OS=Vitis vinifera GN=VIT_08s0007g07070 PE=4 SV=1

Accession: tr|F6HLF4|F6HLF4_VITVI

Unused ProtScore: 5.65

Seq Cov %: 8.4

ITRAQ Changed Ratio:

117: 115=E3TL: D3TL=3.16

116: 114=E5TL: D5TL=1.61

Annotated typical Precursor MS Region:

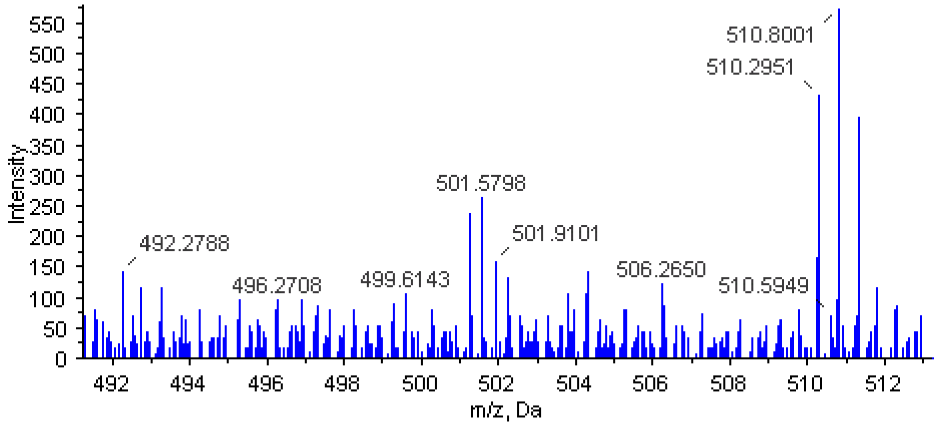

Sequence Coverage and Matched peptides: in different color

MRTSWADSVANSTSDNLVSGSSDNSGFGAGAPSRSGRSSYVPPHLRNRPPSSDPPAPSYTSQASAGYGG
PPGGTRWGGGSRADFGRSGVTTGVTSGGRVGGSSGWNTRSGGWDRGRDREVNPFGDDDNAEPAFSEQEN
TGINFDAYEDIPVETSGDNVPPPVNTFAEIDLGEALNQNIRRCKYVKPTPVQRHAIPISLSGKDLMACA
QTGSGKTAAFCFPIISGIMTGQFAQRPRGAR**TVYPLALILSPTRELSCQIHDEAR**KFSYQTGVKVVVAY
GGAPINQQLRELERGVDILVATPGR**LVDLLER**ARVSLQMIRYLALDEADR**MLDMGFEPQIR**KIVEQMDM
PPPGVRQTMLFSATFPKEIQRLASDFLSSYIFLAVGRVGSSTDLIVQRVEFVHESDKRSHLMDLLHAQR
ANGAHGKQSLTLVFVETKKGADSLEHWLCMNGFPATTIHGDRTQQEREHALRSFK**SGNTPILVATDVAA**
RGLDIPHVAHVVNFDLPNDIDDYVHRIGRTGRAGK**TGLATAFFNENNSSLAR**PLADLMQEANQEVPAWL
TRYASRASYGGGKNRRSGGGRFGGRDFRKDTSFNRGGGATDYYGGNTSSGYGIPGSYGGGYGPGVTSAW
D

Protein No.: I-413

Protein name and Species:

Urease accessory protein ureG OS=Morus alba GN=ureG PE=2 SV=1

Accession: tr|B9A9C9|B9A9C9_MORAL

Unused ProtScore: 5.62

Seq Cov %: 15.1

ITRAQ Changed Ratio:

117: 115=E3TL: D3TL=0.64

116: 114=E5TL: D5TL=1.25

Annotated typical Precursor MS Region:

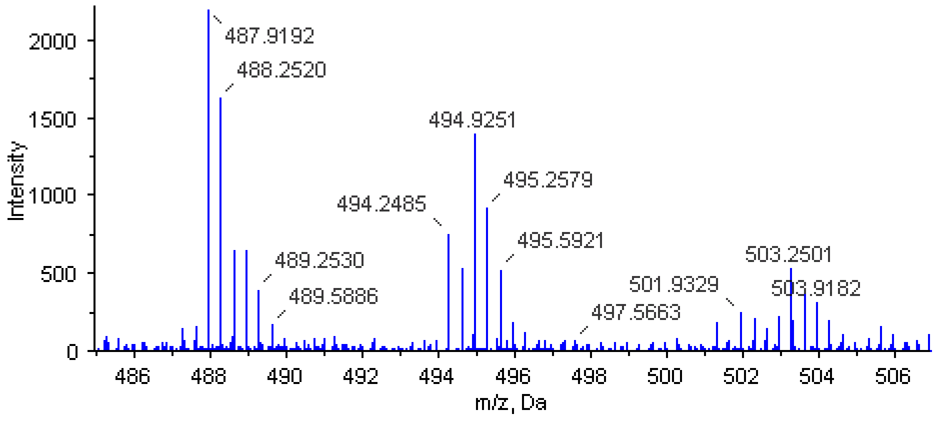

Sequence Coverage and Matched peptides: in different color

MASDDHHHHHDHHHHHDHDHDHDHHGDSRTGAWVGPDGRVYHSHDGLAPHSHEPIYSPGSFSKRAPPLLTRDFNERAFTIGIGGPVGTGKTALMLALCKFLRDK**YSLAAVTNDIFTK**EDGEFLVKNGALPEERIR**AVETGGCPHAAIR**EDISINLGPLEELSNLFKADILLCESGGDNLAANFSRELADYIIYIIDVSAGDKIPRKGGPGITQADLLVINK**TDLAPAVGADLAVMER**DALRMR**DGGPFVFAQVK**HGVGIEEIVNHVLQAWEAATGKKRH

Protein No.: I-414

Protein name and Species:

Predicted protein OS=Populus trichocarpa GN=POPTRDRAFT_580946 PE=4 SV=1

Accession: tr|B9N3J3|B9N3J3_POPTR

Unused ProtScore: 5.61

Seq Cov %: 7.9

ITRAQ Changed Ratio:

117: 115=E3TL: D3TL=0.41

116: 114=E5TL: D5TL=1.27

Annotated typical Precursor MS Region:

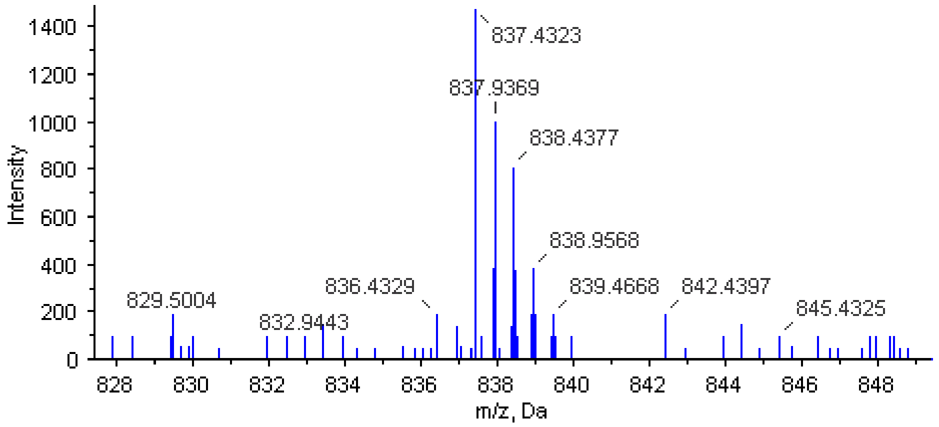

Sequence Coverage and Matched peptides: in different color

MGVDYYNTLKVDKNAKDEDLKKAYRKLAMKWHPDKNPNNKKEAEAKFKQISEAYEVLSDPQKRAIYDQY
GEEGLKGQVPPPETGGPGGATFFSTGDGPATFRFNPRNADDIFAEFFGFSSPFGGMGGGGGGMRGGSRS
FGGMFGDDIFSSFGEARPTNPAPRKAPPIENTLPCSLEELYKGTTKKMKISREIVDVSGKTLPVEEILT
IDIKPGWKRGTKITFPEKGNEQPNVIPADLVFVIDEKPHSTFTREGNDLVVTKKIPLVEALTGCTVHLT
TLDGRTLTIPVNNVIHPNYEEVVAKEGMPIPKDPSRRGNLRIKFDIKFPTRLSAEQKSGIKKLLNS

Protein No.: I-419

Protein name and Species:

Predicted protein OS=Populus trichocarpa GN=POPTRDRAFT_825512 PE=4 SV=1

Accession: tr|B9IJM2|B9IJM2_POPTR

Unused ProtScore: 5.57

Seq Cov %: 11.9

ITRAQ Changed Ratio:

117: 115=E3TL: D3TL=0.41

116: 114=E5TL: D5TL=0.73

Annotated typical Precursor MS Region:

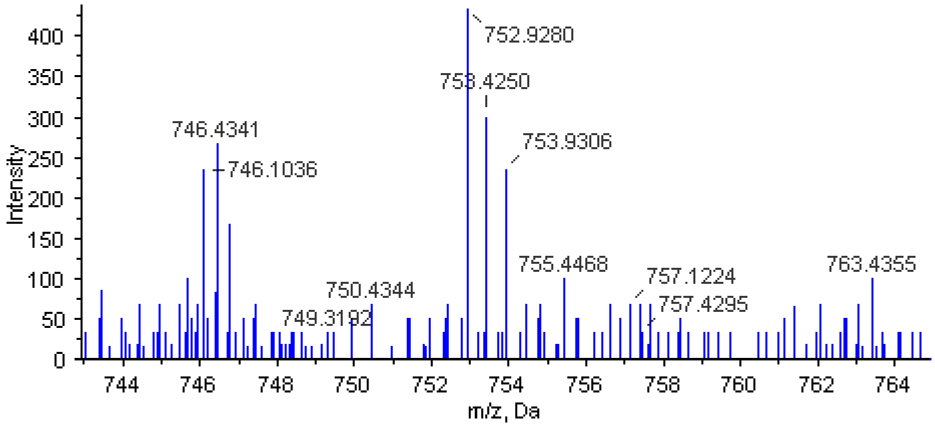

Sequence Coverage and Matched peptides: in different color

MEGAEATRIMMGVNESTIKGYPHASISSR**GAFDWTLQK**IVRSNTSGFKLLFLHVQVPDEDGFDDMDSLY
ASPEDFKNMKHRDRTR**GLHLLEYFVNR**CHEIGVACEAWIKKGDPK**EVICHEVK**RVQPDLLVVGSR**GLGP**
FQRVFVGTVSEFCQK**HAECPVISIK**R**RADETPQDPVDD**

Protein No.: I-422

Protein name and Species:

Aspartic protease (Fragment) OS=Manihot esculenta PE=2 SV=1

Accession: tr|Q94KV2|Q94KV2_MANES

Unused ProtScore: 5.53

Seq Cov %: 43.4

ITRAQ Changed Ratio:

117: 115=E3TL: D3TL=0.25

116: 114=E5TL: D5TL=0.40

Annotated typical Precursor MS Region:

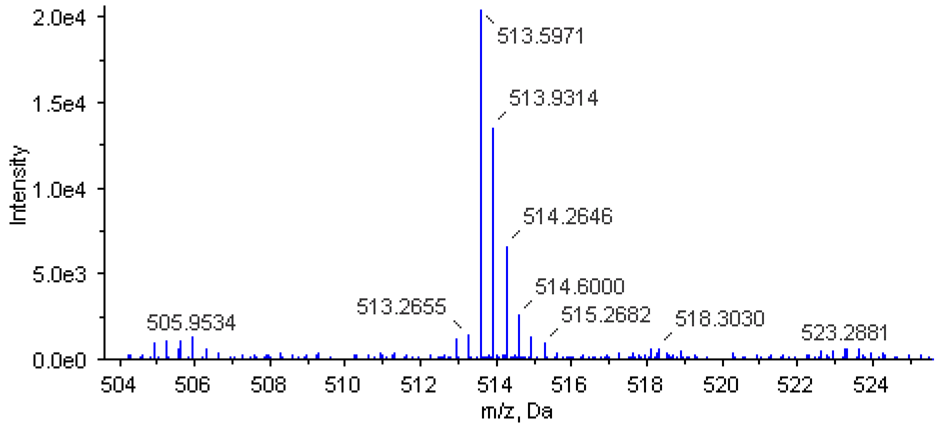

Sequence Coverage and Matched peptides: in different color

CTFDGSRGVSMTIESVVNENSQEVAGSLHDAMCSTCEMAVIWMQNQLKQNATLER**ILNYANELCER**LPS
PMGESAVDCGSLSTMPNVSFTIGGK**VFDLSPEQYVLKVGEGEAAQCISGFTALDVPPPRGPLWILGDVF**
MGRFHTVFDYGNLRVGFAEAA

Protein No.: I-424

Protein name and Species:

Vacuolar H+-ATPase catalytic subunit A; V-type proton ATPase catalytic subunit A-like OS=Populus trichocarpa

Accession: tr|B9HV14|B9HV14_POPTR

Unused ProtScore: 5.45

Seq Cov %: 43.5

ITRAQ Changed Ratio:

117: 115=E3TL: D3TL=3.02

116: 114=E5TL: D5TL=2.08

Annotated typical Precursor MS Region:

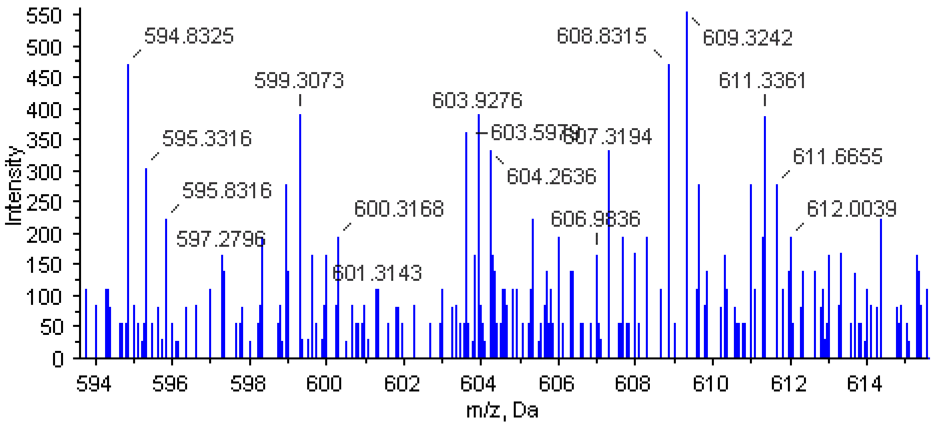

Sequence Coverage and Matched peptides: in different color

MPSVYGAR**LTTFEDSEKESEYGYVR**K**VSGPVVVADGMAGAAMYELVRVGHDNLIGEIIR**LEGHSATIQV
YEETAGLMVNDPVLR**THKPLSVELGPGILGNIFDGIQRPLK**TIARI**SGDVYIPR**GVSVPALDKDTLWEF
QPKKIGEGDLVTGGDLYATVFENTLMQHHVALPPDAMGKITFIAPPGQYSLK**DTVLELEFQGVK**KQFTM
LQTWPVRSPRPVASK**LAADTPLLTGQRVLDALFPSVLGGTCAIPGAFGCGKTVISQALSKYSNSDTVVY**
VGCGERGNEMAEVLMDFPQLTMTLPDGREESVMKR**TTLVANTSNMPVAAR**EASIYTGITIAEYFR**DMGY**
NVSMMADSTSRWAEALREISGR**LAEMPADSGYPAYLAARLASFYER**AGKVKCLGSPERTGSVTIVGAVS
PPGGDFSDPVTSATLSIVQVFWGLDKKLAQRKHFPSVNWLISYSKYSGALESFYERFDPDFINIRTKAR
EVLQR**EDDLNEIVQLVGK**DALAETDKITLETAKLLR**EDYLAQNAFTPYDK**FCPFYKSVWMMRNIIHFCN
LANQAVERGAGMDGQKISYSLIKHR**LGDLFYR**LVSQKFEDPAEGEAVLVEKFSK**LHEDLTAGFRALEDE**
TR

Protein No.: I-426

Protein name and Species:

Enolase 2 OS=Hevea brasiliensis GN=ENO2 PE=1 SV=1

Accession: sp|Q9LEI9|ENO2_HEVBR

Unused ProtScore: 5.44

Seq Cov %: 42.9

ITRAQ Changed Ratio:

117: 115=E3TL: D3TL=0.41

116: 114=E5TL: D5TL=0.52

Annotated typical Precursor MS Region:

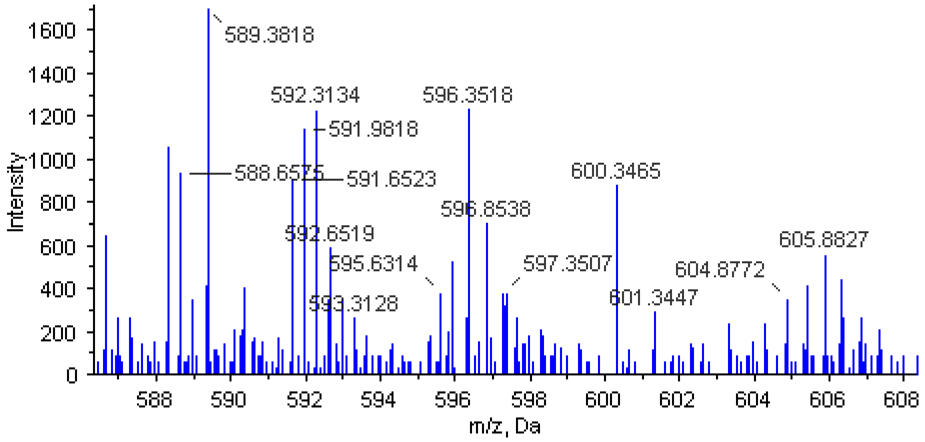

Sequence Coverage and Matched peptides: in different color

M**AITIVSVR**ARQIFDSR**GNPTVEADVKLSDGYLAR**AAVPRGASTGIYEALELR**DGGSDYLGK**GVSK**AVE**
NVNIIIGPALVGKDPTDQVGIDNFMVQQLDGTVNEWGWCKQKLGANAILAVSLAVCK**AGAHVKGIPLYK**
HVANLAGNK**NLVLPVPAFNVINGGSHAGNKLAMQEFMILPVGASSFK**EAMK**MGAEVYHHLK**SVIKK**KYG**
QDATNVGDEGGFAPNIQENKEGLELLKTAIAKAGYTGK**VVIGMDVAASEFYGSDK**TYDLNFKEENNNGS
QKISGDVLKDLYKSFVTEYPIVSIEDPFDQDDWEHYAK**LTSEIGVKVQIVGDDLLVTNPK**RVEKAIKEK
ACNALLLKVNQIGSVTESIEAVKMSKR**AGWGVMASHR**SGETEDTFIADLSVGLATGQIKTGAPCRSERL
AKYNQLLR**IEEELGAEAVYAGANFR**TPVEPY

Protein No.: I-429

Protein name and Species:

alpha-galactosidase-like isoform 1 [Glycine max] OS=Glycine max PE=2 SV=1

Accession: **tr|C6TFR0|C6TFR0_SOYBN**

Unused ProtScore: **5.42**

Seq Cov %: **8.3**

ITRAQ Changed Ratio:

117: 115=E3TL: D3TL=0.43

116: 114=E5TL: D5TL=0.58

Annotated typical Precursor MS Region:

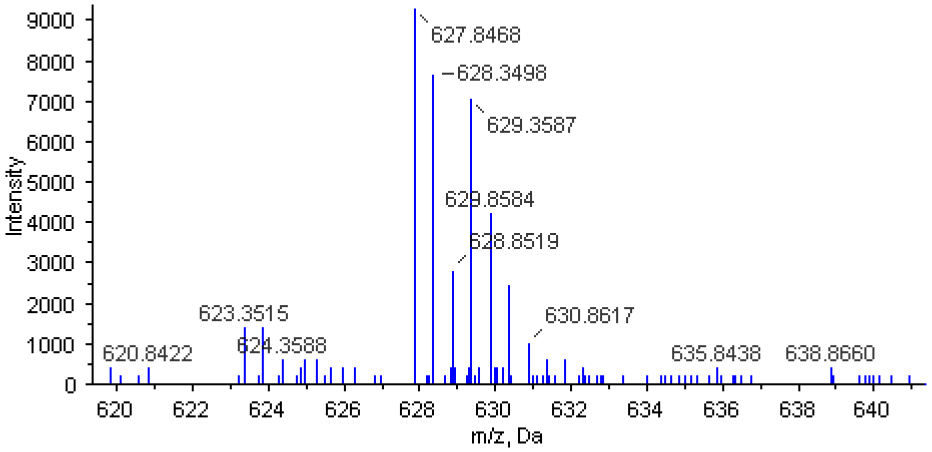

Sequence Coverage and Matched peptides: in different color

MTKQKIRNALLVHLVILFFFSTSVVSARVVSLLQNYGNPIFRSNFHSIYDTSKYGVFQLSNGLGKTPQM
GWNSWNFFACNINEMVIKETADALVSTGLADLGYVYVNIDDCWSSVTRNLKGQLVPDHKTFPSGIK**ALA**
DYVHGKGLKLGIYSDAGVFTCQVRPGSIFHETDDADLFASWGVDYLK**YDNCYNLGIPPK**ERYPPMRDAL
NATGQKIFYSLCEWGVEDPALWADKVGNSWRTTGDINDSWASMTTIADLNDKWAAYAGPGGWNDPDMLE
VGNGGMTYQEYRAHFSIWALAK**APLLIGCDVR**NLTGETLEILSNKEVIAINQDSLGVQGRKVQVSGADG
CRQVWAGPLSGNRLAVALWNRCSKVATIT

Protein No.: I-436

Protein name and Species:

Betaine-aldehyde dehydrogenase OS=Gossypium hirsutum PE=2 SV=1

Accession: tr|Q6S9W9|Q6S9W9_GOSHI

Unused ProtScore: 5.37

Seq Cov %: 7.2

ITRAQ Changed Ratio:

117: 115=E3TL: D3TL=0.65

116: 114=E5TL: D5TL=1.35

Annotated typical Precursor MS Region:

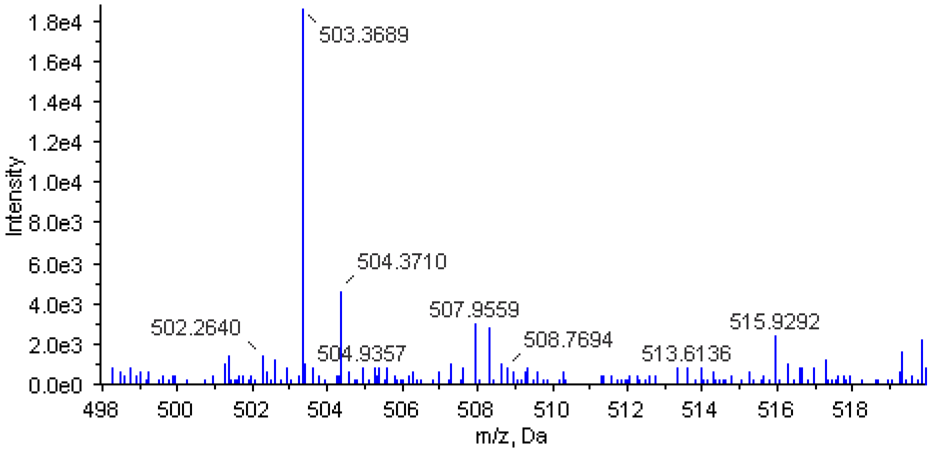

Sequence Coverage and Matched peptides: in different color

MAVQVPSRQLFIDGEWREPILKERLPTINPATEEIIGNIPAATAEDVELAVAAARRALSRNKGKDWATA
PGAVRAKYLRAIAAKVTERKTELAKLEAIDCGKPLDEAVWDIEDVAGCFEYYADLAEGLDARQKAPVSL
PMETFKSYVLKEPIGVVGLITPWNYPLLMATWKVAPSLAAGCAAILKPSELASITCLELAKVVKGSRLP
PGVLNILAGLGPEAGAPLASHPDVDKIAFTGSSATGSKIMAAAAQMVKPVSLELGGK**SPIIVFEDVDLD**
KAAEWTAFGCFWTNGQICSATSRLIVHENIAREFLDRLVKWTKNIKISDPFEEGCR**LGPVVSGGQYEK**V
LKFISTAKSEGATILSGGVRPEHLKKGFFVEPTIITDVTTSMQIWR**EEVFGPVLCVK**TFRTEEEALELA
NDTHYGLGAAVISNDLERCDRVSKNLQAGIVWVNCSQPCFCQAPWGGNKRSGFGRELGEWGLDNYLSVK
QVTQYVSDEPWGWYRSPSKL

Protein No.: I-437

Protein name and Species:

2-hydroxyphytanoyl-CoA lyase, putative OS=Ricinus communis GN=RCOM_0466960 PE=3 SV=1

Accession: **tr|B9SPZ1|B9SPZ1_RICCO**

Unused ProtScore: **5.35**

Seq Cov %: **12.4**

ITRAQ Changed Ratio:

117: 115=E3TL: D3TL=0.45

116: 114=E5TL: D5TL=0.61

Annotated typical Precursor MS Region:

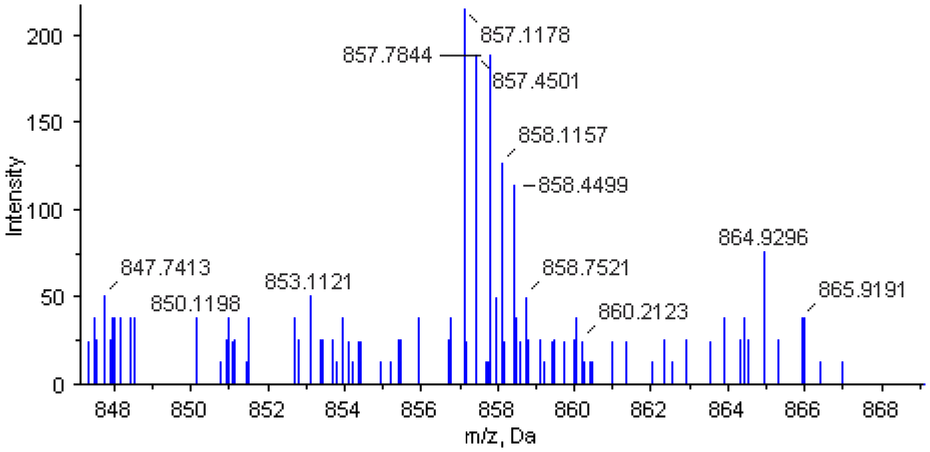

Sequence Coverage and Matched peptides: in different color

MADSNPQNAPESRIDGNILAAKSLARYGVTHMFGVVGIPVTSLANR**AVSLGIR**FVAFHNEQSAGYAASA
YGYLTGKPGLLLTVSGPGCVHGLAGLSNAMVNTWPMVMISGSCDQNDVGKGDFQELDQIEAVKPFVKFS
VKVKDIK**EIPNCVFQVLDR**AVSGRPGGCYLDIPSDVLHQTVDESEAESLLSDAFKPKSIVNEFNTKSSD
FERAAELLRTAER**PLIVFGK**GAAYARAESELKKLVELTGIPFLPTPMGKGILPDNHELAASAARSLAIG
K**CDVALVVGAR**LNWLLHFGQPPKWSKDVKFILVDVCEDEIELRKPHLGLVGDAKKVLEIINKEIKDDPF
CLAKNHPWVEAISSKVKENVSRMESQLAK**NVVPFNFLTPMR**IIR**DAILGVGSPAPIVVSEGANTMDVGR**
AVLVQTEPRTRLDAGTWGTMGVGLGYCIAAAVAEPNRLVVAVEGDSGFGFSAMEVETLVRYKLPVVVIV
FNNGGVYGGDRRSPEEIAGPFKDDPAPTSFVPGAAYHILIKAFGGKGYLVATPDELK**SALSESFSAREP**
TVINVTIDPYAGAESGRLQHKN

Protein No.: I-439

Protein name and Species:

Calcium-dependent protein kinase 5; CDPK OS=Arabidopsis thaliana GN=CPK5 PE=1 SV=1

Accession: sp|Q38871|CDPK5_ARATH

Unused ProtScore: 5.34

Seq Cov %: 9.4

ITRAQ Changed Ratio:

117: 115=E3TL: D3TL=2.58

116: 114=E5TL: D5TL=2.14

Annotated typical Precursor MS Region:

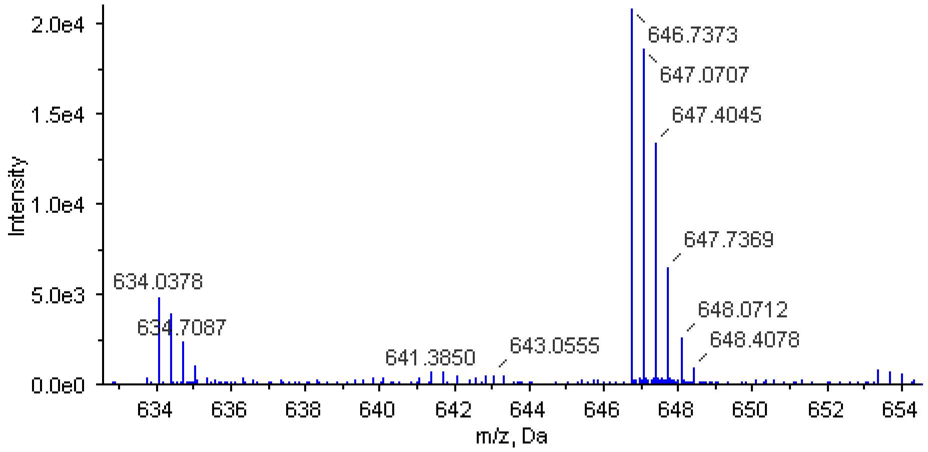

Sequence Coverage and Matched peptides: in different color

MGNSCRGSFKDKLDEGDNNKPEDYSKTSTTNLSSNSDHSPNAADIIAQEFSKDNNSNNNSKDPALVIPL
REPIMRRNPDNQAYYVLGHKTPNIRDIYTLSRKLGQGQFGTTYLCTEIASGVDYACKSISKRKLISKED
VEDVRREIQIMHHLAGHGSIVTIKGAYEDSLYVHIVMELCAGGELFDRIIQRGHYSERKAAELTK**IIVG**
VVEACHSLGVMHRDLKPENFLLVNK**DDDFSLK**AIDFGLSVFFKPGQIFTDVVGSPYYVAPEVLLKRYGP
EADVWTAGVILYILLSGVPPFWAETQQGIFDAVLKGYIDFESDPWPVISDSAKDLIRRMLSSKPAERLT
AHEVLRHPWICENGVAPDRALDPAVLSRLKQFSAMNKLKKMALK**VIAESLSEEEIAGLREMFQAMDTDN**
SGAITFDELKAGLRKYGSTLKDTEIHDLMDAADVDNSGTIDYSEFIAATIHLNKLEREEHLVAAFQYFD
KDGSGFITIDELQQACVEHGMADVF**LEDIIK**EVDQNNDGKIDYGEFVEMMQKGNAGVGRRTMRNSLNIS
MRDA

Protein No.: I-441

Protein name and Species:

O-linked n-acetylglucosamine transferase, ogt, putative OS=Ricinus communis GN=RCOM_1259290 PE=4 SV=1

Accession: tr|B9SX16|B9SX16_RICCO

Unused ProtScore: 5.34

Seq Cov %: 8

ITRAQ Changed Ratio:

117: 115=E3TL: D3TL=0.10

116: 114=E5TL: D5TL=0.39

Annotated typical Precursor MS Region:

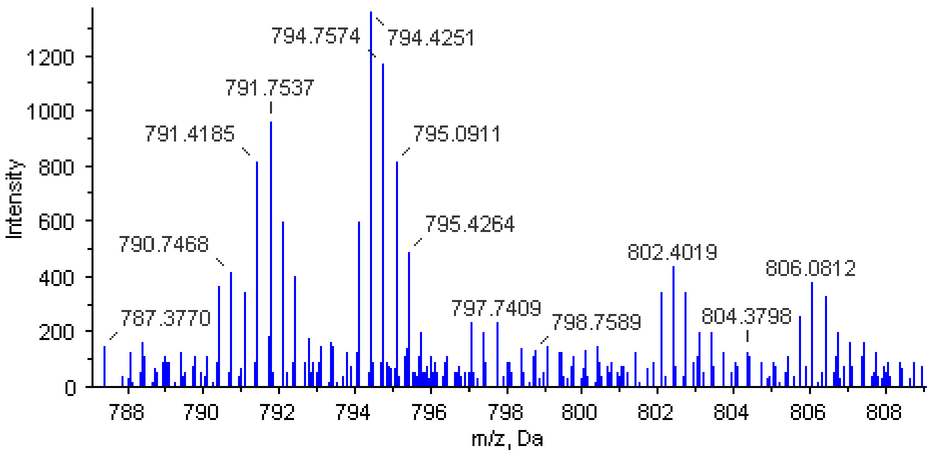

Sequence Coverage and Matched peptides: in different color

MAWTEKNNGNGKEGGPIEDNGFLKGTQEPSPSASGSPVAVAAGLKGIEEKDSLSYANILRSRNK**FVDAL**
AIYESVLEKDSGNVEAYIGKGICLQMQNMGRLAFDSFAEAIK**LDPQNACALTHCGILYK**EEGRLVEAAE
SYQKALRADPLYKPAAECLSIVLTDLGTSLK**LSGNTQEGIQK**YYEALKIDPHYAPAYYNLGVVYSEMMQ
YDTALNCYEKAALERPMYAEAYCNMGVIYKNR**GDLESAIACYER**CLAVSPNFEIAKNNMAIALTDLGTK
VKLEGDINQGIAYYKKALYYNWHYADAMYNLGVAYGEMLKFDNAIVFYELAFHFNPHCAEACNNLGVIY
KDRDNLDKAVECYQTALSIKPNFSQSLNNLGVVYTVQGKMDAAASMIEK**AIMANPTYAEAYNNLGVLYR**
DAGNIPMAINAYEQCLKIDPDSRNAGQNRLLAMNYINEGHDEKLFEAHRDWGRRFMRLYPQYTMWDNPK
DLDRPLVIGYVSPDYFTHSVSYFIEAPLVYHDYANYKVVVYSAVVKADAKTIRFREKVLKQGGIWRDIY
GIDEKKVASMVREDNVDILVELTGHTANNKLGMMACRPAPIQVTWIGYPNTTGLPTIDYRITDSLADPR
DTKQKHVEELVRLPDCFLCYTPSPEAGPVCPTPALANGFITFGSFNNLAKITPKVLQVWARILCAVPNS
RLVVKCKPFCCDSVRQRFLTMLEELGLESLRVDLLPLILLNHDHMQAYSLMDISLDTFPYAGTTTTCES
LYMGVPCVTMAGAIHAHNVGVSLLSKVGLGHLVAQNEDNYVQLALQLASDIPALSNLRMSLRDLMSKSP
VCDGSKFTLGLESSYRDMWHRYCKGDVPSLKRMELLKQQKGSEAVPNENFEPTRNAFPVEGPPESVKLN
GYNIVSSSILNRSSEENVSQTQLNHTTNSDKPS

Protein No.: I-447

Protein name and Species:

Triosephosphate isomerase OS=Vitis vinifera GN=VIT_13s0019g01090 PE=3 SV=1

Accession: **tr|D7TLU7|D7TLU7_VITVI**

Unused ProtScore: **5.22**

Seq Cov %: **16.1**

ITRAQ Changed Ratio:

117: 115=E3TL: D3TL=0.21

116: 114=E5TL: D5TL=0.17

Annotated typical Precursor MS Region:

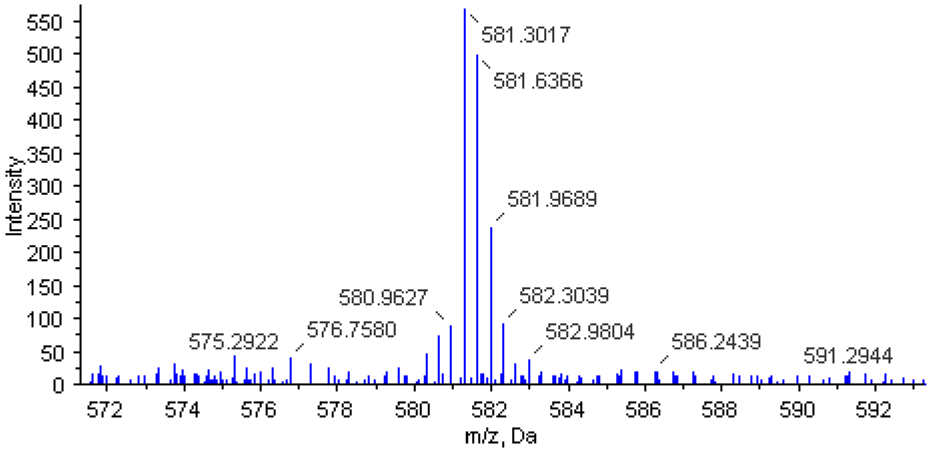

Sequence Coverage and Matched peptides: in different color

MGRKFFVGGNWKCNGTGEEVKKIVSTLNAGEVPSGDVVEVVVSPPFVFLPLVKSTLRPDFHVAAQNCWV
KKGGAFTGEISAEMLVNLGIPWVIIGHSERRLLLNESNEFVGEKVAYALSKGLKVIACVGETLEQRESG
STMEVVAAQTKAIADKVSNWANVVLAYEPVWAIGTGKVATPAQAQEVHSELRNWFQANASPEVAATIRI
IYGGSVSGANCKELAAKPDVDGFLVGGASLKPEFIDIIKSAEVKKNC

Protein No.: I-448

Protein name and Species:

Serine-threonine kinase receptor-associated protein, putative OS=Ricinus communis GN=RCOM_0897280 PE=4 SV=1

Accession: **tr|B9RUZ3|B9RUZ3_RICCO**

Unused ProtScore: **5.22**

Seq Cov %: **17.3**

ITRAQ Changed Ratio:

117: 115=E3TL: D3TL=9.73

116: 114=E5TL: D5TL=2.08

Annotated typical Precursor MS Region:

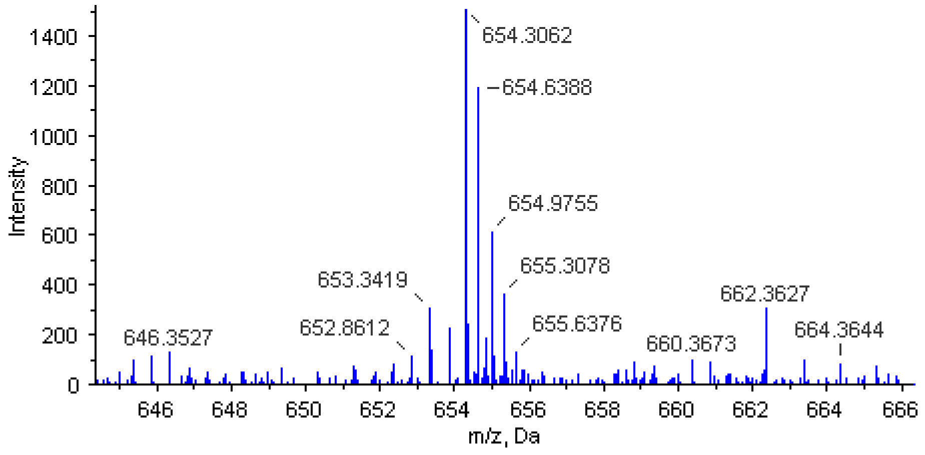

Sequence Coverage and Matched peptides: in different color

MDKKKVAVPLVCHGHSRPVVDLFYSPVTPDGFFLISASKDSSPMLRNGETGDWIGTFEGHKGAVWSCSL
DTKALRAASASADFTAKLWDALTGDELHSFEHRHIVRACAFSEDTHLLLTGGMEKILRLFDLNRPDAPP
REVNSSPGSIRTVAWLHSDQTILSSCTDMGGVRLWDIRSGKIVQTLETKSPVTSAEVSQDGRYITTADG
STVKFWDANHFGLVKSYNMPCNVESASLEPKLGNKFVAGGEDMWIHVFDFHTGEQIGCNKGHHGPVHCL
RFSPGGESYASGSEDGTIRIWQTGPQNHDENGPTGKAMASADDVTQKIKGKGGKTAGTDP

Protein No.: I-449

Protein name and Species:

40S ribosomal protein OS=Populus trichocarpa GN=POPTRDRAFT_814470 PE=2 SV=1

Accession: tr|A9PGL4|A9PGL4_POPTR

Unused ProtScore: 5.22

Seq Cov %: 21.7

ITRAQ Changed Ratio:

117: 115=E3TL: D3TL=1.56

116: 114=E5TL: D5TL=1.71

Annotated typical Precursor MS Region:

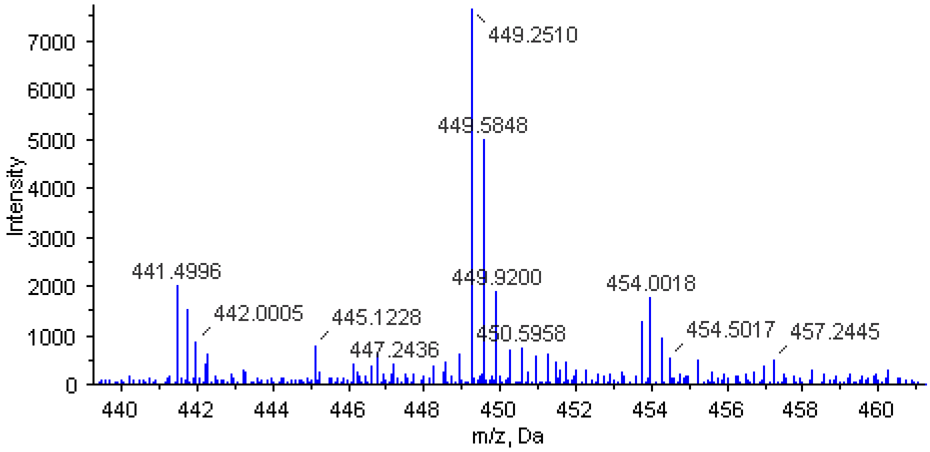

Sequence Coverage and Matched peptides: in different color

MATAKSVK**DVSPHEFVK**AYSAHLKRSGKIELPLWTDIVKTGKFK**ELAPYDPDWYYVR**AASMARKIYIRG
GLGVGAFRRIYGGSKRNGSRPPHFCKSSGSIARHILQQLQNVNIIDIDPKGGRRITSSGQRDLDQVAGR
IVVAP

Protein No.: I-453

Protein name and Species:

quinone oxidoreductase, putative OS=Populus trichocarpa GN=POPTRDRAFT_826614 PE=4 SV=1

Accession: **tr|B9MU39|B9MU39_POPTR**

Unused ProtScore: **5.19**

Seq Cov %: **20.4**

ITRAQ Changed Ratio:

117: 115=E3TL: D3TL=0.38

116: 114=E5TL: D5TL=0.53

Annotated typical Precursor MS Region:

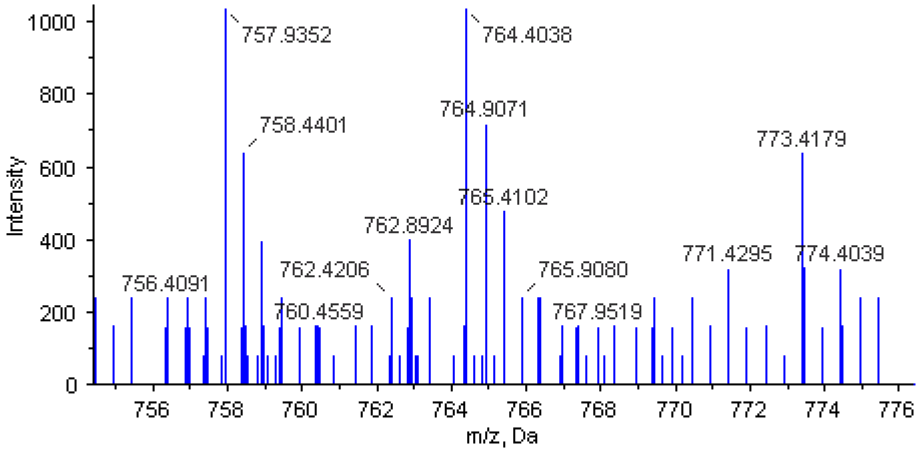

Sequence Coverage and Matched peptides: in different color

MVKAIR**VHELGGPEVLK**WEDVEIGEPGEGEIRVKNKAIGLNFIDVYYRKGVYTASTMPFIPGREAVGEV
IAVGPGLTGRKVGDVVAYAGDPMGSYAEEQILPANKVVSVPESISPVIAASVMLKGMTTQFLLRRCFKV
EPGHTILVHAAAGGVGSLLCQWANALGATVIGTVSTKEKAAQAKEDGCQHVILYQEEDFVARVNEITSC
NGVDVVYDSVGK**DTFQGSLACLK**TR**GYMVSFGQSSGTPDPVPLSALAPK**SLFLTRPSMMHYNQTRDELL
GAAGELFSALASGVLRVR**VNHTYPLSQAAQAHADIESR**KTTGSVVLIP

Protein No.: I-456

Protein name and Species:

Beta-1,3-glucanase OS=Hevea brasiliensis GN=Glu I PE=4 SV=1

Accession: tr|Q7XJ83|Q7XJ83_HEVBR

Unused ProtScore: 5.16

Seq Cov %: 59.1

ITRAQ Changed Ratio:

117: 115=E3TL: D3TL=0.13

116: 114=E5TL: D5TL=0.65

Annotated typical Precursor MS Region:

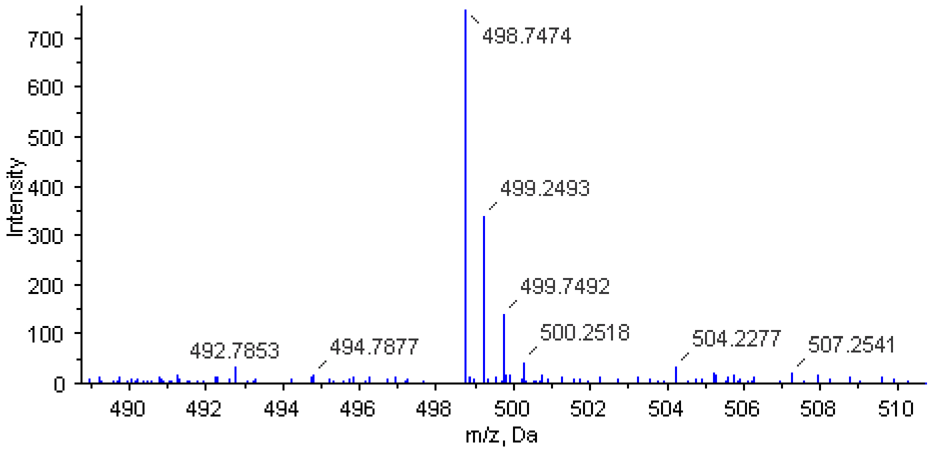

Sequence Coverage and Matched peptides: in different color

MAISSSTSGTSSSLPSRTTVMLLLIFFTASLG**ITDAQVGVCYGMQGNNLPPVSEVIALYK**QSNIKRMR**I**
YDPNRAVLEALRGSNIELILGVPNSDLQSLTNPSNANSWVQKNVR**GFWSSVLFR**YIAVGNEISPVNG**GT**
AWLAQFVLPAMRNIHDAIRSAGLQDQIKVSTAIDLTLVGNSYPPSAGAFRDDVR**SYLDPIIGFLSSIRS**
PLLANIYPYFTYAGNPRDISLPYALFTSPSVVVWDGQRGYK**NLFDATLDALYSALER**ASGGSLEVVVSE
SGWPSAGAF**AATFDNGRTYLSNLIQHVKGG**TPKRPNR**AIETYLFAMFDENKKQPEVEKHFGLFFPDK**RP
K**YNLNFGAEK**NWDISTEHNAT**ILFLK**SDM

Protein No.: I-459

Protein name and Species:

ATP-citrate synthase beta chain OS=Populus trichocarpa GN=POPTRDRAFT_656642 PE=4 SV=1

Accession: tr|B9HIN6|B9HIN6_POPTR

Unused ProtScore: 5.11

Seq Cov %: 38.7

ITRAQ Changed Ratio:

117: 115=E3TL: D3TL=0.47

116: 114=E5TL: D5TL=0.54

Annotated typical Precursor MS Region:

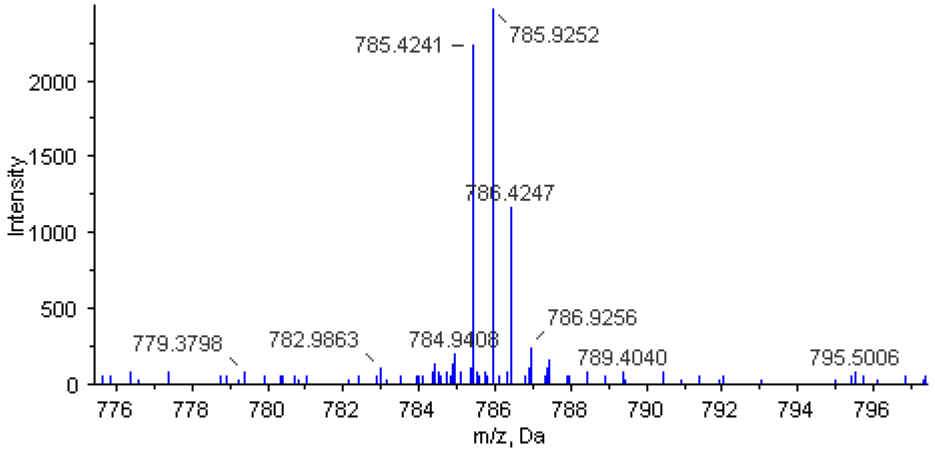

Sequence Coverage and Matched peptides: in different color

MATGQLFSR**TTQALFYNYK**QLPIQR**MLDFDFLCGRELPSVAGIINPGAEGFQK**LFFGQEEIAIPVHSTI
EAACAAHPTADVFINFASFRSAAASSMSALKQPTVR**VVAIIAEGVPEADAK**QLIAYARANNK**VVIGPAT**
VGGIQAGAFKIGDTAGTIDNIIACKLYRPGSVGFVSKSGGMSNELYNSIARVTDGIYEGIAIGGDVFPG
STLSDHVLRFNNIPQVKMIVVLGELGGR**DEYSLVEALK**QGKVSKPVVAWVSGTCARLFK**SEVQFGHAGA**
KSGGEMESAQAKNQALK**DAGAVVPTSYEAFETAIK**ETFEKLVEEGKISSVKEFTPPQIPEDLNIAIKSG
KVR**APTHIISTISDDR**GEEPCYAGVPMSSIVEQGYGVGDVISLLWFKRSLPRYCTHFIEICIMLCADHG
PCVSGAHNTIVTARAGK**DLVSSLVSGLLTIGPRFGGAIDDAAR**YFKDAYDR**GLTPYEFVESMK**KKGIRV
PGIGHRIKRGDNRDK**RVELLQLFAR**THFPSVK**YMEYAVQVETYTLSK**ANNLVLNVDGAIGSLFLDLLAG
SGMFTKQEIDEIVGIGYLNGLFVLAR**SIGLIGHTFDQK**RLKQPLYRHPWEDVLYSK

Protein No.: I-467

Protein name and Species:

COP9 signalosome complex subunit 1 [Vitis vinifera] OS=Vitis vinifera GN=VIT_15s0048g01980 PE=4 SV=1

Accession: tr|D7U7I9|D7U7I9_VITVI

Unused ProtScore: 4.99

Seq Cov %: 7.7

ITRAQ Changed Ratio:

117: 115=E3TL: D3TL=1.59

116: 114=E5TL: D5TL=1.10

Annotated typical Precursor MS Region:

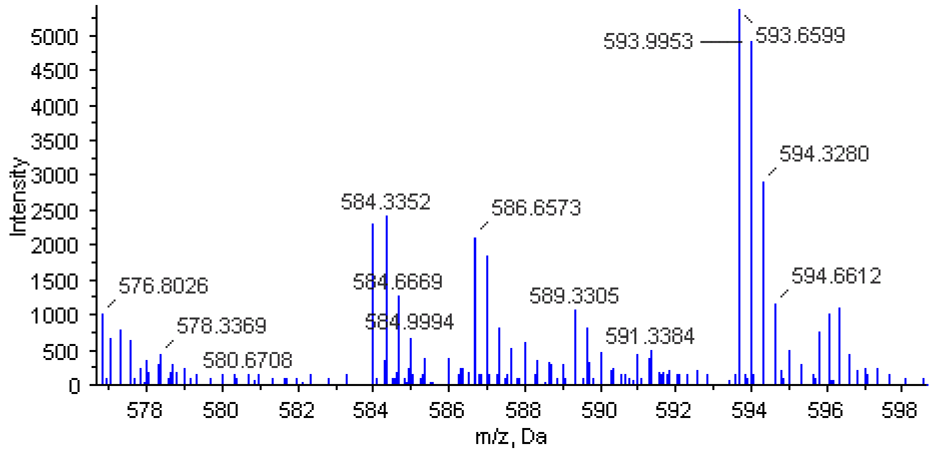

Sequence Coverage and Matched peptides: in different color

MEGEEESSAGAMIDEIYSNGGDAKRNRPIISGEALDIEAYASLYSGRTKITRLLFIADRCENRQMQMEA
YRMAYDEIKKGENTQLYREVVQKIDGRLGPQYGLDLVWTESVERRADQRKEK**LENELNAYR**TNLIKESI
RMGYNDFGDFYYAHGLLADAFKNYVRTRDYCTTSKHIIHMCLNAILVSIEMGQFTHVTSYVSKAEQTPE
ALDPVTVAKLR**CAAGLAHLEAK**KYKLAARKFLETGAELGNNYTEVIAPQDVATYGGLCALASFDRTELK
NKVIDNLNFR**NFLELVPEVR**ELIHDFYSSHYASCLDYLGNLKTNLLLDIHLHDHVEMLYNQIRHKALIQ
YTHPFVSVDLRMMANAFKTSVAGLEK**ELEALITDNQIQAR**IDSHNKILYARHADQRNATFQRVLQTGNE
FDRDVRAMLLRANLLKHEYNLRASRKP

Protein No.: I-475

Protein name and Species:

Amidase, putative OS=Ricinus communis GN=RCOM_1431340 PE=4 SV=1

Accession: tr|B9RF45|B9RF45_RICCO

Unused ProtScore: 4.89

Seq Cov %: 5.6

ITRAQ Changed Ratio:

117: 115=E3TL: D3TL=0.60

116: 114=E5TL: D5TL=0.64

Annotated typical Precursor MS Region:

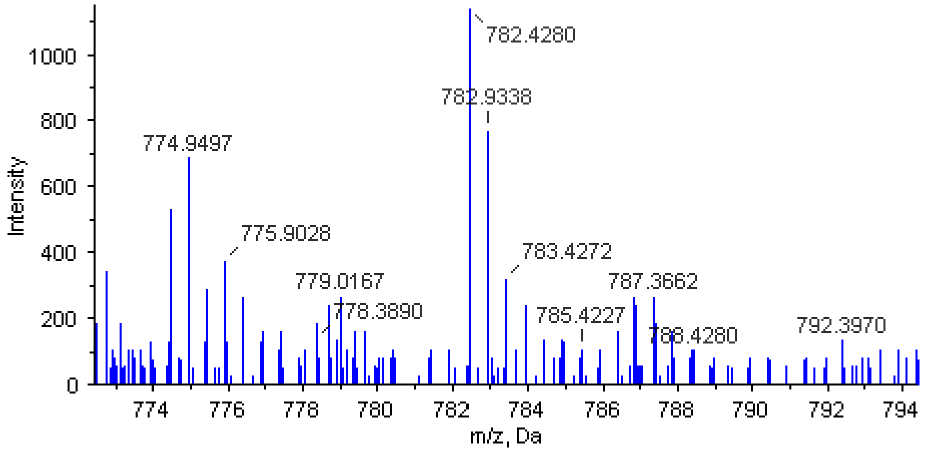

Sequence Coverage and Matched peptides: in different color

MTHQFYELAITLCCSVLSDSNQSPACQWIKGWPTENDQIVLWKIGRPAKAACQIDRESWDRAQSLTVRA
VTDMAISPDYGAFMEKFVLKPSSSSDQLPLHSLTFAVKDIFDVDGYVTGFGNPDWARTHSAATATAPAV
LAVLRGGATCVGKVIMDEMAYSINGENAHYGTPTNPSAPDR**VPGGSSSGSAVAVGAK****LVDFSLGSDTGG**
SVRVPASYCGILGFRPSHGVVSVSGVIPMAQSFDTVGWFSRNPVILKHVGRLLLQLPDVDRTKPNQIFI
AEDCFQLSSIPKK**RVSQVLVKSVEKLFGGDIVK**HVNLGDYVEDKVPSLRHFMSKEIKEQEYNIAPLAAL
SSAMRMLQRYEFKTNHGEWVTTVKPDFGPGLKERIWEAVRSTEENIDAFQSVRTELRAALTNLLEEFGI
IAIPTVPGVPPK**LNTDPTTLEIFR**AKAFSLLSITGVSGFCQVSIPLGMHDGLPVSISLLAKHGSDVFLL
NVVETLYDTLQEEVCNI

Protein No.: I-478

Protein name and Species:

Ligatin, putative OS=Ricinus communis GN=RCOM_1045170 PE=4 SV=1

Accession: **tr|B9SBV7|B9SBV7_RICCO**

Unused ProtScore: **4.86**

Seq Cov %: **5.2**

ITRAQ Changed Ratio:

117: 115=E3TL: D3TL=0.52

116: 114=E5TL: D5TL=1.06

Annotated typical Precursor MS Region:

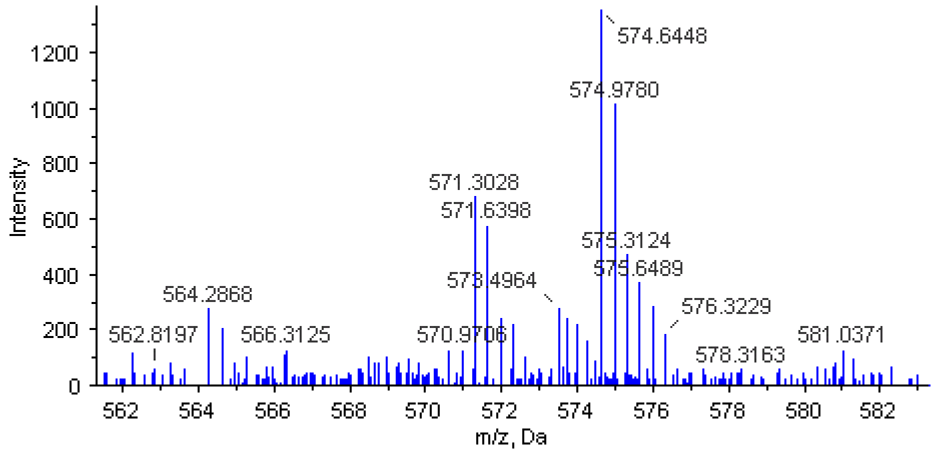

Sequence Coverage and Matched peptides: in different color

MFKKAVEAKSHQRLSGADRKKLKRSIRDKFPRASDADIDTLLPPKADITVAKFQNRVHVYGVEGGFPMF
FDVDGRGTDIFPTVFALWNVPEFLPSFMLKGGEVSRYILGGADLMFPGISIPAEGLPSFSAGEPWAVKV
PGNPAPIAVGSTTMSSSEAIKAGLRGKALRITHYYRDLLWESVEGHYVPNAGFFEDVVFEDPAFTSSIQ
VSDSYETAYDVSNDQLNKVETDEIGESADVNDVISESHPSTITELDSKESKHDIIEQVTAVVSDMKVMD
NDTADESTIEEQHILSTGDVDAYLDKCLLQALHTTVKDKDLPMPGSTLWSNHVLPCRPPGVLLDIKKSS
HKKLSKWLQAKSSAGLISVKEDKYKKETVLISINRSHPDYLSFKPEKRQEQKIDQAGDRATSESQSKKM
LEVAEIYKPSVHVNPIFASVGADTGKLYTASEASDIVFNYIEKENLVKPTNKSFVVLDATLCDALFKGT
VKK**GSTYPTEIHK**KDLGSIFISR**MQAHYIVTR**GSDSAIRKGSLKTIQILTERRQGNKKVTKVSGLEQFL
MDAEALASELQKK**FACSTSVQELPGK**KGNEVLIQGGVIDDVAR**HLVEQYGVPK**R**YIEVLDK**TKK

Protein No.: I-481

Protein name and Species:

Steroid dehydrogenase, putative OS=Ricinus communis GN=RCOM_1082860 PE=3 SV=1

Accession: tr|B9RMR2|B9RMR2_RICCO

Unused ProtScore: 4.82

Seq Cov %: 5.9

ITRAQ Changed Ratio:

117: 115=E3TL: D3TL=3.40

116: 114=E5TL: D5TL=1.38

Annotated typical Precursor MS Region:

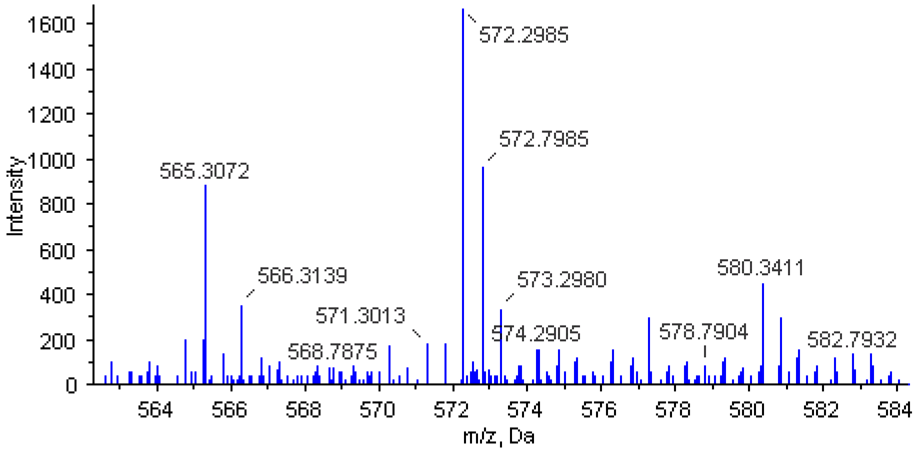

Sequence Coverage and Matched peptides: in different color

MELCFVDKLKSQPLWFLVLFSLGSLSLLRCFFTILNWVYYVNFLRPAKNLKKYGSWALVTGPTDGIGKGF
AFQLARKGLNLVLVARNPDKLKDVSDSILSKYGKVQIKTVVVDFSGDIDEGVKRIKDTVEGLDVGVLIN
NVGVSYPYARFFHEVDEELLKNLIKVNVEGTTKVTHAVLPGMLKRKKGAIINIGSGAAIVIPSDPLYAV
YAATKAYIDQFSRCLYVEYKKSGIDVQCQVPLYVATKMASIRRSSFFVPSTDDYARAGLRWVGYEPRCT
PYWPHSLIWGLISIVPEFAVDAWRYKFNLGIRKRGQQKDARKKE

Protein No.: I-482

Protein name and Species:

Importin alpha-2 subunit, putative OS=Ricinus communis GN=RCOM_0819430 PE=4 SV=1

Accession: tr|B9SGQ4|B9SGQ4_RICCO

Unused ProtScore: 4.81

Seq Cov %: 11.6

ITRAQ Changed Ratio:

117: 115=E3TL: D3TL=0.61

116: 114=E5TL: D5TL=0.56

Annotated typical Precursor MS Region:

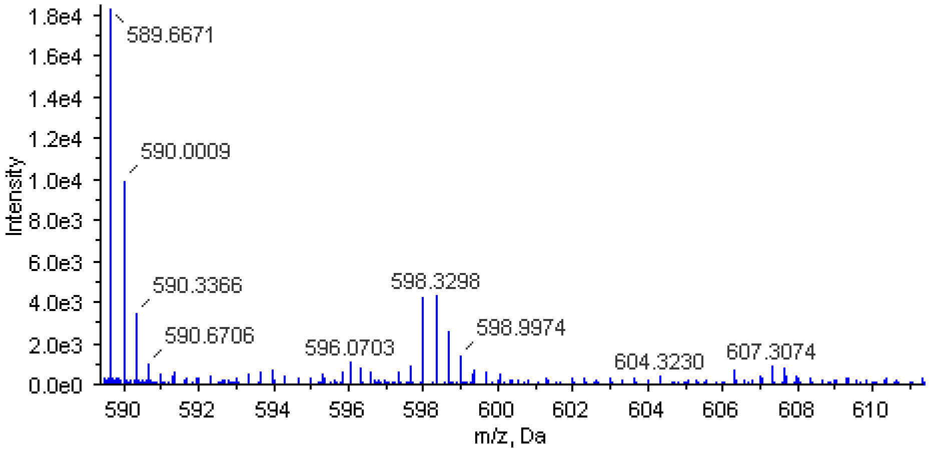

Sequence Coverage and Matched peptides: in different color

MAEEGLTSLKREPIKSSVGNVAAQKRKQNAVAVGKERRESLVRAKRLCRVGTSGDSDSEMIIDEEQSIL
DAQTSSAVEELKSAVAFQGKGAMQKKVGALRELRRLLSRSEFPPIEAAIKAGAIILLVQCLSFGSPDEQ
LLEAAWCLTNIAAGKPEETKALLPALPLLIAHLGERSSSPVAEQCAWALGNVAGEGEDLR**NVLLSQGAL**
PPLARMMLPNRGSTVR**TAAWALSNLIK**GPDPKAATELIRVDGVLDAIVRHLRKADEELATEVAWAVVYL
SALSNVATSMLVKSDVLQLLIQR**LATSNSLQLLIPVLR**SLGNLIAGDSHTISAVLLPGIEITDSIIAVL
VKCLKSEHRVLKKEAAWVLSNIAAGSVEHKQLIYCSEAVPLLLR**LLSTAPFDLR**KEVAYVLGNLCVAPA
EGTGKPDLILEHLVSLVSRGCLPGFIDLVRSVDTEAARLGLQFIELVLRGMPNGEGPKLVEQENGIEAM
ER**FQFHENEDLR**NMANGLVDRYFGEDYGLTELTD

Protein No.: I-485

Protein name and Species:

Adenylyl cyclase-associated protein OS=Populus trichocarpa GN=POPTRDRAFT_722043 PE=3 SV=1

Accession: tr|B9HRN7|B9HRN7_POPTR

Unused ProtScore: 4.79

Seq Cov %: 8.8

ITRAQ Changed Ratio:

117: 115=E3TL: D3TL=0.58

116: 114=E5TL: D5TL=0.72

Annotated typical Precursor MS Region:

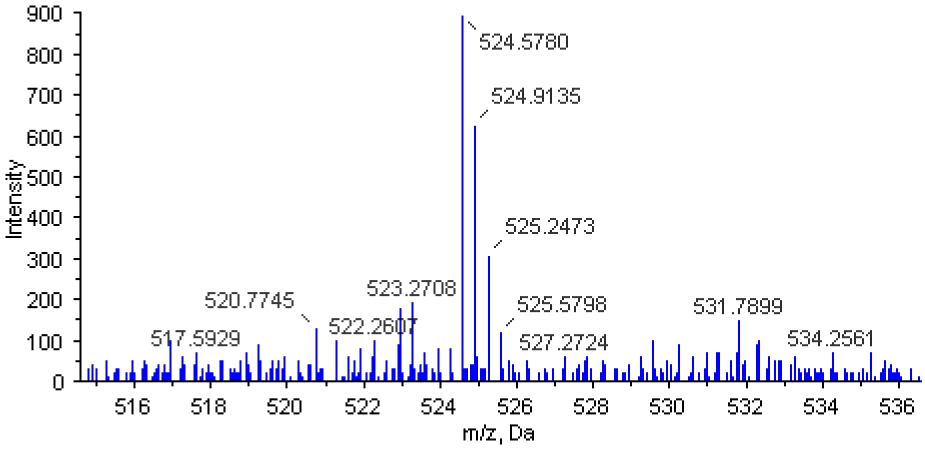

Sequence Coverage and Matched peptides: in different color

MEEKLIERLESAVARLEALSLRSGGLALSGGDDSAATDPSIVAFDDFIGTFFVRVSSAAEKIGGQVLEV
TRILEQALNVQKELLIKIKQTRKPDLAGLAEFLKPLNEVIMKANAMTEGRRSDFFNHLKAAADSLTALA
WIAYTGKDCGMSMPIAHVEESWQMAEFYSNKILVQYKSKDPNHVEWAKALKDLYLPGLRDYVKSHYPLG
PVWSTTGKATASAPSKAPAPPPPPPASLFSSESSQPSSSKPKEGMAAVFQEISSGKSVTTGSLKKVTDD
MKTKNRADRTGVVAVTEKGGRASAPSFSKAGPPK**LELQMGR**KWVVENQIGKKNLVIDDCDAK**QSVYVFG**
CKDSVLQIQGKVNNITIDKCTKMGVVFTDVVAACEIVNCTGVEVQCQGSAPTISVDNTGGCQLYLSK**DS**
LGASITTAKSSEINVLVPGAEPGGDLAEHALPQQFIHAFK**DGQFETTPVSHSGG**

Protein No.: I-487

Protein name and Species:

Mevalonate kinase, putative OS=Ricinus communis GN=RCOM_1431350 PE=3 SV=1

Accession: **tr|B9RF46|B9RF46_RICCO**

Unused ProtScore: **4.74**

Seq Cov %: **10.6**

ITRAQ Changed Ratio:

117: 115=E3TL: D3TL=0.66

116: 114=E5TL: D5TL=0.89

Annotated typical Precursor MS Region:

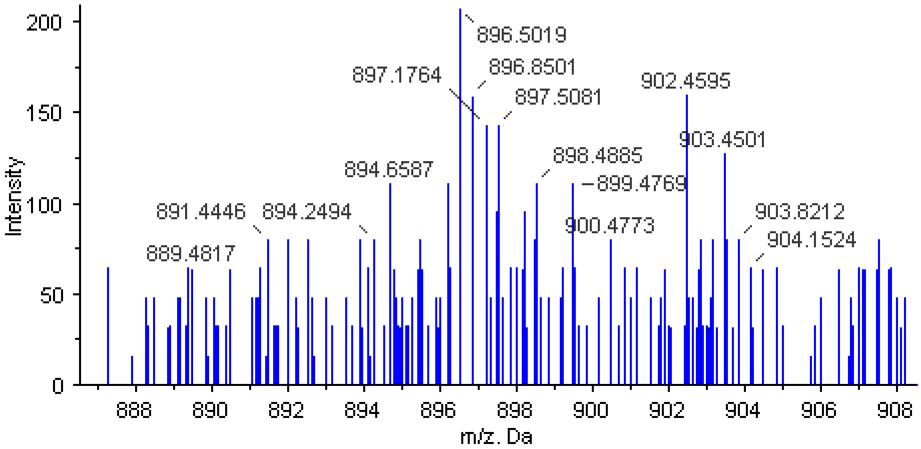

Sequence Coverage and Matched peptides: in different color

MEVKARAPGKIILSGEHAVVHGSTAVAAALDLYTYVSISFPSSGNDGTLKLQLKDLALEFSWPVAKIRE
ALPNLGVPSSSTPTSCSIESLKSISALVEEQDIPEAKIALASGVSAFLWLYTSIQGFEPATVVVTSDLP
LGSGLGSSAAYCVSLSAALLAFSDSVDVDRGHQGWLKFGVPDLELLNKWAFEGEKIIHGK**PSGIDNTVS**
TYGNMIKFRSGNLTRMKSNMPLKMLITNTRVGRNTK**ALVAGVSER**TFRHPNAMSFVFNAVDSISNELAN
IIQSPAPDELSITEKEEMLEELMEMNQGLLQCMGVSHASIETVLQTTLKYKLASK**LTGAGGGGCVLTLL**
PTLLSGTVVDKVIAELESSGFQCLIAGIGGDGAEICFASSS

Protein No.: I-498

Protein name and Species:

Heat shock protein 90-2 OS=Glycine max PE=2 SV=1

Accession: tr|B6EBD6|B6EBD6_SOYBN

Unused ProtScore: 4.69

Seq Cov %: 30.4

ITRAQ Changed Ratio:

117: 115=E3TL: D3TL=0.10

116: 114=E5TL: D5TL=0.44

Annotated typical Precursor MS Region:

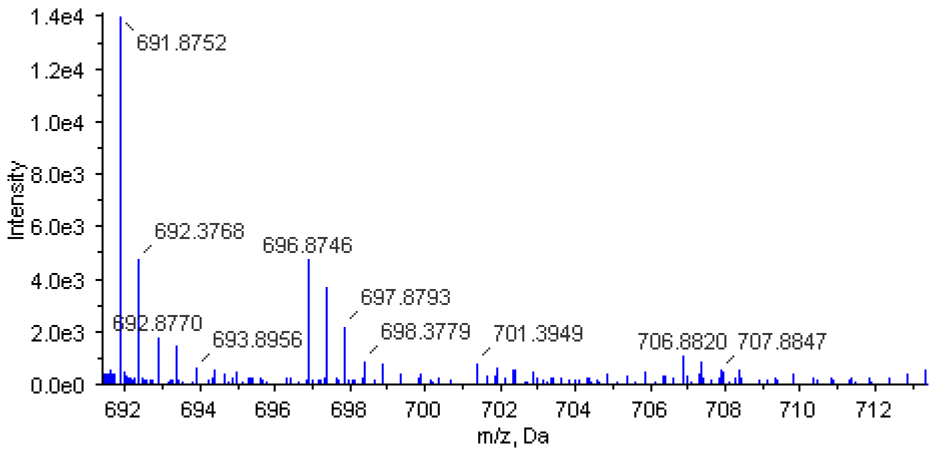

Sequence Coverage and Matched peptides: in different color

MASETETFAFQAEINQLLSLIINTFYSNKEIFLRELISNASDALDKIRFESLTDKSKLDAQPELFIHII
PDKTNNTLSIIDSGIGMTKADLVNNLGTIARSGTKEFMEALAAGADVSMIGQFGVGFYSAYLVADKVIV
TTKHNDDEQYVWESHAGGSFTVTRDTSGENLGRGTKITLFLKEDQLEYLEERRLKDLIKKHSEFISYPI
SLWIEKTTEKEISDDEDEEEKKDEEGKVEDVDEDKEKEEKKKKTIKEVSHEWSLVNKQKPIWMRKPEEI
TKEEYAAFYKSLTNDWEEHLAVKHFSVEGQLEFKAVLFIPKRAPFDLFDTKKKPNNIKLYVRRVFIMDN
CEELMPEYLSFVKGIVDSEDLPLNISREMLQQNKILKVIRKNLVKKCIEMFFEIAENKEDYNKFYEAFS
KNLKLGIHEDSQNKTKLAELLRYHSTKSGDEMTSLKDYVTRMKEGQNDIYYITGESKKAVENSPFLEKL
KKKGFEVLYMVDAIDEYAVGQLKEFEGKKLVSATKEGLKLDESEDEKKKKEELKEKFEGLCHVIKDVLG
DKVEKVVVSDRVVDSPCCLVTGEYGWTANMERIMKAQALRDSSMAGYMSSKKTMEINPENPIMEELRKR
ADADKNDKSVKDLVLLLFETALLTSGFSLDDPNTFGNRIHRMLKLGLSIDEDAGEADADMPPLEDADAD
AEGSKMEEVD

Protein No.: I-503

Protein name and Species:

isoflavone reductase related protein OS=Populus trichocarpa PE=2 SV=1

Accession: tr|A9PF66|A9PF66_POPTR

Unused ProtScore: 4.65

Seq Cov %: 4.9

ITRAQ Changed Ratio:

117: 115=E3TL: D3TL=0.60

116: 114=E5TL: D5TL=0.69

Annotated typical Precursor MS Region:

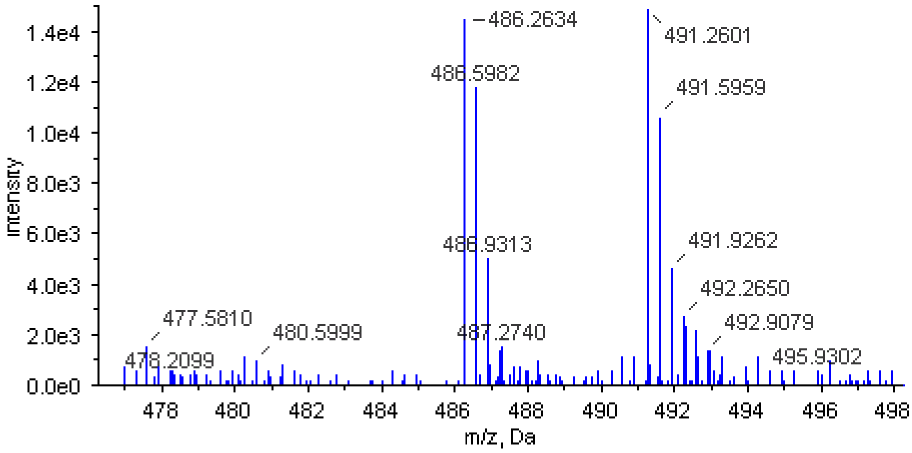

Sequence Coverage and Matched peptides: in different color

MASKILFIGGTGYIGKFIVEASAKAGHPTFVLVRESTLSNPAKSVVIDNFKNLGVNFLIGDLFDHESLV
KAIKQVDVVISTVGHAQLVEQDRIIAAIKEAGNVKRFFPSEFGNDVDRVNAVEPAKSAFATKANVRRAI
EAEGIPYTYVSSNFFSGYFLLSFNQPGATAPPRDKVVILGDGNPKAVFNKEDDIATYTIKAVDDPRTLN
KILYIKPPANTISFNDLVSLWEKKIGKTLERIYVPEEQLLKNIQEASVPVNVVLSIGHSVFVKGDHTNF
EIEPSFGVEASELYPDVKYTTVDEYLKQFV

Protein No.: I-504

Protein name and Species:

Superoxide dismutase (Fragment) OS=Hevea brasiliensis GN=sod PE=2 SV=1

Accession: tr|Q9STB5|Q9STB5_HEVBR

Unused ProtScore: 4.65

Seq Cov %: 13.2

ITRAQ Changed Ratio:

117: 115=E3TL: D3TL=0.44

116: 114=E5TL: D5TL=0.57

Annotated typical Precursor MS Region:

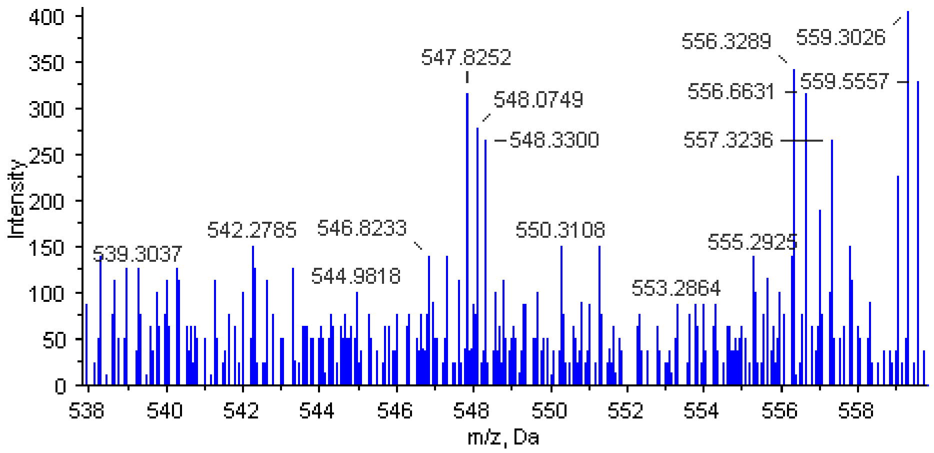

Sequence Coverage and Matched peptides: in different color

QTFSLPDLPYDYGALEPAISGEIMQLHHQKHHQTYITNYNK**ALEQLNDAIEK**GDSAAVVKLQSAIKFNG
GGHVNHSIFWKNLAPVREGGGELPHGSLGWAIDADFGSLEKLIQLMNAEGVALQGSGWVWLALDKELK**K**
LVVETTANQDPLVTKGPTLVPLLGIDVWEHAYYLQYKNVRPDYLKNIWKVMNWKYASEVYAKECPSS

Protein No.: I-506

Protein name and Species:

Cysteine synthase OS=Glycine max PE=2 SV=1

Accession: tr|C6TIP9|C6TIP9_SOYBN

Unused ProtScore: 4.63

Seq Cov %: 16.6

ITRAQ Changed Ratio:

117: 115=E3TL: D3TL=0.63

116: 114=E5TL: D5TL=0.70

Annotated typical Precursor MS Region:

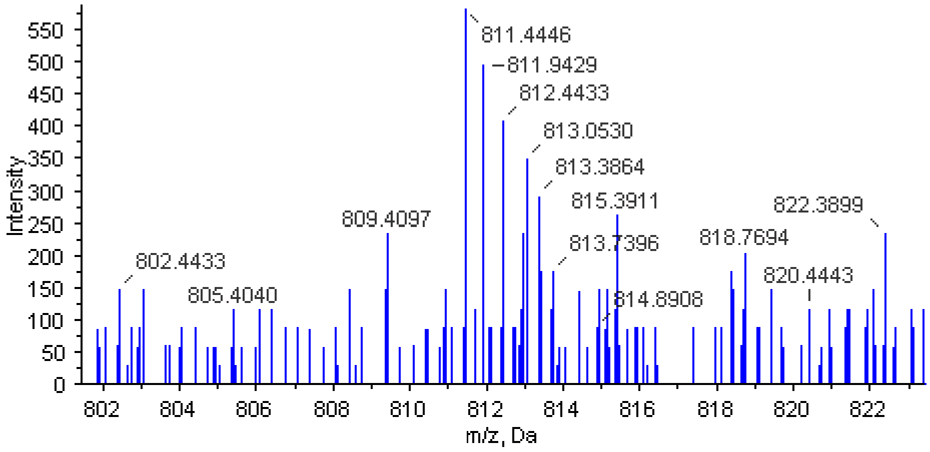

Sequence Coverage and Matched peptides: in different color

MAAEKSNIAK**DVTELIGK**TPLVYLNHIVDGCVAKVAAK**LEMMEPCSSVK**DRIGHSMIVDAEEKGLITPG
ESVLIEPTSGNTGIGLAFMAAAKGYK**LIITMPSSMSLER**RTILRAFGAELVLTDPAKGMKGAVQKAEEI
RDKTPNSYMLQQFENPTNPKVHYETTGPEIWKGSSGK**VDALVSGIGTGGTVTGAGKYLK****EQNPDIK**LYG
IEPVESPILSGGKPGPHKIQGIGAGFIPGVLDVDLLDEVVQISSEEAIETAK**LLALK**EGLLVGISSGAA
AAAAVKIAK**RPENAGK**LVVAVFPSFGER**YLSSVLFESVK**REAESLVFEP

Protein No.: I-510

Protein name and Species:

Fimbrin, putative OS=Ricinus communis GN=RCOM_0549750 PE=4 SV=1

Accession: tr|B9T216|B9T216_RICCO

Unused ProtScore: 4.59

Seq Cov %: 15.8

ITRAQ Changed Ratio:

117: 115=E3TL: D3TL=1.54

116: 114=E5TL: D5TL=1.43

Annotated typical Precursor MS Region:

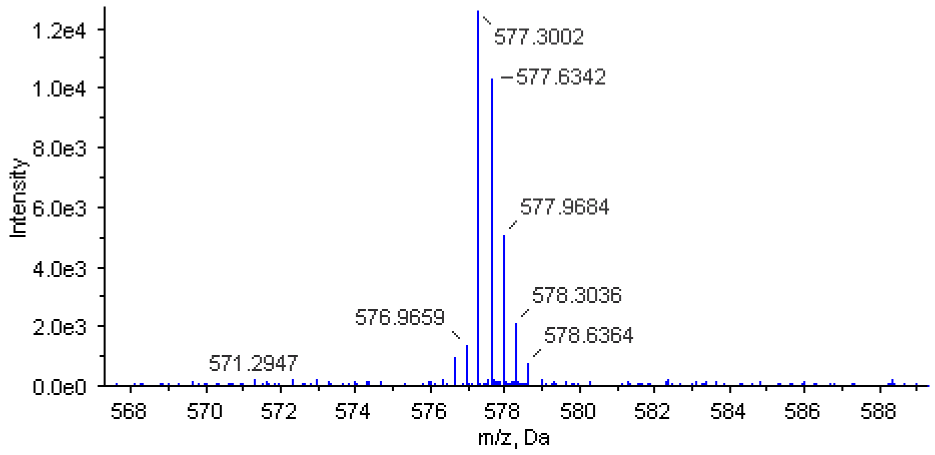

Sequence Coverage and Matched peptides: in different color

MSAGFVGVLVSDPWLQSQFTQVELRTLKAKYVSIRTEGGR**VTVGDLPPVFVK**LR**AFTEVFNEDEIK**AIL
KESYSKAEEELDFETFLR**AYLNVQAR**VPAKSGGKKLRYSSSFLKASTTTFRHNINESEKASYVSHINNH
LGEDSFLKTYLPIDPATNALFDLVKHGVLLCK**LINVAVPGTIDER**AINTKKEMNPWER**NENHTLCLNSA**
KAIGCTVVNIGTQDLAEARPHLVLGLISQIIKIQLLADLNLKK**TPQLVELVDDSK**DVEELMGLPPEKVL
LKWMNFHLKKAGYNK**EVTNFSSDVK**DGEAYVYLLNALAPELSTPDTKAASNPAER**AKVVLEQAEK**LDCK
RYLNANDIVEGSPNLNLAF**VAQIFQHR**NGLSTDTNKMPVAEMMEDDAETSREERCFRLWINSIGIVTYV
NNVFEDVRNGWVLLEVLDKVSPGSVNWKQATKPPIKMPFRKVENCNQAIKIGKELSFSLVNVAGNDIVQ
GNKKLLLAFLWQLMRFTMLQLLRNLRSHSSHSQGK**EITDADILKWANSK**VKKVGK**TSQMESFK**DKTLSN
GVFFLDLLGAVEPRVVNWSVVTKGESDEDKKLNSTYIISVARK**LGCSIFLLPEDIIEVNQK**MMLTLTAS
IMFWSLQHQAAEPESSAAEDNADASSTASMEGENEAALAGEVSDMTMDDAASDSAMSP

Protein No.: I-511

Protein name and Species:

Folylpolyglutamate synthase OS=Populus trichocarpa GN=FPGS2 PE=4 SV=1

Accession: tr|B9HMM6|B9HMM6_POPTR

Unused ProtScore: 4.59

Seq Cov %: 5.2

ITRAQ Changed Ratio:

117: 115=E3TL: D3TL=0.26

116: 114=E5TL: D5TL=1.11

Annotated typical Precursor MS Region:

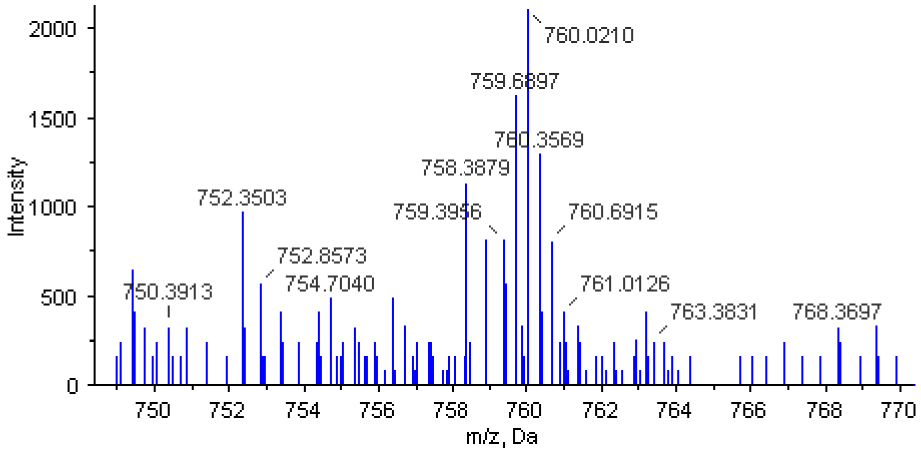

Sequence Coverage and Matched peptides: in different color

MAEGDNNGSSKPTVTPYEEALDALSSLITKRSRADKSNNGDRFDVLFDYLKLEEAISEMKIIHVAGTKG
K**GSTCTFTESILR**NCGFR**TGLFTSPHLIDVR**ERFRLDGKDICEEKFLAYFWWCYDRLKEKSTEDVPMPT
YFRFLA**LLAFK**IFSAEQVDVAILEVGLGGKFDATNVVQKPIVCGVASLGYDHMEILGNTLAQIAGEKAG
IFKDGIPAFTVPQPDEAMNVLEEKASELNVHLQVAEPLDAKLLNGLKLRLEGEHQYLNAGLAIALSFTW
LQRTGHHEFTHMEQASSLPEQFIKGLTTASLQGRAQIVTDQYINAESYGDLVFYLDGAHSPESMEMCAR
WFSLAIKEDSQKRTFNYPPQNNSESTIELVQEHHDERYGKSSMQILLFNCMSVRDPQLLLPPLMKACAS
HGVYFKKALFVANTSVYYKVGSHALPPTDSQFNLSWQFALQRVWENLIQSDKGGEVKHAVAVCEEGKED
AKMSGR**TCENSAVFPSLPLAIK**WLKESVQRNRSVHYQVLVTGSLHLVGDVLRLVKK

Protein No.: I-512

Protein name and Species:

Myosin XI, putative OS=Ricinus communis GN=RCOM_1724180 PE=4 SV=1

Accession: tr|B9S596|B9S596_RICCO

Unused ProtScore: 4.58

Seq Cov %: 5.5

ITRAQ Changed Ratio:

117: 115=E3TL: D3TL=1.61

116: 114=E5TL: D5TL=1.52

Annotated typical Precursor MS Region:

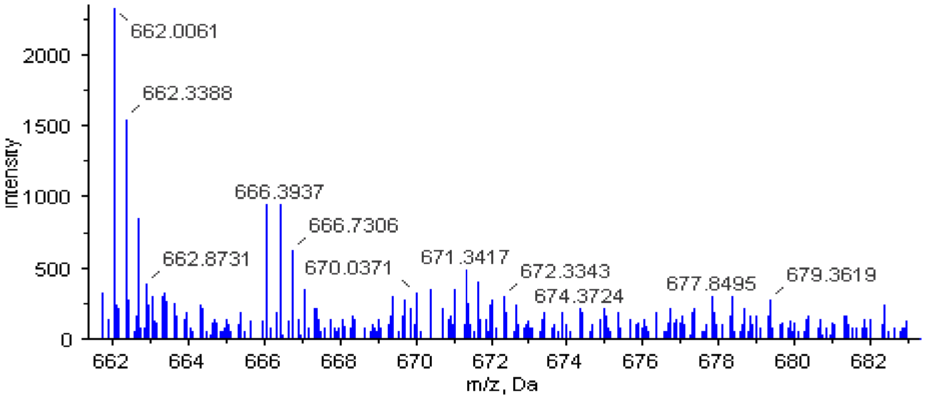

Sequence Coverage and Matched peptides: in different color

MMKQAAAVTLVVGSLVWLEDPDVAWIDGEVVEMKGDNIKVLCTSGKTVVVKASNIYPK**DAEAPPCGVDD**
MTKLAYLHEPGVLQNLRSRYDMNEIYTYTGNILIAVNPFRKLPHLYDSHMMAQYKGAAFGELSPHPFAV
ADAAYRLMINEGI**SQSILVSGESGAGK**TESTKLLMRYLAYMGGRAVAEGR**TVEQQVLESNPVLEAFGNA**
KTVRNNNSSRFGKFVEIQFDQMGRISGAAIRTYLLERSR**VCQVSDPER**NYHCFYMLCAAPQEDVQRYKL
GNPRTFHYLNQSNCYELDGIDDSKEYIATRR**AMDIVGISSDEQDAIFR**VVAAVLHLGNIEFAKGKETDS
SMPKDEKSRFHLRTAAELLMCDVKALEDSLCKR**VIVTR**DETITKWLDPESAVLSRDALAKIVYSRLFDW
IVDKINNSIGQDPDSKSLIGVLDIYGFESFKTNSFEQFCINLTNEKLQQHFNQHVFKMEQEEYTKEEID
WSYIDFVDNQDVLDLIEKKPGGIIALLDEACMFPRSTHETFAQKLYQTFKNHKRFSKPKLARSDFTICH
YAGDVTYQTELFLDKNKDYVVAEHQALLSASTCSFVSGLFPLSAEESSKQSKFSSIGSRFKQQLQSLLE
TLSATEPHYIRCVKPNNLLKPAIFENKNILQQLRCGGVMEAIR**ISCAGYPTR**KPFDEFVDRFSILAPEV
LDGSSDEITACKRLLEKVGLEGYQIGKTKVFLRAGQMADLDTRRSEVLGRSASIIQRKVRSYLSRRSFI
MLRRAAIHIQASCRGQLARQVYENMLREAASLRIQTYLRMYVARKAYIELYCSAISIQTCMRGMAARDE
LRFRRRTRAAIVIQSHCRKYLARLHFMELKKATITAQCAWRGRVARKELRTLKMAARETGALQAAKNKL
EKQVEELTWRLQLEKRMRADLEEAKTQENSKLQSALQEMQLQFKETKEMLVKEREAAKTAKEIIPVIQE
VPVVDNAMLEKLTTENEKLKAMVSSLEKKIDETEKKFEETSKISEERLKQALEAESKIVELKTAMHRLE
EKFTDMETENQILRQQSLLQTPVKKTSDRPPIPAVPNLENGHHVNEEHRASEPQTPVKVFGTESDSKFR
RSHIERQHENIDALINCVMHNIGFSHGKPVAAFTIYKCLLHWKSFEAEKTSVFDRLIQMIGSAIENEDN
NDHMAYWLSNTSTLLFLLQKSLKAAGAGGATPNKKPSSAASLFGRMAMGFRSSPSSSNLAAALAVVRQV
EAKYPALLFKQQLAAYVEKIYGIIRDNLKKELSSLLSLCIQAPRTSKGSALRSGRSFGKDSPSSHWQSI
IDSLNILLSTLKQNFVPPVLIQKIYTQTFSYINVQLFNR

Protein No.: I-514

Protein name and Species:

Cinnamyl alcohol dehydrogenase 1-like [Vitis vinifera] OS=Vitis vinifera GN=VITISV_025556 PE=3 SV=1

Accession: tr|A5C5R6|A5C5R6_VITVI

Unused ProtScore: 4.58

Seq Cov %: 4.5

ITRAQ Changed Ratio:

117: 115=E3TL: D3TL=0.29

116: 114=E5TL: D5TL=0.25

Annotated typical Precursor MS Region:

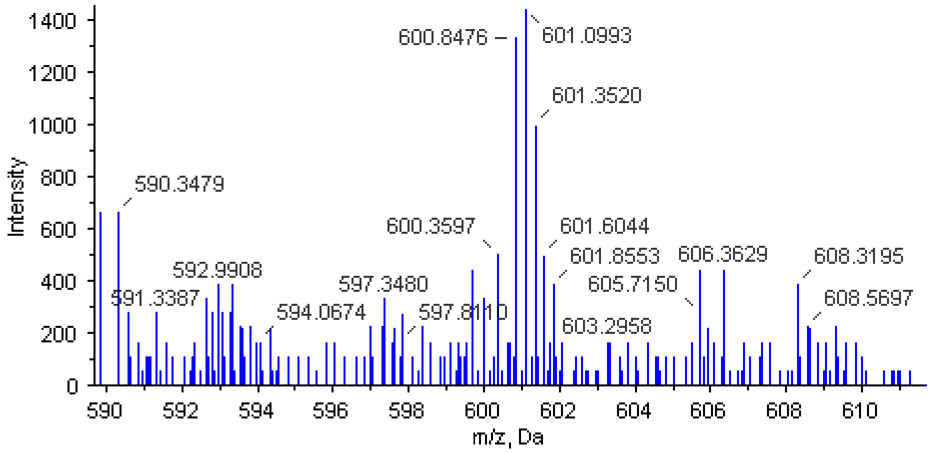

Sequence Coverage and Matched peptides: in different color

MDSVKEKTHGDTKSKKLAQQRSRLQTMSLNWVSCMSKPDMMNNNCLGWAARDESGVLSPYTFTRREVGD
EDVSLKITHCGICYADMLFTKNRLGDSR**YPLVPGHEIVGIVK**EVGSNVHHFKVGHRVGVGAYVNSCREC
ESCNQSLEVYCSKGSVYTSNGVDADGTITN**GGYSSFIVVHER**YCFRIPDEYPLALAAPLLCPGITAYSP
MVRHKMNQPGKSLEVIGLGGVGHLAVKFGKAFGLNVKLFSTSISKKEEALSLLGDDNFVVSSDEQQMKA
MANSLDFIIDTASGDHSLDHYMSCLKTAGVLALVGFPGMKTISGSLVGGVKETQEMLDFCAAHKIYPDI
EIVPIQYVNEAWERLTKGDVKYRVVGSDDVSLKITHCGICYADIIHTRNKFGNSKYPVVPGHDIVGIVK
EVGSNVGVFKVGDSVGVGTYVNSCRECENCEEGLEIHCSKGPVFTFGGVDVDGTITKGGYSSFIVVHQR
YCYRIADDYPLGLAAPLLCAGITVYTPMIRHKMNQPGK**SLGVIGLGGLGHMAVK**FGKAFRLNVTVFSTS
MSKKEEALTLLGADKFVLSSDEQDMKAMAKSLDFIINTASGDHPFDPYMSLLKTAGILEAQKTQEMLDF
CAAHKIYPEVEMVAIQNVNEALERVIKRDVKYRF**VIEIEK**SLK

Protein No.: I-516

Protein name and Species:

Acetylornithine deacetylase, putative OS=Ricinus communis GN=RCOM_0864770 PE=4 SV=1

Accession: tr|B9S1F8|B9S1F8_RICCO

Unused ProtScore: 4.57

Seq Cov %: 12.2

ITRAQ Changed Ratio:

117: 115=E3TL: D3TL=0.14

116: 114=E5TL: D5TL=0.57

Annotated typical Precursor MS Region:

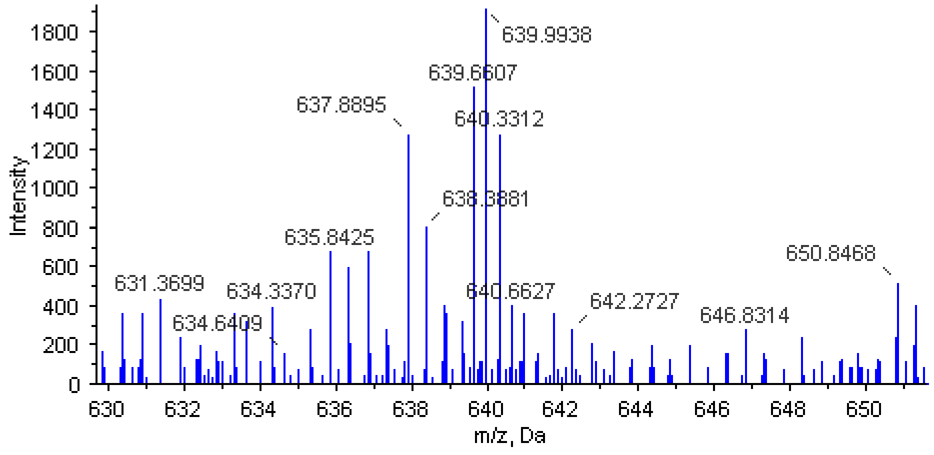

Sequence Coverage and Matched peptides: in different color

MANVEEILGELDKESFVTLLRKIIGESK**HVQNNPPELIPEEDR**VVKHVLDSLLPYSTTSGGGPLIVNHV
SYFPGR**GNLIVEYPGTEPGK**ILSFVGMHMDVVTANPNDWEFDPFSLTINGDKLCGRGTTDCLGHVAVVT
ELMKMLAEKKPKLKSTVVAVFIANEENSAITGVGVDALVQDGLLDKLKGGPLFWIDTADKQPCIGTGGM
IPWKLHVTGKLFHSGLAHKAINALELAMDALQEMQSRFYKDFPPHPREQVYGFATASTMKPTQWSYPGG
GINQIPGECTISGDVR**LTPFYNLVDVK**NM**LQEYVDDINENIEK**LGTRGPVSKYVLPDENLRGSITLTFE
EAMSGVACNLDSRGFHVLCKATEKVVGHVKPY**SITGSLPLIR**ELQEEGFDVQTAGYGLMATYHAKNEYC
LLSDMCQGYRVFVSIISQLED

Protein No.: I-520

Protein name and Species:

Isoleucyl tRNA synthetase, putative OS=Ricinus communis GN=RCOM_0574620 PE=3 SV=1

Accession: tr|B9SUT5|B9SUT5_RICCO

Unused ProtScore: 4.51

Seq Cov %: 5.6

ITRAQ Changed Ratio:

117: 115=E3TL: D3TL=0.38

116: 114=E5TL: D5TL=0.57

nnotated typical Precursor MS Region:

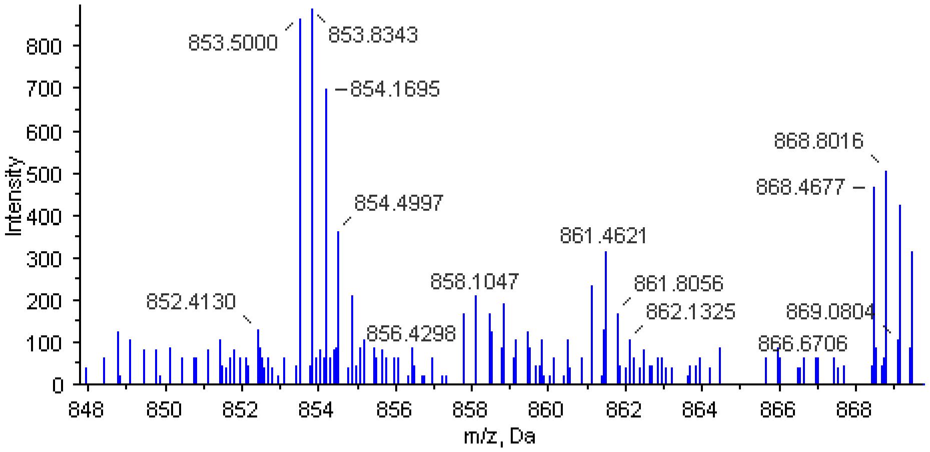

Sequence Coverage and Matched peptides: in different color

MEEVCEGKDFSFPNQEEKILSFWSEIK**AFETQLSR**TESLPEYIFYDGPPFATGLPHYGHILAGTIKDIV
TRYQTMRGHHVTRRFGWDCHGLPVENEIDRKLGIQRRDEVLKMGIDKYNEECRSIVTRYVGEWEKVITR
TGRWIDFKNDYKTMDLKFMESVWWVFYQLFDKGLVYKGFK**VMPYSTGCKTVLSNFEAGQNYK**DVPDPEI
MVAFPIVDDPHNAAFVAWTTTPWTLPSNLALCVNGNFDYVKVRNKYTGK**VYVVAESR**LSALPTEKPKSS
AVNGPAGGKTKGGKTENLMDSFELLEKVKGNELVKKKYVPLFNFFSDFSNTAFRVVADNYVTDDSGTGI
VHCAPAFGEDDYRVCIENQIINKGENLIVAVDDDGCFAERITEFSGRYVKDADKDIIEAVKAKGRLVKS
GTFTHSYPFCWRSDTPLIYRAVPSWFVRVEELKVQLLENNKQTYWVPDYVKEKRFHNWLENARDWAVSR
SRFWGTPLPVWISQDGEEVIVMDSVAKLEKLSGIK**VFDLHR**HHIDHITIPSSRGPEFGVLRRVDDVFDC
WFESGSMPYAYIHYPFENIELFEKSFPGQFIAEGLDQTRGWFYTLMVLSTALFGKPAFK**NLVCNGLVLA
EDGK**KMSKKLKNYPSPMEVIDDFGADALRLYLINSPVVRAETLRFKKEGVYSVVKDVFLPWYNAYRFLV
QNAKRLEVEGLAPFTPLDFGKLQNSSNVLDRWINSATQSLVHFVRQEMDGYRLYTVVPYLLKFLDNLTN
IYVRFNRKRLKGRIGEEDCR**TALSTLYSVLLTSCK**VMSPFTPFFTEVLYQNMRKVSSGAEESIHYCSFP
QEEGERDERIEQSVSR**MMTIIDLAR**NIRERHYKPLKSPLREMIVVHHDADFLDDIAGKLK**EYVLEELNV
R**SLIPCVDTLKYASLRAEPEFSLLGKRLGKAMGVVAKEIKAMSQKDILAFEEAGEVTIASHNLKLADIK
VVREFKRPDGLTEKEIDAAGDGDVLVIMDLRPDESLYEAGVAREVVNRIQKLRKK**VALEPTDVVDVYFE
SLDDDK**SKLGRVLNSQEQYIKDAIGSPLLSSTMMPPEAVVIGEESYHHIYELSFT**IYLAR**AALVFKSDA
ILTLYAGNTKFAKGLETYLLSRDHSNLRSEFQQRNGKITVDCIENQPAADVVLGEHLFLTVGDYFLRTR
SG

Protein No.: I-533

Protein name and Species:

3R-hydroxymyristoyl ACP dehydrase [Arabidopsis thaliana] OS=Arabidopsis lyrata subsp. lyrata GN=ARALYDRAFT_481086 PE=3 SV=1

Accession: tr|D7LDD9|D7LDD9_ARALL

Unused ProtScore: 4.37

Seq Cov %: 9.8

ITRAQ Changed Ratio:

117: 115=E3TL: D3TL=0.31

116: 114=E5TL: D5TL=0.21

Annotated typical Precursor MS Region:

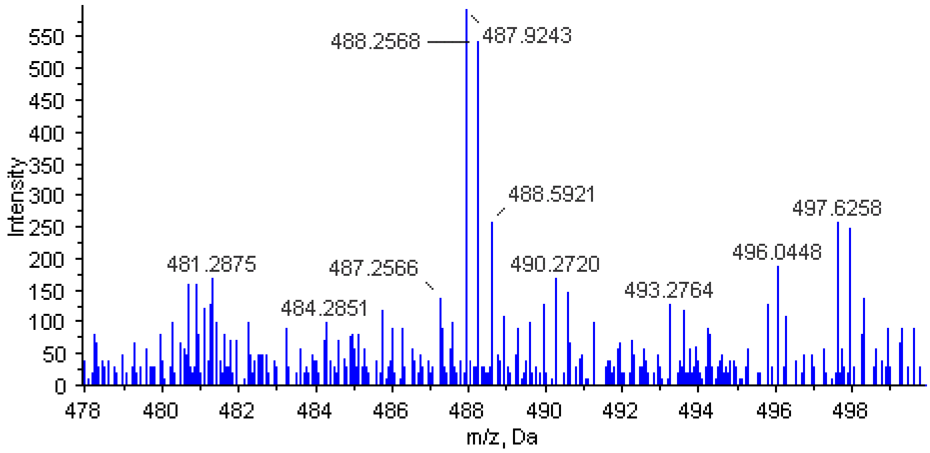

Sequence Coverage and Matched peptides: in different color

MATTSNSVLFLSSDSLSHHHHHHHQQQPLHLPSSRSHSVSLPPNKRSNSLTLRCSTNGDNTSSEKETPI
ELKFPAFPTVMDINQIREILPHRFPFLLVDRVIEYTPGVSAVAIKNVTINDNFFPGHFPERPIMPGVLM
IEAMAQVGGIVMLQPEVGGSQDNFFFAGIDKVRFRKPVIAGDTLVMRMTLLKFQKRFGLAKMEGKAYVG
GALVCEGEFMMVSAGSS

Protein No.: I-534

Protein name and Species:

Glyceraldehyde 3-phosphate dehydrogenase, putative OS=Ricinus communis GN=RCOM_1574030 PE=3 SV=1

Accession: tr|B9RHV9|B9RHV9_RICCO

Unused ProtScore: 4.35

Seq Cov %: 20

ITRAQ Changed Ratio:

117: 115=E3TL: D3TL=0.23

116: 114=E5TL: D5TL=0.36

Annotated typical Precursor MS Region:

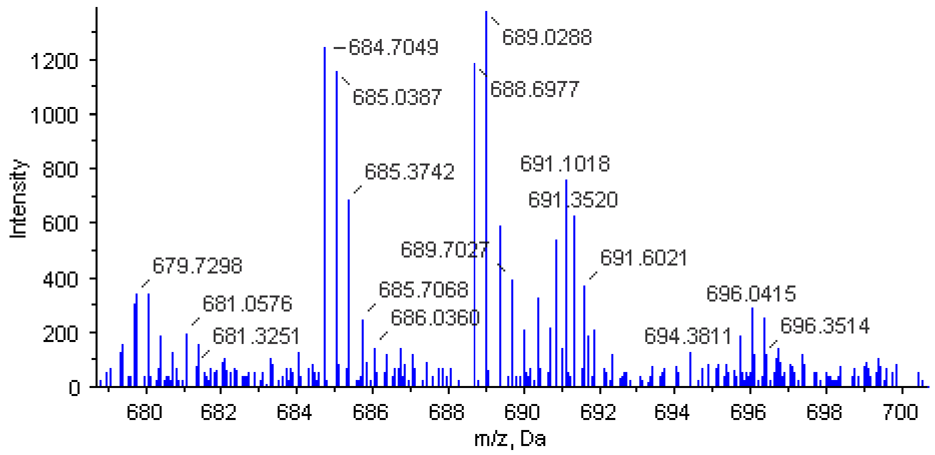

Sequence Coverage and Matched peptides: in different color

MAFSCILRSTATSPLIEASRSDLSPSPSDPLQASSVNFNRDLKASKSFFGTSVPTASSSLEACSARSLQ
PIKATVTEMPPTILKSRSDGRTK**IGINGFGR**IGRLVLRIATLR**DDVDVVAVNDPFIDTK**YMAYMLKYDS
THGLFKGHIKVVDESTLEINGKQIKVSSKRDPAEIPWGDHGAEYVVEASGVFTTIDKASAHKKGGAKKV
VISAPSADAPMFVVGVNEQTYKPNMDIVSNASCTTNCLAPLAKVVHEE**FGIVEGLMTTVH**ATTATQKTV
DGPSMKDWRGGR**GAGQNIIPSSTGAAK**AVGKVLPELNGK**LTGMAFR**VPTPNVSVVDLTCRLEKSASYED
VKAAIKYASEGPLK**GILGYTDEDVVSNDFVGDSRSSIFDAK**AGIGLSAAFMKLVSWYDNEWGYSNRVLD
LIEHMALVAAQN

Protein No.: I-536

Protein name and Species:

Glutathione-S-transferase OS=Rheum australe PE=2 SV=1

Accession: tr|B5M1W3|B5M1W3_RHEAU

Unused ProtScore: 4.35

Seq Cov %: 12.9

ITRAQ Changed Ratio:

117: 115=E3TL: D3TL=0.22

116: 114=E5TL: D5TL=0.56

Annotated typical Precursor MS Region:

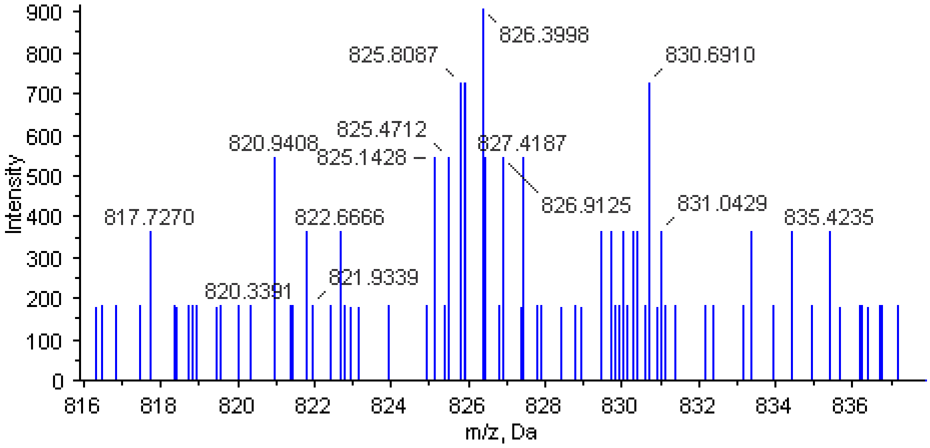

Sequence Coverage and Matched peptides: in different color

MAATLPPALDASSPPPPLFDGTTR**LYTSYTCPFAQR**VWITRNYKGLQEKIKLVGINLQNRPDWYKEKVN
PANK**VPALEHDGK**IIVESLDLIKYVDSNFEGPSLYPDEPAKREFGEELLTYVSEFVPKMYGAFKGGDTT
KVADAEFDYLEKALQKFDDGPFFLGQFSLVDIAYAPFIERFQLVIADVWK**YDLTAGRPK**LAEWVEELNK
IEAYKETKTDPDQLLPYLKSRFVAQQ

Protein No.: I-547

Protein name and Species:

Beta-1,3-glucanase OS=Vitis vinifera GN=VIT_05s0077g01150 PE=2 SV=2

Accession: tr|Q9M563|Q9M563_VITVI

Unused ProtScore: 4.31

Seq Cov %: 15.8

ITRAQ Changed Ratio:

117: 115=E3TL: D3TL=15.42

116: 114=E5TL: D5TL=3.93

Annotated typical Precursor MS Region:

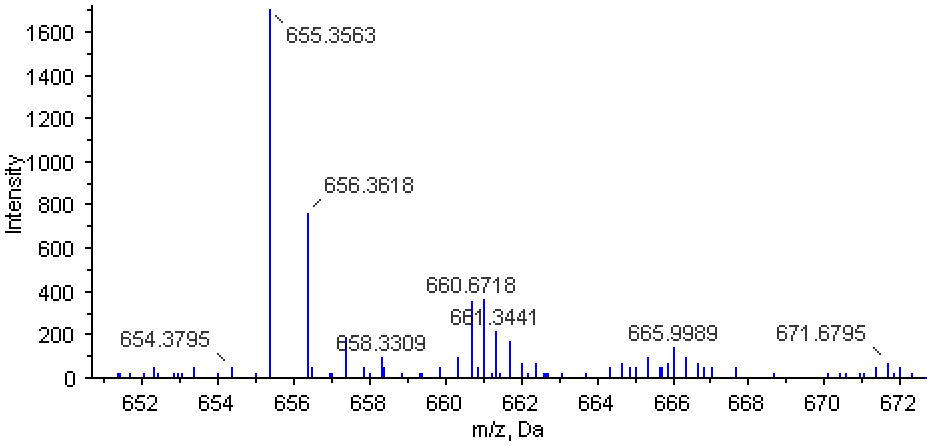

Sequence Coverage and Matched peptides: in different color

MAAMVLLLGFLLATLQITGVKSVGVCYGMLGNNLPPASQVVALYKSRNIDRMR**IYDPNQAALQALR**GSN
IQLMLGVPNSDLQGLATNPSQAQSWVQRNVRNYWPGVSFR**YIAVGNEV**SPVNGGTSRFAQFVLPAMRNI
RAALA**SAGLQDRVKVSTAIDL**TLLGNSYPPSQGAFRGDVRGYLDPIIRFLVDNK**SPLLANIYPYF**SYSG
NPK**DISLPYALFTANSVVVWDGQR**GYKNLFDAMLDALYSALERAGGASLEVVLSESGWPSAGGFGTTVD
NARTYNSNLIRHVKGGTPKRPGR**AIETYLFAMFDENK**KEPQLEK**HFGLFFPNK**QPKYSINFSGEKPWDV
SSENDTNEESLKSDI

Protein No.: I-549

Protein name and Species:

annexin [Populus tomentosa] OS=Populus trichocarpa GN=POPTRDRAFT_654779 PE=3 SV=1

Accession: tr|B9HFG8|B9HFG8_POPTR

Unused ProtScore: 4.3

Seq Cov %: 17.7

ITRAQ Changed Ratio:

117: 115=E3TL: D3TL=0.18

116: 114=E5TL: D5TL=2.47

Annotated typical Precursor MS Region:

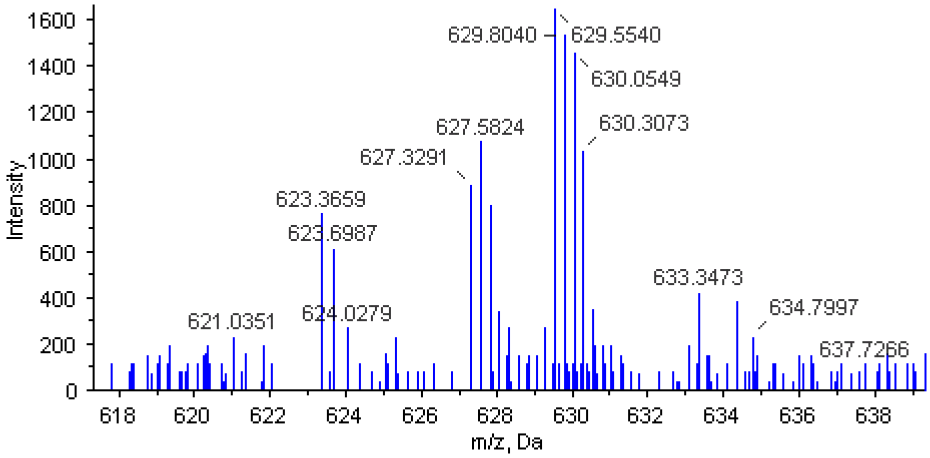

Sequence Coverage and Matched peptides: in different color

MASLKVPASVPPPYEDAEQLHKAFEGWGTNEGLIISILAHRNAAQRNLIRKVYAEAYGQDLLKDLDKEL
SSDFERAVLLWTLDPAERDAYLANEATKRFTSSNWVLMEIACTRSSHDLFKVRQAYHARYKK**SLEEDVA**
YHTTGDFRK**LLVPLVSAFR**YEGEEVNTILAKSEAKILHEKISDKAYSDEEIIRILTTRSK**AQLNATLNH**
YNNAFGNAINKNLKEEADNDFLKLLRATIKCLTYPEKYFEKLLRLSIKKLGTDERALTRVVTTRAEVDM
ERIKEEYHRRNSVTLERDIAGDTSGDYER**MLLALIGHGDA**

Protein No.: I-550

Protein name and Species:

Argininosuccinate synthase, putative OS=Ricinus communis GN=RCOM_0395600 PE=3 SV=1

Accession: tr|B9SS35|B9SS35_RICCO

Unused ProtScore: 4.3

Seq Cov %: 8.2

ITRAQ Changed Ratio:

117: 115=E3TL: D3TL=0.61

116: 114=E5TL: D5TL=0.40

Annotated typical Precursor MS Region:

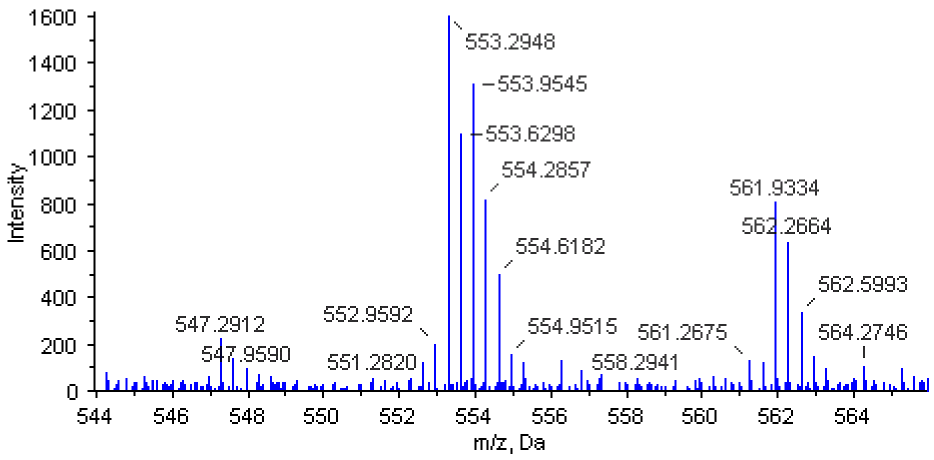

Sequence Coverage and Matched peptides: in different color

MAISSSSFNFAVHDSNRDEFLQNHNIPSIKKSPTIFKKLGAKRGELQGIAFAGTKRVILRTHQNKAIQA
VLSSDREVEVSEASKSKGLRGQLNKVVLAYSGGLDTSVIVPWLRENYGCEVVCFTADVGQGLKELEGLE
EKAKASGACQLVVKDLQEEFVRDYIFPCLRAGAIYERKYLLGTSMARPVIAK**AMVDVAKEVGADAVAHG**
CTGKGNDQVRFELTFFALNPELNVVAPWREWDIKGREDAIEYAKKHNVPVPVTKKSIYSRDRNLWHLSH
EGDILEDPANEPNKDMYMMSVDPEDAPNQPEYIEIGIVSGLPVSVNGK**QLSSASLLSQLNEIGGR**HGIG
R**IDMVENR**LVGMKSRGVYETPGGTILFTAARELESLTLDRETMQVKDSLALK**YAELVYAGR**WFDPLRES
MDAFMEKISEKTTGSVTLKLYKGSVLVTSRTSPYSLYR**QDISSFESGK**IYDQADAAGFIRLYGLPMKVR
AMLENGI

Protein No.: I-552

Protein name and Species:

Glycerophosphodiester phosphodiesterase-like protein (Fragment) OS=Manihot esculenta PE=2 SV=1

Accession: tr|Q5PYQ3|Q5PYQ3_MANES

Unused ProtScore: 4.28

Seq Cov %: 9.2

ITRAQ Changed Ratio:

117: 115=E3TL: D3TL=0.13

116: 114=E5TL: D5TL=0.25

Annotated typical Precursor MS Region:

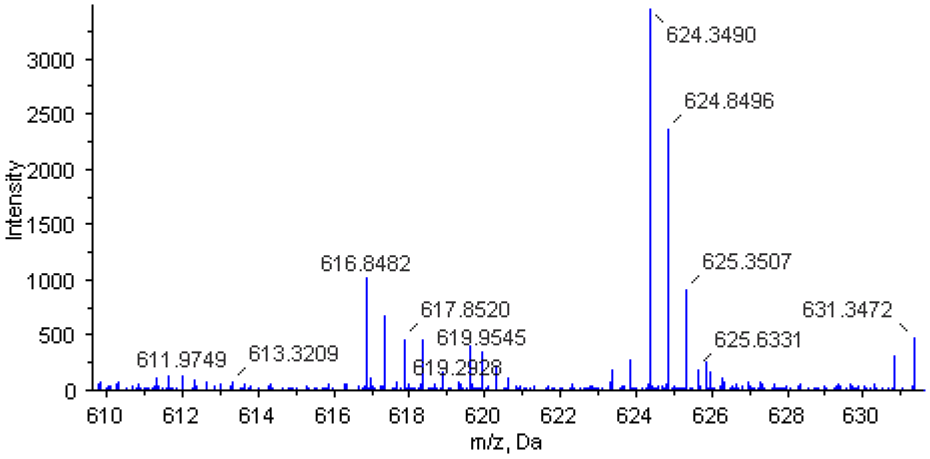

Sequence Coverage and Matched peptides: in different color

MALTCSVLIIFLSLTLVSTATARPIYPLPSKISNGIRKPLQTSRPYNIAHRGSNGEIPEETAAAYMRAI
EEGADFIETDILSSKDGVLICFHDVTLDNTTDIAKHNEFTNRKRTYDVQGVNTTGFFTVDFTLEELKTL
RVKQRHPFRDQQYNGKFSIITFEEFIAIALDASRVVGIYPEMKNPVFINQHVKWPGGKRFEDKFVETLK
KYGYKGSYMSKDWLKQPVFIQSFAPTSLVYISNMTDSPKIFLIDDIDIPTQDTNQSYWEITSDAYLDYI
KNYVVGH

Protein No.: I-556

Protein name and Species:

DNA-binding protein smubp-2, putative OS=Ricinus communis GN=RCOM_0089600 PE=4 SV=1

Accession: tr|B9SL55|B9SL55_RICCO

Unused ProtScore: 4.24

Seq Cov %: 7.6

ITRAQ Changed Ratio:

117: 115=E3TL: D3TL=0.61

116: 114=E5TL: D5TL=0.68

Annotated typical Precursor MS Region:

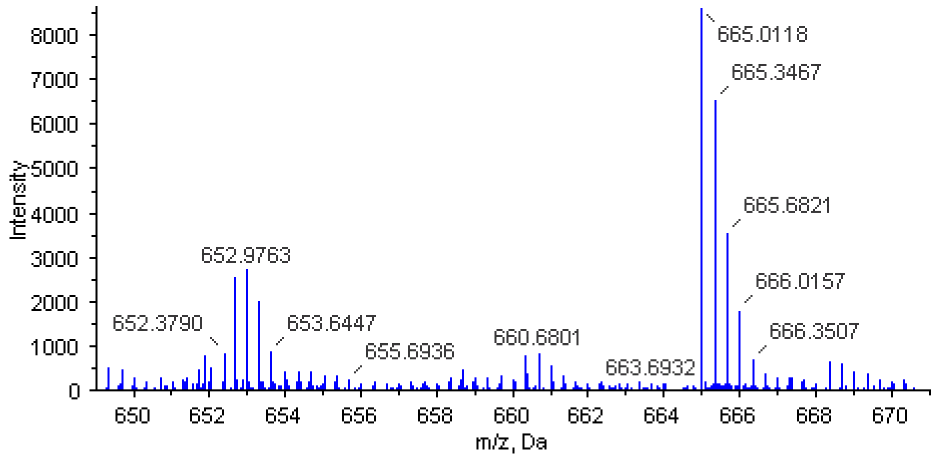

Sequence Coverage and Matched peptides: in different color

MEKDKTKKKLSLQEFVSITSPLLDLEKEAEISASISSGTSRNLDNAQKRGSTILNLKCVDAQTGLMGKT
LLEFQSNKGDVLPSHRFSTHDVVVLKPNKADLGSPALGQGVVYRLKDSSITVAFDDIPEEGLNSPLRLE
KVANEVTYRRMREALIQLSKGVQRGPAADLVPVLFGERQPTMSKKGVTFTPFNSNLDHSQKDAISKALS
SKDVFLLHGPPGTGKTTTVVEIILQEVKRGAK**ILACAASNIAVDNIVER**LVPHRVKLVRLGHPAR**LLPQ**
VLDSALDAQVLKGDNSSLANDIRKEMKALNGKLLKTKDKSTRRDIQKELRTLSKEERKRQQLAVTDVIK
NADVVLTTLTGALSHKLDNTSFDLVIIDEAAQALEIACWIALLKGSRCILAGDHLQLPPTIQSVEAEKK
GLGRTLFERLADLYGDEVTSMLTVQYRMHELIMTWSSKELYNSKIKAHPSIAAHMLSDLEGVKRSSATE
PTLLLIDIAGCDMEEKKDEEESTLNEGEADVAVAHAKRLVENGVQASDIGIITPYAAQVVLLKILRSNE
DKLKDVEISTVDGFQGREKEAIIISMVRSNSKKEVGFLSDRRRMNVAVTRARRQCCLVCDTETVTSDGF
LKRLIEYFEEHGEYLSAAEYCNE

Protein No.: I-561

Protein name and Species:

transportin, putative OS=Populus trichocarpa GN=POPTRDRAFT_570025 PE=4 SV=1

Accession: **tr|B9I337|B9I337_POPTR**

Unused ProtScore: **4.21**

Seq Cov %: **5.1**

ITRAQ Changed Ratio:

117: 115=E3TL: D3TL=1.68

116: 114=E5TL: D5TL=1.63

Annotated typical Precursor MS Region:

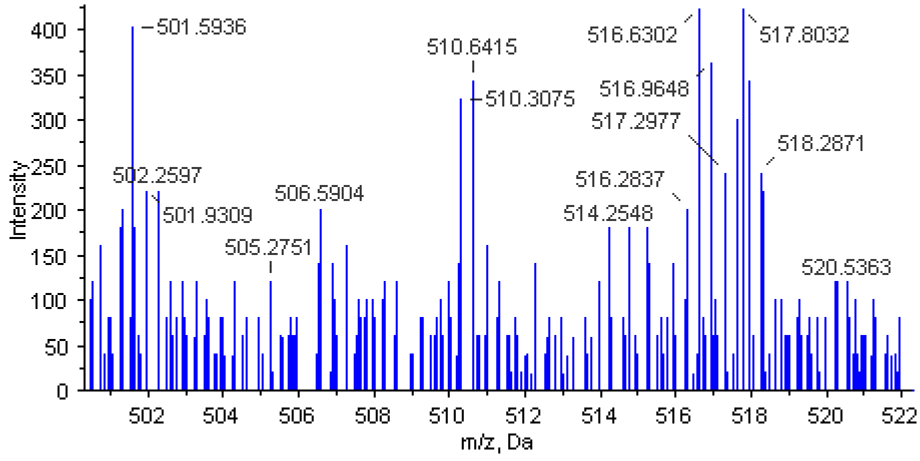

Sequence Coverage and Matched peptides: in different color

MLAVADVLIDAASVLGGDATLRILYVKLAEARTCLGNDHNQWHPAEAALFCIRAISNYVSTVEAEVMPK
IMSLLLELPHEPQLLQTVCLTIGAYSKWLDAALDGFPQLSSVIKILLSGMGKSEDSAAAAAVAFRHICD
DCRRKLCGYFDELFSIYNSAVIGEGSLKVSAGDSLHVVEAFSMVITELPADQAKLALEKLCLPVVTPLQ
EIISQGPDVLEKKLARELTVHIDRLAYIFR**YVNHPEAVADAIQR**LWPILK**AIFDIR**AWDMRTMESLCRA
CKYAVRTSGRLMGITIGAMLEEIQGLYQQHHQPCFLYLSISCHLGMQIFGSDPSCAYYLKNLIEALFKC
TTCLLTNIKDFTARPDIADDCFLLASRCIRYCPQVFIPSAVFPSLVDCSMIGVTVQHREASNSILTFLS
DIFDLAKSSMGEQYLTIRDSVIIPRGSSITR**ILVASLTGALPSSR**LETVTYALLALTRAYGASALEWAR
ESVSLIPSTVVTEVEQTKIFQALTDAASGVDIKTLMGAVEELSDVCRRNRTVQEIVQGALRPLELNLVT
VS

Protein No.: I-562

Protein name and Species:

vacuolar protein sorting-associated protein OS=Populus trichocarpa GN=POPTRDRAFT_833991 PE=4 SV=1

Accession: **tr|B9HYZ8|B9HYZ8_POPTR**

Unused ProtScore: **4.21**

Seq Cov %: **17.7**

ITRAQ Changed Ratio:

117: 115=E3TL: D3TL=1.60

116: 114=E5TL: D5TL=1.66

Annotated typical Precursor MS Region:

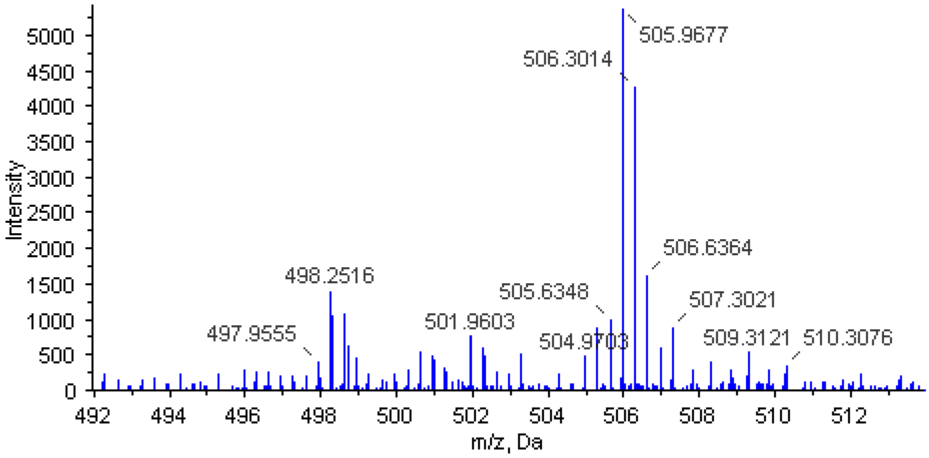

Sequence Coverage and Matched peptides: in different color

MEVKLWNDKREREMYDNFAELYAIIKATEKLEKGYVRDIISSSQYETECQKLIAHFKTLASTFKDTVPS
IERFADTYKMDCPAAINR**LVTSGVPATVEHR**AAAAASSTTSAAVVAECVQNFITAMDSLKLNMVAVDQV
HPLLSDLSASLNK**LSILPPDFEGK**TKMKEWILRLSK**MGAADELTEQQAR**QLHFDLESSYNSFMAALPSA
GT

Protein No.: I-563

Protein name and Species:

Aspartic proteinase, putative OS=Ricinus communis GN=RCOM_1225960 PE=3 SV=1

Accession: tr|B9SFR8|B9SFR8_RICCO

Unused ProtScore: 4.2

Seq Cov %: 16

ITRAQ Changed Ratio:

117: 115=E3TL: D3TL=0.29

116: 114=E5TL: D5TL=0.42

Annotated typical Precursor MS Region:

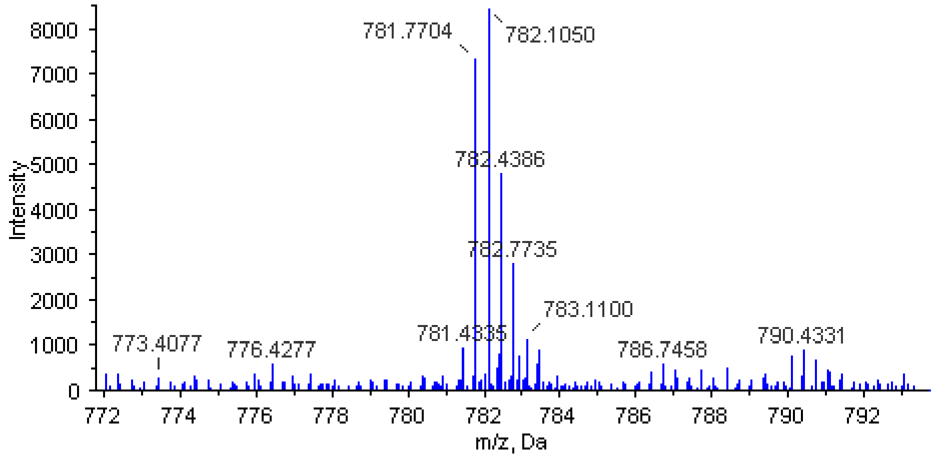

Sequence Coverage and Matched peptides: in different color

MGTKSSALWISFVLLLPVVFSLHNDALVRVGLKKKKFDQVNIPAGTVDFKEGEAMRAATKKYNLVENSD
DVDIVELKNYLDAQYYGEIAIGTPPQTFTVIFDTGSSNLWIPSSK**CYFSVACYFHSK**YKASESSTYQKN
GTSAAIRYGTGSISGFFSQDNVKVGDLVIRNQDFIEATK**EPGVTFLAAKFDGILGLGFQEISVGK**AIPV
WYNMVNEGLVKEQVFSFWLNRNVQAEEGGEIVFGGMDPNHYK**GQHTYVPVTQK**GYWQFDMGEVLIGNEI
TGLCADGCK**AIADSGTSLLAGPTTVITQINHAIGASGIVSQECK**TVVEQYGKFILEMLTAQAQPQKICS
QIGFCTFDGTQGVSTNIESVVDKSKETASDGLQQDSACTVCEMIVVWMQNRLRLNETVDQILNYVNKLC
DRLPSPNGESAVDCSSLSSMPIVSFTIGGKAFKLTADQVKGC

Protein No.: I-567

Protein name and Species:

Profilin-1 OS=Hevea brasiliensis PE=1 SV=1

Accession: sp|O65812|PROF1_HEVBR

Unused ProtScore: 4.17

Seq Cov %: 50.4

ITRAQ Changed Ratio:

117: 115=E3TL: D3TL=0.65

116: 114=E5TL: D5TL=1.98

Annotated typical Precursor MS Region:

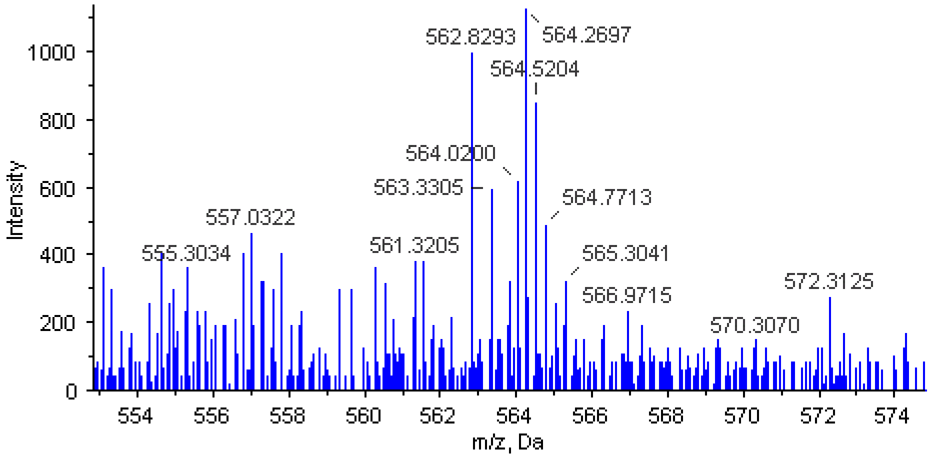

Sequence Coverage and Matched peptides: in different color

MSWQTYVDERLMCEIEGNHLTAAAIIGQDGSVWAQSSNFPQFKSEEITAIMSDFDEPGTLAPTGLHLGG
TKYMVIQGEAGAVIRGKKGPGGVTVRKTNQALIIGIYDEPMTPGQCNMIVERLGDYLLEQGM

Protein No.: I-572

Protein name and Species:

ribosomal protein-like,P0 OS=Solanum tuberosum PE=2 SV=1

Accession: tr|Q307Z8|Q307Z8_SOLTU

Unused ProtScore: 4.15

Seq Cov %: 7.5

ITRAQ Changed Ratio:

117: 115=E3TL: D3TL=7.45

116: 114=E5TL: D5TL=2.63

Annotated typical Precursor MS Region:

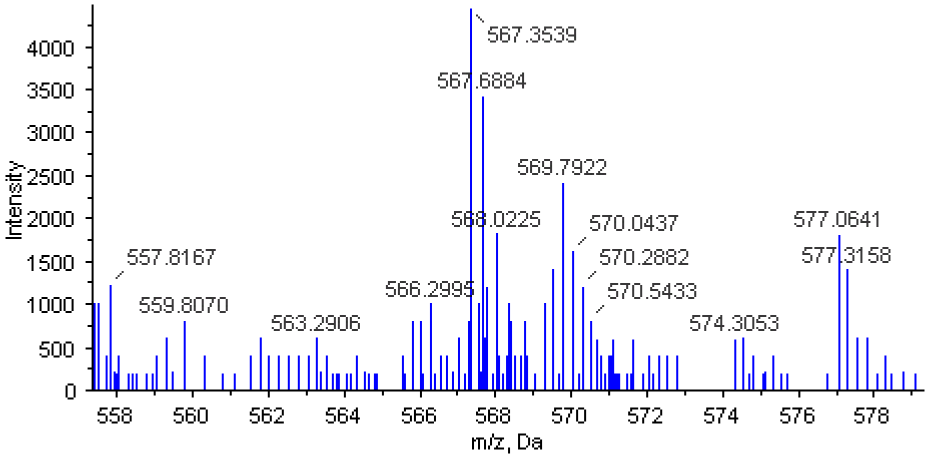

Sequence Coverage and Matched peptides: in different color

MAPKATKAEKKIAYDTKMCQLLDDFTQVLVAAADNVGSNQLQSIRKGLRGDSVVLMGKNTMMKRTIRVH
AEKTGNETILNLIPLLVGNVGLIFTKGDLKEVSEEVAKYKVGAPARVGLVAPVDVVVPPGNTGLDPSQT
SFFQVLNIPTKINKGTVEIITPVELIKKGDKVGSSEAALLAKLGIRPFSYGLVVVSVYDNGSVFSPEVL
DLTEDDLIEKFAMGVSMVTSLALAISYPTLAAAPHMFTNAYKNVLAIAVETDYSFPLADKVKEYLADPS
KFAVAAAPAAAAGSGAAPAAAKEEEKKEEPAEVSDDDMGFSLFD

Protein No.: I-578

Protein name and Species:

Copper transport protein ATOX1 OS=Hevea brasiliensis GN=CCH PE=4 SV=1

Accession: **tr|D4P094|D4P094_HEVBR**

Unused ProtScore: **4.13**

Seq Cov %: **45.4**

ITRAQ Changed Ratio:

117: 115=E3TL: D3TL=0.65

116: 114=E5TL: D5TL=0.52

Annotated typical Precursor MS Region:

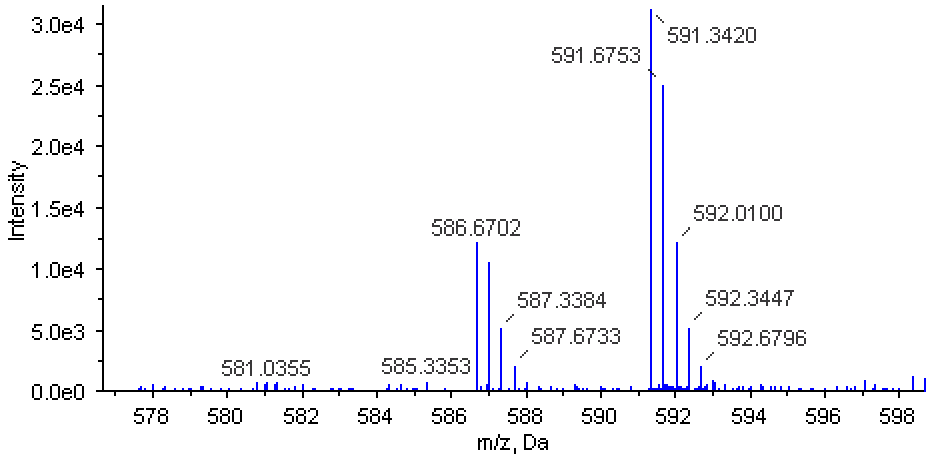

Sequence Coverage and Matched peptides: in different color

MSQTVVLK**VGMSCEGCVGAVK**RVLGK**MEGVESYDIDLK**EQKVTVK**GNVQPEAVLQTVSK**TGKKTTFWEA
EAPAEPETKPAETVTVA

Protein No.: I-580

Protein name and Species:

auxin-induced protein PCNT115 OS=Glycine max PE=2 SV=1

Accession: tr|C6TIG8|C6TIG8_SOYBN

Unused ProtScore: 4.12

Seq Cov %: 10.2

ITRAQ Changed Ratio:

117: 115=E3TL: D3TL=0.60

116: 114=E5TL: D5TL=2.02

Annotated typical Precursor MS Region:

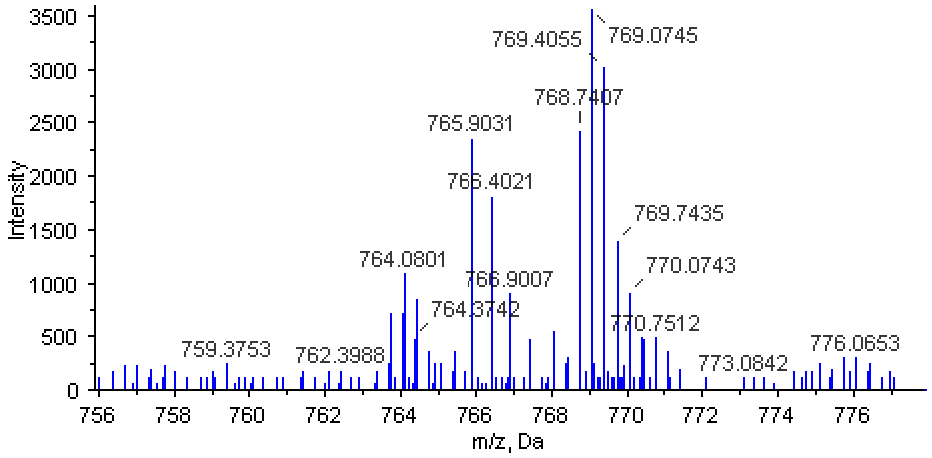

Sequence Coverage and Matched peptides: in different color

MEKLQLHVPRVKLGSQGLEISRLGFGCVGLSGLYNAPLSHEAGCSIIKEAFNMGVTFFDTSDFYGLN**HD**
NEIMIGKALKELPREKVQLATKFGLVRSDGVFAGVKGTPEYVRQCCEASLKRLDVEYIDLYYQHR**VDTS**
VPIEDTMGELKKLVNEGKIKYIGLSQASPDTMKRAHAVHPISALQMEYSLWTR**DIEEEIIPLCR**ELGIG
IVAYSPLGHGFFAGKAAVETLPSQSALAEDARFSGENLEKNKLFYNRIADLASKHSCTPSQLALAWFLH
QGNDIVPIPGTTKIKNLENNVGSVAVKLTNAELSEISDAVPVYEVAGEAPGLGSLSQYTWKFATTPSK

Protein No.: I-584

Protein name and Species:

FH protein interacting protein OS=Populus trichocarpa GN=POPTRDRAFT_809550 PE=4 SV=1

Accession: tr|B9IPT2|B9IPT2_POPTR

Unused ProtScore: 4.09

Seq Cov %: 14

ITRAQ Changed Ratio:

117: 115=E3TL: D3TL=0.64

116: 114=E5TL: D5TL=1.17

Annotated typical Precursor MS Region:

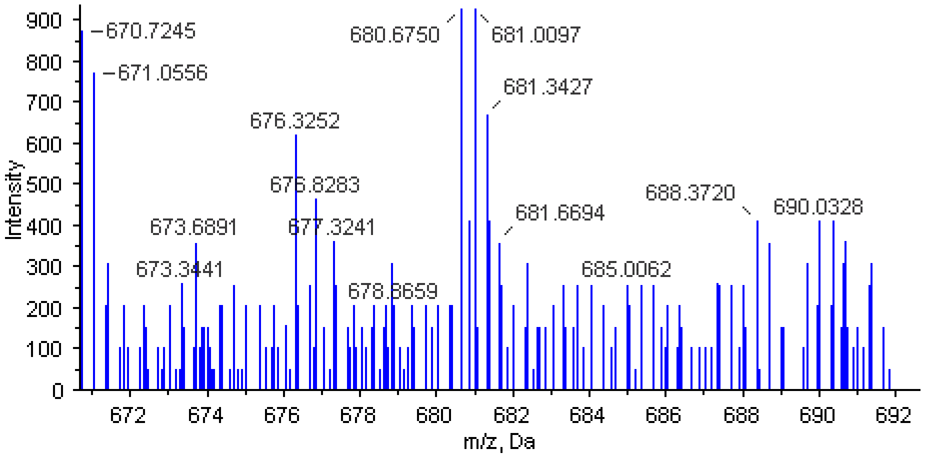

Sequence Coverage and Matched peptides: in different color

MKDHPFSSLIRLNIGGKKFCTTIDTLTQREPDSMLAAMFSGRHTVSEDPDKGYVFIDRDGKHFRHILNW
LR**DGVVPTLTDAEYSELMR**EAVYYQLLGLVEGINSVLTRRREGDELEAELTRADIIKCLQYEKVKFRGV
NFSGLDLSKLDLSYVDFSYASLKNVFFSRANLQCAKFRDVDAEGSIFHNATLRECEFTGANLRGALLAG
ANLKSANLQDACLVDCSFCGADLS**SAHLQTADLTNANLEGANLEGANLK**GAKLNNANLKGANLQRAYLR
HVNLQNTHLEDARLDGANLVGAIR

Protein No.: I-592

Protein name and Species:

Vesicle-associated protein 1 OS=Populus trichocarpa GN=POPTRDRAFT_739862 PE=4 SV=1

Accession: **tr|B9MUU5|B9MUU5_POPTR**

Unused ProtScore: **4.05**

Seq Cov %: **8.7**

ITRAQ Changed Ratio:

117: 115=E3TL: D3TL=1.71

116: 114=E5TL: D5TL=1.69

Annotated typical Precursor MS Region:

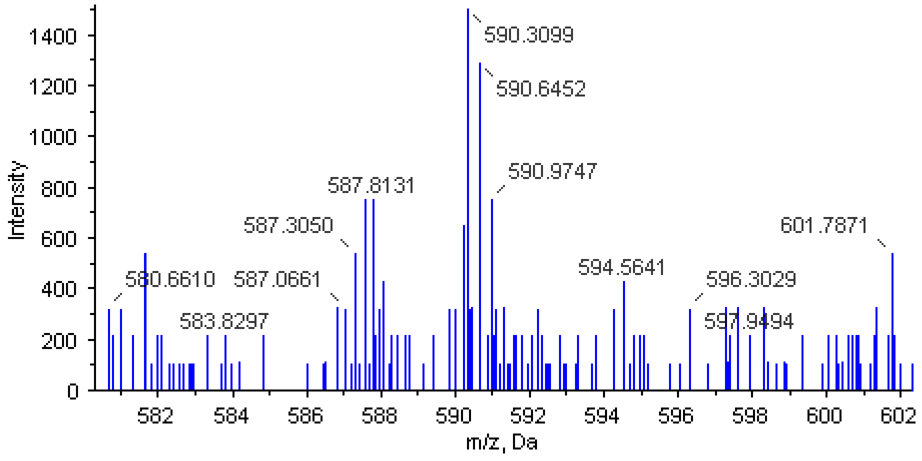

Sequence Coverage and Matched peptides: in different color

MSTGELLGIEPLELK**FPFELK**KQISCSLQLSNK**TDNYVAFK**VKTTNPKKYCVRPNAG**IVLPR****STCDVIV**
TMQAQKEAPPDMQCKDK**FLLQSVK**ANDGVTVKDINAEMFSKEAGHVVEECKLRVLYVSPPQPPSPVLEG
SEEGSPPWGSVSDNGNVNGTDLSTGTRAFVEHLEPQDKPLEAGALISKLTDERNNAIQQNNKLRQE**LEL**
LRCQGNKNRSGASLMFVIIIGLLGLLLGYLMKKT

Protein No.: I-596

Protein name and Species:

Protease C56, putative OS=Ricinus communis GN=RCOM_0763700 PE=4 SV=1

Accession: tr|B9SJL8|B9SJL8_RICCO

Unused ProtScore: 4.05

Seq Cov %: 12.9

ITRAQ Changed Ratio:

117: 115=E3TL: D3TL=0.52

116: 114=E5TL: D5TL=0.31

Annotated typical Precursor MS Region:

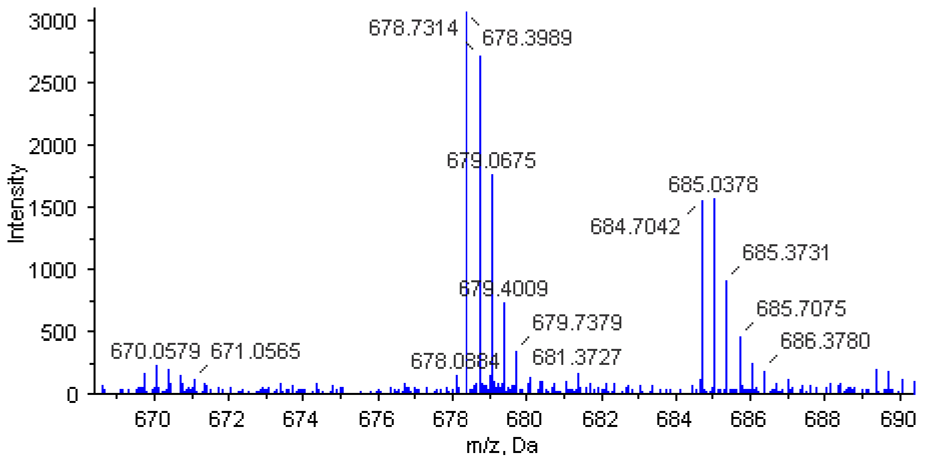

Sequence Coverage and Matched peptides: in different color

MSHQKTVFLLCGDYMEDYEAMVPFQALLAYGVAVDAACPGKKAGDYCRTAIHESSGHQTYSESRGHNFT
LNSTFDDIDFGKYDGLVIPGGR**APEYLAMNESVLDCVR**KFADAGKPIASVCHGQLILAAAGLVKGRKCT
AYPPVRPVLIAAGAHWVEPETMASCVADGNLITGATYEGHPEFIQLFVKALGGSITGADKRILFLCGDY
MEDYEVTVPFQSLQALGCHVNAVCPKKKAGDSCPTAVHDFEGDQTYSEKPGHHFTLTASYVGLDVSSYD
ALVLPGGR**APEYLALDETVIALVK**QFMQSKKPVASICHGQQILAAAGVLKGKKCTAYPAVK**LNVVLSGA**
TWLEPDPIDRCYTDENLVTGAAWPGHPEFISQLMALLGIRVSF

Protein No.: I-602

Protein name and Species:

IAA-amino acid hydrolase ILR1-like 4 OS=Arabidopsis thaliana GN=ILL4 PE=1 SV=2

Accession: sp|O04373|ILL4_ARATH

Unused ProtScore: 4.04

Seq Cov %: 7.3

ITRAQ Changed Ratio:

117: 115=E3TL: D3TL=2.31

116: 114=E5TL: D5TL=1.96

Annotated typical Precursor MS Region:

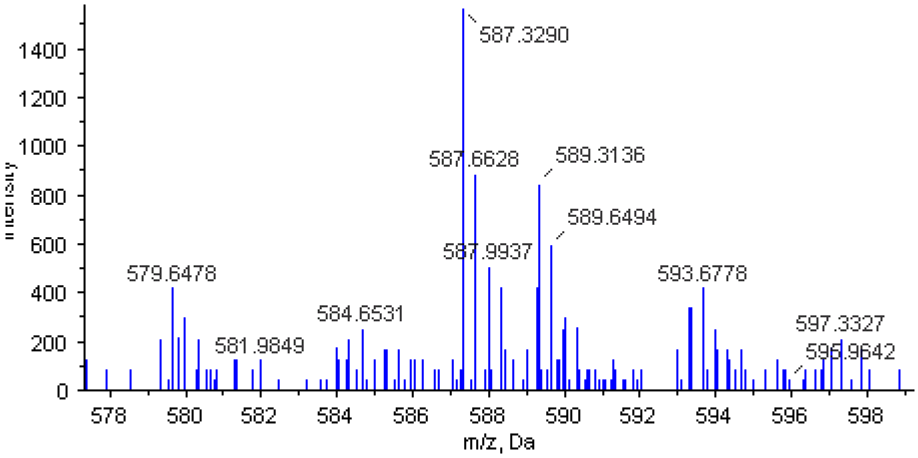

Sequence Coverage and Matched peptides: in different color

MSFFKWVSFVLILHLLNPTLISCSSNGLSQIPSKFLTLAKRNDFFDWMVGIRRRIHENPELGYEEVETS
KLVRAELEKMGVSYKYPVAVTGVVGYVGTGHAPFVALRADMDALAMQEMVEWEHKSKVPGKMHACGHDA
HTTMLLGAAKLLKEHEEELQ**GTVVLVFQPAEEGGGGAK**KIVEAGVLENVSAIFGLHVTNQLALGQVSSR
EGPMLAGSGFFKAKISGKGGHAALPQHTIDPILAASNVIVSLQHLVSR**EADPLDSQVVTVAK**FEGGGAF
NVIPDSVTIGGTFRAFSTKSFMQLKKRIEQVITRQASVNMCNATVDFIEEEKPFFPPTVNDKALHQFFK
NVSGDMLGIENYVEMQPLMGSEDFSFYQQAIPGHFSFVGMQNKARSPMASPHSPYFEVNEELLPYGASL
HASMATRYLLELKASTLNKSNKKDEL

Protein No.: I-605

Protein name and Species:

Putative uncharacterized protein OS=Ricinus communis GN=RCOM_0701460 PE=4 SV=1

Accession: tr|B9SC09|B9SC09_RICCO

Unused ProtScore: 4.03

Seq Cov %: 8.3

ITRAQ Changed Ratio:

117: 115=E3TL: D3TL=0.48

116: 114=E5TL: D5TL=0.56

Annotated typical Precursor MS Region:

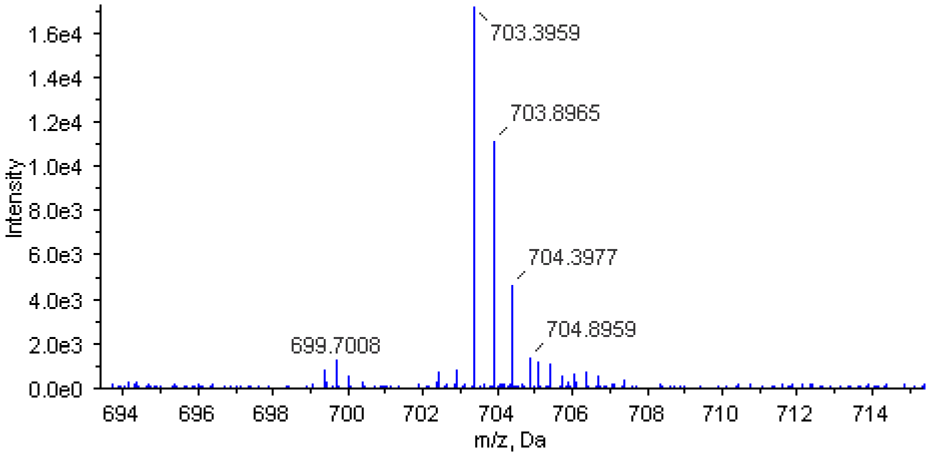

Sequence Coverage and Matched peptides: in different color

MAPMLPKSVAIRSEFSRGYAYYMAENEAVRGFIRCEGENILNPYVKIEVETAQIDSKFVHLRFSHTNKY
WGRSGPGFTQDDYWVSATSDQPEEDTSKWSCTLFEPIFDHGFLFLRHVQTGGRVRTDGPIGATGPVSRL
LLYFRNYDNVPYLDFTFFDLGTLCILPKRVAFRGYNDMYLKAEWIERHEYLQFSSDDVNEKAAGYEITM
MPDGHVRLRSNFFNKFWRRSPNWIWADSTDTVGSDRDTLFWPVK**VNDDTIALR**NAGNNR**FCSSLTAERK**
TDCLNAAVATITREAR**LQVEELVFER**SIFNVRFLMEYARIFDERATVAGWGSAENQSEEAATLSITVGY
EDTTSFTFSNSLAITAGVTVGITTGLPRIAEGKIEISTEVTNTLEWNRTTSETRTASATYLANVPARSR
I**RIDYVATRGTCNIPFSYTQR**DRLSHDGSFATTQHVDGVYTGVNYYSFHFEQPQIVPL

Protein No.: I-606

Protein name and Species:

Lipase class 3 family protein [Arabidopsis thaliana] OS=Arabidopsis thaliana PE=4 SV=1

Accession: tr|Q9LUL3|Q9LUL3_ARATH

Unused ProtScore: 4.03

Seq Cov %: 4.1

ITRAQ Changed Ratio:

117: 115=E3TL: D3TL=0.21

116: 114=E5TL: D5TL=0.40

Annotated typical Precursor MS Region:

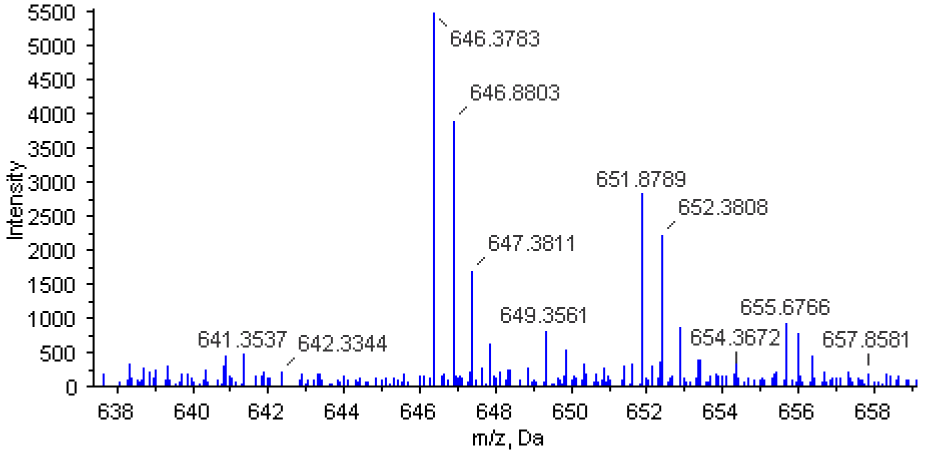

Sequence Coverage and Matched peptides: in different color

MHKDNDSGSGSNPGQVSNYLIVRPHRGGYIDLFRYGVRDDQTSKAKFLEMPDNREWSTITIDEEAEDHR
WVIVVSILVRKIIRLLRTPMEFTGFVVDFFLNLFSANGGFFGLLLRLIQAKVVIPERGSVTFVSTIGQL
DGRISLYKEWNFVEHLEGIDSVDSGRVKIELGSR**GLMDLCVMASKLAYENAK**MNLVEFLDCWNDYQKQM
STQVFVFTDKQK**DANLIVISFR**GTEPFDADDWGTDFDYSWYEVPNVGKLHMGFLEAMGLGNRDDTTTFH
YNLFEQTSSEEENSKKNLLDMVERSAYYAVRVILKRLLSEHENARFVVTGHSLGGALAILFPTLLVLNE
ETEIMKR**LLGVYTFGQPR**IGNREVGLFMKAQLNQPVDRYFRVVYCNDIVPRLPYDDKTFLYKHFGLCLF
YDSFYNETKAEDEPDPNPYGLRYKILGHVIAVWELVRGLTMGYTHGPDYKEGWFRILFRLMGLVIPGLS
DHCMTDYVNSVRLGPDNELQMSSL

Protein No.: I-610

Protein name and Species:

dehydrogenase/reductase SDR family member 12 [Vitis vinifera][Vitis vinifera] OS=Vitis vinifera GN=VIT_18s0157g00200 PE=4 SV=1

Accession: tr|D7SXZ2|D7SXZ2_VITVI

Unused ProtScore: 4.02

Seq Cov %: 15.2

ITRAQ Changed Ratio:

117: 115=E3TL: D3TL=1.91

116: 114=E5TL: D5TL=1.58

Annotated typical Precursor MS Region:

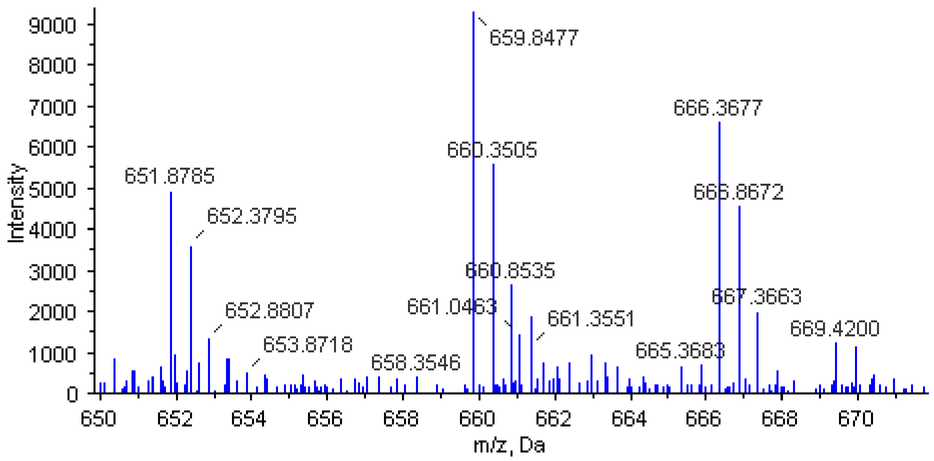

Sequence Coverage and Matched peptides: in different color

MQNPTLHIQIHISIRESIMFILKTWRSIAFGVHGYKNSTKTGFMEHSKNFKSEDMQTQIEGK**NCIVTGA**
NSGIGYATAEGLASRGANVYMVCRNKERGEAALSEIQSK**TGNSNVHLEVCDLSSVSEIK**SFASKFSKKD
VPIHVLVNNAGLLEYNRITTSEGFELNFAVNVLGTFTMTESMLPLLEKAAPDARVITVSSGGMYSVPLT
NDLQFSDDKFDGVTQYARNKR**VQVALTEK**WAEMYKNKGIGFYAMHPGWAETSGLAKSLPGFYKLLSGNL
RTIEEGADTIIWLALQPKEK**LVSGAFYFDR**AEAPKHLMFAATRSSHAMIDSIIGNLRSFSGLSALSS

Protein No.: I-612

Protein name and Species:

UTP-glucose-1-phosphate uridylyltransferase OS=Pyrus pyrifolia PE=2 SV=1

Accession: sp|O64459|UGPA_PYRPY

Unused ProtScore: 4.01

Seq Cov %: 23.6

ITRAQ Changed Ratio:

117: 115=E3TL: D3TL=0.52

116: 114=E5TL: D5TL=0.27

Annotated typical Precursor MS Region:

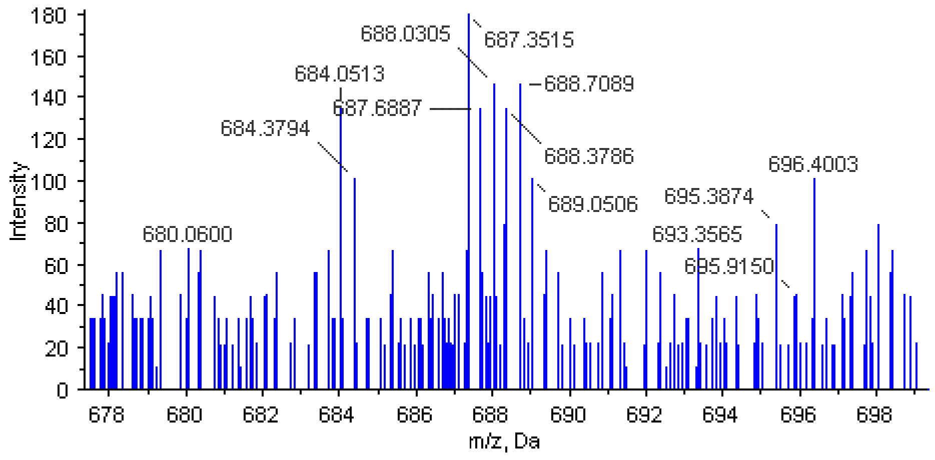

Sequence Coverage and Matched peptides: in different color

MAAVATGNVDKLKSDVASLSQISENEKNGFINLVSRYVSGEEAQHVEWSKIQTPTDEVVVPYDGLAPTP
EDPEEIKKLLDKLVVLK**LNGGLGTTMGCTGPKSVIEVR**NGLTFLDLIVIQIENLNNKYGSCVPLLLMNS
FNTHDDTQKIVEKYSK**SNVQIHTFNQSQYPRLVVEDFSPLPSK**GQTGK**DGWYPPGHGDVFPSLK**NSGKL
DLLLSQGKEYVFIANSDNLGAVVDLKILHHLIQKK**NEYCMEVTPK**TLADVKGGTLISYEGR**VQLLEIAQ
VPDQ**HVNEFKSIEKFKIFNTNNLWVNLNAIKR**LVEADALKMEIIPNPK**EVDGVK**VLQLETAAGAAIRFF
NHAIGINVPR**SRFLPVK**ATSDLLLVQSDLYTLQDGFVTR**NSARKNPENPTIELGPEFKKVGSYLSRFKS
IPSILELESLKVSGDVWFGAGVVLKGKVTITAKSGVKLEIPDNAVIANKDINGPEDL

Protein No.: I-613

Protein name and Species:

Chitinase OS=Euonymus europaeus PE=2 SV=1

Accession: tr|Q7Y237|Q7Y237_EUOEU

Unused ProtScore: 4.01

Seq Cov %: 13.1

ITRAQ Changed Ratio:

117: 115=E3TL: D3TL=0.24

116: 114=E5TL: D5TL=0.21

Annotated typical Precursor MS Region:

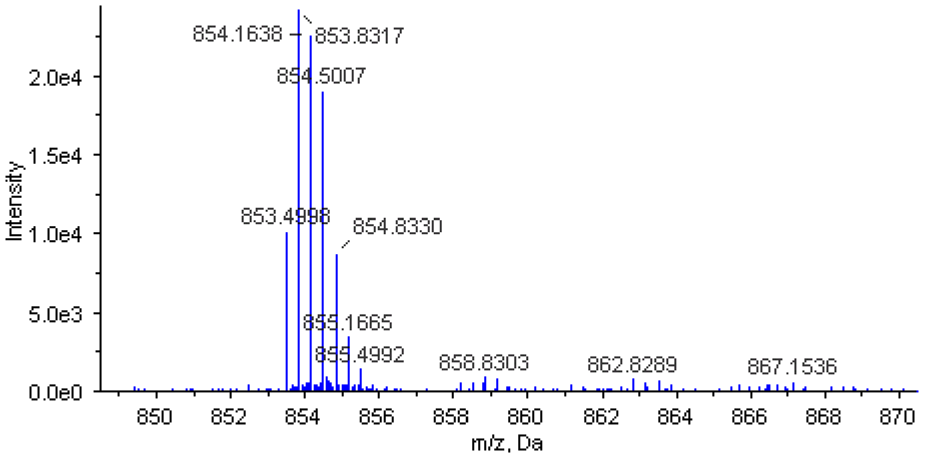

Sequence Coverage and Matched peptides: in different color

MRFWVFTILSLSLSVLGSA**EQCGR**QAGGALCPGGQCCSQYGWCGTTNEYCGQGCQSQCGGSGGGGDIGG
IISRDRFNDILKHRNDNACEGKGFYSHDAFLTAAKAYPAFGTTGDDATRKREIAAFLAQTSHETTGGWP
SAPDGPYSWGYCFVRERNPPSSYCDPNYPCPKQYYGRGPLQLSWNYNYAQAGR**AIGVDLINNPDLVATD
AVISFK**TAIWFWMTAQSPKPSCHDVIIGSWRPSGADTAAGR**VSGYGVITNIINGGIECGK**GQNPQVEDR
IGFFRRYCQILGTGTGNNLDCYNQRPFGARLLLDTI

Protein No.: I-618

Protein name and Species:

HEV1.2 OS=Hevea brasiliensis PE=4 SV=1

Accession: tr|Q6JYQ9|Q6JYQ9_HEVBR

Unused ProtScore: 4

Seq Cov %: 64.6

ITRAQ Changed Ratio:

117: 115=E3TL: D3TL=0.60

116: 114=E5TL: D5TL=0.33

Annotated typical Precursor MS Region:

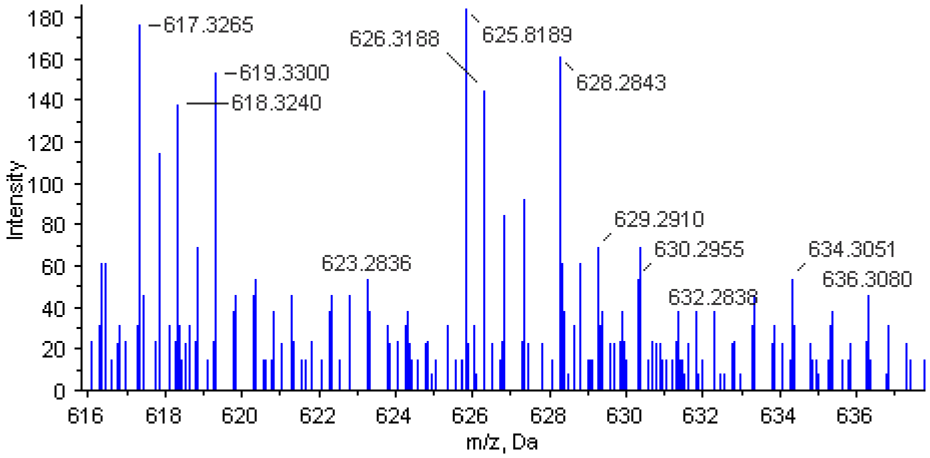

Sequence Coverage and Matched peptides: in different color

MNICMVVLLCLTGVAIAEQCGRQAGGKLCPNNLCCSQYGWCGSSDDYCSPSKNCQSNCKGGGGGGGGSG
GSASNVLATYHLYNPQQHGWDLNAVSAYCSTWDANKPYSWRSKYGWTAFCGPVGAHGQPSCGKCLSVTN
TGTGAKTTVRIVDQCSNGGLDLDVNVFRQLDTDGKGYERGHLTVNYQFVDCGDSFNPLFSIVKSSVIN

Protein No.: I-625

Protein name and Species:

Pyruvate kinase OS=Ricinus communis GN=RCOM_0248610 PE=3 SV=1

Accession: tr|B9ST42|B9ST42_RICCO

Unused ProtScore: 4

Seq Cov %: 14.1

ITRAQ Changed Ratio:

117: 115=E3TL: D3TL=0.65

116: 114=E5TL: D5TL=1.05

Annotated typical Precursor MS Region:

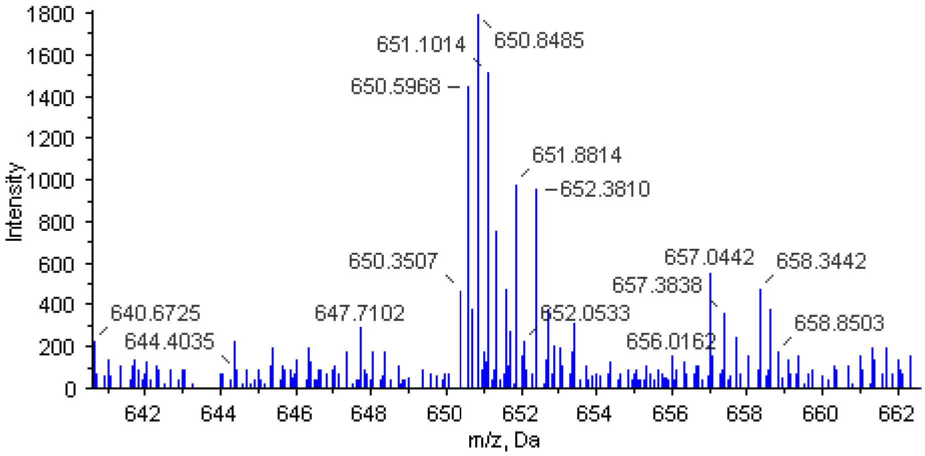

Sequence Coverage and Matched peptides: in different color

MQGGHMVVEEPLRLSSILTPSKPSYLPSLTKIVGTLGPKSRSVETIEACLNAGMSVARFDFSWLDVDYH
QETLNNLRQAMRNAKKLCAVMVDTVGPELPICNQTVKQIELK**ADNRVTITSDVSK**EPSAEILPVNFSGL
SQILKKGDTVFLGQYLFTGSENTSVWLEVLETNGKDVVCLVKNSATLTGFVLMHISQVLSSLPTLTDND
KKVISEWGSCSNVDIISLSYTRNVEDVRELRAFIK**THSLHETQVFAK**IETVEGLKHFDEILQEADGIIL
GR**GNLGIDLPPEK**VFLFQKSAVYK**CNMAGKPVIISR**VVDSMTANLRPTR**AEATDVANAVLDGADGILLG
AETLR**GLYPIDTIRTVGRICAEAEIVCNHSFHFKRISRHVGEPMSHAESVASSAVRAAIKVKAPLIVVF
TSSGAAPRLIAK**YRPPVPVFAVVIPR**LRSNTLKWSFTGSAEARQLLGVRGVYPILASPLVATSGASSEV
STLKVALDHGKSVGLLKPNDQIVVFEKIGDSSVVRIMQLPS

Protein No.: I-628

Protein name and Species:

TBC1 domain family member 13 OS=Populus trichocarpa GN=POPTRDRAFT_174838 PE=4 SV=1

Accession: tr|B9GFV7|B9GFV7_POPTR

Unused ProtScore: 4

Seq Cov %: 8.4

ITRAQ Changed Ratio:

117: 115=E3TL: D3TL=0.22

116: 114=E5TL: D5TL=0.22

Annotated typical Precursor MS Region:

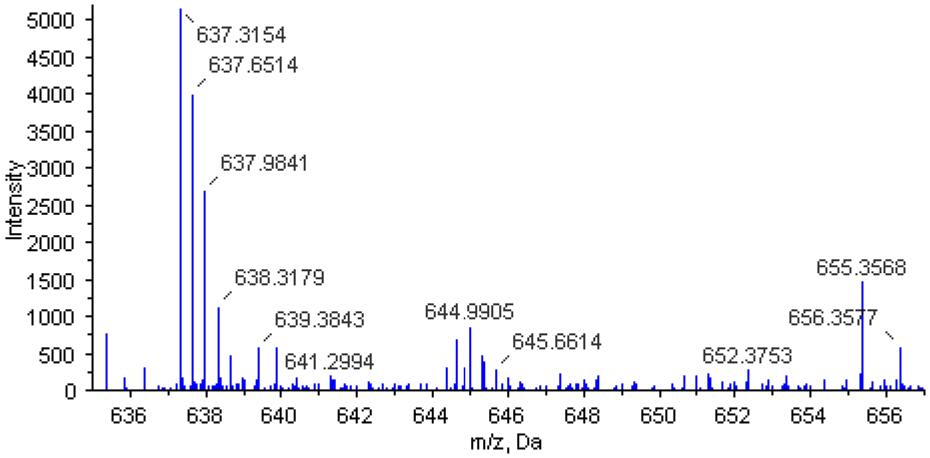

Sequence Coverage and Matched peptides: in different color

SAAEDISKQALLLTELSKKVINMGELRRIASQGIPDGAGIRSTVWK**LLLGYLAPDR**SLWSSELAKKRSQ
YKHFKEDLLMNPSEIARRLEKTTVLNNDNGKSESRCVLSRSEITHGEHPLSLGKSSVWNQFFQDSEIIE
QIDRDVKRTHPDLHFFSGDSSLAKSNQEALRNILTVFAKLNPGIRYVQGMNEILAPLFYIFKNDPDEEM
AACAEADTFFCFVELLSGFR**DHFCQQLDNSVVGIR**STITRLSQLLKEHDEELWRHLEITTKVNPQFYAF
RWITLLLTQEFNFADSLHIWDTLLSDPEGPQETLLRVCCAM**LILIR**RRLLAGDFTSILKLLQNYPPTNI
SHMLYVANKLR

Protein No.: I-631

Protein name and Species:

Cytochrome b5 OS=Camellia sinensis PE=2 SV=1

Accession: tr|F4YFE3|F4YFE3_CAMSI

Unused ProtScore: 4

Seq Cov %: 29.9

ITRAQ Changed Ratio:

117: 115=E3TL: D3TL=3.05

116: 114=E5TL: D5TL=2.04

Annotated typical Precursor MS Region:

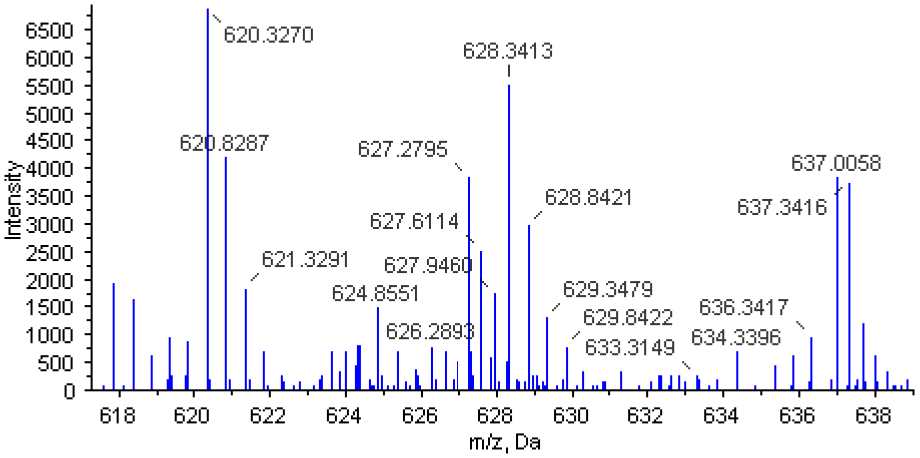

Sequence Coverage and Matched peptides: in different color

MASDPKIHTFEEVAKHNKTKDCWLILSGK**VYDVTPFMDDHPGGDEVLLSSTGKDATNDFEDVGHSDSAR**
EMMEKYYIGGIDSSTVPLKRSYIPPQQSHYKPDKTP**EFVIK**IFQFLVPLLILGLAFGVRYYTKEK

Protein No.: I-634

Protein name and Species:

Putative uncharacterized protei OS=Glycine max PE=2 SV=1

Accession: tr|C6TGB0|C6TGB0_SOYBN

Unused ProtScore: 4

Seq Cov %: 5.7

ITRAQ Changed Ratio:

117: 115=E3TL: D3TL=0.46

116: 114=E5TL: D5TL=1.44

Annotated typical Precursor MS Region:

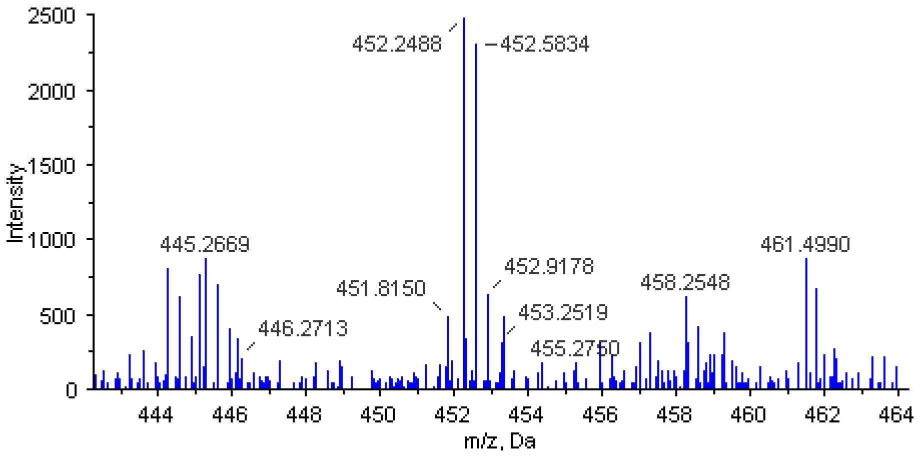

Sequence Coverage and Matched peptides: in different color

MESASELVEFPLLLTPIDSNYRACTIPYRFPSDNPRKPTPTEISWIDLFLNSIPSFKKRAESDTSVPDA
AAKAEKFAQRYADILEDLKKDPESHGGPPDCILLCRLRELVLRELGFRDIFKKVKDEENAKAISLSENV
VH**LNDAIEDEGK**RLENLVRGIFAGNIFDLGSAQLVEVFSKDGMSFLASCQNLVPRPWVIDDLDTFKLKW
SKNPWKKVIIFVDNSGADIILGIMPFARELLRRGSQVVLAANDLPSINDVTCSELVEIISKLKDEDGQL
VGVSTSNLLIANSGNDLPVIDLTRVSQELAYLASDADLVTLEGMGRGIETNLYAQFKCDSLKIGMVK**HP**
EVAQFLGGRLYDCVFKYNEVSS

Protein No.: I-643

Protein name and Species:

Translation machinery-associated protein 7-like OS=Hevea brasiliensis PE=4 SV=1

Accession: tr|D9IZZ6|D9IZZ6_HEVBR

Unused ProtScore: 4

Seq Cov %: 32.8

ITRAQ Changed Ratio:

117: 115=E3TL: D3TL=0.30

116: 114=E5TL: D5TL=1.26

Annotated typical Precursor MS Region:

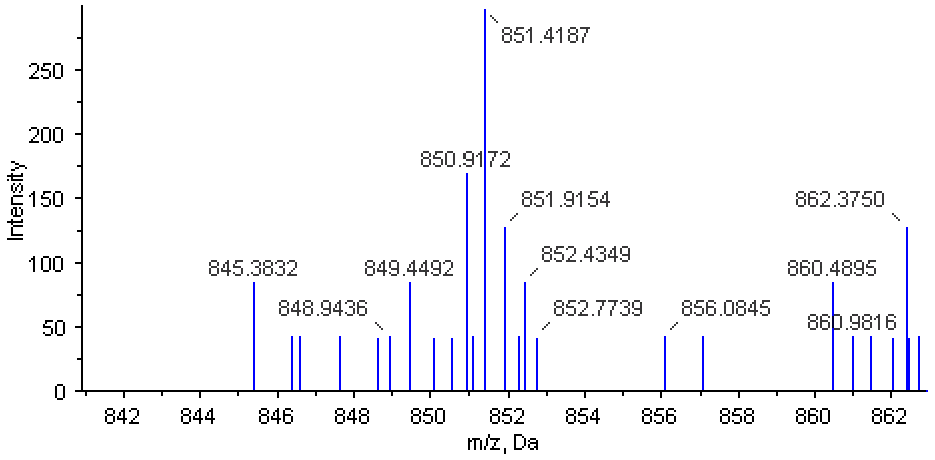

Sequence Coverage and Matched peptides: in different color

MSSKQGGKAKPLEQPKAEKK**DYDETDTANIQK**KKEEEKALKELRAKAAQK**GTFGGSGLK**KSGKK

Protein No.: I-648

Protein name and Species:

20 kDa chaperonin, chloroplastic OS=Vitis vinifera GN=VIT_17s0000g03620 PE=3 SV=1

Accession: tr|D7SIZ7|D7SIZ7_VITVI

Unused ProtScore: 3.96

Seq Cov %: 10.8

ITRAQ Changed Ratio:

117: 115=E3TL: D3TL=0.22

116: 114=E5TL: D5TL=0.59

Annotated typical Precursor MS Region:

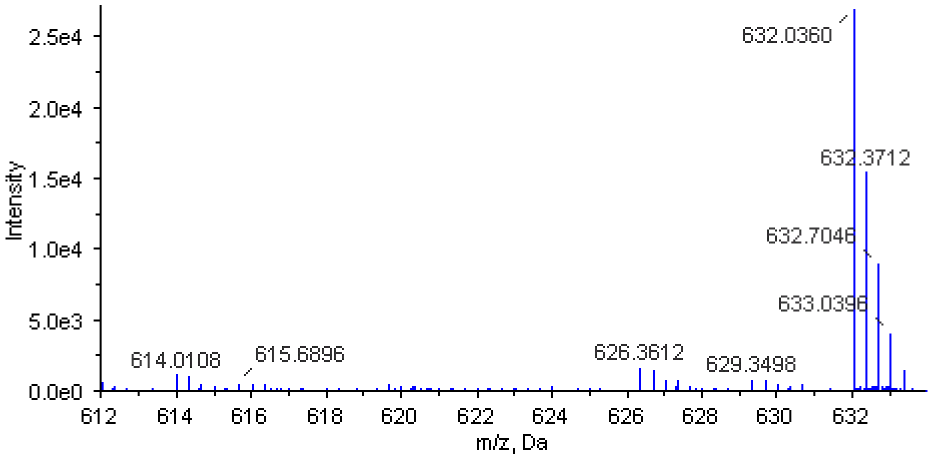

Sequence Coverage and Matched peptides: in different color

MATAPAISTRSLPSFEGLRPSTVRVPLVRLPLRAAGLTQRPVRGLVVRAATSIGPKYTSLKPLADRVLV
KIKTVEEKTGGGILLPTTAQTK**PQGGEVVAVGEGR**TVGKNKVDIGVKTGTSVVYSKYAGTELEFNGSNH
LILK**EDDIVGILETDDIK**DLKPLNDRVLIKVAVAEEKTAGGLLLTEATKEKPSIGTVIAVGPGSLDGEG
NRKPLSLSPGSTIMYSKYAGNEFKGSDGSDYIALRASDVMAVLS

Protein No.: I-650

Protein name and Species:

Ribosomal protein (Fragment) OS=Jatropha curcas PE=2 SV=1

Accession: tr|D1ML39|D1ML39_9ROSI

Unused ProtScore: 3.95

Seq Cov %: 16.5

ITRAQ Changed Ratio:

117: 115=E3TL: D3TL=11.48

116: 114=E5TL: D5TL=2.11

Annotated typical Precursor MS Region:

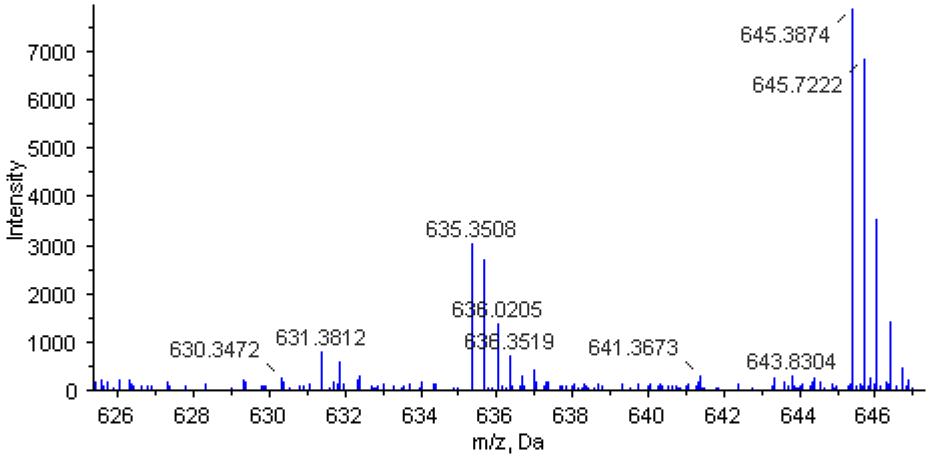

Sequence Coverage and Matched peptides: in different color

QSETLREAISTIMAASVEKKRNFTETIELQ**IGLK**NYDPQKDKCFSGSVKLPHIPRPKMKICMLGDAQHV
EEAEAIGLQWMDVEALKKLNKNKKLVKKLAK**QYHAFLASESVIK**QIPR**LLGPGLNK**AGK**FPTLVTHQES**
LESKVNETKATVKFQLKKVLCMGVAVGNCAMEEKQVFQNVQMSVNFLVSLLKKNWQNVKCLNVKTTMGN
PVRIF

Protein No.: I-651

Protein name and Species:

Calcium lipid binding protein, putative OS=Ricinus communis GN=RCOM_1612600 PE=4 SV=1

Accession: tr|B9RDG1|B9RDG1_RICCO

Unused ProtScore: 3.94

Seq Cov %: 9.3

ITRAQ Changed Ratio:

117: 115=E3TL: D3TL=3.40

116: 114=E5TL: D5TL=2.06

Annotated typical Precursor MS Region:

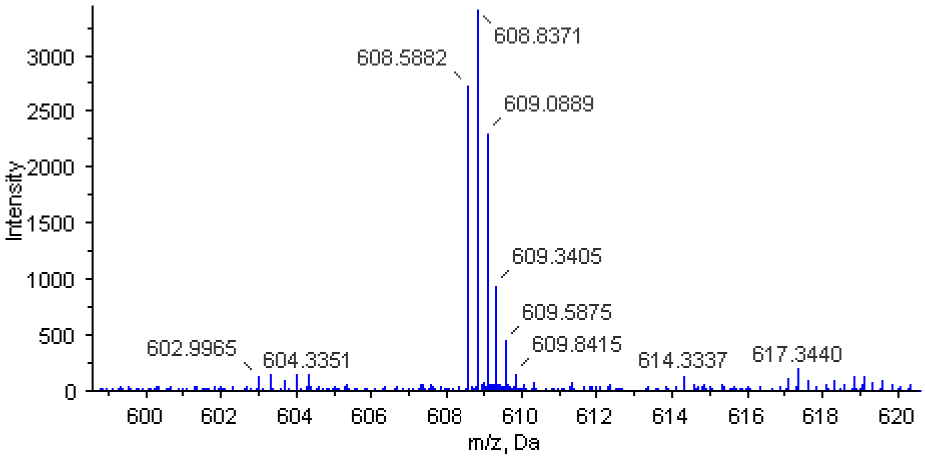

Sequence Coverage and Matched peptides: in different color

MGLISGIFMGMIFGIAVMAGWKHMMRYRSTKRIAKAVDIK**LLGSLNR**DDLKKICGDNFPDWISFPVFEQ
VKWLNKQLSKLWPFVADAATMVVK**ESVEPLLEDYRPPGITSLK**FNKFSLGTVPPKIEGIRVQSLKKGQI
IMDIDFRWGGDPSIILGVEAALVASIPIQLKDLEVYTVIRVIFQLAEEIPCISAVVIALLSEPKPK**IDY**
VLKAVGGSLTAIPGLSDMIDDTVHSIVTDMLQWPHRVVVPIGGIPVDTSELELKPEGK**LTVTVVK**ANDL
KNMEMIGK**SDPYVVLYIR**PMFKVKSRTIENNLNPVWDQTFEMIAEDKETQSLILEVFDKDIGQDKRLGI
AKLPLIELEAETWKQHELRLLPALDMLKIKDKKDGGTLTIKVLYHAFNKEEQLAALEEEKRIIEERKKL
KEAGVIGSTMDALDGAVGLVGTGIASGVGLVGTGIGAGVGLVGTGVGAGVGIVGSGLGAVGSGLSKAGK
FVGRTFTGQSSKR**SGSSTPVNSVQENGGAKPL**

Protein No.: I-653

Protein name and Species:

Quercetin 3-O-methyltransferase 1 OS=Arabidopsis thaliana GN=OMT1 PE=1 SV=1

Accession: sp|Q9FK25|OMT1_ARATH

Unused ProtScore: 3.92

Seq Cov %: 8.3

ITRAQ Changed Ratio:

117: 115=E3TL: D3TL=0.13

116: 114=E5TL: D5TL=0.70

Annotated typical Precursor MS Region:

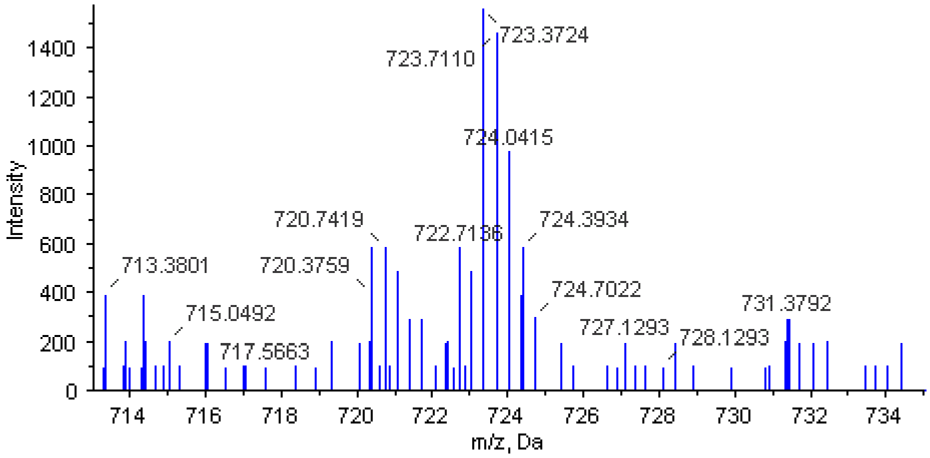

Sequence Coverage and Matched peptides: in different color

MGSTAETQLTPVQVTDDEAALFAMQLASASVLPMALKSALELDLLEIMAKNGSPMSPTEIASKLPTKNP
EAPVMLDRILRLLTSYSVLTCSNRKLSGDGVERIYGLGPVCKYLTKNEDGVSIAALCLMNQDKVLMESW
YHLKDAILDGGIPFNKAYGMSAFEYHGTDPRFNKVFNNGMSNHSTITMKKILETYKGFEGLTSLVDVGG
GIGATLKMIVSKYPNLKGINFDLPHVIEDAPSHPGIEHVGGDMFVSVPKGDAIFMKWICHDWSDEHCVK
FLKNCYESLPEDGKVILAECILPETPDSSLSTKQVVHVDCIMLAHNPGGKERTEKEFEALAKASGFKGI
KVVCDAFGVNLIELLKKL

Protein No.: I-666

Protein name and Species:

glyceraldehyde-3-phosphate dehydrogenase， NADP-dependent OS=Vitis vinifera GN=VIT_11s0037g00070 PE=3 SV=1

Accession: tr|D7U1A1|D7U1A1_VITVI

Unused ProtScore: 3.83

Seq Cov %: 44.6

ITRAQ Changed Ratio:

117: 115=E3TL: D3TL=0.24

116: 114=E5TL: D5TL=0.99

Annotated typical Precursor MS Region:

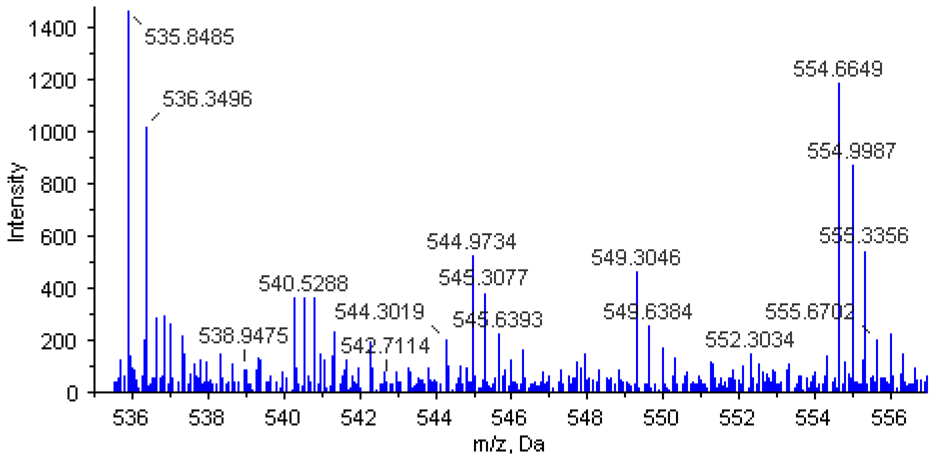

Sequence Coverage and Matched peptides: in different color

MAGTGVFAEIIDGDVYKYYSEGEWRKAVSGK**SVAIINPTTR**KTQYR**VQACSQEEVNK**AMEIAKSAQKIW
AKTPLWKR**AELLHK**AAAILKEHK**APIAECLVK**EIAKPAK**DAVTEVVR****SGDLVSYTAEEGVR**ILGEGK**FL**
VSDSFPGNERSKYCLTSK**IPLGVILAIPPFNYPVNLAVSK**IAPALIAGNSIVLKPPTQGAVSALHMVHC
FHLAGFPKGVISCVTGK**GSEIGDFLTMHPGVN**CISFTGGDTGVAISKK**AGMIPLQMELGGK**DACIVLED
ADLDLVAANIVK**GGFSYSGQR**CTAVKVVLAMESIADTLVEKVNAKVAKLTVGPPEDDCDITPVVSESSA
NFIEGLVMDAKQK**GATFCQEYRR**EGNLIWPLLLDNVRPDMR**IAWEEPFGPVLPVLRINSVEEGIHHCNA**
SNFGLQGCVFTRDINK**AILISDAMETGTVQINSAPARGPDHFPFQGLKDSGIGSQGITNSINMMTK**IK**S**
TVINLPSPSYTMG

Protein No.: I-668

Protein name and Species:

Neutral leucine aminopeptidase preprotein OS=Solanum lycopersicum GN=LapN PE=2 SV=1

Accession: tr|Q8GZD8|Q8GZD8_SOLLC

Unused ProtScore: 3.82

Seq Cov %: 13.7

ITRAQ Changed Ratio:

117: 115=E3TL: D3TL=0.50

116: 114=E5TL: D5TL=1.13

Annotated typical Precursor MS Region:

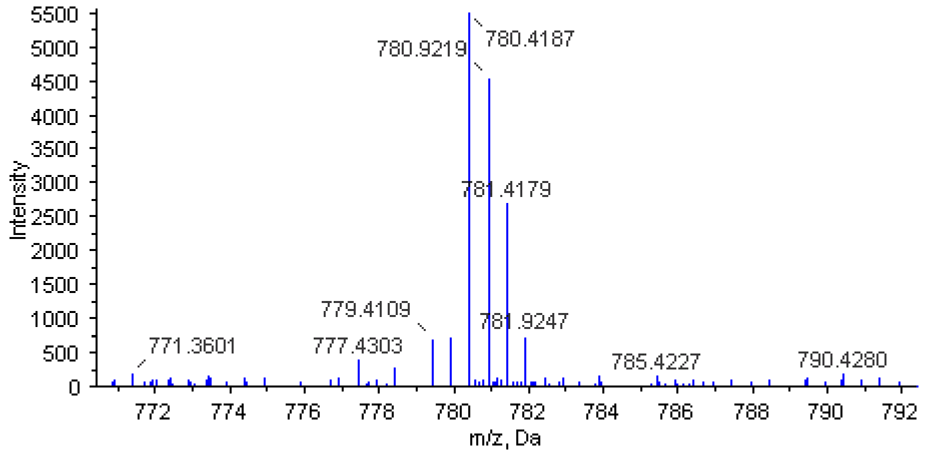

Sequence Coverage and Matched peptides: in different color

MAALRVSSALACSSSSSFHSYPSIFTKFQSSPIWSFSISVTPLCSRRAKRMAHSIARDTLGLTHTNQSD
APKISFAAKEIDLVEWKGDILTVGATEKDLARDGNSKFQNPLLQKLDSKLSGLLSEASSEEDFSGKAGQ
STILR**LPGLGSK**RIALVGLGSPTSSTAAYRCLGEAAAAAAKSAQASNIAIALASTDGLSAELKLSSASA
ITTGAVLGTFEDNRFKSESKKPTLK**SLDILGLGTGPEIEK**KIKYAADVCAGVILGRELVNAPANVLTPA
VLAEEAKKIASTYSDVFSANILDVEQCKELKMGSYLAVAAASANPAHFIHLCYKPSSGEIKKK**IALVGK**
GLTFDSGGYNIKTGPGCSIELMKFDMGGAAAVLGAAKALGQIKPAGVEVHFIVAACENMISGTGMRPGD
IITASNGK**TIEVNNTDAEGRLTLADALVYACNQGVEK**IVDLATLTGACVVALGPSIAGIFTPSDDLAKE
VVAASEVSGEKLWRLPMEDSYWDSMK**SGVADMVNTGGR**PGGAITAALFLKQFVNEKVQWMHIDLAGPVW
SDKKKNATGFGVSTLVEWVLKNSTN

Protein No.: I-680

Protein name and Species:

hydrolase domain-containing protein 4-like [Vitis vinifera] OS=Vitis vinifera GN=VIT_18s0001g13610 PE=4 SV=1

Accession: tr|E0CPI5|E0CPI5_VITVI

Unused ProtScore: 3.74

Seq Cov %: 14.4

ITRAQ Changed Ratio:

117: 115=E3TL: D3TL=9.46

116: 114=E5TL: D5TL=2.53

Annotated typical Precursor MS Region:

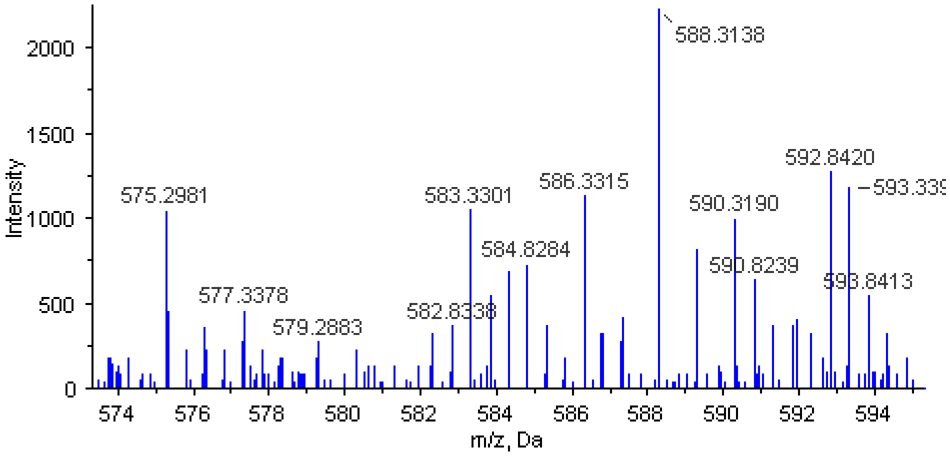

Sequence Coverage and Matched peptides: in different color

MAEEMTKAELGSSSSTTKSRSLWPSLFRWIPTSTDHIIDAEKR**LLSLVK****TPYVQEQVNIGSGPPGSK**VR
WFRSASNEPR**FINTVTFDSK**DDSPTLVMVHGYGASQGFFFRNFDALARRFR**VIAIDQLGWGGSSRPDFT**
CKSTEETEAWFIDSFEEWRKAKNLSNFILLGHSVGGYVAAKYALKHPEHIQHLILVGPAGFSLESDGKS
EWLTRFRATWKGAVLNHLWESNFTPQKLVRGIGPWGPDLVRKYTSARFSSYSTGDLLTEEESKLLTDYV
YHTVAAK**ASGELCLK**YIFSFGAFARLPLLHSASEWKVPTTFIYGFEDWMNYQGAQEARKQMKVPCEIIR
VPQAGHFVFIDNPSGFHSAVLYACRRFLLPDPDSQSLPEGLTSA

Protein No.: I-683

Protein name and Species:

bifunctional polymyxin resistance protein; UDP-D-apiose/UPD-D-xylose synthetase [Vitis vinifera] OS=Vitis vinifera

Accession: tr|D7SN69|D7SN69_VITVI

Unused ProtScore: 3.7

Seq Cov %: 26.6

ITRAQ Changed Ratio:

117: 115=E3TL: D3TL=3.31

116: 114=E5TL: D5TL=2.04

Annotated typical Precursor MS Region:

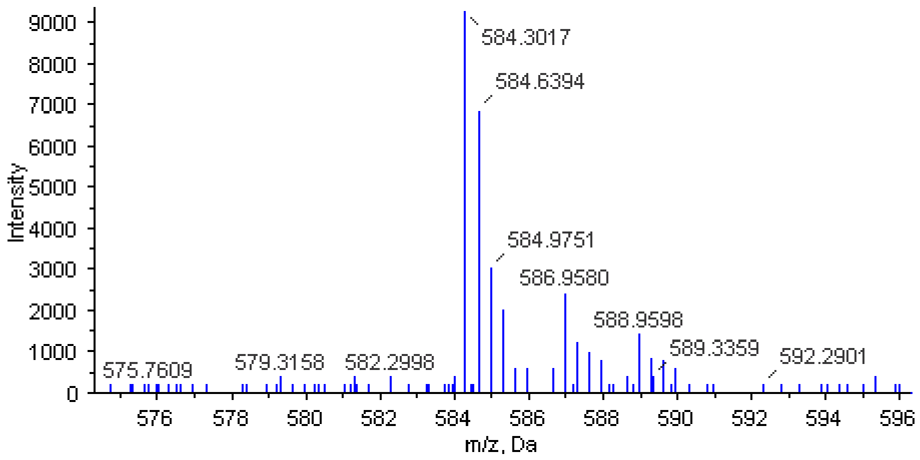

Sequence Coverage and Matched peptides: in different color

MASAARLDLDGNAIKPMTIC**MIGAGGFIGSHLCEK**LMAETMHKVLAVDVYSDKIKHLLEPSTHPWSDRI
QFHRINIKHDSR**LEGLIK****MADLTINLAAICTPADYNTR**PLDTIYSNFIDALPVVKYCSENNKR**LIHFST**
CEVYGK**TIGSFLPK**DSPLWQDPTYYVLK**EDASPCIFGPIEK**QRWSYACAKQLIERLIYAEGAENDLEFT
IVRPFNWIGPR**MDFIPGIDGPSEGVPR**VLACFSNNLLRHEPLKLVDGGQSQRTFVYIKDAIEAVLLMID
NPAR**ANGHIFNVGNPNNEVTVR**QLAEMMTEVYAKVSGEPSLEVPTVDVSSK**EFYGEGYDDSDKR**IPDMT
IINKQLGWNPKTSLWDLLESTLTYQHRTYAEAIKQAIAKPVAS

Protein No.: I-687

Protein name and Species:

V-type proton ATPase subunit F OS=Hevea brasiliensis GN=VHA-F PE=2 SV=1

Accession: tr|B8Y6I1|B8Y6I1_HEVBR

Unused ProtScore: 3.68

Seq Cov %: 21.5

ITRAQ Changed Ratio:

117: 115=E3TL: D3TL=1.69

116: 114=E5TL: D5TL=1.54

Annotated typical Precursor MS Region:

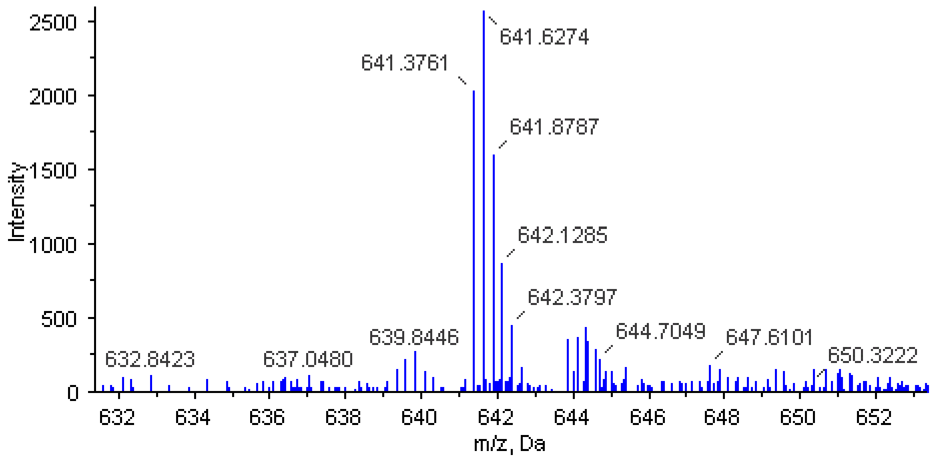

Sequence Coverage and Matched peptides: in different color

MANRVQIPTSNSALIAMIADEDSVVGFLLAGVGNVDLRRKSNYLLVDSKTTVKQIEDAFKDFTTREDIA
IVLISQYVANMVRFLVDSYNKPVPAILEIPSKDHPYDPAQDSVLSRVKHLFSAESVASERR

Protein No.: I-688

Protein name and Species:

argininosuccinate lyase [Vitis vinifera] OS=Vitis vinifera GN=VIT_18s0001g07590 PE=3 SV=1

Accession: tr|E0CRW7|E0CRW7_VITVI

Unused ProtScore: 3.66

Seq Cov %: 8

ITRAQ Changed Ratio:

117: 115=E3TL: D3TL=0.54

116: 114=E5TL: D5TL=0.47

Annotated typical Precursor MS Region:

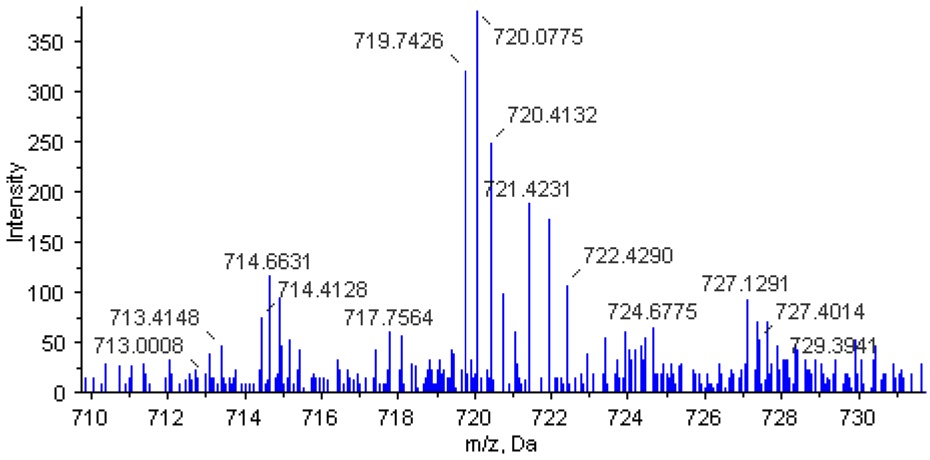

Sequence Coverage and Matched peptides: in different color

MKTSATDPSTKKAKEAKLWGGRFEEGVTDAVER**FTESISFDK**ALYKQDIMGSRAHASMLAHQGLISVSD
RDSILQGLDNIERRIEDGEFVWRTDREDVHMNIEAALTDLIGEPAKKLHTARSRNDQVLTDFRLWCRDA
IDNIVARIEHLQVALVTLAMKNENLIVPGYTHLQRAQPILLQHLLLAYVEQLDRDAGRLLDCRQR**LNFC**
PLGACALAGTGLPIDRFMTSHDLGFNAPMRNSIDAVSDRDFVLEFLSANSITAIHLSRLGEEWVLWASE
EFGFLTPSDSVSTGSSIMPQKKNPDPMELVRGKSARVVGDLVTLLVLCKGLPHAYNRDLQEDKEPTFDS
TKTIIGMLEVCAEFAQNITFNREKIKK**ALPAGHLDATTLADYLVK**KGIPFRTSHDIVGKSVALCVSK**NC**
QLQDLSLDELRSVNPVFDEDVYEFLGVENAVKKFCSYGSTGSECVAEQLDFWVTKLQINTAGHK

Protein No.: I-692

Protein name and Species:

Homogentisate 1,2-dioxygenase, putative OS=Ricinus communis GN=RCOM_1743950 PE=4 SV=1

Accession: tr|B9RXB8|B9RXB8_RICCO

Unused ProtScore: 3.63

Seq Cov %: 7

ITRAQ Changed Ratio:

117: 115=E3TL: D3TL=0.09

116: 114=E5TL: D5TL=0.19

Annotated typical Precursor MS Region:

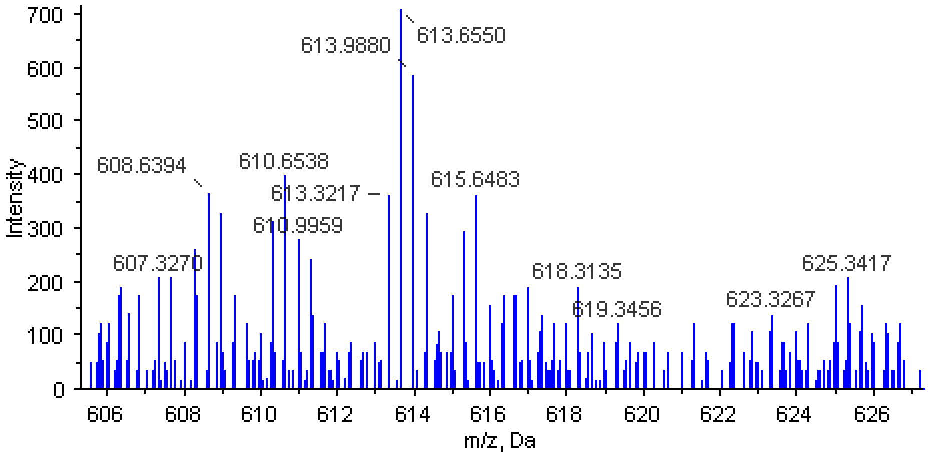

Sequence Coverage and Matched peptides: in different color

MDNPKSNGRDFPSDDHDYLSGFGNTFESEAIHGALPRGQNSPLICPY**GLYAEQISGSSFTSPR**KLSQRS
WLYRIKPSVTHEPFKPRVPSHGK**IVSEFDK**TDSCTTTPTQLRWKPVDIPDSPTDFIDGLFTICGAGSSF
LRHGFAIHMYTANKSMGNCALCNADGDFLVVPQEGRLWITTECGKLQVSPGEVVVLPQGFRFAVDLPDG
PSRGYVAEIFGTHFQLPDLGPIGANGLAAPRDFLVPKAWYEEGPCPGYTIIQK**FGGELFTAK**QDFSPFN
VVAWHGNFVPYKYDLKKFCPYNTVLIDHSDPSINTVLTASTDKPGVALLDFVIFPPRWLVAEHTFRPPY
YHRNCMSEFMGLIYGGYEAKADGFVPGGASLHSCMTPHGPDTKTYEATIARGNDAGPSRITDTMAFMFE
SCLIPRICLWAVESPFIDHDYYQCWIGLKSHFSHGADSKNGGI

Protein No.: I-694

Protein name and Species:

3-isopropylmalate dehydratase, putative OS=Ricinus communis GN=RCOM_1217310 PE=4 SV=1

Accession: tr|B9SQS5|B9SQS5_RICCO

Unused ProtScore: 3.61

Seq Cov %: 8.2

ITRAQ Changed Ratio:

117: 115=E3TL: D3TL=0.48

116: 114=E5TL: D5TL=0.67

Annotated typical Precursor MS Region:

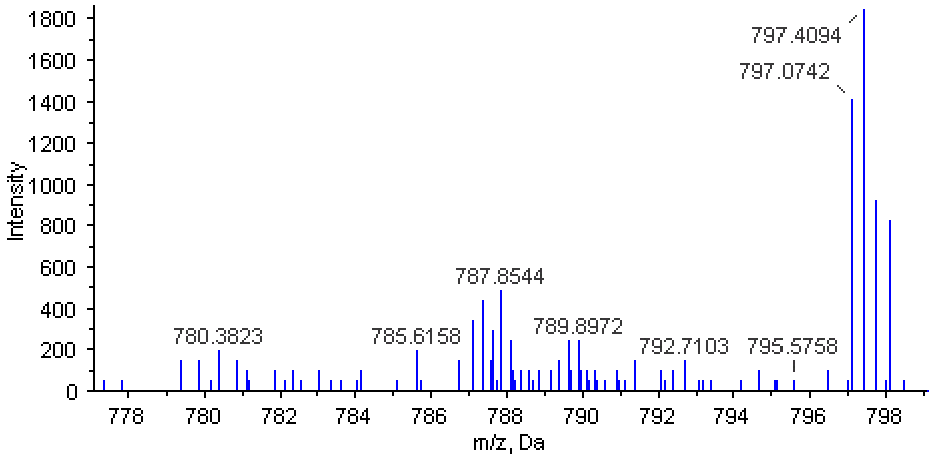

Sequence Coverage and Matched peptides: in different color

MASSAITSPSSS**FITK**KDLGLSAFSSQSSPFYSIPKCKKSISKKVVSVMAPQQSERTPATTGSVKTAMT
MTEKILARASEKSQLSPGENVWVNVDVLMTHDVCGPGSIGIFKREFGQNATVWDREK**IVIIPDHYIFTT**
DERANRNVDILRDFCQEQNVKYFYDIK**DLSNFK**VNPDYKGVCHVALAQEGHCRPGEVLLGTDSHTCTAG
AFGQFATGIGNTDAGFVLGTGKLLLKVPPTLRFVMDGEMPDYLLAKDLILQIIGEISVSGATYKSMEFV
GTTVESLNMEER**MTLCNMVVEAGGK**NGVIPSDSTTFKYLEDKTSVPFEPVYSDEKARFLSEYRFDVSKL
EPLVAKPHSPDNCALARECKDVKIDRVYIGSCTGGKTEDFMAAAKVFLASGKKVKVPTFLVPATQKVWM
DVYSLPVPGSGGK**TCSQIFEEAGCDTPASPSCGACLGGPK**DTYARMNEPMVCVSTTNRNFPGRMGHKEG
QIYLASPYTAAASALTGYVTDPREFLQ

Protein No.: I-700

Protein name and Species:

Protein-L-isoaspartate O-methyltransferase OS=Ricinus communis GN=RCOM_1508020 PE=3 SV=1

Accession: tr|B9RAR0|B9RAR0_RICCO

Unused ProtScore: 3.54

Seq Cov %: 15.2

ITRAQ Changed Ratio:

117: 115=E3TL: D3TL=0.61

116: 114=E5TL: D5TL=0.87

Annotated typical Precursor MS Region:

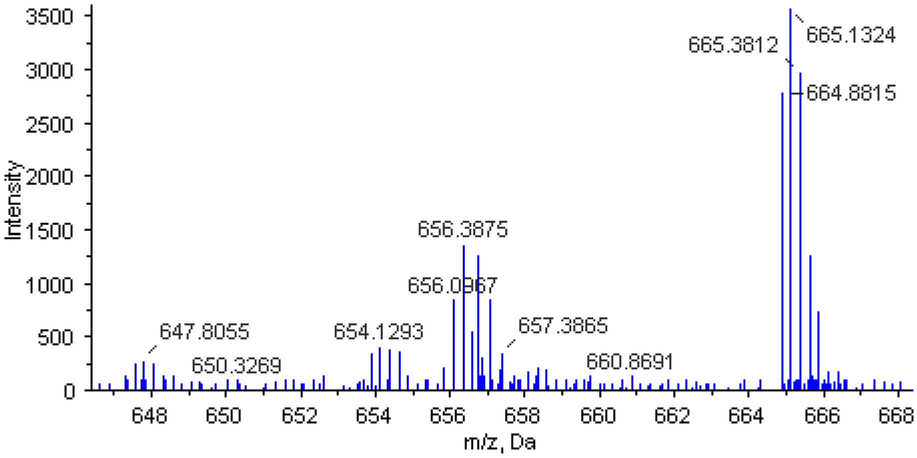

Sequence Coverage and Matched peptides: in different color

MPISPVIAYGCRNYYYVPVPPLKRILTYSFRKNHLHHNNSHRCF**LLLLR**TTTNFHPQFSTFNLIPRKLY
CPFTGNSLFFRMERFFAGSGINKNKAMVEHLQHYGVISSRKVVEVMETIDRALFVPDGTQAYVDSPMPI
GYNATISAPHMHATCLQLLEEHLKPGMHALDVGSGTGYLTACFAIMVGPQGR**AVGVEHIPELVTSSVK**N
ILKSAAAPLLKEGFLSVHIGDGRQGWPEFAPYDAIHVGAAAPEIPQALIDQLKPGGR**MVIPVGNIFQDL**
KVIDKNQDGSISVRSETSVRYVPLTSRDAQLRG

Protein No.: I-703

Protein name and Species:

Heat shock protein 17.5 cytosolic class II OS=Jatropha curcas PE=2 SV=1

Accession: tr|D5JG84|D5JG84_9ROSI

Unused ProtScore: 3.53

Seq Cov %: 19.8

ITRAQ Changed Ratio:

117: 115=E3TL: D3TL=1.50

116: 114=E5TL: D5TL=1.34

Annotated typical Precursor MS Region:

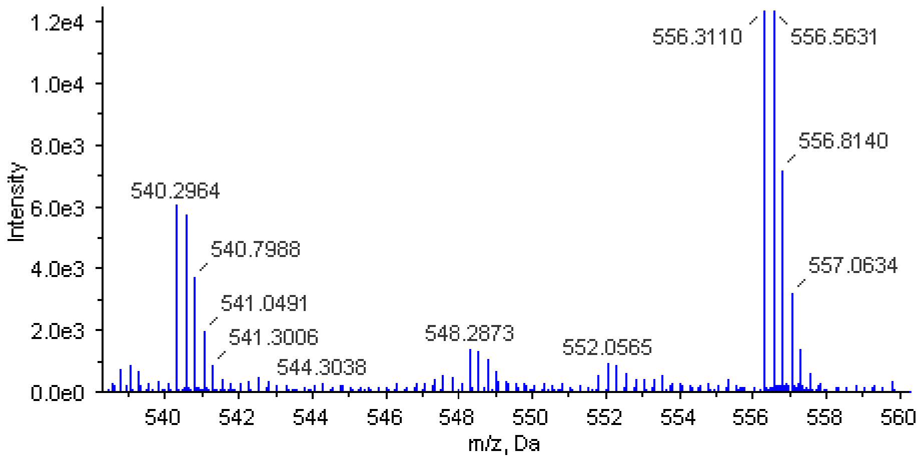

Sequence Coverage and Matched peptides: in different color

MDVRFVGLDSPLLHTLQQMMDIGDDADHKSFNAPTRTYVRDAKAMASTPADVKEYPNSYVFIIDMPGLK
SGDIKVQVEDDNVLLISGERKREEEKEGAKYVRMERRVGKLMRKFALPENANTDAISAVCQDGVLTVTV
QKLPPPEPKKPKTIEVKIA

Protein No.: I-704

Protein name and Species:

Putative uncharacterized protein OS=Vitis vinifera GN=VIT_13s0067g03420 PE=4 SV=1

Accession: tr|D7T494|D7T494_VITVI

Unused ProtScore: 3.52

Seq Cov %: 3

ITRAQ Changed Ratio:

117: 115=E3TL: D3TL=0.10

116: 114=E5TL: D5TL=0.35

Annotated typical Precursor MS Region:

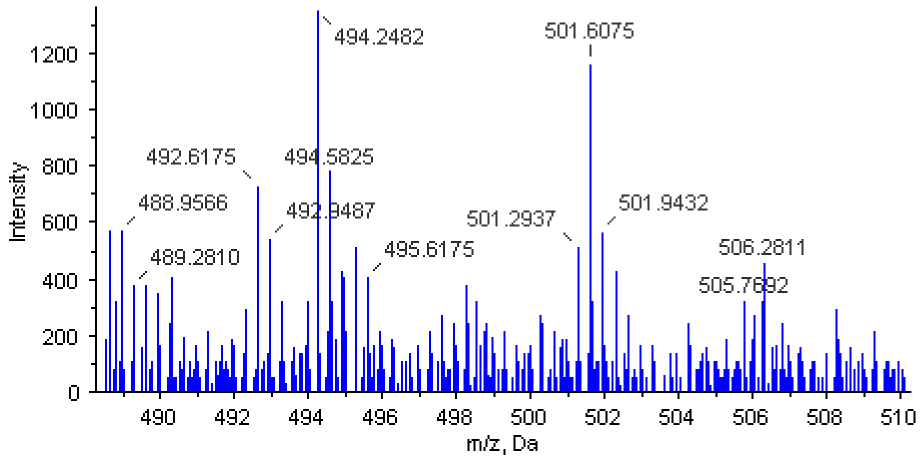

Sequence Coverage and Matched peptides: in different color

MKPKAVVNPLVTFEHKRDAYGFAVRPQHLQRYR**EYANIYKEEEEER**SERWNIFLEQQAESAQLPVNGLS
ADEHNKALHGEATEKDVDANPEKVVQKLGSDDSNENVTEKESQGVAETKTHRIQIWTEIRTSLHAIEEM
MSTRVKKRRDSSKNEKETGLGKHHAPVEEARSLKGVSEEDSEDEFYDVERSDPVQDVPSSDSSNASATA
SAGDVVTLETSFPWKEELECLVRGGVPMALRGELWQAFVGVKARRVERYYQELLASEHNVGNKVEQDSS
QTDSLTDGPIKDSLTVTEKWKGQIEKDLPRTFPGHPALDEDGRNALRRLLTAYARHNPSVGYCQAMNFF
AGLLLLLMPEENAFWALMGIIDDYFDGYYSEEMIESQVDQLAFEDLVRERLPKLVNHLDFLGVQVAWVT
GPWFLSIFMNMLPWESVLRVWDVLLFEGNRVMLFKTALALMELYGPALVTTKDAGDAVTLLQSLAGSTF
DSSELVLTACMGYQNVNEARLQELRDKHRAAVIAAVEERSKGLRAWRDSKGLAHKLYGFKHDPGSLAMD
ANQTEQVVDSQANGDMSHMEPGSANVDGFLIGLTENVEIDSVPDLQEQVRWLKVELCKLLEEKRSALLR
AEELETALMEMVKQDNRRQLSAR**VEQLEQEVSELR**QALADKQEQEHAMLQVLVRVEQEQKLTEDARRFA
EQDAAAQRYAAQVLQEKYEEAITSLAQMEKRVVMAETMLEATLQYQSGQVKAQPSPRSHQDSSSARSNQ
ETPQELPTRKIGLLSRPFALGWRDRNKGKPASEEVSDAKPTNEVENPSAQQVESPSTQQKDANGHEVQE
KQ

Protein No.: I-708

Protein name and Species:

Hydrolase, hydrolyzing O-glycosyl compounds, putative OS=Ricinus communis GN=RCOM_0272270 PE=4 SV=1

Accession: tr|B9T139|B9T139_RICCO

Unused ProtScore: 3.46

Seq Cov %: 6.5

ITRAQ Changed Ratio:

117: 115=E3TL: D3TL=16.00

116: 114=E5TL: D5TL=3.72

Annotated typical Precursor MS Region:

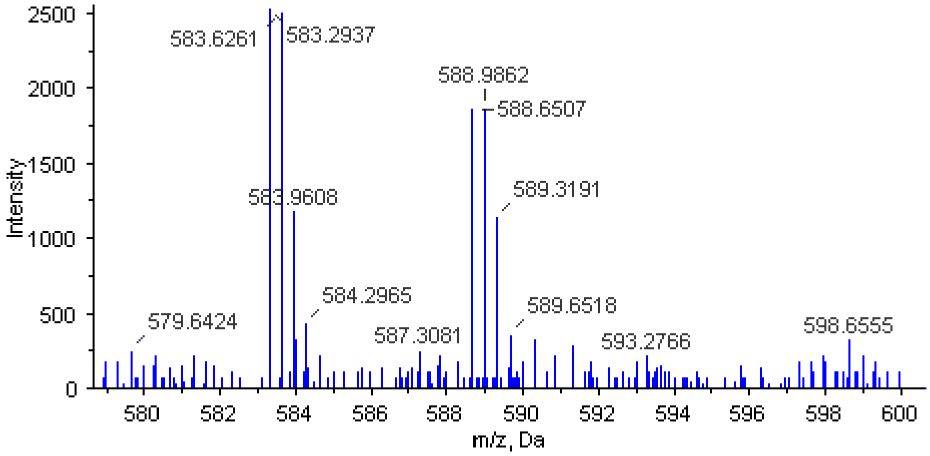

Sequence Coverage and Matched peptides: in different color

MAWSLQKSEIKPTVRKLEKKGGDRQLDAMTIKPAVRISDRKLIVKDRTILTGLQDNVIATSGSSSSPVE
GVFIGAVFDEENSRHVVPLGTLRDVRFMACFRFKLFWMAQKMGDHGRDIPLETQFLMMETKDGSQLESD
GGNEENQIIYTVFLPLIEGSFRACLQGNDNDELELCLESGDVDTKAASFTHPLFIHAGTDPFGTLTEAV
RAVKLHLKSFRQRHEKKLPAIIDYFGWCTWDAFYQEVTQEGVEAGLKSLSEGGTLPKFVIIDDGWQSVG
GDPQEDDEDKPQPLLRLIGIKENEKFRKKDDPTVGIKNIVNIAKEKYGLKYVYVWHAITGYWGGVRPGV
KEMEEYGSLMKYPKVSEGVLENEPTWRTDVLAVQGLGLMNPKAVYKFYNELHNYLASAGIDGVK**VDVQC**
ILETLGAGLGGRVEITR**QYHQALDASVAR**NFPDNGCIACMSHNTDALYCSKQTAVVR**ASDDFFPR**DPVS
HTIHIAAVAYNSVFLGEFMQPDWDMFHSLHPAAEYHASAR**AISGGPVYVSDEPGK**HDFNVLKK**LVLPDG**
SILRARLPGRPTRDCLFSDPARDGISLLKIWNMNKHTGVLGVYNCQGAAWNCVERKNTFHETKSEALTG
AIKGRDVHLIAEAATDSNWNGDCAVYCHQTAELTTVPYNASLPVSLKVLEHEIFTLTPIKVLAPGFSFA
PLGLIAMYNAGGAIEGLKYEVKGVKLVELDEGYKGENSTVSDERVENISSELVGKICMEVKGCGKFGAY
SSTKPRMCIVDSNIAEFEYDSSSGLVTFNLDNLAEEGRLHLVEVEV

Protein No.: I-710

Protein name and Species:

ATP-citrate synthase, putative OS=Populus trichocarpa GN=POPTRDRAFT_827990 PE=4 SV=1

Accession: tr|B9N432|B9N432_POPTR

Unused ProtScore: 3.44

Seq Cov %: 17.3

ITRAQ Changed Ratio:

117: 115=E3TL: D3TL=0.56

116: 114=E5TL: D5TL=0.61

Annotated typical Precursor MS Region:

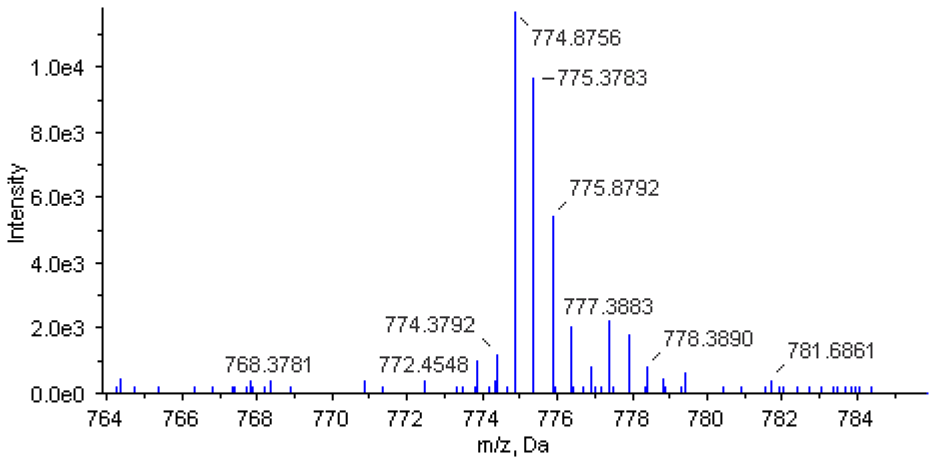

Sequence Coverage and Matched peptides: in different color

MARKKIREYDSKRLLKEHLKRLSGIDLQICSAQVTEGTDFTELTNKEPWLSSTK**LVVKPDMLFGK**RGKS
GLVALNLDLAQVAEFVKARLGAEVEMGGCKAPITTFIVEPFVPHDQEFYISIVSERLGSTISFSECGGI
EIEENWDKVKTIFLPTEKPMTLEACAPLIATLPLEIRGKIGDFIISVFSVFQDLDFSFLEMNPFTLVNG
EPYPLDMR**GELDDTAAFKN**FKKWGNVEFPLPFGRVLSSTESFIHSLDEKTSSSLK**FTVLNPK**GRIWTMV
AGGGASVIYADTVGDLGYASELGNYAEYSGAPNEDEVLQY**ARVVIDCATSDPDGR**KRALLIGGGIAN**FT**
DVAATFNGIIRALREKESKLKAARMHIYVRR**GGPNYQTGLAK**MR**TLGEELGVPLEVYGPEATMTGICK**Q
AIDCIMSTA

Protein No.: I-713

Protein name and Species:

Potassium channel beta, putative OS=Ricinus communis GN=RCOM_1021600 PE=4 SV=1

Accession: tr|B9RWM3|B9RWM3_RICCO

Unused ProtScore: 3.44

Seq Cov %: 7.6

ITRAQ Changed Ratio:

117: 115=E3TL: D3TL=12.13

116: 114=E5TL: D5TL=2.13

Annotated typical Precursor MS Region:

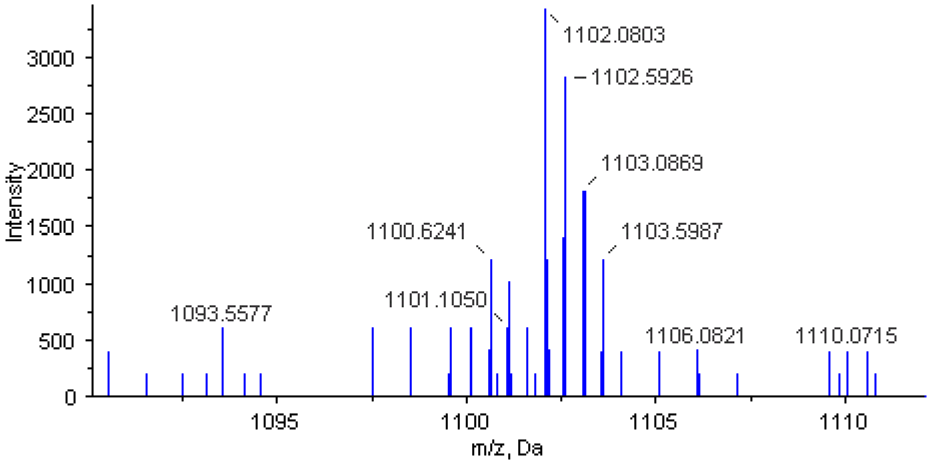

Sequence Coverage and Matched peptides: in different color

MQYKNLGRSGLKVSQLSYGAWVSFGNQLDVKEAK**SLLQCCR**DHGVNFFDNAEVYANGRAEEIMGQAIRE
LGWKRSDIVVSTKIFWGGSGPNDKGLSRKHIVEGTKASLKRLDMDYVDVIYCHRPDSSTPIEETIRAMN
HVIDKGWAFYWGTSEWSAQQITEAWGIAER**LDLVGPIVEQPEYNLLSR**HKVESEYLPLYTNYGLGLTTW
SPLASGVLTGKYSRGAIPPDSRFALENYKNLASRSLIDDVLKKVNGLKPIADELGVPLSQLAIAWCAAN
PNVSSVITGATKESQIQENMKAIDVIPLLTPAVMEKIEAVVQSKPKRPDSYR

Protein No.: I-714

Protein name and Species:

Aldehyde dehydrogenase family 7 member A1 OS=Euphorbia characias PE=2 SV=1

Accession: tr|Q5EBY6|Q5EBY6_EUPCH

Unused ProtScore: 3.43

Seq Cov %: 11.2

ITRAQ Changed Ratio:

117: 115=E3TL: D3TL=0.59

116: 114=E5TL: D5TL=0.68

Annotated typical Precursor MS Region:

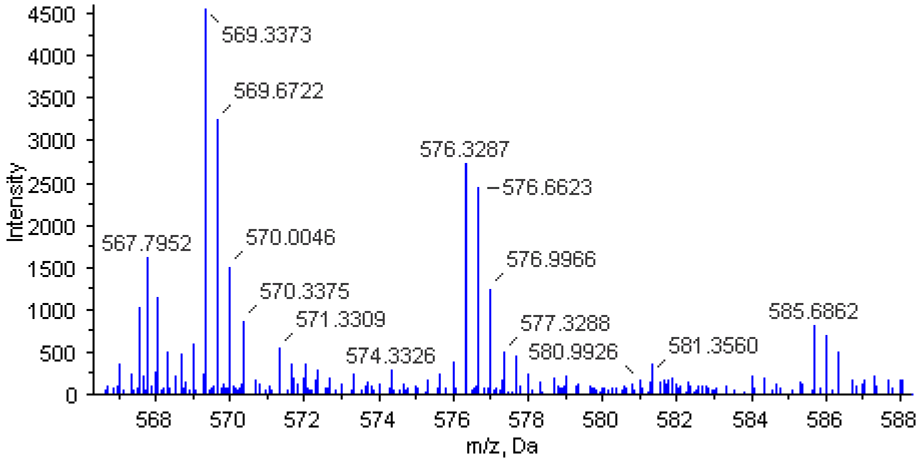

Sequence Coverage and Matched peptides: in different color

MGFARKEYEFLSEIGLSERNLGCYVNGTWKANGPVVTTSNPAN**NQAIAEVVEGSIEDYEEGMK**ACSEAA
KIWMQVPAPKRGDIVRQIGDALRGKLEHLGRLVSLEMGKILAEGIGEVQEIIDMCDFCVGLSRQLNGSI
IPSERPNHAMLEMWNPLGIVGVITAFNFPCAVLGWNACIALVCGNCAVWKGAPTTPLMTIATTKLVAEV
LERNNLPLAIFTSFCGGADIGQAIAKDTR**IPLVSFTGSSK**VGLMVQQTVNQRYGKSLLELSGNNAIIVM
DDADIPLAARSILFAAVGTAGQRCTTCRRLILHEKIYDTVLDQLLKSYKQVKIGDPLEKGTLLGPVHTA
ESRKNFEKG**IELIK**SQGGK**ILTGGSVIESEGNYVQPTIVEISSK**AEVVK**EELFAPVLYVMK**FQTLEEAI
EINNSVPQGLSSSIFTRRPDVIFKWLGPHGSDCGIVNVNIPTNGAEIGGAFGGEKATGGGREAGSDSWK
QYMRASTCTINYGSELPLAQGINFG

Protein No.: I-716

Protein name and Species:

Isoflavone reductase, putative OS=Ricinus communis GN=RCOM_1594880 PE=4 SV=1

Accession: tr|B9R7W5|B9R7W5_RICCO

Unused ProtScore: 3.43

Seq Cov %: 20.1

ITRAQ Changed Ratio:

117: 115=E3TL: D3TL=0.23

116: 114=E5TL: D5TL=0.48

Annotated typical Precursor MS Region:

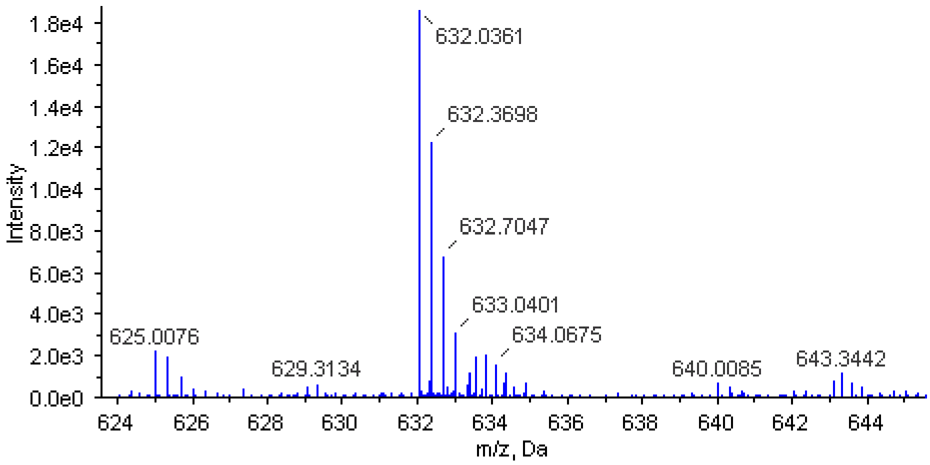

Sequence Coverage and Matched peptides: in different color

MADTSK**ILIIGGTGYIGK**FIVEASAKAGLPTFVLIR**ESTVSDPVK**GKIVENFK**NLGVTLLHGDLYDHES
LVK**AIKQADVVISTLGALQLADQTKVIAAIKEAGNVKR**FFPSEFGTDVDHVHAVEPAK**SAFETKAQIRR
AIEAEGIPYTYAVCNYFASLMIPLLLRPAGDK**VTILGDGNVK**AIFSMEQDIALYTIKAVDDPRTLNKTL
FVNPPMNILTYNELVALEEKKTGKTIEKNYVPEEKVLQDIQTVPMPFNIGLAINHCVFIKGDQTNFEID
PSWGVEASQLYPDVKYTTIAEYFDQTA

Protein No.: I-717

Protein name and Species:

glycine-tRNA ligase 1, mitochondrial OS=Populus trichocarpa GN=POPTRDRAFT_830916 PE=3 SV=1

Accession: tr|B9GZ19|B9GZ19_POPTR

Unused ProtScore: 3.42

Seq Cov %: 10.9

ITRAQ Changed Ratio:

117: 115=E3TL: D3TL=0.61

116: 114=E5TL: D5TL=0.79

Annotated typical Precursor MS Region:

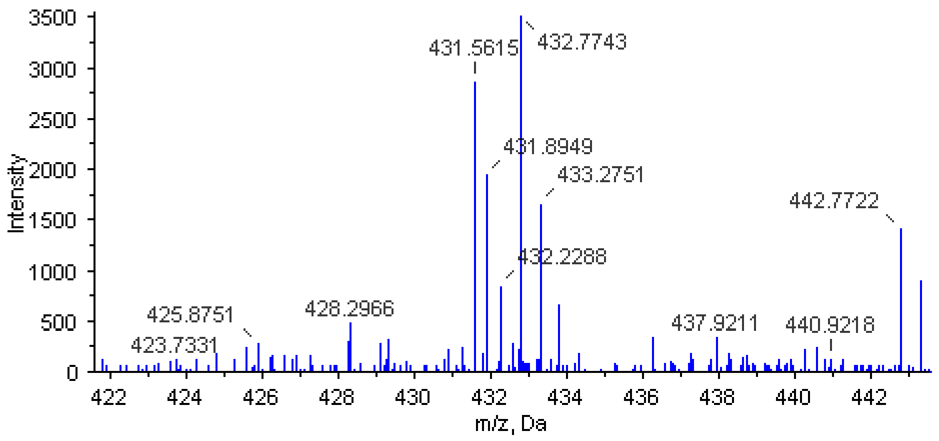

Sequence Coverage and Matched peptides: in different color

MAASEDSLRRALAEKQSSIESQGNTVRALKASNAEKATVDEAIEKLNALKLDKSVMEK**QLQAAVNGNGP**
DGSVNKEAFRQAVVNTLERRLFYIPSFKIYRGVAGLYDYGPPGCAVKSNVLSFWRQHFVLEENMLEVDC
PCVTPEVVLKASGHVDK**FTDLMVK**DEKTGTCCRADHLLKDFCNEKIQKDLSISAEKAAELKHVLAILDD
LSAEELGAKIK**EYGITAPDTK**NPLSDPYPFNLMFQTSIGPSGLSPGYMRPETAQGIFVNFKDLYYYNGN
K**LPFAAAQIGQAFR**NEISPRQGLLRVREFTLAEIEHFVDPEYKSHPK**YSEVADLEFLMFPR**ELQISAQS
AKKIQLGEAVSK**GIVNNETLGYFIGR**VYLFLTRLGIDKDRLRFRQHLANEMAHYAADCWDAEIESSYGW
IECVGIADRSAYDLHAHSEK**SGVPLVAHEK**FSEPK**EVEKLVIAPVK**K**ELGLAFK**GNQKKVVEALEAMNE
KEALDMKASLETKGEVEFYVCTLGEKVTIKKNMVSISKEKKKEHQRTFTPSVIEPSFGIGRIIYCLYEH
SFYMRPSK**AGDEQLNVFR**FPPLVAPIKCTVFPLVQNQQYEDVAKIISKTLTAAGISHK**IDITGTSIGK**R
YARTDELGVPFAITVDSISSVTIRERDSKDQIRVDVEEAAPVVKSVTDGHKTWADVWANFPHHSSGSVE
D

Protein No.: I-718

Protein name and Species:

40S ribosomal protein S25-1, putative OS=Ricinus communis GN=RCOM_1447530 PE=4 SV=1

Accession: tr|B9RH67|B9RH67_RICCO

Unused ProtScore: 3.42

Seq Cov %: 20.4

ITRAQ Changed Ratio:

117: 115=E3TL: D3TL=2.07

116: 114=E5TL: D5TL=0.91

Annotated typical Precursor MS Region:

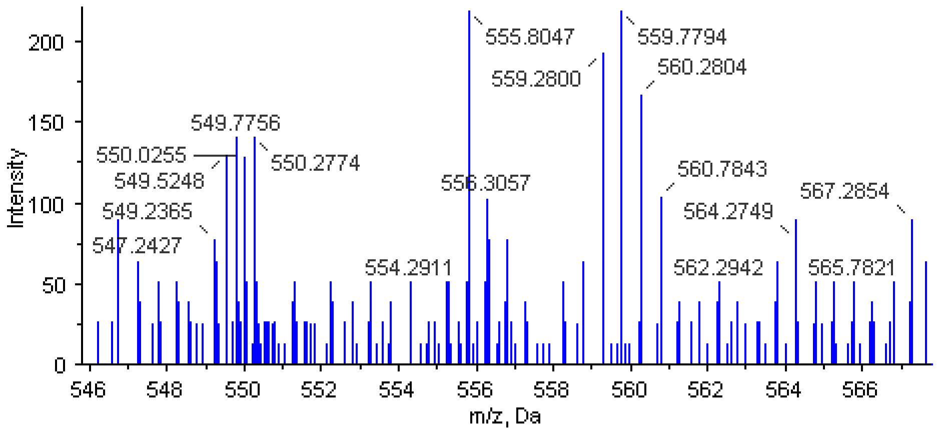

Sequence Coverage and Matched peptides: in different color

MAPKKDKAPPPSSKPAKSGGGKQKKKKWSKGKQKEK**VNNMVLFDQATYDK**LLSEAPKYKLITPSILSDR
LRVNGSLARRAIRELMARGLIRMVSAH**ASQQIYTR**ATNT

Protein No.: I-720

Protein name and Species: clathrin heavy chain 1 [Vitis vinifera]

OS=Vitis vinifera GN=VIT_13s0067g01290 PE=4 SV=1

Accession: tr|A5ACP0|A5ACP0_VITVI

Unused ProtScore: 3.4

Seq Cov %: 15.1

ITRAQ Changed Ratio:

117: 115=E3TL: D3TL=1.63;

116: 114=E5TL: D5TL=1.20

Annotated typical Precursor MS Region:

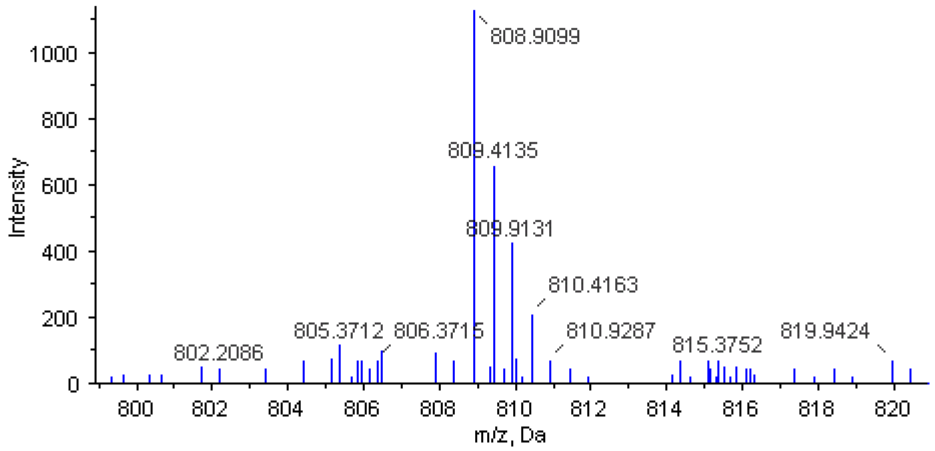

Sequence Coverage and Matched peptides: in different color

MAAANAPITMKEVLTLPSLGISPQFITFTHVTMESDKYLCVRETAPQNSVVIIDMNMPMQPLRRPITAD
SALMNPNTRILALKAQLPGTTQDHLQIFNIEMKAKMKSYQMPEQIVFWKWITPKMLGLVTQTSVYHWSI
EGDSEPVKMFERTANLVNNQIINYRCDPSEKWLVLIGIAPGSPERPQLVKGNMQLFSVEQHRSQALEAH
AASFATFKVPGNDQPCTLIGFATKSFNAGQIVSKLHVIELGSNPGKPGFTKKQADLFFPPDFADDFPVA
MQISHKYGLIYVITKLGLLFVYDLESASAVYRNRISPDPIFLTAEATSIGGFYAINRRGQVLLATVNEA
AIVPFVSGQLNNLELAVNLAKRGNLPGAENLVVQRFQELFAQTKYKEAAELAAESPQGILRTPDTVAKF
QSVPMQSGQTPPLLQYFGTLLTRGKLNAFESLELSRLVVNQNKKNLLENWLAEDKLECSEELGDLVKTV
DTDLALKIYIKARATPKVVAAFAERREFDKILIYSKQVGYTPDYLFLLQTILRSDPQGAVNFALMMSQM
EGGCPIDFNTITDLFLQRNLIREATAFLLDVLKPNLPEHGFLQTKVLEINLVTYPNVADAILANGMFSH
YDRPRIAQLCEKAGLYVRALQHYTELPDIKRVIVNTHAIEPQALVEFFGTLSREWALECMKDLLLVNLR
GNLQIIVQTAKEYSEQLGVDQCVKLFEQFKSYEGLYFFLGSYLSSSEDPDIHFKYIEAAAKTGQIKEVE
RVTRESNFYDAEKTKNFLMEAKLPDARPLINVCDRFGFVPDLTHYLYTNNMLRYIEGYVQKVNPSNAPL
VVGQLLDDECPEDFIKGLILSVRSLLPVEPLVDECEKRNRLRLLTQFLEHLVSEGSQDVHVHNALGKII
IDSNNNPEHFLTTNPYYDSRVVGKYCEKRDPTLAVVAYRRGQCDEELINVTNKNSLFKLQARYVVERMD
SDLWEKVLDPDNDYRRQLIDQVVSTALPESKSPEQVSAAVKAFMTADLPHELIELLEKIVLQNSAFSGN
FNLQNLLILTAIKADPSRVMDYINRLDNFDGPAVGEVAVEAQLFEEAFAIFKKFNLNVQAVNVLLDNIQ
SIERAVEFAFRVEEDAVWSQVAKAQLREGLVSDAIESFIRADDATQFLDVIRAAEDANVYHDLVRYLLM
VRQKAKEPKVDSELIYAYAKIDRLGEIEEFILMPNVANLQNVGDRLYDEALYEAAKIIFAFISNWAKLA
CTLVKLRQFQGAVDAARKANSSKTWKEVCFACVDAEEFRLAQICGLNIIIQVDDLEEVSDYYQNRGCFN
ELISLMESGLGLERAHMGIFTELGVLYARYRPEKLMEHIKLFSTRLNIPKLIRACDEQQHWKELTYLYI
QYDEFDNAATTIMNHSPDAWDHMQFKDVAVKVANVELYYKAVHFYLQEHPDLINDLLNVLALRVDHTRV
VDIMRKAGHLHLVKPYMVAVQSNNVSAVNEALNGIYVEEEDYDRLRESIDMHDNFDQIGLAQKIEKHEL
LEMRRVAAYIYKKAGRWKQSIALSKKDNLYKDAMETASQSGDRELAEELLVYFIEKGKKECFASCLFVC
YDLIRPDIALELAWINNMVDFALPYLLQFIREYAGKVDELVKDKLEALNEVKAKEKEEKDVIAQQNMYA
QLLPLALPAPPMPGMGGAGMAGGFVPPPMGSMGMPPMPPFGMPPMGTY

Protein No.: I-723

Protein name and Species:

Unknown protein 1 (Fragment) OS=Vitis rotundifolia PE=1 SV=1

Accession: sp|P86104|UP01_VITRO

Unused ProtScore: 3.4

Seq Cov %: 100

ITRAQ Changed Ratio:

117: 115=E3TL: D3TL=0.11

116: 114=E5TL: D5TL=0.58

Annotated typical Precursor MS Region:

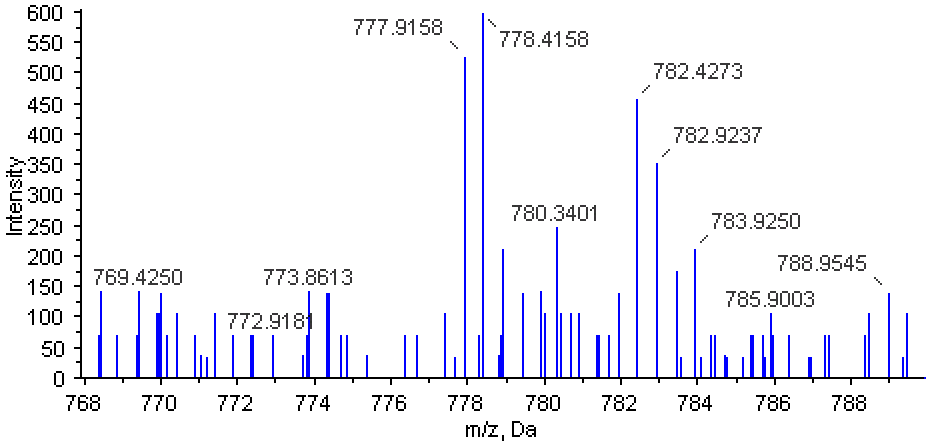

Sequence Coverage and Matched peptides: in different color

TNAENEFVTIKK

Protein No.: I-729

Protein name and Species:

5-methyltetrahydropteroyltriglutamate--homocysteine methyltransferase, putative OS=Ricinus communis GN=RCOM_0952830 PE=3 SV=1

Accession: tr|B9RQ33|B9RQ33_RICCO

Unused ProtScore: 3.37

Seq Cov %: 16.8

ITRAQ Changed Ratio:

117: 115=E3TL: D3TL=0.62

116: 114=E5TL: D5TL=1.09

Annotated typical Precursor MS Region:

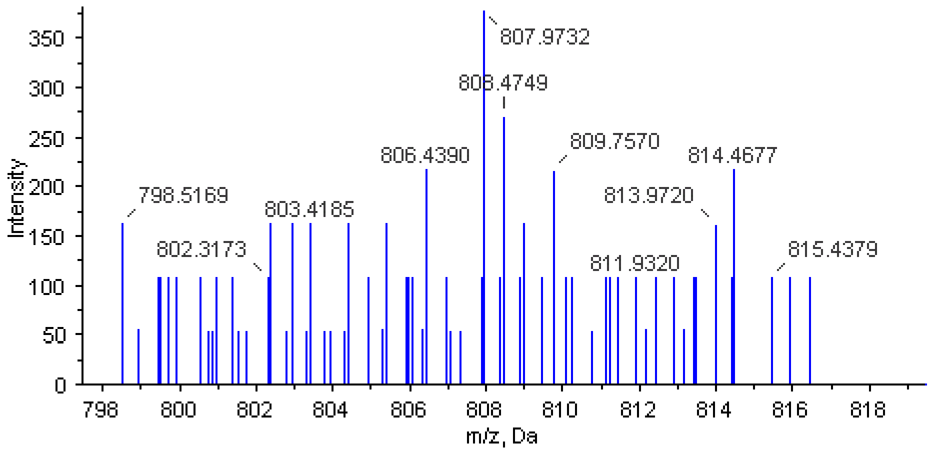

Sequence Coverage and Matched peptides: in different color

MRQVSLNVLAPFTSFRVISLPFSSSTNPSSLSFLPFSFHFPSLSSTSFRAMASHIAGYPRMGPKRELK**F**
ALESFWDGKSSAEDLQKVAAELRASIWKQMADAGTKFIPSNTFSYYDQVLDTTAMLGAVPPRYGWDGGE
IGFDVYFSMAR**GNASVPAMEMTK**WFDTNYHYIVPELGPEVKFSYASHKAVDEYKEAKALGVETVPVLVG
PVSYLLLSKPAKGVEKSFSLLSLIDRILPIYKEVIAELKAAGARWIQFDEPKLVMDLDSHELQAFTNAY
SELEASLSGVHVVVETYFADLPAEAYKTLTSLK**GVTGFGFDLVR**GTKTLDLIKGGFPTGK**FLFAGVVDG**
RNIWANDLASSLDTLHALESAVGKDKVVVSTSCSLLHTAVDLANEPKLDKEIK**SWLAFAAQKVLEVNAL**
AKALAGNRDEAFFSSNALAHASRKSSPR**VTNEAVQQAAAALK**GSDHRRATNVSARLDAQQKKLNLPILP
TTTIGSFPQTLDLRRVRREYKAKKISEDDYVKAIKEEISKVVKIQEELDIDVLVHGEPERNDMVEYFGE
QLSGFAFTANGWVQSYGSR**CVKPPIIYGDVSR**PKAMTVFWSSMAQSMTSRPMK**GMLTGPVTILNWSFVR**
NDQPRHETCYQIALAIKDE**VEDLEKAGITVIQIDEAALR**EGLPLRKSEHAFYLDWAVHSFRITNCGVED
TTQIHTHMCYSNFNDIIHSIINMDADVITIENSRSDEKLLSVFREGVK**YGAGIGPGVYDIHSPRIPSTE**
EIADRINKMLAVLES**NILWVNPDCGLK**TRKYSEVKPALSNMVAATKILRTQLASAK

Protein No.: I-732

Protein name and Species:

Catalytic, putative OS=Ricinus communis GN=RCOM_0300260 PE=4 SV=1

Accession: tr|B9T676|B9T676_RICCO

Unused ProtScore: 3.37

Seq Cov %: 8.5

ITRAQ Changed Ratio:

117: 115=E3TL: D3TL=4.02

116: 114=E5TL: D5TL=11.62

Annotated typical Precursor MS Region:

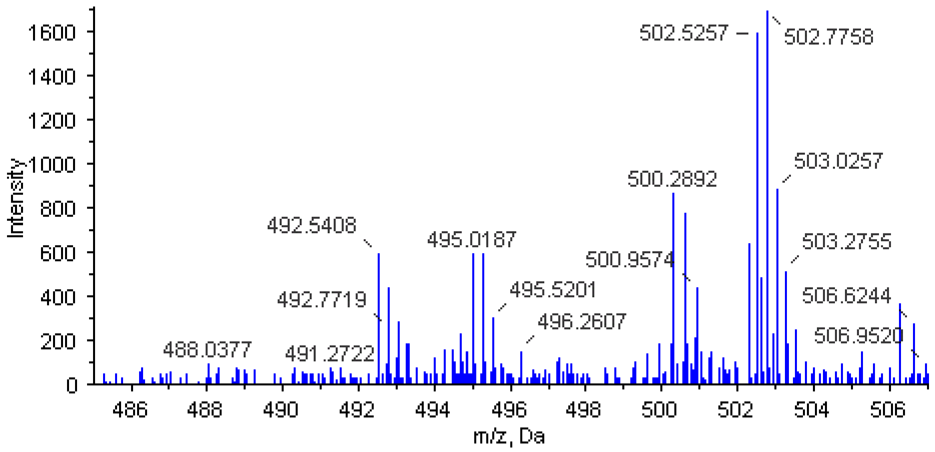

Sequence Coverage and Matched peptides: in different color

MEYEGNQQHVLEKYECLLFDVDDTLYPLRSGLSQHVTKNIQEYMVQKLGIEESKVAELCVSLYKLYGTT
LAGLRAIGHNIDYDDFHSFVHGRLPYDIVKPDPVLRNLLHSLPVRKIVFTNADKTHASRVLSRLGLEDC
FEGILSFETLNNTTKGNESVNEDENGVFDINEYTAAPNAGLALPRSPVVCKPFEDAFEQVFKITSINPQ
RTLFFDDSIRNLQTGKRLGLTTVWVGSSHRTEGVDYALESIHNIKEALPELWDANEKSDGVRYSGRVAI
ETSVKA

Protein No.: I-735

Protein name and Species:

Phagocytic receptor 1b [Vitis vinifera] OS=Vitis vinifera GN=VIT_01s0011g06540 PE=4 SV=1

Accession: tr|D7T8E1|D7T8E1_VITVI

Unused ProtScore: 3.34

Seq Cov %: 3.4

ITRAQ Changed Ratio:

117: 115=E3TL: D3TL=1.71

116: 114=E5TL: D5TL=1.29

Annotated typical Precursor MS Region:

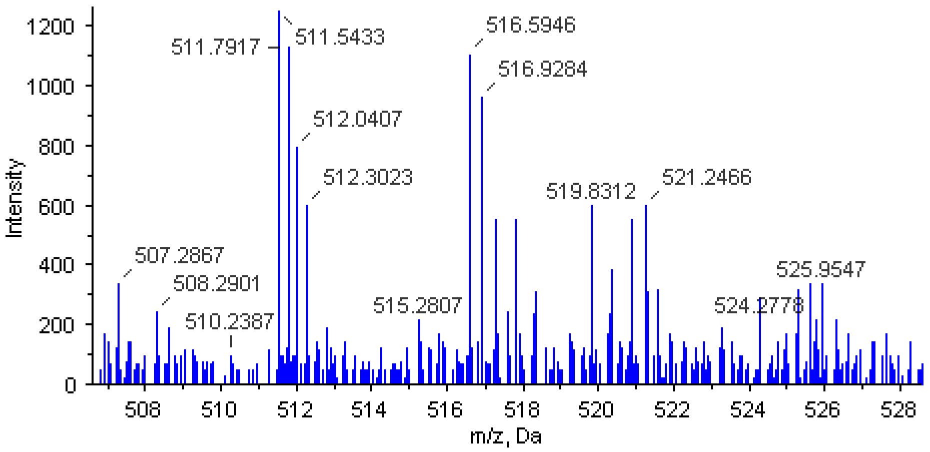

Sequence Coverage and Matched peptides: in different color

MERAMAILVVAVLILLCGSQVRSDASSHRYKAGEAVPLYANK**VGPFHNPSETYR**YFDLPFCVPAHLKEK
KEALGEVLNGDR**LVSAPYK**LDFLREKDSEVVCKKKLKKEEVAQFRAAVAKDYYFQMYYDDLPIWGFIGK
VDKEGKADPSEYKYFLYKHIQFDIRYNKDRVIEINARTDPHSMVDVTDDKDVDVEFMYTVKWTETDAPF
EKRMEKYSQTSSMPHHLEIHWFSIINSCVTVLLLTGFLATILMRVLKNDFIKYAHDEESADDQEETGWK
YIHGDVFRYPKYKSLFAAALGSGTQLFTLTVFIFILALVGVFYPYNRGALFTALVVIYALTSGIAGYTA
TSFYCQLEGTNWVRNLLLTGCLFCAPLFLTFCFLNTVAIIYSATAALPFGTIVVIVLIWTLVTSPLLVL
GGIAGKNSK**AEFQAPCR**TTKYPREIPALPWYRSTIPQMAMAGFLPFSAIYIELYYIFASVWGHRIYTIY
SILFIVFIILLIVTAFITVALTYFQLAAEDHEWWWRSFLCGGSTGLFIYGYCLYYYYARSDMSGFMQTS
FFFGYMACICYGFFLMLGTVGFRASLLFVRHIYRSIKCE

Protein No.: I-738

Protein name and Species:

Dimethylaniline monooxygenase, putative OS=Ricinus communis GN=RCOM_1470740 PE=4 SV=1

Accession: tr|B9RLT7|B9RLT7_RICCO

Unused ProtScore: 3.33

Seq Cov %: 6.1

ITRAQ Changed Ratio:

117: 115=E3TL: D3TL=0.43

116: 114=E5TL: D5TL=0.25

Annotated typical Precursor MS Region:

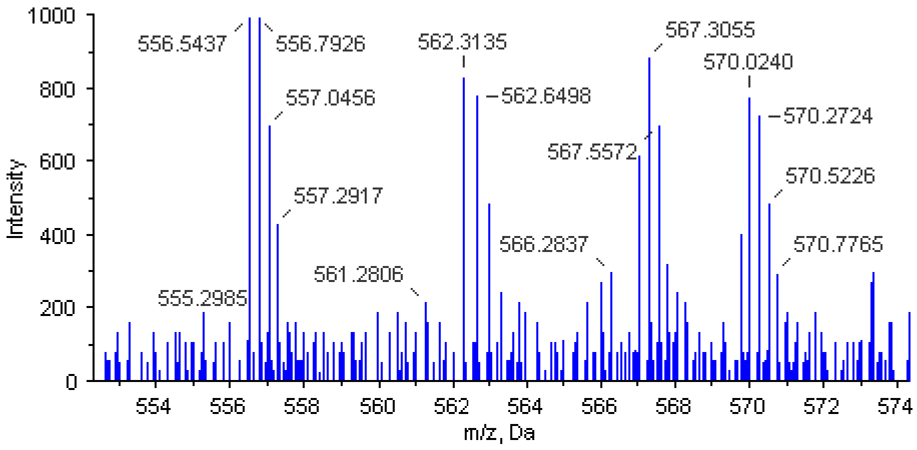

Sequence Coverage and Matched peptides: in different color

MVSDFTRSK**NVCVIGAGPSGLVAAR**ELRKEGHRVVLLEQKDDVGGQWLYEANVESEHPLGKKKFLEVHS
SIYASLRLVSPREIMGYTDFPFLVKKGRDMRRFPGHRELWLYLKDFCDSFGLREMIRFNTRVEYVGMLD
YDELRNDLKWVVKSKEKDSEKAVEEVFDAVVVATGHYSHPKLPFIKGMETWKRKQMHSHIYRVPEPFRN
EVVVVVGNSLSGQDISMELVEVAKEVHLSSKSLDVTLGLSKVISKHENLHLRPQIESLEEDGRVLFADG
SQVIADTILYCTGYTYTFPFLDTKGIVVVEDDRVGPLYEHTFPPSLAPSLSFVGIPRK**IIGFPFFESQA**
KWIAQLLSGKRTLPSWDDMMLSIKQFYQSRDTAGIPKHNTHDIADFEYCDRYADHIGFPHLEEWRKQLC
LSALVNANDNLETYRDSWDDHDLLQEALQSPHFTQLGVQDFIV

Protein No.: I-740

Protein name and Species:

Aminopeptidase M1 [Arabidopsis thaliana] OS=Arabidopsis thaliana GN=APM1 PE=2 SV=1

Accession: tr|Q8VZH2|Q8VZH2_ARATH

Unused ProtScore: 3.31

Seq Cov %: 6.8

ITRAQ Changed Ratio:

117: 115=E3TL: D3TL=0.19

116: 114=E5TL: D5TL=0.38

Annotated typical Precursor MS Region:

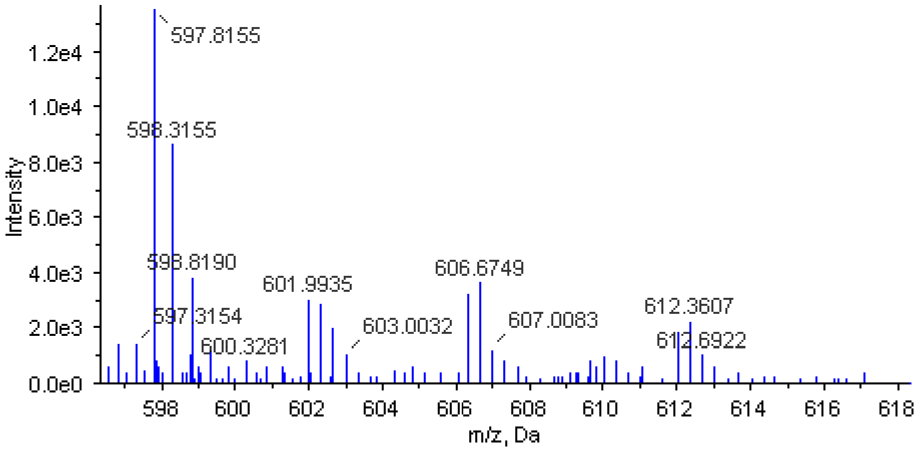

Sequence Coverage and Matched peptides: in different color

MDQFKGEPRLPKFAVPKRYDLRLNPDLIACTFTGTVAIDLDIVADTRFIVLNAADLSVNDASVSFTPPS
SSKALAAPKVVLFEEDEILVLEFGEILPHGVGVLKLGFNGVLNDKMKGFYRSTYEHNGEKK**NMAVTQFE**
PADARRCFPCWDEPACKATFKITLEVPTDLVALSNMPIMEEKVNGNLKIVSYQESPIMSTYLVAIVVGL
FDYVEDHTSDGIKVRVYCQVGKADQGKFALHVGAKTLDLFKEYFAVPYPLPKMDMIAIPDFAAGAMENY
GLVTYRETALLYDEQHSAASNKQRVATVVAHELAHQWFGNLVTMEWWTHLWLNEGFATWVSYLATDSLF
PEWK**IWTQFLDESTEGLR**LDGLEESHPIEVEVNHAAEIDEIFDAISYRKGASVIRMLQSYLGAEVFQK**S**
LAAYIKNHAYSNAKTEDLWAALEAGSGEPVNKLMSSWTKQKGYPVVSAKIKDGKLELEQSRFLSSGSPG
EGQWIVPVTLCCGSYEKRKNFLLESKSGAYDLKELLGCSIADGSDKINGTCSWIKINVDQAGFYRVKYD
DSLAAGLRNATESQSLTSIDRYGILDDSFALTMARQQSLASLLTLCSAYKKELDYTVLSNLIAISYKVV
KIGADANQELMSGIKHFFIGVFQFAAGKLGWDPK**QGESHLDAMLR**GEVLTALAVFGHDETLKEAVRRFD
AFLADR**NTPLLPPDIR**RAAYVAVMQRANKSDK**SGYESLLR**VYRETDLSQEKTRILGSLASCPDPTIVQD
VLNFVLSDEVRNQDALYGLSGVSWEGREVAWKWLQEKWEYIGNTWGSGFLITR**FISAVVSPFASFEK**AK
EVEEFFATRSKPSMARTLKQSIERVHINANWVESIKKEDNLTQLVAQLSSN

Protein No.: I-743

Protein name and Species:

Cytochrome P450, putative OS=Ricinus communis GN=RCOM_1517900 PE=3 SV=1

Accession: tr|B9SDH3|B9SDH3_RICCO

Unused ProtScore: 3.29

Seq Cov %: 2.3

ITRAQ Changed Ratio:

117: 115=E3TL: D3TL=2.11

116: 114=E5TL: D5TL=1.19

Annotated typical Precursor MS Region:

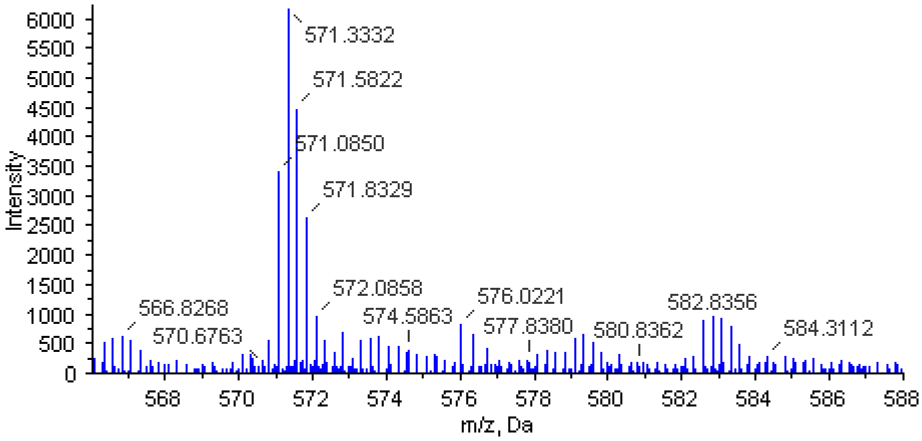

Sequence Coverage and Matched peptides: in different color

MEIWLFIPISLAVSILLKLVINHLLSTKNSTHKLPPGPSPLPILGSSLWLTTTVFDLESALRSLHSKFG
PLLTLHMGSRPAIFIADRSLAHEALIQNSSIFADRPPALGMEKINTSDQHTISSAFYGPTWRLLRRNLT
AKVLHPSNIKSYSHTRKWVVQILKDRLESEFKERGRIQVMGHFQYAMFCLLAFMCFGDNLEEDQIRKIE
EVQRQLLLNFVEFSVFSFWPSVTKVLFRKRWDEYMQIR**KNQENVLIPLLR**SRKAQRDKETDSVLPYVDT
LMDLGLPDEKRKLEEEEIVSLLSEILNGGTETTATALQWIMANLVKYPHIQEKLYMEIRRIVGEGEDLI
KEDELQKMPFLKAVILEGLRRHPPAHMVVPHAVTEDTVLDKYLIPKNGTVNFMVAEMGWDSKVWKDPMA
FKPERFMGSEYEHEVFDITGSREIKMMPFGLGRRMCPGHGLAMLHLEYLVANMVWSYEWKGMDGNGVDL
SEKLEFSVVMK**NPLQAQISPR**RRHKLIIDKI

Protein No.: I-744

Protein name and Species:

cytochrome b5 reductase 1-like，NADH [Vitis vinifera] OS=Vitis vinifera GN=VIT_13s0047g01190 PE=4 SV=1

Accession: tr|F6HIY1|F6HIY1_VITVI

Unused ProtScore: 3.29

Seq Cov %: 6.8

ITRAQ Changed Ratio:

117: 115=E3TL: D3TL=1.61

116: 114=E5TL: D5TL=2.01

Annotated typical Precursor MS Region:

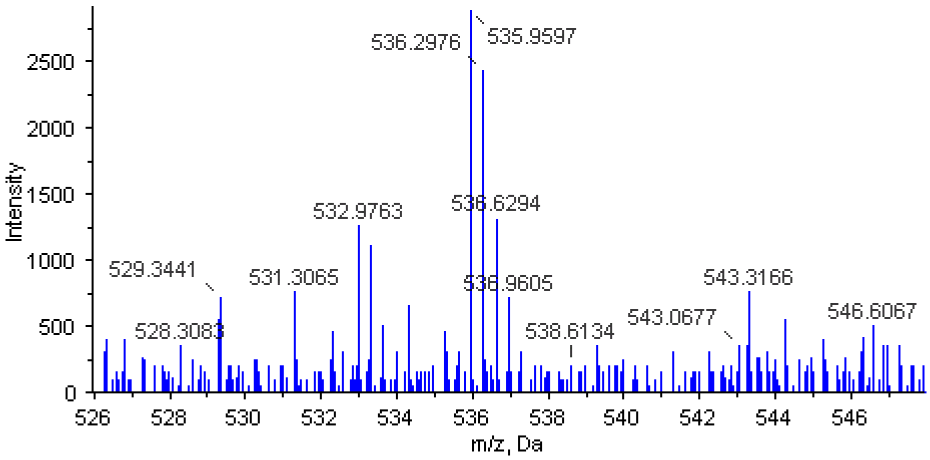

Sequence Coverage and Matched peptides: in different color

MWKMTSTLLPTFRVTWPLKTPGIELSGFAVGLAAVGLAAVYFFYGRRKPKGCLDPERFQDFKLVQKTRI
SHNVGRFRFALPTPTSVFGLPVGQHIICRGKDDQGAEVIRPYTPITLDSDVGFYELVVKMYPKGQMSHH
FRKMREGDTLAVKGPRGRLIYKPGQAKAFGMLAGGTGITPMFQLTR**AILENPK**DKTNVHLIYANISYDD
ILLKDELDGFARKFPNRFKVFYVLSQPPEAWNGGIGHISKEMIEK**HCPAPAPDIQILR**CGPPGMNKAMA
GHLVALGYTPQMQFEF

Protein No.: I-746

Protein name and Species:

40S ribosomal protein S23 OS=Solanum tuberosum PE=2 SV=1

Accession: tr|Q38HT5|Q38HT5_SOLTU

Unused ProtScore: 3.29

Seq Cov %: 11.3

ITRAQ Changed Ratio:

117: 115=E3TL: D3TL=0.62

116: 114=E5TL: D5TL=0.41

Annotated typical Precursor MS Region:

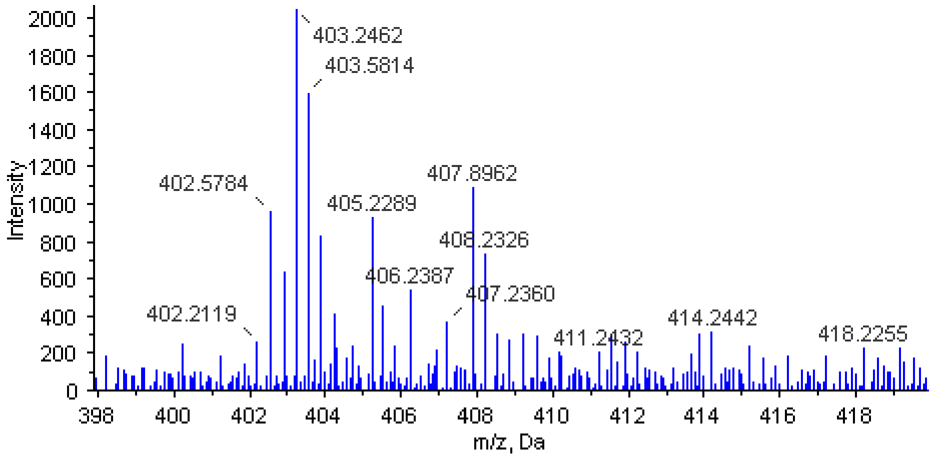

Sequence Coverage and Matched peptides: in different color

MGKTRGMGAGRKLKSHRRRQRWADKSYKKSHLGNEWKKPFAGSSHAKGIVLEKIGIEAKQPNSAIRKCA
RVQLIKNGKKIAAFVPNDGCLNYIEENDEVLIAGFGRK**GHAVGDIPGVR**FKVVKVSGVSLL**ALFK**EKKE
KPRS

Protein No.: I-757

Protein name and Species:

Very-long-chain enoyl-CoA reductase-like OS=Populus trichocarpa GN=POPTRDRAFT_720621 PE=2 SV=1

Accession: tr|A9PDL9|A9PDL9_POPTR

Unused ProtScore: 3.24

Seq Cov %: 7.1

ITRAQ Changed Ratio:

117: 115=E3TL: D3TL=1.94

116: 114=E5TL: D5TL=2.66

Annotated typical Precursor MS Region:

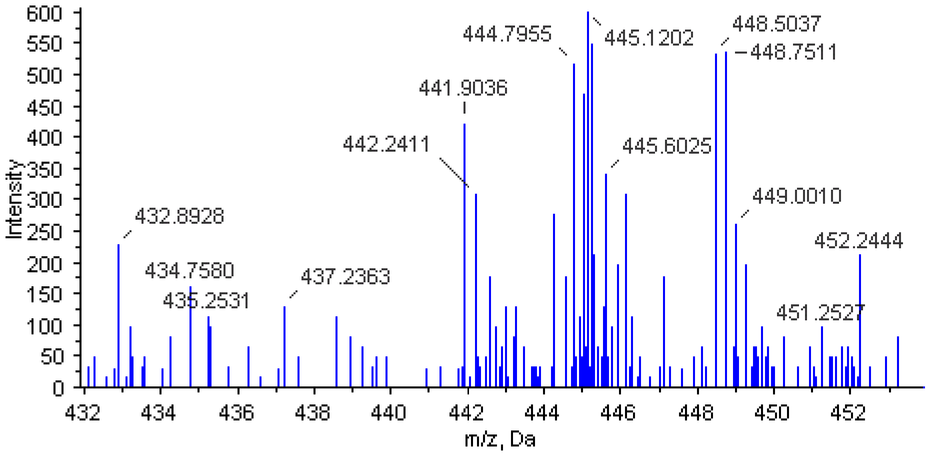

Sequence Coverage and Matched peptides: in different color

MKVTVVSRSGREVIKGGLELSDSATVADLQEAIHKRTKKFYPARQRLTLPLPPGSKERPTVLNYKKNLK
EYQDGNLDSLTILFKDLGPQVSYRTLFFFEYLGPLVLYPFFYYFPVYQFLGYKGEHIMHPVQTYALYYW
CFHYFKRIMETFFVHRFSHATSPLSNVFRNCAYYWTFGSYIAYYVNHPLYTPVGDLQMKIGFGFGLVCQ
LANFYCHILLRNLRRPDGNGGYQIPSGFLFNIVTCANYTTEIYQWLGFNIATQTVAGYIFLVVAASIMT
NWALAKHRRLKKLFDGKDGRPKYPRRWVILPPVL

Protein No.: I-758

Protein name and Species:

Suppression of tumorigenicity, putative OS=Ricinus communis GN=RCOM_0712150 PE=4 SV=1

Accession: tr|B9RRB9|B9RRB9_RICCO

Unused ProtScore: 3.22

Seq Cov %: 6.4

ITRAQ Changed Ratio:

117: 115=E3TL: D3TL=0.52

116: 114=E5TL: D5TL=1.68

Annotated typical Precursor MS Region:

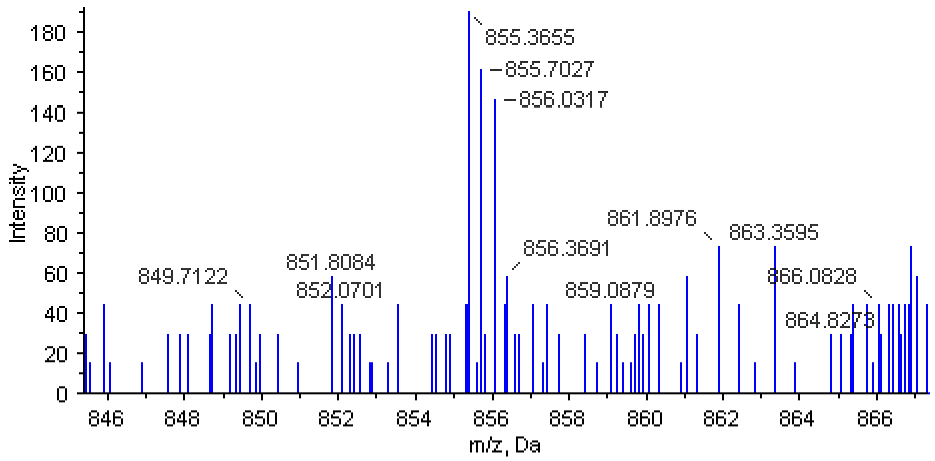

Sequence Coverage and Matched peptides: in different color

MAKNEDTGSPGWR**ASLFLQTTEDVARAVAAAAAAATAPR**SPRPSVVFSSKDDHGDSQFQKLQRHVTRVL
KGFSSPPPEVKAGTYNPEVLTSQKRQWANFQLQYLDHRSLKEPSRLFESMVVVGLHPNCDLQALQRQYV
ARKSEGSARLQGSPGCQNQSRVEPVLEPQVLFVYPPDKQLPLKYKDLLSFCFPGGMEVHAVEKTPSMSE
LNEILLGQEHLKQSDLSFVFRLQVADDSTLYGCCVLVDEIVQKPSGLLSMISDKRSSCSSLSRYVLTTR
RCYCILSRLPFFELHFGVLNSIFTEERLERLTRHIGAFDLESSEGYDKDENLGYNLDSVSPNYIAAEDR
LDGAAETFQSSLRDSTPGEDNKNHMEHCITEEHIHLLQKGVNDNIVVPVESETEIVSNKGESGGGNLED
SDICVDDLSKKQAEERRLPNAVLPLIR**YYQYESSESSSSFQGSPSEDR**NFRSDIDDTETEEASTSGQED
TNDHMDILEWAKVNNYGSLQIICKYYQLHCPSRGSTLRLHPLEHLHPLEYHRTDETVLHIAGSVIDLRT
CSTSLEFAEAHSALSAEEEATALSTWAVACICGSLRLENVLTLFAGALLEKQIVFVCSNLGILSASVLS
IVPLIRPYQWQSLLMPILPDDMLDFLDAPVPYVVGVKNKTSEVQSKLTNVILVDVNKNQVKSHTIPQLP
KHRELFSSLSPYHAKLVGESYLARKRPVHECTDVQVCYSA

Protein No.: I-759

Protein name and Species:

Peroxidase 12, putative OS=Ricinus communis GN=RCOM_0961200 PE=3 SV=1

Accession: tr|B9SEA1|B9SEA1_RICCO

Unused ProtScore: 3.22

Seq Cov %: 7.1

ITRAQ Changed Ratio:

117: 115=E3TL: D3TL=2.49

116: 114=E5TL: D5TL=1.56

Annotated typical Precursor MS Region:

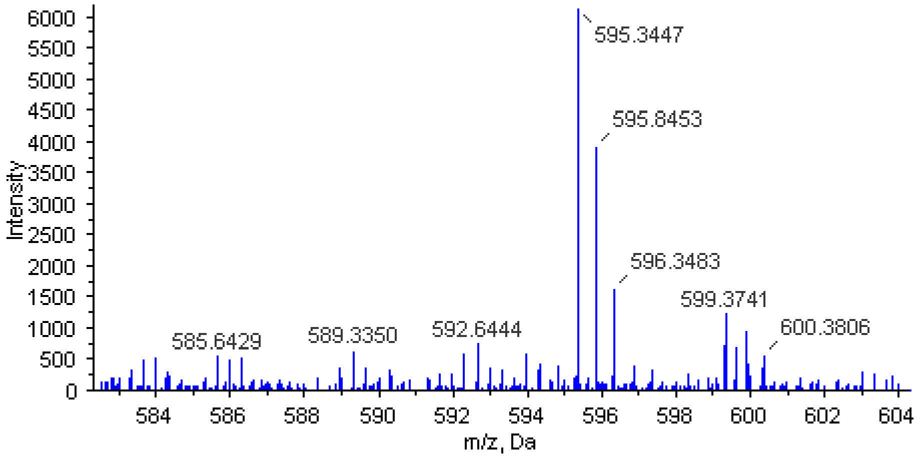

Sequence Coverage and Matched peptides: in different color

MASANSFTSLLLISCLLLAACFSAIEAQTKPPIKNGLSWTFYKTSCPKAESIIRSELKKIFKK**DVGQAA**
GLLRLHFHDCFVLGCDSSVLLDGSAGGPSEKSELPNLTLRKQAFKIVEDLRARLHKECGR**VVSCSDIVA**
IAARDSVVLTGGPEYAIPLGRRDGVKFAEINATFEHLVGPTAKVTEILTKLDRKGLDATDAVALSGGHT
IGIGHCTSFTER**LYPSQDPTMDK**TFANNLKLTCPKLDTTNTTFLDIRSPNKFDNK**YYVDLMNR**QGLFTS
DQDLYTDKRTRSIVTSFAINESLFFEKFIIGMIK**MGQLDVLTGNQGEIR**ANCSAINPKKKYIESVAEEE
LGSSSEMR

Protein No.: I-760

Protein name and Species:

elongation factor 2-like OS=Vitis vinifera GN=VIT_19s0027g00760 PE=4 SV=1

Accession: tr|F6H4T7|F6H4T7_VITVI

Unused ProtScore: 3.21

Seq Cov %: 25

ITRAQ Changed Ratio:

117: 115=E3TL: D3TL=1.63

116: 114=E5TL: D5TL=1.51

Annotated typical Precursor MS Region:

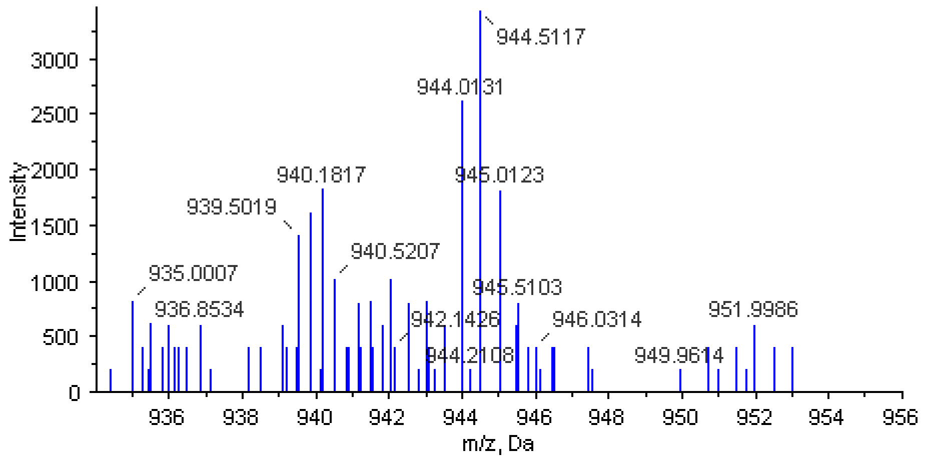

Sequence Coverage and Matched peptides: in different color

MDFNRICLPLWVKFTVEELRRIMDYKHNIRNMSVIAHVDHGKSTLTDSLVAAAGIIAQEVAGDVRMTDT
RQDEAERGITIKSTGISLYYEMSDESLKNYRGERQGNEYLINLIDSPGHVDFSSEVTAALRITDGALVV
VDCVEGVCVQTETVLRQALGERIRPVLTVNKMDRCFLELQVDGEEAYQTFSRVIENANVIMATYEDPLL
GDVQVYPEKGTVAFSAGLHGWAFTLTNFAKMYASKFGVDESKMMERLWGENFFDPSTRKWTTKNTGAPN
CKRGFVQFCYEPIKQIINTCMNDQKDKLWPMLQKLGVTMKSDEKDLMGKALMKRVMQTWLPASTALLEM
MIFHLPSPSTAQKYRVENLYEGPLDDIYATAIRNCDPEGPLMLYVSKMIPASDKGRFFAFGRVFSGKVS
TGLKVRIMGPNYVPGEKKDLYVKSVQRTVIWMGKRQETVEDVPCGNTVAMVGLDQFITKNATLTNEKES
DAHPIRAMKFSVSPVVRVAVQCKVASDLPKLVEGLKRLAKSDPMVVCSIEESGEHIIAGAGELHLEICL
KDLQDDFMGGAEIVKSDPVVSFRETVLEKSCRTVMSKSPNKHNRLYMEARPLEEGLAEAIDDGRVGPRD
DPKARSKILSEEFGWDKDLAKKIWCFGPETTGPNMVVDMCKGVQYLNEIKDSVVAGFQWASKEGALAEE
NMRGICFEVCDVVLHADAIHRGGGQVIPTARRVIYASQLTAKPRLLEPVYLVEIQAPEQALGGIYSVLN
QKRGHVFEEMQRPGTPLYNIKAYLPVVESFGFSGTLRAATSGQAFPQCVFDHWDVMSADPLEAGSTAAQ
LVADIRKRKGLKEQMTPLSEFEDKL

Protein No.: I-763

Protein name and Species:

Cinnamoyl-CoA reductase OS=Hevea brasiliensis GN=CCR PE=2 SV=1

Accession: **tr|E9M776|E9M776_HEVBR**

Unused ProtScore: **3.19**

Seq Cov %: 5.9

ITRAQ Changed Ratio:

117: 115=E3TL: D3TL=0.58

116: 114=E5TL: D5TL=1.02

Annotated typical Precursor MS Region:

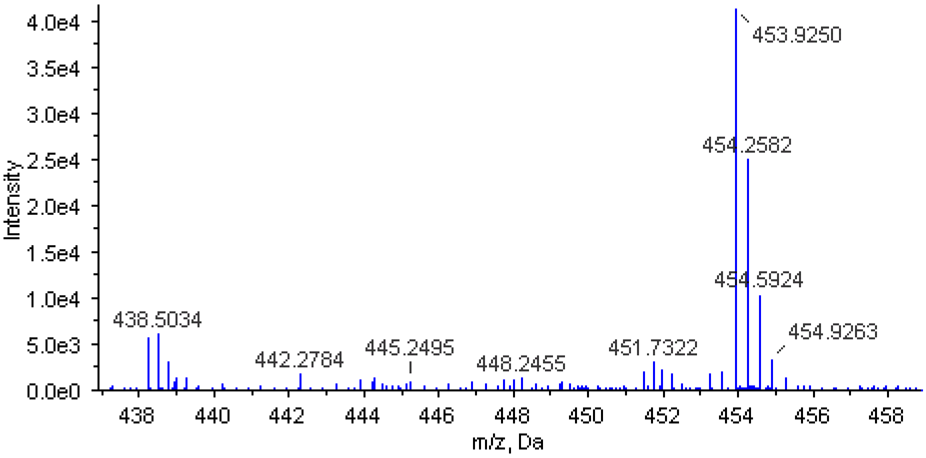

Sequence Coverage and Matched peptides: in different color

MPVDTSSLSGHGQTVCVTGAGGFIASWIVKLLLERGYTVK**GTVRNP**DDPKNSHLRELEGAKERLTLCK**A**
DLLDYESLRKAIMGCDGVFHAASPVTDDPEQMVEPAVNGTKNVVIAA**AEAK**VRRVVFTSSIGAVYMDPN
RNPDVVVDESCWSDLDFCKNTKNWYCYGKAVAEQAAWEVAKEKGVDLVAVNPVLVLGPLLQSTVNASII
HILKYL**TGSAK**TYANSVQAYAHVKDVALAHILVYEIPSASGR**YLCAESVLHR**GEV**VEILAK**SFPEYPIP
TRCSDEKNPRAKPYKFSNQKLKDLGMEFTPVKQCLYETVKSLQERGHLPIPKQPEDSIRIQS

Protein No.: I-771

Protein name and Species:

Multicopy suppressor of IRA1 OS=Nicotiana tabacum GN=MSI1 PE=2 SV=1

Accession: tr|B0LUS9|B0LUS9_TOBAC

Unused ProtScore: 3.09

Seq Cov %: 5.7

ITRAQ Changed Ratio:

117: 115=E3TL: D3TL=0.58

116: 114=E5TL: D5TL=0.52

Annotated typical Precursor MS Region:

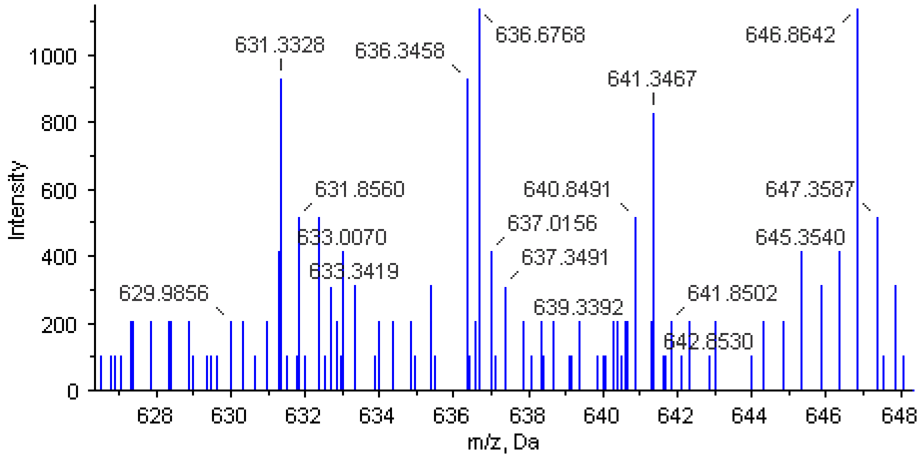

Sequence Coverage and Matched peptides: in different color

MGKDEDEMRGEIEERLINEEYKIWKKNTPFLYDLVITHALEWPSLTVEWLPDREEPPGKDYSVQKMILG
THTSENEPNYLMLAQVQLPLEDAENDARHYDDDRSEFGGFGCANGK**VQIIQQINHDGEVNR**ARYMPQNS
FIIATKTISAEVYVFDYSKHPSKPPLDGACNPDLRLRGHSTEGYGLSWSQFKQGHLLSGSDDAQTCLWD
INATPKNK**ALDAMQIFK**IHEGVVEDVAWHLRHEYLFGSVGDDQYLHIWDLRTPSVTKPIQSVVAHQSEV
NCLAFNPFNEWVVATGSTDKTVKLFDLRKISTALHTLDCHKEEVFQVGWNPKNETILASCCLGRRLMVW
DLSRIDEEQTPEDAEDGPPELLFIHGGHTSKISDFSWNPCEDWVVASVAEDNILQIWQMAENIYHDEDD
LPGDDATKAP

Protein No.: I-772

Protein name and Species:

ubiquitin-associated (UBA) zinc-finger and PB1 domain-containing protein OS=Populus trichocarpa GN=POPTRDRAFT_252306 PE=4 SV=1

Accession: tr|B9IEZ0|B9IEZ0_POPTR

Unused ProtScore: 3.06

Seq Cov %: 5.5

ITRAQ Changed Ratio:

117: 115=E3TL: D3TL=0.13

116: 114=E5TL: D5TL=0.57

Annotated typical Precursor MS Region:

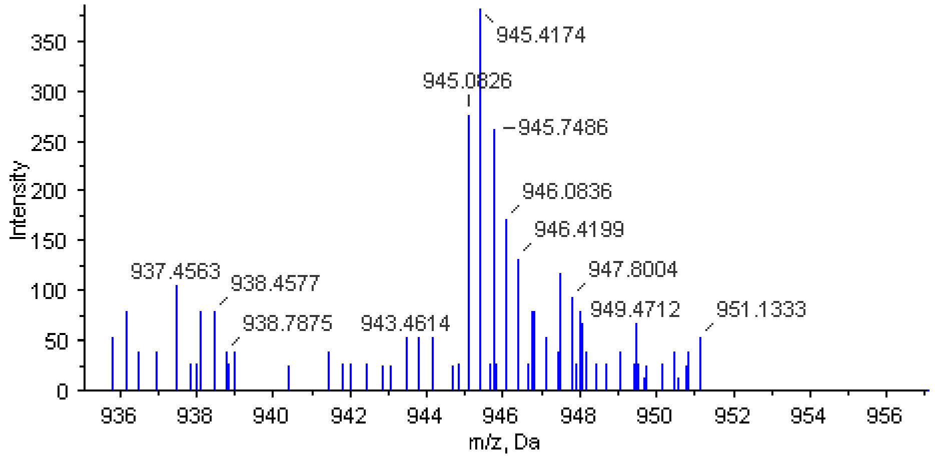

Sequence Coverage and Matched peptides: in different color

IQVKYSDTLRRFNAHVKENEQLDLDMIALREKIFGLFNFPPDADLTLTYIDEDGDVVTLADDDDLRDVM
RQNLKFLRIDVQLNNDKSGKSNARSSGSSTPMRSPRVQSPLPCLNNGVADVLKSVPEPLREVLSKISLD
LTSKAVASNTVLTELVDCFSKMGQYHLNPTSQSHDGIGAQTGATAPTVLNASKDGGLKEDLLNLNSPLK
TSQEERFENGTKTAMSPHTAVPSPVNLNPNPQISNSHVIKRNNSLNNPMVGMFHR**GVQCDGCGVHPITG**
PRYKSKVK**EDYDLCSICFAEMGNEADYIK**MDRPMPYRNRWSFKGFNDPKSWAIPQPLSKGSYGVKGAQP
KLDSRFVLDVNVSDGTMMPTCTPFTKIWRMRNNGSVAWPQGVRLVWIGGDRFFNTDSVEIEIPVNGVPI
DGELDVAADFVSPALPGRYISYWRMAYPSGGKFGQRVWVLIEVDASLKDPFFKYLNLNESPNYIGSKFP
GVLDMNVQPAVDGCFLEPQNNTLLSEPDVPMVDEQPKSQELKFPIDDALLIGHGVSASAPPQAMPSSVP
VLYPMIDISETVPASTELLPAADASTSPEEVIVENAVEKTLLKELKEMGFKQVDLNKEILRRNEYDLEQ
SVDDLCGVSDWDPILEELQEMGFRDKEMNKLLLKKNNGSIKGVVMDILTGKK

Protein No.: I-774

Protein name and Species:

Vacuolar sorting protein, putative OS=Ricinus communis GN=RCOM_0363260 PE=4 SV=1

Accession: tr|B9SYU2|B9SYU2_RICCO

Unused ProtScore: 3.05

Seq Cov %: 2.5

ITRAQ Changed Ratio:

117: 115=E3TL: D3TL=1.96

116: 114=E5TL: D5TL=2.06

Annotated typical Precursor MS Region:

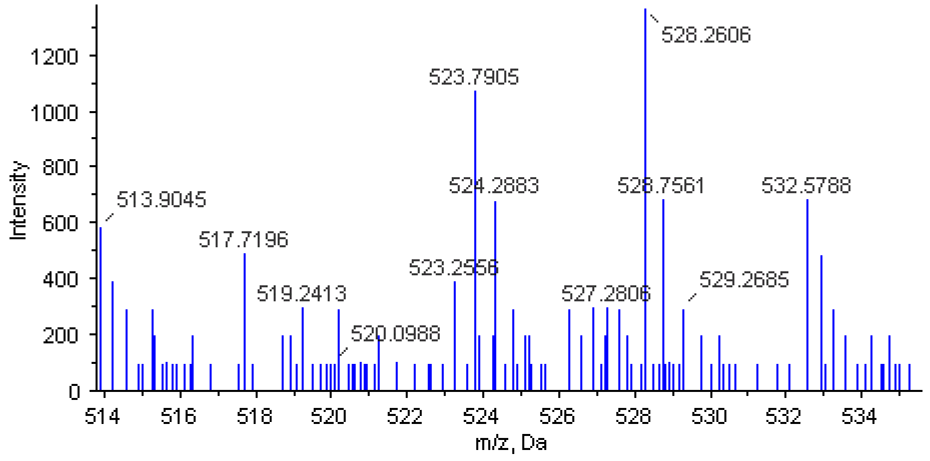

Sequence Coverage and Matched peptides: in different color

MIADGVENEEKWLAAGIAGLQQNAFNMHR**ALDSNNLR**DALKYSAQMLSELRTSKLSPHKYYELYMRAFD
ELRKLEIFFREETRRGCSIIDLYELVQHAGNILPRLYLLCTVGSVYIKSKEAPAKDVLKDLVEMCRGIQ
HPVR**GLFLR**SYLSQVSRDKLPDIGSEYEGDADTVMDAVEFVLQNFTEMNKLWVRMQHQGPAREKEKREK
ERSELRDLVGKNLHVLSQIEGVDLDMYKETVLPRVLEQVVNCKDEIAQFYLMDCIIQVFPDEYHLQTLE
VLLGACPQLQPSVDIKMVLSRLMERLSNYAASSTEVLPEFLQVEAFSKLNHAIGKVIEAQVDMPMFGAV
TLYSSLLTFTLHVHPDRLDYADQVLGACVKKLSNKGKLEDSKATK**QIVALLSAPLEK**YNDVVTALKLSN
YPRVMEYLDNETNKVMATVIIQSIMKNNTRISIADKVEALFELITGLIKDLDGTHEEVDEDDFKEEQNS
VARLIQMLHNDDPEEMYKIICTVRKQIMTGGPKRLPFTVPPLVFSSLKLVRRLQGQEENPFGDESSTTP
KKIFQLLNQMIEALSIVPAPELALRLYLQCAEAANDSDLEPVAYEFFTQAYILYEEDISDSKAQVTALH
LIIGTLQRMHVFGVENRDTLTHKATGYSAKLLKKPDQCRAVYGCAHLFWVDDQDNMKDGERVLICLKRA
LRIANAAQQMANATRGSTGSVTLFVEILNKYLYFFEKGNPQVTVAAIQSLIELITTEMQSDSSTPDPAA
DAFFASTLRYIQFQKQKGGAIGEKYEPLKIVTG

Protein No.: I-778

Protein name and Species:

Phosphoglycerate dehydrogenase OS=Populus trichocarpa GN=POPTRDRAFT_1069632 PE=2 SV=1

Accession: tr|A9PEK1|A9PEK1_POPTR

Unused ProtScore: 3.04

Seq Cov %: 4.2

ITRAQ Changed Ratio:

117: 115=E3TL: D3TL=0.19

116: 114=E5TL: D5TL=0.54

Annotated typical Precursor MS Region:

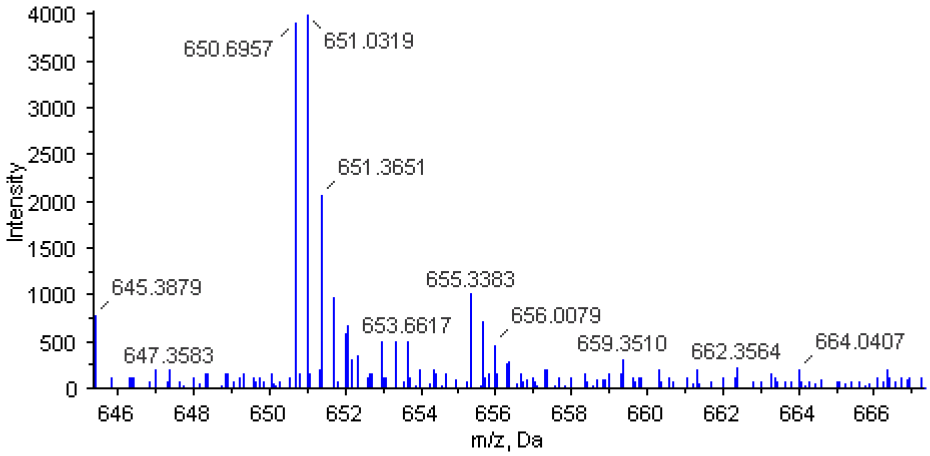

Sequence Coverage and Matched peptides: in different color

MAATTSTAASSLLATKKNLSSLSLTSANKLPAFSPLLTSSRRQRFIVLSASLNSKPTVLVAEKLGEA**GI**
NLLKDFANVDCSYNLSPDELCTKISLCDAIIVRSGTKVSREVFESSGGRLKVVGRAGVGIDNVDLAAAT
EHGCLVVNAPTANTVAAAEHGIALMAAMARNVAQADASVKAGKWERNKYVGVSLVGKTLAVMGFGKVGS
EVARRAK**GLGMHVIAHDPYAPADR**ARAIGVELVSFDEAIATADFISLHMPLTPATAKILNDETFAKMKK
GVRIVNVARGGVIDEDALVRALDAGIVAQAALDVFTVEPPPQDSKLVQHERVTVTPHLGASTKEAQEGV
AIEIAEAVVGALKGELASTSVNAPMVPAEVLTELKPYVELAEKLGRLAVQLVSGGSGVKDVKVTYASAR
APDDLDTRVLRAMITKGLIEPISSVFVNLVNADFSAKQRGLRISEERILGDGSPESPLHFIQVQIANVE
SKFASAISENGEIKVEGRVKDGIPHLTKVGSFEVDVSLEGSIILCRQVDQPGMIGKVGSVLGVENVNVS
FMSVGRIAPRKQAVMAIGVDEQPSKETLKKIGDIPAVEEFVFLKL

Protein No.: I-779

Protein name and Species:

Sucrose synthase [Arabidopsis thaliana] OS=Arabidopsis lyrata subsp. lyrata GN=ARALYDRAFT_494980 PE=4 SV=1

Accession: tr|D7MNN8|D7MNN8_ARALL

Unused ProtScore: 3.03

Seq Cov %: 8.6

ITRAQ Changed Ratio:

117: 115=E3TL: D3TL=0.27

116: 114=E5TL: D5TL=0.33

Annotated typical Precursor MS Region:

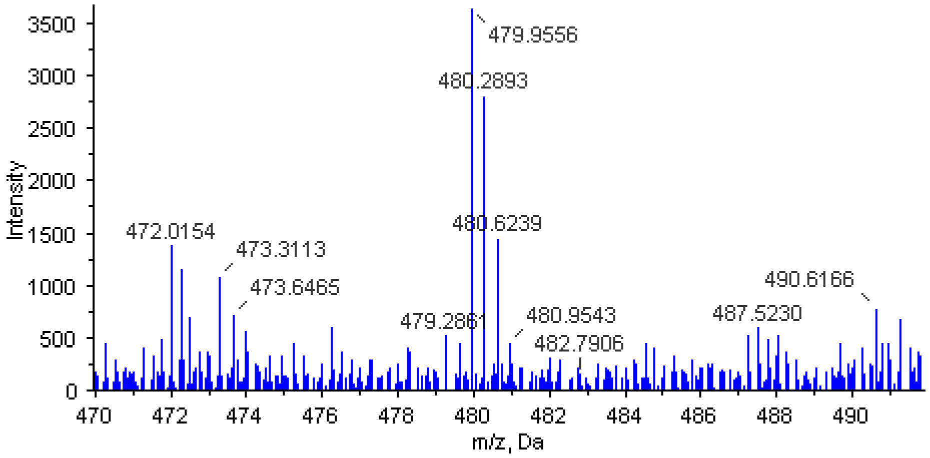

Sequence Coverage and Matched peptides: in different color

MPTGRFETMREWVHDAISAQRNELLSLFSRYVAQGKGILQSHQLIDEFLKTVKVDGTTEDLNKSPFMKV
LQSAEEAIVLPPFVALAIRPRPGVREYVRVNVYELSVDHLTVSEYLRFKEELVNGHANGDYLLELDFEP
FNATLPRPTR**SSSIGNGVQFLNR**HLSSIMFRNKESMEPLLEFLRTHKHDGR**SMMLNDR**IQNIPILQGAL
ARAEEFLSKLPLATPYSEFEFELQGMGFERGWGDTAQKVSEMVHLLLDILQAPDPSVLETFLGRIPMVF
NVVILSPHGYFGQAN**VLGLPDTGGQVVYILDQVRALENEMLLR**IQKQGLEVIPKILIVTRLIPEAKGTT
CNQRLEKVSGTEHAHILRIPFRTEKGILRKWISRFDVWPYLETFAEDASNEISAELQGVPNLIIGNYSD
GNLVASLLASKLGVMQCNIAHALEKTKYPESDIYWRNHEDKYHFSSQFTADLIAMNNADFIITSTYQEI
AGSKNNVGQYESHTAFTMPGLYR**VVHGIDVFDPK**FNIVSPGADMTIYFPYSDKERRLTALHESIEELLF
SAEQNDEHVGLLSDQ**SKPIIFSMAR**LDRVKNLTGLVECYAKNSKLRELANLVIVGGYIDVNQSRDREEM
AEIQKMHSLIEQYDLHGQFRWIAAQMNRARNGELYRYIADTKGVFVQPAFYEAFGLTVVESMTCGLPTF
ATCHGGPAEIIENGVSGFHIDPYHPDQVAATLVSFFETCNTNPNHWVKISEGGLKRIYERYTWKKYSER
LLTLAGVYAFWKHVSKLERRETRRYLEMFYSLKFRDLANSIPLATDEN

Protein No.: I-780

Protein name and Species:

Calcium ATPase OS=Populus trichocarpa GN=POPTRDRAFT_768258 PE=3 SV=1

Accession: tr|B9HPP7|B9HPP7_POPTR

Unused ProtScore: 3.03

Seq Cov %: 2.4

ITRAQ Changed Ratio:

117: 115=E3TL: D3TL=2.01

116: 114=E5TL: D5TL=1.67

Annotated typical Precursor MS Region:

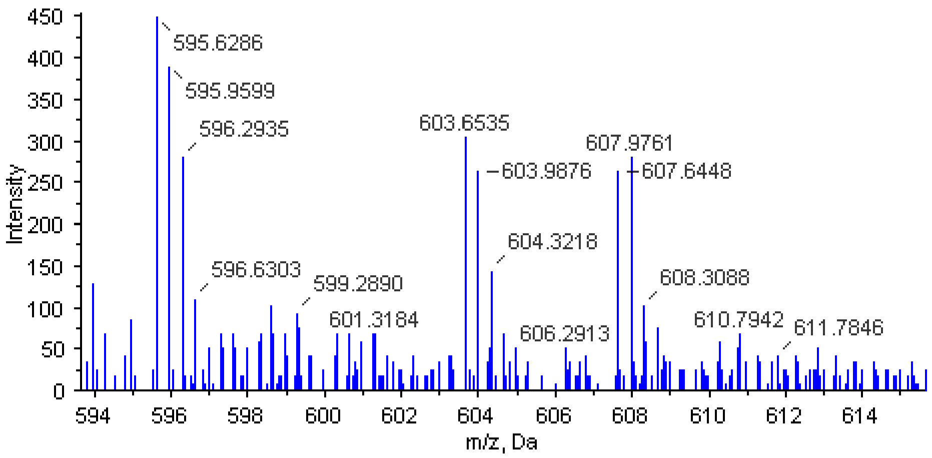

Sequence Coverage and Matched peptides: in different color

MGKGGEDYGERDQNGIESQNQGDIFPAWAKEVKECEEKYAVNREFGLSSADVEKRLKIYGYNELEKHEG
VSIFKLILDQFNDTLVRILLAAAIVSFVLAWYDGEEGGEMGITAFVEPLVIFLILIVNGIVGIWQESNA
EKALEALK**EIQSEHATVIR**DRKKFSSLPAKELVPGDIVELRVGDKVPADMRVLNLISSTLR**VEQGSLTG**
ESEAVSKTAKPVAESTDIQGKKCMVFAGTTVVNGNCICLVTETGMNTEIGKVHSQIHEAAQNEEDTPLK
KKLNEFGEVLTMLIGIICALVWLINLKYFLTWEYVDGWPKNFKFSFEKCTYYFEIAVALAVAAIPEGLP
AVITTCLALGTRKMAQKNALVRKLPSVETLGCTTVICSDKTGTLTTNQMAVSKLVAMGSRVGTLRSFNV
EGTTYSPFDGKIEDWPVGRMDSNLQMIAKIAAVCNDAGVEQSGNHYVAGGMPTEAALKVMVEKMGFPGG
LSKESSLVHEDVLACCRLWNTMEQRIATLEFDRDRKSMGVIVNSSSGKKSLLVKGAVENLLDRSTSIQL
LDGSVVALDRCSKDLILQNLREMSTSALRCLGFAYKEDLSEFRTYSGDEDHPAHQLLLDLHNYSSIESN
LTFVGLAGLRDPPRKEVRQAIEDCKAAGIRVMVITGDNKNTAEAICHEIGVFGPYDDISSKSLTGREFM
GLRDKKTHLRQSGGLLFSRAEPRHKQEIVRLLKEDGEVVAMTGDGVNDAPALKLADIGIAMGIAGTEVA
KEASDMVLADDNFSTIVVAVGEGRSIYNNMKAFIRYMISSNIGEVASIFLTAALGIPEGMIPVQLLWVN
LVTDGPPATALGFNPPDGDVMKKPPRKSDDSLISAWILFRYLVIGFYVGIATVGVFIIWYTRHTFMGID
LSGDGHSLVTYSQLANWGHCESWKNFSASPFTAGSQVFNFDANPCEYLRSGKIKASTLSLTVLVAIEMF
NSLNALSEDCSLVRMPPWVNPWLLLAMSVSFGLHFLILYVPFLAQVFGIVPLSLNEWLLVLAVALPVIL
IDEVLKFVGRLTSGWRHSGSRRPSKSKPE

Protein No.: I-782

Protein name and Species:

Serine/threonine-protein kinase SRK2C OS=Arabidopsis thaliana GN=SRK2C PE=1 SV=1

Accession: sp|Q9M9E9|SRK2C_ARATH

Unused ProtScore: 3.02

Seq Cov %: 6.4

ITRAQ Changed Ratio:

117: 115=E3TL: D3TL=0.54

116: 114=E5TL: D5TL=0.27

Annotated typical Precursor MS Region:

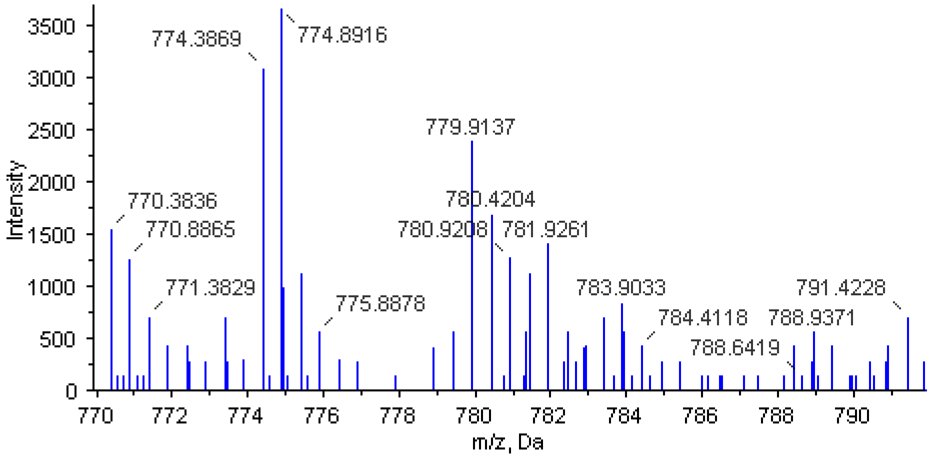

Sequence Coverage and Matched peptides: in different color

MERYEIVKDIGSGNFGVAKLVRDKFSKELFAVKFIERGQKIDEHVQREIMNHRSLIHPNIIRFKEVLLT
ATHLALVMEYAAGGELFGRICSAGRFSEDEARFFFQQLISGVNYCHSLQICHRDLKLENTLLDGSEAPR
VKICDFGYSKSGVLHSQPKTTVGTPAYIAPEVLSTKEYDGKIADVWSCGVTLYVMLVGAYPFEDPSDPK
DFRKTIGRILKAQYAIPDYVRVSDECRHLLSRIFVANPEKRITIEEIKNHSWFLKNLPVEMYEGSLMMN
GPSTQTVEEIVWIIEEARKPITVATGLAGAGGSGGSSNGAIGSSSMDLDDLDTDFDDIDTADLLSPL

Protein No.: I-783

Protein name and Species:

Nonsense-mediated mRNA decay protein, putative OS=Ricinus communis GN=RCOM_0241560 PE=4 SV=1

Accession: tr|B9T5L2|B9T5L2_RICCO

Unused ProtScore: 3.02

Seq Cov %: 4.5

ITRAQ Changed Ratio:

117: 115=E3TL: D3TL=0.62

116: 114=E5TL: D5TL=0.38

Annotated typical Precursor MS Region:

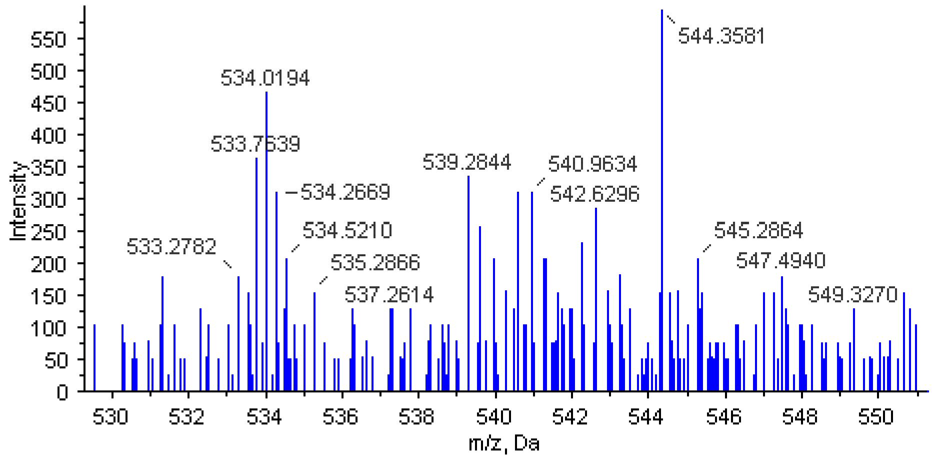

Sequence Coverage and Matched peptides: in different color

MAEEAGMFMVRQTIGSVLCCKCGIPMQPNAVNMCVKCLRSEVDITEGLKKRVIIKHCPECDTYLQPPYT
RIKAQLESKELMTFCVRTLDLKHAGVTLVHAEFIWTEPHSKRIKLRCRVQKEVHRGAVLEQSYIVEFVQ
QESMCESCSRVQANPDQWVASVQLRQHVSHRRTFFYLEQLILKHDAAARAIKIKQMDQGIDFFFGNRSH
GVKFVEFLGKVAPVRSRHDKQLVSHDPKSNNYNYK**YTFSVEISPICR**EDLVCLPPKVAVSLGNLGPLVI
CTKVTNSIALLDPLTLRHCYLDGDQYWRSSFKSLLTSRQLVEYIVLDVDIISPEVNIGSSKYAMAEVQV
ARLSDFGKNDTIFFIKTHLGHILKPGDQALGYDLYGANSNDIELDKYK**GLVLPEAILIK**KSYEEKRQRK
RGKPRAWKLKSLEMEVDDSRGRNDQEKMDTEYEQFLRDLEENAELRF**NVSLYR**NKEYQPSEMASVTDGE
DVPSIPLEELLADLDISEMEDAADDMRE

Protein No.: I-784

Protein name and Species:

ADP-ribosylation factor, putative OS=Ricinus communis GN=RCOM_0910690 PE=3 SV=1

Accession: tr|B9RTH6|B9RTH6_RICCO

Unused ProtScore: 3

Seq Cov %: 15.9

ITRAQ Changed Ratio:

117: 115=E3TL: D3TL=1.64

116: 114=E5TL: D5TL=1.77

Annotated typical Precursor MS Region:

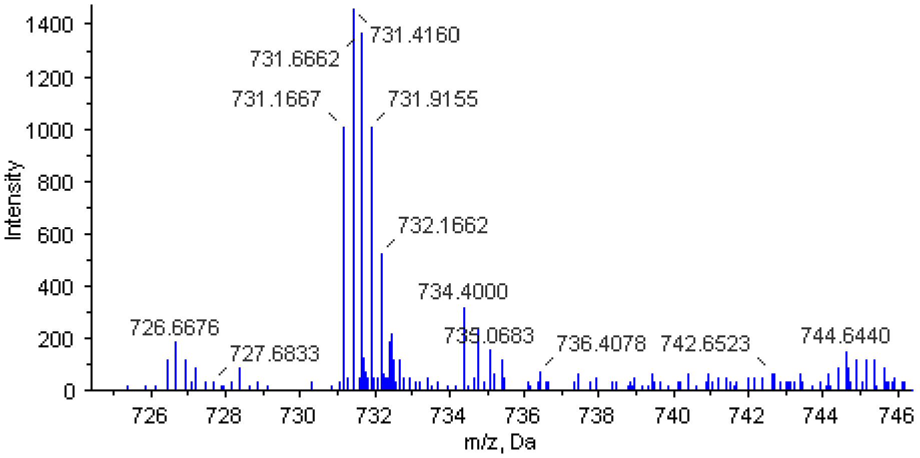

Sequence Coverage and Matched peptides: in different color

MGGFISRFWFMLFPAKEYK**IVVVGLDNAGK**TTTLYKLHLGEVVTTHPTVGSNVEELVYKNIR**FEVWDLG**
GQDRLRTSWATYYRGTHAVIAVIDSTDRARISIMKDELFRLLGHEDLQHSVILVLANKQDLK**DAMTPAE**
ITDALSLHSIKNHDWHIQACCALTGEGLYDGLGWIAQRVTGKAPS

Protein No.: I-789

Protein name and Species:

Putative uncharacterized protein OS=Ricinus communis GN=RCOM_1588080 PE=4 SV=1

Accession: tr|B9R730|B9R730_RICCO

Unused ProtScore: 2.98

Seq Cov %: 10.3

ITRAQ Changed Ratio:

117: 115=E3TL: D3TL=0.10

116: 114=E5TL: D5TL=0.14

Annotated typical Precursor MS Region:

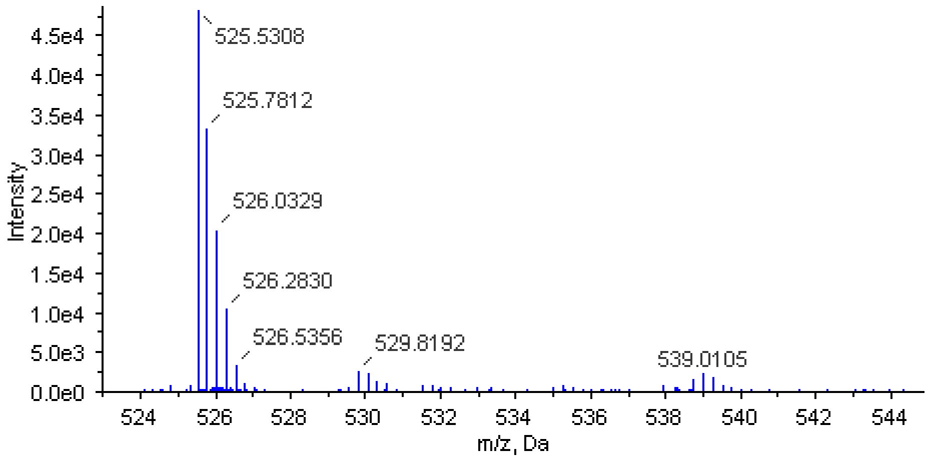

Sequence Coverage and Matched peptides: in different color

MSWWWAGAIGAAKKKQEEDDAPRSFQSVALVLGVTGIVGNSLAEILPLSDTPGGPWKVYGVARRPRPTW
NADHPVEYIQCDISDSAETQSKLSQLTDITHIFYVTWTNRQSEDENCEINGLMFR**NVLQAVIPNAPNLR**
HICLQTGAKHYVGPFESLGK**IQTHDPPFTEDLPR**LDAPNFYYTLEDIMFEEVAKKEGLTWSIHRPDQIF
GFSPYSLMNIIGTLCVYATICK**HEGLPLLFPGTK**AAWNCYSVASDADLIAEHQIWASVDPYAKDEAFNC
NNGDVFKWKHFWKVLAEQFGIEEYGFEEGEKRLSLVEMMKGKEAVWEEIVSENQLQPTKLDEVAVWWFV
DLMLGGEAVISSMNKSK**EHGFLGFR**NSKNSFASWIDKMKAYKIVP

Protein No.: I-790

Protein name and Species:

Cycloartenol synthase OS=Ricinus communis PE=1 SV=1

Accession: sp|Q2XPU6|CAS1_RICCO

Unused ProtScore: 2.98

Seq Cov %: 3.6

ITRAQ Changed Ratio:

117: 115=E3TL: D3TL=0.48

116: 114=E5TL: D5TL=1.16

Annotated typical Precursor MS Region:

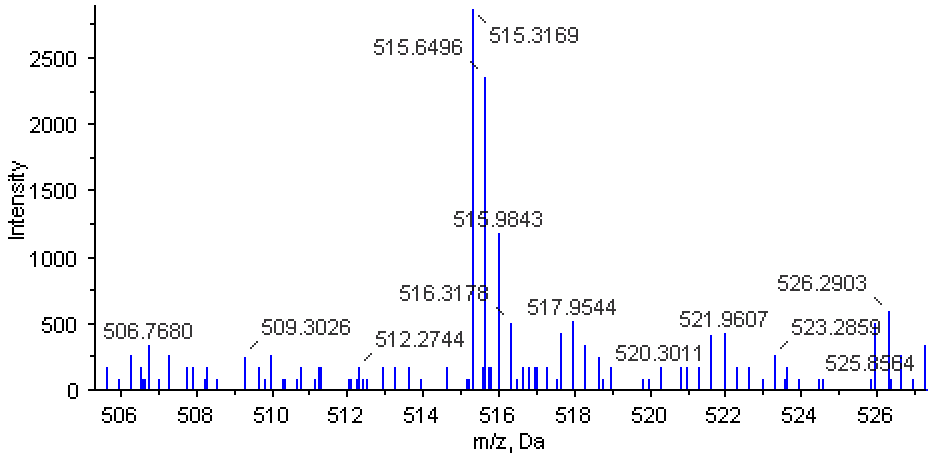

Sequence Coverage and Matched peptides: in different color

MWKLRIAEGSGNPWLRTTNDHIGRQVWEFDSSKIGSPEELSQIENARQNFTKNRFIHKHSSDLLMRIQF
SKENPICEVLPQVKVKESEQVTEEKVKITLRRALNYYSSIQADDGHWPGDYGGPMFLMPGLIIALSITG
ALNAILSEEHKREMCRYLYNHQNRDGGWGLHIEGPSTMFGSVLCYVSLRLLGEGPNEGEGAVERGRNWI
LKHGGATAITSWGKMWLSVLGAYEWSGNNPLPPEMWLLPYILPVHPGRMWCHCR**MVYLPMSYLYGKRFV**
GPITPTVLSLRKELYTVPYHEIDWNQARNQCAKEDLYYPHPMLQDVLWATLHKFVEPILMHWPGKRLRE
KAIQTAIEHIHYEDENTR**YICIGPVNK**VLNMLCCWVEDPNSEAFKLHLPRLYDYLWLAEDGMKMQGYNG
SQLWDTAFAVQAIVSTNLIEEYGPTLKKAHSFIKKMQVLENCPGDLNFWYRHISKGAWPFSTADHGWPI
SDCTAEGIKALMLLSK**IPSEIVGEGLNANR**LYDAVNVVLSLQNGDGGFPTYELSRSYSWLEFINPAETF
GDIVIDYPYVECTSAAIQALTSFRKSYPEHQREEIECCIKKAAKFMEKIQISDGSWYGSWGVCFTYGTW
FGIKGLVAAGKSFGNCSSIRK**ACDFLLSK**QCPSGGWGESYLSCQKKVYSNLEGDRSHVVNTAWAMLSLI
DAGQAERDPTPLHRAARYLINAQMENGDFPQQEIMGVFNRNCMITYAAYRDIFPIWALGEYRCRVLKAS

Protein No.: I-794

Protein name and Species:

Lactoylglutathione lyase [Vitis vinifera] OS=Vitis vinifera GN=VIT_10s0116g01660 PE=4 SV=1

Accession: tr|F6H7L5|F6H7L5_VITVI

Unused ProtScore: 2.95

Seq Cov %: 23

ITRAQ Changed Ratio:

117: 115=E3TL: D3TL=0.24

116: 114=E5TL: D5TL=0.19

Annotated typical Precursor MS Region:

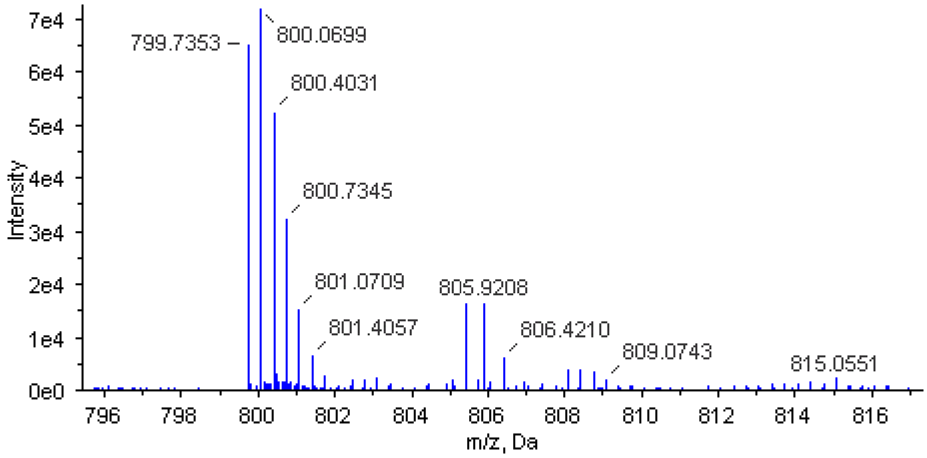

Sequence Coverage and Matched peptides: in different color

MAEAAPVVPSDELLEWPKKDKRR**FLHVVYR**VGDLDRTIK**FYTECFGMK**LLRKRDIPEEKYSNAFLGFGP
EETNFVVELTYKEDDETVLEYLLNCFQQQAPRYQLKLLRKVFDPWKLAAPVVPSDELLEWPKKDKRR**FL**
HVVYRVGDLDRTIK**FYTECFGMK**LLRKRDIPEEKYTNAFLGFGPEETNFVVELTYNYGVDKYDIGTGFG
HFAIATQDVYKMVEDIRAKGGIITREPGPVKGGKSVIAFAK**DPDGYIFELIQRGPTPEPLCQVMLR**VGD
LERSIKFYEKALGMKMVKKTDRPEYKYSIAMMGYAEEHETTVLELTYNYGVTEYTK**GNAYAQVAISTDD**
VYKSAEVVNLVTKELGGK**ITRQPGPIPGLNTKITSFLDPDGWKTVLVDNEDFLK**ELHKEE

Protein No.: I-799

Protein name and Species:

protein argonaute 4A-like [Vitis vinifera] OS=Vitis vinifera GN=VITISV_044457 PE=4 SV=1

Accession: tr|A5BG74|A5BG74_VITVI

Unused ProtScore: 2.93

Seq Cov %: 2.2

ITRAQ Changed Ratio:

117: 115=E3TL: D3TL=0.55

116: 114=E5TL: D5TL=2.21

Annotated typical Precursor MS Region:

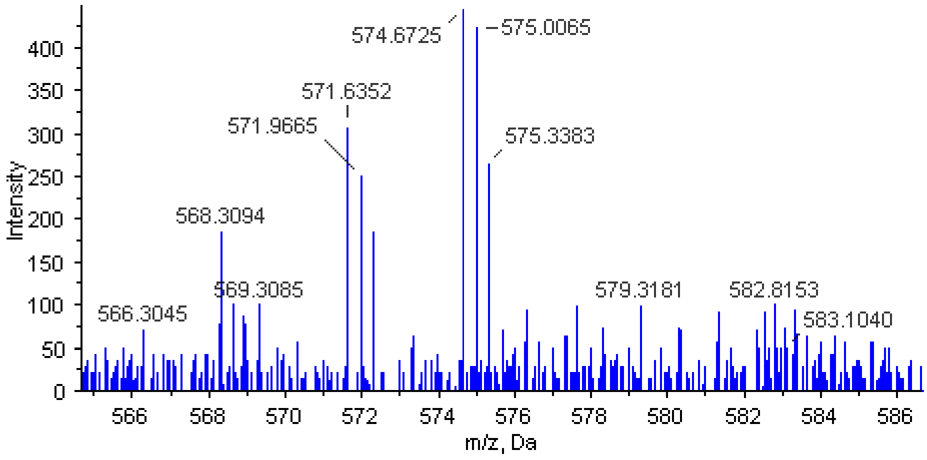

Sequence Coverage and Matched peptides: in different color

MHPRPALMLPCIQPSRPALMLPCIQPLRQSYSSPTSEAHEFHVLQVLYKYLASCNRILKRRLGRFVKNR
TVCPVRWSDRWFERFNAGSTTKRFFETDRTEKLTGRRFDRSDRPNLTMESQXPPNAEPXKRSSSIPNRL
PMARRGLGRTGETIQLVXNHFKVSMHSNANTGAHFYQYNVSLAXEDGHPADAKDIGRKVMDKVHETYHT
EMAXMSFAYDGEKSLFTIGSLPSKKLRFTVVLEDASSNRISTXRNIDXPDDGSDRKRSRRPYHSKTFNV
EISFAAKFPMDSIVRASYGQPSKHLQDAAX**VLDIILR**QHAAKKGCLVVRQSFFDNLPXNFTPLGGGVLG
CRGFNSSFRATQGGLFLNMDVSTTLVIQPDPVRDFLVSNQNVKDMYHIDWSKLCTLVSLQRYTKPLSTQ
QRSSLVEKSRQKPQERMRALKSNKYDANPMLRSSGISISTQFTQVEGRILPTPSSISVVISLQLKSGNG
QDLSPRNGRWNFNNKELAQPTKIDPWLIASFSSRCNMKTLIQDLIKCAKMKGISMGYPAEIFTENPQYM
REPAPVRVDKMISTMMSQFRRLPQFILCILPQKKNCDIYGPWKRQCLSGCGVPTQCIAPSTPV**VNDQYL**
TNLLLKINAKLGGLNSLLTMGYCPSLRLISTIPTLILGMDVSHGSPGRADVPSIAAVVSSRHWPSISQY
RATVRTQSPKLEMIDSLFEPLPNSKDSGIIQACKLLDEQWHPKFMVIIAQKNHHTRFLQNGSPSNVPPG
LSPLYHLQSQVQSGQAGLRITMYCWMSLAFQQMTCSNLCIPCVXLAPVCYAHLAAAQVAQFIKFEDLPE
SSSGHAAAPVPQLPSFHEKVADTMFFC

Protein No.: I-801

Protein name and Species:

S-adenosylmethionine synthase 1 OS=Elaeagnus umbellata GN=SAMS1 PE=2 SV=1

Accession: sp|Q9AT56|METK1_ELAUM

Unused ProtScore: 2.92

Seq Cov %: 26

ITRAQ Changed Ratio:

117: 115=E3TL: D3TL=1.60

116: 114=E5TL: D5TL=0.96

Annotated typical Precursor MS Region:

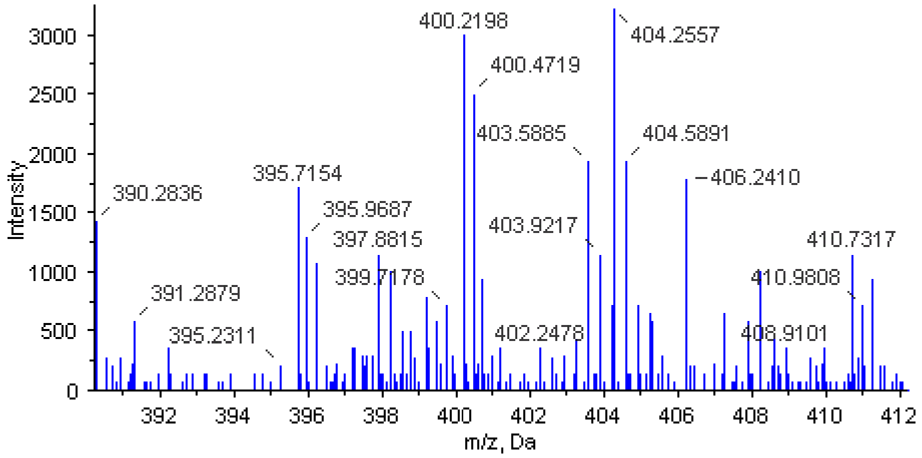

Sequence Coverage and Matched peptides: in different color

METFLFTSESVNEGHPDK**LCDQISDAVLDACLAQDPDSK**VACETCSKTNMVMVFGEITTK**ANVDYEK**IV
RDTCRAIGFVSDDVGLDADNCK**VLVNIEQQSPDIAQGVHGHFTK**RPEEIGAGDQGHMFGYATDETPEYM
PLSHVLATKLGARLTEVRKNGTCPWLRPDGK**TQVTVEYYNENGAMVPVR**VHTVLISTQHDETVTNDEIA
ADLKEHVIKPVIPEKYLDEK**TIFHLNPSGRFVIGGPHGDAGLTGR**KIIIDTYGGWGAHGGGAFSGKDPT
KVDRSGAYIVRQAAK**SIVANGLAR**RCLVQVSYAIGVPEPLSVFVDSYGTGQIPDKEILNIVKENFDFRP
GMITINLDLKRGGNGRFLKTAGYGHFGRDDPDFTWEIVKPLKWEKPQS

Protein No.: I-807

Protein name and Species:

60S ribosomal protein L14, putative OS=Ricinus communis GN=RCOM_1169110 PE=4 SV=1

Accession: tr|B9SS61|B9SS61_RICCO

Unused ProtScore: 2.9

Seq Cov %: 13.4

ITRAQ Changed Ratio:

117: 115=E3TL: D3TL=1.51

116: 114=E5TL: D5TL=0.33

Annotated typical Precursor MS Region:

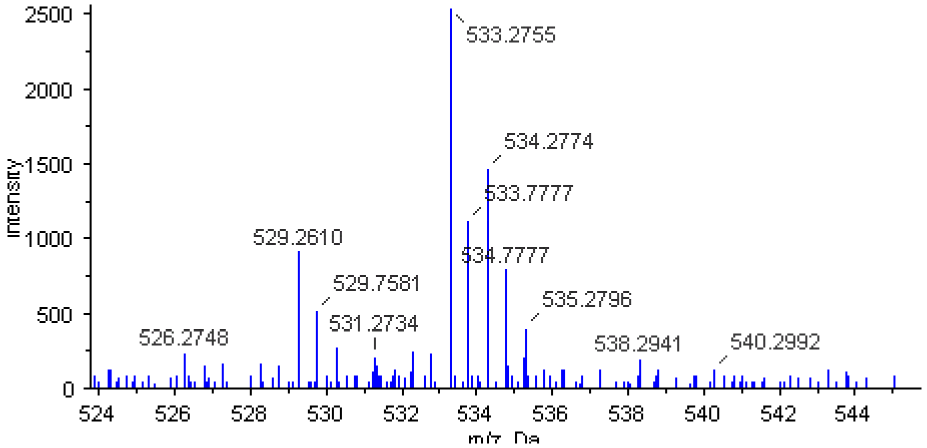

Sequence Coverage and Matched peptides: in different color

MPFKRYVEIGRVALVNYGKDYGKLVVIVDVIDQNRALVDAPDMVRSQMNFKRLQLTDIKIDINRVPKKK
NLIEAMEKADVKNKWESSSWGRKLIVQKRRAALNDFDRFKLMLAKIKRGGLIRQELAKLKKETAA

Protein No.: I-808

Protein name and Species:

Thioredoxin (Fragment) OS=Populus trichocarpa GN=PtrTrxh2 PE=3 SV=1

Accession: tr|B9IJS4|B9IJS4_POPTR

Unused ProtScore: 2.9

Seq Cov %: 25.2

ITRAQ Changed Ratio:

117: 115=E3TL: D3TL=0.08

116: 114=E5TL: D5TL=0.30

Annotated typical Precursor MS Region:

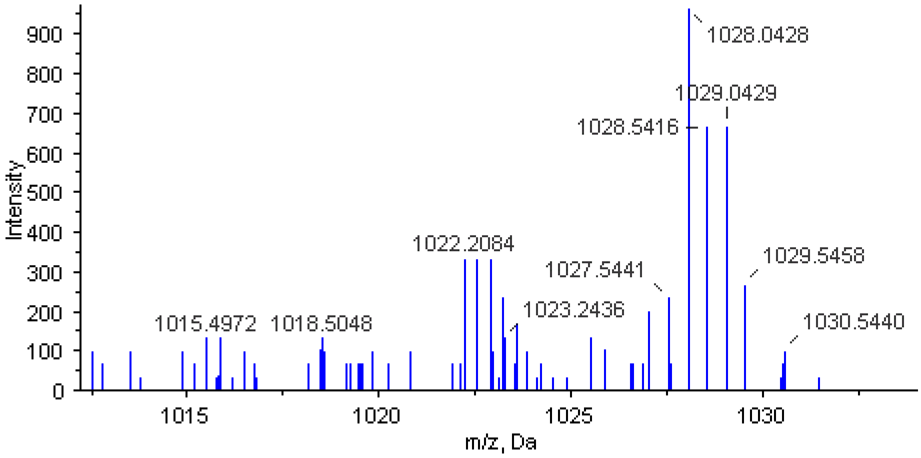

Sequence Coverage and Matched peptides: in different color

IGSILSYLFGASAAGEDSASDGQSGVTAFHSSARWQLHFNSIKNTNQLMVIDFAASWCGPCKHMEPAVH
AMAAK**FTDVQFAKIDVDELPDVAQEFGVQAMPTFVLVK**KGNEVDRVVGAQKEELQRKIEKHR

Protein No.: I-813

Protein name and Species:

Ras-related protein RABA6b-like OS=Populus trichocarpa GN=POPTRDRAFT_835110 PE=3 SV=1

Accession: tr|B9IEB2|B9IEB2_POPTR

Unused ProtScore: 2.87

Seq Cov %: 19.8

ITRAQ Changed Ratio:

117: 115=E3TL: D3TL=1.77

116: 114=E5TL: D5TL=2.51

Annotated typical Precursor MS Region:

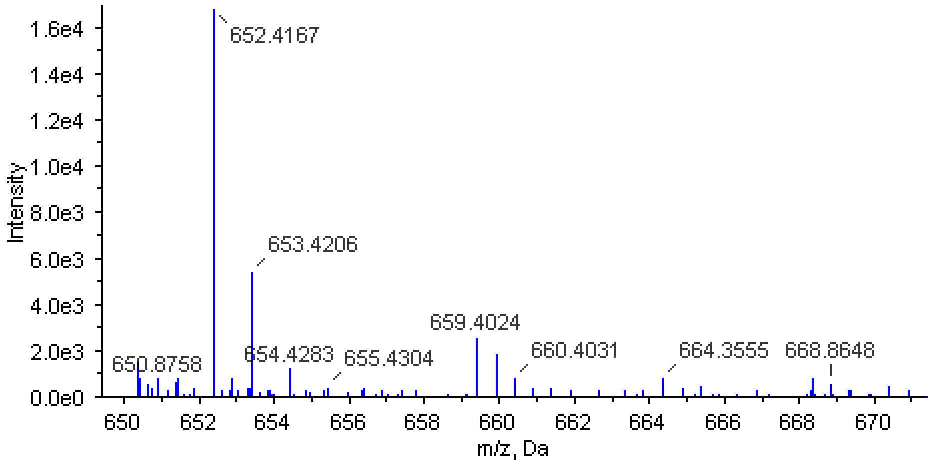

Sequence Coverage and Matched peptides: in different color

MADSYDEECDYLFKAVLIGDSAVGKSNLLSRFSRDEFRLDSKPTIGVEFAYRNIRVGDKLIKAQIWDTA
GQERFRAITSSYYRGALGALLVYDITRRATFDNVKKWLHELRDFGSSDMVVVLVGNKSDISHLREVDED
DGKNLAETEALYFMETSALENLNVEEAFMQMITRIHEIASQKSLEAKKNETTISTIKALPVGKEVINID
DEVSATKQASYCCSY

Protein No.: I-817

Protein name and Species:

Pro-hevein OS=Hevea brasiliensis GN=HEV1 PE=1 SV=2

Accession: sp|P02877|HEVE_HEVBR

Unused ProtScore: 2.85

Seq Cov %: 65.7

ITRAQ Changed Ratio:

117: 115=E3TL: D3TL=0.62

116: 114=E5TL: D5TL=0.34

Annotated typical Precursor MS Region:

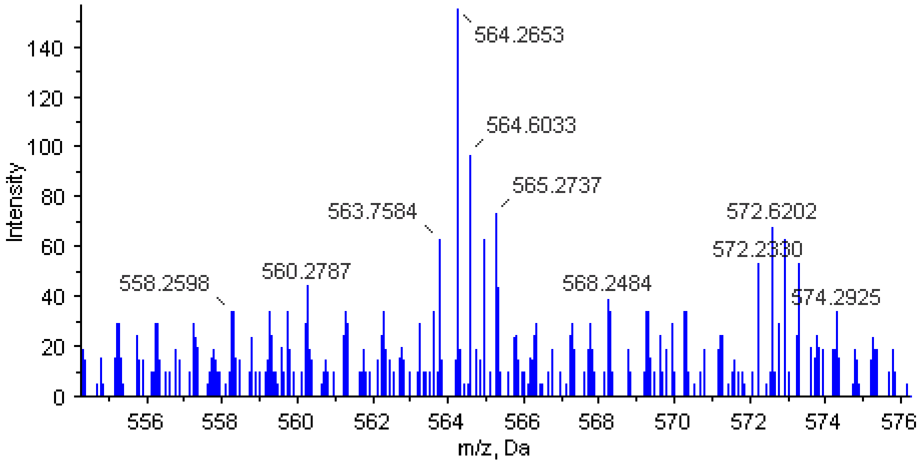

Sequence Coverage and Matched peptides: in different color

MNIFIVVLLCLTGVAIAEQCGRQAGGKLCPNNLCCSQWGWCGSTDEYCSPDHNCQSNCKDSGEGVGGGS
ASNVLATYHLYNSQDHGWDLNAASAYCSTWDANKPYSWRSKYGWTAFCGPVGAHGQSSCGKCLSVTNTG
TGAKTTVRIVDQCSNGGLDLDVNVFRQLDTDGKGYERGHITVNYQFVDCGDSFNPLFSVMKSSVIN

Protein No.: I-819

Protein name and Species:

Monodehydroascorbate reductase I OS=Pisum sativum GN=MDHAR1 PE=4 SV=1

Accession: tr|Q66PF9|Q66PF9_PEA

Unused ProtScore: 2.84

Seq Cov %: 14.3

ITRAQ Changed Ratio:

117: 115=E3TL: D3TL=1.96

116: 114=E5TL: D5TL=1.18

Annotated typical Precursor MS Region:

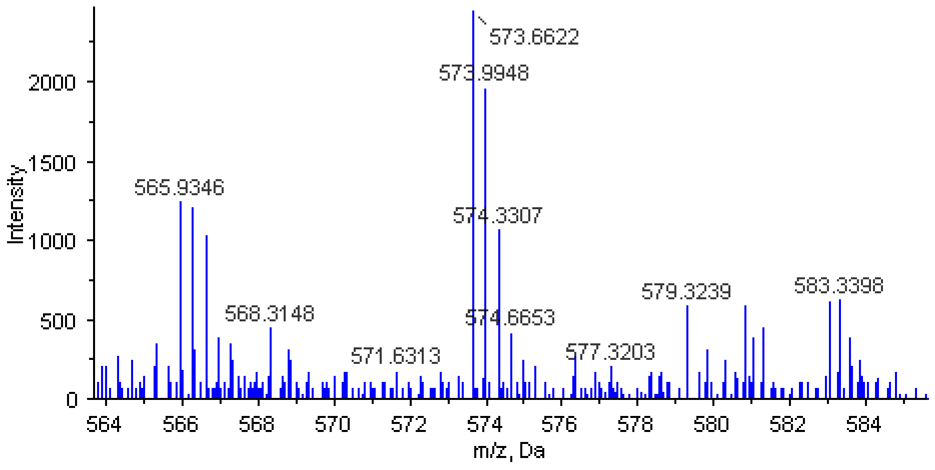

Sequence Coverage and Matched peptides: in different color

MVHSFKYILIGGGVSAGYAAREFVK**QGVHPGELAIISK****EAVAPYERPALSK**AYLFPESPAR**LPGFHTCV**
GSGGERLLPEWYSEKGIQLYLSTEIVSADLAAKFLKSANGEHFDYQTLVIATGSAVIRLTDFGVIGANA
KNIFYLREVDDADKLYEAIKRKKNAKAVVVGGGYIGLELSAVLKLNDLDVTMVYPEPWCMPRLFTSEIA
AFYEGYYANK**GINIIK****GTVAVGFTANSDGEVK**EVKLKDGRVLEADIVIVGVGGRPQISLFKGQVEEQHG
GIKTDSFFK**TSVPDVYAVGDVATFPLK**LYNDVRRVEHVDHARKSAEQAAKVIFAADVRKSVEEYDYLPY
FYSRSFDLSWQFYGDNVGETVLFGDNDPASSKPKFGTYWIKEGK**VVGAFLEGGTPDENK**AIAKVARAKP
AVEDVNQLAEEGLSFASKI

Protein No.: I-825

Protein name and Species:

eukaryotic initiation factor 4A-3-like [Vitis vinifera] OS=Vitis vinifera GN=VIT_06s0080g00550 PE=4 SV=1

Accession: tr|F6HHF5|F6HHF5_VITVI

Unused ProtScore: 2.81

Seq Cov %: 11.8

ITRAQ Changed Ratio:

117: 115=E3TL: D3TL=1.72

116: 114=E5TL: D5TL=1.18

Annotated typical Precursor MS Region:

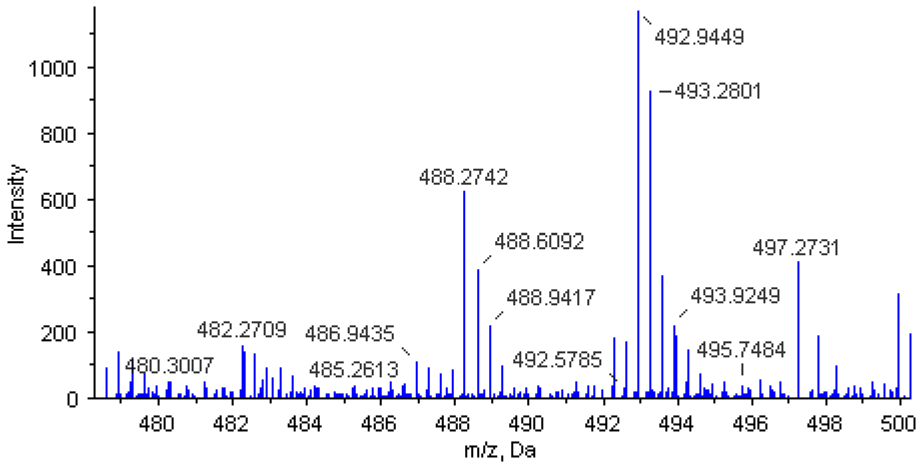

Sequence Coverage and Matched peptides: in different color

MAAAATASVVPANRGGRRNGAVDDEKLVFETTEGVEPIMSFDQMGIKDDLLR**GIYAYGFEKPSAIQQR**A
VLPIIQGR**DVIAQAQSGTGK**TSMIALTVCQMVDTSNR**EVQALILSPTR**ELASQTEKVILAIGDFINIQA
HACIGGKSVGEDIRKLEYGVHIVSGTPGRVCDMIKRRTLRTRAIKLLVLDESDEMLSRGFKDQIYDVYR
YLPPELQVVLISATLPNEILEITNKFMTDPVRILVK**RDELTLEGIK**QFFVAVEREEWKFDTLCDLYDTL
TITQAVIFCNTKRKVDWLTEKMRSNNFTVSSMHGDMPQKERDAIMAEFRSGTTRVLITTDVWARGLDVQ
QASHVSLVINYDLPNNRELYIHRIGRSGRFGRKGVAINFVKTDDIKILRDIEQYYSTQIDEMPMNVADL
I

Protein No.: I-827

Protein name and Species:

Ribosomal protein L5A，60S [Hevea brasiliensis] OS=Populus trichocarpa GN=POPTRDRAFT_732569 PE=3 SV=1

Accession: tr|B9IAZ4|B9IAZ4_POPTR

Unused ProtScore: 2.81

Seq Cov %: 7.7

ITRAQ Changed Ratio:

117: 115=E3TL: D3TL=0.31

116: 114=E5TL: D5TL=0.35

Annotated typical Precursor MS Region:

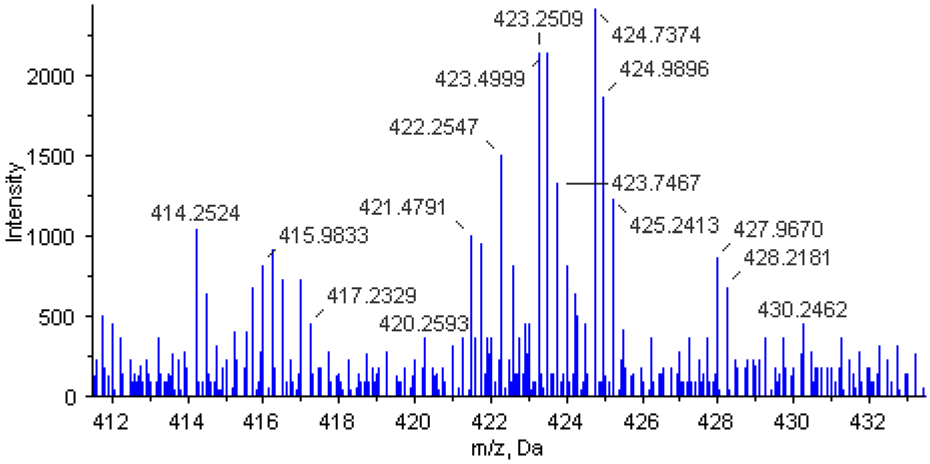

Sequence Coverage and Matched peptides: in different color

MAFAKAQKSRAYYKRFQVKFKRRREGKTDYRARIRLINQDKNKYNTPKYRFVMRFTNKDITAQIIHASM
AGDIVVAAAYSHELPRYGLEVGLTNYAAAYCTGLLLARRVLKKLEMDEEYEGNVEATGEDYSVEPADSR
RPFR**ALLDVGLVR**TTTGNRVFGALK**GALDGGLDIPHSDK**RFAGFNKDGK**QLDAEVHR**KYIYGGHVAAYM
RNLMEDEPEKYQSHFSEYIKR**GIEPDNMEELYK**KVHAAIRADPTAKKSEKQLPKEHKRYNLKKLTYEER
KAKLVERLKALNSAADDDEDDE

Protein No.: I-828

Protein name and Species:

60S ribosomal protein L23A-like [Glycine max] OS=Glycine max PE=2 SV=1

Accession: tr|C6SZQ3|C6SZQ3_SOYBN

Unused ProtScore: 2.81

Seq Cov %: 13.3

ITRAQ Changed Ratio:

117: 115=E3TL: D3TL=1.75

116: 114=E5TL: D5TL=0.48

Annotated typical Precursor MS Region:

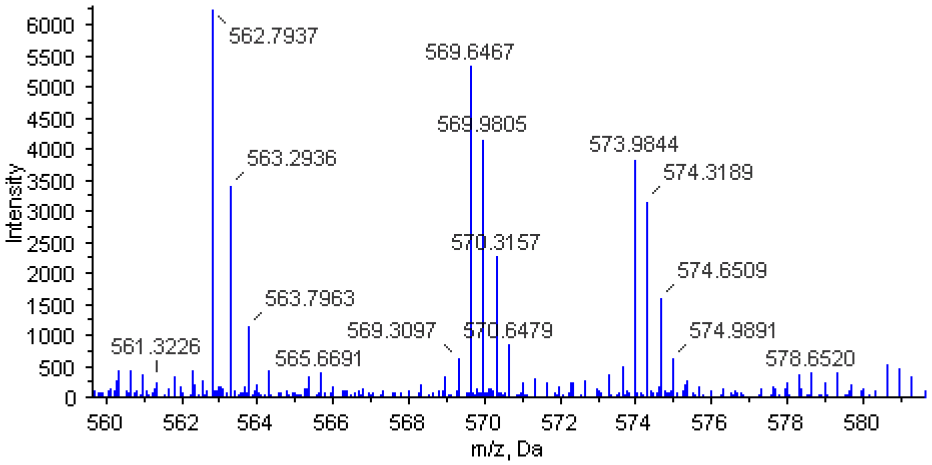

Sequence Coverage and Matched peptides: in different color

MAPKVDKKNDPKAQALKTAKAVKSGATFKKKVKKIRTSVTFHRPKTLKKDRNPKYPRISAPPRNKLDHY
QILKYPLTTESAMKKIEDNNTLVFIVDLRADKKKIKDAVKKMYDIQAKKVNTLIRPDGTKKAYVRLTPD
YDALDVANKIGII

Protein No.: I-832

Protein name and Species:

Long chain acyl-CoA synthetase 1-like OS=Populus trichocarpa GN=POPTRDRAFT_850795 PE=4 SV=1

Accession: tr|B9GSW4|B9GSW4_POPTR

Unused ProtScore: 2.78

Seq Cov %: 3.5

ITRAQ Changed Ratio:

117: 115=E3TL: D3TL=2.21

116: 114=E5TL: D5TL=1.97

Annotated typical Precursor MS Region:

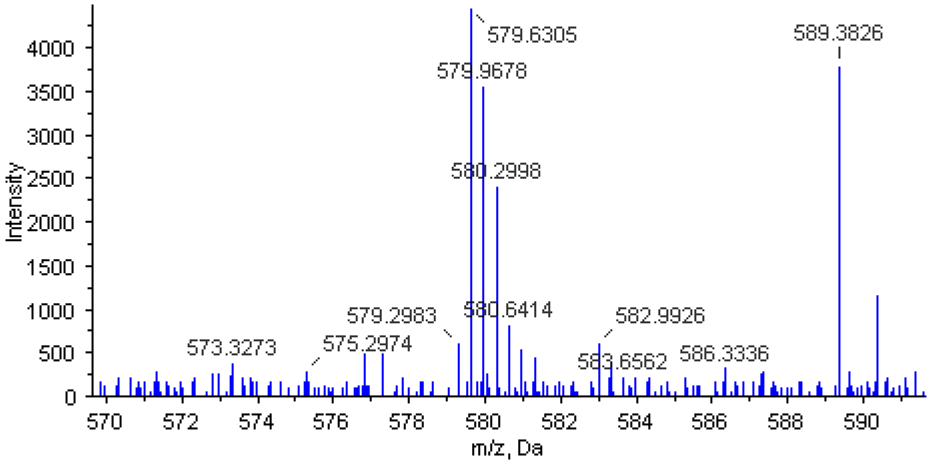

Sequence Coverage and Matched peptides: in different color

MALKAFAAQVEEGREGKDGK**PSVGPVYR**NLLSKKEYPPLDRDVTTSWDIFSSSAKKYPEYKMLGWRKFV
DGKVGPYVWKTYKEVYDEVLQISSALRASGAEPGYRVGIYGSNCPQWIEAMEACNAQSLVCVPLYDTIG
PRAVNFIIDHAEIDFVFVQDKKVKELLDPDCASAQRLKAIVCFTSLTEEEKDRAAQMGIKSYSWEEYLC
MGKENPSEIFPPQPLSICTIMYTSGTSGDPKGVVILHETVAAFVRGIDLFMEQFEDKMTTDDVYLSFLP
LAHVLDRMIEEYFFRK**GASVGYYHGDLNALR**DDLMELKPTCLAGVPRVFEKIQEGMEIPIKALQELSPL
RRNIFDVLYRYKLGWMNRGYRQK**YASPLADLLAFR**KVKAKLGGRLRLIICGGASLSTEVEEFLRVTSCA
FVIQGYGLTESCGPATMAFPDEMCMLGTVGAVAVYNDLCLEEVPEMGYDPLGNPPCGEICLRGKTLFSG
YYKNPELTRESMKDGWFHTGDIGEILPNGVVKIIDRKKNLIKLSQGEYIAIEYLENVYNNTPIVEDIWI
YGDSCKSMLVAVVVLHEENTKSWAYLNGHMGSFTELCSLHQLQKLVLSELKTTAERNKLKGFEHIKGVI
LESCPFDIEK**DLVTATLK**KKRNKLVSHYRVEIDELYQKLAEGRA

Protein No.: I-835

Protein name and Species:

Ubiquitin carboxyl-terminal hydrolase OS=Ricinus communis GN=RCOM_0004440 PE=3 SV=1

Accession: tr|B9T7A3|B9T7A3_RICCO

Unused ProtScore: 2.77

Seq Cov %: 8.1

ITRAQ Changed Ratio:

117: 115=E3TL: D3TL=1.51

116: 114=E5TL: D5TL=1.02

Annotated typical Precursor MS Region:

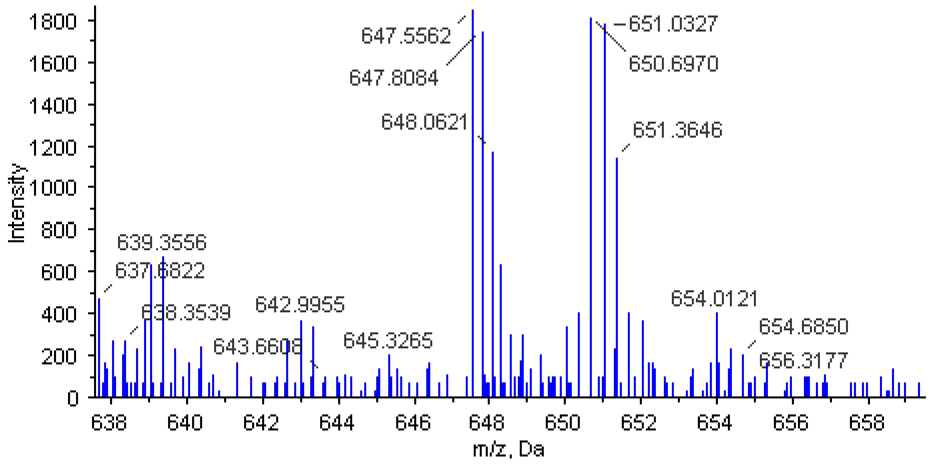

Sequence Coverage and Matched peptides: in different color

MLSVSVKWQKKVFPQVEIDTSQPPYLFKCQLFDLTGVPPERQKIMVKGGLLKDDADWATVGVKEGQK**LM**
MMGTADEIVKAPEK**GPVFMEDLPEEEQVVAVGH**TAGLFNLGNTCYMNSTVQCLHSVPELKSALVKYPSD
RRNDLDQTSHMLTTATR**ELFNELDK**SVKPVAPMQFWMVLRKKYPQFGQLHNGLFMQQDAEECWTQLLYT
LSQSLRPPSPSENPDTVKELFGIDLVSR**IHCQESGEESSETESVYSLK**CHISQDVNHLHEGLKHGLKSE
LEKASPSLGRSAIYLKESCINDLPRYLTVQFVRFFWKRESNQKAKILRKVDYPLELDVYDFCSADLRKQ
LEAPRQILRDEEGRKLGLKVNEKSSGSKDNDVKMTDAEGPSSGSGESSKATSEEGESSHKVSSLTGIYD
LVAVLTHKGRSADSGHYVAWVKQESGKWIEYDDDNPIPQREEDITKLSGGGDWHMAYICMYKARAVPM

Protein No.: I-838

Protein name and Species:

Tubulin beta-7 chain OS=Arabidopsis lyrata subsp. lyrata GN=TUB7 PE=3 SV=1

Accession: tr|D7LLA4|D7LLA4_ARALL

Unused ProtScore: 2.76

Seq Cov %: 6

ITRAQ Changed Ratio:

117: 115=E3TL: D3TL=0.63

116: 114=E5TL: D5TL=1.29

Annotated typical Precursor MS Region:

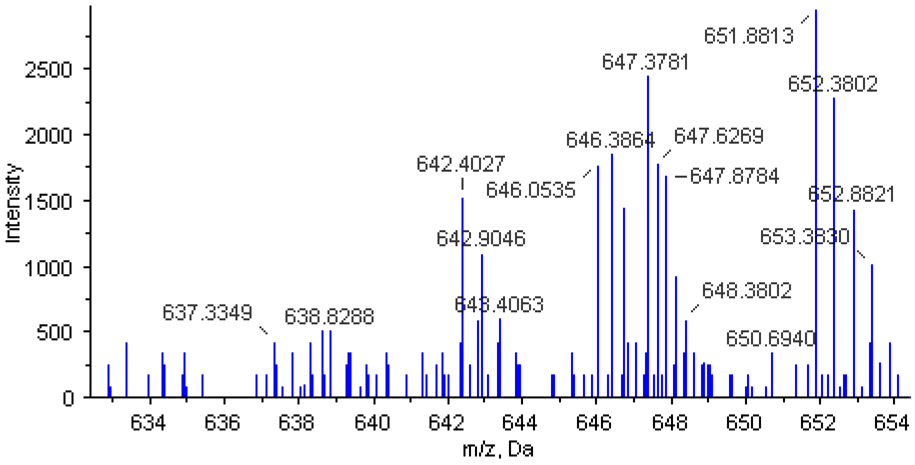

Sequence Coverage and Matched peptides: in different color

MR**EILHIQGGQCGNQIGSK**FWEVVNLEHGIDQTGRYHGDSDLQLERVNVYYNEASCGRYVPRAVLMDLE
PGTMDSVRSGPYGQIFRPDNFVFGQSGAGNNWAKGHYTEGAELIDSVLDVVRKEAENCDCLQGFQVCHS
LGGGTGSGMGTLLISKIREEYPDRMMMTFSVFPSPKVSDTVVEPYNATLSVHQLVENADECMVLDNEAL
YDICFRTLKLSTPSFGDLNHLISATMSGVTCCLRFPGQLNSDLRK**LAVNLIPFPR**LHFFMVGFAPLTSR
GSQQYRNLTVPELTQQMWDAKNMMCAADPRHGRYLTASAMFRGKMSTK**EVDEQMLNVQNK**NSSYFVEWI
PNNVKSTVCDIPPTGLKMASTFIGNSTSIQEMFRRVSEQFTAMFRRKAFLHWYTGEGMDEMEFTEAESN
MNDLVSEYQQYQDATADEEGEYEDEEAEYEQEEGY

Protein No.: I-839

Protein name and Species:

Dipeptidyl peptidase IV, putative OS=Ricinus communis GN=RCOM_1243220 PE=4 SV=1

Accession: tr|B9RSG5|B9RSG5_RICCO

Unused ProtScore: 2.75

Seq Cov %: 6.8

ITRAQ Changed Ratio:

117: 115=E3TL: D3TL=1.77

116: 114=E5TL: D5TL=0.98

Annotated typical Precursor MS Region:

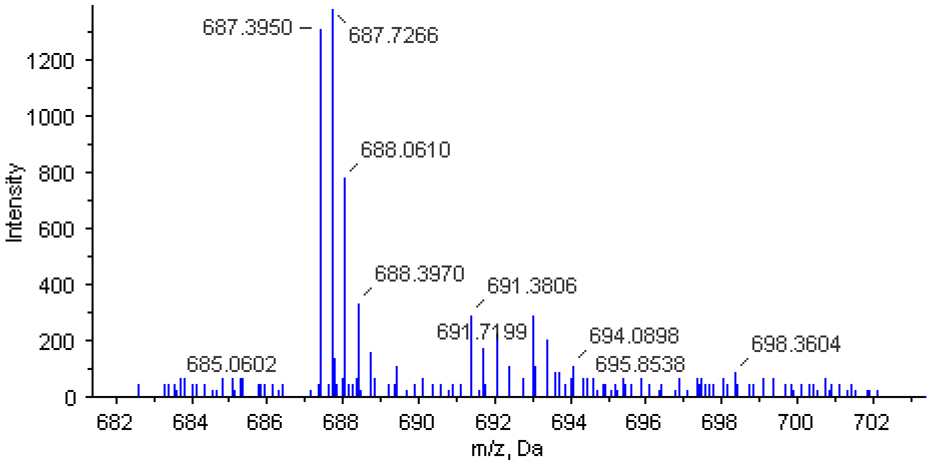

Sequence Coverage and Matched peptides: in different color

MADNTTPQNLDDCILFPVEDIVQSPLPGYGAPTSISFSPDDALITFLFSRK**VFAFDLK**TFK**QELFFSPP**
DGGLDESNISPEEKLRRERSRERGLGVTQYEWVKTSFKKKAVMVPLPAGIYFQELSSSKPELKLPSSPL
SPIIDPHLSPDGTMLAYVK**DSELHVLNLLYNESK**QLTFGSQGNTVTHGLAEYIAQEEMDRKNGYWWSLD
SKFIAFTQVDSSGIPLFRIMHQGKSSVGLESQEDHAYPFAGASNVKVRLGVVSIAGGSITWMDLVCGGT
EELDNEDEYLTRVNWMRGDILTAQVLNRSHTKLRIIKFDIKTGQGKVILVEEQDKWVNLHDCFTPLDKS
VTKYSGGFIWASEKTGFRHLYLHDANGTCLGPITEGEWMVEQIAGVNEAAGLVYFTATLDGPLEFNLYC
TKLFRDDSQNFLGPVRLTHGKGKHVVVLDHHMR**NFVDIHDSLDFPPR**VLYTSLHDGSVIMPLYEQPFTI
PRFKRLELEPPEIVQVQASDGTILYGALYKPDPTKFGPPPYKTLISVYGGPCVQYVCDSWLNTVDMRAQ
FLRSKGILVWKLDNRGSARRGLKFEGSLKYNAGRIDAEDQLTGTEWLIKQGLAKVGHIGVYGWSYGGYM
SAMILARFPDVFRCAVSGAPVTSWDGYDTFYTEKYMGLPSQNPSGYEYSSVMHHVHKLKGRLLLVHGMI
DENVHFRHTARLVNALVAAGKPYELLIFPDERHTLRWHRSRVYMEERIWEFVERSL

Protein No.: I-840

Protein name and Species:

Glutathione-s-transferase theta, gst, putative OS=Ricinus communis GN=RCOM_1299540 PE=3 SV=1

Accession: tr|B9S069|B9S069_RICCO

Unused ProtScore: 2.75

Seq Cov %: 16.7

ITRAQ Changed Ratio:

117: 115=E3TL: D3TL=0.38

116: 114=E5TL: D5TL=1.15

Annotated typical Precursor MS Region:

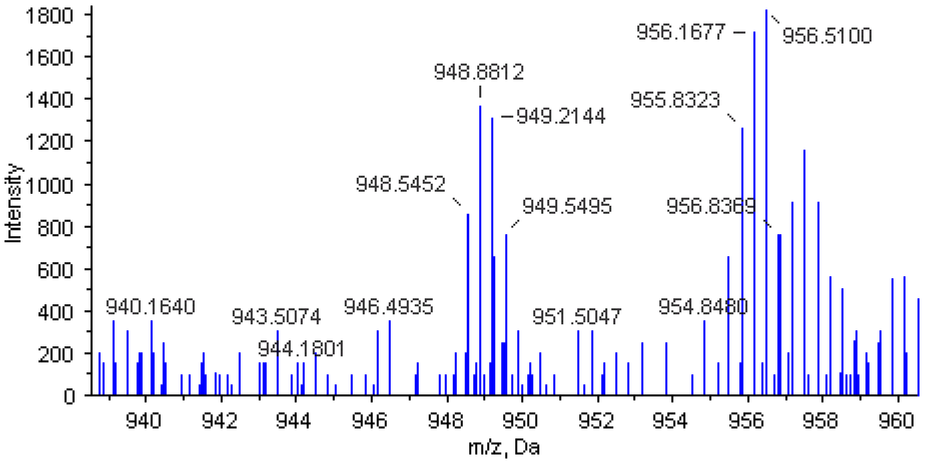

Sequence Coverage and Matched peptides: in different color

MSSVEGKLKLYSYWRSSCSCRVR**IALNLK****GISYQYIPVNLVK**GEQF**TPEFLK**LNPIGYVPVLVDGDIVI
SDSFAILMYLEDKYPQHPLLPSDLQKK**AISIQAANIVSSSIQPLQNLAVLK**FIEEKVGPDQKVPWVQFH
IEKGFTALEKLLKDHAGKYATGDEVSLADVFLEPQIHAAINRFDVDMNKFPLLLRLHEAYNELPAFQNG
MPDKQLDAPSSSSS

Protein No.: I-844

Protein name and Species:

Pyridoxine kinase, putative OS=Ricinus communis GN=RCOM_1384840 PE=4 SV=1

Accession: tr|B9S7R8|B9S7R8_RICCO

Unused ProtScore: 2.72

Seq Cov %: 16.4

ITRAQ Changed Ratio:

117: 115=E3TL: D3TL=0.61

116: 114=E5TL: D5TL=0.51

Annotated typical Precursor MS Region:

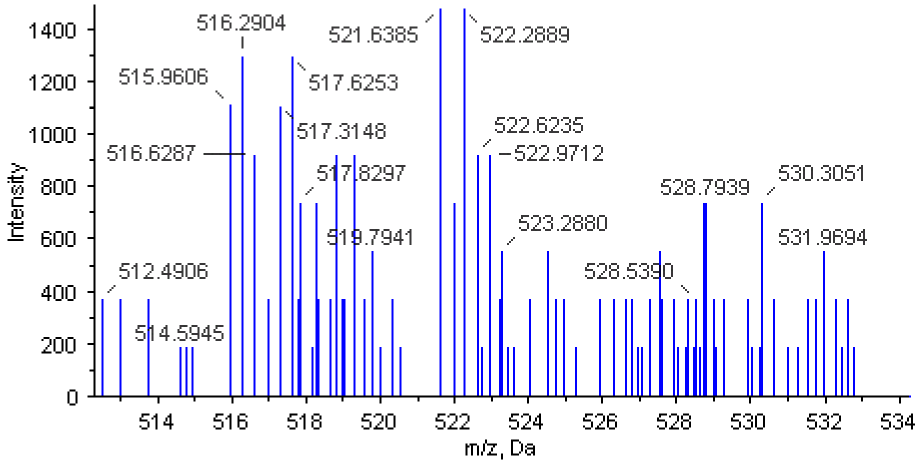

Sequence Coverage and Matched peptides: in different color

MAPPILSLALPSGTGRVLSIQSHTVQGYVGNKSAVFPLQILGYDVDPINSVQFSNHTGYPSVKGQVLNG
QQLWDLIEGLEGNDLLYYTHLLTGYIGSVSFLNTILEVVNKLRSINPK**LTYVCDPVMGDEGKLYVPPEL**
VAVYREK**VVPVASMLTPNQFEAEQLTGFR**IVTEKDGREACNTLHAAGPSKVIITSINIDGNLLLVGSHQ
KEKDQPPEQFKIEIPKIPAYFTKYPDNLGTAAELAVSSLQALLQRTVNDYRSVGYDPQSSSLEIRLIQS
QDDIRHPQVKFKAEKYD

Protein No.: I-845

Protein name and Species:

rhamnogalacturonate lyase OS=Populus

Accession: tr|B9H6S4|B9H6S4_POPTR

Unused ProtScore: 2.72

Seq Cov %: 8.4

ITRAQ Changed Ratio:

117: 115=E3TL: D3TL=0. 21

116: 114=E5TL: D5TL=0. 18

Annotated typical Precursor MS Region:

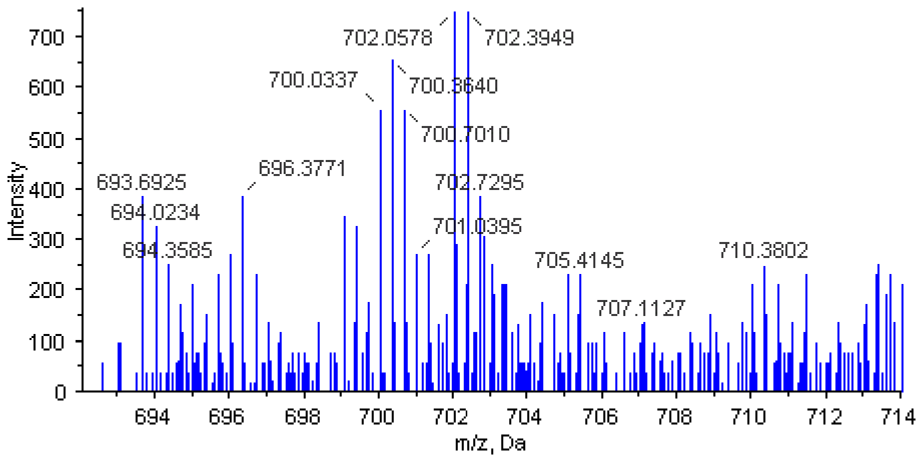

Sequence Coverage and Matched peptides: in different color

MDNGIVQVTLSKPAGIVTGIR**YDGIDNLLEVLNR**ETNRGYWDLHWNPPGGNGIFDVISGTSFRVVVEND
EQVELSFTRMWDSSLEGKYIPLNIDKRFILLRGSSGFYSYAIYEHLQEWPGFDIGETRITFKLRKDKFQ
YMAIADNRQRLMPLPDDRLPGR**CQALAYPEAVILVNPK**LPELTGEVDDKYQYSCENKDNQVHGWICFKP
PVGFWQITPSDEFRTAGPLKQNLTSHVGPTTLAMFHSSHYAGKDLVLSISPGEPWKKVFGPVFIYLNSA
SNGEDPLFLWEDAKMQMMAEVQSWPYSFPASEDFQKSEQRGNVCGRLLVKDRNISDDYILASGAYVGLA
PPGDVGSWQTECKDYQFWTR**ADENGYFSIK**NIRTGDYNLYAWVPGFLGDYRWDAIVNIISGCDMDMGDL
VYEPPR**DGPTLWEIGIPDR**SAAEFYIPGPDPKFMNNLYANHPDRFRQYGLWGRYADLYPDTDLVYTVGL
SDYRKDWFFAQVVRRKDDDTQVGTTWQIKFKLDKVDRNSSYKLRVAIASATLAELQVRVNDAKAQRPLF
TSGLIGRDNAIARHGIHGLYR**LYNVNIPGARLVEGENTIFLTQPR**CTSPFQGLMYDYIRLEGPPFSGSS
NEASSSFK

Protein No.: I-864

Protein name and Species:

Glutaredoxin, grx, putative OS=Ricinus communis GN=RCOM_0936190 PE=4 SV=1

Accession: tr|B9RZ84|B9RZ84_RICCO

Unused ProtScore: 2.65

Seq Cov %: 9.4

ITRAQ Changed Ratio:

117: 115=E3TL: D3TL=0.62

116: 114=E5TL: D5TL=1.00

Annotated typical Precursor MS Region:

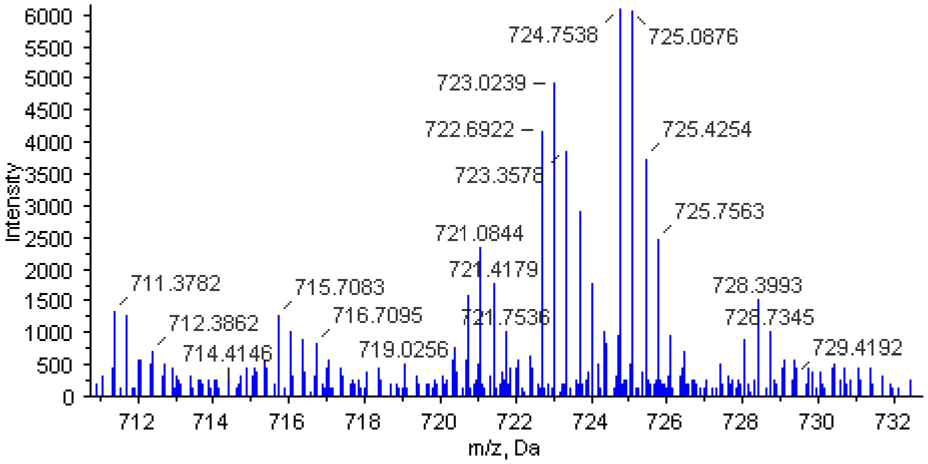

Sequence Coverage and Matched peptides: in different color

MGGSVKEVHSKTELDDVRSSGVPVIVHFWASWCDASKHMDQVFSHLSTDFPNAHFLRVEAEEQPEISEE
FSVSAVPFFVFFKDGKKVDSLEGADPSSLANKVAKAAGSVKSGEPAAPASLGMAAGPSILETVKELAKD
NGPSQVSNKVQPGLNDALEKRLQQLINSHPVMLFMKGSPEAPRCGFSQKIVDILKDEAVK**FGSFDILSD**
NEIREGLKKFSNWPTFPQLYCKGELLGGCDIAIAMHESGELKDVFRDHGVDTNSSEEVKVSEGGNGKGG
ISESTGLSSTLTSRLESLINSSPVMLFMKGKPDEPKCGFSRKVVDILREEK**VNFDSFDILSDDEVR**QGL
KVYSNWSSYPQLYIKGELIGGSDIVLEMQKSGELKRVLVEKGISPKGTLEDRLRSLVASSHVMLFMKGS
PDAPRCGFSSKVVNALR**EEGVSFGSFDILSDEEVR**QGLKVFSNWPTFPQLYYKGELIGGCDIIMELKNN
GELKSTLSE

Protein No.: I-868

Protein name and Species:

programmed cell death protein 5-like [Vitis vinifera] OS=Vitis vinifera GN=VIT_10s0003g02790 PE=4 SV=1

Accession: tr|D7TK03|D7TK03_VITVI

Unused ProtScore: 2.65

Seq Cov %: 23.5

ITRAQ Changed Ratio:

117: 115=E3TL: D3TL=0. 54

116: 114=E5TL: D5TL=0. 47

Annotated typical Precursor MS Region:

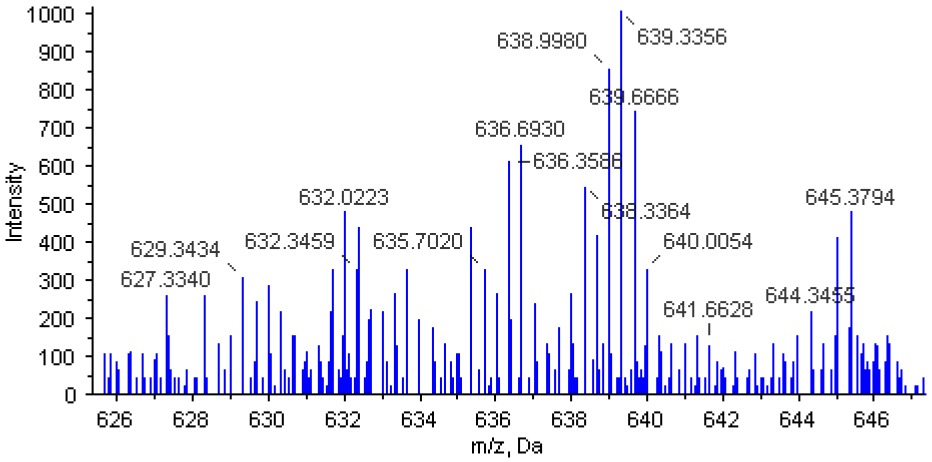

Sequence Coverage and Matched peptides: in different color

MQELMAQHGVGNHQNSEQQKAQEDAKREADERR**QMMLSQILSSEAR**ERLARIALVKPEKAR**GVEDVLLR**
AAQMGQIVEKVSEER**LISLLEQINNQTTK**QTKVTIQRRRSVLEDDD

Protein No.: I-869

Protein name and Species:

14 kDa zinc-binding protein-like [Vitis vinifera] OS=Vitis vinifera GN=VITISV_029944 PE=4 SV=1

Accession: tr|A5BDT4|A5BDT4_VITVI

Unused ProtScore: 2.64

Seq Cov %: 5.7

ITRAQ Changed Ratio:

117: 115=E3TL: D3TL=0. 30

116: 114=E5TL: D5TL=0. 34

Annotated typical Precursor MS Region:

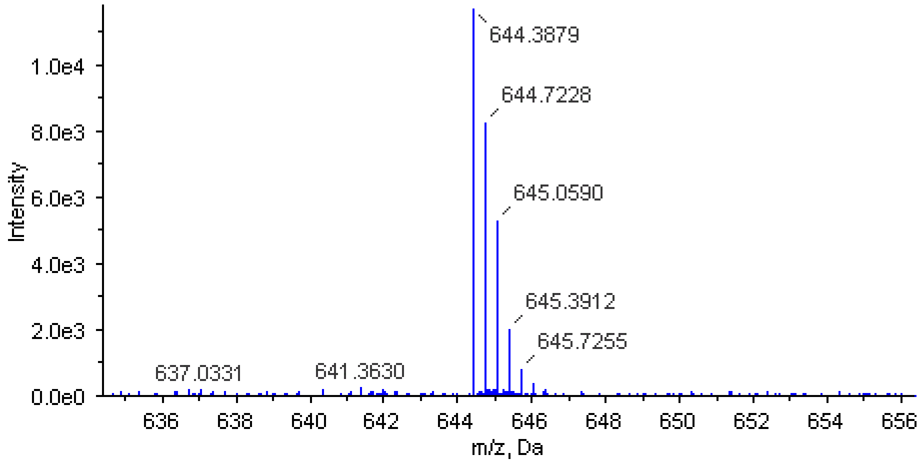

Sequence Coverage and Matched peptides: in different color

MNPSNKSRNQSRVGDINSRLGVIASHLSSAYMASEKGAAVAATPSDSPTIFDKIINKEIPATIVYEDDK
VLAFR**DIAPQAPTHILLIPK**VKDGLTGLSKYIFSSVLYTGQLKTPRHKCYCALHFERTAIHETEYCFLL
RFNLGESLSLNHMGLTLGCICSVDLVMESVGRLLANWRRHYMSIGSRVTFNRVLLIKCSYALHILLIPG
SIRNTWEIGDGFSLVRVRPRRELFDWLELGV**VLAIR**DLERMTEALLGKWLWKFVVEXDSSXRVVFESQC
CYSRELRDFDVCGDHGTQSLPMVQPMRGGRITPTRLACARRMGRAWIIYQFPRNSEAYVGKLTWTIWQV
VLMVFRGFMXFIFQAEERHSVILGHLLYTAKLVAK**QEGLEDGFR**IVINDGPSACQSVYHIHVHLLGGRQ
MNWPPG

Protein No.: I-870

Protein name and Species:

Adenylosuccinate synthetase 2, chloroplastic OS=Ricinus communis GN=PURA2 PE=3 SV=1

Accession: sp|B9SL58|PURA2_RICCO

Unused ProtScore: 2.64

Seq Cov %: 7.2

ITRAQ Changed Ratio:

117: 115=E3TL: D3TL=0.53

116: 114=E5TL: D5TL=2.38

Annotated typical Precursor MS Region:

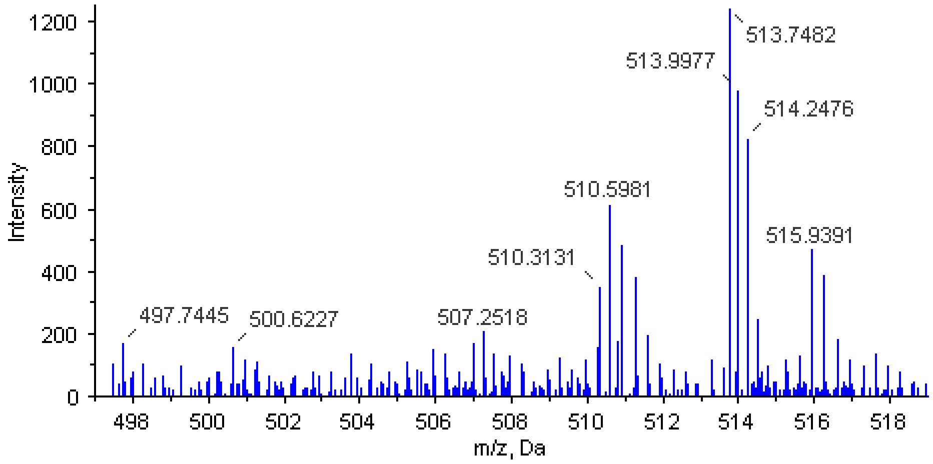

Sequence Coverage and Matched peptides: in different color

MSFTSSLRLDTNPITSPKWVHRQVPLRPHRNFVVCSIKSPSTPSLNLATDSASTDSLSRINSLSQVSGV
LGCQWGDEGKGKLVDILAQHFDIVAR**CQGGANAGHTIYNSEGK**KFALHLVPSGILNEDTLCVIGNGVVV
HLPGLFKEIDGLESSGVSCTGRILVSDRAHLLFDFHQEVDGLREAELAKSFIGTTRRGIGPCYASKAIR
NGIRVSDLRHMDTFPQKLDILLSDAASRFQGFKYGPEMLREEVEKYKRYAERLEPFIADTVHVMNESIE
QKKKILVEGGQATMLDIDFGTYPFVTSSSPSAGGICTGLGIAPRAVGDLIGVVKAYTTRVGSGPFPTEI
LGQGGDLLRFAGQEFGTTTGRPRRCGWLDIVALKYCCQINGFSSLNLTKLDVLSDLPEIQLGVAYKHAD
GTPFKSFPADLR**LLEQLK**VEYEVLPGWKSDISSIRNYADFPKAAQQYVER**IEELVGVPVHYIGVGPGR**D
ALIYK

Protein No.: I-871

Protein name and Species:

60S ribosomal protein L4, putative OS=Ricinus communis GN=RCOM_0819810 PE=4 SV=1

Accession: **tr|B9SGS2|B9SGS2_RICCO**

Unused ProtScore: **2.63**

Seq Cov %: **11**

ITRAQ Changed Ratio:

117: 115=E3TL: D3TL=2. 68

116: 114=E5TL: D5TL=0. 54

Annotated typical Precursor MS Region:

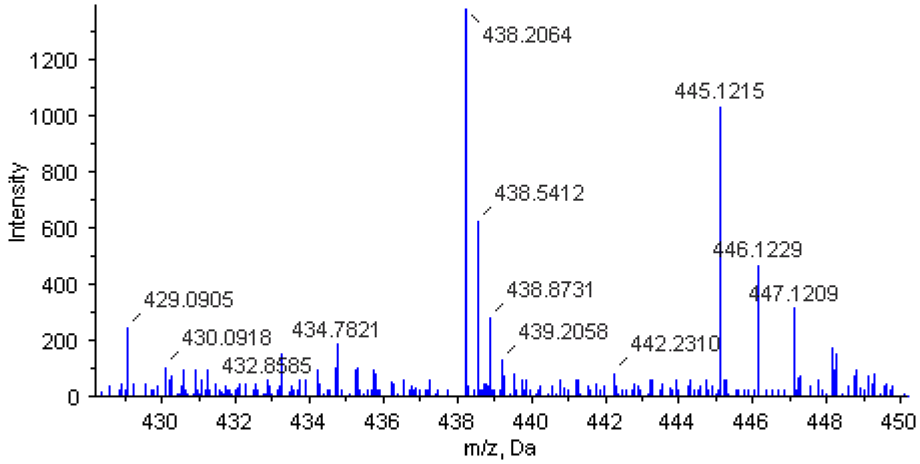

Sequence Coverage and Matched peptides: in different color

MAAAAAAARPLVTVQSLPSQNDMATDAVTTVPLPDSMKASLRPDIVSFVHAQMSKNSRQPYAVSKRAGH
QTSAESWGTGRAVSRIPRVPGGGTHR**AGQGAFGNMCR**GGRMFAPTKIWRRWHRKINVNQKRYALVSAIA
ASAIPSLVMARGHK**VESVPEMPLVISDSAESIEK**TSTALKVLKEIGAYPDVEKAKDSHAIRPGKGKMRN
RRYISRKGPLIVYGSEGAKLVKAFRNIPGVEVANVERLNLLKLAPGGHLGRFVIWTKSAFEK**LDSIYGS**
FQKSSEKKKGYVLPRSKMVNADISRIINSDEVQSVVNPIKKEVNRAPLKKNPLK**NLNVMLK**LNPYAKAA
RRMSLLAEAQRVKAKKEKLDKKRKTVTKEEAAAIRAAGKAWYQTM**ISDSDYTEFENFSK**WLNVSQ

Protein No.: I-879

Protein name and Species:

Phosphofructokinase;pyrophosphate-fructose 6-phosphate 1-phosphotransferase OS=Populus trichocarpa GN=POPTRDRAFT_761666 PE=4 SV=1

Accession: tr|B9H5X2|B9H5X2_POPTR

Unused ProtScore: 2.61

Seq Cov %: 30.2

ITRAQ Changed Ratio:

117: 115=E3TL: D3TL=0. 30

116: 114=E5TL: D5TL=1.01

Annotated typical Precursor MS Region:

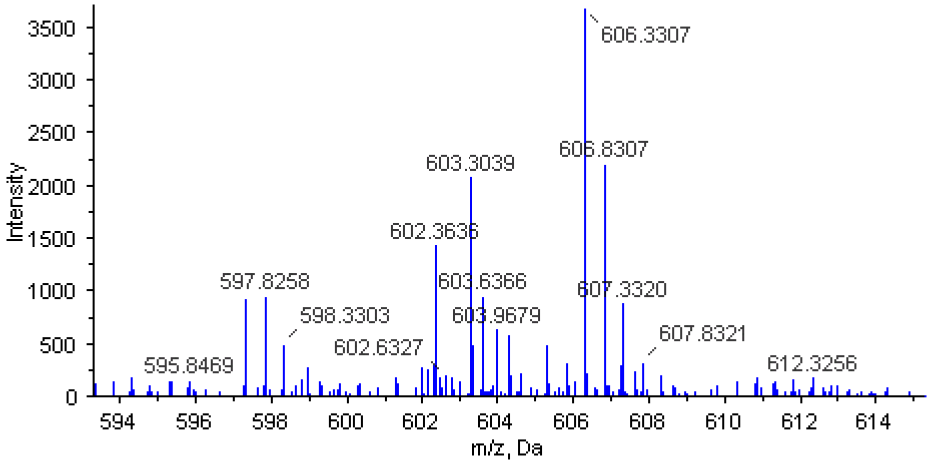

Sequence Coverage and Matched peptides: in different color

MDSDFGIPRELSDLQKLR**SLYQPELPPCLQGTAVR**VELGDATTAADPADVHSISRSFPL**TYGQPLAHFL**
RASAKVSDAQIITDHPAFR**VGIVFCGR**QSPGGHNVIWGLHNALKIHNPN**STLLGFLGGSEGLFAQKTLE**
ITDDILSTYKNQGGYDLLGRTKDQIR**TTEQVNAALTACK**DLK**LDGLVIIGGVTSNTDAAQLAETFAEAK**
CPTK**VVGVPVTLNGDLKNQFVETNVGFDTICKVNSQLISNVCTDALSAEK**YYYFIRLMGRK**ASHVALEC**
TLQSHPNMVILGEEVAASKLTLFDLTTQICDAVQARAVQDKNHGVILLPEGLIESIPEVYALLK**EIHGL**
LRHGVAPDNISSQLSPWTSALFEFLPPYIKKQLLLHPESDDSAQLSQIETEKLLAHLVETEMNKRLKEG
TYTGKK**FNAICHFFGYQAR**GSLPSKFDCDYAYVLGHISYHILAAGLNGYMATVTNLKNPVNKWR**CGAAP**
ITAMMTVKRWAQSPGASSIGK**PAIHPATVDLK**GKAYELLRQNAGRFWMDDLYRNPGPLQFDGPGADSKA
VTLCVEDQDYMGGIKKLQEYLDKVRNIVKPGCSQEVLKAALSVMSSVTDVLSVMSSTSSNGQTPL

Protein No.: I-885

Protein name and Species:

Thioredoxin OS=Jatropha curcas PE=3 SV=1

Accession: tr|E9JFW4|E9JFW4_9ROSI

Unused ProtScore: 2.61

Seq Cov %: 15.3

ITRAQ Changed Ratio:

117: 115=E3TL: D3TL=1. 77

116: 114=E5TL: D5TL=0. 79

Annotated typical Precursor MS Region:

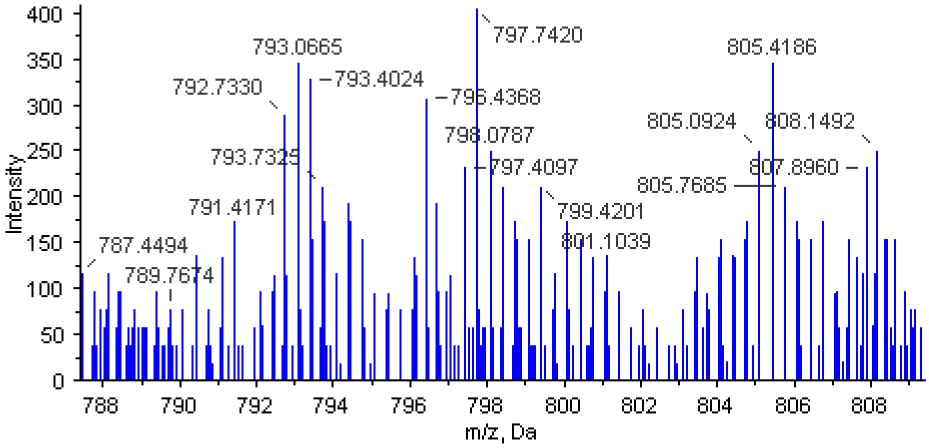

Sequence Coverage and Matched peptides: in different color

MAEEGQVIACHTVEAWNEQLEKGKESKTLIVVDFTATWCGPCRFITPILQDLAKK**MPHVTFLK**VDVDEL
R**TVAEDWAVEAMPTFMFLK**EGKIVDKVVGAKKEELQMTIVKHATEVAAA

Protein No.: I-886

Protein name and Species:

Nucleoside diphosphate kinase OS=Vitis vinifera GN=VIT_02s0025g01500 PE=3 SV=1

Accession: **tr|A5BVN4|A5BVN4_VITVI**

Unused ProtScore: **2.6**

Seq Cov %: **8.9**

ITRAQ Changed Ratio:

117: 115=E3TL: D3TL=0. 64

116: 114=E5TL: D5TL=0. 97

Annotated typical Precursor MS Region:

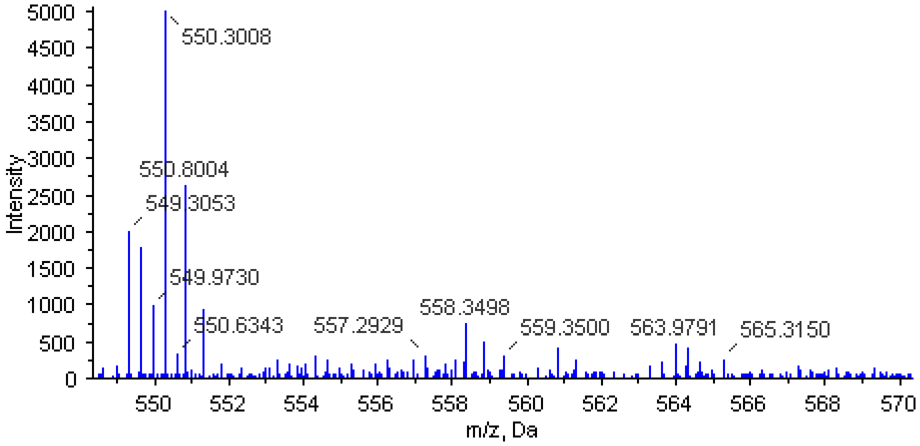

Sequence Coverage and Matched peptides: in different color

MSSQICRSASRAARSLLSASKSSNLLAEGRAVAAVAALSSRGKPFLSSFGNAGSGNAYRGWLSSVLALP
AAAYMMQEQELHATEMER**TFIAIKPDGVQRGLIAEILSR**FERKGFKLVAIKIVVPSKDFAQKHYHDLKE
RPFFNGLCDFLSSGPVVAMVWEGEGVIKYGRKLIGATDPQKSEPGTIRGDLAVVVGRNIIHGSDGPETA
KDEINLWFKPEELVNYSSNAEKWIYGVN

Protein No.: I-888

Protein name and Species:

Coated vesicle membrane protein, putative OS=Ricinus communis GN=RCOM_0911000 PE=3 SV=1

Accession: **tr|B9RTJ5|B9RTJ5_RICCO**

Unused ProtScore: 2.59

Seq Cov %: 10

ITRAQ Changed Ratio:

117: 115=E3TL: D3TL=7. 31

116: 114=E5TL: D5TL=2. 19

Annotated typical Precursor MS Region:

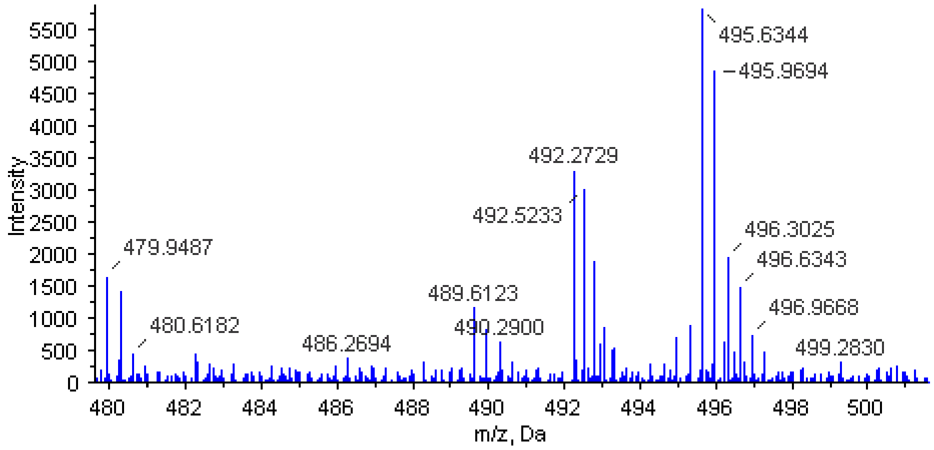

Sequence Coverage and Matched peptides: in different color

MTRRQIYVVGILLLMNLIGRISSLSVTVNDVECVYEYVLYEGDTVSGNFVVVDHDIFWSSDHPGIDLTV
TSPADNVVHTVKGTSGDKFEFKAPRSGMYKFCFHNPYSTPETVSFYIHVGHIPNAHDLAK**DEHLNPINV**
KIAELR**EALESVTAEQK**YLKARDARHRRTNESTRKRVIGYTVGEYILLGLVSTLQVVYIRRLFSKSVAY
NRV

Protein No.: I-900

Protein name and Species:

Glutathione transferase GSTL3 [Populus trichocarpa] OS=Populus trichocarpa GN=POPTRDRAFT_281791 PE=3 SV=1

Accession: tr|B9MT66|B9MT66_POPTR

Unused ProtScore: 2.53

Seq Cov %: 15

ITRAQ Changed Ratio:

117: 115=E3TL: D3TL=0. 47

116: 114=E5TL: D5TL=0. 24

Annotated typical Precursor MS Region:

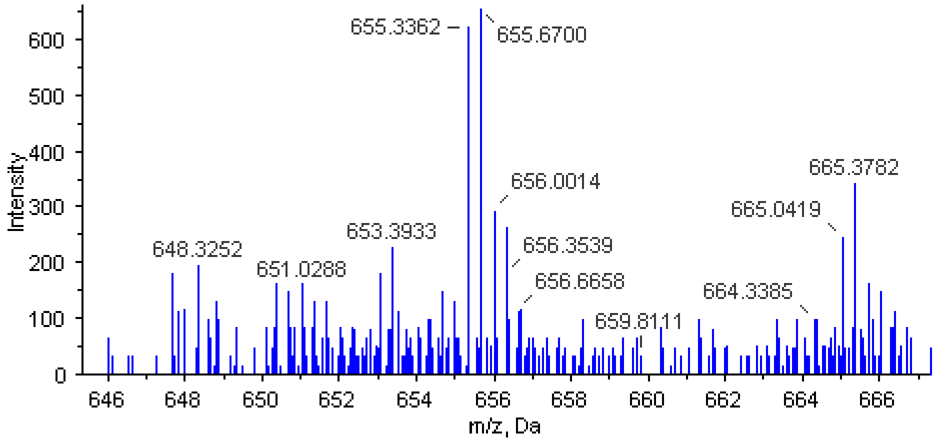

Sequence Coverage and Matched peptides: in different color

PLDATAEQPPLFDGTTKLYTCYTCPFAQRVWITRNFKGLQDEIKLVPLILQNRPAWYPEKVYPPNKVPS
LEHNGKITGESLDLIKYLESNFEGPSLLPKDPAKKEFAEELFSYTDKFNGTVYTAFKGDLAKEAGPAFD
YLENALHKFDDGPFFLGKEFSLVDIAYIPFVERLNIFLLEVFKYDIAAGRQKLAAWIEEVNKIEAYKQT
KTDPKELVEFYKK

Protein No.: I-903

Protein name and Species:

177 protein OS=Arabidopsis thaliana GN=LSH10 PE=2 SV=1

Accession: tr|Q9S7R3|Q9S7R3_ARATH

Unused ProtScore: 2.53

Seq Cov %: 11.9

ITRAQ Changed Ratio:

117: 115=E3TL: D3TL=0. 46

116: 114=E5TL: D5TL=0. 22

Annotated typical Precursor MS Region:

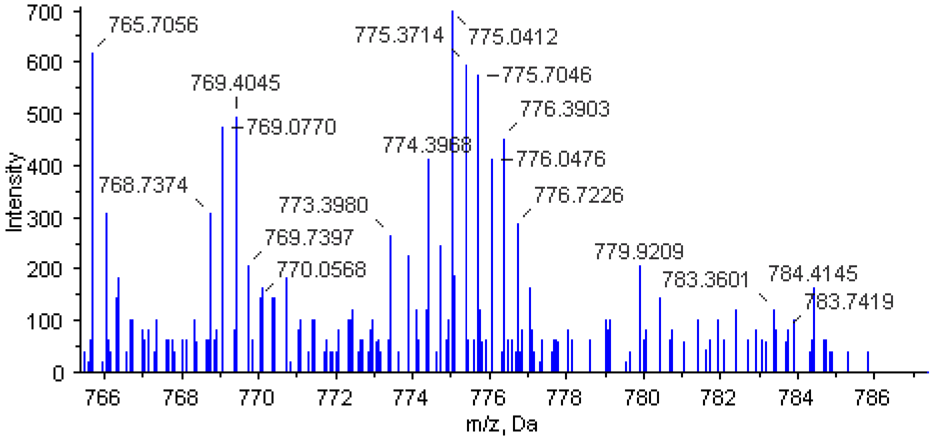

Sequence Coverage and Matched peptides: in different color

MSSPRERGKSLMESSGSEPPVTPSRYESQKRRDWNTFGQYLKNQRPPVPMSHCSCNHVLDFLRYLDQFG
KTKVHVPGCMFYGQPEPPAPCTCPLRQAWGSLDALIGRLRAAYEENGGPPETNPFASGAIRVYLREVRE
CQAKARGIPYKKKKKKKPTPEMGGGREDSSSSSSSFSFS

Protein No.: I-904

Protein name and Species:

Protein disulfide isomerase OS=Gossypium hirsutum GN=GHPDI12 PE=2 SV=1

Accession: tr|G1ED19|G1ED19_GOSHI

Unused ProtScore: 2.53

Seq Cov %: 7.2

ITRAQ Changed Ratio:

117: 115=E3TL: D3TL=2. 36

116: 114=E5TL: D5TL=1. 50

Annotated typical Precursor MS Region:

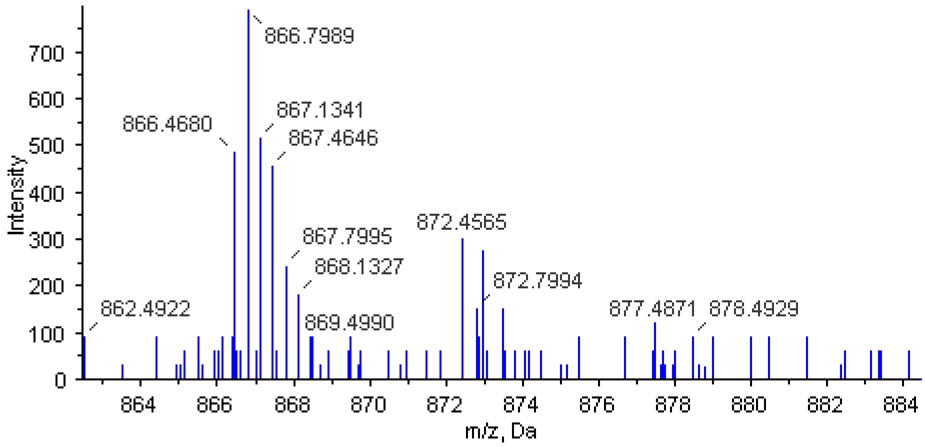

Sequence Coverage and Matched peptides: in different color

MKSQICLAFGTLALLLASALADDVVVLTEANFDKEVGQDRGVLIEFYAPWCGHCKKLAPEYEKLGATFK
KAKSVLIGKVDCDEHKSLCSKYGVQGYPTVQWFPKGSLEPKKYEGTSTAEAPVEFVNTEGGTNVKIATL
PSNVAVLNADNFDEIVLDETKDVLVEFYAPWCGHCKNLAPTYEKVATAFK**SEEDVVIANLDADK**YRDLA
EKYGISGFPTLKFFPKGNKAGEDYDGGR**DLDDFVSFINEK**CGTSRDAKGQLTSTAGILSSLDALVKEFV
AASADEKKVVFSK**IEEEVEK**LKGSTARHGKIYLKAAKSCLEKGADYPKKEIERLQRMLDKSISPAKADE
FTLKKNILSTFASS

Protein No.: I-905

Protein name and Species:

Glutathione S-transferase,2,4-D inducible OS=Glycine max GN=GSTa PE=1 SV=1

Accession: tr|O49235|O49235_SOYBN

Unused ProtScore: 2.52

Seq Cov %: 9.1

ITRAQ Changed Ratio:

117: 115=E3TL: D3TL=0. 35

116: 114=E5TL: D5TL=0. 16

Annotated typical Precursor MS Region:

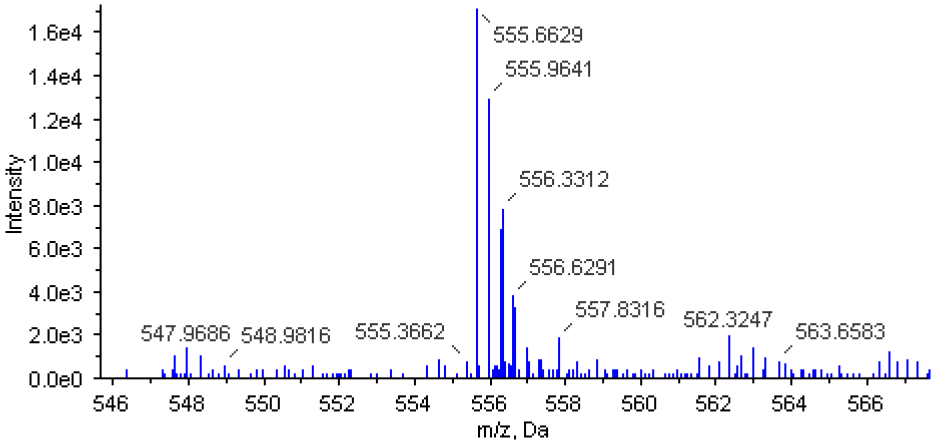

Sequence Coverage and Matched peptides: in different color

MSDEVVLLDFWPSPFGMRVR**IALAEK**GIK**YEYKEEDLR**NK**SPLLLQMNPVHK**K**IPVLIHNGK**PICESLI
AVQYIEEVWNDRNPLLPSDPYQRAQTRFWADYVDKK**IYDLGR**KIWTSKGEEK**EAAK****KEFIEALK**LLEEQ
LGDKTYFGGDNLGFVDIALVPFYTWFKAYETFGTLNIESECPKFIAWAKRCLQKESVAKSLPDQQKVYE
F**IMDLR**KKLGIE

Protein No.: I-907

Protein name and Species:

Cysteine synthase OS=Medicago truncatula PE=2 SV=1

Accession: tr|B7FKU7|B7FKU7_MEDTR

Unused ProtScore: 2.5

Seq Cov %: 19.1

ITRAQ Changed Ratio:

117: 115=E3TL: D3TL=0. 59

116: 114=E5TL: D5TL=1. 07

Annotated typical Precursor MS Region:

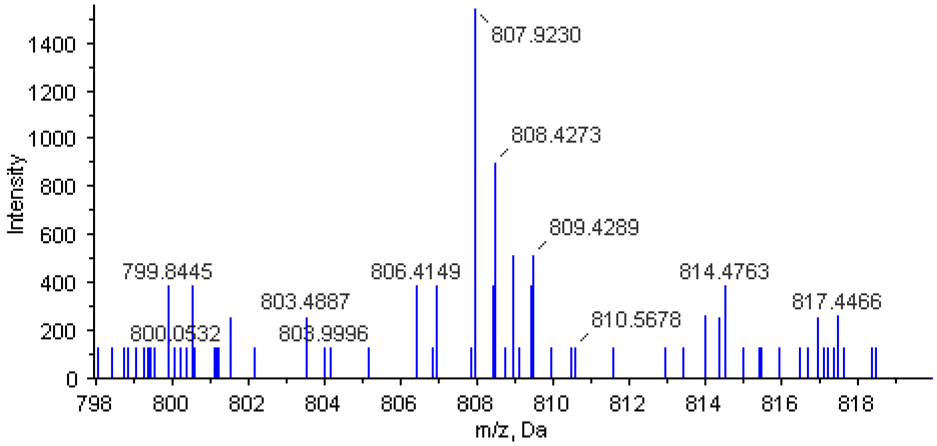

Sequence Coverage and Matched peptides: in different color

MAVERSGIAKDVTELIGKTPLVYLNRLADGCVARVAAKLELMEPCSSVKDRIGYSMIADAEEKGLITPG
QSVLIEPTSGNTGIGLAFMAAAKGYKLIITMPASMSLERRIILLAFGAELVLTDPAKGMKGAVQKAEEL
LAKTPNAYILQQFENPANPKVHYETTGPEIWKGTDGKIDAFVSGIGTGGTITGAGKYLKEQNSNIKLIG
VEPVESPVLSGGKPGPHKIQGIGAGFVPGVLEVNLIDEVVQISSDEAIETAKLLALKEGLFVGISSGAA
AAAAIKIAKRPENAGKLIVVVFPSFGERYLSSVLFESVRREAETMTFEP

Protein No.: I-914

Protein name and Species:

trehalose-phosphate synthase [UDP-forming] 9 [Arabidopsis thaliana] OS=Arabidopsis lyrata subsp. lyrata GN=ARALYDRAFT_889930 PE=4 SV=1

Accession: **tr|D7KNJ9|D7KNJ9_ARALL**

Unused ProtScore: 2.48

Seq Cov %: 3.5

ITRAQ Changed Ratio:

117: 115=E3TL: D3TL=1. 89

116: 114=E5TL: D5TL=1. 96

Annotated typical Precursor MS Region:

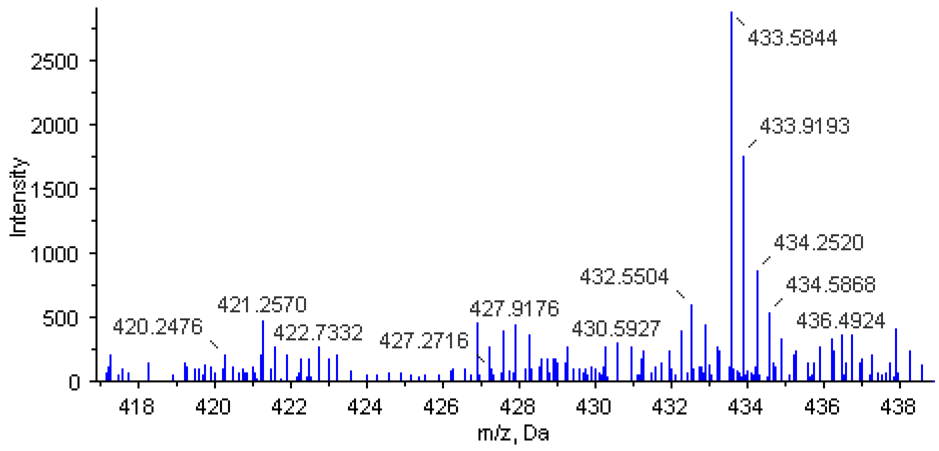

Sequence Coverage and Matched peptides: in different color

MVSRSCANFIDLASWDLLDFPQTQRALPRVMTVPGIISELDGGYSDGSSDVNSSSSSRERKIIVANMLP
LQAKRDTESGQWCFSWDEDSLLLQLRDGFSSDTEFVYIGSLNADIGTSEQEEVSHKLLLDFNCVPTFLP
KEMQEKFYLGFCKHHLWPLFHYMLPMFPDHGDRFDRRLWQAYVSANKIFSDRVMEVINPEEDYVWIHDY
HLMVLPTFLRKRFNRIKLGFFLHSPFPSSEIYRTLPVRDDLLRGLLNCDLIGFHTFDYARHFLSCCSRM
LGLDYESKR**GHIGLDYFGR**TVFIKILPVGIHMGRLESVLNLPSTAAKMKEIQEQFKGKKLILGVDDMDI
FKGISLKLIAMERLFETYWHMRGKLVLIQIVNPARATGKDVEEAKRETYSTVKRINERYGSAGYQPVIL
IDRLVPRYEKTAYYAMADCCLVNAVRDGMNLVPYKYIICRQGTPGMDKAMGISHDSPRT**SMLVVSEFIG**
CSPSLSGAIRVNPWDVDAVSEAVNLALTMGETEKRLRHEKHYHYVSTHDVGYWAKSFMQDLERACREHY
NKRCWGIGFGLSFRVLSLSPSFRKLSIDHIVSTYRTTQRRAIFLDYDGTLVPESSIIKTPNAEVLSVLK
SLCGDPKNTVFVVSGRGWESLSDWLSPCENLGIAAEHGYFIRWSSKREWETCYSSAEAEWKTMVEPVMR
SYMDATDGSTIEFKESALVWHHQEADPDFGACQAKELLDHLESVLANEPVVVKRGQHIVEVKPQGVSKG
LAVEKVIHRMVEDGNPPDMVMCIGDDRSDEDMFESILSTVTNPDLPMPPEIFACTVGRKPSKAKYFLDD
VSDVLKLLGGLAAASSSRKPEDQQQSSSLHTQVAFESII

Protein No.: I-921

Protein name and Species:

Adaptin ear-binding coat-associated protein, putative OS=Ricinus communis GN=RCOM_0553580 PE=4 SV=1

Accession: tr|B9SGK0|B9SGK0_RICCO

Unused ProtScore: 2.45

Seq Cov %: 6.2

ITRAQ Changed Ratio:

117: 115=E3TL: D3TL=0. 27

116: 114=E5TL: D5TL=0. 99

Annotated typical Precursor MS Region:

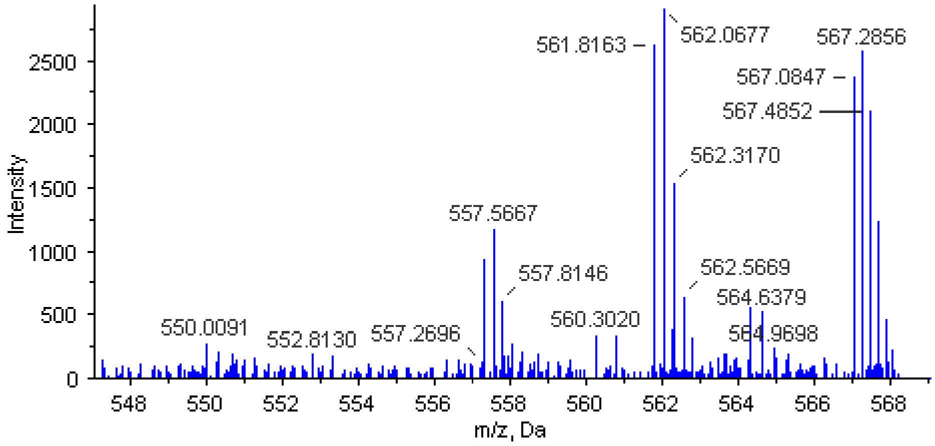

Sequence Coverage and Matched peptides: in different color

MLRKHLGPVHSPISSQPPPVSDDSIILPQPEAVLDRRVIQKGKYRPKSDVLIKWKGAPREDATWENEWR
FTKSYLDYLNPFHAYDEGNLSWMAAAEAEPATMSMVRRAIPNASSLEEGNRVLRGNWLKTIEQHHQQYS
GKSIISDILDVGCSVGVSTRFLADKFPLAKVTGLDLSPHFLSVAQFKEKKGAPRKNPINWIHANGEDTG
LPSKSYDLVTIAFVVHECPERAINGLLREAYRLLRPGGTIALTDSSPKSKILQIMEIEVKDLNDQEKET
EETEAIELVLFHVSECYVYLIPPRKSAASCRADEWNVNKWAWEGMLKVISKGEECIIKLEDKTTGELYA
RAFLR**DGEPHPVEPVIDSSR**YFVLR**IEENIGGR**LRHAFIGIGFRERTEAYDFQAALHDHMKYLNKKK**TA**
EEMEQHFQKTSTVDYSLKEGETLVLQIKNKSGSGMKSKILDQGLNNLSLEEKGEQKELILGIKLPPPPP
APVSPVTSPRFPSDLPPKISLDGTSIDNSPRLRKEEAKEQHSPDNESTQDIPDDDFGDFQAAG

Protein No.: I-929

Protein name and Species:

Putative uncharacterized protein OS=Ricinus communis GN=RCOM_0186330 PE=4 SV=1

Accession: **tr|B9T8C2|B9T8C2_RICCO**

Unused ProtScore: **2.41**

Seq Cov %: **6.6**

ITRAQ Changed Ratio:

117: 115=E3TL: D3TL=0. 61

116: 114=E5TL: D5TL=0. 29

Annotated typical Precursor MS Region:

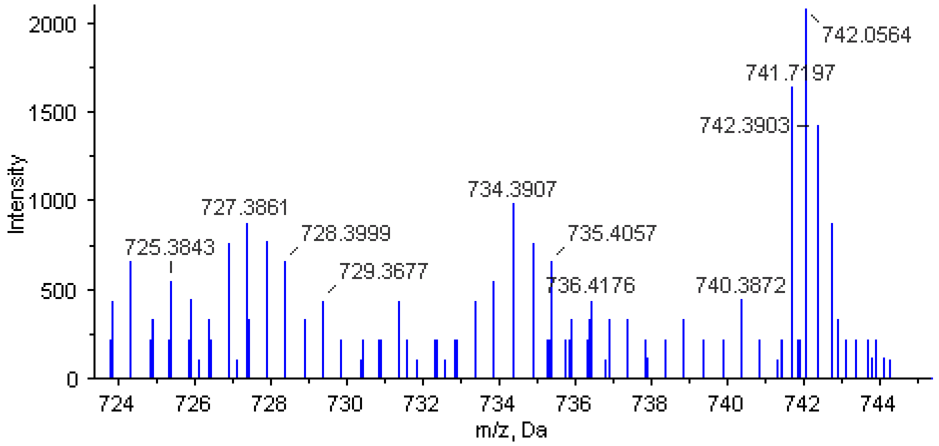

Sequence Coverage and Matched peptides: in different color

MSMLDSFFNKGFKATKCKTLLKLTIPRIKLLRNRREIQIKQMRRDIAKLLETGQEATARIRVEHIIREE
NMMAAQEILELFCELISVRLPIIEAQRECPLDLK**EAISSICFAAPR**CADLQELLQVQMLFVSKYGREFV
AAATELMPECGVNRQLIELLSVRAPSPDVKLKLLKEIAEEHELDWDPAASETELLKKHEDLLNGPTQFV
SGSKLPLPEEKHEEALNSVPDQAHNEQPDSDSDFETLDFPEVPKVALRPSANTASAPEMLPIPPAAQRG
IEHEPSNLSANYENLAQECHLENEDLTEEEPVATKDGTANIVDAKEEKQFLPFISPPVSSTSFSTRPST
SPPISRTK**SEFNVDLQDVIAAAQAAAETAER**AAAAARLAATLAQARISVLTKKNSEKFPECSDENPFHV
DTPDQSPTTEKPHFGHQHSFDGPSGVSSYLHLHQQHEDHQPSELHDLPSFEKLKMEYDSPPSDLVHEQQ
SVRHQPQRLPSMDDDAYFSYPNLFTSQNPNLGSSAQSGTGNSHSAHDV

Protein No.: I-938

Protein name and Species:

Glutathione-s-transferase theta (Fragment) OS=Jatropha curcas PE=2 SV=1

Accession: tr|D6BRD9|D6BRD9_9ROSI

Unused ProtScore: 2.37

Seq Cov %: 15.9

ITRAQ Changed Ratio:

117: 115=E3TL: D3TL=2. 27

116: 114=E5TL: D5TL=2. 11

Annotated typical Precursor MS Region:

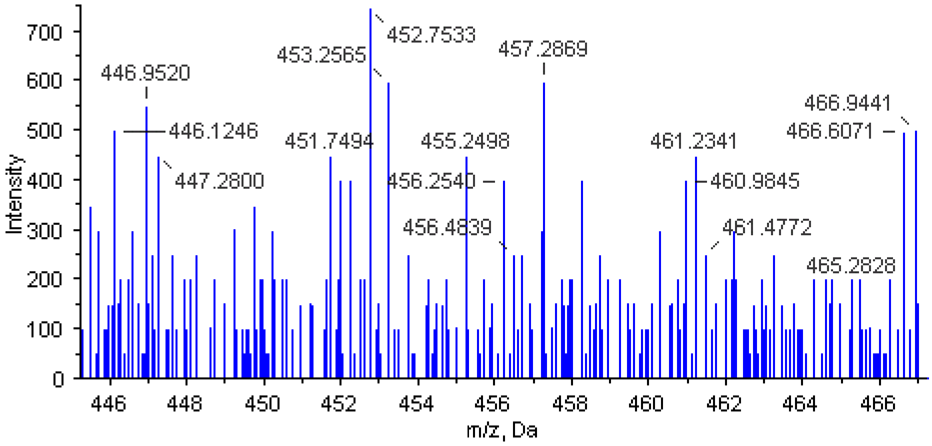

Sequence Coverage and Matched peptides: in different color

MAVIKVHGNPISTATQRVLATLYEKDLEFEFVHIDLATGEHKR**EPFISLNPFGQVPALEHGDLK**LFESR
AITQYITHENPDKGTQLLCPGKVKMPILTVWMEVEAQKFEPAASKLNWEVFLKPIFGLTTDSAAVEANE
AELSKVLDVYEARLAQSKYLACDCFTLVDLHHLPNLQLLLLTQSKKLIESRPHVSAWAADITARPAWAK
VLAMQRV

Protein No.: I-944

Protein name and Species:

Fructose-bisphosphate aldolase OS=Arabidopsis lyrata subsp. lyrata GN=ARALYDRAFT_482625 PE=3 SV=1

Accession: tr|D7LJ40|D7LJ40_ARALL

Unused ProtScore: 2.35

Seq Cov %: 28.2

ITRAQ Changed Ratio:

117: 115=E3TL: D3TL=0. 52

116: 114=E5TL: D5TL=0. 22

Annotated typical Precursor MS Region:

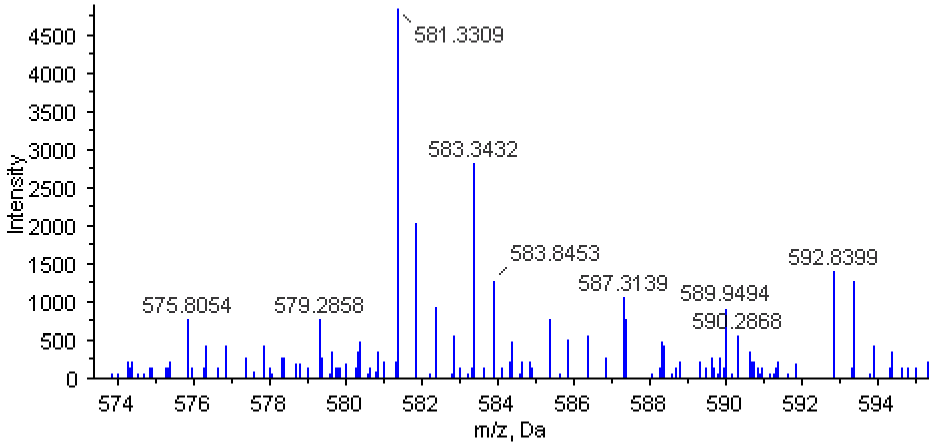

Sequence Coverage and Matched peptides: in different color

MSCFTTK**FADELIANAAYIGTPGKGILAADESTGTIGKRLASINVENVESNRR**ALRELLFTTPGALPCL
SGVILFEETLYQKSSNGTPFVDMLKSAGVLPGIKVDKGTVELAGTNAE**TTTQGLDGLGDR**CKK**YYEAGA**
RFAKWRAVLKIGQNEPSELAIHENAYGLARYAVICQENGLVPIVEPEILVDGSHDIHKCAAVTER**VLAA**
CYKALSDHHVLLEGTLLKPNMVTPGSESA**KVAPEVIAEHTVR**ALQR**TVPAAVPAIVFLSGGQSEEEATR**
NLNAMNKLKTKKPW**SLSFSFGRALQQSTLK**TWGGKEENVK**KAQEAFLVR**CK**ANSEATLGTYK**GDAKLGE
GAAESLHVKDYKY

Protein No.: I-945

Protein name and Species:

coatomer subunit delta OS=Populus trichocarpa GN=POPTRDRAFT_824854 PE=4 SV=1

Accession: tr|B9ICR4|B9ICR4_POPTR

Unused ProtScore: 2.35

Seq Cov %: 5.9

ITRAQ Changed Ratio:

117: 115=E3TL: D3TL=0. 20

116: 114=E5TL: D5TL=0. 47

Annotated typical Precursor MS Region:

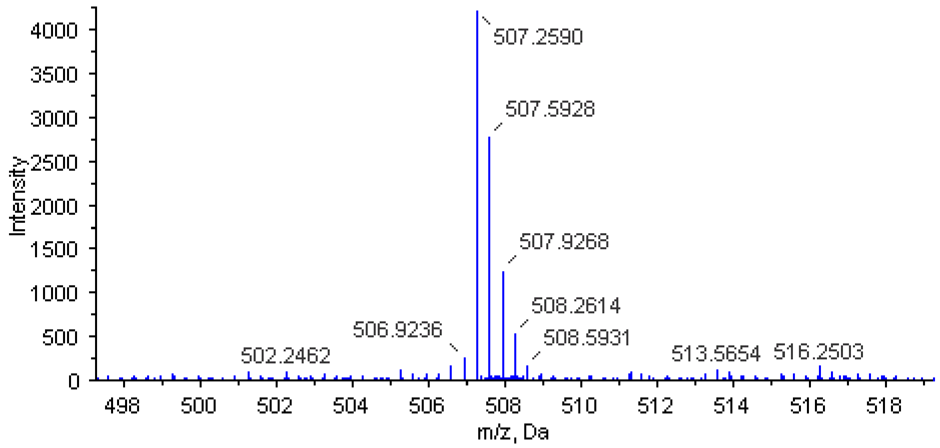

Sequence Coverage and Matched peptides: in different color

MVVLAASIVSKNGKVLVSRQFVDMSRIR**IEGLLAAFPK**LIGSGK**QHTYVETENVR**YVYQPIEAMYLLLV
TNKQSNILEDLDTLRLLSKLVPEYTMSLDEEGICQTAFELIFAFDEVISLGHK**ENVTVAQVKQYCEMES**
HEEKLHKLVLQNKIDETKRRMKEEASKIDQMKIEKNRGNKGGFMSSMGSGRIESSFNDMSISSGGGGGF
GSGSGFGLTSDVDSFSSKPKGRQPSSATAPPKGLGMKLGKTQR**TNQFLESLK**AEGEMIVEDVQPSKSTQ
YTSAAQKLTDPVTLTAEEKLNVTLKRDGGMSHFDVQGHLSLQILNQEDGLIQVQIETGGNPGVIFKTHP
NVNKELFANENILGLRDPSRPFPACQTGDAGVGLLKWRMQSVDESMVPLTINCWPSESGNETYVSIEYE
ASSMFDLRNVVIAVPLPALREAPNVRQIDGEWRYDARNSILEWSILLIDNSNRSGSMEFVVPSGDSSAF
FPITVQ**FSATSTYSELK**VANILPLKGGAPPKFSQRTQLITENYQVV

Protein No.: I-948

Protein name and Species:

Adenylyl cyclase-associated protein OS=Populus trichocarpa GN=POPTRDRAFT_556459 PE=3 SV=1

Accession: tr|B9H0Q2|B9H0Q2_POPTR

Unused ProtScore: 2.34

Seq Cov %: 8.2

ITRAQ Changed Ratio:

117: 115=E3TL: D3TL=0. 40

116: 114=E5TL: D5TL=0. 54

Annotated typical Precursor MS Region:

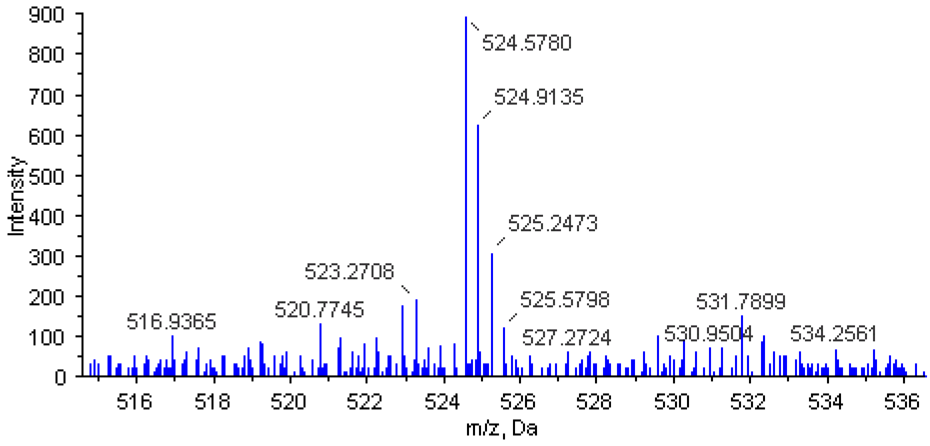

Sequence Coverage and Matched peptides: in different color

MEERLMARLESAVARLEALSLRGGSVAGSGGDDASATDPSIVAFEDFMGAYLGRVSSAGEKIGGQVLAV
TKILEQALNMQKELLIKIKQTQKPDLAGLAEFLKPLNEVIMKANSMTEGRRSDFFNHLKAAADSSTALA
WIAYTGKDCGMSMPIAHVEESWQMAEFYNNK**ILVEYK**SKDPNHVEWAKALK**ELYLPGLR**DYVKSHYPLG
PVWSATAKAKAAAPSAPAPPPPPPASLFTSESSSPSSSKPKVGMAAVFQEISSGKPVTSGLRKVTDNMK
TKNRADRTGVVGASEKESRTSSPSFSKAGPPK**LELQMGR**KWVVENQIGRKNLVIDDCDAK**QSVYIFGCK**
DSVLQVQGKVNNITIDKCTKMGVVFTDVVAACEIVNCSGVEVQCQGSAPTISVDNTGGCQLYLSKGSLG
ASITTAKSSEINILVPGAEPDGDLVEHALPQQFIHTFK**DGQFETTPVSHSGG**

Protein No.: I-949

Protein name and Species:

Annexin-like protein RJ4 OS=Fragaria ananassa PE=2 SV=2

Accession: sp|P51074|ANX4_FRAAN

Unused ProtScore: 2.34

Seq Cov %: 6.7

ITRAQ Changed Ratio:

117: 115=E3TL: D3TL=0. 09

116: 114=E5TL: D5TL=0. 37

Annotated typical Precursor MS Region:

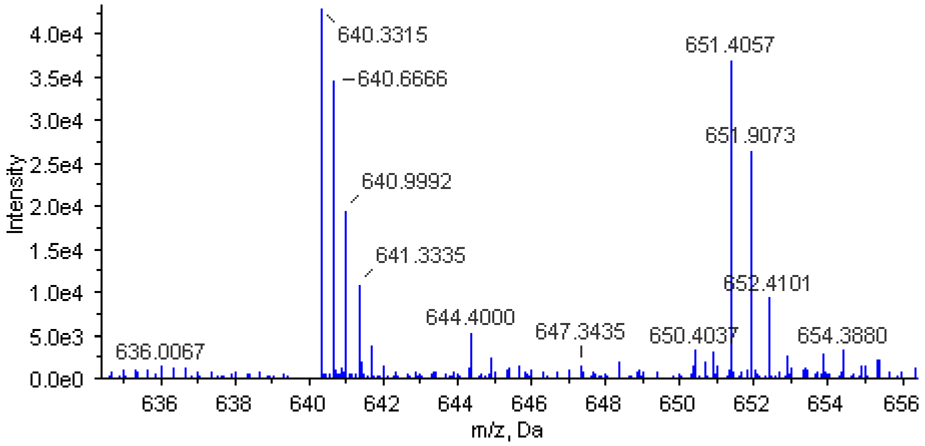

Sequence Coverage and Matched peptides: in different color

MATLVSPPNFCAKEDAEALRKSVKGWGTNEKAIISILGHRNAGQRKEIRAAYEQLYQEDLLKPLESELS
GDFEKAVYRWTLDPADRDAVLANVAIKKSTDVYNVIIEISCIHSPEELLAVRRAYQLRYKHSVEEDLAA
HTTGDIRKLLVALVTAYRYDGHEINAKLANSEADILHDAIKDKAFNHEEIIRILSTRSKTQLMATFNKY
RDDQGISISKNLLEEGANDFQKALHTAIRCLNDPKKYFEKVLRNAIKRVGTDEDALTRVIVTRAERDLR
DIKEVYYKKNSVPLEQAVAKDTSGDYKAFLLTLLGKED

Protein No.: I-952

Protein name and Species:

Fructose-bisphosphate aldolase OS=Codonopsis lanceolata PE=2 SV=1

Accession: **tr|Q2WFK8|Q2WFK8_9ASTR**

Unused ProtScore: **2.33**

Seq Cov %: **23.5**

ITRAQ Changed Ratio:

117: 115=E3TL: D3TL=0. 47

116: 114=E5TL: D5TL=0. 33

Annotated typical Precursor MS Region:

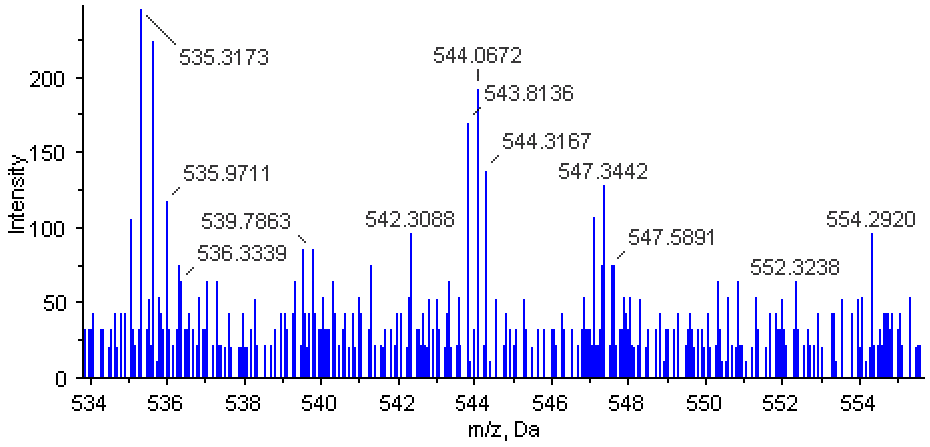

Sequence Coverage and Matched peptides: in different color

MSCYKGK**YADELIANATYIGTPGKGILAADESTGTIGKR**LSSINVENIESNRWALRELLFCTPGALQYL
SGVILFEETLYQKTAAGKPFVDVLKENGVLPGIKVDKGTVVLAGTNGETTTQGLDGLGARCAQYYAAGA
RFAKWRAVLN**IGPNEPSQLAINENANGLAR**YAIICQENGLVPIVEPEILVDGAHDIAKCADVTER**VLAA
CYKALNDHHVLLEGTLLKPN**MVTPGSDSAKVAPEVVAEYTIRALQRTMPAAVPAVVFLSGGQSEEQATV
NLNAINKYKGKKPWSLTFSFGR**ALQQSTLK**AWAGKEENVAKAQAAFLARCKANSEATLGTYEGGASLSE
GAAESLHVKDYKY

Protein No.: I-975

Protein name and Species:

dolichol-phosphate mannosyltransferase OS=Populus trichocarpa GN=POPTRDRAFT_801608 PE=4 SV=1

Accession: tr|B9H5H4|B9H5H4_POPTR

Unused ProtScore: 2.28

Seq Cov %: 14.7

ITRAQ Changed Ratio:

117: 115=E3TL: D3TL=1. 77

116: 114=E5TL: D5TL=2. 07

Annotated typical Precursor MS Region:

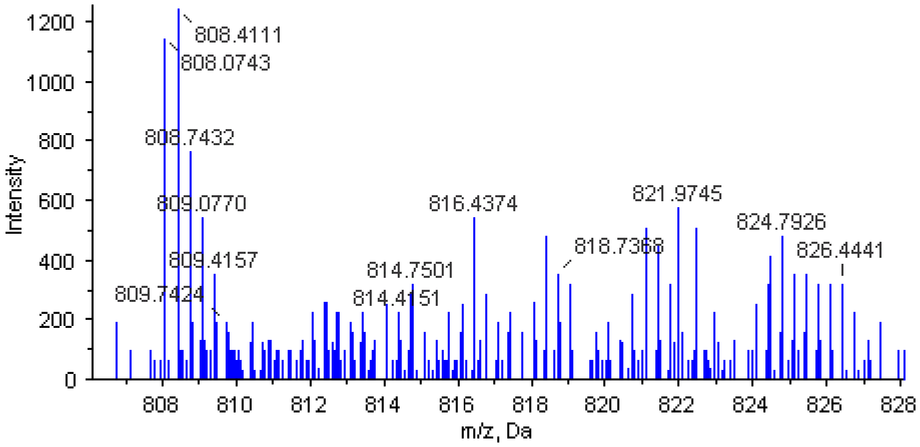

Sequence Coverage and Matched peptides: in different color

MEKNNRYSIIVPTYNERLNIALIVYLIFKHLQDVEFEIIVVDDGSPDGTQEVVKQLQKVYGEDRILLRP
RAKKLGLGTAYIHGLKHASGNFVVIMDADLSHHPK**YLPSFIK**K**QLETGASIVTGTR**YVKGGGVHGWNLM
RKLTSR**GANVLAQTLLWPGVSDLTGSFR**LYKK**SVLEDIISSVVSK**GYVFQMEMIVRASRKGYQIEEVPI
TFVDRV**FGSSK****LGGSEIVEYLK**GLAYLLVTT

Protein No.: I-981

Protein name and Species:

Predicted protein OS=Populus trichocarpa GN=POPTRDRAFT_818483 PE=4 SV=1

Accession: tr|B9H739|B9H739_POPTR

Unused ProtScore: 2.26

Seq Cov %: 9.5

ITRAQ Changed Ratio:

117: 115=E3TL: D3TL=0. 61

116: 114=E5TL: D5TL=1. 05

Annotated typical Precursor MS Region:

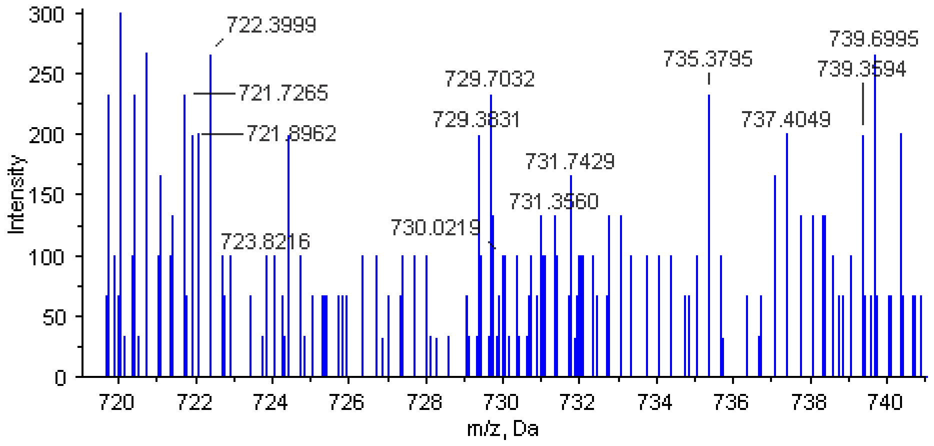

Sequence Coverage and Matched peptides: in different color

MERSTPVRKPHTSTADLLTWSETPPPDSPSVGSAPRPTRSHQPSDGISK**VVFGGQVTEEEFESLNK**RKP
CSGYKMKEMTGSGIFAANGEDDTAESGSANPIPNSKTGLRMYQQAIAGISHISFAEDDSVSPKKPTTLP
EVAKQRELSGTLESESDAKLKKQISGAKSKELSGHDIFAPPPEILPRPTTVRALALQESIQLGEPSPHD
PVGGQQPSEESVVKTAKKINNQK**FNELSGNDIFK**GDAPPSSAEKPLSVAKLREMSGNDIFADGKVESRD
FLGGVRKPPGGESSIALV

Protein No.: I-982

Protein name and Species:

Phospholipase D OS=Fragaria ananassa PE=2 SV=1

Accession: tr|Q533V0|Q533V0_FRAAN

Unused ProtScore: 2.25

Seq Cov %: 11.6

ITRAQ Changed Ratio:

117: 115=E3TL: D3TL=0. 29

116: 114=E5TL: D5TL=0. 24

Annotated typical Precursor MS Region:

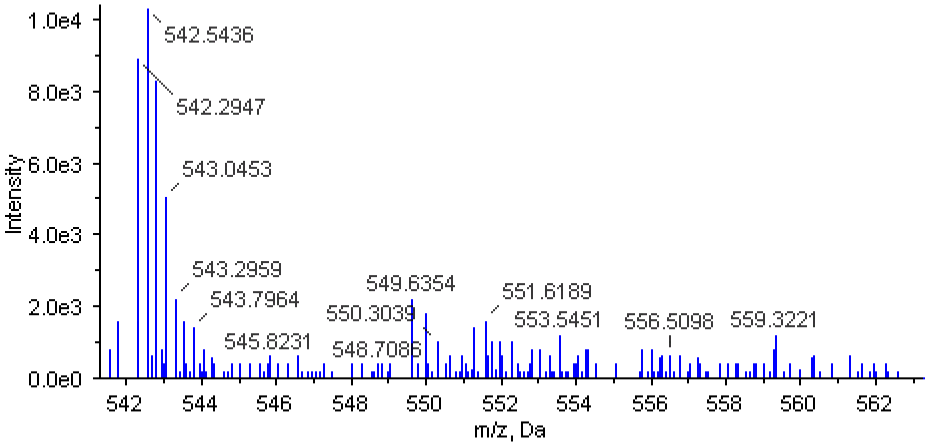

Sequence Coverage and Matched peptides: in different color

MGDSAEHWLHGTLHATIYEVDKLHGSSGNFLRKITGKLEETVGLGKGVSKLYATVDLERARVGRTRVIE
KEPSNPNWSESFHIYCAHVAANVIFTVKESNPIGASLIGRAYVPVEQLIEGEEVDTWAKILDDKKNPVH
GEPKIHVKLQFFHVSKDRSWGQGIKSPKFPGVPFTFFSQRQGCRVSLFQDAHVPDKFIPK**IPLAGGK**YY
EPHRCWEDIFDAITNAKHLIYITGWSVYTEISLIRDSRRPK**SGGDITIGELLK**KKASEGVR**VLMLVWDD**
R**TSVGLLK**KDGLMATHDEETAQFFQNTDVNCVLCPRNPDGGGSIVQGAQISTMFTHHQKIVVVDSEMPN
GGSQSRR**IVSFVGGLDLCDGRYDTPFHSLFR**TLDTAHHDDFHQPNFTGASITKGGPREPWHDIHSRLEG
PIAWDVLFNFEQRWRKQGGKDVLVQLRELDNVIIPPSPVMFPDDHETWNVQLFR**SIDGGAAFGFPDSPE**
DAARAGLVSGKDNIIDR**SIQDAYIHAIR**RAKNFIYIENQYFLGSSFAWAADGIKPEDIGASHVIPRELS
LKIADKIANGERFTVYVVVPMWPEGIPESASVQAILDWQRRTMEMMYKDIKQALDKHGVEEDPR**NYLTF**
FCLGNREVKKDGEYEPSEAPEADSDYIRAQEARRFMIYVHTK**MMIVDDEYIIIGSANINQR**SMDGARDS
EIAMGAYQPHHLSVREPARGQIHGFRMALWYEPLGMLDETFLQPESVECIK**KVNQIADK**YWDLYSSETL
EHDLPGHLLRYPVGVTSEGEVTELPGFEFFPDTKARVLGAKSDYLPPILTT

Protein No.: I-990

Protein name and Species:

Malic enzyme OS=Populus trichocarpa GN=POPTRDRAFT_737670 PE=3 SV=1

Accession: tr|B9ILT5|B9ILT5_POPTR

Unused ProtScore: 2.23

Seq Cov %: 7.3

ITRAQ Changed Ratio:

117: 115=E3TL: D3TL=0.59

116: 114=E5TL: D5TL=1.25

Annotated typical Precursor MS Region:

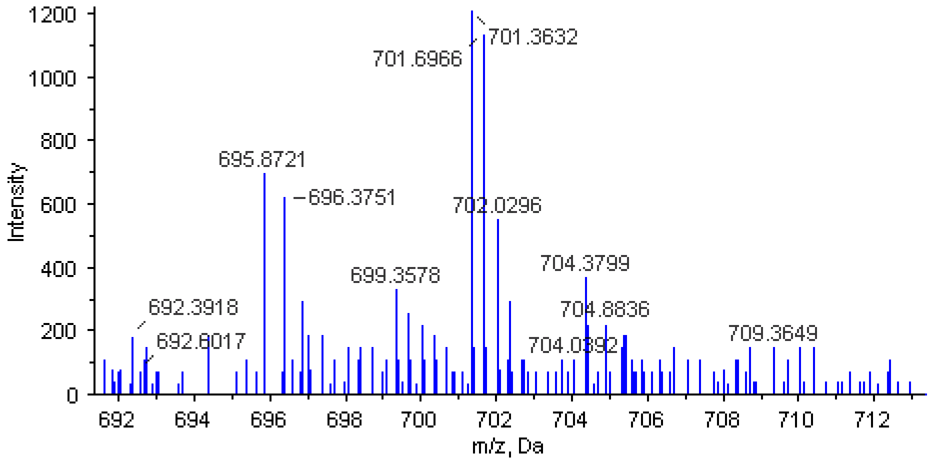

Sequence Coverage and Matched peptides: in different color

MESTLKEMRDGASVLDMDPKSTVGGGVEDVYGEDRATEDQLVTPWTISVASGFTLLRDPQHNKGLAFTE
KERDAHYLRGLLPPATISQQLQEKKLMNTIRQYQLPLQKYTAMMELEERNERLFYKLLIDNVEELLPVV
YTPTVGEACQK**YGSIFK**RPQGLYISLKEKGKVLDVLKNWPQKSIQVIVVTDGER**ILGLGDLGCQGMGIP**
VGKLSLYTALGGVRPSACLPVTIDVGTNNEQLLKDEF**YIGLR**QKRATGQEYSELLHEFMTAVKQNYGEK
VLIQFEDFANHNAFDLLAKYGTTHLVFNDDIQGTAAVVLAGLISALKLLGGSLADHTFLFLGAGEAGTG
IAELIALEMSRRSKTPLEETRKKIWLTDSKGLIVSSRKESLQHFKKPWAHEHEPVKG**LLEVVK**AIKPTV
LIGTSGVGKTFTKEVIEAMASFNEKPLILALSNPTSQSECTAEEAYTWTKGK**AIFASGSPFDPVEYEGK**
VFVPGQSNNAYIFPGLGLGLVISGAIRVHDDMLLAAAEALAGQIKEEYLAKGLIYPPLSNIRKISVQ**IA**
ANVAAK**AYELGLATR**LPRPENLVKHAESCMYSPAYRYYR

Protein No.: I-993

Protein name and Species:

Signal recognition particle 68 kDa protein, putative OS=Ricinus communis GN=RCOM_1503780 PE=4 SV=1

Accession: tr|B9RA70|B9RA70_RICCO

Unused ProtScore: 2.23

Seq Cov %: 6.9

ITRAQ Changed Ratio:

117: 115=E3TL: D3TL=2. 91

116: 114=E5TL: D5TL=0.86

Annotated typical Precursor MS Region:

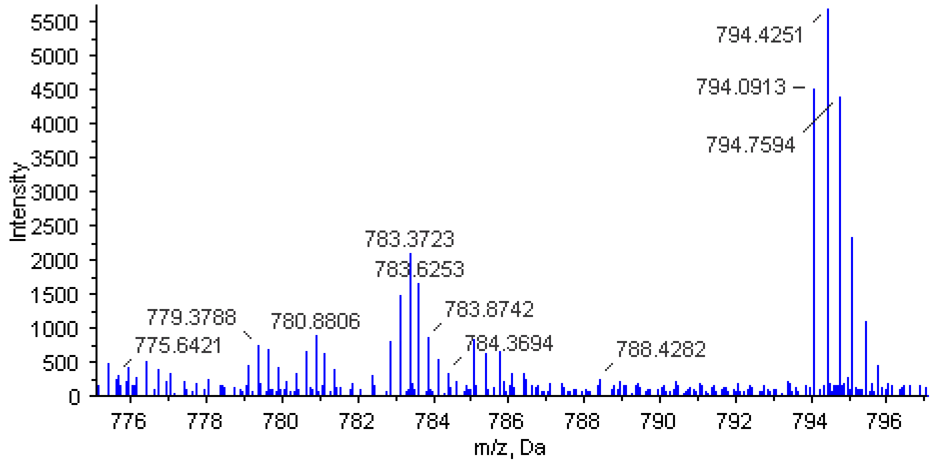

Sequence Coverage and Matched peptides: in different color

MAMDIDGPKSNNSEQISPRYSINVLQLLKSAQMQHGLRHGDYTRYRRYCTARLRRLYKSLKFTHGRGKY
TRRAITESTVTEVRFLHLVLYSAERAWSHAMEKRQLPDGPNARQRIYLIGRLRKAVQWANLFSQLCAAK
GDSRTSLEAEAYAAYMNGNLLFEQDRNWDTALKNFISARAVYEELGKYGDLENQVLCRERVEELEPSIR
YCRHKIGQSNLQTSDLLQIGEMEGPALDLFKTKLEAVMAEARSQQAASLTEFHWLGHKFPISNAKTRVA
ILKAQELEKDLHGSAADSLPAEKKLVVFDKIFAAYHEARSCIR**SDLVSAGNADSVKDDLSGLDK**AVSAV
LGQRTIERNQLLVSIAKSKFIKRRDDKNEKLTRPEELVR**LYDLLLQNTSDLSDLVSSGR**DTKPEEVAFS
EDCALRSLAFRAQRCFYLAKSYSLAGKRTEAYSLYCRARSLSENALQKFQSLNNTDEIMVKELKTLYNE
CRSNSCIEHASGIMEDEKAPENLSKKVSAISLSGGDKKVEKFLLEKLDVYESAVGDPHVKAPPRIEVFP
PAFQTIPRNPIILDLAYNYIDFPSLENRMKKDKKGFISRLWR

Protein No.: I-1002

Protein name and Species:

Hydrolase； Leukotriene A-4 hydrolase, putative OS=Ricinus communis GN=RCOM_1071200 PE=4 SV=1

Accession: tr|B9SD61|B9SD61_RICCO

Unused ProtScore: 2.2

Seq Cov %: 5.2

ITRAQ Changed Ratio:

117: 115=E3TL: D3TL=6. 19

116: 114=E5TL: D5TL=3. 09

Annotated typical Precursor MS Region:

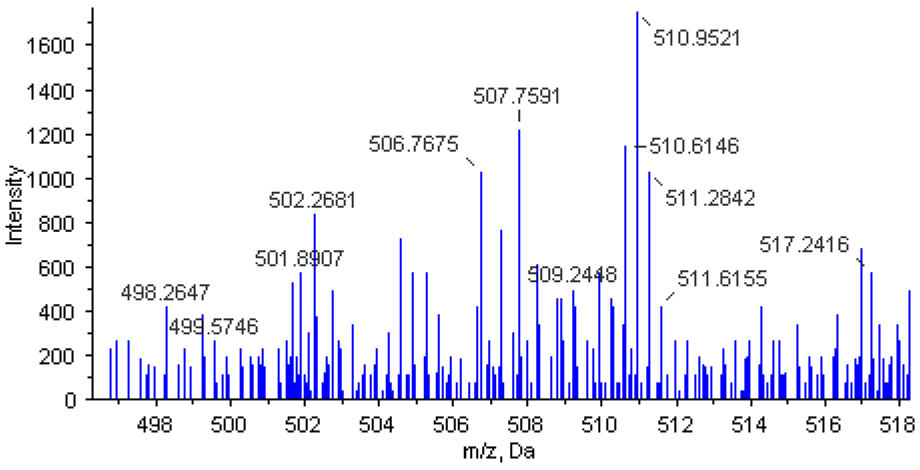

Sequence Coverage and Matched peptides: in different color

MAPVDPHSYTDSTHPLTTHISLSLYFDFSASTIHGTALFSLSTPHTGHISLDTRSLTVHKIADPVTRAL
IPFSLSPEIDPIKGCHLTLSLQNQSSFLILYSTSPSSSALQWLSPPQTFGKAQPFVYTQCQSIHARSVF
PCQDTPAARICYSARLNIPRQLAAVMSARHCHRRAPVSGDVENSIDEGTDFDLKSIWCGEGRVIEEFVM
EQPIPPYLFAF**AVGELGFR**EVGPRTRIYSENVGDVLDAAAREFSGTEEMIRQGEKLFGEYEWERFDLLV
LPPSFPYGGMENPRMVFLTPTVIKGDSSGAQVVAHELAHSWTGNLITNTNNNHFWLNEGFTTYAERRIV
EVVQGEDKAVLNIGIGWRGLNEEVERFKDNMEFTKLK**TNQENADPDDMYSQVPYEK**GFQFLWRIERQIG
RPAFDEFLKKYIATFKFKSIDTDMFLDFLKANVPGIEKDIDLQLWTEGTGIPPDAYEPVSNVYTKIISL
AHEFKLGTMPGEVEVADWQGQEWELYLENLPK**SVEASQIIALDAR**YRLSESKDYEVKVAFLELAILSGC
KDYFSAVEKTLKEVGRMKYLRPLYTALVQGTGKEEEKILAKRVFAEARDCYHPIAQGVVEAIFAKHL

Protein No.: I-1008

Protein name and Species:

Alanine aminotransferase [Medicago truncatula] OS=Medicago truncatula PE=2 SV=1

Accession: tr|B7FM77|B7FM77_MEDTR

Unused ProtScore: 2.19

Seq Cov %: 5.9

ITRAQ Changed Ratio:

117: 115=E3TL: D3TL=5. 06

116: 114=E5TL: D5TL=1. 54

Annotated typical Precursor MS Region:

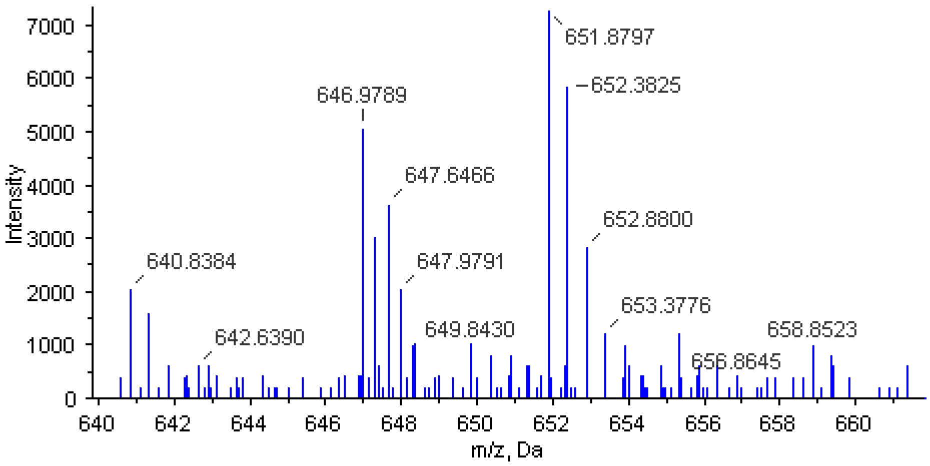

Sequence Coverage and Matched peptides: in different color

MRKSAADRFRHLFNRSLVFVRNQNQQYHHPSPLRSLSSMASDSPFPVTAQNINPQVLKCQYAVRGEIVT
LAQNLQKALQANPDAHSFDEIIYCNIGNPQSLGQQPITFFREVLALCDYPALLDKSETQGLFSADSIER
AWQLVDQIPGRATGAYSHSQGIQGLRDTIAAGIEERDGFPCNANDIFLTDGASPAVHMMMQLLIRSEKD
GILCPIPQYPLYSASITLHGGHLVPYYLDEATGWGLEISELKKQLEEAKSKGISVRALAAINPGNPTGQ
VLAEDNQR**AIVEFCKQEGLVLLADEVYQENVYVPEK**KFHSFKKVSRSMGYGDNDICLVSFQSVSKGYHG
ECGKR**GGYMEVTGFSPDVR**EQIYKVASANLCSNITGQILASLIMSPPKVGDESYESFMAERGAILSSLT
TRAKALEEALNKLEGVTCNK**AEGAMYLFPR**IRLPEKAIKAAEAEKSAPDAFYCKRLLNATGIVVVPGSG
FGQVPGTWHFRCTILPQEDRIPAIVTRLTEFHQKFMDEFRD

Protein No.: I-1016

Protein name and Species:

Ras-related protein Rab11B OS=Nicotiana tabacum GN=RAB11B PE=2 SV=1

Accession: sp|Q40521|RB11B_TOBAC

Unused ProtScore: 2.18

Seq Cov %: 33.2

ITRAQ Changed Ratio:

117: 115=E3TL: D3TL=0. 35

116: 114=E5TL: D5TL=1. 21

Annotated typical Precursor MS Region:

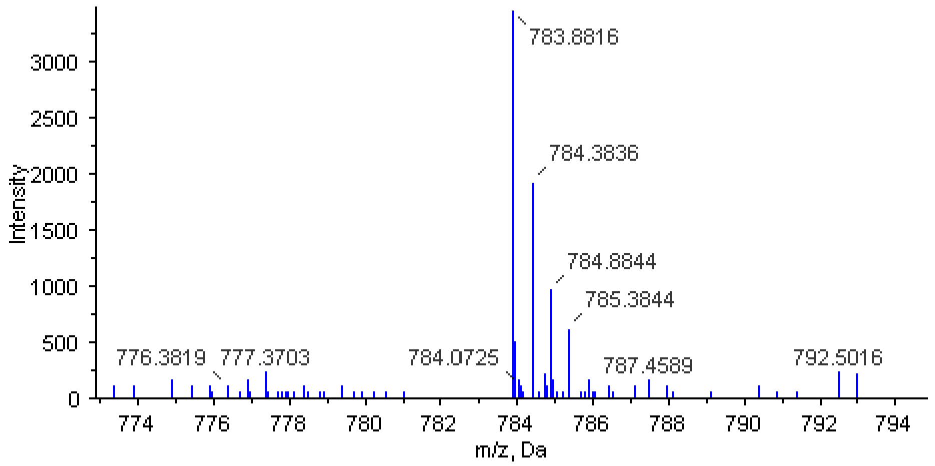

Sequence Coverage and Matched peptides: in different color

MAGGYR**AEDDYDYLFKLVLIGDSGVGK**SNLLSRFSRNEFNLESK**STIGVEFATR**SIRVDDKIVK**AQIWD
TAGQER**YR**AITSAYYR**GAVGALVVYDITR**HVTFENVER**WLKELRDHTDQNIVIMLVGNKADLRHLRAVS
TEDAKAFAERENTFFMETSALESLNVENAFTEVLTEIYKVVCR**KALEVGDDPAALPK**GQTINVGKDDVS
AVKKVGCCSS

Protein No.: I-1019

Protein name and Species:

guanine nucleotide-binding protein subunit beta OS=Vitis vinifera GN=VIT_17s0000g02750 PE=4 SV=1

Accession: **tr|A5BV59|A5BV59_VITVI**

Unused ProtScore: **2.17**

Seq Cov %: **12.2**

ITRAQ Changed Ratio:

117: 115=E3TL: D3TL=1. 67

116: 114=E5TL: D5TL=0. 30

Annotated typical Precursor MS Region:

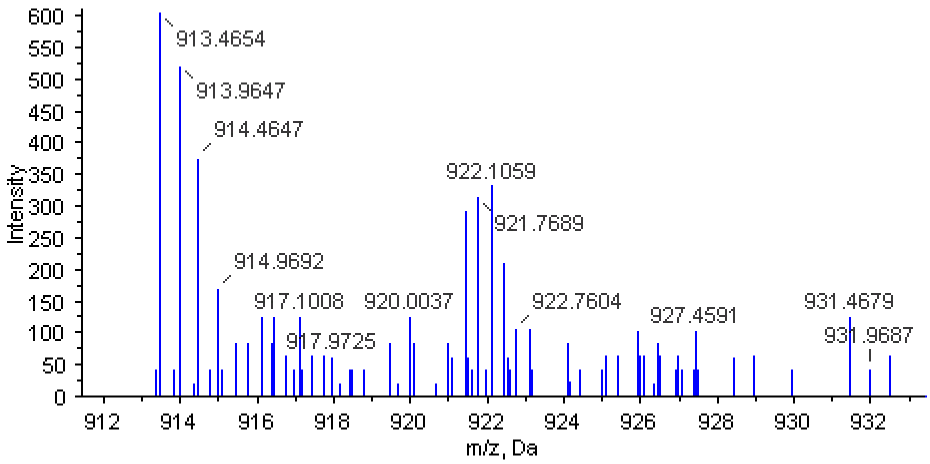

Sequence Coverage and Matched peptides: in different color

MAETLVLRGTMRAHTDMVTAIATPIDNSDMIVTSSRDKSIILWHLTKDDK**TYGVPR**RRLTGHSHFVQDV
VLSSDGQFALSGSWDGELRLWDLAAGTSARRFVGHTKDVLSVAFSVDNRQIVSASRDRTIKLWNTLGEC
KYTIQDADAHSDWVSCVRFSPNNLQPTIVSASWDRTVKVWNLTNCKLR**ATLAGHGGYVNTVAVSPDGSL**
CASGGKDGVILLWDLAEGKRLYSLDAGAIIHALCFSPNRYWLCAATEQSIKIWDLESK**SIVEDLK**VDLK
TEAEKSEDTTTTAKKKGKIIYCTSLSWSADGSTLFSGYTDGVVRVWGIGRY

Protein No.: I-1026

Protein name and Species:

Indole-3-acetic acid-amido synthetase GH3.5, putative OS=Ricinus communis GN=RCOM_0236810 PE=4 SV=1

Accession: **tr|B9T6N8|B9T6N8_RICCO**

Unused ProtScore: **2.15**

Seq Cov %: **3.4**

ITRAQ Changed Ratio:

117: 115=E3TL: D3TL=2. 38

116: 114=E5TL: D5TL=1. 60

Annotated typical Precursor MS Region:

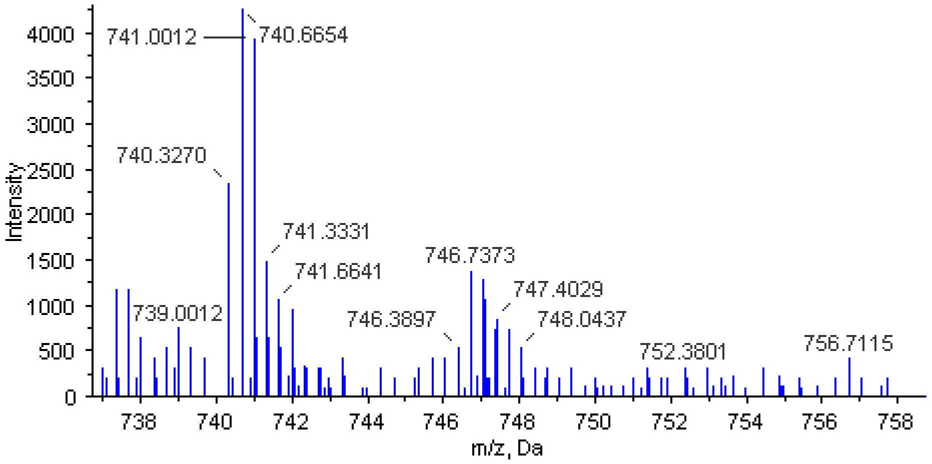

Sequence Coverage and Matched peptides: in different color

MTKNAERVQKETLKK**ILEENGSAEYLQNLGLDGR**TDPESFKICVPICTHGDLEPYIQRIADGDSSPVLT
GKPITTISLSSGTTQGKPKYVPFTDQLLDNTLQIFRTSFAYRNREFPLENGKALEFVFSSKQGKTK**GGL**
AAGTATTNLFRNPNYKTALEELQFKSCSPREVIFGSDFHQSLYCHLLCGLIFREDIQFVFSTFAHSIVL
AFRTFEQVWEELCDDIRNGELSSRITAPSIRTAMSHLLKPNAELADLIHTKCSGLSNWYGLIPELFPNA
KYIYGIMTGSMEPYLKKLRHYAGELPLLSADYGASEGWIAANVNPLLPPELATFAVLPDIGYFEFIPLR
RNGDHIYSEPKPIGLSDVKIGEEYEILVTNFAGFYRYRLGDVVKVMGFHNSTPELKFVCRRSLLLSINI
DKNTEK**DLQLVVEEAAK**LLADEKLEVVDFSSLADRSTDPGHYVIFWEISGEPTAEVLQECCNCLDR**SFL**
DAGYVTSRKVKAIGPLELRVVHRGTFQKILDHYLGLGAAVSQFKTPRCVGPANNVVSQILSNNVAKSYV
STAF

Protein No.: I-1029

Protein name and Species:

Vesicle transport protein-lik OS=Populus trichocarpa GN=POPTRDRAFT_584135 PE=4 SV=1

Accession: **tr|B9N7F9|B9N7F9_POPTR**

Unused ProtScore: **2.15**

Seq Cov %: **11.9**

ITRAQ Changed Ratio:

117: 115=E3TL: D3TL=1. 66

116: 114=E5TL: D5TL=1. 74

Annotated typical Precursor MS Region:

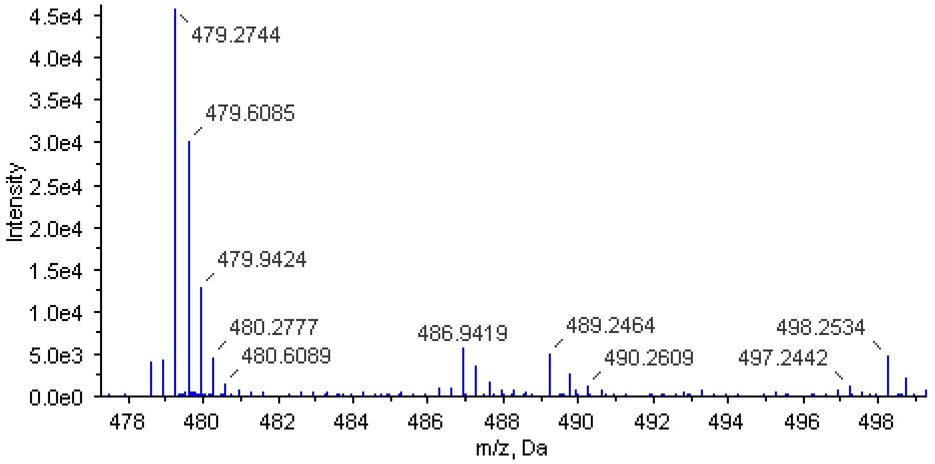

Sequence Coverage and Matched peptides: in different color

MVKLTMIARVTDGLPLAEGLDDGRDVKDAEMYKQQVKALFKNLASGHNDASRMSVETGPYVFHYIIEGR
VCYLTMCDRSYPKKLAFQYLEDLKYEFERVNGAQIETAARPYAFIKFDTFIQKTKKLYQDTRTQRNVAK
LNDELYEVHQIMTRNVQEVLGVGEKLDQVSQMSSRLTSESRIYAEKARDLNRQALIRKWAPVAIVLGVV
FLLFWVKTKLW

Protein No.: I-1030

Protein name and Species:

Proteasome subunit alpha type OS=Populus trichocarpa GN=POPTRDRAFT_818935 PE=2 SV=1

Accession: **tr|A9PCM5|A9PCM5_POPTR**

Unused ProtScore: **2.14**

Seq Cov %: **10**

ITRAQ Changed Ratio:

117: 115=E3TL: D3TL=0. 27

116: 114=E5TL: D5TL=0. 38

Annotated typical Precursor MS Region:

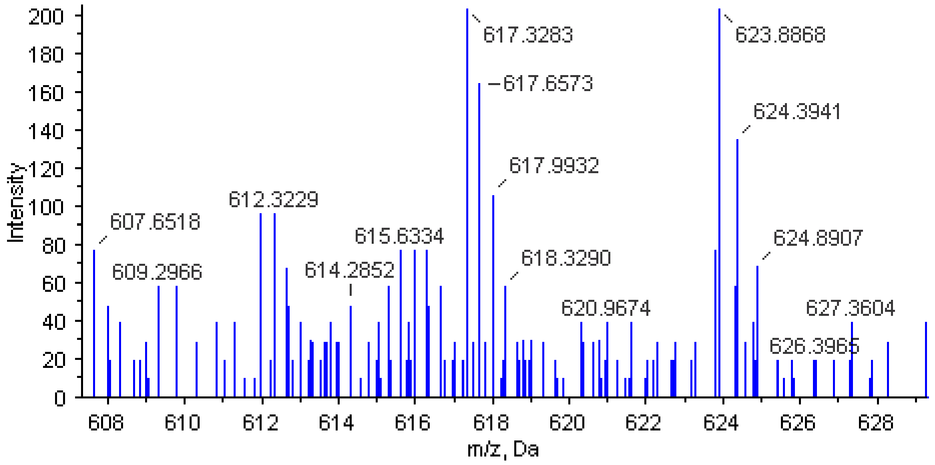

Sequence Coverage and Matched peptides: in different color

MSRRYDSRTTIFSPEGRLYQVEYAMEAIGNAGSAIGILSKDGVVLVGEKKVTSKLLQTSTSTEKMYKID
DHVACAVAGIMSDANILINTARIQAQRYTFAYQEPMPVEQLVQSLCDTKQGYTQFGGLRPFGVSFLFAG
WDKNYGFQLYMSDPSGNYSGWK**AGAIGANNQAAQSMLKQDYKDEITREEAVQLALK**VLSKTMDSTSLTS
EKLELAEVFLSPSGNVKYQVCSPDVLSKLLVKFGVTQPAAETS

Protein No.: I-1037

Protein name and Species:

Aminoacyl-tRNA synthetase-like OS=Populus trichocarpa GN=POPTRDRAFT_552617 PE=3 SV=1

Accession: tr|B9GTA1|B9GTA1_POPTR

Unused ProtScore: 2.13

Seq Cov %: 18.7

ITRAQ Changed Ratio:

117: 115=E3TL: D3TL=0. 28

116: 114=E5TL: D5TL=0. 22

Annotated typical Precursor MS Region:

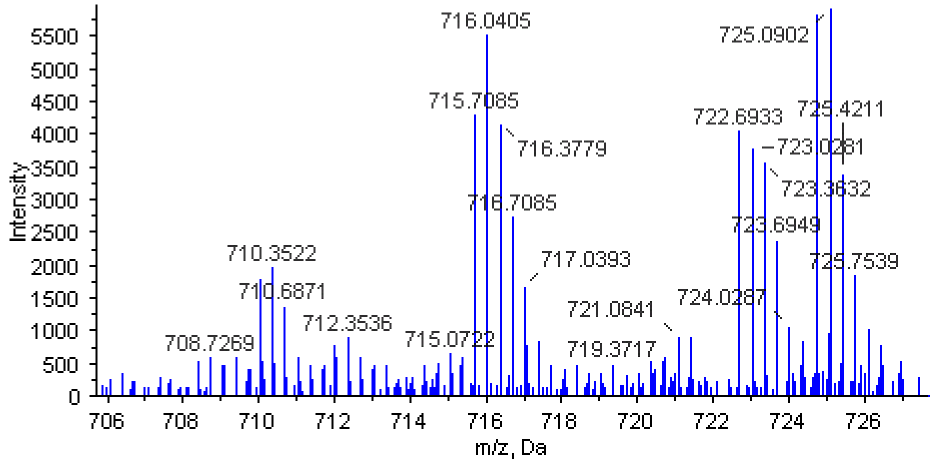

Sequence Coverage and Matched peptides: in different color

MADGKKKEVKKETGLGLTFKKEENFGEWYSEVVVNGEMIEYYDISGCYILRPWSMSIWETMQAFFDVEI
KKMKIK**NCYFPLFVSSSVLQK**EKDHIEGFAPEVAWVTKAGKSDLEVPVAIR**PTSETVMYPYFSK**WIRGH
RDLPLK**LNQWCNVVR**WEFSNPTPFIRSREFLWQEGHTAFASKQEADEEVLQILELYRR**IYEEFLAIPVI**
KGKKSEMEK**FAGGLYTTSVEAFIPNTGRGIQGATSHCLGQNFAKMFEINFENEK**GEKGMAWQNSWAYST
RTIGVMVMVHGDDKGLVLPPK**VASVQVIVVPVPYK**DADTQGIFDACAATVDTLCEAGIRAEADFRENYS
PGWKYSHWEMKGVPLRIEIGPKDLANNQVRAVRRDNAAKVDISRDSLMEKVKDMLDDIQQRLFDAAKQK
RDACIQVVKTWGEFKEALSQRKMILAPWCDEEEVERDVKERTRGEMGAAKSLCSPFDQPELPEGTACFA
SGKPAKKWTYWGRSY

Protein No.: I-1044

Protein name and Species:

Chorismate synthase OS=Vitis vinifera GN=VIT_13s0019g04190 PE=3 SV=1

Accession: tr|D7TMA3|D7TMA3_VITVI

Unused ProtScore: 2.12

Seq Cov %: 6.9

ITRAQ Changed Ratio:

117: 115=E3TL: D3TL=0. 60

116: 114=E5TL: D5TL=1. 09

Annotated typical Precursor MS Region:

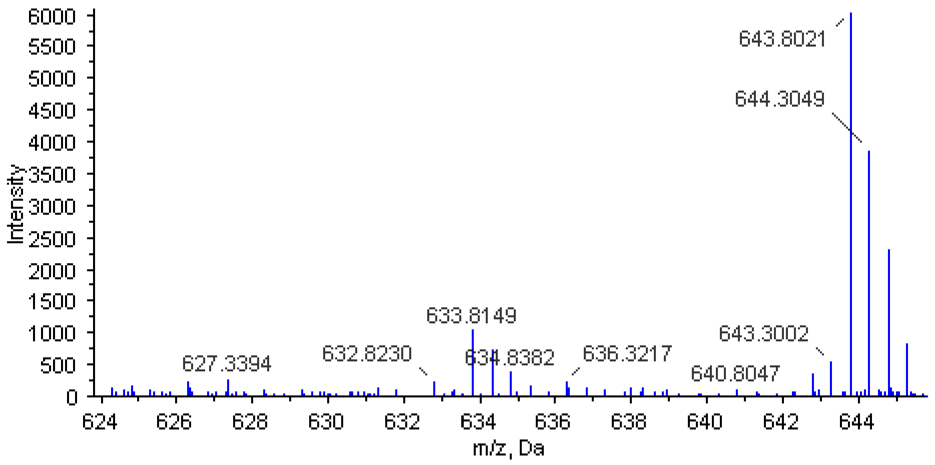

Sequence Coverage and Matched peptides: in different color

MASSLSSKPFLTSPSTDGASGFGSLRKLSFPSVRYAIRPTHRRTLEIQA**AGSTYGTYFR**VTTYGESHGG
GVGCIIDGCPPRLPLTEADMQIDLDRRRPGQSRITTPRKETDTCRIYSGVAEGMTTGSPIMVNVPNTDQ
RGNDYTEMSKAYRPSHADATYDFKYGVRSVQGGGRSSARETIGRVAAGAVAKKILKNFSGTEVLAYVSQ
VHKVVLPEDLVDHETLALDQIESNIVRCPDPEYAEKMIAAIDAVRVR**GDSVGGVVTCIVR**NIPRGLGSP
VFDK**LEAELAK**AALSLPATKGFEFGSGFAGTFMTGSEHNDEFFTDAHGRIRTRTNRSGGIQGGISNGEI
INIRIGFKPTSTIGKKQHTVTRDKQETELIARGRHDPCVVPRAVPMVEAAVALVLVDQLMAQYAQCELF
PINPALQEPLEFRDPELAGTHI

Protein No.: I-1049

Protein name and Species:

pantothenate kinase OS=Vitis vinifera GN=VIT_08s0007g07660 PE=4 SV=1

Accession: tr|F6HLA1|F6HLA1_VITVI

Unused ProtScore: 2.12

Seq Cov %: 3.8

ITRAQ Changed Ratio:

117: 115=E3TL: D3TL=0. 47

116: 114=E5TL: D5TL=1. 01

Annotated typical Precursor MS Region:

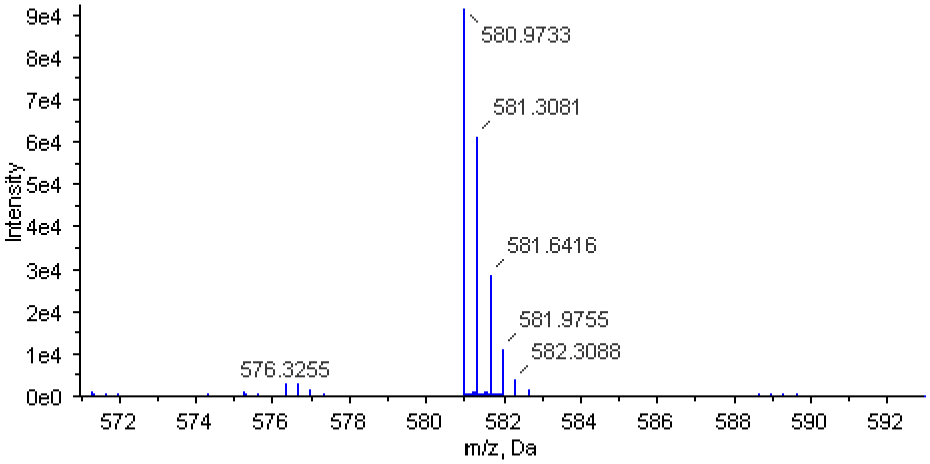

Sequence Coverage and Matched peptides: in different color

MEFPFGHHHHTHHHRPEYGAGEEEEYLQPPPPPPLIHPPPPPPAYFHGEDEPPPPAYFYGEDEPPPPRA
QVHHISHADPPRPPPSSHVHHISHVEDQGFGRENYTHEGPPYARPPPVSYSASAPVRHVSHHLNPQQPQ
GTEQVETHHLHHLPGFLHHHNEAHGVGSNLSNKPTVRVFCKAKPNHSLTILDGKVYLAPSNKTDMLQHW
IKDEK**YSTSVKDEEGFPSFALVNK**ATGQAMKHSIGASHPVQLIPYNPDVLDESVLWTESKDLGDNFRSI
RMINNIHLNVDALHGDRSHGSVHDCTTIVLNKWKKGDNQLWKISLYFENPFPSQLTQSIPLSLNLSLCE
C

Protein No.: I-1051

Protein name and Species:

Carotenoid cleavage dioxygenase 1 OS=Rosa damascena GN=CCD1 PE=2 SV=1

Accession: tr|A9Z0V7|A9Z0V7_ROSDA

Unused ProtScore: 2.11

Seq Cov %: 4.7

ITRAQ Changed Ratio:

117: 115=E3TL: D3TL=0. 60

116: 114=E5TL: D5TL=0. 93

Annotated typical Precursor MS Region:

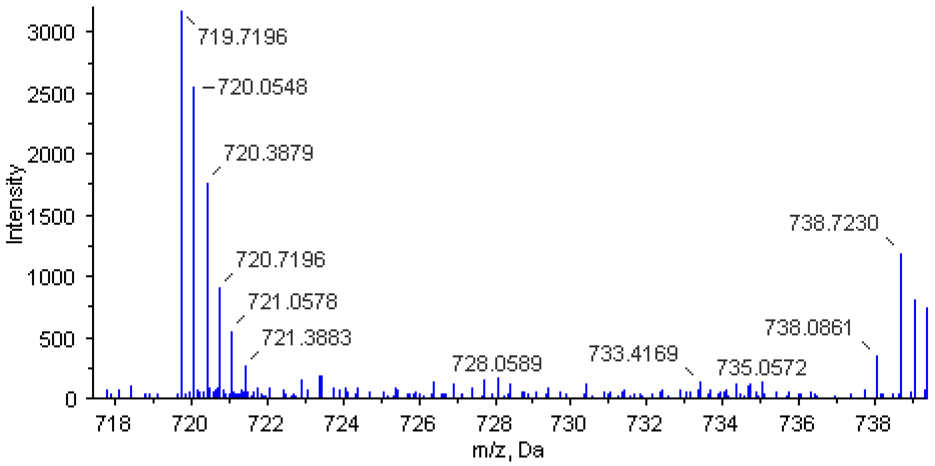

Sequence Coverage and Matched peptides: in different color

MAEVVEKRHDDDVKEKLKSNGIVVPNPKPSKGVTSTLVDTVEKLIVKLMYDSSQPHHYLAGNFAPVIEE
TPPTKDLHVIGHLPDCLNGEFVRVGPNPKFAPVAGYHWFDGDGMIHGMRIKDGKATYVSRYVKTSRLKQ
EEYFGGAKFMKVGDLKGLFGLLMVYMQQLRAKLKVLDLSYGHGTGNTAFVYHHGKLLALSEGDKPYVLK
VLEDGDLQTVGLLDYDKRLKHSFTAHPKVDPFTGEMFTFGYSHDPPYVTYRVISKDGFMHDPIPITVPA
PVMMHDFAITENYAIFMDLPLYFRPKEMVKEGKLIFSFDETKKARFGVLPRYAKDDLLIRWFELPNCFI
FHNANAWEEEDEVVLMTCRLENPDLDMVNGPIKKKLDNFKNELYEMRFNLKTGLATQKKLSESAVDFPR
VNESYTGRKQRYVYGTTLDSIAKVTGIVKFDLHAAPEIGKTKIEVGGNVQGLYDLGPGRFGSEAIFVPR
VPGITSEEDDGYLIFFVHDENTGKSAIHVLDAKTMSGDPVAIVELPHRVPYGFHAFFVTEEQLQEQAKF

Protein No.: I-1059

Protein name and Species:

Nucleoredoxin, putative OS=Ricinus communis GN=RCOM_1599380 PE=4 SV=1

Accession: tr|B9R8F0|B9R8F0_RICCO

Unused ProtScore: 2.1

Seq Cov %: 11.6

ITRAQ Changed Ratio:

117: 115=E3TL: D3TL=0.56

116: 114=E5TL: D5TL=0.75

Annotated typical Precursor MS Region:

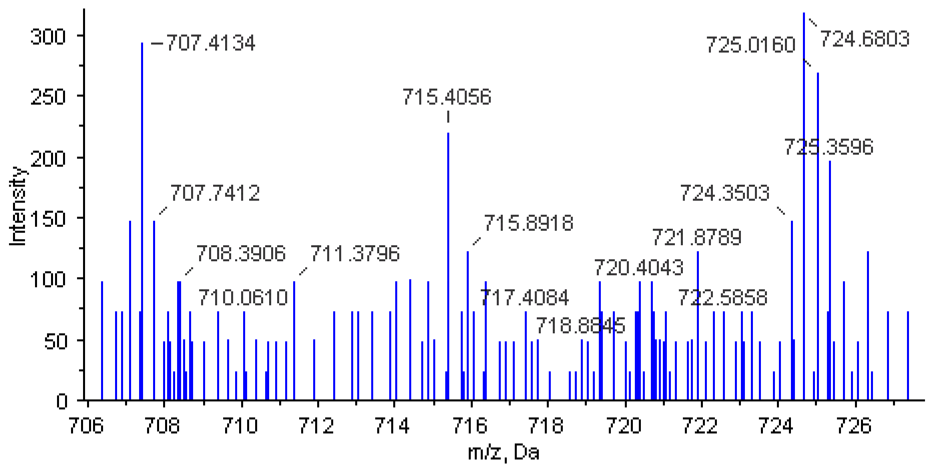

Sequence Coverage and Matched peptides: in different color

MCHYLRLPLALHLRYKVKVSELEGKVIGLYFSANWYPPCRNFNQVLAGVYEQLKENGSNFEVVFVSSDE
NLDAFNNYRALMPWLSIPFSDLETKKALDRKFNIEGVPCLVILQPKDDKEEATLHDGVDLLYR**FGVQAF**
PFTKERLEELKMQEKEKHESQTLTNLLTNHDRDYLFAHPAPKQVPVASLIGKTIGLFFSAQWCRPGMKF
TPKLISIYHKIKQMLRERESEDFEIVFVSTDRDQEGFDSYFNTMPWLALPFGDPTIKTLTK**YFDVQGIP**
CLIIIGPNGKTITKNGR**NLINLYQENAYPFTEAK**VELLEKQMEEEFKSLPRSEYHVGHKHELNLVTEGT
GGGPYICCDCDEQGSGWAYQCLECGYEVHPKCVRVVEPGSTRAR

Protein No.: I-1062

Protein name and Species:

Structural molecule, putative OS=Ricinus communis GN=RCOM_1168960 PE=4 SV=1

Accession: **tr|B9SS56|B9SS56_RICCO**

Unused ProtScore: **2.09**

Seq Cov %: **11.2**

ITRAQ Changed Ratio:

117: 115=E3TL: D3TL=0. 65

116: 114=E5TL: D5TL=1. 15

Annotated typical Precursor MS Region:

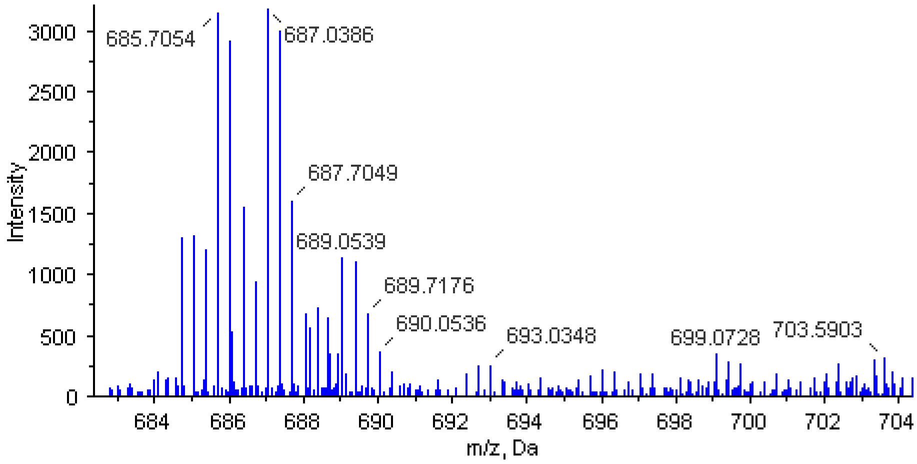

Sequence Coverage and Matched peptides: in different color

MDTLTSLLHPPSFSSSFINHSSSSSSSFTIKAFPKRRLFYINNNTGKSLPVVKSALDEVPVLDPPPPPP
PPPPPFGNERENNKLVASLKLKLLSIVSGLNRGLAASEDDLQKADAAAKELEAVGGLVDLSNDIDKLQG
RWKLIYSSAFSSRTLGGSRPGPPTGRLLPITLGQVFQRIDVLNKDFDNIVELQLGAPWPLPPVEVTATL
AHKFELIGSAKVKITFEKTTVKTTGNLSQLPPLEIPRIPDALRPPSNTGSGDFEVTYVDADTRITRGDR
GELRVFVIA

Protein No.: I-1065

Protein name and Species:

Ribosomal protein L3A OS=Nicotiana tabacum GN=RPL3A PE=2 SV=1

Accession: tr|Q6TKR0|Q6TKR0_TOBAC

Unused ProtScore: 2.08

Seq Cov %: 8.7

ITRAQ Changed Ratio:

117: 115=E3TL: D3TL=1. 66

116: 114=E5TL: D5TL=2. 75

Annotated typical Precursor MS Region:

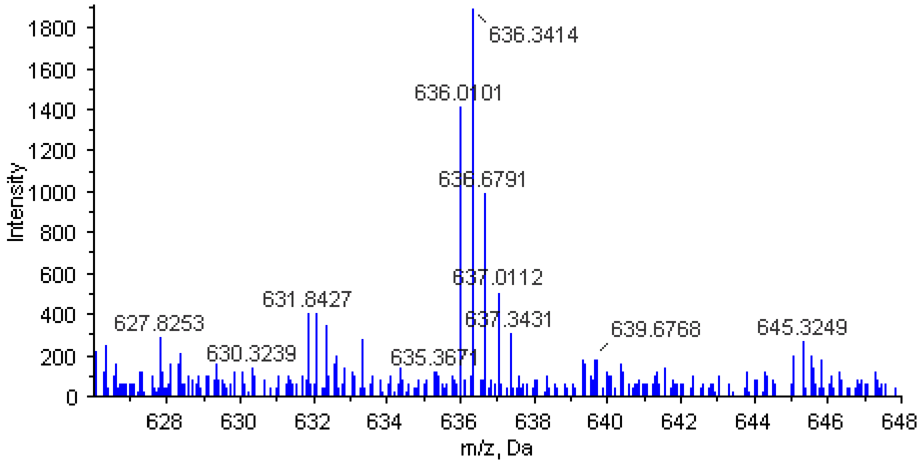

Sequence Coverage and Matched peptides: in different color

MSHRKFEHPR**HGSLGFLPR**KRAARHRGKVKAFPKDDPNKPCK**LTAFLGYK**AGMTHIVRDVEKPGSKLHK
KETCEAVTIIETPPMVIVGVVGYVKTPRGLRCLNTVWAQHLSEELKRRFYKNWCKSKKKAFLKYSKKYE
SDEGKKDIQTQLEKLKKYACVIR**VLAHTQIR**KMKGLKQKKAHLMEIQVNGGTIAQK**VDFAYGFFEK****QVP**
VDAVFQKDEMIDIIGVTKGKGYEGVVTRWGVTRLPRKTHRGLRKVACIGAWHPARVSYTVARAGQNGYH
HRTEMNKKVYKLGKAGQESHAAVTDFDRTEK**DITPMGGFPHYGVVK**DDYLLIKGCCVGPKKRVVTLRQS
LLNQTSR**VALEEIK**LKFIDTSSKFGHGRFQTTQEKQKFYGRLKG

Protein No.: I-1066

Protein name and Species:

Glutamate decarboxylase isozyme 3 OS=Nicotiana tabacum PE=2 SV=1

Accession: tr|Q94KK8|Q94KK8_TOBAC

Unused ProtScore: 2.08

Seq Cov %: 8.1

ITRAQ Changed Ratio:

117: 115=E3TL: D3TL=2. 21

116: 114=E5TL: D5TL=0. 92

Annotated typical Precursor MS Region:

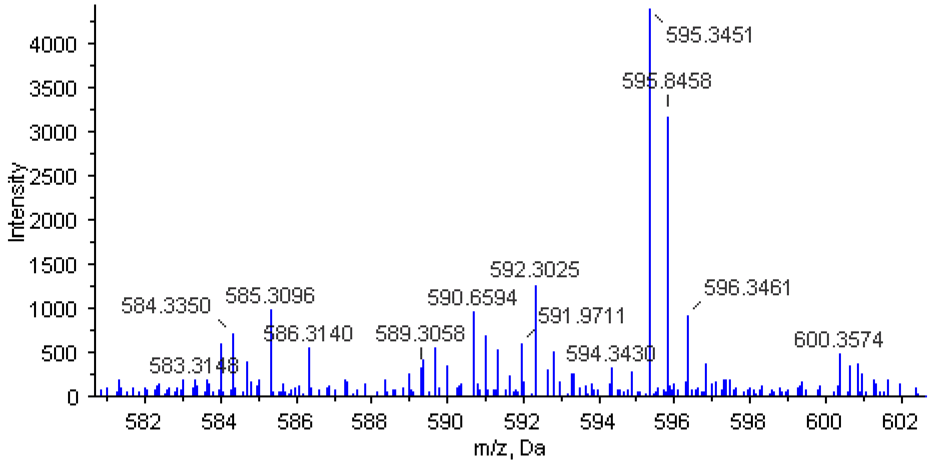

Sequence Coverage and Matched peptides: in different color

MVLSKTSSESDVSVHSTFASRYVRTSLPRFEMAENSIPKEAAFQIINDELMLDGNPRLNLASFVTTWME
PECDKLMMDSINK**NYVDMDEYPVTTELQNR**CVNMIARLFNAPLEEKETAVGVGTVGSSEAIMLAGLAFK
RNWQNKRKAEGKPYNKPNIVTGANVQVCWEKFANYFEVELKEVKLREGYYVMDPVQAVEMVDENTICVA
AILGSTLNGEFEDVK**LLNDLLIEK**NKQTGWNTPIHVDAASGGFIAPFLYPELEWDFRLPLVKSINVSGH
KYGLVYAGIGWVIWRTKQDLPEELIFHINYLGADQPTFTLNFSK**GSSQVIAQYYQLIR**LGYEGYRNVME
NCRENAIVLREGLEKTGRFNIVSKDEGVPLVAFSLKDNSRHNEFEVSETLRRFGWIVPAYTMPADAQHV
TVLRVVIREDFSRTLAERLVLDIVKVLHELDTLPARLSAKLEEVKLVKNGKKFELEVQREVTNYWKKFV
LARKAPVC

Protein No.: I-1067

Protein name and Species:

Aminopeptidase OS=Citrus unshiu GN=ORF12 PE=4 SV=1

Accession: tr|F8WL79|F8WL79_CITUN

Unused ProtScore: 2.08

Seq Cov %: 4.6

ITRAQ Changed Ratio:

117: 115=E3TL: D3TL=0. 59

116: 114=E5TL: D5TL=0. 41

Annotated typical Precursor MS Region:

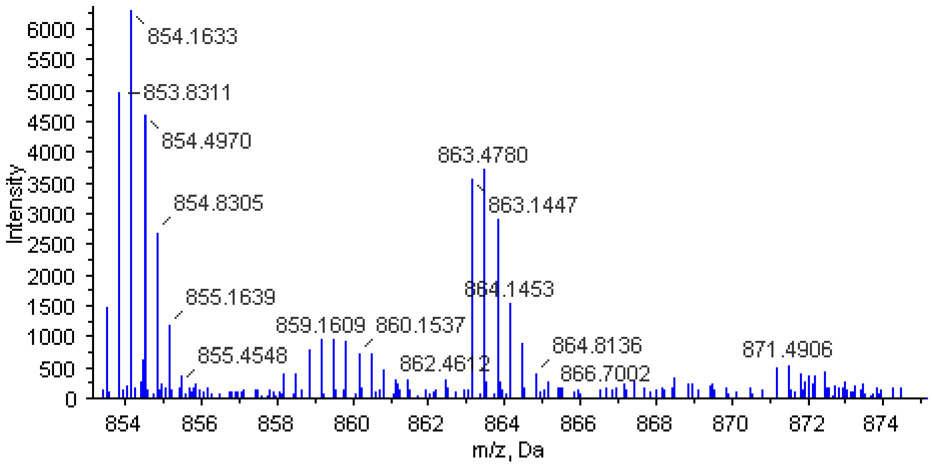

Sequence Coverage and Matched peptides: in different color

MEEFKGQPRLPKFAVPKRYDIRLTPDLTSCKFGGSVAIDVDVVGDTKFIVLNAADLTINNRSVSFTNKA
SSKALEPTKVELVEADEILVLEFAETLPTGMGVLAIGFEGVLNDKMKGFYRSSYELNGEKK**NMAVTQFE**
PADARRCFPCWDEPACKATFK**ITLDVPSELVALSNMPVIDEK**VDGNMKTVSYQESPIMSTYLVAVVIGL
FDYVEDHTSDGTVTISIDSHGIKVRVYCQVGKANQGKFALNVAVKTLELYKEYFAVPYSLPKLDMIAIP
DFAAGAMENYGLVTYRETALLYDDQHSAAANKQRVATVVAHELAHQWFGNLVTMEWWTHLWLNEGFATW
VSYLAADSLFPEWK**IWTQFLDECTEGLR**LDGLAESHPIEVEVNHTGEIDEIFDAISYRKGASVIRMLQN
YLGAECFQRSLASYIKKYACSNAKTEDLWAALEEGSGEPVNKLMNSWTKQKGYPVISVKVREEKLELEQ
ELLGCSISKEGDNGGWIKLNVNQTGFYRVKYDKDLAARLGYAIEKKQLSETDRFGILDDHFALCMARQQ
TLTSLLTLMASYSEETEYTVLSNLITISYKIGRIAADARPELLDYLKQFFISLFQNSAELFICRKLGWD
SKPGESHLDALLRGEIFTALALLGHKETLNEASKRFHAFLADRTTPLLPPDIRKAAYVAVMQKVSASDR
SGYESLLRVYRETDLSQEKTRILSSLASCPDVNIVLEVLNFLLSSEVRSQDAVYGLAVSIEGRETAWKW
LKASGVLSKEVNFDPIPSSINGRISSITGTTSQRPGVLVFLSHALSAQLSHRFVVYTSQIMCSVFQLLH
MCTSNVFPLIPFLFLSAVWHAQFASYEKVREVEEFFSSRCKPYIARTLRQSIERVQINAKWVESIRNEG
HLAEAVKELAYRKY

Protein No.: I-1071

Protein name and Species:

serine/threonine-protein phosphatase 2A 65 kDa regulatory subunit A beta isoform [Vitis vinifera] OS=Vitis vinifera GN=VIT_17s0000g03870 PE=4 SV=1

Accession: tr|D7SIX7|D7SIX7_VITVI

Unused ProtScore: 2.07

Seq Cov %: 14.8

ITRAQ Changed Ratio:

117: 115=E3TL: D3TL=0. 16

116: 114=E5TL: D5TL=0. 28

Annotated typical Precursor MS Region:

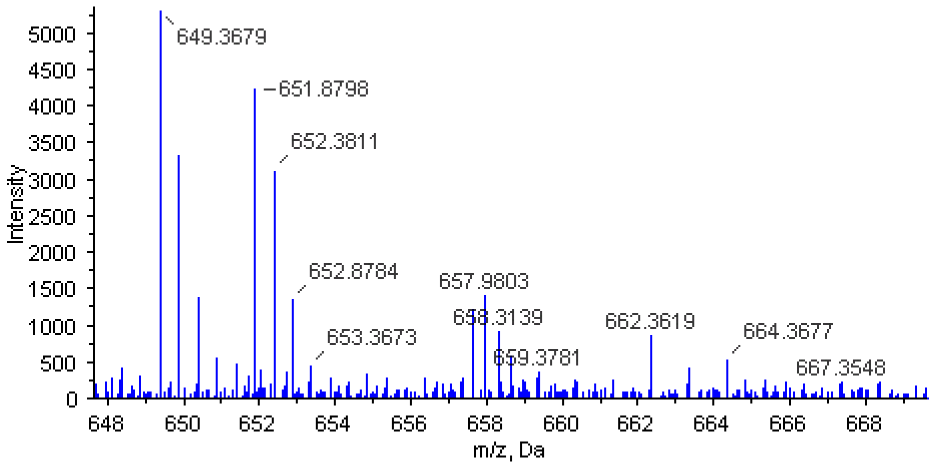

Sequence Coverage and Matched peptides: in different color

MKDTDFFFSALLKLVEGSSLSSPKFQDGLRLPHLRSQILLFPNCPFLFSHFHCSEMAMIDEPLYPIAVL
IDELKNEDIQLRLNSIRRLSTIARALGEERTRKELIPFLSENNDDDDEVLLAMAEELGVFIPYVGGVEH
ANVLLPPLETLCTVEETCVRDK**AVESLCR**IGAQMREPDLVESFIPLVKRLAAGEWFTARVSSCGLFHIA
YPSAPETLKTELR**AIYSQLCQDDMPMVR**RSAASNLGKFAATVEAAHLK**ADIMSIFEDLTQDDQDSVRLL**
AVEGCAALGKLLEPQDCVAH**ILPVIVNFSQDK**SWRVR**YMVANQLYELCEAVGPEPTR**SDLVPAYVRLLR
DNEAEVRIAAAGKVTKFCR**ILNPELAIQHILPC**VKELSSDSSQHVR**SALASVIMGMAPVLGK**DATIDQL
LPIFLSLLKDEFPDVRLNIISKLDQVNQVIGIDLLSQSLLPAIVELAEDRHWRVRLAIIEYIPLLASQL
GVGFFDDKLGALCMQWLKDKVYSIRDAAANNVKRLAEEFGPDWAMQHIIPQVLDMINNPHYLYRMTILH
AISLLAPVMGPEITCSKLLPVVINAAKDRVPNIKFNVAK**VLQSLTPIVDQSVVDKTIRPCLVELSEDPD**
VDVRFFASQALQASDQIMMSS

Protein No.: I-1072

Protein name and Species:

Long-chain acyl-CoA synthetase 4 OS=Ricinus communis GN=LACS4 PE=2 SV=1

Accession: **tr|B3TZ27|B3TZ27_RICCO**

Unused ProtScore: **2.07**

Seq Cov %: **6.5**

ITRAQ Changed Ratio:

117: 115=E3TL: D3TL=0. 42

116: 114=E5TL: D5TL=1. 36

Annotated typical Precursor MS Region:

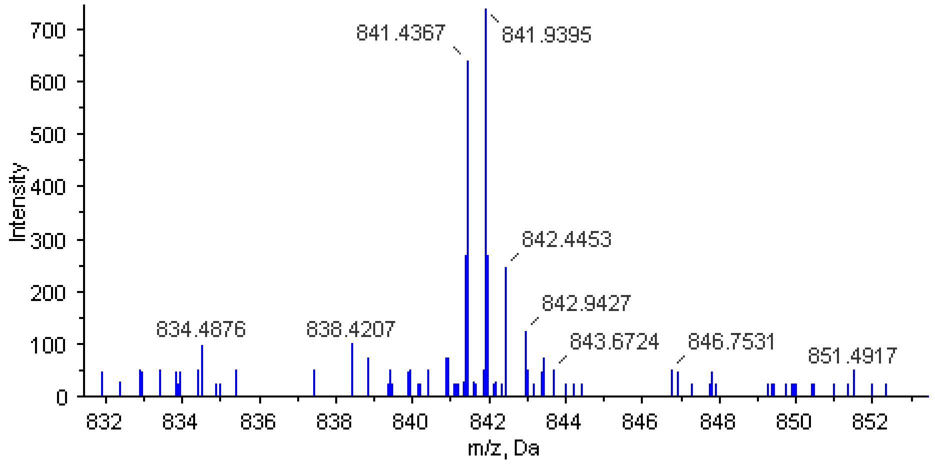

Sequence Coverage and Matched peptides: in different color

MAQQRQRKYLIEVEKAKEAKDGK**PSVGPVYR**SLFAKDGFPPPIPGLDSCWDVFRMSVEKYPNNPMLGHR
EFVNGKAGKYVWQTYKQVYDLVIKVGNAIRSCGVEPGEKCGIYGANSAEWIMSMEACNAHGLYCVPLYD
TLGAGAVEYIICHAEVSIAFVEEKKIPELLKTFPSAAQYIKTIVSFGNIAREQREEMEKFGLVAYSWED
FLKLGENKQYDLPEKKKSDICTIMYTSGTTGDPKGVMISNDSIVTIIAGVRRLLESVNEQLTSEDVYLS
YLPLAHIFDRVIEELFISHGASIGFWRGDVK**LLIEDIGELKPTIF**CAVPRVLDRIHSGLTQKISSGGFL
KNKLFNLAYSYKLSCMKKGLAHDEASPLSDKLVFDKVKQGLGGKVRLILSGAAPLAIHVEAFLRVVSCA
HVLQGYGLTETCAGTFVSLPNEMAMLGTVGPPVPNVDVCLESVPEMNYDALSSTPRGEICVRGSTVFAG
YYKREDLTKEVLIDGWFHTGDIGEWQADGSLKIIDRKKNIFKLSQGEYVAVENLENIYGLASDVDSIWV
YGNSFESFLVAVVNPNKQALEHWAQENSVDGDFKSLCENPRAK**QYIIGELTK**IGKEKKLKGFESIK**AVH**
LDPEPFDIERDLLTPTYKKKRPQLLKYYQKVIDDMYKNASKPSA

Protein No.: I-1081

Protein name and Species:

Malate dehydrogenase OS=Vitis vinifera GN=VIT_07s0005g03350 PE=3 SV=1

Accession: tr|F6HZK0|F6HZK0_VITVI

Unused ProtScore: 2.06

Seq Cov %: 27.4

ITRAQ Changed Ratio:

117: 115=E3TL: D3TL=0. 28

116: 114=E5TL: D5TL=0. 62

Annotated typical Precursor MS Region:

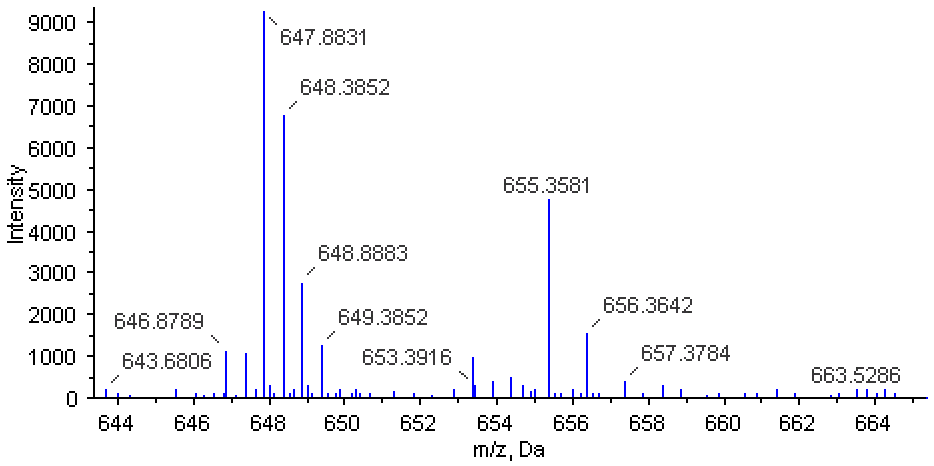

Sequence Coverage and Matched peptides: in different color

MAEDVPRGQTSGLTKSSGTQRRFPLPCSPAIPTMAKEPVR**VLVTGAAGQIGYALVPMIAR**GVMLGADQP
VILH**MLDIPPAAEALNGVKMELVDAAFPLLK**GVVATTDVVEACTGVNIAVM**VGGFPR**KEGMERKDVMSK
NVSIYKSQASALEN**HAAANCKVLVVANPANTNALILKEFAPSIPEK**NISCLTRLDHNRALGQVSER**LNV
QVSDVKNVIIWGNHSSTQYPDVNHATVK**TPAGEKPVRGLVGDDAWLNGEFITTVQQRGAAIIKARKLSS
ALSAASAACDHIRDWVLGTPEGTWVSMGVYSDGSYNVPAGLIYSFPVTCCAGEWK**IVQGLHIDEFSR**KK
LDLTAQELSEEKELAYSCLS

Protein No.: I-1082

Protein name and Species:

Aspartyl aminopeptidase-like protein OS=Populus trichocarpa GN=POPTRDRAFT_835204 PE=3 SV=1

Accession: tr|B9IF79|B9IF79_POPTR

Unused ProtScore: 2.06

Seq Cov %: 8

ITRAQ Changed Ratio:

117: 115=E3TL: D3TL=2. 05

116: 114=E5TL: D5TL=1. 06

Annotated typical Precursor MS Region:

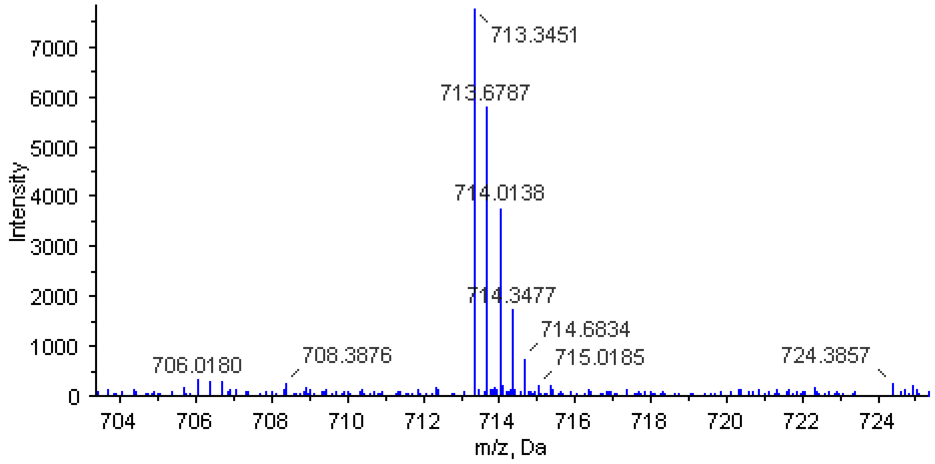

Sequence Coverage and Matched peptides: in different color

MANNNKEQEYCVASDLIDFLNASPTAFHAVEEAKRRLKNAGYEQVSERHDWKLEAGKRYFFTRNHSTIL
AFAIGKK**YVAGNGFYIVGAHTDSPCLK**LKPVSKVTKGGFLEVGVQTYGGGLWHTWFDRDLTVAGRVIVK
EEKDGLVSYSHRLVRIEEPIMRVPTIAIHLERTVNTDGFKVNTQSQLLPVLATSIKAELNKAAAENGPV
KSEEVQADGKKSDKGTISSKHHSLVLEMIANQIGCKVDDICDFELQACDTQPSLIAGAAKEFIFSGRLD
NLCSSFCSLK**ALIDATSSESDLEDESGVR**MVALFDHEEIGSDSAQGAGSPVMLDALSRITSSFNSDSKL
LPKAIQKSFLVSADMAHALHPNYMDKHEENHQPKLHGGLVIKHNANQR**YATNAITSFLFR**EIATKHDLP
TQDFVVRNDMGCGSTIGPILASGVGIR**TVDVGAPQLSMHSIR**EMCSVDDVKYSYEHFKAFFQDISHLDA
KITVDM

Protein No.: I-1085

Protein name and Species:

L-allo-threonine aldolase, putative OS=Arabidopsis thaliana GN=T27C4.17 PE=4 SV=1

Accession: tr|Q9M835|Q9M835_ARATH

Unused ProtScore: 2.06

Seq Cov %: 7.6

ITRAQ Changed Ratio:

117: 115=E3TL: D3TL=1. 58

116: 114=E5TL: D5TL=1. 04

Annotated typical Precursor MS Region:

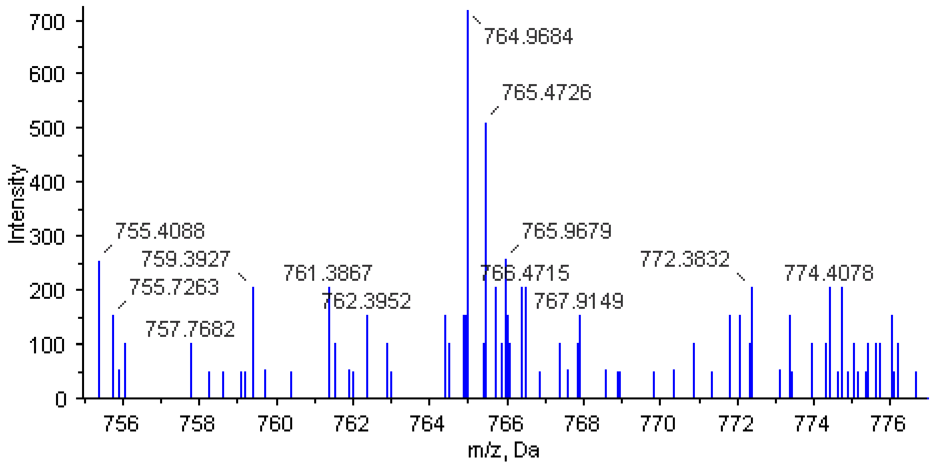

Sequence Coverage and Matched peptides: in different color

MVTPTTIRTVDLRSDTVTKPTESMRSAMANAEVDDDVLGNDPTALRLEKEVAEIAGKEAAMFVPSGTMG
NLISVLVHCDERGSEVILGDDSHIHIYENGGVSSLGGVHPRTVKNEEDGTMEIGAIEAAVRSPKGDLHH
PVTK**LICLENTQANCGGR**CLPIEYIDKVGELAKKHGLKLHIDGARIFNASVALGVPVKRIVQAADSVSI
CLSK**GIGAPVGSVIVGSK**KFITKARWLRKTLGGGMRQIGVLCAAALVALHENVAKLEDDHKKARVLAEG
LNRIERLRVNVAAVETNIVNPKFGAEEACKSLEDVGVLVIPQATFRIRIVLHHQISDVDVEYVLSCFEV
CSLLSSTDMCFDIFNFFATLYSFID

Protein No.: I-1120

Protein name and Species:

Exportin-1 OS=Solanum lycopersicum GN=Xpo1 PE=2 SV=1

Accession: tr|D1MAF2|D1MAF2_SOLLC

Unused ProtScore: 2.03

Seq Cov %: 10.2

ITRAQ Changed Ratio:

117: 115=E3TL: D3TL=0. 60

116: 114=E5TL: D5TL=4. 17

Annotated typical Precursor MS Region:

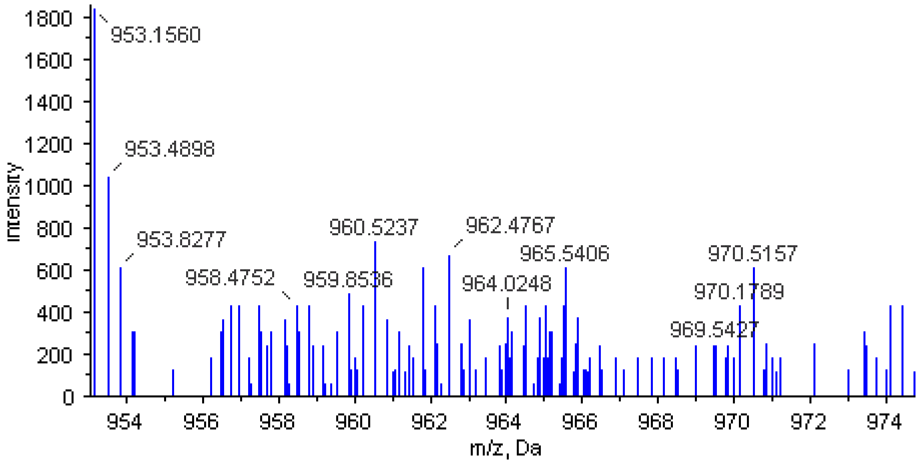

Sequence Coverage and Matched peptides: in different color

MAAEKLR**DLSQPIDVSLLDATVAAFYGTGSK**EERAAADHILRDLQNNPDMWLQVVHILSSTQSLNTKFF
ALQVLEGVIKYRWNALPVEQRDGMKNYISEVIVKLSSDEASLRRERLYISKLNIILVQILKHEWPARWR
SFIPDLVAAAKTSETICENCMAILK**LLSEEVFDFSR**GEMTQQKIKELKQSLNSEFQLIHELCLYVLSVS
HRTELIRATLATLHAFLSWIPLGYIFESTLLEILLKFFPLPSYRNLTLQCLTEVAALNFGDFYNEQYIK
MYTIFMGQLQSVLPPNTSIPEAYANGSNEEQAFIQNLALFFTSFFKSHIRVLESSQENIGALLVGLEYL
INISYVDDTEVFKVCLDYWNSLVLELFEAAHHNLDNPAMTTNMMGLQMPLLSGMNDGLGAQLMQRRQLY
SGPMSKLRLLMISR**MAKPEEVLIVEDENGNIVR**ETMK**DNDVLVQYK**IMRETLIYLSHLDHDDTEKQMLK
KLSNQLNGEDWSWNNLNTLCWAIGSISGSMVEEQENRFLVMVIR**DLLNLCEITK**GKDNK**AVIASNIMYV**
VGQYPRFLRAHWKFLKTVVNKLFEFMHETHPGVQDMACDTFLKIVQKCKRKFVVVQVGENEPFVSELLT
TLPTTIADLEPHQIHTFYESVGQMIQAEPDPQKRDEYLQRLMELPNQRWNEIIGQARQSVDYLKDQDVI
RAVLNILQTNTSAASSLGTYFLPQISLIFLDMLNVYR**MYSELISTSIAQGGPYASR**TSIVKLLRSVKRE
TLK**LIETFLDK**AEDQSHIGK**QFVPPMMDPVLGDYAR**NVPDARESEVLSLFATIINKYKGAMIEDVPRIF
EAAFQCTLEMITKNFEDYPEHRLKFFSLLRAIATHCFAALIRLSSEQLKLVMDSIIWAFRHTERNIAET
GLNLLLAMLKNFQNSEFANQFYRTYYLTIEQEIFAVLTDTFHKPGFKLHVLVLQHLFCMVVMLSEPLWD
ASTVPCSYPNNVEFVREYTIKLLSTSFPNMTAAEVTQFVSGLFDSTNDLPTFKNHIRDFLVQSK**EFSAQ**
DNKDLYAEEAAAQRERERQRMRSIPGLIAPNEIQDEMVDS

Protein No.: I-1121

Protein name and Species:

Glucose 4-6-dehydratase, putative OS=Ricinus communis GN=RCOM_0464520 PE=4 SV=1

Accession: tr|B9SR17|B9SR17_RICCO

Unused ProtScore: 2.03

Seq Cov %: 25.7

ITRAQ Changed Ratio:

117: 115=E3TL: D3TL=2. 13

116: 114=E5TL: D5TL=0. 82

Annotated typical Precursor MS Region:

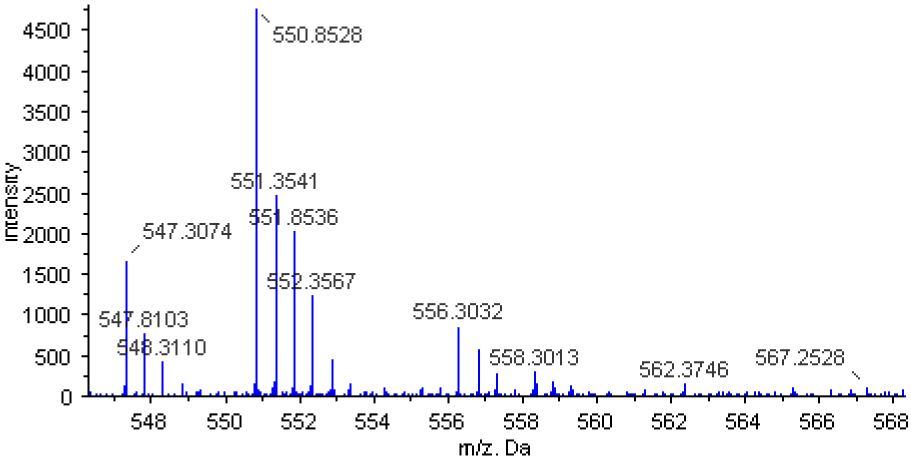

Sequence Coverage and Matched peptides: in different color

MAKEVSNGDHSSASKPPPTPSPLRFSKFFQSNMR**ILVTGGAGFIGSHLVDK**LMENEK**NEVIVVDNYFTG**
SKDNLKKWIGHPRFELIRHDVTEPLLIEVDQIYHLACPASPIFYKYNPVKTIK**TNVIGTLNMLGLAK**RV
GARILLTSTSEVYGDPLVHPQEESYWGNVNPIGVRSCYDEGKR**VAETLMFDYHR**QHGIEIRIAR**IFNTY**
GPRMNIDDGR**VVSNFIAQAVR****NEPLTVQAPGTQTRSFCYVSDMVDGLIR**LMEGENTGPINIGNPGEFTM
LELAETVKELINPDVEIAKVENTPDDPRQRKPDITKAKELLGWEPKIKLR**DGLPLMEDDFR**LRLGVPRK
K

Protein No.: I-1130

Protein name and Species:

copper transport protein [Arabidopsis thaliana] OS=Arabidopsis thaliana PE=4 SV=1

Accession: **tr|Q8LCK3|Q8LCK3_ARATH**

Unused ProtScore: **2.03**

Seq Cov %: **23.3**

ITRAQ Changed Ratio:

117: 115=E3TL: D3TL=0. 42

116: 114=E5TL: D5TL=0. 88

Annotated typical Precursor MS Region:

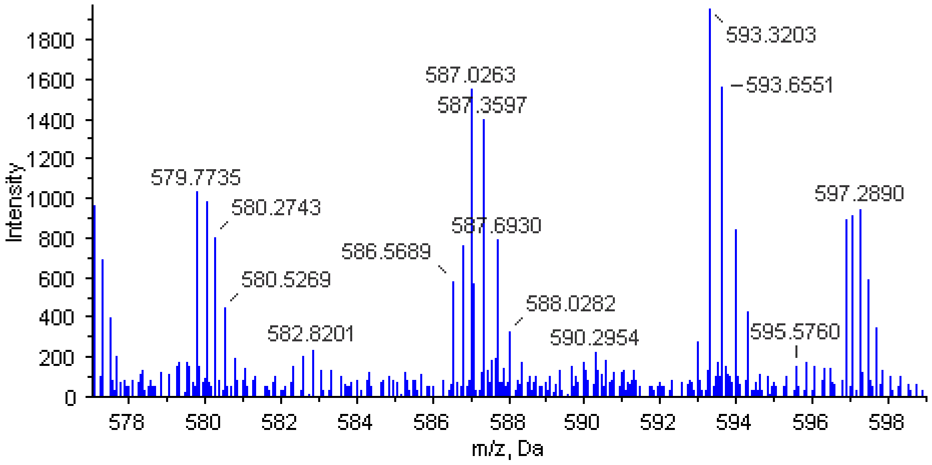

Sequence Coverage and Matched peptides: in different color

MAQKVVLK**VLTMTDDK**TKQKAIEAAADIFGVDSIAADMKDQK**LTVIGLMDAVAVVK**KLKKVGK**VDLISV**
GPAKEEKKEEKKEEKKKEKKEEKKEEKKEEEPKK

Protein No.: I-1136

Protein name and Species:

V-type H+-transporting ATPase subunit I; At2g21410/F3K23.17 OS=Arabidopsis thaliana GN=VHA-A2 PE=2 SV=1

Accession: tr|Q9SJT7|Q9SJT7_ARATH

Unused ProtScore: 2.02

Seq Cov %: 3

ITRAQ Changed Ratio:

117: 115=E3TL: D3TL=0. 51

116: 114=E5TL: D5TL=1. 13

Annotated typical Precursor MS Region:

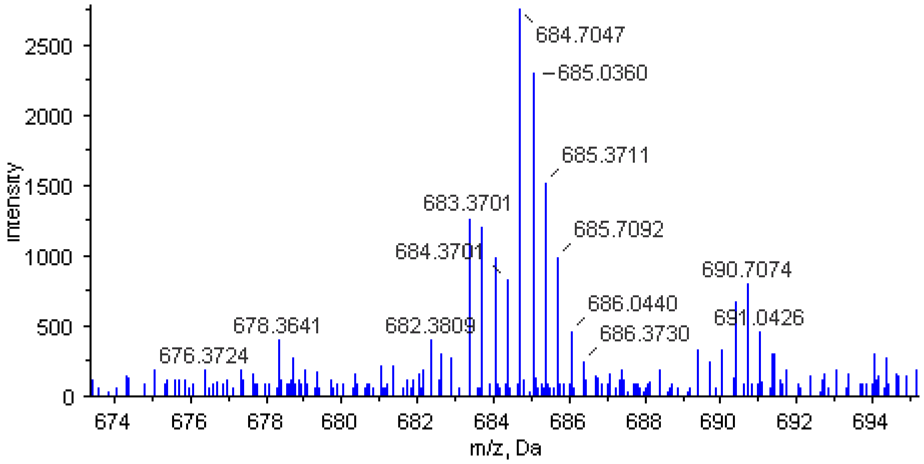

Sequence Coverage and Matched peptides: in different color

MAESHGGGGGCCPPMDLMRSEPMQLVQVIVPMESAHLTVSYLGDLGLVQFKDLNSEKSPFQRTYAAQIK
RCGEMARKIRFFKEQMSKAGVTPKETLDRENDIDLDDVEVKLEELEAELVEINANNDKLQRSYNELVEY
KLVLEKAGEFFASAHRSATAQQSEIETEQVGEDLLEAPLLQEEKSVDPTKQVKLGFLTGLVPREKSMVF
ERILFRATRGNIFIRQSVIEESVVDPNSGEKAEKNVFVVFYSGERAKSKILK**ICEAFGANR**YPFSEDLG
KQAQMMTEVSGRLSELKTTIGAGLDQRNILLETIGDKFEQWNLKIRKEKAIYHTLNMLSLDVTKKCLVG
EGWSPVFAATEIQDALHRAAVDSNSQVGSIFQVLRTKEMPPTFFRTNK**FTTAFQEIVDAYGVAK**YQEAN
PSVFTIVTFPFLFAVMFGDWGHGICLLLATMYLILREKKLSSQKLGDIMEMAFGGRYVIFMMSLFSIYT
GLIYNEFFSIPYPLFASSAYDCRDVSCSEATTIGLIKTRDTYPFGVDPVWHGTRSELPFLNSLKMKMSI
LIGVAQMNLGIIMSFFNAKFFKSAVNIWFQFVPQMIFLNCLFGYLSVLIIIKWCTGSQADLYHVMIYMF
LSPMDDLGENQLFPNQKIVQLTFLFLALVSVPWMLLPKPFILKKQHEARHQGLSYAQLDETDESLQVET
NGGGHGHEEFEFSEIFVHQLIHTIEFVLGAVSNTASYLRLWALSLAHSELSSVFYEKVLLMAWGFNNVF
IWIVGILVFIFATVGVLLVMETLSAFLHALRLHWVEYQNK**FYEGDGYK**FAPFTFTLVGNEDE

Protein No.: I-1137

Protein name and Species:

Translation elongation factor OS=Populus trichocarpa GN=POPTRDRAFT_814408 PE=3 SV=1

Accession: tr|B9IF97|B9IF97_POPTR

Unused ProtScore: 2.02

Seq Cov %: 26.2

ITRAQ Changed Ratio:

117: 115=E3TL: D3TL=0. 61

116: 114=E5TL: D5TL=0. 53

Annotated typical Precursor MS Region:

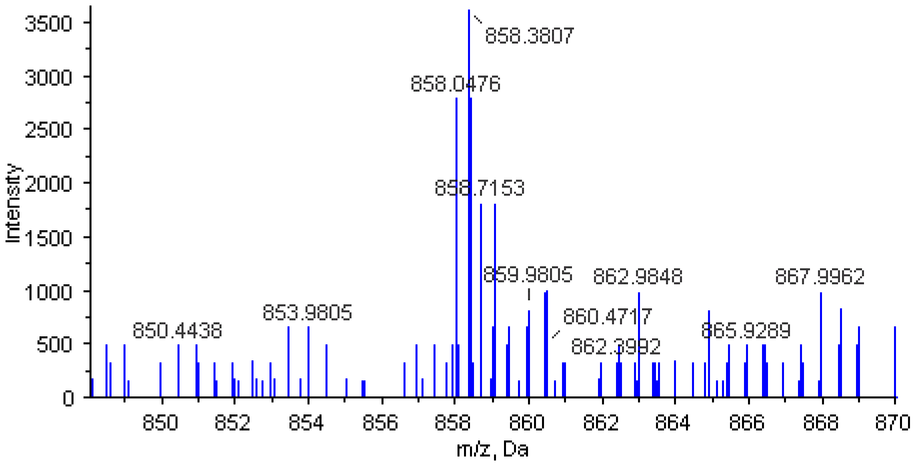

Sequence Coverage and Matched peptides: in different color

MAVTFYDLTSAAGLKKLDDFLLSRSYISGYQASKDDLTVYSALSSAPSAEHVNVYRWYTHIDALLRISG
VEAEGCGVVVKGSAPITEEAIATPPSAETKAAEDDDDDVDLFGEETEEEKKAAEERAATVKAASKKKE
SGKSSVLLDVKPWDDETDMKKLEEAVRSVEMEGLLWGASKLVPVGYGIKKLTIMLTIVDDLVSVDTLIE
ERLTTEPINEYVQSCDIVAFNKKGSR

Protein No.: I-1155

Protein name and Species:

Galactinol-sucrose galactosyltransferase OS=Populus trichocarpa GN=POPTRDRAFT_762165 PE=4 SV=1

Accession: tr|B9HA87|B9HA87_POPTR

Unused ProtScore: 2.01

Seq Cov %: 8.5

ITRAQ Changed Ratio:

117: 115=E3TL: D3TL=11. 07

116: 114=E5TL: D5TL=3. 94

Annotated typical Precursor MS Region:

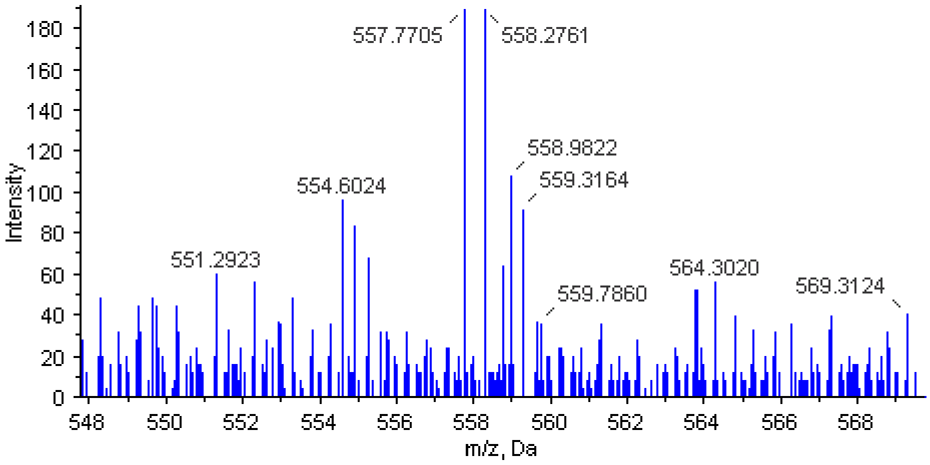

Sequence Coverage and Matched peptides: in different color

MTIKPAVRISDGKLIVKDRTILTGVPDNVIATSGSTSGPVEGVFLGAVFDQENSRHVTSLGALRDVRFM
ACFRFKLWWMAQKMGDQGR**DIPLETQFLLVETK**DGSHLESDGGDEDNQVVYTVFLPLIEGSFRACLQGN
VSDELELCLESGDAETKTSSFTHTLFIHAGTDPFRTITEAVRAVKLHLKTFRQRHEKRLPGIIDHFGWC
TWDAFYQEVTQEGVEAGLQSLASGGTPPKFVIIDDGWQSVGGDPEEETNGQDVKKQDQQPLLRLTGIKE
NAKFQKKDDPAAGIKSIVNIAKEKYGLKYVYVWHAITGYWGGVRPGVKEMEEYGSMMKYPMVSKGVVEN
EPIWKNDALTLQGLGLVNPKNVYRFYNELHSYLAAAGIDGVK**VDVQCILETLGAGLGGR**VELTR**QYHQA**
LDASVARNFLDNGCIACMSHNTDALYCSKQTAVVR**ASDDFYPR**DPVSHTIHIAAVAYNSVFLGEFMQPD
WDMFHSLHAAAEYHASARAISGGPIYVSDAPGKHNFELLKKVVLPDGSILRARLPGRPTSDCLFSDPAR
DGVSLLKIWNMNKFTGVLGVYNCQGAAWSSTERKNAFHQTTTEALTGTIRGRDVHLVAEAATDPNWDGN
CAFYCHRTGELITLPYNAALPVSLK**VLEHDIFTVTPIK**VLAPGFSFAPLGLINMFNAGGAIEGLKYEVK
GKVCVEVKGCGKFGAYSSAKPRKCIVDSNVVDFVYDSNSGLVGFNLDSLLEEGKLRIVEIEL

Protein No.: I-1157

Protein name and Species:

Pyrophosphate-energized membrane proton pump 3 [Vitis vinifera] OS=Vitis vinifera GN=VIT_09s0054g00700 PE=3 SV=1

Accession: tr|D7SS21|D7SS21_VITVI

Unused ProtScore: 2.01

Seq Cov %: 3.8

ITRAQ Changed Ratio:

117: 115=E3TL: D3TL=1. 60

116: 114=E5TL: D5TL=1. 63

Annotated typical Precursor MS Region:

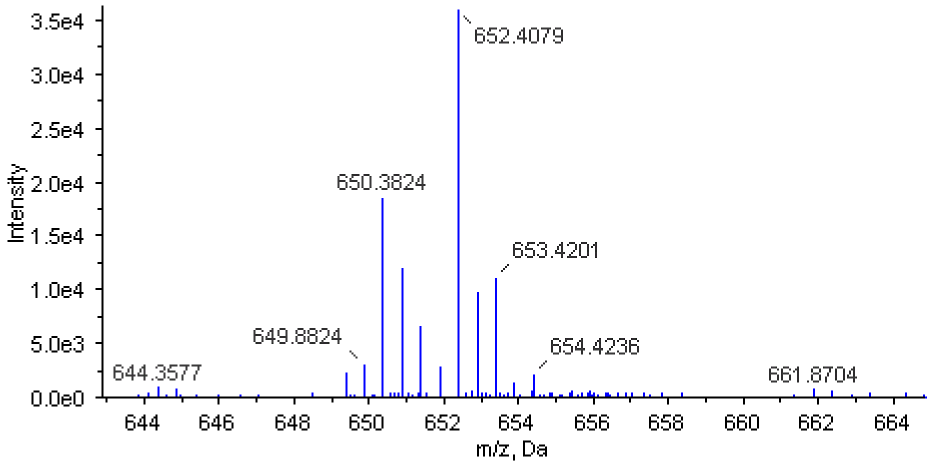

Sequence Coverage and Matched peptides: in different color

MDDDVESGALGPYQDKPRIFPNMRSKSYTPLIFRIFMGINVRVLFVLLLLGLGAVFYVGARTSPILVFV
FSVCIISFLLSVYLTKWVLAKDEGPPEMAQISDAIRDGAEGFFRTQYGTISKMAMLLALVILSIYLFRS
TTPQQESSGIGRSTTAYITVAAFLLGALCSGIAGYVGMWVSVRANVRVSSAARRSAREALQIAVRAGGF
SALVVVGMAVIGVAILYATFYVWLGVDSTGSMKVTDLPLLLVGYGFGASFVALFAQLGGGIYTK**AADVG**
ADLVGKVEQGIPEDDPR**NPAVIADLVGDNVGDCAAR**GADLFESIAAEIISAMILGGTMAQRCKIEDPSG
FILFPLVIHSFDLVISSVGIFSIRGTRDSGVKSPVEDPMAILQKGYSITIILAVLTFGLSTRWLLYTEQ
APSAWMNFALCGLVGIMTAYVFVWITKYYTDYKHEPVRTLALSSSTGHGTNIIAGVSLGLESTALPVIV
ISISIVSAFWLGQTSGLVDETGNPTGGLFGTAVATMGMLSTAAYVLTMDMFGPIADNAGGIVEMSQQPE
SVREITDLLDAVGNTTKATTKGFAIGSAALASFLLFSAYMDEVSAFAHEPFKQVDIAIPEVFVGGLLGS
MLIFLFSAWACSAVGRTAQEVVNEVRRQFIERPGIMDYKEKPDYGRCVAIVASASLREMIKPGALAIVS
PIVVGFLFRILGYYTGHPLLGAKVVASMLMFATVAGILMALFLNTAGGAWDNAKKYIETGALGGKGSDA
HKAAVTGDTVGDPFKDTAGPSLHVLIKMLATITLVMAPVFL

Protein No.: I-1171

Protein name and Species:

Glyceraldehyde-3-phosphate dehydrogenase OS=Dimocarpus longan PE=2 SV=1

Accession: tr|C0LDX2|C0LDX2_9ROSI

Unused ProtScore: 2

Seq Cov %: 33.9

ITRAQ Changed Ratio:

117: 115=E3TL: D3TL=0. 61

116: 114=E5TL: D5TL=0. 22

Annotated typical Precursor MS Region:

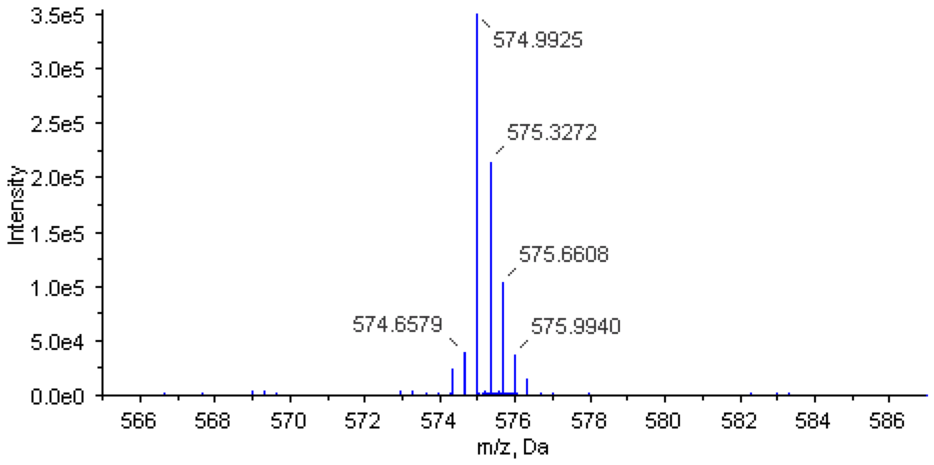

Sequence Coverage and Matched peptides: in different color

MGKIK**IGINGFGR**IGRLVARVILQSDDVELVAVNDPFIDTTYMTYMFKYDSVHGPWKHHELKVKDSKTL
LFGEKAVTVFGIR**NPEEIPWQETGAEYVVESTGVFTDK**EK**AAAHLK**GGAK**KVVISAPSK**DAPMFVVGVN
EH**EYKPEIDIVSNASCTTNCLAPLAK**VINDK**FGIVEGLMTTVHSITATQK**TVDGPSMKDWRGGR**AASFN
IIPSSTGAAK**AVGK**VLPSLNGKLTGMAFR**VPTVDVLVVDLTVRLEKKASYDEIKAAIKEAADGKLK**GIL
GYTEDDVVSTDFVGDNRSSIFDAK**AGIALNDFFVKLVSWYDNEWGYSSRVVDLIRHMAKC

Protein No.: I-1172

Protein name and Species:

Ras-related protein Rab7 OS=Populus trichocarpa GN=POPTRDRAFT_712938 PE=2 SV=1

Accession: tr|A9P8Q6|A9P8Q6_POPTR

Unused ProtScore: 2

Seq Cov %: 73.3

ITRAQ Changed Ratio:

117: 115=E3TL: D3TL=0.62

116: 114=E5TL: D5TL=1.21

Annotated typical Precursor MS Region:

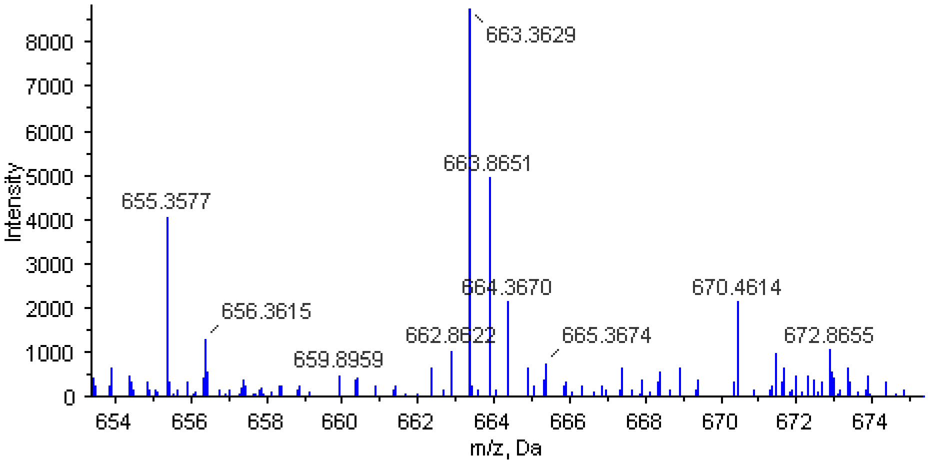

Sequence Coverage and Matched peptides: in different color

MPSRRRTLLK**VIILGDSGVGKTSLMNQYVNK**KFSNQYK**ATIGADFLTKEVQFEDRLFTLQIWDTAGQER**
FQSLGVAFYRGADCCVLVYDVNSMKSFDHLNNWR**EEFLIQASPSDPENFPFVVLGNK**VDVDGGNSRVVS
EKKARAWCASK**GNIPYFETSAKEGVNVEEAFQCIAKNALKSGEEEEIYLPDTIDVGSSSQPR**STGCEC

Protein No.: I-1173

Protein name and Species:

Heat shock protein 80 (Fragment) OS=Euphorbia esula PE=2 SV=1

Accession: tr|Q9M5X2|Q9M5X2_EUPES

Unused ProtScore: 2

Seq Cov %: 40.9

ITRAQ Changed Ratio:

117: 115=E3TL: D3TL=3.22

116: 114=E5TL: D5TL=1.02

Annotated typical Precursor MS Region:

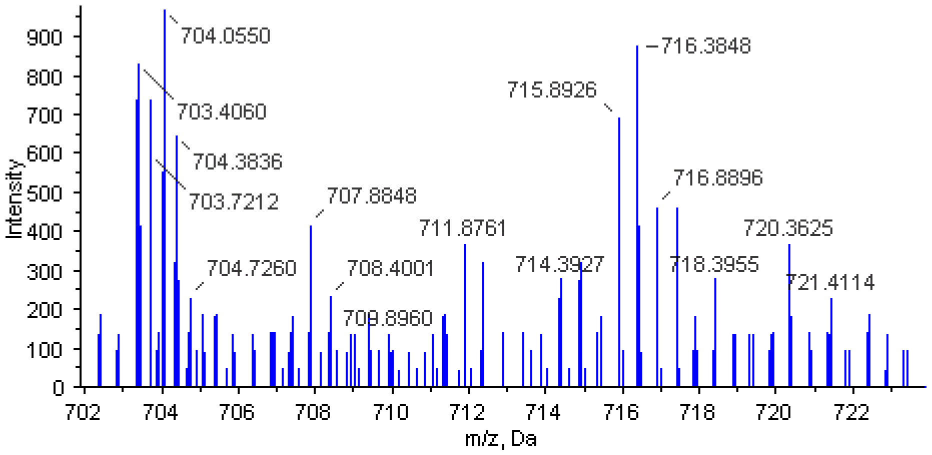

Sequence Coverage and Matched peptides: in different color

VKVIRKNLVKKCIELFQEIAENKEDYNKFYESFSKNLKLGIHEDSQNKTKLAELLRYHSTKSGDEMTSL
KDYVTRMKEGQSDIYYITGESKKAVENSPFLEKLKKKGYEVLFMVDAIDEYAVGQLKEFEGKKLVSATK
EGLKIDESEDEKKKKDELKEQFEGLCKVIKDVLGDRVEKVVVSDRVVDSPCCLVTGEYGWTANMERIMK
AQALRDSSMAGYMSSKKTMEINPENPIMDELRKRADADKNDKSVKDLVLLLFETALLTSGFSLDDPNTF
GNRIHRMLKLGLSIDEDAGEGDADMPALEEADADAEGSKMEEVD

Protein No.: I-1179

Protein name and Species:

Coatomer subunit gamma OS=Populus trichocarpa GN=POPTRDRAFT_821185 PE=3 SV=1

Accession: tr|B9HRP1|B9HRP1_POPTR

Unused ProtScore: 2

Seq Cov %: 11.3

ITRAQ Changed Ratio:

117: 115=E3TL: D3TL=1. 58

116: 114=E5TL: D5TL=0. 71

Annotated typical Precursor MS Region:

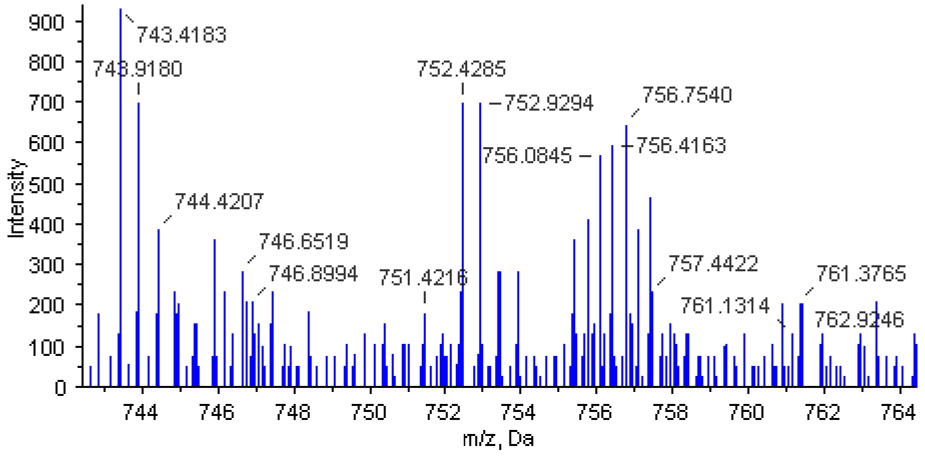

Sequence Coverage and Matched peptides: in different color

MAQPLVKKDDDHDDEAEYSPFLGIEKGAVLQEARVFNDPQLDPRRCSQVITKLLYLLNQGDYFTKTEAT
EVFFSVTKLFQSKDFGLRRMVYLIIKELSPSADEVIIVTSSLMKDMNSKTDMYRANAIRVLCR**ITDGTL**
LTQIERYLKQAIVDKNPVVASAALVSGIHLLQTNPEIVKRWSNEVQEAVQSRAALVQFHALALLQQIRQ
NDRLAVSKLVTSLTRGTVRSPMAQCLLIRYASQVIRESANTQTGDRPFYDFLESCLRHK**AEMVIFEAAR**
AITELSGVTNRELTPAITVLQLFLSSSKPVLRFAAVRTLNKVAMTHPMAVTNCNIDMESLISDQNR**SIA**
TLAITTLLKTGNESSVDRLMKQITNFMSDIADEFKIVVVEAIRSLCLKFPLKYRSLMNFLSNILREEGG
FEYKKAIVDSIVILIRDIPEAKESGLLHLCEFIEDCEFTYLSTQILHFLGIEGPKTTDPSKYIRYIYNR
VHLENATVRAAAVSTLAKFGAMVDALKPR**IFVLLR**R**CIFDSDDEVRDR**TTLYLSTLGGDGEVVETDRDT
KTFLFGDLDIPLVNLETSLK**NYEPSEEPFDIDSVPK**EVKSQPLAEKKAPGK**KPTGLGAPPAGPPSTVDA**
YERLLSSIPEFSDFGKPFK**SSAPVELTEAETEYAVNVVK**HIFDRHVVFQYNCTNTIPEQLLENVSVIVD
SSEADNFAEVASKPLRSLPYDTPGQTFVAFEKPKGITAVGKFSNTLRFIVKEVDPTTGEAEEDGVEDEY
QLEDLEVVAADYMMK**VGVSNFR**NAWESMGDEFEH**VDEYGLGPR**ENLAEAVIAVINLLGMQPCEGTEVVA
TNSRSHTCLLSGVFLGNVRVLARLQFGIHGSRDVAMKLAVRSEDEAVSDTIHEIVSSG

Protein No.: I-1180

Protein name and Species:

Actin-like protein (Fragment) OS=Eperua grandiflora GN=Act PE=3 SV=1

Accession: **tr|D5K0Z8|D5K0Z8_9FABA**

Unused ProtScore: **2**

Seq Cov %: **37**

ITRAQ Changed Ratio:

117: 115=E3TL: D3TL=0. 60

116: 114=E5TL: D5TL=0. 29

Annotated typical Precursor MS Region:

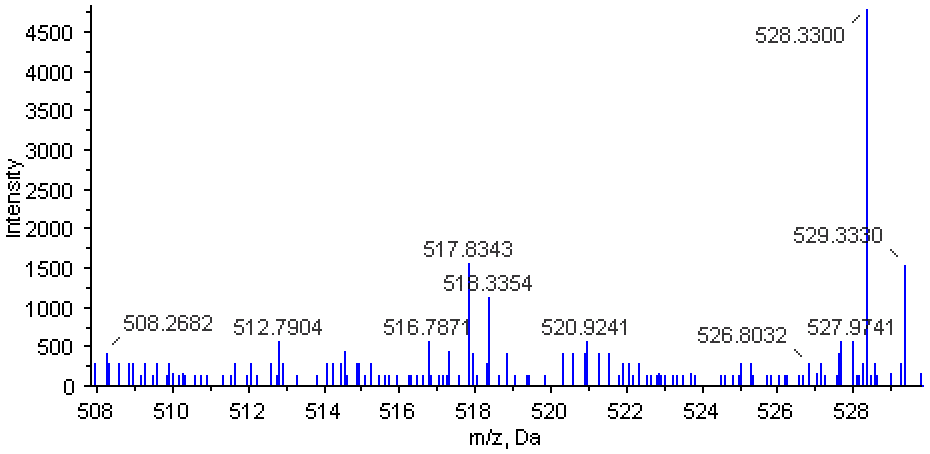

Sequence Coverage and Matched peptides: in different color

FNAPAMYVAIQAVLSLYASGR**TTGIVLDSGDGVSHTVPIYEGYALPHAILR**LDLAGR**DLTDALMK**ILTE
RGYSFTTTAEREIVRDMKEKLAYIALDYEQELETSKTSSAVEK**SYELPDGQVITIGNER**FRCPEVLFQP
SMIGMEAA

Protein No.: I-1186

Protein name and Species:

Luminal-binding protein 5 OS=Populus trichocarpa GN=POPTRDRAFT_570800 PE=3 SV=1

Accession: tr|B9I7J3|B9I7J3_POPTR

Unused ProtScore: 2

Seq Cov %: 8.5

ITRAQ Changed Ratio:

117: 115=E3TL: D3TL=0. 60

116: 114=E5TL: D5TL=0. 25

Annotated typical Precursor MS Region:

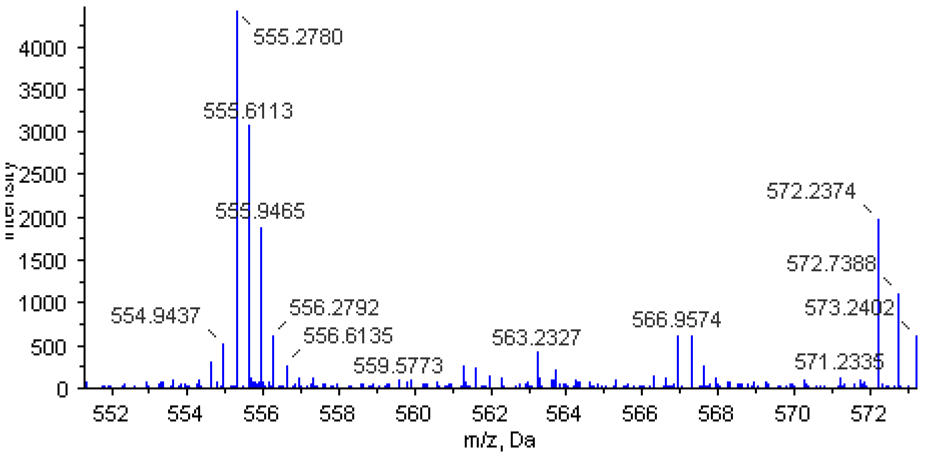

Sequence Coverage and Matched peptides: in different color

MRVKHKAAFAFLFLFVSEYYFLGLVVAADDGPKLGTVIGIDLGTTYSCVAVSR**DGHVEIIANDQGNR**VT
PSWVAFTDTERLIGEAAKNQAPMNPERTIFGVKRLIGRKFDDPEVQRDIKFLPYKVVNKDGKSYIQVKV
KGETKVFSPEEISAMILGKMKETAESYLGKK**IKNAVVTVPAYFNDAQR**QATKDAGIIAGLNVPR**IINEP**
TAAAIAYGLDKKGGDMNILVYDLGGGTFDVSILTIDNGVFEVLSTSGDTHLGGEDFDQRLMDYFINLVK
KKYNKDMSKDKKALGKLRRECERAKRALSSQHQVRVEIESLIDGIDFSEPITRARFEELNMDLFKKTLG
IVKKAMDDAGLKKADIKEIVLVGGSTRIPKVQEMLKEYFDGKEPNKGVNPDEAVAYGAAVQGGILSGEG
GEETKGLLLLDVTPLSLGIETVGGVMTKLIPRNTVIPTKKSQIFTTYQDQQTTVSIKVYEGERSLTKDC
RELGRFDLSGIPPAPRGVPQIEVTFEVDANGILHVKAEDKAAKKSQS**ITITNDK**GR**LSQEEIDR**MVK**EA**
EEMAEEDKKVREKIDARNKLETYIYNMRSTINDKDKLADKIDSDDKERIETALKEALEWLDDNQNAEKD
DYEEKLKEVEEVCDPVIKQVYEKSGSSADSEYEEPNDEL

Protein No.: I-1188

Protein name and Species:

Glyceraldehyde 3-phosphate dehydrogenase (Fragment) OS=Mallotus pierrei GN=G3pdh PE=3 SV=1

Accession: tr|B2X0F6|B2X0F6_9ROSI

Unused ProtScore: 2

Seq Cov %: 45.1

ITRAQ Changed Ratio:

117: 115=E3TL: D3TL=0.65

116: 114=E5TL: D5TL=0.53

Annotated typical Precursor MS Region:

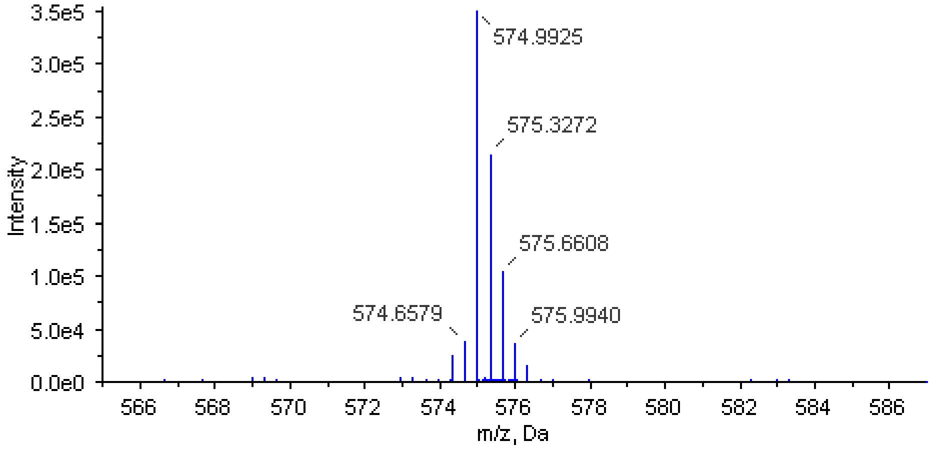

Sequence Coverage and Matched peptides: in different color

VXSITATQKTVDGPSMKDWRGGRAASFNIIPSSTGAAKAVGKVLPSLNGKLTGMSFRVPTVDVSVVDLT
VRLEKEASYDDIKTAIKEESEGKLKGILGYTED

Protein No.: I-1191

Protein name and Species:

Myosin-Vb-like [Vitis vinifera] OS=Vitis vinifera GN=VIT_13s0320g00070 PE=4 SV=1

Accession: tr|F6GZ28|F6GZ28_VITVI

Unused ProtScore: 2

Seq Cov %: 2.9

ITRAQ Changed Ratio:

117: 115=E3TL: D3TL=2. 58

116: 114=E5TL: D5TL=1. 98

Annotated typical Precursor MS Region:

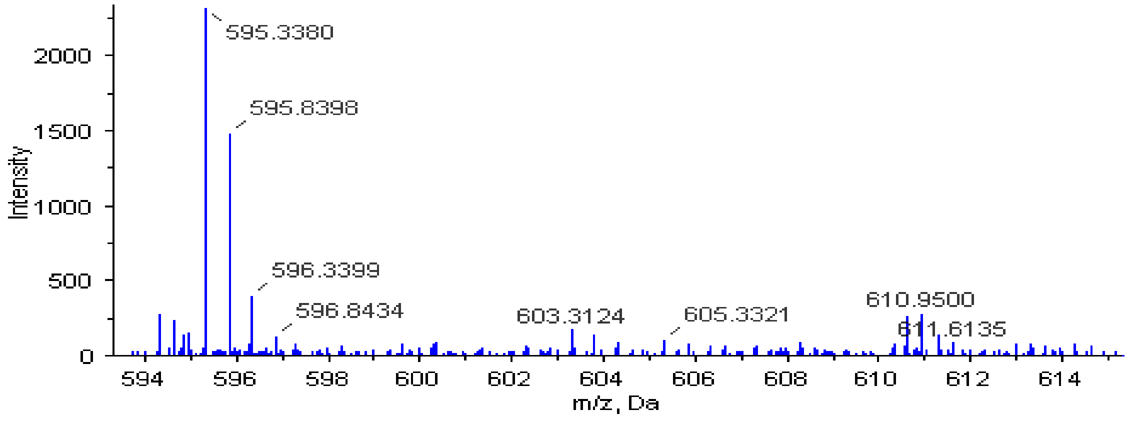

Sequence Coverage and Matched peptides: in different color

MIKGTPVNIIVGSHAWAEDPDAAWIDGEVIGIEGRNATIVTTDGKTIVADISNIYPKDTEAPPAGVDDM
TKLAYLHEPGVLHNLASRFALNEIYTYTGNILIAVNPFQRLPHLYDIHMMGQYKGAAFGELSPHLFAVA
DTCYRAMINEQK**SQSILVSGESGAGK**TETTKMLMRYLAFMGGRSGTEGR**TVEQQVLESNPVLEAFGNAK**
TVKNNNSSRFGKFVEIQFDKHGKISGAAVRTYLLERSR**VCQVSDPER**NYHCFYMLCAAPPEDVKKFKLG
DPRSFHYLNQTNCYEVANVNDAREYLETRNAMDVVGISQDEQDAIFRVVAAILHLGNIGFIKGKEADSS
KLKDEKALYHLRTAAELLMCDEKALEDSLCQRVIVTPDGNITKPLDPDLAVFSRDALAKTVYSRLFDWI
VDKINSSIGQDPNATSIIGVLDIYGFESFKINSFEQLCINLTNEKLQQHFNQHVFKMEQEEYKREEINW
SYVEFIDNQDVLDLIEKKPGGIIALLDEACMFPKSTHETFAQKMYQTYKGHKRFSKPKLARTDFTINHY
AGDVIYQADQFLDKNK**DYVVAEHQALLNASK**CPFVANLFPLLSEEASKQSKFSSIGTRFKQQLQALMET
LSTTEPHYIRCVKPNAVLKPAIFENFNVLNQLRCGGVLEAIRISCAGYPTKRTFDEFFDRFGMLAPDVL
DGADEKSACIAICDRMGLKGYQIGKTKVFLR**AGQMAELDAR**RTEVLANAARRIQRQIQTHLTRKEFIRQ
RRATIHMQKLWRAQLARKLYESMRREAASVCVQKNVRAHTARRNYTNLQASAMAIQTGLRAMAARNEFR
YRRRTKAATLIQTQWRGFQAFSAYNQQKKATLTLQCLWRGRAARKELRKLRMAAR**ETGALK**EAKDKLEK
RVEELTWRLEFEKHLRIDVEEAKGQEISKLQNALLEMQVQLEEAHAAIIREKEAAKIAIEQAPPVLKEV
PVVDNTKMDLLKNQNEELEGEVSELKKMVAEFEQKYCEAQKENTARLKEAEESFTRTSQLQETIERLEL
NLSNLEAENQVLRQQALVASTNEDLFEEMKILKDKIANLESENEVLRNQPTSIEQVAALERVPPQVKSF
DNGHKMEEELQTTKELVPFAPILTKQRSLTDRQQENHDVLIKCLMEDKRFDKNRPVAACIVYKALLQWR
SFEAEKTNIFDRIIHTIRSSIESQESISNLAYWLSTTSTLLFLVQSTLKASNTPNVTSFRSRNSPTTLF
GRMAQGLRSSSFPMGVSSGYSGMVGKPNTHSKVEPKYPALLFKQHLTAYLEKIYGMIRDSLKKEISPFL
NLCIQAPRSTRARSIRGSSKNIHSNIVAKQQASNIHWQNIVNSLDHTLGIMSENHVPSMITRKIFSQVF
SFINVQLFNSLLLRRECCSFSNGEYVKAGLQELEQWCFKAKDEFAGSSWDELQHIRQAVGFLVLHQKPQ
KFLDDITNELCPMLSIPQIYRIGTMFWDDKYGTHGLSPDVIGKMRVLMTEDSINMPNNSFLLDVDSRIP
FSMEEMSRSLIDINLSYVDPPPLLRQRSDFHFLLQPTD

Protein No.: I-1195

Protein name and Species:

Fructokinase-5-like [Vitis vinifera] OS=Vitis vinifera GN=VIT_15s0048g01260 PE=3 SV=1

Accession: tr|D7U7P5|D7U7P5_VITVI

Unused ProtScore: 2

Seq Cov %: 11.3

ITRAQ Changed Ratio:

117: 115=E3TL: D3TL=2.05

116: 114=E5TL: D5TL=1.05

Annotated typical Precursor MS Region:

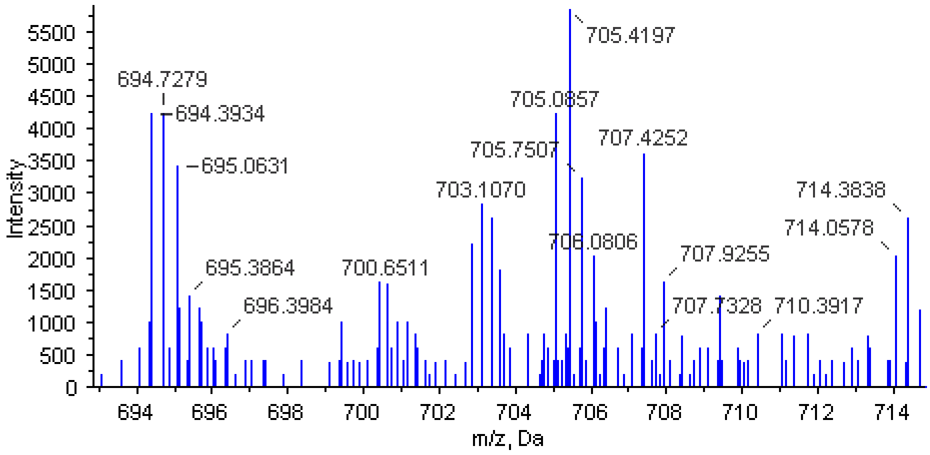

Sequence Coverage and Matched peptides: in different color

MSKSNRSKDDEARMSGCCFPVNLDRSMKSGLSFKKSSSGSFKGESQEKRPLVVCFGEMLIDFVPTVGGV
SLSESPAFKKAPGGAPANVAVGISR**LGGSSAFIGK**VGEDEFGFMLADILKQNNVDSSGMRFDRNAR**TAL**
AFVTLRDDGEREFMFFRNPSADMLLRESELDANLIKKASIFHYGSISLIEEPCKSAHLAAMNIARKSGS
ILSYDPNLRLPLWPSPETARKTIMSIWNQADLIK**ISEEEITFLTGGDDPNDDNVVLK**KLYHSNLKLLVV
TEGSNGCRYYTKEFKGRVTGVKVKAVDTTGAGDAFVGGILNSLAADLNLYKNEEKLREALLFANACGAI
TVTERGAIPALPTKEAVLQILPKANA

Protein No.: I-1196

Protein name and Species:

Ripening-reduced alpha-L-arabinofuranosidase OS=Carica papaya PE=2 SV=1

Accession: tr|C9EA44|C9EA44_CARPA

Unused ProtScore: 2

Seq Cov %: 5.8

ITRAQ Changed Ratio:

117: 115=E3TL: D3TL=0.65

116: 114=E5TL: D5TL=0.53

Annotated typical Precursor MS Region:

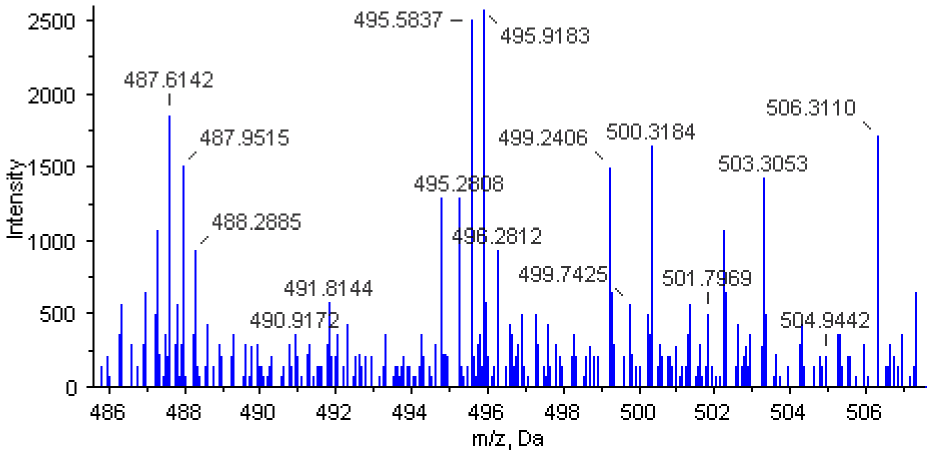

Sequence Coverage and Matched peptides: in different color

MLWADYAKFLCGEVTMSSCKAASSFSSFLCFFLGACCVYQCFALEVGANVTARLIVDASQGASRPMPET
LFGIFFEQEINHAGAGGLWDELVSNRGGFEAGGPNTPSNIDPWSIIGDESSVTVSTERSSCFERNKVAL
R**MEVLCDSR**GANICPSGGVGVYNPGFWGIFLCQNIEQGKKYKVVLYVRSPGAIDLSVSLTSSNGSRTLA
SSNVIASSSDVSNWTKKELLLEAKGTDPHSRLQLTTARKGVIWLDQVSAMPLDTYKVQGHGFRSDLVEM
LLDIKPRFIRFPGGGCFVEGEWLRNAFRWKESIGPWEERPGHFGDVWNYWTDDGLGYFEFLQVGLAEDL
GALPIWVFNNGIGISHNDQVDTSTVLPFVQLQEALDSIEFARGDPDSRWGSVR**AAMGHPEPFDLR**FVAL
GNEDCWKKNYWPGNYLRFYYAIKRAFPDIK**IISNCDGSSR**QLDHPTDYYDFHVVYTSSSNLFSMSHKFD
HASRNGPKVW**AFVSEYAVTGK**DAGTGSLLAALAEAGF**LIGLEK**NSDLVEMTSYAPLFVNVNDRRWNPDA
IVFDSYRQYGTPSYWVQQFFAESSGATLLNSTLQIDSTSLIASAISWQDEKADKKYIRINVNFGSNAVT
LRIYVDGMDPNVARLSGLTETVLRSDNVMDENSFENPEKVRLLFLFLLENAGEDIEVELSPHSFTSFDL
LKEYANIRIAGFDSFSKSST

Protein No.: I-1203

Protein name and Species:

Ras-related protein RHN1-like [Glycine max] OS=Glycine max PE=2 SV=1

Accession: tr|C6TED4|C6TED4_SOYBN

Unused ProtScore: 2

Seq Cov %: 16.3

ITRAQ Changed Ratio:

117: 115=E3TL: D3TL=1. 85

116: 114=E5TL: D5TL=1. 08

Annotated typical Precursor MS Region:

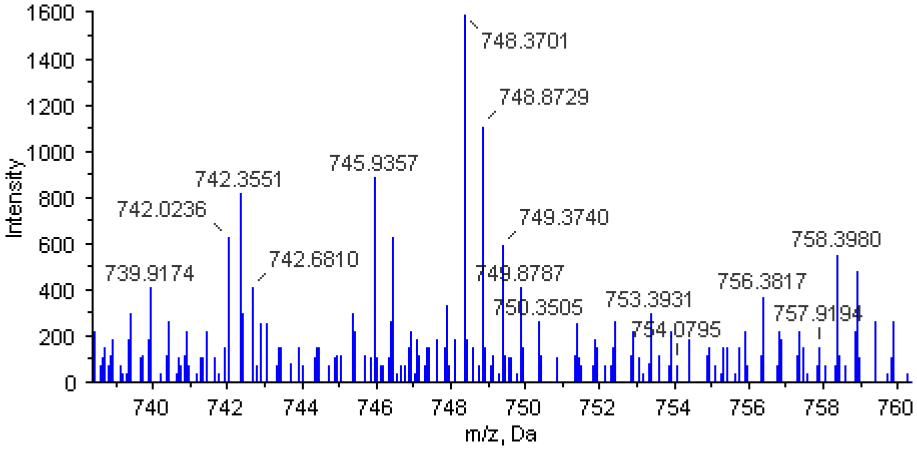

Sequence Coverage and Matched peptides: in different color

MATIGHNNLNAKLVGPSSLFSISFCFFFLSQSFSIPMMFSPCSFHQVLLGDMGAGKSSLVLRFVKGQFL
EFQESTIGAAFFSQTLAVNDATVKFEIWDTAGQERYHSLAPMYYRGAAAAIIVYGITSSDSFTRAKKWV
QELQKQGNPNMVMALAGNKADLEDKRKVTAEEARVYAEENGLFFMETSAKTASNVNDIFYEIAKRLPRA
QPAQNPAGMVLVDRPAEGTRAASCCS

Protein No.: I-1206

Protein name and Species:

Enolase OS=Helianthus annuus GN=ENO1 PE=2 SV=1

Accession: tr|A9P745|A9P745_HELAN

Unused ProtScore: 2

Seq Cov %: 6.1

ITRAQ Changed Ratio:

117: 115=E3TL: D3TL=0. 19

116: 114=E5TL: D5TL=0. 23

Annotated typical Precursor MS Region:

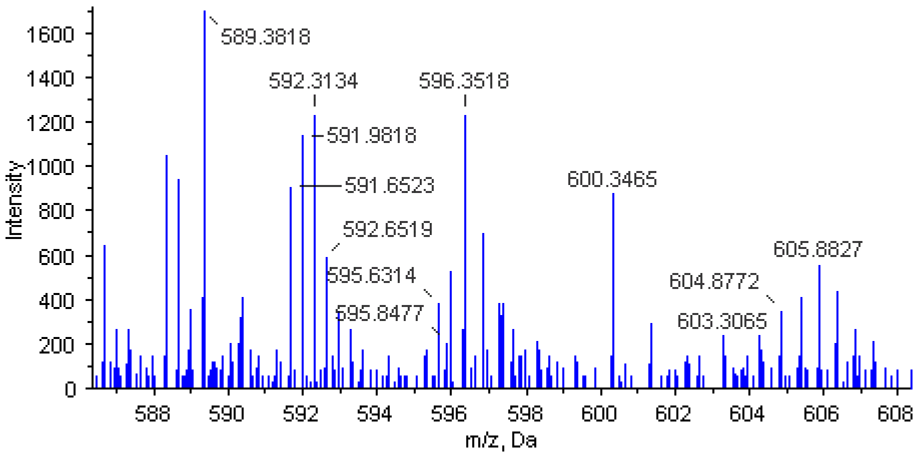

Sequence Coverage and Matched peptides: in different color

MALAPPTTTTSLSNPLLSSKPSLSSPFVNLPTVSRRSPTKFTVRNSVSVAQSPSVSAAKSSTVKSVKAR
QIIDSRGNPTVEVDLVTDGLYRSAVPSGASTGIYEALELRDGDKSVYGGKGVLNAVKNINEVLGPKLIG
VDVRNQAEFDGIMLEIDGTPNKSKLGANAILGVSLSACRAGAGAKSIPLYKHIQEISGTKELVMPVPAF
NVINGGSHAGNNLAMQEFMILPVGATSFAEALRMGSEVYHTLKGIIKAK**YGQDACNVGDEGGFAPNVQD**
NREGLVLLIDAIEKAGYTGKIKIGMDVAASE**FLTK**DGKYDLNFKKQPNDGAHVLSAPSLGDLYREFVRD
FPIVSIEDPFDQDDWTSWTSLQSSVDIQLV**GDDLLVTNPK**RIAEGIQKK**ACNALLLK**VNQIGTVTESIQ
AALDSKAAGWGVMVSHRSGETEDNFIADLSVGLASGQIKTGAPCRSERLAKYNQLLRIEEELGNVRYAG
EAFRSP

Protein No.: I-1207

Protein name and Species:

Rubber elongation factor protein, putative OS=Ricinus communis GN=RCOM_1433280 PE=4 SV=1

Accession: tr|B9RFA8|B9RFA8_RICCO

Unused ProtScore: 2

Seq Cov %: 11.2

ITRAQ Changed Ratio:

117: 115=E3TL: D3TL=1. 51

116: 114=E5TL: D5TL=0. 72

Annotated typical Precursor MS Region:

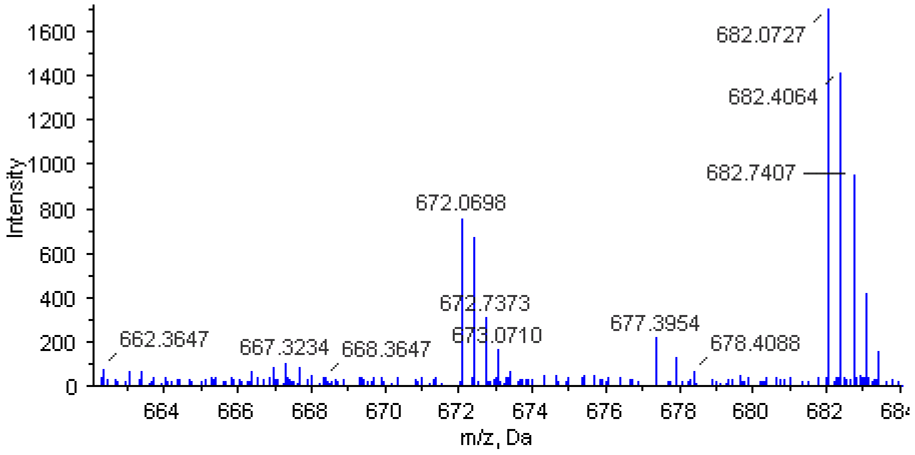

Sequence Coverage and Matched peptides: in different color

MAEQNVNLQQQMAKENEERLKYLEFVQVAALHAVMTFANLYVYAKDK**AGPLKPGVETVEGTVK****SVVGPV**
YDKFHDVPIEVLKFVDRKVDESVTKLDRRVPPVVK**QVSAQAYSVAR**EAPVAARAVASEVQRTGVKETAS
GLAKTLYAMYEPKAKELYSKYEPKAEQCAVSAWRK**LNQLPLFPQVAQVFVPTAAYCSEK**YNQTVVSTAE
KGYR**VSSYLPLVPTQK**IAQVFRNEVPESAPIASS

Protein No.: I-1210

Protein name and Species:

RNA recognition motif-containing protein [Arabidopsis thaliana]

OS=Arabidopsis thaliana GN=At4g17720 PE=2 SV=1

Accession: **tr|Q8LA96|Q8LA96_ARATH**

Unused ProtScore: **2**

Seq Cov %: **6.7**

ITRAQ Changed Ratio:

117: 115=E3TL: D3TL=5. 40

116: 114=E5TL: D5TL=1. 25

Annotated typical Precursor MS Region:

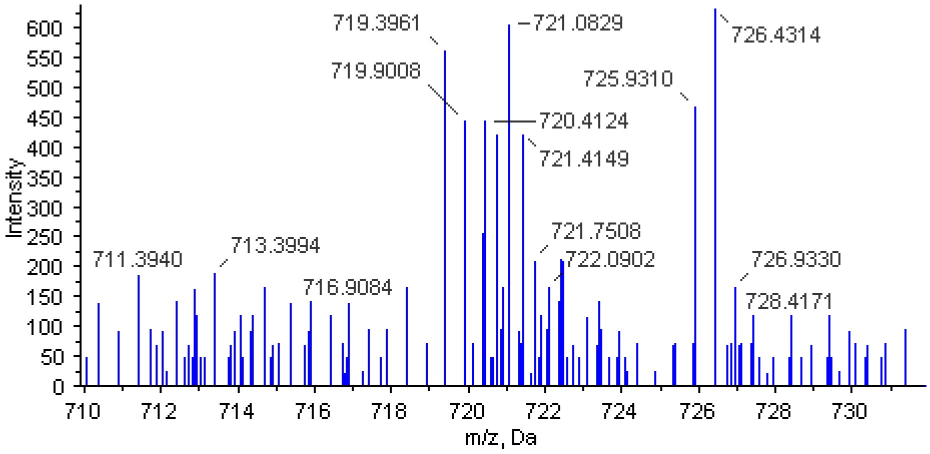

Sequence Coverage and Matched peptides: in different color

MTMTTVKVSNVSLGATDRDLKEFFSFSGDILYLETQSETERTKLAYVTFKDLQGAETAVLLSGATIVDS
SVIVSMAPDYQLSPEALASLEPKDSNKSPKAGDSVLRK**AEDVVSSMLAK**GFILGKDAIAKAKSVDEKHQ
LTSTASAKVASFDKKIGFTDKINTGTVVVGEKVREVDQKYQVSEKTKSAIAAAEQT**VSNAGSAIMK**NRY
VLTGATWVTGAFNKVAKAAEEVGQKAKEKVGMAEEEDKRKVVDEFARVHLSESPKAASSTQEAERESKL
SESPEAKKDSEHLEPQSKPLQQQSPPPMASAPAPAQP

Protein No.: I-1211

Protein name and Species:

GTP-binding protein OS=Nicotiana tabacum GN=SUP1 PE=2 SV=1

Accession: **tr|Q40581|Q40581_TOBAC**

Unused ProtScore: **2**

Seq Cov %: **5.2**

ITRAQ Changed Ratio:

117: 115=E3TL: D3TL=0. 20

116: 114=E5TL: D5TL=0. 51

Annotated typical Precursor MS Region:

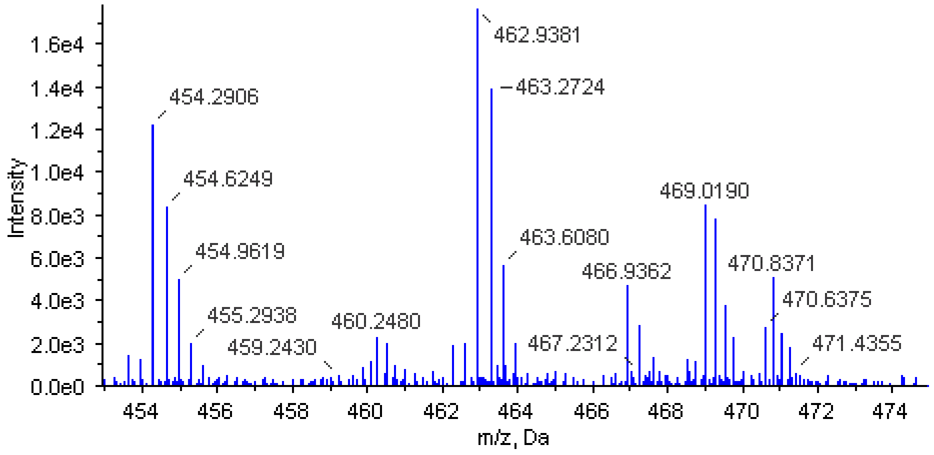

Sequence Coverage and Matched peptides: in different color

MVNVEDGKPEEAIKPEKTNGEDSAMAEEGEAGDDEKPDIPEDELKDEAHPDLEPKNVEPKGIAGKEASP
PEDIQDEVEVNKKRHLNVVFIGHVDAGK**STIGGQILLLSGQVDDR**TIQKYEREAKDKNRESWYMAYIMD
TNEEERVKGITVEVGRAHFETDTTR**FTILDAPGHK**SYVPNMISGRSQADIGLLVISARKGEFETGYERG
GQTREHVQLAKTLGVTKFLVVVNKMDDPTVNWSKERYDEIESKMVPFLRSSGYNVKKDVQFLPISGLLG
SNLKTRMEKSVCPWWNGPCLFETLDAVEVPPRDPNGPLRMPIIDKFKDMGTVVMGKIESGSIHEGDNLL
IIPNKAAVKVLAIFCDEDKVRHVGPGENVRVRLSGVEEDDILSGFVLCSVAKPIHAVTEFVAQLQILEL
LDNAIFTAGYKAVLHIHSVVEECEIVELMQQIDLKTKKPMKKKPLFVKNGAIVLCRVQVNNLICVEKFS
DFAQLGRFTLRTEGKTVAVGKITALPTVADNA

Protein No.: I-1214

Protein name and Species: **FAS-associated factor 2 OS=Populus trichocarpa GN=POPTRDRAFT_548279 PE=4 SV=1**

Accession: **tr|B9GK93|B9GK93_POPTR**

Unused ProtScore: **2**

Seq Cov %: **6.3**

ITRAQ Changed Ratio:

117: 115=E3TL: D3TL=0. 48

116: 114=E5TL: D5TL=1. 01

Annotated typical Precursor MS Region:

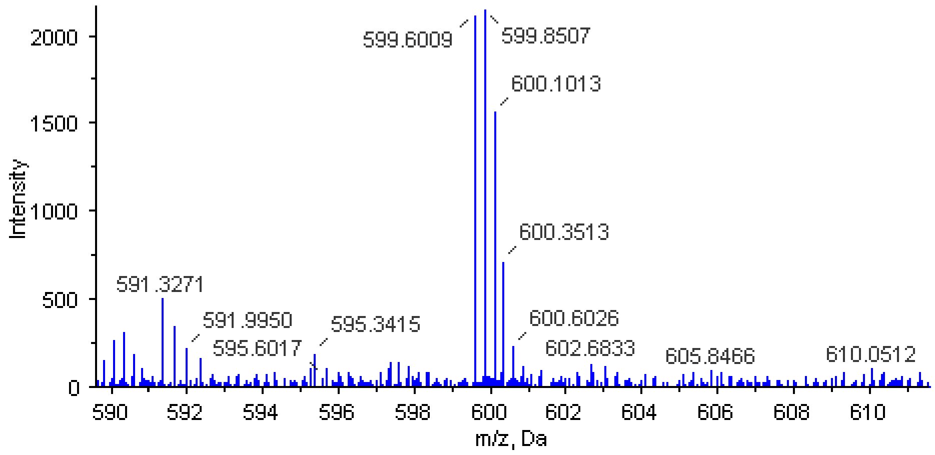

Sequence Coverage and Matched peptides: in different color

MVDVADKLACFRAITGLEDSDLCTEILQAHNWDLELAISSFTSNHSDNHPFSSSYTATDTTATAVDPSN
ATIHRSDSTSIVPAGAPPPGLAWKIVTLPISVISGSLGLISGAVGFGLWAAGGVLSYSLGFIGLGSNSG
RGGDSSAQLVTVSAASREAMEFVAQFERDYGSGSSTRPNFVSEGFMDALTRSRNTFKLLFVYLHSPDHL
DTPAFCEGTLCSEVLSAFVNENFVAWGGSIRGSEGFKMSNSLKASRFPFCAVVMSSTNQRIVLLQHVEG
PK**SPEEMLVILQR**VLEQSAPVLVTARLEAEERTTNMR**LREEQDAAYR**AALEADQARERQQREEQERLER
EAAEAERKRREEEEARDRAASEAAENEAALARMRQEK**ALSLGAEPEKGPDVTQVLVR**FPTGERKERRFH
CAATIQSLYDYVDSLGSLNVENYSLVSNFPRVVYGTDKVSLSLKEAGLHPQASLFVELN

Protein No.: I-1225

Protein name and Species:

carboxymethylenebutenolidase, putative OS=Vitis vinifera GN=VITISV_041925 PE=4 SV=1

Accession: **tr|A5AEX6|A5AEX6_VITVI**

Unused ProtScore: **2**

Seq Cov %: **3.7**

ITRAQ Changed Ratio:

117: 115=E3TL: D3TL=0. 66

116: 114=E5TL: D5TL=0. 63

Annotated typical Precursor MS Region:

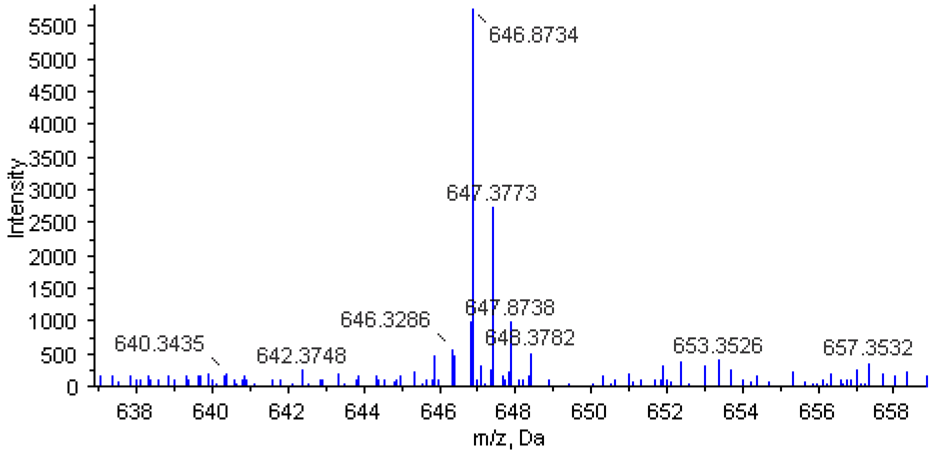

Sequence Coverage and Matched peptides: in different color

MAESASSPFKKIQIQR**DDTTFDAYVVGK**EDAPGIVVVQEWWGVDYEVKNHALK**ISQLDPGFK****ALIPDLY**
RGKVGLDVAEAQHLMDGLDWQGAVKDICASVNWLKANGSQKVGVTGYCMGGALSIASSVLLPEVDAVVA
FYGVPSSELADPAKAKAPVQAHFGELDSFVGFSDVTAAKALEEKLKASGVPYEVHIYPGNGHAFMNRSP
EGIQRRKGMGMPDEDEDAVGLAWSRFRAWMSCYLSP

Protein No.: I-1228

Protein name and Species:

Prefoldin subuni OS=Populus trichocarpa GN=POPTRDRAFT_714512 PE=4 SV=1

Accession: tr|B9H401|B9H401_POPTR

Unused ProtScore: 2

Seq Cov %: 16.8

ITRAQ Changed Ratio:

117: 115=E3TL: D3TL=0. 61

116: 114=E5TL: D5TL=0. 52

Annotated typical Precursor MS Region:

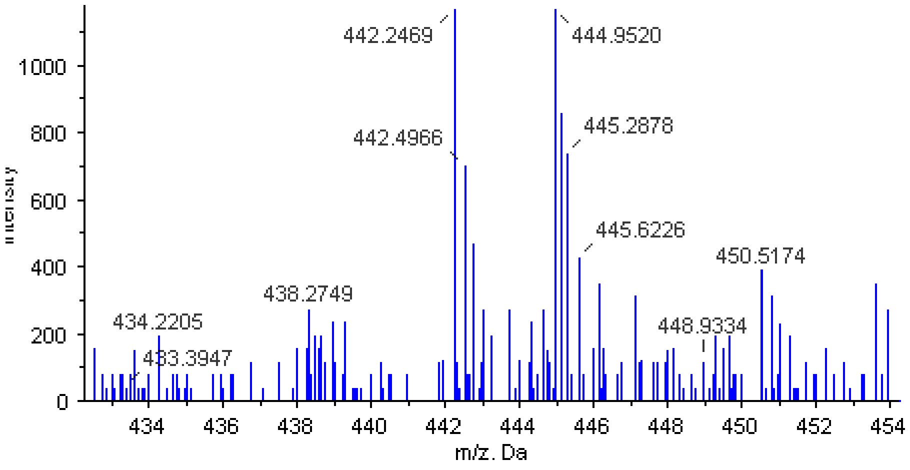

Sequence Coverage and Matched peptides: in different color

AIRELQRDLENKANDLSKLQK**DIAK**NHQVRKK**YTIQLGENELVLK**ELDLLNEGANVYKLIGPVLVK**QDL
AEANANVSKRIEYISAELKR**LDGTLQDLEEK**QNSKKDAIFKLQQRIQSFQAGKAKA

Protein No.: I-1231

Protein name and Species:

60S ribosomal protein L14-2-like [Vitis vinifera] OS=Vitis vinifera GN=VIT_05s0049g01310 PE=4 SV=1

Accession: **tr|D7SZI9|D7SZI9_VITVI**

Unused ProtScore: **2**

Seq Cov %: **8.2**

ITRAQ Changed Ratio:

117: 115=E3TL: D3TL=2. 29

116: 114=E5TL: D5TL=3. 08

Annotated typical Precursor MS Region:

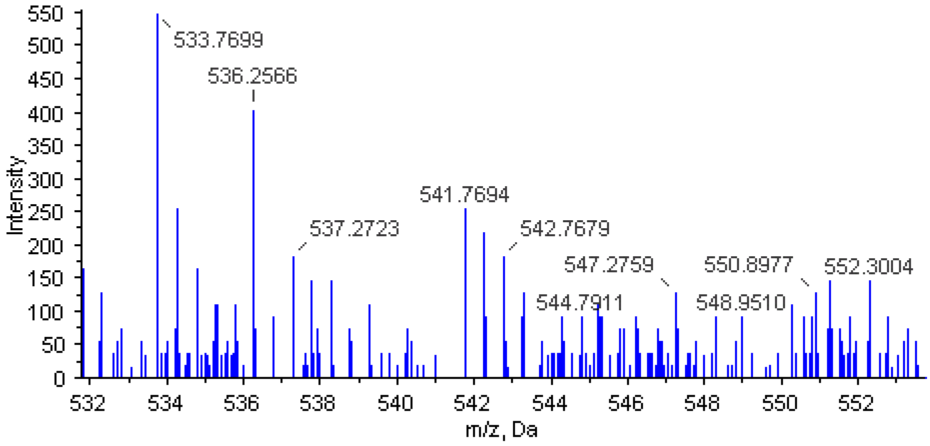

Sequence Coverage and Matched peptides: in different color

MEFFNLYNLHGHKVLNQIRLISGFNSPKKAQRASKPNTIVTQRSRTQIGPKRTIKKPRKTLSPVSPSTP
LASSIFPLPAVSRALANMPFKRYVEVGRVALVNYGKDYGRLVVIVDVIDQNRALVDAPDMVRCQMNFKR
LSLTDIKIDIKRVPKKKTLIEAMTAADVKNKWENSSWGRKLIVQKRRASLNDFDRFKIMLAKIKRGGVI
RQELAKLKKQTAS

Protein No.: I-1236

Protein name and Species:

chitinase 1 OS=Populus trichocarpa GN=POPTRDRAFT_557015 PE=4 SV=1

Accession: **tr|B9HQZ2|B9HQZ2_POPTR**

Unused ProtScore: **2**

Seq Cov %: **6**

ITRAQ Changed Ratio:

117: 115=E3TL: D3TL=0. 52

116: 114=E5TL: D5TL=0. 37

Annotated typical Precursor MS Region:

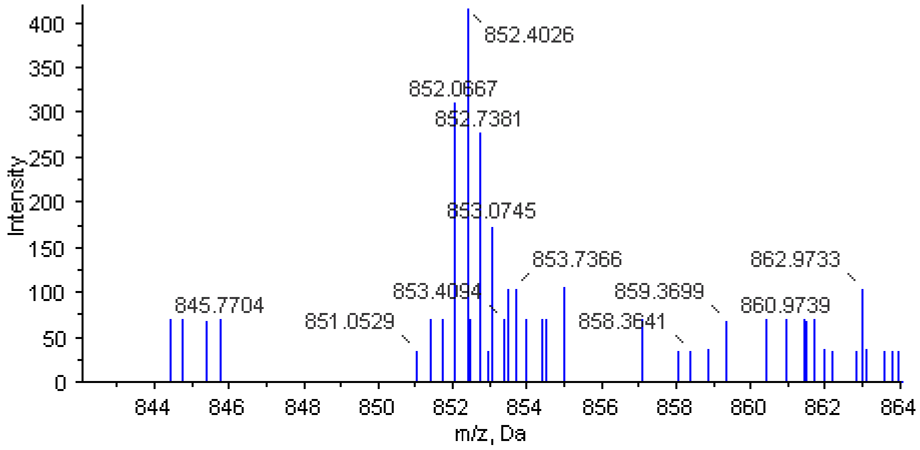

Sequence Coverage and Matched peptides: in different color

MKMWVFTVFSLLLSLLLGGSAEQCGSQAGGALCPGGLCCSQFGWCGSTNDYCGNGCQSQCGGASGGDLG
SIISSEKFDEMLKHRNDGGCPGKGFYTYSAFISAANAFPGFGTTGDADTRKREIAAFLGQTSHETTGGW
QTAPDGPYAWGYCFVKEQNPGSYCSPSSTYPCADGKQYYGRGPVQLSWNYNYGQCGKAIGVDLLNNPDL
VATDPVISFKTAIWFWMTAQSPKPSCHSVITGNWSPSGADSAAGRVPGYGVLTNIINGGLECGMGWKQQ
VEDRIGFYKRYCDLLGVGYGNNLDCYNQKSFANGLLDMVDSM

Protein No.: I-1247

Protein name and Species:

ADP-ribosylation factor, putative OS=Ricinus communis GN=RCOM_0060350 PE=3 SV=1

Accession: **tr|B9SYE9|B9SYE9_RICCO**

Unused ProtScore: **2**

Seq Cov %: **8.2**

ITRAQ Changed Ratio:

117: 115=E3TL: D3TL=0. 34

116: 114=E5TL: D5TL=0. 82

Annotated typical Precursor MS Region:

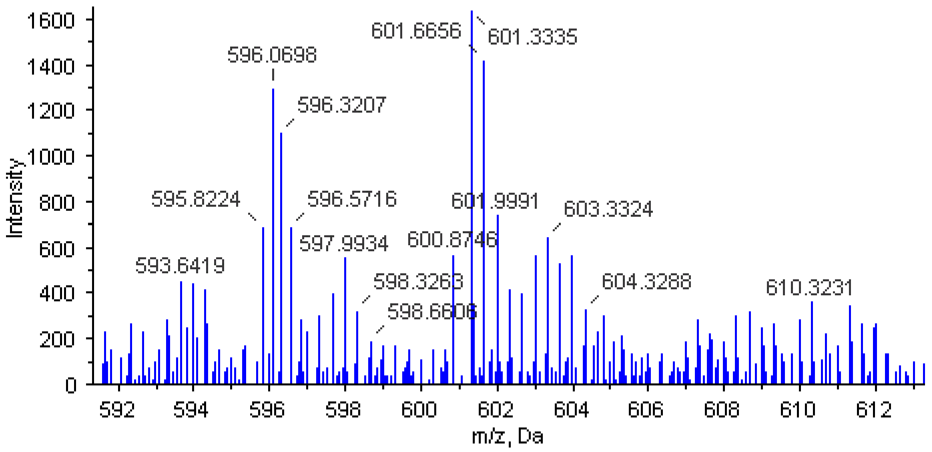

Sequence Coverage and Matched peptides: in different color

MGLLDSLLNWLRSLFFKQEMELSLVGLQNAGKTSLVNAVATGGYSEDMIPTVGFNMRKVTKGNVTIKLW
DLGGQRRFRTMWERYCRGVSAILYVVDAADRDSVPIARSELHDLVVKPSLSGIPLLVLGNKIDKSEALS
K**QALVDQLGLESITDR**EVCCYMISCKDSVNIDVVIDWLIKHSKTAK

Protein No.: I-1275

Protein name and Species:

V-type proton ATPase subunit F OS=Ricinus communis GN=RCOM_1381970 PE=3 SV=1

Accession: tr|B9S7V3|B9S7V3_RICCO

Unused ProtScore: 2

Seq Cov %: 9.2

ITRAQ Changed Ratio:

117: 115=E3TL: D3TL=0. 61

116: 114=E5TL: D5TL=1. 16

Annotated typical Precursor MS Region:

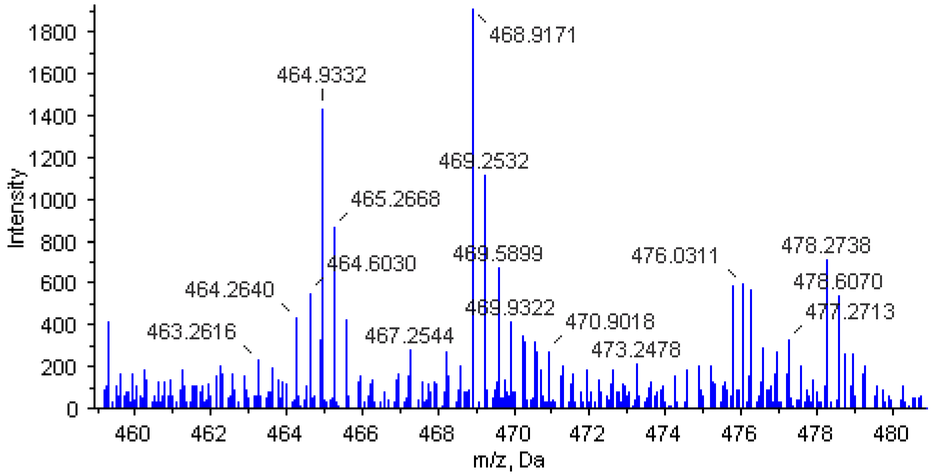

Sequence Coverage and Matched peptides: in different color

MANRNQIRTNNSALIAMIADEDTVVGFLLAGVGNVDLRRKTNYLLVDSKTTVKQIEDAFKDFTTREDIA
IVMISQYVANMIRFVVDSYNKPVPAILEIPSKDHPYDPSQDSVLSRVK**HLFSAESVASGR**R

Protein No.: I-1278

Protein name and Species:

nuclear transport factor 2 isoform 1 OS=Populus trichocarpa GN=POPTRDRAFT_548036 PE=2 SV=1

Accession: tr|A9PBT2|A9PBT2_POPTR

Unused ProtScore: 2

Seq Cov %: 27.6

ITRAQ Changed Ratio:

117: 115=E3TL: D3TL=0.39

116: 114=E5TL: D5TL=0.95

Annotated typical Precursor MS Region:

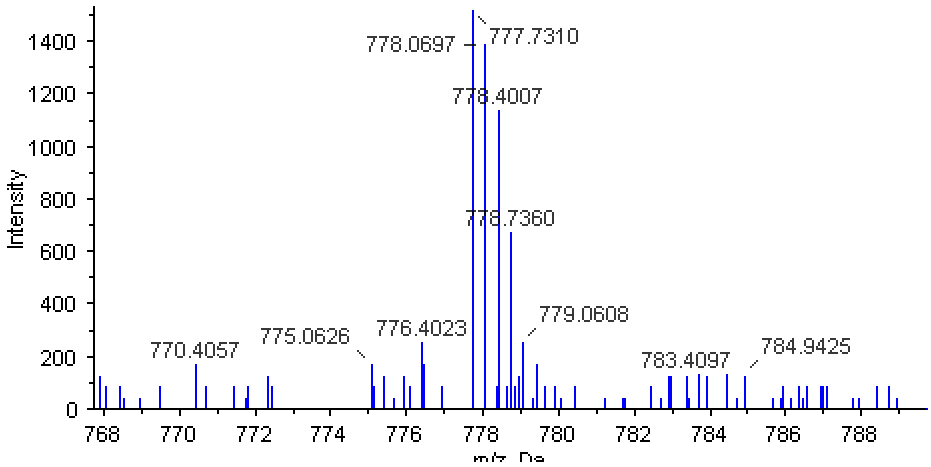

Sequence Coverage and Matched peptides: in different color

MDPDQVAKAFVEHYYSTFDANRAGLANLYQDGSMLTFEGQKTQGSQNIVAKLIALPFQQCKHLITTVDC
QPSGPAGGMLVFVSGNLQLAGEQHALKFSQMFHLMPTPQGSFYVFNDIFRLNYA

Protein No.: I-1281

Protein name and Species:

nuclear transport factor, putative OS=Populus trichocarpa PE=4 SV=1

Accession: **tr|A9PE14|A9PE14_POPTR**

Unused ProtScore: **2**

Seq Cov %: **29.8**

ITRAQ Changed Ratio:

117: 115=E3TL: D3TL=0. 51

116: 114=E5TL: D5TL=1. 43

Annotated typical Precursor MS Region:

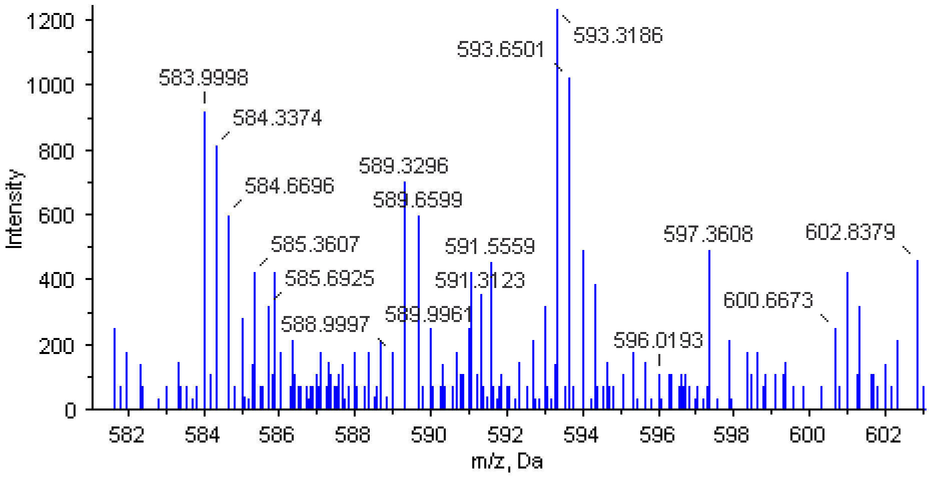

Sequence Coverage and Matched peptides: in different color

MDPDTVAK**AFVEHYY**NMFDSNR**AGLANLYQDASMLTFEGQK**TQGSQNIVAKLTALPFHQCKHHITTVDC
QPSGPAGGMLVF**VSGNLQLAGEQHALK**FSQGSYYVYNDIFRLNYA

Protein No.: I-1318

Protein name and Species:

Putative uncharacterized protein OS=Vitis vinifera GN=VIT_01s0011g00830 PE=4 SV=1

Accession: **tr|F6HF70|F6HF70_VITVI**

Unused ProtScore: **2**

Seq Cov %: **8.9**

ITRAQ Changed Ratio:

117: 115=E3TL: D3TL=0. 52

116: 114=E5TL: D5TL=0. 12

Annotated typical Precursor MS Region:

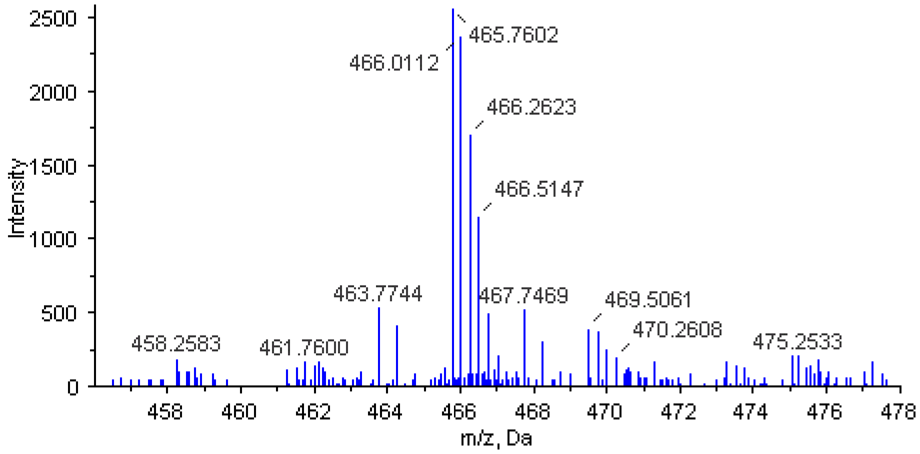

Sequence Coverage and Matched peptides: in different color

MMMVKQHGHDTVKEASPPHISTGEGGVKASSPLFDPSLCKTSSEFLSFQTSMDPTSHHRPGSHPQKHHQ
PSSSELLSSAKVVAEAAQASFHHESEKVDKSR**VAGAAADILGAASHYGK**LEEKSFGKYVEKAETYLHQY
QSSHSTTTTINSGHSTTNTTETHSSSHSGGGDSHSGGGYGDYLKMAEGFLKKY

Protein No.: I-1320

Protein name and Species:

heat shock factor-binding protein-like OS=Ricinus communis GN=RCOM_1439840 PE=4 SV=1

Accession: tr|B9T0W0|B9T0W0_RICCO

Unused ProtScore: 2

Seq Cov %: 16.7

ITRAQ Changed Ratio:

117: 115=E3TL: D3TL=2. 07

116: 114=E5TL: D5TL=1. 03

Annotated typical Precursor MS Region:

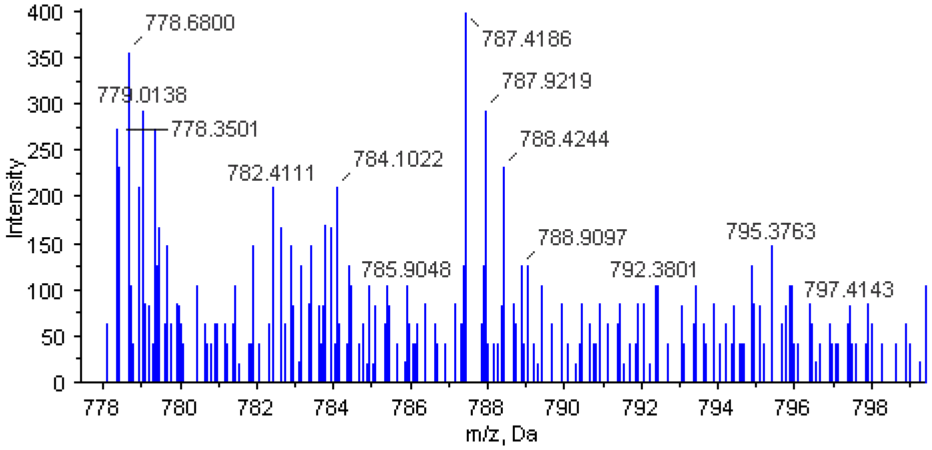

Sequence Coverage and Matched peptides: in different color

MDGHDSEDPKQSTADMTVFVQNLLQQMMGTR**INDLEQSINDLR**SEMGVEGSPSPLCQSKQKSGEENQDD
ALA

Protein No.: I-1336

Protein name and Species:

Calcium lipid binding protein, putative OS=Ricinus communis GN=RCOM_0491050 PE=4 SV=1

Accession: tr|B9SXV3|B9SXV3_RICCO

Unused ProtScore: 1.95

Seq Cov %: 11.3

ITRAQ Changed Ratio:

117: 115=E3TL: D3TL=1. 67

116: 114=E5TL: D5TL=4. 33

Annotated typical Precursor MS Region:

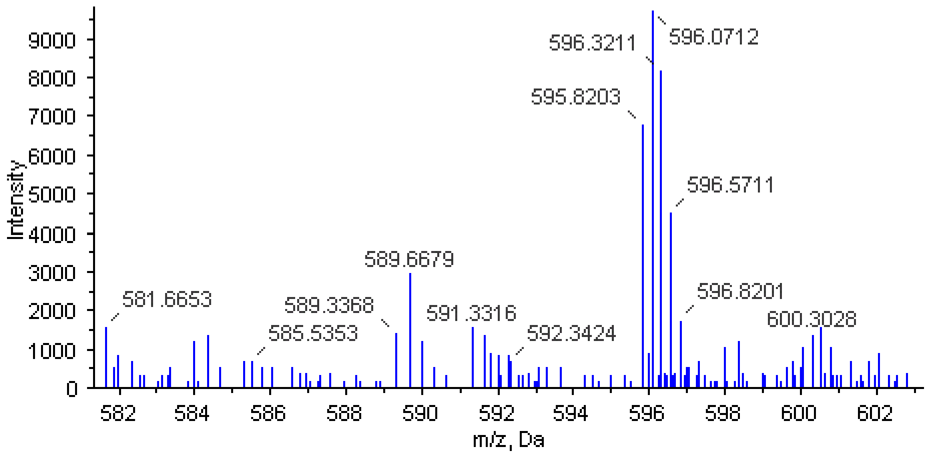

Sequence Coverage and Matched peptides: in different color

MGFFSTIFGFCGFGVGISMGLVIGYYLFIFFQPTDVKEPEIRPLVEEDSETLQRMLPEIPLWVKNPDYD
RIDWLNKFLEYMWPYLDKAICKTAKNIATPIIAEQIPKYKIDSVEFETLTLGTLPPTFSGMKVYVTDEK
ELILEPCLK**WAGNPNVTVAVK**AFGLK**ATAQVVDLQVFALPR**ITLKPLVPSFPCFATIYVSLMEKPHVDF
GLK**LSGADIMSIPGLYR**L**VQEIIK**DQVANMYLWPKTLEVQVMDPAKALKRPVGILNVKVVRAMKLKKK**D**
LLGASDPYVKIK**LTEDKLPSK**KTTVKHKNLNPEWNEEFNLVVKDPETQAVEFHVYDWEQVGKHDK**MGMN**
VVPLKELSPEEPKLTTLDLLKNMDLNDSQNEKSRGQWRLH**VEVVSTSSR**MGLLHPK**ESLGYVDISLADV**
VSNKRINEKFHLIDSKNGRIQIELQWRTSS

Protein No.: I-1337

Protein name and Species:

heat shock 70 kDa protein, mitochondrial-like [Vitis vinifera] OS=Vitis vinifera GN=VIT_00s0415g00030 PE=3 SV=1

Accession: tr|F6HCT7|F6HCT7_VITVI

Unused ProtScore: 1.94

Seq Cov %: 4.5

ITRAQ Changed Ratio:

117: 115=E3TL: D3TL=1. 96

116: 114=E5TL: D5TL=1. 54

Annotated typical Precursor MS Region:

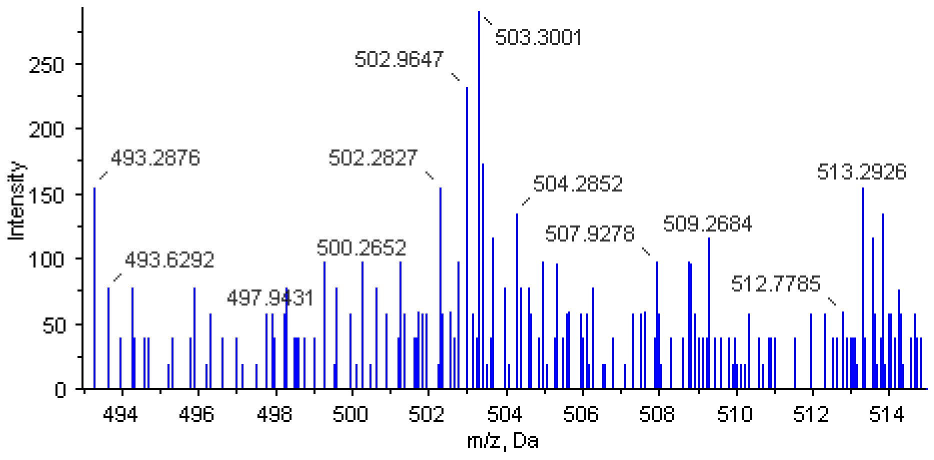

Sequence Coverage and Matched peptides: in different color

MATSVLLRCLKRRDLQSSSLAAYRSLTGNAKTSVSTSPAVQKWASYIRPFSSKPAGNDVIGIDLGTTNS
CVSVMEGKNAKVIENSEGARTTPSVVAINQKGELIVGTPAKRQAVTNPTNTVFGTKRLIGRRFDDPQTQ
KEMKMVPFKIVRAPNGDAWVEANGQQYSPSQIGAFVLTKMKETAEAYLGKTVSKAVITVPAYFNDAQRQ
ATKDAGRIAGLDVQRIINEPTAAALSYGLNNKEGLIAVFDLGGGTFDVSILEISNGVFEVKATNGDTFL
GGEDFDNALLEFLVSEFKRTEGIDLTKDRLALQRLREAAEKAKIELSSTSQTDINLPFITADASGAKHL
NITLTRSKFEALVDKLIERTRNPCKSCLKDAGISVKEVDEVLLVGGMTRVPK**VQEVVAEIFGK**SPSK**GV**
NPDEAVAMGAAIQGGILRGDVKELLLLDVTPLSLGIETLGGIFTRLINRNTTIPTKKSQVFSTAADNQT
QVGVKVLQGEREMATDNKLLGEFELVGIPPAPRGMPQIEVTFDIDANGIVTVSAKDKSTGKEQQITIRS
SGGLSDDEIEKMVKEAELHAQRDQERKALIDIRNSADTTIYSIEKSLDEYRDKIPAEVAKEIEAAVADL
RDAMQKENIDEIKAKLDAANKAVSKIGQHMSGGSGGAASGGSEGSTSSDQTPEAEYEQAKK

Protein No.: I-1341

Protein name and Species:

Predicted protein OS=Populus trichocarpa GN=POPTRDRAFT_1115857 PE=4 SV=1

Accession: tr|B9N560|B9N560_POPTR

Unused ProtScore: 1.92

Seq Cov %: 11.2

ITRAQ Changed Ratio:

117: 115=E3TL: D3TL=0. 62

116: 114=E5TL: D5TL=0. 58

Annotated typical Precursor MS Region:

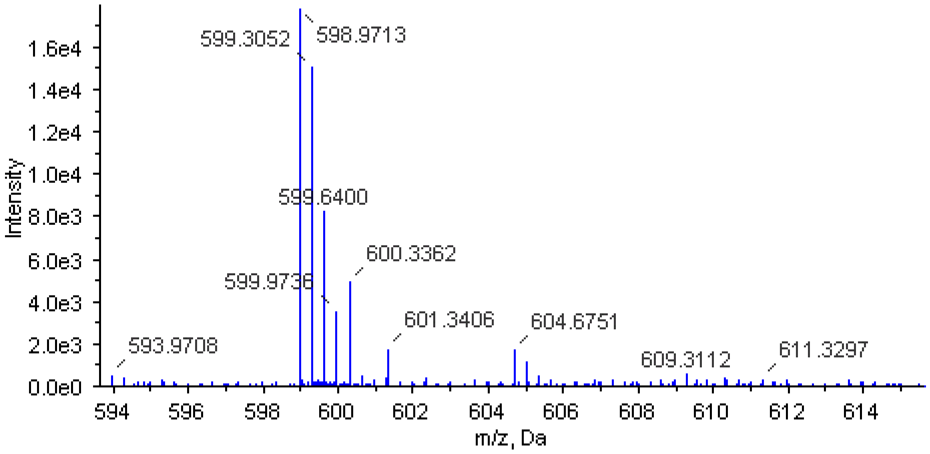

Sequence Coverage and Matched peptides: in different color

MNPQQQQQQQQQQQQNPVDPDQPLLPTIKIHHHPSPPRHPHPPSATPTLTPTTRRK**IGVAVDLSDESAY**
AVRWSVHHYIRPGDSVILLHVSPTSVLLGADWGPLPLSTPTQSQLDLLNNNSKFNSEIDSKTKNENSEK
PQPRQEDDFDAFTASKAADIARPLKEAQIPYKIHIVKDHDMKERLCLEIER**LGLSAVIMGSR**GFGAAIR
GSDERLGSVSDYCVHHCFCPVVVVRYPEDKDCGRD

Protein No.: I-1346

Protein name and Species:

Serine/threonine-protein kinase, putative OS=Ricinus communis GN=RCOM_1590280 PE=4 SV=1

Accession: tr|B9R7B8|B9R7B8_RICCO

Unused ProtScore: 1.91

Seq Cov %: 5.7

ITRAQ Changed Ratio:

117: 115=E3TL: D3TL=1. 71

116: 114=E5TL: D5TL=3. 28

Annotated typical Precursor MS Region:

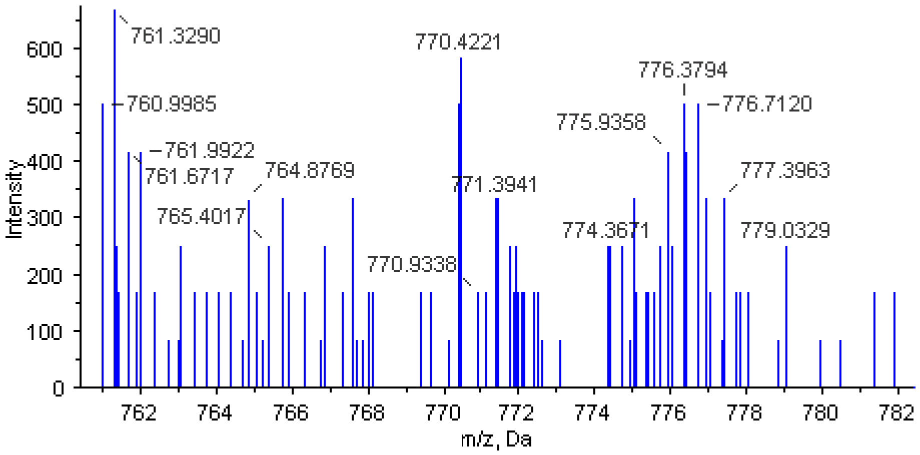

Sequence Coverage and Matched peptides: in different color

MDGGDGTVRLGALNLRPDR**SGFDSGPDVSVSSPVTR**QKAAAAKQFIENHYKNYLQGLQDRKDRRQALQR
RAQEARISNEEQEEMMRNLERRETEYMRLQRRKIGIDDFEQLTVIGKGAFGEVRLCRAKSTGEIFAMKK
LKKSEMLSRGQVEHVRSERNLLAEVDSRCIVKLFYSFQDSDFLYLIMEYLPGGDIMTLLMREDILSEDV
ARFYIAESILAIQSIHQHSYVHRDIKPDNLILDKNGHLK**LSDFGLCK**PLDDKYSSILLENEDISQEGTN
EAGGHSGSEAPWSMPKEVLQQWKRNRRALAYSTVGTLDYMAPEVLLKKGYGMECDWWSLGAILYEMLLG
YPPFCSDDPRITCRKIINWKTCLKFPDEPKISNEAKDLICHLLCDVETRLGTRGVDELKAHPWFRCTQW
DMLYEMEAAYKPVVNGDLDTQNFEK**FPDLEGPPSAIPR**VGPWRKMLTSKDTNFIGFTFKKSDVINSMES
SGTYAKSNASSKAPSLISLLGRIDLQETAELEGEHTLQS

Protein No.: I-1348

Protein name and Species:

Putative uncharacterized protein OS=Ricinus communis GN=RCOM_0632890 PE=4 SV=1

Accession: tr|B9S119|B9S119_RICCO

Unused ProtScore: 1.89

Seq Cov %: 10.3

ITRAQ Changed Ratio:

117: 115=E3TL: D3TL=0. 33

116: 114=E5TL: D5TL=1. 16

Annotated typical Precursor MS Region:

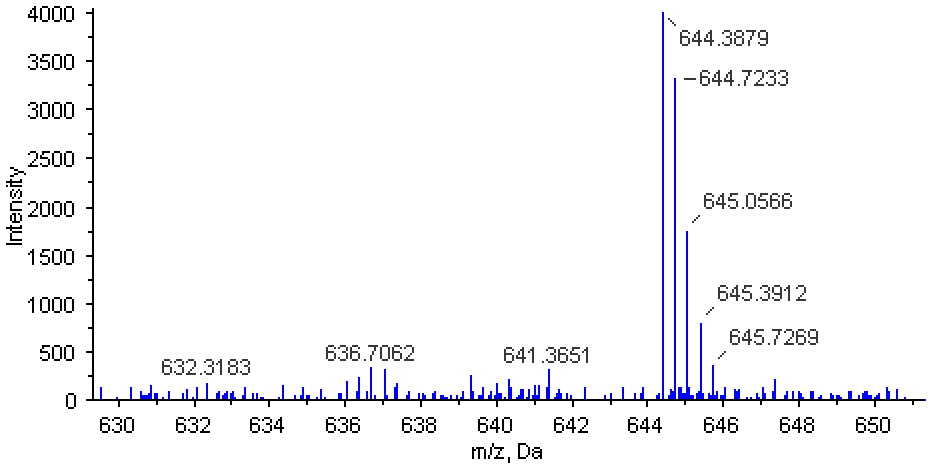

Sequence Coverage and Matched peptides: in different color

MASQRIANHRDEAEIYLGESLCKQKSIELLEEIRLPKGLLPLDDIVEVGYNRTTGFVWLKQKNSKNHRF
REIGR**NVNYDTEVTAFVEDR**RMKRLTGVKSKELLIWVTISDIYVDHNDNSK**ITFGNPSGISR**TFPVSAF
QLQEEKK

Protein No.: I-1353

Protein name and Species:

chitinase，class I (Fragment) OS=Hevea brasiliensis GN=rq30 PE=2 SV=1

Accession: tr|Q949H3|Q949H3_HEVBR

Unused ProtScore: 1.87

Seq Cov %: 7.1

ITRAQ Changed Ratio:

117: 115=E3TL: D3TL=0. 45

116: 114=E5TL: D5TL=0. 51

Annotated typical Precursor MS Region:

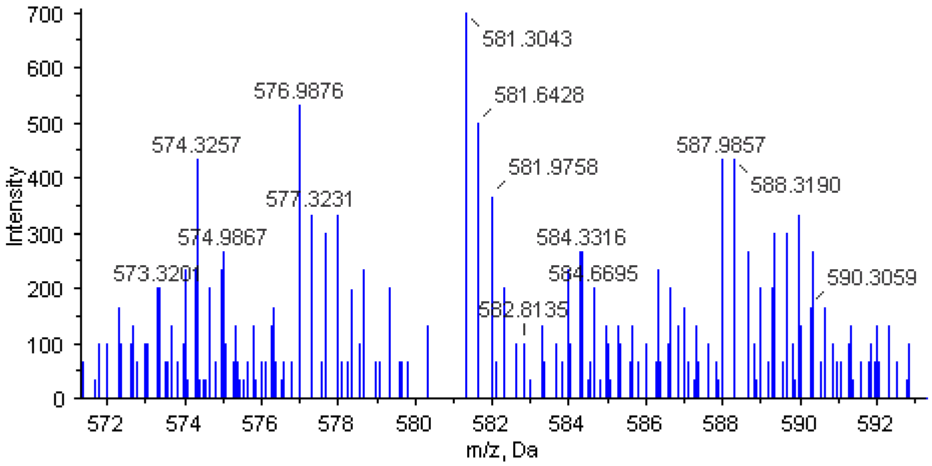

Sequence Coverage and Matched peptides: in different color

EQCGRQAGGALCPGGLCCSQYGWCANTPEYCGSGCQSQCDGGGGGEDGGIDLGSIISRSTFEEMLKHRN
DAACPAKGFYTYDAFISAAKAFPAFGTTGDVDTCKREIAAFFGQTSHATTGGWPTAPDGPYAWGYCYKE
ELNQASSYCSPSPAYPCAPGKKYYGRGPIQLSWNYNYGQCGQALGLDLLNNPDLVATDRVISFKAAIWF
WMTPQFPKPSCHDVITGQWSPTGHDISAGRAPGYGVITNIINGGLECGRGWDARVEDRIGFYKRYCDMF
AVGYGSNLDCYNQTPFGLG

Protein No.: I-1355

Protein name and Species:

GTP-binding protein OS=Populus trichocarpa GN=POPTRDRAFT_1073333 PE=3 SV=1

Accession: tr|B9H023|B9H023_POPTR

Unused ProtScore: 1.86

Seq Cov %: 22

ITRAQ Changed Ratio:

117: 115=E3TL: D3TL=2. 75

116: 114=E5TL: D5TL=0. 78

Annotated typical Precursor MS Region:

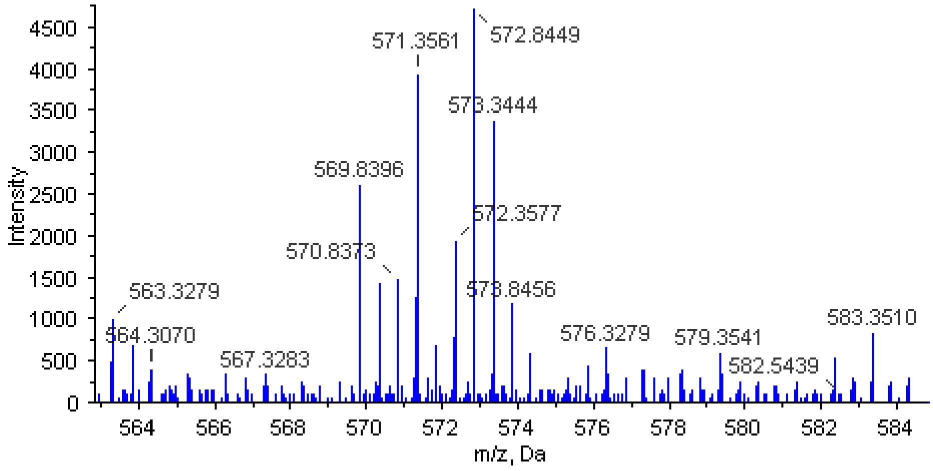

Sequence Coverage and Matched peptides: in different color

MSNSSENEVESLPQSIEEKHQELVVSHVPIVSSFNERIR**PLLDAVDK**LRHLQ**VMKEGIQLPTIVVVGDQ**
SSGKSSVLESLAGISLPRGQGICTRVPLIMRLQHHTAPEPEFSLEFNGKTVPTSEAK**IANAISLATDEI**
AGNAKGISNTPLTLVVKKNGVPDLTMVDLPGITRVPVHGQPENIYEQIADIIMEYIRPDESIILNVLSA
TVDFTTCESIRMSQKVDKNGERTLAVVTKADR**APEGLLEKVTADDVNIGLGYVCVR**NRIGDESYEEARK
EEADLFENHPLLSKIDK**SMVGIPVLAQKLVQIQATIIAR**CLPEIVRKINEK**LNASISELNR**MPKTLSSV
GEALTTFMSIVGSAKESLNKIIVR**GEYDEYLEDKNMHCTAR**LVEMLNQYSGELHNCSENDLTGNFLMDE
IQVLEEAKGIELPNFLPRTTFLSILQKKVEKVSHIPVAFVEKVWTYIEGVVISVLMHHSENYHQLQLST
RRAGHSLIARMKEHSRNWVTEIVQMEKLTDYTSNPEYMNDWNKLMAQQHDFTRNVLENGYVATFKIEGL
GVVPIAGLRGYEQHVLLQAFDLKMRMTAYWKIVLRRLVDFMALHLQF**CARNLVNK**EMEEEIVQELVGRH
DGAIERMLEESPAVAAKREKLNVSIKLLRESNN**VLGNIMDK**IASNI

Protein No.: I-1387

Protein name and Species:

ubiquitin 11 OS=Medicago truncatula PE=2 SV=1

Accession: tr|B7FLK9|B7FLK9_MEDTR

Unused ProtScore: 1.77

Seq Cov %: 77.3

ITRAQ Changed Ratio:

117: 115=E3TL: D3TL=0.24

116: 114=E5TL: D5TL=0.82

Annotated typical Precursor MS Region:

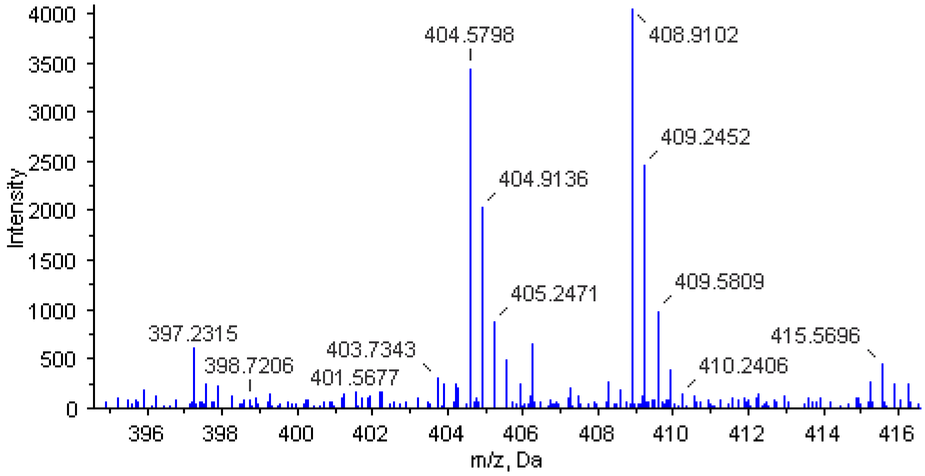

Sequence Coverage and Matched peptides: in different color

MQIFVKTLTGK**TITLEVESSDTIDNVK**AK**IQNKEGIPPDQQRLIFAGKQLEDGRTLADYNIQKESTLHL**
VLRLR**GGMQIFVKT**LTGK**TITLEVESSDTIDNVK**AK**IQDKEGIPPDQQRLIFAGKQLEDGRTLADYNIQ**
KESTLHLVLRLR**GGMQIFVKT**LTGK**TITLEVESSDTIDNVK**AK**IQDKEGIPPDQQRLIFAGKQLEDGRT**
LADYNIQKESTLHLVLRLRGGF

Protein No.: I-1404

Protein name and Species:

Conserved hypothetical protein [Ricinus communis] OS=Ricinus communis GN=RCOM_1445010 PE=4 SV=1

Accession: tr|B9RGW6|B9RGW6_RICCO

Unused ProtScore: 1.7

Seq Cov %: 7

ITRAQ Changed Ratio:

117: 115=E3TL: D3TL=2. 94

116: 114=E5TL: D5TL=3. 98

Annotated typical Precursor MS Region:

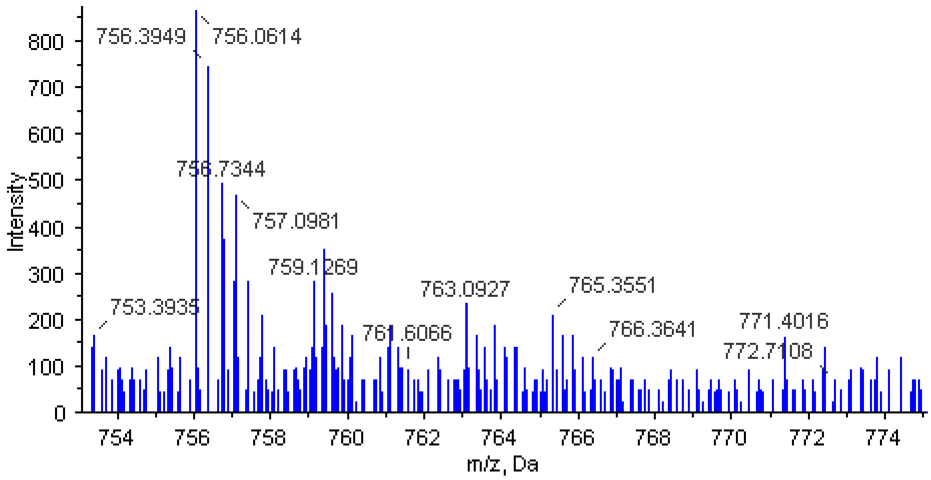

Sequence Coverage and Matched peptides: in different color

MDKNTTKKKEVIRLERESVIPILKPKLVMTLANLIEHTSDRAEFLKLCKRIEYTIRAWYLLQFEDLMQL
YSLFDPVSGAQKLQQQNLSPEEIDVLEQNFLTYLFQVMDKSNFKIATEEEIEVAHSGQYLLNLPISVDE
SKLDKEVLKKYFAAHPREDLPD**FVDK**YVIFRRGIGIDRTTDYFIMEKVDMLIARFWAYILRLTRVEKLL
RRRSSMRCKKDPKKNDEINSEADQNDLCVERIRLENMELSVRNLLSSTTIQEPTFDRIIVVYRRASSKL
KKERGIYVKHFKNIPMADMEIVLPEKKNPGLTPMDWVKFLGSAIVGLVALVSSLEMPKADLWVIFAVLS
AVIGYFAKTYFTFQANLAAYQNLITQSMYDKQLDSGK**GTLLHLCDDVIQQEVK**EVIISFFILMEQGKAT
MQDLDLR**CEELIQEEFGESCNFDVDDAVLK**LEK**LGIVAR**DTIGRYYCVGLKRANEIIGTTTEELVLKAK
QGARRRTKSEVKPAERSKTQEQKILMHHIRRLQGQRRKAMGSLCSIISYLCDFFGLNSNQIPGSAGVWS
CYG

Protein No.: I-1407

Protein name and Species:

Predicted protein OS=Populus trichocarpa GN=POPTRDRAFT_806336 PE=3 SV=1

Accession: tr|B9I2G0|B9I2G0_POPTR

Unused ProtScore: 1.69

Seq Cov %: 1.5

ITRAQ Changed Ratio:

117: 115=E3TL: D3TL=0. 59

116: 114=E5TL: D5TL=0. 56

Annotated typical Precursor MS Region:

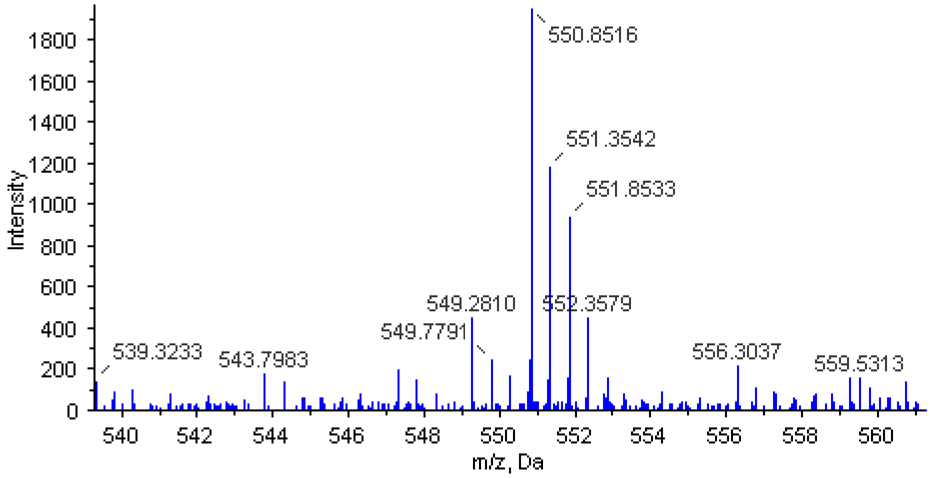

Sequence Coverage and Matched peptides: in different color

MKNFLKKLHIMPNQSQDAEGSNSSRGHKSSNESSSDNKFLHSRLQENKPFSGLSNWLSSVANRKKGARR
DSVSSTSRDPDVEEEFQIQLALELSAR**EDPEAVQIEAVK**QISLGSCAPEHTLAELIAYRYWNYNALSYD
DKVLDGFYDLYGIMTESTSDKMPSLVDLQATPVSGGVTWEAVLVNRAADANLLKLEKKALEIAVKSRSE
SQVFIGSALVRRLAVLVSDYMGGAVGDPSNLSRAWRSLSYSLKANLGSMVLPLGSLTIGLPRHRALMFK
VLADSVGIPCRLVKGHLYTGSDDVAMNFVKIDDGREYIVDLTADPGTLIPSDAAGSHIEYDDSFFSSSP
FSRDIDSYRIASSSSGHTSDSHEGASLTKLSKGEEESTISLNDFGKISIAEKVPVRELPGRPIYPSAHA
RSPSWTEGVSSPSVRRMKVKDVSQYMIDAAKENPQLAQKLHDVLLESGVVAPPNLFTEIYAEQLNASTA
EATSPTEGKDGHKQRTEIRYVKDQDDLVPARFFPLLPPNELPYKSSSPGNQPEQSKPSEFTPVKYVKNV
PVAAAAAAAAAVVASSMVVAAAKSSTDSNLELPVAAAATATAAAVMATTAAVNKQYVQGARSDGDADSA
GYEPHGSGDKGSGGRGKGERISDRLAVNVRSKSDAGLDDVAECEIPWEEITLGERIGLGSYGEVYRGDW
HGTEVAVKRFLDQDITGEALAEFRSEVRIMKRVRHPNVVLFMGAVTRAPNLSIVTEFIPRGSLYRLLHR
PNNQLDDRRRLRMALDAARGMNYLHSCTPMIVHRDLKSPNLLVDKNWVVK**VCDFGLSR**IKNSTFLSSRS
TAGTAEWMAPEVLRNEPSDEKCDVYSFGVILWELSTLQQPWGGMNPMQVVGAVGFQHRSLDIPNDMDPA
IADIIRKCWQTDPRLRPTFAEIMAALKLLQKPITGPQVPRPNAPLRSGH

Protein No.: I-1418

Protein name and Species:

aspartate-tRNA ligase, cytoplasmic-like OS=Populus trichocarpa GN=POPTRDRAFT_652970 PE=3 SV=1

Accession: tr|B9HBL6|B9HBL6_POPTR

Unused ProtScore: 1.65

Seq Cov %: 7.5

ITRAQ Changed Ratio:

117: 115=E3TL: D3TL=0. 10

116: 114=E5TL: D5TL=0. 21

Annotated typical Precursor MS Region:

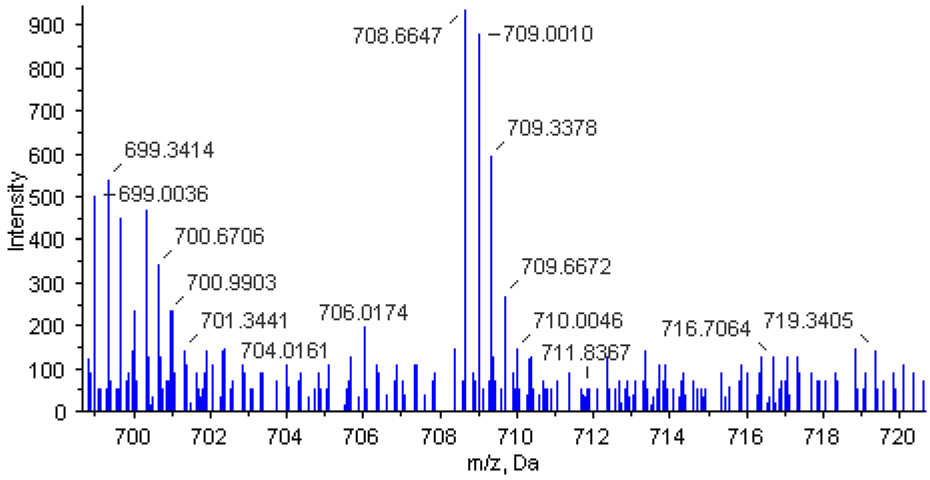

Sequence Coverage and Matched peptides: in different color

MNPQNQRAKKLQRRKQQSKRSYVAVRKPLWPAAAASSLSIEEDPLAANYGNIPLQDLQSKVEADLKAWT
QVGDLTHELKEKEVLIRGRAQTTRAVGKNMAFVVVREKGFTVQCVVTARPDVVSKQMVKFAAGLSRESI
VDIHGVVSVPSIAIKGTTQQVEIQVSKLYCVDK**AMPTLPINIEDASR**SEK**EIEEALEAGEQLVR**VNQDT
RLNYRVLDFRTSANQGIFRIQCQVCNIFRQFLLSEDFVEIHTPKLIAGSSEGGSAVFKLDYKGQAACLA
QSPQLHKQMAICGDFGRVFEIGPVFRAEDSYTHRHLCEFTGLDVEMEIKQHYTEVMDIVDHLFVTMFDH
LNKKCSKYLEAVGRQYPFEPLKYLPKTLRLRFEEGVQMLK**EAGVEIDPYGDLNTESER**K**LGQLVLEK**YG
TEFYILHRYPLAVRPFYTMPCYDDPKYSNSFDVFIRGEEIISGAQRVHVPEFLVERAQACGIDVSTIST
YIDSFRYGAPPHGGFGAGLERVVMLFCGLNNIRKTSLFPRDPLRIAP

Protein No.: I-1426

Protein name and Species:

random slug protein 5 isoform 2 [Vitis vinifera] OS=Vitis vinifera GN=VIT_18s0001g12420 PE=4 SV=1

Accession: **tr|F6H1I3|F6H1I3_VITVI**

Unused ProtScore: **1.61**

Seq Cov %: **8.3**

ITRAQ Changed Ratio:

117: 115=E3TL: D3TL=0. 17

116: 114=E5TL: D5TL=0. 93

Annotated typical Precursor MS Region:

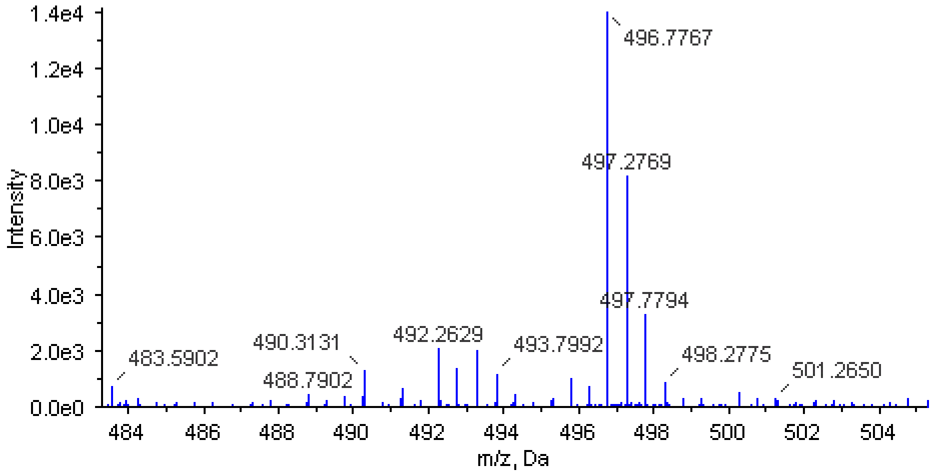

Sequence Coverage and Matched peptides: in different color

MISEEVSVFLDAHLSQQKYPTMLRLWGKSHHDQEKDLQKSDSKVHELK**AALGPLSGR**SLQYCNDACLKR
YLEARNWNVDKSKKMLEETLTWRSTYKPEEIRWSDVATEGETGKVFRANFHDRHGRTVLILRPGKQNTT
ALDNQVRHLVYLLENAILNLPEDQEQMVWLIDFTGMTFSNSVPIKTARDTIN**ILQNHYPER**LFLAFLYS
PPRIFEAFWKAVKYLLDAKTFQKVKFVYPKDKNSVELMSSYFDVENLPTDFGGKATMN**YDHEEFSR**LMT
QDDVKSANLWGFGDKLQHVSNGHSVAEVSPEPACLAPAS

Protein No.: I-1434

Protein name and Species:

HMG-CoA synthase 2 OS=Hevea brasiliensis GN=HMGS2 PE=2 SV=1

Accession: tr|Q6QLW8|Q6QLW8_HEVBR

Unused ProtScore: 1.58

Seq Cov %: 32.5

ITRAQ Changed Ratio:

117: 115=E3TL: D3TL=0. 58

116: 114=E5TL: D5TL=0. 73

Annotated typical Precursor MS Region:

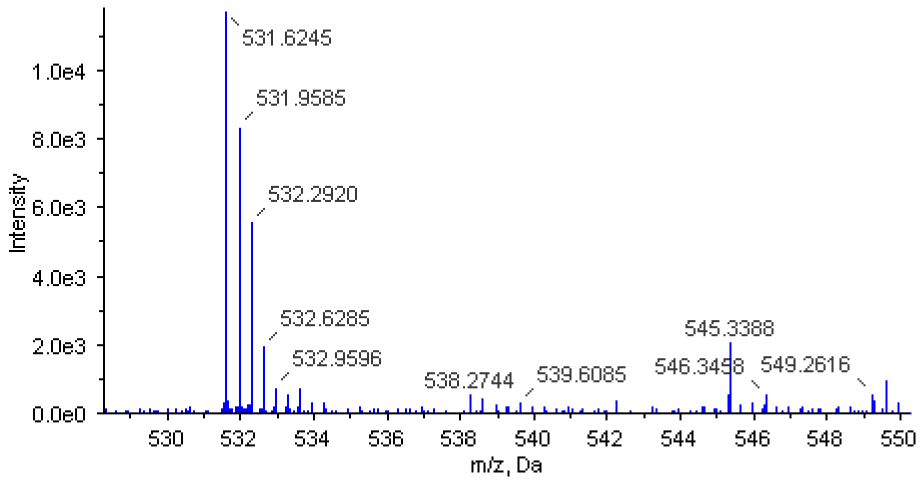

Sequence Coverage and Matched peptides: in different color

MAKNVGILAMDIYFPPTYVQQEALEAHDGASKGKYTIGLGQDCMAFCTEVEDVISMSLTAVTSLLEK**YN**
IDPNQIGRLEVGSETVIDKSKSIK**TFLMQIFEK**FGNTDIEGVDSANACYGGTAALFNCVNWVESNSWDG
R**YGLVVCTDSAVYAEGPAR**PTGGAAAIAMLIGPEAPIAFESKFRGSHMSHAYDFYK**PNLASEYPVVDGK**
LSQTCYLMALDSCYKHFCAKYEKLEGKQFSISDAEYFVFHSPYNKLVQKSFAR**LVFNDFVRNASSIDDA**
AKEK**LAPFSTLSGDESYQNR**DLEK**VSQQVAKPLYDAKVQPTTLIPK**QVGNMYTASLYAAFASLLHNKHT
ELAGKRVILFSYGSGLTATMFSLR**LHEGQHPFSLSNIATVMNVAGK**LKTRHEFPPEK**FAVIMK**LMEHRY
GAKDFVTSKDCSILAPGTYYLTEVDTMYRRFYAQK**AVGDTVENGLLANGH**

Protein No.: I-1437

Protein name and Species:

methyl transferase [Populus tomentosa] OS=Populus trichocarpa GN=POPTRDRAFT_1101018 PE=4 SV=1

Accession: **tr|B9IEB9|B9IEB9_POPTR**

Unused ProtScore: **1.57**

Seq Cov %: **5.2**

ITRAQ Changed Ratio:

117: 115=E3TL: D3TL=3. 73

116: 114=E5TL: D5TL=7. 78

Annotated typical Precursor MS Region:

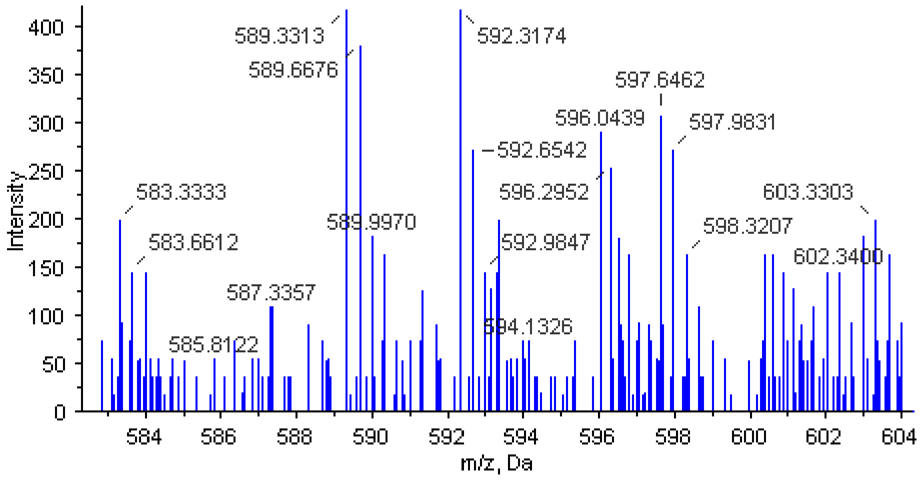

Sequence Coverage and Matched peptides: in different color

MDNISDLSVNAMMLDSKASELDKEERPEILSLLPPYEGKTVLELGAGIGRFTGELAQKAGQVVALDFIE
SAIKKNENINGHYKNVKFMCADVTSPDLNISEGSVDLIFSNWLLMYLSDKEVENLVERMVKWVKVDGFI
FFRESCFHQSGDSKRKYNPTHYREPRFYTKVFKECHTRDGSGDSFELSLVGCKCISAYVKNKKNQNQAM
KLDHLILWICWIWQKVSSYEDK**GFQRFLDNVQYK**SNGILRYERVFGQGYVSTGGIETTKEFVGKLDLKP
GQKVLDVGCGIGGGDFYMAENFDVEVVGIDLSINMISFALERAIGLKCSVEFEVADCTTK**TYPDNTFDV**
IYSRDTILHIQDKPALFRSFFKWLKPGGKVLISDYCKCDGTPSPEFAEYIKQRGYDLHDVKAYGQMLRD
AGFDEVVAEDRTDQFNKVLQRELNAIEKDKDEFIHDFSEGDYNDIVGGWKAKLIRSSSGEQRWGLFIAK
KK

Protein No.: I-1453

Protein name and Species:

chitinase Class Ib, OS=Limonium bicolor PE=2 SV=1

Accession: **tr|Q1W6C5|Q1W6C5_9CARY**

Unused ProtScore: **1.53**

Seq Cov %: **5.9**

ITRAQ Changed Ratio:

117: 115=E3TL: D3TL=0. 64

116: 114=E5TL: D5TL=0. 51

Annotated typical Precursor MS Region:

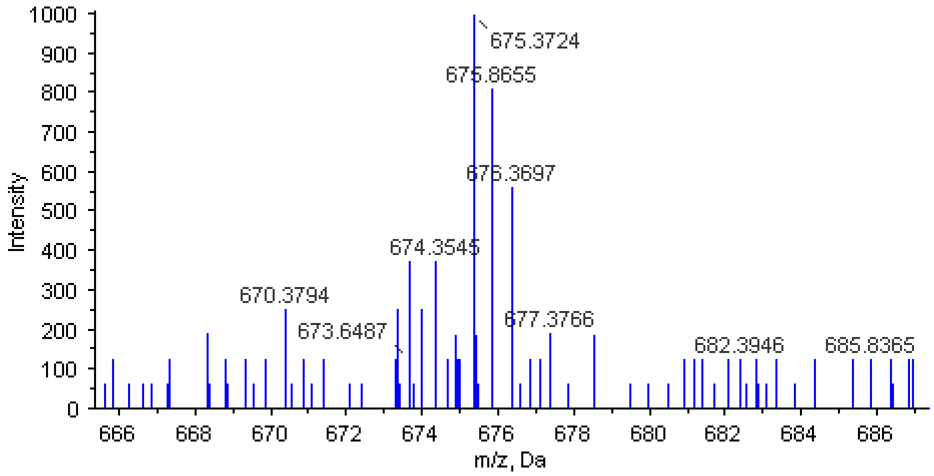

Sequence Coverage and Matched peptides: in different color

MKTTLILTAAALPFLLLISFSSAEQCGSQAGGAVCPNGLCCSKYGWCGSTDTYCKDGCQSQCGGTTPTP
TTPTPTPTQGGDVSSIISQSNFDEMLKHRNDNACSGHGFYTYAAFISAAQSFPGFGTTGDTDTRKRELA
AFLGQTSHETTGGWAAAPDGPYAWGYCFVQEQGASGAFCTSPEYPCAPGKQYYGRGPIQLTHNYNYAQA
GTAINQDLINNPDLVATDPTISFKTAIWFWMTPQSNKPSSHDVITGKWTPSSADSAAGR**VPGYGVITNI**
INGGLECGKGSIAKQEDRIGFYKRYCDVLGVGYGNNLDCNSQRPFA

Protein No.: I-1459

Protein name and Species:

Dynamin, putative OS=Ricinus communis GN=RCOM_0718390 PE=3 SV=1

Accession: tr|B9SBH8|B9SBH8_RICCO

Unused ProtScore: 1.51

Seq Cov %: 8.9

ITRAQ Changed Ratio:

117: 115=E3TL: D3TL=0.60

116: 114=E5TL: D5TL=0. 56

Annotated typical Precursor MS Region:

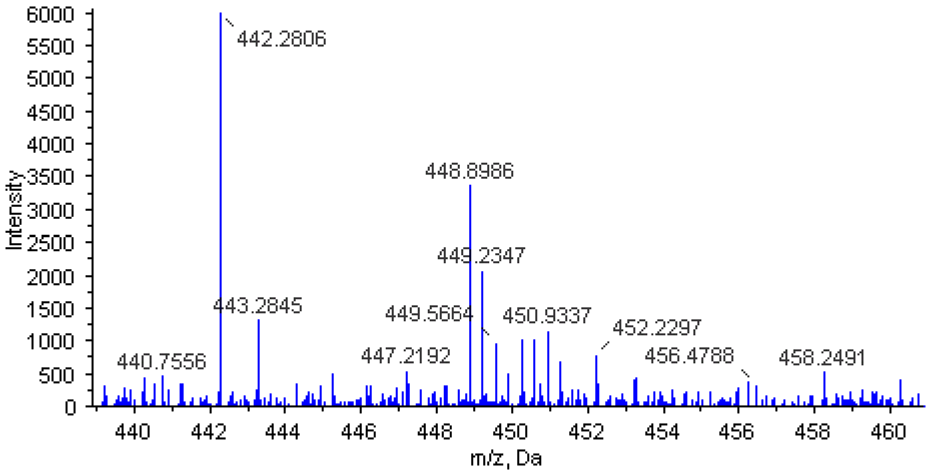

Sequence Coverage and Matched peptides: in different color

MENLISLVNKIQRACTALGDHGEESALPTLWDSLPSIAVVGGQSSGKSSVLESIVGKDFLPRGAGIVTR
RPLVLQLHRVDEGKEYAEFMHLPRKKFSDFAAVRKEISDETDRETGRSKQISTVPIHLSIYSPNVVNLT
LIDLPGLTKVAVDGQPESIVLDIENMIRSYIEKPNCIILAISPANQDLATSDAIKISREVDPKGERTFG
VLTKVDLMDK**GTDAVDILEGK**SYKLQFPWIGVVNRSQADINKSVDMIAARRKEREYFQNSTEYRHLAHR
MGSEHLGKMLSKHLEQVIKSR**IPGLQSLINK**TIAELEGELSRLGKPVATDAGGKLYMIMEICRSFDQIF
KEHLDGIRPGGDKIYLVFDNQLPAALKRLQFDKHLSIDNVRK**LITEADGYQPHLIAPEQGYR**RLIESTL
VTIRGPAEAAVDAVHVLLKELVQKSINETLELKQYPTLR**VEVSNAAIESLDR**MREESKKATLQLVDMEC
CYLTVEFFRKLPQDIEK**GGNPTHSIFDR**YNDSYLRRVGSNVLSYVNMVCGSLRNSIPKSVVYCQVRDAK
RSLLDYFFAELGKKETKQLSSLLDEDPAVMQRRISLAKRLELYRSAQAEIDAVAWAK

Protein No.: I-1464

Protein name and Species:

Alanine aminotransferase 1 OS=Glycine max GN=AlaAT1 PE=2 SV=1

Accession: **tr|A8IKE1|A8IKE1_SOYBN**

Unused ProtScore: **1.5**

Seq Cov %: **7.2**

ITRAQ Changed Ratio:

117: 115=E3TL: D3TL=0. 60

116: 114=E5TL: D5TL=0. 94

Annotated typical Precursor MS Region:

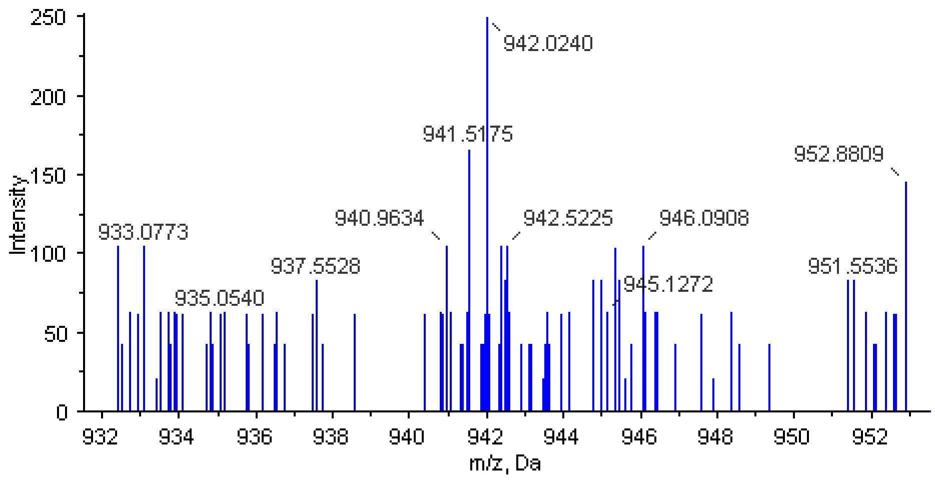

Sequence Coverage and Matched peptides: in different color

MASDSPFPVTAQNINPKVLKCEYAVRGEVVTLAQNLQKDLQANPGSHPFDEILYCNIGNPQSLGQQPIT
FFR**EVLALCDHPAILDKSETQGLFSTDAIQR**AWQIVDQIPGRATGAYSHSQGVKGLRDTIAAGIEERDG
FPANPDDIFMTDGASPAVHNMMQLLIRSENDGILCPIPQYPLYSASIALHGGCLVPYYLDEATGWGLEI
PELKKQLEAAKSKGINVRALVVINPGNPTGQVLGEANQRDIVEFCK**QEGLVLLADEVYQENVYVPEK**KF
HSFKKVSRSMGYGENDITLVSFQSVSKGYHGECGKR**GGYMEVTGFSAEVR**EQIYKVASVNLCSNISGQI
LASLVMSPPKVGDESYDSFMAEKENILASLARRAK**TLEDAFNKLEGVTCNK**AEGAMYLFPQIRLSEKAI
KAAEAANATPDNFYCKRLLNATGVVVVPGSGFGQVPGTWHFRCTILPPEEKIPAIVTRLTEFHEKFMDE
FRD

Protein No.: I-1467

Protein name and Species:

L-lactate dehydrogenase OS=Arabidopsis thaliana PE=2 SV=1

Accession: tr|Q8LBD5|Q8LBD5_ARATH

Unused ProtScore: 1.49

Seq Cov %: 20.7

ITRAQ Changed Ratio:

117: 115=E3TL: D3TL=0. 52

116: 114=E5TL: D5TL=0. 24

Annotated typical Precursor MS Region:

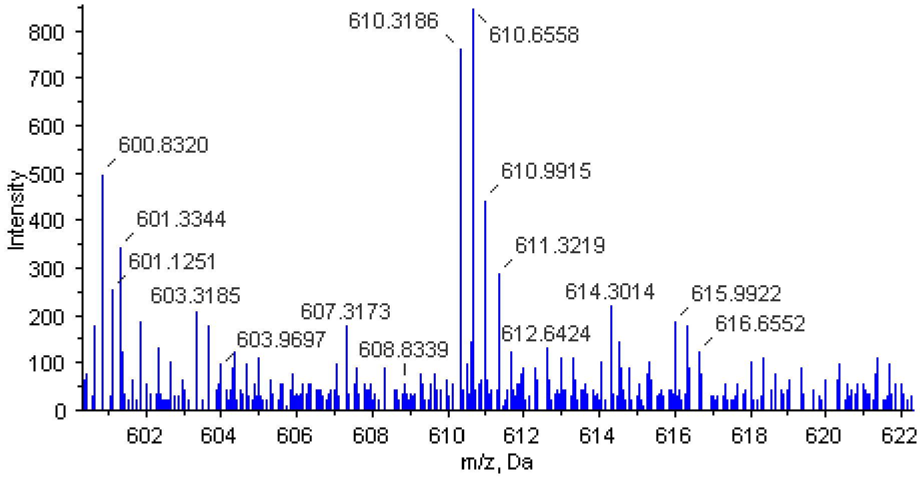

Sequence Coverage and Matched peptides: in different color

MEKNASTSSLKDLGPSGLDLTSAFFKPIHNSDPSLPSNRRTKVSVVGVGNVGMAIAQ**TILTQDLADEIA**
LVDAKPDKLR**GEMLDLQHAAAFLPR**TK**ITASVDYEVTAGSDLCIVTAGAR**QNPGESR**LNLLQR**NVALFR
HIIPPLAKASPDSILIIVSNPVDVLTYVAWKLSGFPVNR**VLGSGTNLDSSR**FRFLIADHLDVNAQDVQA
FIVGEHGDSSVALWSSISVGGIPVLSFLEKNQIAYEKQTLEDIHQAVVGSAYEVIGLKGYTSWAIGYSV
ANLARTILRDQRKIHPVTVLARGFYGVDGGDVFLSLPALLGRNGVVAVTNVHMTDEEAEKLQKSAKTIL
EMXSQLGL

Protein No.: I-1479

Protein name and Species:

S-formylglutathione hydrolase-like [Glycine max] OS=Glycine max PE=2 SV=1

Accession: tr|C6TB50|C6TB50_SOYBN

Unused ProtScore: 1.43

Seq Cov %: 14.7

ITRAQ Changed Ratio:

117: 115=E3TL: D3TL=0.64

116: 114=E5TL: D5TL=0. 79

Annotated typical Precursor MS Region:

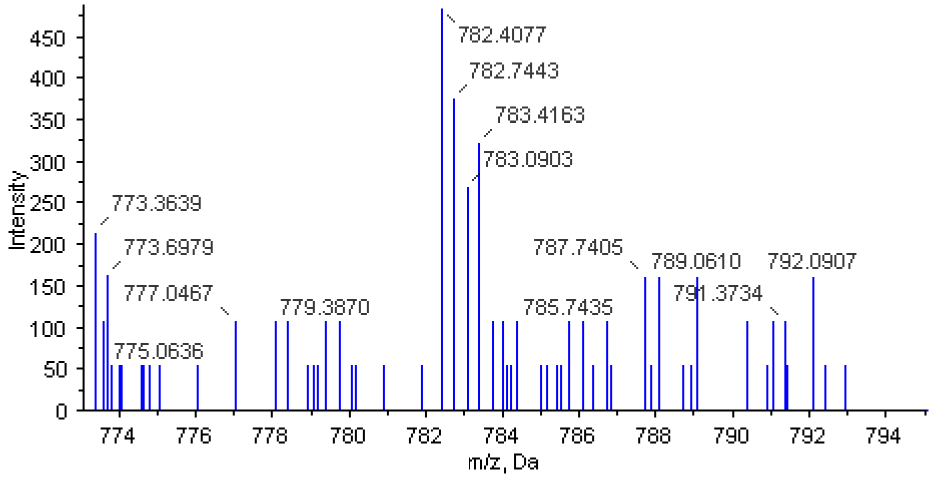

Sequence Coverage and Matched peptides: in different color

MDTRPTEISSGK**MFGGYNK**RFKHFSLTLGCSMNFHIYFPPSPSPSHKFPVLYFLSGLTCTDENFIFKSG
AQR**AASAEGVALVAPDTSPR**GLNVEGEADSWDLGVGAGFYLNATQEKWKNWRMYDYVVKELPKLLSDNF
PQLEISK**ASIFGH**SMGGHGA**LTIFLK**NQDKYK**SVSAFAPIANPINCPWGQK**AFSNYLGDNKFEWEDYDA
TRLVTKFPNVSSTILIDQGEDDKFLPDQLLPHKFEEACKKANVPLLLRFQPGYDHLYYFIATFIDDHIR
HHAQAFRLN

Protein No.: I-1487

Protein name and Species:

Aminotransferase YbdL-like OS=Vitis vinifera GN=VIT_18s0001g03300 PE=4 SV=1

Accession: tr|E0CR92|E0CR92_VITVI

Unused ProtScore: 1.4

Seq Cov %: 5.1

ITRAQ Changed Ratio:

117: 115=E3TL: D3TL=2. 25

116: 114=E5TL: D5TL=1. 82

Annotated typical Precursor MS Region:

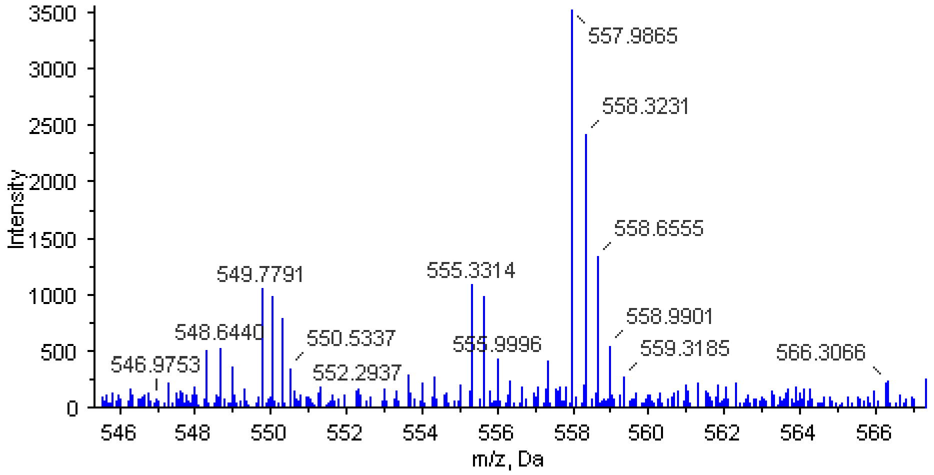

Sequence Coverage and Matched peptides: in different color

MASASTVSAPNTEAEQTHNPPQPLQVAKRLEKFKTTIFTQMSMLAIKHGAINLGQGFPNFDGPEFVKEA
AIQAIKDGKNQYARGYGVPDLNSAVADRFKKDTGLVVDPEKEVTVTSGCTEAIAATMLGLINPGDEVIL
FAPFYDSYEATLSMAGAQIKSITLRPPDFAVPMDELKSAISKNTR**AILINTPHNPTGK**MFTREELNVIA
SLCIENDVLVFTDEVYDKLAFEMDHISMASLPGMYERTVTMNSLGKTFSLTGWKIGWTVAPPHLTWGVR
QAHSFLTFATCTPMQWAAATALR**APDSYYEELK**RDYSAKK**AILVEGLK**AVGFRVYPSSGTYFVVVDHTP
FGLKDDIAFC**EYLIK**EVGVVAIPTSVFYLHPEDGKNLVRFTFCKDEGTLRAAVERMKEKLKPKQ

Protein No.: I-1494

Protein name and Species:

Putative uncharacterized protein OS=Vitis vinifera GN=VIT_19s0014g01520 PE=4 SV=1

Accession: tr|F6H2P8|F6H2P8_VITVI

Unused ProtScore: 1.39

Seq Cov %: 6.5

ITRAQ Changed Ratio:

117: 115=E3TL: D3TL=0. 13

116: 114=E5TL: D5TL=0. 29

Annotated typical Precursor MS Region:

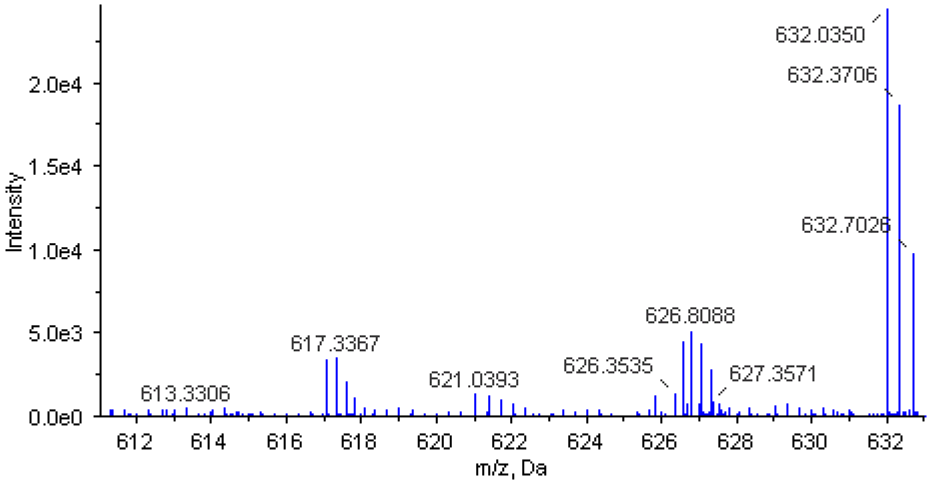

Sequence Coverage and Matched peptides: in different color

MALRHLTPLSPLSPFTRIPPRRCFTQKPFSLSVSASMGSSSRKVLVPIAHGSEPMEAVII**IDVLR**R**AGA**
DVTVASVEKRLQVDACHGVKIVADALISDCADTGFDLISLPGGMPGAATLRDCGMLESMVKKHAADGQL
YAGICAAPAVALGSWGLMKGLKATCYPSFMEQLSSTATTVESRVQQDGKVVTSRGPGTTMEFSVSLVEQ
LYGKEKANEVSGPLVMCSNLGDKFIMAELNPIDWKCDNPQILVPIANGTEEMEAVIIIDFLRRAK**ANVV**
VASVEDKLEIVASRKVKLVADVLLDEAVK**LSYDLIVLPGGLGGAQ**AFASSEKLVNLLKNQRESNKPYGA
ICASPALVLEPHGLLKGKKATAFPALCSKLSDQSEIENRVLVDGNLITSRGPGTSMEFALAIIEKFFGH
GK**ALELAK**VMLFSSQ

Protein No.: I-1523

Protein name and Species:

succinyl-CoA ligase [ADP-forming] subunit alpha-1, mitochondrial-like [Glycine max] OS=Glycine max PE=2 SV=1

Accession: tr|C6TNV4|C6TNV4_SOYBN

Unused ProtScore: 1.31

Seq Cov %: 3.1

ITRAQ Changed Ratio:

117: 115=E3TL: D3TL=2.11

116: 114=E5TL: D5TL=1.52

Annotated typical Precursor MS Region:

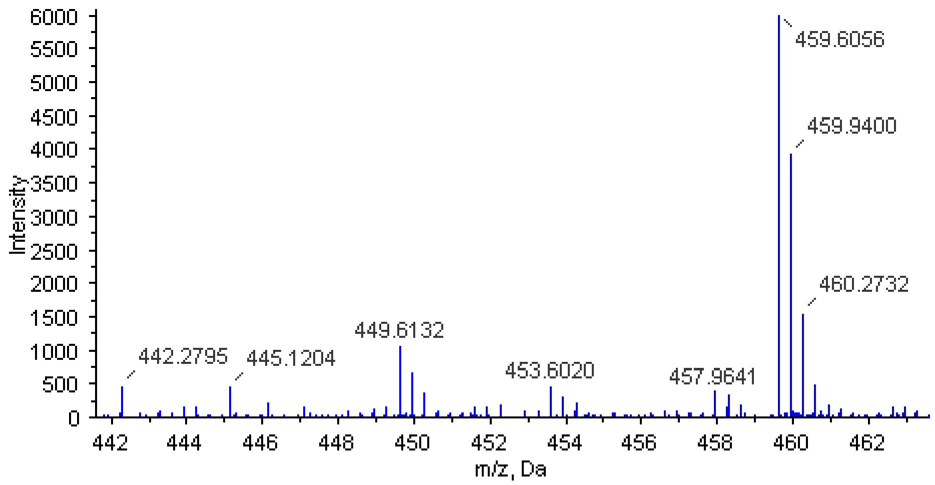

Sequence Coverage and Matched peptides: in different color

MGRQAVTKLLSSIASRRHGLRLFSAASPAV**FVDK**STRVICQGITGKNGTFHTEQAIEYGTNMVGGVTPK
KGGTEHLGLPVFNTVAEAKAETKANASVIYVPPPFAAAAIMEAMEAELDLVVCITEGIPQHDMVRVKAA
LNRQSKTRLIGPNCPGIIKPGECKIGIMPGYIHKPGRIGIVSRSGTLTYEAVFQTTAVGLGQSTCVGIG
GDPFNGTNFVDCLQKFLTDPQTEGIILIGEIGGTAEEDAAALIKDSGTEKPVVAFIAGLTAPPGRRMGH
AGAIVSGGKGTAQDKIKTLREAGVMVVESPAK**IGAAMLDVFK**QRGLVQ

Supplementary Figure S5:

Supplemental spectra and MALDI TOF/TOF MS /MS identification information

Annotated spectra for the 59 phosphorlated latex proteins on identified by TOF, TOF/TOF and LC-MALDI MS/MS.

CID: collision induced desorption;

MALDI TOF:

matrix assisted laser desorption/ionization time of flight;

MS: mass spectrometry;

PMF: peptide mass fingerprinting;

PFF: peptide fragment fingerprinting.

Spot No.: **P1**

NCBI accession No.: **gi|123650**

Species: *Petunia x hybrida*

Protein name: **Heat shock cognate 70 kDa protein**

Mascot score: **97**

Sequence coverage %: **40**

Matched peptides No.: **24**

Total peptides No.: **41**

Calculated Mr: **71581**

Calculated *p*I: **5.11**

Probability Based Mowse Score:

Protein score is -10*Log(P), where P is the probability that the observed match is a random event. Protein scores greater than 73 are significant (p<0.05).

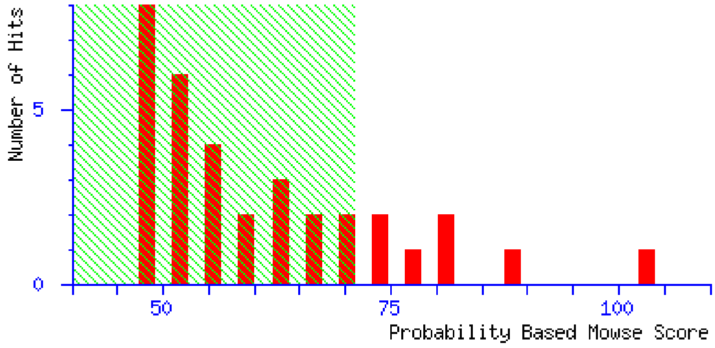

Matched peptide sequences: shown in Bold Red:

1 EGPAIGIDLG TTYSCVGVWQ HDRVEIIAND QGNRTTPSYV GFTDTERLIG
51 DAAKNQVAMN PINTVFDAKR LIGRRFSDPS VQSDIKLWPF KVIPGPGDKP
101 MIVVTYKGEE KQFAAEEISS MVLTKMKEIA EAYLGTTIKN AVVTVPAYFN
151 DSQRQATKDA GVIAGLNVMR IINEPTAAAI AYGLDKKASS AGEKNVLIFD
201 LGGGTFDVSL LTIEEGIFEV KATAGDTHLG GEDFDNRMVN HFVQEFKRKN
251 KKDISGNPRA LRRLRTACER AKRTLSSTAQ TTIEIDSLYE GIDFYSTITR
301 ARFEELNMDL FRKCMEPVEK CLRDAKMDKS SVHDVVLVGG STRIPKVQQL
351 LQDFFNGKEL CKSINPDEAV AYGAAVQAAI LSGEGNEKVQ DLLLLDVTPL
401 SLGLETAGGG MTVLIPRNTT IPTKKEQVFS TYSDNQPGVL IQVYEGERAR
451 TKDNNLLGKF ELSGIPPAPR GVPQITVCFD IDANGILNVS AEDKTTGQKN
501 KITITNDKGR LSKEEIERMV QEAEKYKSED EELKKKVEAK NALENYAYNM
551 RNTIKDDKIN SQLSAADKKR IEDAIDEAIK WLDNNQLAEA DEFEDKMKEL
601 ESICNPIIAK MYQGGAGGAT MDEDGPSVGG SAGSQTGAGP KIEEVD

Spot No.: **P2**

NCBI accession No.: **gi| 249262** Species: *Manihot esculenta*

PFF score: [54]

Protein name: linamarase

Matched peptides No.: [1] Sequence coverage %: [3]

Matched sequences: R.ILDGSNGDVAVDFYNR.Y

Calculated Mr: **61448** Calculated *p*I: **5.52**

Probability Based Mowse Score:

Ions score is -10*Log(P), where P is the probability that the observed match is a random event.
Individual ions scores > 45 indicate identity or extensive homology (p<0.05).
Protein scores are derived from ions scores as a non-probabilistic basis for ranking protein hits.

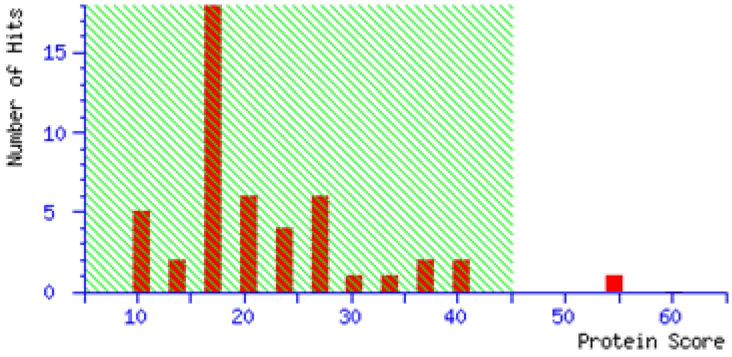

Matched peptide sequences: shown in Bold Red:

```
  1 MLVLFISLLA LTRPAMGTDD DDDNIPDDFS RKYFPDDFIF GTATSAYQIE
 51 GEATAKGRAP SVWDIFSKET PDRILDGSNG DVAVDFYNRY IQDIKNVKKM
101 GFNAFRMSIS WSRVIPSGRR REGVNEEGIQ FYNDVINEII SNGLEPFVTI
151 FHWDTPQALQ DKYGGFLSRD IVYDYLQYAD LLFERFGDRV KPWMTFNEPS
201 AYVGFAHDDG VFAPGRCSSW VNRQCLAGDS ATEPYIVAHN LLLSHAAAVH
251 QYRKYYQGTQ KGKIGITLFT FWYEPLSDSK VDVQAAKTAL DFMFGLWMDP
301 MTYGRYPRTM VDLAGDKLIG FTDEESQLLR GSYDFVGLQY YTAYYAEPIP
351 PVDPKFRRYK TDSGVNATPY DLNGNLIGPQ AYSSWFYIFP KGIRHFLNYT
401 KDTYNDPVIY VTENGVDNYN NESQPIEEAL QDDFRISYYK KHMWNALGSL
451 KNYGVKLKGY FAWSYLDNFE WNIGYTSRFG LYYVDYKNNL TRYPKKSAHW
501 FTKFLNISVN ANNIYELTSK DSRKVGKFYV M
```

Spot No.: **P3**

NCBI accession No.: **gi| 224100473** Species: *Populus trichocarpa*

PFF score: [112]

Protein name: predicted protein

Matched peptides No.: [1] Sequence coverage %: [3]

Matched sequences: R.SPPIEEVIQAGVVPR.F

Calculated Mr: **46093** Calculated *p*I: **4.54**

Probability Based Mowse Score:

Ions score is -10*Log(P), where P is the probability that the observed match is a random event.
Individual ions scores > 46 indicate identity or extensive homology (p<0.05).
Protein scores are derived from ions scores as a non-probabilistic basis for ranking protein hits.

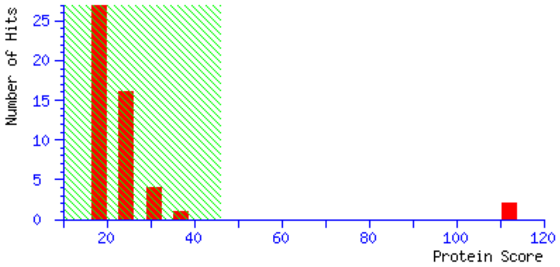

Matched peptide sequences: shown in Bold Red:

1 GVWSEDGNLQ LESTTQFRKL LSIER**SPPIE** **EVIQAGVVPR** FVEFLVREDF
51 PQLQFEAAWA LTNIASGTSE NTKVVIDHGA VPIFVKLLGS PSDDVREQAV
101 WALGNVAGDS PKCRDLVLSH GALIPLLAQL NEHAKLSMLR NATWTLSNFC
151 RGKPQPPFEQ VRPALPALER LVHSTDEEVL TDSCWALSYL SDGTDDKIQA
201 VIEAGVCPRL VELLLHPSPS VLVPALRTVG NIVTGDDMQT QVKALLAFCL
251 FVPSFAVIEN GLIGPLVNLL QNAEFDIKKE AAWAVSNATS GGTHEQIKFL
301 VSQGCIKPLC DLLVSPDPRI VTVSLEGLEN ILKVGEAEKN LGNSGDANFY
351 AQMIDDAEGL EKIENLQSHD NNEIYEKAVK ILETYWLEED DETLPSGDGA
401 QQGFQFGGNG VQVPSGGFNF

Spot No.: **P4**

NCBI accession No.: **gi| 249262** Species: *Manihot esculenta*

PFF score: [56]

Protein name: linamarase

Matched peptides No.: [1] Sequence coverage %: [3]

Matched sequences: R.ILDGSNGDVAVDFYNR.Y;

Calculated Mr: **61448** Calculated *p*I: **5.52**

Probability Based Mowse Score:

Ions score is -10*Log(P), where P is the probability that the observed match is a random event.
Individual ions scores > 45 indicate identity or extensive homology (p<0.05).
Protein scores are derived from ions scores as a non-probabilistic basis for ranking protein hits.

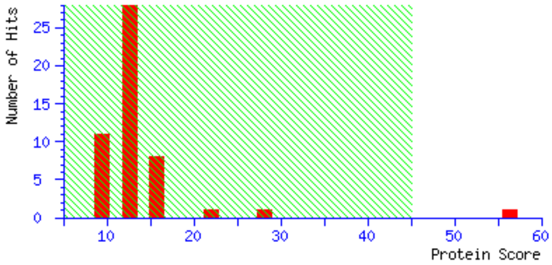

Matched peptide sequences: shown in Bold Red:

```
  1 MLVLFISLLA LTRPAMGTDD DDDNIPDDFS RKYFPDDFIF GTATSAYQIE
 51 GEATAKGRAP SVWDIFSKET PDRILDGSNG DVAVDFYNRY IQDIKNVKKM
101 GFNAFRMSIS WSRVIPSGRR REGVNEEGIQ FYNDVINEII SNGLEPFVTI
151 FHWDTPQALQ DKYGGFLSRD IVYDYLQYAD LLFERFGDRV KPWMTFNEPS
201 AYVGFAHDDG VFAPGRCSSW VNRQCLAGDS ATEPYIVAHN LLLSHAAAVH
251 QYRKYYQGTQ KGKIGITLFT FWYEPLSDSK VDVQAAKTAL DFMFGLWMDP
301 MTYGRYPRTM VDLAGDKLIG FTDEESQLLR GSYDFVGLQY YTAYYAEPIP
351 PVDPKFRRYK TDSGVNATPY DLNGNLIGPQ AYSSWFYIFP KGIRHFLNYT
401 KDTYNDPVIY VTENGVDNYN NESQPIEEAL QDDFRISYYK KHMWNALGSL
451 KNYGVKLKGY FAWSYLDNFE WNIGYTSRFG LYYVDYKNNL TRYPKKSAHW
501 FTKFLNISVN ANNIYELTSK DSRKVGKFYV M
```

Spot No.: **P5**

NCBI accession No.: **gi| 29170601** Species: *Hevea brasiliensis*

PFF score: [502]

Protein name: Small rubber particle protein

Matched peptides No.: [12] Sequence coverage %: [53]

Calculated Mr: **12769** Calculated *p*I: **4.61**

Matched sequences:

Query	Start – End	Observed	Mr(expt)	Mr(calc)	ppm	M	Score	Expect	Rank	U	Peptide
24	6 – 20	1865.8870	1864.8797	1864.7711	58.3	0	128	2.6e-10	1	U	K.ENENFQQEANEQEEK.L
25	6 – 20	1865.8879	1864.8806	1864.7711	58.7	0	135	6.1e-11	1	U	K.ENENFQQEANEQEEK.L
26	6 – 20	1865.9290	1864.9217	1864.7711	80.8	0	98	2.6e-07	1	U	K.ENENFQQEANEQEEK.L
18	47 – 64	1810.9230	1809.9157	1809.9472	-17.4	1	110	2.3e-08	1	U	K.DNSGPLKPGVETIEGVAK.T
19	47 – 64	1810.9230	1809.9157	1809.9472	-17.4	1	110	2.3e-08	1	U	K.DNSGPLKPGVETIEGVAK.T
20	47 – 64	1810.9230	1809.9157	1809.9472	-17.4	1	110	2.3e-08	1	U	K.DNSGPLKPGVETIEGVAK.T
21	47 – 64	1811.0605	1810.0532	1809.9472	58.6	1	153	1.4e-12	1	U	K.DNSGPLKPGVETIEGVAK.T
22	47 – 64	1811.0624	1810.0551	1809.9472	59.6	1	149	3e-12	1	U	K.DNSGPLKPGVETIEGVAK.T
23	47 – 64	1811.1030	1810.0957	1809.9472	82.0	1	142	1.6e-11	1	U	K.DNSGPLKPGVETIEGVAK.T
28	84 – 101	1902.9020	1901.8947	1902.0462	-79.7	0	85	5.6e-06	1	U	R.AVDASFTTLQNIVPSVLK.Q
29	84 – 101	1903.1536	1902.1463	1902.0462	52.6	0	41	0.14	1	U	R.AVDASFTTLQNIVPSVLK.Q
30	84 – 101	1903.1600	1902.1527	1902.0462	56.0	0	113	8.7e-09	1	U	R.AVDASFTTLQNIVPSVLK.Q
6	102 – 113	1347.7456	1346.7383	1346.6500	65.6	0	96	5.5e-07	1	U	K.QLPTQACDTSVK.E
7	102 – 113	1347.7468	1346.7395	1346.6500	66.5	0	102	1.4e-07	1	U	K.QLPTQACDTSVK.E

Probability Based Mowse Score:

Ions score is -10*Log(P), where P is the probability that the observed match is a random event.
Individual ions scores > 46 indicate identity or extensive homology (p<0.05).
Protein scores are derived from ions scores as a non-probabilistic basis for ranking protein hits.

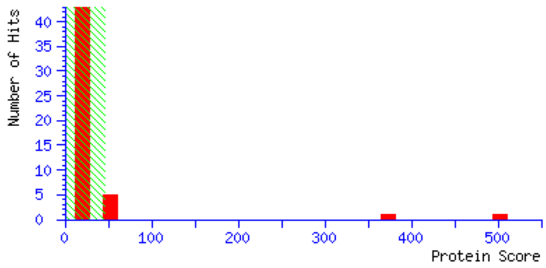

Matched peptide sequences: shown in Bold Red:

```
  1 MAEGKENENF QQEANEQEEK LKYLEFVQAT TDNAVTALSN IYLYAKDNSG
 51 PLKPGVETIE GVAKTVVIPA SKIPTEAIKF ADRAVDASFT TLQNIVPSVL
101 KQLPTQACDT SVKESAE
```

Spot No.: **P6**

NCBI accession No.: **gi| 29170601** Species: *Hevea brasiliensis*

PFF score: [429]

Protein name: Small rubber particle protein

Matched peptides No.: [18] Sequence coverage %: [53]

Calculated Mr: **12769** Calculated *p*I: **4.61**

Matched sequences:

Query	Start – End	Observed	Mr(expt)	Mr(calc)	ppm	M	Score	Expect	Rank	U	Peptide
28	6 – 20	1865.7990	1864.7917	1864.7711	11.1	0	67	0.00033	1	U	K.ENENFQQEANEQEEK.L
29	6 – 20	1865.7990	1864.7917	1864.7711	11.1	0	67	0.00033	1	U	K.ENENFQQEANEQEEK.L
30	6 – 20	1865.8870	1864.8797	1864.7711	58.3	0	104	7.5e-08	1	U	K.ENENFQQEANEQEEK.L
31	6 – 20	1865.8879	1864.8806	1864.7711	58.7	0	114	7.6e-09	1	U	K.ENENFQQEANEQEEK.L
32	6 – 20	1865.9290	1864.9217	1864.7711	80.8	0	74	7.8e-05	1	U	K.ENENFQQEANEQEEK.L
22	47 – 64	1810.9230	1809.9157	1809.9472	-17.4	1	78	3.9e-05	1	U	K.DNSGPLKPGVETIEGVAK.T
23	47 – 64	1810.9230	1809.9157	1809.9472	-17.4	1	78	3.9e-05	1	U	K.DNSGPLKPGVETIEGVAK.T
24	47 – 64	1810.9230	1809.9157	1809.9472	-17.4	1	78	3.9e-05	1	U	K.DNSGPLKPGVETIEGVAK.T
25	47 – 64	1811.0605	1810.0532	1809.9472	58.6	1	111	1.7e-08	1	U	K.DNSGPLKPGVETIEGVAK.T
26	47 – 64	1811.0624	1810.0551	1809.9472	59.6	1	121	2e-09	1	U	K.DNSGPLKPGVETIEGVAK.T
27	47 – 64	1811.1030	1810.0957	1809.9472	82.0	1	94	8.9e-07	1	U	K.DNSGPLKPGVETIEGVAK.T
34	84 – 101	1902.9020	1901.8947	1902.0462	-79.7	0	67	0.00034	1	U	R.AVDASFTTLQNIVPSVLK.Q
35	84 – 101	1903.1536	1902.1463	1902.0462	52.6	0	40	0.16	1	U	R.AVDASFTTLQNIVPSVLK.Q
36	84 – 101	1903.1600	1902.1527	1902.0462	56.0	0	109	2.2e-08	1	U	R.AVDASFTTLQNIVPSVLK.Q
7	102 – 113	1347.6600	1346.6527	1346.6500	2.00	0	16	53	1	U	K.QLPTQACDTSVK.E
8	102 – 113	1347.6600	1346.6527	1346.6500	2.00	0	16	53	1	U	K.QLPTQACDTSVK.E
9	102 – 113	1347.7456	1346.7383	1346.6500	65.6	0	84	1e-05	1	U	K.QLPTQACDTSVK.E
10	102 – 113	1347.7468	1346.7395	1346.6500	66.5	0	86	6e-06	1	U	K.QLPTQACDTSVK.E

Probability Based Mowse Score:

Ions score is -10*Log(P), where P is the probability that the observed match is a random event.
Individual ions scores > 45 indicate identity or extensive homology (p<0.05).
Protein scores are derived from ions scores as a non-probabilistic basis for ranking protein hits.

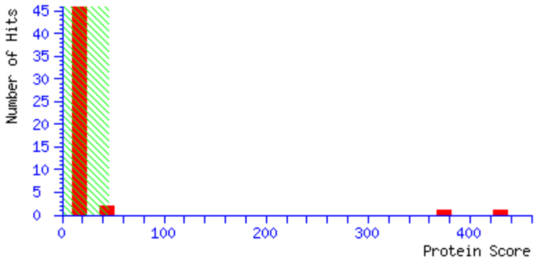

Matched peptide sequences: shown in Bold Red:

```
  1 MAEGKENENF QQEANEQEEK LKYLEFVQAT TDNAVTALSN IYLYAKDNSG
 51 PLKPGVETIE GVAKTVVIPA SKIPTEAIKF ADRAVDASFT TLQNIVPSVL
101 KQLPTQACDT SVKESAE
```

Spot No.: **P7**

NCBI accession No.: **gi| 224071449** Species: *Populus trichocarpa*

PFF score: [75]

Protein name: predicted protein

Matched peptides No.: [1]

Sequence coverage %: [2]

Matched sequences: K.NKIDVIPHYPGR.L;

K.IDVIPHYPGR.L

Calculated Mr: **47121**

Calculated *p*I: **5.28**

Probability Based Mowse Score:

Ions score is -10*Log(P), where P is the probability that the observed match is a random event.
Individual ions scores > 45 indicate identity or extensive homology (p<0.05).
Protein scores are derived from ions scores as a non-probabilistic basis for ranking protein hits.

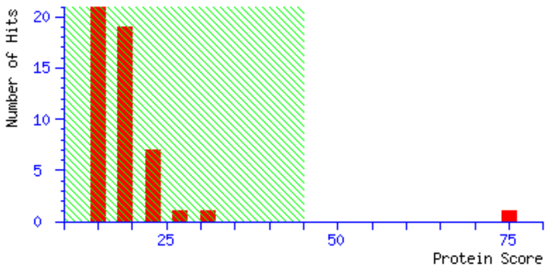

Matched peptide sequences: shown in **Bold Red:**

```
  1 MDGTSEATWP EILGSRNWDN LLDPLDLSLR KLILRCGDFC QATYDAFNND
 51 QNSRYCGTSR YGKRNFFHKV MLDNPENYQV SSFLYATARV SLPEAFLLHS
101 LSRDSWDRET NWIGYIAVTS DEQTKTLGRR EIYIAFRGTT RNYEWVDILG
151 AKLKSAKPLL RGATSTTHDQ ESSSSSDDDD DKVPKVMLGW LTMYISDDPN
201 SPFTKLSARA QLLAHIKELR ERYKDDDLSI IFTGHSLGAS LSILSAFDLV
251 ENGITDIPVS AFVFGSPQVG NKEFNERFNK YPNLKVLHIK NKIDVIPHYP
301 GRLMGYVYTG IEFEIDTRKS PSLKDSKNPS DWHNLQAMLH IVAGWNGEEQ
351 EFELKVKRSL ALVNKSSEFL KDECLVPGIW WVEKNKGMVR NEDGEWVLAP
401 PDEEDLPVPE C
```

Spot No.: **P8**

NCBI accession No.: **gi| 32186890**   Species: *Gossypium hirsutum*

PFF score: [519]

Protein name: actin

Matched peptides No.: [8]   Sequence coverage %: [37]

Matched sequences: K.YPIEHGIVSNWDDMEK.I;
K.IWHHTFYNELR.V;
R.VAPEEHPVLLTEAPLNPK.A;
R.TTGIVLDSGDGVSHTVPIYEGYALPHAILR.L;
K.LAYVALDYEQELETAK.S;
K.NYELPDGQVITIGAER.F;
R.KDLYGNIVLSGGSTMFPGIADR.M + Oxidation (M);
K.GEYDESGPSIVHR.K

Calculated Mr: **41917**   Calculated *p*I: **5.31**

Probability Based Mowse Score:

Ions score is -10*Log(P), where P is the probability that the observed match is a random event.
Individual ions scores > 45 indicate identity or extensive homology (p<0.05).
Protein scores are derived from ions scores as a non-probabilistic basis for ranking protein hits.

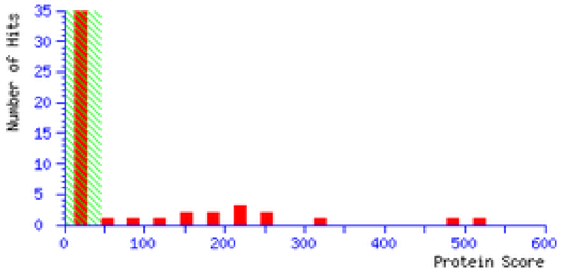

Matched peptide sequences: shown in Bold Red:

```
  1 MADGEDIQPL VCDNGTGMVK AGFAGDDAPR AVFPSIVGRP RHTGVMVGMG
 51 QKDAYVGDEA QSKRGILTLK YPIEHGIVSN WDDMEKIWHH TFYNELRVAP
101 EEHPVLLTEA PLNPKANREK MTQIMFETFN VPAMYVAIQA VLSLYASGRT
151 TGIVLDSGDG VSHTVPIYEG YALPHAILRL DLAGRDLTDA LMKILTERGY
201 MFTTTAEREI VRDMKEKLAY VALDYEQELE TAKSSSSVEK NYELPDGQVI
251 TIGAERFRCP EVLFQPSFIG MEAAGIHETT YNSIMKCDVD IRKDLYGNIV
301 LSGGSTMFPG IADRMSKEIT ALAPSSMKIK VVAPPERKYS VWIGGSILAS
351 LSTFQQMWIS KGEYDESGPS IVHRKCF
```

Spot No.: **P9**

NCBI accession No.: **gi| 14423933**   Species: *Hevea brasiliensis*

PFF score: [384]

Protein name: Small rubber particle protein

Matched peptides No.: [5]   Sequence coverage %: [36]

Calculated Mr: **22331**   Calculated *p*I: **4.80**

Matched sequences:

Query	Start – End	Observed	Mr(expt)	Mr(calc)	ppm	M	Score	Expect	Rank	U	Peptide
27	36 – 53	1881.1389	1880.1316	1880.0255	56.4	1	86	4.6e-06	1	U	K.DISGPLKPGVDTIENVVK.T
12	91 – 105	1620.8983	1619.8910	1619.7903	62.2	0	118	3.3e-09	1	U	K.QVSAQTYSVAQDAPR.I
31	106 – 124	1934.1237	1933.1164	1933.0157	52.1	0	95	5.3e-07	1	U	R.IVLDVASSVFNTGVQEGAK.A
3	134 – 143	1236.7086	1235.7013	1235.6299	57.8	0	61	0.002	1	U	K.AEQYAVITWR.A
5	183 – 194	1346.8149	1345.8076	1345.7493	43.3	0	25	8.1	1	U	R.VSSYLPLLPTEK.I

Probability Based Mowse Score:

Ions score is -10*Log(P), where P is the probability that the observed match is a random event.
Individual ions scores > 45 indicate identity or extensive homology (p<0.05).
Protein scores are derived from ions scores as a non-probabilistic basis for ranking protein hits.

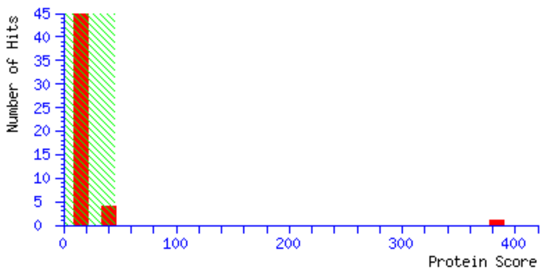

Matched peptide sequences: shown in Bold Red:

1 MAEGKENENF QQEANEQEEK LKYLEFVQAT TDNAVTALSN IYLYAKDNSG
51 PLKPGVETIE GVAKTVVIPA SKIPTEAIKF ADRAVDASFT TLQNIVPSVL
101 KQLPTQACDT SVKESAE

Spot No.: **P10**

NCBI accession No.: **gi| 224071449** Species: *Populus trichocarpa*

PFF score: [71]

Protein name: predicted protein

Matched peptides No.: [1] Sequence coverage %: [2]

Matched sequences: K.IDVIPHYPGR.L;

Calculated Mr: **47121** Calculated *p*I: **5.28**

Probability Based Mowse Score:

Ions score is -10*Log(P), where P is the probability that the observed match is a random event.
Individual ions scores > 45 indicate identity or extensive homology (p<0.05).
Protein scores are derived from ions scores as a non-probabilistic basis for ranking protein hits.

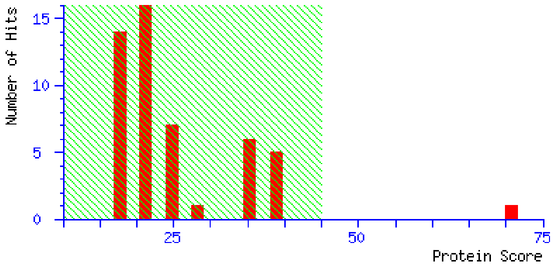

Matched peptide sequences: shown in Bold Red:

```
  1 MDGTSEATWP EILGSRNWDN LLDPLDLSLR KLILRCGDFC QATYDAFNND
 51 QNSRYCGTSR YGKRNFFHKV MLDNPENYQV SSFLYATARV SLPEAFLLHS
101 LSRDSWDRET NWIGYIAVTS DEQTKTLGRR EIYIAFRGTT RNYEWVDILG
151 AKLKSAKPLL RGATSTTHDQ ESSSSSDDDD DKVPKVMLGW LTMYISDDPN
201 SPFTKLSARA QLLAHIKELR ERYKDDDLSI IFTGHSLGAS LSILSAFDLV
251 ENGITDIPVS AFVFGSPQVG NKEFNERFNK YPNLKVLHIK NKIDVIPHYP
301 GRLMGYVYTG IEFEIDTRKS PSLKDSKNPS DWHNLQAMLH IVAGWNGEEQ
351 EFELKVKRSL ALVNKSSEFL KDECLVPGIW WVEKNKGMVR NEDGEWVLAP
401 PDEEDLPVPE C
```

Spot No.: **P11**

NCBI accession No.: **gi| 224071449** Species: *Populus trichocarpa*

PFF score: [52]

Protein name: predicted protein

Matched peptides No.: [1]

Sequence coverage %: [2]

Matched sequences: K.IDVIPHYPGR.L;

Calculated Mr: **47121**

Calculated *p*I: **5.28**

Probability Based Mowse Score:

Ions score is -10*Log(P), where P is the probability that the observed match is a random event.
Individual ions scores > 44 indicate identity or extensive homology (p<0.05).
Protein scores are derived from ions scores as a non-probabilistic basis for ranking protein hits.

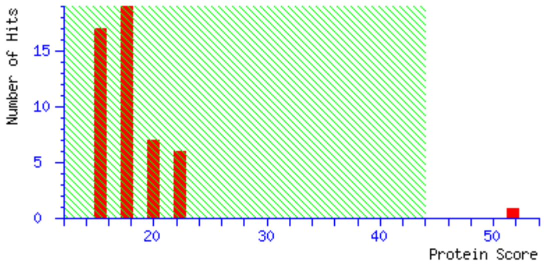

Matched peptide sequences: shown in Bold Red:

```
  1 MDGTSEATWP EILGSRNWDN LLDPLDLSLR KLILRCGDFC QATYDAFNND
 51 QNSRYCGTSR YGKRNFFHKV MLDNPENYQV SSFLYATARV SLPEAFLLHS
101 LSRDSWDRET NWIGYIAVTS DEQTKTLGRR EIYIAFRGTT RNYEWVDILG
151 AKLKSAKPLL RGATSTTHDQ ESSSSSDDDD DKVPKVMLGW LTMYISDDPN
201 SPFTKLSARA QLLAHIKELR ERYKDDDLSI IFTGHSLGAS LSILSAFDLV
251 ENGITDIPVS AFVFGSPQVG NKEFNERFNK YPNLKVLHIK NKIDVIPHYP
301 GRLMGYVYTG IEFEIDTRKS PSLKDSKNPS DWHNLQAMLH IVAGWNGEEQ
351 EFELKVKRSL ALVNKSSEFL KDECLVPGIW WVEKNKGMVR NEDGEWVLAP
401 PDEEDLPVPE C
```

Spot No.: **P12**

NCBI accession No.: **gi| 224071449** Species: *Populus trichocarpa*

PFF score: [106]

Protein name: predicted protein

Matched peptides No.: [2]   Sequence coverage %: [2]

Matched sequences: K.NKIDVIPHYPGR.L;

K.IDVIPHYPGR.L;

Calculated Mr: **47121**   Calculated *p*I: **5.28**

Probability Based Mowse Score:

Ions score is -10*Log(P), where P is the probability that the observed match is a random event.
Individual ions scores > 43 indicate identity or extensive homology (p<0.05).
Protein scores are derived from ions scores as a non-probabilistic basis for ranking protein hits.

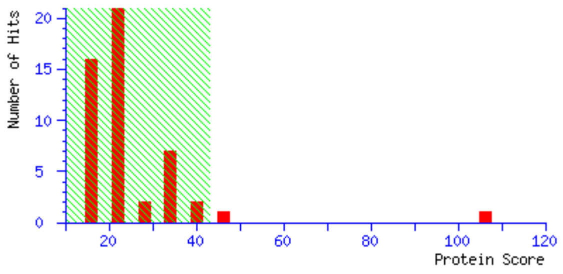

Matched peptide sequences: shown in Bold Red:

```
  1 MDGTSEATWP EILGSRNWDN LLDPLDLSLR KLILRCGDFC QATYDAFNND
 51 QNSRYCGTSR YGKRNFFHKV MLDNPENYQV SSFLYATARV SLPEAFLLHS
101 LSRDSWDRET NWIGYIAVTS DEQTKTLGRR EIYIAFRGTT RNYEWVDILG
151 AKLKSAKPLL RGATSTTHDQ ESSSSSDDDD DKVPKVMLGW LTMYISDDPN
201 SPFTKLSARA QLLAHIKELR ERYKDDDLSI IFTGHSLGAS LSILSAFDLV
251 ENGITDIPVS AFVFGSPQVG NKEFNERFNK YPNLKVLHIK NKIDVIPHYP
301 GRLMGYVYTG IEFEIDTRKS PSLKDSKNPS DWHNLQAMLH IVAGWNGEEQ
351 EFELKVKRSL ALVNKSSEFL KDECLVPGIW WVEKNKGMVR NEDGEWVLAP
401 PDEEDLPVPE C
```

Spot No.: **P13**

NCBI accession No.: **gi| 1550740** Species: *Arabidopsis thaliana*

PFF score: [51]

Protein name: GDP-associated inhibitor

Matched peptides No.: [1] Sequence coverage %: [5]

Matched sequences: R.FQGGSPYIYPLYGLGELPQAFAR.L;

Calculated Mr: **50269** Calculated *p*I: **5.49**

Probability Based Mowse Score:

Ions score is -10*Log(P), where P is the probability that the observed match is a random event.
Individual ions scores > 43 indicate identity or extensive homology (p<0.05).
Protein scores are derived from ions scores as a non-probabilistic basis for ranking protein hits.

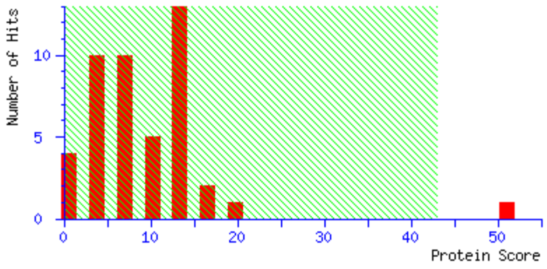

Matched peptide sequences: shown in **Bold Red:**

```
  1 MDEEYEVIVL GTGLKECILS GLLSVDGLKV LHMDRNDYYG GESTSLNLNQ
 51 LWKKFRGEEK APAHLGSSRD YNVDMMPKFM MANGKLVRVL IHTDVTKYLS
101 FKAVDGSYVF VQGKVQKVPA TPMEALKSPL MGIFEKRRAG KFFSYVQEYD
151 EKDPKTHDGM DLRRVTTKDL IAKFGLKEDT IDFIGHAVAL HCNDNHLHQP
201 AYDTVMRMKL YAESLARFQG GSPYIYPLYG LGELPQAFAR LSAVYGGTYM
251 LNKPECKVEF DEEGKVSGVT SEGETAKCKK VVCDPSYLTN KVRKIGRVAR
301 AIAIMSHPIP NTNDSQSVQV ILPQKQLGRK SDMYVFCCSY SHNVAPKGKF
351 IAFVSTDAET DNPQTELQPG IDLLGPVDEL FFDIYDRYEP VNEPTLDNCF
401 ISTSYDATTH FDTTVVDVLN MYKLITGKEL DLSVDLNPAS AAEEE
```

Spot No.: **P14**

NCBI accession No.: **gi| 255552291** Species: *Ricinus communis*

PFF score: [144]

Protein name: short chain dehydrogenase, putative

Matched peptides No.: [3] Sequence coverage %: [11]

Matched sequences: K.VALVTGGDSGIGR.A;
K.AEGAKDPIAIPTDVGFEENCR.K;
K.DPIAIPTDVGFEENCR.K

Calculated Mr: **32148** Calculated *p*I: **6.50**

Probability Based Mowse Score:

Ions score is -10*Log(P), where P is the probability that the observed match is a random event.
Individual ions scores > 45 indicate identity or extensive homology (p<0.05).
Protein scores are derived from ions scores as a non-probabilistic basis for ranking protein hits.

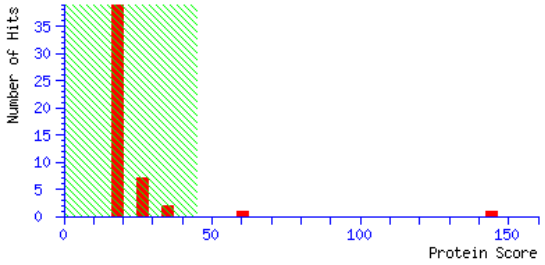

Matched peptide sequences: shown in Bold Red:

```
  1 MATRRGYRFP PQSQDKQPGK EYLMHPLPEF INPHYKPSNK LQDKVALVTG
 51 GDSGIGRAVS YYFTLEGATV AFTYVKGRED KDKDHILKIL HEVKAEGAKD
101 PIAIPTDVGF EENCRKVIDQ IMSEYGKIDI LVNNAGEAHY STTIEDITDP
151 MLERVFRTNI FGHFFMSRHA LKHMKEGGCI INTASVAAYA GFSFMVDYSS
201 TKGAVVAFTR SLALQLIDRG IRVNAVAPGS VWTPFQPSTL SAEQVAQVGS
251 GVPMDRAAQP YEIAPSFVFL ASNDCSSYFT GQVLHPNGMF
```

Spot No.: **P15**

NCBI accession No.: **gi| 218157**Species: ***Oryza sativa***

PFF score: [174]

Protein name: cytoplasmic aldolase

Matched peptides No.: [2] Sequence coverage %: [3]

Matched sequences: K.KVAPEVIAEYTVR.T;

K.VAPEVIAEYTVR.T;

Calculated Mr: **39151** Calculated *p*I: **6.56**

Probability Based Mowse Score:

Ions score is -10*Log(P), where P is the probability that the observed match is a random event.
Individual ions scores > 45 indicate identity or extensive homology (p<0.05).
Protein scores are derived from ions scores as a non-probabilistic basis for ranking protein hits.

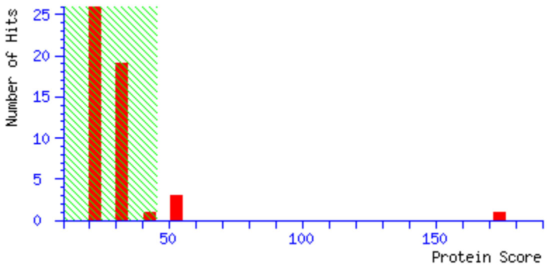

Matched peptide sequences: shown in Bold Red:

```
  1 MSAYCGKYKD ELIKNAAYIG TPGKGILAAD ESTGTIGKRF ASINVENVED
 51 NRRAFRELLF CTPGALQYIS GVILFDETLY QKTKDGKPFV DILKEAGALP
101 GIKVDKGTIE VAGTDKETTT QGHDDLGKQC AKYYEAGARF AKWRAVLKIG
151 PNQPSQLAID LNAQGLACYA IICQENGLVP IVEPEILVDG PHDIDRCAYV
201 SEVVLAACYK ALNEHHVLLE GTLLKPNMVT PGSDAKKVAP EVIAEYTVRT
251 LQRTVPPAVP AIVFLSGGQS EEEATLNLNA MNKLSAKKPW SLSFSFGRAL
301 QQSTLKAWAG KTENVEKARA AFLVRCKANS EATLGTYKGD AVLGEGAAES
351 LHVKDYKY
```

Spot No.: **P16**

NCBI accession No.: **gi| 218157** Species: *Oryza sativa Japonica Group*

PFF score: [125]

Protein name: cytoplasmic aldolase

Matched peptides No.: [2]

Sequence coverage %: [3]

Matched sequences: K.KVAPEVIAEYTVR.T;

K.VAPEVIAEYTVR.T;

Calculated Mr: **39151**

Calculated *p*I: **6.56**

Probability Based Mowse Score:

Ions score is -10*Log(P), where P is the probability that the observed match is a random event.
Individual ions scores > 45 indicate identity or extensive homology (p<0.05).
Protein scores are derived from ions scores as a non-probabilistic basis for ranking protein hits.

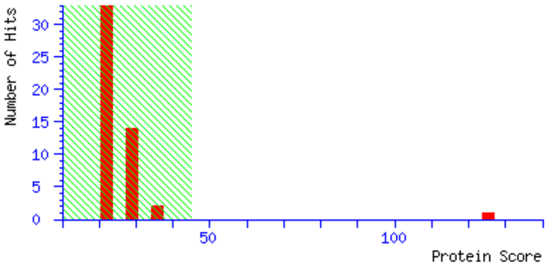

Matched peptide sequences: shown in Bold Red:

```
  1 MSAYCGKYKD ELIKNAAYIG TPGKGILAAD ESTGTIGKRF ASINVENVED
 51 NRRAFRELLF CTPGALQYIS GVILFDETLY QKTKDGKPFV DILKEAGALP
101 GIKVDKGTIE VAGTDKETTT QGHDDLGKQC AKYYEAGARF AKWRAVLKIG
151 PNQPSQLAID LNAQGLACYA IICQENGLVP IVEPEILVDG PHDIDRCAYV
201 SEVVLAACYK ALNEHHVLLE GTLLKPNMVT PGSDAKKVAP EVIAEYTVRT
251 LQRTVPPAVP AIVFLSGGQS EEEATLNLNA MNKLSAKKPW SLSFSFGRAL
301 QQSTLKAWAG KTENVEKARA AFLVRCKANS EATLGTYKGD AVLGEGAAES
351 LHVKDYKY
```

Spot No.: **P17**

NCBI accession No.: **gi| 14423933** Species: *Hevea brasiliensis*

PFF score: [171]

Protein name: Small rubber particle protein

Matched peptides No.: [3] Sequence coverage %: [19]

Calculated Mr: **22331** Calculated *p*I: **4.80**

Matched sequences:

Query	Start – End	Observed	Mr(expt)	Mr(calc)	ppm	M	Score	Expect	Rank	U	Peptide
8	36 – 53	1881.1389	1880.1316	1880.0255	56.4	1	86	3.3e-06	1	U	K.DISGPLKPGVDTIENVVK.T
2	134 – 143	1236.7086	1235.7013	1235.6299	57.8	0	61	0.0016	1	U	K.AEQYAVITWR.A
4	183 – 194	1346.8149	1345.8076	1345.7493	43.3	0	25	6.5	1	U	R.VSSYLPLLPTEK.I

Probability Based Mowse Score:

Ions score is -10*Log(P), where P is the probability that the observed match is a random event.
Individual ions scores > 45 indicate identity or extensive homology (p<0.05).
Protein scores are derived from ions scores as a non-probabilistic basis for ranking protein hits.

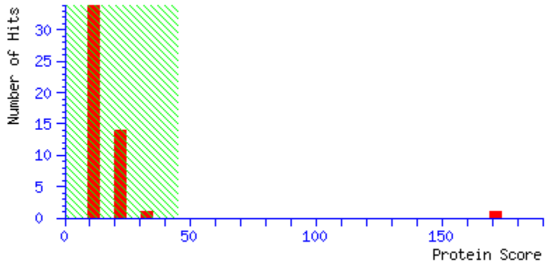

Matched peptide sequences: shown in Bold Red:

1 MAEEVEEERL KYLDFVRAAG VYAVDSFSTL YLYAK**DISGP** **LKPGVDTIEN**
51 **VVK**TVVTPVY YIPLEAVKFV DKTVDVSVTS LDGVVPPVIK QVSAQTYSVA
101 QDAPRIVLDV ASSVFNTGVQ EGAKALYANL EPK**AEQYAVI** **TWR**ALNKLPL
151 VPQVANVVVP TAVYFSEKYN DVVRGTTEQG YR**VSSYLPLL** **PTEK**ITKVFG
201 DEAS

Spot No.: **P18**

NCBI accession No.: **gi| 118489480**

Species: *Populus trichocarpa x Populus deltoids*

PFF score: [74]

Protein name: unknown

Matched peptides No.: [2]

Sequence coverage %: [3]

Matched sequences: R.KLLVALVSAYR.Y;

K.LLVALVSAYR.Y

Calculated Mr: **35859**

Calculated *p*I: **8.42**

Probability Based Mowse Score:

Ions score is -10*Log(P), where P is the probability that the observed match is a random event.
Individual ions scores > 45 indicate identity or extensive homology (p<0.05).
Protein scores are derived from ions scores as a non-probabilistic basis for ranking protein hits.

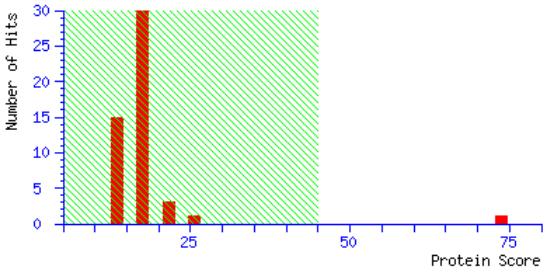

Matched peptide sequences: shown in Bold Red:

```
  1 MAHLEALAKA FTGLGVDEKS LIENLGKSHP EQRTLFRKKT PQLFIEDERS
 51 FERWNDHCVR LLKHEFVRFK NALVLWAMHP WERDARLVKE ALKKGPQSYG
101 VIVEIACTRS SEELLGARKA YHSLFDQSIE EDVATHIHGS ERKLLVALVS
151 AYRYEGPKVK EDAAKSEAKI LANAIKNGNK KNPIEDEEVI RILSTRSKAH
201 LKVACKHYKE VSGNNIHEDL DPSDLILKET VECLCTPHAY FSKVLDEAMS
251 SNAHKNTKKG LTRVIVTRAD VDMKEIKEEY MNLFGVSLSK KIEEKANGNY
301 RDFLVTLITR DN
```

Spot No.: **P19**

NCBI accession No.: **gi|132270**   Species: *Hevea brasiliensis*

PFF score: [341]

Protein name: Rubber elongation factor protein

Matched peptides No.: [3]   Sequence coverage %: [19]

Matched sequences:

Query	Start - End	Observed	Mr(expt)	Mr(calc)	ppm	M	Score	Expect	Rank	U	Peptide
29	41 - 58	1849.2604	1848.2531	1847.9993	137	1	169	1.6e-14	1	U	K.DKSGPLQPGVDIIEGPVK.N
16	43 - 58	1606.0936	1605.0863	1604.8774	130	0	121	1.3e-09	1	U	K.SGPLQPGVDIIEGPVK.N
1	59 - 67	1045.7102	1044.7029	1044.5716	126	0	51	0.021	1	U	K.NVAVPLYNR.F

Calculated Mr: **14713**   Calculated *p*I: **5.04**

Probability Based Mowse Score:

Ions score is -10*Log(P), where P is the probability that the observed match is a random event.
Individual ions scores > 44 indicate identity or extensive homology (p<0.05).
Protein scores are derived from ions scores as a non-probabilistic basis for ranking protein hits.

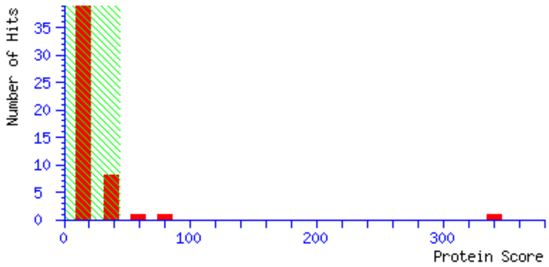

Matched peptide sequences: shown in Bold Red:

```
  1 MAEDEDNQQG QGEGLKYLGF VQDAATYAVT TFSNVYLFAK DKSGPLQPGV
 51 DIIEGPVKNV AVPLYNRFSY IPNGALKFVD STVVASVTII DRSLPPIVKD
101 ASIQVVSAIR AAPEAARSLA SSLPGQTKIL AKVFYGEN
```

Spot No.: **P20**

NCBI accession No.: **gi|132270**   Species: *Hevea brasiliensis*

PFF score: [597]

Protein name: Rubber elongation factor protein

Matched peptides No.: [16]   Sequence coverage %: [38]

Matched sequences:

Query	Start - End	Observed	Mr(expt)	Mr(calc)	ppm	M	Score	Expect	Rank	U	Peptide
94	41 - 58	1849.0222	1848.0149	1847.9993	8.46	1	119	1.4e-09	1	U	K.DKSGPLQPGVDIIEGPVK.N
95	41 - 58	1849.1149	1848.1076	1847.9993	58.6	1	115	4.3e-09	1	U	K.DKSGPLQPGVDIIEGPVK.N
97	41 - 58	1849.2604	1848.2531	1847.9993	137	1	169	1.6e-14	1	U	K.DKSGPLQPGVDIIEGPVK.N
68	43 - 58	1606.0936	1605.0863	1604.8774	130	0	121	1.3e-09	1	U	K.SGPLQPGVDIIEGPVK.N
2	59 - 67	1045.5845	1044.5772	1044.5716	5.38	0	71	0.00021	1	U	K.NVAVPLYNR.F
3	59 - 67	1045.6366	1044.6293	1044.5716	55.3	0	71	0.00023	1	U	K.NVAVPLYNR.F
4	59 - 67	1045.6639	1044.6566	1044.5716	81.4	0	68	0.00043	1	U	K.NVAVPLYNR.F
5	59 - 67	1045.7102	1044.7029	1044.5716	126	0	51	0.021	1	U	K.NVAVPLYNR.F
6	59 - 67	1045.7162	1044.7089	1044.5716	131	0	77	5.8e-05	1	U	K.NVAVPLYNR.F
69	78 - 92	1621.9017	1620.8944	1620.8723	13.6	0	127	2.8e-10	1	U	K.FVDSTVVASVTIIDR.S
70	78 - 92	1621.9749	1620.9676	1620.8723	58.8	0	43	0.077	1	U	K.FVDSTVVASVTIIDR.S
71	78 - 92	1621.9778	1620.9705	1620.8723	60.6	0	109	2e-08	1	U	K.FVDSTVVASVTIIDR.S
18	100 - 110	1158.6577	1157.6504	1157.6404	8.64	0	80	2.3e-05	1	U	K.DASIQVVSAIR.A
19	100 - 110	1158.7130	1157.7057	1157.6404	56.4	0	85	6.8e-06	1	U	K.DASIQVVSAIR.A
21	100 - 110	1158.7190	1157.7117	1157.6404	61.6	0	56	0.0056	1	U	K.DASIQVVSAIR.A
23	100 - 110	1158.7994	1157.7921	1157.6404	131	0	103	1e-07	1	U	K.DASIQVVSAIR.A

Calculated Mr: **14713**   Calculated *p*I: **5.04**

Probability Based Mowse Score:

Ions score is -10*Log(P), where P is the probability that the observed match is a random event.
Individual ions scores > 45 indicate identity or extensive homology (p<0.05).
Protein scores are derived from ions scores as a non-probabilistic basis for ranking protein hits.

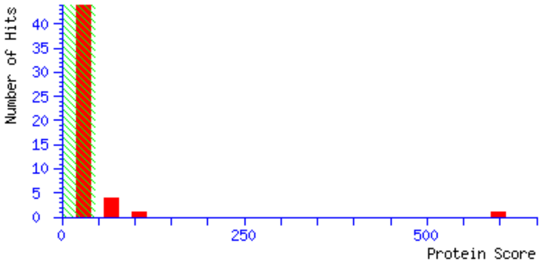

Matched peptide sequences: shown in **Bold Red:**

```
  1 MAEDEDNQQG QGEGLKYLGF VQDAATYAVT TFSNVYLFAK DKSGPLQPGV
 51 DIIEGPVKNV AVPLYNRFSY IPNGALKFVD STVVASVTII DRSLPPIVKD
101 ASIQVVSAIR AAPEAARSLA SSLPGQTKIL AKVFYGEN
```

Spot No.: **P21**

NCBI accession No.: **gi|132270**   Species: *Hevea brasiliensis*

PFF score: [672]

Protein name: Rubber elongation factor protein

Matched peptides No.: [23]   Sequence coverage %: [46]

Matched sequences:

Query	Start – End	Observed	Mr(expt)	Mr(calc)	ppm	M	Score	Expect	Rank	U	Peptide
134	41 – 58	1849.0222	1848.0149	1847.9993	8.46	1	119	1.9e-09	1	U	K.DKSGPLQPGVDIIEGPVK.N
135	41 – 58	1849.0710	1848.0637	1847.9993	34.9	1	148	2.9e-12	1	U	K.DKSGPLQPGVDIIEGPVK.N
136	41 – 58	1849.0801	1848.0728	1847.9993	39.8	1	157	3.4e-13	1	U	K.DKSGPLQPGVDIIEGPVK.N
137	41 – 58	1849.1149	1848.1076	1847.9993	58.6	1	115	5.9e-09	1	U	K.DKSGPLQPGVDIIEGPVK.N
139	41 – 58	1849.2604	1848.2531	1847.9993	137	1	169	2.3e-14	1	U	K.DKSGPLQPGVDIIEGPVK.N
100	43 – 58	1605.9473	1604.9400	1604.8774	39.0	0	128	3.3e-10	1	U	K.SGPLQPGVDIIEGPVK.N
101	43 – 58	1606.0936	1605.0863	1604.8774	130	0	121	1.8e-09	1	U	K.SGPLQPGVDIIEGPVK.N
5	59 – 67	1045.5845	1044.5772	1044.5716	5.38	0	71	0.00023	1	U	K.NVAVPLYNR.F
6	59 – 67	1045.6366	1044.6293	1044.5716	55.3	0	71	0.00026	1	U	K.NVAVPLYNR.F
7	59 – 67	1045.6639	1044.6566	1044.5716	81.4	0	68	0.00048	1	U	K.NVAVPLYNR.F
8	59 – 67	1045.7102	1044.7029	1044.5716	126	0	51	0.023	1	U	K.NVAVPLYNR.F
9	59 – 67	1045.7162	1044.7089	1044.5716	131	0	77	6.5e-05	1	U	K.NVAVPLYNR.F
102	78 – 92	1621.6825	1620.6752	1620.8723	-122	0	49	0.024	1	U	K.FVDSTVVASVTIIDR.S
103	78 – 92	1621.9017	1620.8944	1620.8723	13.6	0	127	3.8e-10	1	U	K.FVDSTVVASVTIIDR.S
104	78 – 92	1621.9475	1620.9402	1620.8723	41.9	0	95	6.1e-07	1	U	K.FVDSTVVASVTIIDR.S
105	78 – 92	1621.9749	1620.9676	1620.8723	58.8	0	43	0.1	1	U	K.FVDSTVVASVTIIDR.S
106	78 – 92	1621.9778	1620.9705	1620.8723	60.6	0	109	2.7e-08	1	U	K.FVDSTVVASVTIIDR.S
30	100 – 110	1158.6577	1157.6504	1157.6404	8.64	0	80	2.7e-05	1	U	K.DASIQVVSAIR.A
31	100 – 110	1158.7063	1157.6990	1157.6404	50.6	0	110	2.5e-08	1	U	K.DASIQVVSAIR.A
32	100 – 110	1158.7130	1157.7057	1157.6404	56.4	0	85	7.9e-06	1	U	K.DASIQVVSAIR.A
34	100 – 110	1158.7190	1157.7117	1157.6404	61.6	0	56	0.0065	1	U	K.DASIQVVSAIR.A
36	100 – 110	1158.7994	1157.7921	1157.6404	131	0	103	1.2e-07	1	U	K.DASIQVVSAIR.A
18	118 – 128	1088.6615	1087.6542	1087.5873	61.5	0	61	0.0021	1	U	R.SLASSLPGQTK.I

Calculated Mr: **14713**   Calculated *p*I: **5.04**

Probability Based Mowse Score:

Ions score is -10*Log(P), where P is the probability that the observed match is a random event.
Individual ions scores > 46 indicate identity or extensive homology (p<0.05).
Protein scores are derived from ions scores as a non-probabilistic basis for ranking protein hits.

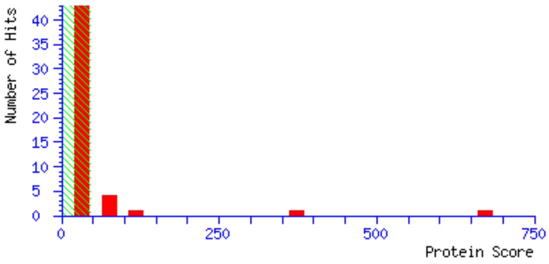

Matched peptide sequences: shown in **Bold Red:**

```
  1 MAEDEDNQQG QGEGLKYLGF VQDAATYAVT TFSNVYLFAK DKSGPLQPGV
 51 DIIEGPVKNV AVPLYNRFSY IPNGALKFVD STVVASVTII DRSLPPIVKD
101 ASIQVVSAIR AAPEAARSLA SSLPGQTKIL AKVFYGEN
```

Spot No.: **P22**

NCBI accession No.: **gi| 38154482** Species: *Nicotiana benthamiana*

PFF score: [197]

Protein name: molecular chaperone Hsp90-1

Matched peptides No.: [5] Sequence coverage %: [8]

Matched sequences: K.ADLVNNLGTIAR.S;
K.HNDDEQYVWESQAGGSFTVTR.D;
K.RAPFDLFDTR.K;
R.APFDLFDTR.K;
K.GIVDSEDLPLNISR.E

Calculated Mr: **80340** Calculated *p*I: **4.94**

Probability Based Mowse Score:

Ions score is -10*Log(P), where P is the probability that the observed match is a random event.
Individual ions scores > 45 indicate identity or extensive homology (p<0.05).
Protein scores are derived from ions scores as a non-probabilistic basis for ranking protein hits.

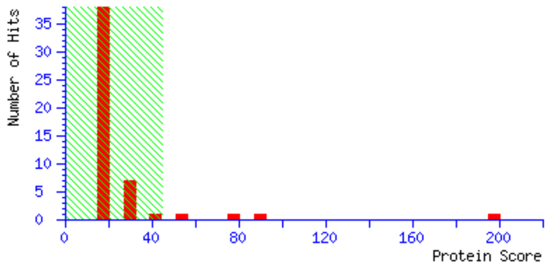

Matched peptide sequences: shown in **Bold Red:**

```
  1 MAEAETFAFQ AEINQLLSLI INTFYSNKEI FLRELISNSS DALDKIRFES
 51 LTDKSKLDAQ PELFIHIIPD KTNNSLTIID SGIGMTKADL VNNLGTIARS
101 GTKEFMEALA AGADVSMIGQ FGVGFYSAYL VAEKVIVTTK HNDDEQYVWE
151 SQAGGSFTVT RDTSGDNLGR GTKITLFLKE DQLEYLEERR LKDLIKKHSE
201 FISYPISLWV EKTIEKEISD DEEEEEKKDE EGKVEEVDEE KEKEEKKKKK
251 VKEASNEWSL VNKQKPIWMR KPDEITKEEY AAFYKSLTND WEEHLAVKHF
301 SVEGQLEFKA ILFVPKRAPF DLFDTRKKPN NIKLYVRRVF IMDNCEELIP
351 EYLSFVKGIV DSEDLPLNIS REMLQQNKIL KVIRKNLVKK CVELFFEIAE
401 NKEDYDKFYE AFSKNLKLGI HEDSQNRSKF AELLRYHSTK SGDEMTSLKD
451 YVTRMKEGQN DIYYITGESK KAVENSPFLE KLKKKGYEVL YMVDAIDEYS
501 IGQLKEFEGK KLVSATKEGL KLDESEDEKK KQEELKEKFE GLCKVIKDVL
551 GDKVEKVVVS DRVVDSPCCL VTGEYGWTAN MERIMKAQAL RDSSMAGYMS
601 SKKTMEINPE NAIMEELRKR ADADKNDKSV KDLVLLLFET ALLTSGFSLE
651 EPNTFGNRIH RMLKLGLSID DDSGDADVDM PALEDAEADA EGSKMEEVD
```

Spot No.: **P23**

NCBI accession No.: **gi| 1708314**   Species: *Ipomoea nil*

PFF score: [126]

Protein name: RecName: Full=Heat shock protein 83

Matched peptides No.: [5]   Sequence coverage %: [8]

Matched sequences: K.LDAQPELFIR.L;
K.ADLVNNLGTIAR.S;
K.ITLFLKEDQLEYLEER.R;
K.RAPFDLFDTR.K;
K.LGIHEDSQNR.A

Calculated Mr: **81055**   Calculated *p*I: **4.95**

Probability Based Mowse Score:

Ions score is -10*Log(P), where P is the probability that the observed match is a random event.
Individual ions scores > 46 indicate identity or extensive homology (p<0.05).
Protein scores are derived from ions scores as a non-probabilistic basis for ranking protein hits.

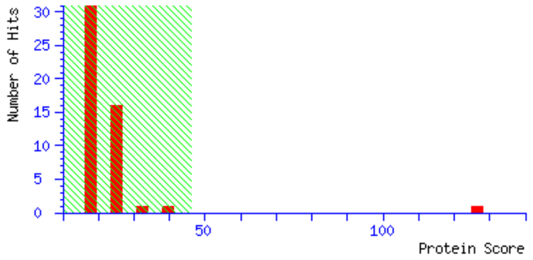

Matched peptide sequences: shown in Bold Red:

1 MADVQMAEAE TFAFQAEINQ LLSLIINTFY SNKEIFLREL ISNASDALDK
51 IRFESLTDKS K**LDAQPELFI** **R**LVPDKTNKT LSIIDSGVGM AK**ADLVNNLG**
101 **TIAR**SGTKEF MEALQAGADV SMIGQFGVGF YSAYLVAEKV IVTTKHNDDE
151 QYIWESQAGG SFTVTRDVDG EQLGRGTK**IT** **LFLKEDQLEY** **LEER**RIKDLV
201 KKHSEFISYP IYLWTEKTTE KEISDDEDDE PKKEEEGDIE EVDEDKEKEG
251 KKKKKIKEVS HEWQLINKQK PIWLRKPEEI TKEEYASFYK SLTNDWEDHL
301 AVKHFSVEGQ LEFKAILFVP K**RAPFDLFDT** **R**KKMNNIKLY VRRVFIMDNC
351 EELIPEYLGF VKGVVDSDDL PLNISREMLQ QNKILKVIRK NLVKKCIEMF
401 NEIAENKDDY NKFYEAFSKN LK**LGIHEDSQ** **NR**AKLADLLR YYSTKSGDEL
451 TSLKDYVTRM KEGQKDIYYI TGESKKAVEN SPFLERLKKK GYEVLFMVDA
501 IDEYAVGQLK EYDGKKLVSA TKEGLKLEDD DEEEKKKREE KKKSFENLCK
551 IIKDILGDKV EKVVVSDRIV DSPCCLVTGE YGWTANMERI MKAQALRDSS
601 MSSYMSSKKT MEINPDNGIM EELRKRAEAD KNDKSVKDLV LLLFETALLT
651 SGFSLDDPNT FGARIHRMLK LGLSIDEEEA GDDADMPALE EEAGEESKME
701 EVD

Spot No.: **P24**

NCBI accession No.: **gi| 20559** Species: *Petunia x hybrida*

PFF score: [136]

Protein name: hsp70 (AA 6 - 651)

Matched peptides No.: [4] Sequence coverage %: [11]

Matched sequences: K.NAVVTVPAYFNDSQR.Q;
R.TLSSTAQTTIEIDSLYEGIDFYSTITR.A;
R.ARFEELNMDLFR.K;
K.EQVFSTYSDNQPGVLIQVYEGER.A

Calculated Mr: **71137** Calculated *p*I: **5.07**

Probability Based Mowse Score:

Ions score is -10*Log(P), where P is the probability that the observed match is a random event.
Individual ions scores > 45 indicate identity or extensive homology (p<0.05).
Protein scores are derived from ions scores as a non-probabilistic basis for ranking protein hits.

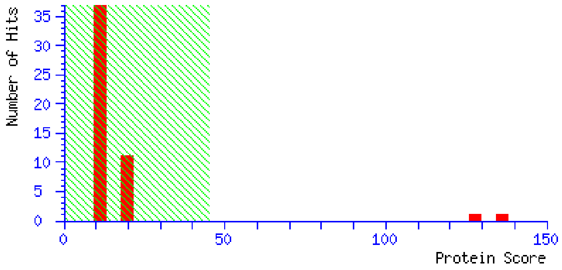

Matched peptide sequences: shown in **Bold Red:**

```
  1 EGPAIGIDLG TTYSCVGVWQ HDRVEIIAND QGNRTTPSYV GFTDTERLIG
 51 DAAKNQVAMN PINTVFDAKR LIGRRFSDPS VQSDIKLWPF KVIPGPGDKP
101 MIVVTYKGEE KQFAAEEISS MVLTKMKEIA EAYLGTTIKN AVVTVPAYFN
151 DSQRQATKDA GVIAGLNVMR IINEPTAAAI AYGLDKKASS AGEKNVLIFD
201 LGGGTFDVSL LTIEEGIFEV KATAGDTHLG GEDFDNRMVN HFVQEFKRKN
251 KKDISGNPRA LRRLRTACER AKRTLSSTAQ TTIEIDSLYE GIDFYSTITR
301 ARFEELNMDL FRKCMEPVEK CLRDAKMDKS SVHDVVLVGG STRIPKVQQL
351 LQDFFNGKEL CKSINPDEAV AYGAAVQAAI LSGEGNEKVQ DLLLLDVTPL
401 SLGLETAGGG MTVLIPRNTT IPTKKEQVFS TYSDNQPGVL IQVYEGERAR
451 TKDNNLLGKF ELSGIPPAPR GVPQITVCFD IDANGILNVS AEDKTTGQKN
501 KITITNDKGR LSKEEIERMV QEAEKYKSED EELKKKVEAK NALENYAYNM
551 RNTIKDDKIN SQLSAADKKR IEDAIDEAIK WLDNNQLAEA DEFEDKMKEL
601 ESICNPIIAK MYQGGAGGAT MDEDGPSVGG SAGSQTGAGP KIEEVD
```

Spot No.: **P25**

NCBI accession No.: **gi| 255566038**   Species: *Ricinus communis*

PFF score: [118]

Protein name: Phospholipase C 3 precursor, putative

Matched peptides No.: [2]   Sequence coverage %: [4]

Matched sequences: K.TVVVLVQENR.S;

K.HCEKGELPNYVVIEPR.Y

Calculated Mr: **59111**   Calculated *p*I: **5.08**

Probability Based Mowse Score:

Ions score is -10*Log(P), where P is the probability that the observed match is a random event.
Individual ions scores > 45 indicate identity or extensive homology (p<0.05).
Protein scores are derived from ions scores as a non-probabilistic basis for ranking protein hits.

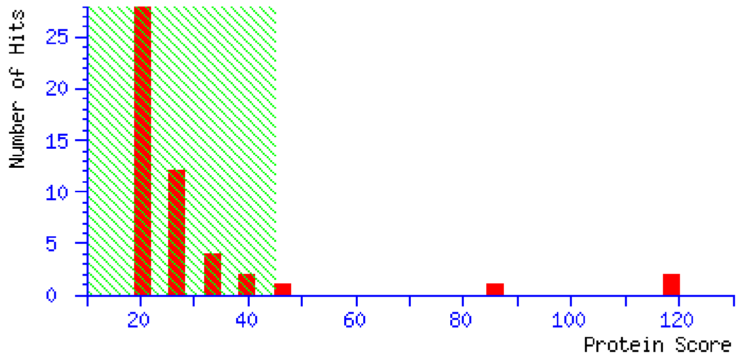

Matched peptide sequences: shown in Bold Red:

```
  1 MAAESSTSAN EYPIKTVVVL VQENRSFDHM LGWLKTLNPE IDGVTGQESN
 51 PISTSDLNSS LVFFRDQAAY VDPDPGHSIQ AIYEQVFGVE WTEAALSSEN
101 AVSPKMNGFA QNAERTQQGM AETVMNGFRP EAVPVYKELA MNFAVCDRWF
151 ASIPTSTQPN RLYLHSATSH GATSNDTKML AEGFPQKTIF ESMHEAGFSF
201 GIYFQQVPTT LFYRNLRKIK YLKNFHQFDL QFKKHCEKGE LPNYVVIEPR
251 YYDLLSIPAN DDHPSHDVSE GQKFVKEVYE ALRASPQWNE MLFIIIYDEH
301 GGFYDHVPTP ATGIPTPDDI VGPAPYLFKF DRLGVRVPAV LISPWIEPGT
351 VLHGPSGPQE TSEFEHSSIA ATVKKLFNLK EFLTKRDEWA GTFESVLTRT
401 SPRTDCPVTL TEPVKLRSRG ANEEANISEF QQELVQLAAV LNGDHGTDIY
451 PHKLVESMKV SEAANYVENA FKRFYEEYEK ARDGGVDESE VIALVKTEAL
501 AIITNTSASS KSFIQKLFSC IVCDN
```

Spot No.: **P26**

NCBI accession No.: **gi| 4235430**   Species: *Hevea brasiliensis*

PFF score: [80]

Protein name: latex-abundant protein

Matched peptides No.: [3]   Sequence coverage %: [9]

Matched sequences: R.SAEPGDLLFVHYSGHGTR.L;

R.EFVDQVPHGCR.I;

K.QTIQDAFESR.G

Calculated Mr: **46443**   Calculated *p*I: **5.01**

Probability Based Mowse Score:

Ions score is -10*Log(P), where P is the probability that the observed match is a random event.
Individual ions scores > 45 indicate identity or extensive homology (p<0.05).
Protein scores are derived from ions scores as a non-probabilistic basis for ranking protein hits.

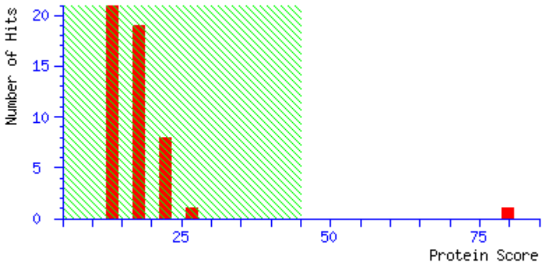

Matched peptide sequences: shown in **Bold Red:**

```
  1 MAKKAVLIGI NYPGTKAELK GCINDVKRMY RCLVDRYGFS EEDITVLIDI
 51 DESYIQPTGK NIRRVLTDLV RSAEPGDLLF VHYSGHGTRL PAETGEDDDT
101 GFDECIVPCD MNLITDDDFR EFVDQVPHGC RITVVSDSCH SGGLIDEAKE
151 QIGESTKRKE EESESGFGFK SFLKQTIQDA FESRGVHLPS DLHHHHGHRD
201 EEDFDNRVVE EDYGDSGYVK SKSLPLSTLI EILKQKTGKD DIDVGKLRPT
251 LFDMFGDDAS PKVKKFMKVI LNKLRHGDGE SGGGGFLGMV GSLAQEFLKH
301 KLDENDESYV KPALETEVDS KQEVYAGKTK RSLPDGGILI SGCQTDQTSA
351 DASPSGKSSE AYGALSNAIQ TIIAETDGAV TNQELVLKAR KMLKKQGFTQ
401 KPGLYCSDDH VEASFVC
```

Spot No.: **P27**

NCBI accession No.: **gi| 297833008** Species: *Arabidopsis lyrata subsp. Lyrata*

PFF score: [53]

Protein name: phosphoesterase family protein

Matched peptides No.: [2] Sequence coverage %: [3]

Matched sequences: K.TVVVLVQENR.S;

K.LPNYVVIEPR.Y

Calculated Mr: **59395** Calculated *p*I: **5.21**

Probability Based Mowse Score:

Ions score is -10*Log(P), where P is the probability that the observed match is a random event.
Individual ions scores > 45 indicate identity or extensive homology (p<0.05).
Protein scores are derived from ions scores as a non-probabilistic basis for ranking protein hits.

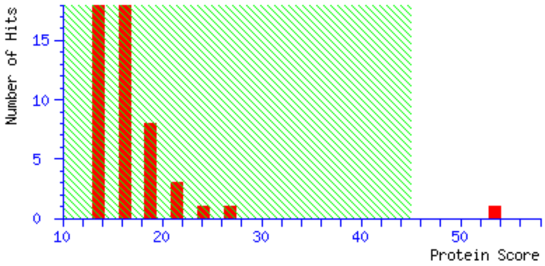

Matched peptide sequences: shown in Bold Red:

```
  1 MGEETSSGGG SSSSPIKTVV VLVQENRSFD HMLGWFKELN PEIDGVSESE
 51 PRSNPISTSD PNSAQVFFGK ESQNIDPDPG HSFQAIYEQV FGKPFSDESP
101 YPYPKMNGFV QNAEAITKGM SEKVVMQGFP PEKLPVFKEL VQEFAVCDRW
151 FSSLPSSTQP NRLYVHAATS NGAFSNDTNT LVRGFPQRTV FESLEESGFT
201 FGIYYQSFPN CLFYRNMRKL KYVDNFHQYH LSFKRHCKEG KLPNYVVIEP
251 RYFNILSAPA NDDHPKNDVA EGQNLVKEIY EALRASPQWN QILFVVVYDE
301 HGGYYDHVPT PVTGVPNPDG LVGPEPYNFK FDRLGVRVPA LLISPWIEPK
351 TVLHEPNGPE PTSQFEHSSI PATLKKIFNL KSFLTKRDEW AGTFDAVINR
401 TSPRTDCPVT LPELPRARDI DIETQEEDED LTDFQIELIQ AAAVLNGDHI
451 KDIYPFKLAD NMKVLDAARY VEEAFTRFHG ESKKAKEGGL DEHEIVDLSK
501 GSTRHSSPKS FVQKFFSCLI CDN
```

Spot No.: **P28**

NCBI accession No.: **gi| 226530579** Species: *Zea mays*

PFF score: [108]

Protein name: uncharacterized protein LOC100272911

Matched peptides No.: [4] Sequence coverage %: [11]

Matched sequences: K.NAVITVPAYFNDSQR.Q;
R.IINEPTAAAIAYGLDKK.A;
K.ATAGDTHLGGEDFDNR.L;
K.SQIHDVVLVGGSTR.I

Calculated Mr: **62054** Calculated *p*I: **5.23**

Probability Based Mowse Score:

Ions score is -10*Log(P), where P is the probability that the observed match is a random event.
Individual ions scores > 45 indicate identity or extensive homology (p<0.05).
Protein scores are derived from ions scores as a non-probabilistic basis for ranking protein hits.

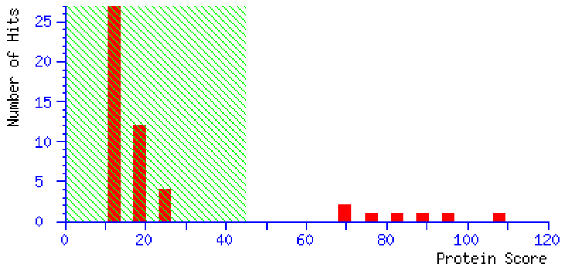

Matched peptide sequences: shown in Bold Red:

```
  1 MKMWPFKVVP GPADKPMIVV TYKGEEKKFS AEEISSMVLT KMKEIAEAYL
 51 STTIKNAVIT VPAYFNDSQR QATKDAGVIA GLNVTRIINE PTAAAIAYGL
101 DKKATSTGEK NVLIFDLGGG TFDVSILTIE EGIFEVKATA GDTHLGGEDF
151 DNRLVNHFVM EFKRKHKKDI SGNPRALRRL RTACERAKRT LSSTAQTTIE
201 IDSLYEGIDF YATITRARFE ELNMDLFRKC MEPVEKCLRD AKMDKSQIHD
251 VVLVGGSTRI PKVQQLLQDF FNGKELCKSI NPDEAVAYGA AVQAAILSGE
301 GNQKVQDLLL LDVTPLSLGL ETAGGVMTVL IPRNTTIPTK KEQVFSTYSD
351 NQPGVLIQVY EGERTRTKDN NLLGKFELTG IPPAPRGVPQ INVTFDIDAN
401 GILNVSAEDK TTGKKNKITI TNDKGRLSKE EIERMVQEAE KYKTEDEEVK
451 RKVEARNALE NYAYNMRNTV RDEKIASKLP ADDKKKIEDT IEDAIKWLDG
501 NQLAEAEEFE DKMKELESIC NPIISQMYQG GAGAAGMDED VPGGGAGNGG
551 GSGAGPKIEE VD
```

Spot No.: **P29**

NCBI accession No.: **gi|149938954** Species: *Actinidia chinensis*

PFF score: [133]

Protein name: G6PD1

Matched peptides No.: [3] Sequence coverage %: [7]

Matched sequences: R.QGFLQSNEVHIFGYAR.T;

R.GGYFDEYGIIR.D;

R.YQGVTIPEAYER.L

Calculated Mr: **59276** Calculated *p*I: **6.75**

Probability Based Mowse Score:

Ions score is -10*Log(P), where P is the probability that the observed match is a random event.
Individual ions scores > 45 indicate identity or extensive homology ($p<0.05$).
Protein scores are derived from ions scores as a non-probabilistic basis for ranking protein hits.

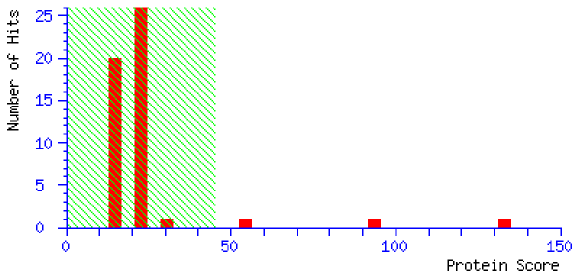

Matched peptide sequences: shown in **Bold Red:**

1 MGSGAWTIEK RSTFRNESFS KEYVTVSETG CLSIIVLGAS GDLAKKKTFP
51 ALFNLYR**QGF** **LQSNEVHIFG** **YAR**TKISDDE LRNRIRGYLI QGSGSSDKQL
101 EEVSKFLQLI KYISGSYDSG EGFQLLDKEI SGYEFSKNTV EGSSRRLFYL
151 ALPPSVYPSV CKMIKNNCMN KSDLGGWTRI VVEKPFGKDL GSAEQLSAQI
201 GELFDEPQIY RIDHYLGKEL VQNLLVLRFA NRFFLPLWNR DNIANVQIVF
251 REDFGTEGR**G** **GYFDEYGIIR** DIIQNHLLQV FCLVAMEKPV SLKPEHIRDE
301 KVKVLQSVVP IKDEEVVLGQ YKGYRDDPTV PDNSNTPTFA TVVLRVHNER
351 WEGVPFILKA GKALNSRKAE IRIQFKDVPG DIFKCQKQGR NEFVIRLQPL
401 EAMYMKLTVK QPGLEMSTVQ SELDLSYGQR **YQGVTIPEAY** **ER**LILDTIRG
451 DQQHFVRRDE LKAAWEIFTP LLHRIDDGEV KPLTYQLGSR GPAEADQLLE
501 RAGYVQTHGY IWIPPTL

Spot No.: **P30**

NCBI accession No.: **gi|226493285** Species: *Zea mays*

PFF score: [53]

Protein name: flavonol 4-sulfotransferase

Matched peptides No.: [12] Sequence coverage %: [38]

Matched sequences: -.MAAAACNPVAPSGPVPFK.D;-.MAAAACNPVAPSGP VPFKDVDDGSVPEHTPK.E;M.AAAACNPVAPSGPVPF;K.DR.LYQGFWLPEHWVPGTIVFQ R.R;R.LYQGFWLPEHWVPGTIVFQRR.F;K.ALAFATAAR.T;R.LAEFMGR.P + Oxidation (M); R.PFSAAEEAAGDVAAVVELCSFDEM;K.G + Oxidation (M);R.YRPMPR.D;K.GVAGDWANH MTPEMAAR.L + 2 Oxidation (M);K.GVAGDWANHMTPEMAARLDGIFR.E;K.LQGTGLIFS.-

Calculated Mr: **38183** Calculated *p*I: **6.26**

Probability Based Mowse Score:

Protein score is -10*Log(P), where P is the probability that the observed match is a random event. Protein scores greater than 73 are significant (p<0.05).

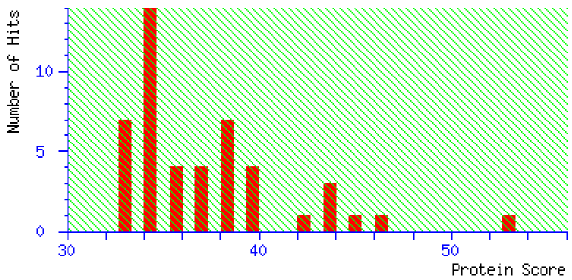

Matched peptide sequences: shown in Bold Red:

```
  1 MAAAACNPVA PSGPVPFKDV DDGSVPEHTP KEEFGDLVAA LPSRQQSILE
 51 LRLYQGFWLP EHWVPGTIVF QRRFTPRPDD VILASYPKCG TTWLKALAFA
101 TAARTAYPPG GARHPLRRLN PHDCVPFIDE IFASGEEAKL EALPSPRLMN
151 THLPYALLPA PVTATATGCR VAYVCRDPKD MVVSLWHFLR RAKPDLLFAD
201 TFESVCDGTV VVGPVWDHVL SYWRASVAAP DRALFLRYED MLRDPGGNVR
251 RLAEFMGRPF SAAEEAAGDV AAVVELCSFD EMKGLEVNRP GSGTAGRYRP
301 MPRDAFFRKG VAGDWANHMT PEMAARLDGI FREKLQGTGL IFS
```

Spot No.: **P31**

NCBI accession No.: **gi|224071449** Species: *Populus trichocarpa*

PFF score: [104]

Protein name: predicted protein

Matched peptides No.: [2] Sequence coverage %: [2]

Matched sequences: K.NKIDVIPHYPGR.L;

K.IDVIPHYPGR.L

Calculated Mr: **47121** Calculated *p*I: **5.28**

Probability Based Mowse Score:

Ions score is -10*Log(P), where P is the probability that the observed match is a random event.
Individual ions scores > 45 indicate identity or extensive homology (p<0.05).
Protein scores are derived from ions scores as a non-probabilistic basis for ranking protein hits.

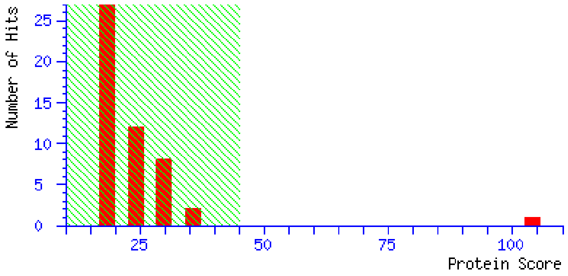

Matched peptide sequences: shown in Bold Red:

```
  1 MDGTSEATWP EILGSRNWDN LLDPLDLSLR KLILRCGDFC QATYDAFNND
 51 QNSRYCGTSR YGKRNFFHKV MLDNPENYQV SSFLYATARV SLPEAFLLHS
101 LSRDSWDRET NWIGYIAVTS DEQTKTLGRR EIYIAFRGTT RNYEWVDILG
151 AKLKSAKPLL RGATSTTHDQ ESSSSSDDDD DKVPKVMLGW LTMYISDDPN
201 SPFTKLSARA QLLAHIKELR ERYKDDDLSI IFTGHSLGAS LSILSAFDLV
251 ENGITDIPVS AFVFGSPQVG NKEFNERFNK YPNLKVLHIK NKIDVIPHYP
301 GRLMGYVYTG IEFEIDTRKS PSLKDSKNPS DWHNLQAMLH IVAGWNGEEQ
351 EFELKVKRSL ALVNKSSEFL KDECLVPGIW WVEKNKGMVR NEDGEWVLAP
401 PDEEDLPVPE C
```

Spot No.: **P32**

NCBI accession No.: gi|1916805 Species: *Hevea brasiliensis*

PFF score: [219]

Protein name: latex patatin homolog

Matched peptides No.: [5] Sequence coverage %: [21]

Matched sequences: K.LQDLDGPDAR.I;

R.DNYDPIHSIGPIYDGEYLR.E;

K.SLDCEDYYLR.I;

R.IQDDTLTGEESSGHIATEENLQR.L;

R.INLDTGRLESIPGAPTNEAAIAK.F

Calculated Mr: **43035** Calculated *p*I: **5.00**

Probability Based Mowse Score:

Ions score is -10*Log(P), where P is the probability that the observed match is a random event.
Individual ions scores > 45 indicate identity or extensive homology (p<0.05).
Protein scores are derived from ions scores as a non-probabilistic basis for ranking protein hits.

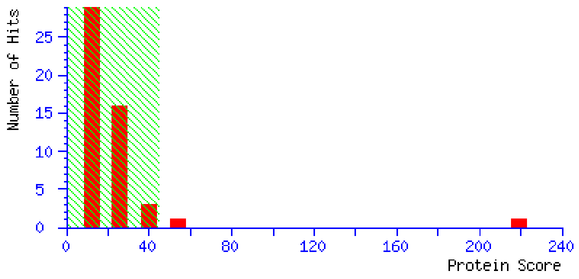

Matched peptide sequences: shown in Bold Red:

```
  1 MATGSTTLTQ GKKITVLSID GGGIRGIIPG IILASLESKL QDLDGPDARI
 51 ADYFDIIAGT STGGLITTML TAPNEDKKPM YQAKDIKDFY LENCPKIFPK
101 ESRDNYDPIH SIGPIYDGEY LRELCNNLLK DLTVKDTSTD VIIPTFDIKL
151 LLPVIFPSDD AKCNALKNAR LADVCISTSA APVLLPAHSF TTEDDKNIHT
201 FELIDGGVAA TNPTLLALTH IRNEIIRQNP RFIGANLTES KSRLVLSLGT
251 GKSEYKEKYN ADMTSKWRLY NWALYNGNSP AVDIFSNASS DMVDSHLSAL
301 FKSLDCEDYY LRIQDDTLTG EESSGHIATE ENLQRLVEIG TELLEKQESR
351 INLDTGRLES IPGAPTNEAA IAKFAKLLSE ERKLRQLK
```

Spot No.: **P33**

NCBI accession No.: **gi|6707018** Species: *Hevea brasiliensis*

PFF score: [196]

Protein name: latex protein allergen Hev b 7

Matched peptides No.: [5] Sequence coverage %: [22]

Matched sequences: K.LQDLDGPDAR.I;
R.DNYDPIHSIGPIYDGEYLR.E;
K.LLLPVIFSSDDAK.C;
R.IQDDTLTGEESSGHIATEENLQR.L;
R.INLDTGRLESIPGAPTNEAAIAK.F

Calculated Mr: **43107** Calculated *p*I: **5.00**

Probability Based Mowse Score:

Ions score is -10*Log(P), where P is the probability that the observed match is a random event.
Individual ions scores > 45 indicate identity or extensive homology (p<0.05).
Protein scores are derived from ions scores as a non-probabilistic basis for ranking protein hits.

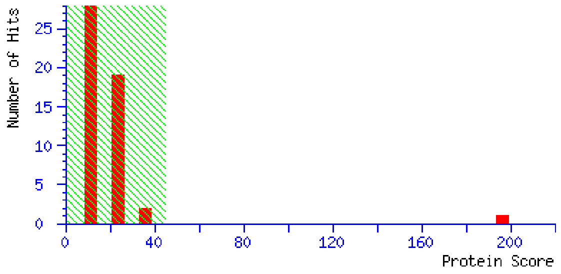

Matched peptide sequences: shown in **Bold Red:**

1 MATGSTPLTQ GKKITVLSID GGGIRGIIPG IILASLESK**L** **QDLDGPDAR**I
51 ADYFDIIAGT STGGLITTML TAPNEDKKPM YQAKDIKDFY LENCPKIFPK
101 ESR**DNYDPIH** **SIGPIYDGEY** **LR**ELCNNLLK DLTVKDTLTD VIIPTFDIK**L**
151 **LLPVIFSSDD** **AK**CNALKNAR LADVCISTSA APVLLPAHSF TTEDDKNIHT
201 FELIDGGVAA TNPTLLALTH IRNEIIRQNP RFIGANLTES KSRLVLSLGT
251 GKSEYKEKYN ADMTSKWRLY NWALYNGNSP AVDIFSNASS DMVDFHLSAL
301 FKSLDCEDYY LR**IQDDTLTG** **EESSGHIATE** **ENLQR**LVEIG TELLEKQESR
351 **INLDTGRLES** **IPGAPTNEAA** **IAK**FAKLLSE ERKLRQLK

Spot No.: **P34**

NCBI accession No.: **gi|32186890** Species: *Gossypium hirsutum*

PFF score: [392]

Protein name: actin

Matched peptides No.: [7] Sequence coverage %: [29]

Matched sequences: K.IWHHTFYNELR.V;
R.VAPEEHPVLLTEAPLNPK.A;
R.TTGIVLDSGDGVSHTVPIYEGYALPHAILR.L;
K.NYELPDGQVITIGAER.F;
R.KDLYGNIVLSGGSTMFPGIADR.M + Oxidation (M);
K.DLYGNIVLSGGSTMFPGIAD;
R.M + Oxidation (M);
K.GEYDESGPSIVHR.K

Calculated Mr: **41917** Calculated *p*I: **5.31**

Probability Based Mowse Score:

Ions score is -10*Log(P), where P is the probability that the observed match is a random event.
Individual ions scores > 45 indicate identity or extensive homology (p<0.05).
Protein scores are derived from ions scores as a non-probabilistic basis for ranking protein hits.

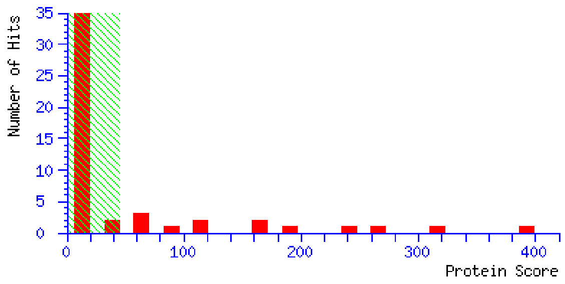

Matched peptide sequences: shown in Bold Red:

```
  1 MADGEDIQPL VCDNGTGMVK AGFAGDDAPR AVFPSIVGRP RHTGVMVGMG
 51 QKDAYVGDEA QSKRGILTLK YPIEHGIVSN WDDMEKIWHH TFYNELRVAP
101 EEHPVLLTEA PLNPKANREK MTQIMFETFN VPAMYVAIQA VLSLYASGRT
151 TGIVLDSGDG VSHTVPIYEG YALPHAILRL DLAGRDLTDA LMKILTERGY
201 MFTTTAEREI VRDMKEKLAY VALDYEQELE TAKSSSSVEK NYELPDGQVI
251 TIGAERFRCP EVLFQPSFIG MEAAGIHETT YNSIMKCDVD IRKDLYGNIV
301 LSGGSTMFPG IADRMSKEIT ALAPSSMKIK VVAPPERKYS VWIGGSILAS
351 LSTFQQMWIS KGEYDESGPS IVHRKCF
```

Spot No.: **P35**

NCBI accession No.: gi|255552291   Species: *Ricinus communis*

PFF score: [141]

Protein name: short chain dehydrogenase, putative

Matched peptides No.: [2]   Sequence coverage %: [10]

Matched sequences: K.VALVTGGDSGIGR.A;

K.DPIAIPTDVGFEENCR.K

Calculated Mr: **32148**   Calculated *p*I: **6.50**

Probability Based Mowse Score:

Ions score is -10*Log(P), where P is the probability that the observed match is a random event.
Individual ions scores > 44 indicate identity or extensive homology ($p<0.05$).
Protein scores are derived from ions scores as a non-probabilistic basis for ranking protein hits.

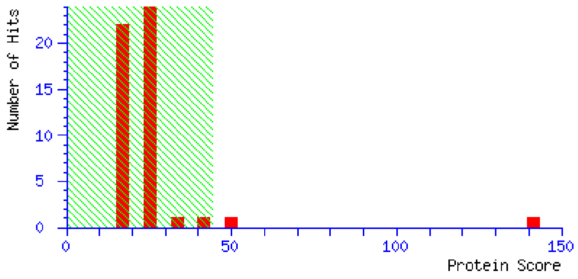

Matched peptide sequences: shown in Bold Red:

1 MATRRGYRFP PQSQDKQPGK EYLMHPLPEF INPHYKPSNK LQDK**VALVTG**
51 **GDSGIGR**AVS YYFTLEGATV AFTYVKGRED KDKDHILKIL HEVKAEGAK**D**
101 **PIAIPTDVGF EENCR**KVIDQ IMSEYGKIDI LVNNAGEAHY STTIEDITDP
151 MLERVFRTNI FGHFFMSRHA LKHMKEGGCI INTASVAAYA GFSFMVDYSS
201 TKGAVVAFTR SLALQLIDRG IRVNAVAPGS VWTPFQPSTL SAEQVAQVGS
251 GVPMDRAAQP YEIAPSFVFL ASNDCSSYFT GQVLHPNGMF

Spot No.: **P36**

NCBI accession No.: **gi|38122474** Species: *Hevea brasiliensis*

PFF score: [393]

Protein name: Rubber elongation factor protein

Matched peptides No.: [9] Sequence coverage %: [26]

Matched sequences:

Query	Start - End	Observed	Mr(expt)	Mr(calc)	ppm	M	Score	Expect	Rank	U	Peptide
95	57 - 69	1516.7605	1515.7532	1515.7722	-12.5	0	103	1.1e-07	1	U	K.YLDFVQAATVYAR.A
96	57 - 69	1516.8470	1515.8397	1515.7722	44.6	0	110	2.4e-08	1	U	K.YLDFVQAATVYAR.A
83	83 - 96	1474.7504	1473.7431	1473.7576	-9.82	1	53	0.012	1	U	K.SGPFKPGVNTVESR.F
84	83 - 96	1474.8275	1473.8202	1473.7576	42.5	1	112	1.6e-08	1	U	K.SGPFKPGVNTVESR.F
13	99 - 107	1061.6001	1060.5928	1060.6029	-9.52	1	17	57	2	U	K.SVVRPVYNK.F
14	99 - 107	1061.6669	1060.6596	1060.6029	53.5	1	41	0.23	1	U	K.SVVRPVYNK.F
65	122 - 132	1306.7577	1305.7504	1305.7041	35.5	1	64	0.00089	1	U	R.RVDAYVTVLDR.I
27	123 - 132	1150.6016	1149.5943	1149.6030	-7.54	0	53	0.012	1	U	R.VDAYVTVLDR.I
28	123 - 132	1150.6576	1149.6503	1149.6030	41.2	0	66	0.00054	1	U	R.VDAYVTVLDR.I

Calculated Mr: **19570** Calculated *p*I: **5.06**

Probability Based Mowse Score:

Ions score is -10*Log(P), where P is the probability that the observed match is a random event.
Individual ions scores > 44 indicate identity or extensive homology (p<0.05).
Protein scores are derived from ions scores as a non-probabilistic basis for ranking protein hits.

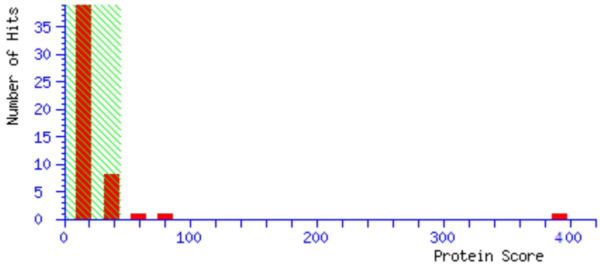

Matched peptide sequences: shown in **Bold Red:**

```
  1 MAEGEEEVNI QEEANKGEEN PQEEANIQEE TNKGEENIQE EANIEEEANK
 51 EEESLKYLDF VQAATVYARA SFSKLYLFAK DKSGPFKPGV NTVESRFKSV
101 VRPVYNKFQP VPNKVLKFAD RRVDAYVTVL DRIVPPIVKR ASIQAYSVAP
151 GAALAVASYL PLHTKRLSKV LYGDG
```

Spot No.: P37

NCBI accession No.: **gi|334854634** Species: *Hevea brasiliensis*

PFF score: [288] Protein name: elicitor-responsive protein

Matched peptides No.: [3] Sequence coverage %: [26]

Matched sequences:

Start - End	Observed	Mr(expt)	Mr(calc)	ppm	Miss	Sequence
2 - 14	1295.8593	1294.8520	1294.7860	51	0	M.PLGTVEVLLVGAK.G (Ions score 90)
47 - 55	1075.5344	1074.5271	1074.4618	61	0	K.GSEPEWNEK.F (Ions score 53)
56 - 70	1687.8898	1686.8825	1686.7989	50	0	K.FSFEVSDGDTELTLK.I (Ions score 145)

Calculated Mr: **15249** Calculated *p*I: **4.06**

Probability Based Mowse Score:

Ions score is -10*Log(P), where P is the probability that the observed match is a random event.
Individual ions scores > 44 indicate identity or extensive homology (p<0.05).
Protein scores are derived from ions scores as a non-probabilistic basis for ranking protein hits.

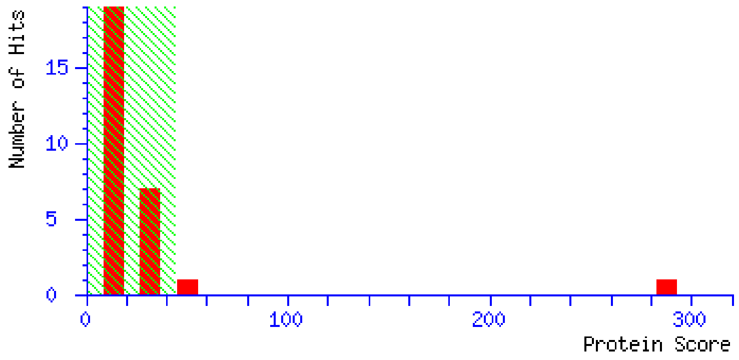

Matched peptide sequences: shown in Bold Red

```
  1 MPLGTVEVLL VGAKGLENTD FLNGVDPYVV LACRTQEQKS SVASGKGSEP
 51 EWNEKFSFEV SDGDTELTLK IMDSDVGAAD DFVGEATIPL EPLFLEGNLP
101 STAYKVVKEQ EYKGEITVGL TFTPEVEMDN VGVDGYDFRL
```

Spot No.: **P38**

NCBI accession No.: **gi| 14423933** Species: *Hevea brasiliensis*

PFF score: [353]

Protein name: Small rubber particle protein

Matched peptides No.: [9] Sequence coverage %: [38]

Calculated Mr: **22331** Calculated *p*I: **4.80**

Matched sequences:

Query	Start – End	Observed	Mr(expt)	Mr(calc)	ppm	M	Score	Expect	Rank	U	Peptide
10	6 – 20	1865.7990	1864.7917	1864.7711	11.1	0	96	4.2e-07	1	U	K.ENENFQQEANEQEEK.L
11	6 – 20	1865.7990	1864.7917	1864.7711	11.1	0	96	4.2e-07	1	U	K.ENENFQQEANEQEEK.L
12	6 – 20	1865.9290	1864.9217	1864.7711	80.8	0	98	2.6e-07	1	U	K.ENENFQQEANEQEEK.L
7	47 – 64	1810.9230	1809.9157	1809.9472	-17.4	1	110	2.3e-08	1	U	K.DNSGPLKPGVETIEGVAK.T
8	47 – 64	1811.0605	1810.0532	1809.9472	58.6	1	153	1.4e-12	1	U	K.DNSGPLKPGVETIEGVAK.T
9	47 – 64	1811.1030	1810.0957	1809.9472	82.0	1	142	1.6e-11	1	U	K.DNSGPLKPGVETIEGVAK.T
2	102 – 113	1347.6600	1346.6527	1346.6500	2.00	0	25	7.6	1	U	K.QLPTQACDTSVK.E
3	102 – 113	1347.6600	1346.6527	1346.6500	2.00	0	25	7.6	1	U	K.QLPTQACDTSVK.E
4	102 – 113	1347.7468	1346.7395	1346.6500	66.5	0	102	1.4e-07	1	U	K.QLPTQACDTSVK.E

Probability Based Mowse Score:

Ions score is -10*Log(P), where P is the probability that the observed match is a random event.
Individual ions scores > 46 indicate identity or extensive homology (p<0.05).
Protein scores are derived from ions scores as a non-probabilistic basis for ranking protein hits.

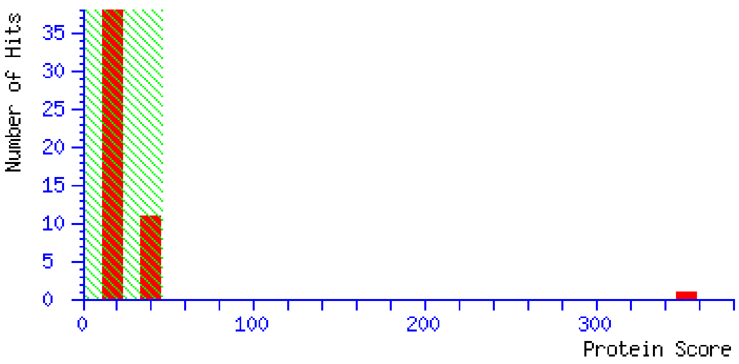

Matched peptide sequences: shown in Bold Red:

```
  1 MAEGKENENF QQEANEQEEK LKYLEFVQAT TDNAVTALSN IYLYAKDNSG
 51 PLKPGVETIE GVAKTVVIPA SKIPTEAIKF ADRAVDASFT TLQNIVPSVL
101 KQLPTQACDT SVKESAE
```

Spot No.: **P39**

NCBI accession No.: **gi| 14423933** Species: *Hevea brasiliensis*

PFF score: [342]

Protein name: Small rubber particle protein

Matched peptides No.: [7] Sequence coverage %: [36]

Calculated Mr: **22331** Calculated *p*I: **4.80**

Matched sequences:

Query	Start - End	Observed	Mr(expt)	Mr(calc)	ppm	M	Score	Expect	Rank	U	Peptide
25	36 - 53	1881.1389	1880.1316	1880.0255	56.4	0	57	0.0022	1	U	K.DISGPLKPGVDTIENVVK.T
11	91 - 105	1620.8983	1619.8910	1619.7903	62.2	0	102	8.7e-08	1	U	K.QVSAQTYSVAQDAPR.I
27	106 - 124	1934.1237	1933.1164	1933.0157	52.1	0	87	2e-06	1	U	R.IVLDVASSVFNTGVQEGAK.A
28	106 - 124	1934.1260	1933.1187	1933.0157	53.3	0	108	1.7e-08	1	U	R.IVLDVASSVFNTGVQEGAK.A
29	106 - 124	1934.1260	1933.1187	1933.0157	53.3	0	108	1.7e-08	1	U	R.IVLDVASSVFNTGVQEGAK.A
2	134 - 143	1236.7086	1235.7013	1235.6299	57.8	0	59	0.0024	1	U	K.AEQYAVITWR.A
4	183 - 194	1346.8149	1345.8076	1345.7493	43.3	0	16	44	1	U	R.VSSYLPLLPTEK.I

Probability Based Mowse Score:

Ions score is -10*Log(P), where P is the probability that the observed match is a random event.
Individual ions scores > 44 indicate identity or extensive homology (p<0.05).
Protein scores are derived from ions scores as a non-probabilistic basis for ranking protein hits.

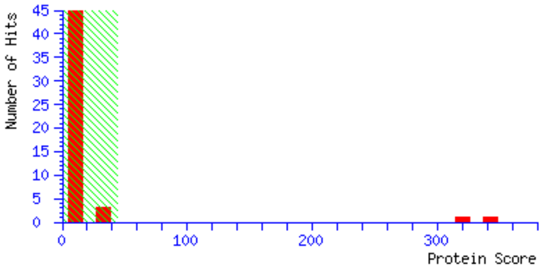

Matched peptide sequences: shown in Bold Red:

```
  1 MAEEVEEERL KYLDFVRAAG VYAVDSFSTL YLYAKDISGP LKPGVDTIEN
 51 VVKTVVTPVY YIPLEAVKFV DKTVDVSVTS LDGVVPPVIK QVSAQTYSVA
101 QDAPRIVLDV ASSVFNTGVQ EGAKALYANL EPKAEQYAVI TWRALNKLPL
151 VPQVANVVVP TAVYFSEKYN DVVRGTTEQG YRVSSYLPLL PTEKITKVFG
201 DEAS
```

Spot No.: **P40**

NCBI accession No.: **gi| 29170601** Species: *Hevea brasiliensis*

PFF score: [316]

Protein name: Small rubber particle protein

Matched peptides No.: [12] Sequence coverage %: [53]

Calculated Mr: **12769** Calculated *p*I: **4.61**

Matched sequences:

Query	Start - End	Observed	Mr(expt)	Mr(calc)	ppm	M	Score	Expect	Rank	U	Peptide
13	6 - 20	1865.7990	1864.7917	1864.7711	11.1	0	55	0.0043	1	U	K.ENENFQQEANEQEEK.L
14	6 - 20	1865.7990	1864.7917	1864.7711	11.1	0	55	0.0043	1	U	K.ENENFQQEANEQEEK.L
15	6 - 20	1865.9290	1864.9217	1864.7711	80.8	0	48	0.021	1	U	K.ENENFQQEANEQEEK.L
8	47 - 64	1810.9230	1809.9157	1809.9472	-17.4	0	54	0.0051	1	U	K.DNSGPLKPGVETIEGVAK.T
9	47 - 64	1810.9230	1809.9157	1809.9472	-17.4	0	54	0.0051	1	U	K.DNSGPLKPGVETIEGVAK.T
10	47 - 64	1810.9230	1809.9157	1809.9472	-17.4	0	54	0.0051	1	U	K.DNSGPLKPGVETIEGVAK.T
11	47 - 64	1811.0624	1810.0551	1809.9472	59.6	0	121	9.1e-10	1	U	K.DNSGPLKPGVETIEGVAK.T
12	47 - 64	1811.1030	1810.0957	1809.9472	82.0	0	36	0.31	1	U	K.DNSGPLKPGVETIEGVAK.T
16	84 - 101	1902.9020	1901.8947	1902.0462	-79.7	0	56	0.0028	1	U	R.AVDASFTTLQNIVPSVLK.Q
1	102 - 113	1347.6600	1346.6527	1346.6500	2.00	0	6	3.8e+02	5	U	K.QLPTQACDTSVK.E
2	102 - 113	1347.6600	1346.6527	1346.6500	2.00	0	6	3.8e+02	5	U	K.QLPTQACDTSVK.E
3	102 - 113	1347.7456	1346.7383	1346.6500	65.6	0	84	6.8e-06	1	U	K.QLPTQACDTSVK.E

Probability Based Mowse Score:

Ions score is -10*Log(P), where P is the probability that the observed match is a random event.
Individual ions scores > 44 indicate identity or extensive homology (p<0.05).
Protein scores are derived from ions scores as a non-probabilistic basis for ranking protein hits.

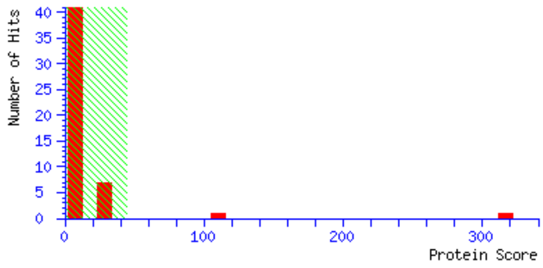

Matched peptide sequences: shown in Bold Red:

```
  1 MAEGKENENF QQEANEQEEK LKYLEFVQAT TDNAVTALSN IYLYAKDNSG
 51 PLKPGVETIE GVAKTVVIPA SKIPTEAIKF ADRAVDASFT TLQNIVPSVL
101 KQLPTQACDT SVKESAE
```

Spot No.: **P41**

NCBI accession No.: **gi|2832430** Species: *Hevea brasiliensis*

PFF score: [261]

Protein name: prohevein

Matched peptides No.: [3] Sequence coverage %: [26]

Matched sequences: K.YGWTAFCGPVGAHGQPSCGK.C;

R.IVDQCSNGGLDLDVNVFR.Q;

R.QLDTDGKGYER.G

Calculated Mr: **20852** Calculated *p*I: **5.64**

Probability Based Mowse Score:

Ions score is -10*Log(P), where P is the probability that the observed match is a random event.
Individual ions scores > 44 indicate identity or extensive homology (p<0.05).
Protein scores are derived from ions scores as a non-probabilistic basis for ranking protein hits.

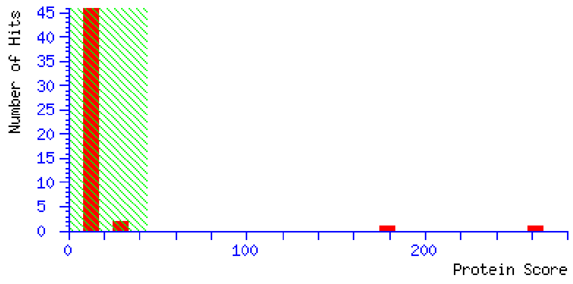

Matched peptide sequences: shown in Bold Red:

```
  1 EQCGRQAGGK LCPNNLCCSQ WGWCGSTDEY CSPDHNCQSN CKDSGEGVGG
 51 GSASNVLATY HLYNSQDHGW DLNAASAYCS TWDANKPYSW RSKYGWTAFC
101 GPVGAHGQPS CGKCLSVTNT GTGAKATVRI VDQCSNGGLD LDVNVFRQLD
151 TDGKGYERGH LTVNYQFVDC GDSFNPLFSV MKSSVIN
```

Spot No.: **P42 (S9)**

NCBI accession No.: **gi|388503664** Species: *Lotus japonicus*

Protein name： **unknown**

Mascot score: **93** Sequence coverage %: **45**

Matched peptides No.: **13** Total peptides No.: **48**

Calculated Mr: **29537** Calculated *p*I: **4.79**

Probability Based Mowse Score:

Protein score is -10*Log(P), where P is the probability that the observed match is a random event.
Protein scores greater than 73 are significant (p<0.05).

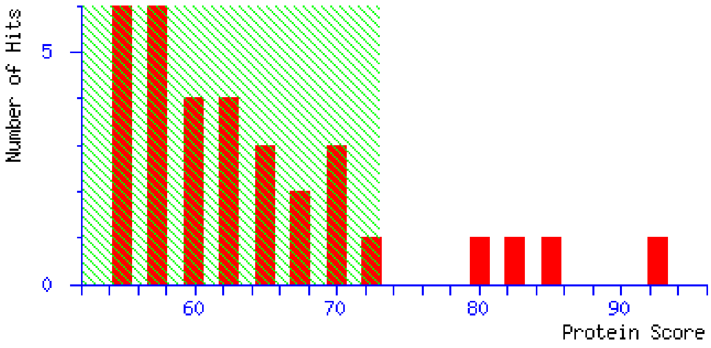

Matched peptide sequences: shown in Bold Red:

```
  1 MASSKDRGTF VYVAKLAEQA ERYEEMVDSM KKVANLDVEL TVEERNLLSV
 51 GYKNVIGARR ASWRILSSIE QKEETKGNDV NAKRIKEYRQ KVESELADIC
101 NDVMRVIDEH LIPSATAGES TVFYYKMKGD YYRYLAEFKS GNEKKEAADQ
151 SMKAYESATT AAEAELPPTH PIRLGLALNF SVFYYEILNS PERACHLAKQ
201 AFDEAISELD TLNEESYKDS TLIMQLLRDN LTLWTSDIPE EGEDSLKAND
251 TAKVGGGEDA E
```

Spot No.: **P43**

NCBI accession No.: **gi|326694865** Species: *Hevea brasiliensis*

PFF score: [294]

Protein name: 14-3-3 protein 2

Matched peptides No.: [7] Sequence coverage %: [32]

Matched sequences:

Query	Start – End	Observed	Mr(expt)	Mr(calc)	ppm	M	Score	Expect	Rank	U	Peptide
38	17 – 33	2131.7244	2130.7171	2130.9601	-114	1	4	5e+02	3		K.LAEQAERYEEMVEFMEK.V
16	24 – 33	1334.4153	1333.4080	1333.5570	-112	0	21	12	1		R.YEEMVEFMEK.V
20	37 – 48	1418.5446	1417.5373	1417.6936	-110	0	84	6.7e-06	1		K.TVDVEELTVEER.N
6	80 – 89	1125.4647	1124.4574	1124.5826	-111	0	28	2.7	1		R.GNEDHVTIIK.E
33	160 – 176	1788.7595	1787.7522	1787.9530	-112	0	78	2e-05	1		K.SAQDIALADLAPTHPIR.L
40	177 – 196	2330.9053	2329.8980	2330.1947	-127	0	30	1.3	1		R.LGLALNFSVFYYEILNSPDR.A
9	222 – 231	1189.5359	1188.5286	1188.6536	-105	0	50	0.02	2		K.DSTLIMQLLR.D

Calculated Mr: **29931** Calculated *p*I: **4.76**

Probability Based Mowse Score:

Ions score is -10*Log(P), where P is the probability that the observed match is a random event.
Individual ions scores > 45 indicate identity or extensive homology (p<0.05).
Protein scores are derived from ions scores as a non-probabilistic basis for ranking protein hits.

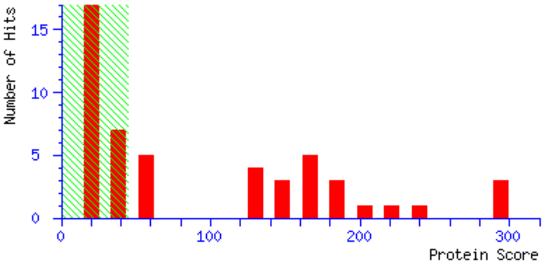

Matched peptide sequences: shown in Bold Red:

```
  1 MSPTESSREE NVYMAKLAEQ AERYEEMVEF MEKVAKTVDV EELTVEERNL
 51 LSVAYKNVIG ARRASWRIIS SIEQKEESRG NEDHVTIIKE YRGKIESELS
101 KICDGILSLL ESHLIPSASS AESKVFYLKM KGDYHRYLAE FKTAGERKEA
151 AESTLLAYKS AQDIALADLA PTHPIRLGLA LNFSVFYYEI LNSPDRACNL
201 AKQAFDEAIS ELDTLGEESY KDSTLIMQLL RDNLTLWTSD ITDEAGDEIK
251 EASKRESGEG QPQQ
```

Spot No.: P44 (S85)

NCBI accession No.: **gi|224099853** Species: ***Populus trichocarpa***

PFF score: [69] Protein name: **putative protein**

Matched peptides No.: [2] Sequence coverage %: [5]

Matched sequences: S FEHPLNELKP VPR

Calculated Mr: **27448** Calculated *p*I: **5.84**

Probability Based Mowse Score:

Ions score is -10*Log(P), where P is the probability that the observed match is a random event.
Individual ions scores > 45 indicate identity or extensive homology (p<0.05).
Protein scores are derived from ions scores as a non-probabilistic basis for ranking protein hits.

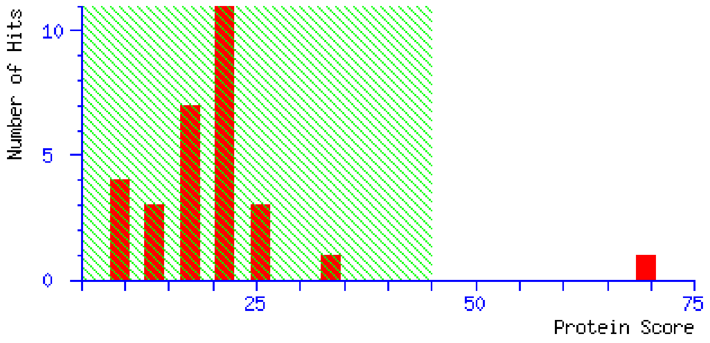

Matched peptide sequences: shown in Bold Red

```
  1 MLAVFDNTVA KCPDALQSPH SAPASSALKD GFLANHFASQ HPGSVTVNLG
 51 TSGLISHSVE KQNPFLPRLF AVVDDIFCLF QGHIDNVAVL KQQYGLNKTA
101 NEVIVVIEAY RTLRDRGPYP ADQVVKDIQG KFAFILYDST SKATFFAADA
151 DGSVPFFWGA DSEGNLVLSD DVQIVQKGCG KSFAPFPKGC FFTTSGGLRS
201 FEHPLNELKP VPRVDSSGQV CGATFKVDAE TKKESVGMPR VDSSYNWSSN
251 Y
```

Spot No.: P45

NCBI accession No.: **gi|124365253** Species: ***Hevea brasiliensis***

PFF score: [745] Protein name: beta-1,3-glucanase

Matched peptides No.: [16] Sequence coverage %: [44]

Matched sequences:

Query	Start – End	Observed	Mr(expt)	Mr(calc)	ppm	M	Score	Expect	Rank	U	Peptide
10	69 – 81	1501.8600	1500.8527	1500.7936	39.4	0	82	1.1e-05	1	U	R.IYDPNQAVLEALR.G
11	69 – 81	1501.9630	1500.9557	1500.7936	108	0	66	0.00036	1	U	R.IYDPNQAVLEALR.G
12	69 – 81	1502.0040	1500.9967	1500.7936	135	0	78	2.7e-05	1	U	R.IYDPNQAVLEALR.G
20	82 – 106	2581.6631	2580.6558	2580.3395	123	0	78	2e-05	1		R.GSNIELILGVPNSDLQSLTNPSNAK.S
1	115 – 121	838.5450	837.5377	837.4133	149	0	27	3.9	1	U	R.GFWSSVR.F
9	124 – 136	1431.9120	1430.9047	1430.7518	107	0	65	0.0006	1		R.YIAVGNEISPVNR.G
17	167 – 188	2236.2949	2235.2876	2235.1535	60.0	0	85	3.5e-06	1		K.VSTAIDLTLVGNSYPPSAGAFR.D
18	167 – 188	2236.3291	2235.3218	2235.1535	75.3	0	125	3.9e-10	1		K.VSTAIDLTLVGNSYPPSAGAFR.D
3	193 – 200	975.6890	974.6818	974.5549	130	0	24	11	1		R.SYLNPIIR.F
4	193 – 200	975.7050	974.6977	974.5549	147	0	36	0.68	1		R.SYLNPIIR.F
5	193 – 200	975.7600	974.7527	974.5549	203	0	46	0.065	1		R.SYLNPIIR.F
19	225 – 245	2350.3850	2349.3777	2349.2005	75.4	0	30	1.1	1		R.DISLPYALFTSPSVVVWDGQR.G
15	249 – 264	1812.0800	1811.0727	1810.9101	89.8	0	104	4.3e-08	1	U	K.NLFDATLDALYSALER.A
21	265 – 293	2826.4260	2825.4187	2825.3257	32.9	0	135	2.3e-11	1		R.ASGGSLEVVVSESGWPSAGAFAATFDNGR.T
7	294 – 304	1315.9490	1314.9417	1314.7296	161	0	30	1.8	1		R.TYLSNLIQHVK.R
8	294 – 304	1316.0090	1315.0017	1314.7296	207	0	55	0.0051	1		R.TYLSNLIQHVK.R

Calculated Mr: **41543** Calculated *p*I: **9.40**

Probability Based Mowse Score:

Ions score is -10*Log(P), where P is the probability that the observed match is a random event.
Individual ions scores > 44 indicate identity or extensive homology (p<0.05).
Protein scores are derived from ions scores as a non-probabilistic basis for ranking protein hits.

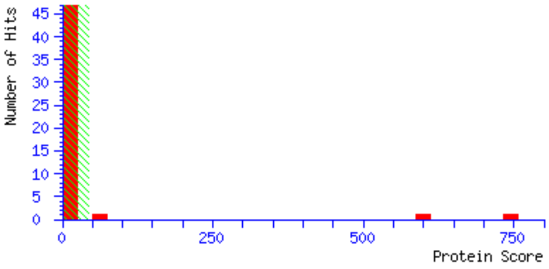

Matched peptide sequences: shown in Bold Red

1 MAISSSTSGT SSSLPSRTTV MLLLFFFTAS VGITDAQVGV CYGMQGNNLP
51 PVSEVIALYK KSNITRMRIY DPNQAVLEAL RGSNIELILG VPNSDLQSLT
101 NPSNAKSWVQ KNVRGFWSSV RFRYIAVGNE ISPVNRGTAW LAQFVLPAMR
151 NIHDAIRSAG LQDQIKVSTA IDLTLVGNSY PPSAGAFRDD VRSYLNPIIR
201 FLSSIRSPLL ANIYPYFTYA GNPRDISLPY ALFTSPSVVV WDGQRGYKNL
251 FDATLDALYS ALERASGGSL EVVVSESGWP SAGAFAATFD NGRTYLSNLI
301 QHVKRGTPKR PKRAIETYLF AMFDENKKQP EVEKHFGLFF PNKWQKYNLN
351 FSAEKNWDIS TEHNATILFL KSDM

Spot No.: **P46**

NCBI accession No.: **gi| 224102193**

Species: ***Populus trichocarpa***

PFF score: [347]

Protein name: NAD-dependent malate dehydrogenase

Matched peptides No.: [5]   Sequence coverage %: [19]

Matched sequences:

Query	Start – End	Observed	Mr(expt)	Mr(calc)	ppm	M	Score	Expect	Rank	U	Peptide
13	127 – 142	1650.0818	1649.0745	1648.9876	52.7	0	30	1.2	1		K.VLVVANPANTNALILK.E
20	212 – 231	2434.3748	2433.3675	2433.2176	61.6	1	191	7.2e-17	1	U	R.ELVKDDEWLNAEFITTVQQR.G
18	216 – 231	1965.0579	1964.0506	1963.9276	62.7	0	50	0.012	1	U	K.DDEWLNAEFITTVQQR.G
15	240 – 256	1774.0004	1772.9931	1772.8839	61.6	1	67	0.00026	1		R.KLSSALSAASSACDHIR.D
11	312 – 324	1490.8445	1489.8372	1489.7511	57.8	1	9	1.8e+02	1		K.KLDLTADELSEEK.A

Calculated Mr: **36093**   Calculated *p*I: **6.11**

Probability Based Mowse Score:

Ions score is -10*Log(P), where P is the probability that the observed match is a random event.
Individual ions scores > 44 indicate identity or extensive homology (p<0.05).
Protein scores are derived from ions scores as a non-probabilistic basis for ranking protein hits.

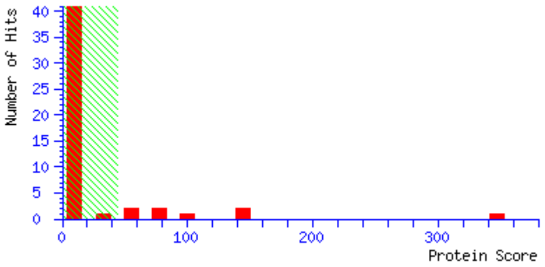

Matched peptide sequences: shown in **Bold Red:**

```
  1 MAKEPVRVLV TGAAGQIGYA LVPMIARGVM LGPDQPVILH MLDIPPAAEA
 51 LNGVKMELVD AAFPLLKGVV ATTDVVEACT GVNIAVMVGG FPRKEGMERK
101 DVMSKNVSIY KSQASALEKH AAANCKVLVV ANPANTNALI LKEFAPSISE
151 KNITCLTRLD HNRALGQISE RLSVQVCDVK NVIIWGNHSS SQYPDVNHAT
201 VKTPAGEKPV RELVKDDEWL NAEFITTVQQ RGAAIIKARK LSSALSAASS
251 ACDHIRDWVL GTPEGTWVSM GVYSDGSYNV PAGLIYSFPV TCQNGEWKIV
301 QGLSIDEFSR KKLDLTADEL SEEKALAYSC LS
```

Spot No.: **P47**

NCBI accession No.: **gi| 255578278**

Species: ***Ricinus communis***

PFF score: [75]

Protein name: **hypothetical protein RCOM_053778**

Matched peptides No.: [2] Sequence coverage %: [6]

Matched sequences: **K.QESEYGSGGYGR.R; R.KPSYGEEGGYGER.T**

Calculated Mr: **41389** Calculated *p*I: **4.65**

Probability Based Mowse Score:

Ions score is -10*Log(P), where P is the probability that the observed match is a random event.
Individual ions scores > 44 indicate identity or extensive homology ($p<0.05$).
Protein scores are derived from ions scores as a non-probabilistic basis for ranking protein hits.

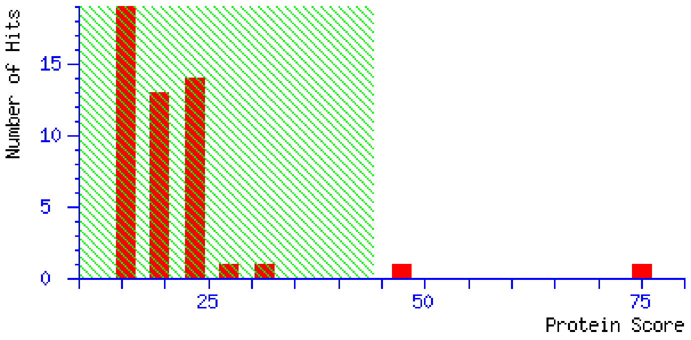

Matched peptide sequences: shown in Bold Red:

1 MPYYTRSEDD VNDFDEFDPT PYGGGYDLAL TYGRPLPPSD ETCYQNSAIA
51 DDDVDYDRPN FTSYAEPSAY NDDILQEEYN SYSRPKPRPG FIPGGGIGGD
101 PYSRPHAAPG FQPGSGGYGG VSEYEKPSGY GRRPDSEYGS GGYGGGTEYE
151 RPSGEDYGSG HGRKQDSEYG SGGYGRRPES EYGSGGGYER PSSEEYGSGY
201 GRK**QESEYGS** **GGYGR**RPEAE YGSGYGGRPE TEYGGGGGSE YGGGYGR**KPS**
251 **YGEEGGYGER** TEYERPSYGD DPPRRPGYGR QDDEYERPSY ERKSDDDNDG
301 SRRKYGGDDD EKGYQKKYGG YGDDDDEEGS RRKYGYGGEE ESYGRKKYGD
351 DGSDDDEKKH HRHKHHQHKH SDDE

Spot No.: **P48**

NCBI accession No.: **gi|255552291** Species: *Ricinus communis*

PFF score: [201]

Protein name: short chain dehydrogenase, putative

Matched peptides No.: [2] Sequence coverage %: [7]

Matched sequences: K.AEGAKDPIAIPTDVGFEENCR.K;

K.DPIAIPTDVGFEENCR.K

Calculated Mr: **32148** Calculated *p*I: **6.50**

Probability Based Mowse Score:

Ions score is -10*Log(P), where P is the probability that the observed match is a random event.
Individual ions scores > 45 indicate identity or extensive homology (p<0.05).
Protein scores are derived from ions scores as a non-probabilistic basis for ranking protein hits.

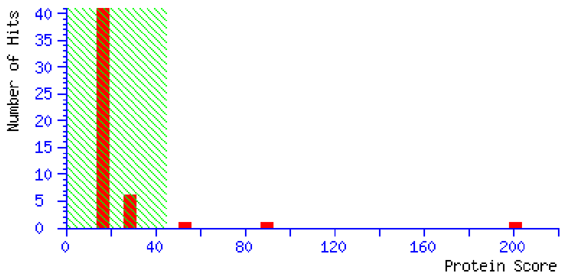

Matched peptide sequences: shown in Bold Red:

```
  1 MATRRGYRFP PQSQDKQPGK EYLMHPLPEF INPHYKPSNK LQDKVALVTG
 51 GDSGIGRAVS YYFTLEGATV AFTYVKGRED KDKDHILKIL HEVKAEGAKD
101 PIAIPTDVGF EENCRKVIDQ IMSEYGKIDI LVNNAGEAHY STTIEDITDP
151 MLERVFRTNI FGHFFMSRHA LKHMKEGGCI INTASVAAYA GFSFMVDYSS
201 TKGAVVAFTR SLALQLIDRG IRVNAVAPGS VWTPFQPSTL SAEQVAQVGS
251 GVPMDRAAQP YEIAPSFVFL ASNDCSSYFT GQVLHPNGMF
```

Spot No.: **P49**

NCBI accession No.: **gi|255581753** Species: *Ricinus communis*

PFF score: [175]

Protein name: adenosine kinase, putative

Matched peptides No.: [3] Sequence coverage %: [11]

Matched sequences: K.RPENWALVEK.A;

K.VLPYMDYVFGNETEAR.T + Oxidation (M);

R.AGCYAANVIIQR.S

Calculated Mr: **38058** Calculated *p*I: **5.17**

Probability Based Mowse Score:

Ions score is -10*Log(P), where P is the probability that the observed match is a random event.
Individual ions scores > 45 indicate identity or extensive homology (p<0.05).
Protein scores are derived from ions scores as a non-probabilistic basis for ranking protein hits.

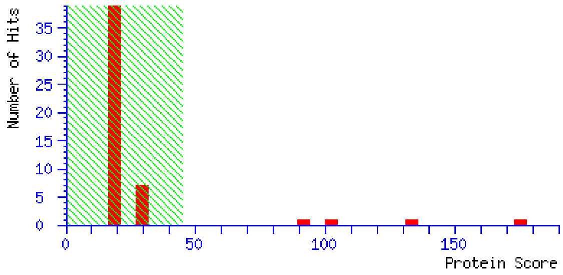

Matched peptide sequences: shown in Bold Red:

```
  1 MASYEGILLG MGNPLLDISA VVDEDFLNKY EIKLNNAILA EDKHLPMYEE
 51 MANNSNVEYI AGGATQNSIK VAQWMLQIPG ATSYMGCIGK DKFGEEMTKN
101 SKKAGVNVHY YEDETAPTGT CGVCVVGGER SLVANLSAAN CYKSEHLKRP
151 ENWALVEKAK YFYIAGFFLT VSPESIQLVA EHAAANNKIF TMNLSAPFIC
201 EFFKDAQEKV LPYMDYVFGN ETEARTFAKV HGWETDNVEE IAKKISQWPK
251 ASGTHKRITV ITQGADPVVV AEDGKVKLFP VILLPKEKLV DTNGAGDAFV
301 GGFLSQLVQE KPIEECVRAG CYAANVIIQR SGCTYPEKPD FS
```

Spot No.: **P50**

NCBI accession No.: **gi|4588474** Species: *Brassica napus*

PFF score: [96]

Protein name: phosphoinositide-specific phospholipase C

Matched peptides No.: [2] Sequence coverage %: [3]

Matched sequences: R.VIELDIWPNSNKNDIDVLHGR.T;

K.NDIDVLHGR.T

Calculated Mr: **66632** Calculated *p*I: **5.79**

Probability Based Mowse Score:

Ions score is -10*Log(P), where P is the probability that the observed match is a random event.
Individual ions scores > 45 indicate identity or extensive homology (p<0.05).
Protein scores are derived from ions scores as a non-probabilistic basis for ranking protein hits.

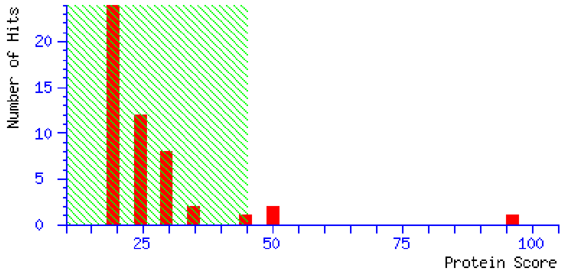

Matched peptide sequences: shown in **Bold Red:**

```
  1 MSKQTYKVCF CFNRRFRYTA SEAPRDVKTL FDKYSENGVM TVDHLQRFLI
 51 DVQKQDKATK EDAQSIINAA SSLLHSNGLH LDAFFKYLFG DSNPPLALHE
101 VHQDMDAPIS HYFIFTGHNS YLTGNQLSSD CSEVPIIDAL KKGVRVIELD
151 IWPNSNKNDI DVLHGRTLTS PVELIKCLRA IKTHAFEVSD YPVVVTLEDH
201 LTPELQSKVA EMVTEIFGEI LFTPPVGESL KEFPSPNSLK RRIIISTKPP
251 KEYKEGKDED VVQKGKALGD EEVWGREVPS FIERNKSGDK DDLDDEEDND
301 EDDDVEKFKK NAPPQYKHLI AIHAGKPKGS ITACLKVDPD KVRRLSLSEE
351 QLEKAAEKYA KQIVRFTQQN LLRIYPKGTR VTSSNYNPLV GWSHGAQMVA
401 FNMQGYGRSL WLMQGMFRAN GGCGYIKKPD ILLKGGSDSD IFDPKTTLPV
451 KTTLRVTIYM GEGWYFDFRH THFDQYSPPD FYTRVGIAGV PADTVMKKTK
501 TLEDNWVPSW DEVFEFPLTV PELALLRLEV HEYDMSEKDD FGGQTCLPVW
551 ELQEGIRSFP LHNRKEEKYK SVKLLVKVEF V
```

(Bold red residues: VIELD at 146–150; IWPNSNKNDI DVLHGR at 151–166.)

Spot No.: **P51**

NCBI accession No.: **gi|255578278** Species: *Ricinus communis*

PFF score: [95]

Protein name: hypothetical protein RCOM_0537780

Matched peptides No.: [2] Sequence coverage %: [6]

Matched sequences: K.QESEYGSGGYGR.R;

R.KPSYGEEGGYGER.T

Calculated Mr: **41389** Calculated *p*I: **4.65**

Probability Based Mowse Score:

Ions score is -10*Log(P), where P is the probability that the observed match is a random event.
Individual ions scores > 45 indicate identity or extensive homology (p<0.05).
Protein scores are derived from ions scores as a non-probabilistic basis for ranking protein hits.

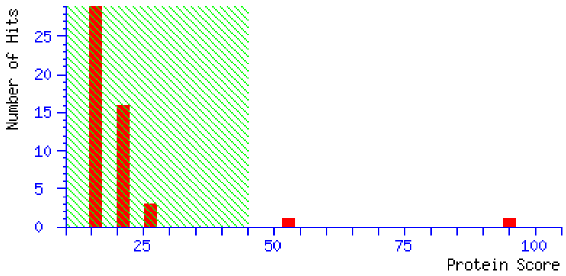

Matched peptide sequences: shown in Bold Red:

1 MPYYTRSEDD VNDFDEFDPT PYGGGYDLAL TYGRPLPPSD ETCYQNSAIA
51 DDDVDYDRPN FTSYAEPSAY NDDILQEEYN SYSRPKPRPG FIPGGGIGGD
101 PYSRPHAAPG FQPGSGGYGG VSEYEKPSGY GRRPDSEYGS GGYGGGTEYE
151 RPSGEDYGSG HGRKQDSEYG SGGYGRRPES EYGSGGGYER PSSEEYGSGY
201 GRK**QESEYGS** **GGYGR**RPEAE YGSGYGGRPE TEYGGGGGSE YGGGYGR**KPS**
251 **YGEEGGYGER** TEYERPSYGD DPPRRPGYGR QDDEYERPSY ERKSDDDNDG
301 SRRKYGGDDD EKGYQKKYGG YGDDDDEEGS RRKYGYGGEE ESYGRKKYGD
351 DGSDDDEKKH HRHKHHQHKH SDDE

Spot No.: **P52**

NCBI accession No.: **gi|255552291** Species: *Ricinus communis*

PFF score: [67]

Protein name: short chain dehydrogenase, putative

Matched peptides No.: [3] Sequence coverage %: [11]

Matched sequences: K.VALVTGGDSGIGR.A;

K.AEGAKDPIAIPTDVGFEENCR.K;

K.DPIAIPTDVGFEENCR.K

Calculated Mr: **32148** Calculated *p*I: **6.50**

Probability Based Mowse Score:

Ions score is -10*Log(P), where P is the probability that the observed match is a random event.
Individual ions scores > 45 indicate identity or extensive homology (p<0.05).
Protein scores are derived from ions scores as a non-probabilistic basis for ranking protein hits.

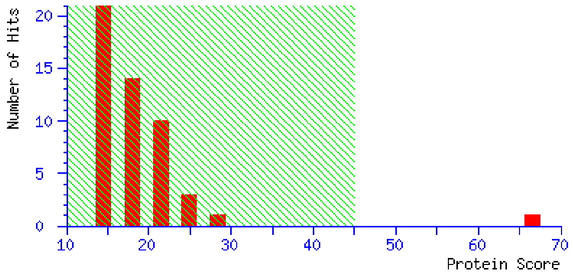

Matched peptide sequences: shown in Bold Red:

```
  1 MATRRGYRFP PQSQDKQPGK EYLMHPLPEF INPHYKPSNK LQDKVALVTG
 51 GDSGIGRAVS YYFTLEGATV AFTYVKGRED KDKDHILKIL HEVKAEGAKD
101 PIAIPTDVGF EENCRKVIDQ IMSEYGKIDI LVNNAGEAHY STTIEDITDP
151 MLERVFRTNI FGHFFMSRHA LKHMKEGGCI INTASVAAYA GFSFMVDYSS
201 TKGAVVAFTR SLALQLIDRG IRVNAVAPGS VWTPFQPSTL SAEQVAQVGS
251 GVPMDRAAQP YEIAPSFVFL ASNDCSSYFT GQVLHPNGMF
```

Spot No.: **P53**

NCBI accession No.: **gi|224057068** Species: *Populus trichocarpa*

PFF score: [279]

Protein name: predicted protein

Matched peptides No.: [7] Sequence coverage %: [35]

Matched sequences: K.CPDTNYLFMGDYVDR.G + Oxidation (M);
R.QITQVYGFYDECLR.K;
R.VQEVPHEGPMCDLLWSDPDDR.C + Oxidation (M);
R.GAGYTFGQDISEQFNHTNNLK.L;
R.AHQLVMEGYNWGHEQK.V + Oxidation (M);
K.VVTIFSAPNYCYR.C;
K.GHTFIQFEPAPR.R

Calculated Mr: **36330** Calculated *p*I: **5.08**

Probability Based Mowse Score:

Ions score is -10*Log(P), where P is the probability that the observed match is a random event.
Individual ions scores > 45 indicate identity or extensive homology (p<0.05).
Protein scores are derived from ions scores as a non-probabilistic basis for ranking protein hits.

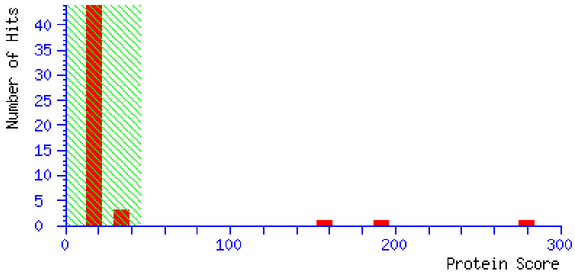

Matched peptide sequences: shown in Bold Red:

1 MSLDSVATNA HGNLDEEISQ LMQCKPLSEQ EVRALCEKAK EILMDESNVQ
51 PVKSPVTICG DIHGQFHDLA ELFRIGGKCP DTNYLFMGDY VDRGYYSVET
101 VTLLVALKVR YPQRITILRG NHESRQITQV YGFYDECLRK YGNANVWKIF
151 TDLFDYFPLT ALVESEIFCL HGGLSPSIET LDNIRNFDRV QEVPHEGPMC
201 DLLWSDPDDR CGWGISPRGA GYTFGQDISE QFNHTNNLKL IARAHQLVME
251 GYNWGHEQKV VTIFSAPNYC YRCGNMASIL EVDDCKGHTF IQFEPAPRRG
301 EPDVTRRTPD YFL

Spot No.: **P54**

NCBI accession No.: **gi|2832430**   Species: *Hevea brasiliensis*

PFF score: [181]

Protein name: prohevein

Matched peptides No.: [3]   Sequence coverage %: [26]

Matched sequences: K.YGWTAFCGPVGAHGQPSCGK.C;

R.IVDQCSNGGLDLDVNVFR.Q;

R.QLDTDGKGYER.G

Calculated Mr: **20852**   Calculated *p*I: **5.64**

Probability Based Mowse Score:

Ions score is -10*Log(P), where P is the probability that the observed match is a random event.
Individual ions scores > 44 indicate identity or extensive homology (p<0.05).
Protein scores are derived from ions scores as a non-probabilistic basis for ranking protein hits.

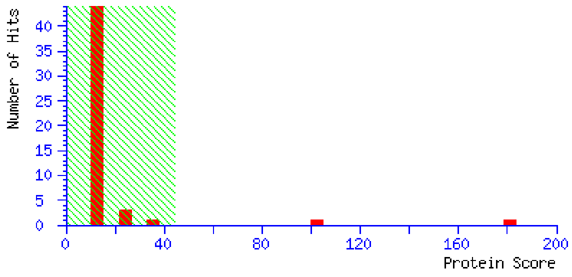

Matched peptide sequences: shown in **Bold Red:**

```
  1 EQCGRQAGGK LCPNNLCCSQ WGWCGSTDEY CSPDHNCQSN CKDSGEGVGG
 51 GSASNVLATY HLYNSQDHGW DLNAASAYCS TWDANKPYSW RSKYGWTAFC
101 GPVGAHGQPS CGKCLSVTNT GTGAKATVRI VDQCSNGGLD LDVNVFRQLD
151 TDGKGYERGH LTVNYQFVDC GDSFNPLFSV MKSSVIN
```

Spot No.: **P55**

NCBI accession No.: **gi|224071449** Species: *Populus trichocarpa*

PFF score: [55]

Protein name: predicted protein

Matched peptides No.: [2] Sequence coverage %: [2]

Matched sequences: K.NKIDVIPHYPGR.L;

K.IDVIPHYPGR.L

Calculated Mr: **47121** Calculated *p*I: **5.28**

Probability Based Mowse Score:

Ions score is -10*Log(P), where P is the probability that the observed match is a random event.
Individual ions scores > 45 indicate identity or extensive homology (p<0.05).
Protein scores are derived from ions scores as a non-probabilistic basis for ranking protein hits.

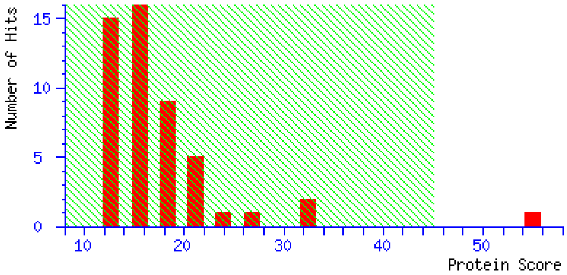

Matched peptide sequences: shown in Bold Red:

```
  1 MDGTSEATWP EILGSRNWDN LLDPLDLSLR KLILRCGDFC QATYDAFNND
 51 QNSRYCGTSR YGKRNFFHKV MLDNPENYQV SSFLYATARV SLPEAFLLHS
101 LSRDSWDRET NWIGYIAVTS DEQTKTLGRR EIYIAFRGTT RNYEWVDILG
151 AKLKSAKPLL RGATSTTHDQ ESSSSSDDDD DKVPKVMLGW LTMYISDDPN
201 SPFTKLSARA QLLAHIKELR ERYKDDDLSI IFTGHSLGAS LSILSAFDLV
251 ENGITDIPVS AFVFGSPQVG NKEFNERFNK YPNLKVLHIK NKIDVIPHYP
301 GRLMGYVYTG IEFEIDTRKS PSLKDSKNPS DWHNLQAMLH IVAGWNGEEQ
351 EFELKVKRSL ALVNKSSEFL KDECLVPGIW WVEKNKGMVR NEDGEWVLAP
401 PDEEDLPVPE C
```

Spot No.: **P56**

NCBI accession No.: **gi|218157** Species: *Oryza sativa Japonica Group*

PFF score: [58]

Protein name: cytoplasmic aldolase

Matched peptides No.: [2] Sequence coverage %: [3]

Matched sequences: K.KVAPEVIAEYTVR.T;

K.VAPEVIAEYTVR.T

Calculated Mr: **39151** Calculated *p*I: **6.56**

Probability Based Mowse Score:

Ions score is -10*Log(P), where P is the probability that the observed match is a random event.
Individual ions scores > 46 indicate identity or extensive homology (p<0.05).
Protein scores are derived from ions scores as a non-probabilistic basis for ranking protein hits.

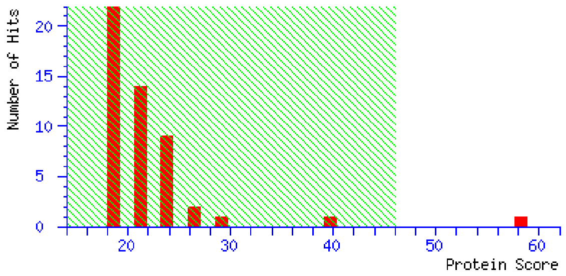

Matched peptide sequences: shown in Bold Red:

```
  1 MSAYCGKYKD ELIKNAAYIG TPGKGILAAD ESTGTIGKRF ASINVENVED
 51 NRRAFRELLF CTPGALQYIS GVILFDETLY QKTKDGKPFV DILKEAGALP
101 GIKVDKGTIE VAGTDKETTT QGHDDLGKQC AKYYEAGARF AKWRAVLKIG
151 PNQPSQLAID LNAQGLACYA IICQENGLVP IVEPEILVDG PHDIDRCAYV
201 SEVVLAACYK ALNEHHVLLE GTLLKPNMVT PGSDAKKVAP EVIAEYTVRT
251 LQRTVPPAVP AIVFLSGGQS EEEATLNLNA MNKLSAKKPW SLSFSFGRAL
301 QQSTLKAWAG KTENVEKARA AFLVRCKANS EATLGTYKGD AVLGEGAAES
351 LHVKDYKY
```

Spot No.: **P57**

NCBI accession No.: **gi|15219412**   Species: *Arabidopsis thaliana*

PFF score: [120]

Protein name: phosphoglycerate kinase

Matched peptides No.: [3]   Sequence coverage %: [6]

Matched sequences: K.YSLKPLVPR.L;

K.KLAALADVYVNDAFGTAHR.A;

K.LAALADVYVNDAFGTAHR.A

Calculated Mr: **42162**   Calculated *p*I: **5.49**

Probability Based Mowse Score:

Ions score is -10*Log(P), where P is the probability that the observed match is a random event.
Individual ions scores > 45 indicate identity or extensive homology (p<0.05).
Protein scores are derived from ions scores as a non-probabilistic basis for ranking protein hits.

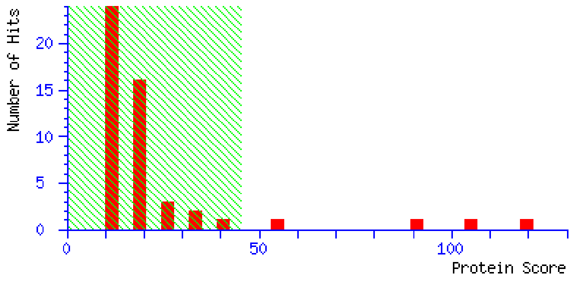

Matched peptide sequences: shown in Bold Red:

```
  1 MATKRSVGTL KEADLKGKSV FVRVDLNVPL DDNSNITDDT RIRAAVPTIK
 51 YLMGNGSRVV LCSHLGRPKG VTPKYSLKPL VPRLSELLGV EVVMANDSIG
101 EEVQKLVAGL PEGGVLLLEN VRFYAEEEKN DPEFAKKLAA LADVYVNDAF
151 GTAHRAHAST EGVAKFLKPS VAGFLMQKEL DYLVGAVANP KKPFAAIVGG
201 SKVSTKIGVI ESLLNTVDIL LLGGGMIFTF YKAQGLSVGS SLVEEDKLDL
251 AKSLMEKAKA KGVSLLLPTD VVIADKFAPD ANSKIVPATA IPDGWMGLDI
301 GPDSIKTFSE ALDTTKTIIW NGPMGVFEFD KFAAGTEAVA KQLAELSGKG
351 VTTIIGGGDS VAAVEKVGLA DKMSHISTGG GASLELLEGK PLPGVLALDE
401 A
```

Spot No.: **P58**

NCBI accession No.: **gi|356513072** Species: *Glycine max*

PFF score: [121]

Protein name: phosphoglucomutase,cytoplasmic-like

Matched peptides No.: [4] Sequence coverage %: [6]

Matched sequences: R.VETTPFDGQKPGTSGLR.K;
K.SIFDFESIR.K;
K.SIFDFESIRK.L;
K.LVTVEDIVR.Q

Calculated Mr: **63702** Calculated *p*I: **5.33**

Probability Based Mowse Score:

Ions score is -10*Log(P), where P is the probability that the observed match is a random event.
Individual ions scores > 45 indicate identity or extensive homology ($p<0.05$).
Protein scores are derived from ions scores as a non-probabilistic basis for ranking protein hits.

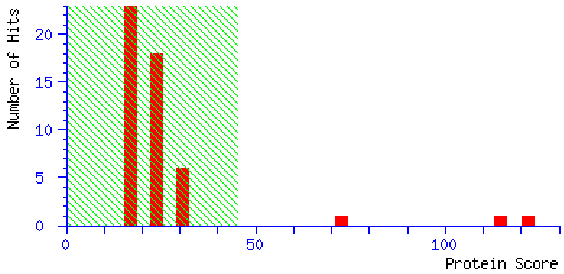

Matched peptide sequences: shown in Bold Red:

1 MVLFNVSR**VE** **TTPFDGQKPG** **TSGLR**KKVKV FVQPHYLHNF VQSTFNALTV
51 EKVRGATLVV SGDGRYFSKE AIQIITKMSA ANGVRRVWIG QNGLLSTPAV
101 SAVIRERVGA DGSRATGAFI LTASHNPGGP HEDFGIKYNM ENGGPAPEGI
151 TDKIYENTTT INEYLIASDL PDVDITTTGV TSFTGPEGPF DVEVFDSASD
201 YIKLMK**SIFD** **FESIRK**LLSS PKFTFCYDAL HGVGGAYAKS IFVDELGAQE
251 SSLLNCTPKE DFGGGHPDPN LTYAKELVAR MGLGKSEPQD DPPEFGAASD
301 GDADRNMILG KRFFVTPSDS VAIIAANAVE AIPYFSAGLK GVARSMPTSA
351 ALDVVAKHLN LKFFEVPTGW KFFGNLMDAG LCSVCGEESF GTGSDHIREK
401 DGIWAVLAWL SILAYKNKDK LEDK**LVTVED** **IVR**QHWATYG RHYYTRYDYE
451 NVDAGAAKEL MAYLVKLQSS LSEVNQIIKG IRSDVSNVVH GDEFEYNDPV
501 DGSISSHQGI RYLFEDGSRL IFRLSGTGSE GATIRLYIEQ YEKDPSKIGR
551 LSNEALAPLV EVALKLSKME EFTGRSAPTV IT

Spot No.: **P59**

NCBI accession No.: **gi|255568794** Species: *Ricinus communis*

PFF score: [95]

Protein name: nucleoredoxin, putative

Matched peptides No.: [3] Sequence coverage %: [5]

Matched sequences: R.VEYFRQEEENAK.K;

K.LIEAYHEIK.A;

K.KCDFDLHPK.C

Calculated Mr: **63728** Calculated *p*I: **5.46**

Probability Based Mowse Score:

Ions score is -10*Log(P), where P is the probability that the observed match is a random event.
Individual ions scores > 45 indicate identity or extensive homology (p<0.05).
Protein scores are derived from ions scores as a non-probabilistic basis for ranking protein hits.

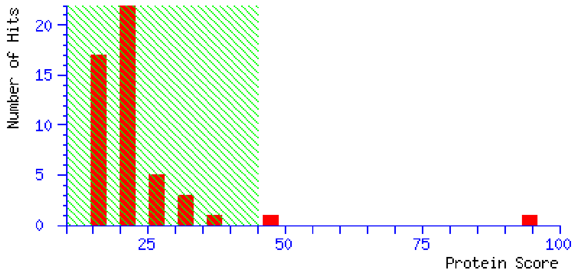

Matched peptide sequences: shown in Bold Red:

```
  1 MADDPVIDVS HDLLSLLSSD DRDFLIRSNG DQVKISNLVG KIVGLYFSGS
 51 WCGPCRHFTP NLVQVYEELS LKGDFEVVFI SSDRDAESFD AYFSKMPWLA
101 IPFSDQETCK HLKDLFKVRG IPNLVFLDAD GKVSCDQGVR FIREYGAEGY
151 PFTPERVEYF RQEEENAKKN QTLSSILVSS SRDFLISKDG TKIPVSELEG
201 KMVGLYFSVH SHRLCLDFTP RLEEVYKKLK EKGEKFEVVL ISMDYDENNF
251 KQGLETMPWL ALPFEDKSRE RLARYFELSA LPTLVIIGED GKTLNKNVAE
301 LIEGHGIQAY PFTPEKLVEL AEIEKARLEA QTLESVLVHG DKDFVIEESG
351 SKVPVSELVG KNILLYFSAK WCPPCRAFLP KLIEAYHEIK AKDNAFEIIF
401 ISSDRDQSSF DEFYTEMPWL ALPFGDDRKT ILQRKFKIKG IPAAIAISPT
451 GKTLTKEARE HITAYGADAY PFNEDHLKQL NDKQEEIAKG WPEKVRHELH
501 PEHELVRMKR NGYGCDGCKE AGSGWSFYCK KCDFDLHPKC ALKKEENGEK
551 VKK
```

Determination of the phosphorylated amino sites of small rubber particle protein (SRPP) in protein spot P6.

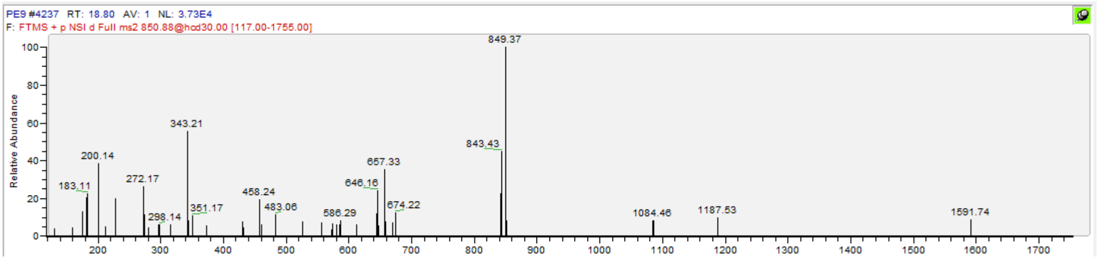

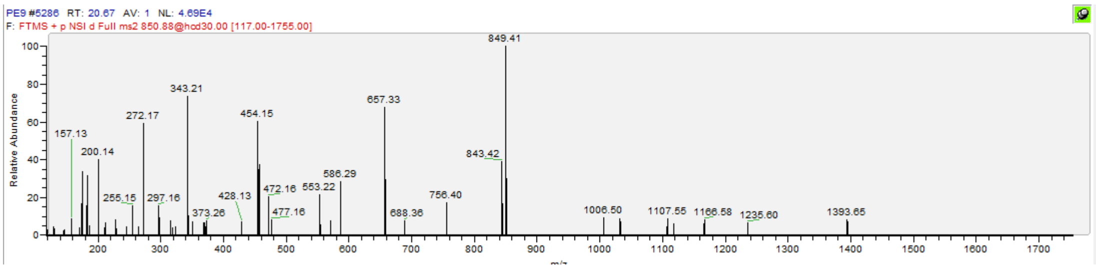

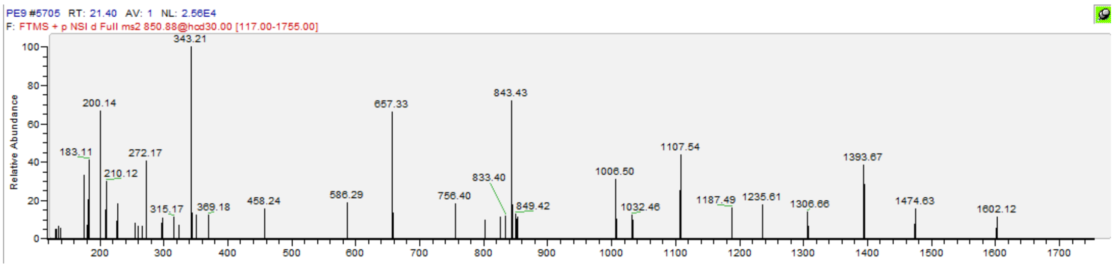

MS/MS Fragmentation of **QVSpAQTYpSVAQDAPR**

#	b	b++	b*	b*++	b0	b0++	Seq.	y	y++	y*	y*++	y0	y0++	#
1	129.0659	65.0366	112.0393	56.5233			Q							15
2	228.1343	114.5708	211.1077	106.0575			V	1572.7054	786.8563	1555.6788	778.3431	1554.6948	777.8510	14
3	315.1663	158.0868	298.1397	149.5735	297.1557	149.0815	S	1473.6370	737.3221	1456.6104	728.8088	1455.6264	728.3168	13
4	386.2034	193.6053	369.1769	185.0921	368.1928	184.6001	A	1386.6049	693.8061	1369.5784	685.2928	1368.5944	684.8008	12
5	514.2620	257.6346	497.2354	249.1214	496.2514	248.6293	Q	1315.5678	658.2876	1298.5413	649.7743	1297.5573	649.2823	11
6	615.3097	308.1585	598.2831	299.6452	597.2991	299.1532	T	*1187.5092*	594.2583	1170.4827	585.7450	1169.4987	585.2530	10
7	858.3393	429.6733	841.3128	421.1600	840.3288	420.6680	Y	1086.4616	543.7344	1069.4350	535.2211	1068.4510	534.7291	9
8	945.3714	473.1893	928.3448	464.6760	927.3608	464.1840	S	*843.4319*	422.2196	826.4054	413.7063	825.4213	413.2143	8
9	1044.4398	522.7235	1027.4132	514.2102	1026.4292	513.7182	V	756.3999	378.7036	739.3733	370.1903	738.3893	369.6983	7
10	1115.4769	558.2421	1098.4503	549.7288	1097.4663	549.2368	A	*657.3315*	329.1694	640.3049	320.6561	639.3209	320.1641	6
11	1243.5355	622.2714	1226.5089	613.7581	1225.5249	613.2661	Q	*586.2944*	293.6508	569.2678	285.1375	568.2838	284.6455	5
12	1358.5624	679.7848	1341.5359	671.2716	1340.5518	670.7796	D	*458.2358*	229.6215	441.2092	221.1083	440.2252	220.6162	4
13	1429.5995	715.3034	1412.5730	706.7901	1411.5890	706.2981	A	*343.2088*	172.1081	326.1823	163.5948			3
14	1526.6523	763.8298	1509.6257	755.3165	1508.6417	754.8245	P	*272.1717*	136.5895	255.1452	128.0762			2
15							R	175.1190	88.0631	158.0924	79.5498			1

#	b	b++	b*	b*++	b0	b0++	Seq.	y	y++	y*	y*++	y0	y0++	#
1	129.0659	65.0366	112.0393	56.5233			Q							15
2	228.1343	114.5708	211.1077	106.0575			V	1474.7285	737.8679	1457.7019	729.3546	1456.7179	728.8626	14
3	297.1557	149.0815	280.1292	140.5682	279.1452	140.0762	S	1375.6601	688.3337	1358.6335	679.8204	1357.6495	679.3284	13
4	368.1928	184.6001	351.1663	176.0868	350.1823	175.5948	A	1306.6386	653.8229	1289.6121	645.3097	1288.6280	644.8177	12
5	496.2514	248.6293	479.2249	240.1161	478.2409	239.6241	Q	1235.6015	618.3044	1218.5749	609.7911	1217.5909	609.2991	11
6	597.2991	299.1532	580.2726	290.6399	579.2885	290.1479	T	1107.5429	554.2751	1090.5164	545.7618	1089.5324	545.2698	10
7	760.3624	380.6849	743.3359	372.1716	742.3519	371.6796	Y	1006.4952	503.7513	989.4687	495.2380	988.4847	494.7460	9
8	847.3945	424.2009	830.3679	415.6876	829.3839	415.1956	S	843.4319	422.2196	826.4054	413.7063	825.4213	413.2143	8
9	946.4629	473.7351	929.4363	465.2218	928.4523	464.7298	V	756.3999	378.7036	739.3733	370.1903	738.3893	369.6983	7
10	1017.5000	509.2536	1000.4734	500.7404	999.4894	500.2483	A	657.3315	329.1694	640.3049	320.6561	639.3209	320.1641	6
11	1145.5586	573.2829	1128.5320	564.7696	1127.5480	564.2776	Q	586.2944	293.6508	569.2678	285.1375	568.2838	284.6455	5
12	1260.5855	630.7964	1243.5590	622.2831	1242.5749	621.7911	D	458.2358	229.6215	441.2092	221.1083	440.2252	220.6162	4
13	1331.6226	666.3149	1314.5961	657.8017	1313.6121	657.3097	A	343.2088	172.1081	326.1823	163.5948			3
14	1428.6754	714.8413	1411.6488	706.3281	1410.6648	705.8360	P	272.1717	136.5895	255.1452	128.0762			2
15							R	175.1190	88.0631	158.0924	79.5498			1

#	b	b++	b*	b*++	b0	b0++	Seq.	y	y++	y*	y*++	y0	y0++	#
1	129.0659	65.0366	112.0393	56.5233			Q							15
2	228.1343	114.5708	211.1077	106.0575			V	1572.7054	786.8563	1555.6788	778.3431	1554.6948	777.8510	14
3	395.1326	198.0700	378.1061	189.5567	377.1221	189.0647	S	1473.6370	737.3221	1456.6104	728.8088	1455.6264	728.3168	13
4	466.1697	233.5885	449.1432	225.0752	448.1592	224.5832	A	1306.6386	653.8229	1289.6121	645.3097	1288.6280	644.8177	12
5	594.2283	297.6178	577.2018	289.1045	576.2178	288.6125	Q	1235.6015	618.3044	1218.5749	609.7911	1217.5909	609.2991	11
6	695.2760	348.1416	678.2494	339.6284	677.2654	339.1364	T	1107.5429	554.2751	1090.5164	545.7618	1089.5324	545.2698	10
7	858.3393	429.6733	841.3128	421.1600	840.3288	420.6680	Y	1006.4952	503.7513	989.4687	495.2380	988.4847	494.7460	9
8	945.3714	473.1893	928.3448	464.6760	927.3608	464.1840	S	843.4319	422.2196	826.4054	413.7063	825.4213	413.2143	8
9	1044.4398	522.7235	1027.4132	514.2102	1026.4292	513.7182	V	756.3999	378.7036	739.3733	370.1903	738.3893	369.6983	7
10	1115.4769	558.2421	1098.4503	549.7288	1097.4663	549.2368	A	657.3315	329.1694	640.3049	320.6561	639.3209	320.1641	6
11	1243.5355	622.2714	1226.5089	613.7581	1225.5249	613.2661	Q	586.2944	293.6508	569.2678	285.1375	568.2838	284.6455	5
12	1358.5624	679.7848	1341.5359	671.2716	1340.5518	670.7796	D	458.2358	229.6215	441.2092	221.1083	440.2252	220.6162	4
13	1429.5995	715.3034	1412.5730	706.7901	1411.5890	706.2981	A	343.2088	172.1081	326.1823	163.5948			3
14	1526.6523	763.8298	1509.6257	755.3165	1508.6417	754.8245	P	272.1717	136.5895	255.1452	128.0762			2
15							R	175.1190	88.0631	158.0924	79.5498			1

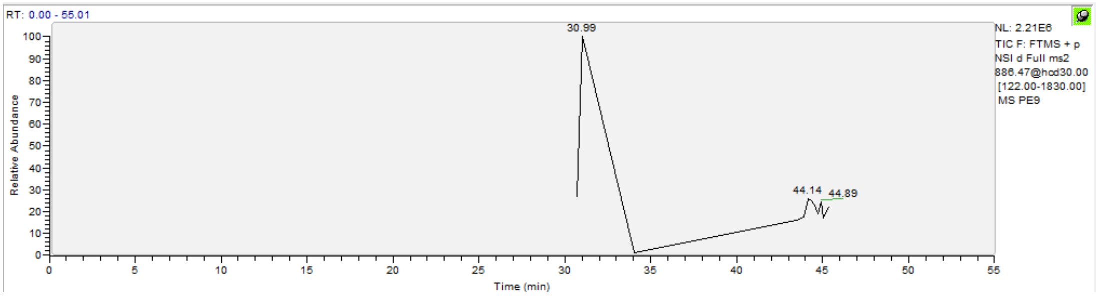

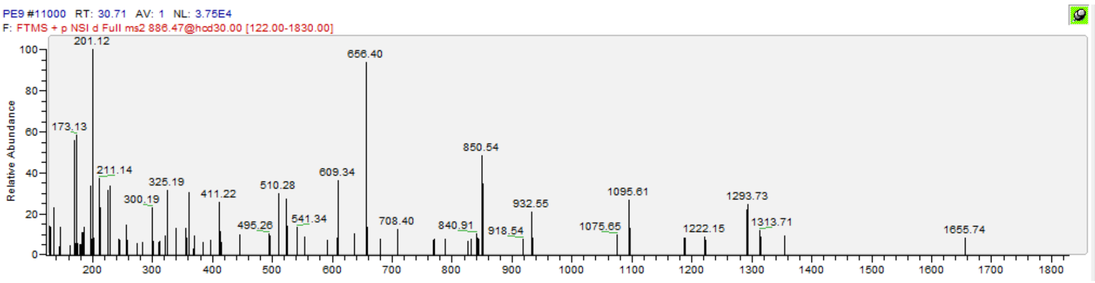

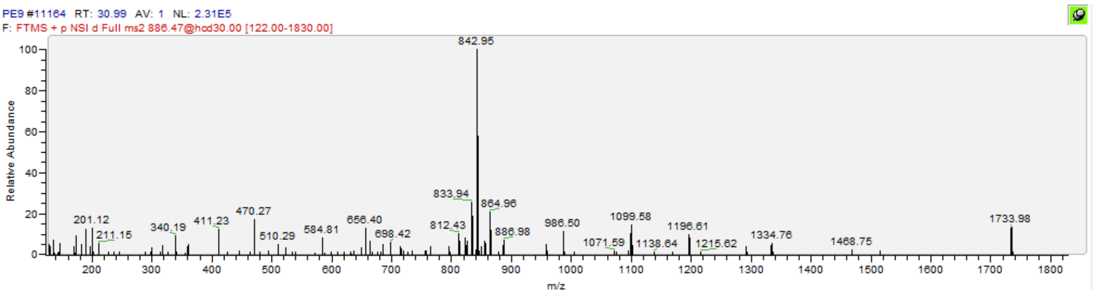

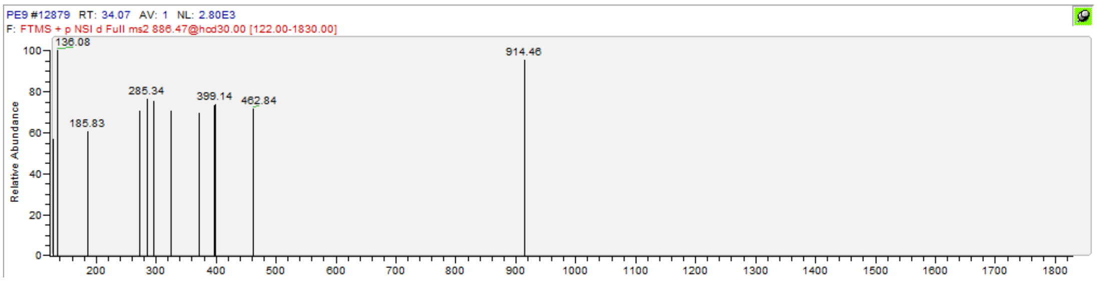

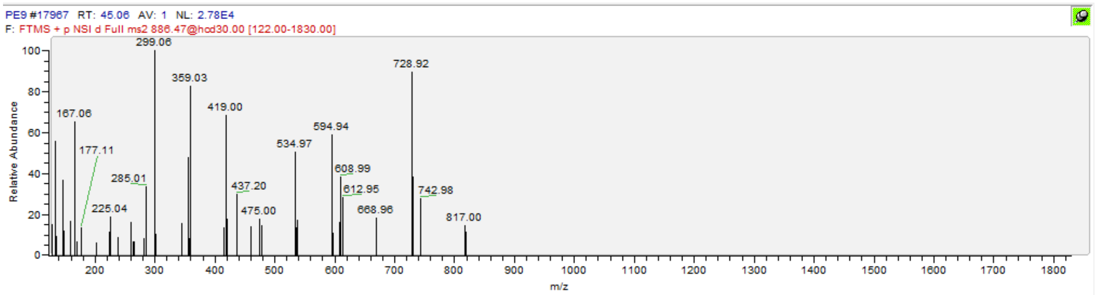

MS/MS Fragmentation of TVVTpPVYYIPLEAVK

#	b	b++	b0	b0++	Seq.	y	y++	y*	y*++	y0	y0++	#
1	102.0550	51.5311	84.0444	42.5258	T							15
2	201.1234	101.0653	183.1128	92.0600	V	1670.8805	835.9439	1653.8539	827.4306	1652.8699	826.9386	14
3	300.1918	150.5995	282.1812	141.5942	V	1571.8121	786.4097	1554.7855	777.8964	1553.8015	777.4044	13
4	481.2058	241.1065	463.1952	232.1013	T	1472.7437	736.8755	1455.7171	728.3622	1454.7331	727.8702	12
5	578.2586	289.6329	560.2480	280.6276	P	1291.7297	646.3685	1274.7031	637.8552	1273.7191	637.3632	11
6	677.3270	339.1671	659.3164	330.1618	V	1194.6769	597.8421	1177.6503	589.3288	1176.6663	588.8368	10
7	840.3903	420.6988	822.3797	411.6935	Y	1095.6085	548.3079	1078.5819	539.7946	1077.5979	539.3026	9
8	1003.4536	502.2305	985.4431	493.2252	Y	932.5451	466.7762	915.5186	458.2629	914.5346	457.7709	8
9	1116.5377	558.7725	1098.5271	549.7672	I	769.4818	385.2445	752.4553	376.7313	751.4713	376.2393	7
10	1213.5905	607.2989	1195.5799	598.2936	P	656.3978	328.7025	639.3712	320.1892	638.3872	319.6972	6
11	1326.6745	663.8409	1308.6640	654.8356	L	559.3450	280.1761	542.3184	271.6629	541.3344	271.1708	5
12	1455.7171	728.3622	1437.7065	719.3569	E	446.2609	223.6341	429.2344	215.1208	428.2504	214.6288	4
13	1526.7542	763.8808	1508.7437	754.8755	A	317.2183	159.1128	300.1918	150.5995			3
14	1625.8226	813.4150	1607.8121	804.4097	V	246.1812	123.5942	229.1547	115.0810			2
15					K	147.1128	74.0600	130.0863	65.5468			1

Mascot Search Results

Peptide View

MS/MS Fragmentation of **QVSAQTYSVAQDAPR**
Found in **SRPP_HEVBR** in **SwissProt**, Small rubber particle protein OS=Hevea brasiliensis GN=SRPP PE=1 SV=1
Match to Query 9859: 1699.749968 from(850.882260,2+) intensity(277497.1200) scans(5705) rawscans(sn5705) rtinseconds(1283.9872) index(3303)
Title: 3304: Scan 5705 (rt=1283.99)
Data file tempfile

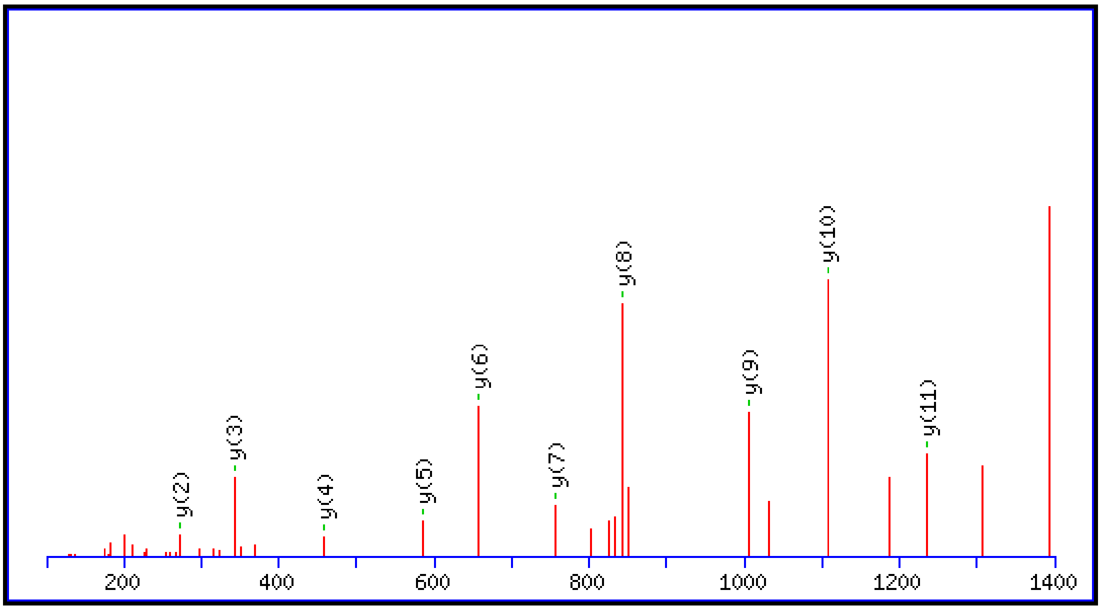

Monoisotopic mass of neutral peptide Mr(calc): 1699.7567
Fixed modifications: Carbamidomethyl (C) (apply to specified residues or termini only)
Variable modifications:
S3 : Phospho (ST), with neutral losses 0.0000(shown in table), 97.9769
Ions Score: 91 Expect: 2.8e-009
Matches : 10/242 fragment ions using 13 most intense peaks

#	b	b^{++}	b*	b^{*++}	b^0	b^{0++}	Seq.	y	y^{++}	y*	y^{*++}	y^0	y^{0++}	#
1	129.0659	65.0366	112.0393	56.5233			Q							15
2	228.1343	114.5708	211.1077	106.0575			V	1572.7054	786.8563	1555.6788	778.3431	1554.6948	777.8510	14
3	395.1326	198.0700	378.1061	189.5567	377.1221	189.0647	S	1473.6370	737.3221	1456.6104	728.8088	1455.6264	728.3168	13
4	466.1697	233.5885	449.1432	225.0752	448.1592	224.5832	A	1306.6386	653.8229	1289.6121	645.3097	1288.6280	644.8177	12
5	594.2283	297.6178	577.2018	289.1045	576.2178	288.6125	Q	***1235.6015***	618.3044	1218.5749	609.7911	1217.5909	609.2991	11
6	695.2760	348.1416	678.2494	339.6284	677.2654	339.1364	T	***1107.5429***	554.2751	1090.5164	545.7618	1089.5324	545.2698	10
7	858.3393	429.6733	841.3128	421.1600	840.3288	420.6680	Y	***1006.4952***	503.7513	989.4687	495.2380	988.4847	494.7460	9
8	945.3714	473.1893	928.3448	464.6760	927.3608	464.1840	S	***843.4319***	422.2196	826.4054	413.7063	825.4213	413.2143	8
9	1044.4398	522.7235	1027.4132	514.2102	1026.4292	513.7182	V	***756.3999***	378.7036	739.3733	370.1903	738.3893	369.6983	7
10	1115.4769	558.2421	1098.4503	549.7288	1097.4663	549.2368	A	***657.3315***	329.1694	640.3049	320.6561	639.3209	320.1641	6
11	1243.5355	622.2714	1226.5089	613.7581	1225.5249	613.2661	Q	***586.2944***	293.6508	569.2678	285.1375	568.2838	284.6455	5
12	1358.5624	679.7848	1341.5359	671.2716	1340.5518	670.7796	D	***458.2358***	229.6215	441.2092	221.1083	440.2252	220.6162	4
13	1429.5995	715.3034	1412.5730	706.7901	1411.5890	706.2981	A	***343.2088***	172.1081	326.1823	163.5948			3
14	1526.6523	763.8298	1509.6257	755.3165	1508.6417	754.8245	P	***272.1717***	136.5895	255.1452	128.0762			2
15							R	175.1190	88.0631	158.0924	79.5498			1

All matches to this query

Score	Mr(calc)	Delta	Sequence	Site Analysis
91.2	1699.7567	-0.0067	QVSAQTYSVAQDAPR	Phospho S3 98.48%
73.0	1699.7567	-0.0067	QVSAQTYSVAQDAPR	Phospho T6 1.48%
56.8	1699.7567	-0.0067	QVSAQTYSVAQDAPR	Phospho Y7 0.04%
41.0	1699.7567	-0.0067	QVSAQTYSVAQDAPR	Phospho S8 0.03%

Supplementary Figure S6: Antibody information

Antibody name: WR-1

Protein name: sucrose transporter 3

Plant species: *Hevea brasiliensis*

NCBI Accession No.: gi |227809927

Antibody tag sequence: ISGMLVQPIVGYHSC

Protein sequence：

1 metdptkesk lpsseplppp pplvppppas sklrkivmva siaagiqfgw alqlslltpy
61 vqllgiphtw aafiwlcgpi sgmlvqpivg yhsdrctsrf grrrpfiagg alavavavfl
121 igyaadlgql sgdpvakspk praiavfvvg fwildvannm lqgpcralla dlsganqkkt
181 rianalysff mavgnvlgfa agsythlyki fpftktkacd vycanlkscf fisivllltl
241 tvlaliyvre kpwspergnt aagdeeeede gasesspmpf fseifaslkn lqkpmwilll
301 vtclnwvawf pfllfdtdwm grevyggdsn gnpvqvrlyd rgvragalgl mlnsvvlgft
361 slgvevlara vggvkrlwgi vnfilsfclf mtilitkmae shrrfatvgg gatvplpppg
421 dvkagalalf avmgvpqait ysipfalasi fcntagagqg lslgvlnlsi vipqmvvsvv
481 sgpwdalfgg gnlpafvvgg vaaaasgifa ftllpspqpd apsaktsrav taafh

Gene sequence：

1 atggagactg atcctacgaa agaatccaaa ctcccctctt ctgaacctct tccacctcct
61 ccgcccctag ttcctccacc acccgcttca agcaagctta gaaagattgt aatggtggcc
121 tccatagctg ctggtatcca gtttggctgg gctttgcagc tctctctttt gacaccttac
181 gttcagcttc ttggtatacc tcacacatgg gctgctttca tctggctatg tggacccatt
241 tcaggaatgc ttgtccagcc aattgtcggc tatcatagtg atcgttgcac ctcccgtttt
301 ggccgtcgcc gcccttttat tgcaggcggt gctttggccg ttgctgtagc tgttttcctt
361 atcggttatg ctgctgacct tggtcaactc tctggtgacc ctgttgccaa gagccccaaa
421 ccacgagcta tagccgtgtt tgtcgttggg ttctggattc ttgacgtcgc taataacatg
481 ctccagggtc cttgccgtgc tctcctcgcc gatctttctg gtgccaatca gaagaagacg
541 cgtatagcta atgcactgta ctccttcttc atggccgtcg ggaacgttct tggctttgcc
601 gctggatcct acactcattt atataaaata tttccattca caaagacgaa agcttgtgac
661 gtttactgtg caaatctgaa atcttgtttc tttatctcca ttgtcttgct tttaactctc
721 actgtattag ctctcattta cgttcgtgaa aagccttggt ccccagagcg aggaaacacc
781 gctgccggtg acgaggagga ggaggacgag ggggcgtcgg agtcttctcc gatgccgttc
841 ttcagtgaga tatttgcatc tttgaagaat ctgcaaaaac ccatgtggat ccttcttctg
901 gtgacgtgcc tgaactgggt tgcatggttt ccattcttgt tgttcgatac tgattggatg
961 ggtagagagg tatacggcgg agattcaaac ggaaatcccg ttcaagtgag gttgtatgat
1021 cgtggggtgc gcgctggtgc actgggtctg atgcttaact cggtggtttt ggggttcacc

1081 tcccttggtg tggaggtgct ggcgcgtgct gtcggaggag tgaaaagatt gtggggtata
1141 gtgaatttca ttctttcatt ttgtttgttc atgacgatct tgatcaccaa aatggctgaa
1201 tcccacagaa ggttcgccac cgtgggaggc ggcgccacgg ttcccttgcc accacctgga
1261 gacgtcaagg ctggtgcatt ggcccttttt gcggtgatgg gtgtacctca agctataact
1321 tacagtattc cttttgcttt ggcatcaata ttttgtaaca ctgctggtgc tggtcaagga
1381 ctttctttgg gagttctgaa tctttcaatt gttataccac agatggtggt gtcagtagta
1441 agcggaccat gggatgcatt atttggaggg ggtaatctac cagcttttgt ggtgggaggg
1501 gttgctgccg cggccagtgg aatatttgca ttcaccttac tcccatctcc gcagcctgat
1561 gccccctccg ccaagacttc aagggctgtc acagctgcct tccattga

Antibody name: WR-3

Protein name: **hydroxymethylglutaryl coenzyme A reductase**

Plant species: *Hevea brasiliensis*

NCBI Accession No.: gi |16417954

Antibody tag sequence: CEDRSPTTPKASDA

Protein sequence:

1 mdttgrlhhr khatpvedrs pttpkasdal plplyltnav fftlffsvay yllhrwrdki
61 rnstplhivt lseivaivsl iasfiyllgf fgidfvqsfi arashdvwdl edtdpnylid
121 edhrlvtcpp anistkttii aaptklptse pliaplvsee demivnsvvd gkipsysles
181 klgdckraaa irrealqrmt rrsleglpve gfdyesilgq ccempvgyvq ipvgiagpll
241 lngreysvpm attegclvas tnrgckaiyl sggatsvllk dgmtrapvvr fasatraael
301 kffledpdnf dtlavvfnks srfarlqgik csiagknlyi rfscstgdam gmnmvskgvq
361 nvleflqsdf sdmdvigisg nfcsdkkpaa vnwiegrgks vvceaiikee vvkkvlktnv
421 aslvelnmlk nlagsavaga lggfnahagn ivsaifiatg qdpaqnvess hcitmmeavn
481 dgkdlhisvt mpsievgtvg ggtqlasqsa clnllgvkga nkespgsnsr llaaivagsv
541 lagelslmsa iaagqlvksh mkynrsskdm skaas

Gene sequence:

1 atggacacca ccggccggct ccaccaccga aagcatgcta cacccgttga ggaccgttct
61 ccgaccactc cgaaagcgtc ggacgcgctt ccgcttcccc tctacctgac caacgcggtt
121 ttcttcacgc tgttcttctc ggtggcgtat tacctccttc accggtggcg cgacaagatc
181 cgcaactcca ctccccttca tatcgttact ctctctgaaa ttgttgctat tgtctccctc
241 attgcctctt tcatttacct cctaggattc ttcggtatcg attttgtgca gtcattcatt
301 gcacgcgcct cccatgacgt gtgggacctc gaagatacgg atcccaacta cctcatcgat
361 gaagatcacc gtctcgttac ttgccctccc gctaatatat ctactaagac taccattatt
421 gccgcaccta ccaaattgcc tacctcggaa cccttaattg cacccttagt ctcggaggaa
481 gacgaaatga tcgtcaactc cgtcgtggat gggaagatac cctcctattc tctggagtcg
541 aagctcgggg actgcaaacg agcggctgcg attcgacgcg aggctttgca gaggatgaca
601 aggaggtcgc tggaaggctt gccagtagaa gggttcgatt acgagtcgat tttaggacaa
661 tgctgtgaaa tgccagtggg atacgtgcag attccggtgg ggattgcggg gccgttgttg
721 ctgaacggcc gggagtactc tgttccaatg gcgaccacgg agggttgttt ggtggcgagc
781 actaatagag ggtgtaaggc catttacttg tcaggtgggg ccaccagcgt tttgttgaag
841 gatggcatga caagagcgcc tgttgttaga ttcgcgtcgg cgactagagc cgcggagttg
901 aagttcttct tggaggatcc tgacaatttt gataccttgg ccgtagtttt taacaagtct
961 agtagatttg cgaggctcca aggcattaaa tgctcaattg ctggtaagaa tctttatata
1021 agattcagct gcagcactgg cgatgcaatg gggatgaaca tggtttctaa aggggttcaa
1081 aacgttcttg aatttcttca aagtgatttt tctgatatgg atgtcattgg catctcagga
1141 aatttttgtt cggataagaa gcctgctgct gtaaattgga ttgaaggacg tggcaaatca

1201 gttgtttgtg aggcaattat caaggaagag gtggtgaaga aggtgttgaa aaccaatgtg
1261 gcctccctag tggagcttaa catgctcaag aatcttgctg gttctgctgt tgctggtgct
1321 ttgggtggat ttaatgccca tgcaggcaac atcgtatctg caatctttat tgccactggc
1381 caggatccag cacagaatgt tgagagttct cattgcatta ccatgatgga agctgtcaat
1441 gatggaaagg atctccatat ctctgtgacc atgccctcca ttgaggtggg tacagtcgga
1501 ggtggaactc aacttgcatc tcagtctgct tgtctcaatt tgcttggggt gaagggtgca
1561 aacaaagagt cgccaggatc aaactcaagg ctccttgctg ccatcgtagc tggttcagtt
1621 ttggctggtg agctctcctt gatgtctgcc attgcagctg ggcagcttgt caagagtcac
1681 atgaagtaca acagatccag caaagatatg tctaaagctg catcttag

Antibody name: WR-4

Protein name: **hydroxymethylglutaryl coenzyme A synthase**

Plant species: *Hevea brasiliensis*

NCBI Accession No.: gi |16417956

Antibody tag sequence: YKHFCAKYEKFEGK

Protein sequence:

1 maknvgilav diyfpptfvq qealeahdga skgkytiglg qdcmafctev edvismslta
61 vtslldkyni dpkqigrlev gsetvidksk siktflmqif ekfgntdieg vdstnacygg
121 taalfncvnw vessswdgry glvvctdsav yaegparptg gaaaiailvg pdapiafesk
181 frgshmshay dfykpnlase ypvvdgklsq tcylmaldsc ykhfcakyek fegkqfsisd
241 aeyfvfhspy nklvqksfar lvfndfvrna ssidetakek lapfsnlsgd esyqnrdlek
301 vsqqvakply dakvkpttli pkqvgnmyta slyaafasll hskhtelagk rvtlfsygsg
361 ltatmfslrl hegqhpfsls niasvmnvag klkarhelpp ekfvdimklm ehrygakdfv
421 tskdcsllas gtyyltevds lyrrfyaqka vgntvengll angh

Gene sequence:

1 atggcaaaga atgtgggaat tctcgctgtg gacatctact ttcctcctac ctttgttcag
61 caggaagcac tggaggctca tgatggtgca agcaaaggga aatacaccat tggacttgga
121 caggattgca tggcattttg tactgaggtg gaagatgtca tctcaatgag tttgactgca
181 gttacttcac tcctcgacaa gtataatatt gatcctaaac aaatcggtcg tctggaagtt
241 ggcagtgaga ctgtgatcga caagagcaaa tctattaaaa ccttcttgat gcaaattttc
301 gagaaattcg gaaacactga cattgaaggc gttgactcaa caaatgcatg ttatgggggg
361 actgcagctt tattcaactg tgtcaattgg gttgagagca gttcatggga tggacgctat
421 ggacttgtag tgtgtactga cagtgcggtc tatgcagagg gtccagcccg accaactgga
481 ggagctgcag ccattgcgat tttagtaggt ccagatgcac ctattgcttt tgaaagcaaa
541 tttaggggga gccatatgtc tcatgcttat gatttttaca agcccaacct ggctagtgaa
601 tatccagttg tggatggcaa gctttcccaa acatgctacc tcatggctct tgattcttgc
661 tacaaacatt tctgtgccaa gtatgagaaa tttgaaggca agcaattctc tatttctgat
721 gctgaatatt ttgtatttca ttctccttac aacaagcttg tacagaaaag ctttgctcgt
781 ttggtgttca atgactttgt gaggaatgcc aggtctattg atgagactgc taaagaaaag
841 ctggcaccgt tttcaaattt atctggtgat gaaagctacc aaaaccggga tcttgaaaag
901 gtatcccaac aagttgccaa gcccctttat gatgcgaaag tgaaaccaac cactttgata
961 ccaaagcaag ttggcaatat gtacactgca tctttgtatg cagcatttgc atccctcctt
1021 cacagtaaac atactgaatt ggcaggcaag cgggtgacac tgttctctta tgggagtggg
1081 ttgacagcca caatgttctc attgcgacta catgaaggcc aacatccctt tagcttgtca
1141 aacattgcat ctgtgatgaa tgttgcagga aagttgaagg caagacatga gcttccccca
1201 gagaagtttg taaacatcat gaagctaatg gagcaccggt acggagctaa agactttgtg
1261 agaagcaagg attgcagcct cttggcttct ggaacatact atctcacaga agttgacagc

1321 ttgtatcgaa gattctatgc ccagaaggct gttggcaaca cagttgagaa tggtttgctg
1381 gctaatggtc attga

Antibody name: WR-5

Protein name: **phosphomevalonate kinase**

Plant species: *Hevea brasiliensis*

NCBI Accession No.: gi |16417948

Antibody tag sequence: DEIKPDSWAWAWTDC

Protein sequence:

1 mavvasapgk vlmtggylil erpnagivls tnarfyaivk piydeikpds wawawtdvkl
61 tspqlaresl yklslknlal qcvsssasrn pfveqavqfa vaaahatldk dkknvlnkll
121 lqglditilg tndfysyrne ieacglpltp eslaalpsfs sitfnveean gqnckpevak
181 tglgssaamt tavvaallhh lglvdlsssc kekkfsdldl vhiiaqtahc iaqgkvgsgf
241 dvssavygsh ryvrfspevl ssaqdagkgi plqevisnil kgkwdhertm fslpplmsll
301 lgepgtggss tpsmvgalkk wqksdtqksq etwrklsean saletqfnil sklaeehwda
361 ykcvidscst knsekwieqa tepsreavvk allgsrnaml qirnymrqmg eaagvpiepe
421 sqtrlldttm nmdgvllagv pgaggfdavf avtlgdsgtn vakawsslnv lallvredpn
481 gvllesgdpr tkeittavfa vhi

Gene sequence:

1 atggctgtag ttgcttctgc tccgggtaag gtgttgatga ctgggggtta cctcatattg
61 gaaagaccca atgcagggat tgtactcagc acaaatgctc gattctatgc cattgtgaag
121 cctatttacg atgaaatcaa acctgatagt tgggcatggg catggactga tgtgaaatta
181 acatctcccc aactagcaag ggaaagcttg tacaaattgt cactgaaaaa tttagctctt
241 cagtgtgtct cttcaagtgc atcaaggaac ccatttgtgg aacaagcagt gcaatttgct
301 gtagcagctg cacatgcaac acttgacaaa gataagaaga atgtcttaaa caagctactc
361 ttgcaaggtc ttgatattac aatattaggt accaatgact tctattcata ccgaaatgag
421 attgaagcat gtggactccc tttgacacca gaatcattgg ctgcacttcc ttctttttcc
481 tcaatcacct tcaatgtaga ggaagcaaat ggacaaaact gcaagcctga ggtagctaaa
541 actggattgg gttcatcagc agcaatgacc actgctgtag ttgctgcttt acttcatcac
601 cttggattgg ttgatctttc atcctcttgt aaagagaaga aattttctga tcttgatttg
661 gtacatataa tagcccaaac tgcccattgt attgcacaag ggaaagtcgg cagtggattt
721 gatgttagtt ctgcagttta tggcagtcat cgatacgtgc gcttctctcc agaagtgctt
781 tcctctgctc aggatgctgg gaaaggaatt ccattacagg aagtcatttc taacatccta
841 aaaggaaaat gggaccatga gaggactatg ttttccttgc caccattgat gagcctgcta
901 ctaggtgagc caggaactgg aggatcttcc acgccatcaa tggtaggtgc tctaaagaaa
961 tggcagaagt ctgatactca gaaatcccaa gaaacatgga gaaagttgtc agaggcaaat
1021 tcagcacttg aaacgcaatt caatatttta agcaagctcg cagaagaaca ttgggacgcg
1081 tataaatgtg tgatagacag ttgcagcaca aaaaactcag agaagtggat tgagcaggca
1141 actgaaccca gccgagaagc agttgttaaa gcattattag gatcaagaaa tgccatgctt
1201 cagatcagaa attacatgcg ccagatgggt gaggctgcag gtgttccgat agagcctgaa

1261 tcacagactc gacttttgga tactactatg aatatggatg gagtcttgtt ggctggagtt
1321 cctggagcag gtgggtttga tgcagtcttc gctgttacct taggggactc tggtaccaat
1381 gtggcaaaag cttggagttc actcaatgtt ctggccctgt tggttagaga agaccctaat
1441 ggtgttttgt tagaaagcgg cgatccaaga accaaggaaa tcacaacagc tgtttttgca
1501 gttcatattt ga

Antibody name: WR-6

Protein name: **mevalonate kinase, MEVK**

Plant species: *Hevea brasiliensis*

NCBI Accession No.: gi |16417946

Antibody tag sequence: DVSITEKEEKLEEC

Protein sequence:

1 mevkarapgk iilsgehavv hgstavaasi nlytyvtlsf ataenddslk lqlkdlalef
61 swpigrirea lsnlgapsss trtscsmesi ktisalveee nipeakialt sgvsaflwly
121 tsiqgfkpat vvvtsdlplg sglgssaafc valsaallaf sdsvnvdtkh lgwsifgesd
181 lellnkwale gekiihgkps gidntvsayg nmikfksgnl triksnmplk mlvtntrvgr
241 ntkalvagvs ertlrhpnam sfvfnavdsi snelaniiqs papddvsite keekleelme
301 mnqgllqcmg vshasietvl rttlkyklas kltgaggggc vltllptlls gtvvdkaiae
361 lescgfqcli agiggngvef cfggss

Gene sequence:

1 atggaagtta aagcaagagc tccagggaaa atcattctct ccggtgaaca cgcagtggtg
61 cacggatcca ctgcagtcgc tgcatccatt aatctctaca cctatgtcac cctttctttt
121 gctactgctg agaatgatga ttcactgaaa cttcagctca aggatctggc actagaattt
181 tcatggccaa ttggtagaat cagagaggca ttatctaact taggtgctcc ttcctcttca
241 acacgcacct cttgctcgat ggaatcaatt aagacaattt cagctttggt tgaagaagaa
301 aatatcccag aggcaaaaat tgcactcact tctggagtgt cagccttttt atggttatat
361 acttctattc aaggatttaa gcctgccacc gtagttgtca cttctgatct tccactgggt
421 tcaggcctag gatcatctgc tgcattttgt gttgccctct cagctgctct gcttgctttc
481 tcagactctg taaatgtgga cacaaagcac ctagggtggt caatatttgg agagtctgac
541 cttgaattat taaacaaatg ggctctcgaa ggtgaaaaga taattcatgg aaagccatct
601 ggaatagaca acactgtcag cgcatatggc aacatgatca agttcaagtc tggtaatctg
661 actcgcatca agtccaacat gccgctcaaa atgctcgtca ctaacacaag agttgggagg
721 aacacaaaag cactggttgc tggtgtttca gagagaacct tacggcaccc taatgccatg
781 agttttgttt ttaatgccgt tgattctatc agtaatgagc tggctaacat catccagtca
841 cctgctccag atgatgtgtc cataactgag aaggaagaga agctagaaga gttaatggaa
901 atgaatcaag gcttgcttca atgcatgggg gtcagccatg cttctataga aactgttctt
961 cggacaactt tgaaatacaa gttagcttcc aagctgactg gagcaggggg tggggggtgc
1021 gtgctgacac tgttaccaac cctgctatca ggaacagttg ttgacaaagc aattgctgaa
1081 ttggagtcat gcggatttca atgtttgatt gctggaatcg gtgggaatgg tgttgagttt
1141 tgctttggtg gttcatcctg a

Antibody name: WR-7

Protein name: **farnesyl diphosphate synthase, FADS**

Plant species: *Hevea brasiliensis*

NCBI Accession No.: gi |22676905

Antibody tag sequence: IPRILKKHFRGKAYC

Protein sequence:

1 madlkstflk vysvlkqell edpafewtpd srqwvermld ynvpggklnr glsvidsykl
61 lkegqeltee eiflasalgw ciewlqayfl vlddimdssh trrgqpcwfr vpkvgliaan
121 dgillrnhip rilkkhfrgk ayyvdlldlf nevefqtasg qmidlittle gekdlskytl
181 slhrrivqyk tayysfylpv acalliagen ldnhivvkdi lvqmgiyfqv qddyldcfgd
241 petigkigtd iedfkcswlv vkalelcnee qkkvlyehyg kadpasvakv kvlynelklq
301 gvfteyenes ykklvtsiea hpskpvqavl ksflakiykr qk

Gene sequence:

1 atggcggatc tgaagtcaac tttcttgaag gtctactctg tcctcaagca ggagctcctt
61 gaggatccgg ctttcgaatg gacaccagat tcccgtcaat gggttgagcg gatgttggac
121 tacaatgtgc ctggagggaa gctgaatagg gggctttctg taattgacag ctacaaattg
181 ttgaaagaag gacaggaatt aacagaagaa gagatctttc ttgcaagtgc tcttggttgg
241 tgtattgaat ggcttcaagc ctattttctt gtacttgatg acattatgga tagctctcat
301 acacgacgtg gtcagccttg ctggtttagg gtgcccaagg ttggtctgat tgcagcaaat
361 gatgggattt tgcttcgcaa tcacattccc aggattctta aaaagcactt ccgagggaag
421 gcatactatg tagatcttct agatttgttt aatgaggtgg agtttcaaac agcctcagga
481 cagatgatag atctaattac aacacttgaa ggagaaaagg atttatccaa atacactttg
541 tcactccacc ggagaattgt tcagtacaaa actgcctact actcatttta ccttcctgtt
601 gcttgtgcat tgctcatagc gggtgagaat ctggacaatc atattgttgt aaaagacatt
661 cttgttcaga tgggaatcta cttccaagta caggatgatt atttggattg ctttggtgat
721 cccgagacaa ttggtaagat aggaacagat atagaagatt ttaagtgttc atggttggtc
781 gtgaaggctt tagaactttg caatgaagaa caaaagaaag tgttatatga gcactatggg
841 aaagctgacc cagccagtgt agcaaaggtg aaggtccttt ataatgagct gaagcttcag
901 ggggtattta cggagtatga gaatgaaagc tataagaaac tagtaacctc tattgaagct
961 catcctagca agccggtgca agcagtgttg aagtcctttt tggccaaaat ttacaagaga
1021 cagaaataa

Antibody name: WR-8

Protein name: **cis-prenyltransferase, CIST**

Plant species: *Hevea brasiliensis*

NCBI Accession No.: gi |40716449

Antibody tag sequence: CDNFRRKPHEVQYV

Protein sequence:

1 mklytgerps vfrllgkymr kglygiltqg pipthlafil dgnrrfakkh klpeggghka
61 gflallnvlt ycyelgvkya tiyafsidnf rrkphevqyv mnlmlekieg mimeesiina
121 ydicvrfvgn lkllsepvkt aadkimrata nnskcvllla vcytstdeiv haveesseln
181 snevcnnqel eeanatgsgt viqtenmesy sgiklvdlek ntyinpypdv lirtsgetrl
241 snyllwqttn cilyspyalw peiglrhvvw svinfqrhys ylekhkeylk

Gene sequence:

1 atgaaattat acaccggtga gaggccaagt gtgttcagac ttttagggaa gtatatgaga
61 aaagggttat atggcatcct aacccagggt cccatcccta ctcatcttgc cttcatattg
121 gatggaaaca ggaggtttgc taagaagcat aaactgccag aaggaggtgg tcataaggct
181 ggatttttag ctcttctgaa cgtgctaact tattgctatg agttaggagt gaaatatgcg
241 actatctatg cctttagcat cgataatttt cgaaggaaac ctcatgaggt tcagtacgta
301 atgaatctaa tgctggagaa gattgaaggg atgatcatgg aagaaagtat catcaatgca
361 tatgatattt gcgtacgttt tgtgggtaac ctgaagcttt taagtgagcc agtcaagacc
421 gcagcagata agattatgag ggctactgcc aacaattcca aatgtgtgct tctccttgct
481 gtatgctata cttcaactga tgagatcgtg catgctgttg aagaatcctc tgaattgaac
541 tccaatgaag tttgtaacaa tcaagaattg gaggaggcaa atgcaactgg aagcggtact
601 gtgattcaaa ctgagaacat ggagtcgtat tctggaataa aacttgtaga ccttgagaaa
661 aacacctaca taaatcctta tcctgatgtt ctgattcgaa cttctgggga gacccgtctg
721 agcaactact tactttggca gactactaat tgcatactgt attctcctta tgcactgtgg
781 ccagagattg gtcttcgaca cgtggtgtgg tcagtaatta acttccaacg tcattattct
841 tacttggaga aacataagga atacttaaaa taa

Antibody name: WR-9

Protein name: Rubber elongation factor

Plant species: *Hevea brasiliensis*

NCBI Accession No.: gi |132270

Antibody tag sequence: drslppivkd asiqvvsa

Protein sequence:

1 maedednqqg qgeglkylgf vqdaatyavt tfsnvylfak dksgplqpgv diiegpvknv
61 avplynrfsy ipngalkfvd stvvasvtii drslppivkd asiqvvsair aapeaarsla
101 sslpgqtkil akvfygen

Gene sequence:

1 ttttgatctt ccatttttgc aaaaggaaat cttcgattat ggctgaagac gaagacaacc
61 aacaagggca gggggagggg ttaaaatatt tgggttttgt gcaagacgcg gcaacttatg
121 ctgtgactac cttctcaaac gtctatcttt ttgccaaaga caaatctggt ccactgcagc
181 ctggtgtcga tatcattgag ggtccggtga agaacgtggc tgtacctctc tataataggt
241 tcagttatat tcccaatgga gctctcaagt ttgtagacag cacggttgtt gcatctgtca
301 ctattataga tcgctctctt cccccaattg tcaaggacgc atctatccaa gttgtttcag
361 caattcgagc tgccccagaa gctgctcgtt ctctggcttc ttctttgcct gggcagacca
421 agatacttgc taaggtgttt tatggagaga attgagcccc aatttgcacc aattgcttcc
481 aactaagcaa gttaatgata tgctcaagaa tatatatcta ttgtgagctt tttttatgtt
541 ctcatcctga gtgttgagac tatgttttcg tttgaatatt atactgtgtt ttattatgtg
601 ttttgaatat tcataatgag aataaagggc caattgaatt attggccaat atgtaatgat
661 acataaattt cgtgattgag t

Antibody name: WR-10

Protein name: Small rubber particle protein

Plant species: *Hevea brasiliensis*

NCBI Accession No.: gi |14423933

Antibody tag sequence: gvvppvik qvsaqtysv

Protein sequence:

1 maeeveeerl kyldfvraag vyavdsfstl ylyakdisgp lkpgvdtien vvktvvtpvy

61 yipleavkfv dktvdvsvts ldgvvppvik qvsaqtysva qdaprivldv assvfntgvq

121 egakalyanl epkaeqyavi twralnklpl vpqvanvvvp tavyfsekyn dvvrgtteqg
181 yrvssylpll ptekitkvfg deas

Gene sequence:

1 ataatcagtt gatagcttcc acagtgtttt ccgaaaggca aatctttttt caaacttcag

61 cgactgcgtt ttgaatttgt gatttttaaa ggaaattttc aattatggct gaagaggtgg

121 aggaagagag gctaaagtat ttggattttg tgcgagcggc tggagtttat gctgtagatt

181 ctttctcaac tctctacctt tatgccaagg acatatctgg tccattaaaa cctggtgtcg

241 atactattga gaatgtggtg aagaccgtgg ttactcctgt ttattatatt ccccttgagg

301 ctgtcaagtt tgtagacaaa acggtggatg tatcggtcac tagcctagat ggcgttgttc

361 ccccagttat caagcaggtg tctgcccaaa cttactcggt agctcaagat gctccaagaa

421 ttgttcttga tgtggcttct tcagttttca acactggtgt gcaggaaggc gcaaaagctc

481 tgtacgctaa tcttgaacca aaagctgagc aatatgcggt cattacctgg cgtgccctca

541 ataagctgcc actagttcct caagtggcaa atgtagttgt gccaaccgct gtttatttct

601 ctgaaaagta caacgatgtt gttcgtggca ctactgagca gggatataga gtgtcctctt

661 atttgccttt gttgcccact gagaaaatta ctaaggtgtt tggagatgag gcatcataat

721 ctgcactgga tttggttatt tatctattgt gagctttttt atatgtactt attcagtgtt

781 tagaataagt ctttggtggt gtgttttgga tgtggaataa agggccaatt gcattgttgg

841 tcaatatata attatgtata acatttcgtg atttgagttg gaatctaaag gttttacaaa

901 aaaaaaaaaa aaaaaaaaaa aa

Antibody name: WR-11

Protein name: **hydroxynitrile lyase**

Plant species: *Hevea brasiliensis*

NCBI Accession No.: gi |**1223884**

Antibody tag sequence: CIENYKPDKVYKVE

Protein sequence:

1 mafahfvlih tichgawiwh klkpllealg hkvtaldlaa sgvdprqiee igsfdeysep
61 lltflealpp gekvilvges cgglniaiaa dkycekiaaa vfhnsvlpdt ehcpsyvvdk
121 lmevfpdwkd ttyftytkdg keitglklgf tllrenlytl cgpeeyelak mltrkgslfq
181 nilakrpfft kegygsikki yvwtdqdeif lpefqlwqie nykpdkvykv eggdhklqlt
241 ktkeiaeilq evadtyn

Gene sequence:

1 atggcattcg ctcattttgt tcttattcat accatatgcc acggtgcatg gatttggcac
61 aagctcaaac ccctccttga ggcacttggc cacaaggtta ctgcactgga ccttgcagca
121 agcggcgttg acccaaggca aattgaggag attggctcat ttgatgagta ttctgaaccc
181 ttgttgacgt tcttggaggc actccctcca ggggaaaagg tgattctggt tggcgagagc
241 tgtggaggac tcaatatagc aattgctgct gataaatact gtgaaaagat tgcagctgct
301 gttttccaca attcagtatt gccagacacc gagcactgcc catcttacgt cgtggataag
361 ctcatggagg tgtttcccga ctggaaagac accacgtatt ttacgtacac taaagatggc
421 aaggagataa ctggattgaa actgggcttc acgcttctga gggaaaattt atataccctt
481 tgcggtcctg aggaatatga actggcgaag atgttgacaa ggaagggatc attatttcaa
541 aatattttag ctaagcgacc attcttcact aaggaaggtt acggatcgat taagaaaatt
601 tatgtgtgga ccgaccaaga cgaaatattt ttacctgaat ttcaactctg gcaaatagaa
661 aactataaac cagacaaggt ttataaggtc gaaggtggag atcataaatt gcagcttaca
721 aagactaagg agatcgctga aattctccaa gaggtggctg atacctataa ttga

Antibody name: WR-12

Protein name: **superoxide dismutase (manganese)**

Plant species: *Hevea brasiliensis*

NCBI Accession No.: gi |**348137**

Antibody tag sequence: CQLHHQKHHQTYIT

Protein sequence:

1 malrslvtrk nlpsafkaat glgqlrglqt fslpdlpydy galepaisge imqlhhqkhh
61 qtyitnynka leqlndaiek gdsaavvklq saikfngggh vnhsifwknl apvregggel
121 phgslgwaid adfgslekli qlmnaegaal qgsgwvwlal dkelkklvve ttanqdplvt
181 kgptlvpllg idvwehayyl qyknvrpdyl kniwkvmnwk yasevyakec pss

Gene sequence:

1 atggctctgc gatctctagt gacccgaaag aacctaccct cagccttcaa ggccgccaca
61 gggctagggc aactgcgggg attgcagacc ttctctctcc ccgatctccc ttacgactat
121 ggtgccctgg agcccgcgat tagcggcgag ataatgcagc tgcatcacca gaaacatcac
181 cagacttata ttactaacta caacaaggct ctcgagcaac tcaacgacgc tatagagaag
241 ggcgattctg ctgccgtcgt caagttgcag agcgccatca agttcaacgg tggaggtcat
301 gtcaaccact ccattttctg gaagaatctt gcccccgtcc gtgaaggagg tggtgaactc
361 ccgcatggtt cccttggttg ggctattgac gcagattttg gctcattgga aaagttgata
421 caattaatga atgcagaagg tgctgcgctg caaggatctg gatgggtgtg gcttgccttg
481 gacaaagaat tgaagaagct tgtggttgag accactgcaa accaggatcc attggtaact
541 aaaggaccaa ctttagttcc tttgcttggt attgatgttt gggagcatgc atactactta
601 cagtacaaaa atgtcagacc agactatcta aagaacatat ggaaagtaat gaattggaag
661 tatgcaagtg aagtgtatgc caaagaatgc ccttcttctt ag

Antibody name: WR-13

Protein name: **rubber peroxidase 1**

Plant species: *Hevea brasiliensis*

NCBI Accession No.: gi |109809965

Antibody tag sequence: NFNNTNSPDPTLNC

Protein sequence:

1 mhisplmlva ialyailvgs srplayaqls ptfydqscpn vsniirgviq eslqsdsrig
61 aslirlhfhd cfvngcdasi lldntdties ekqaaannns argfdvvdtm karlesacpg
121 ivscadiltv saqqsvdlag gptwtnllgr rdsltasrsq anlsipgpfe tldqlkskft
181 avglnnntdl valsgahtfg raqcrtfspr lynfnntnsp dptlnttylq tlqqicpqgg
241 ngsvitnldl ttsdtfdney fsnllvgegl lqsdqelfnt tgadtvaivq nfsanqtaff
301 esfvesmlrm gnlsvltgti geirlncskv ngnssagaet llvssm

Gene sequence:

1 atgcatatct ctccattgat gttggtggcg atagctttat atgcaatttt agttggatca
61 tcaagacccc tggcctacgc tcagctatct ccaacatttt atgatcaaag ctgcccaaac
121 gtctccaaca ttattcgtgg agtcatccaa gaatccttgc aatctgattc caggattggt
181 gccagcctca tcaggcttca tttccatgac tgctttgtca atggttgcga tgcatcaatt
241 ttgttggaca atactgatac tatagagagt gagaaacaag ctgctgcaaa taacaactca
301 gctagaggtt ttgatgttgt tgatacaatg aaggctagat tggagagtgc ttgtcctggc
361 attgtttctt gtgctgatat tctaactgtt tcagctcaac aatctgttga tttggcagga
421 gggccaacat ggacaaatct acttggaaga agagatagct taacagcaag cagaagtcaa
481 gctaatctta gcattccagg tcctttcgag acccttgacc agctcaagtc caagttcacc
541 gccgtaggcc tcaacaacaa taccgatcta gttgccctat ctggtgctca cacgtttgga
601 agagctcaat gcagaacatt tagccccegt ttgtacaact tcaataacac taattctcca
661 gacccaacat tgaacacaac atatctgcaa acgcttcaac aaatatgtcc acaaggagga
721 aatgggagtg taataactaa tcttgatctc acaacttctg atacctttga taatgaatat
781 ttctccaatc ttctagttgg agaaggcctt ctccagagtg atcaagaact atttaacact
841 actggggctg atactgttgc tattgttcaa aattttagtg ctaatcagac tgcttttttt
901 gaaagctttg tggagtccat gctcagaatg ggaaatctca gcgttttaac aggaactatt
961 ggagagatta gattgaattg cagcaaagtt aatggaaatt catcagctgg agcagaaact
1021 cttcttgtta gctccatgta g

Antibody name: WR-14

Protein name: **calcium-dependent protein kinase 1, CDPK**

Plant species: *Hevea brasiliensis*

NCBI Accession No.: gi |289413843

Antibody tag sequence: CRQNQQQQSYYQQPE

Protein sequence:

1 mgccsskekp skseankgyr sggtgnlrqn qqqqsyyqqp eyqqqqavqh pkmtvpqtqt
61 qtpqtrpqqp qastpppvra aptnltarsv qtpqtilgkp ledikqyysl gkelgrgqfg
121 vtylctenst ghiyacksil krklinkgdr edikrevqim qhlsgqpniv efrgsyedrq
181 svhvvmelca ggelfdriia kghyserdaa rickdivnvv hachfmgvmh rdlkpenfll
241 askdegamlk ttdfglsvfi eegktyrniv gsayyvapev lrrsygkeid iwsagvilyi
301 llsgvppfwa etekgifdai legyidfess pwpsisdsak dlvrrmltqd pkrritsaqv
361 ldhpwikdgg aasdkpidsa vlsrmkqfra mnklkklalk viaenlseee ikglkamftn
421 mdtdksgtit yeelktglar lgsrlsetev kqlmeaadvd gngaidyief isatmhryrl
481 erdehlykaf qyfdkdssgy itrdelesam meygmgdeas ikeiisevdt dndgkinyee
541 fctmmrrgvq qpgklf

Gene sequence:

1 atgggttgtt gtagcagcaa ggagaagcct tcaaaatcag aggctaataa agggtacagg
61 tcaggaggca ctggtaattt aaggcagaac caacaacaac aatcttatta tcaacaacca
121 gaataccagc aacaacaggc cgtgcagcac ccaaaaatga ccgtccctca aactcaaaca
181 caaaccccgc aaacaaggcc ccaacaacct caggcatcaa ccccccctcc agtaagagca
241 gcacctacga accttacagc aagatcagtt caaacgccac agaccattct gggcaaaccc
301 ttggaagaca ttaagcagta ctacagtcta gggaaagaac tgggtagagg ccaatttggt
361 gttacttatt tgtgcacaga gaattccact ggccacattt atgcctgcaa atccatactg
421 aagagaaaac taatcaataa gggtgacaga gaagatatca agagagaggt tcagattatg
481 cagcacttgt ctgggcagcc aaatattgta gagtttaggg gttcctatga ggataggcaa
541 tctgttcatg ttgtgatgga gctttgtgct ggaggggaac tttttgatcg tattattgct
601 aaagggcatt attcggagag agatgccgct aggatttgca aggatattgt gaatgtggtg
661 cacgcttgcc attttatggg agtgatgcac cgtgatctca agccggagaa tttcttgctg
721 gctagcaagg atgaggggagc aatgttgaag accactgatt ttggactgtc tgtgttcatt
781 gaggaaggga aaacataccg taatatagtt ggtagtgctt attatgttgc tcctgaagta
841 ttgaggcgta gttatggaaa ggaaatagat atttggagtg caggagttat tttgtatatt
901 ctactcagtg gtgtacctcc attttgggct gaaactgaaa aaggaatatt tgatgctatt
961 ctagaaggat acattgattt tgaaagttca ccatggccat caatatctga cagtgccaaa
1021 gatctggtca ggagaatgct gactcaggac ccaaaaaggc gaattacttc agcacaagtt
1081 cttgaccatc catggattaa agatggaggt gcagcatcag acaagccaat agatagtgca
1141 gttctctcta gaatgaagca attcagggca atgaataagc tcaagaagct agcattaaag

1201 gttattgctg aaaatctctc tgaagaagaa attaaaggtc ttaaagcaat gttcacaaac
1261 atggacactg acaagagtgg cacaatcacc tatgaagagc tgaagacagg tttagctcga
1321 cttgggtcaa ggctctctga aactgaagtt aaacaattaa tggaagctgc cgatgtggat
1381 ggaaatggag caatcgacta cattgagttt atctctgcta caatgcatag atatcgactg
1441 gaaagagatg agcatctata caaagcattt cagtactttg ataaggatag cagtgggtat
1501 ataacaagag atgaattaga gtctgccatg atggagtatg gtatgggaga tgaggccagt
1561 attaaggaaa taatttctga ggtggataca gataatgatg ggaaaatcaa ctatgaagaa
1621 ttctgcacta tgatgagacg tggagtacaa caaccaggaa agcttttcta g

Antibody name: WR-15

Protein name: **ethylene-inducible protein, ETHI**

Plant species: *Hevea brasiliensis*

NCBI Accession No.: gi |1209317

Antibody tag sequence: GLGEAMVGINLNDKC

Protein sequence:

1 magtgvvavy gngaitetkk spfsvkvgla qmlrggvimd vvnpeqaria eeagacavma
61 lervpadira qggvarmsdp qlikeikqsv tipvmakari ghfveaqile aigidyvdes
121 evltpadeen hinkhnfrip fvcgcrnlge alrriregaa mirtkgeagt gnvieavrhv
181 rsvmgdirll rnmdddevft fakkiaapyd lvmqtkqlgr lpvvqfaagg vatpadaalm
241 mqlgcdgvfv gsgvfksgdp arraraivqa vthysdpdml aevscglgea mvginlndkk
301 verfanrse

Gene sequence:

1 atggctggaa ctggggtggt ggcagtttac ggcaacggag caatcaccga aaccaaaaag
61 tcccccttt ccgtgaaggt gggtctagcc cagatgctaa gaggcggtgt tatcatggac
121 gtcgtaaatc ccgagcaggc ccgcatagcc gaggaggctg gcgcttgtgc tgtcatggct
181 ttggagcgtg tgccagccga tatccgcgct caaggtggcg tcgcgcgcat gagcgaccct
241 cagcttatca aagaaattaa acagtccgtt accatccccg tcatggccaa ggcccgcatc
301 ggccacttcg tggaggccca aattcttgaa gccataggca tcgattacgt cgacgagagc
361 gaagtgctta cccctgccga cgaagagaac cacatcaaca aacataattt ccgtatcccc
421 ttcgtttgcg gttgtaggaa cttaggcgag gcgcttcgaa ggatacgcga aggagccgcc
481 atgattagga ccaaaggtga agcaggaaca gggaatgtta tcgaagcggt taggcacgtg
541 aggtctgtga tgggtgatat taggcttttg aggaatatgg acgacgatga ggtgttcact
601 ttcgccaaga agattgctgc gccgtacgat ttggttatgc agacgaagca gctagggagg
661 ctacctgtgg tgcagttcgc ggcgggaggg gtcgcgacgc cggcggatgc ggctttgatg
721 atgcagttgg gttgcgatgg ggtgtttgtg gggtctgggg tgttcaagag cgggggacccc
781 gcaaggaggg ccagagcaat tgtgcaggcg gtgacgcatt atagcgatcc cgacatgctc
841 gcggaggtga gttgcggtct cggtgaggct atggtgggta tcaatttaaa cgacaagaag
901 gtggaaaggt tcgcaaacag gtcggaatag

Antibody name: WR-16

Protein name: **ethylene receptor, ETHR**

Plant species: *Hevea brasiliensis*

NCBI Accession No.: gi |56900932

Antibody tag sequence: QQNPVGYTVPIQLPC

Protein sequence:

1 meacnciepq wpadellmky qyisdffial ayfsipleli yfvkksavfp yrwvlvqfga
61 fivlcgathl inlwtftmhs rtvaivmtta kvltaavsca talmlvhiip dllsvktrel
121 ilknkaaeld remglirtqe etgrhvrmlt heirstldrh tilkttlvel grtlaleeca
181 lwmptrsgle lqlsytlrqq npvgytvpiq lpvinkvfsc nravkispnc pvarirpfag
241 kympgevvav rvpllhlsnf qindwpelst kryalmvlml psdsarqwha yelelvevva
301 dqvavalsha aileesmrar dllmeqnval dlarreaeta irarndflav mnhemrtpmh
361 aiialssllq etgltpeqrl mvetilkssn llatlindvl dlsrledgsl qldlgtfnlr
421 avfrevlnli kpiasvkklp vtlnlapdlp eyaigdekrl mqtilnvvgn avkfskegni
481 sitafvarpe slrdlrfpdf fpvpsddhfy lrvqvkdegl ginlqdipkl ftkfaqnqsl
541 atrnsggsgl glaickrfvn lmeghiwies eglgkgctai fvvklailec sneskhpfmq
601 ngpkpnqgak qtsfmgpess llmddtggyq dgnkrtfwgt lxcdxttvss sdeclrvvxl
661 dhkvvfmdvc mpdglellsa ymkdftkrhe rplivalywn ankvtkencm kvgmdgvilk
721 pvsvdkmrgv lsdllehrvl feam

Gene sequence:

1 atggaggcat gcaattgtat tgagccacaa tggccagcgg atgaattgtt gatgaagtac
61 caatatattt cagatttctt cattgctctt gcttatttct ccattcctct agagctcatt
121 tattttgtta agaaatcagc agtttttcct tatagatggg tgctggtgca gtttggtgct
181 tttatagttt tatgtggggc aacacatctt attaacttat ggacttttac catgcattca
241 cgaactgtgg ctattgtaat gaccacagca aaagttttga ctgctgcagt ttcatgcgca
301 accgccctta tgcttgtaca cataattcct gatcttttga gtgttaaaac tagagaacta
361 attttgaaaa acaaggctgc agagcttgat agagagatgg gcctgatccg tacacaggaa
421 gaaacaggcc ggcatgttag gatgctgact catgagatca ggagcacact tgatagacat
481 accatactaa agaccacact tgttgaactg ggaaggacat tggcattgga agagtgtgcc
541 ttatggatgc ccaccaggtc tggattagag cttcaacttt cctacactct tcgtcagcag
601 aatcctgttg gatatactgt gcctatccag cttcctgtga ttaataaagt attcagttgt
661 aaccgtgctg taaaaatatc acctaactgt ccagtggcaa gaatacgtcc ttttgcagga
721 aaatatatgc ctggagaggt ggttgctgtt cgagtcccac ttcttcatct ctctaatttc
781 caaattaatg attggcctga gctttctacc aaacgctatg ctttgatggt tttgatgctt
841 ccctcagata gtgcaaggca gtggcatgct tatgagttgg agcttgttga agtagttgct
901 gaccaggtgg ctgttgctct atcacatgct gctatcttag aagagtcaat gagggcaagg
961 gatcttctta tggagcagaa tgttgcactt gatcttgcaa ggagagaagc agaaacagct

1021 atccgtgctc gcaatgattt cttagctgtc atgaaccatg agatgagaac tcccatgcat
1081 gcaattattg cactttcttc cttactgcaa gaaactggac tgacgcctga gcagcgcctg
1141 atggttgaga caattcttaa gagtagtaac ctgttggcta ctttaataaa tgatgtatta
1201 gacctttcaa ggcttgaaga tggcagtctt cagcttgacc tgggaacttt taatctacgt
1261 gctgtattca gggaggtcct taacttgatc aagcctattg catctgttaa aaagttgcct
1321 gttactttaa atttagctcc agatttgcca gaatatgcca ttggtgatga gaaacgtctt
1381 atgcaaacca ttttaaatgt tgttggtaat gctgtgaagt tctctaaaga aggcaacatc
1441 tcaataactg cttttgttgc tagaccagaa tcattaagag atcttcggtt tcctgatttt
1501 ttcccagtgc caagtgatga tcacttttat ttgcgtgtac aggtaaaaga tgagggacta
1561 ggtataaacc tccaagatat tcctaagttg tttactaaat ttgcacaaaa tcaatcattg
1621 gcaaccagaa attctggtgg cagtggactt ggccttgcaa tttgtaagag gtttgtaaat
1681 cttatggaag gacacatctg gatagagagt gaaggacttg gcaagggatg cactgctatc
1741 tttgttgtaa aacttgctat tcttgagtgc tcaaatgaat cgaagcatcc tttcatgcag
1801 aatgggccca agccaaatca aggggccaaa caaacttctt ttatggggcc tgaaagttct
1861 ttgttaatgg atgatacggg gggttaccag gacggtaaca aaaggacttt ttggggcact
1921 ttggnttgtg atgngaccac tgtgagctcc agcgatgagt gtttacgagt agtttntctg
1981 gaccacaagg tggtatttat ggatgtgtgc atgcctgatg gtttggagtt gctatccgca
2041 tacatgaaag atttcacaaa acgtcatgaa aggccattaa tagtggcgct ttactggaac
2101 gcaaacaagg tgacaaagga aaactgcatg aaggtcggca tggatggtgt aatactgaaa
2161 cctgtctctg ttgacaagat gaggggtgtt ttatcagatc ttctggagca tcgggttcta
2221 ttcgaggcca tgtaa

Antibody name: WR-17

Protein name: **glutathione reductase, GLUR**

Plant species: *Hevea brasiliensis*

NCBI Accession No.: gi |186920319

Antibody tag sequence: CMKNTISGRQEKTVM

Protein sequence:

1 avfcipplsv vglseeqaie qakndilvft stfnpmknti sgrqektvmk lvvdaetdkv
61 lgasmcgpda peiiqgiava lkcgatkaql dstvgihpst aeefctm

Gene sequence:

1 gcagtgtttt gcataccacc tttgtctgta gtaggcctca gtgaagagca ggcaatagag
61 caagcaaaaa atgatatatt ggttttcact tcaaccttca atcctatgaa gaatactatc
121 tctggacggc aagagaagac agtgatgaag cttgttgttg atgctgagac agataaagtt
181 cttggagctt caatgtgtgg gcctgatgct cctgaaatta tacagggaat tgctgttgca
241 ttgaagtgcg gagcaacaaa ggcccaattg gacagcacgg tgggaataca cccttctact
301 gctgaggaat tctgtaccat g

Antibody name: WR-23

Protein name: **latex cyanogenic beta glucosidase, LACG**

Plant species: *Hevea brasiliensis*

NCBI Accession No.: gi |31580730

Antibody tag sequence: LDDKLRVRYFKGYLC

Protein sequence:

1 mmkekflkeh pyllekevsr sdfppnflfg vatsayqieg gcreggrgps iwdafshtkg
61 tildgsngdv avdhyhryke dieliaklgf dayrfslsws rifpdglgtk vneegiafyn
121 niinallekg iepyitlyhw dlplhlqesm ggwlnkeivk yfaiyadtcf asfgdrvkkw
181 itlneplqta vngfdtgila pgkhehsyte pflashhqil ahatavsiyr smykdnqgge
241 vglvvdcewa esnsdkiedk aaaakrlefq lgwylhplyy gdypevmrki lggglpkfse
301 edkellrnsl dfiglnhyss rfikhvtdsp aecyyykaqe ierlakwedg epigeraase
361 wlyvrpwglr kvlnyivqry nnpiiyvten gmddedssap lhemlddklr vryfkgylaa
421 vaqaikdgad vrgyfawsll dnfewaqgyt krfgliyvdy knglarhpks saywfmrflk
481 gdegkngke

Gene sequence:

1 atgatgaagg agaaattctt gaaggaacat ccatatttgc tggaaaagga ggtctctcgc
61 tctgattttc ctcctaattt tctctttggt gttgccactt ctgcctatca gattgaaggt
121 ggctgcaggg aaggtggtag aggtcctagt atatgggatg ctttttcaca caccaaagga
181 actattcttg atggaagcaa tggtgatgtg gcagtggatc attatcatcg atacaaggaa
241 gacattgaac tcatcgctaa gttggggttt gatgcttatc gattttcctt atcatggtct
301 cgcattttcc ctgatggttt gggaaccaaa gtcaatgaag aagggatagc attctataac
361 aatatcatta atgctcttct tgaaaagggt atagagccat atataactct gtaccattgg
421 gatctcccct tgcatcttca agagtcaatg ggagggtggt tgaataagga aattgtaaaa
481 tactttgcaa tctatgcaga cacttgcttt gcaagttttg gtgatagagt taagaaatgg
541 attactctaa atgagcctct ccaaacagca gtaaatggat ttgatactgg aatacttgct
601 cctggaaaac atgaacattc atatacagaa ccatttttgg cttcacacca ccagatcttg
661 gcccatgcaa cagctgtttc catataccgc agtatgtaca aagacaatca aggaggagaa
721 gtaggcttgg tggtggactg tgaatgggct gaatctaatt cagataaaat tgaagataaa
781 gctgctgctg caaaacggct tgagtttcag cttggatggt atttgcatcc tttatattat
841 ggagactatc ctgaagttat gcgtaaaata ctgggaggtg ggctcccaaa attctcagag
901 gaagataagg aattacttag gaactcactt gacttcattg gtctaaatca ctattcttca
961 agattcatca agcatgttac agatagccct gcagaatgct attactataa agcccaagaa
1021 attgagagac ttgctaaatg ggaagatggt gagccaatcg gcgagagggc agcatcagaa
1081 tggctctatg ttcgtccttg gggacttcgg aaggttctca attacatagt ccagagatac
1141 aataatccca taatttacgt tactgagaat ggtatggatg atgaagactc cagtgcccct
1201 ctccatgaaa tgctagatga caaactgaga gttcgctatt ttaaaggata ccttgctgca

1261 gttgctcagg caatcaaaga cggagcagat gtgaggggat attttgcatg gtcattgttg
1321 gacaattttg aatgggctca aggttatacc aagcgttttg gtttgatcta tgtggattac
1381 aagaatggac ttgctcgcca cccaaagtct tctgcttatt ggttcatgag gttcttgaaa
1441 ggtgatgaag ggaaaaatgg caaagagtga

Antibody name: WR-25

Protein name: **beta-cyanoalanine synthase, CYAS**

Plant species: *Hevea brasiliensis*

NCBI Accession No.: gi |326909405

Antibody tag sequence: CQRIRDLPKNLPGTK

Protein sequence:

1 matlrnllkk ksltsnelai rrfvsseaaa espsfaqrir dlpknlpgtk iktevsqlig
61 rtplvylnkm segcgayiav kqemmqptas ikdrpafsmi ndaekknlit pgktvliept
121 sgnmgismaf maamkgykmv ltmpsytsle rrvtmkafga elivtdptkg mggtvkkayd
181 llestpnafm lqqfsnpans kihfettgpe iwedtlghvd ifvmgigsgg tvsgvgqylk
241sqnpnvkiyg vepaesnvln ggkpgphqim gngvgfkpdi ldmdvmekvl evssedavkm
301 arrlaleegl mvgissgant vaalrlarmp enkgklivtv hpsfgeryls svlfeelrye
361 aanmqpvpvd

Gene sequence:

1 atggctactc ttaggaactt gttgaagaaa aaatctttaa cgtccaacga gcttgctata
61 aggaggttcg tctcttccga ggctgctgct gaatctcctt cttttgctca aagaatcagg
121 gatctgccca agaatctccc tggaactaaa atcaagactg aagtttctca acttattggg
181 agaactcccc ttgtttatct taacaaaatg agtgaaggat gtggagctta catagccgtc
241 aagcaagaga tgatgcaacc aactgccagc atcaaagaca ggccggcgtt ttcaatgatc
301 aatgatgcag aaaagaagaa tttaatcact cccgggaaga cggtcttgat agagccaaca
361 tctggtaata tggggattag tatggctttt atggcagcca tgaaaggata caagatggtt
421 ctaaccatgc cctcttacac aagcttggag agaagggtga ctatgaaggc atttggagct
481 gagctaattg tcactgatcc caccaagggg atgggtggaa cagttaagaa ggcctatgat
541 cttttggaat ccacaccaaa tgctttcatg ctacaacaat tttcaaatcc tgcaaattct
601 aagatccatt ttgaaacgac aggtcctgaa atttgggagg atacacttgg tcatgttgac
661 atctttgtaa tgggaatagg cagtggagga acagtctctg gcgttgggca gtaccttaaa
721 tctcaaaatc ctaatgttaa gatatatggg gtggagcctg ctgaaagtaa tgtgctcaac
781 ggtggtaaac caggtcctca tcaaattatg ggtaacggag ttggatttaa accagacata
841 ttggacatgg atgtaatgga aaaggttctt gaggttagca gtgaagatgc agtaaaaatg
901 gctaggagat tggcattgga ggaggggctt atggtgggaa tatcatctgg agccaacaca
961 gttgctgcac ttagacttgc tagaatgcca gagaacaaag gaaaactcat cgtgactgtt
1021 catccaagtt tcggggagcg atacttgtca tctgtcctgt ttgaagaact gagatatgag
1081 gctgcaaaca tgcaaccagt tccagttgac taa

Antibody name: WR-28

Protein name: ascorbate peroxidase, ACPX

Plant species: *Hevea brasiliensis*

NCBI Accession No.: gi |27449248

Antibody tag sequence: HPGREDKPEPPPEC

Protein sequence:

1 mtknypkvse eyqkaidkak rklrgfiaek gcaplmlria whsagtydan tktggpfgtm
61 rhaaeqahaa nngldiavrl lepikqqfpi lsyadfyqla gvvaveitgg peipfhpgre
121 dkpepppegr lpnatkgadh lrevfgktmg lsdkdivvls gghtlgrchk ersgfdgpwt
181 anplifdnsf ftellagqke gllqlptdtv lvtdpvfrpy vekyaadeda ffadyaeahv
241 klselgfaea

Gene sequence:

1 atgacgaaga actacccaaa agttagtgaa gagtaccaga aggccattga taaggccaag
61 aggaagctca ggggtttcat cgctgagaag ggctgtgctc ctctcatgct ccgtatcgca
121 tggcactcag ctggaactta cgacgcgaac accaagactg gaggtccatt tggaaccatg
181 aggcacgcag cggagcaggc ccatgctgct aacaatgggc tggatattgc tgtcagactc
241 cttgagccca tcaagcagca gttccctatc ctctcctatg ctgacttcta tcagcttgct
301 ggtgttgttg ccgttgagat cactggtggg cctgagatcc cattccaccc tggcagagag
361 gacaagcctg aaccacctcc agaaggtcgt ctgcctaatg ctactaaagg tgctgatcac
421 ttgagggagg tctttggcaa aaccatgggt ctcagtgaca aggatatcgt tgttctctct
481 ggtggccaca ccctgggaag gtgccacaag gagcgctctg gttttgacgg gccctggact
541 gctaacccac tcatctttga caattctttc ttcacggagc tcttggctgg acagaaggaa
601 ggccttctac aattgccaac tgacaccgtt cttgttaccg atcctgtctt ccgcccatat
661 gttgaaaaat atgctgctga tgaagatgca ttctttgctg attatgctga ggcccatgtg
721 aagctctccg agctggggtt tgctgaggct taa

Antibody name: WR-29

Protein name: **heat-shock protein 70, HSP70**

Plant species: *Hevea brasiliensis*

NCBI Accession No.: gi |186898205

Antibody tag sequence: EKYKAEDEEVKKKC

Protein sequence:

1 mapktegkai gidlgttysc vgvwqndrve iiandqgnrt tpsyvaftdt erligdaakn
61 qvamnpqntv fdakrligrr fsdpsvqsdm khwpfkvisg pgdkpmivvq ykgeekqfap
121 eeissmvltk mkeiaeaylg qtvknavitv payfndsqrq atkdagaiag vnvmriinep
181 taaaiaygld kkgsstgekn vlifdlgggt fdvslltiee gifevkatag dthlggedfd
241 nrlvnhfvae fkgkhkkdis tnaralrrlr tarerakrtl sstsqttiei dslyegidfy
301 atitrarfee lnmdlfrkcm epvekclrds kidksqvhdv vlvggstrip kvqqllqdif
361 ngkelcksin pdeavaygaa vqaailsgeg dekvqdllll dvtplslgie taggvmtvli
421 prnttiptkk eqifstysdn qpgvliqvye gerartkdnn llgkfeltgi ppaprgvpqi
481 nvcfdidang ivnvsaedkt agvknkitit ndkgrlskee iermvqeaek ykaedeevkk
541 kveaknslen yaynmrntvk ddkfagklap adkgkiekai detiewldrn qlaeveefed
601 klkeleglcn piiakmyqgg aggdvpvgga empnsnygkt ssggsgagpk ieevd

Gene sequence:

1 atggcgccaa aaactgaagg caaggccatt ggtatcgatc ttggcaccac ctacagctgc
61 gtgggtgtgt ggcagaacga ccgggttgag atcatagcca atgatcaggg caacagaact
121 actccttctt atgttgcctt cactgacacc gagcgtctca ttggggatgc agctaagaac
181 caagttgcta tgaaccctca aaacactgtc tttgatgcca aacgtctcat tggtaggcgg
241 ttttctgatc cttctgtgca gagcgacatg aagcactggc ctttcaaggt tatttctggt
301 cctggtgata agcctatgat tgtggtgcaa tacaaaggtg aagaaaagca atttgcgcct
361 gaggagattt cttctatggt gctaaccaag atgaaggaga ttgcagaggc ttatctgggt
421 cagacagtga agaatgccgt gatcacagtg cctgcttatt tcaatgactc gcaaaggcag
481 gccaccaagg atgctggtgc tattgcaggg gtgaacgtca tgagaattat caatgagcct
541 acagctgcag ccattgctta cggtttggac aagaaaggtt cgagtactgg agaaaagaac
601 gtgttgattt tcgatcttgg tggagggaca tttgatgttt ctcttctgac gatcgaggag
661 ggtatatttg aagtgaaggc aacagctgga gacacccacc ttggaggaga ggacttcgat
721 aataggcttg tcaaccattt cgttgcagag ttcaagggga agcacaagaa ggatattagt
781 actaatgcga gagccttgag aaggttgaga accgcccgcg agagggcaaa gaggactctg
841 tcttcaacct cgcaaacaac cattgagatt gattctctct acgaagggat tgatttctat
901 gcgaccatca ctagagcaag atttgaagag ctgaacatgg atttgtttag aaagtgcatg
961 gaacccgtgg agaaatgtct tcgtgactcc aagatcgata agagtcaagt tcatgacgtt
1021 gtgctcgttg gtgggtcaac aagaattcct aaagttcagc aacttttgca agatatcttc
1081 aacggaaagg agctctgcaa gagcataaac cccgacgagg ctgtcgctta tggagcagct

1141 gtccaagctg caattcttag tggtgaaggg gatgagaaag ttcaagattt acttctcttg
1201 gatgttactc ctctcagcct cggaattgag actgctggtg gtgtaatgac agtcttgatt
1261 ccaaggaaca ccactattcc taccaaaaaa gagcaaatct tctctacata ctctgataat
1321 caaccaggag tgcttattca ggtgtatgaa ggtgaaagag ctagaaccaa ggataataat
1381 cttcttggaa aattcgagct caccggaatc cctcctgcac ccagaggcgt tcctcagatt
1441 aatgtgtgtt tcgacattga cgccaatggt attgtgaatg tctctgctga ggataagact
1501 gctggagtga agaacaagat aacaatcact aacgataaag gaagattgag caaggaagag
1561 attgaaagaa tggtgcaaga agcagagaag tacaaagcag aggatgagga ggtgaagaag
1621 aaggtggagg ccaagaactc tttggagaac tacgcataca acatgagaaa tacagtgaag
1681 gatgacaagt tcgccggaaa attggctcca gccgacaagg ggaagattga aaaggcaatt
1741 gatgagacta ttgagtggtt ggacagaaac caattagcag aggtggagga gtttgaggac
1801 aagttgaagg agcttgaggg cttgtgcaat ccaattattg caaagatgta tcagggcggt
1861 gctgggggtg acgtaccagt gggtggcgcc gaaatgccga acagcaacta tggcaagact
1921 agctccggcg gttctggtgc cgggcccaag atcgaagaag ttgattaa

Antibody name: WR-30

Protein name: **aquaporin**

Plant species: *Hevea brasiliensis*

NCBI Accession No.: gi |307826678

Antibody tag sequence: CQSQDDKDYTEPPA

Protein sequence:

1 megkeedvrl gankyretqp igtsaqsqdd kdyteppaap lfepteltsw sfyragiaef
61 iatflflyis vltvmgvvka ptkcstvgiq giawsfggmi falvyctagi sgghinpavt
121 fglflarkls ltralyymvm qclgaicgag vvkgfegrhq ytllgggans vnpgytkgdg
181 lgaeivgtfv lvytvfsatd akrnardshv pilaplpigf avflvhlati pitgtginpa
241 rslgaaiifn kdkgwddhwi fwvgpfigaa laalyhqvvi raipfkk

Gene sequence:

1 atggagggca aggaagaaga tgttagattg ggagctaaca aatataggga gacgcagccc
61 attggtacgt cagctcagag ccaagatgac aaggactaca ctgaaccacc agcagcgccg
121 ctgttcgagc caacggagct cacttcttgg tccttttaca gggctggtat tgcagagttc
181 atagccactt tcttgttctt atacatctct gttttgactg tcatgggtgt agttaaggca
241 cccaccaagt gttcgactgt tgggattcag gggatagctt ggtcctttgg tggcatgatc
301 ttcgctcttg tctattgtac tgctggcatt tcaggaggtc acataaaccc ggcggtgacg
361 tttgggctgt ttttggcaag gaaactgtcc ttgacaaggg ctttgtacta catggtgatg
421 cagtgccttg gagccatatg tggtgctggt gtagtgaaag gatttgaagg gcgtcaccag
481 tatactttgt tgggtggtgg tgccaatagt gtgaacccag gttacaccaa aggtgatggg
541 cttggtgctg aaattgttgg cacctttgtt cttgtctaca ctgtcttctc tgctactgat
601 gccaaacgta atgccagaga ctcccatgtt cctattttgg cacctttgcc aattgggttc
661 gctgtgttct tggtgcactt ggctaccatc ccaattacag gaactggtat caacccagct
721 cgtagtcttg gtgcagcaat catcttcaac aaggacaagg gctgggatga tcactggatt
781 ttctgggtgg gtccattcat tggagcagca cttgcagctc tataccacca agttgtgatc
841 agagccatcc ctttcaagaa gtga

Antibody name: WR-31

Protein name: **Geranylgeranyl diphosphate synthase, GGDS**

Plant species: *Hevea brasiliensis*

NCBI Accession No.: gi |14325238

Antibody tag sequence: QEQLAGFDPEKAAPC

Protein sequence:

1 mssvnlgswv htsyvlnqat rsrsksksfs lpfnplksla isfayrkser pissvsaiit
61 keeetlqeeq nnpppsfdfk symlqkgnsi nqaleaaipl qepakihesm rysllaggkr
121 vrpalclaac elvggndsma mpaacavemi htmslihddl pcmdnddlrr gkptnhivfg
181 edvavlagda llafafehia vstlnvssar ivravgelak aigaeglvag qvvdinsegs
241 sevdleklef ihihktakll egavvlgail gggtdeevek lrkyardigl lfqvvddild
301 vtkssqelgk tagkdlvadk vtypkllgie ksrefaekln keaqeqlagf dpekaaplia
361 lanyiahrqn

Gene sequence:

1 atgagttcag tgaatttggg ctcatgggtt cacacctcct acgtcttaaa ccaagctacc
61 agatccagat ccaaatccaa atccttctct ctacctttca atcctctaaa aagtttagca
121 atttcctttg cttatagaaa atcagagcga cccatttcat ctgtctctgc gattattacc
181 aaggaagaag aaactcttca agaagagcag aataatccac caccctcttt tgatttcaaa
241 tcctacatgc tccaaaaagg caattccatt aaccaagctt tagaagctgc cattccactc
301 caagaacccg ctaaaattca cgagtctatg cgttattccc tcttggccgg cggcaagagg
361 gtacgaccgg ccctctgcct cgctgcgtgt gagcttgttg gtgggaatga ctccatggcg
421 atgcctgctg catgcgctgt ggaaatgatt catactatgt ctcttatcca tgatgacctc
481 ccttgcatgg ataacgacga tctccgccgt ggcaagccca ccaatcacat cgtgtttgga
541 gaggacgtgg cggttctcgc cggtgacgca ctcctagcat ttgcttttga acacatcgct
601 gtttctactt taaatgtttc ttctgctaga attgtccggg cagttgggga attagcgaag
661 gcgatcgggg cagaagggtt agttgctggc caagtagttg atataaattc tgagggctca
721 tctgaggtgg atttagagaa gcttgaattt attcacatcc acaagaccgc taagttgttg
781 gagggggctg tggtgctagg ggctatattg ggcggaggaa ccgatgagga agtggagaaa
841 ttgaggaaat atgctaggga tattgggttg ttgttccaag ttgttgacga tattcttgat
901 gtgactaaat catcccaaga attggggaaa actgcaggca aggacttggt ggcggacaag
961 gttacatatc ccaagctttt ggggattgag aagtcgaggg aatttgcaga gaagctgaat
1021 aaggaagctc aggagcagct ggctggattt gatcctgaaa aggcagctcc attgattgct
1081 ttggctaatt acatcgctca caggcaaaac taa

Supplementary Figure S7: **Primers used for RT-PCR**

Protein name: 14-3-3 protein

Spot No.: I-27

Accession No.: tr|F2YZ43|F2YZ43_HEVBR

Primer sequences:

Primer-up: CGTGGATGTTGAGGAGCTAACTG

Primer-down: CAGAAGTCCAGAGTGTCAGATTGTC

Protein name: (ACAT) Acetyl-CoA C-acetyltransferase

Spot No.: I-13

Accession No.: tr|A9ZMZ4|A9ZMZ4_HEVBR

Primer sequences:

Primer-up: GGTTGGATGCTTCTCAAGTTGATTAC

Primer-down: CAAGAGTTCGCCCCATCGTG

Protein name: (ACPX) Ascorbate peroxidase

Spot No.: I-228

Accession No.: tr|Q8GZP1|Q8GZP1_HEVBR

Primer sequences:

Primer-up: GGCTGGATATTGCTGTCAGACTC

Primer-down: CGGAAGACAGGATCGGTAACAAG

Protein name: (ADPF) actin-depolymerizing factor

Spot No.: S-170

Accession No.: gi|298362843

Primer sequences:

Primer-up: TGCGGCTACTGTATCCAAACC

Primer-down: CTGCTTCACCATCCCACAAAC

Protein name: (ABAS) Abscisic acid stress ripening-related protein 1

Spot No.: I-160

Accession No.: tr|Q6XNP8|Q6XNP8_HEVBR

Primer sequences:

Primer-up: GGCACAAGATAAAAGAGGAGGTAGC

Primer-down: GCGTATGGAGTTAAAAGGGATGG

Protein name: (ATPS) ATP synthase subunit beta

Spot No.: I-188

Accession No.: sp|P29685|ATPBM_HEVBR

Primer sequences:

Primer-up: GTTGGTCTAACTGGGCTAACTGTGG

Primer-down: TCTTGGTGGTTGTAATACGCTCTTG

Protein name: (CIAD)Cinnamyl alcohol dehydrogenase

Spot No.: I-254

Accession No.: tr|E9M774|E9M774_HEVBR

Primer sequences:

Primer-up: TGGTCCTATAATGATGTCTACACCGAT

Primer-down: CTTGTTCAACCCAAAGTGGCTAAG

Protein name: (CIBP)Citrate-binding protein

Spot No.: S-145

Accession No.: sp|Q39962|CBPR_HEVBR

Primer sequences:

Primer-up: GATCCAACTGATGGGTTCACTGAG

Primer-down: GCTTGATGTTTTTCCACCTTGATTC

Protein name: Copper transport protein ATOX1

Spot No.: I-578

Accession No.: tr|D4P094|D4P094_HEVBR

Primer sequences:

Primer-up: GAAAGGAAACGTGCAGCCAGAG

Primer-down: ACCGAGTGTCGTATAAGGGTGCT

Protein name: chitinase

Spot No.: D-72

Accession No.: gi|3451147

Primer sequences:

Primer-up: GCAACGAAGGAACTCTAACACAAAC

Primer-down: GAATCCGAGAAATCAGCACATCC

Protein name: caffeic acid 3-O-methyltransferase

Spot No.: D-125

Accession No.: gi|380468126

Primer sequences:

Primer-up: AGTTGATGTGGGCGGTAATAGTG

Primer-down: TGTCGGGTCCTCTCTTTTCCTC

Protein name: Cysteine protease

Spot No.: I-252

Accession No.: tr|Q155L4|Q155L4_HEVBR

Primer sequences:

Primer-up: CTTGTTCTTCGCTTCCACCTTGT

Primer-down: GGTCCGATTCTCCGAGTTATGCT

Protein name: Cis-prenyl transferase

Spot No.: I-351

Accession No.: tr|A9ZN23|A9ZN23_HEVBR

Primer sequences:

Primer-up: GGTCCCATCCCTACTCATCTTGC

Primer-down: CTCATACTCTGATCTGCTGCGGTC

Protein name: Cis-prenyl transferase OS=Hevea brasiliensis

Spot No.: I-1585

Accession No.: tr|Q8W3U4|Q8W3U4_HEVBR

Primer sequences:

Primer-up: GGAGGTGGTCATAAGGCTGGAT

Primer-down: TCTGCTGCGGTCTTGAGTGG

Protein name: Cu/Zn superoxide dismutase

Spot No.: D-17

Accession No.: gi|27449246

Primer sequences:

Primer-up: GTTATTACCAGCAGTGAGGGCATTAG

Primer-down: GACCTTCGTGAAAAACAACTGACC

Protein name: eukaryotic translation initiation factor 5A

Spot No.: S-91

Accession No.: gi|33325129

Primer sequences:

Primer-up: CGCAAGAATGGTTACATCGTC

Primer-down: TCCCTTCCACTCAATAGCAATC

Protein name: Eukaryotic translation initiation factor

Spot No.: I-98

Accession No.: tr|B9SJV6|B9SJV6_RICCO

Primer sequences:

Primer-up: CGCAAGAATGGTTACATCGTC

Primer-down: TCCCTTCCACTCAATAGCAATC

Protein name: Eukaryotic translation initiation factor 1Aa

Spot No.: I-673

Accession No.: tr|F8RW94|F8RW94_HEVBR

Primer sequences:

Primer-up: AAGGGAAAGGGAGGTAAGAATAGGA

Primer-down: GATAGTCACGCAGCCCAACAAGT

Protein name: eukaryotic translation elongation factor

Spot No.: D-8

Accession No.: gi|255565836

Primer sequences:

Primer-up: TGCCACGGCTCAGAAGTATCG

Primer-down: GCAACCTTACATTGAACAGCCAC

Protein name: Enolase 1

Spot No.: D-6

Accession No.: gi|14423688

Primer sequences:

Primer-up: CGTTGCTAACCTCGCTGGTAAC

Primer-down: GCATAGTGCTCCCAGTCATCTTG

Protein name: Esterase

Spot No.: D-95

Accession No.: gi|51315784

Primer sequences:

Primer-up: GATTTTATCGCCGAGAGTTTCAATC

Primer-down: ACCGTCGTCTGCTGTAACTGTATCT

Protein name: Elicitor-responsive protein

Spot No.: I-30

Accession No.: tr|F8SKC5|F8SKC5_HEVBR

Primer sequences:

Primer-up: AATGCCTCTAGGAACTGTTGAAGTC

Primer-down: ACCGAAAATCGTATCCATCCAC

Protein name: Farnesyl diphosphate synthase

Spot No.: I-133

Accession No.: tr|Q8L7F4|Q8L7F4_HEVBR

Primer sequences:

Primer-up: ATGGGTTGAGCGGATGTTGG

Primer-down: CTGGGAATGTGATTGCGAAGC

Protein name: Gamma-glutamylcysteine synthetase

Spot No.: I-1621

Accession No.: tr|D5LYI6|D5LYI6_HEVBR

Primer sequences:

Primer-up: TGCCAGCATTTTGGGTAGGAT

Primer-down: GTTCTAACCACCTCAGCCACTTCA

Protein name: Beta-1,3-glucanase

Spot No.: I-2

Accession No.: tr|D1M8S7|D1M8S7_HEVBR

Primer sequences:

Primer-up: ATCTCCAAAGCCTTACCAATCC

Primer-down: TGGTGAAGTGAACAAAGCATAGG

Protein name: Glutaredoxin

Spot No.: I-264

Accession No.: tr|B3FNP8|B3FNP8_HEVBR

Primer sequences:

Primer-up: TGGATACTGAGGGCGATGGAA

Primer-down: CTTTTAAGCAGAAGCCTTAGCAAGAG

Protein name: Glyoxylase I

Spot No.: I-812

Accession No.: tr|E9KK00|E9KK00_HEVBR

Primer sequences:

Primer-up: CTCCAAGTGACCCAGTTGAAAGA

Primer-down: GAAATACCAATATGTCCAAAGCCAC

Protein name: (GLUS) glutamine synthetase

Spot No.: S-56

Accession No.: gi|2213877

Primer sequences:

Primer-up: TGGTACGGCATTGAGCAAGAATAC

Primer-down: AGCAACGCCCCATAAGAAAGTG

Protein name: (GSTF) glutathione-S-transferase tau 1

Spot No.: D-53

Accession No.: gi|333690873

Primer sequences:

Primer-up: ATGGACTACAAAGGGAGATGAGCA

Primer-down: GCACCTCTTAGCCCAAGCAATC

Protein name: Pro-hevein

Spot No.: I-817

Accession No.: sp|P02877|HEVE_HEVBR

Primer sequences:

Primer-up: CTATGTTGTAGCCAGTATGGGTGG

Primer-down: GTAGTTCACTGTAAGATGACCTCGTTC

Protein name: Hevein

Spot No.: I-1357

Accession No.: tr|Q7M1L9|Q7M1L9_HEVBR

Primer sequences:

Primer-up: CTATGTTGTAGCCAGTATGGGTGG

Primer-down: GTAGTTCACTGTAAGATGACCTCGTTC

Protein name: Hevamine

Spot No.: D-93

Accession No.: gi|157831407

Primer sequences:

Primer-up: CATAGAGCATGGTTCAACCCTGTAC

Primer-down: GACCAAAGCATAACACCTCCGTAC

Protein name: (Hev b5) Major latex allergen Hev b 5

Spot No.: I-5

Accession No.: sp|Q39967|ALL5_HEVBR

Primer sequences:

Primer-up: CAAGAAACCGCCGATGCTACA

Primer-down: CGGAACTTCTGTGGTGGCTGTC

Protein name: (HMGR) Hydroxymethylglutaryl coenzyme A reductase

Spot No.: I-119

Accession No.: tr|Q547W6|Q547W6_HEVBR

Primer sequences:

Primer-up: TCCGTCGTGGATGGGAAGAT

Primer-down: TGGTCGCCATTGGAACAGAGT

Protein name: (HMGS) Hydroxymethylglutaryl coenzyme A synthase

Spot No.: I-22

Accession No.: tr|Q944F8|Q944F8_HEVBR

Primer sequences:

Primer-up: CAGTGCGGTCTATGCAGAGGGT

Primer-down: GCAAGAATCAAGAGCCATGAGGT

Protein name: (HMGS-2) HMG-CoA synthase 2

Spot No.: I-1434

Accession No.: tr|Q6QLW8|Q6QLW8_HEVBR

Primer sequences:

Primer-up: TGATCCTAACCAAATCGGTCGTC

Primer-down: GCGTCCATCCCATGAATTACTCT

Protein name: Heat shock protein

Spot No.: I-11

Accession No.: tr|Q71EE1|Q71EE1_HEVBR

Primer sequences:

Primer-up: GGAGAATATGGCTGGACTGCTAAC

Primer-down: CGTCAATGCTCAGACCCAGTTTC

Protein name: latex protein allergen Hev b 7

Spot No.: D-76

Accession No.: gi|6707018

Primer sequences:

Primer-up: TTCTTCTCCCAGTCATCTTCTCATC

Primer-down: CTTTCAAGCCTGCCAGTATCC

Protein name: (LAP1) latex abundant protein 1

Spot No.: D-111

Accession No.: gi|37901124

Primer sequences:

Primer-up: ACTATGATTATCCCGACTTCACCTG

Primer-down: TCACATAGATTGCACAACGGAAC

Protein name: (LAC) latex cystatin

Spot No.: D-102

Accession No.: gi|268321205

Primer sequences:

Primer-up: GGCAAAACTAGGAGGTGTTAAGGAAG

Primer-down: GCTGTAGAGTCTGAAGGGGCATC

Protein name: (MEVD) Mevalonate disphosphate decarboxylase

Spot No.: I-18

Accession No.: tr|Q944G0|Q944G0_HEVBR

Primer sequences:

Primer-up: CACCACAGGAATGCGTGAGACT

Primer-down: CGTTCATGTAGAAAATTGGAGGACA

Protein name: (MEVK) Mevalonate kinase

Spot No.: I-1957

Accession No.: tr|Q944G2|Q944G2_HEVBR

Primer sequences:

Primer-up: TCTGATCTTCCACTGGGTTCAGG

Primer-down: CTTGATGCGAGTCAGATTACCAGAC

Protein name: MYC2

Spot No.: I-159

Accession No.: tr|D2CRK4|D2CRK4_HEVBR

Primer sequences:

Primer-up: AAGAGGAAGAACCCAACTGAACG

Primer-down: ATCGGTGCTCTGATTGTCGGT

Protein name: (NUDK) nucleoside diphosphate kinase 1

Spot No.: S-93

Accession No.: gi|312985291

Primer sequences:

Primer-up: CTAATCACTGTGGAGCGCTTG

Primer-down: CGGGTGAAGGCTGCTTGA

Protein name: Invertase 2

Spot No.: I-50

Accession No.: tr|E9KF95|E9KF95_HEVBR

Primer sequences:

Primer-up: CACCAGAGGCGGTCTTGATTACT

Primer-down: TCAGAAAAGCCAGGGCACTAGG

Protein name: (PDBP) Pyridoxal biosynthesis protein PDX1

Spot No.: I-150

Accession No.: sp|Q39963|PDX1_HEVBR

Primer sequences:

Primer-up: AGTCCGTTACCATCCCCGTCA

Primer-down: CATTCCCTGTTCCTGCTTCACC

Protein name: (PHOT)Phosphoglyceride transfer family protein

Spot No.: I-175

Accession No.: tr|Q6J6I5|Q6J6I5_HEVBR

Primer sequences:

Primer-up: GATTTGATGAGAAGCCAACCCAC

Primer-down: CATATTAGCCTGGTTGCTTTAATCG

Protein name: (PROI) Protease inhibitor protein

Spot No.: I-366

Accession No.: tr|B3FNP9|B3FNP9_HEVBR

Primer sequences:

Primer-up: TCGGGACAAACGGGGACAT

Primer-down: GGTAATAAATTCGTTGTTTAGCCAATG

Protein name: (PPCI) Peptidyl-prolyl cis-trans isomerase

Spot No.: I-33

Accession No.: tr|B3FNQ1|B3FNQ1_HEVBR

Primer sequences:

Primer-up: GCGGATACAACGCCACGAAC

Primer-down: CCCTCCACAACTCTACCAAACACC

Protein name: Profilin-1

Spot No.: I-567

Accession No.: sp|O65812|PROF1_HEVBR

Primer sequences:

Primer-up: CTGCCATTATGAGTGACTTTGACG

Primer-down: TGTCTTCCTAACAGTAACACCACCAG

Protein name: prohevein

Spot No.: D-10

Accession No.: gi|2832430

Primer sequences:

Primer-up: GAAGGTGTTGGTGGTGGAAGTG

Primer-down: TGATGATTTCATAACGGAGAATAGAGG

Protein name: Pseudo-hevein

Spot No.: D-140

Accession No.: tr|Q6KF83|Q6KF83_HEVBR

Primer sequences:

Primer-up: GATGGGATTTGAACGCTGTAAGTG

Primer-down: TGGAGAATAGAGGGTTGAAGGAATC

Protein name: Rab4

Spot No.: I-227

Accession No.: tr|F2VXU6|F2VXU6_HEVBR

Primer sequences:

Primer-up: CTTCCTCTTGCCTGTTGCCTCT

Primer-down: GCAGCTTAATTGTCTTGCCATCC

Protein name: (REF-1) rubber elongation factor

Spot No.: D-85

Accession No.: gi|38122474

Primer sequences:

Primer-up: CAAGGACAAGTCTGGTCCATTC

Primer-down: TATGAACATCCAAAACACACGAC

Protein name: Rubber elongation factor

Spot No.: D-139

Accession No.: gi|132270

Primer sequences:

Primer-up: CAAAGACAAATCTGGTCCACTG

Primer-down: AAACATAGTCTCAACACTCAGGATG

Protein name: (SRPP-1) small rubber particle protein

Spot No.: D-99

Accession No.: gi|29170601

Primer sequences:

Primer-up: CCGTTGAAGCCTGGGGTC

Primer-down: CCACATCCAAAACACACCACC

Protein name: Small rubber particle protein

Spot No.: I-66

Accession No.: tr|Q84T87|Q84T87_HEVBR

Primer sequences:

Primer-up: CGTGTTCCGCCAGTTGTCAAG

Primer-down: GTAGGCACCAAAGGCAAATAAGAG

Protein name: (SRPP-3) Small rubber particle protein

Spot No.: D-52

Accession No.: gi|14423933

Primer sequences:

Primer-up: TGAAGAGGTGGAGGAAGAGAGG

Primer-down: CCACATCCAAAACACACCACC

Protein name: (TMAP) Translation machinery-associated protein

Spot No.: I-643

Accession No.: tr|D9IZZ6|D9IZZ6_HEVBR

Primer sequences:

Primer-up: AGCAAGGTGGAAAAGCAAAGC

Primer-down: CTTGAGCCCAGAACCTCCGA

Protein name: Ubiquitin-protein ligase 2a

Spot No.: I-943

Accession No.: tr|F8RWT0|F8RWT0_HEVBR

Primer sequences:

Primer-up: AGACTGATGAGGGATTTTAGGAGGT

Primer-down: TGACTGGATTGATGTGAGTATGGC

Protein name: (VHA-F) V-type proton ATPase subunit F

Spot No.: I-687

Accession No.: tr|B8Y6I1|B8Y6I1_HEVBR

Primer sequences:

Primer-up: TGCTGATGAGGACTCAGTAGTTGGA

Primer-down: CCTGTGCAGGATCATAAGGATGG

Protein name: (50KP) 50 kDa protein; major latex allergen Hev b 4

Spot No.: D-2

Accession No.: gi|37719658

Primer sequences:

Primer-up: GGAAGATTTTCTGATGGTCGCAC

Primer-down: CACGATATGTCGAATTGCCACAG

Protein name: (VHAE) V-type proton ATPase subunit E

Spot No.: D-58

Accession No.: gi|356528948

Primer sequences:

Primer-up: ATGGTCAGATTCATCCGTCAGG

Primer-down: TGTGGTTAATGCCATGAAAGAGG

Protein name: calmodulin

Spot No.: D-105

Accession No.: gi|354318

Primer sequences:

Primer-up: TCACTGGGACAGAATCCTACCG

Primer-down: CTTCTCGCCAAGGTTTGTCATC

Protein name: (CDCP) cell division cycle protein

Spot No.: D-130

Accession No.: gi|351727028

Primer sequences:

Primer-up: GGTGGTGTTAGAAAACAGATGGC

Primer-down: CCAATGACAATAACATGAGCACG

The abbreviations for the proteins were:

ATPS, ATP synthase subunit beta; ACAT, Acetyl-CoA C-acetyltransferase; REF, rubber elongation factor; SRPP, small rubber particle protein; VHAF, V-type proton ATPase subunit; ACPX, Ascorbate peroxidase; GGCS, Gamma-glutamylcysteine synthetase; VHAE, V-type proton ATPase subunit E; GLUS, glutamine synthetase; GSTF, glutathione-S-transferase;HSP70, heat shock protein 70; PDBP, Pyridoxal biosynthesis protein; CAMT, caffeic acid 3-O-methyltransferase;CYSP,Cysteine protease; CIPT, Cis-prenyl

transferase; ETIF, eukaryotic translation initiation factor; ETEF, eukaryotic translation elongation factor; ELRP, Elicitor-responsive protein; LAC, latex cystatin; TMAP, Translation machinery-associated protein; COPT, Copper transport protein; 50KP, 50 kDa protein; Gluc, beta-1,3-glucanase; CDCP, cell division cycle protein; ABAS, Abscisic acid stress ripening-related protein; ADPF, actin-depolymerizing factor; PROI, Protease inhibitor protein; HMGR, Hydroxymethylglutaryl coenzyme A reductase; HMGS, Hydroxymethylglutaryl coenzyme A synthase; PPCI, Peptidyl-prolyl cis-trans isomerase; MEVD, Mevalonate disphosphate decarboxylase; MEVK, Mevalonate kinase; ProH, prohevein; PseuH, Pseudo-hevein; FADS, Farnesyl diphosphate synthase; GLUD, Glutaredoxin; LAP, latex abundant protein; CIAD, Cinnamyl alcohol dehydrogenase; CIBP, Citrate-binding protein; SOD, Superoxide dismutase; NUDK, nucleoside diphosphate kinase; PHOT, Phosphoglyceride transfer family protei; SUCT, sucrose transporter; PHOK, phosphomevalonate kinase; HYDL, hydroxynitrile lyase; CDPK, calcium-dependent protein kinase; ETHI, ethylene-inducible protein; GLUR, glutathione reductase; LACG, latex cyanogenic beta glucosidase; CYAS, beta-cyanoalanine synthase; GGDS, geranylgeranyl diphosphate synthase.

Supplementary Table S1 MS identification of high abundant rubber latex proteins 2-DE gels

Spot No.[a]	Vol%[b]	Exper.[c] *p*I/M*r*	Thero.[d] *p*I/M*r*	M.S.[e] Score	NCBI No.[f]	Plant species	Protein name
**1**	4.47	4.96/14	5.04/14.7	122	132270	*H. brasiliensis*	Rubber elongation factor protein
**2**	4.18	4.92/13	5.04/14.7	142	132270	*H. brasiliensis*	Rubber elongation factor protein
**3**	3.09	4.88/13	5.04/14.7	95	132270	*H. brasiliensis*	Rubber elongation factor protein
**4**	2.69	4.8/22	4.8/22.3	119	14423933	*H. brasiliensis*	Small rubber particle protein
**5**	1.12	4.9/23	4.8/22.3	122	14423933	*H. brasiliensis*	Small rubber particle protein
**6**	1.24	4.82/23	4.8/22.3	135	14423933	*H. brasiliensis*	Small rubber particle protein
**7**	0.0296	4.72/14	4.61/12.8	[121]	29170601	*H. brasiliensis*	small rubber particle protein
**8**	0.034	4.74/14	4.61/12.8	[110]	29170601	*H. brasiliensis*	small rubber particle protein
**9**	0.0969	4.88/26	4.79/29.5	93	388503664	*L. japonicus*	unknown
**10**	0.434	4.98/40	5.0/43.0	209	3288200	*H. brasiliensis*	latex allergen
**11**	0.834	5.01/40	5.0.43.1	172	6707018	*H. brasiliensis*	latex protein allergen Hev b 7
**12**	0.214	5.07/49	5.01/46.4	82	4235430	*H. brasiliensis*	latex-abundant protein
**13**	0.135	5.05/49	5.01/46.6	96	4235430	*H. brasiliensis*	latex-abundant protein
**14**	0.335	5.09/54	5.07/71.1	103	20559	*P. hybrida*	hsp70 (AA 6 - 651)
**15**	0.513	5.16/40	5.31/42.0	142	58013197	*I. tinctoria*	actin
**16**	0.0655	5.21/52	5.2/69.1	174	225429795	*V. vinifera*	V-type proton ATPase, subunit A
**17**	0.137	5.29/47	7.56/59.9	92	357480067	*M. truncatula*	Leucine aminopeptidase
**18**	0.139	5.31/44	5.91/30.8	81	219885949	*Z. mays*	unknown
**19**	0.309	5.28/33	5.93/34.3	[101]	242059605	*S. bicolor*	hypothetical protein
**20**	0.145	5.18/15	8.01/11.2	[86]	30575570	*C. paradisi*	HSP19 class II, partial
**21**	0.458	5.33/16	5.73/15.7	83	27449246	*H. brasiliensis*	Cu/Zn superoxide dismutase
**22**	0.656	5.61/15	8.29/35.1	[60]	37806305	*O. sativa*	hypothetical protein
**23**	0.0912	5.55/33	9.46/35.3	85	10946499	*H. brasiliensis*	beta-1,3-glucanase
**24**	0.363	5.62/36	6.01/36.0	82	78216493	*M. domestica*	cytosolic malate dehydrogenase
**25**	0.567	5.77/32	6.41/36.0	[81]	257219562	*J. curcas*	annexin-like protein
**26**	0.852	5.69/27	8.72/34.1	[56]	3451147	*H. brasiliensis*	chitinase
**27**	0.442	5.75/30	9.45/38.9	97	4469175	*H. brasiliensis*	beta-1,3-glucanase
**28**	0.652	6.89/33	9.4/41.5	99	124365253	*H. brasiliensis*	beta-1,3-glucanase
**29**	0.803	6.85/34	9.4/41.5	109	124365253	*H. brasiliensis*	beta-1,3-glucanase
**30**	0.728	6.75/33	7.1/36.9	86	255540341	*R. communis*	glyceraldehyde 3-phosphate dehydrogenase
**31**	0.447	6.87/35	7.7/36.8	121	4539543	*N. tabacum*	glyceraldehyde-3-phosphate dehydrogenase
**32**	0.139	5.6/45	5.68/51.9	99	32527831	*P. tremuloides*	UDP-glucose pyrophosphorylase
**33**	0.104	5.55/39	5.0/43.1	171	6707018	*H. brasiliensis*	latex protein allergen Hev b 7
**34**	0.111	5.49/48	5.49/48.0	123	158144895	*G. hirsutum*	enolase
**35**	0.131	4.95/40	5.0/43.0	[81]	3288200	*H. brasiliensis*	latex allergen
**36**	0.0215	4.91/39	5.0/43.0	[138]	1916805	*H. brasiliensis*	latex patatin homolog
**37**	0.122	5.03/40	5.12/43.0	152	41581137	*H. brasiliensis*	latex allergen hev b 7.02
**38**	0.0897	5.06/40	5.12/43.0	142	41581137	*H. brasiliensis*	latex allergen hev b 7.02
**39**	0.0134	5.11/72	5.13/90.4	136	255556934	*R. communis*	Transitional endoplasmic reticulum ATPase
**40**	0.183	5.16/42	5.31/41.9	125	149938964	*A. deliciosa*	ACT1
**41**	0.0147	5.39/46	9.34/41.4	83	159489771	*C. reinhardtii*	predicted protein

**42**	0.0412	5.46/44	5.57/48.0	97	14423688	*H. brasiliensis*	Enolase 1
**43**	0.0132	5.54/62	5.62/111	90	23503558	*L. japonicus*	phosphoenolpyruvate carboxylase
**44**	0.0841	5.51/38	9.61/62.6	82	382929047	*H. hirsuta*	maturase K (chloroplast)
**45**	0.142	5.52/34	9.45/38.9	156	4469175	*H. brasiliensis*	beta-1,3-glucanase
**46**	0.045	5.65/33	9.45/38.9	126	4469175	*H. brasiliensis*	beta-1,3-glucanase
**47**	0.0134	5.65/27	6.18/35.9	[65]	225438145	*V. vinifera*	malate dehydrogenase, cytoplasmic
**48**	0.177	5.63/28	6.18/35.9	[85]	225438145	*V. vinifera*	malate dehydrogenase, cytoplasmic
**49**	0.0563	5.2/33	6.5/32.1	[75]	255552291	*R. communis*	short chain dehydrogenase, putative
**50**	0.057	4.75/15	4.61/12.8	[240]	29170601	*H. brasiliensis*	small rubber particle protein
**51**	0.638	5.05/13	5.04/14.7	103	132270	*H. brasiliensis*	Rubber elongation factor protein
**52**	0.339	5.15/13	5.04/14.7	115	132270	*H. brasiliensis*	Rubber elongation factor protein
**53**	0.0984	5.28/17	5.57/17.5	[61]	115187464	*A. hypogaea*	thioredoxin fold
**54**	0.0297	5.64/16	6.22/34.3	84	34538473	*H. vulgare*	caleosin 1
**55**	0.201	5.72/15	9.57/19.2	88	225440278	*V. vinifera*	hypothetical protein
**56**	0.0897	5.07/54	5.81/39.5	83	2213877	*H. brasiliensis*	glutamine synthetase
**57**	0.0301	5.1/54	5.21/71.5	102	172050753	*C. spinosa*	70 kDa heat shock protein
**58**	0.0356	5.12/54	5.18/71.9	[151]	762844	*S. lycopersicum*	Hsc70
**59**	0.0783	5.0/57	4.98/80.4	107	356552478	*G. max*	heat shock cognate protein 80-like
**60**	0.0132	5.01/56	5.07/52.1	[51]	356510919	*G. max*	UDP-glycosyltransferase 75D1-like
**61**	0.0163	5.03/49	5.49/47.1	144	255560725	*R. communis*	dead box ATP-dependent RNA helicase
**62**	0.0271	5.06/46	5.01/46.4	[142]	4235430	*H. brasiliensis*	latex-abundant protein
**63**	0.0401	4.91/46	4.65/41.4	[76]	255578278	*R. communis*	hypothetical protein
**64**	0.0176	5.14/46	5.15/47.1	[98]	5302811	*A. thaliana*	serine protease-like protein
**65**	0.0171	5.13/51	5.85/63.3	102	2506277	*P. sativum*	60 kDa chaperonin subunit beta
**66**	0.0193	5.16/54	5.27/72.0	88	255543357	*R. communis*	heat shock protein, putative
**67**	0.025	5.18/48	4.54/46.1	[91]	224100473	*P. trichocarpa*	predicted protein
**68**	0.0265	5.21/48	5.72/56.3	84	31580730	*H. brasiliensis*	latex cyanogenic beta glucosidase
**69**	0.0379	5.27/44	5.44/50.1	83	2501850	*N. tabacum*	GDP dissociation inhibitor
**70**	0.0152	5.3/44	5.44/50.1	[202]	2501850	*N. tabacum*	GDP dissociation inhibitor
**71**	0.549	5.21/43	5.42/47.2	147	359478860	*V. vinifera*	26S protease regulatory subunit 6B
**72**	0.126	5.13/42	5.28/47.1	[128]	224071449	*P. trichocarpa*	phospholipase A
**73**	0.0841	5.12/40	5.37/41.9	178	32186912	*G. hirsutum*	actin
**74**	0.0304	4.98/34	4.79/48.0	[70]	297816956	*A. lyrata*	hypothetical protein
**75**	0.0404	4.9/32	4.72/28.9	[74]	3264769	*P. armeniaca*	late embryogenesis-like protein
**76**	0.0574	4.93/32	5.24/42.0	81	168066110	*P. patens*	predicted protein
**77**	0.0683	4.89/30	4.79/29.5	109	388503664	*L. japonicus*	unknown
**78**	0.0174	5.0/35	4.84/24.3	[71]	115468882	*O. sativa*	Os06g0608500
**79**	0.0627	5.06/33	5.51/61.1	[104]	356532109	*G. max*	phosphoglycerate mutase
**80**	0.0124	5.05/31	5.11/59.2	90	224056489	*P. trichocarpa*	predicted protein
**81**	0.185	5.14/34	6.5/32.1	[174]	255552291	*R. communis*	short chain dehydrogenase, putative
**82**	0.133	5.29/38	5.49/39.3	91	2073448	*L. japonicus*	glutamine synthetase
**83**	0.0563	5.1/31	6.07/27.5	[204]	192912956	*E. guineensis*	aluminum-induced protein
**84**	0.0517	5.06/28	5.12/32.9	[254]	255559601	*R. communis*	alpha-soluble nsf attachment protein
**85**	0.0808	4.98/27	5.84/27.4	[69]	224099853	*P. trichocarpa*	predicted protein

**86**	0.438	5.17/21	5.06/19.6	[410]	38122474	*H. brasiliensis*	rubber elongation factor
**87**	0.0478	5.21/22	5.06/19.6	[226]	38122474	*H. brasiliensis*	rubber elongation factor
**88**	0.0734	5.13/21	5.06/19.6	116	38122474	*H. brasiliensis*	rubber elongation factor
**89**	0.0575	5.14/23	5.06/19.6	99	38122474	*H. brasiliensis*	rubber elongation factor
**90**	1.88	4.62/19	4.06/15.2	[496]	334854634	*H. brasiliensis*	elicitor-responsive protein
**91**	0.156	5.35/17	5.6/17.7	[95]	33325129	*H. brasiliensis*	eukaryotic translation initiation factor 5A
**92**	0.0403	5.37/16	5.39/18.0	[180]	224144943	*P. trichocarpa*	predicted protein
**93**	0.299	6.68/13	6.85/16.3	84	312985291	*H. brasiliensis*	nucleoside diphosphate kinase 1
**94**	0.376	6.74/16	5.63/22.7	96	123062	*H. brasiliensis*	Pro-hevein
**95**	0.274	6.61/15	7.77/20.7	[70]	313586569	*H. brasiliensis*	hypothetical protein 19
**96**	0.0897	6.65/14	7.77/20.7	94	313586569	*H. brasiliensis*	hypothetical protein 19
**97**	0.414	6.78/16	8.94/40.7	84	4835766	*A. thaliana*	transfactor (WERBP-1)
**98**	0.0367	5.42/22	5.06/19.6	99	38122474	*H. brasiliensis*	rubber elongation factor
**99**	0.107	5.34/24	5.71/26.1	[73]	3377751	*M. crystallinum*	glutathione S-transferase
**100**	0.0804	5.35/26	6.17/27.5	[49]	1709761	*S. oleracea*	Proteasome subunit alpha type-4
**101**	0.0483	5.47/26	5.78/27.5	[434]	27449248	*H. brasiliensis*	ascorbate peroxidase
**102**	0.142	5.48/26	6.0/27.5	[193]	211906460	*G. hirsutum*	triosephosphate isomerase
**103**	0.0357	5.22/21	5.06/19.6	125	38122474	*H. brasiliensis*	rubber elongation factor
**104**	0.345	5.75/19	8.72/34.1	153	3451147	*H. brasiliensis*	chitinase
**105**	0.15	5.75/23	8.44/29.9	93	157831407	*H. brasiliensis*	Chain A, Crystal Structures Of Hevamine
**106**	0.231	5.74/24	8.44/29.9	99	157831407	*H. brasiliensis*	Chain A, Crystal Structures Of Hevamine
**107**	0.148	5.77/20	8.72/34.1	83	3451147	*H. brasiliensis*	chitinase
**108**	0.0327	5.52/33	5.89/27.9	[89]	3127890	*C. arietinum*	cysteine synthase, O-acetyl-lyase
**109**	0.0761	5.39/37	5.44/40.0	[163]	114199044	*M. domestica*	caffeic acid O-methyltransferase
**110**	0.0405	5.38/44	5.57/48.0	158	14423688	*H. brasiliensis*	Enolase 1
**111**	0.0239	5.39/44	5.57/48.0	175	14423688	*H. brasiliensis*	Enolase 1
**112**	0.0339	5.38/44	6.05/25.1	85	226897734	*T. flavum*	norcoclaurine 6-O-methyltransferase
**113**	0.0262	5.38/49	5.53/54.1	[49]	255565071	*R. communis*	conserved hypothetical protein
**114**	0.0502	5.47/51	5.52/61.0	96	1346735	*R. communis*	Phosphoglyceromutase
**115**	0.0152	5.51/61	5.87/95.0	141	255565836	*R. communis*	eukaryotic translation elongation factor
**116**	0.0926	5.02/39	5.0/43.1	147	6707018	*H. brasiliensis*	latex protein allergen Hev b 7
**117**	0.0363	5.05/39	5.0/43.9	[59]	51315784	*H. brasiliensis*	Esterase
**118**	0.0152	5.29/21	5.06/19.6	98	38122474	*H. brasiliensis*	rubber elongation factor
**119**	0.0968	5.34/46	5.41/47.8	[51]	3023685	*A. glutinosa*	Enolase
**120**	0.0518	5.28/41	4.83/22.0	81	351723039	*G. max*	uncharacterized protein
**121**	0.0992	5.02/27	7.63/31.6	[55]	255554865	*R. communis*	lactoylglutathione lyase, putative
**122**	0.139	5.35/26	5.59/18.4	[83]	118485535	*P. trichocarpa*	unknown
**123**	0.152	5.51/26	5.93/27.6	86	15225839	*A. thaliana*	proteasome subunit alpha type-3
**124**	0.179	5.49/23	6.06/23.0	81	10862818	*H. brasiliensis*	IgE-binding protein Mn SOD
**125**	0.153	5.17/24	7.7/25.7	[53]	1737447	*E. globulus*	auxin-induced protein
**126**	0.104	4.45/15	4.61/12.8	[97]	29170601	*H. brasiliensis*	small rubber particle protein
**127**	0.119	5.46/13	5.64/20.9	[84]	2832430	*H. brasiliensis*	prohevein
**128**	0.065	5.55/14	6.82/15.0	[68]	224054224	*P. trichocarpa*	predicted protein
**129**	0.178	5.31/12	5.89/13.3	[61]	329750601	*H. brasiliensis*	thioredoxin H-type 2

**130**	0.532	5.01/11	5.45/11.2	81	268321205	*H. brasiliensis*	latex cystatin
**131**	1.01	5.02/13	5.04/14.7	[539]	132270	*H. brasiliensis*	Rubber elongation factor protein
**132**	0.105	5.45/13	5.04/14.7	138	132270	*H. brasiliensis*	Rubber elongation factor protein
**133**	0.117	5.16/15	6.4/8.6	81	242083244	*S. bicolor*	hypothetical protein
**134**	0.0976	4.94/13	4.61/12.8	[85]	29170601	*H. brasiliensis*	small rubber particle protein
**135**	0.0865	4.86/15	4.61/12.8	[493]	29170601	*H. brasiliensis*	small rubber particle protein
**136**	0.448	4.13/13	4.1/16.9	[128]	354318	*T. aestivum*	calmodulin
**137**	0.0284	4.12/21	4.8/22.3	85	14423933	*H. brasiliensis*	Small rubber particle protein
**138**	0.0653	4.45/24	4.8/22.3	165	14423933	*H. brasiliensis*	Small rubber particle protein
**139**	0.0351	5.36/47	5.57/48.0	107	14423688	*H. brasiliensis*	Enolase 1
**140**	0.149	4.87/19	5.04/14.7	[52]	132270	*H. brasiliensis*	Rubber elongation factor protein
**141**	0.0517	5.38/45	5.57/48.0	165	14423688	*H. brasiliensis*	Enolase 1
**142**	0.083	5.43/44	5.92/48.1	[766]	14423687	*H. brasiliensis*	Enolase 2
**143**	0.115	5.01/26	5.48/25.6	[52]	333690873	*H. brasiliensis*	glutathione-S-transferase tau 1
**144**	0.0957	5.33/53	8.78/43.5	82	242080619	*S. bicolor*	hypothetical protein
**145**	0.101	5.09/15	5.95/17.6	[87]	140083605	*R. hybrid*	cytosolic class II small HSP17.5
**146**	0.0217	4.02/41	5.01/14.6	[88]	37901124	*H. brasiliensis*	latex abundant protein 1
**147**	0.0256	4.35/40	4.53/38.0	[173]	359806300	*G. max*	uncharacterized protein
**148**	0.0301	4.28/38	4.53/38.1	[166]	37625031	*V. aestivalis*	ankyrin-repeat protein
**149**	0.0356	4.39/38	4.45/37.5	[71]	13310811	*N. tabacum*	ankyrin-repeat protein HBP1
**150**	0.518	5.18/42	5.64/42.2	[180]	129916	*T. aestivum*	Phosphoglycerate kinase
**151**	0.0258	4.31/36	4.5/25.3	[117]	378464888	*A. mongolicus*	translation elongation factor
**152**	0.0335	4.43/32	4.5/25.1	[107]	225456295	*V. vinifera*	elongation factor 1-delta-like
**153**	0.0201	4.83/30	5.26/35.7	[132]	22261807	*S. tuberosum*	Light-induced protein
**154**	0.0125	4.89/31	4.82/28.9	83	345103803	*G. thurberi*	14-3-3-like protein, partial
**155**	0.0549	5.08/26	4.76/29.9	[384]	326694865	*H. brasiliensis*	14-3-3 protein 2
**156**	0.0563	4.78/33	5.02/30.9	[53]	225458231	*V. vinifera*	proteasome subunit alpha
**157**	0.0338	4.89/31	5.69/32.6	[80]	211906514	*G. hirsutum*	lactoylglutathione lyase
**158**	0.186	5.02/33	5.5/33.2	80	359483362	*V. vinifera*	lactoylglutathione lyase
**159**	0.0491	5.12/37	5.08/53.7	80	340033945	*C. maritimum*	ATP synthase CF1 beta subunit
**160**	0.0565	5.15/36	5.41/49.2	84	4388534	*S. bicolor*	F1-ATP synthase, beta subunit
**161**	0.0209	5.13/30	5.64/27.4	[136]	147805616	*V. vinifera*	hypothetical protein
**162**	0.0607	5.06/36	8.89/54.7	83	326492265	*Ho. Vulgare*	predicted protein
**163**	0.06679	4.89/46	4.99/54.4	[343]	225459744	*V. vinifera*	V-type proton ATPase subunit B
**164**	0.0157	5.08/46	5.04/54.4	[377]	225428086	*V. vinifera*	V-type proton ATPase subunit B 1
**165**	0.116	4.78/42	5.28/47.1	[59]	224071449	*P. trichocarpa*	phospholipase A
**166**	0.0203	4.81/16	5.91/13.4	81	116789736	*P. sitchensis*	unknown
**167**	0.0414	5.35/17	5.98/51.0	[51]	115392253	*C. betuloides*	polyphenol oxidase
**168**	0.0691	5.36/19	5.39/18.0	[210]	225430941	*V. vinifera*	regulator of ribonuclease
**169**	0.179	5.23/16	5.64/20.9	[152]	2832430	*H. brasiliensis*	prohevein
**170**	0.14	5.18/15	5.92/16.2	[110]	298362843	*H. brasiliensis*	actin-depolymerizing factor
**171**	0.477	4.81/19	9.23/58.4	[104]	255539441	*R. communis*	actin depolymerizing factor
**172**	0.125	5.18/18	5.64/20.9	[252]	2832430	*H. brasiliensis*	prohevein
**173**	0.0849	5.12/23	5.17/24.9	101	255574159	*R. communis*	proteasome subunit beta type

**174**	0.0317	4.89/31	5.93/34.3	[117]	242059605	*S. bicolor*	hypothetical protein
**175**	0.0295	5.22/27	6.44/66.4	86	2832654	*A. thaliana*	chloroplast protein cs/ch-42
**176**	0.0419	5.38/26	5.78/27.5	[487]	27449248	*H. brasiliensis*	ascorbate peroxidase
**177**	0.0415	5.32/24	6.06/22.6	[369]	357480975	*M. truncatula*	Proteasome subunit beta type
**178**	0.054	5.35/25	6.08/24.9	[302]	255541320	*R. communis*	proteasome subunit beta type
**179**	0.19	6.98/15	6.56/9.7	82	350537255	*S. lycopersicum*	small zinc finger-like protein
**180**	0.0431	5.56/27	8.99/18.9	107	37622210	*H. brasiliensis*	small rubber particle protein
**181**	0.0442	5.87/38	5.8/61.3	81	168038495	*P. patens*	predicted protein
**182**	0.18	6.35/33	6.04/61.4	82	164472662	*T. aestivum*	calcium-dependent protein kinase
**183**	0.145	5.86/45	5.68/51.9	92	32527831	*P. tremula*	UDP-glucose pyrophosphorylase
**184**	0.0292	5.91/45	6.04/51.8	92	356576773	*G. max*	seryl-tRNA synthetase-like
**185**	0.0442	5.48/34	8.64/36.3	[51]	357514973	*M. truncatula*	Annexin D4
**186**	0.0553	5.35/38	6.48/42.1	[63]	1351887	*M. domestica*	Alcohol dehydrogenase
**187**	0.0362	5.51/37	5.54/41.0	[123]	380468126	*H. brasiliensis*	caffeic acid 3-O-methyltransferase
**188**	0.0275	5.28/36	5.54/41.0	[117]	380468126	*H. brasiliensis*	caffeic acid 3-O-methyltransferase
**189**	0.069	5.11/26	5.41/27.5	[159]	284433794	*J. curcas*	glutathione S-transferase omega
**190**	0.0311	5.56/28	6.09/30.8	[52]	255567721	*R. communis*	carboxymethylenebutenolidase
**191**	0.0405	5.48/29	10.36/29.7	[59]	125573095	*O. sativa*	hypothetical protein OsJ_04535
**192**	0.0954	5.44/33	5.5/34.4	82	255542380	*R. communis*	cysteine synthase, putative
**193**	0.0329	5.23/32	5.56/32.4	[57]	255562276	*R. communis*	pyridoxine kinase, putative
**194**	0.0813	5.85/33	6.5/32.1	[303]	255552291	*R. communis*	short chain dehydrogenase
**195**	0.0796	5.17/54	5.27/72.0	102	255543357	*R. communis*	heat shock protein, putative
**196**	0.0398	5.07/53	7.9/68.9	82	224139936	*P. trichocarpa*	predicted protein
**197**	0.0121	5.09/72	5.18/90.5	174	351727028	*G. max*	cell division cycle protein 48 homolog
**198**	0.0331	5.25/48	5.01/46.4	180	4235430	H. brasiliensis	latex-abundant protein
**199**	0.0648	5.51/47	4.96/48.7	[57]	198400319	*Camellia sinensis*	senescence-related protein
**200**	0.0702	5.15/40	5.6/42.4	[282]	255570457	*R. communis*	protease C56, putative
**201**	0.0708	5.46/41	5.44/41.9	149	32186896	*G. hirsutum*	actin
**202**	0.0756	5.41/42	5.49/47.1	109	255560725	*R. communis*	dead box ATP-dependent RNA helicase
**203**	0.0199	5.35/48	5.57/48.0	[229]	14423688	*H. brasiliensis*	Enolase 1
**204**	0.0168	5.38/55	5.27/72.0	[311]	186898205	*H. brasiliensis*	heat-shock protein 70
**205**	0.0392	5.68/58	5.6/65.4	[63]	255537027	*R. communis*	HSP70-interacting protein
**206**	0.0262	6.32/51	8.96/65.0	81	302853426	*V. carteri*	hypothetical protein
**207**	0.0208	6.14/67	5.6/65.4	[266]	255537027	*R. communis*	HSP70-interacting protein
**208**	0.0133	6.35/67	5.6/65.4	[68]	255537027	*R. communis*	HSP70-interacting protein
**209**	0.0489	5.36/57	5.52/61.0	[161]	1346735	*R. communis*	phosphoglycerate mutase
**210**	0.0132	5.38/68	5.6/65.4	[69]	255537027	*R. communis*	HSP70-interacting protein
**211**	0.0415	5.35/72	5.13/90.4	[1185]	255556934	*R. communis*	Transitional endoplasmic reticulum ATPase
**212**	0.0352	5.36/53	5.79/66.6	[166]	4588474	*B. napus*	phosphoinositide-specific phospholipase C
**213**	0.0419	5.38/55	5.79/66.6	[116]	4588474	*B. napus*	phosphoinositide-specific phospholipase C
**214**	0.0365	5.33/52	5.46/63.7	[138]	255568794	*R. communis*	nucleoredoxin, putative
**215**	0.0159	5.37/51	5.66/53.8	[289]	32967699	*A. thaliana*	S-adenosyl-L-homocystein hydrolase
**216**	0.0354	5.61/49	5.69/52.8	[327]	336390551	*G. max*	adenosylhomocysteinase

**217**	0.0327	5.78/48	5.2/51.8	[52]	6136111	*H. vulgare*	glucose-1-phosphate uridylyltransferase
**218**	0.0876	6.28/38	6.86/51.8	[802]	14916100	*H. brasiliensis*	hydroxymethylglutaryl Co-A synthase
**219**	0.0746	5.65/34	5.92/34.8	[181]	307707110	*P. armeniaca*	NAD-dependent malate dehydrogenase
**220**	0.091	5.54/38	5.64/42.2	[357]	357137038	*B. distachyon*	phosphoglycerate kinase, cytosolic-like
**221**	0.031	5.25/34	8.64/36.3	[99]	357514973	*M. truncatula*	Annexin D4
**222**	0.0629	5.41/37	5.49/42.2	[314]	21536853	*A. thaliana*	phosphoglycerate kinase, putative
**223**	0.0416	5.68/39	5.82/41.6	[77]	255547137	*R. communis*	Alpha-1,4-glucan-protein synthase
**224**	0.0396	5.29/37	5.54/41.0	[922]	380468126	*H. brasiliensis*	caffeic acid 3-O-methyltransferase
**225**	0.0375	5.25/39	5.6/42.4	[237]	255570457	*R. communis*	protease C56, putative
**226**	0.0626	5.13/27	5.24/26.2	[172]	224131618	*P. trichocarpa*	predicted protein
**227**	0.166	5.31/25	5.48/25.6	[386]	333690873	*H. brasiliensis*	glutathione-S-transferase tau 1
**228**	0.0928	5.38/26	6.0/27.5	[243]	211906460	*G. hirsutum*	triosephosphate isomerase
**229**	0.0256	5.21/27	5.13/27.5	[93]	255568460	*R. communis*	glutathione-s-transferase omega
**230**	0.0459	5.15/25	5.62/25.8	[104]	2970051	*V. radiata*	ARG10
**231**	0.0957	5.23/21	5.64/20.9	[217]	2832430	*H. brasiliensis*	prohevein
**232**	0.136	5.35/20	5.64/20.9	[112]	2832430	*H. brasiliensis*	prohevein
**233**	0.0722	4.98/23	4.8/22.3	[384]	14423933	*H. brasiliensis*	Small rubber particle protein
**234**	0.185	5.01/15	5.04/14.7	[379]	132270	*H. brasiliensis*	Rubber elongation factor protein
**235**	0.0425	5.56/15	6.32/17.1	[128]	123550	*P. sativum*	heat shock protein, 17.1 kDa class II
**236**	0.212	5.36/14	5.96/16.5	[272]	312985293	*H. brasiliensis*	nucleoside diphosphate kinase 2
**237**	0.234	5.67/13	5.04/14.7	[90]	132270	*H. brasiliensis*	Rubber elongation factor protein
**238**	1.121	5.54/13	5.64/20.9	[344]	2832430	*H. brasiliensis*	prohevein
**239**	0.0902	5.01/41	5.6/42.4	[193]	255570457	*R. communis*	protease C56, putative
**240**	0.0535	5.58/46	5.68/47.0	[58]	727357	*A. thaliana*	DnaJ homolog
**241**	0.0674	5.34/48	5.46/63.7	[100]	255568794	*R. communis*	nucleoredoxin, putative
**242**	1.13	4.75/51	4.6/41.5	93	37719658	*H. brasiliensis*	50 kDa protein
**243**	0.354	4.78/49	4.6/41.5	85	37719658	*H. brasiliensis*	50 kDa protein
**244**	1.05	4.55/50	4.6/41.5	95	37719658	*H. brasiliensis*	50 kDa protein
**245**	1.02	4.73/47	4.6/41.5	101	37719658	*H. brasiliensis*	50 kDa protein
**246**	0.121	4.77/47	4.6/41.5	118	37719658	*H. brasiliensis*	50 kDa protein
**247**	0.215	5.35/13	5.63/22.7	[158]	123062	*H. brasiliensis*	Pro-hevein
**248**	1.41	6.96/13	5.64.20.9	[446]	2832430	*H. brasiliensis*	prohevein
249	0.428	5.01/13	5.04/14.7	[405]	132270	*H. brasiliensis*	Rubber elongation factor protein
**250**	0.478	5.24/13	5.04/14.7	[340]	132270	*H. brasiliensis*	Rubber elongation factor protein
**251**	0.401	5.11/13	5.04/14.7	[470]	132270	*H. brasiliensis*	Rubber elongation factor protein
**252**	0.788	5.69/14	5.04/14.7	[402]	132270	*H. brasiliensis*	Rubber elongation factor protein
**B1**	---	---	5.04/14.7	[282]	132270	*H. brasiliensis*	Rubber elongation factor protein
**B2**	---	---	5.14/90.3	90	225440045	*V. vinifera*	cell division cycle protein 48 homolog
**B3**	---	---	5.54/92.3	[100]	88193697	*A. hypogaea*	phospholipase D
**B4**	---	---	5.17/71.4	102	225440324	*V. vinifera*	heat shock cognate 70 protein
**B5**	---	---	6.77/23.7	95	253760141	*S. bicolor*	hypothetical protein
**B6**	---	---	4.8/22.3	[348]	14423933	*H. brasiliensis*	Small rubber particle protein
**B7**	---	---	9.4/41.5	125	124365253	*H. brasiliensis*	beta-1,3-glucanase

**B8**	---	---	9.46/35.3	187	124365253	*H. brasiliensis*	beta-1,3-glucanase
**B9**	---	---	7.1/36.9	103	255540341	*R. communis*	glyceraldehyde 3-phosphate dehydrogenase
**B10**	---	---	4.8/22.3	160	14423933	*H. brasiliensis*	small rubber particle protein
**B11**	---	---	5.06/19.6	106	38122474	*H. brasiliensis*	rubber elongation factor
**B12**	---	---	5.0/43.0	87	3288200	*H. brasiliensis*	latex allergen
**B13**	---	---	5.04/14.9	[271]	334854628	*H. brasiliensis*	rubber elongation factor
**B14**	---	---	5.57/48.0	117	14423688	*H. brasiliensis*	Enolase 1
**B15**	---	---	5.27/72.0	85	255543357	*R. communis*	heat shock protein, putative
**B16**	---	---	5.12/91.0	88	225456951	*V. vinifera*	cell division cycle protein 48 homolog
**B17**	---	---	5.22/21.0	[67]	66735596	*A. hypogaea*	phospholipase D alpha
**B18**	---	---	5.02/80.4	90	33326375	*H. brasiliensis*	heat shock protein
**B19**	---	---	5.11/70.4	107	450880	*A. thaliana*	heat shock cognate 70-1
**B20**	---	---	7.01/28.4	81	379068234	*R. formosanum*	leucine-rich repeat protein
**B21**	---	---	5.57/48.0	101	14423688	*H. brasiliensis*	enolase 1
**B22**	---	---	5.0/43.0	121	3288200	*H. brasiliensis*	latex allergen
**B23**	---	---	5.64/42.2	[81]	113217	*Daucus carota*	Actin-1
**B24**	---	---	97.2/6.1	84	147835357	*V. vinifera*	hypothetical protein
**B25**	---	---	5.67/11.7	81	170525118	*P. mendocina*	geranylgeranyl reductase
**B26**	---	---	8.11/43.2	83	145346735	*O. lucimarinus*	predicted protein
**B27**	---	---	4.79/29.0	[101]	37903393	*S. hybrid*	14-3-3-like protein
**B28**	---	---	9.46/35.5	181	10946499	*H. brasiliensis*	beta-1,3-glucanase
**B29**	---	---	9.4/41.5	105	124365253	*H. brasiliensis*	beta-1,3-glucanase
**B30**	---	---	4.8/22.3	130	14423933	*H. brasiliensis*	small rubber particle protein
**B31**	---	---	7.77/20.7	132	313586569	*H. brasiliensis*	hypothetical protein 19
**B32**	---	---	6.56/8.5	162	327492451	*M. crystallinum*	N-terminal end of polyubiquitin
**B33**	---	---	5.73.15.7	87	27449246	*H. brasiliensis*	Cu/Zn superoxide dismutase SOD
**B34**	---	---	4.61/12.8	84	29170601	*H. brasiliensis*	small rubber particle protein
**B35**	---	---	5.01/10.3	[118]	242080683	*S. bicolor*	hypothetical protein

Note:

[a]Assigned spot (S1-S252) or band (B1-B35) number as indicated in Fig. 2.

[b]The statistical percent of volue value (Vol%) of the target protein spot in all the proteins in the 2-DE gels produced by ImageMaster.

[c, d]The experimental (c) and theoretical (d) values for the mass (kDa) and *p*I of identified proteins. The theoretical values were retrieved from the protein database, and the experimental values were produced by ImageMaster.

[e]Mascot searched scores (M.S.) against NCBInr for the MS and [MS/MS] searched results.

[f]Database accession (gi|) numbers according to NCBInr. ---, the values were not detected.

Supplementary Table S2 MS identification of the differential latex proteins in DIGE gels

Spot NO.[a]	Changed[b] 3 | 5-day	Exper.[c] *p*I/M*r*	Thero.[d] *p*I/M*r*	M.S.[e] Score	NCBI No.[f]	Plant species	Protein name
**D1**	-1.23/-1.85	5.19/30	4.71/28.6	[278]	255545912	*R. communis*	14-3-3 protein, putative
**D2**	-1.47/-1.97	4.75/51	4.6/41.5	93	37719658	*H. brasiliensis*	50 kDa protein
**D3**	-1.51/-2.09	4.78/49	4.6/41.5	85	37719658	*H. brasiliensis*	50 kDa protein
**D4**	-1.24/-4.02	5.87/62	5.64/27.4	[324]	147805616	*V. vinifera*	hypothetical protein VITISV_028800
**D5**	-1.24/-1.68	6.33/68	5.3/35.8	[122]	224137430	*P. trichocarpa*	predicted protein
**D6**	1.31/1.59	5.16/47	5.57/48.0	[229]	14423688	*H. brasiliensis*	Enolase 1
**D7**	-1.52/-2.74	4.55/50	4.6/41.5	95	37719658	*H. brasiliensis*	50 kDa protein
**D8**	1.11/1.54	5.45/61	5.87/95.0	123	255565836	*R. communis*	eukaryotic translation elongation factor
**D9**	1.52/1.34	4.93/41	5.047/54.4	146	225428086	*V. vinifera*	V-type proton ATPase subunit B 1
**D10**	-1.27/-1.57	6.42/16	5.65/20.9	[446]	2832430	*H. brasiliensis*	Pro-hevein
**D11**	-1.23/-1.54	6.61/15	5.63/22.7	[158]	123062	*H. brasiliensis*	Pro-hevein
**D12**	-1.52/1.31	5.87/71	5.42/45.9	[77]	15237159	*A. thaliana*	regulatory particle triple-A ATPase 3
**D13**	-1.53/1.19	6.0/21	5.63/22.7	[129]	123062	*H. brasiliensis*	Pro-hevein
**D14**	-1.09/1.56	5.94/54	5.75/35.6	[196]	225446010	*V. vinifera*	glucose-6-phosphate 1-epimerase
**D15**	1.51/-1.53	5.25/97	5.15/75.5	[338]	1143427	*C. sativus*	heat shock protein 70
**D16**	-1.43/-1.87	6.23/48	4.94/28.6	[198]	255558037	*R. communis*	nucleic acid binding protein, putative
**D17**	-1.27/-1.52	6.35/18	5.73/15.7	[105]	27449246	*H. brasiliensis*	Cu/Zn superoxide dismutase
**D18**	-1.07/-1.55	6.96/14	5.64/20.9	[446]	2832430	*H. brasiliensis*	pro-hevein
**D19**	-1.57/-1.53	6.6/99	6.08/59.6	[301]	255551408	*R. communis*	chaperonin containing t-complex protein 1
**D20**	-1.51/-1.82	6.02/55	6.18/35.6	[75]	225438145	*V. vinifera*	malate dehydrogenase, cytoplasmic
**D21**	1.75/-1.98	5.6/43	5.19/56.6	[116]	224056377	*R. communis*	Aspartic proteinase precursor, putative
**D22**	1.15/-1.55	5.59/54	7.63/31.6	[145]	255554865	*R. communis*	lactoylglutathione lyase, putative
**D23**	-1.95/1.55	6.23/64	5.83/27.7	[466]	356517215	*G. max*	uncharacterized protein LOC100817735
**D24**	1.52/1.14	5.55/98	5.1/71.2	[505]	356569000	*G. max*	heat shock cognate 70 kDa protein
**D25**	1.54/1.52	5.01/13	5.04/14.7	[405]	132270	*H. brasiliensis*	Rubber elongation factor protein
**D26**	1.51/-1.11	6.53/42	7.81/31.3	[79]	6624721	*P. sativum*	cysteine protease, putative
**D27**	1.55/-1.1	5.24/13	5.04/14.7	[340]	132270	*H. brasiliensis*	Rubber elongation factor protein
**D28**	3.01/1.06	5.96/87	5.47/46.2	[115]	314991144	*G. hirsutum*	mitogen-activated protein kinase
**D29**	-2.92/1.05	6.01/33	5.59/18.4	[82]	118485535	*P. trichocarpa*	unknown
**D30**	1.26/1.54	5.11/13	5.04/14.7	[470]	132270	*H. brasiliensis*	Rubber elongation factor protein
**D31**	1.58/-1.15	6.61/41	6.43/36.7	[80]	470127750	*F. vesca*	uncharacterized protein LOC101300446
**D32**	1.53/1.19	5.69/14	5.04/14.7	[398]	132270	*H. brasiliensis*	Rubber elongation factor protein
**D33**	1.67/1.21	6.37/102	5.31/47.9	[106]	351724891	*G. max*	enolase
**D34**	1.56/1.32	5.27/68	4.65/41.4	[154]	255578278	*R. communis*	hypothetical protein RCOM_0537780
**D35**	1.53/1.19	6.63/41	5.15/75.5	[378]	1143427	*C. sativus*	heat shock protein 70
**D36**	1.55/1.54	6.54/82	6.96/41.2	[120]	164604960	*H. brasiliensis*	acetyl-CoA C-acetyltransferase
**D37**	-1.58/-1.53	6.54/41	8.99/18.9	[77]	37622210	*H. brasiliensis*	small rubber particle protein
**D38**	-1.49/1.52	5.24/35	4.87/30.8	[109]	255555641	*R. communis*	26S proteasome atpase regulatory subunit
**D39**	-1.59/-2.53	6.32/31	6.08/24.9	[157]	255541320	*R. communis*	proteasome subunit beta type
**D40**	-1.55/-1.95	5.21/97	5.35/75.4	[459]	255570990	*R. communis*	heat shock protein, putative
**D41**	-1.26/-1.55	6.53/24	5.86/22.7	[97]	351726646	*G. max*	uncharacterized protein

**D42**	1.51/1.87	5.49/72	4.98/54.8	[111]	166627	*A. thaliana*	nucleotide-binding subunit of V-ATPase
**D43**	-1.51/-1.53	6.21/14	5.04/14.7	[55]	132270	*H. brasiliensis*	Rubber elongation factor protein
**D44**	1.53/-1.39	6.71/34	5.84/27.5	[366]	255583952	*R. communis*	proteasome subunit alpha type, putative
**D45**	-1.21/-1.58	5.97/28	5.48/26.3	[189]	356547978	*G. max*	eukaryotic translation initiation factor 3
**D46**	-1.17/-1.56	6.51/31	6.4/23.7	[78]	310772392	*M. glabra*	dehydroascorbate reductase
**D47**	1.63/-1.29	5.54/19	5.39/17.9	[370]	255547584	*R. communis*	Regulator of ribonuclease activity A
**D48**	1.24/-1.55	6.72/85	6.29/35.5	[128]	255568992	*R. communis*	conserved hypothetical protein
**D49**	-1.52/-1.54	6.24/91	5.78/54.6	[206]	356522666	*G. max*	ubiquitin carboxyl-terminal hydrolase 6
**D50**	1.54/-1.23	5.51/47	5.84/27.4	[120]	224099853	*P. trichocarpa*	predicted protein
**D51**	-1.12/1.58	6.13/90	5.46/63.7	[380]	255568794	*R. communis*	nucleoredoxin, putative
**D52**	1.53/1.58	5.51/31	4.8/22.3	[137]	14423933	*H. brasiliensis*	Small rubber particle protein
**D53**	-1.01/1.53	5.9/45	5.48/25.6	[132]	333690873	*H. brasiliensis*	glutathione-S-transferase tau 1
**D54**	-1.27/1.55	6.01/62	5.71/38.4	[105]	356543702	*G. max*	pulegone reductase-like
**D55**	1.21/1.52	6.4/20	5.82/18.5	[56]	351721881	*G. max*	heat shock protein,18.5 kDa class I
**D56**	-1.09/1.56	5.61/96	5.02/48.4	[96]	462406561	*P. persica*	hypothetical protein
**D57**	1.12/1.59	6.64/35	7.6/30.6	[56]	255570177	*R. communis*	proteasome subunit beta type
**D58**	1.13/-1.54	6.75/16	5.93/18.3	[197]	3834310	*A. thaliana*	Ubiquitin-conjugating enzyme
**D59**	1.02/1.56	6.7/18	6.2/16.7	[256]	315937232	*J. curcas*	ubiquitin-conjugating family protein
**D60**	1.27/1.52	5.53/72	5.07/54.1	[271]	226531470	*Z. mays*	vacuolar ATP synthase subunit B
**D61**	-1.78/-2.65	6.34/22	5.64/20.9	[175]	2832430	*H. brasiliensis*	pro-hevein
**D62**	1.61/1.31	6.32/56	5.54/40.1	[195]	114199044	*M. domestica*	caffeic acid O-methyltransferase
**D63**	1.12/1.57	5.74/29	8.33/27.7	[77]	255538084	*R. communis*	Adenine phosphoribosyltransferase
**D64**	1.52/1.51	5.79/35	5.55/26.0	[94]	255546213	*R. communis*	o-methyltransferase, putative
**D65**	1.53/1.52	4.88/13	5.04/14.7	95	132270	*H. brasiliensis*	Rubber elongation factor protein
**D66**	1.57/1.51	4.9/23	4.8/22.3	122	14423933	*H. brasiliensis*	Small rubber particle protein
**D67**	1.56/1.53	4.82/23	4.8/22.3	135	14423933	*H. brasiliensis*	Small rubber particle protein
**D68**	1.54/1.51	4.88/26	4.79/29.5	93	388503664	*L. japonicus*	unknown
**D69**	1.57/1.14	5.05/49	5.01/46.6	96	4235430	*H. brasiliensis*	latex-abundant protein
**D70**	1.03/-1.52	5.18/15	8.01/11.2	[86]	30575570	*C. paradisi*	HSP19 class II, partial
**D71**	-1.54/-1.7	5.61/15	8.29/35.1	[60]	37806305	*O. sativa*	hypothetical protein
**D72**	-1.55/-1.56	5.69/27	8.72/34.1	[56]	3451147	*H. brasiliensis*	chitinase
**D73**	-1.59/-1.58	6.75/33	7.1/36.9	86	255540341	*R. communis*	glyceraldehyde 3-phosphate dehydrogenase
**D74**	1.01/-1.55	4.95/40	5.0/43.0	[81]	3288200	*H. brasiliensis*	latex allergen
**D75**	-1.22/1.53	4.91/39	5.0/43.0	[138]	1916805	*H. brasiliensis*	latex patatin homolog
**D76**	-1.11/3.13	5.03/40	5.12/43.0	152	41581137	*H. brasiliensis*	latex allergen hev b 7.02
**D77**	-1.07/2.02	5.06/40	5.12/43.0	142	41581137	*H. brasiliensis*	latex allergen hev b 7.02
**D78**	-1.27/-1.58	3.65/33	9.45/38.9	156	4469175	*H. brasiliensis*	beta-1,3-glucanase
**D79**	-1.17/-1.61	5.64/16	6.22/34.3	84	34538473	*H. vulgare*	caleosin 1
**D80**	1.54/1.16	5.03/49	5.49/47.1	144	255560725	*R. communis*	dead box ATP-dependent RNA helicase
**D81**	1.52/1.1	4.91/46	4.65/41.4	[76]	255578278	*R. communis*	hypothetical protein RCOM_0537780
**D82**	-1.04/1.68	5.27/44	5.44/50.1	83	2501850	*N. tabacum*	GDP dissociation inhibitor
**D83**	-1.06/-1.52	4.9/49	4.72/28.9	[74]	3264769	*P. armeniaca*	late embryogenesis-like protein
**D84**	1.04/1.55	4.98/27	5.84/27.4	[69]	224099853	*P. trichocarpa*	predicted protein
**D85**	-1.56/-1.03	5.17/21	5.06/19.6	[410]	38122474	*H. brasiliensis*	rubber elongation factor

**D86**	-1.52/2.18	5.21/22	5.06/19.6	[226]	38122474	*H. brasiliensis*	rubber elongation factor
**D87**	1.53/1.52	4.62/19	4.06/15.2	[496]	334854634	*H. brasiliensis*	elicitor-responsive protein
**D88**	-1.57/-1.62	5.37/16	5.39/18.0	[180]	224144943	*P. trichocarpa*	predicted protein
**D89**	-1.58/-1.28	6.74/16	5.63/22.7	96	123062	*H. brasiliensis*	Pro-hevein
**D90**	-1.58/-4	5.42/22	5.06/19.6	99	38122474	*H. brasiliensis*	rubber elongation factor
**D91**	-1.02/-1.61	5.35/26	6.17/27.5	[49]	1709761	*S. oleracea*	Proteasome subunit alpha type-4
**D92**	-1.58/1.02	5.22/22	5.06/19.6	125	38122474	*H. brasiliensis*	rubber elongation factor
**D93**	-1.56/-1.52	5.75/23	8.44/29.9	93	157831407	*H. brasiliensis*	Chain A, Crystal Structures Of Hevamine
**D94**	1.03/-1.58	5.52/33	5.89/27.9	[89]	3127890	*C. arietinum*	cysteine synthase, O-acetyl-(thiol)-lyase
**D95**	-1.24/3.05	5.05/39	5.0/43.9	[59]	51315784	*H. brasiliensis*	Esterase
**D96**	1.39/5.24	5.29/21	5.06/19.6	98	38122474	*H. brasiliensis*	rubber elongation factor
**D97**	1.04/1.52	5.02/27	7.63/31.6	[55]	255554865	*R. communis*	lactoylglutathione lyase, putative
**D98**	1.58/1.39	5.51/26	5.93/27.6	86	15225839	*A. thaliana*	proteasome subunit alpha type-3
**D99**	1.55/1.02	4.45/15	4.61/12.8	[97]	29170601	*H. brasiliensis*	small rubber particle protein
**D100**	-1.09/-1.63	5.46/13	5.64/20.9	[84]	2832430	*H. brasiliensis*	pro-hevein
**D101**	-1.67/-1.16	5.55/14	6.82/15.0	[68]	224054224	*P. trichocarpa*	predicted protein
**D102**	1.13/1.72	5.01/11	5.45/11.2	81	268321205	*H. brasiliensis*	latex cystatin
**D103**	-1.62/-1.02	5.16/15	6.4/8.6	81	242083244	*S. bicolor*	hypothetical protein
**D104**	1.57/1.12	4.94/13	4.61/12.8	[85]	29170601	*H. brasiliensis*	small rubber particle protein
**D105**	-2.51/-3.62	4.13/13	4.1/16.9	[128]	354318	*T. aestivum*	calmodulin
**D106**	-1.51/-8.05	4.12/21	4.8/22.3	85	14423933	*H. brasiliensis*	Small rubber particle protein
**D107**	-2.36/1.06	4.45/24	4.8/22.3	165	14423933	*H. brasiliensis*	Small rubber particle protein
**D108**	-1.62/-1.1	5.36/47	5.57/48.0	107	14423688	*H. brasiliensis*	Enolase 1
**D109**	1.61/1.65	5.01/26	5.48/25.6	[52]	333690873	*H. brasiliensis*	glutathione-S-transferase tau 1
**D110**	-1.12/-1.57	5.09/15	5.95/17.6	[87]	140083605	*R. hybrid*	cytosolic class II HSP17.5
**D111**	-2.32/-11.52	4.02/41	5.01/14.6	[88]	37901124	*H. brasiliensis*	latex abundant protein 1
**D112**	-1.27/-1.87	4.35/40	4.53/38.0	[173]	359806300	*G. max*	uncharacterized protein
**D113**	-8.92/-8.16	4.28/38	4.53/38.1	[166]	37625031	*V. aestivalis*	ankyrin-repeat protein
**D114**	1.02/-1.7	4.39/38	4.45/37.5	[71]	13310811	*N. tabacum*	ankyrin-repeat protein HBP1
**D115**	-1.64/-1.6	4.31/36	4.5/25.3	[117]	378464888	*A. mongolicus*	translation elongation factor
**D116**	-1.08/-1.94	4.83/30	5.26/35.7	[132]	22261807	*S. tuberosum*	Light-induced protein, chloroplastic
**D117**	1.21/1.73	5.13/30	5.64/27.4	[136]	147805616	*V. vinifera*	hypothetical protein VITISV_028800
**D118**	1.63/1.17	5.08/46	5.04/54.4	[377]	225428086	*V. vinifera*	V-type proton ATPase subunit B 1
**D119**	1.01/1.56	4.78/42	5.28/47.1	[59]	224071449	*P. trichocarpa*	phospholipase A
**D120**	-1.57/1.52	4.81/16	5.91/13.4	81	116789736	*P. sitchensis*	unknown
**D121**	-1.12/-1.65	5.35/17	5.98/51.0	[51]	115392253	*C. betuloides*	polyphenol oxidase
**D122**	-1.21/-1.9	5.36/19	5.39/18.0	[210]	225430941	*V. vinifera*	regulator of ribonuclease-like protein 2
**D123**	-1.81/1.07	4.81/19	9.23/58.4	[104]	255539441	*R. communis*	actin depolymerizing factor, putative
**D124**	-1.1/2.05	5.32/24	6.06/22.6	[369]	357480975	*M. truncatula*	Proteasome subunit beta type
**D125**	-1.21/1.65	5.51/37	5.54/41.0	[123]	380468126	*H. brasiliensis*	caffeic acid 3-O-methyltransferase
**D126**	-1.05/1.55	5.28/36	5.54/41.0	[117]	380468126	*H. brasiliensis*	caffeic acid 3-O-methyltransferase
**D127**	-1.59/1.15	5.11/26	5.41/27.5	[159]	284433794	*J. curcas*	glutathione S-transferase omega
**D128**	-1.05/-1.64	5.56/28	6.09/30.8	[52]	255567721	*R. communis*	carboxymethylenebutenolidase, putative
**D129**	-1.74/1.13	5.44/33	5.5/34.4	82	255542380	*R. communis*	cysteine synthase, putative

**D130**	-1.62/-1.64	5.09/72	5.18/90.5	174	351727028	*G. max*	cell division cycle protein 48 homolog
**D131**	-1.65/-1.12	6.28/38	6.86/51.8	[802]	14916100	*H. brasiliensis*	hydroxymethylglutaryl CoA synthase
**D132**	-1.62/-1.01	5.25/34	8.64/36.3	[99]	357514973	*M. truncatula*	Annexin D4
**D133**	1.62/1.06	5.41/37	5.49/42.2	[314]	21536853	*A. thaliana*	phosphoglycerate kinase, putative
**D134**	1.71/1.16	5.68/39	5.82/41.6	[77]	255547137	*R. communis*	Alpha-1,4-glucan-protein synthase
**D135**	-1.02/1.57	5.31/25	5.48/25.6	[386]	333690873	*H. brasiliensis*	glutathione-S-transferase tau 1
**D136**	1.1/1.72	5.15/25	5.62/25.8	[104]	2970051	*V. radiata*	ARG10
**D137**	-1.66/1.63	5.23/21	5.64/20.9	[217]	2832430	*H. brasiliensis*	pro-hevein
**D138**	-1.1/-1.71	4.98/23	4.8/22.3	[384]	14423933	*H. brasiliensis*	Small rubber particle protein
**D139**	1.63/1.09	5.01/15	5.04/14.7	[379]	132270	*H. brasiliensis*	Rubber elongation factor protein
**D140**	-1.56/-1.62	5.54/13	5.64/20.9	[344]	2832430	*H. brasiliensis*	pro-hevein
**D141**	-1.63/-1.54	5.35/13	5.63/22.7	[158]	123062	*H. brasiliensis*	Pro-hevein
**D142**	1.63/1.51	5.01/13	5.04/14.7	[405]	132270	*H. brasiliensis*	Rubber elongation factor protein
**D143**	1.61/1.64	5.11/13	5.04/14.7	[470]	132270	*H. brasiliensis*	Rubber elongation factor protein

Note:

[a]Assigned spot number (D1-D143) as indicated in Fig. 3.

[b]The relatively changed ratios of the target protein spots in the 3 and 5 days latex from rubber trees treated by water and ethylene. All the protein spots in the 2-D DIGE gels were analyzed by DeCyder 7.0 software.

[c, d]The experimental (c) and theoretical (d) values for the mass (kDa) and *p*I of identified proteins. The theoretical values were retrieved from the protein database, and the experimental values were produced by ImageMaster.

[e]Mascot searched scores (M.S.) against NCBInr for the MS and [MS/MS] searched results.

[f]Database accession (gi|) numbers according to NCBInr.

Supplementary Table S3 MS information of ethylene responsive latex proteins by iTRAQ

Protein No. I-[a]	Un[b] Score	Cov.[c] 95%	Peptides[d] (95%)	Ratio[e] E3/D3	Ratio[e] E5/D5	Accession species[f]	Protein name
**2**	66.5	67.1	135	0.38	0.57	tr|D1M8S7|D1M8S7_HEVBR	Beta-1,3-glucanase
**5**	58.21	95.4	66	3.25	1.54	sp|Q39967|ALL5_HEVBR	Major latex allergen Hev b 5
**7**	54.77	71.6	105	0.33	0.30	tr|Q706V4|Q706V4_HEVBR	Latex allergen hev b 7.02
**10**	44.55	56.2	49	0.39	0.75	tr|E5FR85|E5FR85_MORAL	Actin
**11**	43.62	38.3	34	1.58	1.42	tr|Q71EE1|Q71EE1_HEVBR	Heat shock protein
**12**	42.03	35.4	37	0.46	0.56	tr|Q5VLJ6|Q5VLJ6_HEVBR	50 kDa protein
**13**	41.88	76	55	0.65	0.45	tr|A9ZMZ4|A9ZMZ4_HEVBR	Acetyl-CoA C-acetyltransferase
**15**	39.75	45.2	55	0.30	0.37	sp|Q9LEJ0|ENO1_HEVBR	Enolase 1
**17**	37.25	65.5	49	0.52	0.30	tr|Q9ZSP8|Q9ZSP8_HEVBR	Latex-abundant protein
**18**	34.69	43.4	22	0.49	0.35	tr|Q944G0|Q944G0_HEVBR	Mevalonate disphosphate decarboxylase
**19**	32.73	46.6	41	0.50	0.77	sp|P81406|GAPN_PEA	glyceraldehyde-3-phosphate dehydrogenase
**22**	31.1	42.7	22	0.19	0.44	tr|Q944F8|Q944F8_HEVBR	Hydroxymethylglutaryl CoA synthase
**23**	30.18	25.5	30	2.17	1.57	tr|B9SI90|B9SI90_RICCO	Methyl transferase
**24**	30.15	42	36	0.31	0.59	tr|B9RZR0|B9RZR0_RICCO	ATP-citrate synthase
**25**	29.94	42.5	31	0.34	0.36	tr|B9R8U5|B9R8U5_RICCO	Phosphofructokinase, putative
**27**	27.55	51.5	17	0.53	0.86	tr|F2YZ43|F2YZ43_HEVBR	14-3-3 protein 2
**28**	27.3	65.4	20	0.38	0.39	tr|Q40208|Q40208_LOTJA	RAB2A
**30**	27.13	90	24	0.33	0.40	tr|F8SKC5|F8SKC5_HEVBR	Elicitor-responsive protein
**31**	27.02	37.6	16	1.60	1.32	tr|C6TEK4|C6TEK4_SOYBN	phospholipase D alpha 1
**32**	27.02	22.7	28	0.63	0.55	tr|B9RV56|B9RV56_RICCO	Phospholipase D
**33**	26.07	64.5	48	0.06	0.28	tr|B3FNQ1|B3FNQ1_HEVBR	Peptidyl-prolyl cis-trans isomerase
**39**	24.89	97.4	54	0.39	0.61	tr|Q84T88|Q84T88_HEVBR	Small rubber particle protein
**40**	24.28	45.2	40	2.07	1.56	tr|Q8H9B1|Q8H9B1_9ROSI	eukaryotic translation elongation factor 1
**42**	24.12	36.8	28	1.87	1.11	tr|B9SP64|B9SP64_RICCO	Phosphoglucomutase, putative
**43**	23.88	22.7	15	0.59	0.70	tr|B9S8D6|B9S8D6_RICCO	Trehalose-6-phosphate synthase
**49**	22.33	12.4	11	1.84	2.19	tr|B9I8G4|B9I8G4_POPTR	Exportin-1
**50**	22.29	26	14	1.51	2.20	tr|E9KF95|E9KF95_HEVBR	Invertase 2
**53**	21.98	15.9	14	0.57	0.62	tr|A9NIV2|A9NIV2_MANES	Sucrose synthase
**54**	21.97	32.8	19	0.31	0.92	tr|B9NBW3|B9NBW3_POPTR	Malate dehydrogenase
**55**	21.39	30.8	18	1.51	1.63	tr|B9RSN1|B9RSN1_RICCO	Chaperonin containing t-complex protein 1
**57**	21.05	24.2	17	3.50	2.24	tr|E4MXI2|E4MXI2_THEHA	Luminal binding protein
**58**	21.05	22.7	12	0.36	0.70	tr|B9RM60|B9RM60_RICCO	Glu-fructose-6-phosphate aminotransferase
**60**	20.86	29.1	28	0.29	0.40	tr|B9STR1|B9STR1_RICCO	Leucine aminopeptidase, putative1
**61**	20.57	41.2	17	0.63	0.39	tr|B9RIY3|B9RIY3_RICCO	Rab GDP dissociation inhibitor
**64**	19.65	36.3	16	0.60	0.44	tr|B9RLK7|B9RLK7_RICCO	Alpha-1,4-glucan-protein synthase
**69**	18.74	19.2	18	0.58	0.57	tr|B9RBP6|B9RBP6_RICCO	Heat shock protein 70
**70**	18.66	38.1	12	0.52	0.45	tr|Q9ZSZ6|Q9ZSZ6_HEVBR	DnaJ protein
**71**	18.31	33.9	13	0.54	0.63	tr|B9RHY3|B9RHY3_RICCO	Phosphoglycerate kinase
**73**	18.11	13.1	13	0.63	0.52	tr|B9SYR5|B9SYR5_RICCO	Alanyl-tRNA synthetase, putative
**76**	17.33	21.8	9	1.72	1.29	tr|B9I5Y4|B9I5Y4_POPTR	T-complex protein 1 subunit theta
**77**	17.27	42.2	15	2.23	1.96	tr|Q8L3U6|Q8L3U6_HEVBR	Cis-prenyl transferase II

**80**	16.77	39.6	10	0.47	0.72	tr|B9S943|B9S943_RICCO	GTP-binding protein ras-like protein
**83**	16.69	7.1	7	0.23	0.66	tr|F6HJZ9|F6HJZ9_VITVI	cullin-associated protein 1
**85**	16.45	40.7	14	1.71	1.12	tr|B9RR89|B9RR89_RICCO	Proteasome subunit alpha type
**86**	16.41	37.5	8	2.07	1.87	tr|C6TKI4|C6TKI4_SOYBN	Proteasome subunit alpha type
**87**	16.33	16.6	10	0.41	0.26	tr|B9R7S8|B9R7S8_RICCO	Glucose-6-phosphate isomerase
**89**	16.31	19.8	12	2.11	1.37	tr|B9S5K6|B9S5K6_RICCO	26S proteasome regulatory subunit
**95**	16.17	27.3	13	0.47	0.74	tr|D2D2Z1|D2D2Z1_GOSHI	UDP-D-glucose pyrophosphorylase
**98**	15.62	14.3	12	0.61	0.59	tr|B9SJV6|B9SJV6_RICCO	Eukaryotic translation initiation factor 2c
**99**	15.49	11.9	10	1.80	1.49	tr|A5AHP0|A5AHP0_VITVI	Coatomer subunit beta
**102**	15.18	18.4	15	0.42	0.59	tr|B9SH99|B9SH99_RICCO	Nucleoredoxin, putative
**106**	14.96	32.3	13	0.44	0.76	tr|B9SIT3|B9SIT3_RICCO	L-lactate dehydrogenase
**108**	14.87	27.6	10	1.51	1.47	tr|A5BEF3|A5BEF3_VITVI	26S proteasome regulatory subunit 4
**109**	14.75	17.6	10	0.55	0.72	tr|B9SS36|B9SS36_RICCO	Chaperonin containing t-complex protein 1
**112**	14.47	30	13	0.08	0.10	tr|B9RXK1|B9RXK1_RICCO	Lactoylglutathione lyase, putative
**115**	14.31	28.7	9	3.10	1.52	tr|B9SN65|B9SN65_RICCO	Glucose 4-6-dehydratase, putative
**117**	14.13	42.8	7	0.30	0.37	tr|A9PFQ6|A9PFQ6_POPTR	Regulator of ribonuclease activity A
**119**	14.02	22.1	8	0.22	0.28	tr|Q547W6|Q547W6_HEVBR	Hydroxymethylglutaryl CoA reductase
**122**	13.95	13.5	11	0.58	1.19	tr|B9II82|B9II82_POPTR	villin-2
**125**	13.43	11.8	11	0.17	0.40	tr|F6HTU8|F6HTU8_VITVI	Cysteine synthase
**129**	13.13	39.2	7	0.52	0.66	tr|C6TGU2|C6TGU2_SOYBN	Proteasome subunit alpha type
**131**	12.95	31.6	7	2.47	1.62	sp|O22518|RSSA_SOYBN	40S ribosomal protein SA
**132**	12.91	19.8	9	2.27	1.66	tr|D2D2Z2|D2D2Z2_GOSHI	UDP-D-glucose dehydrogenase
**133**	12.91	25.4	9	0.56	0.73	tr|Q8L7F4|Q8L7F4_HEVBR	Farnesyl diphosphate synthase
**134**	12.79	27.6	9	0.21	0.77	tr|F6HPS0|F6HPS0_VITVI	Serine/threonine-protein phosphatase
**135**	12.78	18.2	12	0.63	1.20	tr|B9RCK2|B9RCK2_RICCO	Calcium-dependent protein kinase
**136**	12.7	18.5	6	0.64	0.79	tr|D7TKK5|D7TKK5_VITVI	obg-like ATPase 1
**138**	12.64	19.3	9	0.46	0.69	tr|D7SUQ2|D7SUQ2_VITVI	GTP-binding nuclear protein Ran-3
**141**	12.36	27	10	0.55	0.47	tr|D7SUD7|D7SUD7_VITVI	Aspartate aminotransferase
**145**	12.21	34.9	15	0.29	0.38	sp|Q39962|CBPR_HEVBR	Citrate-binding protein
**148**	12	61.8	41	0.55	0.66	tr|A9ZMZ5|A9ZMZ5_HEVBR	Acetyl-CoA C-acetyltransferase
**150**	11.87	29.8	10	2.40	1.42	sp|Q39963|PDX1_HEVBR	Pyridoxal biosynthesis protein PDX1
**151**	11.81	27.5	9	1.75	1.51	tr|B9RC03|B9RC03_RICCO	NAD dependent epimerase/dehydratase
**152**	11.77	50	23	0.61	0.63	tr|B1NDK1|B1NDK1_9ERIC	Calmodulin
**160**	11.38	20.7	7	0.44	0.62	tr|B9RAM4|B9RAM4_RICCO	26S proteasome non-atpase
**161**	11.37	21.3	22	0.31	0.42	tr|Q6XNP8|Q6XNP8_HEVBR	Abscisic acid stress ripening-related protein
**167**	11.12	18	7	10.47	2.51	tr|C7B9C0|C7B9C0_RICCO	Myo-inositol-1-phosphate synthase
**168**	10.98	22.2	11	0.45	0.64	tr|B9SZU0|B9SZU0_RICCO	Catalytic, putative
**169**	10.97	15.2	5	1.53	1.66	tr|B9SWY5|B9SWY5_RICCO	Proliferation-associated 2g4
**170**	10.95	26.1	12	0.30	0.52	tr|B9SVA7|B9SVA7_RICCO	Aspartic proteinase, putative
**175**	10.83	33.5	8	0.08	0.59	tr|Q6J6I5|Q6J6I5_HEVBR	Phosphoglyceride transfer protein
**182**	10.63	15.2	10	1.75	1.24	tr|B9RYE9|B9RYE9_RICCO	Polyadenylate-binding protein,
**186**	10.47	29	7	1.60	1.41	tr|B9SPS6|B9SPS6_RICCO	Proteasome subunit beta type
**187**	10.45	42.2	8	0.52	0.70	tr|B9GTP5|B9GTP5_POPTR	ras-related protein RABC1 isoform
**188**	10.41	17.3	7	7.52	1.63	sp|P29685|ATPBM_HEVBR	ATP synthase subunit beta

**191**	10.38	7	5	0.58	0.60	tr|B9RUL1|B9RUL1_RICCO	Acid phosphatase, putative
**195**	10.23	4.2	5	5.50	2.29	tr|B9RN01|B9RN01_RICCO	Putative uncharacterized protein
**197**	10.18	24.9	10	0.55	0.59	tr|B9SIC4|B9SIC4_RICCO	chitinase Class I, putative
**198**	10.16	20.1	9	0.52	0.31	tr|B9RTJ4|B9RTJ4_RICCO	Stem-specific protein TSJT1SV=1
**204**	9.98	21.7	9	1.85	1.56	tr|A9PFJ7|A9PFJ7_POPTR	Pyruvate kinase
**205**	9.88	66.3	40	0.66	0.75	tr|F8RW92|F8RW92_HEVBR	Peptidyl-prolyl cis-trans isomerase
**208**	9.79	6.4	6	3.31	2.11	tr|B9T1E9|B9T1E9_RICCO	Importin beta-3, putative
**210**	9.66	31.3	12	1.67	1.53	tr|Q2XPH2|Q2XPH2_MANIN	Profilin
**212**	9.6	37.5	7	0.35	0.72	tr|Q8GZP2|Q8GZP2_HEVBR	Superoxide dismutase [Cu-Zn]
**213**	9.57	13.9	8	0.66	0.53	tr|B9RKX9|B9RKX9_RICCO	Seryl-tRNA synthetase, putative
**216**	9.47	18.8	21	0.36	0.77	tr|B9GH43|B9GH43_POPTR	Phospholipase D
**219**	9.44	47.7	12	0.65	0.66	tr|F2YZ44|F2YZ44_HEVBR	14-3-3 protein 3
**220**	9.44	19.2	6	0.61	0.63	tr|Q14VV8|Q14VV8_BOENI	Caffeic acid O-methyltransferase
**221**	9.34	35.3	10	0.28	0.53	tr|D2JWP4|D2JWP4_POPDE	Pathogenesis-related thaumatin
**224**	9.24	32.6	7	0.52	0.61	tr|D2WL72|D2WL72_POPTR	Glutathione transferase
**226**	9.2	32.7	26	0.57	0.52	tr|E8Z9H3|E8Z9H3_HEVBR	Latex cystatin
**227**	9.17	40.6	14	0.17	0.40	tr|F2VXU6|F2VXU6_HEVBR	Rab4
**230**	9.12	14.9	7	0.54	0.62	tr|B9H8X6|B9H8X6_POPTR	6-phosphofructokinase 3
**241**	8.78	35.9	6	0.40	0.58	tr|D6BR55|D6BR55_9ROSI	Acyl-CoA-binding protein
**242**	8.71	61.9	13	0.58	0.58	tr|D9I9X9|D9I9X9_HEVBR	Actin-depolymerizing factor
**244**	8.65	23.3	6	1.61	1.50	tr|B9R7X1|B9R7X1_RICCO	Proteasome subunit alpha type
**245**	8.65	41.8	7	0.65	0.70	tr|F8RWS9|F8RWS9_HEVBR	ubiquitin-protein ligase 1
**246**	8.6	12.1	6	1.79	0.69	tr|B9HR67|B9HR67_POPTR	Plant synaptotagmin
**247**	8.57	6.7	4	2.03	1.66	tr|B9SE11|B9SE11_RICCO	Delta 1-pyrroline-5-carboxylate synthetase
**248**	8.56	16.7	6	0.34	0.50	tr|F6HNF4|F6HNF4_VITVI	adenosine kinase 2
**256**	8.36	19.2	6	0.66	0.75	tr|B9HJ73|B9HJ73_POPTR	Fructose-bisphosphate aldolase
**261**	8.15	14.2	8	0.53	0.96	tr|B9RKG2|B9RKG2_RICCO	Arginyl-tRNA synthetase, putative
**262**	8.15	10.3	5	0.20	0.65	tr|E2DQG6|E2DQG6_MANES	Carotenoid cleavage dioxygenase 1
**264**	8.09	43	5	0.28	0.31	tr|B3FNP8|B3FNP8_HEVBR	Glutaredoxin
**266**	8.05	42	8	0.46	0.58	tr|B9T509|B9T509_RICCO	ras-related protein RABA3
**267**	8.02	29	6	0.39	0.12	tr|B9RKM9|B9RKM9_RICCO	Peroxiredoxin, putative
**268**	8	9.3	4	1.53	1.67	tr|B9S6T7|B9S6T7_RICCO	Ubiquilin, putative
**269**	7.99	7.5	5	0.61	0.57	tr|B9IGY9|B9IGY9_POPTR	2,3-bispho-phosphoglycerate mutase
**278**	7.82	20.6	10	0.58	0.62	tr|B9SXW5|B9SXW5_RICCO	26S proteasome non-atpase
**279**	7.78	14.4	7	0.47	0.67	tr|B9GXV5|B9GXV5_POPTR	FAS-associated factor 2
**280**	7.72	3.6	5	0.34	0.50	tr|B9RRX1|B9RRX1_RICCO	Putative uncharacterized protein
**281**	7.7	12.3	5	3.25	0.90	tr|B9SR96|B9SR96_RICCO	Glutamate decarboxylase, putative
**284**	7.66	15	5	0.38	1.00	tr|B2Z6Q5|B2Z6Q5_POPTR	hydroxycinnamoyltransferase
**289**	7.53	7.3	6	1.50	1.39	tr|B9SCN8|B9SCN8_RICCO	Eukaryotic translation initiation factor 2c
**290**	7.51	21.9	22	0.66	1.37	tr|O04975|O04975_MESCR	Fructose-bisphosphate aldolase
**293**	7.44	18.2	5	0.11	0.68	tr|B9S3Y6|B9S3Y6_RICCO	Nuclear protein localization
**297**	7.32	21.9	5	0.50	0.64	tr|B9S228|B9S228_RICCO	Nucleic acid binding protein
**299**	7.28	28.7	9	1.57	0.84	tr|B9HUT6|B9HUT6_POPTR	Proteasome subunit beta type
**306**	7.1	12.1	5	2.78	1.59	tr|B9HIC5|B9HIC5_POPTR	differentiation associated protein

**310**	7.05	18.2	4	1.51	1.12	tr|B9RIC4|B9RIC4_RICCO	40S ribosomal protein S2, putative
**314**	6.98	13.9	4	0.47	0.52	tr|A9PEP6|A9PEP6_POPTR	DNA damage-inducible protein
**315**	6.98	5	4	0.44	0.60	tr|F6H158|F6H158_VITVI	Putative uncharacterized protein
**321**	6.86	6.5	3	1.80	1.08	tr|B9SG36|B9SG36_RICCO	Ubiquitin-protein ligase, putative
**322**	6.86	20.2	7	0.44	2.25	tr|B9GXZ6|B9GXZ6_POPTR	alanine aminotransferase
**324**	6.85	19.6	5	0.51	0.71	tr|A5AQP2|A5AQP2_VITVI	ATP-citrate synthase alpha chain
**327**	6.81	33.3	36	0.65	0.70	tr|B2MW33|B2MW33_HEVBR	Heat shock protein 70
**328**	6.77	17.7	5	0.32	2.32	tr|B9SCD6|B9SCD6_RICCO	Esterase D, putative
**329**	6.77	30.3	6	0.23	0.75	tr|B9H2B8|B9H2B8_POPTR	Predicted protein
**335**	6.7	24.3	7	0.37	0.53	tr|B9RLJ0|B9RLJ0_RICCO	L-asparaginase, putative
**338**	6.66	12.1	4	0.61	1.26	tr|B9HLM0|B9HLM0_POPTR	Chorismate synthase
**343**	6.6	17.6	4	2.09	0.82	tr|B9SKG4|B9SKG4_RICCO	40S ribosomal protein S11, putative
**345**	6.54	6.8	4	0.33	1.27	tr|E6NU17|E6NU17_9ROSI	JHL07K02.17 protein
**348**	6.52	7	4	31.33	2.51	tr|B9SUE9|B9SUE9_RICCO	Hydrolase, O-glycosyl compounds
**349**	6.51	31.9	4	2.42	1.28	tr|B9SWB0|B9SWB0_RICCO	60S ribosomal protein L12, putative
**350**	6.49	14.7	4	0.48	0.84	tr|B9HG22|B9HG22_POPTR	GTP-binding protein
**351**	6.48	37.8	14	0.39	0.72	tr|A9ZN23|A9ZN23_HEVBR	Cis-prenyl transferase
**355**	6.46	22.7	3	2.27	0.86	tr|B9S120|B9S120_RICCO	40S ribosomal protein S14, putative
**357**	6.44	64	14	0.51	1.17	tr|Q6T708|Q6T708_HEVBR	Rubber elongation factor
**364**	6.34	13.9	5	0.60	0.62	tr|Q9ZTT8|Q9ZTT8_GOSHI	Chitinase, Class I
**366**	6.31	67.1	9	2.78	1.56	tr|B3FNP9|B3FNP9_HEVBR	Protease inhibitor protein
**369**	6.2	51.7	17	2.61	1.12	tr|A9P8X5|A9P8X5_POPTR	Ras-related protein RABB1b
**370**	6.18	11.2	7	1.64	1.17	tr|F6HGX0|F6HGX0_VITVI	Asparaginyl-tRNA synthetase
**371**	6.18	17.6	5	0.63	1.25	tr|O24328|O24328_RICCO	S-adenosyl-methionine hyltransferase
**372**	6.14	8.1	3	2.31	2.14	tr|F6HQV3|F6HQV3_VITVI	60S ribosomal protein L4
**373**	6.14	13.6	3	0.28	0.31	tr|B9SE47|B9SE47_RICCO	Malate dehydrogenase
**374**	6.12	18.5	7	0.09	0.29	tr|B9SUZ4|B9SUZ4_RICCO	Alcohol dehydrogenase
**375**	6.11	17.5	14	0.39	0.51	tr|G3G858|G3G858_CAMSI	Methionine synthase
**382**	6.03	24.2	4	1.71	1.47	tr|B9SA27|B9SA27_RICCO	Trafficking protein particle complex subunit
**383**	6.02	9.9	3	0.50	0.58	tr|Q5J3P0|Q5J3P0_MALDO	Sucrose phosphate phosphatase
**386**	6	19.1	3	2.19	1.74	tr|B9RYZ1|B9RYZ1_RICCO	60S ribosomal protein L9
**389**	6	6.5	3	6.19	1.20	tr|B9IBL7|B9IBL7_POPTR	Predicted protein
**390**	5.99	18	7	0.39	0.71	tr|F6I510|F6I510_VITVI	Putative uncharacterized protein
**391**	5.98	11.8	5	0.54	1.06	tr|B9IER7|B9IER7_POPTR	Carbonic anhydrase
**392**	5.95	7.6	4	5.06	1.78	tr|B9SN36|B9SN36_RICCO	Protein SEY1;root hair defective 3
**395**	5.85	17.4	3	1.75	1.46	tr|B9SU88|B9SU88_RICCO	Receptor for activated protein kinase
**401**	5.79	22.9	4	1.61	3.14	tr|G0YC94|G0YC94_9ROSI	Fumarylacetoacetate hydrolase
**408**	5.72	65.7	8	1.51	1.34	sp|Q6XNP7|HPI_HEVBR	Protease inhibitor
**409**	5.7	13.6	8	0.58	0.50	tr|B9T5Y1|B9T5Y1_RICCO	bisphosphate phosphodiesterase
**410**	5.68	8.9	4	2.29	2.13	tr|B9GU26|B9GU26_POPTR	Protein disulfide isomerase, putative
**412**	5.65	8.4	4	3.16	1.62	tr|F6HLF4|F6HLF4_VITVI	DEAD-box ATP-dependent RNA helicase
**413**	5.62	15.1	3	0.64	1.24	tr|B9A9C9|B9A9C9_MORAL	Urease accessory protein ureG
**414**	5.61	7.9	3	0.41	1.27	tr|B9N3J3|B9N3J3_POPTR	Predicted protein
**419**	5.57	11.9	2	0.41	0.73	tr|B9IJM2|B9IJM2_POPTR	Predicted protein

**422**	5.53	43.4	9	0.25	0.39	tr|Q94KV2|Q94KV2_MANES	Aspartic protease
**424**	5.45	43.5	31	3.02	2.08	tr|B9HV14|B9HV14_POPTR	Vacuolar H+-ATPase catalytic subunit A
**426**	5.44	42.9	55	0.41	0.51	sp|Q9LEI9|ENO2_HEVBR	Enolase 2
**429**	5.42	8.3	4	0.43	0.58	tr|C6TFR0|C6TFR0_SOYBN	alpha-galactosidase-like isoform 1
**436**	5.37	7.2	3	0.65	1.35	tr|Q6S9W9|Q6S9W9_GOSHI	Betaine-aldehyde dehydrogenase
**437**	5.35	12.4	5	0.45	0.61	tr|B9SPZ1|B9SPZ1_RICCO	2-hydroxyphytanoyl-CoA lyase
**439**	5.34	9.4	5	2.58	2.14	sp|Q38871|CDPK5_ARATH	Calcium-dependent protein kinase 5
**441**	5.34	8	6	0.10	0.39	tr|B9SX16|B9SX16_RICCO	acetylglucosamine transferase
**447**	5.22	16.1	4	0.21	0.17	tr|D7TLU7|D7TLU7_VITVI	Triosephosphate isomerase
**448**	5.22	17.3	4	9.73	2.08	tr|B9RUZ3|B9RUZ3_RICCO	Serine-threonine kinase receptor
**449**	5.22	21.7	3	1.56	1.71	tr|A9PGL4|A9PGL4_POPTR	40S ribosomal protein
**453**	5.19	20.4	5	0.38	0.53	tr|B9MU39|B9MU39_POPTR	quinone oxidoreductase, putative
**456**	5.16	59.1	79	0.13	0.65	tr|Q7XJ83|Q7XJ83_HEVBR	Beta-1,3-glucanase
**459**	5.11	38.7	34	0.47	0.54	tr|B9HIN6|B9HIN6_POPTR	ATP-citrate synthase beta chain
**467**	4.99	7.7	3	1.58	1.11	tr|D7U7I9|D7U7I9_VITVI	COP9 signalosome complex 1
**475**	4.89	5.6	2	0.60	0.64	tr|B9RF45|B9RF45_RICCO	Amidase, putative
**478**	4.86	5.2	3	0.52	1.06	tr|B9SBV7|B9SBV7_RICCO	Ligatin, putative
**481**	4.82	5.9	2	3.40	1.38	tr|B9RMR2|B9RMR2_RICCO	Steroid dehydrogenase, putative
**482**	4.81	11.6	5	0.61	0.56	tr|B9SGQ4|B9SGQ4_RICCO	Importin alpha-2 subunit, putative
**485**	4.79	8.8	4	0.58	0.72	tr|B9HRN7|B9HRN7_POPTR	Adenylyl cyclase-associated protein
**487**	4.74	10.6	2	0.66	0.89	tr|B9RF46|B9RF46_RICCO	Mevalonate kinase, putative
**498**	4.69	30.4	34	0.10	0.44	tr|B6EBD6|B6EBD6_SOYBN	Heat shock protein 90-2
**503**	4.65	4.9	2	0.60	0.69	tr|A9PF66|A9PF66_POPTR	isoflavone reductase related protein
**504**	4.65	13.2	3	0.44	0.57	tr|Q9STB5|Q9STB5_HEVBR	Superoxide dismutase
**506**	4.63	16.6	7	0.63	0.70	tr|C6TIP9|C6TIP9_SOYBN	Cysteine synthase
**510**	4.59	15.8	15	1.54	1.43	tr|B9T216|B9T216_RICCO	Fimbrin, putative
**511**	4.59	5.2	2	0.26	1.11	tr|B9HMM6|B9HMM6_POPTR	Folylpolyglutamate synthase
**512**	4.58	5.5	6	1.61	1.52	tr|B9S596|B9S596_RICCO	Myosin XI, putative
**514**	4.58	4.5	3	0.29	0.25	tr|A5C5R6|A5C5R6_VITVI	Cinnamyl alcohol dehydrogenase 1
**516**	4.57	12.2	5	0.14	0.57	tr|B9S1F8|B9S1F8_RICCO	Acetylornithine deacetylase
**520**	4.51	5.6	5	0.38	0.57	tr|B9SUT5|B9SUT5_RICCO	Isoleucyl tRNA synthetase, putative
**533**	4.37	9.8	2	0.31	0.21	tr|D7LDD9|D7LDD9_ARALL	3R-hydroxymyristoyl ACP dehydrase
**534**	4.35	20	10	0.23	0.36	tr|B9RHV9|B9RHV9_RICCO	Glyceraldehyde 3-phosphate dehydrogenase
**536**	4.35	12.9	5	0.22	0.55	tr|B5M1W3|B5M1W3_RHEAU	Glutathione-S-transferase
**547**	4.31	15.8	8	15.42	3.93	tr|Q9M563|Q9M563_VITVI	Beta-1,3-glucanase
**549**	4.3	17.7	5	0.18	2.47	tr|B9HFG8|B9HFG8_POPTR	annexin1
**550**	4.3	8.2	3	0.61	0.39	tr|B9SS35|B9SS35_RICCO	Argininosuccinate synthase, putative
**552**	4.28	9.2	4	0.13	0.25	tr|Q5PYQ3|Q5PYQ3_MANES	Glycerophosphodiester phosphodiesterase
**556**	4.24	7.6	3	0.61	0.68	tr|B9SL55|B9SL55_RICCO	DNA-binding protein smubp-2
**561**	4.21	5.1	2	1.67	1.63	tr|B9I337|B9I337_POPTR	transportin, putative
**562**	4.21	17.7	3	1.60	1.65	tr|B9HYZ8|B9HYZ8_POPTR	vacuolar protein sorting-associated protein
**563**	4.2	16	7	0.29	0.42	tr|B9SFR8|B9SFR8_RICCO	Aspartic proteinase, putative
**567**	4.17	50.4	7	0.65	1.98	sp|O65812|PROF1_HEVBR	Profilin-1
**572**	4.15	7.5	2	7.45	2.63	tr|Q307Z8|Q307Z8_SOLTU	ribosomal protein-like

**578**	4.13	45.4	5	0.65	0.52	tr|D4P094|D4P094_HEVBR	Copper transport protein ATOX1
**580**	4.12	10.2	4	0.60	2.02	tr|C6TIG8|C6TIG8_SOYBN	auxin-induced protein PCNT115
**584**	4.09	14	2	0.64	1.17	tr|B9IPT2|B9IPT2_POPTR	FH protein interacting protein
**592**	4.05	8.7	2	1.71	1.69	tr|B9MUU5|B9MUU5_POPTR	Vesicle-associated protein 1
**596**	4.05	12.9	4	0.52	0.30	tr|B9SJL8|B9SJL8_RICCO	Protease C56, putative
**602**	4.04	7.3	2	2.31	1.96	sp|O04373|ILL4_ARATH	IAA-amino acid hydrolase ILR1-like 4
**605**	4.03	8.3	4	0.48	0.56	tr|B9SC09|B9SC09_RICCO	Putative uncharacterized protein
**606**	4.03	4.1	4	0.21	0.39	tr|Q9LUL3|Q9LUL3_ARATH	Lipase class 3 family protein
**610**	4.02	15.2	6	1.91	1.58	tr|D7SXZ2|D7SXZ2_VITVI	dehydrogenase/reductase
**612**	4.01	23.6	3	0.52	0.27	sp|O64459|UGPA_PYRPY	UTP-phosphate uridylyltransferase
**613**	4.01	13.1	12	0.24	0.21	tr|Q7Y237|Q7Y237_EUOEU	Chitinase
**618**	4	64.6	79	0.60	0.33	tr|Q6JYQ9|Q6JYQ9_HEVBR	HEV1.2
**625**	4	14.1	6	0.65	1.05	tr|B9ST42|B9ST42_RICCO	Pyruvate kinase
**628**	4	8.4	3	0.22	0.22	tr|B9GFV7|B9GFV7_POPTR	TBC1 domain family member 13
**631**	4	29.9	2	3.05	2.04	tr|F4YFE3|F4YFE3_CAMSI	Cytochrome b5
**634**	4	5.7	2	0.46	1.44	tr|C6TGB0|C6TGB0_SOYBN	Putative uncharacterized protein
**643**	4	32.8	4	0.30	1.26	tr|D9IZZ6|D9IZZ6_HEVBR	Translation machinery-associated protein 7
**648**	3.96	10.8	2	0.22	0.59	tr|D7SIZ7|D7SIZ7_VITVI	20 kDa chaperonin, chloroplastic
**650**	3.95	16.5	3	11.48	2.11	tr|D1ML39|D1ML39_9ROSI	Ribosomal protein
**651**	3.94	9.3	3	3.40	2.06	tr|B9RDG1|B9RDG1_RICCO	Calcium lipid binding protein
**653**	3.92	8.3	4	0.13	0.70	sp|Q9FK25|OMT1_ARATH	Quercetin 3-O-methyltransferase 1
**666**	3.83	44.6	37	0.24	0.99	tr|D7U1A1|D7U1A1_VITVI	glyceraldehyde-3-phosphate dehydrogenase
**668**	3.82	13.7	9	0.50	1.13	tr|Q8GZD8|Q8GZD8_SOLLC	aminopeptidase preprotein
**680**	3.74	14.4	4	9.46	2.53	tr|E0CPI5|E0CPI5_VITVI	hydrolase domain-containing protein
**683**	3.7	26.6	8	3.31	2.04	tr|D7SN69|D7SN69_VITVI	bifunctional polymyxin resistance protein
**687**	3.68	21.5	2	1.69	1.53	tr|B8Y6I1|B8Y6I1_HEVBR	V-type proton ATPase subunit F
**688**	3.66	8	3	0.54	0.47	tr|E0CRW7|E0CRW7_VITVI	argininosuccinate lyase
**692**	3.63	7	3	0.09	0.19	tr|B9RXB8|B9RXB8_RICCO	Homogentisate 1,2-dioxygenase
**694**	3.61	8.2	2	0.48	0.67	tr|B9SQS5|B9SQS5_RICCO	3-isopropylmalate dehydratase
**700**	3.54	15.2	5	0.61	0.87	tr|B9RAR0|B9RAR0_RICCO	Protein-L-isoaspartate O-methyltransferase
**703**	3.53	19.8	3	1.50	1.34	tr|D5JG84|D5JG84_9ROSI	Heat shock protein 17.5 class II
**704**	3.52	3	2	0.10	0.35	tr|D7T494|D7T494_VITVI	Putative uncharacterized protein
**708**	3.46	6.5	4	16.00	3.72	tr|B9T139|B9T139_RICCO	Hydrolase
**710**	3.44	17.3	6	0.55	0.61	tr|B9N432|B9N432_POPTR	ATP-citrate synthase, putative
**713**	3.44	7.6	2	12.13	2.13	tr|B9RWM3|B9RWM3_RICCO	Potassium channel beta, putative
**714**	3.43	11.2	3	0.59	0.68	tr|Q5EBY6|Q5EBY6_EUPCH	Aldehyde dehydrogenase family 7
**716**	3.43	20.1	7	0.23	0.48	tr|B9R7W5|B9R7W5_RICCO	Isoflavone reductase, putative
**717**	3.42	10.9	7	0.61	0.79	tr|B9GZ19|B9GZ19_POPTR	glycine-tRNA ligase 1
**718**	3.42	20.4	2	2.07	0.91	tr|B9RH67|B9RH67_RICCO	40S ribosomal protein S25-1
**720**	3.4	15.1	26	1.63	1.20	tr|A5ACP0|A5ACP0_VITVI	clathrin heavy chain 1
**723**	3.4	100	4	0.11	0.58	sp|P86104|UP01_VITRO	Unknown protein 1
**729**	3.37	16.8	19	0.62	1.09	tr|B9RQ33|B9RQ33_RICCO	5-methyl-homocysteine methyltransferase
**732**	3.37	8.5	2	4.02	11.62	tr|B9T676|B9T676_RICCO	Catalytic, putative

**735**	3.34	3.4	2	1.71	1.29	tr|D7T8E1|D7T8E1_VITVI	Phagocytic receptor 1b
**738**	3.33	6.1	2	0.43	0.25	tr|B9RLT7|B9RLT7_RICCO	Dimethylaniline monooxygenase
**740**	3.31	6.8	6	0.19	0.38	tr|Q8VZH2|Q8VZH2_ARATH	Aminopeptidase M1
**743**	3.29	2.3	2	2.11	1.19	tr|B9SDH3|B9SDH3_RICCO	Cytochrome P450, putative
**744**	3.29	6.8	2	1.61	2.01	tr|F6HIY1|F6HIY1_VITVI	cytochrome b5 reductase 1-like
**746**	3.29	11.3	2	0.62	0.41	tr|Q38HT5|Q38HT5_SOLTU	40S ribosomal protein S23
**757**	3.24	7.1	2	1.94	2.65	tr|A9PDL9|A9PDL9_POPTR	Very-long-chain enoyl-CoA reductase
**758**	3.22	6.4	3	0.52	1.67	tr|B9RRB9|B9RRB9_RICCO	Suppression of tumorigenicity
**759**	3.22	7.1	2	2.49	1.56	tr|B9SEA1|B9SEA1_RICCO	Peroxidase 12, putative
**760**	3.21	25	21	1.63	1.51	tr|F6H4T7|F6H4T7_VITVI	elongation factor 2-like
**763**	3.19	5.9	2	0.58	1.02	tr|E9M776|E9M776_HEVBR	Cinnamoyl-CoA reductase
**771**	3.09	5.7	2	0.58	0.52	tr|B0LUS9|B0LUS9_TOBAC	Multicopy suppressor
**772**	3.06	5.5	3	0.13	0.57	tr|B9IEZ0|B9IEZ0_POPTR	ubiquitin domain-containing protein
**774**	3.05	2.5	2	1.96	2.06	tr|B9SYU2|B9SYU2_RICCO	Vacuolar sorting protein
**778**	3.04	4.2	2	0.19	0.54	tr|A9PEK1|A9PEK1_POPTR	Phosphoglycerate dehydrogenase
**779**	3.03	8.6	6	0.27	0.32	tr|D7MNN8|D7MNN8_ARALL	Sucrose synthase
**780**	3.03	2.4	2	2.01	1.67	tr|B9HPP7|B9HPP7_POPTR	Calcium ATPase
**782**	3.02	6.4	2	0.54	0.27	sp|Q9M9E9|SRK2C_ARATH	Serine/threonine-protein kinase
**783**	3.02	4.5	2	0.62	0.38	tr|B9T5L2|B9T5L2_RICCO	Nonsense-mediated mRNA decay protein
**784**	3	15.9	4	1.64	1.77	tr|B9RTH6|B9RTH6_RICCO	ADP-ribosylation factor
**789**	2.98	10.3	3	0.10	0.14	tr|B9R730|B9R730_RICCO	Putative uncharacterized protein
**790**	2.98	3.6	3	0.48	1.16	sp|Q2XPU6|CAS1_RICCO	Cycloartenol synthase
**794**	2.95	23	10	0.24	0.19	tr|F6H7L5|F6H7L5_VITVI	Lactoylglutathione lyase
**799**	2.93	2.2	2	0.55	2.21	tr|A5BG74|A5BG74_VITVI	protein argonaute 4A-like
**801**	2.92	26	10	1.60	0.96	sp|Q9AT56|METK1_ELAUM	S-adenosylmethionine synthase 1
**807**	2.9	13.4	3	1.51	0.33	tr|B9SS61|B9SS61_RICCO	60S ribosomal protein L14
**808**	2.9	25.2	2	0.08	0.30	tr|B9IJS4|B9IJS4_POPTR	Thioredoxin
**813**	2.87	19.8	4	1.77	2.51	tr|B9IEB2|B9IEB2_POPTR	Ras-related protein RABA6b
**817**	2.85	65.7	63	0.62	0.34	sp|P02877|HEVE_HEVBR	Pro-hevein OS=Hevea brasiliensis
**819**	2.84	14.3	5	1.96	1.18	tr|Q66PF9|Q66PF9_PEA	Monodehydroascorbate reductase I
**825**	2.81	11.8	6	1.72	1.18	tr|F6HHF5|F6HHF5_VITVI	eukaryotic initiation factor 4A-3
**827**	2.81	7.7	2	0.31	0.35	tr|B9IAZ4|B9IAZ4_POPTR	Ribosomal protein L5A, 60S
**828**	2.81	13.3	2	1.75	0.48	tr|C6SZQ3|C6SZQ3_SOYBN	60S ribosomal protein L23A
**832**	2.78	3.5	2	2.21	1.97	tr|B9GSW4|B9GSW4_POPTR	Long chain acyl-CoA synthetase 1
**835**	2.77	8.1	2	1.51	1.02	tr|B9T7A3|B9T7A3_RICCO	Ubiquitin carboxyl-terminal hydrolase
**838**	2.76	6	2	0.63	1.29	tr|D7LLA4|D7LLA4_ARALL	Tubulin beta-7 chain
**839**	2.75	6.8	4	1.77	0.98	tr|B9RSG5|B9RSG5_RICCO	Dipeptidyl peptidase IV
**840**	2.75	16.7	3	0.38	1.15	tr|B9S069|B9S069_RICCO	Glutathione-s-transferase theta
**844**	2.72	16.4	4	0.61	0.51	tr|B9S7R8|B9S7R8_RICCO	Pyridoxine kinase, putative
**845**	2.72	8.4	5	0.21	0.18	tr|B9H6S4|B9H6S4_POPTR	rhamnogalacturonate lyase
**864**	2.65	9.4	4	0.62	0.98	tr|B9RZ84|B9RZ84_RICCO	Glutaredoxin, grx, putative
**868**	2.65	23.5	3	0.54	0.47	tr|D7TK03|D7TK03_VITVI	programmed cell death protein 5
**869**	2.64	5.7	3	0.30	0.34	tr|A5BDT4|A5BDT4_VITVI	14 kDa zinc-binding protein
**870**	2.64	7.2	2	0.53	2.38	sp|B9SL58|PURA2_RICCO	Adenylosuccinate synthetase 2

**871**	2.63	11	3	2.68	0.54	tr|B9SGS2|B9SGS2_RICCO	60S ribosomal protein L4
**879**	2.61	30.2	23	0.30	1.01	tr|B9H5X2|B9H5X2_POPTR	Phosphofructokinase
**885**	2.61	15.3	3	1.77	0.79	tr|E9JFW4|E9JFW4_9ROSI	Thioredoxin
**886**	2.6	8.9	2	0.64	0.97	tr|A5BVN4|A5BVN4_VITVI	Nucleoside diphosphate kinase
**888**	2.59	10	2	7.31	2.19	tr|B9RTJ5|B9RTJ5_RICCO	Coated vesicle membrane protein
**900**	2.53	15.9	2	0.47	0.24	tr|B9MT66|B9MT66_POPTR	Glutathione transferase GSTL3
**903**	2.53	11.9	3	0.46	0.22	tr|Q9S7R3|Q9S7R3_ARATH	177 protein
**904**	2.53	7.2	2	2.36	1.50	tr|G1ED19|G1ED19_GOSHI	Protein disulfide isomerase
**905**	2.52	9.1	3	0.35	0.16	tr|O49235|O49235_SOYBN	Glutathione S-transferase
**907**	2.5	19.1	10	0.59	1.07	tr|B7FKU7|B7FKU7_MEDTR	Cysteine synthase
**914**	2.48	3.5	2	1.89	1.96	tr|D7KNJ9|D7KNJ9_ARALL	trehalose-phosphate synthase
**921**	2.45	6.2	3	0.27	0.99	tr|B9SGK0|B9SGK0_RICCO	Adaptin ear-binding coat-associated protein
**929**	2.41	6.6	2	0.61	0.29	tr|B9T8C2|B9T8C2_RICCO	Putative uncharacterized protein
**938**	2.37	15.9	2	2.27	2.11	tr|D6BRD9|D6BRD9_9ROSI	Glutathione-s-transferase theta
**944**	2.35	28.2	20	0.52	0.22	tr|D7LJ40|D7LJ40_ARALL	Fructose-bisphosphate aldolase
**945**	2.35	5.9	3	0.20	0.47	tr|B9ICR4|B9ICR4_POPTR	coatomer subunit delta
**948**	2.34	8.2	4	0.40	0.54	tr|B9H0Q2|B9H0Q2_POPTR	Adenylyl cyclase-associated protein
**949**	2.34	6.7	5	0.09	0.37	sp|P51074|ANX4_FRAAN	Annexin-like protein RJ4
**952**	2.33	23.5	18	0.47	0.33	tr|Q2WFK8|Q2WFK8_9ASTR	Fructose-bisphosphate aldolase
**975**	2.28	14.7	2	1.77	2.07	tr|B9H5H4|B9H5H4_POPTR	dolichol-phosphate mannosyltransferase
**981**	2.26	9.5	3	0.61	1.05	tr|B9H739|B9H739_POPTR	Predicted protein
**982**	2.25	11.6	13	0.29	0.24	tr|Q533V0|Q533V0_FRAAN	Phospholipase D
**990**	2.23	7.3	5	0.59	1.25	tr|B9ILT5|B9ILT5_POPTR	Malic enzyme
**993**	2.23	6.9	2	2.91	0.86	tr|B9RA70|B9RA70_RICCO	Signal recognition particle 68 kDa protein
**1002**	2.2	5.2	2	6.19	3.09	tr|B9SD61|B9SD61_RICCO	Hydrolase; Leukotriene hydrolase
**1008**	2.19	5.9	2	5.06	1.54	tr|B7FM77|B7FM77_MEDTR	Alanine aminotransferase
**1016**	2.18	33.2	7	0.35	1.21	sp|Q40521|RB11B_TOBAC	Ras-related protein Rab11B
**1019**	2.17	12.2	2	1.67	0.30	tr|A5BV59|A5BV59_VITVI	guanine nucleotide-binding protein
**1026**	2.15	3.4	2	2.38	1.60	tr|B9T6N8|B9T6N8_RICCO	Indole-3-acetic acid-amido synthetase
**1029**	2.15	11.9	2	1.66	1.74	tr|B9N7F9|B9N7F9_POPTR	Vesicle transport protein-lik
**1030**	2.14	10	5	0.27	0.38	tr|A9PCM5|A9PCM5_POPTR	Proteasome subunit alpha type
**1037**	2.13	18.7	8	0.28	0.22	tr|B9GTA1|B9GTA1_POPTR	Aminoacyl-tRNA synthetase
**1044**	2.12	6.9	3	0.60	1.09	tr|D7TMA3|D7TMA3_VITVI	Chorismate synthase
**1049**	2.12	3.8	5	0.47	1.01	tr|F6HLA1|F6HLA1_VITVI	pantothenate kinase
**1051**	2.11	4.7	4	0.60	0.93	tr|A9Z0V7|A9Z0V7_ROSDA	Carotenoid cleavage dioxygenase 1
**1059**	2.1	11.6	3	0.56	0.75	tr|B9R8F0|B9R8F0_RICCO	Nucleoredoxin, putative
**1062**	2.09	11.2	2	0.65	1.15	tr|B9SS56|B9SS56_RICCO	Structural molecule, putative
**1065**	2.08	8.7	4	1.66	2.75	tr|Q6TKR0|Q6TKR0_TOBAC	Ribosomal protein L3A
**1066**	2.08	8.1	4	2.21	0.92	tr|Q94KK8|Q94KK8_TOBAC	Glutamate decarboxylase isozyme 3
**1067**	2.08	4.6	5	0.59	0.41	tr|F8WL79|F8WL79_CITUN	Aminopeptidase
**1071**	2.07	14.8	13	0.16	0.28	tr|D7SIX7|D7SIX7_VITVI	serine/threonine-protein phosphatase
**1072**	2.07	6.5	4	0.42	1.36	tr|B3TZ27|B3TZ27_RICCO	Long-chain acyl-CoA synthetase 4
**1081**	2.06	27.4	19	0.28	0.62	tr|F6HZK0|F6HZK0_VITVI	Malate dehydrogenase
**1082**	2.06	8	2	2.05	1.06	tr|B9IF79|B9IF79_POPTR	Aspartyl aminopeptidase

**1085**	2.06	7.6	4	1.58	1.04	tr|Q9M835|Q9M835_ARATH	L-allo-threonine aldolase, putative
**1120**	2.03	10.2	8	0.60	4.17	tr|D1MAF2|D1MAF2_SOLLC	Exportin-1
**1121**	2.03	25.7	7	2.13	0.82	tr|B9SR17|B9SR17_RICCO	Glucose 4-6-dehydratase, putative
**1130**	2.03	23.3	4	0.42	0.88	tr|Q8LCK3|Q8LCK3_ARATH	copper transport protein
**1136**	2.02	3	2	0.51	1.13	tr|Q9SJT7|Q9SJT7_ARATH	V-type H+-transporting ATPase subunit I
**1137**	2.02	26.2	8	0.61	0.53	tr|B9IF97|B9IF97_POPTR	Translation elongation factor
**1155**	2.01	8.5	5	11.07	3.94	tr|B9HA87|B9HA87_POPTR	Galactinol-sucrose galactosyltransferase
**1157**	2.01	3.8	2	1.60	1.63	tr|D7SS21|D7SS21_VITVI	Pyrophosphate-energized membrane proton
**1171**	2	33.9	18	0.61	0.22	tr|C0LDX2|C0LDX2_9ROSI	Glyceraldehyde-3-pho- dehydrogenase
**1172**	2	73.3	18	0.62	1.21	tr|A9P8Q6|A9P8Q6_POPTR	Ras-related protein Rab7
**1173**	2	40.9	17	3.22	1.02	tr|Q9M5X2|Q9M5X2_EUPES	Heat shock protein 80
**1179**	2	11.3	10	1.58	0.71	tr|B9HRP1|B9HRP1_POPTR	Coatomer subunit gamma
**1180**	2	37	8	0.60	0.29	tr|D5K0Z8|D5K0Z8_9FABA	Actin-like protein
**1186**	2	8.5	8	0.60	0.25	tr|B9I7J3|B9I7J3_POPTR	Luminal-binding protein 5
**1188**	2	45.1	5	0.65	0.53	tr|B2X0F6|B2X0F6_9ROSI	Glyceraldehyde 3-phosphate dehydrogenase
**1191**	2	2.9	3	2.58	1.98	tr|F6GZ28|F6GZ28_VITVI	Myosin-Vb-like
**1195**	2	11.3	3	2.05	1.05	tr|D7U7P5|D7U7P5_VITVI	Fructokinase-5-like
**1196**	2	5.8	4	0.65	0.53	tr|C9EA44|C9EA44_CARPA	Ripening-reduced arabinofuranosidase
**1203**	2	16.3	4	1.85	1.08	tr|C6TED4|C6TED4_SOYBN	Ras-related protein RHN1-like
**1206**	2	6.1	2	0.19	0.23	tr|A9P745|A9P745_HELAN	Enolase
**1207**	2	11.2	5	1.51	0.72	tr|B9RFA8|B9RFA8_RICCO	Rubber elongation factor protein
**1210**	2	6.7	2	5.40	1.25	tr|Q8LA96|Q8LA96_ARATH	RNA recognition motif-containing protein
**1211**	2	5.2	2	0.20	0.51	tr|Q40581|Q40581_TOBAC	GTP-binding protein
**1214**	2	6.3	4	0.48	1.01	tr|B9GK93|B9GK93_POPTR	FAS-associated factor 2
**1225**	2	3.7	3	0.66	0.63	tr|A5AEX6|A5AEX6_VITVI	carboxymethylenebutenolidase
**1228**	2	16.8	2	0.61	0.52	tr|B9H401|B9H401_POPTR	Prefoldin subuni
**1231**	2	8.2	3	2.29	3.08	tr|D7SZI9|D7SZI9_VITVI	60S ribosomal protein
**1236**	2	6	2	0.52	0.37	tr|B9HQZ2|B9HQZ2_POPTR	chitinase 1
**1247**	2	8.2	2	0.34	0.82	tr|B9SYE9|B9SYE9_RICCO	ADP-ribosylation factor, putative
**1275**	2	9.2	2	0.61	1.16	tr|B9S7V3|B9S7V3_RICCO	V-type proton ATPase subunit F
**1278**	2	27.6	6	0.39	0.95	tr|A9PBT2|A9PBT2_POPTR	nuclear transport factor 2 isoform 1
**1281**	2	29.8	6	0.51	1.43	tr|A9PE14|A9PE14_POPTR	nuclear transport factor
**1318**	2	8.9	5	0.52	0.12	tr|F6HF70|F6HF70_VITVI	Putative uncharacterized protein
**1320**	2	16.7	2	2.07	1.03	tr|B9T0W0|B9T0W0_RICCO	heat shock factor-binding protein
**1336**	1.95	11.3	4	1.67	4.33	tr|B9SXV3|B9SXV3_RICCO	Calcium lipid binding protein
**1337**	1.94	4.5	2	1.96	1.54	tr|F6HCT7|F6HCT7_VITVI	heat shock 70 kDa protein
**1341**	1.92	11.2	2	0.62	0.58	tr|B9N560|B9N560_POPTR	Predicted protein
**1346**	1.91	5.7	2	1.71	3.28	tr|B9R7B8|B9R7B8_RICCO	Serine/threonine-protein kinase
**1348**	1.89	10.3	3	0.33	1.16	tr|B9S119|B9S119_RICCO	Putative uncharacterized protein
**1353**	1.87	7.1	4	0.45	0.51	tr|Q949H3|Q949H3_HEVBR	Chitinase, class I
**1355**	1.86	22	19	2.75	0.78	tr|B9H023|B9H023_POPTR	GTP-binding protein
**1387**	1.77	77.3	20	0.24	0.82	tr|B7FLK9|B7FLK9_MEDTR	ubiquitin 11
**1404**	1.7	7	2	2.94	3.98	tr|B9RGW6|B9RGW6_RICCO	Conserved hypothetical protein
**1407**	1.69	1.5	2	0.59	0.56	tr|B9I2G0|B9I2G0_POPTR	Predicted protein

**1418**	1.65	7.5	3	0.10	0.21	tr|B9HBL6|B9HBL6_POPTR	aspartate-tRNA ligase, cytoplasmic
**1426**	1.61	8.3	3	0.17	0.93	tr|F6H1I3|F6H1I3_VITVI	random slug protein 5 isoform 2
**1434**	1.58	32.5	19	0.58	0.73	tr|Q6QLW8|Q6QLW8_HEVBR	HMG-CoA synthase 2
**1437**	1.57	5.2	2	3.73	7.78	tr|B9IEB9|B9IEB9_POPTR	methyl transferase
**1453**	1.53	5.9	2	0.64	0.51	tr|Q1W6C5|Q1W6C5_9CARY	chitinase Class Ib
**1459**	1.51	8.9	4	0.60	0.56	tr|B9SBH8|B9SBH8_RICCO	Dynamin, putative
**1464**	1.5	7.2	3	0.60	0.94	tr|A8IKE1|A8IKE1_SOYBN	Alanine aminotransferase 1
**1467**	1.49	20.7	6	0.52	0.24	tr|Q8LBD5|Q8LBD5_ARATH	L-lactate dehydrogenase
**1479**	1.43	14.7	3	0.64	0.79	tr|C6TB50|C6TB50_SOYBN	S-formylglutathione hydrolase-like
**1487**	1.4	5.1	2	2.25	1.82	tr|E0CR92|E0CR92_VITVI	Aminotransferase YbdL-like
**1494**	1.39	6.5	2	0.13	0.29	tr|F6H2P8|F6H2P8_VITVI	Putative uncharacterized protein
**1523**	1.31	3.1	2	2.11	1.52	tr|C6TNV4|C6TNV4_SOYBN	succinyl-CoA ligase subunit alpha-1

Note:

[a]The protein number (I-) obtained from the iTRAQ experiments.

[b]The Unused Score produced by the ProteinPilot™ Software.

[c]The percent of sequence coverage value for the matched peptides with higher than 95% confidence.

[d]The number of matched matched peptides with higher than 95% confidence.

[e]The changed ration of target protein in the E-3 and D-3 rubber latex identified by iTRAQ.

[f]Accession numbers and plant species in the Swiss-Prot Database.

Supplementary Table S4 MS identification of the 59 phosphorlated latex proteins

Spot P-.[a]	Changed ratio[b] E3/D3:E3/D0:D3/D0	Thero.[c] *p*I/M*r*	M.S.[d] Score	NCBI No.[e]	Plant species	Protein name
**P1**	0.26: 0.36: 2.03	5.11/71.6	97	123650	*P. hybrida*	Heat shock cognate 70 kDa protein
**P2**	3.75: 7.78: 2.48	5.52/61.4	[54]	249262	*M. esculenta*	linamarase
**P3**	0.46: 0.56: 0.92	4.54/46.1	[112]	224100473	*P. trichocarpa*	predicted protein
**P4**	1.31: 0.57: 0.45	5.52/61.4	[56]	249262	*M. esculenta*	linamarase
**P5**	4.48: 2.82: 0.48	4.61/12.8	[502]	29170601	*H. brasiliensis*	Small rubber particle protein
**P6**	15.04: 5.74: 0.21	4.61/12.8	[429]	29170601	*H. brasiliensis*	Small rubber particle protein
**P7**	0.46: 1.01: 1.77	5.28/47.1	[75]	224071449	*P. trichocarpa*	phospholipase A
**P8**	5.26: 7.09: 2.01	5.31/42.0	[519]	32186890	*G. hirsutum*	actin
**P9**	0.28: 0.64: 3.21	4.8/22.3	[384]	14423933	*H. brasiliensis*	Small rubber particle protein
**P10**	0.36: 0.43: 1.30	5.28/47.1	[71]	224071449	*P. trichocarpa*	phospholipase A
**P11**	1.13: 0.45: 0.62	5.28/47.1	[52]	224071449	*P. trichocarpa*	phospholipase A
**P12**	0.47: 0.37: 0.46	5.28/47.1	[106]	224071449	*P. trichocarpa*	phospholipase A
**P13**	1.95: 2.15: 1.10	5.49/50.3	[51]	1550740	*A. thaliana*	GDP-associated inhibitor
**P14**	0.37: 0.37: 1.67	6.5/32.1	[144]	255552291	*R. communis*	short chain dehydrogenase, putative
**P15**	0.49: 0.38: 0.87	6.56/39.2	[174]	218157	*O. sativa*	cytoplasmic aldolase
**P16**	2.47: 0.73: 0.38	6.56/39.2	[125]	218157	*O. sativa*	cytoplasmic aldolase
**P17**	4.66: 15.17: 4.53	4.8/22.3	[171]	14423933	*H. brasiliensis*	Small rubber particle protein
**P18**	1.15: 2.32: 3.97	8.42/35.9	[74]	118489480	*P. trichocarpa*	unknown
**P19**	3.29: 3.33: 1.07	5.04/14.7	[341]	132270	*H. brasiliensis*	Rubber elongation factor protein
**P20**	2.81: 2.11: 0.71	5.04/14.7	[597]	132270	*H. brasiliensis*	Rubber elongation factor protein
**P21**	2.33: 1.87: 0.72	5.04/14.7	[672]	132270	*H. brasiliensis*	Rubber elongation factor protein
**P22**	0.18: 0.41: 2.34	4.94/80.3	[197]	38154482	*N. benthamiana*	Hsp 90-1
**P23**	1.42: 3.24: 2.76	4.95/81.1	[126]	1708314	*I. nil*	Heat shock protein 83
**P24**	2.38: 2.65: 1.37	5.07/71.1	[136]	20559	*P. hybrida*	hsp70 (AA 6 - 651)
**P25**	1.55: 2.67: 2.61	5.08/59.1	[118]	255566038	*R. communis*	Phospholipase C 3 precursor
**P26**	2.10: 3.98: 1.61	5.01/46.4	[80]	4235430	*H. brasiliensis*	latex-abundant protein
**P27**	0.61: 3.71: 6.87	5.21/59.4	[53]	297833008	*A. lyrata*	phosphoesterase family protein
**P28**	2.70: 1.93: 0.37	5.23/62.1	[108]	226530579	*Z. mays*	uncharacterized protein
**P29**	2.39: 0.75: 0.41	6.75/59.3	[133]	149938954	*A. chinensis*	G6PD1; glucose-6-phosphate dehydrogenase
**P30**	1.04: 2.15: 3.11	6.26/38.2	[53]	226493285	*Z. mays*	flavonol 4-sulfotransferase
**P31**	0.51: 0.31: 1.34	5.28/47.1	[104]	224071449	*P. trichocarpa*	phospholipase A
**P32**	0.93: 1.36: 2.07	5.0/43.0	[219]	1916805	*H. brasiliensis*	latex patatin homolog
**P33**	0.51: 0.27: 0.41	5.0/43.1	[196]	6707018	*H. brasiliensis*	latex protein allergen Hev b 7
**P34**	0.46: 0.83: 1.33	5.31/41.9	[392]	32186890	*G. hirsutum*	actin
**P35**	5.06: 6.54: 0.89	6.5/32.1	[141]	255552291	*R. communis*	short chain dehydrogenase, putative
**P36**	3.77: 1.01: 0.22	5.06/19.6	[393]	38122474	*H. brasiliensis*	Rubber elongation factor protein
**P37**	0.14: 0.21: 1.29	4.06/15.2	[288]	334854634	*H. brasiliensis*	elicitor-responsive protein
**P38**	7.23: 7.56: 2.19	4.8/22.3	[353]	14423933	*H. brasiliensis*	Small rubber particle protein
**P39**	4.51: 5.10: 1.94	4.8/22.3	[342]	14423933	*H. brasiliensis*	Small rubber particle protein
**P40**	2.06: 2.04: 1.51	4.61/12.8	[316]	29170601	*H. brasiliensis*	Small rubber particle protein
**P41**	0.88: 9.80: 10.89	5.64/20.9	[261]	2832430	*H. brasiliensis*	prohevein

**P42**	2.11: 2.06: 1.12	4.79/29.5	93	388503664	*L. japonicus*	unknown
**P43**	2.33: 3.97: 2.19	4.76/29.9	[294]	326694865	*H. brasiliensis*	14-3-3 protein 2
**P44**	1.55: 3.01: 2.80	5.84/27.4	[69]	224099853	*P. trichocarpa*	predicted protein
**P45**	1.35: 2.18: 1.71	9.4/41.5	[745]	124365253	*H. brasiliensis*	beta-1,3-glucanase
**P46**	1.63: 2.23: 1.35	6.11/36.1	[347]	224102193	*P. trichocarpa*	malate dehydrogenase
**P47**	0.67: 0.46: 0.74	4.65/41.4	[75]	*255578278*	*R. communis*	hypothetical protein
**P48**	2.41: 6.72: 3.69	6.5/32.1	[201]	255552291	*R. communis*	short chain dehydrogenase, putative
**P49**	1.14: 2.77: 2.94	5.17/30.1	[175]	255581753	*R. communis*	adenosine kinase, putative
**P50**	1.39: 1.18: 2.23	5.79/66.6	[96]	4588474	*B. napus*	phosphoinositide phospholipase C
**P51**	1.43: 5.81: 11.34	4.65/41.4	[95]	255578278	*R. communis*	hypothetical protein
**P52**	5.02: 7.38: 1.33	6.5/32.1	[67]	255552291	*R. communis*	short chain dehydrogenase, putative
**P53**	1.26: 2.25: 2.17	5.08/36.3	[279]	224057068	*P. trichocarpa*	predicted protein
**P54**	0.44: 0.43: 0.89	5.64/20.9	[181]	2832430	*H. brasiliensis*	prohevein
**P55**	0.41: 0.46: 0.95	5.28/47.1	[55]	224071449	*P. trichocarpa*	phospholipase A
**P56**	0.24: 0.58: 3.23	6.56/39.2	[58]	218157	*O. sativa*	cytoplasmic aldolase
**P57**	0.40: 0.50: 1.64	5.49/42.2	[120]	15219412	*A. thaliana*	phosphoglycerate kinase
**P58**	5.28: 1.23: 0.22	5.33/63.7	[121]	356513072	*G. max*	phosphoglucomutase, cytoplasmic
**P59**	2.65: 1.03: 0.37	5.46/63.7	[95]	255568794	*R. communis*	nucleoredoxin, putative

Note:

[a]Assigned spot number (P1-P59) of the differentially phosphorylated proteins were indicated in Fig. 5.

[b]The relatively changed ratios (E-3/D-3; E-3/D-0; D-3/D-0) of the phosphorylated protein spots among the D-0, D-3 and E-3 latex proteins from the rubber trees treated by water and ethylene. All the protein spots in the 2-DE gels were analyzed by ImageMaster software.

[c]The theoretical values for the *p*I and mass (kDa)of the identified proteins. The theoretical values were retrieved from the protein database.

[d]Mascot searched scores (M.S.) against NCBInr for the MS and [MS/MS] searched results.

[e]Database accession (gi|) numbers according to NCBInr.
